# VCD Analysis of Axial Chirality in Synthetic Stereoisomeric Biaryl-Type *bis*-Isochroman Heterodimers with Isolated Blocks of Central and Axial Chirality †

**DOI:** 10.3390/ijms25179657

**Published:** 2024-09-06

**Authors:** Zoltán Czenke, Attila Mándi, Sándor Balázs Király, Attila Kiss-Szikszai, Anita Kónya-Ábrahám, Anna Kurucz-Szabados, Krisztián Cserepes, Attila Bényei, Changsheng Zhang, Máté Kicsák, Tibor Kurtán

**Affiliations:** 1 Department of Organic Chemistry, University of Debrecen, P. O. Box 400, 4002 Debrecen, Hungary; 2 Doctoral School of Chemistry, University of Debrecen, Egyetem tér 1., 4032 Debrecen, Hungary; 3 Department of Physical Chemistry, University of Debrecen, Egyetem tér 1., 4032 Debrecen, Hungary; 4 Key Laboratory of Tropical Marine Bio-Resources and Ecology, Guangdong Key Laboratory of Marine Materia Medica, Institutions of South China Sea Ecology and Environmental Engineering, South China Sea Institute of Oceanology, Chinese Academy of Sciences, Guangzhou 510301, China

**Keywords:** stereogenic biaryl axis, stereoisomeric *bis*-isochromans, isolated blocks of chirality, VCD, ECD

## Abstract

Optically active heterodimeric 5,5′-linked *bis*-isochromans, containing a stereogenic *ortho*-trisubstituted biaryl axis and up to four chirality centers, were synthesized stereoselectively by using a Suzuki–Miyaura biaryl coupling reaction of optically active isochroman and 1-arylpropan-2-ol derivatives, providing the first access to synthetic biaryl-type isochroman dimers. Enantiomeric pairs and stereoisomers up to seven derivatives were prepared with four different substitution patterns, which enabled us to test how OR, ECD, and VCD measurements and DFT calculations can be used to determine parallel central and axial chirality elements in three isolated blocks of chirality. In contrast to natural penicisteckins A–D and related biaryls, the ECD spectra and OR data of (a*S*) and (a*R*) atropodiastereomers did not reflect the opposite axial chirality, but they were characteristic of the central chirality. The atropodiastereomers showed consistently near-mirror-image VCD curves, allowing the determination of axial chirality with the aid of DFT calculation or by comparison of characteristic VCD transitions.

## 1. Introduction

The chiral 3-alkyl or 3-methylisochroman (3-metyl-3,4-dihydro-1*H*-isochromene) scaffold is a common subunit in optically active benzene-condensed *O*-heterocyclic secondary metabolites, in which the condensed benzene ring usually contains hydroxyl and methoxy substituents [1,2,3]. Optically active 1-aryl-3-methylisochromans containing phenolic hydroxyl groups were identified as natural components of olive with antioxidant, anti-inflammatory, and neuroprotective activity [4,5,6].

Benzene-condensed chiral *O*-heterocycles often form biaryl-type homo- or heterodimeric natural products with a stereogenic biaryl axis, the formation of which is aided by the presence of activating substituents of the benzene ring such as hydroxyl or alkoxy groups [7]. Depending on the substitution pattern, the hindered rotation along the biaryl axis can result in atropisomers with axial chirality [7] or interconverting conformers with different biaryl helicity [8]. The axial chirality of biaryls is mainly governed by the number and size of the *ortho*-substituents, which impose strong steric repulsion in the *periplanar* transition state, and, thus, hinder the rotation about the biaryl axis. The axial chirality of biaryl natural products often plays a fundamental role in the bioactivity, since atropodiastereomers can possess markedly different activity [9,10,11,12,13].

Although chiral isochroman natural products often contain activating hydroxyl or alkoxy groups on the condensed benzene ring, which would promote oxidative biaryl coupling reactions, there are only two reports on biaryl-type *bis*-isochroman natural products produced by oxidative biaryl coupling [14,15]. The homodimeric 7,7′-linked asperbiphenyl (**1**), the first axially chiral *bis*-isochroman, was isolated from the marine fungus *Aspergillus* sp. and it contains an *ortho*-tetrasubstituted stereogenic biaryl axis and four chirality centers [14].

The absolute configurations (ACs) of the central and axial chirality elements were not determined in asperbiphenyl (**1**). Penicisteckins A–D (**2**–**5**), two pairs of atropodiastereomeric biaryl-type hetero- and homodimeric *bis*-isochromans with 7,5′- and 7,7′-linkages, and a pair of atropodiastereomeric 2-(isochroman-5-yl)-1,4-benzoquinone derivatives [penicisteckin E (**6**) and F (**7**)] were reported as novel biaryl scaffolds containing both central and axial chirality elements from *Penicillium steckii* HNNU-5B18 [15].

The absolute configurations of penicisteckins A–D (**2**–**5**) were determined by single-crystal X-ray diffraction analysis, while those of penicisteckin E (**6**) and F (**7**) by TDDFT (time-dependent density functional theory)-ECD calculations of the atropodiastereomers [15]. The configurational assignment of both central and axial stereogenic elements in biaryl natural products is still a challenging task when single-crystal X-ray analysis and chemical correlation is not applicable. In this case, the combination of electronic (ECD) and vibrational circular dichroism (VCD), and optical rotation (OR) calculations may offer a solution. Biaryl-type atropodiastereomers usually have near-mirror-image ECD spectra when their biaryl chromophore is not symmetrical [16], which can be used for the assignment of axial chirality with the aid of TDDFT-ECD calculations [17,18,19].

The sign and magnitude of the biaryl dihedral angle of the two aryl units in axially chiral biaryls are reflected in the interaction of the two aromatic chromophores, giving rise to exciton-coupled ECD bands. The signs of the exciton-coupled ECD couplets are usually characteristic of the axial chirality, as exemplified by the mirror-image ECD spectra of flavomannin A and B, atropodiastereomeric dihydroanthracenone dimers [20]. However, ECD data do not reflect the absolute configuration of the central chirality elements when they are also present in the condensed heterocyclic rings of biaryls. When the optically active monomeric units are also coisolated with the dimers, they can be analyzed independently by chiroptical methods to assign the central chirality [16,17,18,19,20,21]. The central and axial chirality elements of cephalochromin, a homodimeric naphthpyranone natural product, could be determined simultaneously by VCD calculations [22], but the same approach failed to determine the central chirality elements of flavomannin A [20]. Recently, the axial chirality of 9,10-phenanthrenequinone dimers has been determined by ECD calculations, while the central chirality of the side chains were assigned by TDDFT-OR calculations [23].

**Scheme 1:**
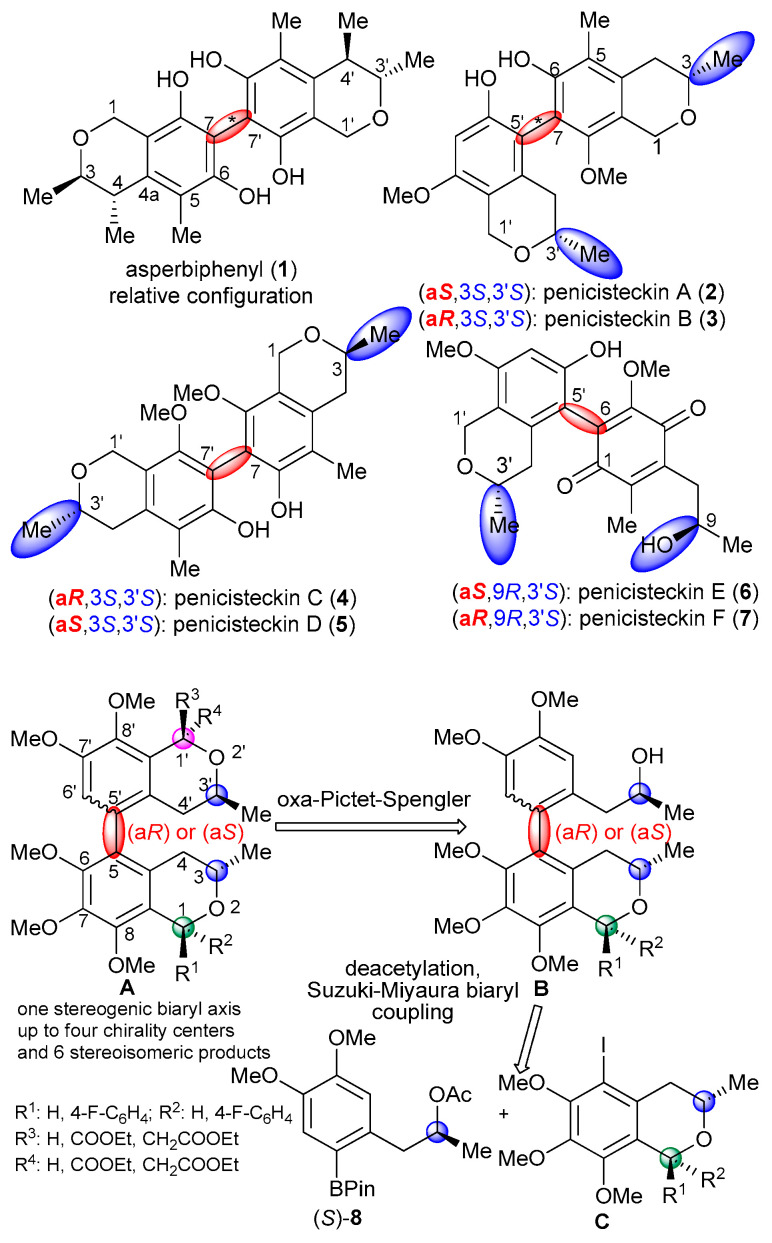
Retrosynthetic scheme for the preparation of axially chiral heterodimeric *bis*-ischromans.

In the current work, we carried out the synthesis of optically active 5,5′-linked *bis*-isochromans with known absolute configuration and different substitution pattern at C-1 and C-1′, which contain an *ortho*-trisubstituted stereogenic biaryl axis and up to four chirality centers (Scheme 1). This is the first synthesis of the *bis*-isochroman scaffold with central and axial chirality.

The stereoselective synthesis enabled us to change the absolute configurations of both axial and central chirality elements and to prepare up to seven stereoisomeric products, including enantiomeric pairs and atropodiastereomers. The stereogenic elements constituted three isolated blocks of chirality, which consisted of the axial chirality and central chirality elements of the two isochroman subunits. The correlation of the configuration for these blocks of chirality is a very common and significant challenge in the stereochemical assignment of axially chiral biaryl-type homo- and heterodimers containing benzene-condensed heterocycles as monomeric units. The stereoisomeric *bis*-isochromans served as model compounds to test the combination of ECD, VCD, and OR measurements and calculations to determine the parallel axial and central chirality elements. With the stereoisomeric *bis*-isochromans in hand, we could test how the different methods could distinguish the different stereoisomers, which may be extended to the stereochemical study of other related axially chiral biaryl natural products. There are several examples available in the literature, in which VCD calculations were utilized to distinguish more than two stereoisomers by exploiting the large number of VCD transitions, characteristic of different stereogenic elements [24,25,26,27]. However, in these examples, only one experimental VCD spectrum was compared to the computed VCDs of the considered stereoisomers, and experimental VCD data could not be recorded for all the stereoisomers to identify differences. In this work, experimental VCD spectra of up to seven stereoisomeric *bis*-isochromans differing in central and axial chirality elements were recorded and used for comparison with the computed VCD spectra. The comparison of experimental VCD data of stereoisomers differing in one to five stereogenic elements could allow the identification of even minor differences in the VCD transitions reporting the stereochemical differences. Moreover, VCDs of structurally closely related *bis*-isochromans with four different substitution patterns were studied, which enabled us to evaluate the effect of structural variations on the characteristic VCD bands.

In addition to the chiroptical approach, the combination of NOE correlations and DFT conformational analysis was also tested to correlate the relative configuration of axial and central chirality elements. The planar structure and absolute configuration were confirmed independently by single-crystal X-ray analysis for three *bis*-isochroman derivatives.

According to our retrosynthetic plan, the target *bis*-isochromans **A** were obtained by an oxa-Pictet–Spengler cyclization reaction of the axially chiral isochroman/1-arylpropan-2-ol conjugate **B**, which diastereoselectively established a new C-1′ chirality center and allowed the variation of the C-1 substituent (Scheme 1). The key step of the scheme is the Suzuki–Miyaura biaryl cross-coupling reaction of *cis*- or *trans*-5-iodoisochromans **C** with the pinacolatoboronate ester derivative (*S*)-**8**, which produced the *ortho*-trisubstituted stereogenic biaryl axis [**C** + (*S*)-**8** → **B**]. Chirality transfer from central to axial is expected to take place during formation of the biaryl axis. The boronate ester derivative (*S*)-**8** and the 5-iodoisochromans **C** could be traced back to (*S*)-propylenoxide [(*S*)-**11**] [28] in two separate three- and four-step sequences.

During the synthesis of the target *bis*-isochromans A, we could change the absolute configuration of the C-3 and C-3′ chirality centers by using (*R*)-propylene oxide [(*R*)-**11**] [29] instead of (*S*)-**11**, which allowed the preparation of enantiomeric pairs. The formation of new axial and central chirality stereogenic elements during the biaryl cross-coupling and oxa-Pictet–Spengler reactions produced a series of stereoisomeric bis-isochromans, which were analyzed by ECD, VCD, OR, NOE measurements, X-ray analysis, and DFT calculations.

## 2. Results and Discussion

### 2.1. Synthesis

**Scheme 2:**
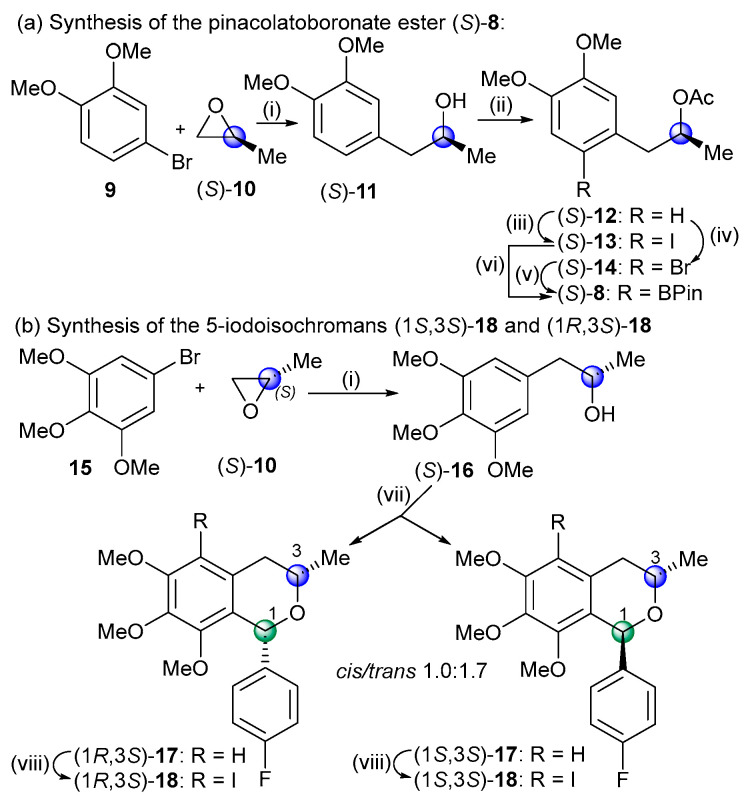
Synthesis of the cross-coupling partners for the Suzuki reaction. Reagents and conditions: (i) (a) *n*-BuLi, Ar/N_2_, THF, −80 °C, 20 min; (b) (*S*)-propylene oxide, Ar/N_2_, −80 °C, 20 min; (c) BF_3_^.^Et_2_O, Ar/N_2_, 80 °C, 30 min, (*S*)-**11** (66%), (*S*)-**16** [30] (90%); (ii) AcCl, C_5_H_5_N, CH_2_Cl_2_, rt, 2.5 h, (*S*)-**12** [31] (84%); (iii) NIS, F_3_CCOOH, MeCN, rt, 16 h, (*S*)-**13** (96%); (iv) NBS, MeCN, rt, 16 h, (*S*)-**14** (97%); (v) (a) (Ph_3_P)_2_PdCl_2_, PPh_3_, KOAc, Ar/N_2_, DMF, rt, 15 min, (b) B_2_Pin_2_, Ar/N_2_, 150 °C, 3 h, (*S*)-**8** (95%); (vi) (a) (Ph_3_P)_2_PdCl_2_, PPh_3_, NaOAc, Ar/N_2_, DMF, rt, 15 min, (b) B_2_Pin_2_, Ar/N_2_, 150 °C, 1 h, (*S*)-**8** (56%); (vii) 4-F-C_6_H_4_CHO, BF_3_^.^Et_2_O, CH_2_Cl_2_, 0 °C, 2 h → rt, 4 h, *cis*-(1*R*,3*S*)-**17** (36%), *trans*-(1*S*,3*S*)-**17** (61%); (viii) NIS, F_3_CCOOH, MeCN, rt, 16 h, *cis*-(1*R*,3*S*)-**18** (69%), *trans*-(1*S*,3*S*)-**18** (92%).

The optically active coupling partners of the biaryl cross-coupling reactions, the boronate ester (*S*)-**8,** and the diastereomeric 1-aryl-5-iodoisochromans (1*S*,3*S*)-**18** and (1*R*,3*S*)-**18** were prepared in short sequences from (*S*)-propylene oxide (Scheme 2).

In the first step, the aryl lithium reagents, formed in situ in the reaction of 4-bromoveratrole (**9**) or 1-bromo-3,4,5-trimethoxy-benzene (**15**) with *n*-butyllithium, opened the epoxide ring, regioselectively producing the (*S*)-**2**-arylpropan-2-ol derivatives (*S*)-**11** and (*S*)-**16** [30]. Acetylation of (*S*)-**11** [28] and subsequent regioselective halogenation with *N*-halosuccinimide afforded the substrates (*S*)-**13** and (*S*)-**14** of the Miyaura borylation. The Miyaura borylation of the iodo derivative (*S*)-**13** resulted in the product (*S*)-**8** with low yield (56%) due to the competing dehalogenation side reaction. Thus, the bromo derivative was used instead as the substrate, which improved the yield of (*S*)-**8** to 95%.

**Scheme 3:**
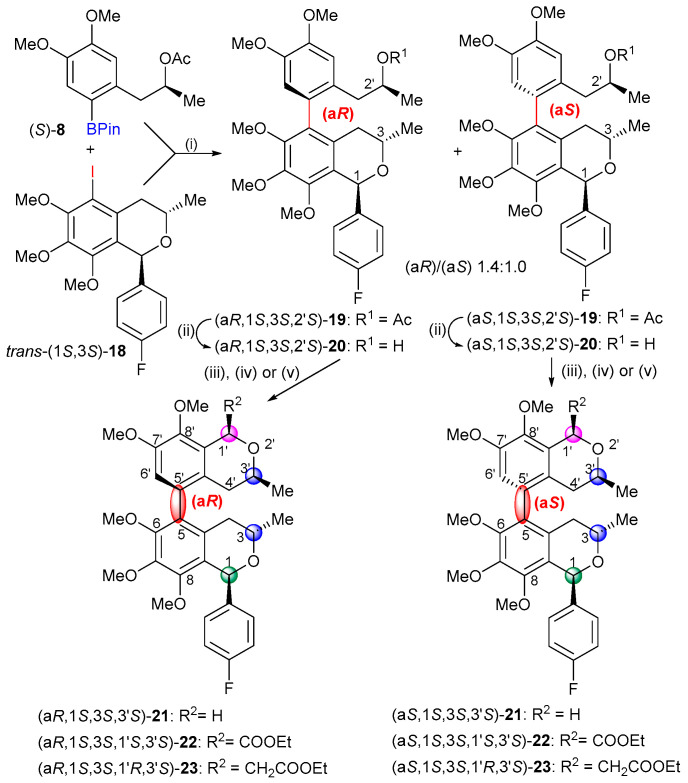
Suzuki cross-coupling reactions of *trans*-(1*S*,3*S*)-**18** and subsequent cyclizations. Reagents and conditions: (i) (a) *trans*-(1*S*,3*S*)-**18**, Xantphos, Pd(OAc)_2_, Ar/N_2_, DMF, rt, 1 h; (b) (*S*)-**8**, CsF, Ar/N_2_, DMF, rt 30 min; (c) a + b, Ar/N_2_, 150 °C, 1.5 h, mixture of (a*S*,1*S*,3*S*,2′*S*)-**19** and (a*R*,1*S*,3*S*,2′*S*)-**19** 79% (*dr* 1.0:1.4); (ii) LiOH, MeOH:THF:H_2_O (5:2:3), rt, 4h, (a*S*,1*S*,3*S*,2′*S*)-**20** (40%), (a*R*,1*S*,3*S*,2′*S*)-**20** (53%); (iii) MOMCl, ZnCl_2_, Et_2_O, 0 °C, 4 h, (a*R*,1*S*,3*S*,3′*S*)-**21** (99%), 6 h, (a*S*,1*S*,3*S*,3′*S*)-**21** (96%); (iv), (Et_2_O)_2_CHCOOEt, BF_3_^.^Et_2_O, toluene, see actual conditions in the SI, (a*R*,1*S*,3*S*,1′*S*,3′*S*)-**22** (71%), (a*S*,1*S*,3*S*,1′*S*,3′*S*)-**22** (38%); (v) (Et_2_O)_2_CHCH_2_COOEt, BF_3_^.^Et_2_O, toluene, 0 °C → rt, 3 h, (a*R*,1*S*,3*S*,1′*R*,3′*S*)-**23** (94%), 4 h, (a*S*,1*S*,3*S*,1′*R*,3′*S*)-**23** (90%).

For the preparation of the 1-aryl-5-iodoisochroman coupling partners, an oxa-Pictet–Spengler cyclization of (*S*)-**16** was carried out with 4-fluorobenzaldehyde to produce the diastereomers *cis*-(1*R*,3*S*)-**17** and *trans*-(1*S*,3*S*)-**17** with a ratio 1.0:1.7. The *cis*- and *trans*-diastereomers were separated by column chromatography, and the absolute configuration of the *cis*-(1*R*,3*S*) isomer was determined by the NOE correlation of the 1-H and 3-H protons. The Suzuki biaryl coupling of *trans*-(1*S*,3*S*)-**18** and (*S*)-**8** was carried out with Pd(OAc)_2_ and different phosphine ligands such as SPhos (66%, *dr* 1.0:1.2), Xantphos (79%, *dr* 1.0:1.4), and (*S*)-BINAP (54%, *dr* 1.0:1.7), resulting in the mixture of axially chiral biaryls (a*R*)- and (a*S*)-**19** with slight atropodiastereoselectivity, favoring the (a*R*) atropodiastereomers (Scheme 3). The duplication of some characteristic ^1^H- and ^13^C-NMR signals such as those of the C-3 methyl indicated the presence of the atropodiastereomers due to the *ortho*-trisubstituated biaryl axis. The low atropodiastereoselectivity was exploited to prepare two series of atropodiastereomeric *bis*-isochromans, since after the removal of the *O*-acetyl group, (a*R*)- and (a*S*)-**20** could be separated by column chromatography and they were used for further cyclization.

Then, oxa-Pictet–Spengler cyclization of (a*R*)- and (a*S*)-**20** with methoxy-methyl chloride (MOMCl) afforded the 5,5′-linked atropodiastereomeric *bis*-isochromans heterodimers (a*R*)- and (a*S*)-**21** with identical absolute configuration at the three chirality centers but different axial chirality. The cyclization was also performed with ethyl diethoxyacetate and ethyl 3,3-diethoxypropionate in the presence of boron trifluoride to produce the atropodiastereomeric pairs (a*R*)-/(a*S*)-**22** and (a*R*)-/(a*S*)-**23**, respectively, in which an additional C-3′ chirality center was introduced with 1,3 *cis*-diastereoselectivity. The observed NOE correlation of the axial 1′-H and 3′-H protons allowed the determination of their *cis* relative configuration, which, on the basis of the known (3′*S*) absolute configuration, also afforded the absolute configuration of the C-1′ chirality center. The planar structure and absolute configuration of (a*R*,1*S*,3*S*,3′*S*)-**21** (ω_C-6,C-5,C-5′,C-4a′_ = −74.8°) were also confirmed by single-crystal X-ray diffraction analysis (Figure S269, CCDC deposition no.: 2249290).

**Scheme 4:**
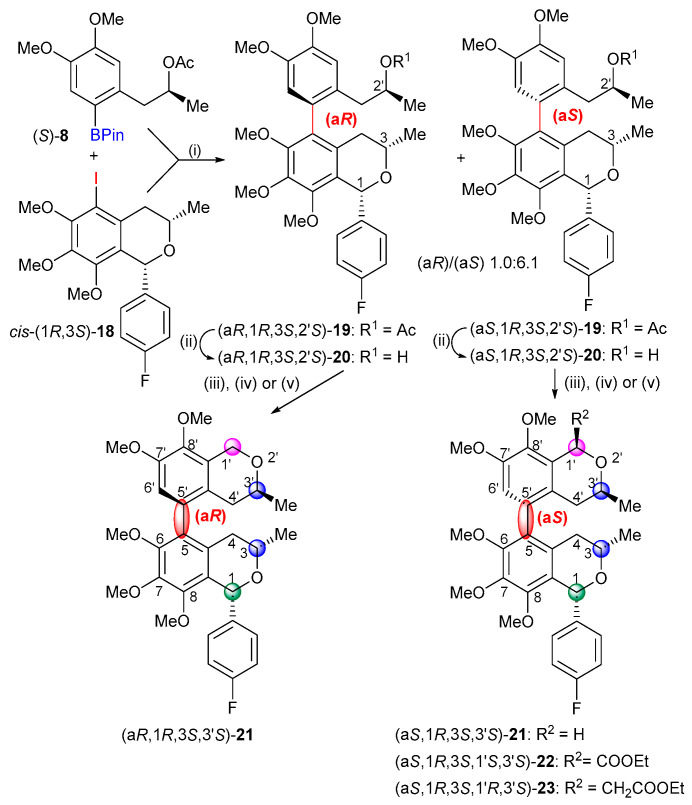
Suzuki cross-coupling reaction of *cis*-(1*R*,3*S*)-**18** and subsequent cyclizations. Reagents and conditions: (i) (a) *cis*-(1*R*,3*S*)-**18**, Xantphos, Pd(OAc)_2_, N_2_, DMF, rt, 1 h; (b) (*S*)-**8**, CsF, N_2_, DMF, rt 30 min; (c) a + b, N_2_, 150 °C, 1.5 h, (a*R*,1*R*,3*S*,2′*S*)-**19** (11%) and (a*S*,1*R*,3*S*,2′*S*)-**19** (68%); (ii) LiOH, MeOH:THF:H_2_O (1:0.8:0.5), rt, 4 h, (a*R*,1*R*,3*S*,2′*S*)-**20** (94%) and LiOH, MeOH:THF:H_2_O (5:2:3), rt, 4 h, (a*S*,1*R*,3*S*,2′*S*)-**20** (96%). (iii) MOMCl, ZnCl_2_, Et_2_O, 0 °C, 3 h, (a*R*,1*R*,3*S*,3′*S*)-**21** (89%), 4 h, (a*S*,1*R*,3*S*,3′*S*)-**21** (97%); (iv) (Et_2_O)_2_CHCOOEt, BF_3_^.^Et_2_O, toluene, see actual conditions in SI, (a*S*,1*R*,3*S*,1′*S*,3′*S*)-**22** (23%); (v) (Et_2_O)_2_CHCH_2_COOEt, BF_3_^.^Et_2_O, toluene, 0 °C → rt, 16 h, (a*S*,1*R*,3*S*,1′*R*,3′*S*)-**23** (91%).

The Suzuki biaryl coupling of *cis*-(1*R*,3*S*)-**17** and (*S*)-**8** was carried out with Pd(OAc)_2_ and Xantphos to yield the atropodiastereomers (a*R*,1*R*,3*S*,2′*S*)-**19** (11%) and (a*S*,1*R*,3*S*,2′*S*)-**19** (68%) with a diastereomeric ratio of 1.0:6.1, favoring the (a*S*) atropodiastereomer (Scheme 4). The atropodiastereomers could be separated by column chromatography and after deacylation (**19** → **20**); both of them were cyclized with MOMCl to produce the *bis*-isochromans heterodimers (a*R*,1*R*,3*S*,3′*S*)- and (a*S*,1*R*,3*S*,3′*S*)-**21**. The oxa-Pictet–Spengler cyclizations of (a*S*,1*R*,3*S*,2′*S*)-**20** with ethyl diethoxyacetate and ethyl 3,3-diethoxypropionate afforded the *bis*-isochromans (a*S*,1*R*,3*S*,1′*S*,3′*S*)-**22** and (a*S*,1*R*,3*S*,1′*R*,3′*S*)-**23**, respectively.

We repeated the sequences presented in Scheme 2, Scheme 3 and Scheme 4 starting from the (*R*)-propylene oxide [(*R*)-**10**] to produce enantiomeric pairs for **21**–**23**, which could help in identifying weak transitions and artefacts in the VCD spectra and, hence, validated our chiroptical approach to determine parallel axial and central chirality. The planar structure and absolute configuration (a*S*,1*R*,3*R*,3′*R*)-**21** (ω_C-6,C-5,C-5′,C-4a′_ = 75.1°, Figure S270, CCDC deposition no.: 2249291) and (a*S*,1*R*,3*R*,1′*R*,3′*R*)-**22** (ω_C-6,C-5,C-5′,C-4a′_ = 80.7°, Figure S271, CCDC deposition no.: 2249292) were also determined by single-crystal X-ray diffraction analysis.

**Scheme 5:**
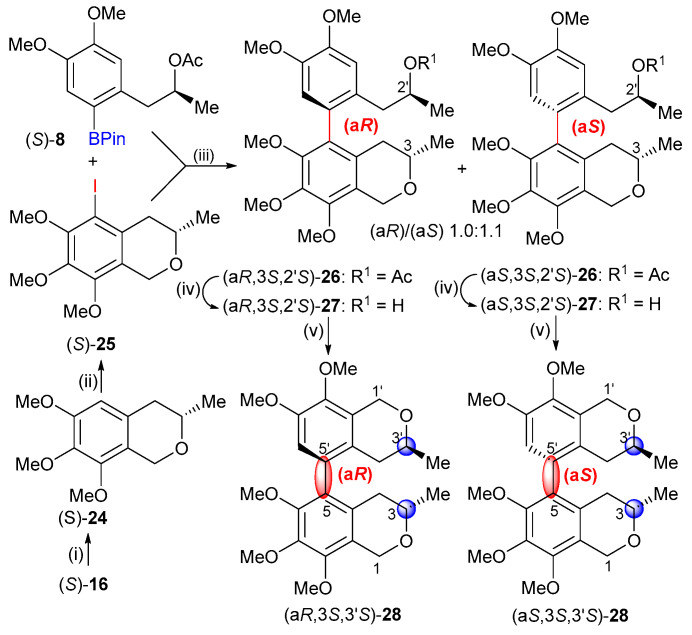
Suzuki cross-coupling reaction of (*S*)-**25** and subsequent cyclization. Reagents and conditions: (i) MOMCl, ZnCl_2_, Et_2_O, 0 °C, 2 h, (*S*)-**24** (91%); (ii) NIS, F_3_CCOOH, MeCN, rt, 1.5 h, (*S*)-**25** (93%) (iii) (a) (*S*)-**25**, SPhos, Pd(OAc)_2_, Ar, DMF, rt, 1 h; (b) (*S*)-**8**, CsF, Ar, DMF, rt, 30 min; (c) a + b, Ar, 150 °C, 1.5 h, (a*R*,3*S*,2′*S*)-**26** and (a*S*,3*S*,2′*S*)-**26** (66%); (ii) LiOH, MeOH:THF:H_2_O (7:2:2), rt, 3 h, mixture of (a*R*,3*S*,2′*S*)-**27** and (a*S*,3*S*,2′*S*)-**27** (91%); (v) MOMCl, ZnCl_2_, Et_2_O, 0 °C, 4 h, mixture of (a*R*,3*S*,2′*S*)-**28** and (a*S*,3*S*,2′*S*)-**28** (86%).

In order to prepare axially chiral *bis*-isochromans lacking both the C-1 and C-1′ chirality centers, we converted the 1-arylpropan-2-ol derivative (*S*)-**16** to the substituted 5-iodoisochroman (*S*)-**25** in two steps, which was used for the Suzuki cross-coupling reaction with (*S*)-**8** (Scheme 5). The two atropodiastereomers (a*R*,3*S*,2′*S*)-**26** and (a*S*,3*S*,2′*S*)-**26** were obtained in a 1.0:1.1 ratio as a mixture, which could not be separated by column chromatography. The diastereomeric mixture was deacetylated (**26** → **27**) and the oxa-Pictet–Spengler cyclization with MOMCl afforded the 5,5′-linked atropodiastereomeric *bis*-isochroman heterodimers (a*R*,3*S*,3′*S*)-**28** and (a*S*,3*S*,3′*S*)-**28**, which contained a stereogenic biaryl axis and only two chirality centers. Preparative chiral HPLC analysis using Chiralpak IC column allowed the separation of (a*R*,3*S*,3′*S*)-**28** and (a*S*,3*S*,3′*S*)-**28**, which were used for chiroptical analysis.

### 2.2. Stereochemical Analysis

Seven stereoisomers of **21**, containing three known chirality centers along with the stereogenic biaryl axis, were used as model compounds for ECD, VCD, and OR measurements and calculations to test how to determine the axial chirality in the presence of central chirality elements or vice versa. Six stereoisomers of both **22** and **23**, consisting of three enantiomeric pairs, were prepared for the stereochemical analysis, which contained four known chirality centers along with the stereogenic biaryl axis. We studied two atropodiastereomers of **28**, which had only two central chirality elements with the same (*S*) absolute configuration.

**Figure 1:**
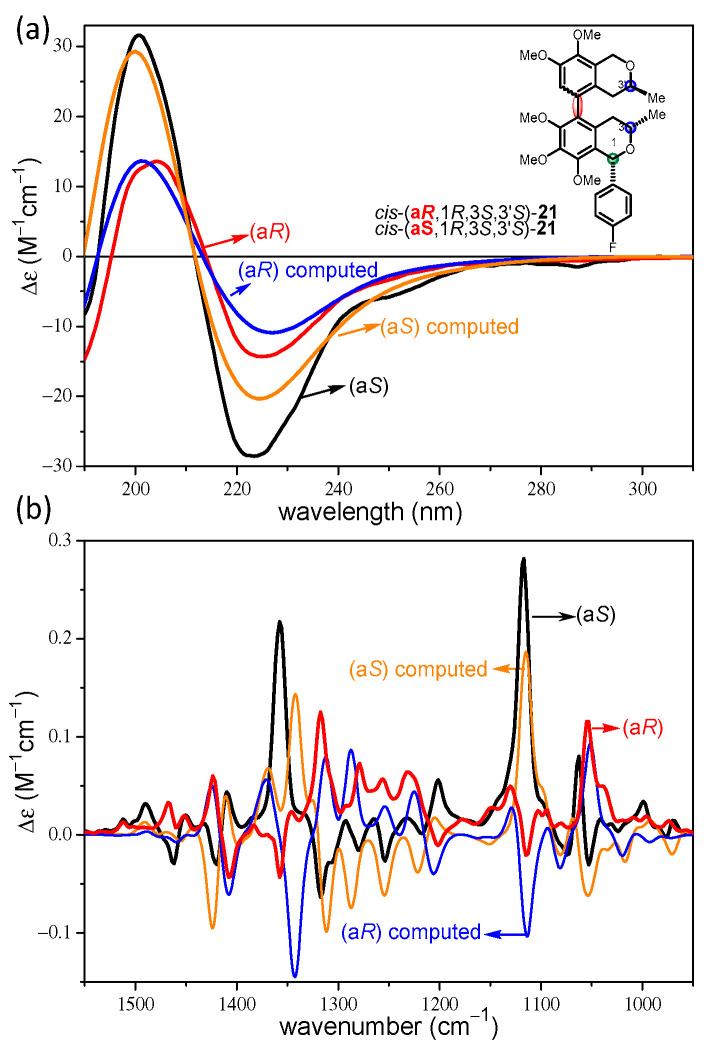
(**a**) Comparison of the experimental ECD spectra of *cis*-(a*R*,1*R*,3*S*,3′*S*)- (red) and *cis*-(a*S*,1*R*,3*S*,3′*S*)-**21** (black) with the corresponding computed B3LYP/TZVP PCM/MeCN ECD spectra (blue and orange). (**b**) Comparison of the experimental VCD spectra of *cis*-(a*R*,1*R*,3*S*,3′*S*)- (red) and *cis*-(a*S*,1*R*,3*S*,3′*S*)-**21** (black) with the corresponding computed B3LYP/TZVP PCM/CHCl_3_ VCD spectra (blue and orange).

The enantiomeric pairs of **21**–**23** provided mirror-image experimental ECD and VCD curves, which allowed the validation of the weak Cotton effects (CEs) and artefacts as well as our VCD measurement protocol (Figures S153–158, S173–178, S185–190). Experimental and computed ECD, VCD, and OR data of atropodiastereomers of **21** were compared to identify chiroptical features that can deduce the axial chirality in the presence of central chirality elements. Surprisingly, the atropodiastereomeric *cis*-(a*R*,1*R*,3*S*,3′*S*)- and *cis*-(a*S*,1*R*,3*S*,3′*S*)-**21** showed near-identical ECD spectra with a broad negative couplet below 300 nm (Figure 1a), which were not suitable to distinguish the atropodiastereomers and assign the axial chirality. The experimental ECD curves of *cis*-(a*R*,1*R*,3*S*,3′*S*)- and *cis*-(a*S*,1*R*,3*S*,3′*S*)-**21** differed only in the intensities and they were reproduced well by the corresponding computed B3LYP/TZVP PCM/MeCN ECD spectra. In axially chiral biaryl natural products, the sign and magnitude of the biaryl dihedral angle usually primarily governs the exciton-coupled ECD spectrum, the pattern of which is characteristic of the axial chirality even in the presence of central chirality elements. The anomalous ECD behavior of the atropodiastereomeric *cis*-(a*R*,1*R*,3*S*,3′*S*)- and *cis*-(a*S*,1*R*,3*S*,3′*S*)-**21** could be attributed to either the presence of the C-1 4-fluorophenyl group, which could interact with the biaryl chromophore with exciton coupling, or the substitution pattern of the 5,5′-linked biaryl, which provided near parallel orientation of the interacting electric transition moments of the two isochroman subunits. In contrast, the experimental VCD spectra of *cis*-(a*R*,1*R*,3*S*,3′*S*)- and *cis*-(a*S*,1*R*,3*S*,3′*S*)-**21** showed almost mirror-image curves in the wavenumber range 1100–1450 cm^−1^, which reflected the different axial chirality of the atropodiastereomers, and they could be reproduced well by the VCD calculations (Figure 1b). On the basis of the good agreements, the axial chirality could be assigned to the near-mirror-image VCD curves.

**Figure 2:**
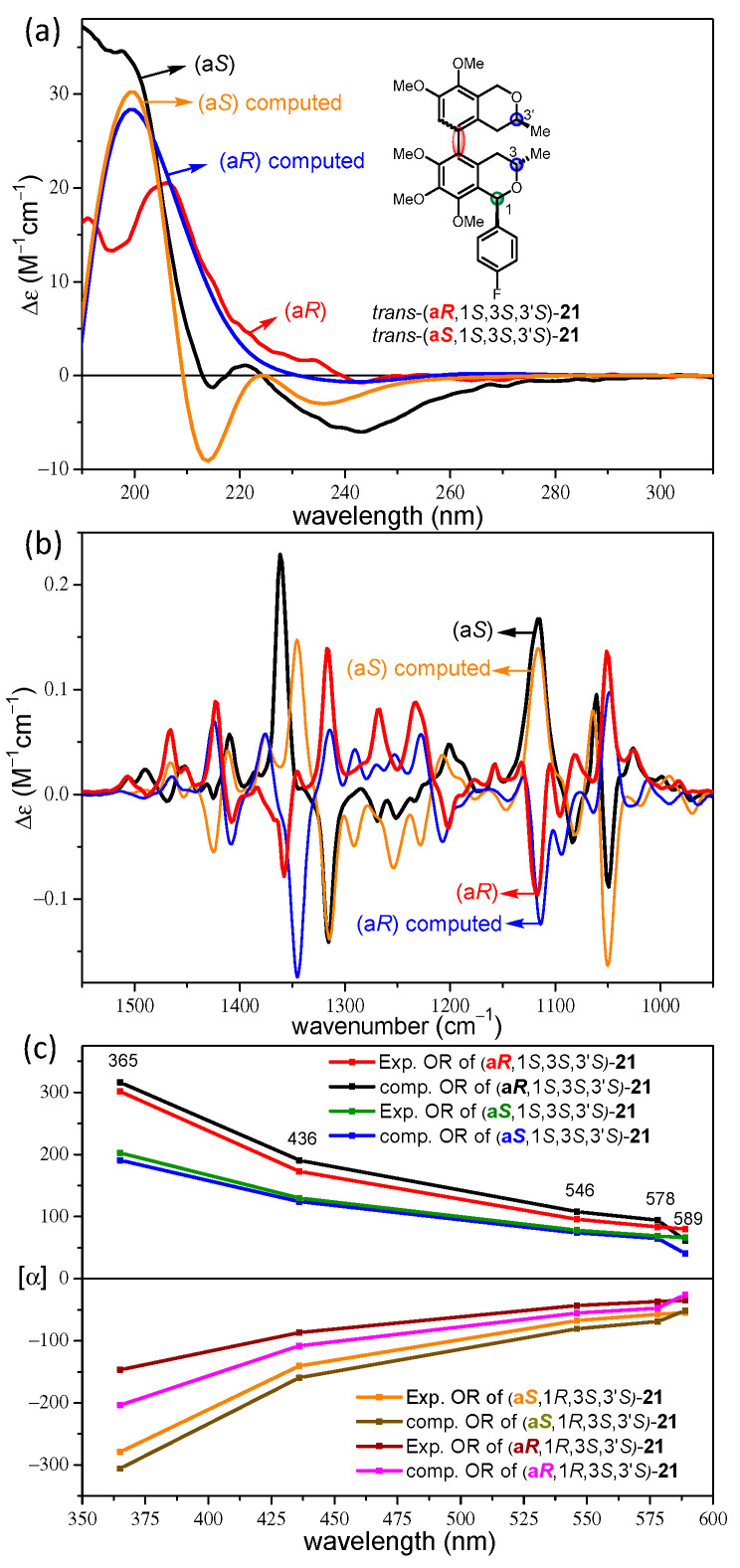
(**a**) Comparison of the experimental ECD spectra of *trans*-(a*R*,1*S*,3*S*,3′*S*)- (red) and *trans*-(a*S*,1*S*,3*S*,3′*S*)-**21** (black) with the corresponding computed B3LYP/TZVP PCM/MeCN ECD spectra (blue and orange). (**b**) Comparison of the experimental VCD spectra of *trans*-(a*R*,1*S*,3*S*,3′*S*)- (red) and *trans*-(a*S*,1*S*,3*S*,3′*S*)-**21** (black) with the corresponding computed B3LYP/TZVP PCM/CHCl_3_ VCD spectra (blue and orange). (**c**) Experimental and computed OR values of *cis*-(a*R*/a*S*,1*R*,3*S*,3′*S*)- and *trans*-(a*R*/a*S*,1*S*,3*S*,3′*S*)-**21** at 589, 578, 546, 436 and 365 nm.

The ECD spectra of the atropodiastereomeric *trans*-(a*R*,1*R*,3*S*,3′*S*)- and *trans*-(a*S*,1*R*,3*S*,3′*S*)-**21** also had quite a similar pattern; they had a negative CE around 240 nm and a more intense positive one below 210 nm (Figure 2a). The weak negative CE at 215 nm of *trans*-(a*S*,1*S*,3*S*,3′*S*)-**21** represented the sole slight difference that could be reproduced well by the ECD calculations to differentiate the (a*S*) and (a*R*) atropodiastereomers. However, the experimental ECD spectra of both atropodiastereomers and enantiomers were required for this assignment, and if only one of the two atropodiastereomers was available for ECD measurement, a reliable assignment of the axial chirality could not be achieved by the ECD calculations. There were much more apparent differences in the experimental VCD spectra of *trans*-(a*R*,1*R*,3*S*,3′*S*)- and *trans*-(a*S*,1*R*,3*S*,3′*S*)-**21**, since near-mirror-image VCD transitions were recorded in the range of 1050–1450 cm^−1^ (Figure 2b). The VCD transitions were clearly determined by the opposite axial chirality, which were reproduced well by the DFT VCD calculations. The agreement of the experimental and computed VCD spectra was utilized to determine the axial chirality.

The OR values of the four stereoisomers *cis*-(a*R*/a*S*,1*R*,3*S*,3′*S*)- and *trans*-(a*R*/a*S*,1*S*,3*S*,3′*S*)-**21** were recorded in acetonitrile at 589, 578, 546, 436, and 365 nm and computed with the BH&HLYP/TZVP PCM/MeCN method (Figure 2c).

Both atropodiastereomers *cis*-(a*R*/a*S*,1*R*,3*S*,3′*S*)-**21** gave monotonously increasing positive ORD curves in the 589–365 nm wavelength range, with *cis*-(a*R*,1*R*,3*S*,3′*S*)-**21** giving significantly larger magnitudes, which could be reproduced well by the OR calculations. The reproduction of the differences in the intensity of the positive OR values of *cis*-(a*R*/a*S*,1*R*,3*S*,3′*S*)-**21** by OR calculations may be used to assign the (a*R*)/(a*S*) axial chirality. However, this approach would be only feasible when the experimental OR values of both atropodiastereomers are available, which is usually not the case. The two atropodiastereomers *trans*-(a*R*/a*S*,1*S*,3*S*,3′*S*)-**21** showed a monotonously decreasing ORD curve with the same negative signs but different intensities. The ORD curve of *trans*-(a*R*,1*S*,3*S*,3′*S*)-**21** ran above that of *trans*-(a*S*,1*S*,3*S*,3′*S*)-**21**, and were reproduced well by the OR calculations. The OR values recorded at five wavelengths showed identical positive and negative signs for the atropodiastereomeric pairs *cis*-(a*R*/a*S*,1*R*,3*S*,3′*S*)- and *trans*-(a*R*/a*S*,1*S*,3*S*,3′*S*)-**21**, respectively. Although there were consistent differences in the magnitudes, they could not be used to distinguish and assign the atropodiastereomers efficiently. In contrast, the C-1 epimeric pairs *cis*-(a*R*,1*R*,3*S*,3′*S*)/*trans*-(a*R*,1*S*,3*S*,3′*S*)-**21** and *cis*-(a*S*,1*R*,3*S*,3′*S*)/*trans*-(a*S*,1*S*,3*S*,3′*S*)-**21** differing only in the absolute configuration of the C-1 chirality center had oppositely signed OR values at all the tested wavelengths, which could be used to assign the AC of C-1.

**Figure 3:**
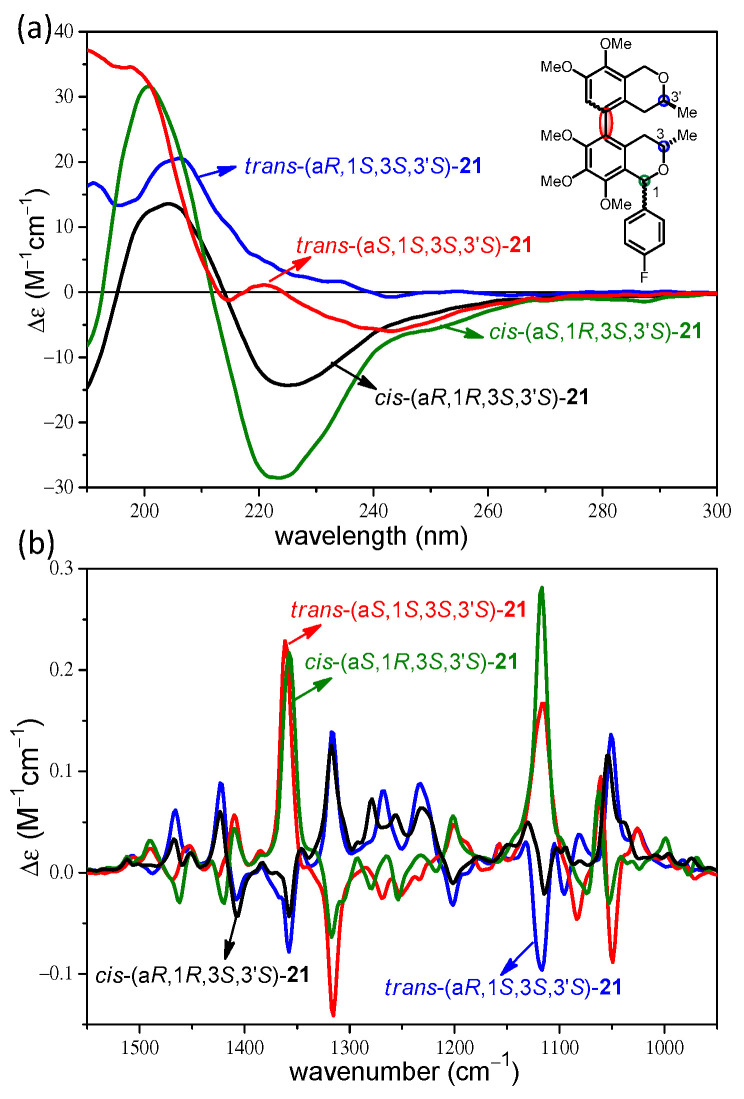
(**a**) Overlapped experimental ECD spectra and (**b**) VCD spectra of *trans*-(a*R*,1*S*,3*S*,3′*S*)- (blue), *trans*-(a*S*,1*S*,3*S*,3′*S*)-**21** (red), *cis*-(a*R*,1*R*,3*S*,3′*S*)- (black), and *cis*-(a*S*,1*R*,3*S*,3′*S*)-**21** (olive).

The comparison of the experimental ECD of the four stereoisomers *cis*-(a*R*/a*S*,1*R*,3*S*,3′*S*)- and *trans*-(a*R*/a*S*,1*S*,3*S*,3′*S*)-**21** showed that there are significant differences in the sign, shape, and intensity of the ECD bands for the C-1 epimeric pairs *cis*-(a*R*,1*R*,3*S*,3′*S*)/*trans*-(a*R*,1*S*,3*S*,3′*S*)-**21** and *cis*-(a*S*,1*R*,3*S*,3′*S*)/*trans*-(a*S*,1*S*,3*S*,3′*S*)-**21** in the wavelength range 210–230 nm (Figure 3a). For instance, the *cis*-(a*S*,1*R*,3*S*,3′*S*)-**21** had an intense negative CE at 224 nm, while the epimeric *trans*-(a*S*,1*S*,3*S*,3′*S*)-**21** showed a weak positive CE at 221 nm and a weak negative one at 215 nm. Similarly, the *cis*-(a*R*,1*R*,3*S*,3′*S*)-**21** exhibited an intense negative CE at 226 nm, while the epimeric *trans*-(a*R*,1*S*,3*S*,3′*S*)-**21** had a positive shoulder at that wavelength. When aided with TDDFT-ECD calculations, the ECD spectra could distinguish and assign efficiently the C-1 epimers of **21** but they cannot be used to determine the axial chirality.

**Figure 4:**
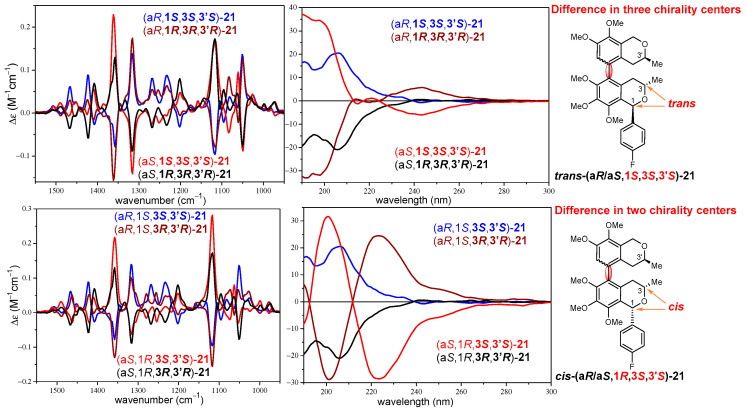
Comparison of the experimental ECD and VCD spectra of stereoisomers of 21 differing in three or two central chirality centers.

**Figure 5:**
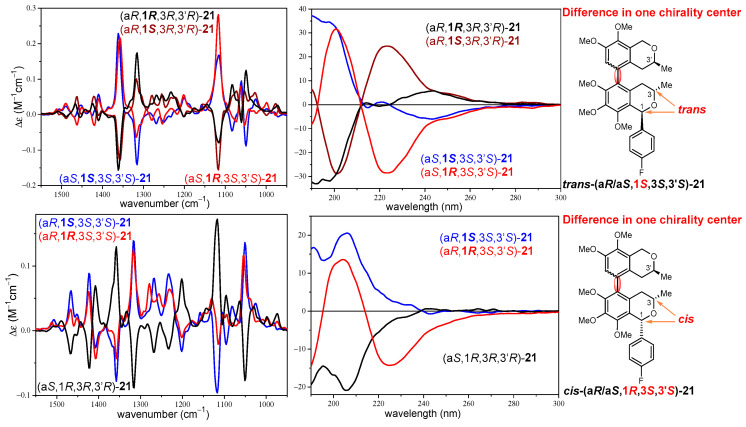
Comparison of the experimental ECD and VCD spectra of C-1 epimeric pairs of 21 (*cis versus trans*).

In contrast, the VCD spectra of the four stereoisomers *cis*-(a*R*/a*S*,1*R*,3*S*,3′*S*)- and *trans*-(a*R*/a*S*,1*S*,3*S*,3′*S*)-**21** showed near-mirror-image VCD transitions for the (a*R*)/(a*S*) atropodiastereomeric pairs allowing the assignment of the axial chirality with the aid of DFT VCD calculations (Figure 3b). Regarding the C-1 epimeric pairs *cis*-(a*R*,1*R*,3*S*,3′*S*)/*trans*-(a*R*,1*S*,3*S*,3′*S*)-**21** and *cis*-(a*S*,1*R*,3*S*,3′*S*)/*trans*-(a*S*,1*S*,3*S*,3′*S*)-**21**, the VCD showed only minor differences in weak transitions in the range of 1250–1270 cm^−1^. When considering diastereomeric pairs differing in the absolute configuration of one, two, or three chirality centers but having identical axial chirality, the ECD spectra were markedly different or near mirror image (Figure S163), while the VCD spectra showed no or only minor differences, especially in the 1200–1300 cm^−1^ wavenumber range (Figures S161–S172). For instance, stereoisomers differing in all the three chirality centers such as *trans*-(a*R*,1*S*,3*S*,3′*S*)-**21** and *trans*-(a*R*,1*R*,3*R*,3′*R*)-**21** had quite different ECD curves with opposite CE below 220 nm, while the main VCD transitions had the same signs and only small differences could be observed in the range of 1200–1300 cm^−1^ (Figure 4). Similarly, stereoisomers differing in the C-3 and C-3′ chirality centers such as *trans*-(a*R*,1*S*,3*S*,3′*S*)-**21** and *cis*-(a*R*,1*S*,3*R*,3′*R*)-**21** showed opposite CEs in the ECD spectra below 220 nm, while minor VCD differences were again restricted to the range of 1200–1300 cm^−1^ (Figure 4). Experimental ECD and VCD spectra of three C-1 epimeric pairs of **21** were compared in Figure 5, distinguishing the *cis/trans* configuration of the trimethoxyisochroman subunit. The ECD spectra of the C-1 epimers had significant differences in the 220–240 nm range, while minor differences appeared for the weak C–H deformation vibrations (1200–1300 cm^−1^) in the VCD spectra.

**Figure 6:**
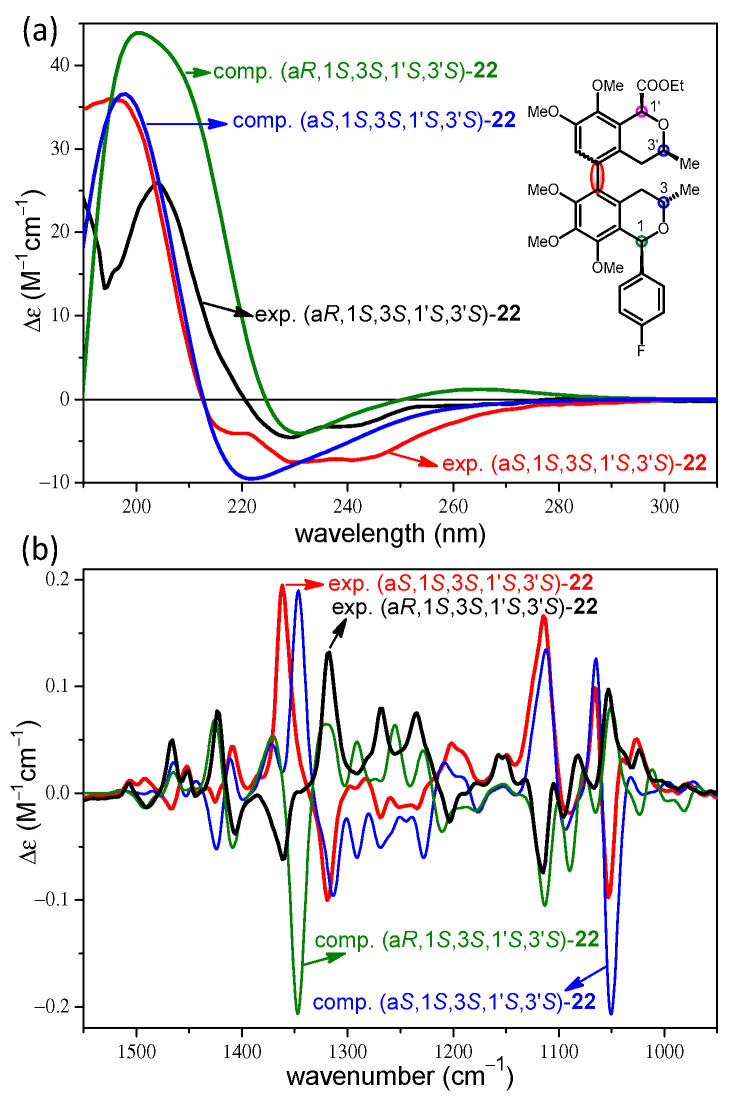
(**a**) Comparison of the experimental ECD and (**b**) VCD spectra of atropodiastereomeric (a*R*,1*S*,3*S*,1′*S*,3′*S*)-**22** (black) and (a*S*,1*S*,3*S*,1′*S*,3′*S*)-**22** (red) with the corresponding computed B3LYP/TZVP PCM/MeCN ECD or B3LYP/TZVP PCM/CHCl_3_ VCD spectra (olive and blue).

Compared to the stereoisomers of **21**, the atropodiastereomeric (a*R*,1*S*,3*S*,1′*S*,3′*S*)-**22** and (a*S*,1*S*,3*S*,1′*S*,3′*S*)-**22** possessed an additional chirality center at C-1′ with an ethoxycarbonyl substituent and both of them had *cis* relative configuration in the dimethoxyisochroman subunit and *trans* one in the trimethoxyisochroman. The ECD spectra of the (a*R*) and (a*S*) atropodiastereomers of **22** were quite similar, showing negative CEs above 210 nm and an intense positive one below it (Figure 6a). The differences in the ECD spectra in the relative intensities and shape could not be used to assign the axial chirality even with the aid of TDDFT-ECD calculations, which, however, reproduced the experimental ECD curves well.

The experimental VCD spectra of the atropodiastereomeric (a*R*,1*S*,3*S*,1′*S*,3′*S*)-**22** and (a*S*,1*S*,3*S*,1′*S*,3′*S*)-**22** differing only in the axial chirality had near-mirror-image VCD transitions in the 1000–1500 cm^−1^ wavenumber range (Figure 6b). The VCD calculations gave good agreements with the experimental curves, which allowed the determination of the axial chirality. Due to the large similarity with the VCD spectra of the stereoisomers of **21**, the axial chirality of (a*R*)- and (a*S*)-**22** could have been deduced by simple comparison of the characteristic VCD transitions.

**Figure 7:**
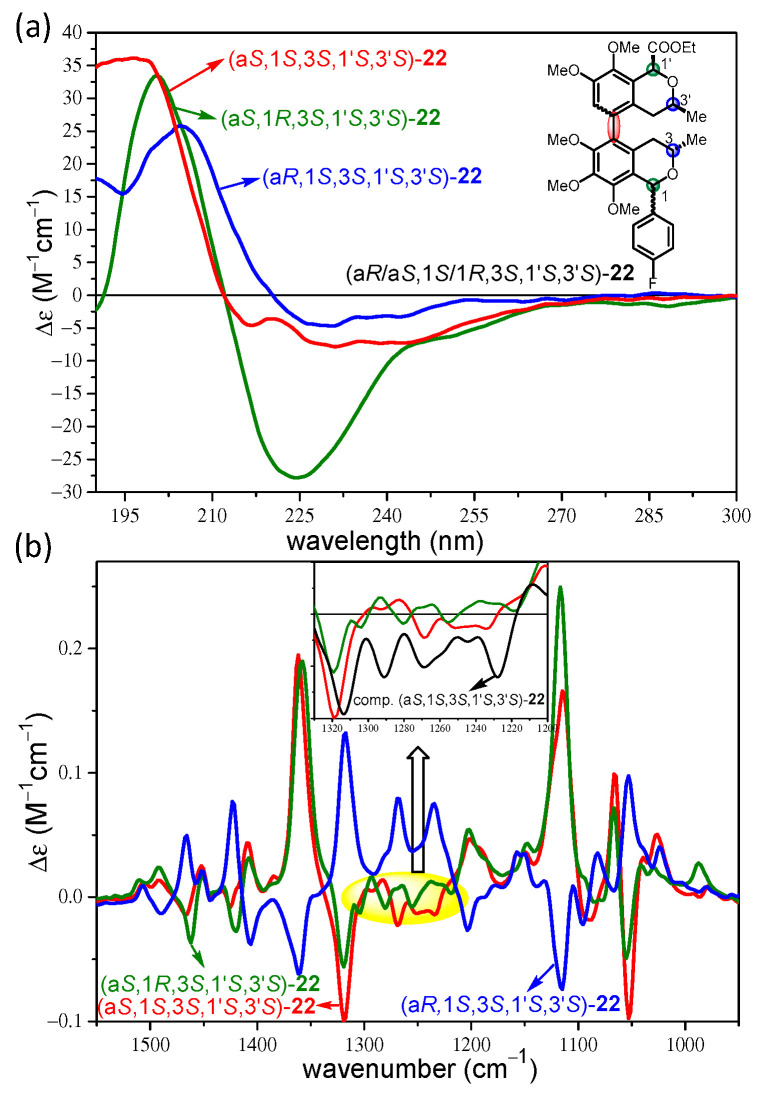
(**a**) Overlapped experimental ECD spectra and (**b**) VCD spectra of (a*R*,1*S*,3*S*,1′*S*,3′*S*)-**22** (blue), (a*S*,1*S*,3*S*,1′*S*,3′*S*)-**22** (red) and (a*S*,1*R*,3*S*,1′*S*,3′*S*)-**22** (olive).

The experimental ECD and VCD spectra of the (a*S*,1*R*,3*S*,1′*S*,3′*S*)-**22** were also compared with those of (a*R*,1*S*,3*S*,1′*S*,3′*S*)-**22** and (a*S*,1*S*,3*S*,1′*S*,3′*S*)-**22** (Figure 7a). The ECD spectra of the C-1 epimeric (a*S*,1*R*,3*S*,1′S,3′*S*)-**22** and (a*S*,1*S*,3*S*,1′*S*,3′*S*)-**22** had similarly negative CEs above and positive ones below 210 nm. However, the broad negative transition of (a*S*,1*R*,3*S*,1′*S*,3′*S*)-**22** (225 nm Δε = −27.82) was much more intense than the corresponding one of (a*S*,1*S*,3*S*,1′*S*,3′*S*)-**22** (231 nm Δε = −7.84), and they had also different shapes and shoulders. These distinct differences can be used to assign the AC of the C-1 chirality centers in the C-1 epimers.

The VCD spectra of the C-1 epimeric (a*S*,1*R*,3*S*,1′*S*,3′*S*)-**22** and (a*S*,1*S*,3*S*,1′*S*,3′*S*)-**22** showed the same signs for most of the CEs in the range of 1000–1500 cm^−1^, reflecting their identical (a*S*) axial chirality, and VCD differences were even more subtle than those in the ECD spectra (Figure 7b). These differences consisted of three weak oppositely signed VCD transitions in the 1220–1290 cm^−1^ range, highlighted in Figure 7b, and the B3LYP/TZVP PCM/CHCl_3_ VCD spectrum of (a*S*,1*S*,3*S*,1′*S*,3′*S*)-**22** reproduced the signs and shapes for one of them, which can be used to distinguish the C-1 epimers of **22** with VCD.

Stereoisomeric pairs with identical axial chirality but different AC of three or four chirality centers such as (a*S*,1*S*,3*S*,1′*S*,3′*S*)-**22**/(a*S*,1*R*,3*R*,1′*R*,3′*R*)-**22**, (a*R*,1*S*,3*S*,1′*S*,3′*S*)-**22**/(a*R*,1*R*,3*R*,1′*R*,3′*R*)-**22** (Figures S179 and S180), (a*R*,1*S*,3*S*,1′*S*,3′*S*)-**22**/(a*R*,1*S*,3*R*,1′*R*,3′*R*)-**22**, (a*S*,1*R*,3*S*,1′*S*,3′*S*)-**22**/(a*S*,1*R*,3*R*,1′*R*,3′*R*)-**22** (Figures S183 and S184) had near-mirror-image ECD, reflecting the different central chirality elements and almost congruent VCD spectra with minor differences in the 1200–1300 cm^−1^ wavelength range.

**Figure 8:**
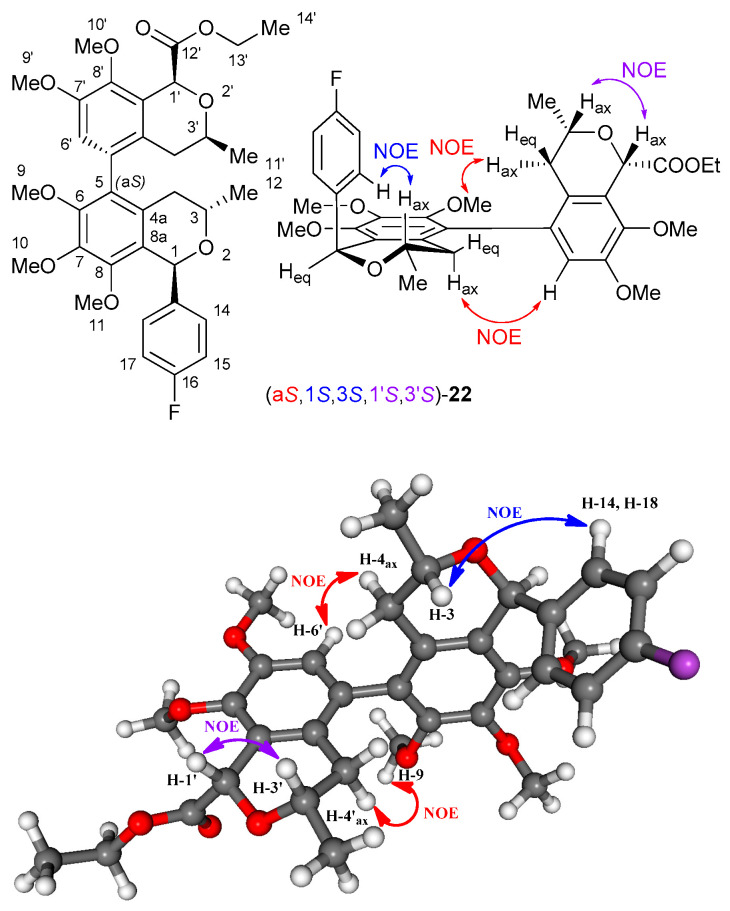
Characteristic NOE correlations of (a*S*,1*S*,3*S*,1′*S*,3′*S*)-**22**, suggesting (1*S*,3*S*,1′*S*,3′*S*) configurations of the isochroman subunits and (a*S*) axial chirality as shown on the lowest-energy computed conformer. The red colored arrows shows the NOE correlations for determining of axial chirality, while blue and purple ones for the central chirality of the two difference isochromans.

In some cases, the ^1^H NMR signals of the diastereotopic 4-H_a_/4-H_b_ and 4′-H_a_/4′-H_b_ protons appeared separately, and their characteristic NOE correlations could be measured with aromatic or methoxy protons of the other isochroman residue, which could be used to determine the axial chirality in the knowledge of the AC of the central chirality elements (Figures S63, S70, S77, S84). As an example, the characteristic NOE correlations are shown for (a*S*,1*S*,3*S*,1′*S*,3′*S*)-**22,** suggesting (a*S*) axial chirality (Figure 8). In contrast to the VCD spectra, this approach could not be applied universally to assign the axial chirality.

**Figure 9:**
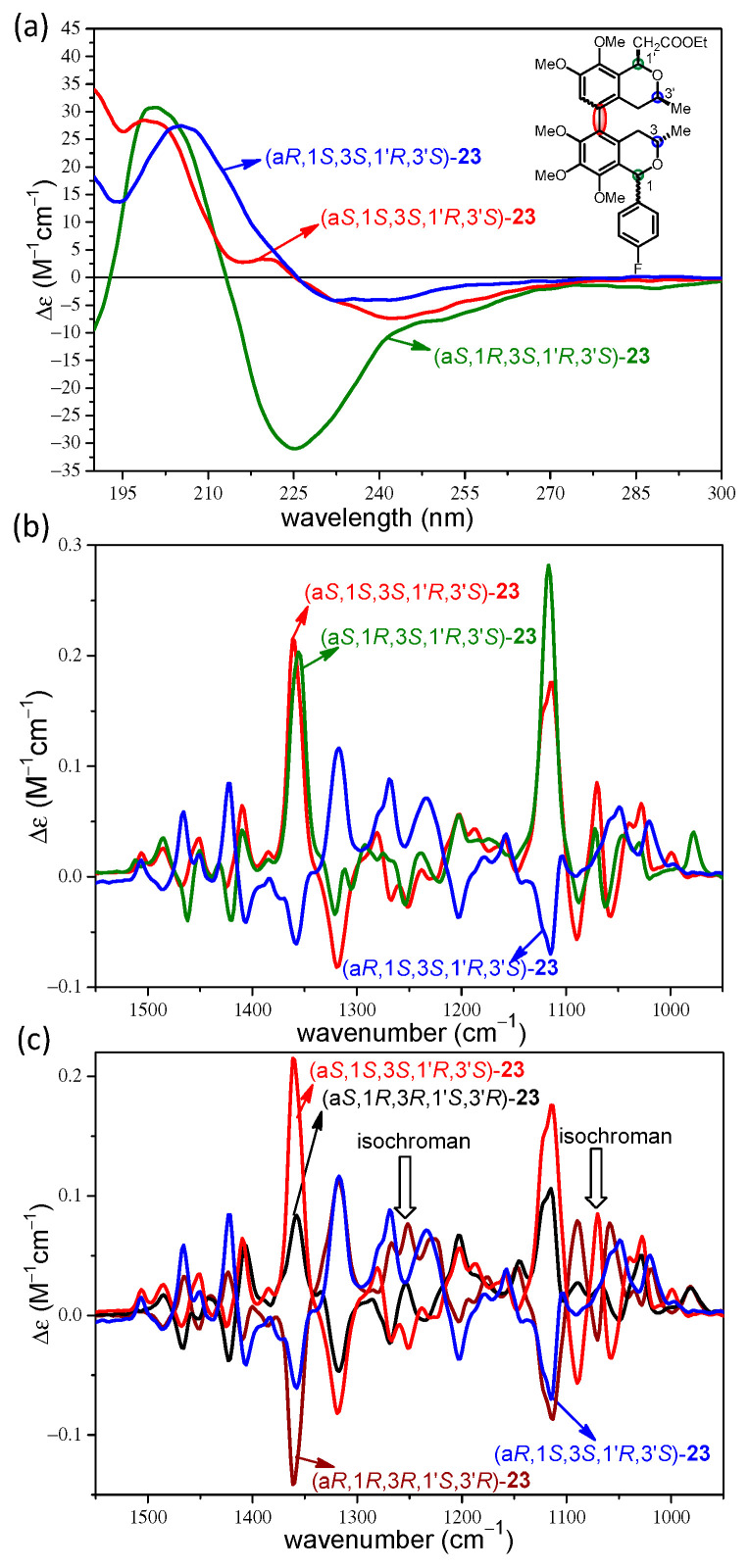
Overlapped experimental (**a**) ECD spectra and (**b**) VCD spectra of (a*R*,1*S*,3*S*,1′*R*,3′*S*)-**23** (blue), (a*S*,1*S*,3*S*,1′*R*,3′*S*)-**23** (red), and (a*S*,1*R*,3*S*,1′*R*,3′*S*)-**23** (olive). (**c**) Overlapped experimental VCD spectra of (a*S*,1*S*,3*S*,1′R,3′*S*)-**23** (red), (a*S*,1*R*,3*R*,1′*S*,3′*R*)-**23** (black), (a*R*,1*R*,3*R*,1′*S*,3′*R*)-**23** (wine), and (a*R*,1*S*,3*S*,1′*R*,3′*S*)-**23** (blue).

The stereoisomers (a*R*,1*S*,3*S*,1′*R*,3′*S*)-**23**, (a*S*,1*S*,3*S*,1′*R*,3′*S*)-**23** and (a*S*,1*R*,3*S*,1′*R*,3′*S*)-**23** had a C-1 ethoxycarbonylmethyl substituent and they were homochiral with the corresponding stereoisomers of **22**. Similarly to the stereoisomers of **22**, the experimental ECD spectra of atropodiastereomeric (a*R*,1*S*,3*S*,1′*R*,3′*S*)-**23**, (a*S*,1*S*,3*S*,1′*R*,3′*S*)-**23** showed the same pattern, by which they could not be distinguished (Figure 9a). In contrast, the C-1 epimeric (a*S*,1*S*,3*S*,1′*R*,3′*S*)-**23** and (a*S*,1*R*,3*S*,1′*R*,3′*S*)-**23** had quite different ECD spectra, since (a*S*,1*R*,3*S*,1′*R*,3′*S*)-**23** showed an intense broad negative band at 225 nm [225 nm (−25.77), 250sh (−6.47)], while (a*S*,1*S*,3*S*,1′*R*,3′*S*)-**23** exhibited a weak positive transition at 220 nm (2.65), a negative trough at 244 nm (−6.93) with a shoulder at 264 nm (−2.30) (Figure 9a).

The VCD spectra of atropodiastereomeric isomers (a*S*,1*S*,3*S*,1′*R*,3′*S*)-**23**/(a*R*,1*S*,3*S*,1′*R*,3′*S*)-**23** and (a*R*,1*R*,3*R*,1′S,3′*R*)-**23**/(a*S*,1*R*,3*R*,1′*S*,3′*R*)-**23** showed opposite CEs for most of the VCD transitions, while the C-1 epimers (a*S*,1*S*,3*S*,1′*R*,3′*S*)-**23** and (a*S*,1*R*,3*S*,1′*R*,3′*S*)-**23** had only minor differences in the 1200–1350 cm^−1^ wavelength range (Figure 9b,c). Stereoisomeric pairs differing in the AC of all the four central chirality elements but having identical axial chirality, represented by the pairs (a*S*,1*R*,3*R*,1′*S*,3′*R*)-**23**/(a*S*,1*S*,3*S*,1′*R*,3′*S*)-**23** and (a*R*,1*S*,3*S*,1′*R*,3′*S*)-**23**/(a*R*,1*R*,3*R*,1′*S*,3′*R*)-**23,** had the same VCD sign for most of the CEs, including the most intense ones reflecting the axial chirality (Figure 9c). However, some distinct VCD transitions could be identified in C–H deformation vibration region with opposite CE for the pairs, which reflected the opposite configuration of the isochroman residues.

**Figure 10:**
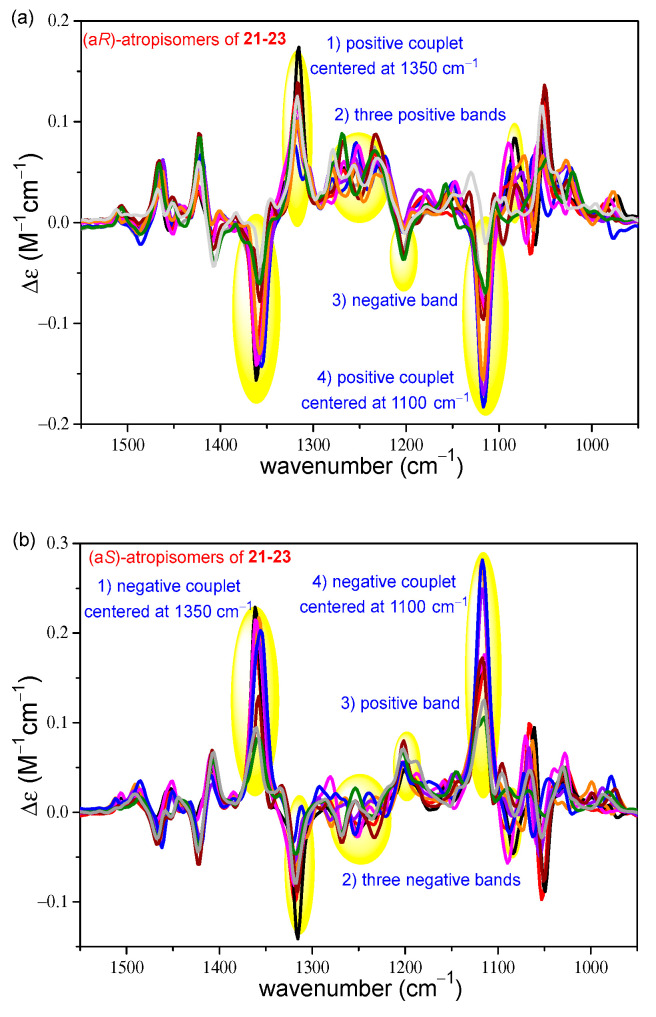
Overlapped ten and nine VCD spectra of the (**a**) (a*R*) and (**b**) (a*S*) atropisomers of **21**–**23** differing in the AC of the central chirality elements and the C-1′ substitution.

The VCD spectra of the (a*R*) and (aS) atropisomers of **21**–**23** were compared (Figure 10), and it was found that regardless of the type of the C-1′ substitution (H, COOEt, or CH_2_COOEt) and AC of the central chirality elements, there are conserved characteristic VCD transitions, which reflect the different axial chirality of the biaryl axis. Four characteristic wavelength ranges are highlighted in Figure 10 but there are additional characteristic bands above 1400 cm^−1^ and below 1100 cm^−1^. For example, two intense positive VCD couplets were identified, centered around 1350 cm^−1^ and 1100 cm^−1^ for the (a*R*) atropisomers of **21**–**23** (Figure 10a), which had opposite signs for the corresponding (a*S*) atropodiastereomers (Figure 10b). The analysis of the computed VCD transitions revealed that almost all the VCD bands had some contributions from different vibrational modes of the biaryl subunit, especially from carbon–carbon stretching vibrations. These contributions are responsible for the mirror-image VCD signals of the atropodiastereomers, while VCD bands reflecting central chirality elements had significant vibrational components from the aliphatic moieties.

**Figure 11:**
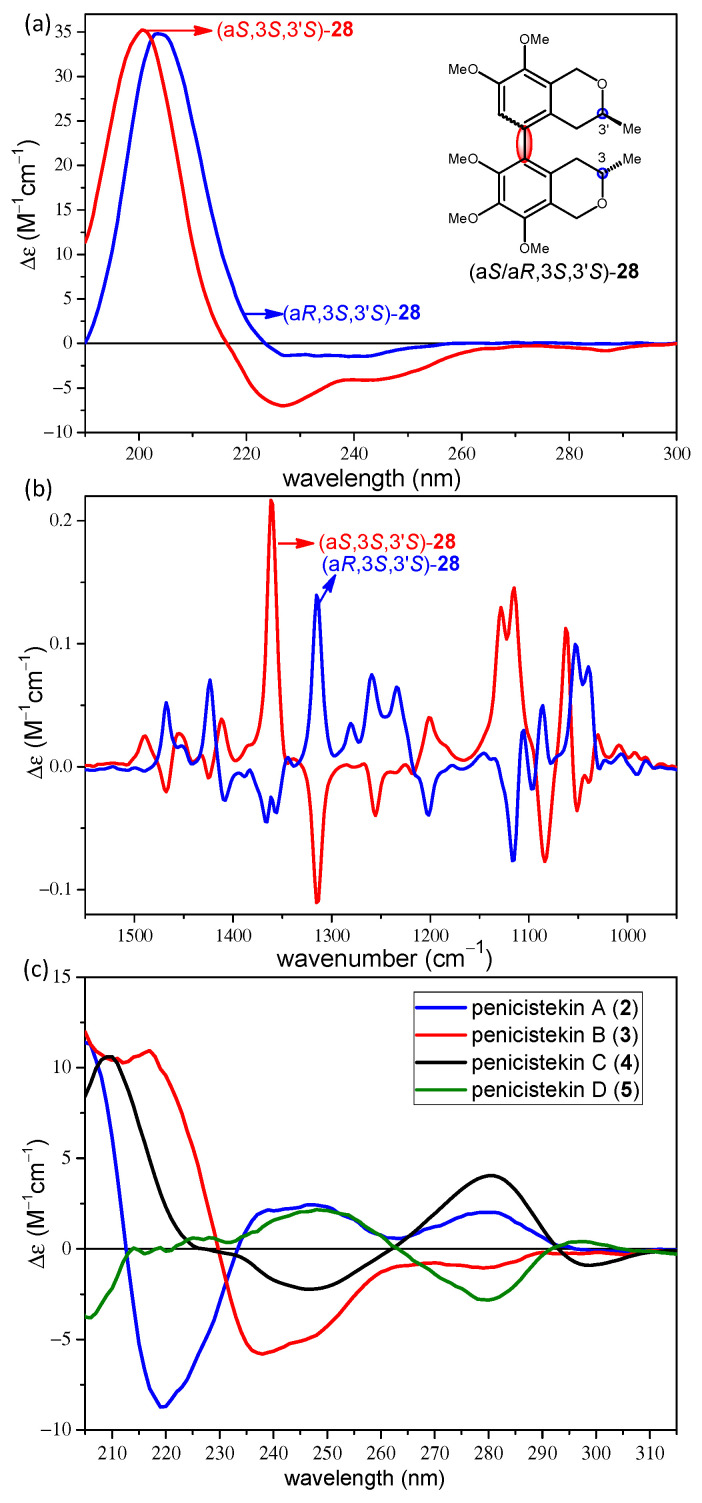
Experimental (**a**) ECD and (**b**) VCD spectra of (a*R*,3*S*,3′*S*)-**28** and (a*S*,3*S*,3′*S*)-**28**. (**c**) Experi-mental ECD spectra of penicisteckins A–D (**2**–**5**).

In order to test the effect of the C-1 aryl substituent, which may have an exciton-coupled ECD interaction with the substituted biphenyl chromophore and influence VCD spectrum as well, ECD and VCD spectra of the atropodiastereomeric (a*R*,3*S*,3′*S*)-**28** and (a*S*,3*S*,3′*S*)-**28** were analyzed (Figure 11). The experimental ECD spectra of the atropodiastereomeric (a*R*,3*S*,3′*S*)-**28** and (a*S*,3*S*,3′*S*)-**28b** showed the same pattern with negative CEs at higher and a positive one at lower wavelength (Figure 11a), which confirmed that it is not the presence of the C-1 aryl group and a C-1 chirality center that is responsible for the near-identical ECD spectra of the atropodiastereomers. ECD spectra were characteristic of the C-3 and C-3′ chirality centers, which determined the preferred helicity of the condensed heteroring.

The (a*S*) and (a*R*) atropodiastereomers of all the prepared axially chiral *bis*-isochromans showed anomalous ECD behavior, since their ECD spectra were not mirror-image curves and they did not reflect the chirality of the stereogenic biaryl axis. The near-identical ECD spectra of atropodiastereomeric (a*R*,3*S*,3′*S*)-**28** and (a*S*,3*S*,3′*S*)-**28** proved that 5,5′ biaryl linkage and the 6,7,8,7′,8′-pentamethoxy substitution pattern were responsible for the ECD discrepancy, which oriented the electric transition moments of the isochroman residues in a way that efficient exciton-coupled interaction was not possible. Thus, the ECD spectrum was governed by the central chirality elements, which is usually not the case in biaryls containing both central and axial chirality elements.

Interestingly, the experimental ECD spectra of the atropoisomeric penicisteckins A,B (**2**,**3**) and penicisteckins C,D (**4**,**5**), biaryl-type hetero- and homodimeric *bis*-isochroman natural products with 7,5′- and 7,7′-linkages differing only in the axial chirality, showed opposite CEs for the corresponding ECD transitions of the atropodiastereomers (Figure 11c). Similarly to **28**, penicisteckins A–D (**2**–**5**) had a methylene group at C-1 and C-1′, but the different ECD behavior was attributed to the different biaryl linkage and aromatic substitution pattern, which enabled an enhanced interaction of the two isochroman moieties.

The VCD spectra of the atropodiastereomers (a*R*,3*S*,3′*S*)-**28** and (a*S*,3*S*,3′*S*)-**28** had oppositely signed CEs for the major transitions, and a simple comparison with those of the stereoisomers of **21**–**23** allowed the assignment of the axial chirality. The (a*S*,3*S*,3′*S*)-**28** stereoisomer had two intense negative VCD couplets centered at 1350 (negative CE at 1315 cm^−1^, positive CE at 1362 cm^−1^) and 1100 cm^−1^ and (negative CE at 1084 cm^−1^ and a positive one at 1115 cm^−1^).

## 3. Materials and Methods

### 3.1. General Information

Chemicals were purchased puriss p.a. from commercial suppliers. Thin-layer chromatography (TLC) was performed on Silica gel 60 F_254_ (Merck) with visualization by UV light (254 nm) and immersing into aqueous solution of sulfuric acidic ammonium molybdate or 5% ethanolic phosphomolybdic acid solution followed by heating. Flash column chromatography was performed on Silica gel 60 (Merck 0.040–0.063 mm). Melting points were determined on a Kofler hot-stage apparatus and were uncorrected. Anhydrous solvents were used for all the reactions and distilled solvents were used as eluent for flash chromatography. HPLC-grade solvents were used for chiral HPLC separations. Preparative chiral HPLC was performed by Agilent 1260 Infinity II apparatus using a Chiralpak IC column. The ^1^H NMR (400 MHz) and ^13^C NMR (100 MHz) spectra were recorded with a Bruker Avance I 400 MHz spectrometer at 298 K. Chemical shifts are referenced to Me_4_Si (0.00 ppm for ^1^H) and to the residual solvent signals (CDCl_3_: 77.16 ppm for ^13^C). Chemical shifts are reported as *δ* in ppm, and ^1^*J*_C,F_, ^2^*J*_H,H_ and ^3^*J*_H,H_ coupling constants in Hz. IR spectra were recorded on a JASCO FT/IR-4100 spectrometer and absorption bands are presented as wavenumber in cm^−1^. Optical rotations were measured at room temperature with a Perkin-Elmer 241 automatic polarimeter (*c* (g/100 mL)). ECD spectra were recorded on a J-810 spectropolarimeter. VCD measurements were performed on a BioTools ChiralIR-2X spectrometer at a resolution of 4 cm^−1^ under ambient temperature for 18 × 3000 scans, respectively. Samples were dissolved in CDCl_3_ and the solutions were placed in a 100 μm BaF_2_ cell. For spectroscopic measurements, spectroscopic grade solvents were used. Diffraction intensity data were collected at room temperature and the in case of (a*S*,1*R*,3*R*,1′*R*,3′*R*)-**22** at low temperature (120 K) using a Bruker-D8 Venture diffractometer (Bruker AXS GmbH, Karlsruhe, Germany) equipped with INCOATEC IμS 3.0 (Incoatec GmbH, Geesthacht, Germany) dual (Cu and Mo) sealed tube microsources and a Photon II Charge-Integrating Pixel Array detector (Bruker AXS GmbH, Karlsruhe, Germany) using Mo Kα (λ = 0.71073 Å) radiation. Electrospray quadrupole time-of-flight HRMS measurements were performed with a MicroTOF-Q type QqTOF MS instrument equipped with an ESI source from Bruker (Bruker Daltoniks, Bremen, Germany).

### 3.2. Computational Section

Mixed torsional/low-frequency mode conformational searches were carried out by means of the Macromodel 10.8.011 software, using the Merck molecular force field (MMFF) with an implicit solvent model for CHCl_3_ [32]. All quantum chemical calculations were carried out with the Gaussian 09 software package [33,34]. The B3LYP (VCD) and ωB97X [35] (ECD) functionals with the TZVP basis set and PCM solvent model for CHCl_3_ (VCD) and MeCN (ECD) were used to reoptimize the initial MMFF geometries. TDDFT-ECD and -OR calculations were performed at the B3LYP/TZVP, BH&HLYP/TZVP, CAM-B3LYP/TZVP, and the PBE0/TZVP levels of theory with the PCM solvent model for MeCN. ECD spectra were generated as sums of Gaussians with 2100–3000 cm^−1^ widths at half-height, using dipole-velocity-computed rotational strength values [36]. VCD calculations were performed at the B3LYP/TZVP PCM/CHCl_3_ level, while the spectra were gained by applying a 8 cm^−1^ half-height width and scaled by a factor of 0.985. Boltzmann distributions were estimated from the B3LYP and ωB97X energies. The MOLEKEL 5.4 software package was used for visualization of the results [37].

### 3.3. X-ray Analysis

X-ray-quality crystals were grown by slow evaporation of the sample dissolved in a mixture of hexane and EtOH. A crystal well-looking in polarized light microscope was fixed under a microscope onto a Mitegen loop using high-density oil. Diffraction intensity data were collected at room temperature and in case of (a*S*,1*R*,3*R*,1′*R*,3′*R*)-**22** at low temperature (120 K) using a Bruker-D8 Venture diffractometer (Bruker AXS GmbH, Karlsruhe, Germany) equipped with INCOATEC IμS 3.0 (Incoatec GmbH, Geesthacht, Germany) dual (Cu and Mo) sealed tube micro sources and a Photon II Charge-Integrating Pixel Array detector (Bruker AXS GmbH, Karlsruhe, Germany) using Mo Kα (λ = 0.71073 Å) radiation. The absolute configurations were assigned relative of the known configuration of C-3 and hence the applied radiation the Flack parameter is meaningless [38], but the assignment is unambiguous. Other spectroscopic methods (VCD, ECD) support the 100% enantiopurity of the samples. High multiplicity data collection and integration were performed using APEX4 (version 2021-4.0, Bruker AXS Inc., 2021, Madison, WI, USA) software. Data reduction and multi-scan absorption correction were performed using SAINT (version 8.40B, Bruker AXS Inc., 2019, Madison, USA). The structures were routinely solved using direct methods and refined on F^2^ using the SHELXL program [39] incorporated into the APEX4 suite. Refinement was performed anisotropically for all non-hydrogen atoms. Hydrogen atoms were placed into geometric positions except for the hydrogen atoms of solvent ethanol molecules in the structure of (a*S*,1*R*,3*R*,1′*R*,3′*R*)-**22**. These atoms could be found on the difference electron density map and the respective O-H distances should be restrained. Multi-scan absorption correction had to be applied because of the irregular shape of the crystals especially in the case of (a*S*,1*R*,3*R*,3′*R*)-**21** (large crystals) and (a*S*,1*R*,3*R*,1′*R*,3′*R*)-**22** (very small needle crystals). In latter case, RIGU command was used to regulate the refinement resulting in significant number of restrains. Further experimental details are shown in Table S1. The CIF file was manually edited using Publcif software [40], while graphics were prepared by using the Mercury program [41]. The results for the X-ray diffraction structure determinations were good enough and acceptable according to the Checkcif functionality of the PLATON software (Utrecht University, Utrecht, The Netherlands) [42]. Structural parameters such as bond length and angle data were in the expected range (Figure S269 and Table S2, Figure S270 and Table S3 as well as Figure S271 and Table S4 for (a*R*,1*S*,3*S*,3′*S*)-**21**, (a*S*,1*R*,3*R*,3′*R*)-**21** and (a*S*,1*R*,3*R*,1′*R*,3′*R*)-**22**, respectively). The solid-state structures are stabilized by van der Waals interactions as well as by weak C-H∙∙O and in case of (a*S*,1*R*,3*R*,1′*R*,3′*R*)-**22** by strong O-H∙∙O hydrogen bonds. As expected, (a*R*,1*S*,3*S*,3′*S*)-**21** and (a*S*,1*R*,3*R*,3′*R*)-**21** crystallized in unit cells of identical geometric parameters. The most interesting structure is (a*S*,1*R*,3*R*,1′*R*,3′*R*)-**22** in which there were three molecules in the asymmetric unit together with two (slightly disordered) solvent ethanol molecules. There were only minor conformational differences among the molecules (Figure S272) and they have the same axial chirality. The supplementary crystallographic data can be obtained free of charge from the Cambridge Crystallographic Data Centre via http://www.ccdc.cam.ac.uk/data_request/cif (accessed on 28 August 2024), using reference deposition number 2249290 for (a*R*,1*S*,3*S*,3′*S*)-**21**, 2249291 for (a*S*,1*R*,3*R*,3′*R*)-**21**, and 2249292 for (a*S*,1*R*,3*R*,1′*R*,3′*R*)-**22**.

## 4. Conclusions

Stereoselective synthesis of heterodimeric 5,5′-linked bis-isochromans, containing a stereogenic *ortho*-trisubstituted biaryl axis and up to four chirality centers, was achieved using a Suzuki–Miyaura biaryl coupling reaction of optically active isochroman and 1-arylpropan-2-ol derivatives. Up to seven stereoisomers of the four target compounds differing in the C-1 and C-1′ substitution were prepared, which were used as model compounds with three isolated blocks of chirality to determine axial and central chirality elements parallel by OR, ECD, and VCD measurements and DFT calculations. Experimental VCD spectra of seven stereoisomers could be compared, which contained three chirality centers and a stereogenic biaryl axis in three isolated blocks of chirality. The effect of different C-1 and C-1′ substitution on the VCD spectra was also checked and characteristic VCD bands were identified, which reported the axial chirality regardless of different substitution pattern and configuration of the chirality centers. The stereochemical analysis was also supported by single-crystal X-ray analysis and NOE measurements. In contrast to related biaryl-type dimers such a penicisteckins A–D, we found that the ECD spectra and OR data of (a*S*) and (a*R*) atropodiastereomers were quite similar and did not reflect the opposite axial chirality. However, the atropodiastereomers showed consistently near-mirror-image VCD curves, allowing the determination of axial chirality with the aid of DFT calculation. The ECD spectra were dependent on the configuration of the chirality centers, which also governed the helicity of the heteroring in the isochroman subunits. Weak VCD transitions were identified in the C–H deformation vibration region, which could serve to distinguish stereoisomers with different central chirality elements. In the chiroptical analysis of our *bis*-isochromans, we demonstrated the complimentary nature of ECD, OR, and VCD methods to determine the parallel AC of central and axial chirality elements in three isolated blocks of chirality.

## Data Availability

The original contributions presented in the study are included in the article/Supplementary Material, further inquiries can be directed to the corresponding author/s.

## Supplementary Materials

The following supporting information can be downloaded at: https://www.mdpi.com/article/10.3390/ijms25179657/s1.
