# Supplementary material for: VCD Analysis of Axial Chirality in Synthetic Stereoisomeric Biaryl-Type bis-Isochroman Heterodimers with Isolated Blocks of Central and Axial Chirality
Source: Int J Mol Sci. 2024 Sep 6;25(17):9657. doi: 10.3390/ijms25179657 (PMC11395685; doi:10.3390/ijms25179657)
Supplement: Supplementary file 1 [file ijms-25-09657-s001.zip › ijms-3157990-supplementary.pdf]

Supporting information

VCD Analysis of Axial Chirality in Synthetic Stereoisomeric Biaryl-Type *bis*-Isochroman Heterodimers with Isolated Blocks of Central and Axial Chirality

Zoltán Czenke [1,2], Attila Mándi [1], Sándor Balázs Király [1], Attila Kiss-Szikszai [1], Anita Kónya-Ábrahám [1], Anna Kurucz-Szabados [1,2], Krisztián Cserepes [1,2], Attila Bényei [3], Changsheng Zhang [4], Máté Kicsák [1,*] and Tibor Kurtán [1,*]

[1] Department of Organic Chemistry, University of Debrecen, P. O. Box 400, 4002 Debrecen, Hungary
[2] Doctoral School of Chemistry, University of Debrecen, Egyetem tér 1., 4032 Debrecen, Hungary
[3] Department of Physical Chemistry, University of Debrecen, Egyetem tér 1., 4032 Debrecen, Hungary
[4] Key Laboratory of Tropical Marine Bio-Resources and Ecology, Guangdong Key Laboratory of Marine Materia Medica, Institutions of South China Sea Ecology and Environmental Engineering, South China Sea Institute of Oceanology, Chinese Academy of Sciences, Guangzhou 510301, China
* Correspondence: kicsak.mate@science.unideb.hu (M.K.); kurtan.tibor@science.unideb.hu (T.K.)

1. Table of Contents

1. Table of Contents 2

1.1. Table of Figures 3

1.2. List of Tables 12

2. General Information 13

2.1. Computational section 13

2.2. Syntheses and characterization of the compounds 14

2.2.1. General procedure for synthesis of chiral non-racemic 1-arylpropan-2-ols 14

2.2.2. General procedure for acetylation of chiral non-racemic 1-arylpropan-2-ols 16

2.2.3. General procedure for the halogenation of 1-arylpropan-2-yl acetates or isochroman derivatives with *N*-halosuccinimides (NXS, X = I or Br) 18

2.2.4. General procedure for Miyaura borylation of chiral non-racemic 1-(2-haloaryl)propan-2-yl acetates 23

2.2.5. General procedure for oxa-Pictet-Spengler reactions of chiral non-racemic 1-arylpropan-2-ol derivatives 25

2.2.6. General procedure for the Suzuki coupling reaction of chiral non-racemic 1-[(2-pinacolatoboryl)aryl]propan-2-yl acetate and 5-iodoisochroman derivatives 49

2.2.7. General procedure for deacetylation of chiral non-racemic 1-[2-(6,7,8-trimethoxy-3-methylisochroman-5-yl)-4,5-dimethoxyphenyl]propan-2-yl acetate derivatives 55

3. Spectra of the compounds 62

3.1. NMR spectra 62

3.2. ECD and VCD spectra 144

3.3. MS spectra 170

3.4. IR spectra 180

4. X-ray analysis 197

4.1. Data of the X-ray analysis 198

5. Structures, populations and Cartesian coordinates of the low-energy DFT conformers 229

6. References 429

1.1. Table of Figures

Figure S1. ^{1}H NMR (400 MHz) spectrum of (*S*)-11 in $CDCl_3$ 62
Figure S2. ^{13}C NMR (100 MHz) spectrum of (*S*)-11 in $CDCl_3$ 62
Figure S3. ^{1}H NMR (400 MHz) spectrum of (*S*)-**12** in $CDCl_3$ 63
Figure S4. ^{13}C NMR (100 MHz) spectrum of (*S*)-**12** in $CDCl_3$ 63
Figure S5. ^{1}H NMR (400 MHz) spectrum of (*S*)-**13** in $CDCl_3$ 64
Figure S6. ^{13}C NMR (100 MHz) spectrum of (*S*)-**13** in $CDCl_3$ 64
Figure S7. ^{1}H NMR (400 MHz) spectrum of (*S*)-**14** in $CDCl_3$ 65
Figure S8. ^{13}C NMR (100 MHz) spectrum of (*S*)-**14** in $CDCl_3$ 65
Figure S9. ^{1}H NMR (400 MHz) spectrum of (*S*)-**8** in $CDCl_3$ 66
Figure S10. ^{13}C NMR (100 MHz) spectrum of (*S*)-**8** in $CDCl_3$ 66
Figure S11. ^{1}H NMR (400 MHz) spectrum of (*S*)-**16** in $CDCl_3$ 67
Figure S12. ^{13}C NMR (100 MHz) spectrum of (*S*)-**16** in $CDCl_3$ 67
Figure S13. ^{1}H NMR (400 MHz) spectrum of *cis*-(1*R*,3*S*)-**17** in $CDCl_3$. 68
Figure S14. ^{13}C NMR (100 MHz) spectrum of *cis*-(1*R*,3*S*)-**17** in $CDCl_3$. 68
Figure S15. ^{1}H-^{1}H COSY NMR (400 MHz) spectrum of *cis*-(1*R*,3*S*)-**17** in $CDCl_3$. 69
Figure S16. ^{1}H-^{13}C HSQC NMR (400 MHz) spectrum of *cis*-(1*R*,3*S*)-**17** in $CDCl_3$. 69
Figure S17. ^{1}H-^{13}C HMBC NMR (400 MHz) spectrum of *cis*-(1*R*,3*S*)-**17** in $CDCl_3$. 70
Figure S18. Characteristic NOE correlation shown on the structure of *cis*-(1*R*,3*S*)-**17** suggesting (1*R*,3*S*) configuration of isochroman unit. 71
Figure S19. ^{1}H-^{1}H NOESY NMR (400 MHz) spectrum of *cis*-(1*R*,3*S*)-**17** in $CDCl_3$. 71
Figure S20. ^{1}H NMR (400 MHz) spectrum of *trans*-(1*S*,3*S*)-**17** in $CDCl_3$. 72
Figure S21. ^{13}C NMR (100 MHz) spectrum of *trans*-(1*S*,3*S*)-**17** in $CDCl_3$. 72
Figure S22. ^{1}H-^{1}H COSY NMR (400 MHz) spectrum of *trans*-(1*S*,3*S*)-**17** in $CDCl_3$. 73
Figure S23. ^{1}H-^{13}C HSQC NMR (400 MHz) spectrum of *trans*-(1*S*,3*S*)-**17** in $CDCl_3$. 73
Figure S24. ^{1}H-^{13}C HMBC NMR (400 MHz) spectrum of *trans*-(1*S*,3*S*)-**17** in $CDCl_3$. 74
Figure S25. Characteristic NOE correlation shown on the structure of *trans*-(1*S*,3*S*)-**17** suggesting (1*S*,3*S*) configuration of isochroman unit. 75
Figure S26. ^{1}H-^{1}H NOESY NMR (400 MHz) spectrum of *trans*-(1*S*,3*S*)-**17** in $CDCl_3$. 75
Figure S27. ^{1}H NMR (400 MHz) spectrum of *cis*-(1*R*,3*S*)-**18** in $CDCl_3$. 76
Figure S28. ^{13}C NMR (100 MHz) spectrum of *cis*-(1*R*,3*S*)-**18** in $CDCl_3$. 76
Figure S29. ^{1}H NMR (400 MHz) spectrum of *trans*-(1*S*,3*S*)-**18** in $CDCl_3$. 77
Figure S30. ^{13}C NMR (100 MHz) spectrum of *trans*-(1*S*,3*S*)-**18** in $CDCl_3$. 77
Figure S31. ^{1}H NMR (400 MHz) spectrum of the mixture of (a*R*,1*S*,3*S*,2'*S*)-**19** and (a*S*,1*S*,3*S*,2'*S*)-**19** in $CDCl_3$. 78
Figure S32. ^{13}C NMR (100 MHz) spectrum of the mixture of (a*R*,1*S*,3*S*,2'*S*)-**19** and (a*S*,1*S*,3*S*,2'*S*)-**19** in $CDCl_3$. 78
Figure S33. ^{1}H NMR (400 MHz) spectrum of (a*S*,1*R*,3*S*,2'*S*)-**19** in $CDCl_3$. 79
Figure S34. ^{13}C NMR (100 MHz) spectrum of (a*S*,1*R*,3*S*,2'*S*)-**19** in $CDCl_3$. 79
Figure S35. ^{1}H-^{1}H COSY NMR (400 MHz) spectrum of (a*S*,1*R*,3*S*,2'*S*)-**19** in $CDCl_3$. 80
Figure S36. ^{1}H-^{13}C HSQC NMR (400 MHz) spectrum of (a*S*,1*R*,3*S*,2'*S*)-**19** in $CDCl_3$. 80
Figure S37. ^{1}H-^{13}C HMBC NMR (400 MHz) spectrum of (a*S*,1*R*,3*S*,2'*S*)-**19** in $CDCl_3$. 81
Figure S38. ^{1}H-^{1}H NOESY NMR (400 MHz) spectrum of (a*S*,1*R*,3*S*,2'*S*)-**19** in $CDCl_3$. 81
Figure S39. ^{1}H NMR (400 MHz) spectrum of (a*R*,1*R*,3*S*,2'*S*)-**19** in $CDCl_3$. 82
Figure S40. ^{13}C NMR (100 MHz) spectrum of (a*R*,1*R*,3*S*,2'*S*)-**19** in $CDCl_3$. 82
Figure S41. ^{1}H NMR (400 MHz) spectrum of (a*S*,1*S*,3*S*,2'*S*)-**20** in $CDCl_3$. 83
Figure S42. ^{13}C NMR (100 MHz) spectrum of (a*S*,1*S*,3*S*,2'*S*)-**20** in $CDCl_3$. 83

Figure S43. ^{1}H-^{1}H COSY NMR (400 MHz) spectrum of (a*S*,1*S*,3*S*,2'*S*)-**20** in $CDCl_3$. 84
Figure S44. ^{1}H-^{13}C HSQC NMR (400 MHz) spectrum of (a*S*,1*S*,3*S*,2'*S*)-**20** in $CDCl_3$. 84
Figure S45. ^{1}H-^{13}C HMBC NMR (400 MHz) spectrum of (a*S*,1*S*,3*S*,2'*S*)-**20** in $CDCl_3$. 85
Figure S46. ^{1}H-^{1}H NOESY NMR (400 MHz) spectrum of (a*S*,1*S*,3*S*,2'*S*)-**20** in $CDCl_3$. 85
Figure S47. ^{1}H NMR (400 MHz) spectrum of (a*R*,1*S*,3*S*,2'*S*)-**20** in $CDCl_3$. 86
Figure S48. ^{13}C NMR (100 MHz) spectrum of (a*R*,1*S*,3*S*,2'*S*)-**20** in $CDCl_3$. 86
Figure S49. ^{1}H-^{1}H COSY NMR (400 MHz) spectrum of (a*R*,1*S*,3*S*,2'*S*)-**20** in $CDCl_3$. 87
Figure S50. ^{1}H-^{13}C HSQC NMR (400 MHz) spectrum of (a*R*,1*S*,3*S*,2'*S*)-**20** in $CDCl_3$. 87
Figure S51. ^{1}H-^{13}C HMBC NMR (400 MHz) spectrum of (a*R*,1*S*,3*S*,2'*S*)-**20** in $CDCl_3$. 88
Figure S52. ^{1}H-^{1}H NOESY NMR (400 MHz) spectrum of (a*R*,1*S*,3*S*,2'*S*)-**20** in $CDCl_3$. 88
Figure S53. ^{1}H NMR (400 MHz) spectrum of (a*S*,1*R*,3*S*,2'*S*)-**20** in $CDCl_3$. 89
Figure S54. ^{13}C NMR (100 MHz) spectrum of (a*S*,1*R*,3*S*,2'*S*)-**20** in $CDCl_3$. 89
Figure S55. ^{1}H-^{1}H COSY NMR (400 MHz) spectrum of (a*S*,1*R*,3*S*,2'*S*)-**20** in $CDCl_3$. 90
Figure S56. ^{1}H NMR (400 MHz) spectrum of (a*R*,1*R*,3*S*,2'*S*)-**20** in $CDCl_3$. 90
Figure S57. ^{13}C NMR (100 MHz) spectrum of (a*R*,1*R*,3*S*,2'*S*)-**20** in $CDCl_3$. 91
Figure S58. ^{1}H NMR (400 MHz) spectrum of (a*S*,1*S*,3*S*,3'*S*)-**21** in $CDCl_3$. 91
Figure S59. ^{13}C NMR (100 MHz) spectrum of (a*S*,1*S*,3*S*,3'*S*)-**21** in $CDCl_3$. 92
Figure S60. ^{1}H-^{1}H COSY NMR (400 MHz) spectrum of (a*S*,1*S*,3*S*,3'*S*)-**21** in $CDCl_3$. 92
Figure S61. ^{1}H-^{13}C HSQC NMR (400 MHz) spectrum of (a*S*,1*S*,3*S*,3'*S*)-**21** in $CDCl_3$. 93
Figure S62. ^{1}H-^{13}C HMBC NMR (400 MHz) spectrum of (a*S*,1*S*,3*S*,3'*S*)-**21** in $CDCl_3$. 93
Figure S63. Characteristic NOE correlations shown on the lowest-energy computed conformer of (a*S*,1*S*,3*S*,3'*S*)-**21** suggesting (1*S*,3*S*) configuration of isochroman subunit and (a*S*) axial chirality.... 94
Figure S64. ^{1}H-^{1}H NOESY NMR (400 MHz) spectrum of (a*S*,1*S*,3*S*,3'*S*)-**21** in $CDCl_3$. 94
Figure S65. ^{1}H NMR (400 MHz) spectrum of (a*R*,1*S*,3*S*,3'*S*)-**21** in $CDCl_3$. 95
Figure S66. ^{13}C NMR (100 MHz) spectrum of (a*R*,1*S*,3*S*,3'*S*)-**21** in $CDCl_3$. 95
Figure S67. ^{1}H-^{1}H COSY NMR (400 MHz) spectrum of (a*R*,1*S*,3*S*,3'*S*)-**21** in $CDCl_3$. 96
Figure S68. ^{1}H-^{13}C HSQC NMR (400 MHz) spectrum of (a*R*,1*S*,3*S*,3'*S*)-**21** in $CDCl_3$. 96
Figure S69. ^{1}H-^{13}C HMBC NMR (400 MHz) spectrum of (a*R*,1*S*,3*S*,3'*S*)-**21** in $CDCl_3$. 97
Figure S70. Characteristic NOE correlations shown on the the lowest-energy computed conformer of (a*R*,1*S*,3*S*,3'*S*)-**21** suggesting (1*S*,3*S*) configuration of isochroman subunit and (a*R*) axial chirality.. 98
Figure S71. ^{1}H-^{1}H NOESY NMR (400 MHz) spectrum of (a*R*,1*S*,3*S*,3'*S*)-**21** in $CDCl_3$. 98
Figure S72. ^{1}H NMR (400 MHz) spectrum of (a*S*,1*R*,3*S*,3'*S*)-**21** in $CDCl_3$. 99
Figure S73. ^{13}C NMR (100 MHz) spectrum of (a*S*,1*R*,3*S*,3'*S*)-**21** in $CDCl_3$. 99
Figure S74. ^{1}H-^{1}H COSY NMR (400 MHz) spectrum of (a*S*,1*R*,3*S*,3'*S*)-**21** in $CDCl_3$. 100
Figure S75. ^{1}H-^{13}C HSQC NMR (400 MHz) spectrum of (a*S*,1*R*,3*S*,3'*S*)-**21** in $CDCl_3$. 100
Figure S76. ^{1}H-^{13}C HMBC NMR (400 MHz) spectrum of (a*S*,1*R*,3*S*,3'*S*)-**21** in $CDCl_3$. 101
Figure S77. Characteristic NOE correlations shown on the lowest-energy computed conformer of (a*S*,1*R*,3*S*,3'*S*)-**21** suggesting (1*R*,3*S*) configuration of isochroman subunit and (a*S*) axial chirality. 102
Figure S78. ^{1}H-^{1}H NOESY NMR (400 MHz) spectrum of (a*S*,1*R*,3*S*,3'*S*)-**21** in $CDCl_3$. 102
Figure S79. ^{1}H NMR (400 MHz) spectrum of (a*R*,1*R*,3*S*,3'*S*)-**21** in $CDCl_3$. 103
Figure S80. ^{13}C NMR (100 MHz) spectrum of (a*R*,1*R*,3*S*,3'*S*)-**21** in $CDCl_3$. 103
Figure S81. ^{1}H-^{1}H COSY NMR (400 MHz) spectrum of (a*R*,1*R*,3*S*,3'*S*)-**21** in $CDCl_3$. 104
Figure S82. ^{1}H-^{13}C HSQC NMR (400 MHz) spectrum of (a*R*,1*R*,3*S*,3'*S*)-**21** in $CDCl_3$. 104
Figure S83. ^{1}H-^{13}C HMBC NMR (400 MHz) spectrum of (a*R*,1*R*,3*S*,3'*S*)-**21** in $CDCl_3$. 105
Figure S84. Characteristic NOE correlations shown on the lowest-energy computed conformer of (a*R*,1*R*,3*S*,3'*S*)-**21** suggesting (1*R*,3*S*) configuration of isochroman subunit and (a*R*) axial chirality. 106
Figure S85. ^{1}H-^{1}H NOESY NMR (400 MHz) spectrum of (a*R*,1*R*,3*S*,3'*S*)-**21** in $CDCl_3$. 106
Figure S86. ^{1}H NMR (400 MHz) spectrum of (a*S*,1*R*,3*S*,1'*S*,3'*S*)-**22** in $CDCl_3$. 107

Figure S87. ^{13}C NMR (100 MHz) spectrum of (a*S*,1*R*,3*S*,1'*S*,3'*S*)-**22** in $CDCl_3$. 107
Figure S88. ^{1}H-^{1}H COSY NMR (400 MHz) spectrum of (a*S*,1*R*,3*S*,1'*S*,3'*S*)-**22** in $CDCl_3$. 108
Figure S89. ^{1}H-^{13}C HSQC NMR (400 MHz) spectrum of (a*S*,1*R*,3*S*,1'*S*,3'*S*)-**22** in $CDCl_3$. 108
Figure S90. ^{1}H-^{13}C HMBC NMR (400 MHz) spectrum of (a*S*,1*R*,3*S*,1'*S*,3'*S*)-**22** in $CDCl_3$. 109
Figure S91. Characteristic NOE correlations shown on the structure of (a*S*,1*R*,3*S*,1'*S*,3'*S*)-**22** suggesting (1*R*,3*S*,1'*S*,3'*S*) configurations of isochroman subunits and (a*S*) axial chirality. 110
Figure S92. ^{1}H-^{1}H NOESY NMR (400 MHz) spectrum of (a*S*,1*R*,3*S*,1'*S*,3'*S*)-**22** in $CDCl_3$. 110
Figure S93. ^{1}H NMR (400 MHz) spectrum of (a*S*,1*S*,3*S*,1'*S*,3'*S*)-**22** in $CDCl_3$. 111
Figure S94. ^{13}C NMR (100 MHz) spectrum of (a*S*,1*S*,3*S*,1'*S*,3'*S*)-**22** in $CDCl_3$. 111
Figure S95. ^{1}H-^{1}H COSY NMR (400 MHz) spectrum of (a*S*,1*S*,3*S*,1'*S*,3'*S*)-**22** in $CDCl_3$. 112
Figure S96. ^{1}H-^{13}C HSQC NMR (400 MHz) spectrum of (a*S*,1*S*,3*S*,1'*S*,3'*S*)-**22** in $CDCl_3$. 112
Figure S97. ^{1}H-^{13}C HMBC NMR (400 MHz) spectrum of (a*S*,1*S*,3*S*,1'*S*,3'*S*)-**22** in $CDCl_3$. 113
Figure S98. Characteristic NOE correlations shown on the lowest-energy computed conformer of (a*S*,1*S*,3*S*,1'*S*,3'*S*)-**22** suggesting (1*S*,3*S*,1'*S*,3'*S*) configurations of isochroman subunits and (a*S*) axial chirality 114
Figure S99. ^{1}H-^{1}H NOESY NMR (400 MHz) spectrum of (a*S*,1*S*,3*S*,1'*S*,3'*S*)-**22** in $CDCl_3$. 114
Figure S100. ^{1}H NMR (400 MHz) spectrum of (a*R*,1*S*,3*S*,1'*S*,3'*S*)-**22** in $CDCl_3$. 115
Figure S101. ^{13}C NMR (100 MHz) spectrum of (a*R*,1*S*,3*S*,1'*S*,3'*S*)-**22** in $CDCl_3$. 115
Figure S102. ^{1}H-^{1}H COSY NMR (400 MHz) spectrum of (a*R*,1*S*,3*S*,1'*S*,3'*S*)-**22** in $CDCl_3$. 116
Figure S103. ^{1}H-^{13}C HSQC NMR (400 MHz) spectrum of (a*R*,1*S*,3*S*,1'*S*,3'*S*)-**22** in $CDCl_3$. 116
Figure S104. ^{1}H-^{13}C HMBC NMR (400 MHz) spectrum of (a*R*,1*S*,3*S*,1'*S*,3'*S*)-**22** in $CDCl_3$. 117
Figure S105. Characteristic NOE correlations shown on the lowest-energy computed conformer of (a*R*,1*S*,3*S*,1'*S*,3'*S*)-**22** suggesting (1*S*,3*S*,1'*S*,3'*S*) configurations of isochroman subunits and (a*R*) axial chirality 118
Figure S106. ^{1}H-^{1}H NOESY NMR (400 MHz) spectrum of (a*R*,1*S*,3*S*,1'*S*,3'*S*)-**22** in $CDCl_3$. 118
Figure S107. ^{1}H NMR (400 MHz) spectrum of (a*S*,1*S*,3*S*,1'*R*,3'*S*)-**23** in $CDCl_3$. 119
Figure S108. ^{13}C NMR (100 MHz) spectrum of (a*S*,1*S*,3*S*,1'*R*,3'*S*)-**23** in $CDCl_3$. 119
Figure S109. ^{1}H-^{1}H COSY NMR (400 MHz) spectrum of (a*S*,1*S*,3*S*,1'*R*,3'*S*)-**23** in $CDCl_3$. 120
Figure S110. ^{1}H-^{13}C HSQC NMR (400 MHz) spectrum of (a*S*,1*S*,3*S*,1'*R*,3'*S*)-**23** in $CDCl_3$. 120
Figure S111. ^{1}H-^{13}C HMBC NMR (400 MHz) spectrum of (a*S*,1*S*,3*S*,1'*R*,3'*S*)-**23** in $CDCl_3$. 121
Figure S112. Characteristic NOE correlations shown on the structure of (a*S*,1*S*,3*S*,1'*R*,3'*S*)-**23** suggesting (1*S*,3*S*,1'*R*,3'*S*) configurations of isochroman subunits and (a*S*) axial chirality. 122
Figure S113. ^{1}H-^{1}H NOESY NMR (400 MHz) spectrum of (a*S*,1*S*,3*S*,1'*R*,3'*S*)-**23** in $CDCl_3$. 122
Figure S114. ^{1}H NMR (400 MHz) spectrum of (a*R*,1*S*,3*S*,1'*R*,3'*S*)-**23** in $CDCl_3$. 123
Figure S115. ^{13}C NMR (100 MHz) spectrum of (a*R*,1*S*,3*S*,1'*R*,3'*S*)-**23** in $CDCl_3$. 123
Figure S116. ^{1}H-^{1}H COSY NMR (400 MHz) spectrum of (a*R*,1*S*,3*S*,1'*R*,3'*S*)-**23** in $CDCl_3$. 124
Figure S117. ^{1}H-^{13}C HSQC NMR (400 MHz) spectrum of (a*R*,1*S*,3*S*,1'*R*,3'*S*)-**23** in $CDCl_3$. 124
Figure S118. ^{1}H-^{13}C HMBC NMR (400 MHz) spectrum of (a*R*,1*S*,3*S*,1'*R*,3'*S*)-**23** in $CDCl_3$. 125
Figure S119. Characteristic NOE correlations shown on the structure of (a*R*,1*S*,3*S*,1'*R*,3'*S*)-**23** suggesting (1*S*,3*S*,1'*R*,3'*S*) configurations of isochroman subunits and (a*R*) axial chirality. 126
Figure S120. ^{1}H-^{1}H NOESY NMR (400 MHz) spectrum of (a*R*,1*S*,3*S*,1'*R*,3'*S*)-**23** in $CDCl_3$. 126
Figure S121. ^{1}H NMR (400 MHz) spectrum of (a*S*,1*R*,3*S*,1'*R*,3'*S*)-**23** in $CDCl_3$. 127
Figure S122. ^{13}C NMR (100 MHz) spectrum of (a*S*,1*R*,3*S*,1'*R*,3'*S*)-**23** in $CDCl_3$. 127
Figure S123. ^{1}H-^{1}H COSY NMR (400 MHz) spectrum of (a*S*,1*R*,3*S*,1'*R*,3'*S*)-**23** in $CDCl_3$. 128
Figure S124. ^{1}H-^{13}C HSQC NMR (400 MHz) spectrum of (a*S*,1*R*,3*S*,1'*R*,3'*S*)-**23** in $CDCl_3$. 128
Figure S125. ^{1}H-^{13}C HMBC NMR (400 MHz) spectrum of (a*S*,1*R*,3*S*,1'*R*,3'*S*)-**23** in $CDCl_3$. 129
Figure S126. Characteristic NOE correlations shown on the structure of (a*S*,1*R*,3*S*,1'*R*,3'*S*)-**23** suggesting (1*R*,3*S*,1'*R*,3'*S*) configurations of isochroman subunits and (a*S*) axial chirality. 130

Figure S127. ^{1}H-^{1}H NOESY NMR (400 MHz) spectrum of (a*S*,1*R*,3*S*,1'*R*,3'*S*)-**23** in $CDCl_3$........... 130
Figure S128. ^{1}H NMR (400 MHz) spectrum of (*S*)-**24** in $CDCl_3$.. 131
Figure S129. ^{13}C NMR (100 MHz) spectrum of (*S*)-**24** in $CDCl_3$.. 131
Figure S130. ^{1}H NMR (400 MHz) spectrum of (*S*)-**25** in $CDCl_3$.. 132
Figure S131. ^{13}C NMR (100 MHz) spectrum of (*S*)-**25** in $CDCl_3$.. 132
Figure S132. ^{1}H NMR (400 MHz) spectrum of the mixture of (a*R*,3*S*,2'*S*)-**26** and (a*S*,3*S*,2'*S*)-**26** in $CDCl_3$. 133
Figure S133. ^{13}C NMR (100 MHz) spectrum of the mixture of (a*R*,3*S*,2'*S*)-**26** and (a*S*,3*S*,2'*S*)-**26** in $CDCl_3$. 133
Figure S134. ^{1}H-^{1}H COSY NMR (400 MHz) spectrum of the mixture of (a*R*,3*S*,2'*S*)-**26** and (a*S*,3*S*,2'*S*)-**26** in $CDCl_3$. 134
Figure S135. ^{1}H-^{13}C HSQC NMR (400 MHz) spectrum of the mixture of (a*R*,3*S*,2'*S*)-**26** and (a*S*,3*S*,2'*S*)-**26** in $CDCl_3$. 134
Figure S136. ^{1}H-^{13}C HMBC NMR (400 MHz) spectrum of the mixture of (a*R*,3*S*,2'*S*)-**26** and (a*S*,3*S*,2'*S*)-**26** in $CDCl_3$. 135
Figure S137. ^{1}H NMR (400 MHz) spectrum of the mixture of (a*R*,3*S*,2'*S*)-**27** and (a*S*,3*S*,2'*S*)-**27** in $CDCl_3$. 135
Figure S138. ^{13}C NMR (100 MHz) spectrum of the mixture of (a*R*,3*S*,2'*S*)-**27** and (a*S*,3*S*,2'*S*)-**27** in $CDCl_3$. 136
Figure S139. ^{1}H NMR (400 MHz) spectrum of (a*R*,3*S*,3'*S*)-**28** in $CDCl_3$. 136
Figure S140. ^{13}C NMR (100 MHz) spectrum of (a*R*,3*S*,3'*S*)-**28** in $CDCl_3$. 137
Figure S141. ^{1}H-^{1}H COSY NMR (400 MHz) spectrum of (a*R*,3*S*,3'*S*)-**28** in $CDCl_3$. 137
Figure S142. ^{1}H-^{13}C HSQC NMR (400 MHz) spectrum of (a*R*,3*S*,3'*S*)-**28** in $CDCl_3$. 138
Figure S143. ^{1}H-^{13}C HMBC NMR (400 MHz) spectrum of (a*R*,3*S*,3'*S*)-**28** in $CDCl_3$. 138
Figure S144. Characteristic NOE correlations shown on the structure of (a*R*,3*S*,3'*S*)-**28** suggesting (a*R*) axial chirality. 139
Figure S145. ^{1}H-^{1}H NOESY NMR (400 MHz) spectrum of (a*R*,3*S*,3'*S*)-**28** in $CDCl_3$. 139
Figure S146. ^{1}H NMR (400 MHz) spectrum of (a*S*,3*S*,3'*S*)-**28** in $CDCl_3$... 140
Figure S147. ^{13}C NMR (100 MHz) spectrum of (a*S*,3*S*,3'*S*)-**28** in $CDCl_3$.. 140
Figure S148. ^{1}H-^{1}H COSY NMR (400 MHz) spectrum of (a*S*,3*S*,3'*S*)-**28** in $CDCl_3$. 141
Figure S149. ^{1}H-^{13}C HSQC NMR (400 MHz) spectrum of (a*S*,3*S*,3'*S*)-**28** in $CDCl_3$. 141
Figure S150. ^{1}H-^{13}C HMBC NMR (400 MHz) spectrum of (a*S*,3*S*,3'*S*)-**28** in $CDCl_3$. 142
Figure S151. Characteristic NOE correlation shown on the structure of (a*S*,3*S*,3'*S*)-**28** suggesting (a*S*) axial chirality. 143
Figure S152. ^{1}H-^{1}H NOESY NMR (400 MHz) spectrum of (a*S*,3*S*,3'*S*)-**28** in $CDCl_3$. 143
Figure S153. Experimental ECD spectra of enantiomeric (a*S*,1*S*,3*S*,3'*S*)-**21** (black) and (a*R*,1*R*,3*R*,3'*R*)-**21** (blue) in MeCN.. 144
Figure S154. Experimental VCD spectra of enantiomeric (a*S*,1*S*,3*S*,3'*S*)-**21** (red) and (a*R*,1*R*,3*R*,3'*R*)-**21** (black) in $CDCl_3$. 144
Figure S155. Experimental ECD spectra of enantiomeric (a*R*,1*S*,3*S*,3'*S*)-**21** (black) and (a*S*,1*R*,3*R*,3'*R*)-**21** (blue) in MeCN. 145
Figure S156. Experimental VCD spectra of enantiomeric (a*R*,1*S*,3*S*,3'*S*)-**21** (red) and (a*S*,1*R*,3*R*,3'*R*)-**21** (black) in $CDCl_3$. 145
Figure S157. Experimental ECD spectra of enantiomeric (a*S*,1*R*,3*S*,3'*S*)-**21** (black) and (a*R*,1*S*,3*R*,3'*R*)-**21** (blue) in MeCN. 146
Figure S158. Experimental VCD spectra of enantiomeric (a*S*,1*R*,3*S*,3'*S*)-**21** (red) and (a*R*,1*S*,3*R*,3'*R*)-**21** (black) in $CDCl_3$. 146
Figure S159. Experimental ECD spectrum of (a*R*,1*R*,3*S*,3'*S*)-**21** in MeCN. 147

Figure S160. Experimental VCD spectrum of (a*R*,1*R*,3*S*,3'*S*)-**21** in $CDCl_3$. 147
Figure S161. Overlapped experimental ECD spectra of stereoisomeric (a*S*,1*S*,3*S*,3'*S*)-**21** (red), (a*R*,1*S*,3*S*,3'*S*)-**21** (blue), (a*R*,1*R*,3*R*,3'*R*)-**21** (wine) and (a*S*,1*R*,3*R*,3'*R*)-**21** (black) in MeCN. 148
Figure S162. Overlapped experimental VCD spectra of stereoisomeric (a*S*,1*S*,3*S*,3'*S*)-**21** (red), (a*R*,1*S*,3*S*,3'*S*)-**21** (blue), (a*R*,1*R*,3*R*,3'*R*)-**21** (wine) and (a*S*,1*R*,3*R*,3'*R*)-**21** (black) in $CDCl_3$. 148
Figure S163. Overlapped experimental ECD spectra of stereoisomeric (a*S*,1*R*,3*S*,3'*S*)-**21** (red), (a*R*,1*R*,3*S*,3'*S*)-**21** (blue) and (a*R*,1*S*,3*R*,3'*R*)-**21** (wine) in MeCN. 149
Figure S164. Overlapped experimental VCD spectra of stereoisomeric (a*S*,1*R*,3*S*,3'*S*)-**21** (red), (a*R*,1*R*,3*S*,3'*S*)-**21** (blue) and (a*R*,1*S*,3*R*,3'*R*)-**21** (wine) in $CDCl_3$. 149
Figure S165. Overlapped experimental ECD spectra of stereoisomeric (a*R*,1*S*,3*S*,3'*S*)-**21** (blue), (a*S*,1*R*,3*S*,3'*S*)-**21** (red), (a*S*,1*R*,3*R*,3'*R*)-**21** (black) and (a*R*,1*S*,3*R*,3'*R*)-**21** (wine) in MeCN. 150
Figure S166. Overlapped experimental VCD spectra of stereoisomeric (a*R*,1*S*,3*S*,3'*S*)-**21** (blue), (a*S*,1*R*,3*S*,3'*S*)-**21** (red), (a*S*,1*R*,3*R*,3'*R*)-**21** (black) and (a*R*,1*S*,3*R*,3'*R*)-**21** (wine) in $CDCl_3$. 150
Figure S167. Overlapped experimental ECD spectra of stereoisomeric (a*S*,1*S*,3*S*,3'*S*)-**21** (blue), (a*S*,1*R*,3*S*,3'*S*)-**21** (red), (a*R*,1*R*,3*R*,3'*R*)-**21** (black) and (a*R*,1*S*,3*R*,3'*R*)-**21** (wine) in MeCN. 151
Figure S168. Overlapped experimental VCD spectra of stereoisomeric (a*S*,1*S*,3*S*,3'*S*)-**21** (blue), (a*S*,1*R*,3*S*,3'*S*)-**21** (red), (a*R*,1*R*,3*R*,3'*R*)-**21** (black) and (a*R*,1*S*,3*R*,3'*R*)-**21** (wine) in $CDCl_3$. 151
Figure S169. Overlapped experimental ECD spectra of stereoisomeric (a*R*,1*S*,3*S*,3'*S*)-**21** (blue), (a*R*,1*R*,3*S*,3'*S*)-**21** (red) and (a*S*,1*R*,3*R*,3'*R*)-**21** (black) in MeCN. 152
Figure S170. Overlapped experimental VCD spectra of stereoisomeric (a*R*,1*S*,3*S*,3'*S*)-**21** (blue), (a*R*,1*R*,3*S*,3'*S*)-**21** (red) and (a*S*,1*R*,3*R*,3'*R*)-**21** (black) in $CDCl_3$. 152
Figure S171. Overlapped experimental ECD spectra of stereoisomeric (a*S*,1*S*,3*S*,3'*S*)-**21** (blue), (a*R*,1*R*,3*S*,3'*S*)-**21** (red) and (a*R*,1*R*,3*R*,3'*R*)-**21** (black) in MeCN. 153
Figure S172. Overlapped experimental VCD spectra of stereoisomeric (a*S*,1*S*,3*S*,3'*S*)-**21** (blue), (a*R*,1*R*,3*S*,3'*S*)-**21** (red) and (a*R*,1*R*,3*R*,3'*R*)-**21** (black) in $CDCl_3$. 153
Figure S173. Experimental ECD spectra of enantiomeric (a*S*,1*R*,3*S*,1'*S*,3'*S*)-**22** (black) and (a*R*,1*S*,3*R*,1'*R*,3'*R*)-**22** (blue) in MeCN. 154
Figure S174. Experimental VCD spectra of enantiomeric (a*S*,1*R*,3*S*,1'*S*,3'*S*)-**22** (red) and (a*R*,1*S*,3*R*,1'*R*,3'*R*)-**22** (black) in $CDCl_3$. 154
Figure S175. Experimental ECD spectra of enantiomeric (a*S*,1*S*,3*S*,1'*S*,3'*S*)-**22** (black) and (a*R*,1*R*,3*R*,1'*R*,3'*R*)-**22** (blue) in MeCN. 155
Figure S176. Experimental VCD spectra of enantiomeric (a*S*,1*S*,3*S*,1'*S*,3'*S*)-**22** (red) and (a*R*,1*R*,3*R*,1'*R*,3'*R*)-**22** (black) in $CDCl_3$. 155
Figure S177. Experimental ECD spectra of enantiomeric (a*R*,1*S*,3*S*,1'*S*,3'*S*)-**22** (black) and (a*S*,1*R*,3*R*,1'*R*,3'*R*)-**22** (blue) in MeCN. 156
Figure S178. Experimental VCD spectra of enantiomeric (a*R*,1*S*,3*S*,1'*S*,3'*S*)-**22** (red) and (a*S*,1*R*,3*R*,1'*R*,3'*R*)-**22** (black) in $CDCl_3$. 156
Figure S179. Overlapped experimental ECD spectra of stereoisomeric (a*S*,1*S*,3*S*,1'*S*,3'*S*)-**22** (blue), (a*R*,1*S*,3*S*,1'*S*,3'*S*)-**22** (red), (a*R*,1*R*,3*R*,1'*R*,3'*R*)-**22** (black) and (a*S*,1*R*,3*R*,1'*R*,3'*R*)-**22** (wine) in MeCN. 157
Figure S180. Overlapped experimental VCD spectra of stereoisomeric (a*S*,1*S*,3*S*,1'*S*,3'*S*)-**22** (blue), (a*R*,1*S*,3*S*,1'*S*,3'*S*)-**22** (red), (a*R*,1*R*,3*R*,1'*R*,3'*R*)-**22** (black) and (a*S*,1*R*,3*R*,1'*R*,3'*R*)-**22** (wine) in $CDCl_3$. 157
Figure S181. Overlapped experimental ECD spectra of stereoisomeric (a*S*,1*S*,3*S*,1'*S*,3'*S*)-**22** (blue), (a*S*,1*R*,3*S*,1'*S*,3'*S*)-**22** (red), (a*R*,1*R*,3*R*,1'*R*,3'*R*)-**22** (black) and (a*R*,1*S*,3*R*,1'*R*,3'*R*)-**22** (wine) in MeCN. 158

Figure S182. Overlapped experimental VCD spectra of stereoisomeric (a*S*,1*S*,3*S*,1'*S*,3'*S*)-**22** (blue), (a*S*,1*R*,3*S*,1'*S*,3'*S*)-**22** (red), (a*R*,1*R*,3*R*,1'*R*,3'*R*)-**22** (black) and (a*R*,1*S*,3*R*,1'*R*,3'*R*)-**22** (wine) in $CDCl_3$. 158
Figure S183. Overlapped experimental ECD spectra of stereoisomeric (a*R*,1*S*,3*S*,1'*S*,3'*S*)-**22** (blue), (a*S*,1*R*,3*S*,1'*S*,3'*S*)-**22** (red), (a*S*,1*R*,3*R*,1'*R*,3'*R*)-**22** (black) and (a*R*,1*S*,3*R*,1'*R*,3'*R*)-**22** (wine) in MeCN. 159
Figure S184. Overlapped experimental VCD spectra of stereoisomeric (a*R*,1*S*,3*S*,1'*S*,3'*S*)-**22** (blue), (a*S*,1*R*,3*S*,1'*S*,3'*S*)-**22** (red), (a*S*,1*R*,3*R*,1'*R*,3'*R*)-**22** (black) and (a*R*,1*S*,3*R*,1'*R*,3'*R*)-**22** (wine) in $CDCl_3$. 159
Figure S185. Experimental ECD spectra of enantiomeric (a*S*,1*S*,3*S*,1'*R*,3'*S*)-**23** (black) and (a*R*,1*R*,3*R*,1'*S*,3'*R*)-**23** (blue) in MeCN. 160
Figure S186. Experimental VCD spectra of enantiomeric (a*S*,1*S*,3*S*,1'*R*,3'*S*)-**23** (red) and (a*R*,1*R*,3*R*,1'*S*,3'*R*)-**23** (black) in $CDCl_3$. 160
Figure S187. Experimental ECD spectra of enantiomeric (a*R*,1*S*,3*S*,1'*R*,3'*S*)-**23** (black) and (a*S*,1*R*,3*R*,1'*S*,3'*R*)-**23** (blue) in MeCN. 161
Figure S188. Experimental VCD spectra of enantiomeric (a*R*,1*S*,3*S*,1'*R*,3'*S*)-**23** (red) and (a*S*,1*R*,3*R*,1'*S*,3'*R*)-**23** (black) in $CDCl_3$. 161
Figure S189. Experimental ECD spectra of enantiomeric (a*S*,1*R*,3*S*,1'*R*,3'*S*)-**23** (black) and (a*R*,1*S*,3*R*,1'*S*,3'*R*)-**23** (blue) in MeCN. 162
Figure S190. Experimental VCD spectra of enantiomeric (a*S*,1*R*,3*S*,1'*R*,3'*S*)-**23** (red) and (a*R*,1*S*,3*R*,1'*S*,3'*R*)-**23** (black) in $CDCl_3$. 162
Figure S191. Overlapped experimental ECD spectra of stereoisomeric (a*S*,1*S*,3*S*,1'*R*,3'*S*)-**23** (blue), (a*R*,1*S*,3*S*,1'*R*,3'*S*)-**23** (red), (a*R*,1*R*,3*R*,1'*S*,3'*R*)-**23** (black) and (a*S*,1*R*,3*R*,1'*S*,3'*R*)-**23** (wine) in MeCN. 163
Figure S192. Overlapped experimental VCD spectra of stereoisomeric (a*S*,1*S*,3*S*,1'*R*,3'*S*)-**23** (blue), (a*R*,1*S*,3*S*,1'*R*,3'*S*)-**23** (red), (a*R*,1*R*,3*R*,1'*S*,3'*R*)-**23** (black) and (a*S*,1*R*,3*R*,1'*S*,3'*R*)-**23** (wine) in $CDCl_3$. 163
Figure S193. Overlapped experimental ECD spectra of stereoisomeric (a*S*,1*R*,3*S*,1'*R*,3'*S*)-**23** (blue), (a*S*,1*S*,3*S*,1'*R*,3'*S*)-**23** (red), (a*R*,1*S*,3*R*,1'*S*,3'*R*)-**23** (black) and (a*R*,1*R*,3*R*,1'*S*,3'*R*)-**23** (wine) in MeCN. 164
Figure S194. Overlapped experimental VCD spectra of stereoisomeric (a*S*,1*R*,3*S*,1'*R*,3'*S*)-**23** (blue), (a*S*,1*S*,3*S*,1'*R*,3'*S*)-**23** (red), (a*R*,1*S*,3*R*,1'*S*,3'*R*)-**23** (black) and (a*R*,1*R*,3*R*,1'*S*,3'*R*)-**23** (wine) in $CDCl_3$. 164
Figure S195. Overlapped experimental ECD spectra of stereoisomeric (a*S*,1*R*,3*S*,1'*R*,3'*S*)-**23** (blue), (a*R*,1*S*,3*S*,1'*R*,3'*S*)-**23** (red), (a*R*,1*S*,3*R*,1'*S*,3'*R*)-**23** (black) and (a*S*,1*R*,3*R*,1'*S*,3'*R*)-**23** (wine) in MeCN. 165
Figure S196. Overlapped experimental VCD spectra of stereoisomeric (a*S*,1*R*,3*S*,1'*R*,3'*S*)-**23** (blue), (a*R*,1*S*,3*S*,1'*R*,3'*S*)-**23** (red), (a*R*,1*S*,3*R*,1'*S*,3'*R*)-**23** (black) and (a*S*,1*R*,3*R*,1'*S*,3'*R*)-**23** (wine) in $CDCl_3$. 165
Figure S197. Overlapped experimental VCD spectra of stereoisomeric (a*R*,1*R*,3*R*,3'*R*)-**21** (black), (a*R*,1*S*,3*R*,3'*R*)-**21** (orange), (a*R*,1*S*,3*R*,1'*R*,3'*R*)-**22** (violet), (a*R*,1*R*,3*R*,1'*R*,3'*R*)-**22** (red), (a*R*,1*S*,3*R*,1'*S*,3'*R*)-**23** (blue) and (a*R*,1*R*,3*R*,1'*S*,3'*R*)-**23** (magenta) in $CDCl_3$. 166
Figure S198. Overlapped experimental VCD spectra of stereoisomeric (a*R*,1*S*,3*S*,3'*S*)-**21** (blue), (a*R*,1*R*,3*S*,3'*S*)-**21** (black), (a*R*,1*S*,3*S*,1'*S*,3'*S*)-**22** (red) and (a*R*,1*S*,3*S*,1'*R*,3'*S*)-**23** (magenta) in $CDCl_3$. 166
Figure S199. Overlapped experimental VCD spectra of stereoisomeric (a*S*,1*S*,3*S*,3'*S*)-**21** (black), (a*S*,1*R*,3*S*,3'*S*)-**21** (orange), (a*S*,1*R*,3*S*,1'*S*,3'*S*)-**22** (violet), (a*S*,1*S*,3*S*,1'*S*,3'*S*)-**22** (red), (a*S*,1*R*,3*S*,1'*R*,3'*S*)-**23** (blue) and (a*S*,1*S*,3*S*,1'*R*,3'*S*)-**23** (magenta) in $CDCl_3$. 167

Figure S200. Overlapped experimental VCD spectra of stereoisomeric (a*S*,1*R*,3*R*,3'*R*)-**21** (blue), (a*S*,1*R*,3*R*,1'*R*,3'*R*)-**22** (red) and (a*S*,1*R*,3*R*,1'*S*,3'*R*)-**23** (black) in $CDCl_3$. 167
Figure S201. Experimental ECD spectrum of (a*R*,3*S*,3'*S*)-**28** in MeCN. 168
Figure S202. Experimental VCD spectrum of (a*R*,3*S*,3'*S*)-**28** in $CDCl_3$. 168
Figure S203. Experimental ECD spectrum of (a*S*,3*S*,3'*S*)-**28** in MeCN. 169
Figure S204. Experimental VCD spectrum of (a*S*,3*S*,3'*S*)-**28** in $CDCl_3$. 169
Figure S205. Experimental ESI-HRMS spectrum of (*S*)-**11**. 170
Figure S206. Experimental ESI-HRMS spectrum of (*S*)-**12**. 170
Figure S207. Experimental ESI-HRMS spectrum of (*S*)-**13**. 170
Figure S208. Experimental ESI-HRMS spectrum of (*S*)-**14**. 171
Figure S209. Experimental ESI-HRMS spectrum of (*S*)-**8**. 171
Figure S210. Experimental ESI-HRMS spectrum of (*S*)-**16**. 171
Figure S211. Experimental ESI-HRMS spectrum of *cis*-(1*R*,3*S*)-**17**. 172
Figure S212. Experimental ESI-HRMS spectrum of *trans*-(1*S*,3*S*)-**17**. 172
Figure S213. Experimental ESI-HRMS spectrum of *cis*-(1*R*,3*S*)-**18**. 172
Figure S214. Experimental ESI-HRMS spectrum of *trans*-(1*S*,3*S*)-**18**. 173
Figure S215. Experimental ESI-HRMS spectrum of the mixture of (a*R*,1*S*,3*S*,2'*S*)-**19** and (a*S*,1*S*,3*S*,2'*S*)-**19**. 173
Figure S216. Experimental ESI-HRMS spectrum of the mixture of (a*R*,1*R*,3*S*,2'*S*)-**19** and (a*S*,1*R*,3*S*,2'*S*)-**19**. 173
Figure S217. Experimental ESI-HRMS spectrum of (a*S*,1*S*,3*S*,2'*S*)-**20**. 174
Figure S218. Experimental ESI-HRMS spectrum of (a*R*,1*S*,3*S*,2'*S*)-**20**. 174
Figure S219. Experimental ESI-HRMS spectrum of (a*S*,1*R*,3*S*,2'*S*)-**20**. 174
Figure S220. Experimental ESI-HRMS spectrum of (a*R*,1*R*,3*S*,2'*S*)-**20**. 174
Figure S221. Experimental ESI-HRMS spectrum of (a*S*,1*S*,3*S*,3'*S*)-**21**. 175
Figure S222. Experimental ESI-HRMS spectrum of (a*R*,1*S*,3*S*,3'*S*)-**21**. 175
Figure S223. Experimental ESI-HRMS spectrum of (a*S*,1*R*,3*S*,3'*S*)-**21**. 175
Figure S224. Experimental ESI-HRMS spectrum of (a*R*,1*R*,3*S*,3'*S*)-**21**. 175
Figure S225. Experimental ESI-HRMS spectrum of (a*S*,1*R*,3*S*,1'*S*,3'*S*)-**22**. 176
Figure S226. Experimental ESI-HRMS spectrum of (a*S*,1*S*,3*S*,1'*S*,3'*S*)-**22**. 176
Figure S227. Experimental ESI-HRMS spectrum of (a*R*,1*S*,3*S*,1'*S*,3'*S*)-**22**. 176
Figure S228. Experimental ESI-HRMS spectrum of (a*S*,1*S*,3*S*,1'*R*,3'*S*)-**23**. 176
Figure S229. Experimental ESI-HRMS spectrum of (a*R*,1*S*,3*S*,1'*R*,3'*S*)-**23**. 177
Figure S230. Experimental ESI-HRMS spectrum of (a*S*,1*R*,3*S*,1'*R*,3'*S*)-**23**. 177
Figure S231. Experimental ESI-HRMS spectrum of (*S*)-**24**. 177
Figure S232. Experimental ESI-HRMS spectrum of (*S*)-**25**. 178
Figure S233. Experimental ESI-HRMS spectrum of the mixture of (a*R*,3*S*,2'*S*)-26 and (a*S*,3*S*,2'*S*)-**26**. 178
Figure S234. Experimental ESI-HRMS spectrum of the mixture of (a*R*,3*S*,2'*S*)-**27** and (a*S*,3*S*,2'*S*)-**27**. 178
Figure S235. Experimental ESI-HRMS spectrum of the mixture of (a*R*,3*S*,3'*S*)-**28** and (a*S*,3*S*,3'*S*)-**28**. 179
Figure S236. Experimental IR spectrum of (*S*)-**11** recorded as KBr disc. 180
Figure S237. Experimental IR spectrum of (*S*)-**12** recorded as KBr disc. 180
Figure S238. Experimental IR spectrum of (*S*)-**13** recorded as KBr disc. 181
Figure S239. Experimental IR spectrum of (*S*)-**14** recorded as KBr disc. 181
Figure S240. Experimental IR spectrum of (*S*)-**8** recorded as KBr disc. 182
Figure S241. Experimental IR spectrum of (*S*)-**16** recorded as KBr disc. 182

Figure S242. Experimental IR spectrum of *cis*-(1*R*,3*S*)-**17** recorded as KBr disc. 183
Figure S243. Experimental IR spectrum of *trans*-(1*S*,3*S*)-**17** recorded as KBr disc.......................... 183
Figure S244. Experimental IR spectrum of *cis*-(1*R*,3*S*)-**18** recorded as KBr disc. 184
Figure S245. Experimental IR spectrum of *trans*-(1*S*,3*S*)-**18** recorded as KBr disc.......................... 184
Figure S246. Experimental IR spectrum of the mixture of (a*R*,1*S*,3*S*,2'*S*)-**19** and (a*S*,1*S*,3*S*,2'*S*)-**19** recorded as KBr disc. 185
Figure S247. Experimental IR spectrum of (a*S*,1*R*,3*S*,2'*S*)-**19** recorded as KBr disc........................ 185
Figure S248. Experimental IR spectrum of (a*R*,1*R*,3*S*,2'*S*)-**19** recorded as KBr disc. 186
Figure S249. Experimental IR spectrum of (a*S*,1*S*,3*S*,2'*S*)-**20** recorded as KBr disc. 186
Figure S250. Experimental IR spectrum of (a*R*,1*S*,3*S*,2'*S*)-**20** recorded as KBr disc........................ 187
Figure S251. Experimental IR spectrum of (a*S*,1*R*,3*S*,2'*S*)-**20** recorded as KBr disc........................ 187
Figure S252. Experimental IR spectrum of (a*R*,1*R*,3*S*,2'*S*)-**20** recorded as KBr disc. 188
Figure S253. Experimental IR spectrum of (a*S*,1*S*,3*S*,3'*S*)-**21** recorded as KBr disc. 188
Figure S254. Experimental IR spectrum of (a*R*,1*S*,3*S*,3'*S*)-**21** recorded as KBr disc........................ 189
Figure S255. Experimental IR spectrum of (a*S*,1*R*,3*S*,3'*S*)-**21** recorded as KBr disc........................ 189
Figure S256. Experimental IR spectrum of (a*R*,1*R*,3*S*,3'*S*)-**21** recorded as KBr disc. 190
Figure S257. Experimental IR spectrum of (a*S*,1*R*,3*S*,1'*S*,3'*S*)-**22** recorded as KBr disc................... 190
Figure S258. Experimental IR spectrum of (a*S*,1*S*,3*S*,1'*S*,3'*S*)-**22** recorded as KBr disc. 191
Figure S259. Experimental IR spectrum of (a*R*,1*S*,3*S*,1'*S*,3'*S*)-**22** recorded as KBr disc.................. 191
Figure S260. Experimental IR spectrum of (a*S*,1*S*,3*S*,1'*R*,3'*S*)-**23** recorded as KBr disc.................. 192
Figure S261. Experimental IR spectrum of (a*R*,1*S*,3*S*,1'*R*,3'*S*)-**23** recorded as KBr disc. 192
Figure S262. Experimental IR spectrum of (a*S*,1*R*,3*S*,1'*R*,3'*S*)-**23** recorded as KBr disc. 193
Figure S263. Experimental IR spectrum of (*S*)-**24** recorded as KBr disc. 193
Figure S264. Experimental IR spectrum of (*S*)-**25** recorded as KBr disc. 194
Figure S265. Experimental IR spectrum of the mixture of (a*R*,3*S*,2'*S*)-**26** and (a*S*,3*S*,2'*S*)-**26** recorded as KBr disc. 194
Figure S266. Experimental IR spectrum of the mixture of (a*R*,3*S*,2'*S*)-**27** and (a*S*,3*S*,2'*S*)-**27** recorded as KBr disc. 195
Figure S267. Experimental IR spectrum of (a*R*,3*S*,3'*S*)-**28** recorded as KBr disc............................. 195
Figure S268. Experimental IR spectrum of (a*S*,3*S*,3'*S*)-**28** recorded as KBr disc. 196
Figure S269. ORTEP view of (a*R*,1*S*,3*S*,3'*S*)-**21** at 50% probability level with numbering scheme. 199
Figure S270. ORTEP view of (a*S*,1*R*,3*R*,3'*R*)-**21** at 50% probability level with numbering scheme. 204
Figure S271. ORTEP view of (a*S*,1*R*,3*R*,1'*R*,3'*R*)-**22** at 50% probability level with numbering scheme. Only one molecule from the asymmetric unit is shown and solvent molecules are omitted for clarity. The numbering of the other molecules were systematically changed; *e.g.* C1 corresponds to C21 and C31. 210
Figure S272. Overlay of the three isomers of in the asymmetric unit of structure (a*S*,1*R*,3*R*,1'*R*,3'*R*)-**22**. 211
Figure S273. Structures and populations of the low-energy ωB97X/TZVP PCM/MeCN conformers (≥ 1%) of (*aS*,1*S*,3*S*,3'*S*)-**21**.. 229
Figure S274. Overlapped structures of the 12 low-energy ωB97X/TZVP PCM/MeCN conformers (≥ 1%) of (*aS*,1*S*,3*S*,3'*S*)-**21**.. 229
Figure S275. Structures and populations of the low-energy ωB97X/TZVP PCM/MeCN conformers (≥ 1%) of (*aR*,1*S*,3*S*,3'*S*)-**21**. 230
Figure S276. Structures and populations of the low-energy ωB97X/TZVP PCM/MeCN conformers (≥ 1%) of (*aS*,1*R*,3*S*,3'*S*)-**21**. 231
Figure S277. Structures and populations of the low-energy ωB97X/TZVP PCM/MeCN conformers (≥ 1%) of (*aR*,1*R*,3*S*,3'*S*)-**21**.. 232

Figure S278. Structures and populations of the low-energy ωB97X/TZVP PCM/MeCN conformers (≥ 1%) of (*aS*,1*S*,3*S*,1'*S*,3'*S*)-**22**. 233
Figure S279. Structures and populations of the low-energy ωB97X/TZVP PCM/MeCN conformers (≥ 1%) of (*aR*,1*S*,3*S*,1'*S*,3'*S*)-**22**. 234
Figure S280. Structures and populations of the low-energy ωB97X/TZVP PCM/MeCN conformers (≥ 1%) of (*aS*,3*S*,3'*S*)-**28**. 235
Figure S281. Structures and populations of the low-energy ωB97X/TZVP PCM/MeCN conformers (≥ 1%) of (*aR*,3*S*,3'*S*)-**28**. 236

1.2. List of Tables

Table S1. Experimental details of the structural elucidation by single crystal X-ray diffraction....... 198
Table S2. Geometric parameters (Å, º) for (a*R*,1*S*,3*S*,3'*S*)-**21**... 199
Table S3. Geometric parameters (Å, º) for (a*S*,1*R*,3*R*,3'*R*)-**21**.. 205
Table S4. Geometric parameters (Å, º) for (a*S*,1*R*,3*R*,1'*R*,3'*R*)-**22**... 211
Table S5. Cartesian coordinates and energies of the low-energy conformers calculated at the ωB97X/TZVP PCM/MeCN level. 237
Table S6. Cartesian coordinates and energies of the low-energy conformers calculated at the B3LYP/TZVP PCM/$CHCl_3$ level.. 354

2. General Information

Chemicals were purchased puriss p.a. from commercial suppliers. Thin-layer chromatography (TLC) was performed on Silica gel 60 F_{254} (Merck) with visualization by UV light (254 nm) and immersing into aqueous solution of sulfuric acidic ammonium molybdate or 5 % ethanolic phosphomolybdic acid solution followed by heating. Flash column chromatography was performed on Silica gel 60 (Merck 0.040-0.063 mm). Melting points were determined on a Kofler hot-stage apparatus and are uncorrected. Anhydrous solvents were used for all the reactions and distilled solvents were used as eluent for flash chromatography. HPLC-grade solvents were used for chiral HPLC separations. Preparative chiral HPLC was performed by Agilent 1260 Infinity II apparatus using Chiralpak IC column. The ^{1}H NMR (400 MHz) and ^{13}C NMR (100 MHz) spectra were recorded with Bruker Avance I 400 MHz spectrometer at 298 K. Chemical shifts are referenced to Me_4Si (0.00 ppm for ^{1}H) and to the residual solvent signals ($CDCl_3$: 77.16 ppm for ^{13}C). Chemical shifts were reported as δ in ppm, and $^{1}J_{C,F}$, $^{2}J_{H,H}$ and $^{3}J_{H,H}$ coupling constants in Hz. IR spectra were recorded on a JASCO FT/IR-4100 spectrometer and absorption bands are presented as wavenumber in cm^{-1}. Optical rotations were measured at room temperature with a Perkin-Elmer 241 automatic polarimeter (*c* [g/100ml]). ECD spectra were recorded on a J-810 spectropolarimeter. VCD measurements were performed on a BioTools ChiralIR-2X spectrometer at a resolution of 4 cm^{-1} under ambient temperature for 18 × 3000 scans, respectively. Sample were dissolved in $CDCl_3$ and the solutions were placed in a 100 µm BaF_2 cell. For spectroscopic measurements spectroscopic grade solvents were used. Diffraction intensity data were collected at room temperature and in case of (a*S*,1*R*,3*R*,1'*R*,3'*R*)-**22** at low temperature (120 K) using a Bruker-D8 Venture diffractometer (Bruker AXS GmbH, Karlsruhe, Germany) equipped with INCOATEC IµS 3.0 (Incoatec GmbH, Geesthacht, Germany) dual (Cu and Mo) sealed tube micro sources and a Photon II Charge-Integrating Pixel Array detector (Bruker AXS GmbH, Karlsruhe, Germany) using Mo Kα (λ = 0.71073 Å) radiation. Electrospray quadrupole time-of-flight HRMS measurements were performed with a MicroTOF-Q type QqTOF MS instrument equipped with an ESI source from Bruker (Bruker Daltoniks, Bremen, Germany).

2.1. Computational section

Mixed torsional/low-frequency mode conformational searches were carried out by means of the Macromodel 10.8.011 software, using the Merck Molecular Force Field (MMFF) with an implicit solvent model for $CHCl_3$ [1]. All quantum chemical calculations were carried out with the Gaussian 09 software package [2-3]. The B3LYP (VCD) and ωB97X [4] (ECD) functionals

with the TZVP basis set and PCM solvent model for $CHCl_3$ (VCD) and MeCN (ECD) were used to re-optimize the initial MMFF geometries. TDDFT-ECD and -OR calculations were performed at the B3LYP/TZVP, BH&HLYP/TZVP, CAM-B3LYP/TZVP and the PBE0/TZVP levels of theory with the PCM solvent model for MeCN. ECD spectra were generated as sums of Gaussians with 2100-3000 cm^{-1} widths at half-height, using dipole-velocity-computed rotational strength values [5]. VCD calculations were performed at the B3LYP/TZVP PCM/$CHCl_3$ level, while the spectra were gained by applying a 8 cm^{-1} half-height width and scaled by a factor of 0.985. Boltzmann distributions were estimated from the B3LYP and ωB97X energies. The MOLEKEL 5.4 software package was used for visualization of the results [6].

2.2. Syntheses and characterization of the compounds

2.2.1. General procedure for synthesis of chiral non-racemic 1-arylpropan-2-ols

The corresponding aryl bromide (1.3 or 1.5 equiv) was dissolved in anhydrous THF (~1 g aryl bromide/10 ml anhydrous THF) under Ar or N_2 athmosphere and the solution was cooled to −80 °C. Then 2.5 M *n*-BuLi in hexane (1.3 or 1.5 equiv.) was added and after stirring for 20 minutes, (*S*)- or (*R*)-propylene oxide (1.0 equiv.) was added and the reaction mixture was stirred for 20 minutes at −80 °C. Next $BF_3 \cdot Et_2O$ (1.1 equiv.) was added to the solution, which was stirred further for 30 minutes at −80 °C. Then the cooling was stopped and a saturated solution of NH_4Cl was added to the reaction mixture. The mixture was stirred for 10 minutes and concentrated in *vacuo*. The suspension was diluted with EtOAc and the organic phase was washed twice with brine. The organic phase was dried over anhydrous Na_2SO_4, filtered, and the solvent was evaporated in *vacuo*. The residue was purified by flash chromatography to yield the optically active 1-arylpropan-2-ol derivative.

(*S*)-1-(3,4-dimethoxyphenyl)propan-2-ol [(*S*)-11] [7]

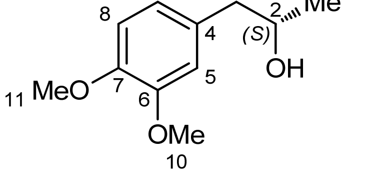

Starting from 4-bromoveratrole **9** (2.00 ml, 3.02 g, 13.90 mmol, 1.3 equiv.): anhydrous THF (30 ml), 2.5 M *n*-BuLi in hexane (5.56 ml, 13.90 mmol, 1.3 equiv.), (*S*)-propylene oxide (749 µl, 621 mg, 10.70 mmol, 1.0 equiv., ≥98.0 *ee*%), $BF_3 \cdot Et_2O$ (1.45 ml, 1.67 g, 11.77 mmol, 1.1

equiv.). (*S*)-**11**: 1.38 g (yield: 66%) yellow syrup; $[\alpha]_D^{20}$ +28 (*c* = 0.50; $CHCl_3$). Flash chromatography (hexane/EtOAc 7:3 → 6:4 → 4:6 → 3:7); R_f = 0.24 (hexane/EtOAc 6:4); ^1H NMR (400 MHz, $CDCl_3$) δ = 6.85 – 6.79 (m, 1H, *H-5* or *H-8* or *H-9*), 6.78 – 6.72 (m, 2H, *H-5*, *H-8*, or *H-5*, *H-9*, or *H-8*, *H-9*), 4.02 – 3.93 (m, 1H, *H-2*), 3.87, 3.85 (2s, 2 x 3H, *H-10*, *H-11*), 2.72 (dd, *J* = 13.6, 4.8 Hz, 1H, *H-1-a*), 2.62 (dd, *J* = 13.6, 8.0 Hz, 1H, *H-1-b*), 1.89 (bs, 1H, O*H*), 1.23 (d, *J* = 6.2 Hz, 3H, *H-3*); ^{13}C NMR (100 MHz, $CDCl_3$) δ = 148.9, 147.7, 131.1 (3C, *C-4*, *C-6*, *C-7*), 121.3, 112.6, 111.4 (3C, *C-5*, *C-8*, *C-9*) 68.9 (1C, *C-2*), 55.9, 55.8 (2C, *C-10*, *C-11*), 45.3 (1C, *C-1*), 22.7 (1C, *C-3*); IR (KBr) ν = 3398 (ν OH), 3061, 3000 (ν Ar =CH), 2963, 2933 (ν_{as} Me, ν_{as} CH_2), 2858, 2841 (ν_s Me, ν_s CH_2), 2007, 1905, 1830, 1789, 1716, 1682 (γ Ar =CH, γ Ar C=C overtone and combination bands), 1604, 1588, 1519, 1464 (ν Ar C=C, δ_{as} Me, β_s CH_2), 1371, 1334 (δ_s Me, δ CH), 1261 (ν_{as} Ar-O-Me), 1119, 1071 (ν C-OH), 1038, 1021 (ν_s Ar-O-Me), 889, 829 (1,2,4-trisubstituated γ_s Ar =CH) cm^{-1}; ESI-TOF-HRMS: *m/z* calculated for $C_{11}H_{16}NaO_3$ $[M+Na]^+$ 219.0992, found 219.0990.

(*R*)-1-(3,4-dimethoxyphenyl)-propan-2-ol [(*R*)-11] [8]

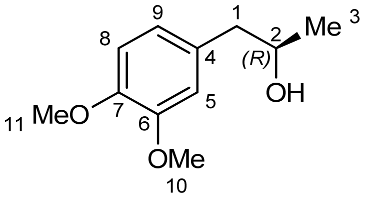

Starting from 4-bromoveratrole **9** (4.31 ml, 6.51 g, 29.98 mmol, 1.5 equiv.): anhydrous THF (65 ml), 2.5 M *n*-BuLi in hexane (11.99 ml, 29.98 mmol, 1.5 equiv.), (*R*)-propylene oxide (1.40 ml, 1.16 g, 19.98 mmol, 1.0 equiv., ≥99.0 *ee*%), $BF_3{\cdot}Et_2O$ (2.71 ml, 3.12 g, 21.98 mmol, 1.1 equiv.). (*R*)-**11**: 2.90 g (yield: 74%) yellow syrup; $[\alpha]_D^{20}$ −36 (*c* = 0.51; $CHCl_3$). Chromatographic and spectral data except for the chiroptical ones were identical with those of (*S*)-**11**.

(*S*)-1-(3,4,5-trimethoxyphenyl)propan-2-ol [(*S*)-16] [9]

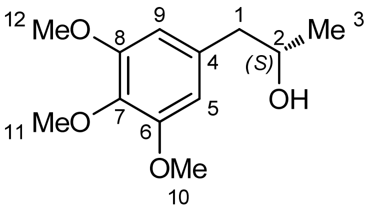

Starting from 5-bromo-1,2,3-trimethoxybenzene **15** (9.52 g, 38.54 mmol, 1.5 equiv.): anhydrous THF (95 ml), 2.5 M *n*-BuLi in hexane (15.42 ml, 38.54 mmol, 1.5 equiv.), (*S*)-propylene oxide (1.80 ml, 1.49 g, 25.69 mmol, 1.0 equiv., ≥98.0 *ee*%), $BF_3{\cdot}Et_2O$ (3.49 ml, 4.01

g, 28.26 mmol, 1.1 equiv.). (*S*)-**16**: 5.21 g (yield: 90%) yellow syrup; $[\alpha]_D^{20}$ +25 (*c* = 0.52; $CHCl_3$). Flash chromatography (hexane/EtOAc 7:3 → 6:4 → 4:6 → 3:7); R_f = 0.33 (hexane/EtOAc 1:1); ^{1}H NMR (400 MHz, $CDCl_3$) δ = 6.43 (s, 2H, *H-5*, *H-9*), 4.06 – 3.97 (m, 1H, *H-2*), 3.85 (s, 6H, *H-10*, *H-12*), 3.83 (s, 3H, *H-11*), 2.73 (dd, *J* = 13.5, 4.6 Hz, 1H, *H-1-a*), 2.61 (dd, *J* = 13.5, 8.2 Hz, 1H, *H-1-b*), 1.96 (bs, 1H, O*H*), 1.26 (d, *J* = 6.2 Hz, 3H, *H-3*); ^{13}C NMR (100 MHz, $CDCl_3$) δ = 153.2, 136.6, 134.4 (4C, *C-4*, *C-6*, *C-7*, *C-8*), 106.3 (2C, *C-5*, *C-9*), 68.8 (1C, *C-2*), 60.9 (1C, *C-11*), 56.1 (2C, *C-10*, *C-12*), 46.2 (1C, *C-1*), 22.9 (1C, *C-3*); IR (KBr) ν = 3444 (ν OH), 2936 (ν_{as} CH_2), 2837 (ν_s CH_2), 2012, 1965 (γ Ar =CH, γ Ar C=C overtone and combination bands), 1589, 1507, 1454 (ν Ar C=C, δ_{as} Me, β_s CH_2), 1371, 1333 (δ_s Me, δ CH), 1237(ν_{as} Ar-O-Me), 1126 (ν C-OH), 1054 (ν_s Ar-O-Me), 852 (1,2,3,5-tetrasubstituated γ_s Ar =CH) cm^{-1}; ESI-TOF-HRMS: *m/z* calculated for $C_{12}H_{18}NaO_4$ $[M+Na]^+$ 249.1097, found 249.1096.

(*R*)-1-(3,4,5-trimethoxy-phenyl)propan-2-ol [(*R*)-16] [9]

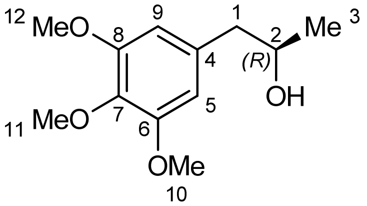

Starting from 5-bromo-1,2,3-trimethoxybenzene **15** (6.35 g, 25.69 mmol, 1.5 equiv.): anhydrous THF (65 ml), 2.5 M *n*-BuLi in hexane (10.28 ml, 25.69 mmol, 1.5 equiv.), (*R*)-propylene oxide (1.20 ml, 1.00 g, 17.13 mmol, 1.0 equiv., ≥99.0 *ee*%), $BF_3 \cdot Et_2O$ (2.33 ml, 2.67 g, 18.84 mmol, 1.1 equiv.). (*R*)-**16**: 3.41 g (yield: 88%) yellow syrup; $[\alpha]_D^{20}$ −31 (*c* = 0.52; $CHCl_3$). Chromatographic and spectral data except for the chiroptical ones were identical with those of (*S*)-**16**.

2.2.2. General procedure for acetylation of chiral non-racemic 1-arylpropan-2-ols

The corresponding 1-arylpropan-2-ol (1.0 equiv.) was dissolved in anhydrous CH_2Cl_2 (20-70 ml) and anhydrous C_5H_5N (3.0 equiv.) and AcCl (2.0 equiv.) were added and the reaction mixture was stirred at room temperature. When the starting material was consumed (2.5-3 hours) on the basis of TLC monitoring, a saturated solution of $NaHCO_3$ was added to the reaction mixture and stirred for 10 minutes. The mixture was diluted with CH_2Cl_2 and the phases were separated in a separatory funnel. The organic phase was washed twice with a 10% solution of $NaHSO_4$, twice with a saturated solution of $NaHCO_3$ and twice with brine. The

organic phase was dried over anhydrous Na_2SO_4, filtered, and the solvent was evaporated in *vacuo*. The residue was purified by flash chromatography to yield the 1-arylpropan-2-yl acetate target derivative.

(*S*)-1-(3,4-dimethoxyphenyl)propan-2-yl acetate [(*S*)-12] [10]

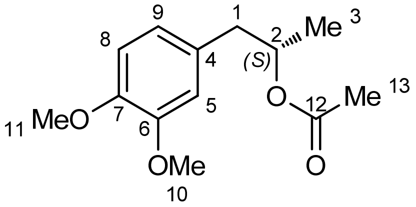

Starting from (*S*)-**11** (1.30 g, 6.63 mmol, 1.0 equiv.): anhydrous CH_2Cl_2 (20 ml), anhydrous C_5H_5N (1.60 ml, 1.57 g, 19.89 mmol, 3.0 equiv.), AcCl (943 ml, 1.04 g, 13.26 mmol, 2.0 equiv.), reaction time: 2.5 hours. (*S*)-**12**: 1.32 g (yield: 84%) yellow syrup; $[\alpha]_D^{20}$ +6 (c = 0.52; $CHCl_3$). Flash chromatography (hexane/EtOAc 8:2 → 7:3); R_f = 0.60 (hexane/EtOAc 6:4); ^1H NMR (400 MHz, $CDCl_3$) δ = 6.81 – 6.77 (m, 1H, *H-5* or *H-8* or *H-9*), 6.76 – 6.71 (m, 2H, *H-5*, *H-8*, or *H-5*, *H-9*, or *H-8*, *H-9*), 5.14 – 5.03 (m, 1H, *H-2*), 3.87, 3.86 (2s, 2 x 3H, *H-10*, *H-11*), 2.88 (dd, J = 13.7, 6.6 Hz, 1H, *H-1-a*), 2.68 (dd, J = 13.7, 6.6 Hz, 1H, *H-1-b*), 2.01 (s, 3H, *H-13*), 1.21 (d, J = 6.3 Hz, 3H, *H-3*); ^{13}C NMR (100 MHz, $CDCl_3$) δ = 170.6 (1C, *C-12*), 148.8, 147.7, 130.3 (3C, *C-4*, *C-6*, *C-7*), 121.5, 112.6, 111.1 (3C, *C-5*, *C-8*, *C-9*), 71.7 (1C, *C-2*), 55.9 (2C, *C-10*, *C-11*), 41.9 (1C, *C-1*), 21.5 (1C, *C-13*), 19.5 (1C, *C-3*); IR (KBr) ν = 2935 (ν_{as} CH_2), 2835 (ν_s CH_2), 2061 (γ Ar =CH, γ Ar C=C overtone and combination bands), 1731 (ν C=O), 1607, 1590, 1517, 1455 (ν Ar C=C, δ_{as} Me, β_s CH_2), 1372, 1331 (δ_s Me, δ CH), 1240 (ν_{as} C-O-C=O, ν_{as} Ar-O-Me), 1029 (ν_s C-O-C=O, ν_s Ar-O-Me), 856, 806 (1,2,4-trisubstituated γ_s Ar =CH) cm^{-1}; ESI-TOF-HRMS: *m/z* calculated for $C_{13}H_{18}NaO_4$ $[M+Na]^+$ 261.1097, found 261.1096.

(*R*)-1-(3,4-dimethoxyphenyl)propan-2-yl acetate [(*R*)-12]

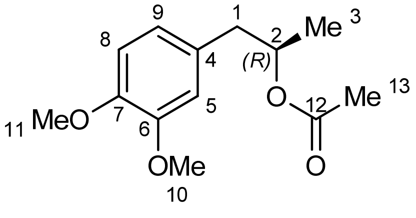

Starting from (*R*)-**11** (3.74 g, 19.07 mmol, 1.0 equiv.): anhydrous CH_2Cl_2 (70 ml), anhydrous C_5H_5N (4.61 ml, 4.53 g, 57.22 mmol, 3.0 equiv.), AcCl (2.71 ml, 2.99 g, 38.15 mmol, 2.0 equiv.), reaction time: 3 hours. (*R*)-**12**: 4.12 g (yield: 91%) yellow syrup; $[\alpha]_D^{20}$ −16 (c = 0.54;

$CHCl_3$). Chromatographic and spectral data except for the chiroptical ones were identical with those of (*S*)-**12**.

2.2.3. General procedure for the halogenation of 1-arylpropan-2-yl acetates or isochroman derivatives with *N*-halosuccinimides (NXS, X = I or Br)

The corresponding 1-arylpropan-2-yl acetate (1.0 equiv.) was dissolved in anhydrous MeCN (5-30 ml), then NIS (1.1 or 1.3 equiv.) and F_3CCOOH (0.3 equiv.) or NBS (1.2 equiv.) were added, and the solution was stirred at room temperature. When the starting material was consumed (1.5-16 hours) on the basis of TLC monitoring, the solvent was evaporated in *vacuo* and the residual solid was dissolved in EtOAc. The organic phase was washed with water, twice with a 10% aqueous solution of $Na_2S_2O_3$ and once with brine. The organic phase was dried over anhydrous Na_2SO_4, filtered, and the solvent was evaporated in *vacuo*. The residue was purified by flash chromatography to yield the 1-(2-halo)arylpropan-2-yl acetate or isochroman target derivatives.

(*S*)-1-(2-iodo-4,5-dimethoxyphenyl)propan-2-yl acetate [(*S*)-13]

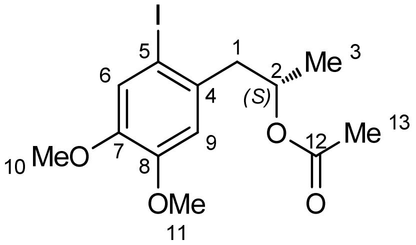

Starting from (*S*)-**12** (1.28 g, 5.39 mmol, 1.0 equiv.): anhydrous MeCN (20 ml), NIS (1.58 g, 7.01 mmol, 1.3 equiv.) and F_3CCOOH (124 μl, 184 mg, 1.62 mmol, 0.3 equiv.), reaction time: 16 hours. (*S*)-**13**: 1.88 g (yield: 96%) yellow syrup; $[\alpha]_D^{20}$ +6 (*c* = 0.55; $CHCl_3$). Flash chromatography (CH_2Cl_2/EtOAc 10:0 → 10:0.05 → 10:0.1); R_f = 0.35 (CH_2Cl_2/EtOAc 10:0.05); ^{1}H NMR (400 MHz, $CDCl_3$) δ = 7.21, 6.74 (2s, 2 x 1H, *H-6*, *H-9*), 5.20 – 5.11 (m, 1H, *H-2*), 3.85 (s, 6H, *H-10*, *H-11*), 2.96 (dd, *J* = 14.0, 7.7 Hz, 1H, *H-1-a*), 2.89 (dd, *J* = 14.0, 5.7 Hz, 1H, *H-1-b*), 1.99 (s, 3H, *H-13*), 1.29 (d, *J* = 6.2 Hz, 3H, *H-3*); ^{13}C NMR (100 MHz, $CDCl_3$) δ = 170.4 (1C, *C-12*), 149.2, 148.3, 133.1 (3C, *C-4*, *C-7*, *C-8*), 121.7, 113.2 (2C, *C-6*, *C-9*), 89.0 (1C, *C-5*), 71.0 (1C, *C-2*), 56.2, 56.0 (2C, *C-10*, *C-11*), 46.0 (1C, *C-1*), 21.4 (1C, *C-13*), 19.8 (1C, *C-3*); IR (KBr) ν = 3078 (ν Ar =CH), 2976, 2933 (ν_{as} Me, ν_{as} CH_2), 2840 (ν_s CH_2), 2048, 1997 (γ Ar =CH, γ Ar C=C overtone and combination bands), 1737 (ν C=O), 1596, 1567, 1507, 1441 (ν Ar C=C, δ_{as} Me, β_s CH_2), 1373, 1334 (δ_s Me, δ CH), 1257 (ν_{as} C-O-C=O, ν_{as} Ar-O-Me), 1051 (ν Ar C-I), 1028 (ν_s C-O-C=O, ν_s Ar-O-Me), 858 (1,2,4,5-tetrasubstituated

γ_s Ar =CH) cm^{-1}; ESI-TOF-HRMS: *m/z* calculated for $C_{13}H_{17}INaO_4$ $[M+Na]^+$ 387.0064, found 387.0064.

(*R*)-1-(2-iodo-4,5-dimethoxyphenyl)propan-2-yl acetate [(*R*)-13]

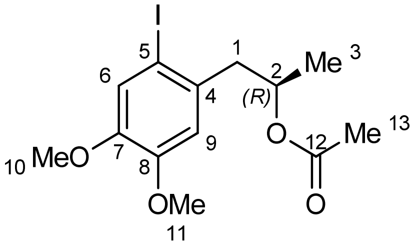

Starting from (*R*)-**12** (1.75 g, 7.35 mmol, 1.0 equiv.): anhydrous MeCN (30 ml), NIS (2.15 g, 9.55 mmol, 1.3 equiv.), F_3CCOOH (169 µl, 251 mg, 2.21 mmol, 0.3 equiv.), reaction time: 16 hours. (*R*)-**13**: 2.47 g (yield: 93%) yellow syrup; $[\alpha]_D^{20}$ −17 (c = 0.53; $CHCl_3$). Chromatographic and spectral data except for the chiroptical ones were identical with those of (*S*)-**13**.

(*S*)-1-(2-bromo-4,5-dimethoxyphenyl)propan-2-yl acetate [(*S*)-14]

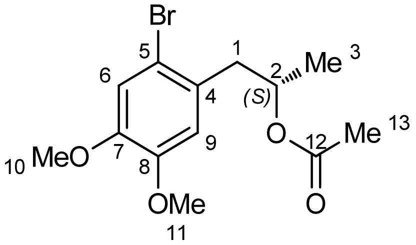

Starting from (*S*)-**12** (1.36 g, 5.71 mmol, 1.0 equiv.): anhydrous MeCN (15 ml), NBS (1.22 g, 6.85 mmol, 1.2 equiv.), reaction time: 16 hours. (*S*)-**14**: 1.76 g (yield: 97%) pale yellow syrup; $[\alpha]_D^{20}$ +8 (c = 0.52; $CHCl_3$). Flash chromatography (CH_2Cl_2/EtOAc 10:0.05 → 10:0.1); R_f = 0.40 (CH_2Cl_2/EtOAc 10:0.05); ^{1}H NMR (400 MHz, $CDCl_3$) δ = 7.00, 6.74 (2s, 2 x 1H, *H-6*, *H-9*), 5.22 – 5.13 (m, 1H, *H-2*), 3.85 (s, 6H, *H-10*, *H-11*), 2.93 (d, J = 6.6 Hz, 2H, *H-1*), 1.99 (s, 3H, *H-13*), 1.27 (d, J = 6.3 Hz, 3H, *H-3*); ^{13}C NMR (100 MHz, $CDCl_3$) δ = 170.5 (1C, *C-12*), 149.2, 148.3, 133.1 (3C, *C-4*, *C-7*, *C-8*), 115.5, 113.9 (2C, *C-6*, *C-9*), 114.9 (1C, *C-5*), 70.8 (1C, *C-2*), 56.2, 56.1 (2C, *C-10*, *C-11*), 41.6 (1C, *C-1*), 21.4 (1C, *C-13*), 19.8 (1C, *C-3*); IR (KBr) ν = 3081 (ν Ar =CH), 2978, 2934 (ν_{as} Me, ν_{as} CH_2), 2842 (ν_s CH_2), 2057 (γ Ar =CH, γ Ar C=C overtone and combination bands), 1736 (ν C=O), 1604, 1573, 1509, 1461 (ν Ar C=C, δ_{as} Me, β_s CH_2), 1373, 1337 (δ_s Me, δ CH), 1244, 1207 (ν_{as} C-O-C=O, ν_{as} Ar-O-Me), 1051, 1031 (ν Ar C-Br, ν_s C-O-C=O, ν_s Ar-O-Me), 859 (1,2,4,5-tetrasubstituated γ_s Ar =CH) cm^{-1}; ESI-TOF-HRMS: *m/z* calculated for $C_{13}H_{17}BrNaO_4$ $[M+Na]^+$ 339.0202, found 339.0201.

(1*R*,3*S*)-1-(4-fluorophenyl)-5-iodo-6,7,8-trimethoxy-3-methylisochroman [*cis*-(1*R*,3*S*)-18]

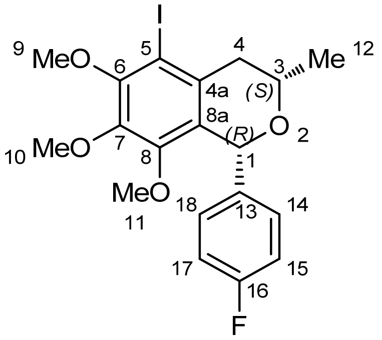

Starting from *cis*-(1*R*,3*S*)-**17** (1.02 g, 3.06 mmol, 1.0 equiv.): anhydrous MeCN (30 ml), NIS (757 mg, 3.36 mmol, 1.1 equiv.), F_3CCOOH (70 µl, 105 mg, 0.92 mmol, 0.3 equiv.), reaction time: 16 hours. *cis*-(1*R*,3*S*)-**18**: 968 mg (yield 69%) white powder; mp 168-172 °C; $[\alpha]_D^{20}$ −5 (*c* = 0.53; $CHCl_3$). Flash chromatography (hexane/CH_2Cl_2 7:3 → 6:4 → 1:1 → 4:6 → 3:7 → 0:10); R_f = 0.22 (hexane/CH_2Cl_2 1:1); ^{1}H NMR (400 MHz, $CDCl_3$) δ = 7.28 – 7.20 (m, 2H, *H-14*, *H-18*), 7.04 – 6.96 (m, 2H, *H-15*, *H-17*), 5.72 (s, 1H, *H-1*), 3.87 (s, 3H, *H-9*), 3.77 (s, 3H , *H-10*), 3.82 – 3.69 (m, 1H, *H-3*), 3.08 (s, 3H, *H-11*), 2.73 (dd, *J* = 16.4, 1.3 Hz, 1H, $H\text{-}4_{eq}$), 2.60 (ddd, *J* = 16.4, 10.6, 1.4 Hz, 1H, $H\text{-}4_{ax}$), 1.38 (d, *J* = 6.2 Hz, 3H, *H-12*); ^{13}C NMR (100 MHz, $CDCl_3$) δ = 162.3 (1C, d, $J_{C\text{-}F}$ = 245.5 Hz, *C-16*), 152.8 (1C, *C-6*), 150.9 (1C, *C-8*), 144.7 (1C, *C-7*), 139.7 (1C, d, $J_{C\text{-}F}$ = 3.0 Hz, *C-13*), 133.9 (1C, *C-4a*), 130.1 (2C, d, $J_{C\text{-}F}$ = 8.1 Hz, *C-14*, *C-18*), 129.2 (1C, *C-8a*), 115.2 (2C, d, $J_{C\text{-}F}$ = 21.4 Hz, *C-15*, *C-17*), 92.5 (1C, *C-5*), 77.5 (1C, *C-1*), 71.0 (1C, *C-3*), 60.8, 60.7, 59.3 (3C, *C-9*, *C-10*, *C-11*), 42.8 (1C, *C-4*), 21.6 (1C, *C-12*); IR (KBr) ν = 3042 (ν Ar =CH), 2974, 2937 (ν_{as} Me, ν_{as} CH_2), 2898, 2858 (ν_s Me, ν_s CH_2, ν CH), 1902 (γ Ar C=C overtone and combination bands), 1604, 1579, 1552, 1513, 1505, 1462 (ν Ar C=C, β_s CH_2, δ_{as} Me), 1385, 1354, 1335, 1304 (δ_s Me, δ CH, γ_s CH_2), 1290, 1274, 1262, 1225, 1153, 1120, 1100 (ν_{as} Ar-O-Me, ν Ar C-F), 1085, 1073, 1051, 1024 (ν_{as} C-O-C, ν Ar C-I, ν_s Ar-O-Me) cm^{-1}; ESI-TOF-HRMS: *m/z* calculated for $C_{19}H_{20}FINaO_4$ $[M+Na]^+$ 481.0283, found 481.0282.

(1*S*,3*R*)-1-(4-fluorophenyl)-5-iodo-6,7,8-trimethoxy-3-methylisochroman [*cis*-(1*S*,3*R*)-18]

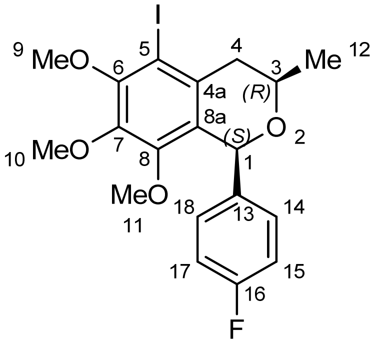

Starting from *cis*-(1*S*,3*R*)-**17** (1.45 g, 4.36 mmol, 1.0 equiv.): anhydrous MeCN (30 ml), NIS (1.08 g, 4.80 mmol, 1.1 equiv.), F_3CCOOH (100 µl, 149 mg, 1.31 mmol, 0.3 equiv.), reaction time: 16 hours. *cis*-(1*S*,3*R*)-**18**: 1.40 g (yield 70%) white powder; mp 168-171 °C; $[\alpha]_D^{20}$ +48 (*c* = 0.52; $CHCl_3$). Chromatographic and spectral data except for the chiroptical ones were identical with those of *cis*-(1*R*,3*S*)-**18**.

(1*S*,3*S*)-1-(4-fluorophenyl)-5-iodo-6,7,8-trimethoxy-3-methylisochroman [*trans*-(1*S*,3*S*)-18]

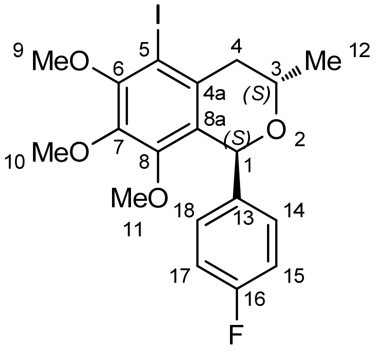

Starting from *trans*-(1*S*,3*S*)-**17** (1.21 g, 3.65 mmol, 1.0 equiv.): anhydrous MeCN (30 ml), NIS (902 mg, 4.01 mmol, 1.1 equiv.), F_3CCOOH (84 µl, 125 mg, 1.09 mmol, 0.3 equiv.), reaction time: 16 hours. *trans*-(1*S*,3*S*)-**18**: 1.54 g (yield: 92%) white solid; mp 55-59 °C; $[\alpha]_D^{20}$ +5 (*c* = 0.53; $CHCl_3$). Flash chromatography (hexane/EtOAc 97:3 → 95:5 → 93:7); R_f = 0.45 (hexane/EtOAc 9:1); ^{1}H NMR (400 MHz, $CDCl_3$) δ = 7.21 – 7.14 (m, 2H, *H-14*, *H-18*), 7.03 – 6.95 (m, 2H, *H-15*, *H-17*), 5.94 (s, 1H, *H-1*), 3.91 (s, 3H, *H-9*), 3.86 (s, 3H, *H-10*), 3.75 – 3.63 (m, 1H, *H-3*), 3.55 (s, 3H, *H-11*), 2.69 (dd, *J* = 17.0, 3.7 Hz, 1H, $H\text{-}4_{eq}$), 2.41 (dd, *J* = 17.0, 10.9 Hz, 1H, $H\text{-}4_{ax}$), 1.23 (d, *J* = 6.1 Hz, 3H, *H-12*); ^{13}C NMR (100 MHz, $CDCl_3$) δ = 162.3 (1C, d, $J_{C\text{-}F}$ = 246.4 Hz, *C-16*), 153.0 (1C, *C-6*), 150.4 (1C, *C-8*), 144.1 (1C, *C-7*), 137.6 (1C, d, $J_{C\text{-}F}$ = 3.0 Hz, *C-13*), 132.9 (1C, *C-4a*), 130.3 (2C, d, $J_{C\text{-}F}$ = 8.0 Hz, *C-14*, *C-18*), 126.7 (1C, *C-8a*), 114.8 (2C, d, $J_{C\text{-}F}$ = 21.2 Hz, *C-15*, *C-17*), 92.8 (1C, *C-5*), 73.3 (1C, *C-1*), 63.8 (1C, *C-3*), 60.8, 60.7, 60.2 (3C, *C-9*, *C-10*, *C-11*), 41.7 (1C, *C-4*), 21.6 (1C, *C-12*); IR (KBr) ν = 3044 (ν Ar

=CH), 2968, 2936 (ν_{as} Me, ν_{as} CH_2), 2890, 2833 (ν_s Me, ν_s CH_2, ν CH), 2021, 1914 (γ Ar C=C overtone and combination bands), 1603, 1557, 1507, 1462 (ν Ar C=C, β_s CH_2, δ_{as} Me), 1383, 1362, 1334 (δ_s Me, δ CH, γ_s CH_2), 1283, 1263, 1225, 1197, 1160, 1141, 1122, 1110 (ν_{as} Ar-O-Me, ν Ar C-F), 1089, 1068, 1047, 1022 (ν_{as} C-O-C, ν Ar C-I, ν_s Ar-O-Me) cm^{-1}; ESI-TOF-HRMS: *m/z* calculated for $C_{19}H_{20}FINaO_4$ $[M+Na]^+$ 481.0283, found 481.0282.

(1*R*,3*R*)-1-(4-fluorophenyl)-5-iodo-6,7,8-trimethoxy-3-methylisochroman [*trans*-(1*R*,3*R*)-18]

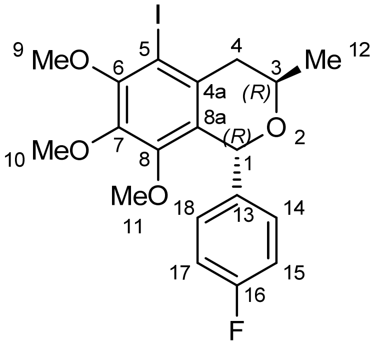

Starting from *trans*-(1*R*,3*R*)-**17** (1.27 g, 3.82 mmol, 1.0 equiv.): anhydrous MeCN (30 ml), NIS (946 mg, 4.20 mmol, 1.1 equiv.), F_3CCOOH (88 µl, 131 mg, 1.15 mmol, 0.3 equiv.), reaction time: 16 hours. *trans*-(1*R*,3*R*)-**18**: 1.63 g (yield: 93%) white solid; mp 57-59 °C; $[\alpha]_D^{20}$ −11 (*c* = 0.53; $CHCl_3$). Chromatographic and spectral data except for the chiroptical ones were identical with those of *trans*-(1*S*,3*S*)-**18**.

(*S*)-5-iodo-6,7,8-trimethoxy-3-methylisochroman [(*S*)-25]

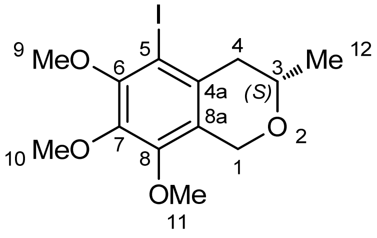

Starting from (*S*)-**24** (556 mg, 2.33 mmol, 1.0 equiv.): anhydrous MeCN (10 ml), NIS (683 mg, 3.03 mmol, 1.3 equiv.), F_3CCOOH (54 µl, 80 mg, 0.70 mmol, 0.3 equiv.) reaction time: 1.5 hours. (*S*)-**25**: 787 mg (yield: 93%) white solid; mp 33-36 °C; $[\alpha]_D^{20}$: +72 (*c* = 0.53; $CHCl_3$). Flash chromatography (hexane/Et_2O 95:5 → 9:1); R_f = 0.47 (hexane/Et_2O 8:2); ^1H NMR (400 MHz, $CDCl_3$) δ = 4.87 (d, *J* = 15.6 Hz, 1H, *H-1-a*), 4.57 (d, *J* = 15.6 Hz, 1H, *H-1-b*), 3.88, 3.86 (2s, 3 x 3H, *H-9*, *H-10*, *H-11*), 3.74 – 3.61 (m, 1H, *H-3*), 2.64 (dd, *J* = 16.7, 2.1 Hz, 1H, *H-4-a*), 2.39 (dd, *J* = 16.8, 10.7 Hz, 1H, *H-4-b*), 1.38 (d, *J* = 6.1 Hz, 3H, *H-12*); ^{13}C NMR (100

MHz, $CDCl_3$) δ = 152.2, 149.7, 143.9, 132.4, 126.3 (5C, *C-4a*, *C-8a*, *C-6*, *C-7*, *C-8*), 92.8 (1C, *C-5*), 71.4 (1C, *C-3*), 64.6 (1C, *C-1*), 61.0, 60.7, 60.5 (3C, *C-9*, *C-10*, *C-11*), 41.6 (1C, *C-4*), 21.5 (1C, *C-12*); IR (KBr) ν = 2963, 2928 (ν_{as} Me, ν_{as} CH_2), 2881, 2837 (ν_s Me, ν_s CH_2), 2018, 1952, 1915, 1893, 1732 (γ Ar C=C overtone and combination bands), 1648, 1588, 1558, 1466, 1416 (ν Ar C=C, β_s CH_2, δ_{as} Me), 1387, 1367, 1350, 1334 (δ_s Me, δ CH, γ_s CH_2), 1261, 1240, 1211 (ν_{as} Ar-O-Me), 1082, 1051, 1025 (ν_{as} C-O-C, ν Ar C-I, ν_s Ar-O-Me) cm^{-1}; ESI-TOF-HRMS: *m/z* calculated for $C_{13}H_{17}INaO_4$ $[M+Na]^+$ 387.0064, found 387.0063.

2.2.4. General procedure for Miyaura borylation of chiral non-racemic 1-(2-haloaryl)propan-2-yl acetates

To the solution of 1-(2-haloaryl)propan-2-yl acetate (1.0 equiv.) in anhydrous DMF (18-37 ml), Ph_3P (0.2 equiv.), $(Ph_3P)_2PdCl_2$ (0.1 equiv.) and anhydrous NaOAc (4.0 equiv.) or annealed KOAc (4.0 equiv.) were added under Ar or N_2 atmosphere, and the solution was stirred for 15 minutes with inert gas bubbling at room temperature. Then B_2Pin_2 (3.0 equiv.) was added to the reaction mixture and the temperature was raised to 150 °C. When the starting material was consumed (1 hour-3 hours) on the basis of TLC monitoring, the reaction mixture was poured on ice and diluted with Et_2O and the mixture was filtered on a short pad of Celite. The Celite was washed with Et_2O and the two layers were separated in a separatory funnel. The aqueous phase was extracted twice with Et_2O and the combined organic layers were washed twice with brine and dried over anhydrous Na_2SO_4, filtered, and concentrated in *vacuo*. The residue was purified by flash chromatography to yield the 1-[2-(pinacolatoboryl)aryl]propan-2-yl acetate target derivative.

(*S*)-1-[4,5-dimethoxy-2-(4,4,5,5-tetramethyl-1,3,2-dioxaborolan-2-yl)phenyl]propan-2-yl acetate [(*S*)-8]

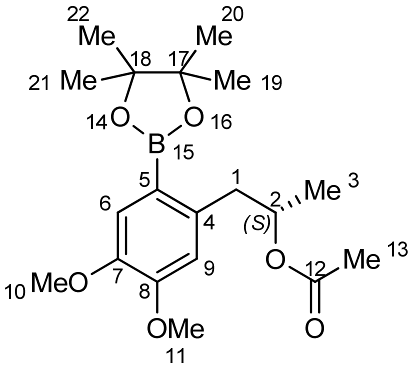

Starting from (*S*)-**13** (939 mg, 2.58 mmol, 1.0 equiv.): anhydrous DMF (20 ml), Ph_3P (135 mg, 0.52 mmol, 0.2 equiv.), $(Ph_3P)_2PdCl_2$ (181 mg, 0.26 mmol, 0.1 equiv.), anhydrous NaOAc (846

mg, 10.31 mmol, 4.0 equiv.), B_2Pin_2 (1.96 g, 7.73 mmol, 3.0 equiv.), reaction time: 1 hour. (*S*)-**8**: 524 mg (yield: 56%) pale yellow syrup.

Starting from (*S*)-**14** (1.76 g, 5.54 mmol, 1.0 equiv.): anhydrous DMF (18 ml), Ph_3P (291 mg, 1.11 mmol, 0.2 equiv.), $(Ph_3P)_2PdCl_2$ (389 mg, 0.55 mmol, 0.1 equiv.), annealed KOAc (2.18 g, 22.16 mmol, 4.0 equiv.), B_2Pin_2 (4.22 g, 16.62 mmol, 3.0 equiv), reaction time: 3 hours. (*S*)-**8**: 1.92 g (yield: 95%) colorless syrup; $[\alpha]_D^{20}$ +14 (*c* = 0.43; $CHCl_3$).

Flash chromatography (hexane/EtOAc 9:1 → 85:15 → 8:2 → 7:3); R_f = 0.43 (hexane/EtOAc 8:2); ^{1}H NMR (400 MHz, $CDCl_3$) δ = 7.27, 6.72 (2s, 2 x 1H, *H-6*, *H-9*), 5.13 – 5.00 (m, 1H, *H-2*), 3.90, 3.88 (2s, 2 x 3H, *H-10*, *H-11*), 3.21 (dd, *J* = 13.2, 5.7 Hz, 1H, *H-1-a*), 3.02 (dd, *J* = 13.2, 7.6 Hz, 1H, *H-1-b*), 1.96 (s, 3H, *H-13*), 1.33 (s, 12H, *H-19*, *H-20*, *H-21*, *H-22*), 1.22 (d, *J* = 6.2 Hz, 3H, *H-3*); ^{13}C NMR (100 MHz, $CDCl_3$) δ = 170.5 (1C, *C-12*), 150.8, 146.8, 138.9 (3C, *C-4*, *C-7*, *C-8*), 118.2, 113.6 (2C, *C-6*, *C-9*), 83.5 (2C, *C-17*, *C-18*), 73.3 (1C, *C-2*), 56.0, 55.8 (2C, *C-10*, *C-11*), 41.2 (1C, *C-1*), 25.0 (4C, *C-19, C-20*, *C-21*, *C-22*), 21.4 (1C, *C-13*), 19.7 (1C, *C-3*); IR (KBr) ν = 2978, 2934 (ν_{as} Me, ν_{as} CH_2), 1736 (ν C=O), 1600, 1571, 1519 (ν Ar C=C), 1371, 1349, 1320 (δ_s Me, δ CH, ν_{as} O-B-O), 1251, 1221, 1204 (ν_{as} C-O-C=O, ν_{as} Ar-O-Me), 1160, 1144 (ν_s O-B-O), 1054 (ν C-O-B, ν_s C-O-C=O, ν_s Ar-O-Me), 860 (1,2,4,5-tetrasubstituated γ_s Ar =CH) cm^{-1}; ESI-TOF-HRMS: *m/z* calculated for $C_{19}H_{29}BNaO_6$ $[M+Na]^+$ 387.1953, found 387.1957.

(*R*)-1-[4,5-dimethoxy-2-(4,4,5,5-tetramethyl-1,3,2-dioxaborolan-2-yl)phenyl]propan-2-yl acetate [(*R*)-8]

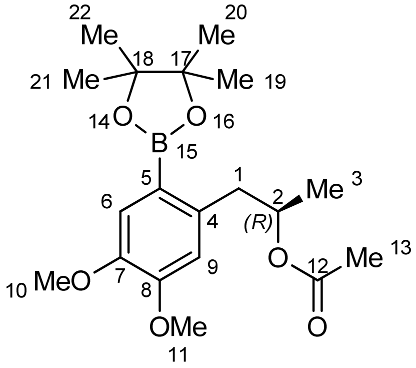

Starting from (*R*)-**13** (2.50 g, 6.85 mmol, 1.0 equiv.): anhydrous DMF (37 ml), Ph_3P (360 mg, 1.37 mmol, 0.2 equiv.), $(Ph_3P)_2PdCl_2$ (481 mg, 0.69 mmol, 0.1 equiv.), anhydrous NaOAc (2.25 g, 27.42 mmol, 4.0 equiv.), B_2Pin_2 (5.22 g, 20.56 mmol, 3.0 equiv), reaction time: 3 hours. (*R*)-**8**: 1.50 g (yield: 60%) pale yellow syrup; $[\alpha]_D^{20}$ −23 (c = 0.44; $CHCl_3$). Chromatographic and spectral data except for the chiroptical ones were identical with those of (*S*)-**8**.

2.2.5. General procedure for oxa-Pictet-Spengler reactions of chiral non-racemic 1-arylpropan-2-ol derivatives

Method A: The 1-arylpropan-2-ol derivative (1.0 equiv.) was dissolved in anhydrous Et_2O (5-20 ml), and the solution was cooled to 0 °C. The reagent MOMCl (5.0-10.0 equiv.) and annealed $ZnCl_2$ (0.3 equiv.) were added to the solution. When the starting material was consumed (2-4 hours) on the basis of TLC monitoring, the reaction was quenched with water and stirred for 15 minutes. Et_2O was added to the mixture and the layers were separated. The organic layer was extracted twice with a saturated solution of $NaHCO_3$ and once with brine. The organic phase was dried over anhydrous Na_2SO_4, filtered, and the solvent was evaporated in *vacuo*. The residue was purified by flash chromatography to yield the chiral non-racemic isochroman target derivative.

Method B: The 1-arylpropan-2-ol derivative (1.0 equiv.) was dissolved in anhydrous CH_2Cl_2 (30-55 ml), and 4-fluorobenzaldehyde (1.2 equiv.) was added to the solution. The solution was cooled to 0 °C and $BF_3{\cdot}Et_2O$ (0.3 equiv.) was added. The reaction mixture was allowed to warm up to room temperature. When the starting material was consumed (4 hours) on the basis of TLC monitoring, the reaction was quenched with water and stirred for 20 minutes. CH_2Cl_2 was added to the mixture and the layers were separated. The organic layer was extracted twice with a saturated solution of $NaHCO_3$ and once with brine. The organic phase was dried over anhydrous Na_2SO_4, filtered, and the solvent was evaporated in *vacuo*. The residue was purified by flash chromatography to yield the chiral non-racemic isochroman target derivatives.

Method C: The 1-arylpropan-2-ol derivative (1.0 equiv.) was dissolved in anhydrous toluene (3 ml) and the solution was cooled to 0 °C. Then ethyl-3,3-diethoxypropionate (90%, technical grade, 2.0 equiv.) and $BF_3{\cdot}Et_2O$ (0.3 equiv.) were added to the solution and the reaction was allowed to warm up to room temperature. When the starting material was consumed (3-16 hours) on the basis of TLC monitoring, the reaction was quenched with a saturated solution of $NaHCO_3$ and stirred for 10 minutes. EtOAc was added to the mixture, and the layers were separated. The organic layer was extracted twice with a saturated solution of $NaHCO_3$ and once with brine. The organic phase was dried over anhydrous Na_2SO_4, filtered, and the solvent was evaporated in *vacuo*. The residue was purified by flash chromatography to yield the chiral non-racemic isochroman target derivatives.

(1*R*,3*S*)-1-(4-fluorophenyl)-6,7,8-trimethoxy-3-methylisochroman [*cis*-(1*R*,3*S*)-17] and (1*S*,3*S*)-1-(4-fluorophenyl)-6,7,8-trimethoxy-3-methylisochroman [*trans*-(1*S*,3*S*)-17]

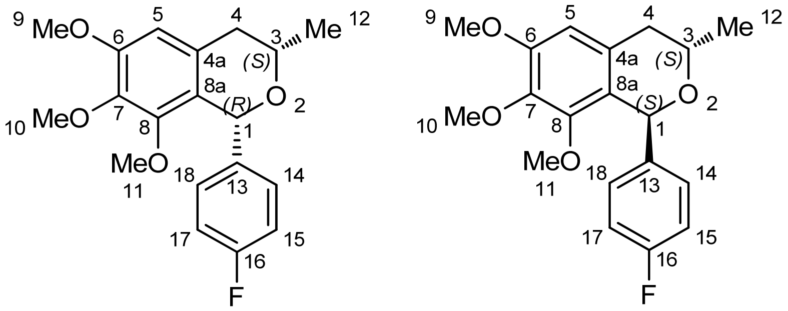

Starting from (*S*)-**16** (2.13 g, 9.39 mmol, 1.0 equiv.) using **method B**: anhydrous CH_2Cl_2 (30 ml), 4-fluorobenzaldehyde (1.21 ml, 1.40 g, 11.27 mmol, 2.0 equiv.), $BF_3{\cdot}Et_2O$ (348 µl, 400 mg, 2.82 mmol, 0.3 equiv.), reaction time: 4 hours. *cis*-(1*R*,3*S*)-**17**: 1.12 g (yield: 36%) colorless syrup; $[\alpha]_D^{20}$ +16 (*c* = 0.47; $CHCl_3$); *trans*-(1*S*,3*S*)-**17**: 1.90g (yield: 61%) white solid; mp 49-51 °C; $[\alpha]_D^{20}$ −18 (*c* = 0.50; $CHCl_3$); *dr cis:trans* = 1.0:1.7. Flash chromatography (hexane/CH_2Cl_2 7:3 → 65:35 → 6:4 → 55:45 → 1:1 → 4:6 → 3:7, then CH_2Cl_2/EtOAc 1:1).

cis-(1*R*,3*S*)-**17**: R_f = 0.38 (CH_2Cl_2); ^{1}H NMR (400 MHz, $CDCl_3$) δ = 7.30 – 7.23 (m, 2H, *H-14*, *H-18*), 7.02 – 6.94 (m, 2H, *H-15*, *H-17*), 6.44 (s, 1H, *H-5*), 5.76 (s, 1H, *H-1*), 3.84 (s, 3H, *H-9*), 3.83 – 3.77 (m, 1H, *H-3*), 3.74 (s, 3H, *H-10*), 3.10 (s, 3H, *H-11*), 2.80 (dd, *J* = 15.8, 10.7 Hz, 1H, $H\text{-}4_{ax}$), 2.61 (d, *J* = 15.6 Hz, 1H, $H\text{-}4_{eq}$), 1.33 (d, *J* = 6.2 Hz, 3H, *H-12*); ^{13}C NMR (100 MHz, $CDCl_3$) δ = 162.2 (1C, d, $J_{C\text{-}F}$ = 245.1 Hz, *C-16*), 152.7 (1C, *C-6*), 150.3 (1C, *C-8*), 140.6 (1C, *C-7*), 140.2 (1C, d, $J_{C\text{-}F}$ = 2.8 Hz, *C-13*), 130.6 (1C, *C-4a*), 130.0 (2C, d, $J_{C\text{-}F}$ = 8.1 Hz, *C-14*, *C-18*), 123.4 (1C, *C-8a*), 115.0 (2C, d, $J_{C\text{-}F}$ = 21.3 Hz, *C-15*, *C-17*), 106.7 (1C, *C-5*), 77.2 (1C, *C-1*), 70.4 (1C, *C-3*), 60.5 (1C, *C-10*), 59.2 (1C, *C-11*), 55.9 (1C, *C-9*), 36.9 (1C, *C-4*), 21.7 (1C, *C-12*); IR (KBr) ν = 3045 (ν Ar =CH), 2970, 2937 (ν_{as} Me, ν_{as} CH_2), 2895, 2840 (ν_s Me, ν_s CH_2), 1894, 1747 (γ Ar C=C overtone and combination bands), 1602, 1583, 1509, 1492, 1457 (ν Ar C=C, β_s CH_2, δ_{as} Me), 1383, 1342, 1305 (δ_s Me, δ CH, γ_s CH_2), 1258, 1240, 1221, 1148, 1114 (ν_{as} Ar-O-Me, ν Ar C-F), 1083, 1055, 1024 (ν_{as} C-O-C, ν_s Ar-O-Me), 859 (1,2,3,4,5-pentasubstituated γ_s Ar =CH) cm^{-1}; ESI-TOF-HRMS: *m/z* calculated for $C_{19}H_{21}FNaO_4$ $[M+Na]^+$ 355.1316, found 355.1315.

trans-(1*S*,3*S*)-**17**: R_f = 0.24 (CH_2Cl_2); ^{1}H NMR (400 MHz, $CDCl_3$) δ = 7.24 – 7.15 (m, 2H, *H-14*, *H-18*), 7.01 – 6.94 (m, 2H, *H-15*, *H-17*), 6.48 (s, 1H, *H-5*), 5.95 (s, 1H, *H-1*), 3.87 (s, 3H, *H-9*), 3.82 (s, 3H, *H-10*), 3.79 – 3.68 (m, 1H, *H-3*), 3.53 (s, 3H, *H-11*), 2.71 – 2.58 (m, 2H, *H-4*), 1.18 (d, *J* = 6.1 Hz, 3H, *H-12*); ^{13}C NMR (100 MHz, $CDCl_3$) δ = 162.1 (1C, d, $J_{C\text{-}F}$ = 245.7 Hz, *C-16*), 152.9 (1C, *C-6*), 149.8 (1C, *C-8*), 140.0 (1C, *C-7*), 138.3 (1C, d, $J_{C\text{-}F}$ = 2.9 Hz, *C-13*), 130.2 (2C, d, $J_{C\text{-}F}$ = 8.0 Hz, *C-14*, *C-18*), 129.8 (1C, *C-4a*), 121.0 (1C, *C-8a*), 114.7 (2C, d, $J_{C\text{-}F}$ = 21.3 Hz, *C-15*, *C-17*), 106.9 (1C, *C-5*), 73.2 (1C, *C-1*), 63.0 (1C, *C-3*), 60.7 (1C, *C-10*), 60.0 (1C, *C-11*), 55.9 (1C, *C-9*), 35.4 (1C, *C-4*), 21.5 (1C, *C-12*); IR (KBr) ν = 3047, 3011

(ν Ar =CH), 2969, 2943 (ν_{as} Me, ν_{as} CH_2), 2882, 2846 (ν_s Me, ν_s CH_2), 1904 (γ Ar C=C overtone and combination bands), 1602, 1584, 1505, 1492, 1460 (ν Ar C=C, β_s CH_2, δ_{as} Me), 1385, 1365, 1344, 1329 (δ_s Me, δ CH, γ_s CH_2), 1265, 1241, 1223, 1201, 1172, 1161, 1145, 1120 (ν_{as} Ar-O-Me, ν Ar C-F), 1097, 1069, 1051, 1023 (ν_{as} C-O-C, ν_s Ar-O-Me), 882 (1,2,3,4,5-pentasubstituated γ_s Ar =CH) cm^{-1}; ESI-TOF-HRMS: *m/z* calculated for $C_{19}H_{21}FNaO_4$ $[M+Na]^+$ 355.1316, found 355.1315.

(1*S*,3*R*)-1-(4-fluorophenyl)-6,7,8-trimethoxy-3-methylisochroman [*cis*-(1*S*,3*R*)-17] and (1*R*,3*R*)-1-(4-fluorophenyl)-6,7,8-trimethoxy-3-methylisochroman [*trans*-(1*R*,3*R*)-17]

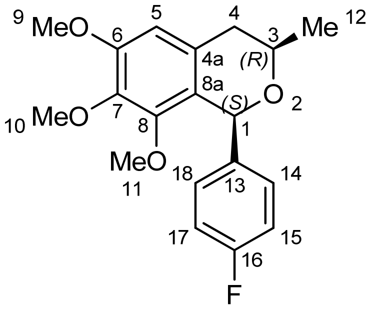

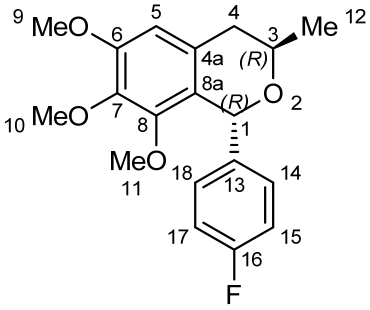

Starting from (*R*)-**16** (3.95 g, 17.48 mmol, 1.0 equiv.) using **method B**: anhydrous CH_2Cl_2 (55 ml), 4-fluorobenzaldehyde (2.25 ml, 2.60 g, 20.97 mmol, 1.2 equiv.), $BF_3{\cdot}Et_2O$ (647 μl, 744 mg, 5.24 mmol, 0.3 equiv.), reaction time: 4 hours. *cis*-(1*S*,3*R*)-**17**: 2.03 g (yield: 35%) colorless syrup; $[\alpha]_D^{20}$ −16 (*c* = 0.50; $CHCl_3$); *trans*-(1*R*,3*R*)-**17**: 3.45 g (yield: 59%) white solid; mp 47-50 °C; $[\alpha]_D^{20}$ +14 (*c* = 0.49; $CHCl_3$); *dr cis:trans* = 1.0:1.7. Chromatographic and spectral data except for the chiroptical ones were identical with those of *cis*-(1*R*,3*S*)-**17** and *trans*-(1*S*,3*S*)-**17**.

(a*S*,1*S*,3*S*,3'*S*)-1-(4-fluorophenyl)-6,7,7',8,8'-pentamethoxy-3,3'-dimethyl-5,5'-*bis*-isochroman [(a*S*,1*S*,3*S*,3'*S*)-21]

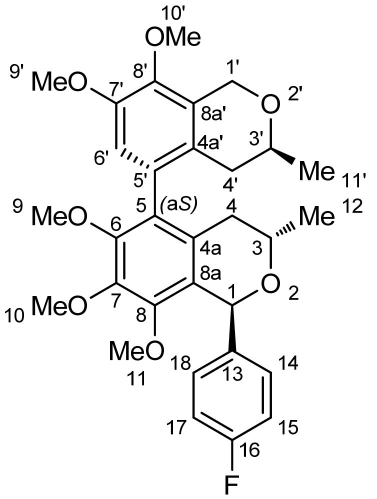

Starting from (a*S*,1*S*,3*S*,2'*S*)-**20** (70 mg, 0.13 mmol, 1.0 equiv.) using **method A**: anhydrous Et_2O (5 ml), MOMCl (100 µl, 106 mg, 1.32 mmol, 10.0 equiv.), annealed $ZnCl_2$ (5 mg, 0.04 mmol, 0.3 equiv.), reaction time: 4 hours. (a*S*,1*S*,3*S*,3'*S*)-**21**: 68 mg (yield: 96%) white amorphous solid foam; $[\alpha]_D^{20}$ +66; $[\alpha]_{578}^{20}$ +69; $[\alpha]_{546}^{20}$ +78; $[\alpha]_{436}^{20}$ +130; $[\alpha]_{365}^{20}$ +203 (*c* = 0.51; MeCN); ECD: (*c* = 1.94×10^{-4} M; MeCN) λ [nm], (Δε) = 287sh (−0.35), 271 (−0.96), 263sh (−1.57), 243 (−5.99), 221 (1.12), 215 (−1.23), 198sh (34.62). Flash chromatography (hexane/EtOAc 9:1 → 85:15 → 8:2 → 7:3); R_f = 0.29 (hexane/EtOAc 8:2); ^{1}H NMR (400 MHz, $CDCl_3$) δ = 7.22 – 7.17 (m, 2H, *H-14*, *H-18*), 7.06 – 6.99 (m, 2H, *H-15*, *H-17)*, 6.61 (s, 1H, *H-6'*), 6.04 (s, 1H, *H-1*), 5.07 (d, *J* = 15.8 Hz, 1H, $H\text{-}1'_{eq}$), 4.77 (d, *J* = 15.8 Hz, 1H, $H\text{-}1'_{ax}$), 3.89 (s, 3H, *H-10*), 3.89 (s, 3H, *H-10'*), 3.83 (s, 3H, *H-9'*), 3.71 – 3.66 (m, 1H, *H-3'*), 3.66 (s, 3H, *H-9*), 3.64 – 3.58 (m, 1H, *H-3*), 3.57 (s, 3H, *H-11*), 2.41 (dd, *J* = 16.4, 10.6 Hz, 1H, $H\text{-}4'_{ax}$), 2.25 (dd, *J* = 17.1, 11.0 Hz, 1H, $H\text{-}4_{ax}$), 2.08 (dd, *J* = 16.4, 2.5 Hz, 1H, $H\text{-}4'_{eq}$), 1.97 (dd, *J* = 17.1, 3.5 Hz, 1H, $H\text{-}4_{eq}$), 1.30 (d, *J* = 6.1 Hz, 1H, *H-11'*), 1.08 (d, *J* = 6.1 Hz, 1H, *H-12*); ^{13}C NMR (100 MHz, $CDCl_3$) δ = 162.3 (d, $J_{C\text{-}F}$ = 246.1 Hz, 1C, *C-16*), 151.2 (1C, *C-6*), 150.0 (1C, *C-7'*), 149.4 (1C, *C-8*), 144.1 (1C, *C-7*), 143.7 (1C, *C-8'*), 138.3 (d, $J_{C\text{-}F}$ = 2.9 Hz, 1C, *C-13*), 131.3 (1C, *C-5'*), 130.3 (d, $J_{C\text{-}F}$ = 8.0 Hz, 2C, *C-14*, *C-18*), 128.8 (2C, *C-5*, *C-8a'*), 128.3 (1C, *C-4a*), 125.1 (1C, *C-4a'*), 124.3 (1C, *C-8a*), 114.9 (d, $J_{C\text{-}F}$ = 21.1 Hz, 2C, *C-15*, *C-17*), 112.6 (1C, *C-6'*), 73.4 (1C, *C-1*), 70.8 (1C, *C-3'*), 64.9 (1C, *C-1'*), 63.3 (1C, *C-3*), 61.2 (1C, *C-9*), 60.8 (1C, *C-10*), 60.3 (1C, *C-10'*), 60.1 (1C, *C-11*), 55.9 (1C, *C-9'*), 34.4 (1C, *C-4*), 33.7 (1C, *C-4'*), 21.8 (2C, *C-11'*, *C-12*); IR (KBr) ν = 2967, 2935 (ν_{as} Me, ν_{as} CH_2), 2838 (ν_s CH_2), 1603, 1578, 1507, 1491, 1464, 1420, 1408 (ν Ar C=C, β_s CH_2, δ_{as} Me), 1383, 1363, 1316, (δ_s Me, δ CH, γ_s CH_2), 1254, 1223, 1199, 1157, 1114 (ν_{as} Ar-O-Me, ν Ar C-F), 1085, 1064, 1028 (ν_{as} C-O-C, ν_s Ar-O-Me), 864 (1,2,3,4,5-pentasubstituated γ_s Ar =CH) cm^{-1}; ESI-TOF-HRMS: *m/z* calculated for $C_{31}H_{35}FNaO_7$ $[M+Na]^+$ 561.2259, found 561.2257.

(a*R*,1*R*,3*R*,3'*R*)-1-(4-fluorophenyl)-6,7,7',8,8'-pentamethoxy-3,3'-dimethyl-5,5'-*bis*-isochroman [(a*R*,1*R*,3*R*,3'*R*)-21]

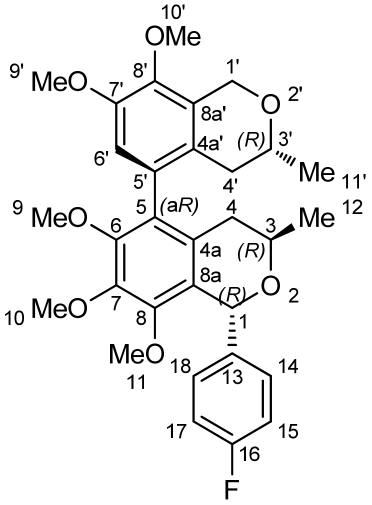

Starting from (a*R*,1*R*,3*R*,2'*R*)-**20** (310 mg, 0.59 mmol, 1.0 equiv.) using **method A**: anhydrous Et_2O (20 ml), MOMCl (447 µl, 474 mg, 5.89 mmol, 10.0 equiv.), annealed $ZnCl_2$ (24 mg, 0.18 mmol, 0.3 equiv.), reaction time: 4 hours. (a*R*,1*R*,3*R*,3'*R*)-**21**: 272 mg (yield: 86%) white amorphous solid foam; $[\alpha]_D^{20}$ −57; $[\alpha]_{578}^{20}$ −59; $[\alpha]_{546}^{20}$ −67; $[\alpha]_{436}^{20}$ −103; $[\alpha]_{365}^{20}$ −158 (*c* = 0.52; MeCN); ECD: (*c* = 1.71×10^{-4} M; MeCN) λ [nm], (Δε) = 287sh (0.45), 271 (0.98), 263sh (1.56), 242 (5.56), 220 (−0.67), 214 (0.77), 198 (−31.91). Chromatographic and spectral data except for the chiroptical ones were identical with those of (a*S*,1*S*,3*S*,3'*S*)-**21**.

(a*R*,1*S*,3*S*,3'*S*)-1-(4-fluorophenyl)-6,7,7',8,8'-pentamethoxy-3,3'-dimethyl-5,5'-*bis*-isochroman [(a*R*,1*S*,3*S*,3'*S*)-21]

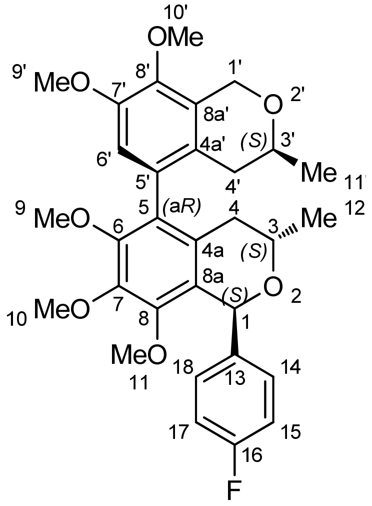

Starting from (a*R*,1*S*,3*S*,2'*S*)-**20** (93 mg, 0.18 mmol, 1.0 equiv.) using **method A**: anhydrous Et_2O (5 ml), MOMCl (100 µl, 106 mg, 1.32 mmol, 7.5 equiv.), annealed $ZnCl_2$ (7 mg, 0.05 mmol, 0.3 equiv.), reaction time: 4 hours. (a*R*,1*S*,3*S*,3'*S*)-**21**: 94 mg (yield: 99%) off-white

solid; mp 147-150 °C; $[\alpha]_D^{20}$ +80; $[\alpha]_{578}^{20}$ +83; $[\alpha]_{546}^{20}$ +96; $[\alpha]_{436}^{20}$ +173; $[\alpha]_{365}^{20}$ +301 (*c* = 0.49; MeCN); ECD: (*c* = 2.24×10^{-4} M; MeCN) λ [nm], (Δε) = 286 (0.09), 271 (−0.43), 264 (−0.51), 259sh (−0.20), 255 (0.12), 243 (−0.73), 234sh (1.68), 219sh (5.73), 206 (20.54). Purification was not required for the crude product. R_f = 0.27 (hexane/EtOAc 8:2); ^{1}H NMR (400 MHz, $CDCl_3$) δ = 7.29 – 7.22 (m, 2H, *H-14*, *H-18*), 7.08 – 6.97 (m, 2H, *H-15*, *H-17*), 6.61 (s, 1H, *H-6'*), 6.01 (s, 1H, *H-1*), 5.06 (d, *J* = 15.8 Hz, 1H, $H\text{-}1'_{eq}$), 4.78 (d, *J* = 15.8 Hz, 1H, $H\text{-}1'_{ax}$), 3.89 (s, 3H, *H-10*), 3.88 (s, 3H, *H-10'*), 3.86 (s, 3H, *H-9'*), 3.76 – 3.67 (m, 1H, *H-3'*), 3.67 (s, 3H, *H-9*), 3.66 – 3.62 (m, 1H, *H-3*), 3.57 (s, 3H, *H-11*), 2.27 (dd, *J* = 16.3, 3.3 Hz, 1H, $H\text{-}4'_{eq}$), 2.26 (dd, *J* = 17.0, 3.7 Hz, 1H, $H\text{-}4_{eq}$), 2.14 (dd, *J* = 16.3, 10.4 Hz, 1H, $H\text{-}4'_{ax}$), 2.05 (dd, *J* = 17.0, 10.7 Hz, 1H, $H\text{-}4_{ax}$), 1.26 (d, *J* = 6.1 Hz, 3H, *H-11'*), 1.08 (d, *J* = 6.1 Hz, 3H, *H-12*); ^{13}C NMR (100 MHz, $CDCl_3$) δ = 162.3 (d, $J_{C\text{-}F}$ = 245.8 Hz, 1C, *C-16*), 150.3 (1C, *C-6*), 149.9 (1C, *C-7'*), 149.4 (1C, *C-8*), 143.9 (1C, *C-7*), 143.8 (1C, *C-8'*), 138.3 (d, $J_{C\text{-}F}$ = 2.9 Hz, 1C, *C-13*), 131.5 (1C, *C-5'*), 130.4 (d, $J_{C\text{-}F}$ = 8.0 Hz, 2C, *C-14*, *C-18*), 129.3 (1C, *C-5*), 129.0 (1C, *C-4a*), 128.9 (1C, *C-8a'*), 125.8 (1C, *C-4a'*), 124.5 (1C, *C-8a*), 114.8 (d, $J_{C\text{-}F}$ = 21.2 Hz, 2C, *C-15*, *C-17*), 112.4 (1C, *C-6'*), 73.4 (1C, *C-1*), 70.8 (1C, *C-3'*), 65.0 (1C, *C-1'*), 63.4 (1C, *C-3*), 61.0 (1C, *C-9*), 60.8 (1C, *C-10*), 60.2 (1C, *C-10'*), 60.1 (1C, *C-11*), 56.1 (1C, *C-9'*), 33.5 (1C, *C-4'*), 33.3 (1C, *C-4*), 21.7 (1C, *C-11'*), 21.6 (1C, *C-12*); IR (KBr) ν = 2963, 2934 (ν_{as} Me, ν_{as} CH_2), 2837 (ν_s CH_2), 1907, 1729 (γ Ar =CH, γ Ar C=C overtone and combination bands), 1602, 1579, 1506, 1489, 1465, 1421, 1408 (ν Ar C=C, β_s CH_2, δ_{as} Me), 1383, 1361, 1315, (δ_s Me, δ CH, γ_s CH_2), 1267, 1253, 1233, 1218, 1198, 1159, 1129, 1117 (ν_{as} Ar-O-Me, ν Ar C-F), 1096, 1086, 1066, 1052, 1027 (ν_{as} C-O-C, ν_s Ar-O-Me) cm^{-1}; ESI-TOF-HRMS: *m/z* calculated for $C_{31}H_{35}FNaO_7$ $[M+Na]^+$ 561.2259, found 561.2257.

(a*S*,1*R*,3*R*,3'*R*)-1-(4-fluorophenyl)-6,7,7',8,8'-pentamethoxy-3,3'-dimethyl-5,5'-*bis*-isochroman [(a*S*,1*R*,3*R*,3'*R*)-21]

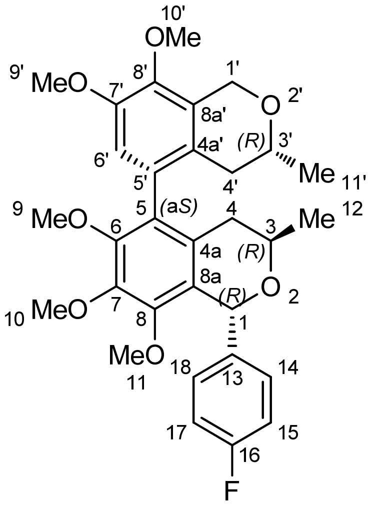

Starting from (a*S*,1*R*,3*R*,2'*R*)-**20** (300 mg, 0.57 mmol, 1.0 equiv.) using **method A**: anhydrous Et_2O (20 ml), MOMCl (325 µl, 344 mg, 4.27 mmol, 7.5 equiv.), annealed $ZnCl_2$ (23 mg, 0.17 mmol, 0.3 equiv.), reaction time: 4 hours. (a*S*,1*R*,3*R*,3'*R*)-**21**: 304 mg (yield: 99%) off-white solid; mp 150-151 °C; $[\alpha]_D^{20}$ −84; $[\alpha]_{578}^{20}$ −88; $[\alpha]_{546}^{20}$ −101; $[\alpha]_{436}^{20}$ −155; $[\alpha]_{365}^{20}$ −298 (*c* = 0.50; MeCN); ECD (*c* = 1.44×10^{-4} M; MeCN) λ [nm], (Δε) = 286 (−0.15), 271 (0.40), 264 (0.48), 259sh (0.16), 253 (−0.06), 243 (0.70), 232sh (−1.62), 219sh (−6.00), 206 (−20.88). Purification was not required for the crude product. Chromatographic and spectral data except for the chiroptical ones were identical with those of (a*R*,1*S*,3*S*,3'*S*)-**21**.

(a*S*,1*R*,3*S*,3'*S*)-1-(4-fluorophenyl)-6,7,7',8,8'-pentamethoxy-3,3'-dimethyl-5,5'-*bis*-isochroman [(a*S*,1*R*,3*S*,3'*S*)-21]

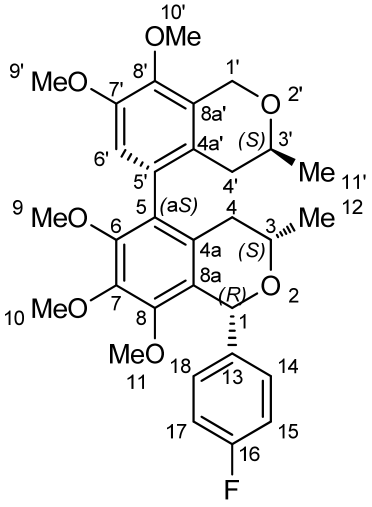

Starting from (a*S*,1*R*,3*S*,2'*S*)-**20** (63 mg, 0.12 mmol, 1.0 equiv.) using **method A**: anhydrous Et_2O (5 ml), MOMCl (91 µl, 97 mg, 1.20 mmol, 10.0 equiv.), annealed $ZnCl_2$ (5 mg, 0.04 mmol, 0.3 equiv.), reaction time: 4 hours. (a*S*,1*R*,3*S*,3'*S*)-**21**: 63 mg (yield: 97%) off-white amorphous solid foam; $[\alpha]_D^{20}$ −55; $[\alpha]_{578}^{20}$ −57; $[\alpha]_{546}^{20}$ −68; $[\alpha]_{436}^{20}$ −141; $[\alpha]_{365}^{20}$ −279 (*c* = 0.49;

MeCN); ECD (c = 1.65×10^{-4} M; MeCN) λ [nm], (Δε) = 287 (−1.45), 270sh (−1.14), 249sh (−5.90), 224 (−28.53), 201 (31.60). Flash chromatography (hexane/EtOAc 9:1 → 85:15); R_f = 0.28 (hexane/EtOAc 85:15); ^{1}H NMR (400 MHz, $CDCl_3$) δ = 7.39 – 7.31 (m, 2H, *H-14*, *H-18*), 7.08 – 6.99 (m, 2H, *H-15*, *H-17*), 6.65 (s, 1H, *H-6'*), 5.82 (s, 1H, *H-1*), 5.07 (d, J = 15.8 Hz, 1H, $H\text{-}1'_{ax}$), 4.77 (d, J = 15.8 Hz, 1H, $H\text{-}1'_{eq}$), 3.90 (s, 3H, *H-10'*), 3.87 (s, 3H, *H-9'*), 3.80 (s, 3H, *H-10*), 3.76 – 3.59 (m, 2H, *H-3*, *H-3'*), 3.57 (s, 3H, *H-9*), 3.15 (s, 3H, *H-11*), 2.41 (dd, J = 16.4, 10.9 Hz, 1H, $H\text{-}4'_{ax}$), 2.41 (dd, J = 16.4, 10.9 Hz, 1H, $H\text{-}4_{ax}$), 1.99 (ddd, J = 16.4, 2.3, 1.2 Hz, 1H, $H\text{-}4'_{eq}$), 1.97 (ddd, J = 16.4, 3.2, 1.0 Hz, 1H, $H\text{-}4_{eq}$), 1.26 (d, J = 6.1 Hz, 3H, *H-11'*), 1.23 (d, J = 6.1 Hz, 3H, *H-12*); ^{13}C NMR (100 MHz, $CDCl_3$) δ = 162.3 (d, $J_{C\text{-}F}$ = 245.0 Hz, 1C, *C-16*), 150.9 (1C, *C-6*), 149.8 (1C, *C-7'*), 149.8 (1C, *C-8*), 144.6 (1C, *C-7*), 143.8 (1C, *C-8'*), 140.2 (d, $J_{C\text{-}F}$ = 2.7 Hz, 1C, *C-13*), 131.3 (1C, *C-5'*), 130.2 (d, $J_{C\text{-}F}$ = 8.1 Hz, 2C, *C-14*, *C-18*), 129.2 (1C, *C-4a*), 128.7 (1C, *C-8a'*), 128.4 (1C, *C-5*), 126.7 (1C, *C-8a*), 125.8 (1C, *C-4a'*), 115.1 (d, $J_{C\text{-}F}$ = 21.3 Hz, 2C, *C-15*, *C-17*), 113.0 (1C, *C-6'*), 77.6 (1C, *C-1*), 70.8, 70.6 (2C, *C-3*, *C-3'*), 65.0 (1C, *C-1'*), 61.0 (1C, *C-9*), 60.7 (1C, *C-10*), 60.3 (1C, *C-10'*), 59.4 (1C, *C-11*), 56.1 (1C, *C-9'*), 35.6 (1C, *C-4*), 33.5 (1C, *C-4'*), 21.8 (1C, *C-12*), 21.7 (1C, *C-11'*); IR (KBr) ν = 2970, 2936 (ν_{as} Me, ν_{as} CH_2), 2843 (ν_s CH_2), 1604, 1575, 1510, 1490, 1463, 1420 (ν Ar C=C, β_s CH_2, δ_{as} Me), 1383, 1361, 1317, (δ_s Me, δ CH, γ_s CH_2), 1279, 1255, 1232, 1200, 1148, 1118 (ν_{as} Ar-O-Me, ν Ar C-F), 1102, 1085, 1029 (ν_{as} C-O-C, ν_s Ar-O-Me) cm^{-1}; ESI-TOF-HRMS: *m/z* calculated for $C_{31}H_{35}FNaO_7$ $[M+Na]^+$ 561.2259, found 561.2256.

(a*R*,1*S*,3*R*,3'*R*)-1-(4-fluorophenyl)-6,7,7',8,8'-pentamethoxy-3,3'-dimethyl-5,5'-*bis*-isochroman [(a*R*,1*S*,3*R*,3'*R*)-21]

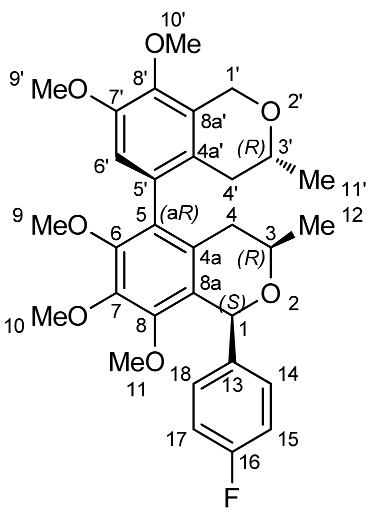

Starting from (a*R*,1*S*,3*R*,2'*R*)-**20** (100 mg, 0.19 mmol, 1.0 equiv.) using **method A**: anhydrous Et_2O (5 ml), MOMCl (144 µl, 153 mg, 1.90 mmol, 10.0 equiv.), annealed $ZnCl_2$ (8 mg, 0.06

mmol, 0.3 equiv.), reaction time: 4 hours. (a*R*,1*S*,3*R*,3'*R*)-**21**: 90 mg (yield: 88%) off-white amorphous solid foam; $[\alpha]_D^{20}$ +49; $[\alpha]_{578}^{20}$ +51; $[\alpha]_{546}^{20}$ +61; $[\alpha]_{436}^{20}$ +116; $[\alpha]_{365}^{20}$ +269 (*c* = 0.49; MeCN); ECD (*c* = 1.94×10^{-4} M; MeCN) λ [nm], (Δε) = 287 (1.13), 270sh (1.00), 248sh (5.02), 223 (24.51), 201 (−28.82). Chromatographic and spectral data except for the chiroptical ones were identical with those of (a*S*,1*R*,3*S*,3'*S*)-**21**.

(a*R*,1*R*,3*S*,3'*S*)-1-(4-fluorophenyl)-6,7,7',8,8'-pentamethoxy-3,3'-dimethyl-5,5'-*bis*-isochroman [(a*R*,1*R*,3*S*,3'*S*)-21]

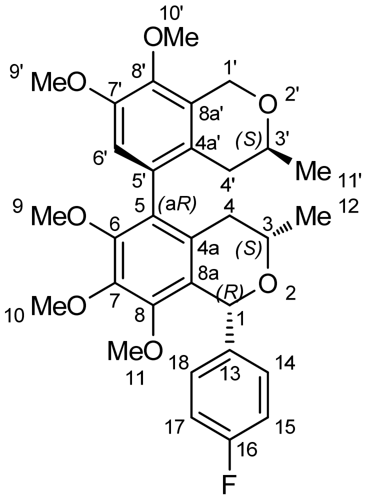

Starting from (a*R*,1*R*,3*S*,2'*S*)-**20** (41 mg, 0.08 mmol, 1.0 equiv.) using **method A**: anhydrous Et_2O (7 ml), MOMCl (30 µl, 31 mg, 0.39 mmol, 5.0 equiv.), annealed $ZnCl_2$ (3 mg, 0.02 mmol, 0.3 equiv.), reaction time: 3 hours. (a*R*,1*R*,3*S*,3'*S*)-**21**: 37 mg (yield: 89%) off-white amorphous solid foam; $[\alpha]_D^{20}$ −35; $[\alpha]_{578}^{20}$ −37; $[\alpha]_{546}^{20}$ −43; $[\alpha]_{436}^{20}$ −87; $[\alpha]_{365}^{20}$ −147 (*c* = 0.46; MeCN); ECD (*c* = 1.98×10^{-4} M; MeCN) λ [nm], (Δε) = 287 (−0.49), 271sh (−0.66), 226 (−14.37), 205 (13.86), 202sh (12.76). Flash chromatography (hexane/EtOAc 9:1 → 85:15); R_f = 0.42 (hexane/EtOAc 8:2); ^{1}H NMR (400 MHz, $CDCl_3$) δ = 7.31 – 7.22 (m, 2H, *H-14*, *H-18*), 7.07 – 6.99 (m, 2H, *H-15*, *H-17*), 6.61 (s, 1H, *H-6'*), 5.83 (s, 1H, *H-1*), 5.08 (d, *J* = 15.8 Hz, 1H, *H-1'$_{ax}$*), 4.79 (d, *J* = 15.8 Hz, 1H, *H-1'$_{eq}$*), 3.89 (s, 3H, *H-10'*), 3.84 (s, 3H, *H-9'*), 3.82 (s, 3H, *H-10*), 3.78 – 3.68 (m, 2H, *H-3*, *H-3'*), 3.61 (s, 3H, *H-9*), 3.17 (s, 3H, *H-11*), 2.35 – 2.18 (m, 4H, *H-4'*, *H-4*), 1.32 (d, *J* = 6.1 Hz, 3H, *H-11'*), 1.22 (d, *J* = 6.1 Hz, 3H, *H-12*); ^{13}C NMR (100 MHz, $CDCl_3$) δ = 162.3 (d, $J_{C\text{-}F}$ = 245.3 Hz, 1C, *C-16*), 150.3 (1C, *C-6*), 150.0 (1C, *C-7'*), 149.7 (1C, *C-8*), 144.6 (1C, *C-7*), 143.8 (1C, *C-8'*), 140.2 (d, $J_{C\text{-}F}$ = 3.0 Hz, 1C, *C-13*), 131.3 (1C, *C-5'*), 130.1, (1C, *C-4a*), 130.0 (d, $J_{C\text{-}F}$ = 8.0 Hz, 2C, *C-14*, *C-18*), 128.9 (1C, *C-8a'*), 128.7 (1C, *C-5*), 126.9 (1C, *C-8a*), 125.6 (1C, *C-4a'*), 115.2 (d, $J_{C\text{-}F}$ = 21.4 Hz, 2C, *C-15*, *C-17*), 112.6 (1C, *C-6'*), 77.3 (1C, *C-1*), 70.7 (1C, *C-3'*), 70.5 (1C, *C-3*), 65.1 (1C, *C-1'*), 60.9 (1C, *C-9*), 60.7 (1C, *C-10*),

60.3 (1C, *C-10'*), 59.3 (1C, *C-11*), 56.0 (1C, *C-9'*), 34.4 (1C, *C-4*), 33.6 (1C, *C-4'*), 21.8 (2C, *C-11'*, *C-12*); IR (KBr) ν = 2970, 2935 (ν_{as} Me, ν_{as} CH_2), 2847 (ν_s CH_2), 1730 (γ Ar =CH, γ Ar C=C overtone and combination bands), 1604, 1577, 1509, 1490, 1463, 1421, 1407 (ν Ar C=C, β_s CH_2, δ_{as} Me), 1384, 1359, 1317, (δ_s Me, δ CH, γ_s CH_2), 1279, 1255, 1223, 1201, 1152, 1116 (ν_{as} Ar-O-Me, ν Ar C-F), 1074, 1054, 1029 (ν_{as} C-O-C, ν_s Ar-O-Me) cm^{-1}; ESI-TOF-HRMS: *m/z* calculated for $C_{31}H_{35}FNaO_7$ $[M+Na]^+$ 561.2259, found 561.2251.

(a*S*,1*R*,3*S*,1'*S*,3'*S*)-ethyl 1-(4-fluorophenyl)-6,7,7',8,8'-pentamethoxy-3,3'-dimethyl-[5,5'-*bis*-isochroman]-1'-carboxylate [(a*S*,1*R*,3*S*,1'*S*,3'*S*)-22] and (a*S*,1*S*,3*S*,1'*S*,3'*S*)-ethyl 1-(4-fluorophenyl)-6,7,7',8,8'-pentamethoxy-3,3'-dimethyl-[5,5'-*bis*-isochroman]-1'-carboxylate [(a*S*,1*S*,3*S*,1'*S*,3'*S*)-22]

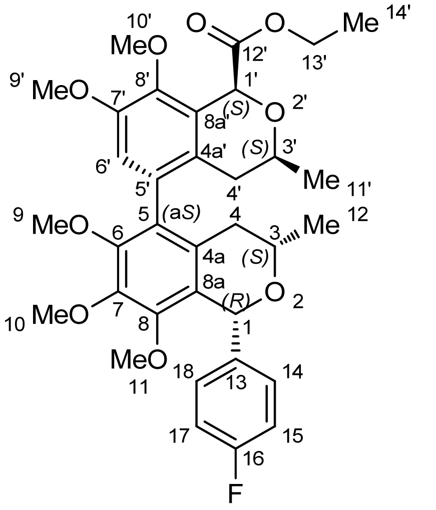

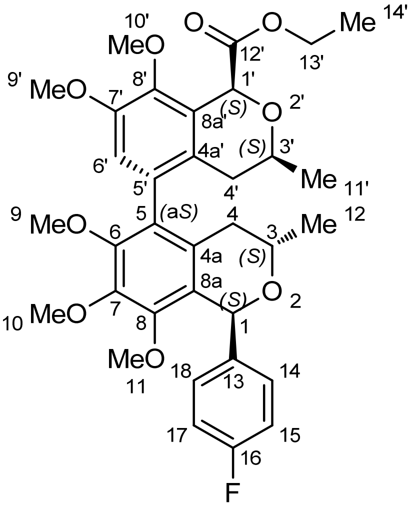

(a*S*,1*R*,3*S*,2'*S*)-**20** (141 mg, 0.27 mmol, 1 equiv.) was dissolved in anhydrous toluene (3 ml), and the solution was cooled to 0 °C. Then ethyl-diethoxyacetate (96 µl, 94 mg, 0.54 mmol, 2 equiv.) and $BF_3 \cdot Et_2O$ (10 µl, 11 mg, 0.08 mmol, 0.3 equiv.) were added to the solution, and the reaction was allowed to warm up to room temperature. After stirring overnight, another portion of $BF_3 \cdot Et_2O$ (10 µl, 11 mg, 0.08 mmol, 0.3 equiv.) was added to the solution and stirred for overnight. The reaction was stirred for 4 hours at 60 °C and allowed to cool down to room temperature. After another day, the reaction was quenched with a saturated solution of $NaHCO_3$ and stirred for 10 minutes. EtOAc was added to the mixture and the layers were separated. The organic layer was extracted twice with a saturated solution of $NaHCO_3$ and once with brine. The organic phase was dried over anhydrous Na_2SO_4, filtered, and the solvent was evaporated in *vacuo*. The residue was purified by flash chromatography (hexane/EtOAc 85:15 → 6:4). (a*S*,1*R*,3*S*,1'*S*,3'*S*)-**22**: 37 mg (yield: 23%) white amorphous solid foam; $[\alpha]_D^{20}$ −49; $[\alpha]_{578}^{20}$ −52;

$[\alpha]^{20}_{546}$ −62; $[\alpha]^{20}_{436}$ −71; $[\alpha]^{20}_{365}$ −261 (*c* = 0.49; MeCN); ECD (*c* = 1.93×10^{-4} M; MeCN) λ [nm], (Δε) = 288 (−1.69), 271sh (−1.44), 252sh (−6.17), 225 (−27.82), 201 (33.46).

(a*S*,1*R*,3*S*,1'*S*,3'*S*)-**22**: R_f = 0.21 (hexane/EtOAc 8:2); ^{1}H NMR (400 MHz, $CDCl_3$) *δ* = 7.40 – 7.29 (m, 2H, *H-14*, *H-18*), 7.09 – 6.98 (m, 2H, *H-15*, *H-17*), 6.72 (s, 1H, *H-6'*), 5.82 (s, 1H, *H-1*), 5.47 (s, 1H, *H-1'*), 4.36 – 4.18 (m, 2H, *H-13'*), 3.88 (s, 3H, *H-10'*), 3.87 (s, 3H, *H-9'*), 3.80 (s, 3H, *H-10*), 3.73 – 3.61 (m, 2H, *H-3*, *H-3'*), 3.54 (s, 3H, *H-9*), 3.15 (s, 3H, *H-11*), 2.56 (ddd, *J* = 16.2, 11.3, 0.9 Hz, 1H, $H\text{-}4'_{ax}$), 2.43 (ddd, *J* = 16.3, 10.9, 1.4 Hz, 1H, $H\text{-}4_{ax}$), 1.99 (dd, *J* = 16.0, 2.5 Hz, 1H, $H\text{-}4'_{eq}$), 1.97 (d, *J* = 16.2 Hz, 1H, $H\text{-}4_{eq}$), 1.32 (t, *J* = 7.2 Hz, 3H, *H-14'*), 1.29 (d, *J* = 6.1 Hz, 3H, *H-11'*), 1.23 (d, *J* = 6.1 Hz, 3H, *H-12*); ^{13}C NMR (100 MHz, $CDCl_3$) *δ* = 171.0 (1C, *C-12'*), 162.3 (d, $J_{C\text{-}F}$ = 245.3 Hz, 1C, *C-16*), 151.2 (1C, *C-6*), 149.9 (2C, *C-7'*, *C-8*), 144.6 (1C, *C-7*), 144.5 (1C, *C-8'*), 140.2 (d, $J_{C\text{-}F}$ = 2.9 Hz, 1C, *C-13*), 130.5 (1C, *C-5'*), 130.2 (d, $J_{C\text{-}F}$ = 8.1 Hz, 2C, *C-14*, *C-18*), 129.2 (1C, *C-4a*), 128.0 (1C, *C-5*), 127.0 (1C, *C-4a'*), 126.7 (1C, *C-8a*), 126.6 (1C, *C-8a'*), 115.1 (d, $J_{C\text{-}F}$ = 21.4 Hz, 2C, *C-15*, *C-17*), 114.3 (1C, *C-6'*), 77.6 (1C, *C-1*), 75.6 (1C, *C-1'*), 71.2 (1C, *C-3'*), 70.6 (1C, *C-3*), 61.4 (1C, *C-13'*), 60.9 (1C, *C-9*), 60.8 (1C, *C-10*), 60.1 (1C, *C-10'*), 59.4 (1C, *C-11*), 56.2 (1C, *C-9'*), 35.8 (1C, *C-4*), 33.4 (1C, *C-4'*), 21.9 (1C, *C-12*), 21.7 (1C, *C-11'*), 14.3 (1C, *C-14'*); IR (KBr) ν = 2973, 2936 (ν_{as} Me, ν_{as} CH_2), 2849 (ν_s CH_2), 1746 (ν C=O), 1604, 1576, 1509, 1492, 1463, 1420 (ν Ar C=C, β_s CH_2, δ_{as} Me), 1384, 1361, 1318 (δ_s Me, δ CH, γ_s CH_2), 1257, 1232, 1183, 1150, 1116 (ν_{as} Ar-O-Me, ν Ar C-F, ν_{as} C-O-C=O), 1068, 1029 (ν_{as} C-O-C, ν_s Ar-O-Me, ν_s C-O-C=O) cm^{-1}; ESI-TOF-HRMS: *m/z* calculated for $C_{34}H_{39}FNaO_9$ $[M+Na]^+$ 633.2470, found 633.2470.

Epimerization of C-1 caused by $BF_3 \cdot Et_2O$ also resulted in (a*S*,1*S*,3*S*,1'*S*,3'*S*)-**22** (52 mg, yield 32%) as pale yellow amorphous solid foam. Characterization is disclosed elsewhere.

(a*R*,1*S*,3*R*,1'*R*,3'*R*)-ethyl 1-(4-fluorophenyl)-6,7,7',8,8'-pentamethoxy-3,3'-dimethyl-[5,5'-*bis*-isochroman]-1'-carboxylate [(a*R*,1*S*,3*R*,1'*R*,3'*R*)-22] and (a*R*,1*R*,3*R*,1'*R*,3'*R*)-ethyl 1-(4-fluorophenyl)-6,7,7',8,8'-pentamethoxy-3,3'-dimethyl-[5,5'-*bis*-isochroman]-1'-carboxylate [(a*R*,1*R*,3*R*,1'*R*,3'*R*)-22]

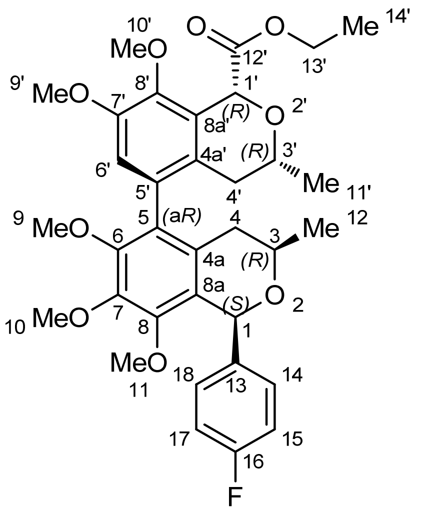

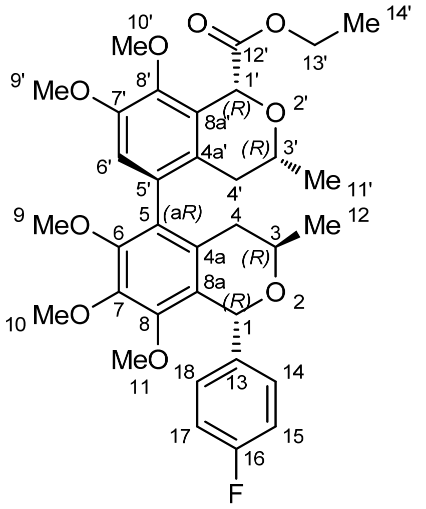

(a*R*,1*S*,3*R*,2'*R*)-**20** (323 mg, 0.61 mmol, 1 equiv.) was dissolved in anhydrous toluene (7 ml), and the solution was cooled to 0 °C. Then ethyl-diethoxyacetate (220 µl, 216 mg, 1.22 mmol, 2 equiv.) and $BF_3 \cdot Et_2O$ (23 µl, 26 mg, 0.18 mmol, 0.3 equiv.) were added to the solution, and the reaction were allowed to warm up to room temperature. After stirring overnight, another portion of $BF_3 \cdot Et_2O$ (11 µl, 13 mg, 0.09 mmol, 0.15 equiv.) was added to the solution and the reaction was stirred for 8 hours at 60 °C. Then it was allowed to cool down to room temperature. After stirring overnight, the mixture was quenched with a saturated solution of $NaHCO_3$ and stirred for 10 minutes. EtOAc was added to the mixture and the layers were separated. The organic layer was extracted twice with a saturated solution of $NaHCO_3$ and once with brine. The organic phase was dried over anhydrous Na_2SO_4, filtered, and the solvent was evaporated in *vacuo*. The residue was purified by flash chromatography. (a*R*,1*S*,3*R*,1'*R*,3'*R*)-**22**: 62 mg (yield: 17%) white amorphous solid foam; $[\alpha]_D^{20}$ +46; $[\alpha]_{578}^{20}$ +49; $[\alpha]_{546}^{20}$ +58; $[\alpha]_{436}^{20}$ +70; $[\alpha]_{365}^{20}$ +276 (*c* = 0.48; MeCN); ECD (*c* = 1.59×10^{-4} M; MeCN) λ [nm], (Δε) = 288 (1.55), 271sh (1.41), 251sh (6.08), 224 (26.63), 201 (−35.87).

Epimerization of C-1 caused by $BF_3 \cdot Et_2O$ also resulted in (a*R*,1*R*,3*R*,1'*R*,3'*R*)-**22** (126 mg, yield 34%) as pale yellow amorphous solid foam. Chromatographic and spectral data except for the chiroptical ones were identical with those of the corresponding enantiomers (a*S*,1*R*,3*S*,1'*S*,3'*S*)-**22** and (a*S*,1*S*,3*S*,1'*S*,3'*S*)-**22**.

(a*S*,1*S*,3*S*,3'*S*)-1-(4-fluorophenyl)-6,7,7',8,8'-pentamethoxy-3,3'-dimethyl-5,5'-*bis*-isochroman [(a*S*,1*S*,3*S*,3'*S*)-21] and (a*S*,1*S*,3*S*,1'*S*,3'*S*)-ethyl 1-(4-fluorophenyl)-6,7,7',8,8'-pentamethoxy-3,3'-dimethyl-[5,5'-*bis*-isochroman]-1'-carboxylate [(a*S*,1*S*,3*S*,1'*S*,3'*S*)-22]

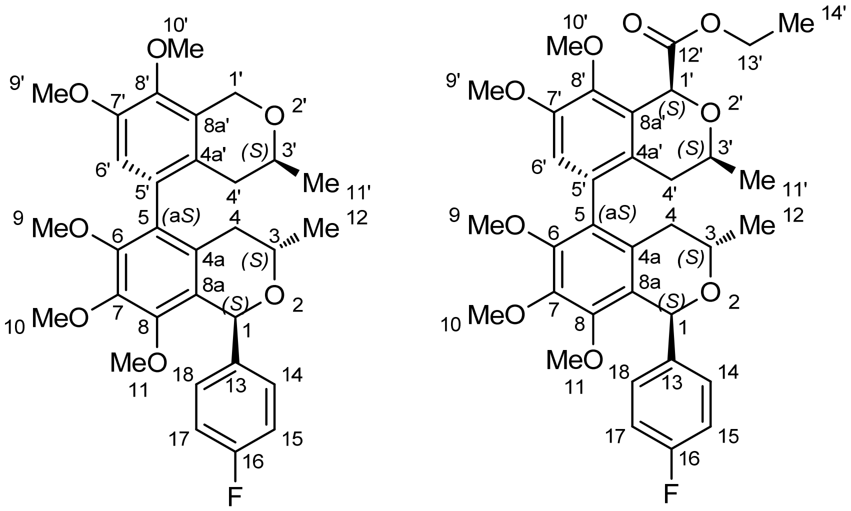

(a*S*,1*S*,3*S*,2'*S*)-**20** (141 mg, 0.27 mmol, 1.0 equiv.) was dissolved in anhydrous toluene (3 ml), and the solution was cooled to 0 °C. Then ethyl-diethoxyacetate (96 µl, 94 mg, 0.53 mmol, 2.0 equiv.) and $BF_3 \cdot Et_2O$ (10 µl, 11 mg, 0.08 mmol, 0.3 equiv.) were added to the solution, and the reaction was allowed to warm up to room temperature. After four days, another portion of ethyl-diethoxyacetate (96 µl, 94 mg, 0.53 mmol, 2.0 equiv.) was added to the reaction mixture. The temperature was raised to 60 °C. After stirring overnight, the reaction was allowed to cool down to room temperature and stirred for another day. The reaction was quenched with a saturated solution of $NaHCO_3$ and stirred for 10 minutes. EtOAc was added to the mixture and the layers were separated. The organic layer was extracted twice with a saturated solution of $NaHCO_3$ and once with brine. The organic phase was dried over anhydrous Na_2SO_4, filtered, and the solvent was evaporated in *vacuo*. The residue was purified by flash chromatography (hexane/EtOAc 95:5 → 9:1 → 85:15→ 75:25 → 7:3) to yield (a*S*,1*S*,3*S*,1'*S*,3'*S*)-**22** (62 mg, 38%) as white amorphous solid foam; $[\alpha]_D^{20}$ +42; $[\alpha]_{578}^{20}$ +43; $[\alpha]_{546}^{20}$ +49; $[\alpha]_{436}^{20}$ +55; $[\alpha]_{365}^{20}$ +119 (*c* = 0.50; MeCN); ECD (*c* = $1.3977 \cdot 10^{-4}$ M; MeCN) λ [nm], (Δε) = 291 (−0.44), 270 (−1.19), 264sh (−2.05), 239sh (−7.35), 231 (−7.84), 217 (−4.58), 197 (36.15).

(a*S*,1*S*,3*S*,1'*S*,3'*S*)-**22**: R_f = 0.35 (hexane/EtOAc 75:25); ^{1}H NMR (400 MHz, $CDCl_3$) δ = 7.22 – 7.16 (m, 2H, *H-14*, *H-18*), 7.06 – 6.99 (m, 2H, *H-15*, *H-17*), 6.67 (s, 1H, *H-6'*), 6.03 (s, 1H, *H-1*), 5.46 (s, 1H, *H-1'*), 4.36 – 4.19 (m, 2H, *H-13'*), 3.89 (s, 3H, *H-10*), 3.87 (s, 3H, *H-10'*), 3.82 (s, 3H, *H-9'*), 3.73 – 3.65 (m, 1H, *H-3'*), 3.63 (s, 3H, *H-9*), 3.62 – 3.58 (m, 1H, *H-3*), 3.57 (s, 3H, *H-11*), 2.54 (ddd, *J* = 16.1, 11.1 Hz, 1.3 Hz, 1H, $H\text{-}4'_{ax}$), 2.26 (dd, *J* = 17.0, 11.0 Hz, 1H, $H\text{-}4_{ax}$), 2.08 (dd, *J* = 16.1, 2.1 Hz, 1H, $H\text{-}4'_{eq}$), 1.93 (dd, *J* = 17.1, 3.5 Hz, 1H, $H\text{-}4_{eq}$), 1.33 (t, *J* = 7.1 Hz, 3H, *H-14'*), 1.33 (d, *J* = 6.1 Hz, 3H, *H-11'*), 1.08 (d, *J* = 6.1 Hz, 3H, *H-12*); ^{13}C NMR (100 MHz, $CDCl_3$) δ = 170.9 (1C, *C-12'*), 162.3 (d, $J_{C\text{-}F}$ = 246.1 Hz, 1C, *C-16*), 151.5 (1C, *C-6*), 150.1 (1C, *C-7'*), 149.5 (1C, *C-8*), 144.4 (1C, *C-8'*), 144.2 (1C, *C-7*), 138.3 (d, $J_{C\text{-}F}$

= 3.0 Hz, 1C, *C-13*), 130.5 (1C, *C-5'*), 130.3 (d, J_{C-F} = 8.0 Hz, 2C, *C-14*, *C-18*), 128.4 (1C, *C-5*), 128.3 (1C, *C-4a*), 126.6 (1C, *C-8a'*), 126.2 (1C, *C-4a'*), 124.2 (1C, *C-8a*), 114.9 (d, J_{C-F} = 21.3 Hz, 2C, *C-15*, *C-17*), 113.9 (1C, *C-6'*), 75.6 (1C, *C-1'*), 73.4 (1C, *C-1*), 71.2 (1C, *C-3'*), 63.2 (1C, *C-3*), 61.4 (1C, *C-13'*), 61.1 (1C, *C-9*), 60.9 (1C, *C-10*), 60.2 (1C, *C-11*), 60.1 (1C, *C-10'*), 56.0 (1C, *C-9'*), 34.5 (1C, *C-4*), 33.6 (1C, *C-4'*), 21.8 (2C, *C-11'*, *C-12*), 14.2 (1C, *C-14'*); IR (KBr) ν = 2974, 2935 (ν_{as} Me, ν_{as} CH_2), 2867 (ν_s Me), 1746 (ν C=O), 1603, 1579, 1507, 1492, 1464, 1420, 1407 (ν Ar C=C, β_s CH_2, δ_{as} Me), 1384, 1363, 1317 (δ_s Me, δ CH, γ_s CH_2), 1282, 1255, 1223, 1182, 1158, 1112 (ν_{as} Ar-O-Me, ν Ar C-F, ν_{as} C-O-C=O), 1066, 1028 (ν_{as} C-O-C, ν_s Ar-O-Me, ν_s C-O-C=O) cm^{-1}; ESI-TOF-HRMS: *m/z* calculated for $C_{34}H_{39}FNaO_9$ $[M+Na]^+$ 633.2470, found 633.2472.

Hydrolysis of ethyl ester and subsequent decarboxylation of C-1' caused by $BF_3{\cdot}Et_2O$ also resulted in (a*S*,1*S*,3*S*,3'*S*)-**21** (42 mg, 29%) as white amorphous solid foam. Characterization is disclosed elsewhere.

(a*R*,1*R*,3*R*,1'*R*,3'*R*)-ethyl 1-(4-fluorophenyl)-6,7,7',8,8'-pentamethoxy-3,3'-dimethyl-[5,5'-*bis*-isochroman]-1'-carboxylate [(a*R*,1*R*,3*R*,1'*R*,3'*R*)-22]

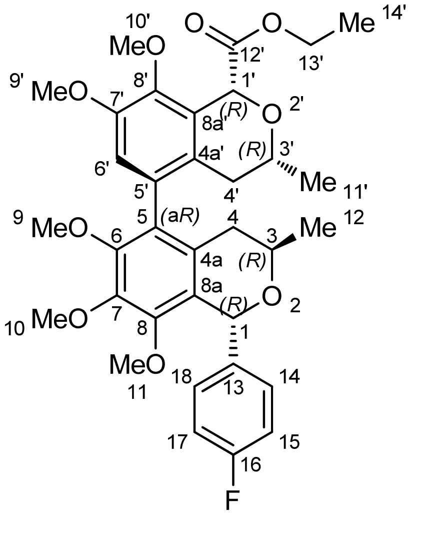

(a*R*,1*R*,3*R*,2'*R*)-**20** (100 mg, 0.19 mmol, 1 equiv.) was dissolved in anhydrous toluene (2 ml), and the solution was cooled to 0 °C. Then ethyl-diethoxyacetate (68 µl, 67 mg, 0.38 mmol, 2 equiv.) and $BF_3{\cdot}Et_2O$ (7 µl, 8 mg, 0.06 mmol, 0.3 equiv.) were added to the solution, and the reaction were allowed to warm up to room temperature. After stirring overnight, another portion of $BF_3{\cdot}Et_2O$ (4 µl, 4 mg, 0.03 mmol, 0.15 equiv.) was added to the solution and the reaction was stirred for 8 hours at 60 °C. Then it was allowed to cool down to room temperature. After stirring overnight, the reaction was quenched with a saturated solution of $NaHCO_3$ and stirred for 10 minutes. EtOAc was added to the mixture and the layers were separated. The organic

layers were extracted twice with a saturated solution of $NaHCO_3$ and once with brine. The organic phase was dried over anhydrous Na_2SO_4, filtered, and the solvent was evaporated in *vacuo*. The residue was purified by flash chromatography to yield (a*R*,1*R*,3*R*,1'*R*,3'*R*)-**22** (45 mg, 39%) as a white amorphous solid foam; $[\alpha]_D^{20}$ −36; $[\alpha]_{578}^{20}$ −38; $[\alpha]_{546}^{20}$ −42; $[\alpha]_{436}^{20}$ −57; $[\alpha]_{365}^{20}$ −72 (*c* = 0.44; MeCN); ECD (*c* = 1.7170·10^{-4} M; MeCN) λ [nm], (Δε) = 288 (0.50), 270 (1.04), 263sh (1.85), 239sh (6.54), 230 (7.19), 215 (3.56), 195 (−29.62). Chromatographic and spectral data except for the chiroptical ones were identical with those of (a*S*,1*S*,3*S*,1'*S*,3'*S*)-**22**.

(a*R*,1*S*,3*S*,1'*S*,3'*S*)-ethyl 1-(4-fluorophenyl)-6,7,7',8,8'-pentamethoxy-3,3'-dimethyl-[5,5'-*bis*-isochroman]-1'-carboxylate [(a*R*,1*S*,3*S*,1'*S*,3'*S*)-22]

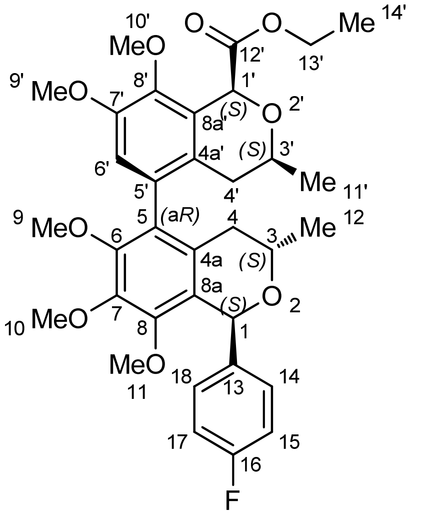

(a*R*,1*S*,3*S*,2'*S*)-**20** (143 mg, 0.27 mmol, 1 equiv.) was dissolved in anhydrous toluene (3 ml), and the solution was cooled to 0 °C. Then ethyl-diethoxyacetate (97 µl, 95 mg, 0.54 mmol, 2 equiv.) and $BF_3 \cdot Et_2O$ (10 µl, 12 mg, 0.08 mmol, 0.3 equiv.) were added to the solution. After stirring for three days at room temperature, the reaction was quenched with a saturated solution of $NaHCO_3$ and stirred for 10 minutes. EtOAc was added to the mixture and the layers were separated. The organic layers were extracted twice with a saturated solution of $NaHCO_3$ and once with brine. The organic phase was dried over anhydrous Na_2SO_4, filtered, and the solvent was evaporated in *vacuo*. The residue was purified by flash chromatography to yield (a*R*,1*S*,3*S*,1'*S*,3'*S*)-**22** (118 mg, 71%) as white amorphous solid foam; $[\alpha]_D^{20}$ +52; $[\alpha]_{578}^{20}$ +54; $[\alpha]_{546}^{20}$ +62; $[\alpha]_{436}^{20}$ +91; $[\alpha]_{365}^{20}$ +174 (*c* = 0.48; MeCN); ECD (*c* = 1.67×10^{-4} M; MeCN) λ [nm], (Δε) = 287 (0.44), 279sh (0.18), 270 (−0.48), 264 (−0.69), 257sh (−0.65), 242sh (−3.31), 231 (−4.71), 205 (25.83). Flash chromatography (hexane/EtOAc 9:1 → 85:15); R_f = 0.32 (hexane/EtOAc 75:25); ^1H NMR (400 MHz, $CDCl_3$) δ = 7.28 – 7.22 (m, 2H, *H-14*, *H-18*), 7.06 – 6.99 (m, 2H, *H-15*, *H-17*), 6.68 (s, 1H, *H-6'*), 6.02 (s, 1H, *H-1*), 5.45 (s, 1H, *H-1'*), 4.34 –

4.20 (m, 2H, *H-13'*), 3.89 (s, 3H, *H-10*), 3.87 (s, 3H, *H-10'*), 3.85 (s, 3H, *H-9'*), 3.78 – 3.72 (m, 1H, *H-3'*), 3.71 – 3.60 (m, 1H, *H-3*), 3.64 (s, 3H, *H-9*), 3.57 (s, 3H, *H-11*), 2.32 (dd, J = 15.9, 11.3 Hz, 1H, $H\text{-}4'_{ax}$), 2.29 (dd, J = 17.1, 3.3 Hz, 1H, $H\text{-}4_{eq}$), 2.23 (dd, J = 16.0, 2.7 Hz, 1H, $H\text{-}4'_{eq}$), 2.11 (dd, J = 17.0, 10.8 Hz, 1H, $H\text{-}4_{ax}$), 1.33 (t, J = 7.1 Hz, 3H, *H-14'*), 1.30 (d, J = 6.0 Hz, 3H, *H-11'*), 1.09 (d, J = 6.1 Hz, 3H, *H-12*); ^{13}C NMR (100 MHz, $CDCl_3$) δ = 171.0 (1C, *C-12'*), 162.3 (d, $J_{C\text{-}F}$ = 245.9 Hz, 1C, *C-16*), 150.4 (1C, *C-6*), 150.0 (1C, *C-7'*), 149.5 (1C, *C-8*), 144.4 (1C, *C-8'*), 143.8 (1C, *C-7*), 138.3 (d, $J_{C\text{-}F}$ = 2.9 Hz, 1C, *C-13*), 130.8 (1C, *C-5'*), 130.3 (d, $J_{C\text{-}F}$ = 8.0 Hz, 2C, *C-14*, *C-18*), 129.3 (1C, *C-4a*), 128.9 (1C, *C-5*), 126.7 (2C, *C-8a'*, *C-4a'*), 124.5 (1C, *C-8a*), 114.8 (d, $J_{C\text{-}F}$ = 21.2 Hz, 2C, *C-15*, *C-17*), 113.7 (1C, *C-6'*), 75.6 (1C, *C-1'*), 73.4 (1C, *C-1*), 71.3 (1C, *C-3'*), 63.4 (1C, *C-3*), 61.3 (1C, *C-13'*), 60.9 (1C, *C-9*), 60.8 (1C, *C-10*), 60.1 (1C, *C-10'*); 60.0 (1C, *C-11*), 56.1 (1C, *C-9'*), 33.3 (2C, *C-4'*, *C-4*), 21.6 (2C, *C-11'*, *C-12*), 14.2 (1C, *C-14'*); IR (KBr) ν = 2974, 2936 (ν_{as} Me, ν_{as} CH_2), 2898 (ν_s Me), 1747 (ν C=O), 1602, 1579, 1507, 1492, 1464, 1421, 1407 (ν Ar C=C, β_s CH_2, δ_{as} Me), 1385, 1362, 1315 (δ_s Me, δ CH, γ_s CH_2), 1282, 1266, 1233, 1183, 1159, 1127, 1113 (ν_{as} Ar-O-Me, ν Ar C-F, ν_{as} C-O-C=O), 1095, 1066, 1053, 1029 (ν_{as} C-O-C, ν_s Ar-O-Me, ν_s C-O-C=O), 872 (1,2,3,4,5-pentasubstituated γ_s Ar =CH) cm^{-1}; ESI-TOF-HRMS: *m/z* calculated for $C_{34}H_{39}FNaO_9$ $[M+Na]^+$ 633.2470, found 633.2461.

(a*S*,1*R*,3*R*,1'*R*,3'*R*)-ethyl 1-(4-fluorophenyl)-6,7,7',8,8'-pentamethoxy-3,3'-dimethyl-[5,5'-*bis*-isochroman]-1'-carboxylate [(a*S*,1*R*,3*R*,1'*R*,3'*R*)-22]

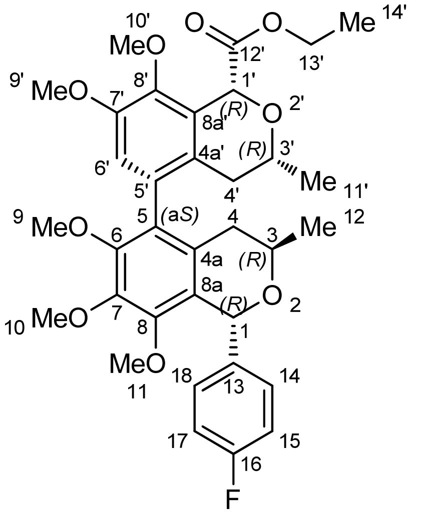

(a*S*,1*R*,3*R*,2'*R*)-**20** (100 mg, 0.19 mmol, 1 equiv.) was dissolved in anhydrous toluene (2 ml), and the solution was cooled to 0 °C. Then ethyl-diethoxyacetate (68 µl, 67 mg, 0.38 mmol, 2 equiv.) and $BF_3{\cdot}Et_2O$ (7 µl, 8 mg, 0.06 mmol, 0.3 equiv.) were added to the solution. After stirring for 24 hours at room temperature, the reaction was quenched with a saturated solution

of $NaHCO_3$ and stirred for 10 minutes. EtOAc was added to the mixture and the layers were separated. The organic layers were extracted twice with a saturated solution of $NaHCO_3$ and once with brine. The organic phase was dried over anhydrous Na_2SO_4, filtered, and the solvent was evaporated in *vacuo*. The residue was purified by flash to yield (a*S*,1*R*,3*R*,1'*R*,3'*R*)-**22** (40 mg, 34%) as white amorphous solid foam; $[\alpha]_D^{20}$ −45; $[\alpha]_{578}^{20}$ −48; $[\alpha]_{546}^{20}$ −54; $[\alpha]_{436}^{20}$ −90; $[\alpha]_{365}^{20}$ −147 (*c* = 0.32; MeCN); ECD (*c* = 1.59×10^{-4} M; MeCN) λ [nm], (Δε) = 287 (−0.34), 279sh (−0.14), 271 (0.45), 264 (0.66), 257sh (0.69), 239sh (3.48), 227 (4.95), 204 (−25.22). Chromatographic and spectral data except for the chiroptical ones were identical with those of (a*R*,1*S*,3*S*,1'*S*,3'*S*)-**22**.

Ethyl 2'-{(a*S*,1*S*,3*S*,1'*R*,3'*S*)-1-(4-fluorophenyl)-6,7,7',8,8'-pentamethoxy-3,3'-dimethyl-[5,5'-*bis*-isochroman]-1'-yl}acetate [(a*S*,1*S*,3*S*,1'*R*,3'*S*)-23]

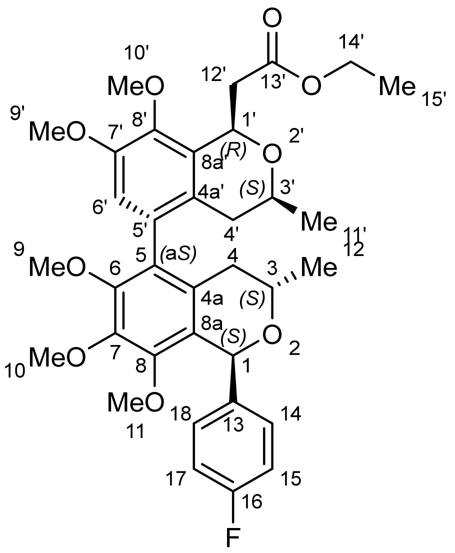

Starting from (a*S*,1*S*,3*S*,2'*S*)-**20** (132 mg, 0.25 mmol, 1.0 equiv.) using **method C**: anhydrous toluene (3 ml), ethyl-3,3-diethoxypropionate (90%, technical grade, 108 µl = 98 µl, 95 mg, 0.50 mmol, 2.0 equiv.), $BF_3{\cdot}Et_2O$ (9 µl, 11 mg, 0.08 mmol, 0.3 equiv.), reaction time: 4 hours. (a*S*,1*S*,3*S*,1'*R*,3'*S*)-**23**: 141 mg (yield: 90%), white amorphous solid foam; $[\alpha]_D^{20}$ +80; $[\alpha]_{578}^{20}$ +83; $[\alpha]_{546}^{20}$ +95; $[\alpha]_{436}^{20}$ +125; $[\alpha]_{365}^{20}$ +223 (*c* = 0.51; MeCN); ECD (*c* = 1.82×10^{-4} M; MeCN) λ [nm], (Δε) = 292 (−0.47), 270 (−1.26), 264sh (−2.30), 242 (−7.40), 220 (3.33), 199 (28.46). Flash chromatography (hexane/EtOAc 9:1 → 85:15 → 8:2 → 75:25 → 7:3); R_f = 0.40 (hexane/EtOAc 75:25); ^{1}H NMR (400 MHz, $CDCl_3$) δ = 7.23 – 7.16 (m, 2H, *H-14*, *H-18*), 7.06 – 6.99 (m, 2H, *H-15*, *H-17*), 6.64 (s, 1H, *H-6'*), 6.04 (s, 1H, *H-1*), 5.44 (dd, *J* = 8.2, 2.9 Hz, 1H, *H-1'*), 4.20 (q, *J* = 7.1 Hz, 2H, *H-14'*), 3.91 (s, 3H, *H-10'*), 3.89 (s, 3H, *H-10*), 3.84 (s, 3H, *H-9'*), 3.66 (s, 3H, *H-9*), 3.64 – 3.58 (m, 2H, *H-3*, *H-3'*), 3.57 (s, 3H, *H-11*), 3.31 (dd, *J* = 15.2,

3.2 Hz, 1H, *H-12'-a*), 2.66 (dd, *J* = 15.2, 8.3 Hz, 1H, *H-12'-b*), 2.42 (dd, *J* = 15.5, 10.9 Hz, 1H, *H-4'ax*), 2.27 (dd, *J* = 17.1, 11.1 Hz, 1H, *H-4ax*), 2.03 (d, *J* = 15.5 Hz, 1H, *H-4'eq*), 1.90 (dd, *J* = 17.1, 3.4 Hz, 1H, *H-4eq*), 1.28 (t, *J* = 7.1 Hz, 3H, *H-15'*), 1.24 (d, *J* = 6.1 Hz, 3H, *H-11'*), 1.09 (d, *J* = 6.1 Hz, 3H, *H-12*); ^{13}C NMR (100 MHz, $CDCl_3$) δ = 171.9 (1C, *C-13'*), 162.2 (d, $J_{C\text{-}F}$ = 246.0 Hz, 1C, *C-16*), 151.3 (1C, *C-6*), 150.3 (1C, *C-7'*), 149.3 (1C, *C-8*), 144.5 (1C, *C-8'*), 144.1 (1C, *C-7*), 138.2 (d, $J_{C\text{-}F}$ = 2.8 Hz, 1C, *C-13*), 130.6 (1C, *C-5'*), 130.4 (1C, *C-8a'*), 130.2 (d, $J_{C\text{-}F}$ = 8.0 Hz, 2C, *C-14*, *C-18*), 128.7 (1C, *C-5*), 128.2 (1C, *C-4a*), 126.8 (1C, *C-4a'*), 124.1 (1C, *C-8a*), 114.8 (d, $J_{C\text{-}F}$ = 8.0 Hz, 2C, *C-15*, *C-17*), 112.8 (1C, *C-6'*), 73.3 (1C, *C-1*), 71.6 (1C, *C-1'*), 69.9 (1C, *C-3'*), 63.1 (1C, *C-3*), 61.0 (1C, *C-9*), 60.7 (1C, *C-10*), 60.3 (1C, *C-10'*), 60.2 (1C, *C-14'*), 60.0 (1C, *C-11*), 55.9 (1C, *C-9'*), 41.9 (1C, *C-12'*), 34.6 (1C, *C-4'*), 34.4 (1C, *C-4*), 21.7 (2C, *C-11'*, *C-12*), 14.3 (1C, *C-15'*); IR (KBr) ν = 2973, 2936 (ν_{as} Me, ν_{as} CH_2), 2841 (ν_s CH_2), 1737 (ν C=O), 1602, 1579, 1507, 1486, 1464, 1420 (ν Ar C=C, β_s CH_2, δ_{as} Me), 1383, 1363, 1314 (δ_s Me, δ CH, γ_s CH_2), 1280, 1254, 1223, 1174, 1159, 1112 (ν_{as} Ar-O-Me, ν Ar C-F, ν_{as} C-O-C=O), 1093, 1067, 1027 (ν_{as} C-O-C, ν_s Ar-O-Me, ν_s C-O-C=O) cm^{-1}; ESI-TOF-HRMS: *m/z* calculated for $C_{35}H_{41}FNaO_9$ $[M+Na]^+$ 647.2627, found 647.2626.

Ethyl 2'-{(a*R*,1*R*,3*R*,1'*S*,3'*R*)-1-(4-fluorophenyl)-6,7,7',8,8'-pentamethoxy-3,3'-dimethyl-[5,5'-*bis*-isochroman]-1'-yl}acetate [(a*R*,1*R*,3*R*,1'*S*,3'*R*)-23]

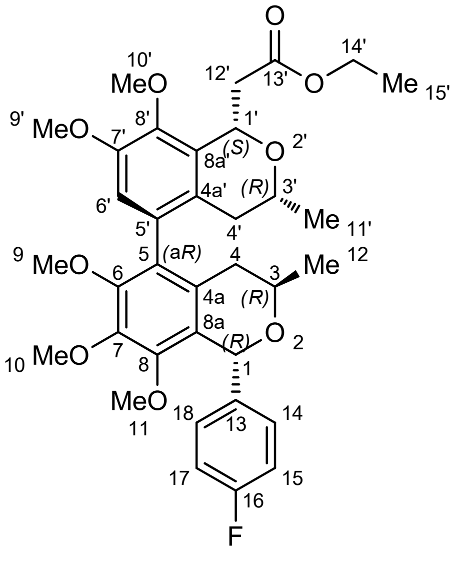

Starting from (a*R*,1*R*,3*R*,2'*R*)-**20** (170 mg, 0.32 mmol, 1.0 equiv.) using **method C**: anhydrous toluene (4 ml), ethyl-3,3-diethoxypropionate (90%, technical grade, 140 µl = 126 µl, 123 mg, 0.65 mmol, 2.0 equiv.), $BF_3{\cdot}Et_2O$ (14 µl, 12 mg, 0.10 mmol, 0.3 equiv.), reaction time: 4 hours. (a*R*,1*R*,3*R*,1'*S*,3'*R*)-**23**: 140 mg (yield: 69%), white amorphous solid foam; $[\alpha]_D^{20}$ −75; $[\alpha]_{578}^{20}$ −78; $[\alpha]_{546}^{20}$ −88; $[\alpha]_{436}^{20}$ −121; $[\alpha]_{365}^{20}$ −202 (*c* = 0.51; MeCN); ECD (*c* = 1.83×10^{-4} M; MeCN) λ

[nm], (Δε) = 291 (0.45), 270 (1.12), 264sh (2.30), 244 (6.93), 220 (−2.65), 200 (−26.80). Chromatographic and spectral data except for the chiroptical ones were identical with those of (a*S*,1*S*,3*S*,1'*R*,3'*S*)-**23**.

Ethyl 2'-{(a*R*,1*S*,3*S*,1'*R*,3'*S*)-1-(4-fluorophenyl)-6,7,7',8,8'-pentamethoxy-3,3'-dimethyl-[5,5'-*bis*-isochroman]-1'-yl}acetate [(a*R*,1*S*,3*S*,1'*R*,3'*S*)-23]

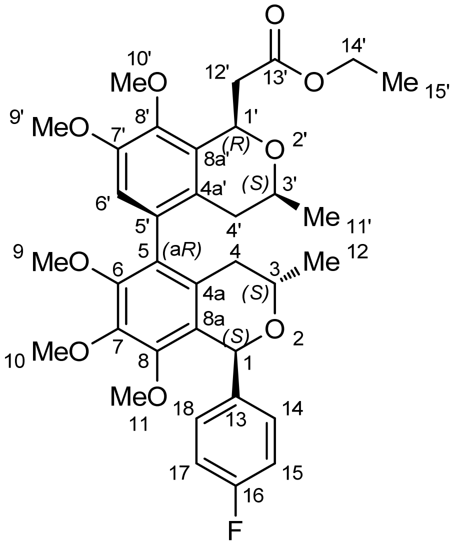

Starting from (a*R*,1*S*,3*S*,2'*S*)-**20** (140 mg, 0.27 mmol, 1.0 equiv.) using **method C**: anhydrous toluene (3 ml), ethyl-3,3-diethoxypropionate (90%, technical grade, 115 µl = 103 µl, 101 mg, 0.53 mmol, 2.0 equiv.), $BF_3 \cdot Et_2O$ (10 µl, 11 mg, 0.08 mmol, 0.3 equiv.), reaction time: 3 hours. (a*R*,1*S*,3*S*,1'*R*,3'*S*)-**23**: 156 mg (yield: 94%), white amorphous solid foam; $[\alpha]_D^{20}$ +72; $[\alpha]_{578}^{20}$ +75; $[\alpha]_{546}^{20}$ +86; $[\alpha]_{436}^{20}$ +122; $[\alpha]_{365}^{20}$ +233 (*c* = 0.50; MeCN); ECD (*c* = 1.66×10^{-4} M; MeCN) λ [nm], (Δε) = 287 (0.18), 270 (−0.61), 264 (−0.97), 257sh (−1.29), 242sh (−4.07), 233 (−4.18), 206 (27.41). Flash chromatography (hexane/EtOAc 9:1 → 85:15 → 8:2 → 7:3 → 6:4); R_f = 0.49 (hexane/EtOAc 75:25); ^{1}H NMR (400 MHz, $CDCl_3$) *δ* = 7.28 – 7.22 (m, 2H, *H-14*, *H-18*), 7.06 – 6.98 (m, 2H, *H-15*, *H-17*), 6.65 (s, 1H, *H-6'*), 6.02 (s, 1H, *H-1*), 5.43 (dd, *J* = 8.0, 3.1 Hz, 1H, *H-1'*), 4.18 (q, *J* = 7.1, 2H, *H-14'*), 3.90 (s, 3H, *H-10'*), 3.89 (s, 3H, *H-10*), 3.87 (s, 3H, *H-9'*), 3.73 – 3.60 (m, 2H, *H-3'*, *H-3*), 3.66 (s, 3H, *H-9*), 3.57 (s, 3H, *H-11*), 3.32 (dd, *J* = 15.1, 3.4 Hz, 1H, *H-12'-a*), 2.66 (dd, *J* = 15.1, 8.1 Hz, 1H, *H-12'-b*), 2.24 (dd, *J* = 17.0, 3.6 Hz, 1H, $H\text{-}4_{eq}$), 2.22 – 2.15 (m, 2H, *H-4'*), 2.11 (dd, *J* = 17.0, 10.7 Hz, 1H, $H\text{-}4_{ax}$), 1.27 (t, *J* = 7.1 Hz, 3H, *H-15'*), 1.21 (d, *J* = 6.1 Hz, 3H, *H-11'*), 1.09 (d, *J* = 6.1 Hz, 3H, *H-12*); ^{13}C NMR (100 MHz, $CDCl_3$) *δ* = 171.8 (1C, *C-13'*), 162.2 (d, $J_{C\text{-}F}$ = 245.9 Hz, 1C, *C-16*), 150.3 (1C, *C-6*), 150.2 (1C, *C-7'*), 149.3 (1C, *C-8*), 144.6 (1C, *C-8'*), 143.8 (1C, *C-7*), 138.2 (d, $J_{C\text{-}F}$ = 2.9 Hz, 1C, *C-13*), 130.9 (1C, *C-5'*), 130.5 (1C, *C-8a'*), 130.3 (d, $J_{C\text{-}F}$ = 8.0 Hz, 2C, *C-14*, *C-18*), 129.2 (2C,

C-4a, *C-5*), 127.4 (1C, *C-4a'*), 124.4 (1C, *C-8a*), 114.7 (d, $J_{C\text{-}F}$ = 21.2 Hz, 2C, *C-15*, *C-17*), 112.7 (1C, *C-6'*), 73.4 (1C, *C-1*), 71.7 (1C, *C-1'*), 69.9 (1C, *C-3'*), 63.3 (1C, *C-3*), 60.8 (1C, *C-9*), 60.7 (1C, *C-10*), 60.3 (1C, *C-10'*), 60.2 (1C, *C-14'*), 60.0 (1C, *C-11*), 55.9 (1C, *C-9'*), 41.8 (1C, *C-12'*), 34.4 (1C, *C-4'*), 32.2 (1C, *C-4*), 21.5 (2C, *C-11'*, *C-12*), 14.3 (1C, *C-15'*); IR (KBr) ν = 2973, 2935 (ν_{as} Me, ν_{as} CH_2), 1738 (ν C=O), 1602, 1507, 1486, 1464, 1421, 1407 (ν Ar C=C, β_s CH_2, δ_{as} Me), 1384, 1360, 1315 (δ_s Me, δ CH, γ_s CH_2), 1267, 1254, 1222, 1174, 1159, 1122 (ν_{as} Ar-O-Me, ν Ar C-F, ν_{as} C-O-C=O), 1093, 1066, 1029 (ν_{as} C-O-C, ν_s Ar-O-Me, ν_s C-O-C=O), 871 (1,2,3,4,5-pentasubstituated γ_s Ar =CH) cm^{-1}; ESI-TOF-HRMS: *m/z* calculated for $C_{35}H_{41}FNaO_9$ $[M+Na]^+$ 647.2627, found 647.2628.

Ethyl 2'-{(a*S*,1*R*,3*R*,1'*S*,3'*R*)-1-(4-fluorophenyl)-6,7,7',8,8'-pentamethoxy-3,3'-dimethyl-[5,5'-*bis*-isochroman]-1'-yl}acetate [(a*S*,1*R*,3*R*,1'*S*,3'*R*)-23]

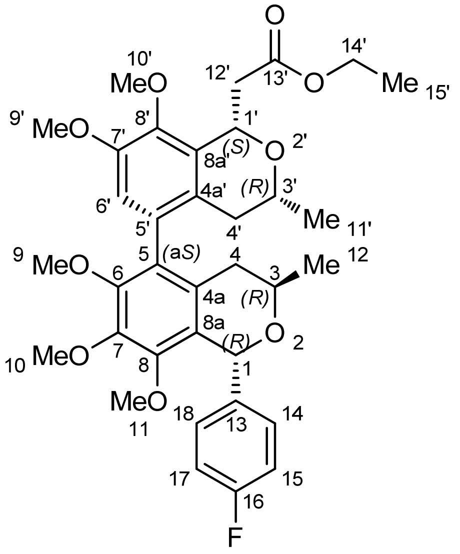

Starting from (a*S*,1*R*,3*R*,2'*R*)-**20** (100 mg, 0.19 mmol, 1.0 equiv.) using **method C**: anhydrous toluene (2 ml), ethyl-3,3-diethoxypropionate (90%, technical grade, 82 μl = 74 μl, 72 mg, 0.38 mmol, 2.0 equiv.), $BF_3 \cdot Et_2O$ (7 μl, 8 mg, 0.06 mmol, 0.3 equiv.), reaction time: 3 hours. (a*S*,1*R*,3*R*,1'*S*,3'*R*)-**23**: 98 mg (yield: 83%), white amorphous solid foam; $[\alpha]_D^{20}$ −70; $[\alpha]_{578}^{20}$ −74; $[\alpha]_{546}^{20}$ −84; $[\alpha]_{436}^{20}$ −131; $[\alpha]_{365}^{20}$ −230 (*c* = 0.50; MeCN); ECD (*c* = 1.87×10^{-4} M; MeCN) λ [nm], (Δε) = 287 (−0.14), 270 (0.55), 263 (0.89), 257sh (1.28), 241 (4.08), 232sh (3.89), 205 (−24.28). Chromatographic and spectral data except for the chiroptical ones were identical with those of (a*R*,1*S*,3*S*,1'*R*,3'*S*)-**23**.

Ethyl 2'-{(a*S*,1*R*,3*S*,1'*R*,3'*S*)-1-(4-fluorophenyl)-6,7,7',8,8'-pentamethoxy-3,3'-dimethyl-[5,5'-*bis*-isochroman]-1'-yl}acetate [(a*S*,1*R*,3*S*,1'*R*,3'*S*)-23]

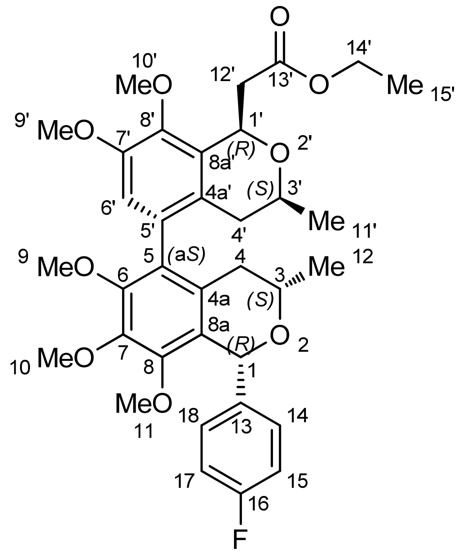

Starting from (a*S*,1*R*,3*S*,2'*S*)-**20** (140 mg, 0.27 mmol, 1.0 equiv.) using **method C**: anhydrous toluene (3 ml), ethyl-3,3-diethoxypropionate (90%, technical grade, 115 µl = 103 µl, 101 mg, 0.53 mmol, 2.0 equiv.), $BF_3 \cdot Et_2O$ (10 µl, 11 mg, 0.08 mmol, 0.3 equiv.), reaction time: 16 hours. (a*S*,1*R*,3*S*,1'*R*,3'*S*)-**23**: 150 mg (yield: 91%), white amorphous solid foam; $[\alpha]^{20}_{D}$ −36; $[\alpha]^{20}_{578}$ −38; $[\alpha]^{20}_{546}$ −46; $[\alpha]^{20}_{436}$ −111; $[\alpha]^{20}_{365}$ −253 (*c* = 0.48; MeCN); ECD (*c* = 1.41×10^{-4} M; MeCN) λ [nm], (Δε) = 289 (−2.02), 250sh (−7.82), 225 (−30.99), 201 (30.75). Flash chromatography (hexane/EtOAc 95:5 → 9:1 → 85:15 → 8:2); R_f = 0.55 (hexane/EtOAc 75:25); ^{1}H NMR (400 MHz, $CDCl_3$) *δ* = 7.38 – 7.32 (m, 2H, *H-14*, *H-18*), 7.07 – 7.00 (m, 2H, *H-15*, *H-17*), 6.69 (s, 1H, *H-6'*), 5.82 (s, 1H, *H-1*), 5.44 (dd, *J* = 8.4, 3.2 Hz, 1H, *H-1'*), 4.20 (qd, *J* = 7.1, 1.2 Hz, 2H, *H-14'*), 3.92 (s, 3H, *H-10'*), 3.88 (s, 3H, *H-9'*), 3.80 (s, 3H, *H-10*), 3.73 – 3.64 (m, 1H, *H-3*), 3.63 – 3.56 (m, 1H, *H-3'*), 3.55 (s, 3H, *H-9*), 3.29 (dd, *J* = 15.1, 3.3 Hz, 1H, *H-12'-a*), 3.15 (s, 3H, *H-11*), 2.64 (dd, *J* = 15.1, 8.3 Hz, 1H, *H-12'-b*), 2.49 – 2.38 (m, 2H, $H\text{-}4_{ax}$, $H\text{-}4'_{ax}$), 1.99 – 1.91 (m, 1H, $H\text{-}4_{eq}$), 1.95 – 1.89 (m, 1H, $H\text{-}4'_{eq}$), 1.28 (t, *J* = 7.1 Hz, 3H, *H-15'*), 1.23 (d, *J* = 6.1 Hz, 3H, *H-12*), 1.20 (d, *J* = 6.1 Hz, 3H, *H-11'*); ^{13}C NMR (100 MHz, $CDCl_3$) *δ* = 172.0 (1C, *C-13'*), 162.2 (d, $J_{C\text{-}F}$ = 245.3 Hz, 1C, *C-16*), 151.1 (1C, *C-6*), 150.1 (1C, *C-7'*), 149.7 (1C, *C-8*), 144.6 (1C, *C-8'*), 144.5 (1C, *C-7*), 140.1 (d, $J_{C\text{-}F}$ = 2.8 Hz, 1C, *C-13*), 130.6 (1C, *C-5'*), 130.3 (1C, *C-8a'*), 130.1 (d, $J_{C\text{-}F}$ = 8.0 Hz, 2C, *C-14*, *C-18*), 129.1 (1C, *C-4a*), 128.4 (1C, *C-5*), 127.6 (1C, *C-4a'*), 126.6 (1C, *C-8a*), 115.0 (d, $J_{C\text{-}F}$ = 21.4 Hz, 2C, *C-15*, *C-17*), 113.3 (1C, *C-6'*), 77.5 (1C, *C-1*), 71.6 (1C, *C-1'*), 70.5 (1C, *C-3*), 69.9 (1C, *C-3'*), 60.9 (1C, *C-9*), 60.6 (1C, *C-10*), 60.4 (1C, *C-10'*), 60.2 (1C, *C-14'*), 59.3 (1C, *C-11*), 56.0 (1C, *C-9'*), 42.0 (1C, *C-12'*), 35.6 (1C, *C-4*), 34.5 (1C, *C-4'*), 21.8 (1C, *C-12*), 21.6 (1C, *C-11'*), 14.3 (1C, *C-15'*); IR (KBr) ν = 2974, 2936 (ν_{as} Me, ν_{as} CH_2), 2847 (ν_s CH_2), 1737 (ν C=O), 1605, 1576, 1510, 1486, 1462, 1420 (ν Ar C=C, β_s CH_2, δ_{as} Me), 1383, 1359, 1315 (δ_s Me, δ CH, γ_s CH_2), 1279,

1256, 1224, 1154, 1117 (ν_{as} Ar-O-Me, ν Ar C-F, ν_{as} C-O-C=O), 1072, 1029 (ν_{as} C-O-C, ν_s Ar-O-Me, ν_s C-O-C=O) cm^{-1}; ESI-TOF-HRMS: *m/z* calculated for $C_{35}H_{41}FNaO_9$ $[M+Na]^+$ 647.2627, found 647.2619.

Ethyl 2'-{(a*R*,1*S*,3*R*,1'*S*,3'*R*)-1-(4-fluorophenyl)-6,7,7',8,8'-pentamethoxy-3,3'-dimethyl-[5,5'-*bis*-isochroman]-1'-yl}acetate [(a*R*,1*S*,3*R*,1'*S*,3'*R*)-23] and ethyl 2'-{(a*R*,1*R*,3*R*,1'*S*,3'*R*)-1-(4-fluorophenyl)-6,7,7',8,8'-pentamethoxy-3,3'-dimethyl-[5,5'-*bis*-isochroman]-1'-yl}acetate [(a*R*,1*R*,3*R*,1'*S*,3'*R*)-23]

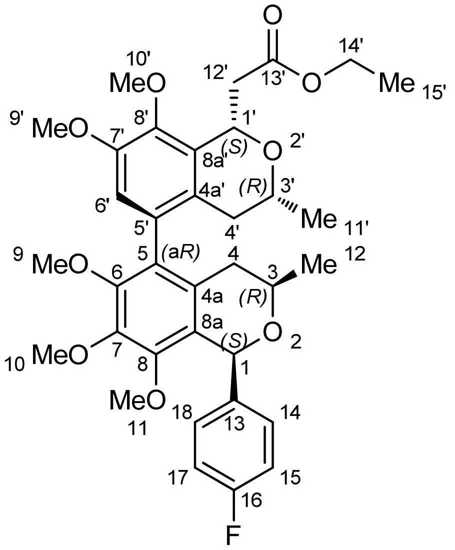

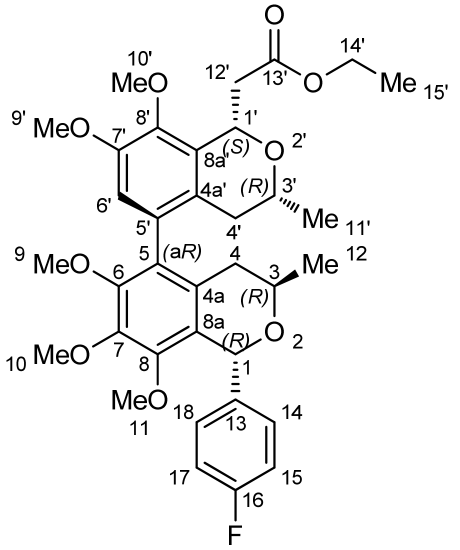

(a*S*,1*S*,3*S*,2'*S*)-**20** (100 mg, 0.19 mmol, 1.0 equiv.) was dissolved in anhydrous toluene (2 ml), and the solution was cooled to 0 °C. Then ethyl-3,3-diethoxypropionate (90%, technical grade, 82 µl = 74 µl, 72 mg, 0.38 mmol, 2.0 equiv.) and $BF_3 \cdot Et_2O$ (7 µl, 8 mg, 0.06 mmol, 0.3 equiv.) were added to the solution, and the reaction was allowed to warm up to room temperature. After two days, another portion of $BF_3 \cdot Et_2O$ (4 µl, 4 mg, 0.03 mmol, 0.15 equiv.) was added to the reaction mixture. The temperature was raised to 60 °C. After stirring 4.5 hours, the reaction was allowed to cool down to room temperature. The reaction was quenched with a saturated solution of $NaHCO_3$ and stirred for 10 minutes. EtOAc was added to the mixture and the layers were separated. The organic layer was extracted twice with a saturated solution of $NaHCO_3$ and once with brine. The organic phase was dried over anhydrous Na_2SO_4, filtered, and the solvent was evaporated in *vacuo*. The residue was purified by flash chromatography to yield the chiral non-racemic isochroman target derivative. (a*R*,1*S*,3*R*,1'*S*,3'*R*)-**23**: 34 mg (yield: 30%), white amorphous solid foam; $[\alpha]_D^{20}$ +27; $[\alpha]_{578}^{20}$ +28; $[\alpha]_{546}^{20}$ +35; $[\alpha]_{436}^{20}$ +52; $[\alpha]_{365}^{20}$ +205 (*c* = 0.39; MeCN); ECD (*c* = 1.81×10^{-4} M; MeCN) λ [nm], (Δε) = 289 (1.57), 250sh (6.47), 225 (25.77), 202 (−28.36).

Epimerization of C-1 caused by $BF_3{\cdot}Et_2O$ also resulted in (a*R*,1*R*,3*R*,1'*S*,3'*R*)-**23**: (38 mg, yield: 33%) as white amorphous solid foam. Chromatographic and spectral data except for the chiroptical ones were identical with those of the corresponding enantiomers (a*S*,1*R*,3*S*,1'*R*,3'*S*)-**23** and (a*S*,1*S*,3*S*,1'*R*,3'*S*)-**23**.

(*S*)-6,7,8-trimethoxy-3-methylisochroman [(*S*)-24]

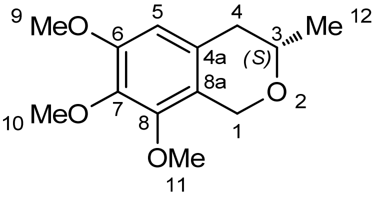

Starting from (*S*)-**16** (600 mg, 2.65 mmol, 1.0 equiv.) using **method A**: anhydrous Et_2O (20 ml), MOMCl (1.01 ml, 1.07 g, 13.26 mmol, 5.0 equiv.), annealed $ZnCl_2$ (109 mg, 0.80 mmol, 0.3 equiv.), reaction time: 2 hours. (*S*)-**24**: 575 mg (yield: 91%), colorless syrup; $[\alpha]_D^{20}$ +112 (*c* = 0.33; $CHCl_3$). Flash chromatography (hexane/EtOAc 95:5 → 93:7 → 9:1); R_f = 0.74 (hexane/EtOAc 6:4); ^{1}H NMR (400 MHz, $CDCl_3$) δ = 6.39 (s, 1H, *H-5*), 4.88 (d, J = 15.3 Hz, 1H, *H-1-a*), 4.62 (d, J = 15.3 Hz, 1H, *H-1-b*), 3.86, 3.83, 3.82 (3s, 3x 3H, *H-9*, *H-10*, *H-11*), 3.78 – 3.65 (m, 1H, *H-3*), 2.59 (d, J = 6.8 Hz, 2H, *H-4*), 1.33 (d, J = 6.2 Hz, 3H, *H-12*); ^{13}C NMR (100 MHz, $CDCl_3$) δ = 152.2, 149.2, 139.9, 129.1, 120.4 (5C, *C-4a*, *C-8a*, *C-6*, *C-7*, *C-8*), 107.2 (1C, *C-5*), 70.5 (1C, *C-3*), 64.4 (1C, *C-1*), 60.8, 60.4, 55.9 (3C, *C-10*, *C-11*, *C-12*), 35.5 (1C, *C-4*), 21.5 (1C, *C-9*); IR (KBr) ν = 2969, 2936 (ν_{as} Me, ν_{as} CH_2), 2837 (ν_s CH_2), 2017, 1725 (γ Ar =CH, γ Ar C=C overtone and combination bands), 1605, 1589, 1495, 1463 (ν Ar C=C, β_s CH_2, δ_{as} Me), 1383, 1365, 1336 (δ_s Me, δ CH), 1263, 1244, 1223 (ν_{as} Ar-O-Me), 1091 (ν_{as} C-O-C), 1050, 1031 (ν_s Ar-O-Me), 866 (1,2,3,4,5-pentasubstituated γ_s Ar =CH) cm^{-1}; ESI-TOF-HRMS: *m/z* calculated for $C_{13}H_{18}NaO_4$ $[M+Na]^+$ 261.1097, found 261.1096.

(a*R*,3*S*,3'*S*)-6,7,7',8,8'-pentamethoxy-3,3'-dimethyl-5,5'-*bis*-isochroman [(a*R*,3*S*,3'*S*)-28] and (a*S*,3*S*,3'*S*)-6,7,7',8,8'-pentamethoxy-3,3'-dimethyl-5,5'-*bis*-isochroman [(a*S*,3*S*,3'*S*)-28]

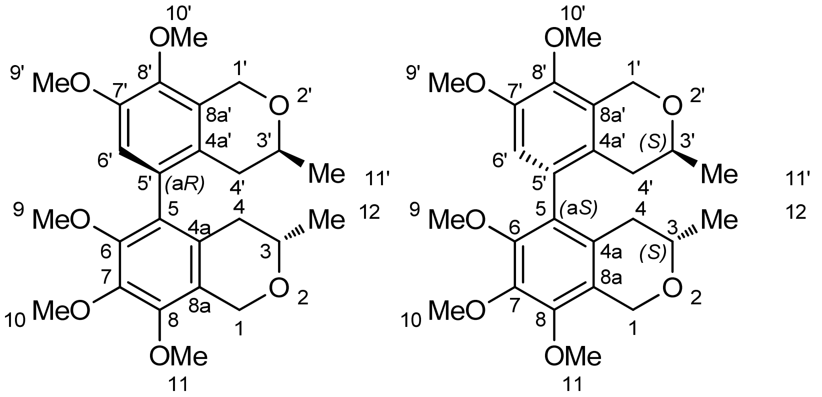

Starting from the mixture of (a*R*,3*S*,2'*S*)-**27** and (a*S*,3*S*,2'*S*)-**27** (136 mg, 0.32 mmol, 1.0 equiv.) using **method A**: anhydrous Et_2O (5 ml), MOMCl (185 µl, 196 mg, 2.44 mmol, 7.7 equiv.), annealed $ZnCl_2$ (13 mg, 0.10 mmol, 0.3 equiv.), reaction time: 4 hours. The mixture of (a*R*,3*S*,2'*S*)-**28** and (a*S*,3*S*,3'*S*)-**28**: 120 mg, (yield: 86%), yellow syrup. Flash chromatography (hexane/EtOAc 85:15 → 8:2 → 7:3); R_f = 0.24 (hexane/EtOAc 8:2); ESI-TOF-HRMS: *m/z* calculated for $C_{25}H_{32}NaO_7$ $[M+Na]^+$ 467.2040, found 467.2040. The atropdiastereomers were separated by preparative chiral HPLC using Chiralpak IC column with isocratic elution (hexane/*i*-PrOH 85:15).

(a*R*,3*S*,3'*S*)-**28**: t_R = 12.29 min $[\alpha]_D^{20}$ +57; $[\alpha]_{578}^{20}$ +60; $[\alpha]_{546}^{20}$ +68; $[\alpha]_{436}^{20}$ +123; $[\alpha]_{365}^{20}$ +197 (*c* = 0.45; MeCN); ECD (*c* = 2.66×10^{-4} M; MeCN) λ [nm], (Δε) = 289 (−0.07), 268 (0.09), 239 (−1.45), 228sh (−1.36), 204 (34.79); ^{1}H NMR (400 MHz, $CDCl_3$) δ = 6.56 (s, 1H, *H-6'*), 5.05 (d, *J* = 15.8 Hz, 1H, *H-1'$_{eq}$*), 4.98 (d, *J* = 15.6 Hz, 1H, *H-1$_{eq}$*), 4.77 (d, *J* = 15.8 Hz, 1H, *H-1'$_{ax}$*), 4.68 (d, *J* = 15.6 Hz, 1H, *H-1$_{ax}$*), 3.93 (s, 3H, *H-11*), 3.92 (s, 3H, *H-10*), 3.87 (s, 3H, *H-10'*), 3.82 (s, 3H, *H-9'*), 3.74 – 3.63 (m, 2H, *H-3*, *H-3'*), 3.62 (s, 3H, *H-9*), 2.27 – 2.01 (m, 3H, *H-4$_{eq}$*, *H-4'*), 2.06 (dd, *J* = 17.1, 10.4 Hz, 1H, *H-4$_{ax}$*) 1.25 (d, *J* = 6.2 Hz, 3H, *H-11'*), 1.22 (d, *J* = 6.3 Hz, 3H, *H-12*); ^{13}C NMR (100 MHz, $CDCl_3$) δ = 149.9 (1C, *C-7'*), 149.7 (1C, *C-6*), 148.7 (1C, *C-8*), 143.9 (1C, *C-7*), 143.7 (1C, *C-8'*), 131.4 (1C, *C-5'*), 129.4 (1C, *C-5*), 128.8 (1C, *C-8a'*), 128.5 (1C, *C-4a*), 125.7 (1C, *C-4a'*), 124.0 (1C, *C-8a*), 112.4 (1C, *C-6'*), 70.8 (2C, *C-3*, *C-3'*), 65.0 (1C, *C-1'*), 64.7 (1C, *C-1*), 61.1 (1C, *C-9*), 61.0 (1C, *C-10*), 60.6 (1C, *C-11*), 60.3 (1C, *C-10'*), 56.0 (1C, *C-9'*), 33.6 (1C, *C-4'*), 33.4 (1C, *C-4*), 21.7 (2C, *C-11'*, *C-12*); IR (KBr) ν = 3051 (ν Ar =CH), 2967, 2934 (ν_{as} Me, ν_{as} CH_2), 2893, 2839 (ν_s Me, ν_s CH_2, ν CH), 1958, 1733 (γ Ar =CH, γ Ar C=C overtone and combination bands), 1598, 1579, 1489, 1466, 1422, 1408 (ν Ar C=C, β_s CH_2, δ_{as} Me), 1384, 1362, 1314 (δ_s Me, δ CH, γ_s CH_2), 1280, 1258, 1233, 1215 (ν_{as} Ar-O-Me), 1131, 1114, 1083 (ν_{as} C-O-C, ν_s Ar-O-Me), 869 (1,2,3,4,5-pentasubstituated γ_s Ar =CH) cm^{-1}.

(a*S*,3*S*,3'*S*)-**28**: t_R = 14.33 min; $[\alpha]_D^{20}$ +69; $[\alpha]_{578}^{20}$ +72; $[\alpha]_{546}^{20}$ +82; $[\alpha]_{436}^{20}$ +135; $[\alpha]_{365}^{20}$ +196 (*c* =

0.44; MeCN); ECD ($c = 2.87\times10^{-4}$ M; MeCN) λ [nm], (Δε) = 287 (−0.81), 242sh (−4.11), 227 (−6.99), 201 (35.19); ^{1}H NMR (400 MHz, $CDCl_3$) δ = 6.57 (s, 1H, *H-6'*), 5.05 (d, J = 15.8 Hz, 1H, *H-1'*$_{eq}$), 4.97 (d, J = 15.5 Hz, 1H, *H-1*$_{eq}$), 4.76 (d, J = 15.8 Hz, 1H, *H-1'*$_{ax}$), 4.69 (d, J = 15.5 Hz, 1H, *H-1*$_{ax}$), 3.93 (s, 3H, *H-11*), 3.91 (s, 3H, *H-10*), 3.88 (s, 3H, *H-10'*), 3.81 (s, 3H, *H-9'*), 3.72 – 3.57 (m, 2H, *H-3'*, *H-3*), 3.58 (s, 3H, *H-9*), 2.37 (dd, J = 16.4, 10.7 Hz, 1H, *H-4'*$_{ax}$), 2.25 (dd, J = 16.8, 10.6 Hz, 1H, *H-4*$_{ax}$), 2.03 (dd, J = 16.5, 2.3 Hz, 1H, *H-4'*$_{eq}$), 1.94 (dd, J = 16.9, 2.0 Hz, 1H, *H-4*$_{eq}$), 1.25 (d, J = 5.9 Hz, 3H, *H-11'*), 1.24 (d, J = 5.9 Hz, 3H, *H-12*); ^{13}C NMR (100 MHz, $CDCl_3$) δ = 150.4 (1C, *C-6*), 149.9 (1C, *C-7'*), 148.7 (1C, *C-8*), 143.9 (1C, *C-7*), 143.7 (1C, *C-8'*), 131.2 (1C, *C-5'*), 129.1 (1C, *C-5*), 128.6 (1C, *C-8a'*), 127.7 (1C, *C-4a*), 125.5 (1C, *C-4a'*), 123.8 (1C, *C-8a*), 112.7 (1C, *C-6'*), 70.9 (1C, *C-3*), 70.8 (1C, *C-3'*), 64.9 (2C, *C-1'*, *C-1*), 61.1 (1C, *C-9*), 61.0 (1C, *C-10*), 60.6 (1C, *C-11*), 60.3 (1C, *C-10'*), 56.0 (1C, *C-9'*), 34.6 (1C, *C-4*), 33.6 (1C, *C-4'*), 21.8 (1C, *C-12*), 21.7 (1C, *C-11'*); IR (KBr) ν = 3051 (ν Ar =CH), 2967, 2934 (ν_{as} Me, ν_{as} CH_2), 2839 (ν_s CH_2), 1956, 1731 (γ Ar =CH, γ Ar C=C overtone and combination bands), 1598, 1579, 1489, 1465, 1421 (ν Ar C=C, β_s CH_2, δ_{as} Me), 1383, 1362, 1314 (δ_s Me, δ CH, γ_s CH_2), 1280, 1258, 1233 (ν_{as} Ar-O-Me), 1131, 1113, 1084, 1062, 1035 (ν_{as} C-O-C, ν_s Ar-O-Me), 865 (1,2,3,4,5-pentasubstituated γ_s Ar =CH) cm^{-1}.

2.2.6. General procedure for the Suzuki coupling reaction of chiral non-racemic 1-[(2-pinacolatoboryl)aryl]propan-2-yl acetate and 5-iodoisochroman derivatives

To the solution of the 5-iodoisochroman derivative (1.0 equiv.) in anhydrous DMF (2-22 ml), phosphine ligand (**method D**: SPhos, 0.2 equiv.; **method E**: (*S*)-BINAP, 0.1 equiv.; **method F**: Xantphos, 0.1 equiv.) and $Pd(OAc)_2$ (0.1 equiv.) were added under Ar or N_2 atmosphere, and the solution was stirred for 1 hour with inert gas bubbling at room temperature. To the solution of the 1-[(2-pinacolatoboryl)aryl]propan-2-yl acetate (1.2 equiv.) in anhydrous DMF (2-14 ml), CsF (4.0 equiv.) was added under Ar or N_2 atmosphere, and the solution was stirred for 0.5 hour with inert gas bubbling at room temperature. The first solution was merged with the second, and the mixture was stirred at 150 °C. When the 5-iodoisochroman derivative was consumed (1.5-3 hours) on the basis of TLC monitoring, the reaction mixture was poured on ice and diluted with Et_2O and the mixture was filtered on a short pad of Celite. The Celite was washed with Et_2O. The two layers were separated in a separatory funnel. The aqueous phase was extracted twice with Et_2O. The combined organic layers were washed twice with brine. The organic phase was dried over anhydrous Na_2SO_4, filtered, and the solvent was evaporated in *vacuo*. The residue was purified by flash chromatography to yield the 1-[2-(6,7,8-trimethoxy-3-methylisochroman-5-yl)-4,5-dimethoxyphenyl]propan-2-yl acetate target derivative.

(2*S*)-1-{2-[(a*S*,1*S*,3*S*)-1-(4-fluorophenyl)-6,7,8-trimethoxy-3-methylisochroman-5-yl]-4,5-dimethoxyphenyl}propan-2-yl acetate and (2*S*)-1-{2-[(a*R*,1*S*,3*S*)-1-(4-fluorophenyl)-6,7,8-trimethoxy-3-methylisochroman-5-yl]-4,5-dimethoxyphenyl}propan-2-yl acetate [the mixture of (a*R*,1*S*,3*S*,2'*S*)-19 and (a*S*,1*S*,3*S*,2'*S*)-19]

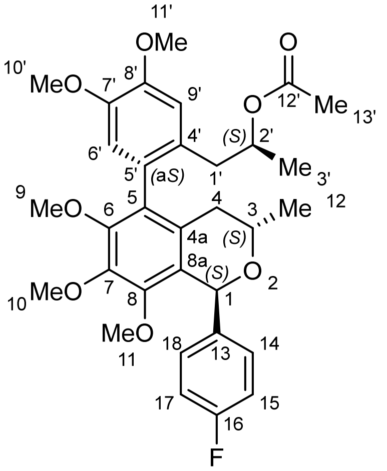

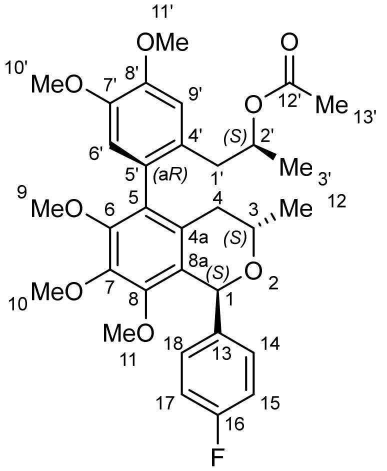

Starting from *trans*-(1*S*,3*S*)-**18** (255 mg, 0.56 mmol, 1.0 equiv.) using **method D**: anhydrous DMF (2.5 ml), SPhos (46 mg, 0.11 mmol, 0.2 equiv.), $Pd(OAc)_2$ (13 mg, 0.06 mmol, 0.1 equiv.), (*S*)-**8** (243 mg, 0.67 mmol, 1.2 equiv.), anhydrous DMF (2 ml), CsF (338 mg, 2.23 mmol, 4.0 equiv.), reaction time: 2 hours. The mixture of (a*R*,1*S*,3*S*,2'*S*)-**19** and (a*S*,1*S*,3*S*,2'*S*)-**19**: 209 mg (yield: 66%, *dr* 1.0:1.2) pale brown amorphous solid foam.

Starting from *trans*-(1*S*,3*S*)-**18** (152 mg, 0.33 mmol, 1.0 equiv.) with **method E**: anhydrous DMF (2 ml), (*S*)-BINAP (21 mg, 0.03 mmol, 0.1 equiv.), $Pd(OAc)_2$ (7 mg, 0.03 mmol, 0.1 equiv.), (*S*)-**8** (145 mg, 0.40 mmol, 1.2 equiv.), anhydrous DMF (2.5 ml), CsF (201 mg, 1.32 mmol, 4.0 equiv.), reaction time: 2 hours. The mixture of (a*R*,1*S*,3*S*,2'*S*)-**19** and (a*S*,1*S*,3*S*,2'*S*)-**19**: 102 mg (yield: 54%, *dr* 1.0:1.7) pale brown amorphous solid foam.

Starting from *trans*-(1*S*,3*S*)-**18** (815 mg, 1.78 mmol, 1.0 equiv.) with **method F**: anhydrous DMF (5 ml), Xantphos (103 mg, 0.18 mmol, 0.1 equiv.), $Pd(OAc)_2$ (40 mg, 0.18 mmol, 0.1 equiv.), (*S*)-**8** (778 mg, 2.14 mmol, 1.2 equiv.), anhydrous DMF (3 ml), CsF (1.08 g, 7.12 mmol, 4.0 equiv.), reaction time: 1.5 hours. The mixture of (a*R*,1*S*,3*S*,2'*S*)-**19** and (a*S*,1*S*,3*S*,2'*S*)-**19**: 797 mg (yield: 79%, *dr* 1.0:1.4) pale brown amorphous solid foam.

Flash chromatography (hexane/EtOAc 85:15 → 8:2 → 75:25 → 7:3); R_f = 0.27 (hexane/EtOAc 75:25); ^{1}H NMR (400 MHz, $CDCl_3$) δ = 7.30 – 7.14 [2m, 2 x 2H, (a*R*), (a*S*) *H-14*, *H-18*], 7.08 – 6.96 [2m, 2 x 2H, (a*R*), (a*S*) *H-15*, *H-17*], 6.91, 6.85 [2s, 2 x 1H, (a*R*), (a*S*) *H-9'*], 6.65, 6.62 [2s, 2 x 1H, (a*R*), (a*S*) *H-6'*], 6.04, 6.03 [2s, 2 x 1H, (a*R*), (a*S*) *H-1*], 5.21 – 5.08 [m, 1H, (a*R*) or (a*S*) *H-2'*], 4.89 – 4.76 [m, 1H, (a*R*) or (a*S*) *H-2'*], 3.94, 3.93, 3.89, 3.86, 3.71, 3.65, 3.57,

3.55 [10s, 10 x 3H, (a*R*), (a*S*) *H-11*, *H-10'*, *H-10*, *H-11'*, *H-9*], 3.69 – 3.62 [2m, 2 x 1H, (a*R*), (a*S*) *H-3*], 2.67, 2.55, 2.32, 2.24, 2.21, 2.05, 2.02 [dd, *J* = 14.1, 7.4 Hz, 1H, dd, *J* = 13.8, 8.0 Hz, 1H, dd, *J* = 13.8, 5.8 Hz, 1H, dd, *J* = 14.1, 6.4 Hz, 1H, dd, *J* = 17.8, 10.2 Hz, 1H, dd, *J* = 17.8, 2.5 Hz, 1H, dd, *J* = 17.9, 4.3 Hz, 1H, dd, *J* = 17.9, 9.8 Hz, 1H, (a*R*), (a*S*) *H-1'-a,b*, *H-4-a,b*], 1.99, 1.95 [2s, 2 x 3H, (a*R*), (a*S*) *H-13'*], 1.10 [d, *J* = 6.2 Hz, 3H, (a*R*) or (a*S*) *H-12*], 1.08 [d, *J* = 6.1 Hz, 3H, (a*R*) or (a*S*) *H-12*], 1.06 [d, *J* = 5.8 Hz, 2 x 3H, (a*R*), (a*S*) *H-3'*]; ^{13}C NMR (100 MHz, $CDCl_3$) δ = 170.5, 170.0 [2C, (a*R*), (a*S*) *C-12'*], 162.3 [d, $J_{C\text{-}F}$ = 245.8 Hz, 2C, (a*R*), (a*S*), *C-16*], 151.1, 150.8 [2C, (a*R*), (a*S*) *C-6*], 149.4 [2C, (a*R*), (a*S*) *C-8*], 148.2, 147.9 [2C, (a*R*), (a*S*) *C-7'*], 147.4, 147.2 [2C, (a*R*), (a*S*) *C-8'*], 144.2, 143.9 [2C, (a*R*), (a*S*) *C-7*], 138.3 [2C, (a*R*), (a*S*) *C-13*], 130.4, 130.3 [2d, $J_{C\text{-}F}$ = 7.8 Hz, 4C, (a*R*), (a*S*) *C-14*, *C-18*], 129.6, 129.3, 129.1, 128.6, 128.3, 128.2 [8C, (a*R*), (a*S*) *C-5'*, *C-4'*, *C-4a*, *C-5*], 124.5, 124.1 [2C, (a*R*), (a*S*) *C-8a*], 114.8 [d, *J* = 21.1 Hz, 4C, (a*R*), (a*S*) *C-15*, *C-17*], 113.5, 113.1, 112.8, 112.1 [4C, (a*R*), (a*S*) *C-6'*, *C-9'*], 73.5, 73.3, 71.4, 70.6 [4C, (a*R*), (a*S*) *C-2'*, *C-3*], 63.3, 63.1 [2C, (a*R*), (a*S*) *C-1*], 61.1, 61.0, 60.8, 60.7, 60.1 [6C, (a*R*), (a*S*) *C-9*, *C-10*, *C-11*], 56.1, 56.0, 55.9, 55.8 [4C, (a*R*), (a*S*) *C-11'*, *C-10'*], 40.1, 38.7 [2C, (a*R*), (a*S*) *C-1'*], 34.4, 33.7 [2C, (a*R*), (a*S*) *C-4*], 21.7, 21.6, 21.5, 21.3 [4C, (a*R*), (a*S*) *C-12*, *C-13'*], 20.1, 19.8 [2C, (a*R*), (a*S*) *C-3'*]; IR (KBr) ν = 2930 (ν_{as} CH_2), 2850 (ν_s CH_2), 1735 (ν C=O), 1604, 1508, 1463, 1422, 1409 (ν Ar C=C, β_s CH_2, δ_{as} Me), 1364, 1329 (δ CH, γ_s CH_2, δ_s Me), 1249, 1158, 1113 (ν_{as} C-O-C=O, ν_{as} Ar-O-Me, ν Ar C-F), 1069, 1025 (ν_{as} C-O-C, ν_s C-O-C=O, ν_s Ar-O-Me) cm^{-1}; ESI-TOF-HRMS: *m/z* calculated for $C_{32}H_{37}FNaO_8$ [M+Na]$^+$ 591.2365, found 591.2364.

(2*R*)-1-{2-[(a*R*,1*R*,3*R*)-1-(4-fluorophenyl)-6,7,8-trimethoxy-3-methylisochroman-5-yl]-4,5-dimethoxyphenyl}propan-2-yl acetate and (2*R*)-1-{2-[(a*S*,1*R*,3*R*)-1-(4-fluorophenyl)-6,7,8-trimethoxy-3-methylisochroman-5-yl]-4,5-dimethoxyphenyl}propan-2-yl acetate [the mixture of (a*R*,1*R*,3*R*,2'*R*)-19 and (a*S*,1*R*,3*R*,2'*R*)-19]

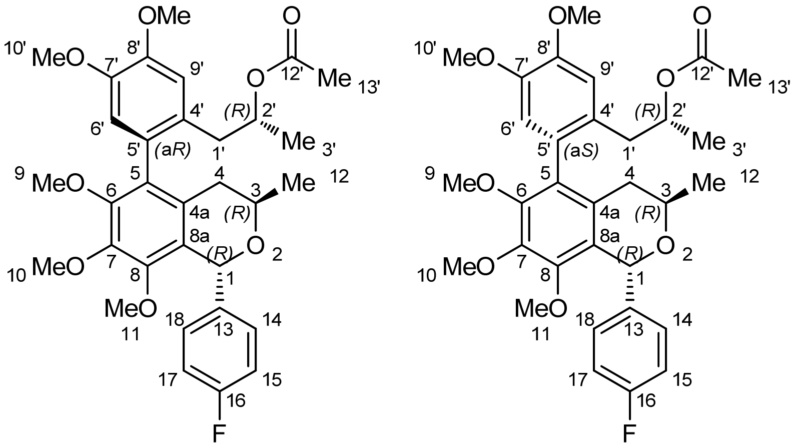

Starting from *trans*-(1*R*,3*R*)-**18** (1.00 g, 2.18 mmol, 1.0 equiv.) using **method F**: anhydrous DMF (6 ml), Xantphos (126 mg, 0.22 mmol, 0.1 equiv.), $Pd(OAc)_2$ (49 mg, 0.22 mmol, 0.1 equiv.), (*R*)-**8** (954 mg, 2.62 mmol, 1.2 equiv.), anhydrous DMF (4 ml), CsF (1.33 g, 8.73 mmol, 4.0 equiv.), reaction time: 3 hours. The mixture of (a*R*,1*R*,3*R*,2'*R*)-**19** and (a*S*,1*R*,3*R*,2'*R*)-**19**: 899 mg (yield: 73%, *dr* 1.0:1.4) pale brown amorphous solid foam. Chromatographic and spectral data except for the chiroptical ones were identical with those of the mixture of (a*R*,1*S*,3*S*,2'*S*)-**19** and (a*S*,1*S*,3*S*,2'*S*)-**19**.

(2*S*)-1-{2-[(a*S*,1*R*,3*S*)-1-(4-fluorophenyl)-6,7,8-trimethoxy-3-methylisochroman-5-yl]-4,5-dimethoxyphenyl}propan-2-yl acetate [(a*S*,1*R*,3*S*,2'*S*)-19] and (2*S*)-1-{2-[(a*R*,1*R*,3*S*)-1-(4-fluorophenyl)-6,7,8-trimethoxy-3-methylisochroman-5-yl]-4,5-dimethoxyphenyl}propan-2-yl acetate [(a*R*,1*R*,3*S*,2'*S*)-19]

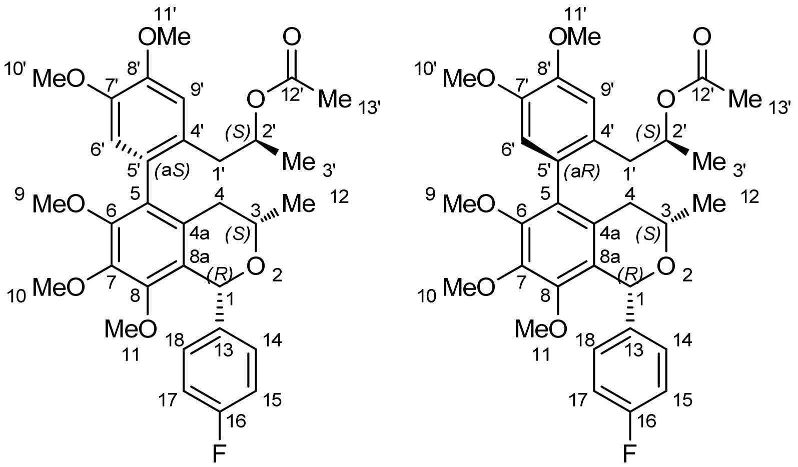

Starting from *cis*-(1*R*,3*S*)-**18** (500 mg, 1.09 mmol, 1.0 equiv.) using **method F**: anhydrous DMF (8 ml) Xantphos (63 mg, 0.11 mmol, 0.1 equiv.), $Pd(OAc)_2$ (25 mg, 0.11 mmol, 0.1 equiv.), (*S*)-**8** (477 mg, 1.31 mmol, 1.2 equiv.), anhydrous DMF (5 ml), CsF (663 mg, 4.36 mmol, 4.0 equiv.), reaction time: 1.5 hours. (a*S*,1*R*,3*S*,2'*S*)-**19**: 423 mg (yield: 68%) off-white amorphous solid foam; $[\alpha]_D^{20}$ −110 (*c* = 0.48; $CHCl_3$). (a*R*,1*R*,3*S*,2'*S*)-**19**: 69 mg (yield: 11%) yellow amorphous solid foam; $[\alpha]_D^{20}$ −45 (*c* = 0.14; $CHCl_3$). Flash chromatography (hexane/EtOAc 9:1 → 85:15 → 8:2 → 75:25 → 7:3 → 65:35); ESI-TOF-HRMS: *m/z* calculated for $C_{32}H_{37}FNaO_8$ $[M+Na]^+$ 591.2365, found 561.2360.

(a*S*,1*R*,3*S*,2'*S*)-**19**: R_f = 0.31 (hexane/EtOAc 8:2); ^1H NMR (400 MHz, $CDCl_3$) δ = 7.40 – 7.31 (m, 2H, *H-14*, *H-18*), 7.08 – 6.98 (m, 2H, *H-15*, *H-17*), 6.84 (s, 1H, *H-9'*), 6.67 (s, 1H, *H-6'*), 5.85 (s, 1H, *H-1*), 4.78 – 4.65 (m, 1H, *H-2'*), 3.94 (s, 3H, *H-11'*), 3.90 (s, 3H, *H-10'*), 3.79 (s, 3H, *H-10*), 3.76 – 3.66 (m, 1H, *H-3*), 3.55 (s, 3H, *H-9*), 3.14 (s, 3H, *H-11*), 2.67 – 2.53 (m, 2H, *H-1'*), 2.39 (ddd, *J* = 16.2, 10.7, 1.3 Hz, 1H, $H\text{-}4_{ax}$), 2.05 (d, *J* = 16.2 Hz, 1H, $H\text{-}4_{eq}$), 1.93 (s,

3H, *H-13'*), 1.21 (d, J = 6.2 Hz, 3H, *H-12*), 1.02 (d, J = 6.2 Hz, 3H, *H-3'*); ^{13}C NMR (100 MHz, $CDCl_3$) δ = 170.2 (1C, *C-12'*), 162.3 (d, $J_{C\text{-}F}$ = 245.1 Hz, 1C, *C-16*), 150.3 (1C, *C-6*), 149.8 (1C, *C-8*), 148.0 (1C, *C-8'*), 147.4 (1C, *C-7'*), 144.6 (1C, *C-7*), 140.3 (d, $J_{C\text{-}F}$ = 3.0 Hz, 1C, *C-13*), 130.3 (d, $J_{C\text{-}F}$ = 7.9 Hz, 2C, *C-14*, *C-18*), 130.2 (1C, *C-4a*), 129.2 (1C, *C-4'*), 128.8 (1C, *C-5*), 128.2 (1C, *C-5'*), 127.0 (1C, *C-8a*), 115.1 (d, $J_{C\text{-}F}$ = 21.4 Hz, 2C, *C-15*, *C-17*), 113.7 (1C, *C-6'*), 113.4 (1C, *C-9'*), 77.5 (1C, *C-1*), 70.8 (1C, *C-3*), 70.7 (1C, *C-2'*), 61.0 (1C, *C-9*), 60.7 (1C, *C-10*), 59.4 (1C, *C-11*), 56.2 (1C, *C-10'*), 56.0 (1C, *C-11'*), 39.9 (1C, *C-1'*), 35.8 (1C, *C-4*), 21.7 (1C, *C-12*), 21.4 (1C, *C-13'*), 20.3 (1C, *C-3'*); IR (KBr) ν = 2974, 2936 (ν_{as} Me, ν_{as} CH_2), 2845 (ν_s CH_2), 1735 (ν C=O), 1606, 1573, 1513, 1462, 1421, 1410 (ν Ar C=C, β_s CH_2, δ_{as} Me), 1371, 1351, 1328 (δ CH, γ_s CH_2, δ_s Me), 1251, 1225, 1171, 1154, 1119, 1102 (ν_{as} C-O-C=O, ν_{as} Ar-O-Me, ν Ar C-F), 1075, 1051, 1027, 1001 (ν_{as} C-O-C, ν_s C-O-C=O, ν_s Ar-O-Me), 861 (1,2,4,5-tetrasubstituated γ_s Ar =CH) cm^{-1}.

(a*R*,1*R*,3*S*,2'*S*)-**19**: R_f = 0.21 (hexane/EtOAc 8:2); ^{1}H NMR (400 MHz, $CDCl_3$) δ = 7.32 – 7.24 (m, 2H, *H-14*, *H-18*), 7.06 – 6.99 (m, 2H, *H-15*, *H-17*), 6.90, 6.62 (2s, 2 x 1H, *H-6'*, *H-9'*), 5.84 (s, 1H, *H-1*), 5.18 – 5.08 (m, 1H, *H-2'*), 3.94, 3.86, 3.81, 3.67, 3.20 (5s, 5 x 3H, *H-9*, *H-10*, *H-11*, *H-10'*, *H-11'*), 3.78 – 3.71 (m, 1H, *H-3*), 2.39 (dd, J = 14.2, 7.7, 1H, *H-4-a* or *H-1'-a*), 2.50 (dd, J = 14.2, 6.3 Hz, 1H, *H-4-b* or *H-1'-b*), 2.30 – 2.18 (m, 2H, *H-4* or *H-1'*), 2.00 (s, 3H, *H-13'*), 1.20, 1.14 (2d, J = 6.1 Hz, J = 6.2 Hz, 2 x 3H, *H-12*, *H-3'*); ^{13}C NMR (100 MHz, $CDCl_3$) δ = 170.5 (1C, *C-12'*), 162.3 (d, $J_{C\text{-}F}$ = 245.4 Hz, 1C, *C-16*), 150.9, 149.7, 148.2, 147.3, 144.6, 130.1, 129.1, 128.9, 128.3, 126.5 (10C, *C-4a*, *C-5*, *C-6*, *C-7*, *C-8*, *C-8a*, *C-4'*, *C-5'*, *C-7'*, *C-8'*), 140.3 (d, $J_{C\text{-}F}$ = 3.0 Hz, 1C, *C-13*), 130.0 (d, $J_{C\text{-}F}$ = 8.1 Hz, 2C, *C-14*, *C-18*), 115.4 (d, $J_{C\text{-}F}$ = 21.4 Hz, 2C, *C-15*, *C-17*), 113.3, 112.3 (2C, *C-6'*, *C-9'*), 77.3 (1C, *C-1*), 71.0, 70.4 (2C, *C-3*, *C-2'*), 61.0, 60.5, 59.4, 56.1, 55.9 (5C, *C-9*, *C-10*, *C-11*, *C-10'*, *C-11'*), 39.0, 34.8 (3C, *C-4*, *C-1'*), 21.7, 21.5, 20.1 (3C, *C-12*, *C-3'*, *C-13'*); IR (KBr) ν = 2973, 2936 (ν_{as} Me, ν_{as} CH_2), 2846 (ν_s CH_2), 1735 (ν C=O), 1604, 1575, 1512, 1463, 1421, 1408 (ν Ar C=C, β_s CH_2, δ_{as} Me), 1371, 1352, 1329 (δ CH, γ_s CH_2, δ_s Me), 1249, 1172, 1154, 1115 (ν_{as} C-O-C=O, ν_{as} Ar-O-Me, ν Ar C-F), 1074, 1050, 1027 (ν_{as} C-O-C, ν_s C-O-C=O, ν_s Ar-O-Me), 863 (1,2,4,5-tetrasubstituated γ_s Ar =CH) cm^{-1}.

(2*R*)-1-{2-[(a*R*,1*S*,3*R*)-1-(4-fluorophenyl)-6,7,8-trimethoxy-3-methylisochroman-5-yl]-4,5-dimethoxyphenyl}propan-2-yl acetate [(a*R*,1*S*,3*R*,2'*R*)-19]

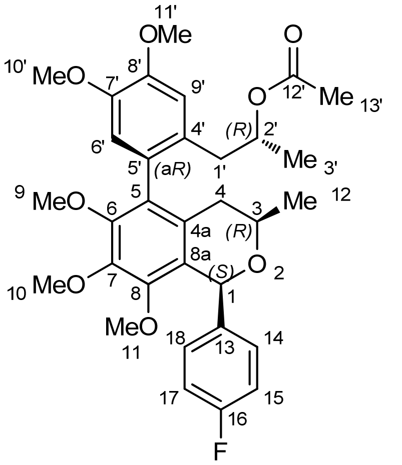

Starting from *cis*-(1*S*,3*R*)-**18** (1.40 g, 3.06 mmol, 1.0 equiv.) using **method F**: anhydrous DMF (22 ml) Xantphos (177 mg, 0.31 mmol, 0.1 equiv.), Pd(OAc)2 (69 mg, 0.31 mmol, 0.1 equiv.), (*R*)-**8** (1.34 g, 3.67 mmol, 1.2 equiv.), anhydrous DMF (14 ml), CsF (1.86 g, 12.22 mmol, 4.0 equiv.), reaction time: 3 hours. (a*R*,1*S*,3*R*,2'*R*)-**19**: 983 mg (yield: 57%) off-white amorphous solid foam; $[\alpha]_D^{20}$ +90 (*c* = 0.47; $CHCl_3$). Chromatographic and spectral data except for the chiroptical ones were identical with those of (a*S*,1*R*,3*S*,2'*S*)-**19**.

(2*S*)-1-{2-[(a*R*,3*S*)-6,7,8-trimethoxy-3-methylisochroman-5-yl]-4,5-dimethoxyphenyl}propan-2-yl acetate and (2*S*)-1-{2-[(a*S*,3*S*)-6,7,8-trimethoxy-3-methylisochroman-5-yl]-4,5-dimethoxyphenyl}propan-2-yl acetate [the mixture of (a*R*,3*S*,2'*S*)-26 and (a*S*,3*S*,2'*S*)-26]

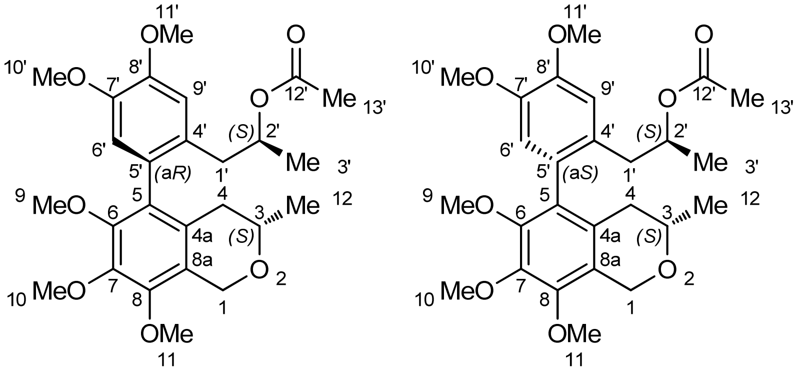

Starting from (*S*)-**25** (415 mg, 1.14 mmol, 1.0 equiv.) using **method D**: anhydrous DMF (4 ml), SPhos (94 mg, 0.23 mmol, 0.2 equiv.), Pd(OAc)2 (26 mg, 0.11 mmol, 0.1 equiv.), (*S*)-**8** (498 mg, 1.37 mmol, 1.2 equiv.), anhydrous DMF (4 ml), CsF (692 mg, 4.55 mmol, 4.0 equiv.), reaction time: 1.5 hours. The mixture of (a*R*,3*S*,2'*S*)-**26** and (a*S*,3*S*,2'*S*)-**26**: 359 mg (yield: 66%), yellow syrup. Flash chromatography (hexane/acetone 95: 5 → 9:1 → 85:15 → 8:2); R_f = 0.46 (hexane/acetone 7:3); ^{1}H NMR (400 MHz, $CDCl_3$) δ = 6.90, 6.84 [2s, 2 x 1H, (a*R*), (a*S*) *H-9'*], 6.60, 6.59 [2s, 2 x 1H, (a*R*), (a*S*) *H-6'*], 5.16 – 5.07 [m, 1H, (a*R*) or (a*S*) *H-2'*], 5.00, 4.96

[2d, *J* = 15.4 Hz, 2 x 1H, (a*R*), (a*S*) *H-1-a*], 4.86 – 4.75 [m, 1H, (a*R*) or (a*S*) *H-2'*], 4.76 – 4.65 [m, 2 x 1H, (a*R*), (a*S*) *H-1-b*], 3.94 [s, 3 x 3H, (a*R*), (a*S*) *H-11*, (a*R*) or (a*S*) *H-10'*], 3.93 [s, 3H, (a*R*) or (a*S*) *H-10'*], 3.91, 3.90 [2s, 2 x 3H, (a*R*), (a*S*) *H-10*], 3.85, 3.84 [2s, 2 x 3H, (a*R*), (a*S*) *H-11'*], 3.68 [s, 3H, (a*R*) or (a*S*) *H-9*], 3.67 – 3.59 [m, 3H, (a*R*), (a*S*) *H-3*], 3.56 [s, 3H, (a*R*), (a*S*) *H-9*], 2.67 [dd, *J* = 14.1, 7.4 Hz, 1H, (a*R*) or (a*S*) *H-1'-a*], 2.59 [qd, *J* = 14.1, 7.0 Hz, 2H, (a*R*) or (a*S*) *H-1'-a,b*], 2.33 [dd, *J* = 14.1, 6.4 Hz, 1H, (a*R*) or (a*S*) *H-1'-b*], 2.35 – 2.14 [m, 2 x 1H, (a*R*), (a*S*) *H-4-a*], 2.08 – 2.01 [m, 2 x 1H, (a*R*), (a*S*) *H-4-b*], 1.98, 1.94 [2s, 2 x 3H, (a*R*), (a*S*) *H-13'*], 1.22 [2d, *J* = 6.1 Hz, 2 x 3H, (a*R*), (a*S*) *H-12*], 1.08, 1.03 [2d, *J* = 6.2 Hz, 2 x 3H, (a*R*), (a*S*) *H-3'*]; ^{13}C NMR (100 MHz, $CDCl_3$) δ = 170.5, 170.2 [2C, (a*R*), (a*S*) *C-12'*], 150.3, 149.8 [2C, (a*R*), (a*S*) *C-6*], 148.6 [2C, (a*R*), (a*S*) *C-8*], 148.0, 147.8 [2C, (a*R*), (a*S*) *C-7'*], 147.2, 147.1 [2C, (a*R*), (a*S*) *C-8'*], 143.8 [2C, (a*R*), (a*S*) *C-7*], 129.7, 129.3 [2C, (a*R*), (a*S*) *C-5'*], 129.1, 128.8 [2C, (a*R*), (a*S*) *C-4'*], 128.5, 128.4 [2C, (a*R*), (a*S*) *C-4a*], 128.2, 128.1 [2C, (a*R*), (a*S*) *C-5*], 123.9, 123.5 [2C, (a*R*), (a*S*) *C-8a*], 113.2, 113.1 [2C, (a*R*), (a*S*) *C-6'*], 112.7, 111.9 [2C, (a*R*), (a*S*) *C-9'*], 71.3 [1C, (a*R*) or (a*S*) *C-2'*], 70.8 [1C, (a*R*) or (a*S*) *C-3*], 70.6 [2C, (a*R*) or (a*S*) *C-3*, (a*R*) or (a*S*) *C-2'*], 64.8, 64.6 [2C, (a*R*), (a*S*) *C-1*], 61.0 [2C, (a*R*), (a*S*) *C-9*], 60.9, 60.7, 60.5 [4C, (a*R*), (a*S*) *C-10*, *C-11*], 55.9 [2C, (a*R*), (a*S*) *C-11'*], 55.8 [2C, (a*R*), (a*S*) *C-10'*], 39.6, 38.6 [2C, (a*R*), (a*S*) *C-1'*], 34.7, 33.7 [2C, (a*R*), (a*S*) *C-4*], 21.6, 21.5 [2C, (a*R*), (a*S*) *C-12*], 21.4, 21.3 [2C, (a*R*), (a*S*) *C-13'*], 20.1, 19.7 [2C, (a*R*), (a*S*) *C-3'*]; IR (KBr) ν = 2970, 2935 (ν_{as} Me, ν_{as} CH_2), 2844 (ν_s CH_2), 1735 (ν C=O), 1606, 1578, 1516, 1465, 1423, 1410 (ν Ar C=C, β_s CH_2, δ_{as} Me), 1364, 1327 (δ CH, γ_s CH_2, δ_s Me), 1252, 1207, 1174, 1129, 1113 (ν_{as} C-O-C=O, ν_{as} Ar-O-Me), 1072, 1050, 1029 (ν_{as} C-O-C, ν_s C-O-C=O, ν_s Ar-O-Me), 864 (1,2,4,5-tetrasubstituated γ_s Ar =CH) cm^{-1}; ESI-TOF-HRMS: *m/z* calculated for $C_{26}H_{34}NaO_8$ $[M+Na]^+$ 497.2146, found 497.2145.

2.2.7. General procedure for deacetylation of chiral non-racemic 1-[2-(6,7,8-trimethoxy-3-methylisochroman-5-yl)-4,5-dimethoxyphenyl]propan-2-yl acetate derivatives

The corresponding 1-[2-(6,7,8-trimethoxy-3-methylisochroman-5-yl)-4,5-dimethoxyphenyl]-propan-2-yl acetate (1.0 equiv.) was dissolved in a mixture of MeOH:THF:H_2O. LiOH (2.0 equiv.) was added to the solution and it was stirred at room temperature. When the starting material was consumed (3-4 hours) on the basis of TLC monitoring, the solvent was evaporated in *vacuo*, and the residue was dissolved in CH_2Cl_2. The organic layer was extracted twice with a 10 % aqueous solution of $NaHSO_4$, twice with a saturated aqueous solution of $NaHCO_3$ and once with brine. The organic phase was dried over anhydrous Na_2SO_4, filtered, and the solvent was evaporated in *vacuo*. The residue was purified by flash chromatography to yield the 1-[2-

(6,7,8-trimethoxy-3-methylisochroman-5-yl)-4,5-dimethoxyphenyl]propan-2-ol target derivative.

(2*S*)-1-{2-[(a*S*,1*S*,3*S*)-1-(4-fluorophenyl)-6,7,8-trimethoxy-3-methylisochroman-5-yl]-4,5-dimethoxyphenyl}propan-2-ol [(a*S*,1*S*,3*S*,2'*S*)-20] and (2*S*)-1-{2-[(a*R*,1*S*,3*S*)-1-(4-fluorophenyl)-6,7,8-trimethoxy-3-methylisochroman-5-yl]-4,5-dimethoxyphenyl}propan-2-ol [(a*R*,1*S*,3*S*,2'*S*)-20]

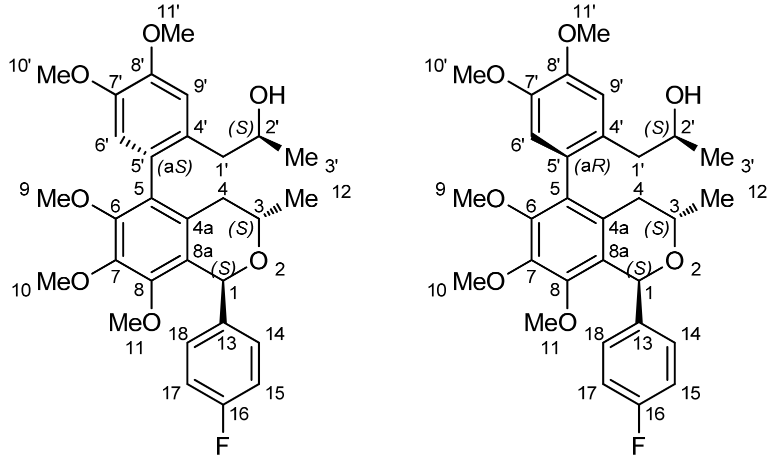

Starting from the mixture of (a*R*,1*S*,3*S*,2'*S*)-**19** and (a*S*,1*S*,3*S*,2'*S*)-**19** (893 mg, 1.57 mmol, 1.0 equiv.), a mixture of MeOH:THF:H_2O (5:2:3 ml), LiOH (75 mg, 3.14 mmol, 2.0 equiv.), reaction time: 4 hours. (a*S*,1*S*,3*S*,2'*S*)-**20**: 327 mg (yield 40%) off-white amorphous solid foam; $[\alpha]_D^{20}$ +36 (*c* = 0.49; $CHCl_3$). (a*R*,1*S*,3*S*,2'*S*)-**20**: 440 mg (yield: 53%) white amorphous solid foam; $[\alpha]_D^{20}$ +76 (*c* = 0.51; $CHCl_3$). Flash chromatography (CH_2Cl_2/EtOAc 10:0.1 → 10:0.3 → 10:1 → 9:1 → 7:3).

(a*S*,1*S*,3*S*,2'*S*)-**20**: R_f = 0.28 (hexane/EtOAc 6:4); ^{1}H NMR (400 MHz, $CDCl_3$) δ = 7.22 – 7.12 (m, 2H, *H-14*, *H-18*), 7.07 – 6.96 (m, 2H, *H-15*, *H-17*), 6.88 (s, 1H, *H-9'*), 6.63 (s, 1H, *H-6'*), 6.03 (s, 1H, *H-1*), 3.95 (s, 3H, *H-11'*), 3.93 – 3.89 (m, 1H, *H-2'*), 3.88 (s, 3H, *H-10*), 3.86 (s, 3H, *H-10'*), 3.68 (s, 3H, *H-9*), 3.63 (s, 3H, H-11), 3.62 – 3.54 (m, 1H, *H-3*), 2.63 (dd, *J* = 13.9, 3.2 Hz, 1H, *H-1'-a*), 2.29 (dd, *J* = 13.9, 9.4 Hz, 1H, *H-1'-b*), 2.24 (dd, *J* = 17.1, 11.0 Hz, 1H, $H\text{-}4_{ax}$), 2.02 (dd, *J* = 17.1, 3.5 Hz, 1H, $H\text{-}4_{eq}$), 1.16 (d, *J* = 6.1 Hz, 3H, *H-3'*), 1.06 (d, *J* = 6.1 Hz, 3H, *H-12*); ^{13}C NMR (100 MHz, $CDCl_3$) δ = 162.8 (d, $J_{C\text{-}F}$ = 246.1 Hz, 1C, *C-16*), 150.4 (1C, *C-6*), 149.4 (1C, *C-8*), 148.5 (1C, *C-8'*), 147.5 (1C, *C-7'*), 144.2 (1C, *C-7*), 138.1 (d, $J_{C\text{-}F}$ = 3.0 Hz, 1C, *C-13*), 130.2 (d, $J_{C\text{-}F}$ = 8.0 Hz, 2C, *C-14*, *C-18*), 129.7 (1C, *C-4'*), 129.6 (1C, *C-5*), 129.0 (1C, *C-4a*), 128.1 (1C, *C-5'*), 124.6 (1C, *C-8a*), 114.9 (d, $J_{C\text{-}F}$ = 21.3 Hz, 2C, *C-15*, *C-17*), 113.0 (1C, *C-6'*), 112.7 (1C, *C-9'*), 73.3 (1C, *C-1*), 68.7 (1C, *C-2'*), 63.2 (1C, *C-3*), 61.3 (1C, *C-9*), 61.0 (1C, *C-10*), 60.3 (1C, *C-11*), 56.1 (1C, *C-10'*), 55.9 (1C, *C-11'*), 43.0 (1C, *C-*

1'), 34.4 (1C, *C-4*), 23.8 (1C, *C-3'*), 21.7 (1C, *C-12*); IR (KBr) ν = 3461 (ν OH), 2967, 2935 (ν_{as} Me, ν_{as} CH_2), 2843 (ν_s CH_2), 1605, 1577, 1508, 1464, 1421, 1409 (ν Ar C=C, β_s CH_2, δ_{as} Me), 1363, 1330 (δ CH, γ_s CH_2), 1250, 1221, 1199, 1171, 1158, 1110 (ν_{as} Ar-O-Me, ν Ar C-F, ν C-OH), 1070, 1024 (ν_{as} C-O-C, ν_s Ar-O-Me) cm^{-1}; ESI-TOF-HRMS: *m/z* calculated for $C_{30}H_{35}FNaO_7$ $[M+Na]^+$ 549.2259, found 549.2255.

(a*R*,1*S*,3*S*,2'*S*)-**20**: R_f = 0.19 (hexane/EtOAc 6:4); ^{1}H NMR (400 MHz, $CDCl_3$) δ = 7.30 – 7.20 (m, 2H, *H-14*, *H-18*), 7.08 – 6.97 (m, 2H, *H-15*, *H-17*), 6.90 (s, 1H, *H-9'*), 6.64 (s, 1H, *H-6'*), 6.02 (s, 1H, *H-1*), 3.94 (s, 3H, *H-11'*), 3.90 – 3.80 (m, 1H, *H-2'*), 3.89 (s, 3H, *H-10'*), 3.84 (s, 3H, *H-10*), 3.73 (s, 3H, *H-9*), 3.73 – 3.60 (m, 1H, *H-3*), 3.53 (s, 3H, *H-11*), 2.43 (dd, *J* = 13.7, 7.7 Hz, 1H, *H-1'-a*), 2.41 (dd, *J* = 13.7, 5.1 Hz, 1H, *H-1'-b*), 2.23 (dd, *J* = 17.0, 3.5 Hz, 1H, $H\text{-}4_{eq}$), 2.03 (dd, *J* = 17.0, 10.7 Hz, 1H, $H\text{-}4_{ax}$), 1.94 (bs, 1H, O*H*), 1.10 (d, *J* = 6.1 Hz, 3H, *H-3'*), 1.06 (d, *J* = 6.1 Hz, 3H, *H-12*); ^{13}C NMR (100 MHz, $CDCl_3$) δ = 162.3 (d, $J_{C\text{-}F}$ = 245.9 Hz, 1C, *C-16*), 151.1 (1C, *C-6*), 149.4 (1C, *C-8*), 148.1 (1C, *C-8'*), 147.4 (1C, *C-7'*), 143.8 (1C, *C-7*), 138.3 (d, $J_{C\text{-}F}$ = 2.9 Hz, 1C, *C-13*), 130.4 (d, $J_{C\text{-}F}$ = 8.0 Hz, 2C, *C-14*, *C-18*), 129.9 (1C, *C-4'*), 129.8 (1C, *C-5*), 128.9 (1C, *C-4a*), 128.3 (1C, *C-5'*), 124.4 (1C, *C-8a*), 114.8 (d, $J_{C\text{-}F}$ = 21.2 Hz, 2C, *C-15*, *C-17*), 113.8 (1C, *C-9'*), 113.2 (1C, *C-6'*), 73.5 (1C, *C-1*), 67.5 (1C, *C-2'*), 63.3 (1C, *C-3*), 61.2 (1C, *C-9*), 60.6 (1C, *C-10*), 60.1 (1C, *C-11*), 56.2 (1C, *C-10'*), 55.9 (1C, *C-11'*), 43.2 (1C, *C-1'*), 33.7 (1C, *C-4*), 23.1 (1C, *C-3'*), 21.6 (1C, *C-12*); IR (KBr) ν = 3430 (ν OH), 2966, 2934 (ν_{as} Me, ν_{as} CH_2), 2843 (ν_s CH_2), 1604, 1578, 1508, 1464, 1421, 1409 (ν Ar C=C, β_s CH_2, δ_{as} Me), 1360, 1330 (δ CH, γ_s CH_2), 1251, 1220, 1158, 1112 (ν_{as} Ar-O-Me, ν Ar C-F, ν C-OH), 1069, 1026 (ν_{as} C-O-C, ν_s Ar-O-Me) cm^{-1}; ESI-TOF-HRMS: *m/z* calculated for $C_{30}H_{36}FO_7$ $[M+H]^+$ 527.2440, found 527.2431.

(2*R*)-1-{2-[(a*R*,1*R*,3*R*)-1-(4-fluorophenyl)-6,7,8-trimethoxy-3-methylisochroman-5-yl]-4,5-dimethoxyphenyl}propan-2-ol [(a*R*,1*R*,3*R*,2'*R*)-20] and (2*R*)-1-{2-[(a*S*,1*R*,3*R*)-1-(4-fluorophenyl)-6,7,8-trimethoxy-3-methylisochroman-5-yl]-4,5-dimethoxyphenyl}propan-2-ol [(a*S*,1*R*,3*R*,2'*R*)-20]

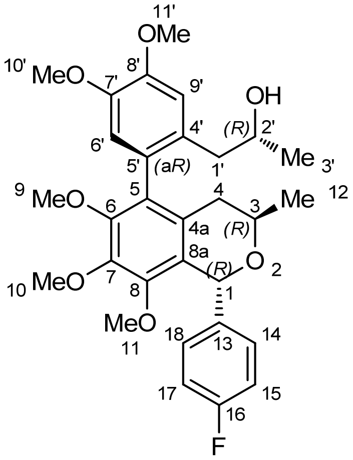 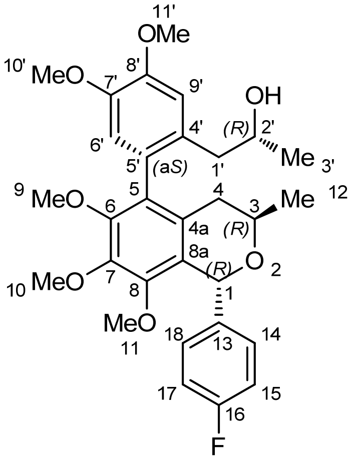

Starting from the mixture of (a*R*,1*R*,3*R*,2'*R*)-**19** and (a*S*,1*R*,3*R*,2'*R*)-**19** (899 mg, 1.58 mmol, 1.0 equiv.), a mixture of MeOH:THF:H_2O (5:2:3 ml), LiOH (76 mg, 3.16 mmol, 2.0 equiv.), reaction time: 4 hours. (a*R*,1*R*,3*R*,2'*R*)-**20**: 353 mg (yield 42%), off-white amorphous solid foam; $[\alpha]_D^{20}$ −48 (c = 0.42; $CHCl_3$). (a*S*,1*R*,3*R*,2'*R*)-**20**: 447 mg (yield: 54%), white amorphous solid foam; $[\alpha]_D^{20}$ −80 (c = 0.52; $CHCl_3$). Chromatographic and spectral data except for the chiroptical ones were identical with those of the mixture of (a*S*,1*S*,3*S*,2'*S*)-**20** and (a*R*,1*S*,3*S*,2'*S*)-**20**.

(2*S*)-1-{2-[(a*S*,1*R*,3*S*)-1-(4-fluorophenyl)-6,7,8-trimethoxy-3-methylisochroman-5-yl]-4,5-dimethoxyphenyl}propan-2-ol [(a*S*,1*R*,3*S*,2'*S*)-20]

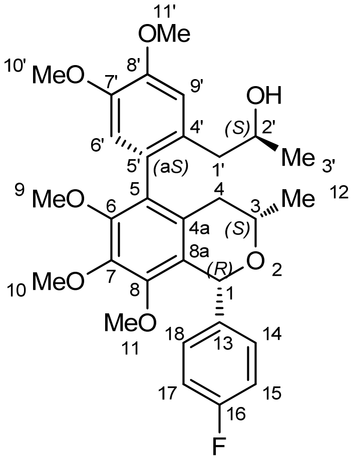

Starting from (a*S*,1*R*,3*S*,2'*S*)-**19** (403 mg, 0.71 mmol, 1.0 equiv.), a mixture of MeOH:THF:H_2O (5:2:3 ml), LiOH (34 mg, 1.42 mmol, 2.0 equiv.), reaction time: 4 hours. (a*S*,1*R*,3*S*,2'*S*)-**20**: 359 mg (yield: 96%), white amorphous solid foam; $[\alpha]_D^{20}$ −92 (c = 0.48; $CHCl_3$). Flash chromatography (hexane/EtOAc 65:35 → 6:4 → 55:45); R_f = 0.21 (hexane/EtOAc 6:4); ^{1}H NMR (400 MHz, $CDCl_3$) δ = 7.40 – 7.30 (m, 2H, *H-14*, *H-18*), 7.09 – 6.98 (m, 2H, *H-15*, *H-17*), 6.88 (s, 1H, *H-9'*), 6.65 (s, 1H, *H-6'*), 5.83 (s, 1H, *H-1*), 4.00 – 3.89

(m), 1H, *H-2'*), 3.95 (s, 3H, *H-11'*), 3.90 (s, 3H, *H-10'*), 3.78 (s, 3H, *H-10*), 3.75 – 3.67 (m, 1H, *H-3*), 3.63 (s, 3H, *H-9*), 3.11 (s, 3H, *H-11*), 2.78 (bs, 1H, O*H*), 2.58 (dd, *J* = 14.1, 3.1 Hz, 1H, *H-1'-a*), 2.36 (ddd, *J* = 16.4, 10.9, 1.4 Hz, 1H, $H\text{-}4_{ax}$), 2.18 (dd, *J* = 14.1, 9.7 Hz, 1H, *H-1'-b*), 2.03 (d, *J* = 16.4 Hz, 1H, $H\text{-}4_{eq}$), 1.21 (d, *J* = 6.1 Hz, 3H, *H-12*), 1.13 (d, *J* = 6.1 Hz, 3H, *H-3'*); ^{13}C NMR (100 MHz, $CDCl_3$) δ = 162.3 (d, $J_{C\text{-}F}$ = 245.2 Hz, 1C, *C-16*), 149.9, 149.8 (2C, *C-6*, *C-8*), 148.6 (1C, *C-8'*), 147.5 (1C, *C-7'*), 144.6 (1C, *C-7*), 140.1 (d, $J_{C\text{-}F}$ = 2.9 Hz, 1C, *C-13*), 130.2 (d, $J_{C\text{-}F}$ = 8.0 Hz, 2C, *C-14*, *C-18*), 130.1, 130.0, 129.1, 128.1, 127.2 (5C, *C-4'*, *C-5'*, *C-4a*, *C-5*, *C-8a*), 115.0 (d, $J_{C\text{-}F}$ = 21.4 Hz, 2C, *C-15*, *C-17*), 113.2, 112.3 (2C, *C-6'*, *C-9'*), 77.5 (1C, *C-1*), 70.5, 68.6 (2C, *C-2'*, *C-3*), 61.2 (1C, *C-9*), 60.8 (1C, *C-10*), 59.4 (1C, *C-11*), 56.2 (1C, *C-10'*), 55.9 (1C, *C-11'*), 42.7 (1C, *C-1'*), 35.4 (1C, *C-4*), 24.0, 21.7 (2C, *C-12*, *C-3'*); IR (KBr) ν = 3493 (ν OH), 2968, 2936 (ν_{as} Me, ν_{as} CH_2), 2845 (ν_s CH_2), 1606, 1574, 1512, 1462, 1421, 1410 (ν Ar C=C, β_s CH_2, δ_{as} Me), 1352, 1329 (δ CH, γ_s CH_2), 1251, 1222, 1171, 1155, 1118 (ν_{as} Ar-O-Me, ν Ar C-F, ν C-OH), 1075, 1049, 1026 (ν_{as} C-O-C, ν_s Ar-O-Me), 854 (1,2,4,5-tetrasubstituated γ_s Ar =CH) cm^{-1}; ESI-TOF-HRMS: *m/z* calculated for $C_{30}H_{35}FNaO_7$ $[M+Na]^+$ 549.2259, found 549.2259.

(2*R*)-1-{2-[(a*R*,1*S*,3*R*)-1-(4-fluorophenyl)-6,7,8-trimethoxy-3-methylisochroman-5-yl]-4,5-dimethoxyphenyl}propan-2-ol [(a*R*,1*S*,3*R*,2'*R*)-20]

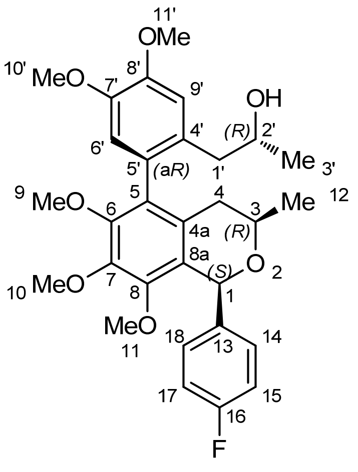

Starting from (a*R*,1*S*,3*R*,2'*R*)-**19** (983 mg, 1.73 mmol, 1.0 equiv.), a mixture of MeOH:THF:H_2O (12:5:7 ml), LiOH (83 mg, 3.46 mmol, 2.0 equiv.), reaction time: 4 hours. (a*R*,1*S*,3*R*,2'*R*)-**20**: 878 mg (yield: 96%) white amorphous solid foam; $[\alpha]_D^{20}$ +71 (*c* = 0.31; $CHCl_3$). Chromatographic and spectral data except for the chiroptical ones were identical with those of (a*S*,1*R*,3*S*,2'*S*)-**20**.

(2*S*)-1-{2-[(a*R*,1*R*,3*S*)-1-(4-fluorophenyl)-6,7,8-trimethoxy-3-methylisochroman-5-yl]-4,5-dimethoxyphenyl}propan-2-ol [(a*R*,1*R*,3*S*,2'*S*)-20]

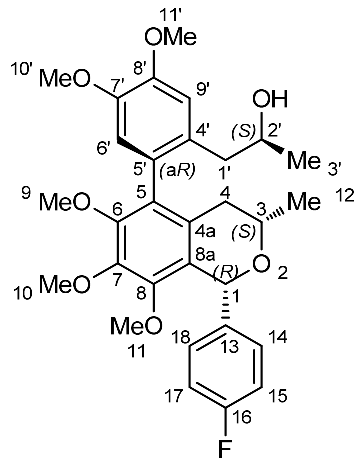

Starting from (a*R*,1*R*,3*S*,2'*S*)-**19** (55 mg, 0.10 mmol, 1.0 equiv.), a mixture of MeOH:THF:H_2O (1:0.8:0.5 ml), LiOH (5 mg, 0.19 mmol, 2.0 equiv.), reaction time: 4 hours. (a*R*,1*R*,3*S*,2'*S*)-**20**: 48 mg (yield: 94%), off-white amorphous solid foam; $[\alpha]_D^{20}$ −36 (*c* = 0.10; $CHCl_3$). Purification was not required for the crude product. R_f = 0.29 (hexane/EtOAc 1:1); ^{1}H NMR (400 MHz, $CDCl_3$) δ = 7.28 – 7.21 (m, 2H, *H-14*, *H-18*), 7.07 – 6.96 (m, 2H, *H-15*, *H-17*), 6.89, 6.65 (2s, 2 x H, *H-6'*, *H-9'*), 5.83 (s, 1H, *H-1*), 3.95, 3.87, 3.80, 3.71, 3.21 (5s, 5 x 3H, *H-9*, *H-10*, *H-11*, *H-10'*, *H-11'*), 3.86 – 3.81 (m, 1H, *H-2'*), 3.78 – 3.72 (m, 1H, *H-3*), 2.56 (dd, *J* = 13.7, 4.6 Hz, 1H, *H-4-a* or *H-1'-a*), 2.49 (dd, *J* = 13.7, 8.3 Hz, 1H, *H-4-b* or *H-1'-b*), 2.32 – 2.19 (m, 2H, *H-4* or *H-1'*), 1.78 (bs, 1H, O*H*), 1.20, 1.13 (2d, *J* = 6.1 Hz, *J* = 6.2 Hz, 2 x 3H, *H-12*, *H-3'*); ^{13}C NMR (100 MHz, $CDCl_3$) δ = 162.3 (d, $J_{C\text{-}F}$ = 245.4 Hz, 1C, *C-16*), 150.9, 149.7, 148.2, 147.4, 144.5, 129.8, 129.1, 128.4, 126.7 (10C, *C-4a*, *C-5*, *C-6*, *C-7*, *C-8*, *C-8a*, *C-4'*, *C-5'*, *C-7'*, *C-8'*) , 140.2 (d, $J_{C\text{-}F}$ = 3.0 Hz, 1C, *C-13*), 129.9 (d, $J_{C\text{-}F}$ = 8.4 Hz, 2C, *C-14*, *C-18*), 115.2 (d, $J_{C\text{-}F}$ = 21.4 Hz, 2C, *C-15*, *C-17*), 113.6, 113.5 (2C, *C-6'*, *C-9'*), 77.2 (1C, *C-1*), 70.4, 67.7 (2C, *C-3*, *C-2'*), 61.1, 60.5, 59.4, 56.2, 56.0 (5C, *C-9*, *C-10*, *C-11*, *C-10'*, *C-11'*), 43.3 (1C, *C-1'*), 34.8 (1C, *C-4*), 23.2, 21.7 (2C, *C-12*, *C-3'*); IR (KBr) ν = 3443 (ν OH), 2967, 2935 (ν_{as} Me, ν_{as} CH_2), 2847 (ν_s CH_2), 1606, 1577, 1512, 1463, 1422, 1408 (ν Ar C=C, β_s CH_2, δ_{as} Me), 1352, 1330 (δ CH, γ_s CH_2), 1251, 1221, 1171, 1154, 1114 (ν_{as} Ar-O-Me, ν Ar C-F, ν C-OH), 1074, 1049, 1027 (ν_{as} C-O-C, ν_s Ar-O-Me), 867 (1,2,4,5-tetrasubstituated γ_s Ar =CH) cm^{-1}; ESI-TOF-HRMS: *m/z* calculated for $C_{30}H_{35}FNaO_7$ $[M+Na]^+$ 549.2259, found 549.2254.

(2*S*)-1-{2-[(a*R*,3*S*)-6,7,8-trimethoxy-3-methylisochroman-5-yl]-4,5-dimethoxy-phenyl}propan-2-ol and (2*S*)-1-{2-[(a*S*,3*S*)-6,7,8-trimethoxy-3-methylisochroman-5-yl]-4,5-dimethoxyphenyl}propan-2-ol [the mixture of (a*R*,3*S*,2'*S*)-27 and (a*S*,3*S*,2'*S*)-27]

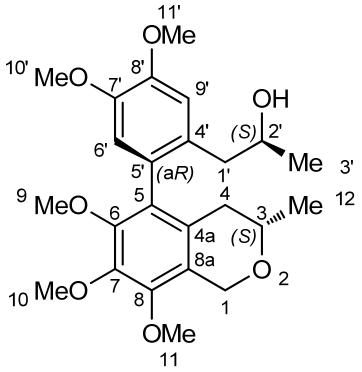

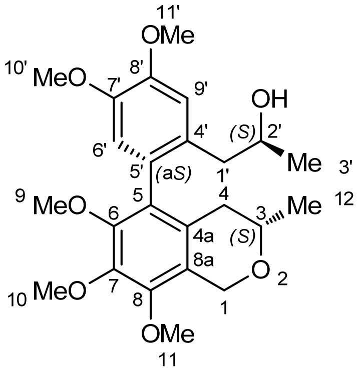

Starting from the mixture of (a*R*,3*S*,2'*S*)-**26** and (a*S*,3*S*,2'*S*)-**26** (338 mg, 0.71 mmol, 1.0 equiv.), MeOH:THF:H_2O (7:2:2 ml), LiOH (34 mg, 1.43 mmol, 2.0 equiv.), reaction time: 3 hours. The mixture of (a*R*,3*S*,2'*S*)-**27** and (a*S*,3*S*,2'*S*)-**27**: 281 mg (yield: 91%), light yellow goo. Purification was not required for the crude product. R_f = 0.18 (hexane/EtOAc 6:4); ^{1}H NMR (400 MHz, $CDCl_3$) δ = 6.91, 6.88 [2s, 2 x 1H, (a*R*), (a*S*) *H-9'*], 6.61, 6.59 [2s, 2 x 1H, (a*R*), (a*S*) *H-6'*], 4.98 [d, *J* = 14.8 Hz, 1H, (a*R*) or (a*S*) *H-1-a*], 4.94 [d, *J* = 14.6 Hz, 1H, (a*R*) or (a*S*) *H-1-a*], 4.70 [d, *J* = 14.7 Hz, 1H, (a*R*) or (a*S*) *H-1-b*], 4.66 [d, *J* = 14.7 Hz, 1H, (a*R*) or (a*S*) *H-1-b*], 3.94, 3.89, 3.88, 3.85, 3.84, 3.69, 3.63 [9s, 10 x 3H, (a*R*), (a*S*) *H-10'*, *H-11'*, *H-9*, *H-10*, *H-11*], 3.87 – 3.78 [m, 2 x 1H, (a*R*), (a*S*) *H-2'* or *H-3*], 3.68 – 3.56 [m, 2 x 1H, (a*R*), (a*S*) *H-2'* or *H-3*], 2.91 [s, 2H, (a*R*), (a*S*) O*H*], 2.56, 2.44, 2.38, 2.32 – 2.16, 2.10 – 1.96 [dd, *J* = 14.0, 3.4 Hz, 1H, dd, *J* = 13.7, 8.0 Hz, dd, *J* = 13.7, 4.9 Hz, 2m, 3H, (a*R*), (a*S*) *H-1'-a,b*, *H-1-a,b*, *H-4-a,b*], 1.21 [2d, *J* = 5.9 Hz, *J* = 5.8 Hz, 2 x 3H, (a*R*), (a*S*) *H-12*], 1.12, 1.07 [2d, *J* = 6.0 Hz, *J* = 6.1 Hz, 2 x 3H, (a*R*), (a*S*) *H-3'*]; ^{13}C NMR (100 MHz, $CDCl_3$) δ = 150.1, 149.2 [2C, (a*R*), (a*S*) *C-6*], 148.4, 148.2, 147.7, 147.1, 147.0 [6C, (a*R*), (a*S*) *C-7'*, *C-8'*, *C-8*], 143.6, 143.5 [2C, (a*R*), (a*S*) *C-7*], 129.8, 129.7, 129.6, 128.1, 128.0, 127.9, 127.8 [8C, (a*R*), (a*S*) *C-4'*, *C-5'*, *C-4a*, *C-5*], 123.9, 123.5 [2C, (a*R*), (a*S*) *C-8a*], 113.4, 112.8, 112.7, 112.1 [4C, (a*R*), (a*S*) *C-6'*, *C-9'*], 70.5, 70.4, 68.3, 67.3 [4C, (a*R*), (a*S*) *C-3*, *C-2'*], 64.5, 64.3 [2C, (a*R*), (a*S*) *C-1*], 60.9, 60.9, 60.8, 60.5, 60.3, 55.8, 55.6 [10C, (a*R*), (a*S*) *C-9*, *C-10*, *C-11*, *C-10'*, *C-11'*], 42.7, 42.4 [2C, (a*R*), (a*S*) *C-1'*], 34.2, 33.4 [2C, (a*R*), (a*S*) *C-4*], 23.6, 22.9 [2C, (a*R*), (a*S*) *C-12*], 21.4, 21.3 [2C, (a*R*), (a*S*) *C-3'*]; IR (KBr) ν = 3499 (ν OH), 2935 (ν_{as} CH_2), 2844 (ν_s CH_2), 2049, 1958, 1715 (γ Ar =CH, γ Ar C=C overtone and combination bands), 1606, 1579, 1515, 1465, 1409 (ν Ar C=C, β_s CH_2, δ_{as} Me), 1391, 1363, 1328 (δ_s Me, δ CH, γ_s CH_2), 1253, 1219, 1174, 1111 (ν_{as} Ar-O-Me, ν C-OH), 1073, 1029 (ν_{as} C-O-C, ν_s Ar-O-Me), 866 (1,2,4,5-tetrasubstituated γ_s Ar =CH) cm^{-1}; ESI-TOF-HRMS: *m/z* calculated for $C_{24}H_{32}NaO_7$ $[M+Na]^+$ 455.2040, found 455.2040.

3. Spectra of the compounds

3.1. NMR spectra

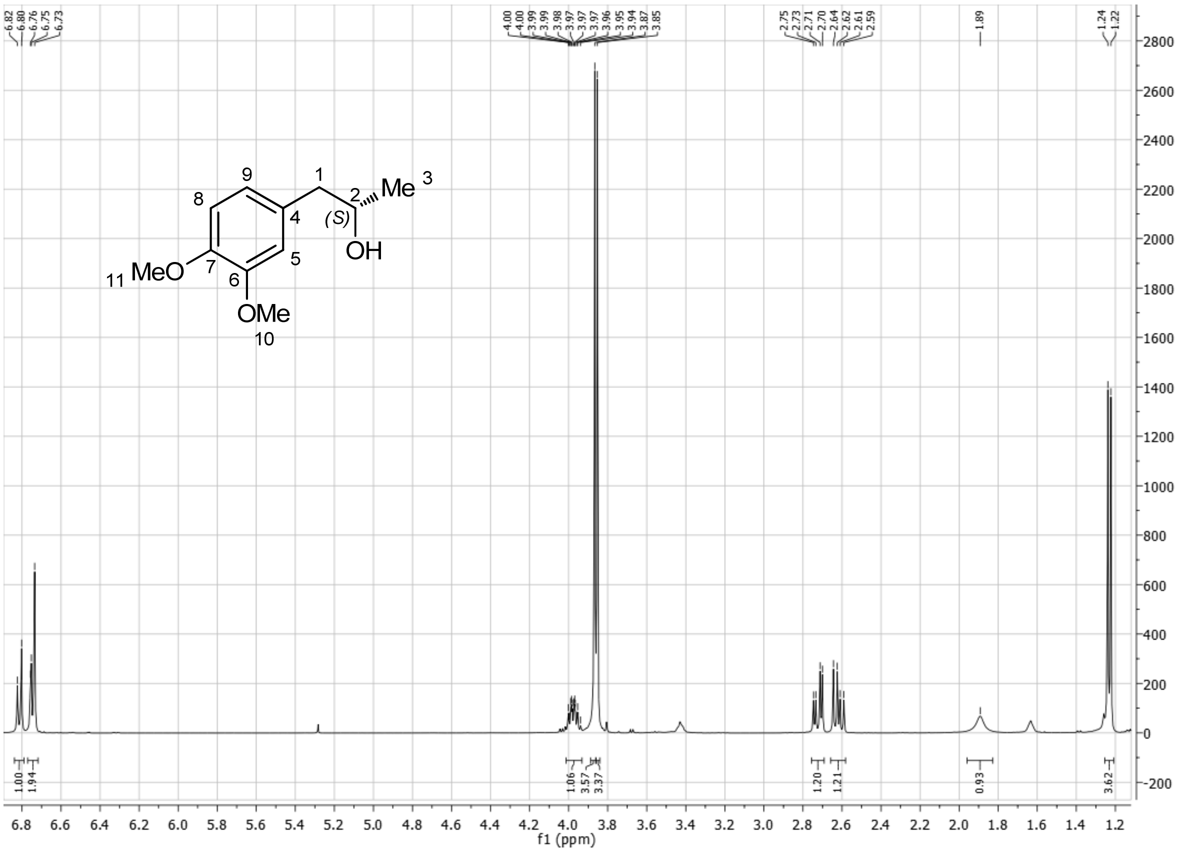

Figure S1. ^{1}H NMR (400 MHz) spectrum of (*S*)-**11** in $CDCl_3$.

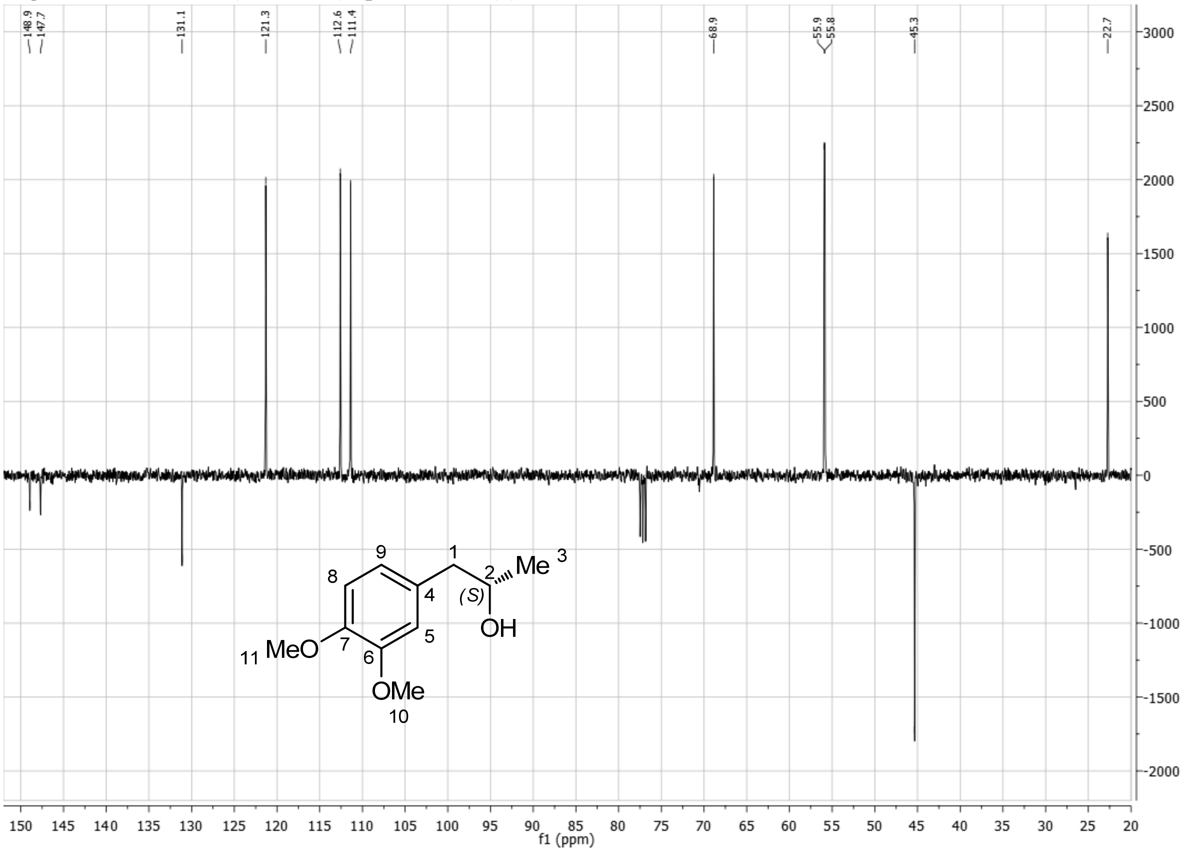

Figure S2. ^{13}C NMR (100 MHz) spectrum of (*S*)-11 in $CDCl_3$.

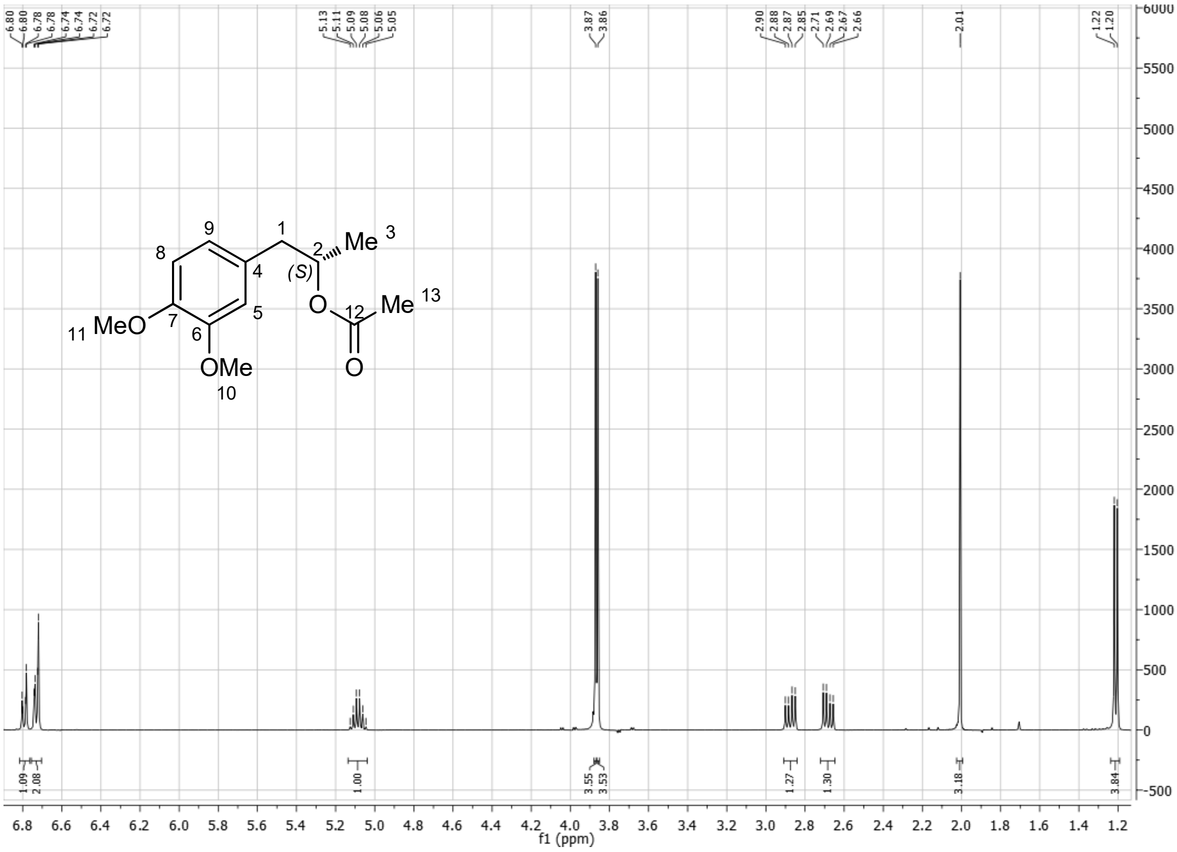

Figure S3. ^{1}H NMR (400 MHz) spectrum of (*S*)-**12** in $CDCl_3$.

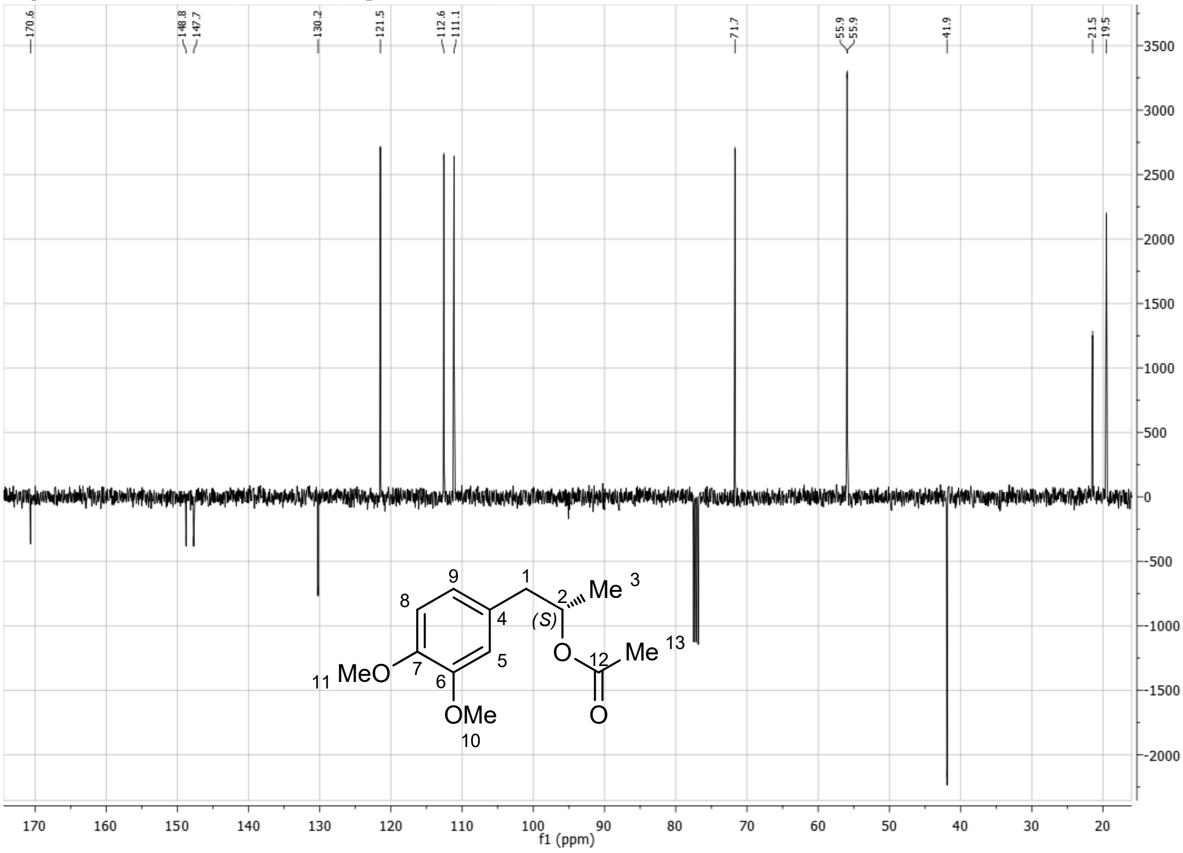

Figure S4. ^{13}C NMR (100 MHz) spectrum of (*S*)-**12** in $CDCl_3$.

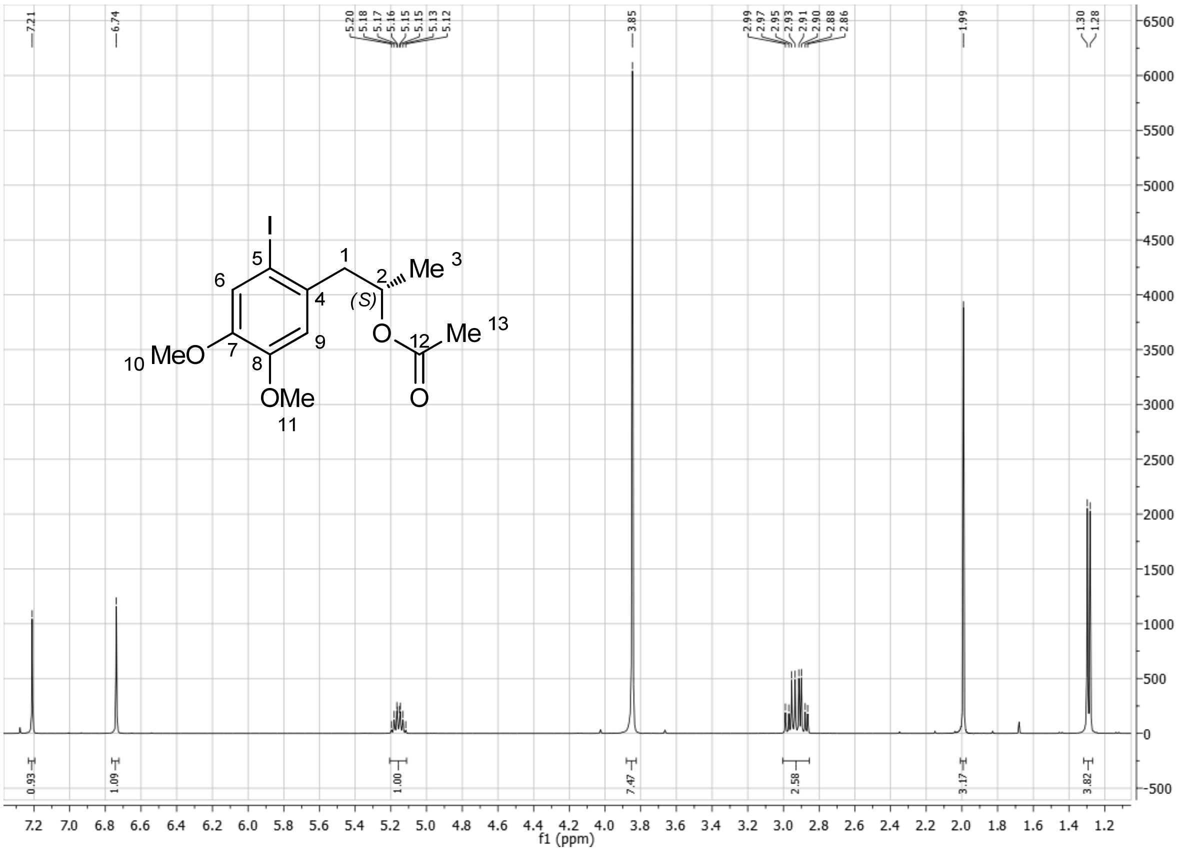

Figure S5. ^{1}H NMR (400 MHz) spectrum of (*S*)-**13** in $CDCl_3$.

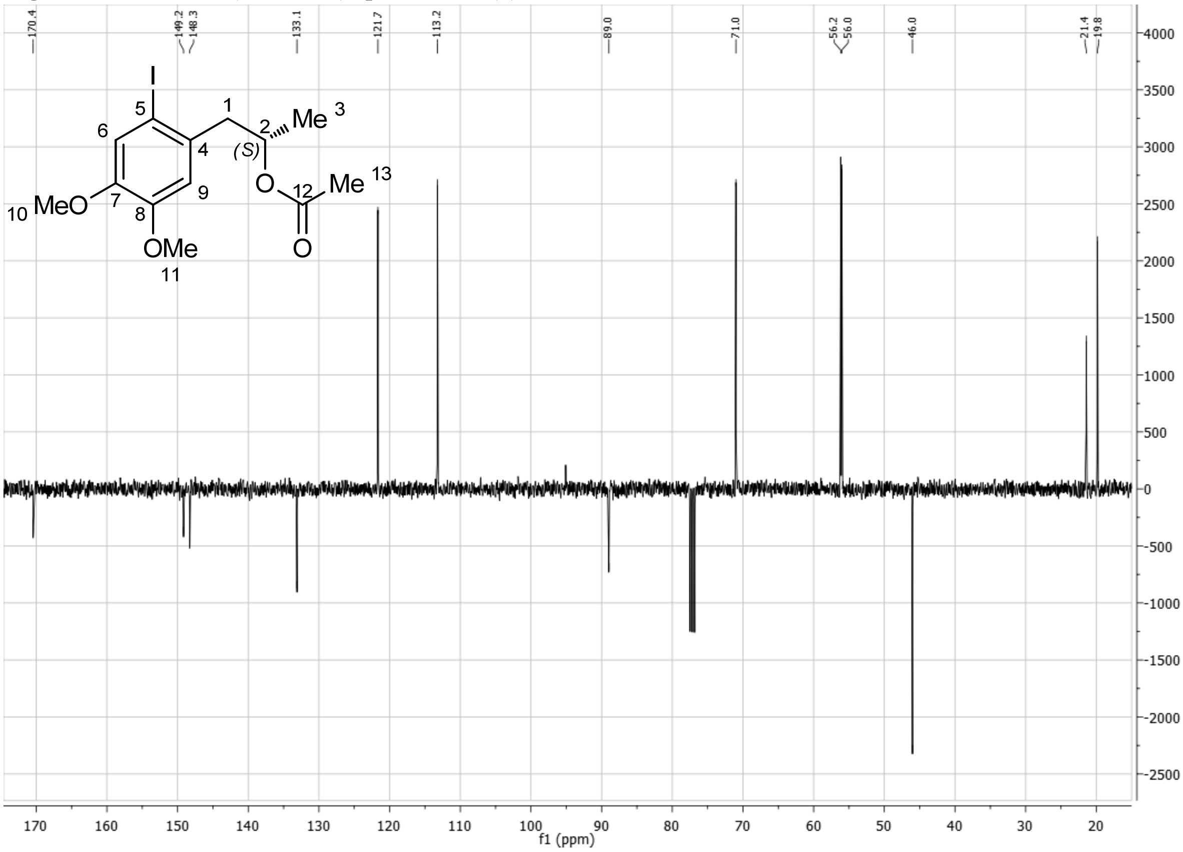

Figure S6. ^{13}C NMR (100 MHz) spectrum of (*S*)-**13** in $CDCl_3$.

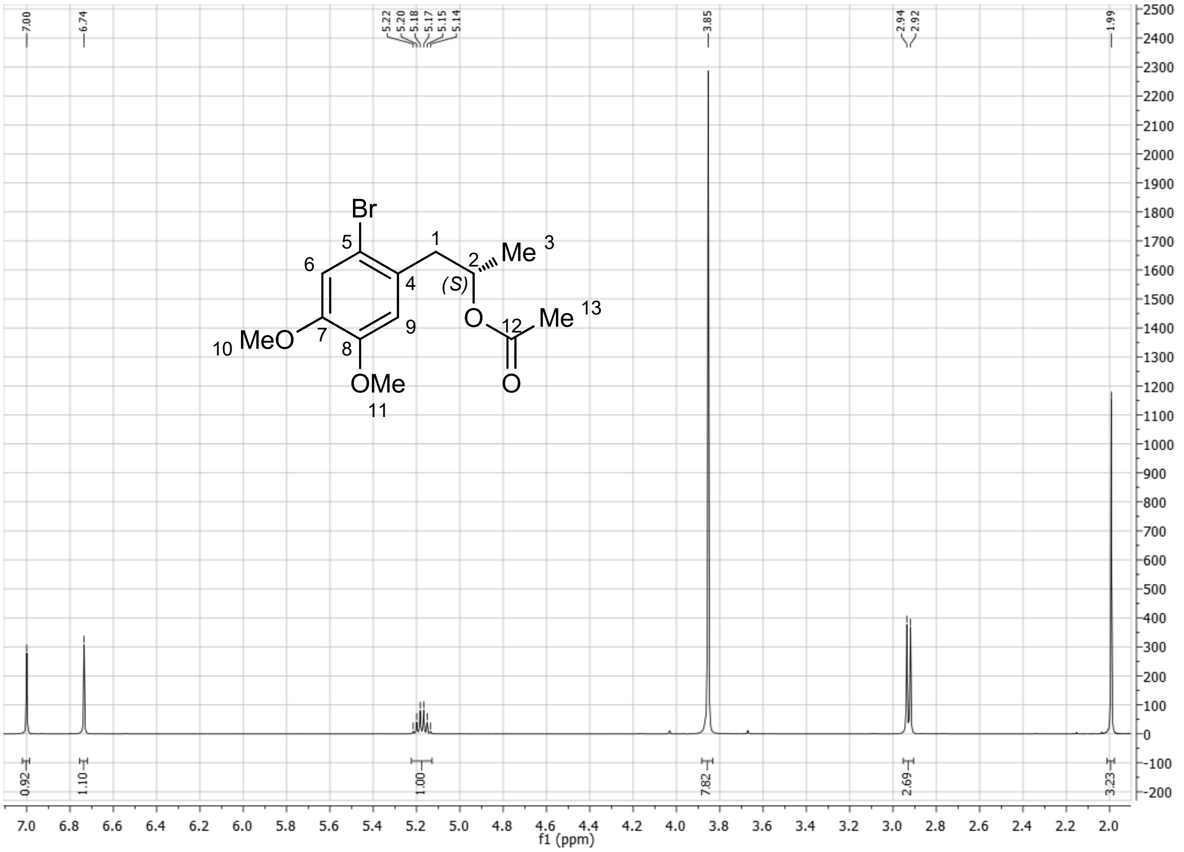

Figure S7. ^{1}H NMR (400 MHz) spectrum of (*S*)-**14** in $CDCl_3$.

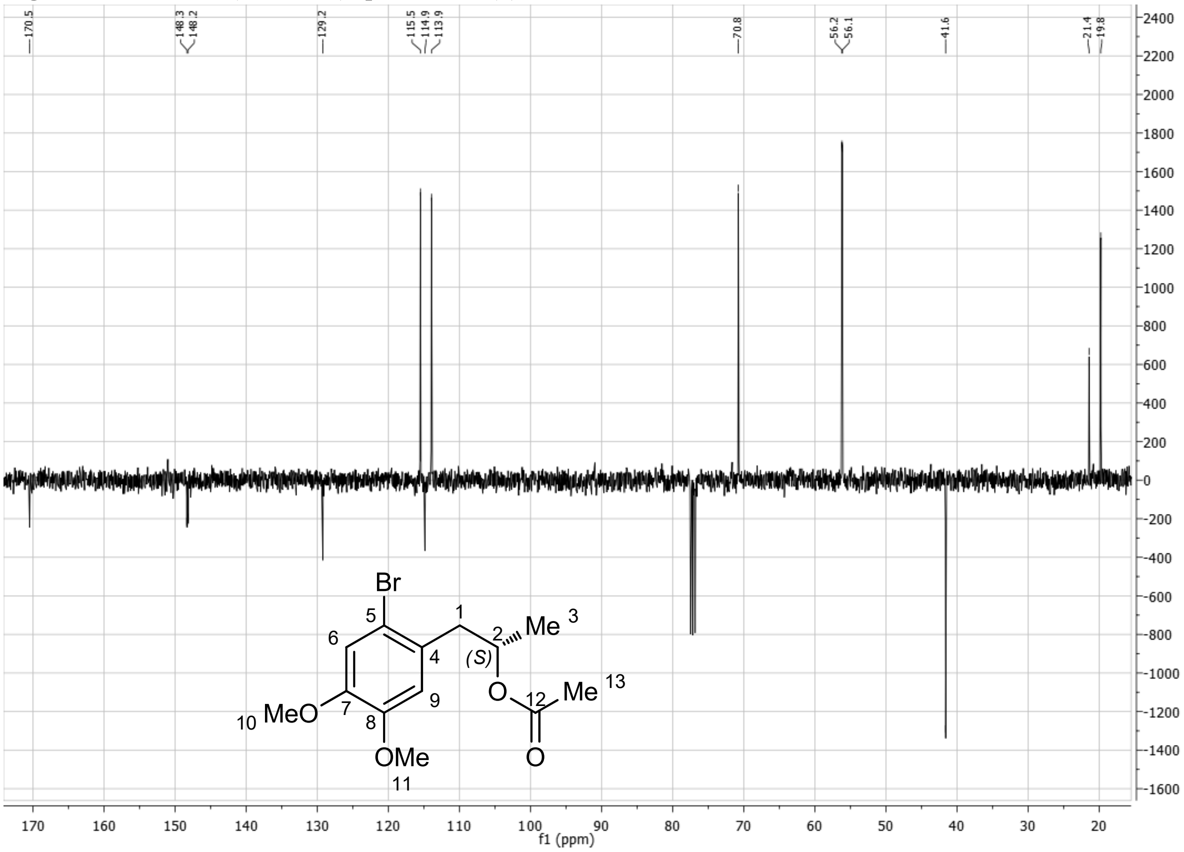

Figure S8. ^{13}C NMR (100 MHz) spectrum of (*S*)-**14** in $CDCl_3$.

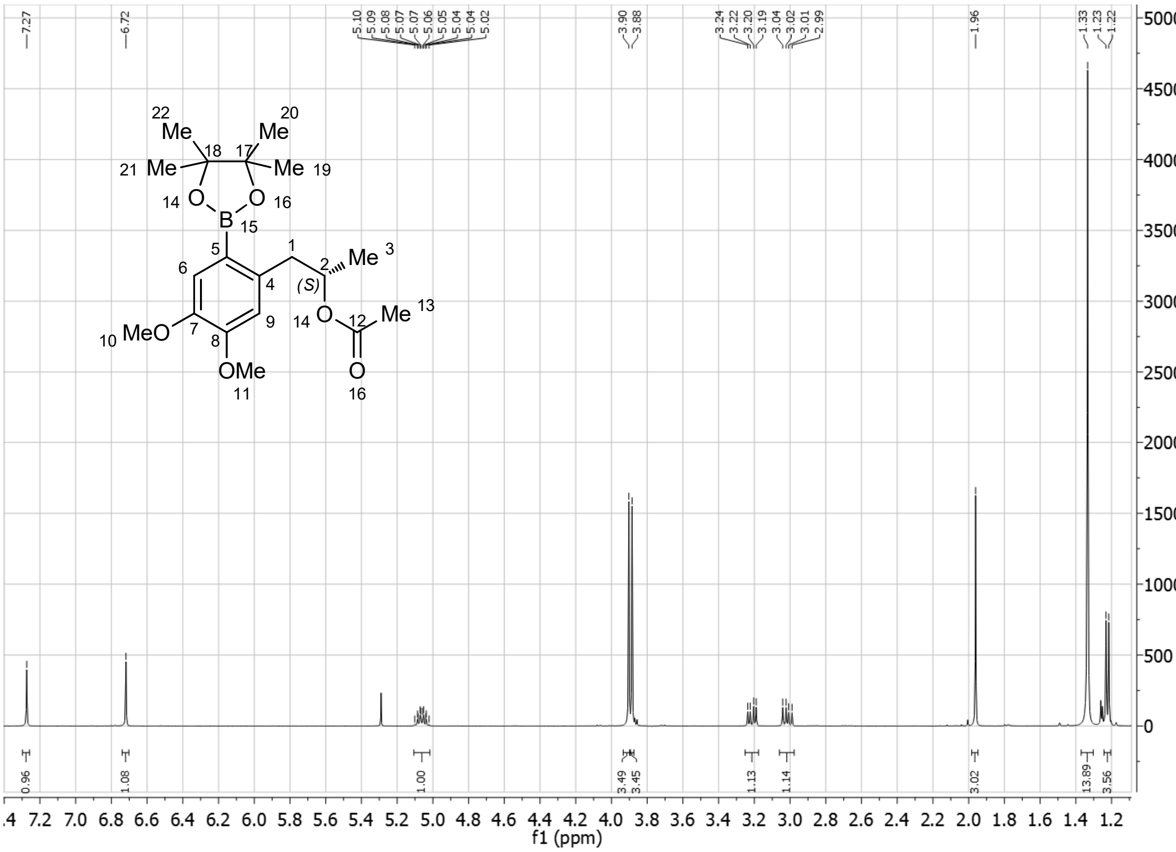

Figure S9. ^{1}H NMR (400 MHz) spectrum of (*S*)-**8** in $CDCl_3$.

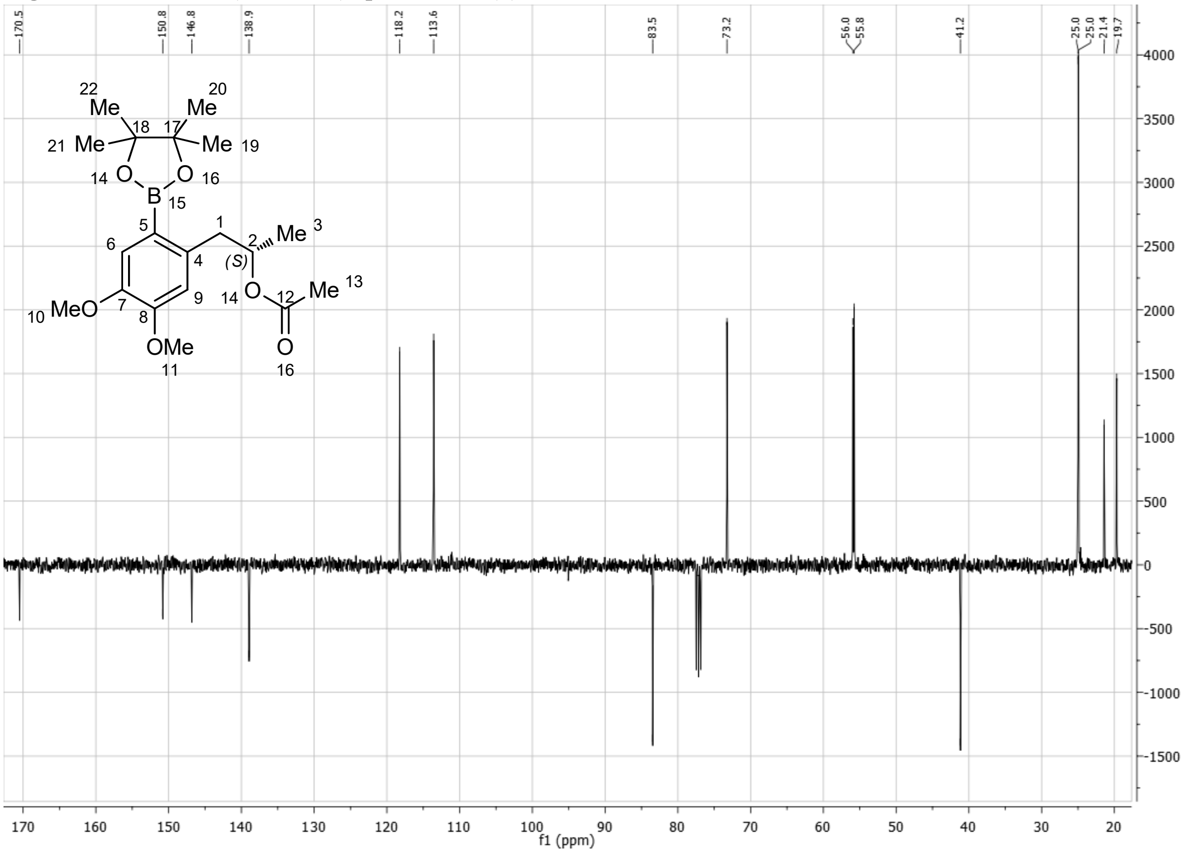

Figure S10. ^{13}C NMR (100 MHz) spectrum of (*S*)-**8** in $CDCl_3$.

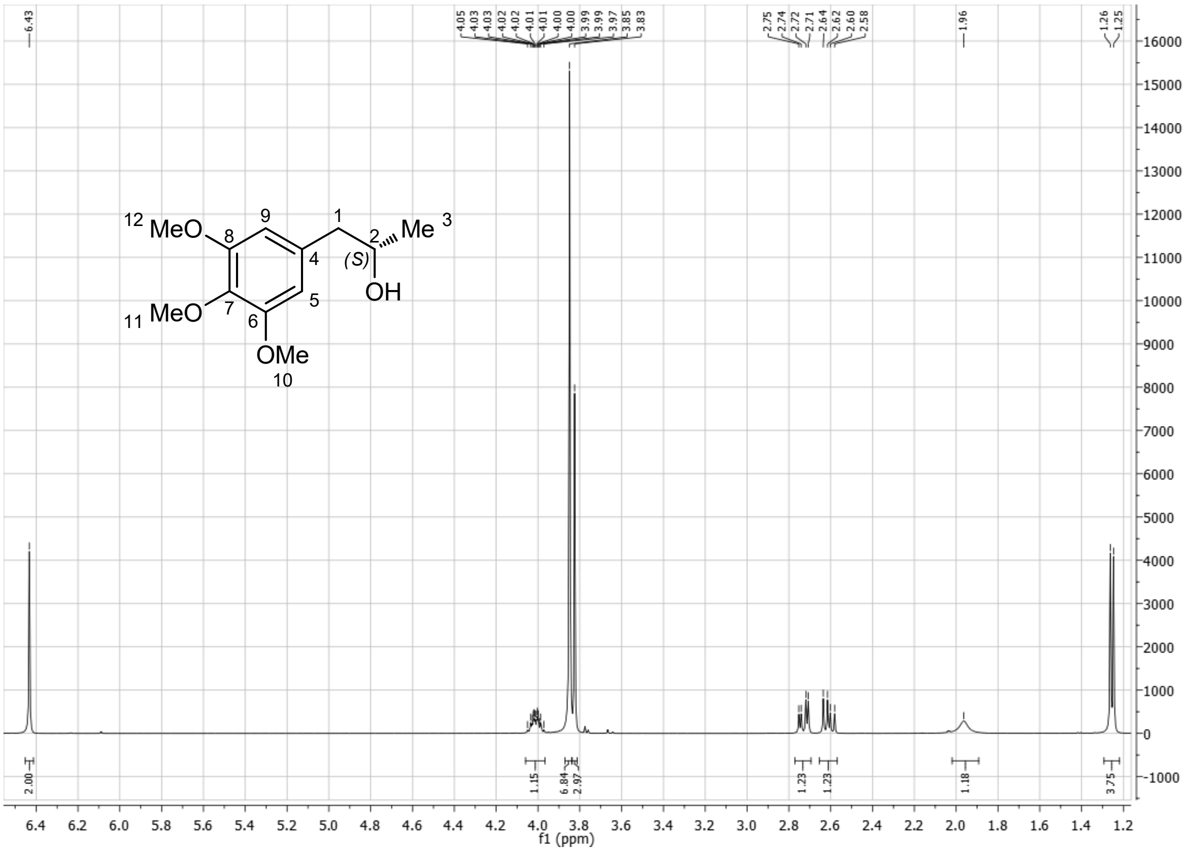

Figure S11. ^{1}H NMR (400 MHz) spectrum of (*S*)-**16** in $CDCl_3$.

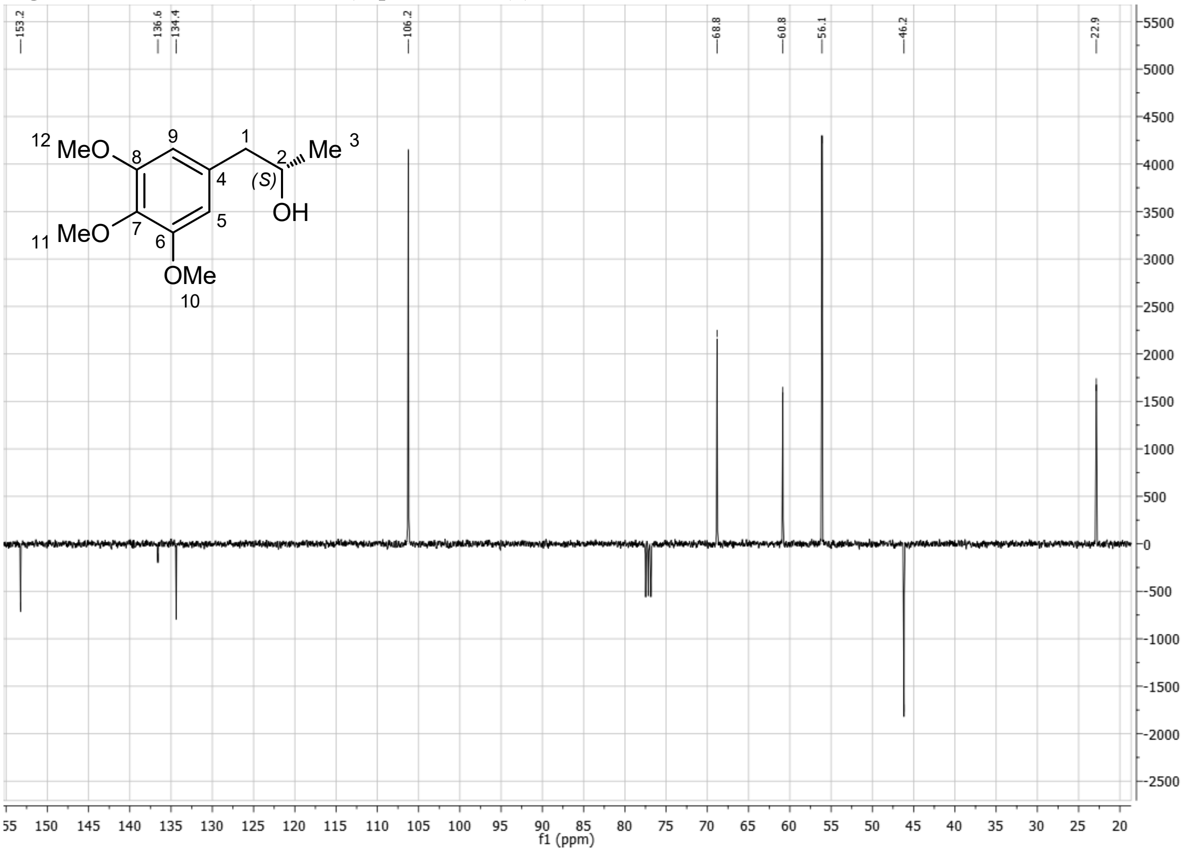

Figure S12. ^{13}C NMR (100 MHz) spectrum of (*S*)-**16** in $CDCl_3$.

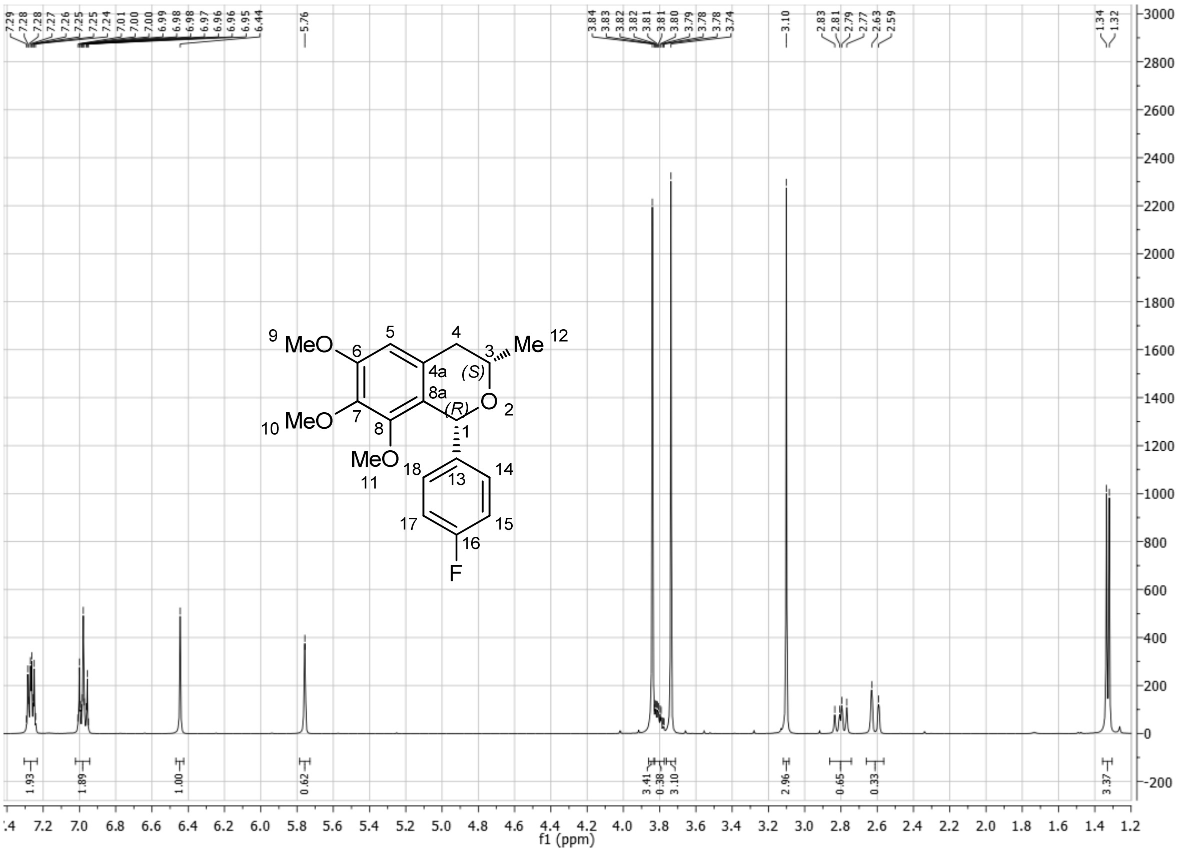

Figure S13. ^{1}H NMR (400 MHz) spectrum of *cis*-(1*R*,3*S*)-**17** in $CDCl_3$.

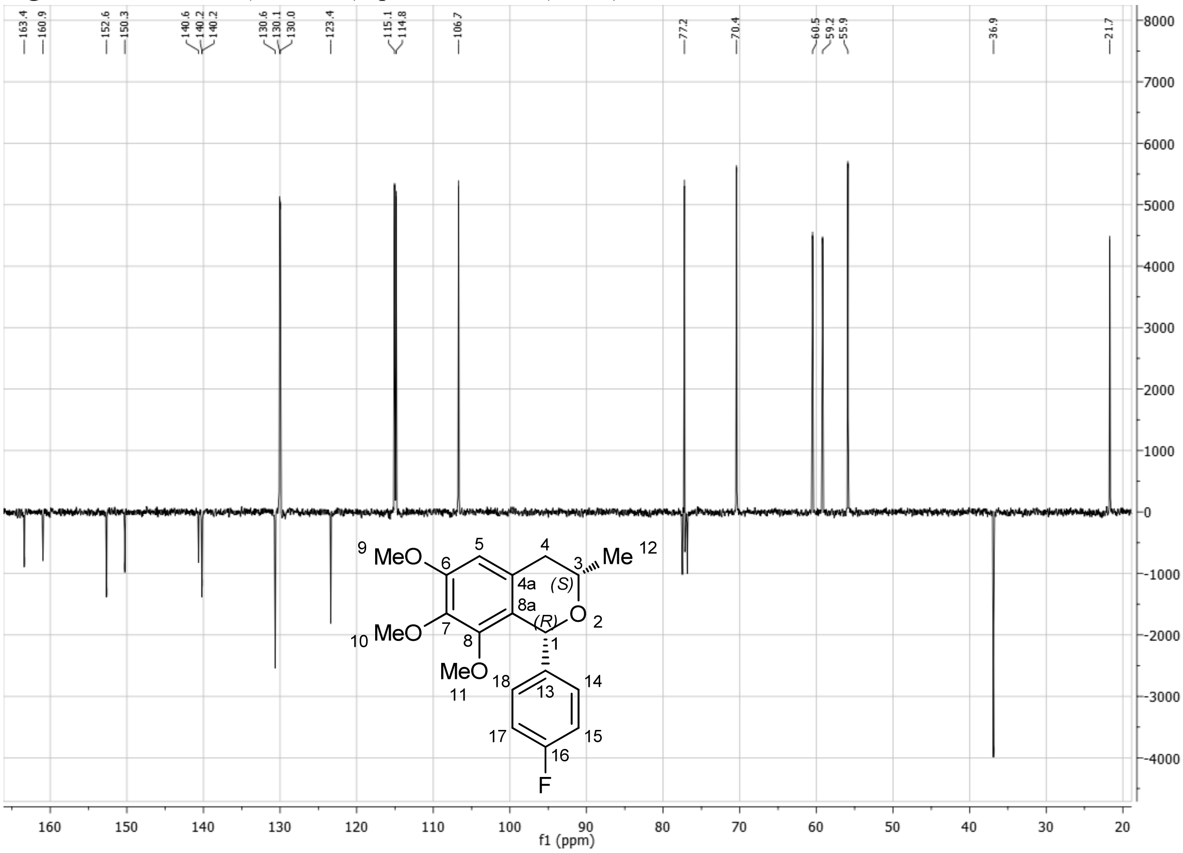

Figure S14. ^{13}C NMR (100 MHz) spectrum of *cis*-(1*R*,3*S*)-**17** in $CDCl_3$.

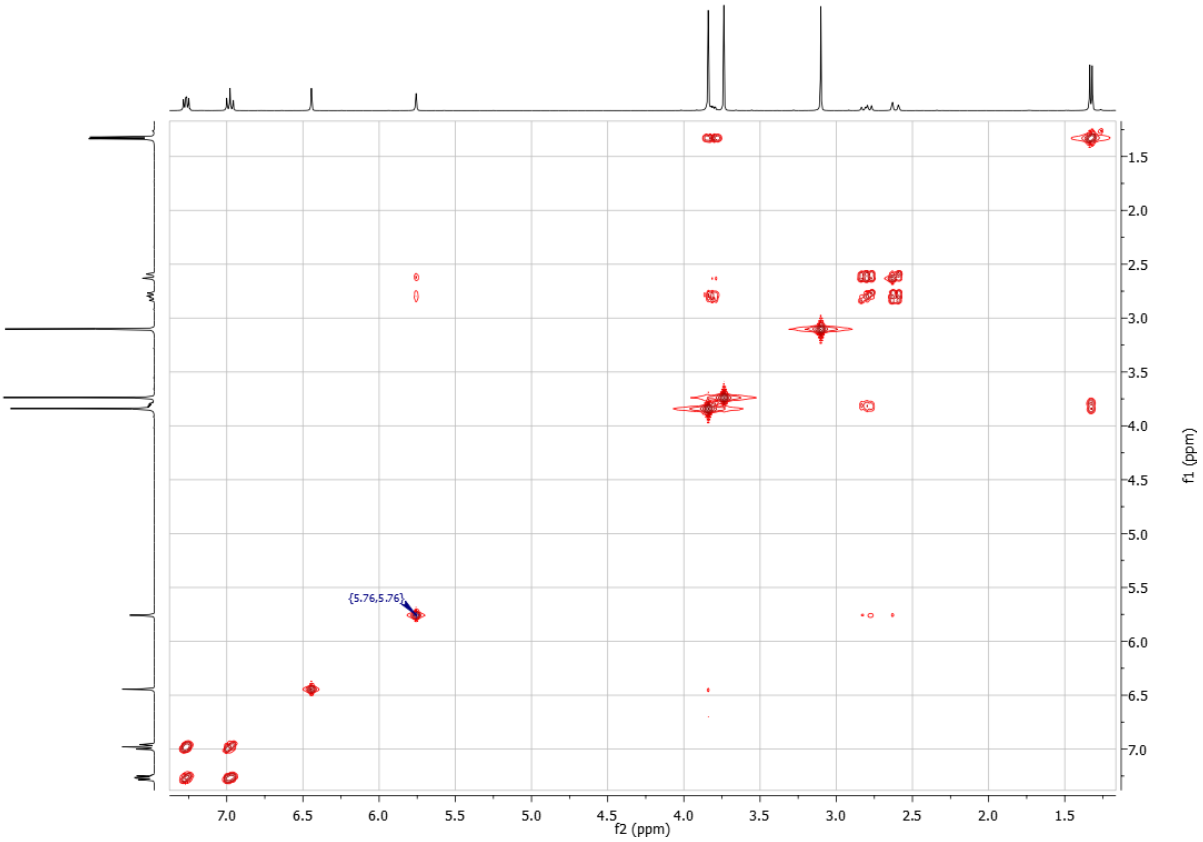

Figure S15. ^{1}H-^{1}H COSY NMR (400 MHz) spectrum of *cis*-(1*R*,3*S*)-**17** in $CDCl_3$.

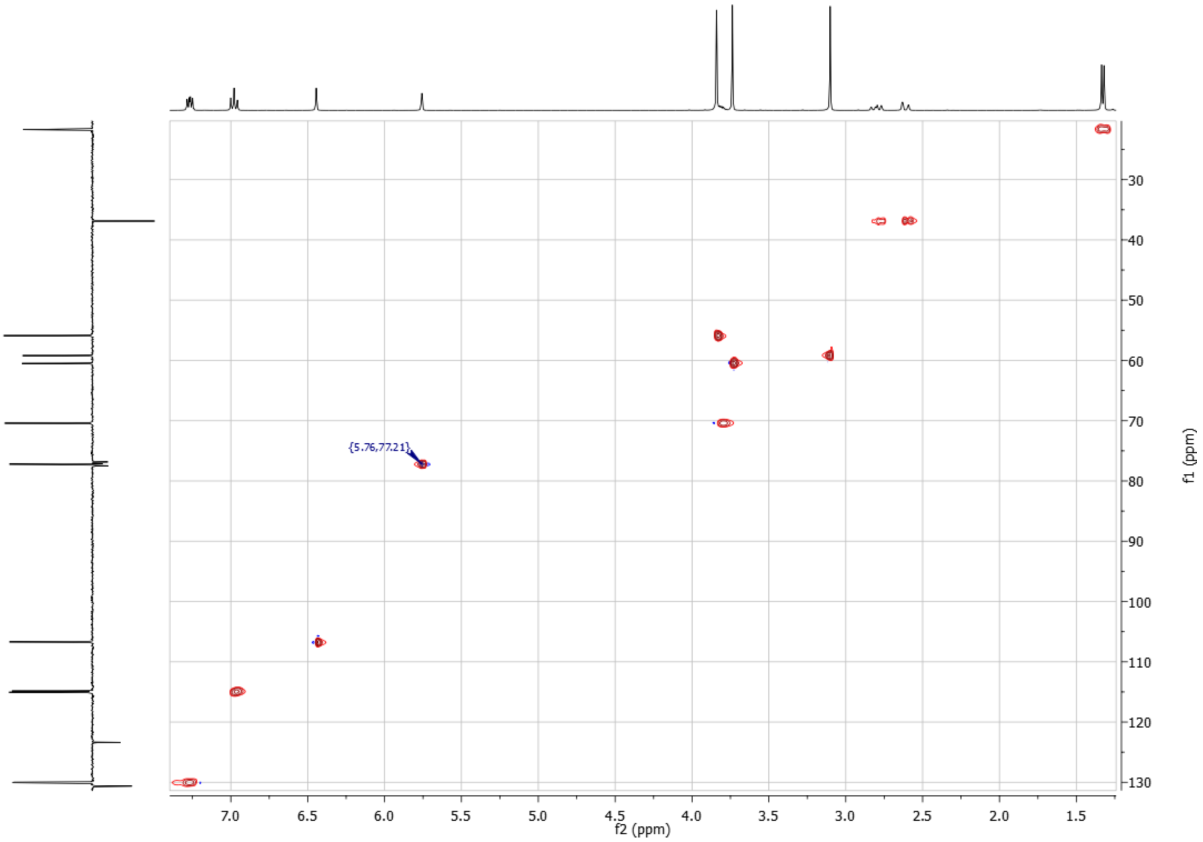

Figure S16. ^{1}H-^{13}C HSQC NMR (400 MHz) spectrum of *cis*-(1*R*,3*S*)-**17** in $CDCl_3$.

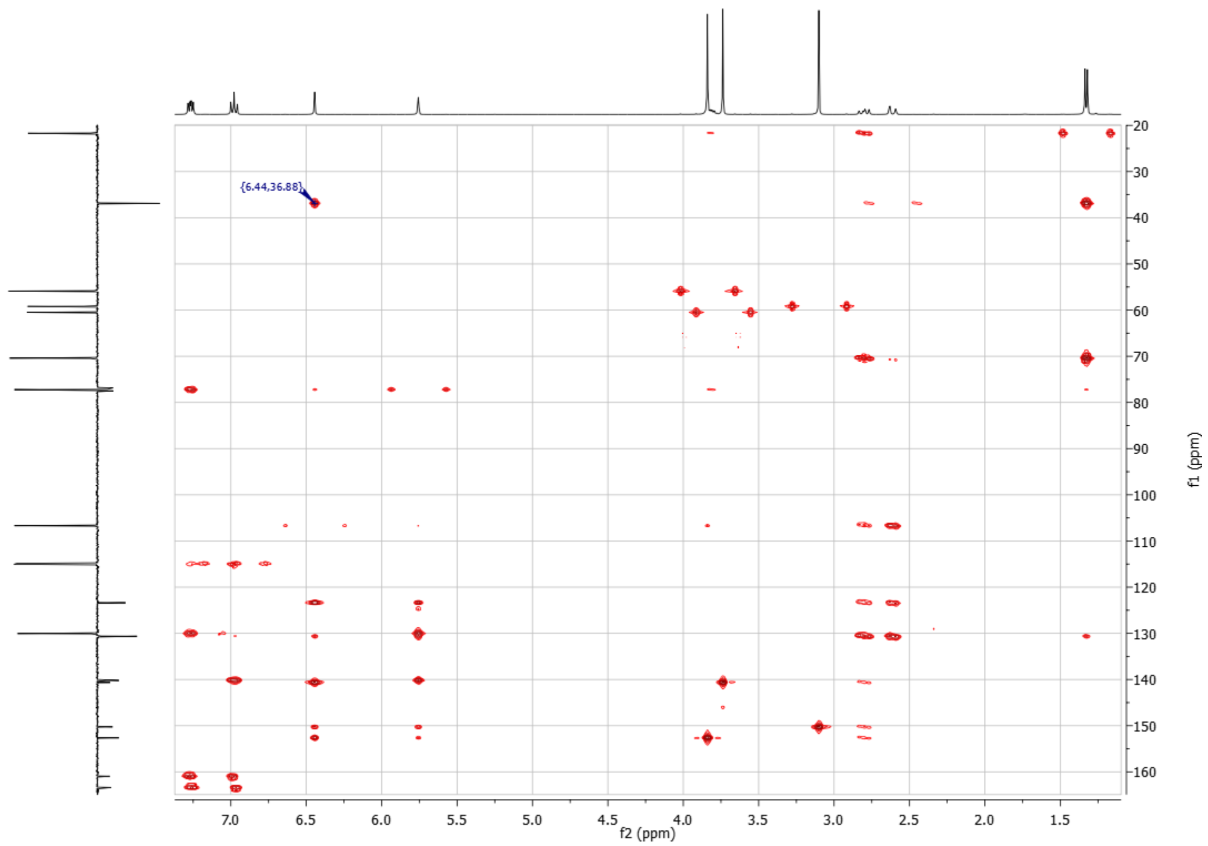

Figure S17. ^{1}H-^{13}C HMBC NMR (400 MHz) spectrum of *cis*-(1*R*,3*S*)-**17** in $CDCl_3$.

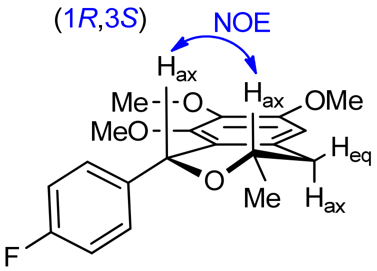

Figure S18. Characteristic NOE correlation shown on the structure of *cis*-(1*R*,3*S*)-**17** suggesting (1*R*,3*S*) configuration of isochroman unit.

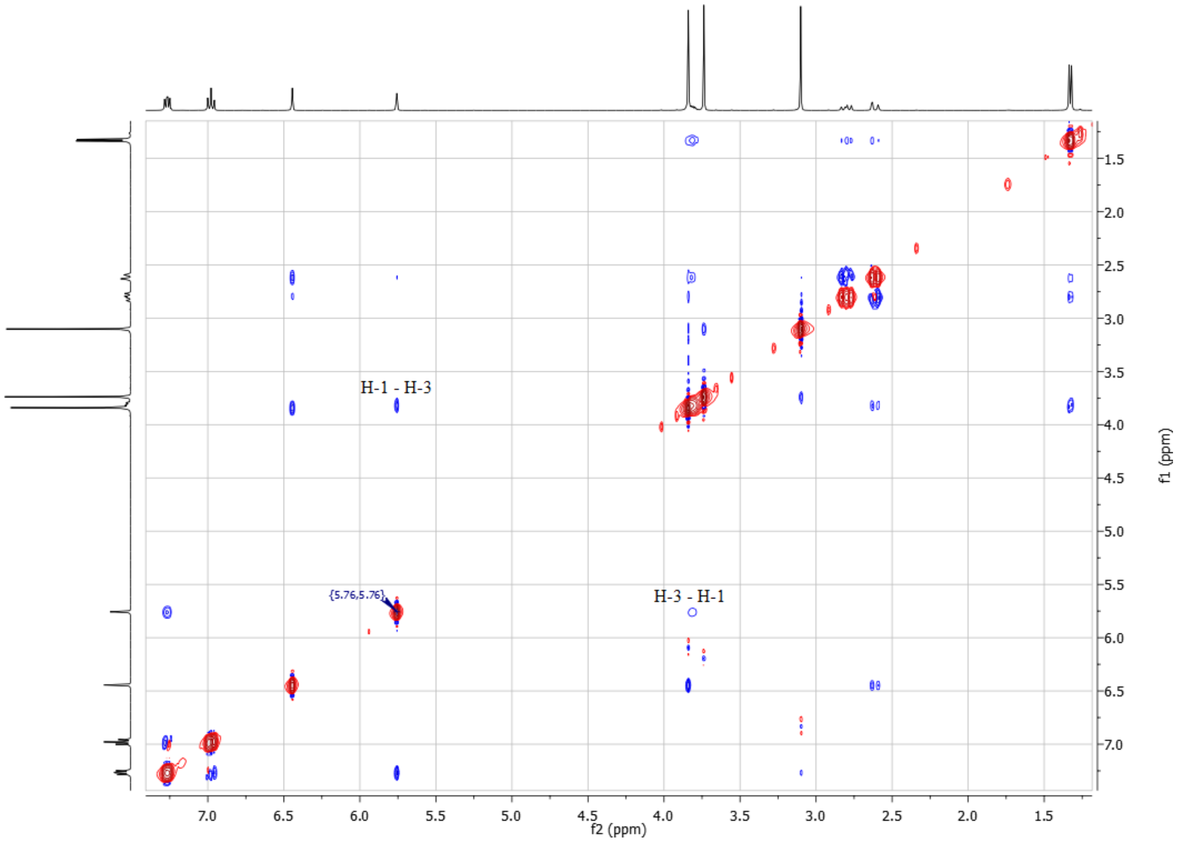

Figure S19. ^{1}H-^{1}H NOESY NMR (400 MHz) spectrum of *cis*-(1*R*,3*S*)-**17** in $CDCl_3$.

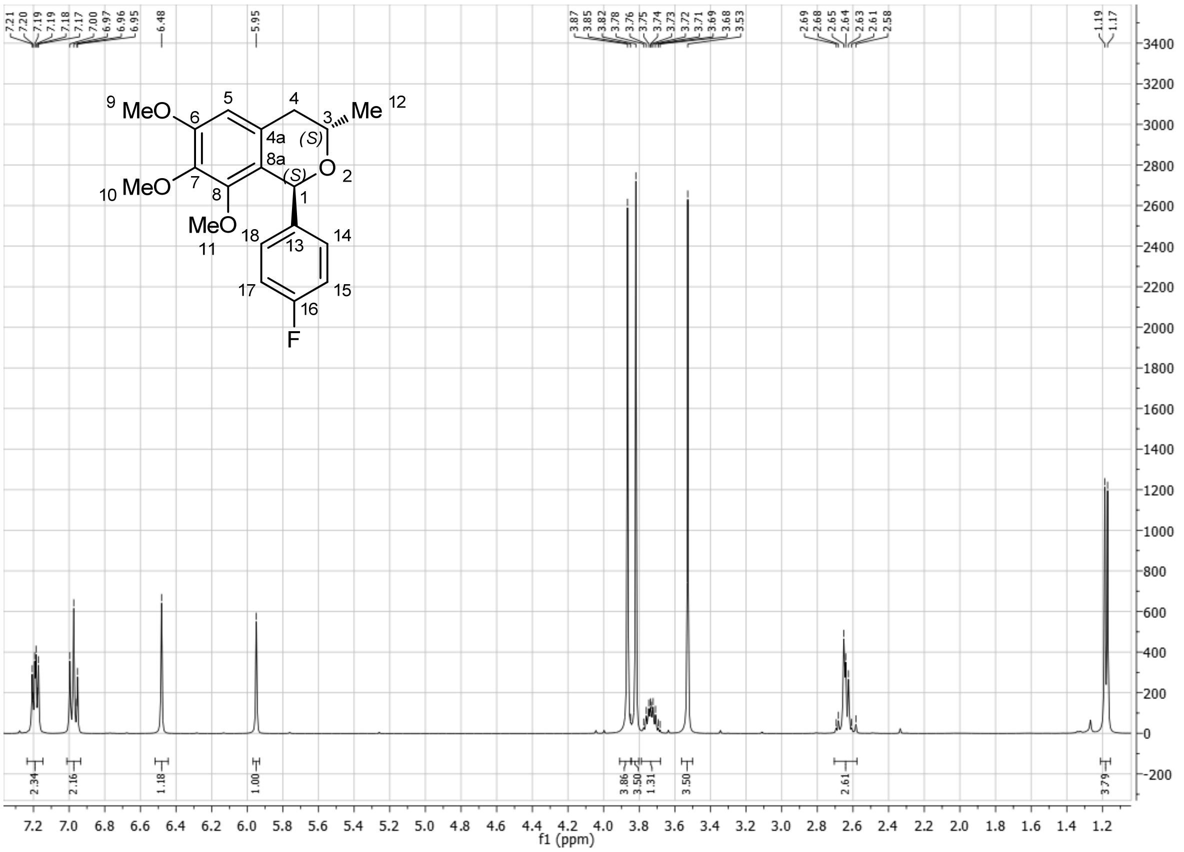

Figure S20. ^{1}H NMR (400 MHz) spectrum of *trans*-(1*S*,3*S*)-**17** in $CDCl_3$.

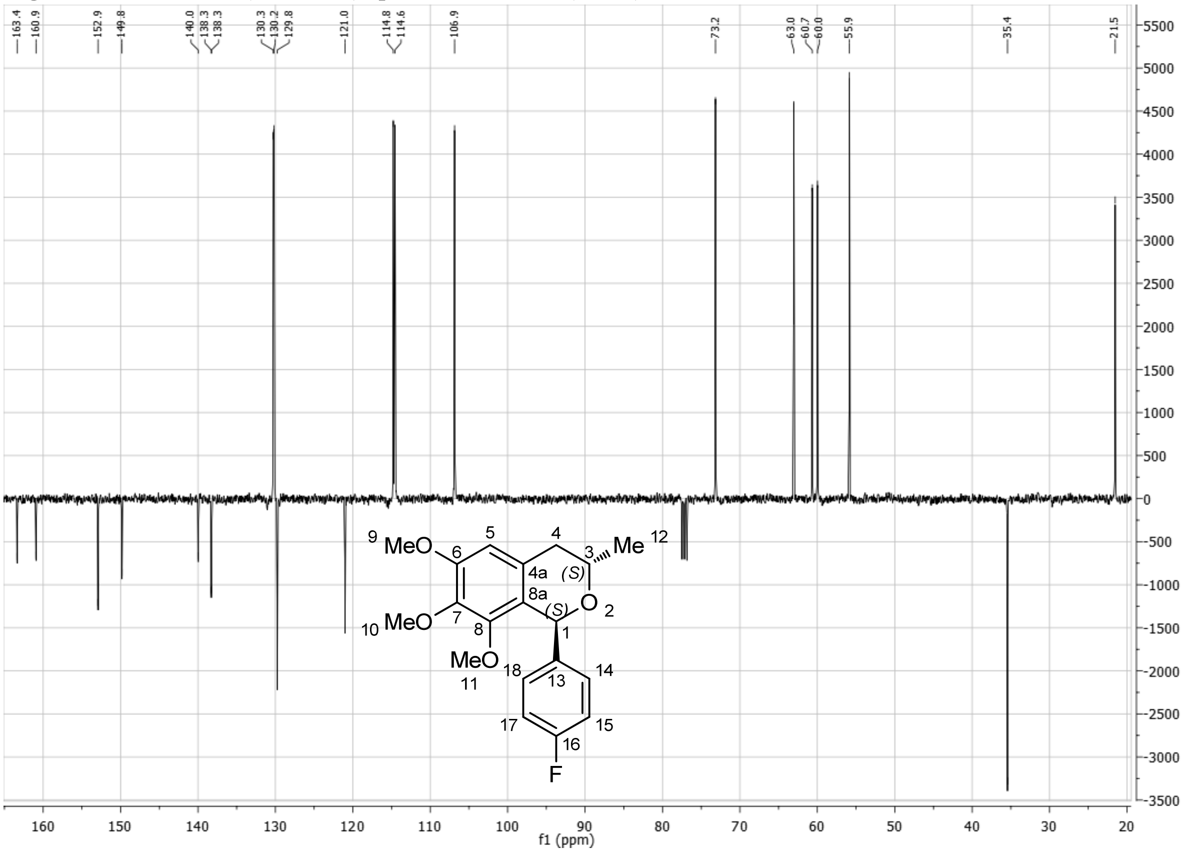

Figure S21. ^{13}C NMR (100 MHz) spectrum of *trans*-(1*S*,3*S*)-**17** in $CDCl_3$.

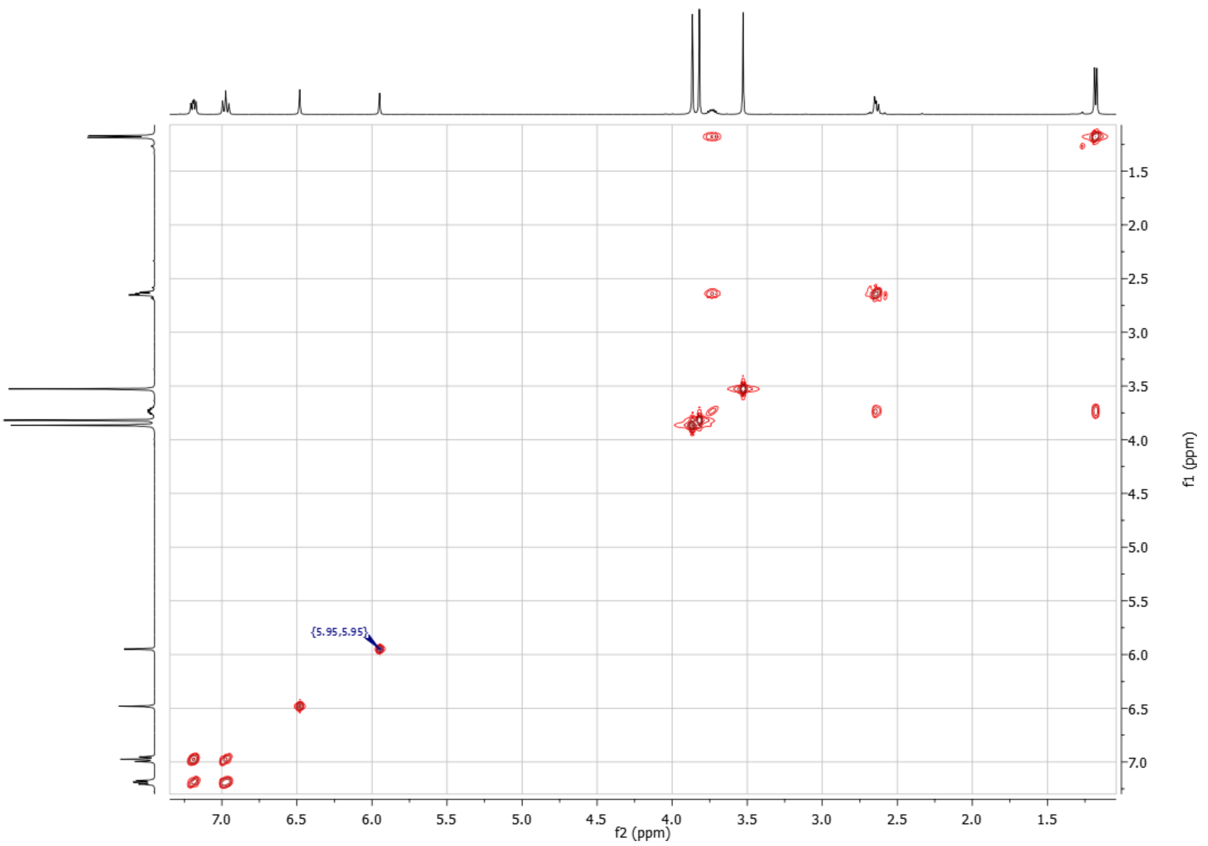

Figure S22. ^{1}H-^{1}H COSY NMR (400 MHz) spectrum of *trans*-(1*S*,3*S*)-**17** in $CDCl_3$.

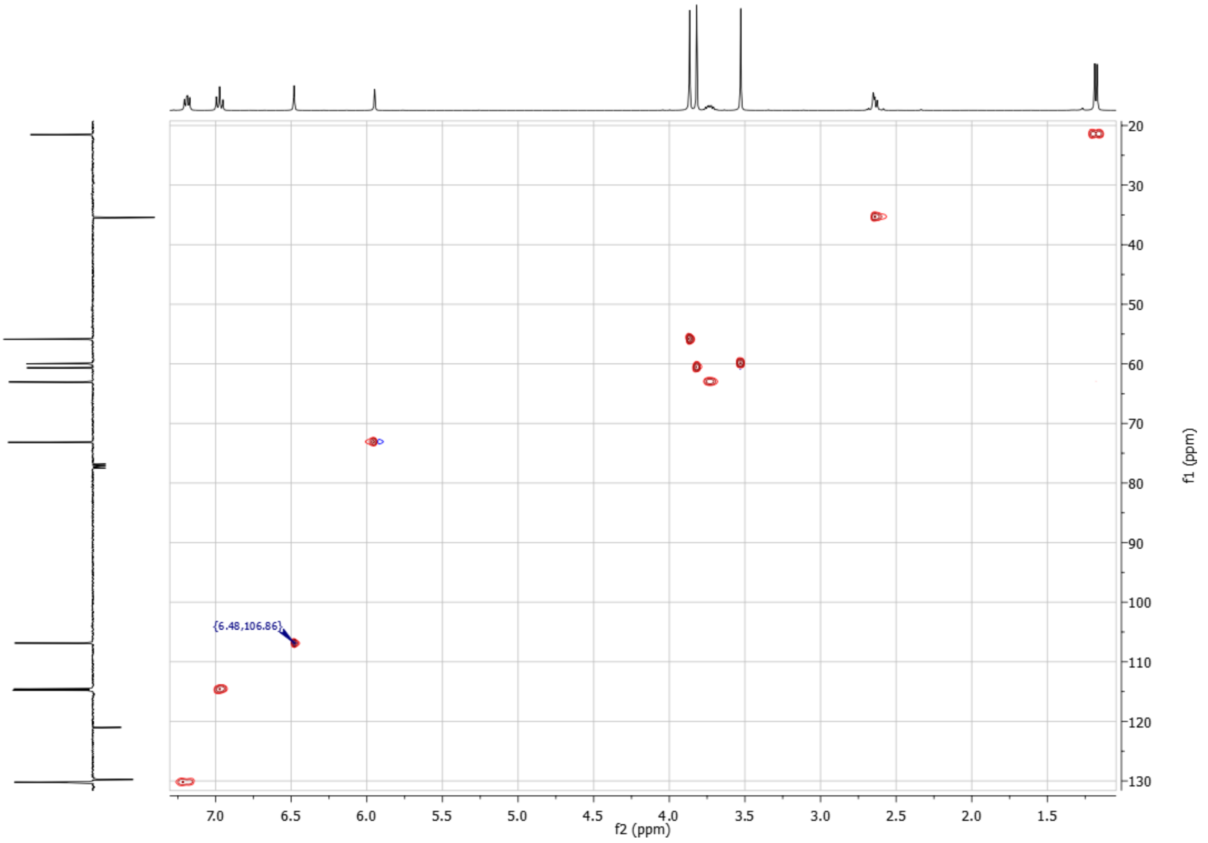

Figure S23. ^{1}H-^{13}C HSQC NMR (400 MHz) spectrum of *trans*-(1*S*,3*S*)-**17** in $CDCl_3$.

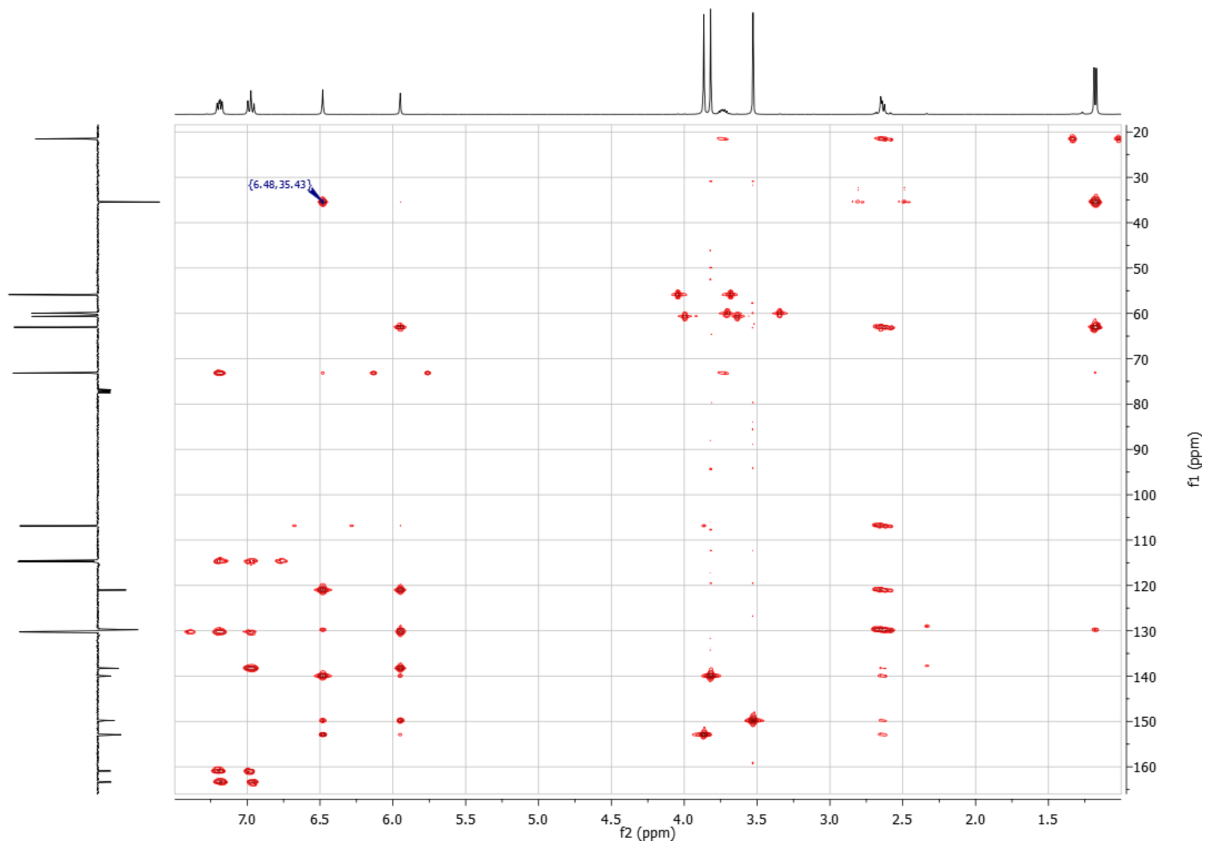

Figure S24. ^{1}H-^{13}C HMBC NMR (400 MHz) spectrum of *trans*-(1*S*,3*S*)-**17** in $CDCl_3$.

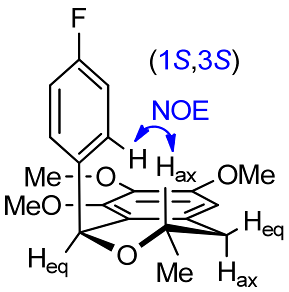

Figure S25. Characteristic NOE correlation shown on the structure of *trans*-(1*S*,3*S*)-**17** suggesting (1*S*,3*S*) configuration of isochroman unit.

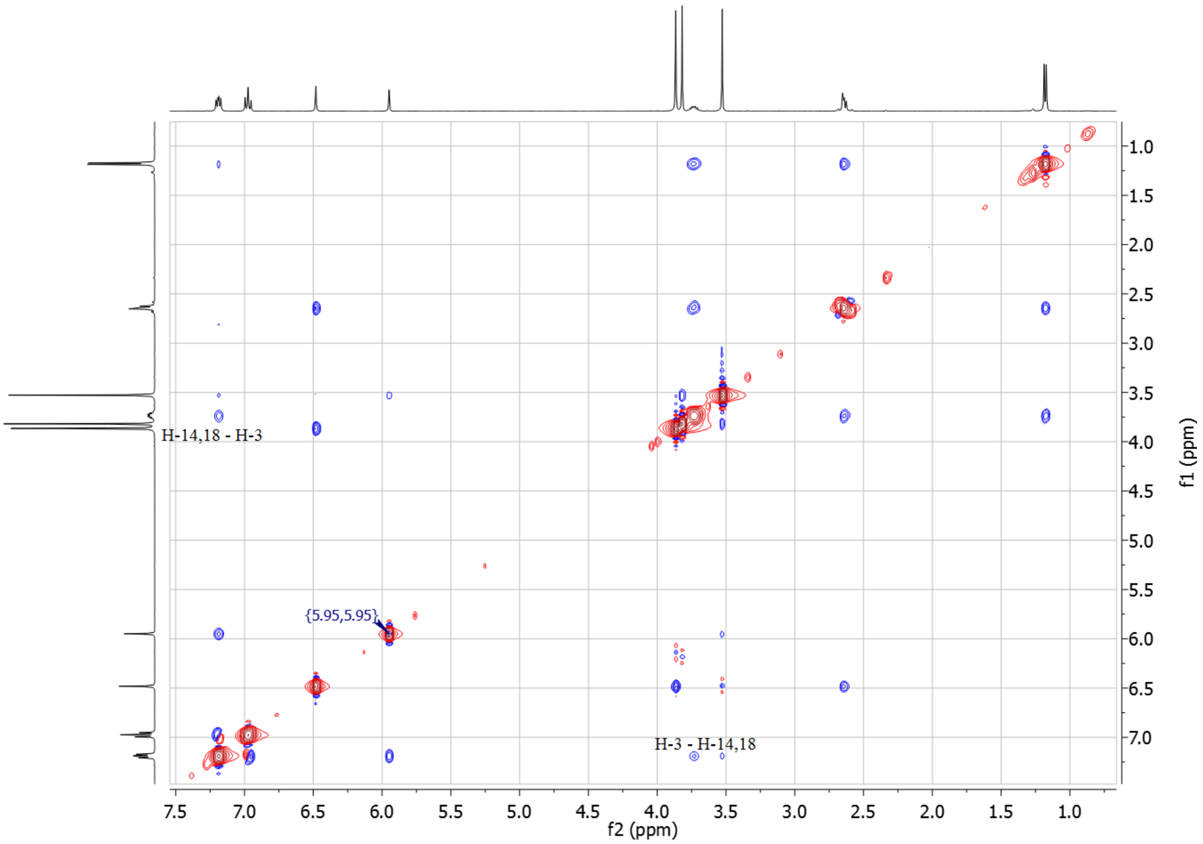

Figure S26. ^{1}H-^{1}H NOESY NMR (400 MHz) spectrum of *trans*-(1*S*,3*S*)-**17** in $CDCl_3$.

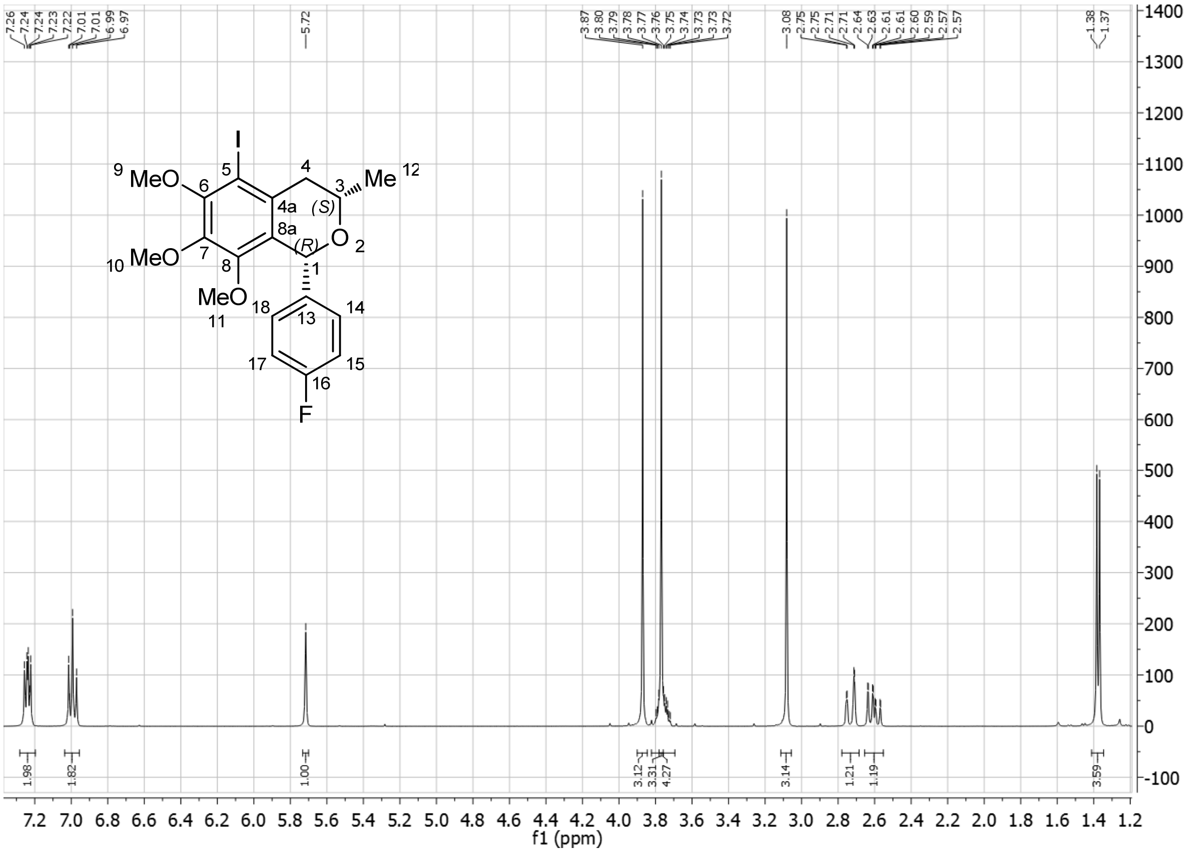

Figure S27. ^{1}H NMR (400 MHz) spectrum of *cis*-(1*R*,3*S*)-**18** in $CDCl_3$.

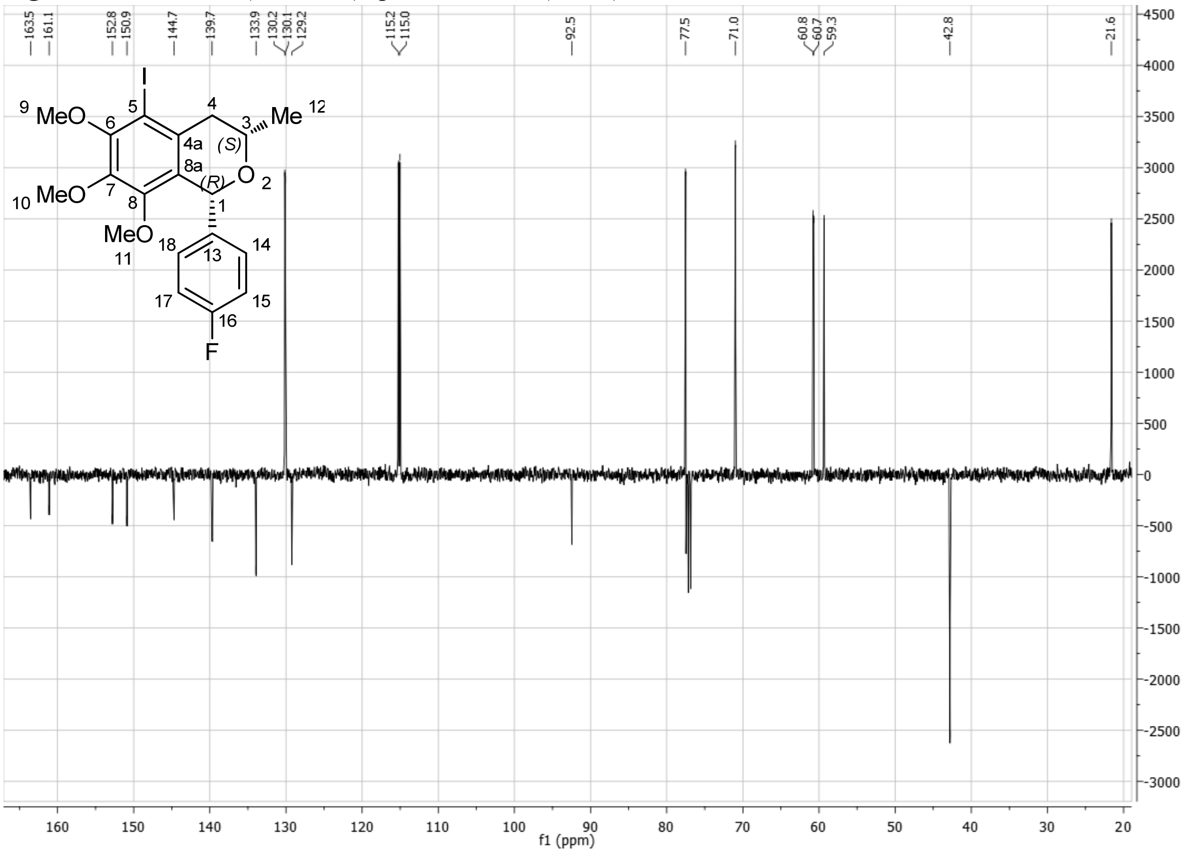

Figure S28. ^{13}C NMR (100 MHz) spectrum of *cis*-(1*R*,3*S*)-**18** in $CDCl_3$.

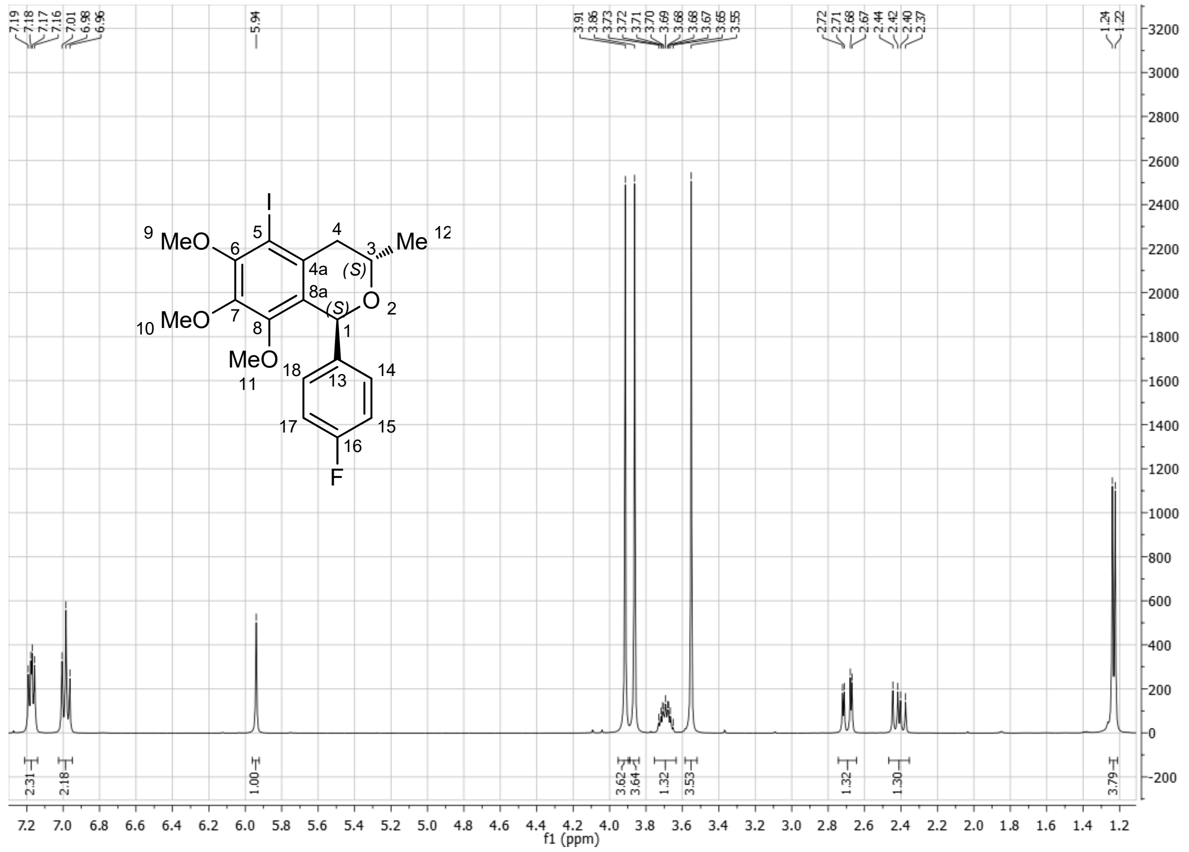

Figure S29. ^{1}H NMR (400 MHz) spectrum of *trans*-(1*S*,3*S*)-**18** in $CDCl_3$.

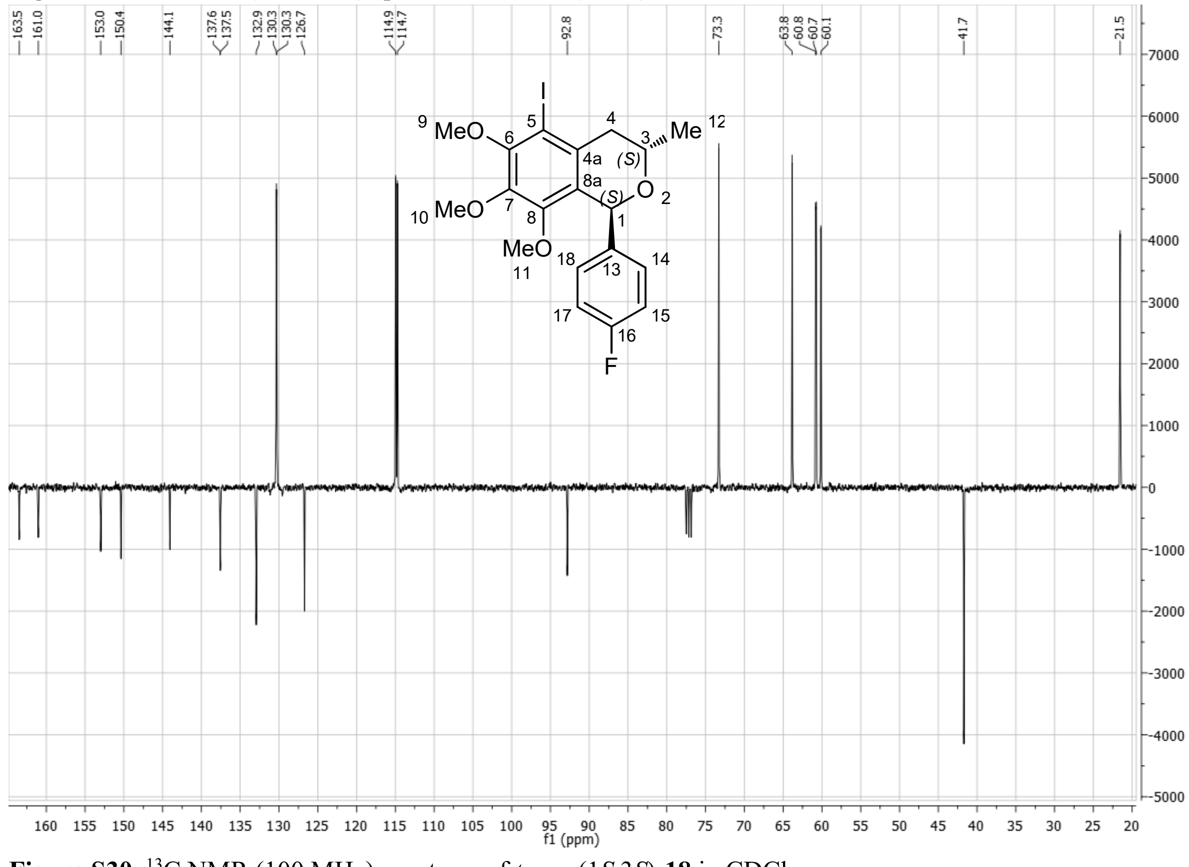

Figure S30. ^{13}C NMR (100 MHz) spectrum of *trans*-(1*S*,3*S*)-**18** in $CDCl_3$.

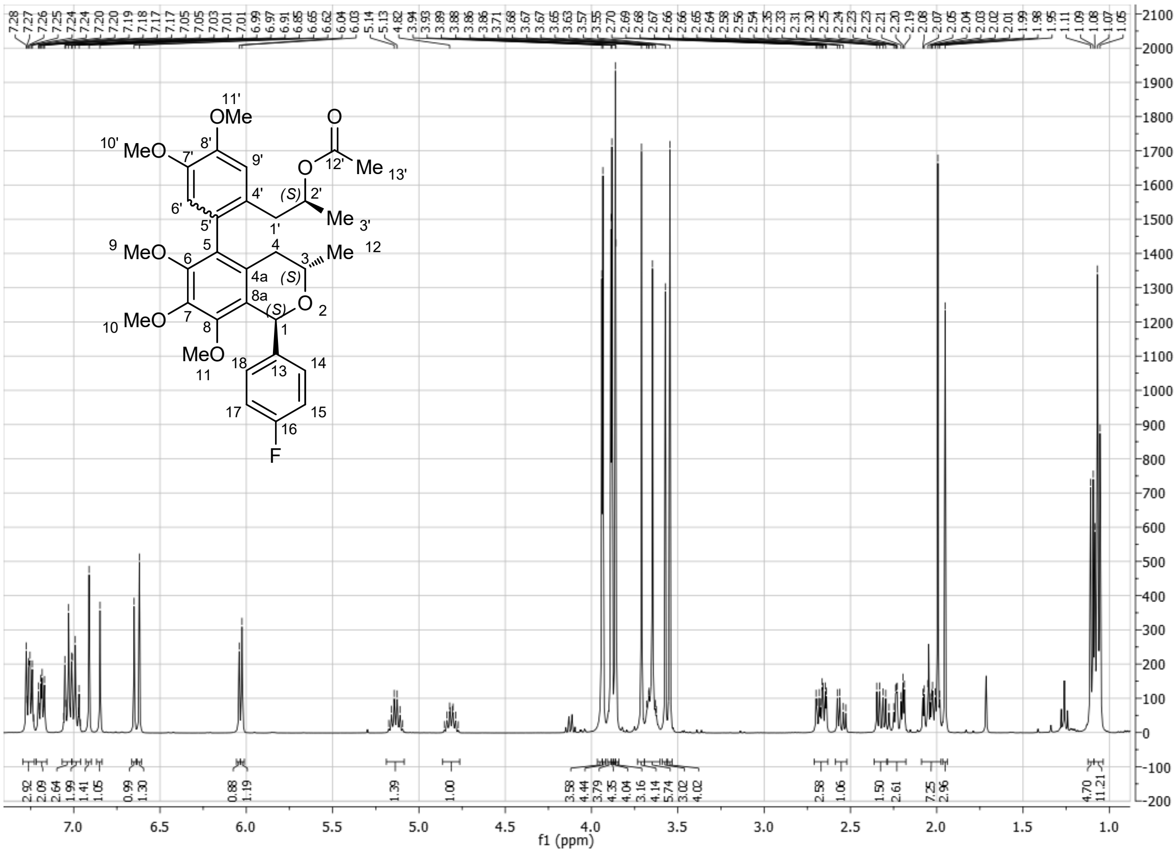

Figure S31. ^{1}H NMR (400 MHz) spectrum of the mixture of (a*R*,1*S*,3*S*,2'*S*)-**19** and (a*S*,1*S*,3*S*,2'*S*)-**19** in $CDCl_3$.

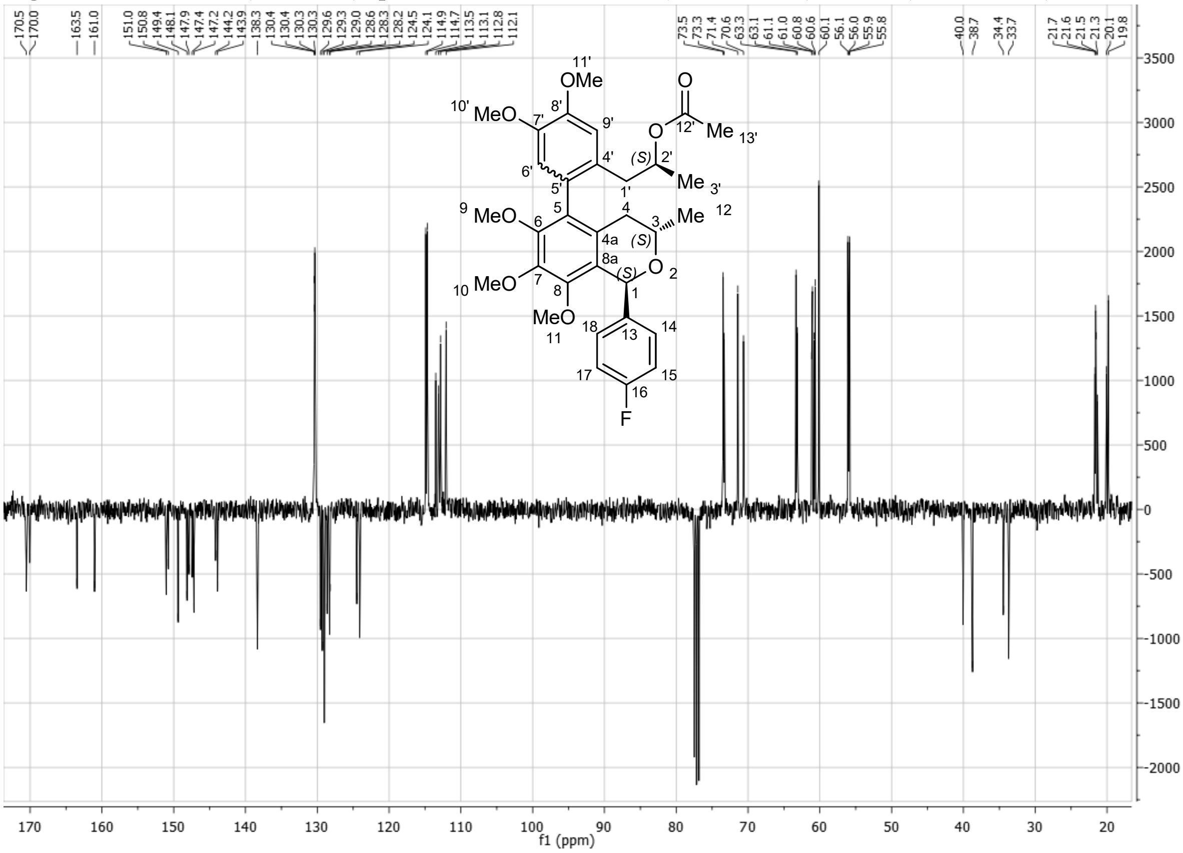

Figure S32. ^{13}C NMR (100 MHz) spectrum of the mixture of (a*R*,1*S*,3*S*,2'*S*)-**19** and (a*S*,1*S*,3*S*,2'*S*)-**19** in $CDCl_3$.

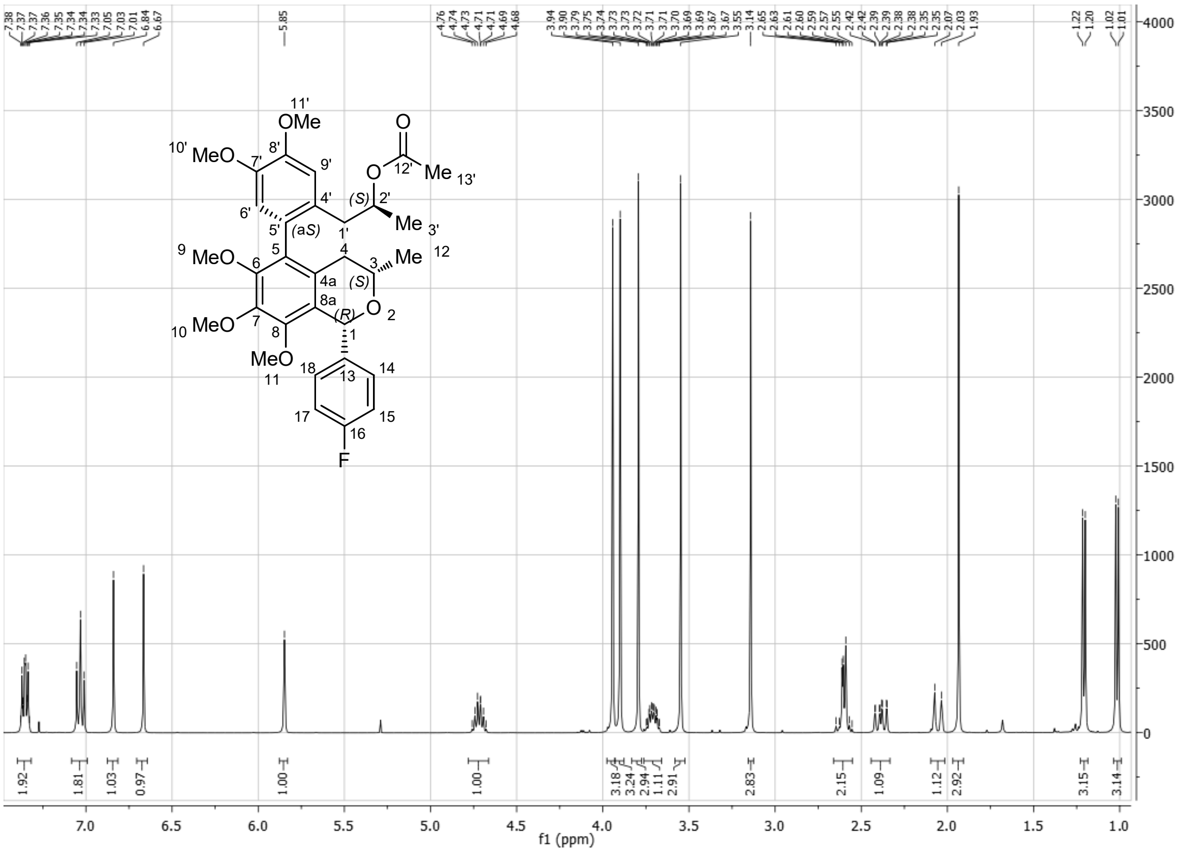

Figure S33. ^{1}H NMR (400 MHz) spectrum of (a*S*,1*R*,3*S*,2'*S*)-**19** in $CDCl_3$.

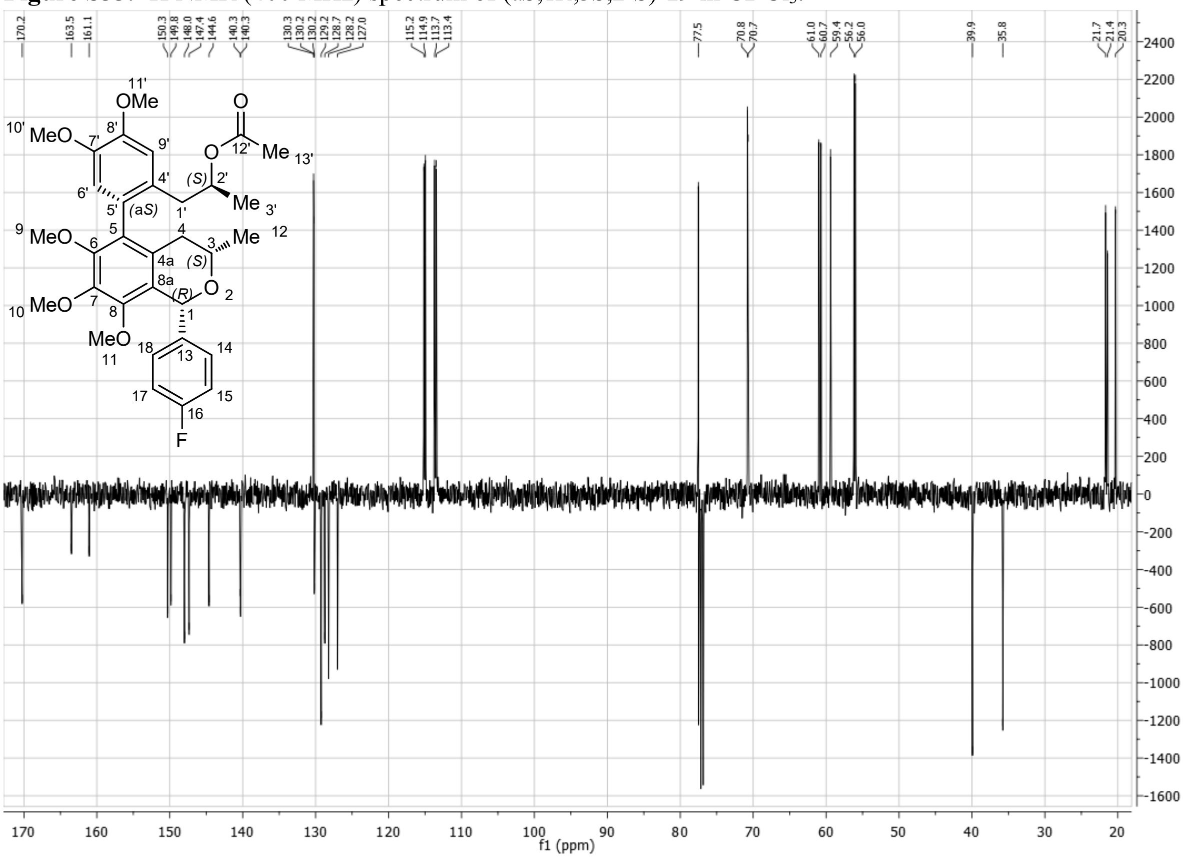

Figure S34. ^{13}C NMR (100 MHz) spectrum of (a*S*,1*R*,3*S*,2'*S*)-**19** in $CDCl_3$.

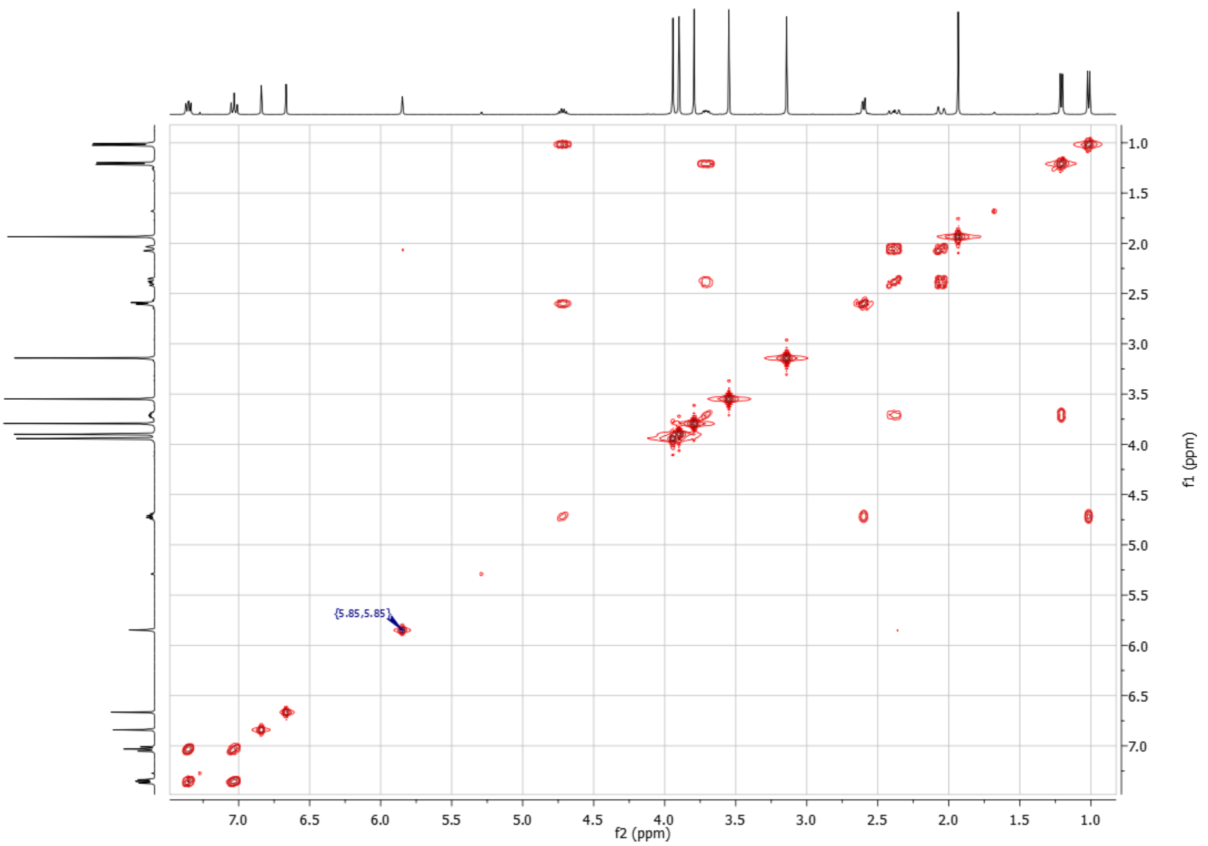

Figure S35. ^{1}H-^{1}H COSY NMR (400 MHz) spectrum of (a*S*,1*R*,3*S*,2'*S*)-**19** in $CDCl_3$.

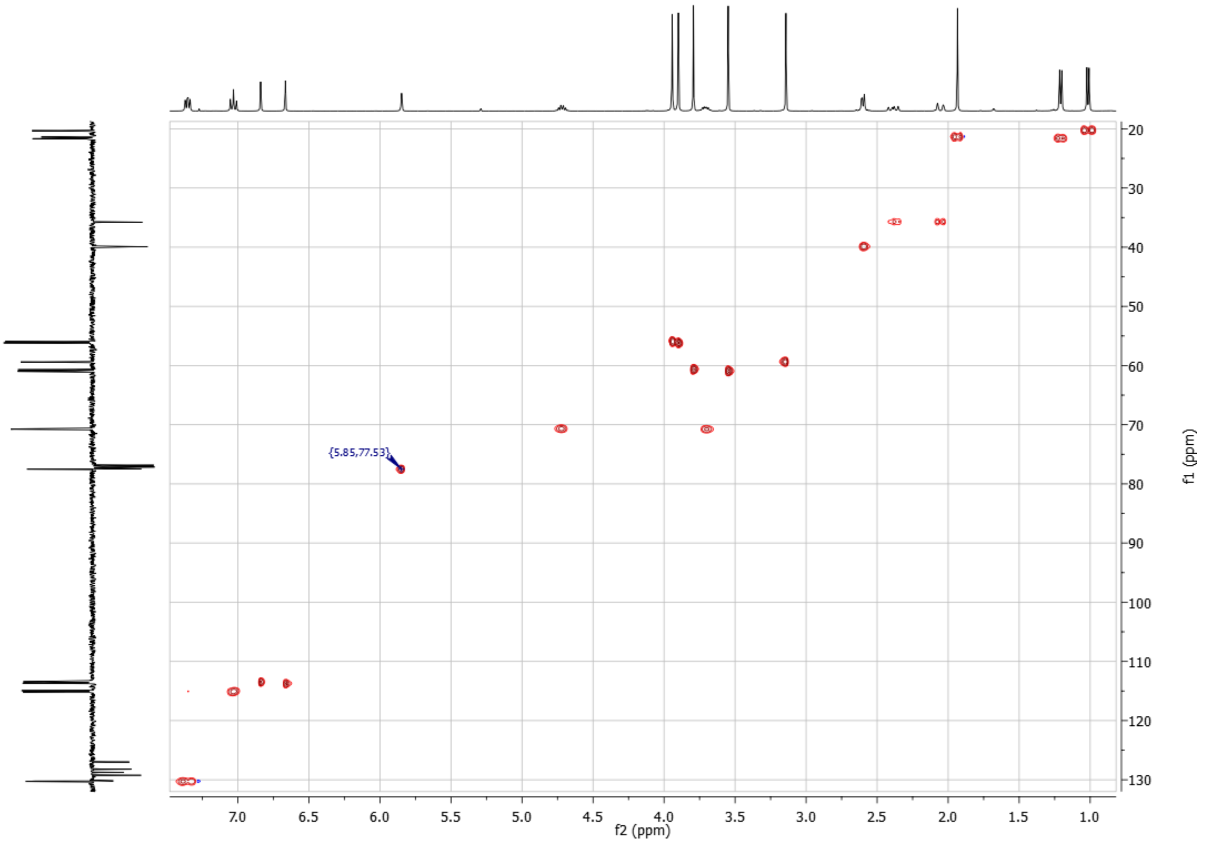

Figure S36. ^{1}H-^{13}C HSQC NMR (400 MHz) spectrum of (a*S*,1*R*,3*S*,2'*S*)-**19** in $CDCl_3$.

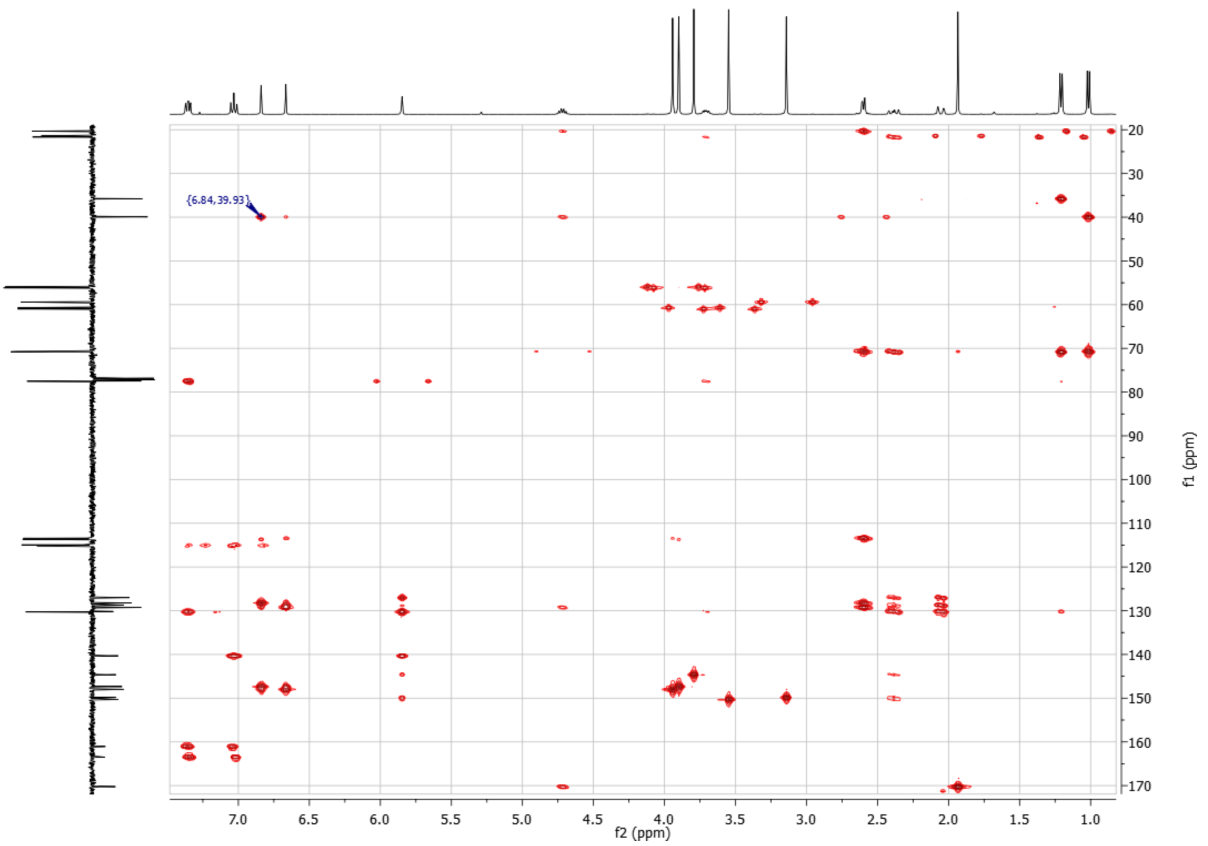

Figure S37. ^{1}H-^{13}C HMBC NMR (400 MHz) spectrum of (a*S*,1*R*,3*S*,2'*S*)-**19** in $CDCl_3$.

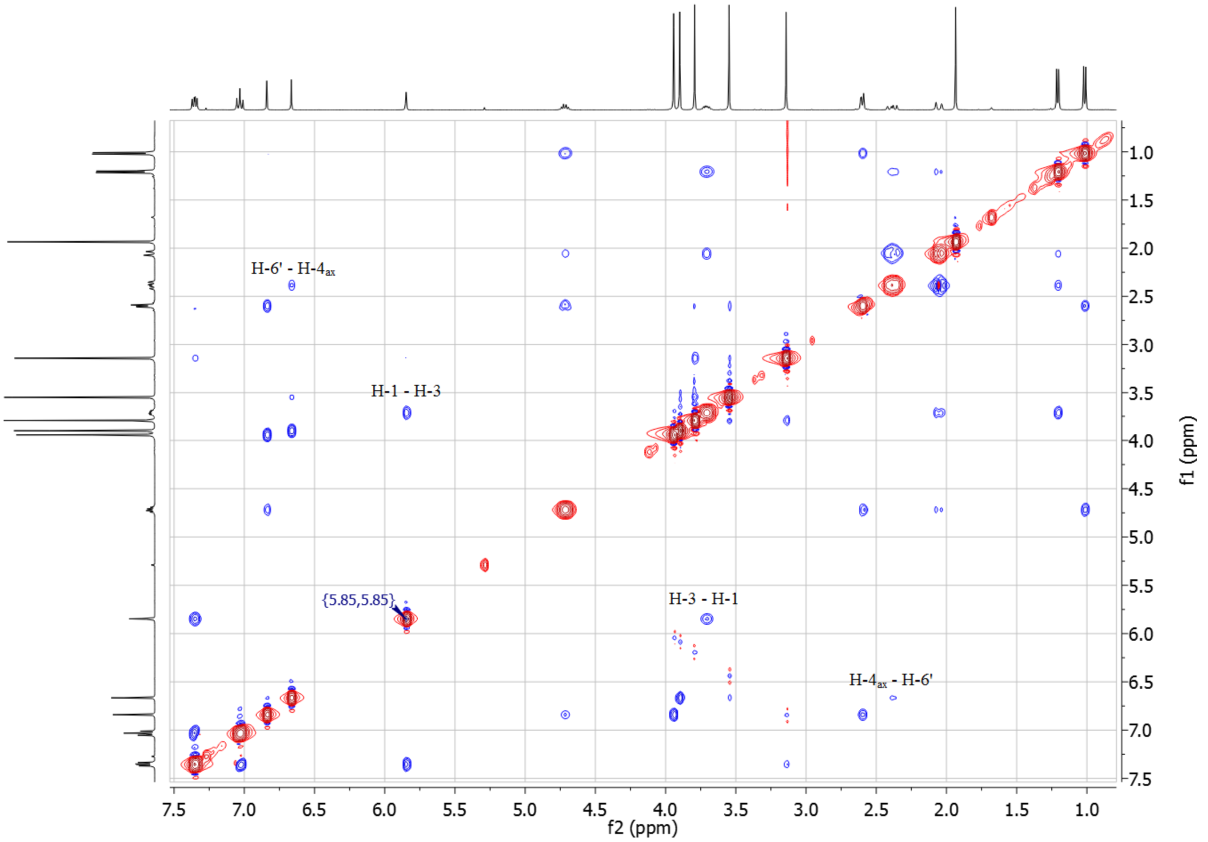

Figure S38. ^{1}H-^{1}H NOESY NMR (400 MHz) spectrum of (a*S*,1*R*,3*S*,2'*S*)-**19** in $CDCl_3$.

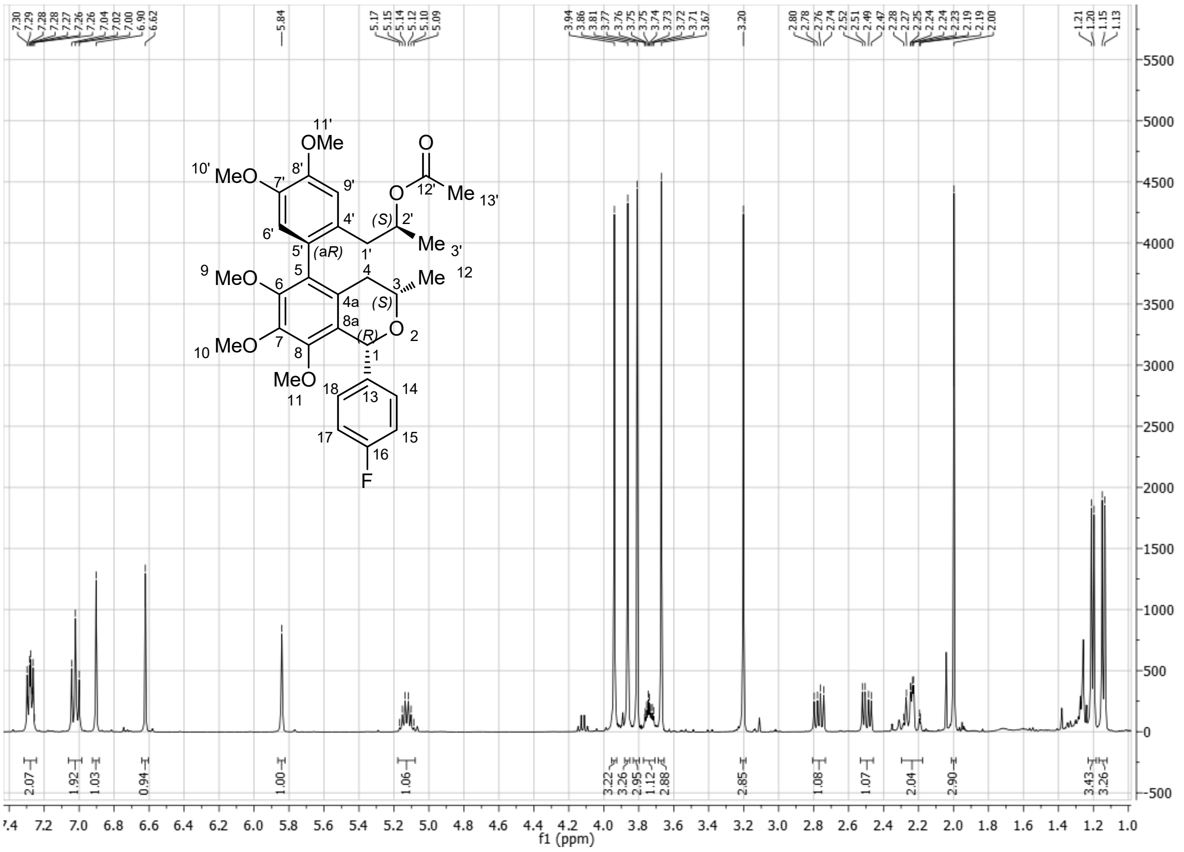

Figure S39. ^{1}H NMR (400 MHz) spectrum of (a*R*,1*R*,3*S*,2'*S*)-**19** in $CDCl_3$.

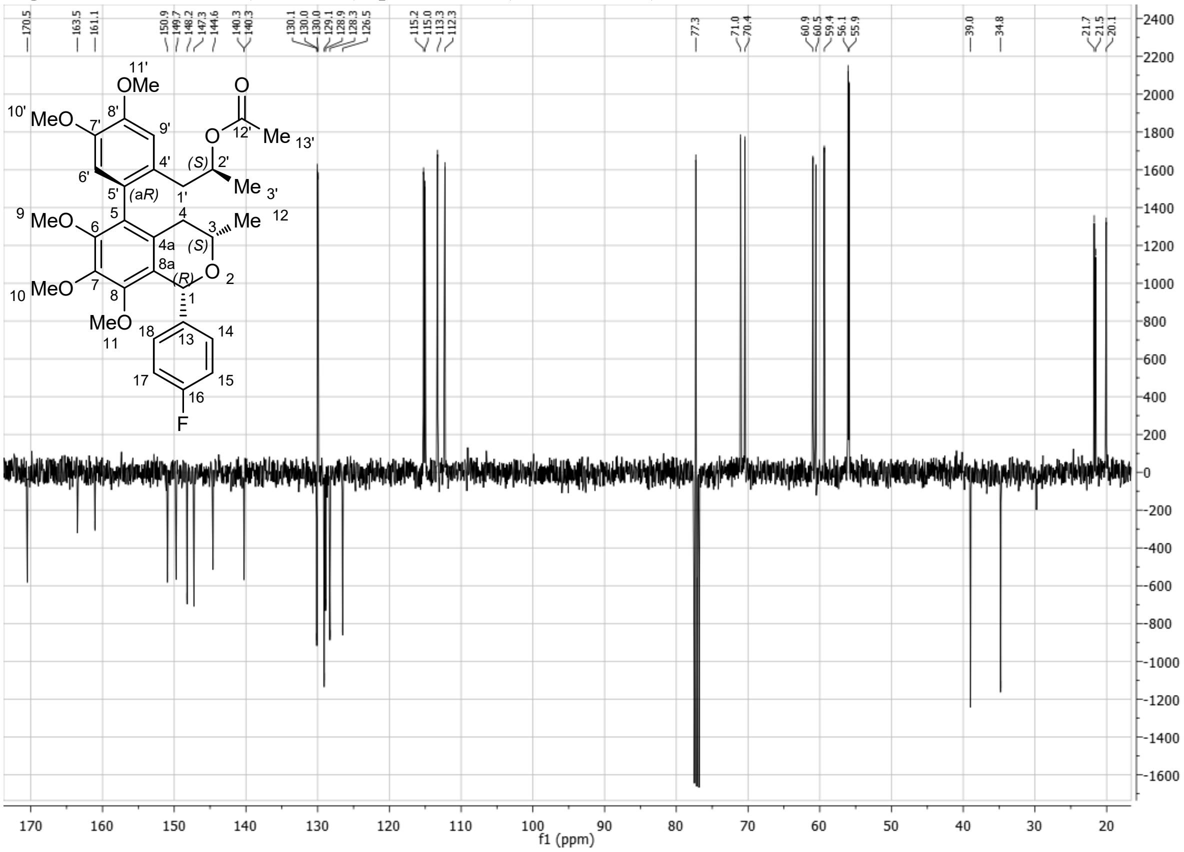

Figure S40. ^{13}C NMR (100 MHz) spectrum of (a*R*,1*R*,3*S*,2'*S*)-**19** in $CDCl_3$.

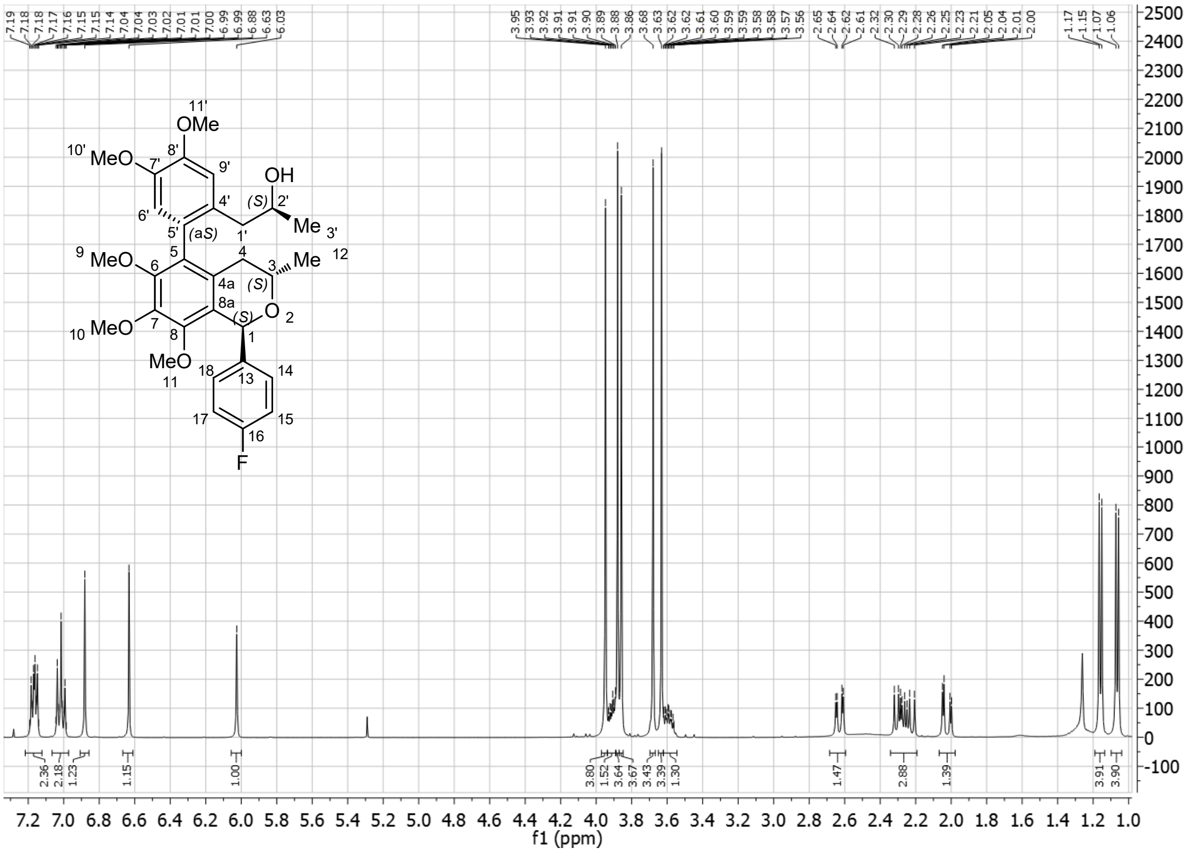

Figure S41. ^{1}H NMR (400 MHz) spectrum of (a*S*,1*S*,3*S*,2'*S*)-**20** in $CDCl_3$.

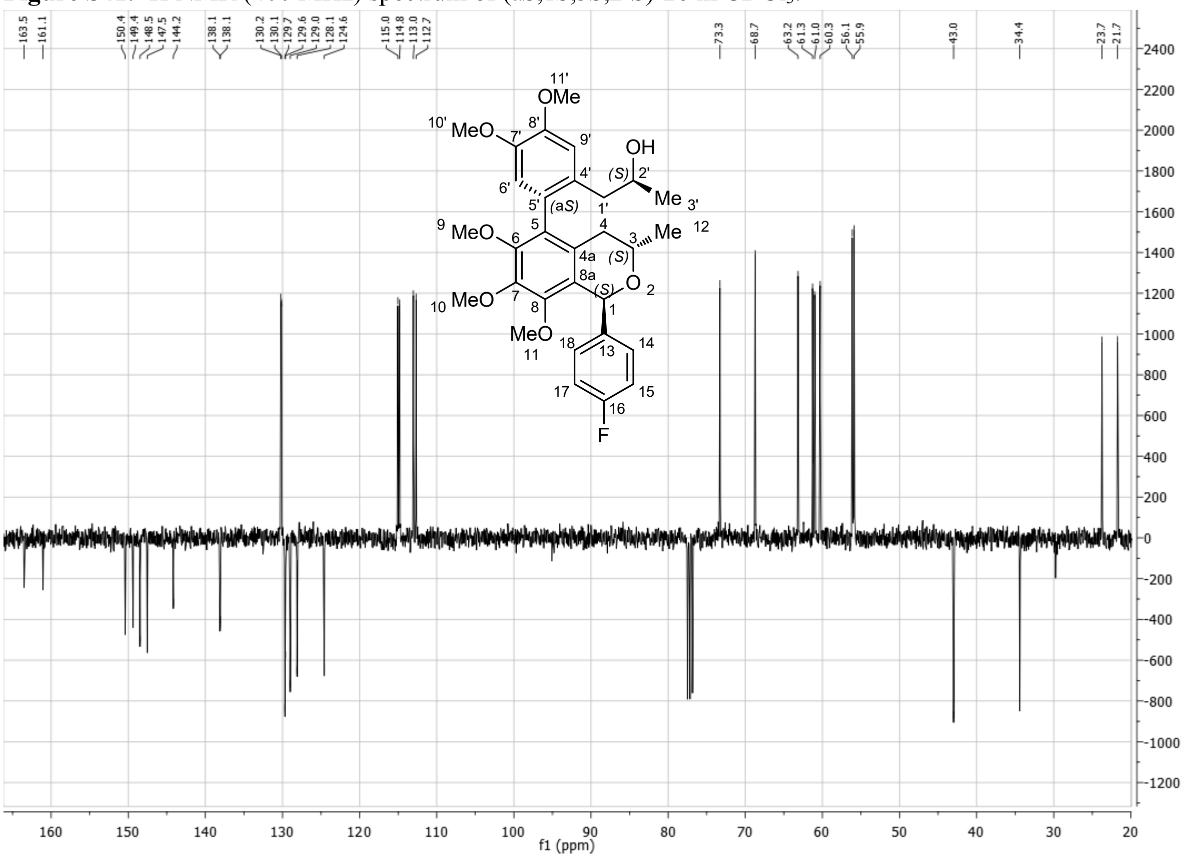

Figure S42. ^{13}C NMR (100 MHz) spectrum of (a*S*,1*S*,3*S*,2'*S*)-**20** in $CDCl_3$.

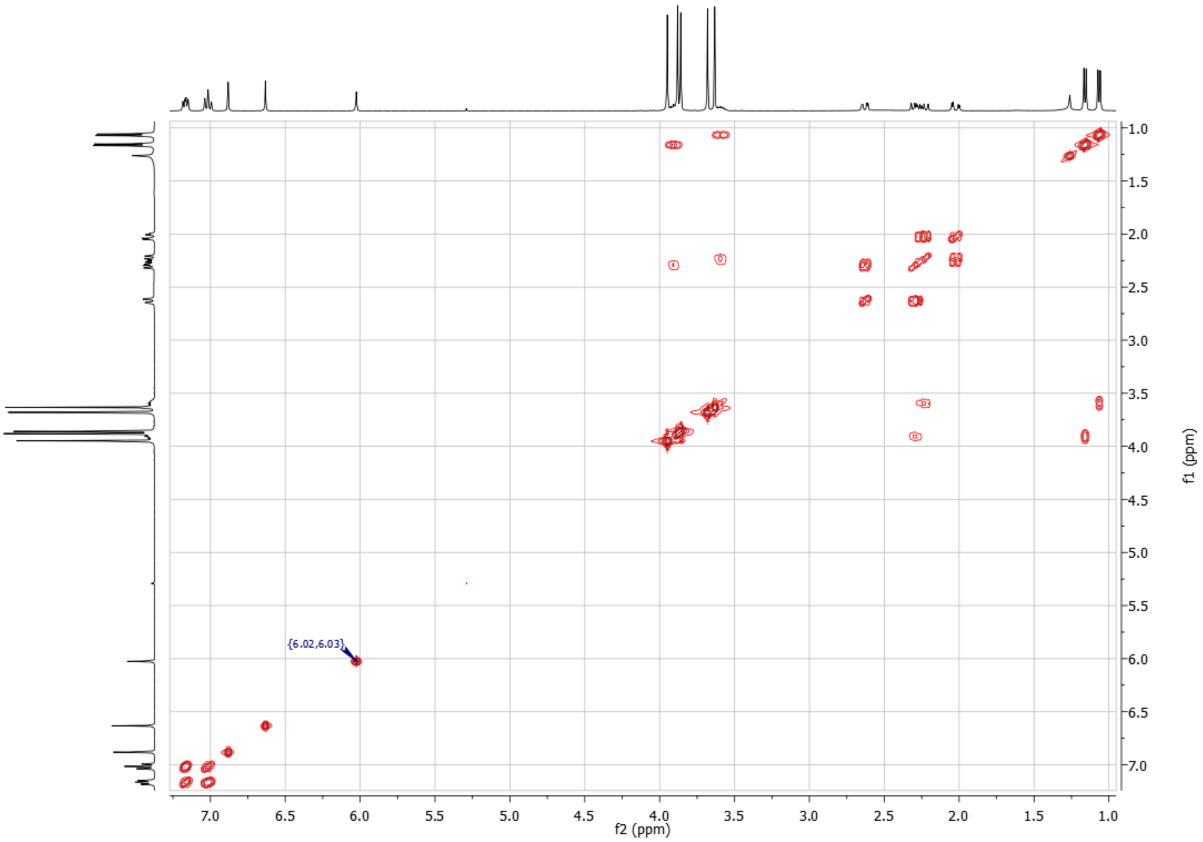

Figure S43. ^{1}H-^{1}H COSY NMR (400 MHz) spectrum of (a*S*,1*S*,3*S*,2'*S*)-**20** in $CDCl_3$.

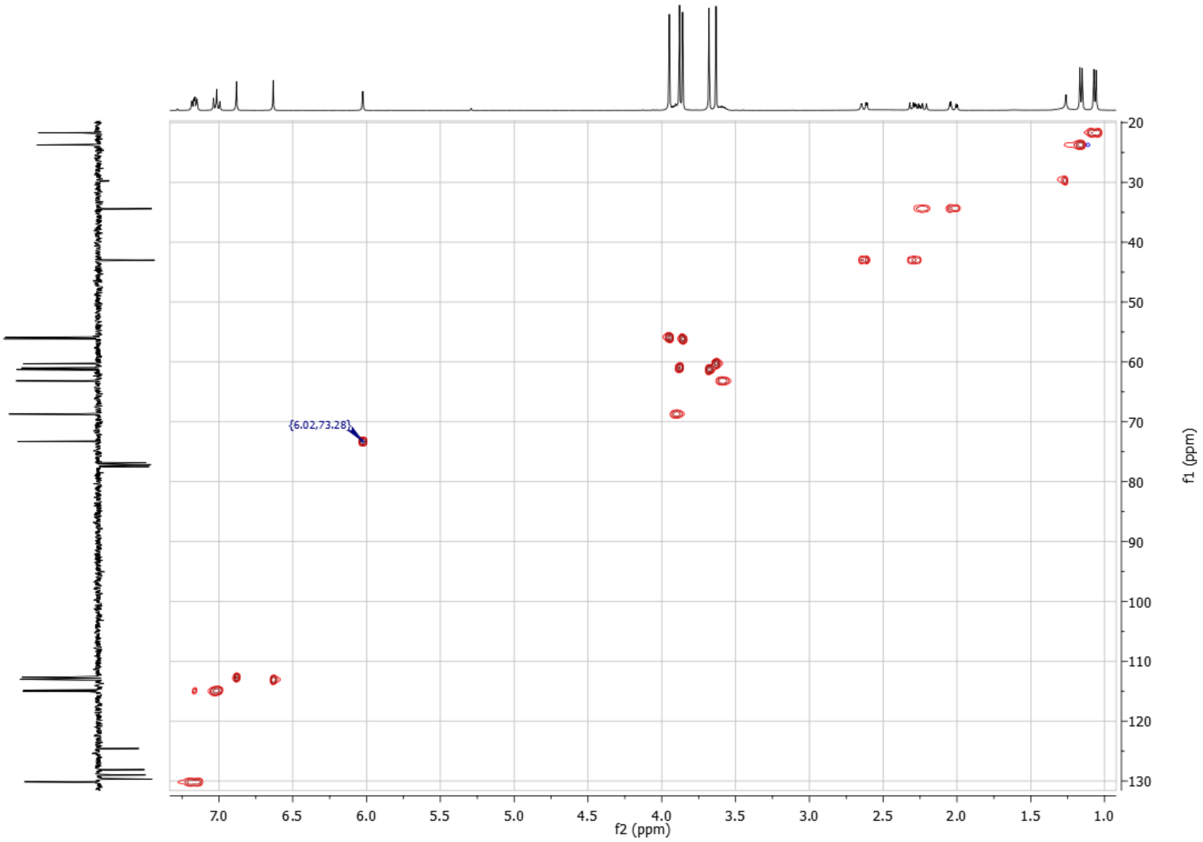

Figure S44. ^{1}H-^{13}C HSQC NMR (400 MHz) spectrum of (a*S*,1*S*,3*S*,2'*S*)-**20** in $CDCl_3$.

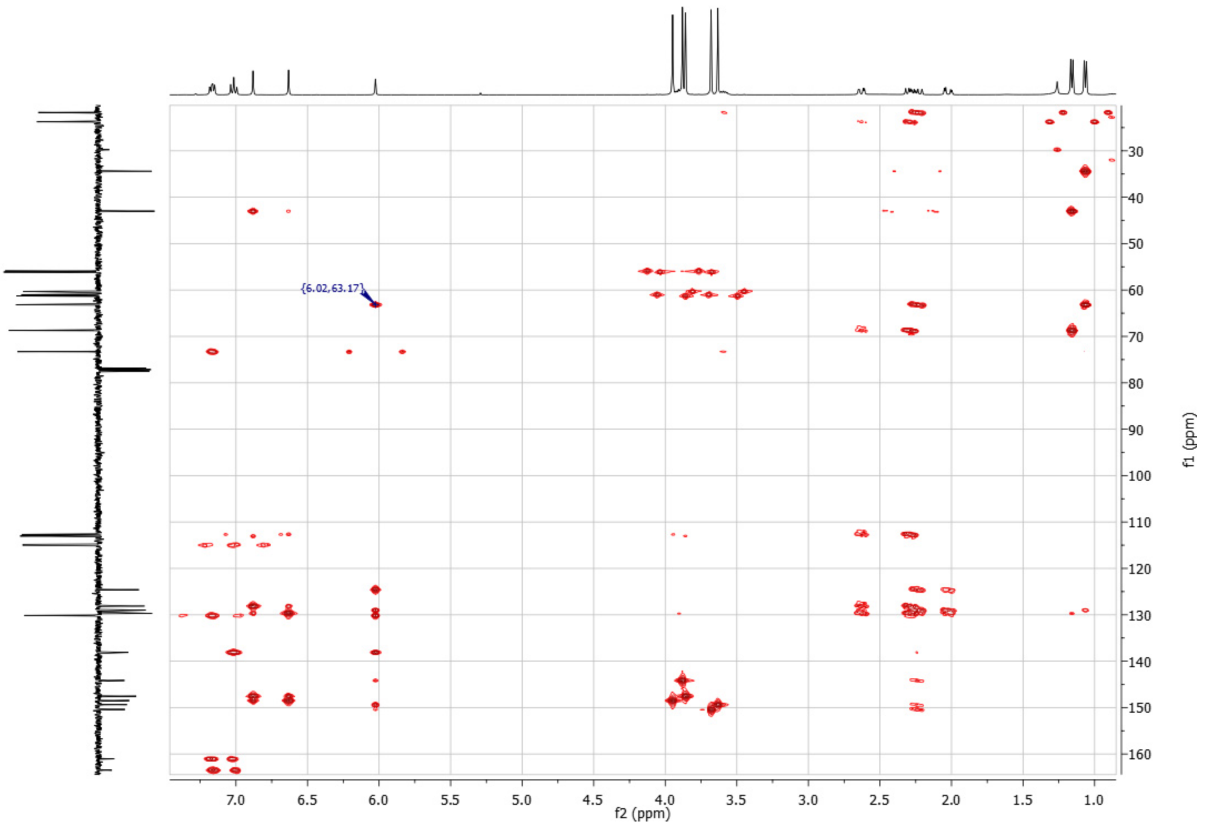

Figure S45. ^{1}H-^{13}C HMBC NMR (400 MHz) spectrum of (a*S*,1*S*,3*S*,2'*S*)-**20** in $CDCl_3$.

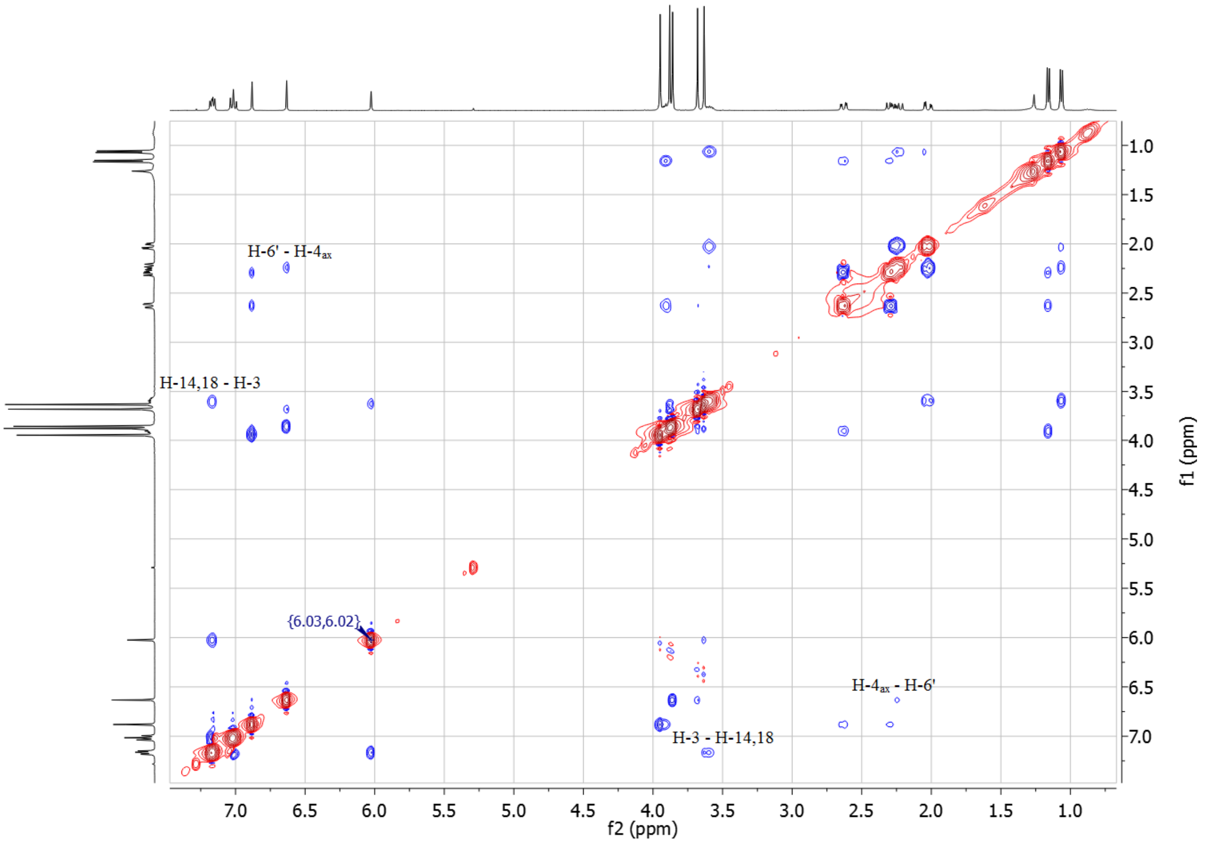

Figure S46. ^{1}H-^{1}H NOESY NMR (400 MHz) spectrum of (a*S*,1*S*,3*S*,2'*S*)-**20** in $CDCl_3$.

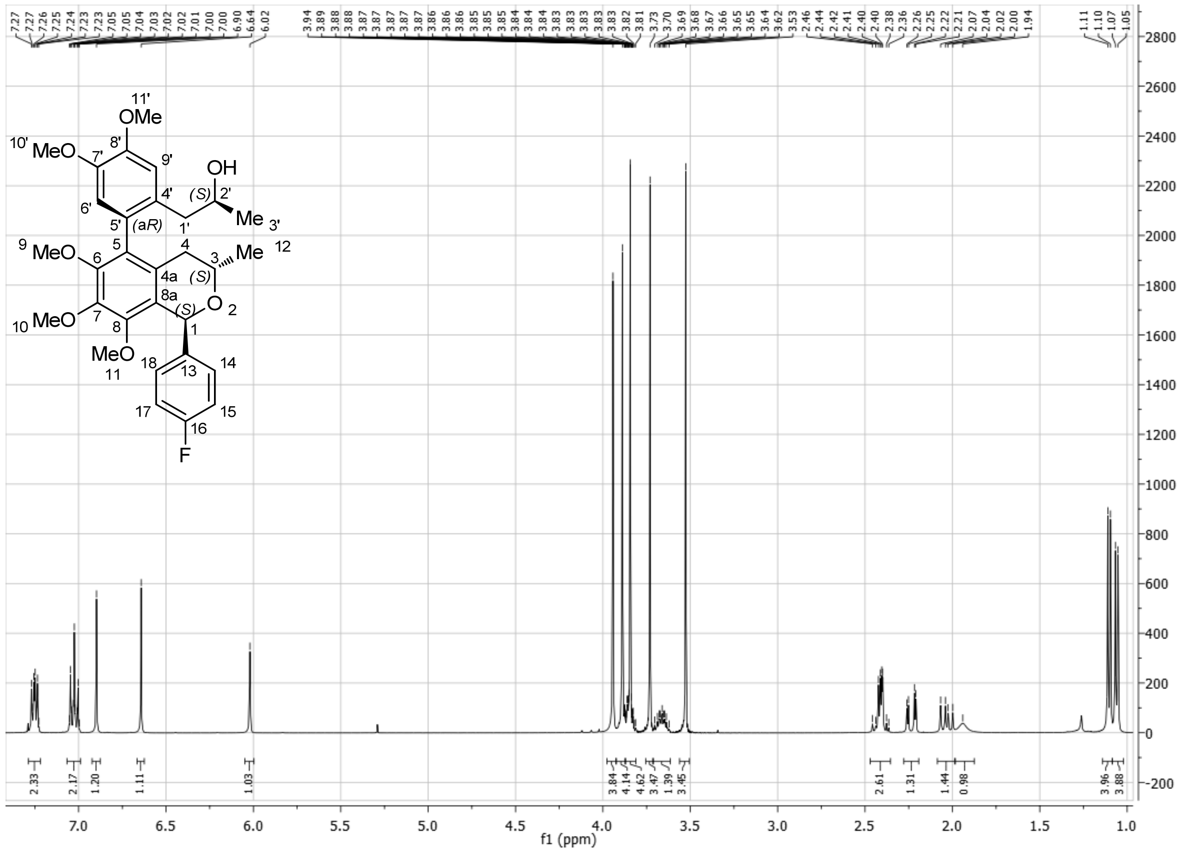

Figure S47. ^{1}H NMR (400 MHz) spectrum of (a*R*,1*S*,3*S*,2'*S*)-**20** in $CDCl_3$.

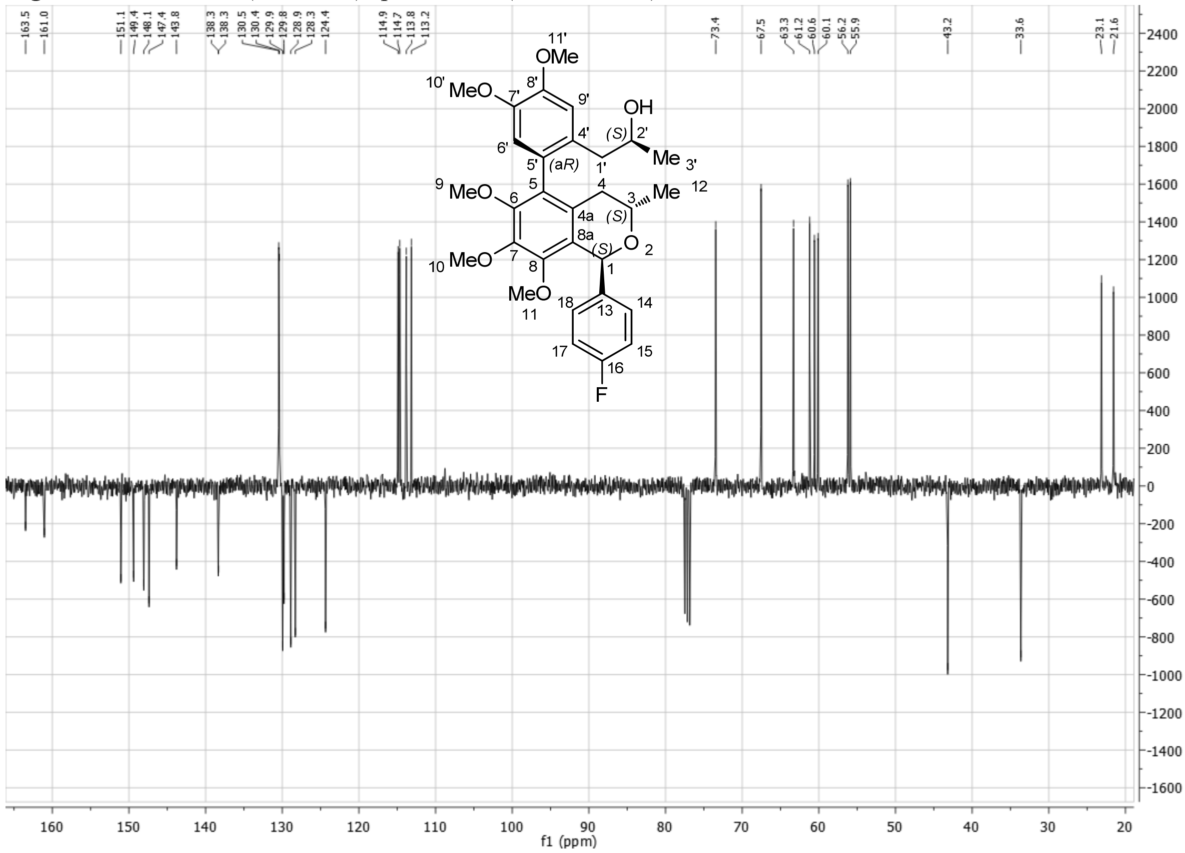

Figure S48. ^{13}C NMR (100 MHz) spectrum of (a*R*,1*S*,3*S*,2'*S*)-**20** in $CDCl_3$.

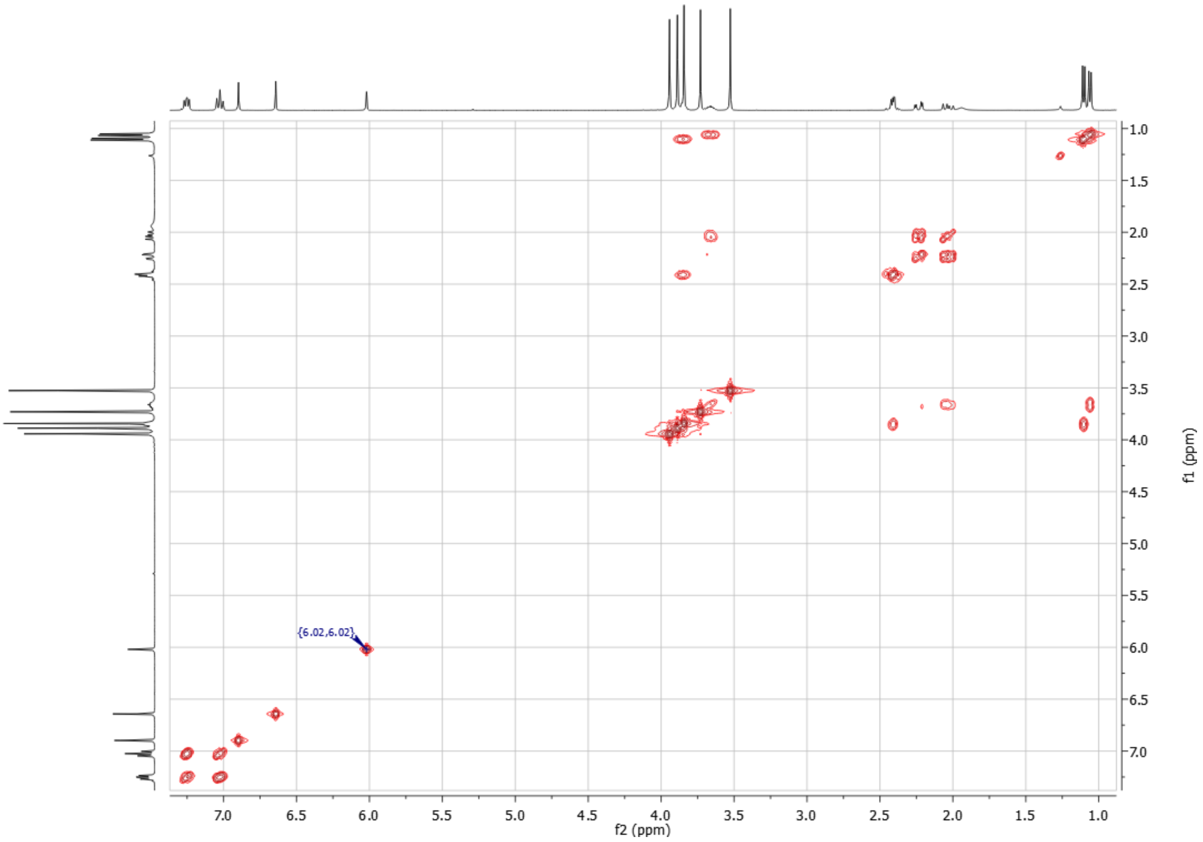

Figure S49. ^{1}H-^{1}H COSY NMR (400 MHz) spectrum of (a*R*,1*S*,3*S*,2'*S*)-**20** in $CDCl_3$.

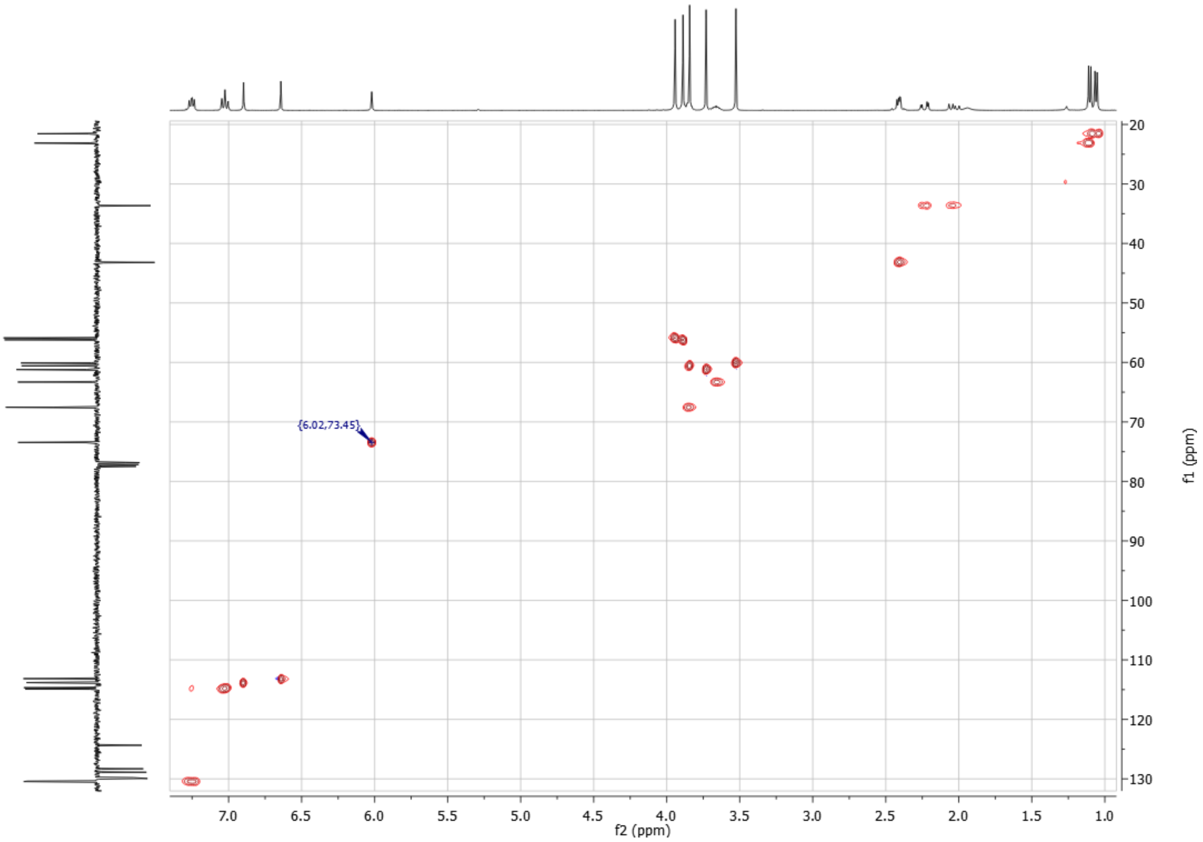

Figure S50. ^{1}H-^{13}C HSQC NMR (400 MHz) spectrum of (a*R*,1*S*,3*S*,2'*S*)-**20** in $CDCl_3$.

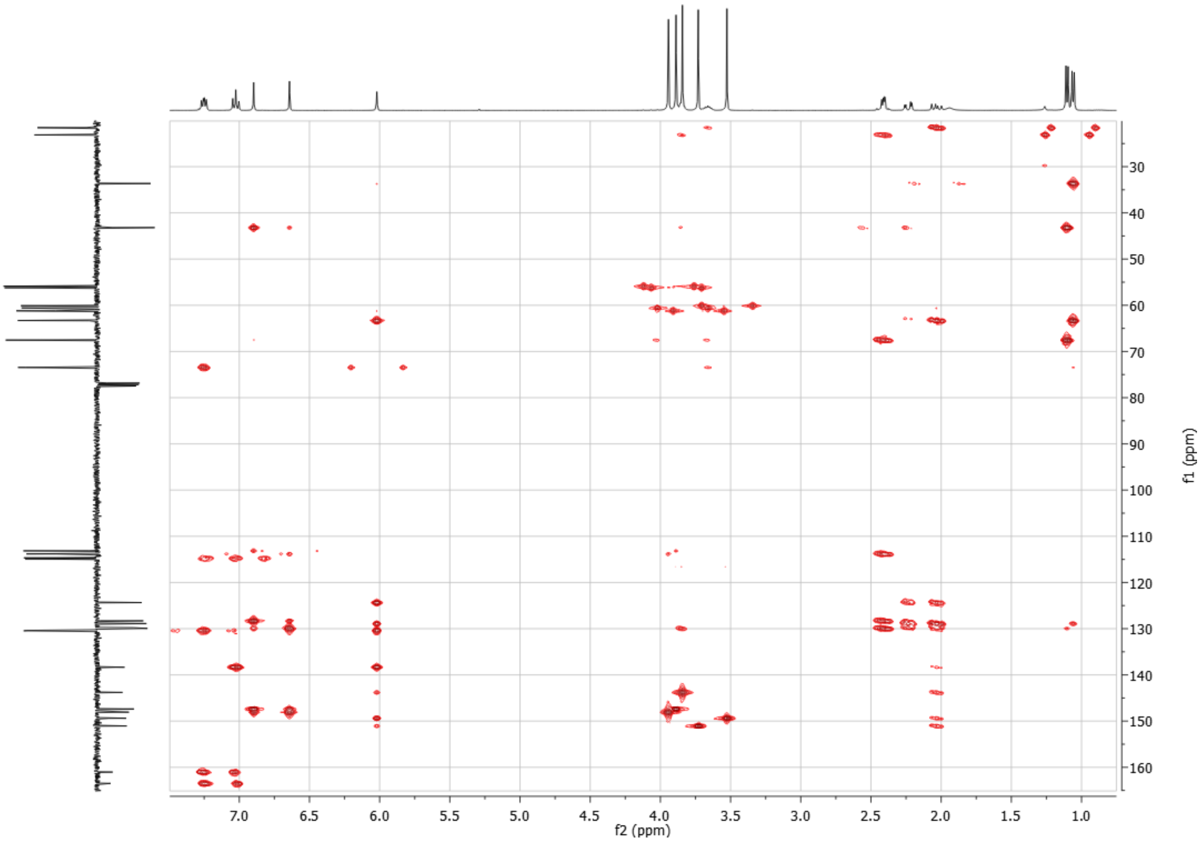

Figure S51. ^{1}H-^{13}C HMBC NMR (400 MHz) spectrum of (a*R*,1*S*,3*S*,2'*S*)-**20** in $CDCl_3$.

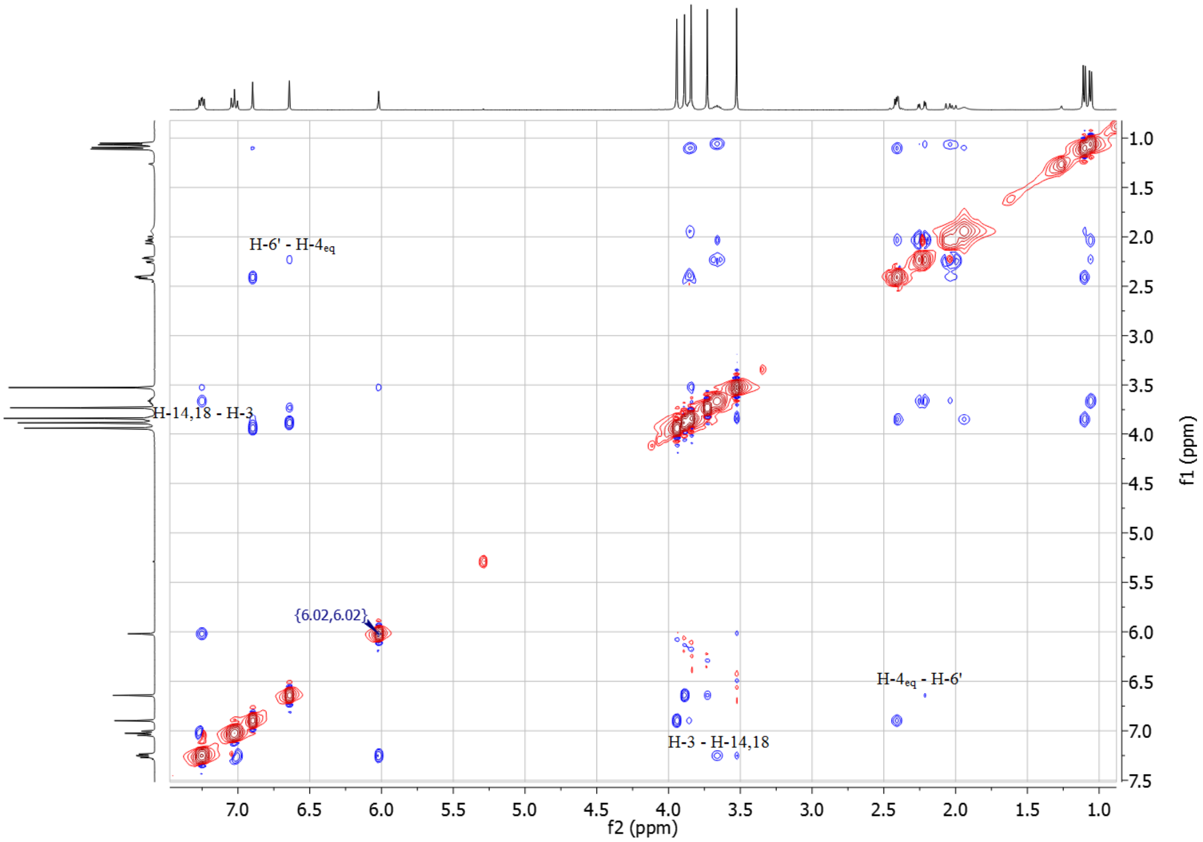

Figure S52. ^{1}H-^{1}H NOESY NMR (400 MHz) spectrum of (a*R*,1*S*,3*S*,2'*S*)-**20** in $CDCl_3$.

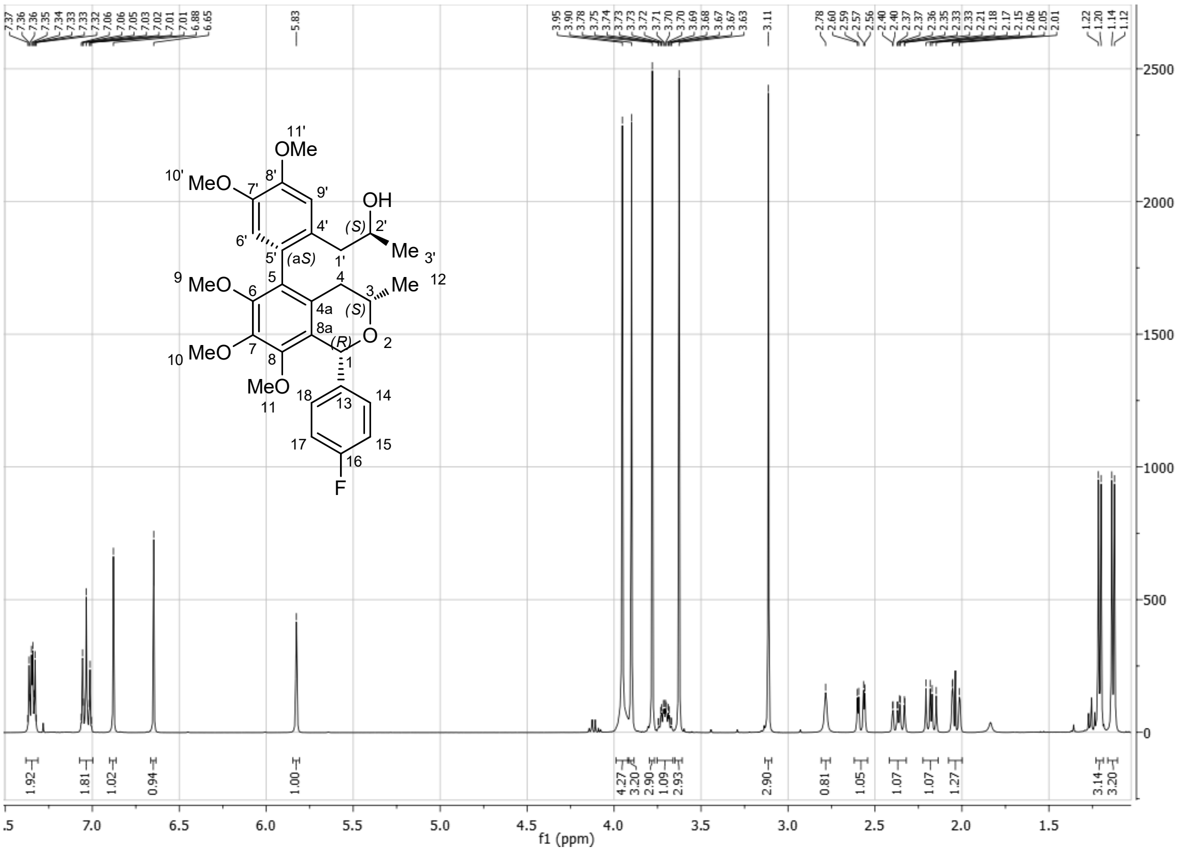

Figure S53. ^{1}H NMR (400 MHz) spectrum of (a*S*,1*R*,3*S*,2'*S*)-**20** in $CDCl_3$.

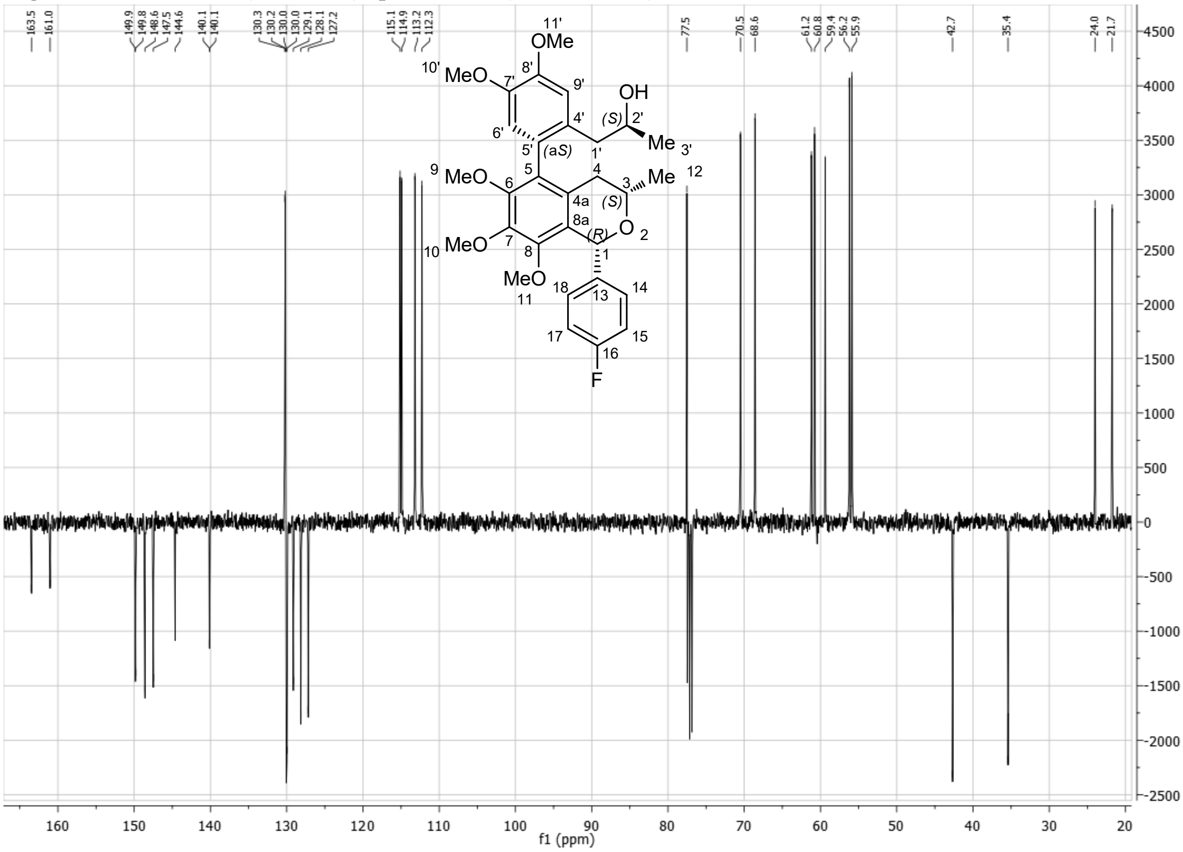

Figure S54. ^{13}C NMR (100 MHz) spectrum of (a*S*,1*R*,3*S*,2'*S*)-**20** in $CDCl_3$.

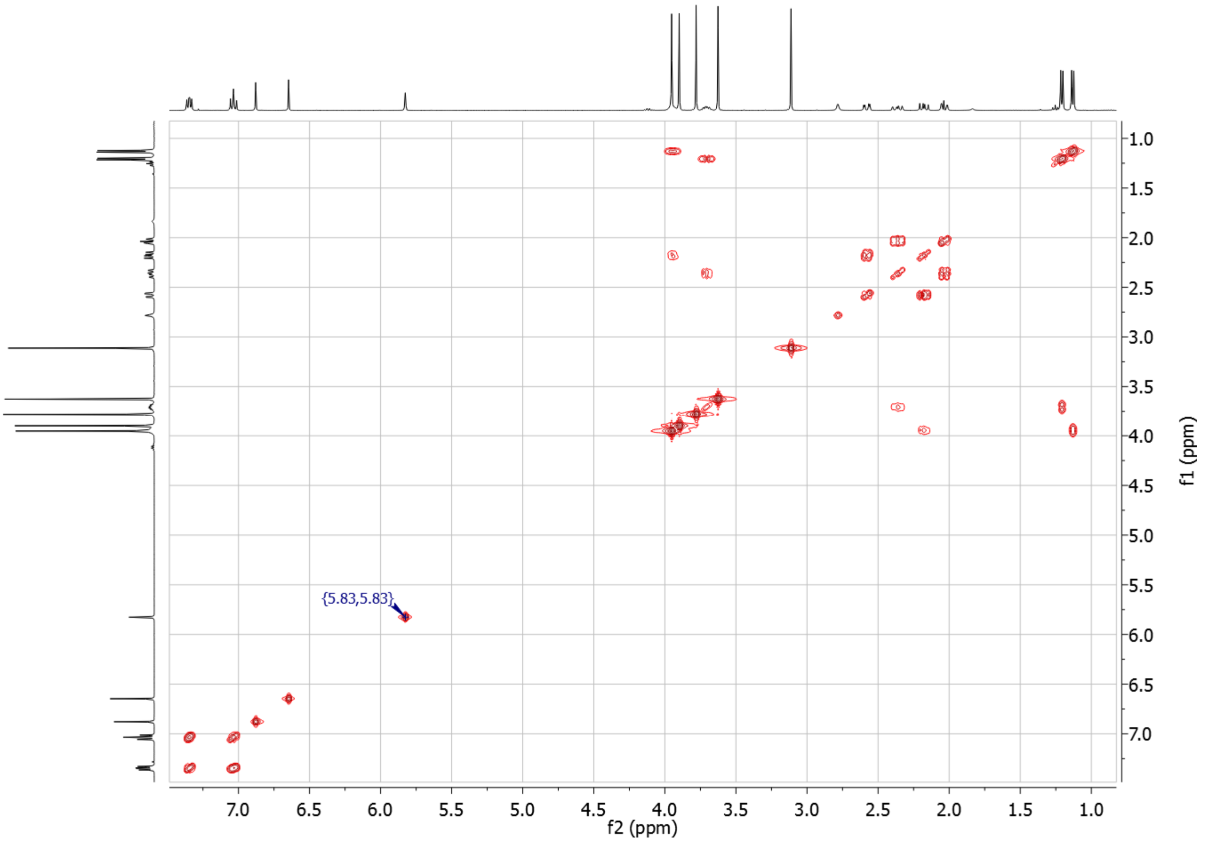

Figure S55. ^{1}H-^{1}H COSY NMR (400 MHz) spectrum of (a*S*,1*R*,3*S*,2’*S*)-**20** in $CDCl_3$.

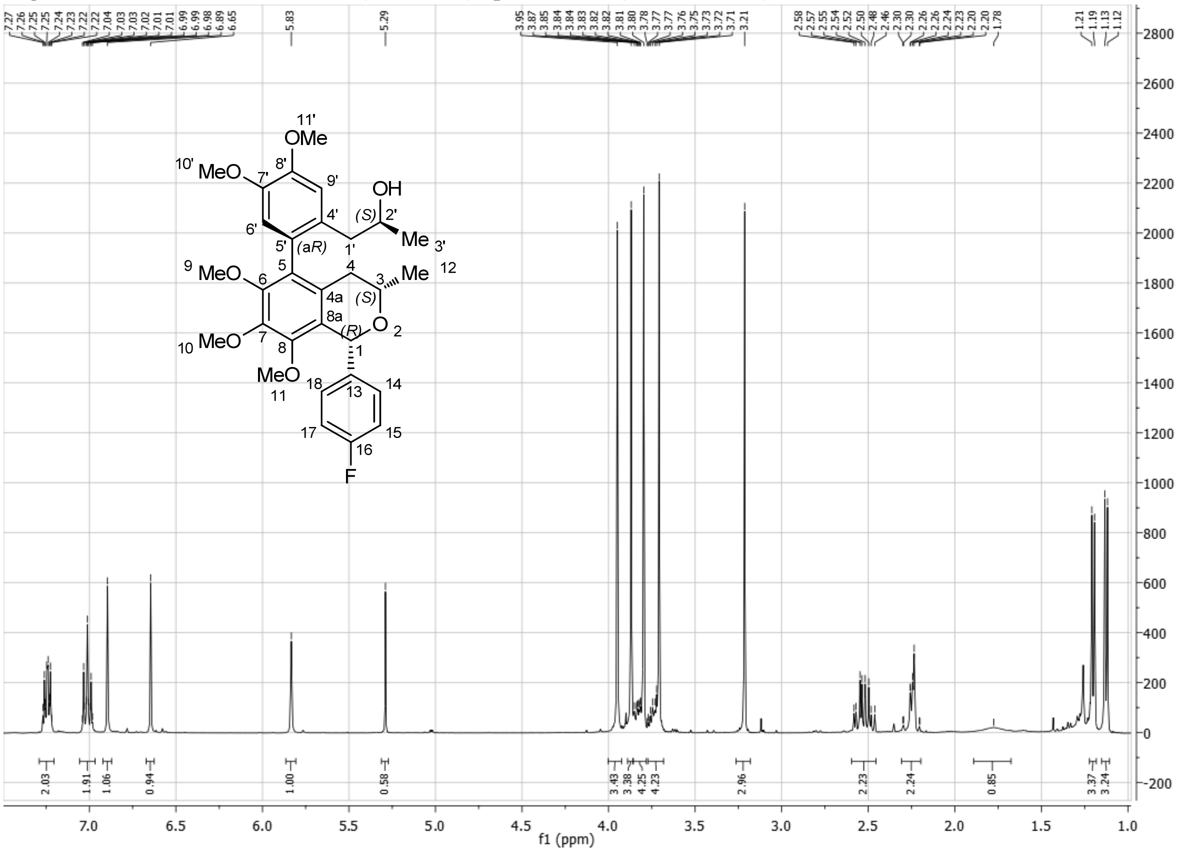

Figure S56. ^{1}H NMR (400 MHz) spectrum of (a*R*,1*R*,3*S*,2’*S*)-**20** in $CDCl_3$.

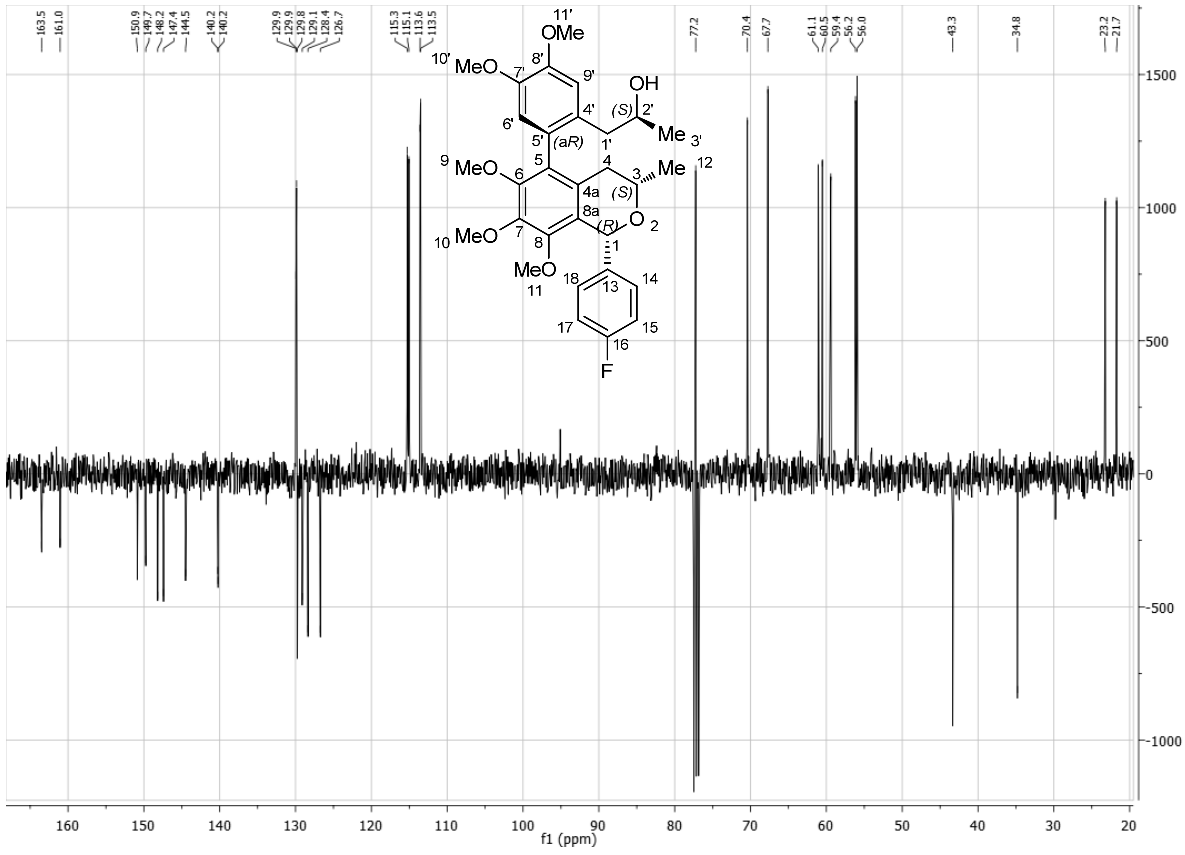

Figure S57. ^{13}C NMR (100 MHz) spectrum of (a*R*,1*R*,3*S*,2'*S*)-**20** in $CDCl_3$.

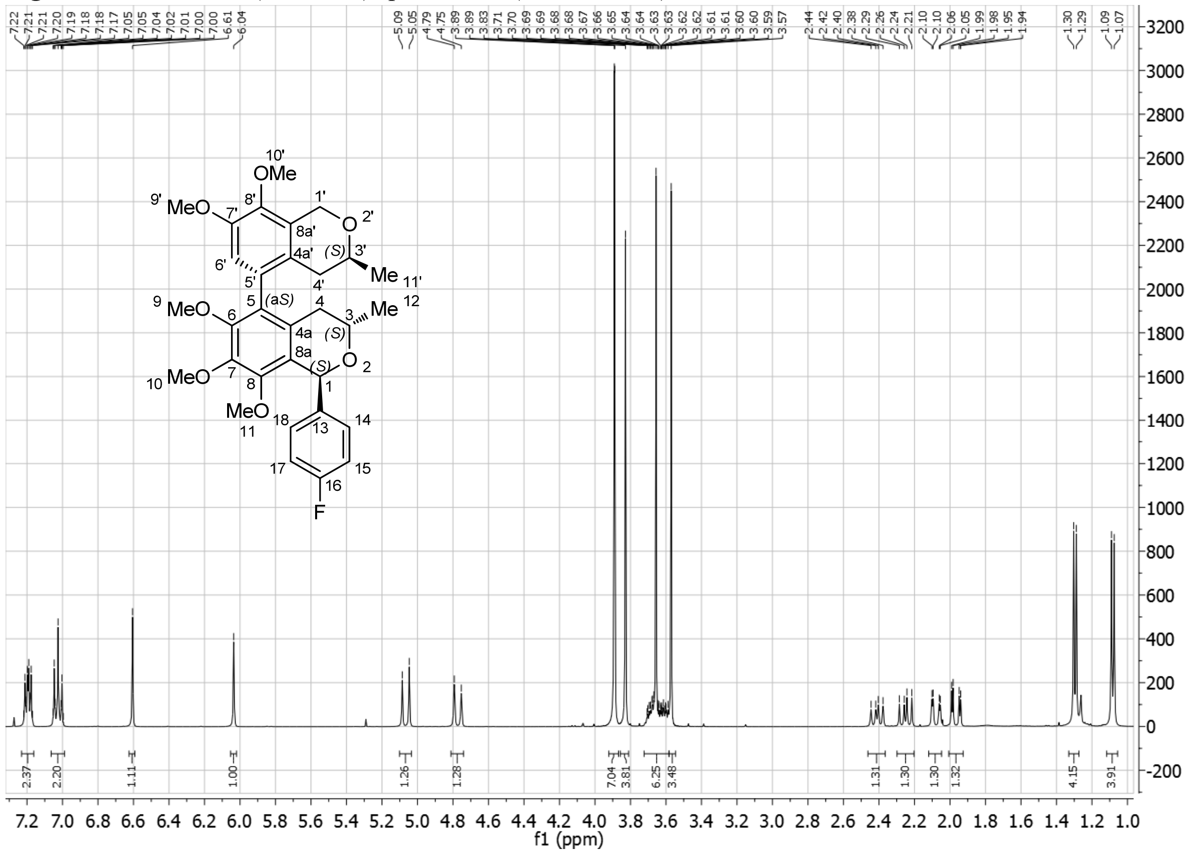

Figure S58. ^{1}H NMR (400 MHz) spectrum of (a*S*,1*S*,3*S*,3'*S*)-**21** in $CDCl_3$.

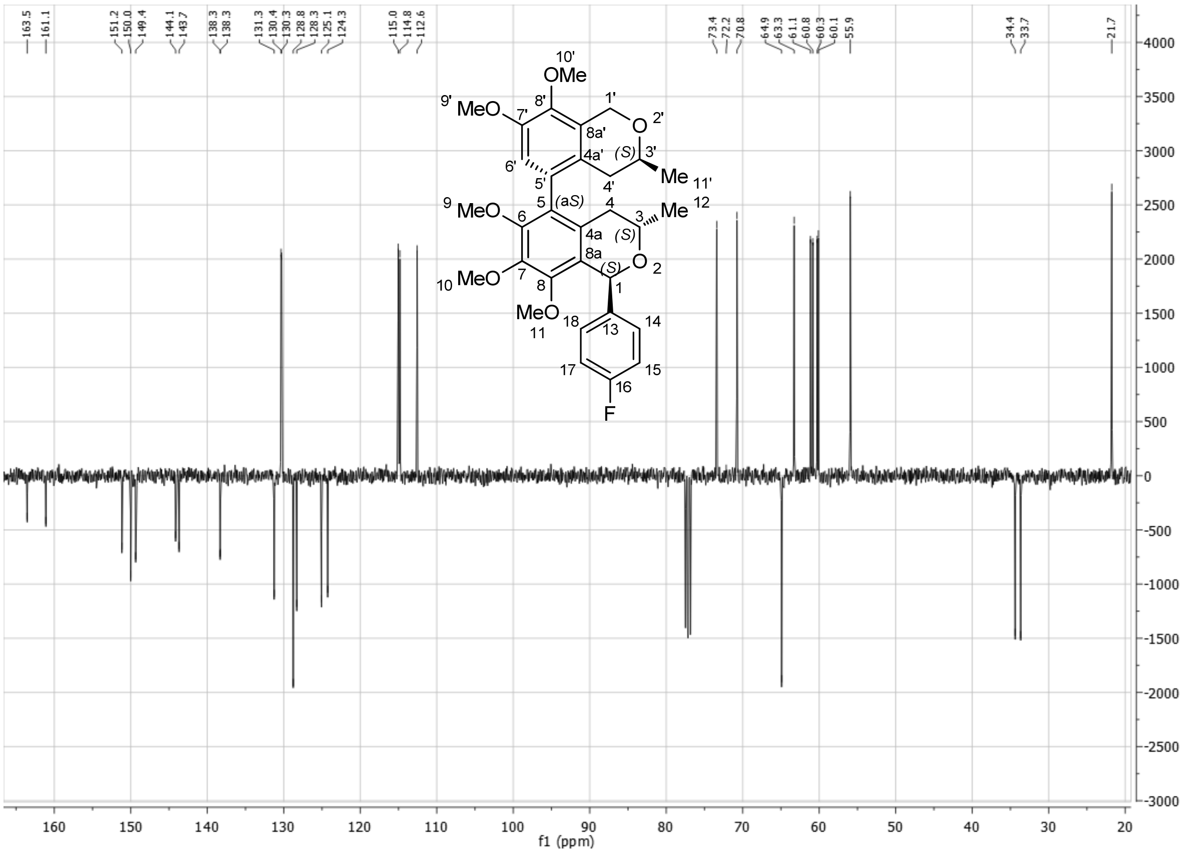

Figure S59. ^{13}C NMR (100 MHz) spectrum of (a*S*,1*S*,3*S*,3'*S*)-**21** in $CDCl_3$.

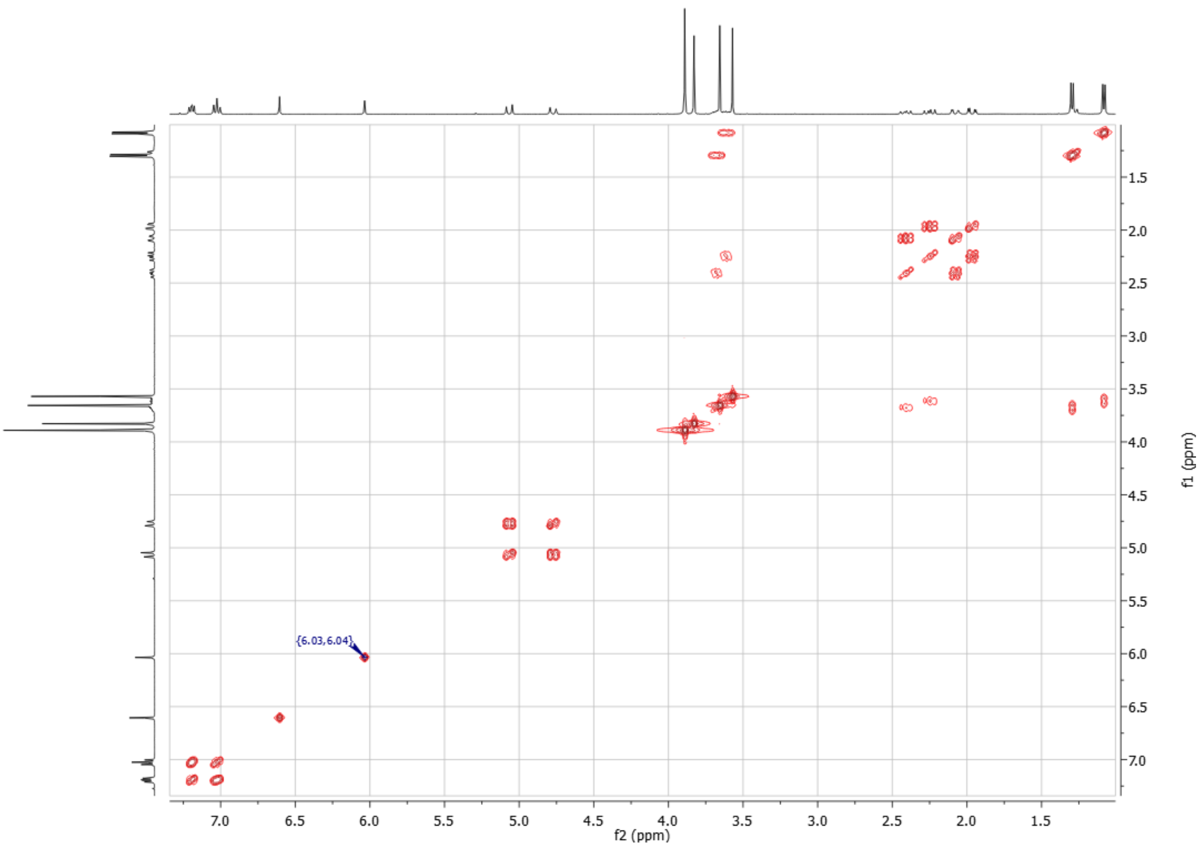

Figure S60. ^{1}H-^{1}H COSY NMR (400 MHz) spectrum of (a*S*,1*S*,3*S*,3'*S*)-**21** in $CDCl_3$.

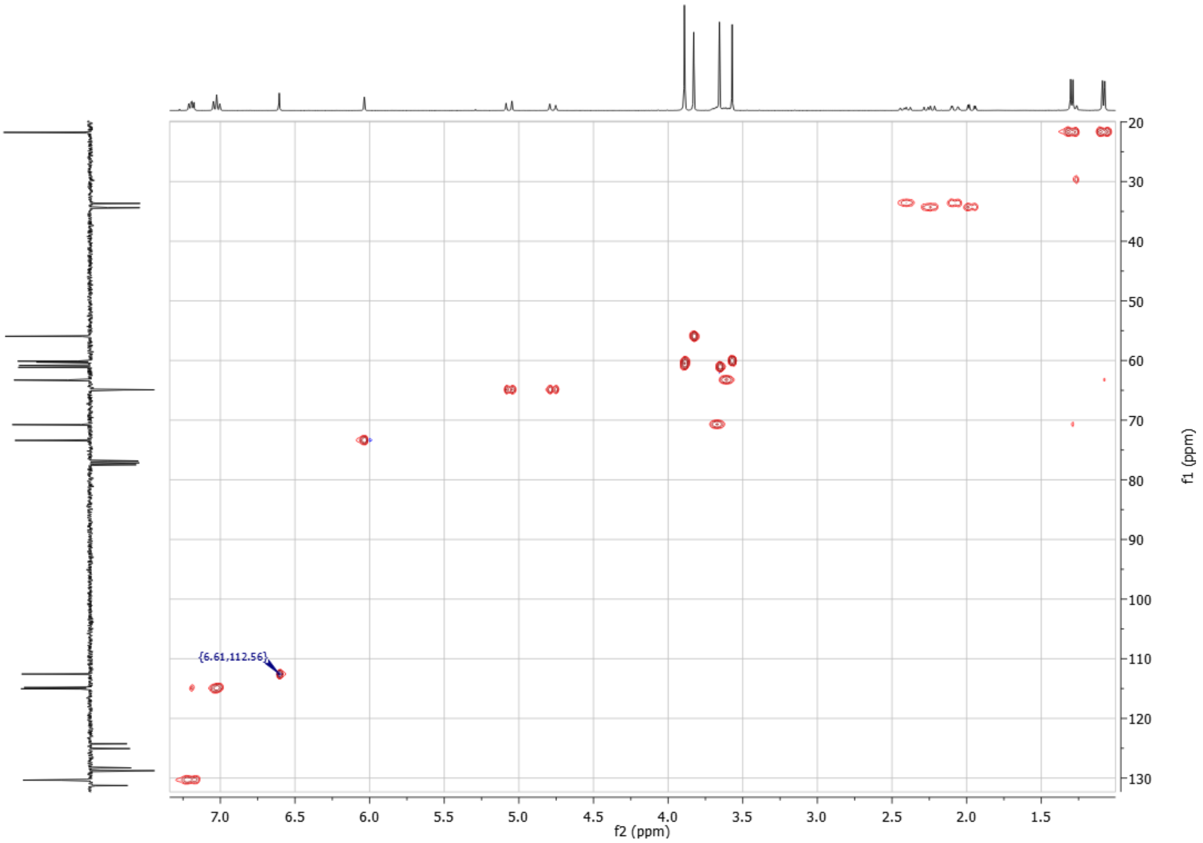

Figure S61. ^{1}H-^{13}C HSQC NMR (400 MHz) spectrum of (aS,1S,3S,3'S)-**21** in $CDCl_3$.

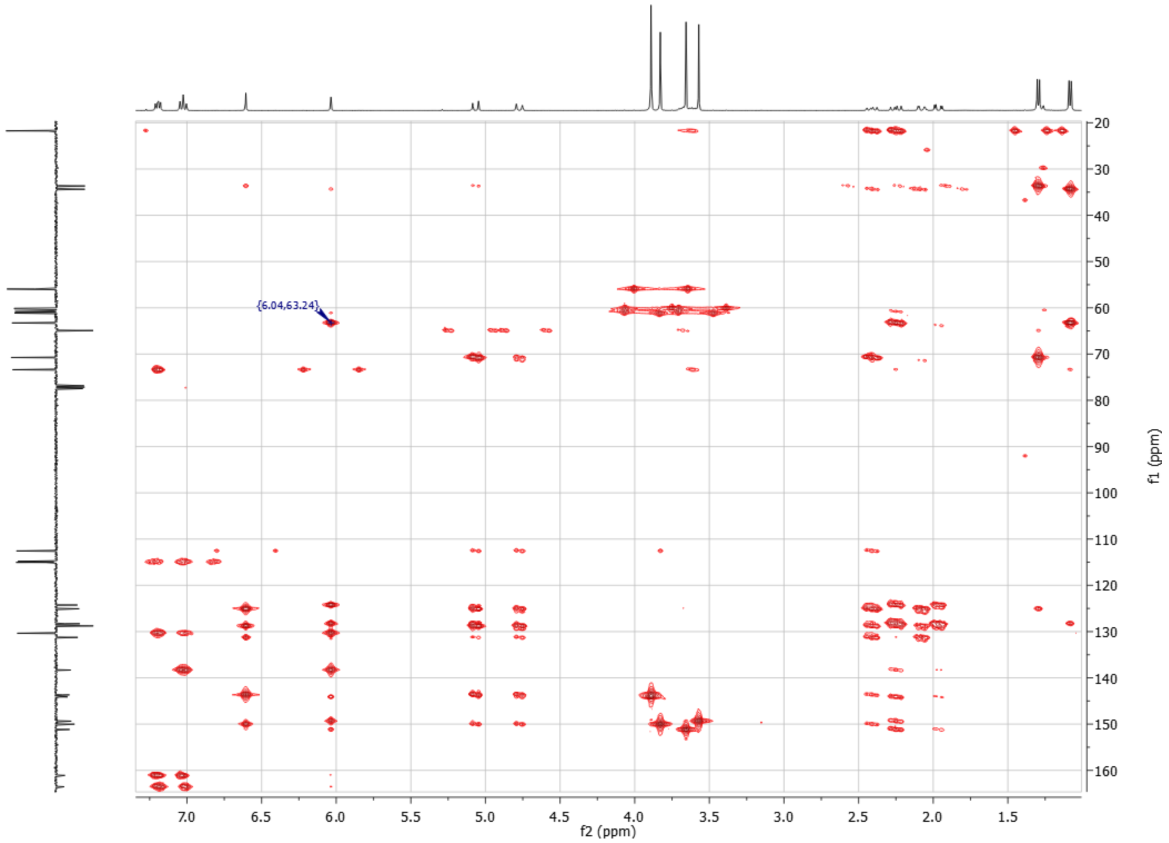

Figure S62. ^{1}H-^{13}C HMBC NMR (400 MHz) spectrum of (aS,1S,3S,3'S)-**21** in $CDCl_3$.

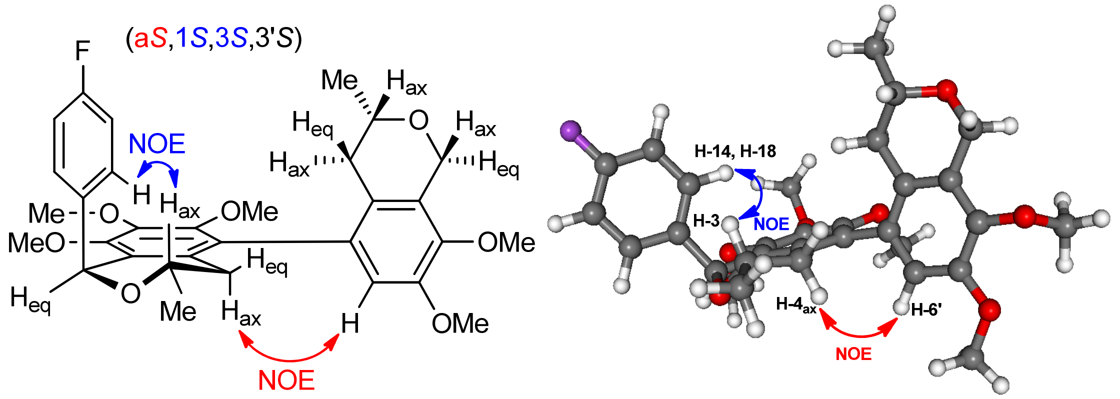

Figure S63. Characteristic NOE correlations shown on the lowest-energy computed conformer of (a*S*,1*S*,3*S*,3'*S*)-**21** suggesting (1*S*,3*S*) configuration of isochroman subunit and (a*S*) axial chirality.

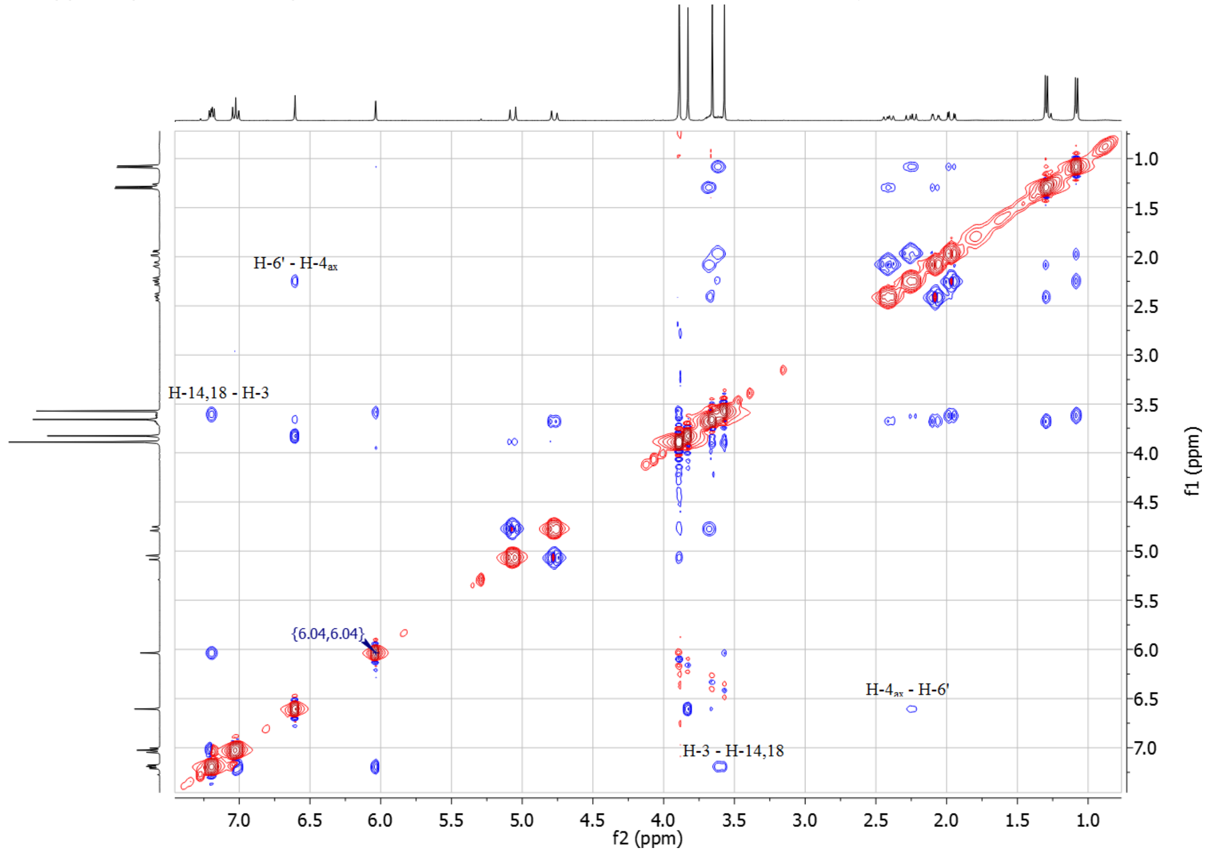

Figure S64. ^{1}H-^{1}H NOESY NMR (400 MHz) spectrum of (a*S*,1*S*,3*S*,3'*S*)-**21** in CDCl$_3$.

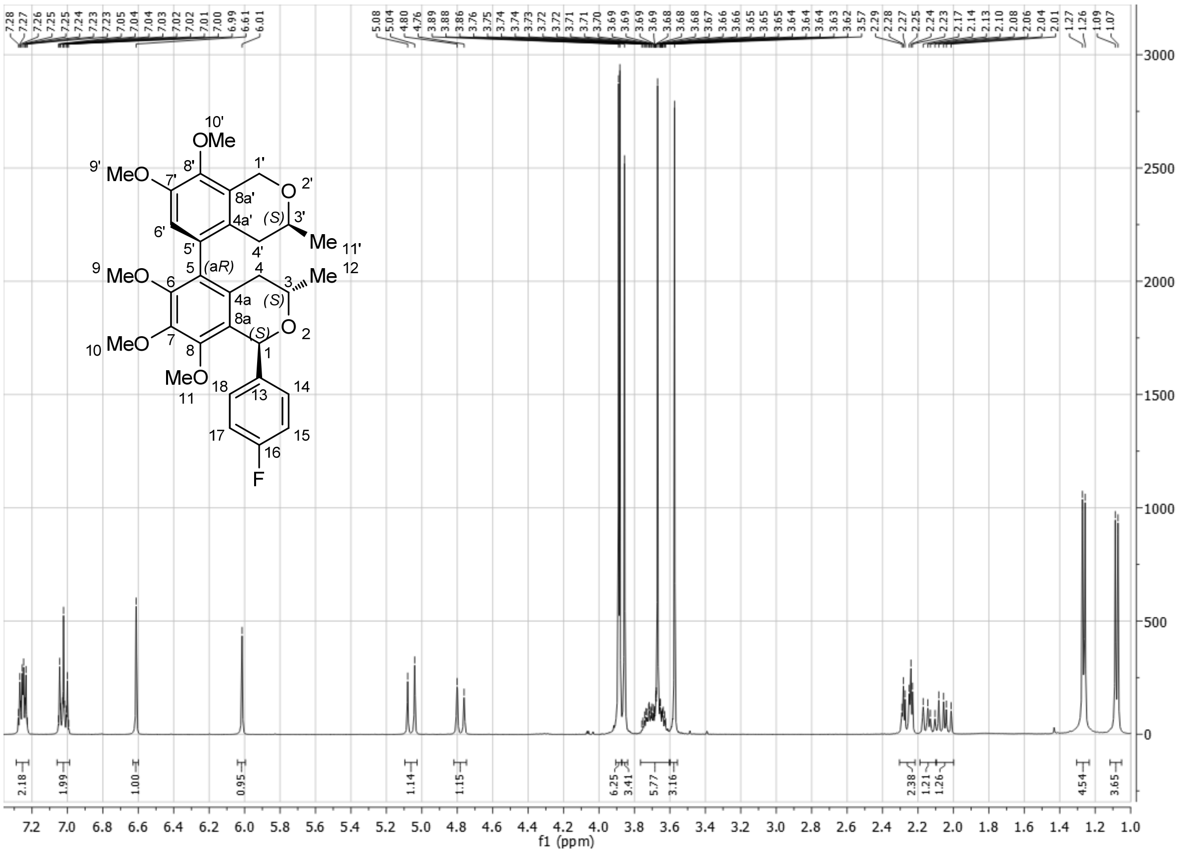

Figure S65. ^{1}H NMR (400 MHz) spectrum of (a*R*,1*S*,3*S*,3'*S*)-**21** in $CDCl_3$.

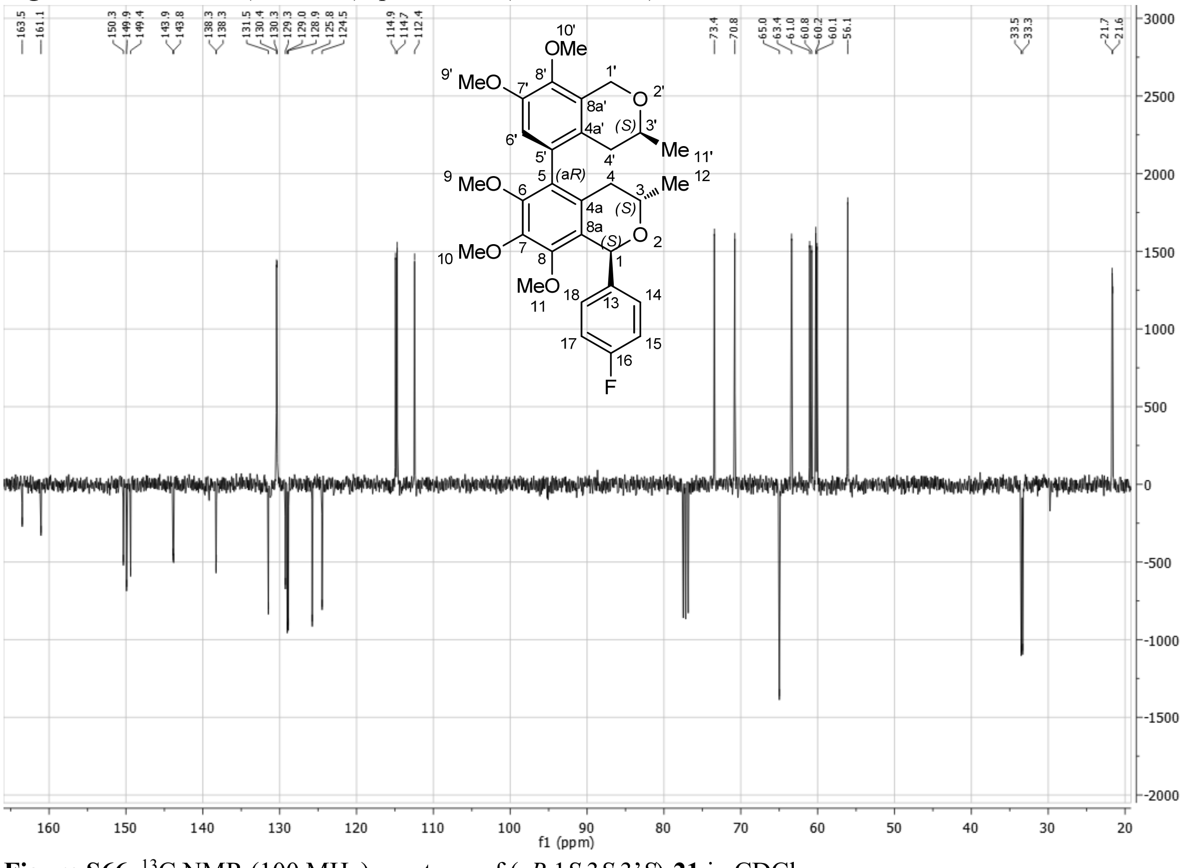

Figure S66. ^{13}C NMR (100 MHz) spectrum of (a*R*,1*S*,3*S*,3'*S*)-**21** in $CDCl_3$.

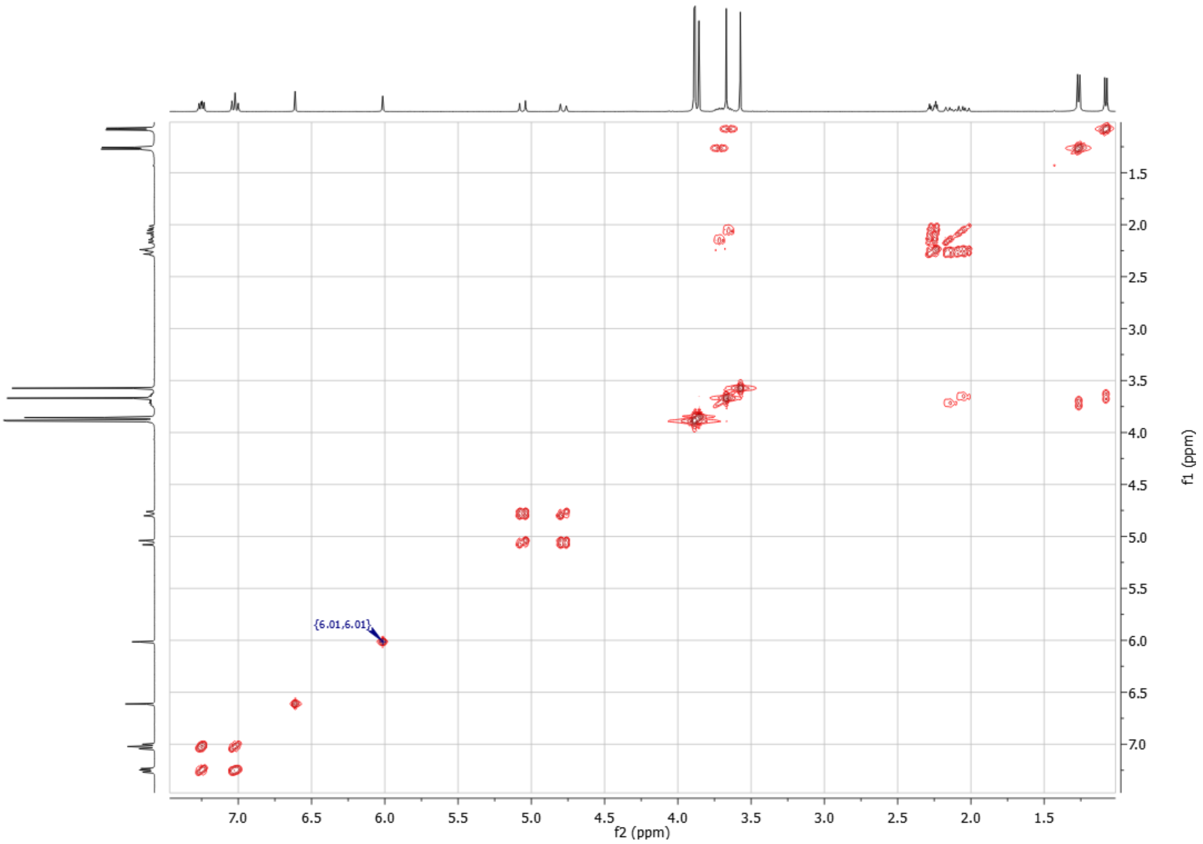

Figure S67. ^{1}H-^{1}H COSY NMR (400 MHz) spectrum of (a*R*,1*S*,3*S*,3'*S*)-**21** in $CDCl_3$.

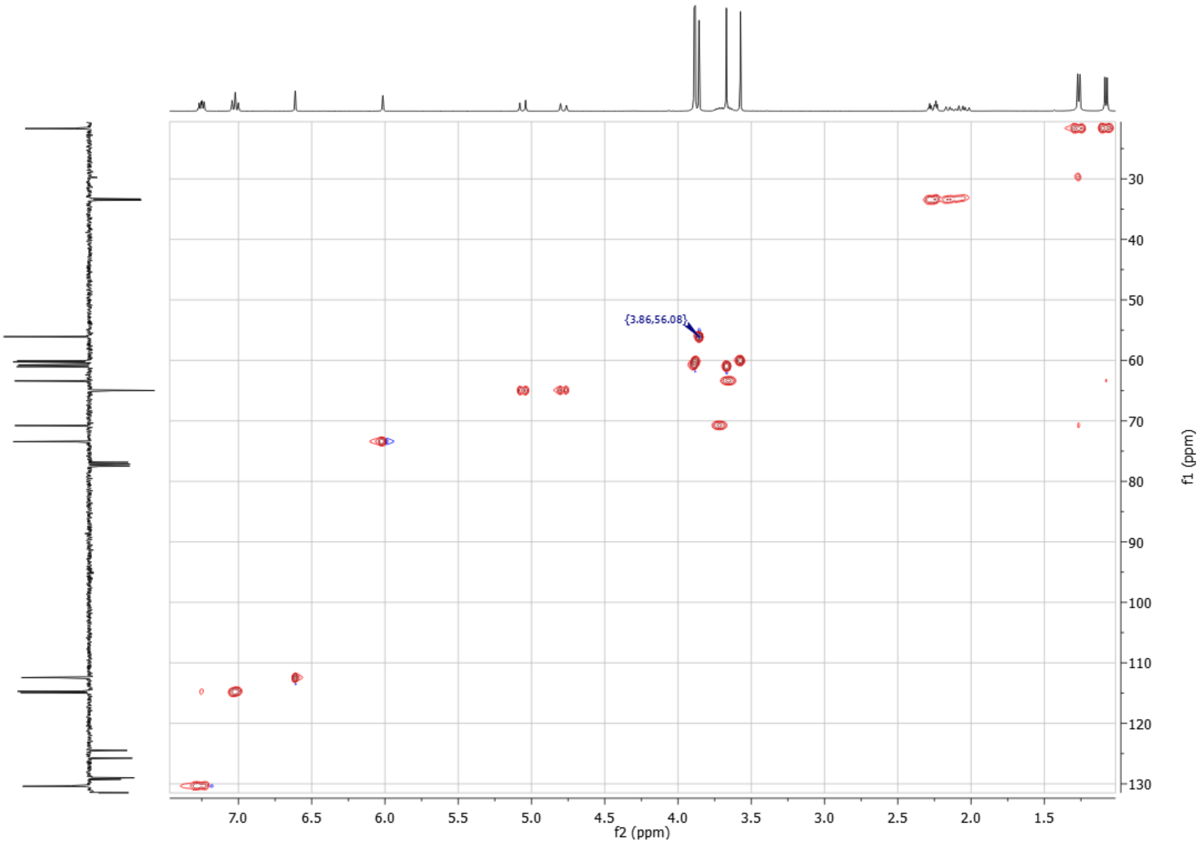

Figure S68. ^{1}H-^{13}C HSQC NMR (400 MHz) spectrum of (a*R*,1*S*,3*S*,3'*S*)-**21** in $CDCl_3$.

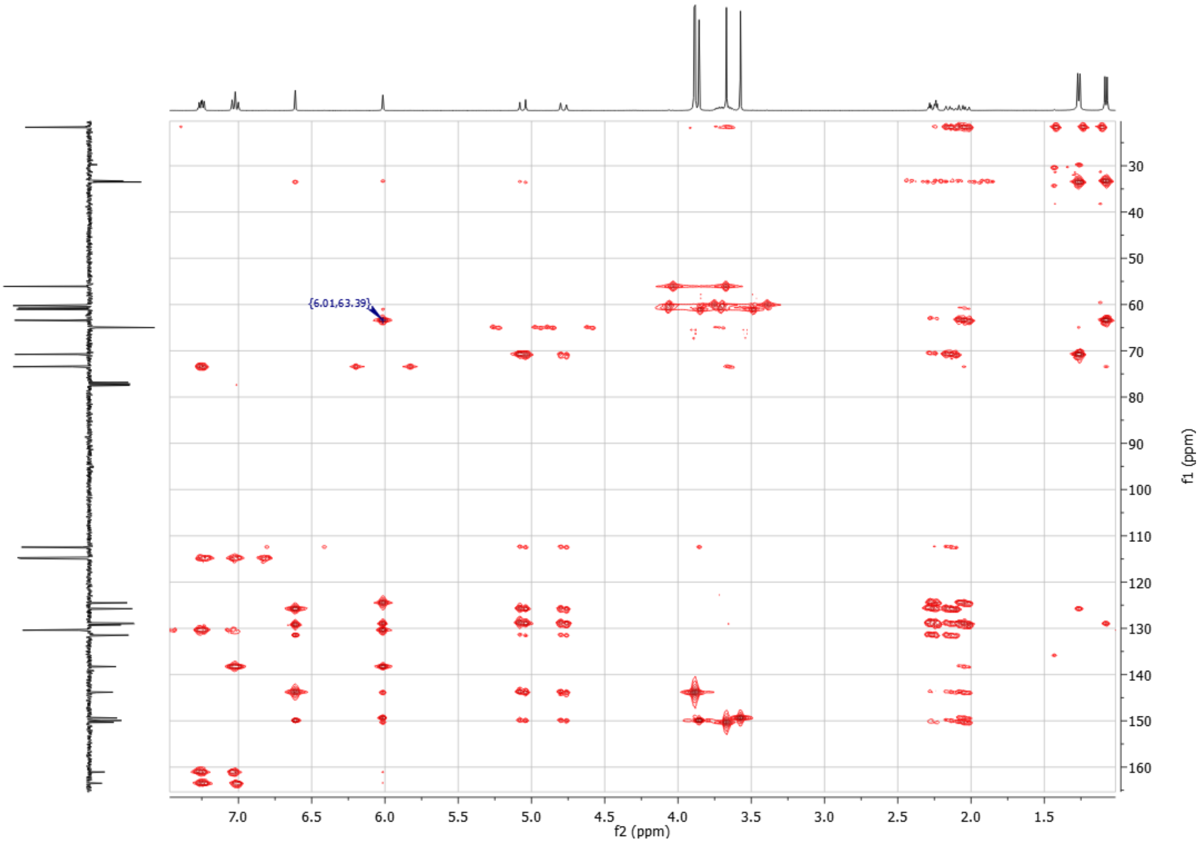

Figure S69. ^{1}H-^{13}C HMBC NMR (400 MHz) spectrum of (a*R*,1*S*,3*S*,3'*S*)-**21** in $CDCl_3$.

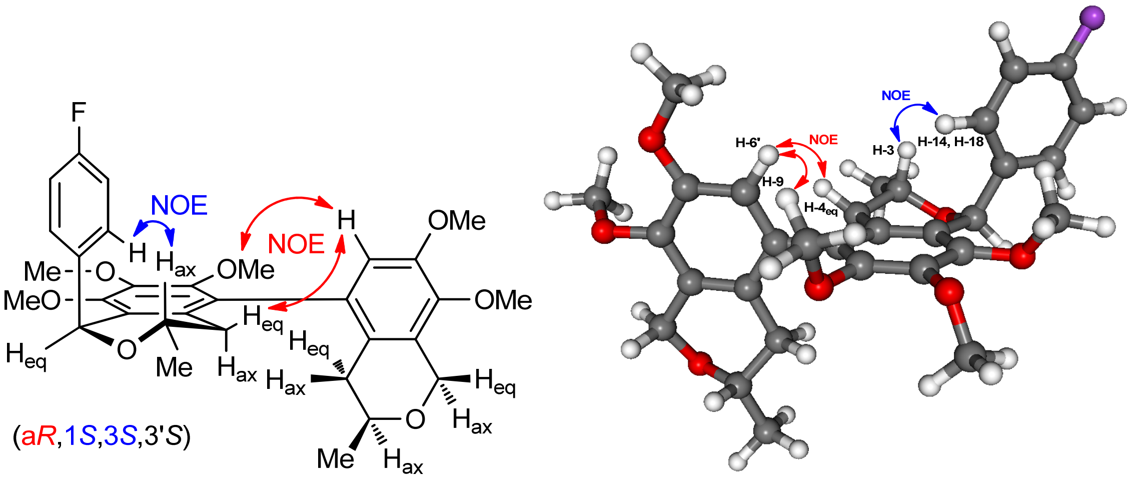

Figure S70. Characteristic NOE correlations shown on the the lowest-energy computed conformer of (a*R*,1*S*,3*S*,3'*S*)-**21** suggesting (1*S*,3*S*) configuration of isochroman subunit and (a*R*) axial chirality.

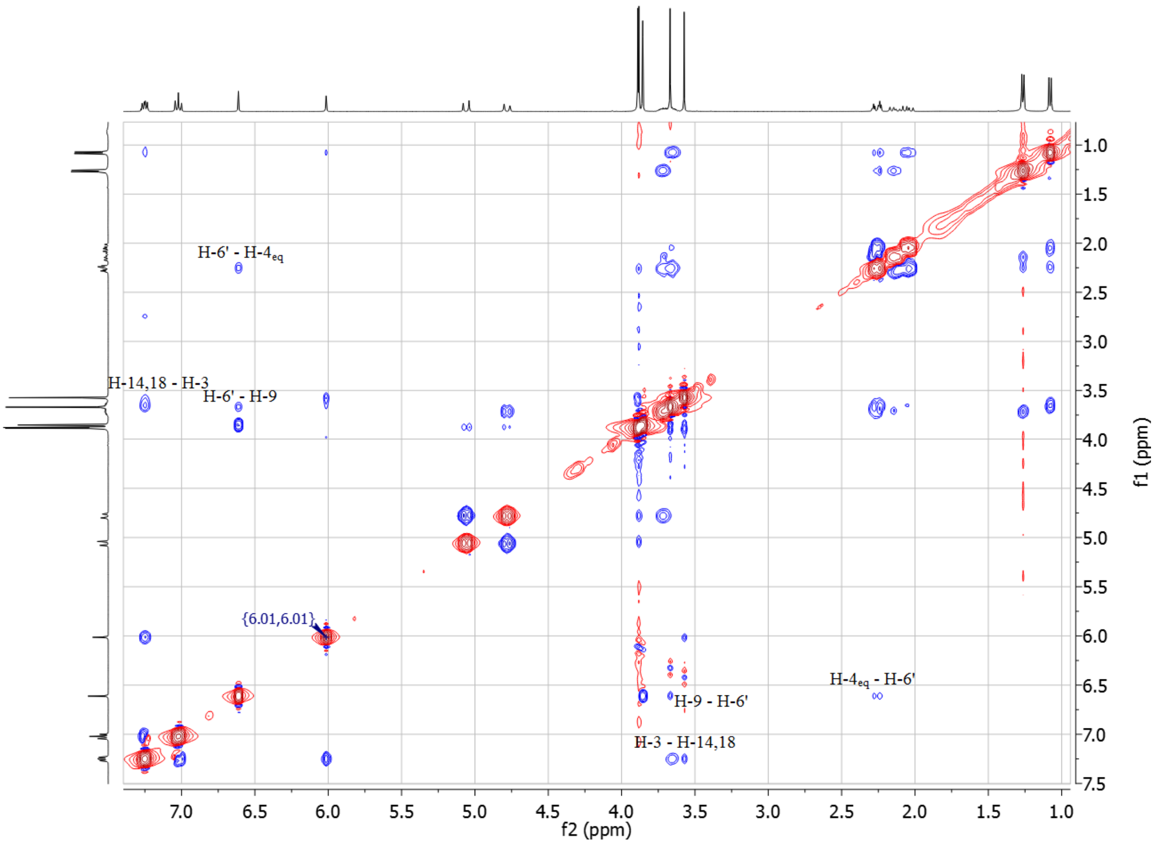

Figure S71. ^{1}H-^{1}H NOESY NMR (400 MHz) spectrum of (a*R*,1*S*,3*S*,3'*S*)-**21** in $CDCl_3$.

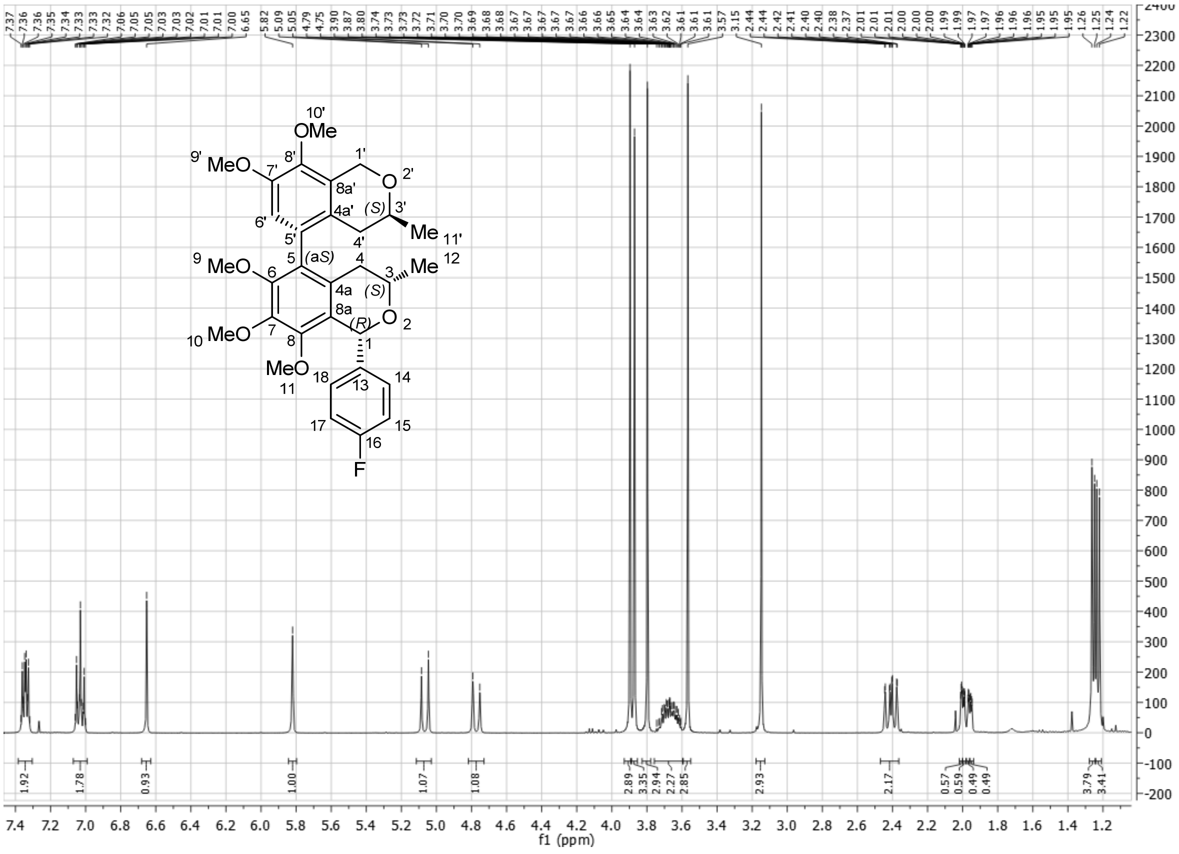

Figure S72. ^{1}H NMR (400 MHz) spectrum of (a*S*,1*R*,3*S*,3'*S*)-**21** in $CDCl_3$.

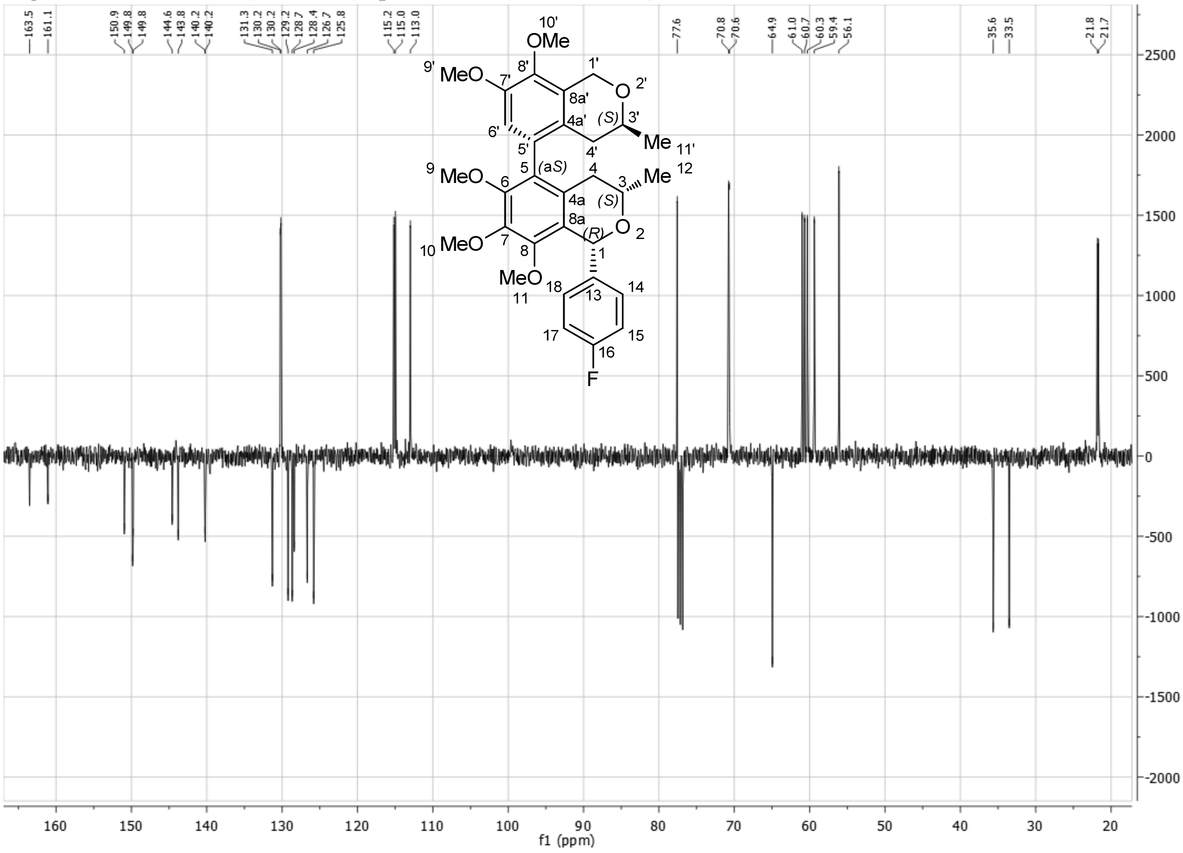

Figure S73. ^{13}C NMR (100 MHz) spectrum of (a*S*,1*R*,3*S*,3'*S*)-**21** in $CDCl_3$.

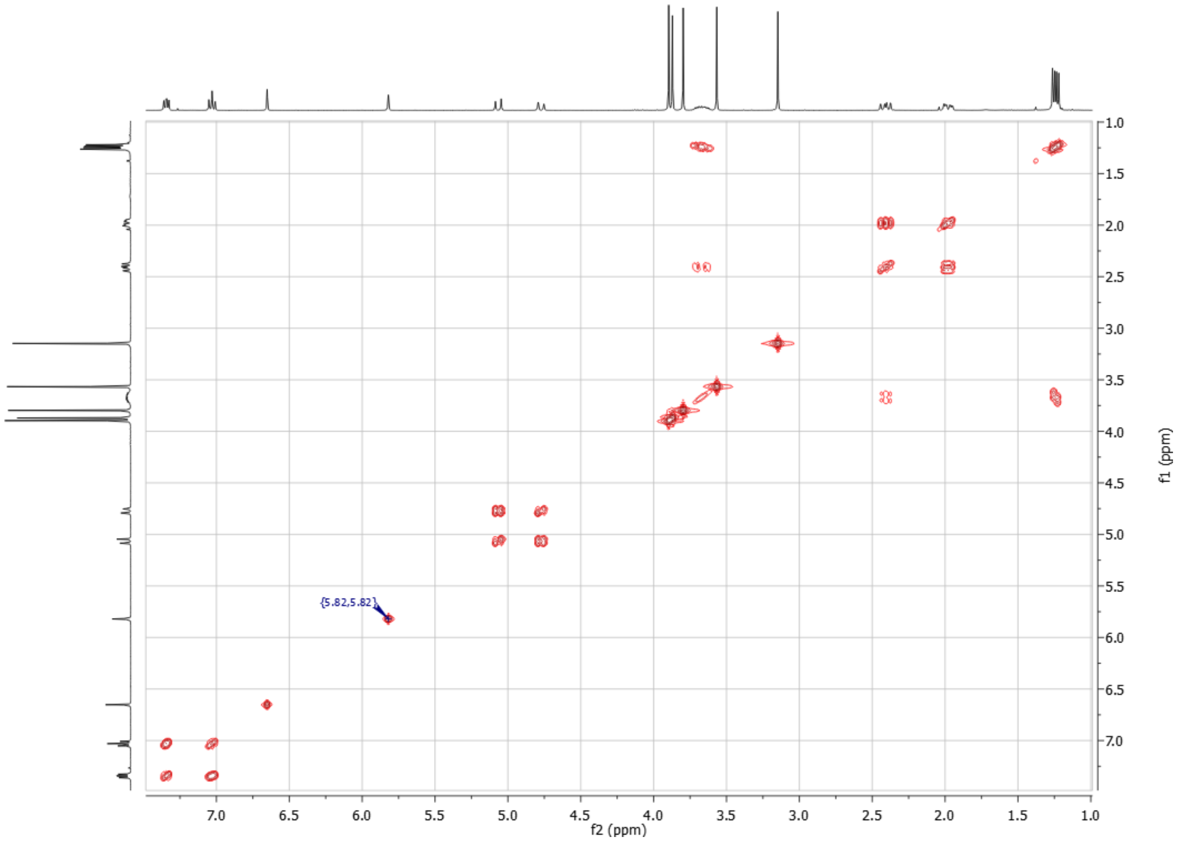

Figure S74. ^{1}H-^{1}H COSY NMR (400 MHz) spectrum of (a*S*,1*R*,3*S*,3'*S*)-**21** in $CDCl_3$.

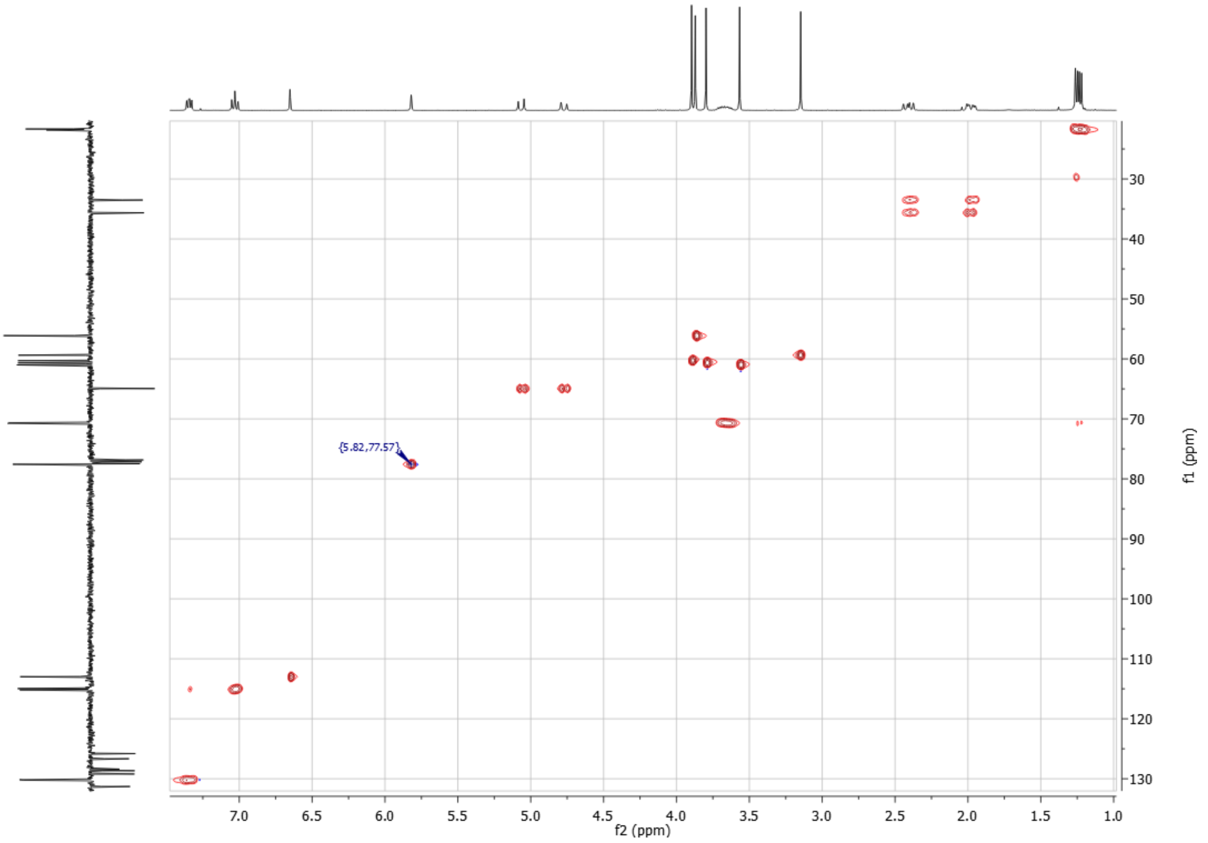

Figure S75. ^{1}H-^{13}C HSQC NMR (400 MHz) spectrum of (a*S*,1*R*,3*S*,3'*S*)-**21** in $CDCl_3$.

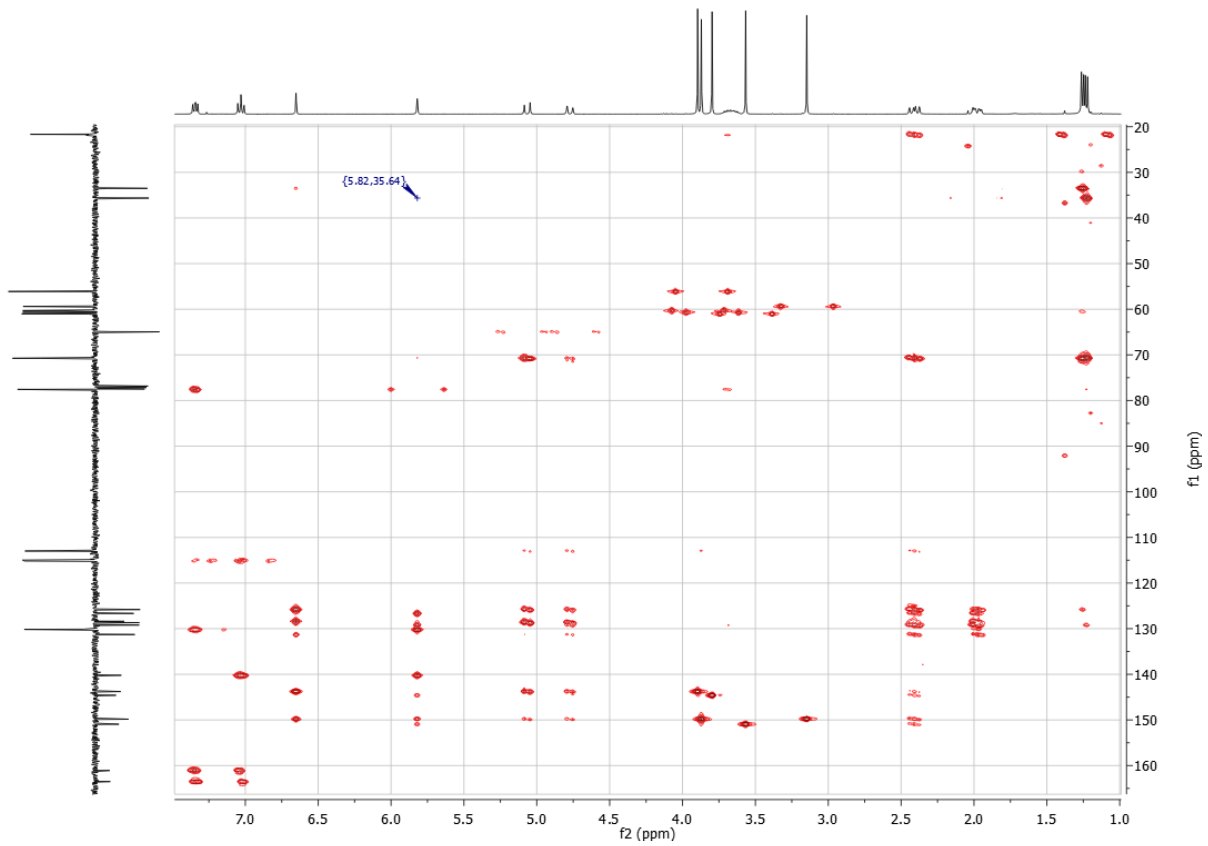

Figure S76. ^{1}H-^{13}C HMBC NMR (400 MHz) spectrum of (a*S*,1*R*,3*S*,3'*S*)-**21** in $CDCl_3$.

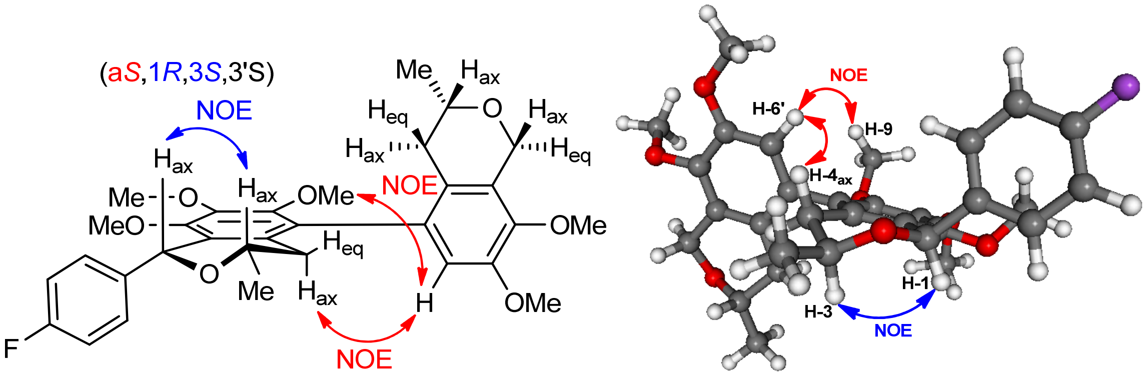

Figure S77. Characteristic NOE correlations shown on the lowest-energy computed conformer of (a*S*,1*R*,3*S*,3'*S*)-**21** suggesting (1*R*,3*S*) configuration of isochroman subunit and (a*S*) axial chirality.

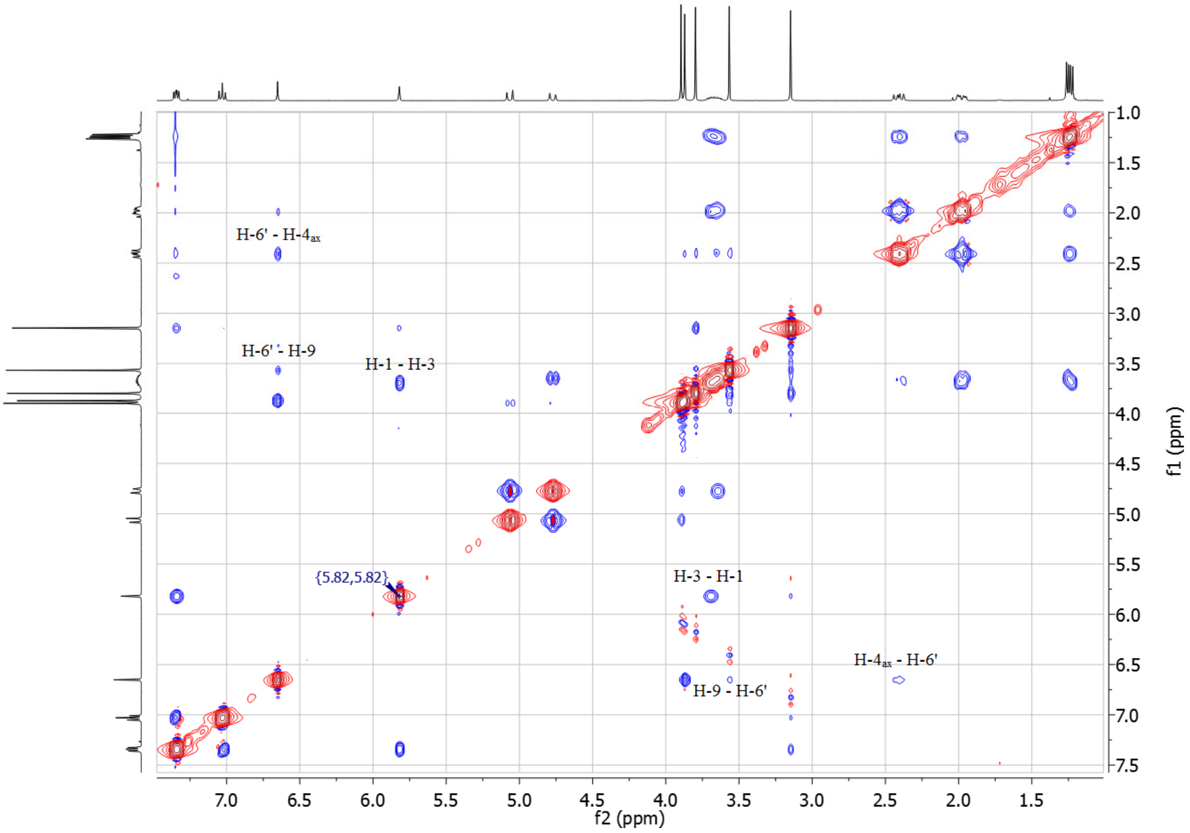

Figure S78. ^{1}H-^{1}H NOESY NMR (400 MHz) spectrum of (a*S*,1*R*,3*S*,3'*S*)-**21** in $CDCl_3$.

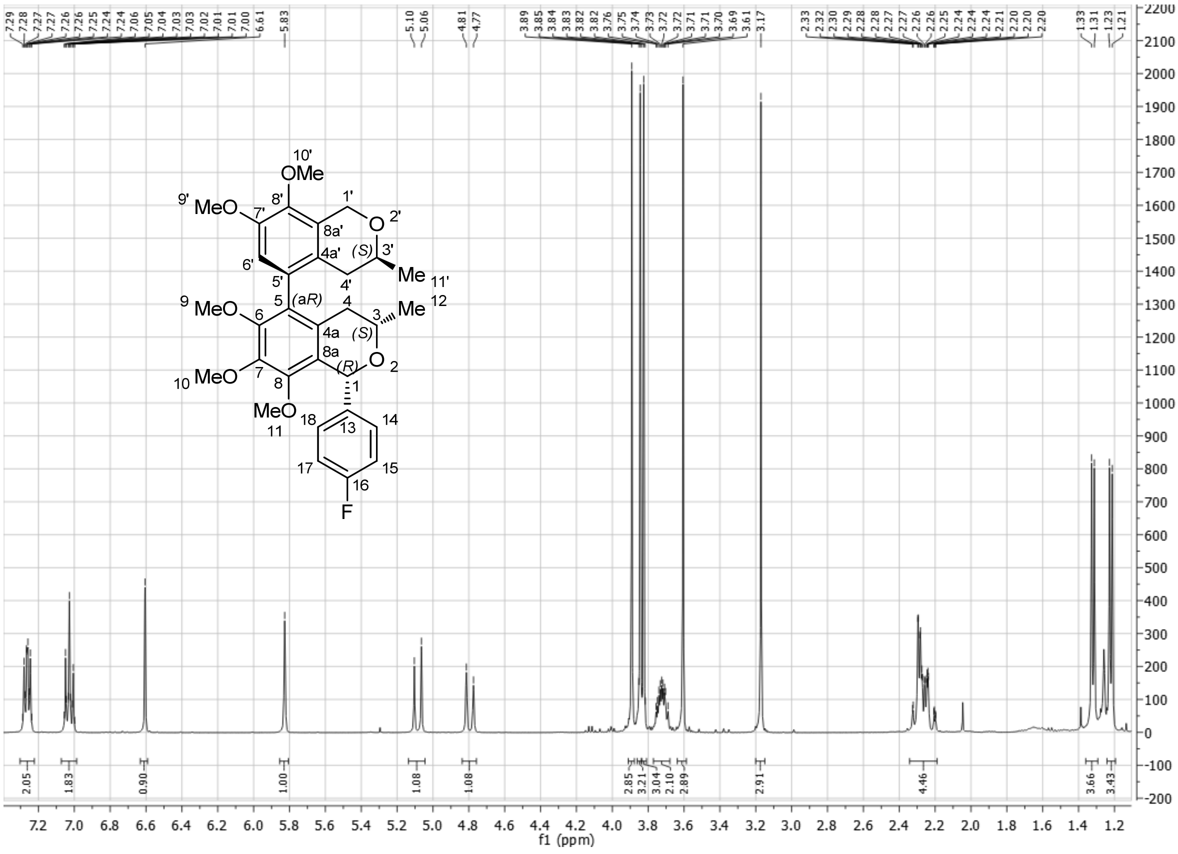

Figure S79. ^{1}H NMR (400 MHz) spectrum of (a*R*,1*R*,3*S*,3'*S*)-**21** in $CDCl_3$.

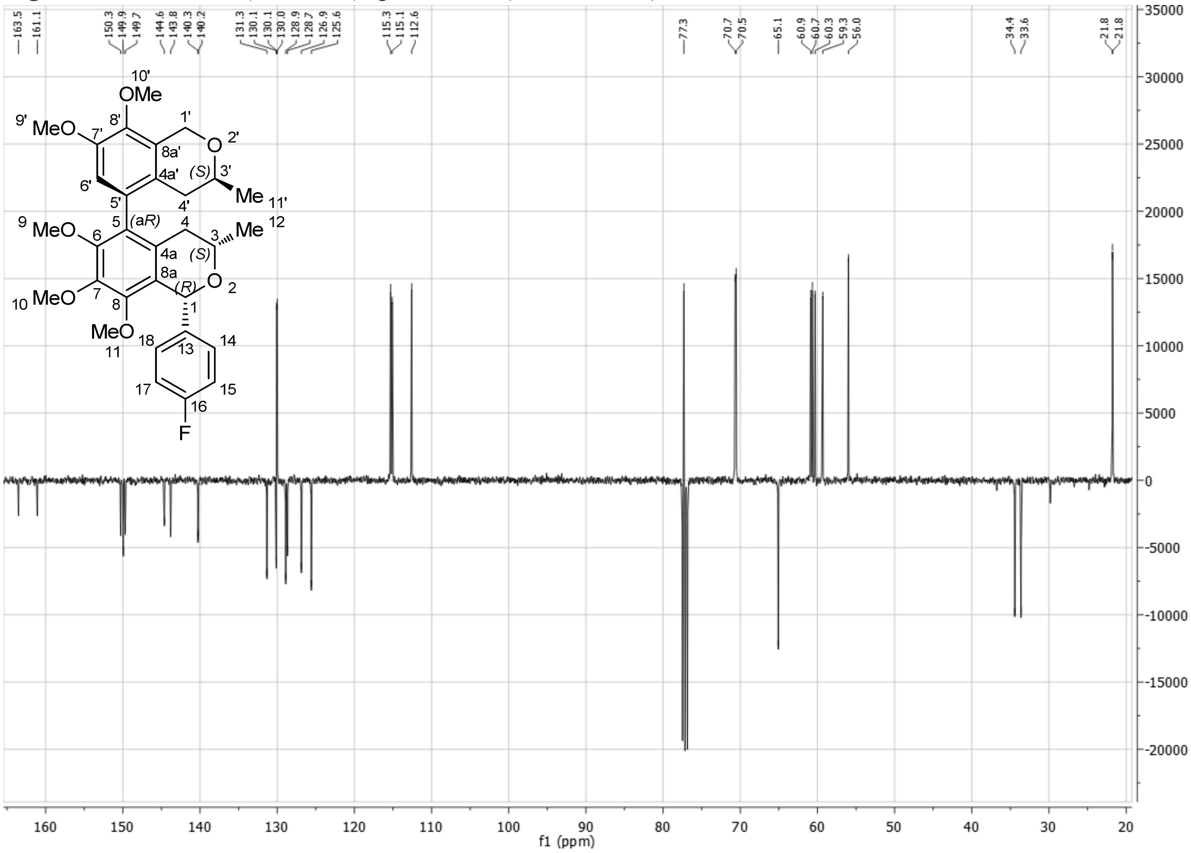

Figure S80. ^{13}C NMR (100 MHz) spectrum of (a*R*,1*R*,3*S*,3'*S*)-**21** in $CDCl_3$.

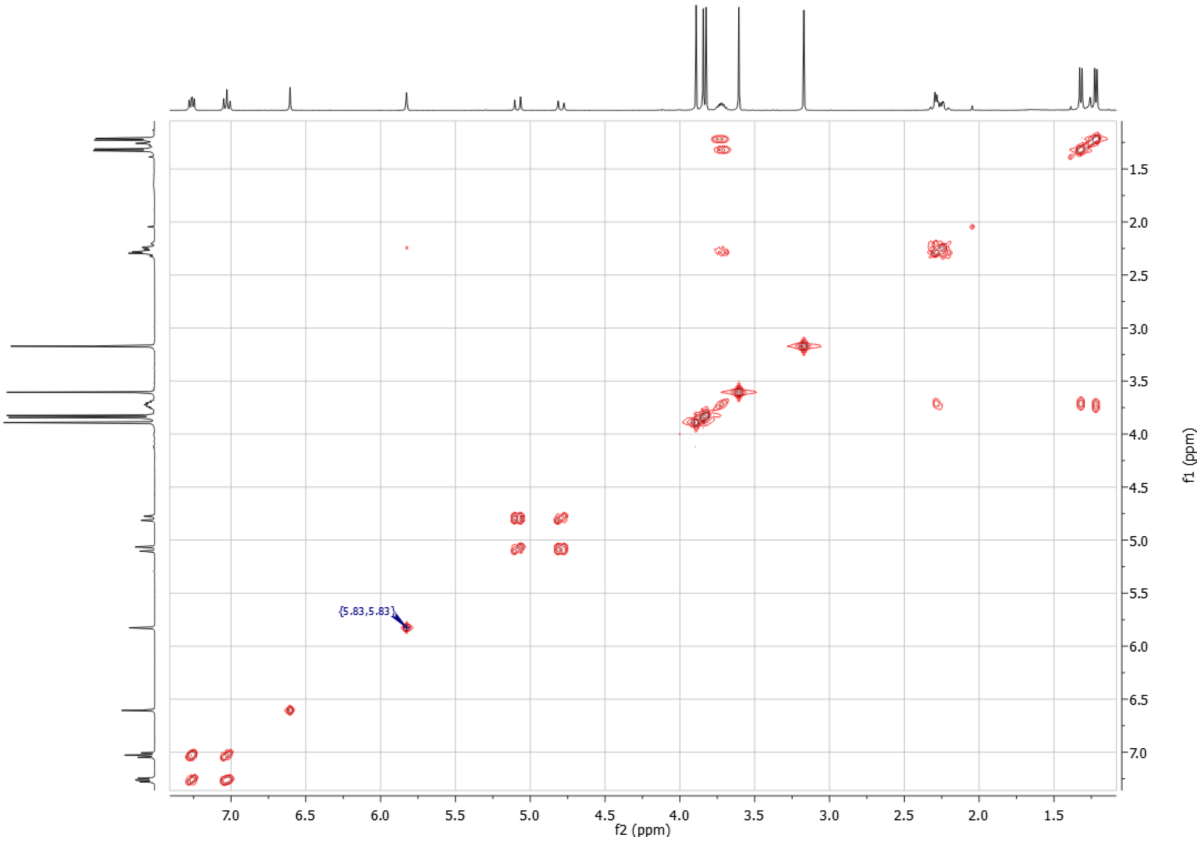

Figure S81. ^{1}H-^{1}H COSY NMR (400 MHz) spectrum of (a*R*,1*R*,3*S*,3'*S*)-**21** in $CDCl_3$.

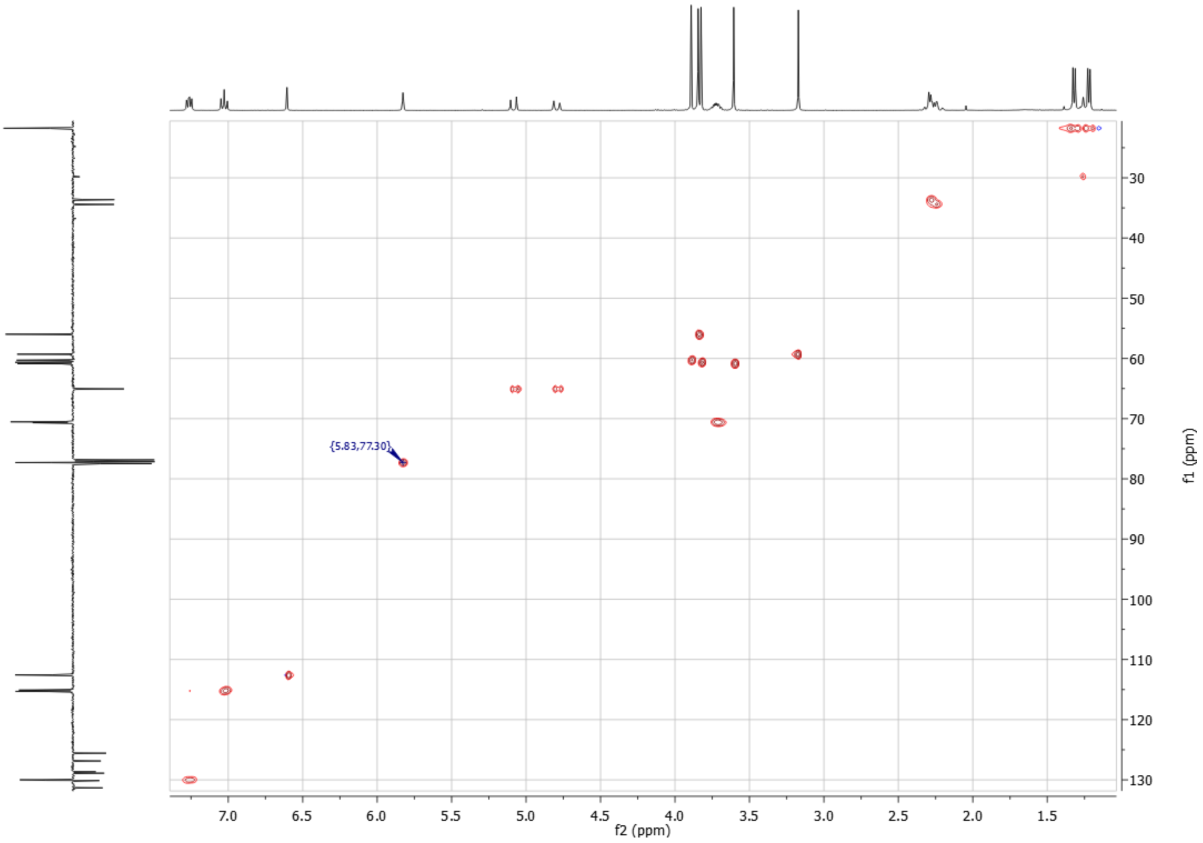

Figure S82. ^{1}H-^{13}C HSQC NMR (400 MHz) spectrum of (a*R*,1*R*,3*S*,3'*S*)-**21** in $CDCl_3$.

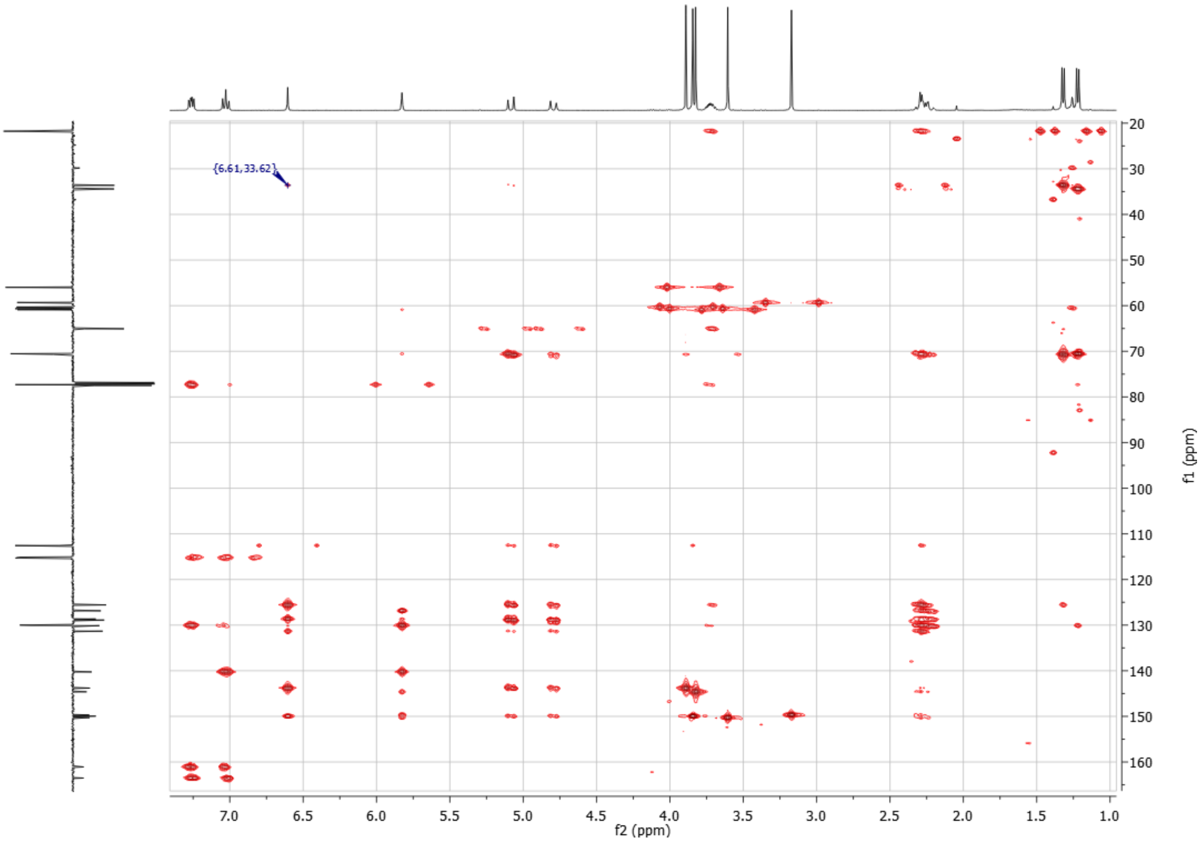

Figure S83. ^{1}H-^{13}C HMBC NMR (400 MHz) spectrum of (a*R*,1*R*,3*S*,3'*S*)-**21** in $CDCl_3$.

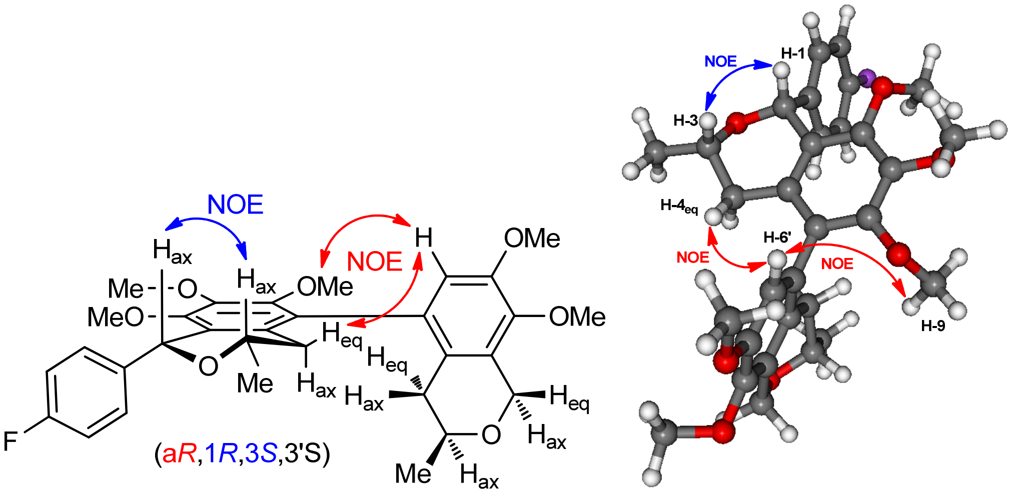

Figure S84. Characteristic NOE correlations shown on the lowest-energy computed conformer of (a*R*,1*R*,3*S*,3'*S*)-**21** suggesting (1*R*,3*S*) configuration of isochroman subunit and (a*R*) axial chirality.

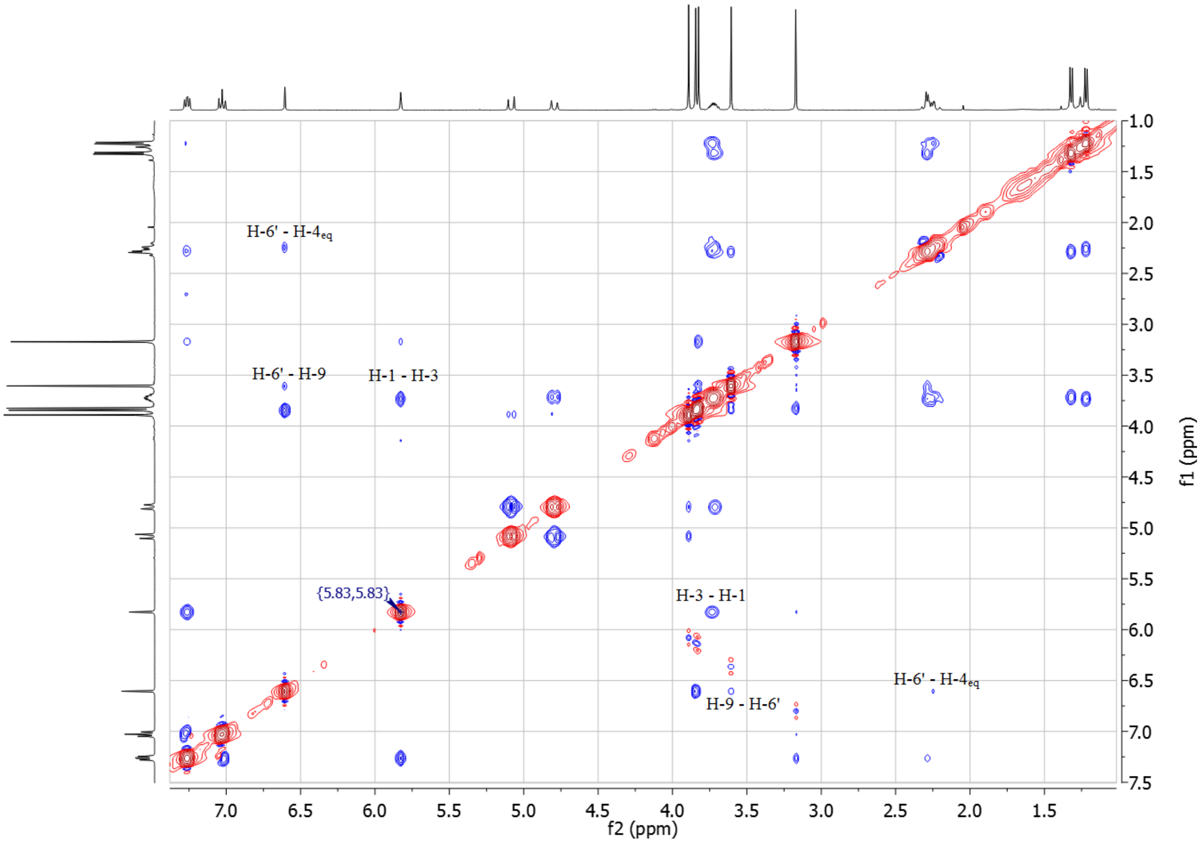

Figure S85. ^{1}H-^{1}H NOESY NMR (400 MHz) spectrum of (a*R*,1*R*,3*S*,3'*S*)-**21** in $CDCl_3$.

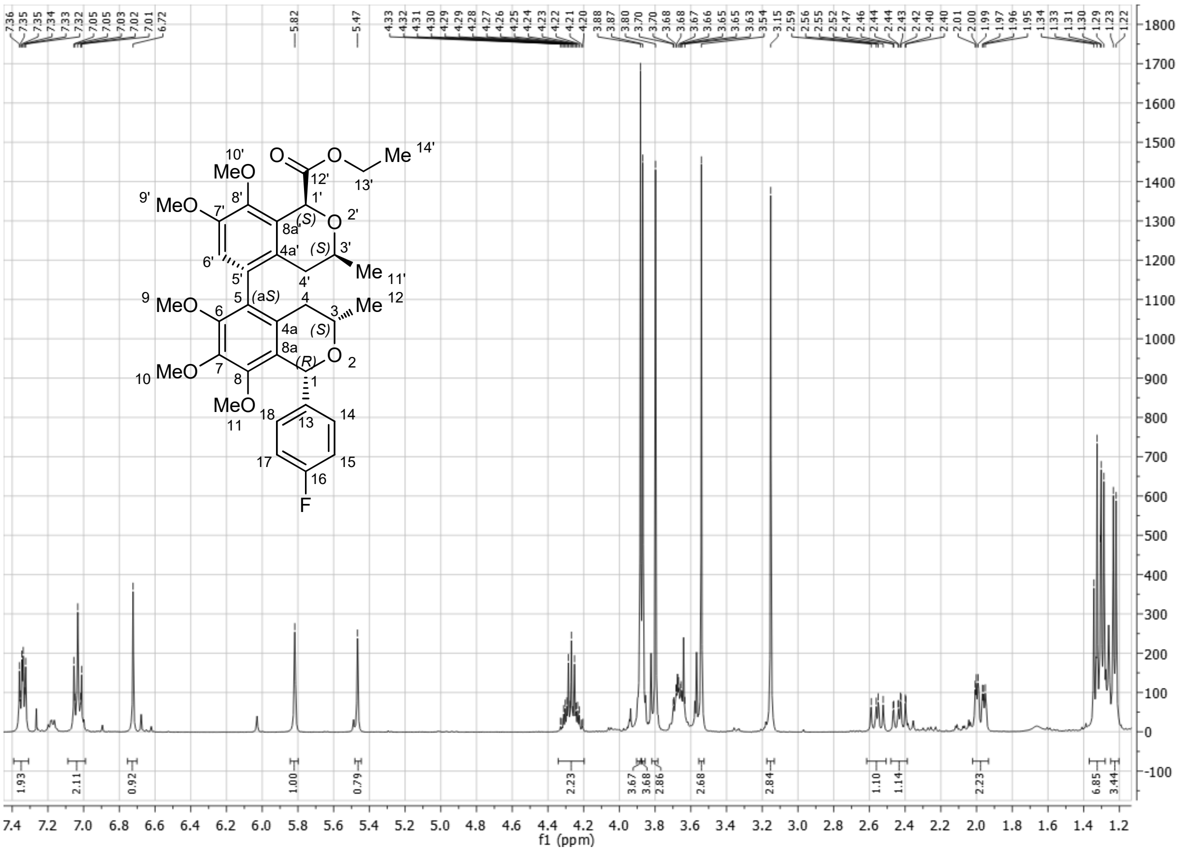

Figure S86. ^{1}H NMR (400 MHz) spectrum of (a*S*,1*R*,3*S*,1'*S*,3'*S*)-**22** in $CDCl_3$.

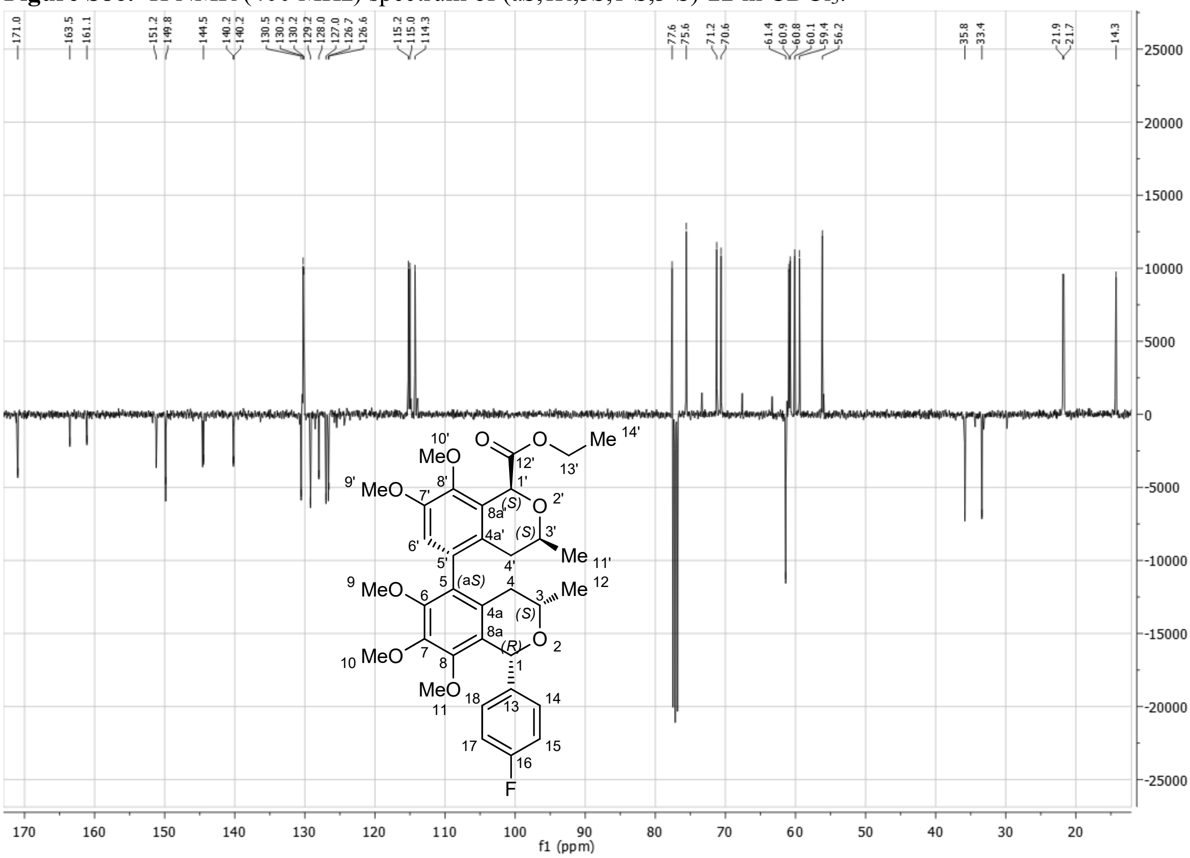

Figure S87. ^{13}C NMR (100 MHz) spectrum of (a*S*,1*R*,3*S*,1'*S*,3'*S*)-**22** in $CDCl_3$.

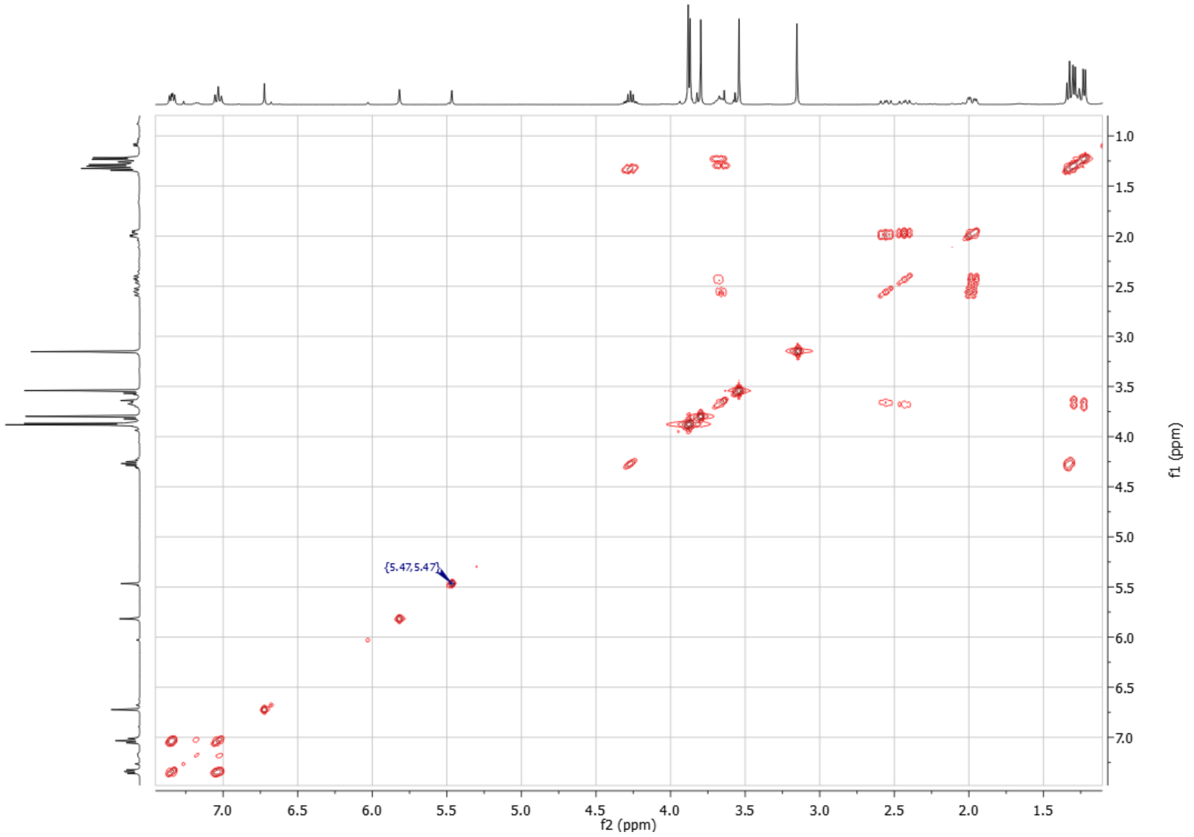

Figure S88. ^{1}H-^{1}H COSY NMR (400 MHz) spectrum of (a*S*,1*R*,3*S*,1'*S*,3'*S*)-**22** in $CDCl_3$.

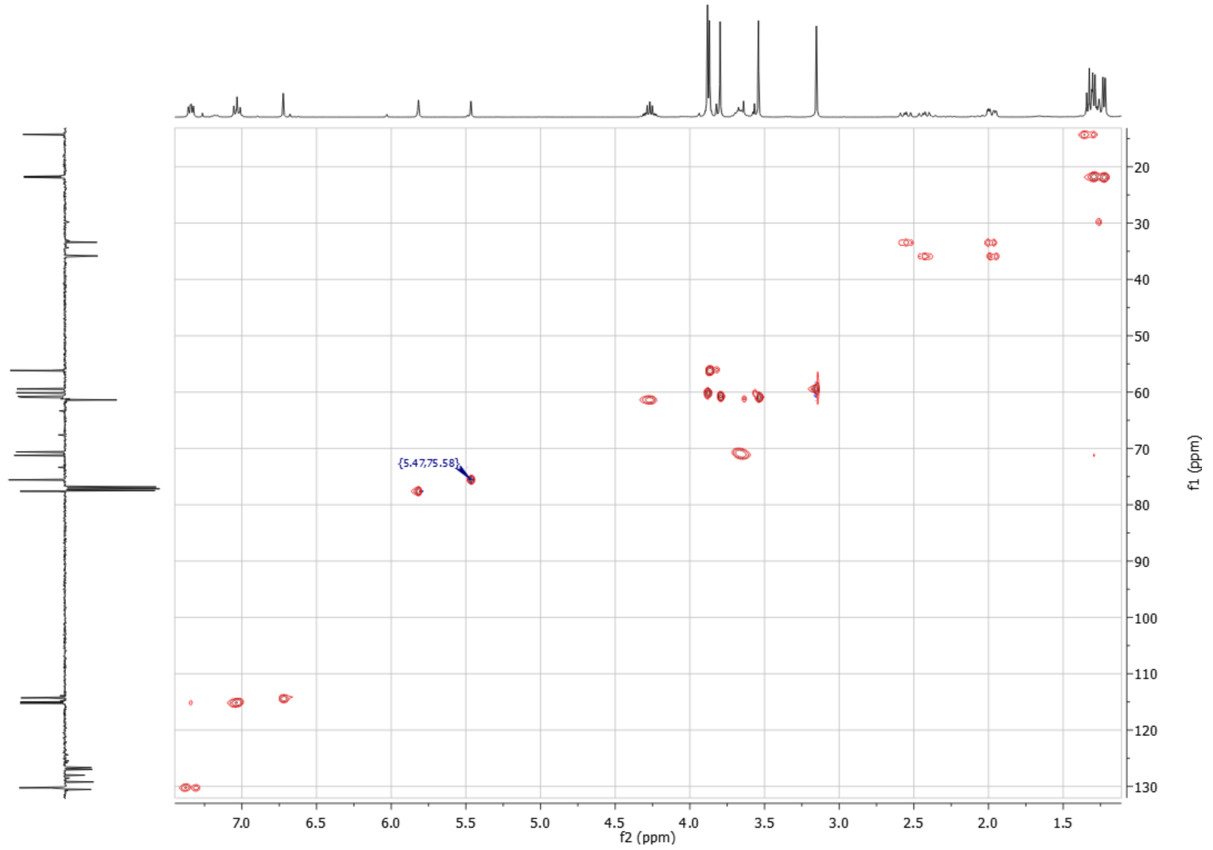

Figure S89. ^{1}H-^{13}C HSQC NMR (400 MHz) spectrum of (a*S*,1*R*,3*S*,1'*S*,3'*S*)-**22** in $CDCl_3$.

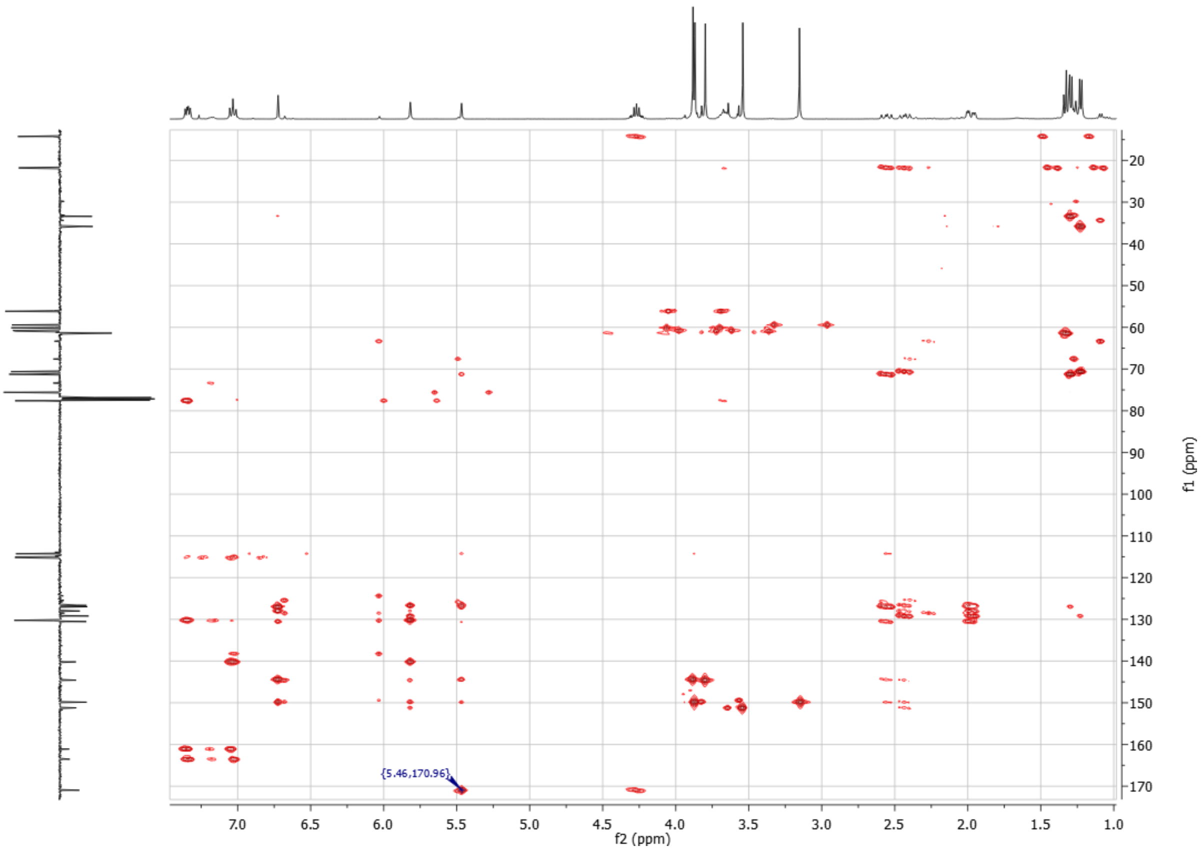

Figure S90. ^{1}H-^{13}C HMBC NMR (400 MHz) spectrum of (a*S*,1*R*,3*S*,1'*S*,3'*S*)-**22** in $CDCl_3$.

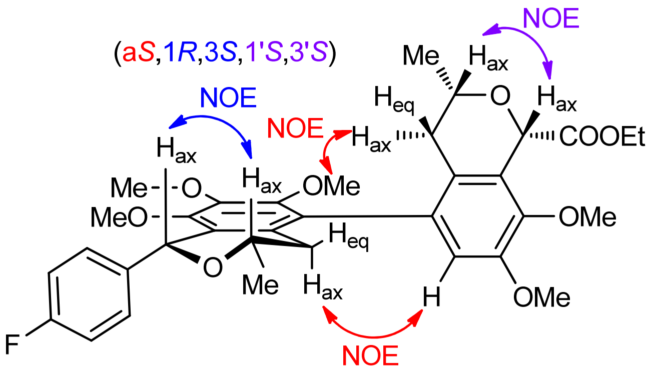

Figure S91. Characteristic NOE correlations shown on the structure of (a*S*,1*R*,3*S*,1'*S*,3'*S*)-**22** suggesting (1*R*,3*S*,1'*S*,3'*S*) configurations of isochroman subunits and (a*S*) axial chirality.

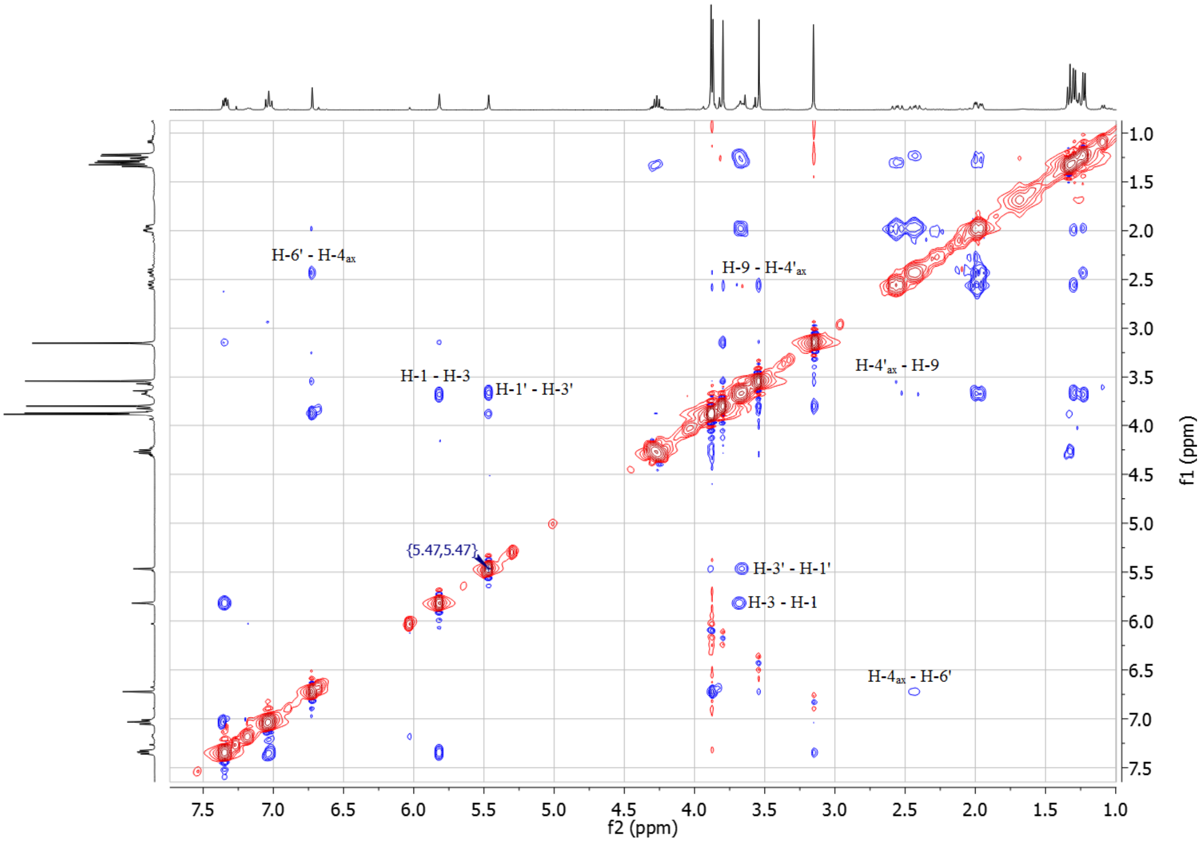

Figure S92. ^{1}H-^{1}H NOESY NMR (400 MHz) spectrum of (a*S*,1*R*,3*S*,1'*S*,3'*S*)-**22** in $CDCl_3$.

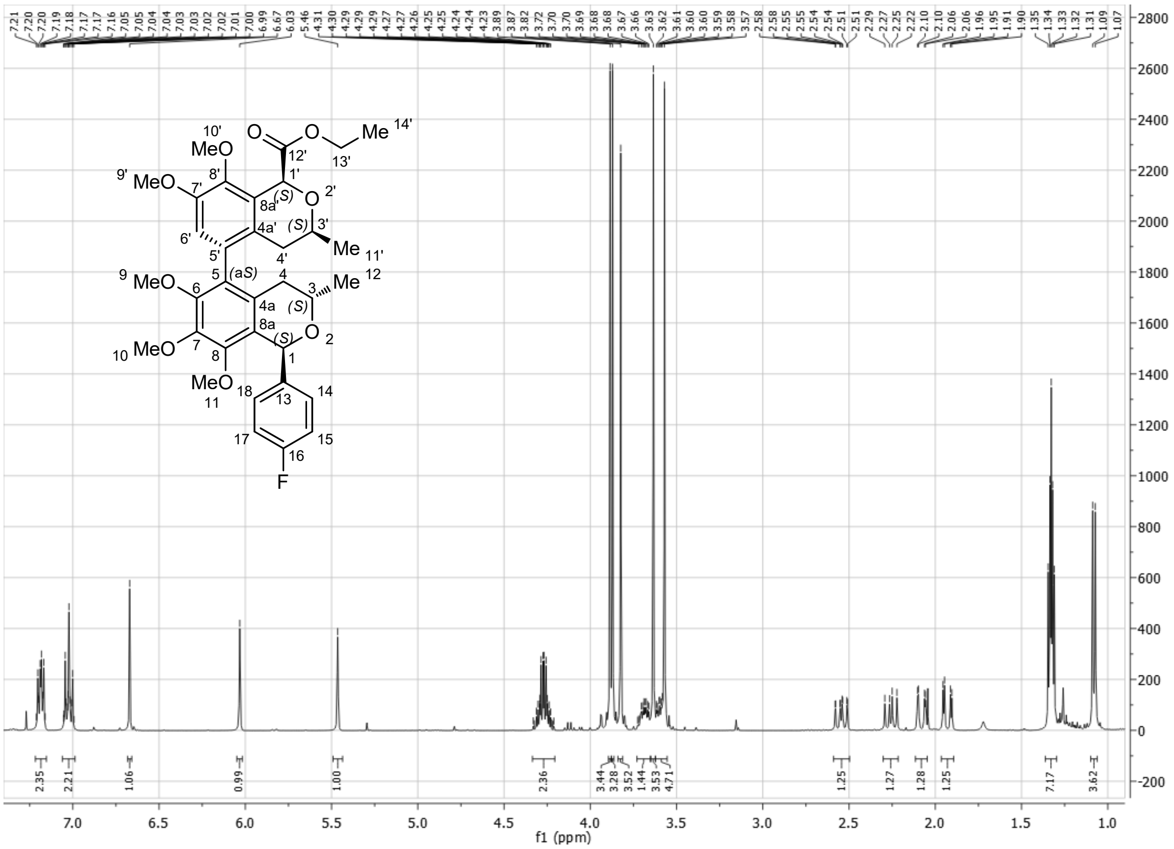

Figure S93. ^{1}H NMR (400 MHz) spectrum of (a*S*,1*S*,3*S*,1'*S*,3'*S*)-**22** in $CDCl_3$.

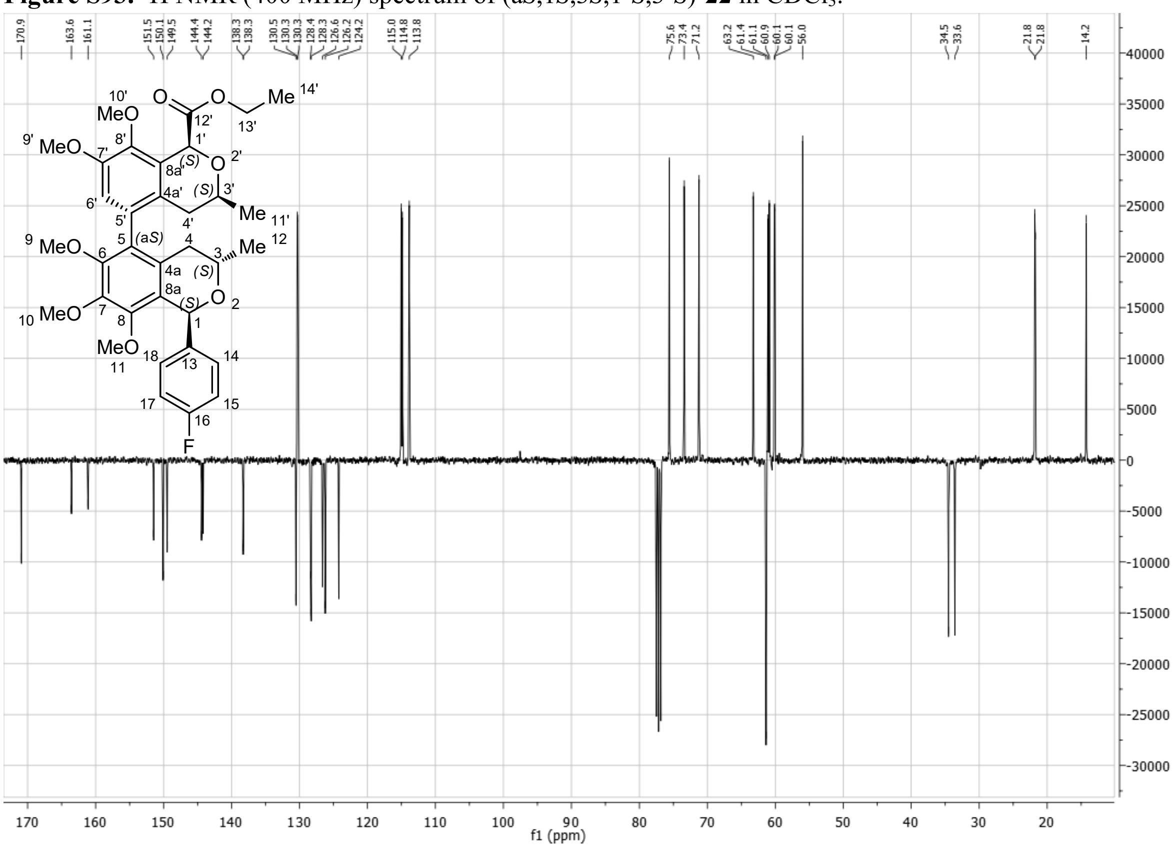

Figure S94. ^{13}C NMR (100 MHz) spectrum of (a*S*,1*S*,3*S*,1'*S*,3'*S*)-**22** in $CDCl_3$.

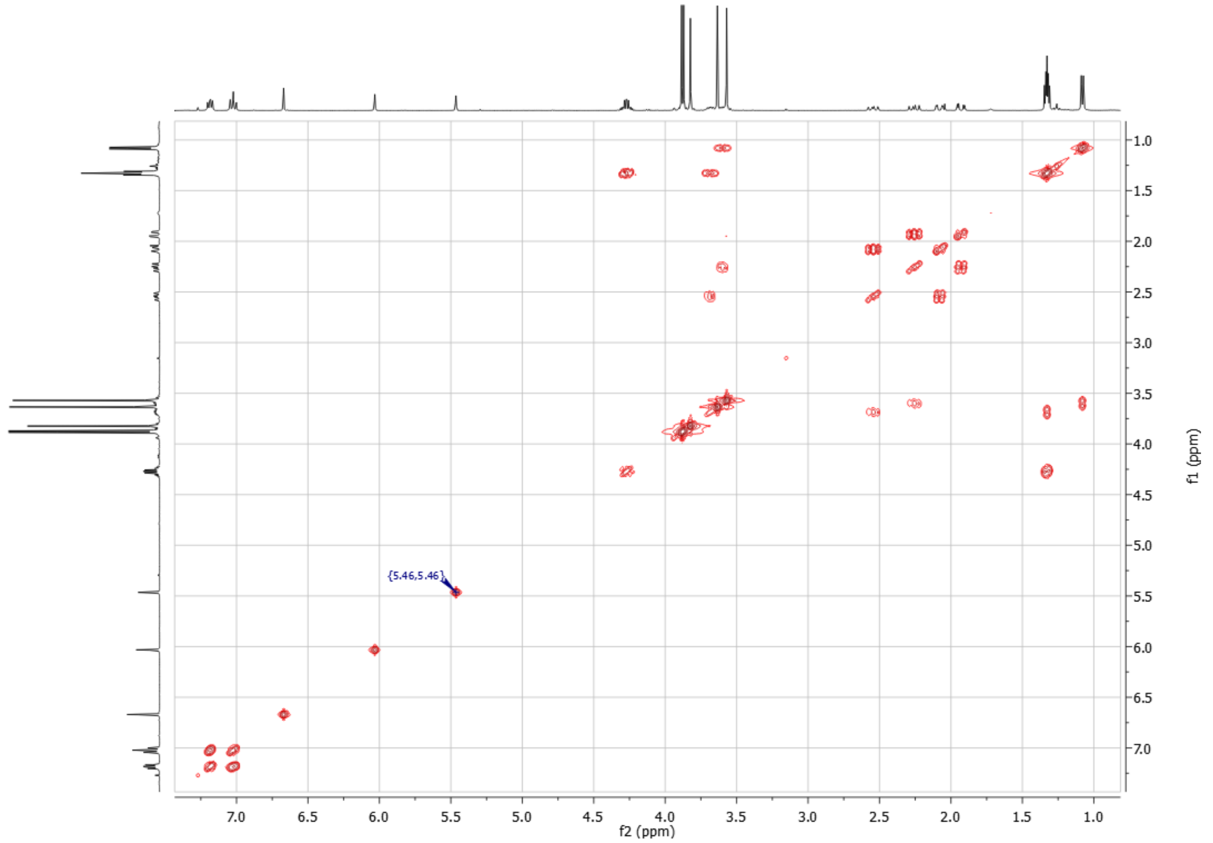

Figure S95. ^{1}H-^{1}H COSY NMR (400 MHz) spectrum of (a*S*,1*S*,3*S*,1'*S*,3'*S*)-**22** in $CDCl_3$.

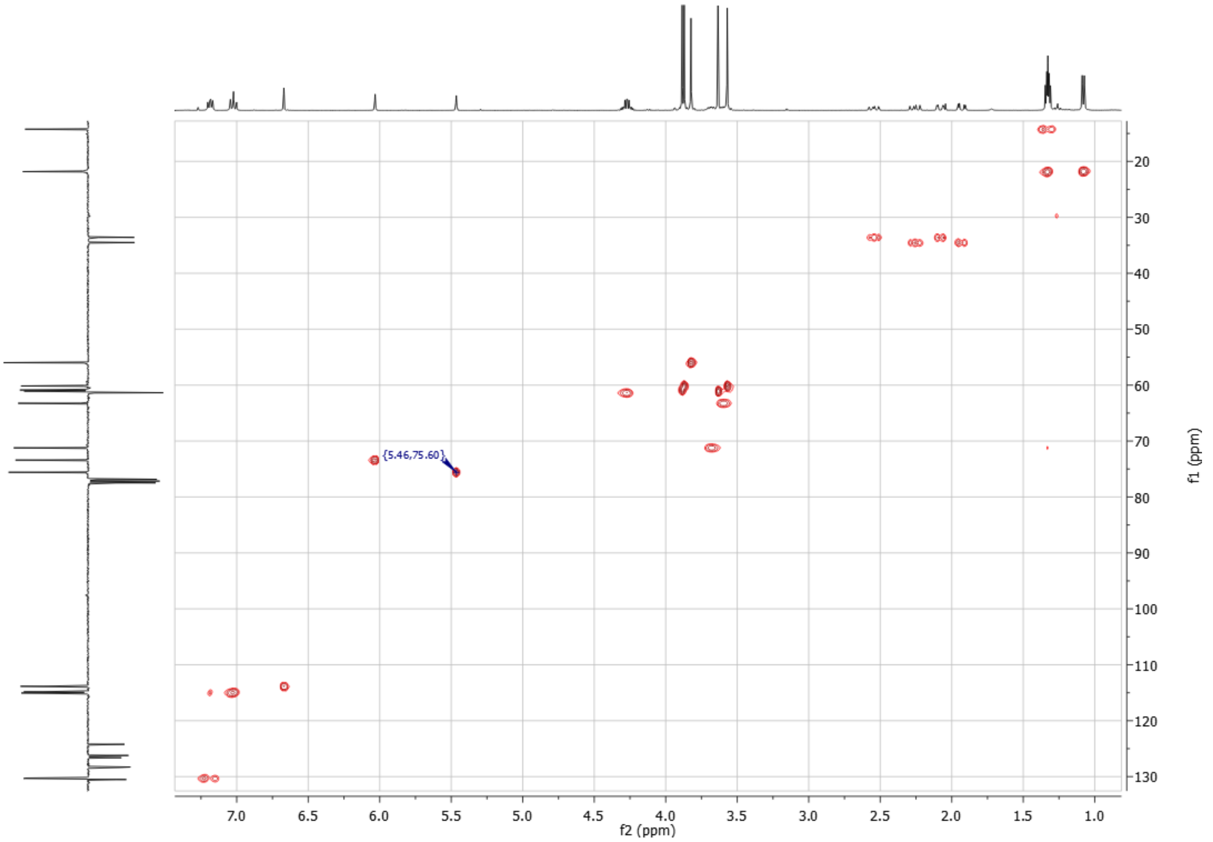

Figure S96. ^{1}H-^{13}C HSQC NMR (400 MHz) spectrum of (a*S*,1*S*,3*S*,1'*S*,3'*S*)-**22** in $CDCl_3$.

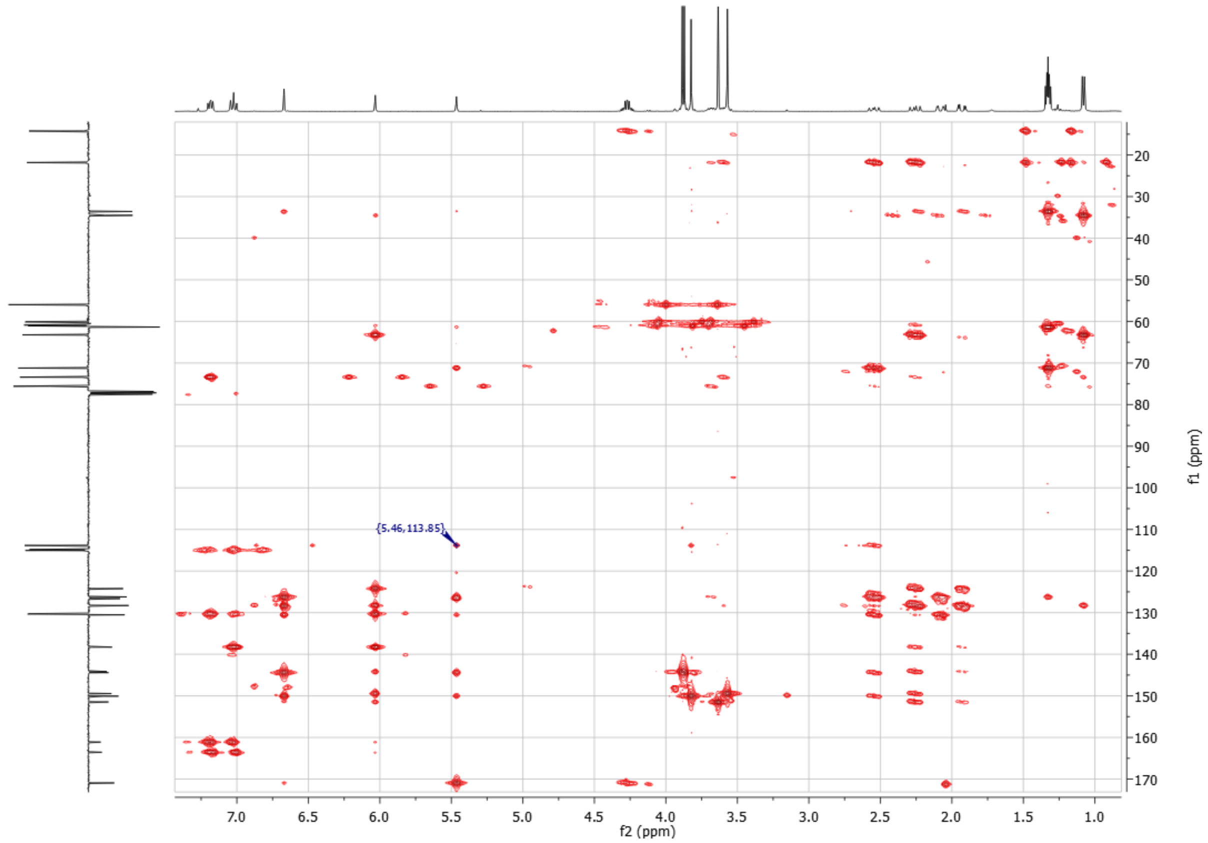

Figure S97. ^{1}H-^{13}C HMBC NMR (400 MHz) spectrum of (a*S*,1*S*,3*S*,1'*S*,3'*S*)-**22** in $CDCl_3$.

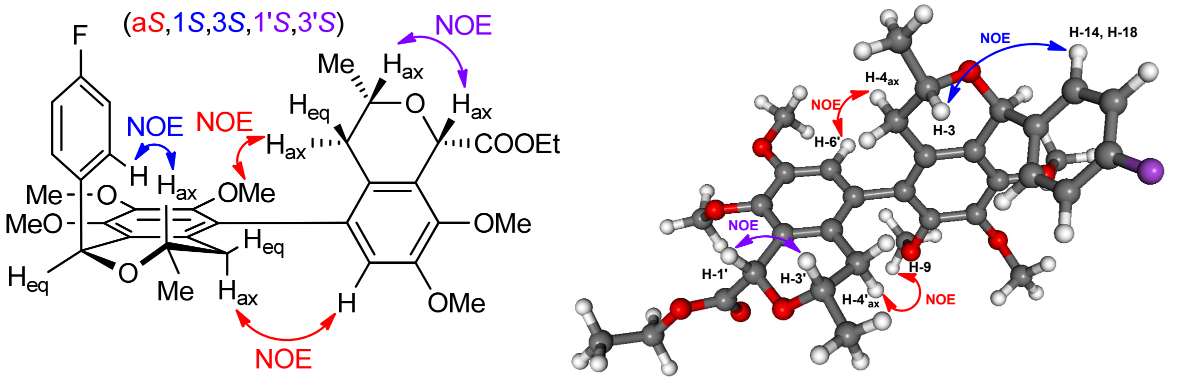

Figure S98. Characteristic NOE correlations shown on the lowest-energy computed conformer of (a*S*,1*S*,3*S*,1'*S*,3'*S*)-**22** suggesting (1*S*,3*S*,1'*S*,3'*S*) configurations of isochroman subunits and (a*S*) axial chirality.

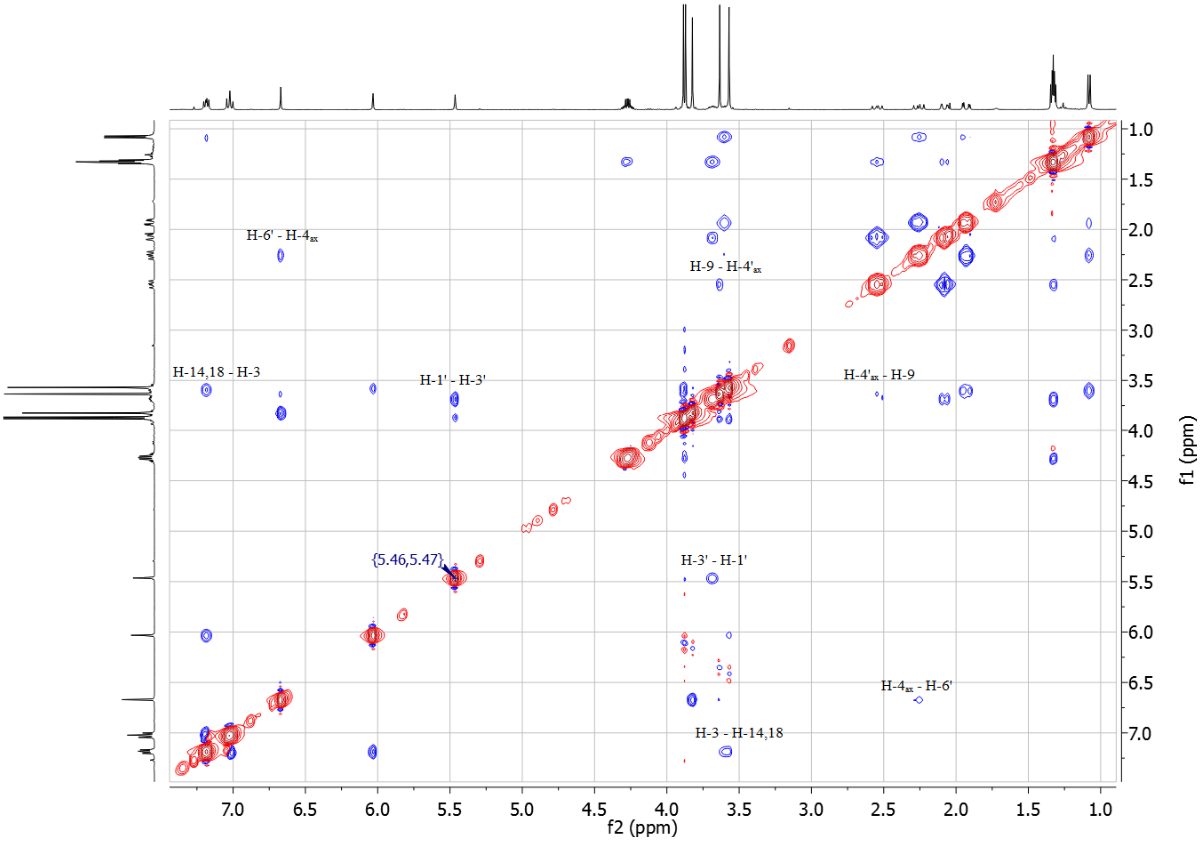

Figure S99. ^{1}H-^{1}H NOESY NMR (400 MHz) spectrum of (a*S*,1*S*,3*S*,1'*S*,3'*S*)-**22** in $CDCl_3$.

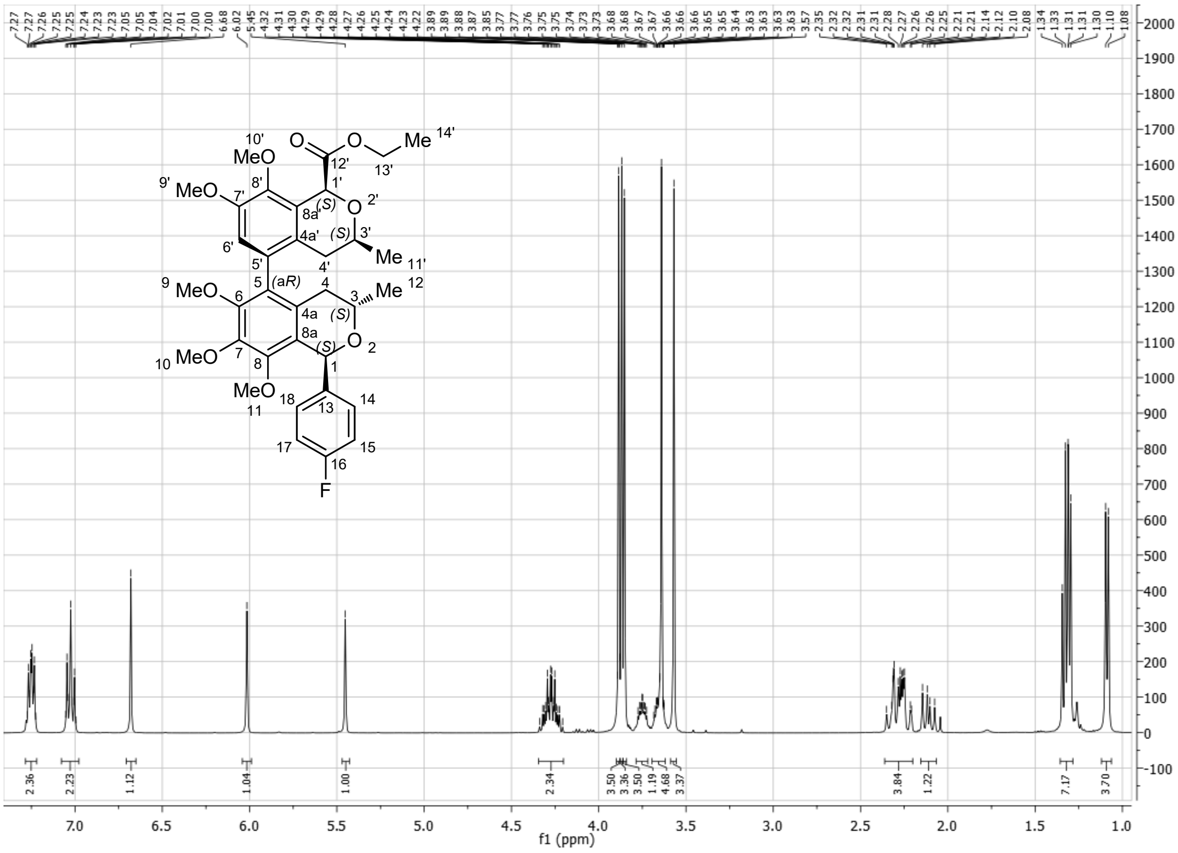

Figure S100. ^{1}H NMR (400 MHz) spectrum of (a*R*,1*S*,3*S*,1'*S*,3'*S*)-**22** in $CDCl_3$.

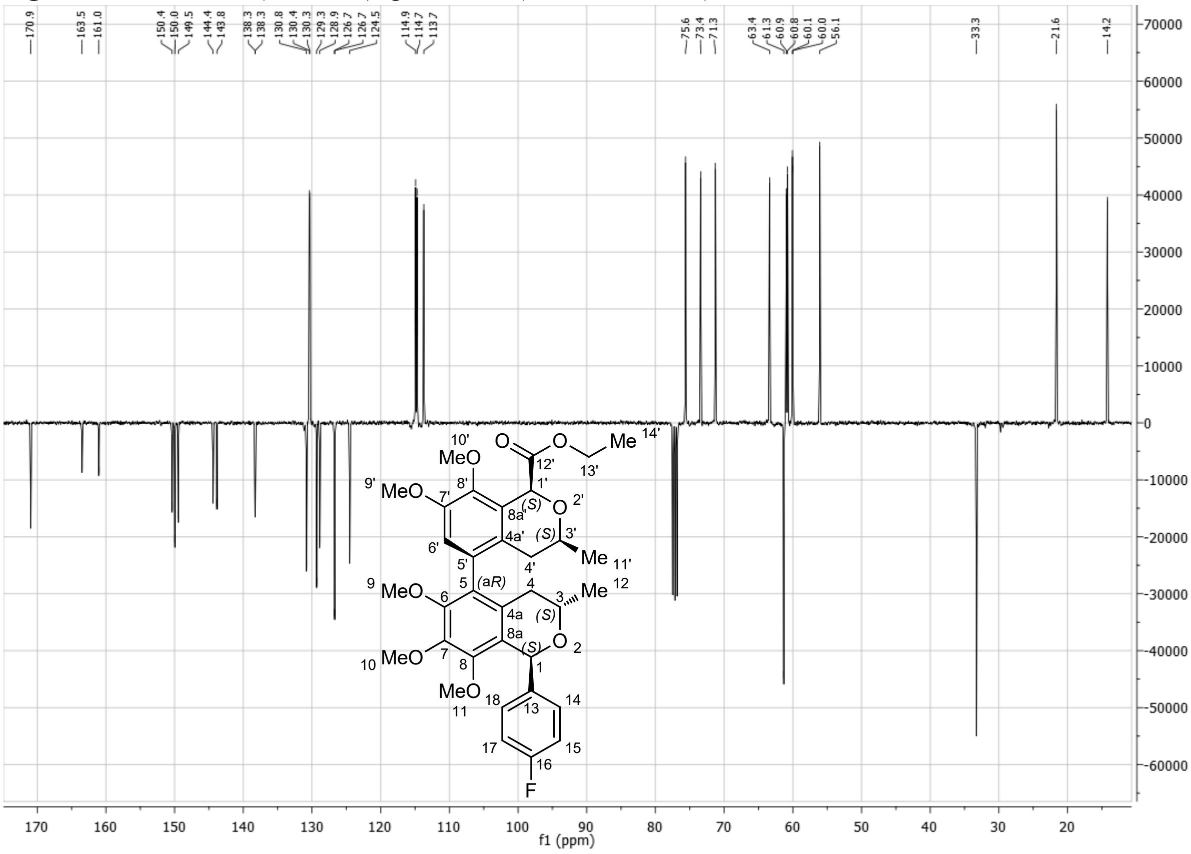

Figure S101. ^{13}C NMR (100 MHz) spectrum of (a*R*,1*S*,3*S*,1'*S*,3'*S*)-**22** in $CDCl_3$.

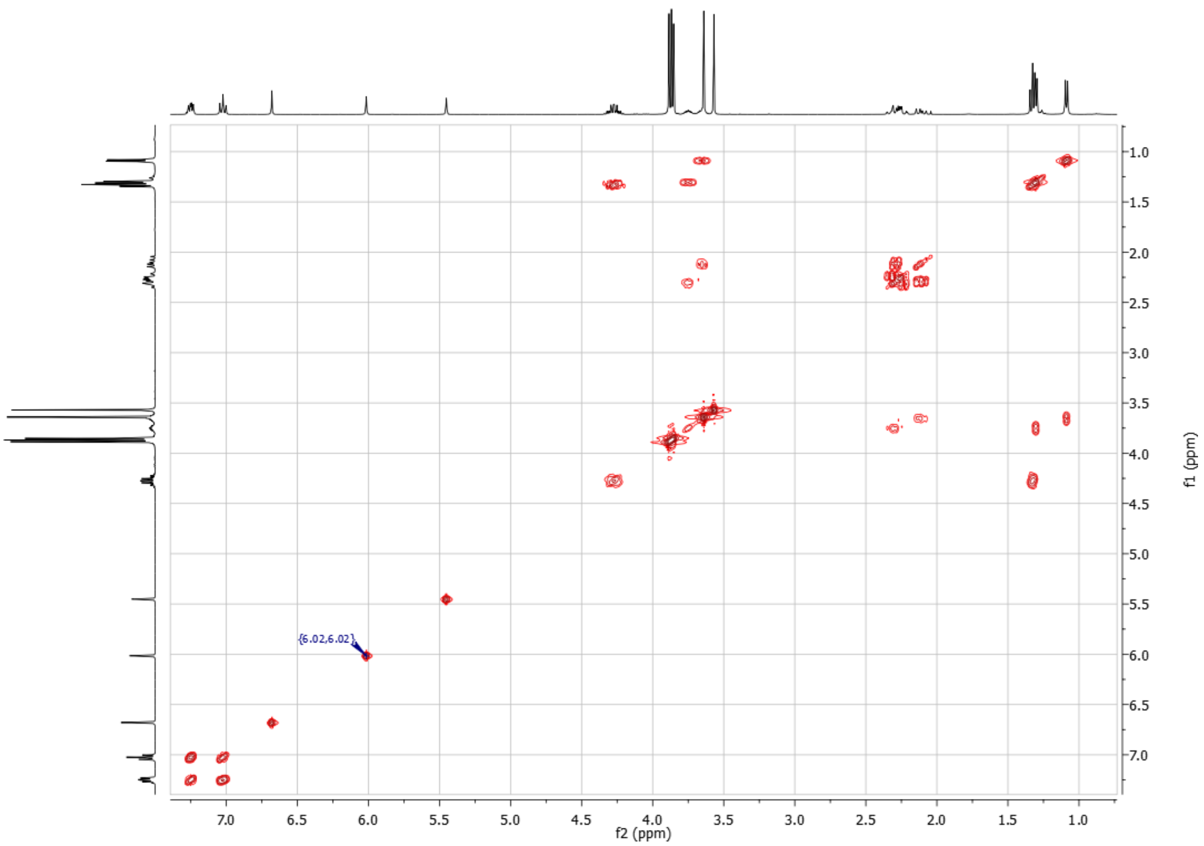

Figure S102. ^{1}H-^{1}H COSY NMR (400 MHz) spectrum of (a*R*,1*S*,3*S*,1'*S*,3'*S*)-**22** in $CDCl_3$.

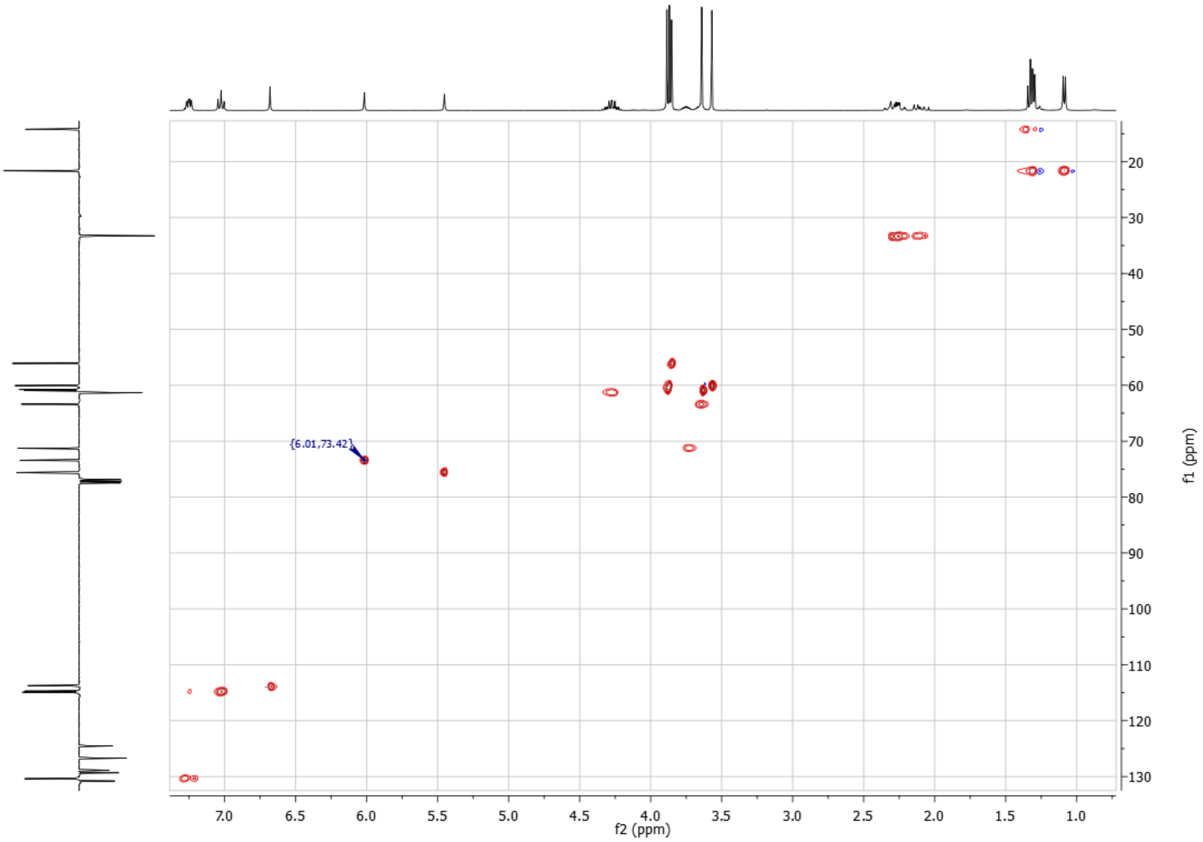

Figure S103. ^{1}H-^{13}C HSQC NMR (400 MHz) spectrum of (a*R*,1*S*,3*S*,1'*S*,3'*S*)-**22** in $CDCl_3$.

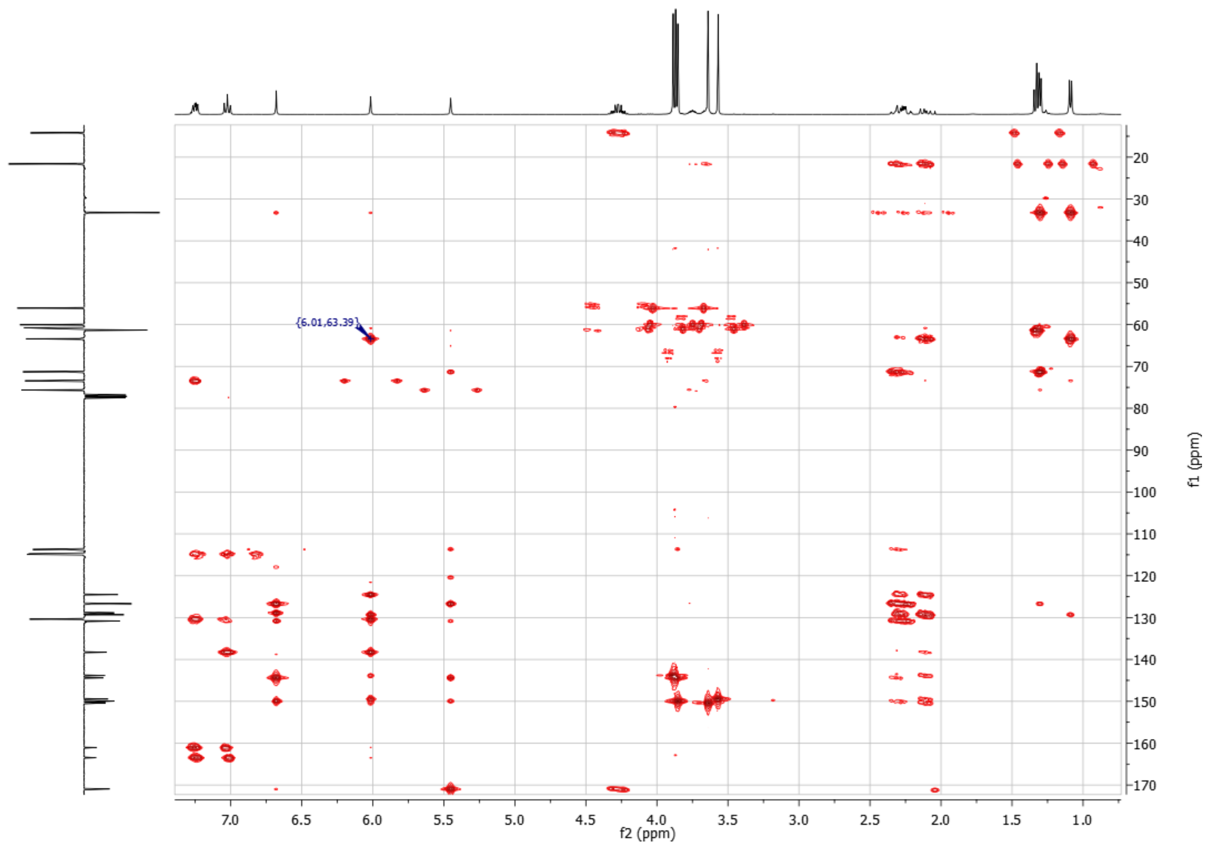

Figure S104. ^{1}H-^{13}C HMBC NMR (400 MHz) spectrum of (a*R*,1*S*,3*S*,1'*S*,3'*S*)-**22** in $CDCl_3$.

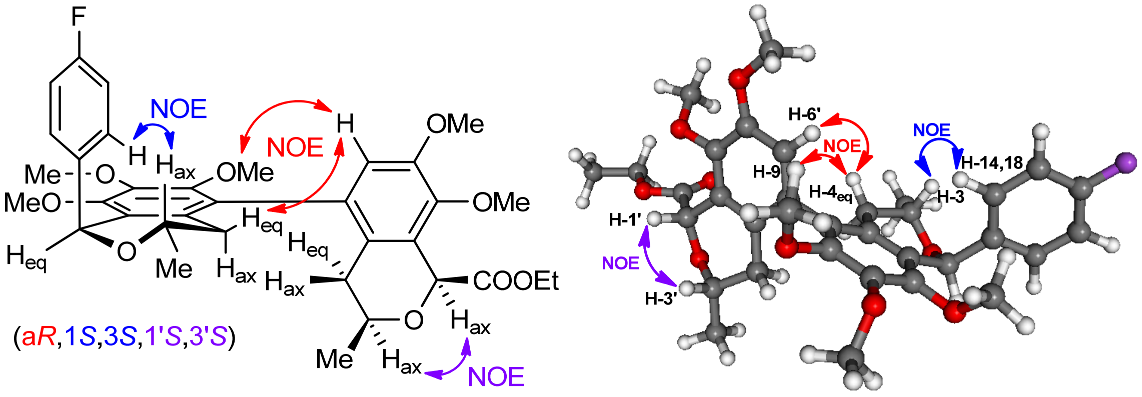

Figure S105. Characteristic NOE correlations shown on the lowest-energy computed conformer of (a*R*,1*S*,3*S*,1'*S*,3'*S*)-**22** suggesting (1*S*,3*S*,1'*S*,3'*S*) configurations of isochroman subunits and (a*R*) axial chirality.

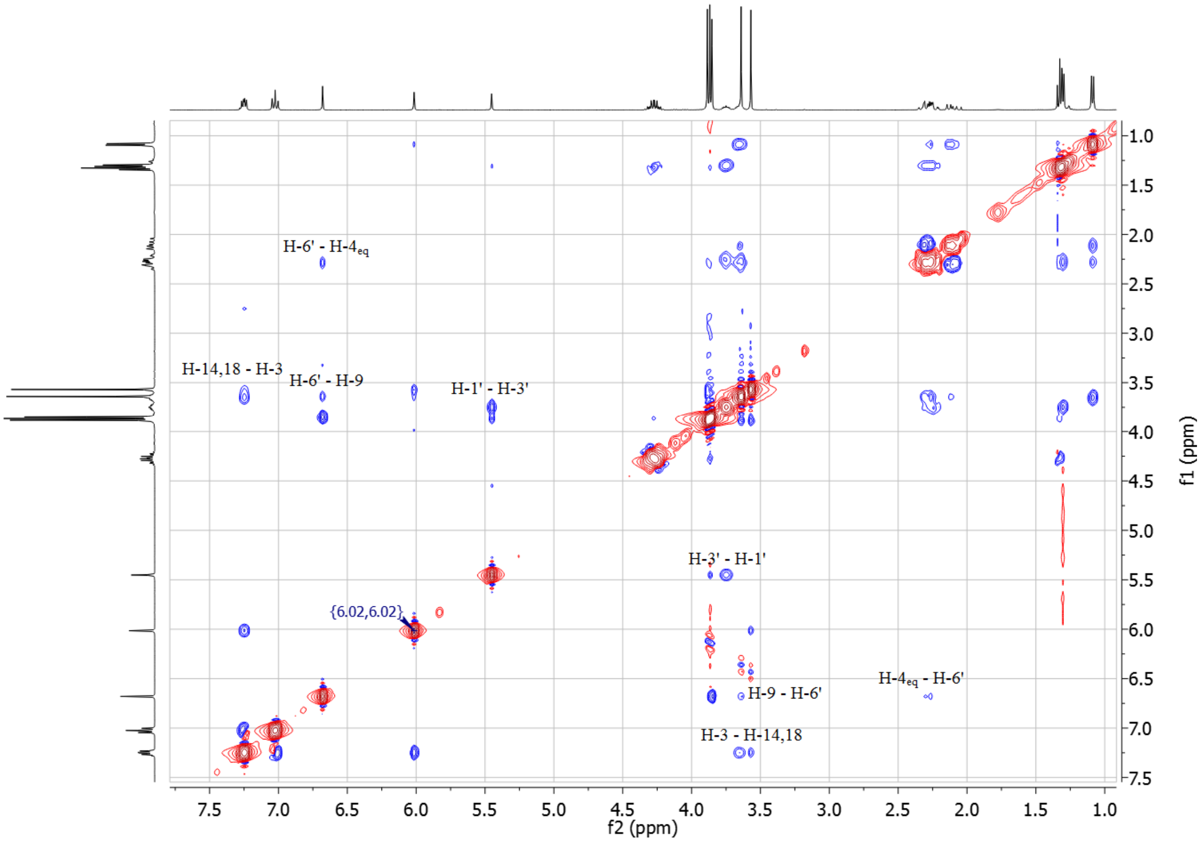

Figure S106. ^{1}H-^{1}H NOESY NMR (400 MHz) spectrum of (a*R*,1*S*,3*S*,1'*S*,3'*S*)-**22** in $CDCl_3$.

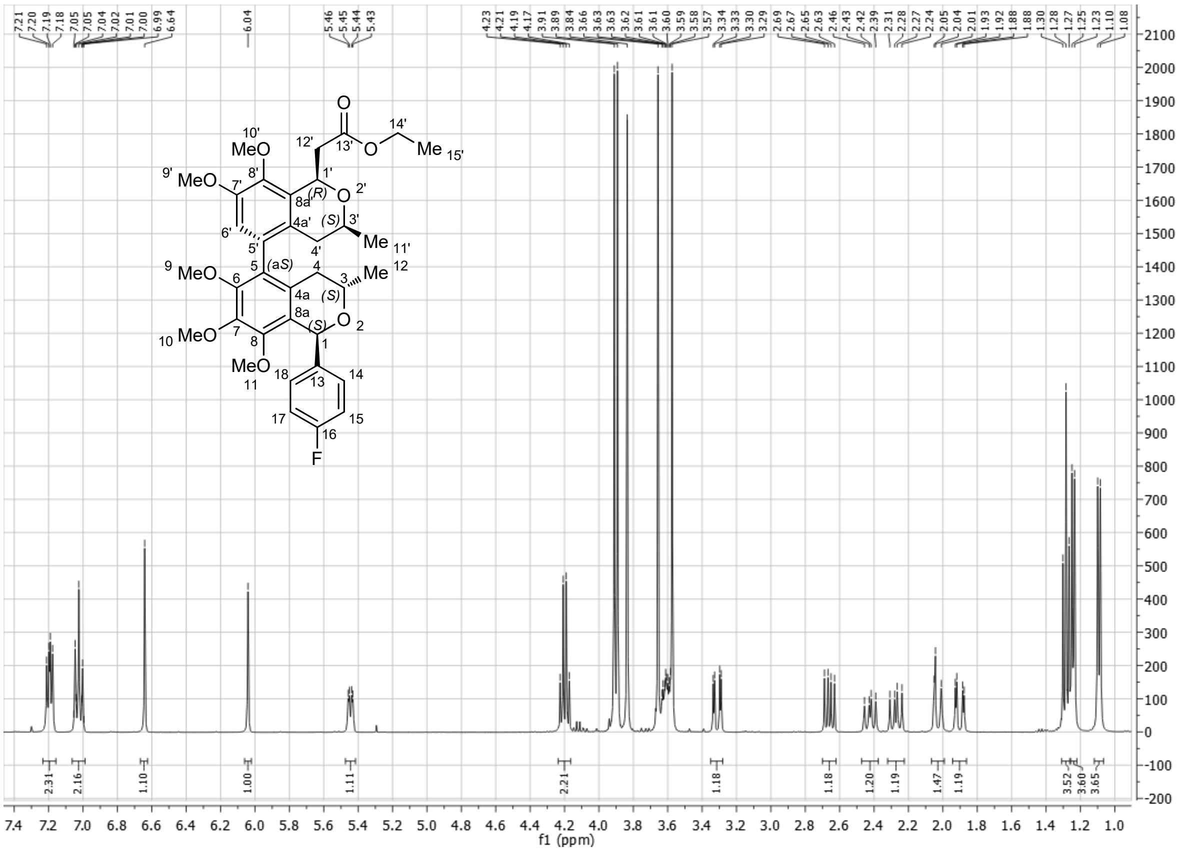

Figure S107. ^{1}H NMR (400 MHz) spectrum of (a*S*,1*S*,3*S*,1'*R*,3'*S*)-**23** in $CDCl_3$.

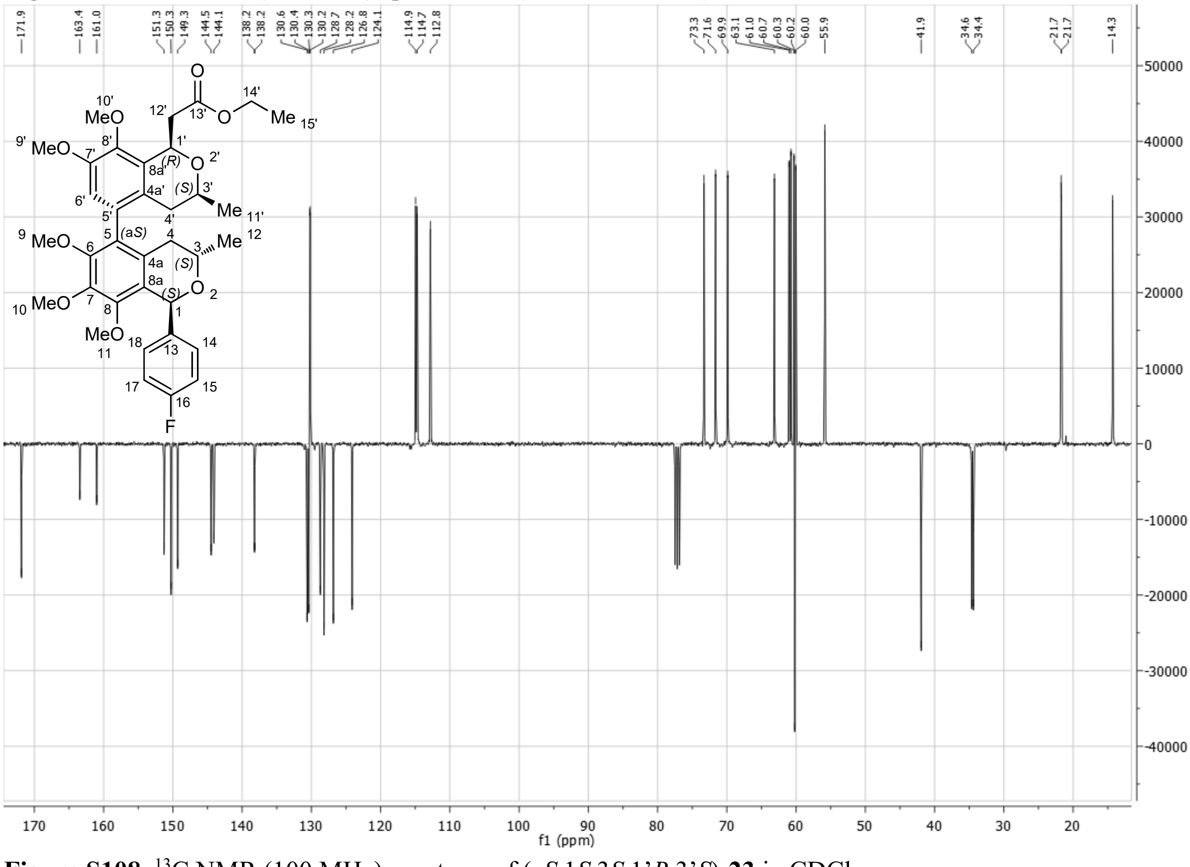

Figure S108. ^{13}C NMR (100 MHz) spectrum of (a*S*,1*S*,3*S*,1'*R*,3'*S*)-**23** in $CDCl_3$.

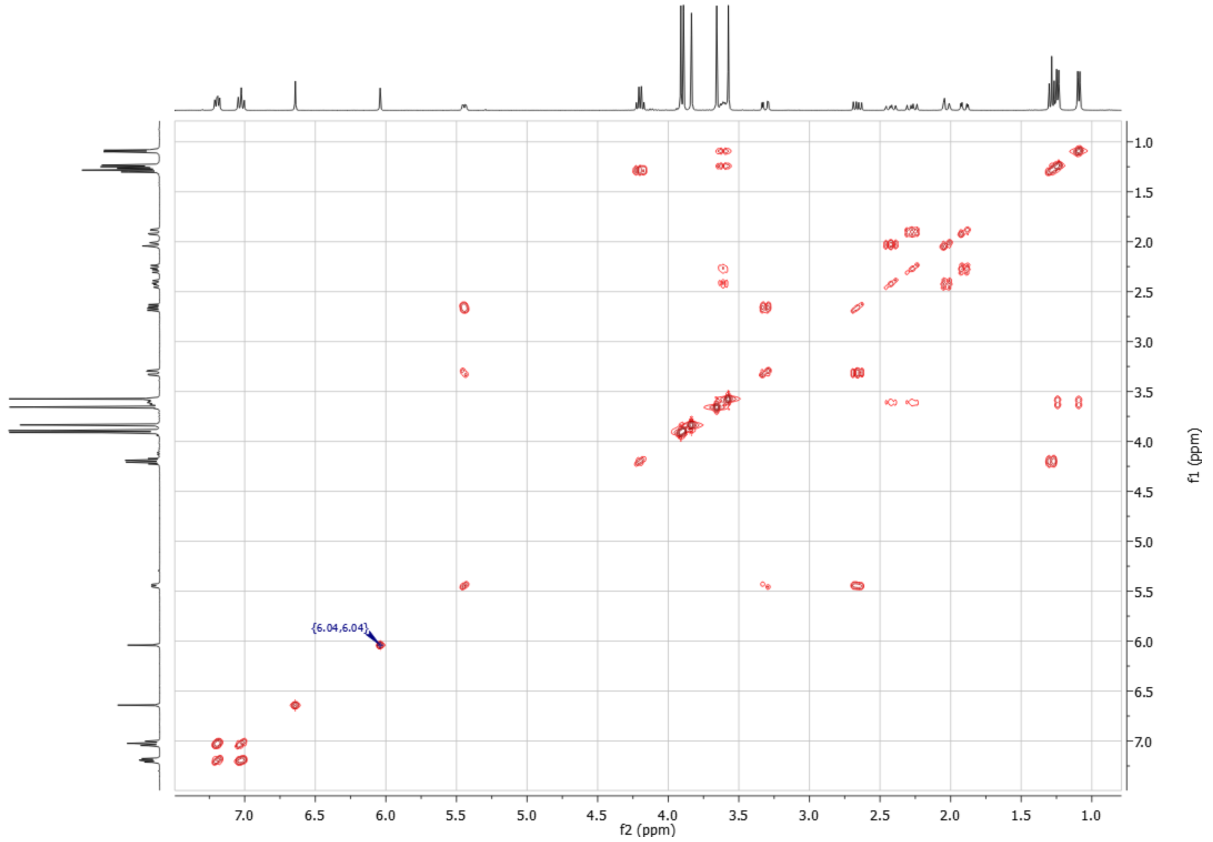

Figure S109. ^{1}H-^{1}H COSY NMR (400 MHz) spectrum of (a*S*,1*S*,3*S*,1'*R*,3'*S*)-**23** in $CDCl_3$.

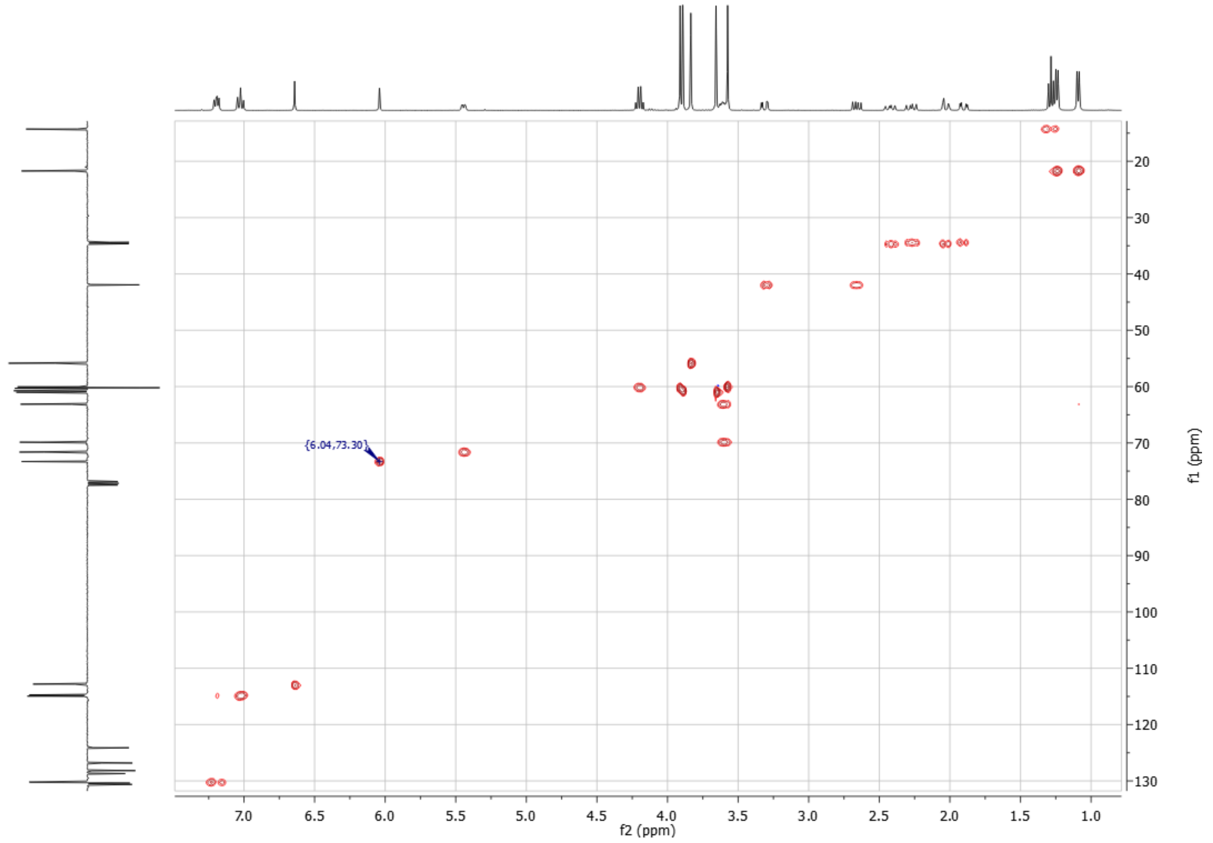

Figure S110. ^{1}H-^{13}C HSQC NMR (400 MHz) spectrum of (a*S*,1*S*,3*S*,1'*R*,3'*S*)-**23** in $CDCl_3$.

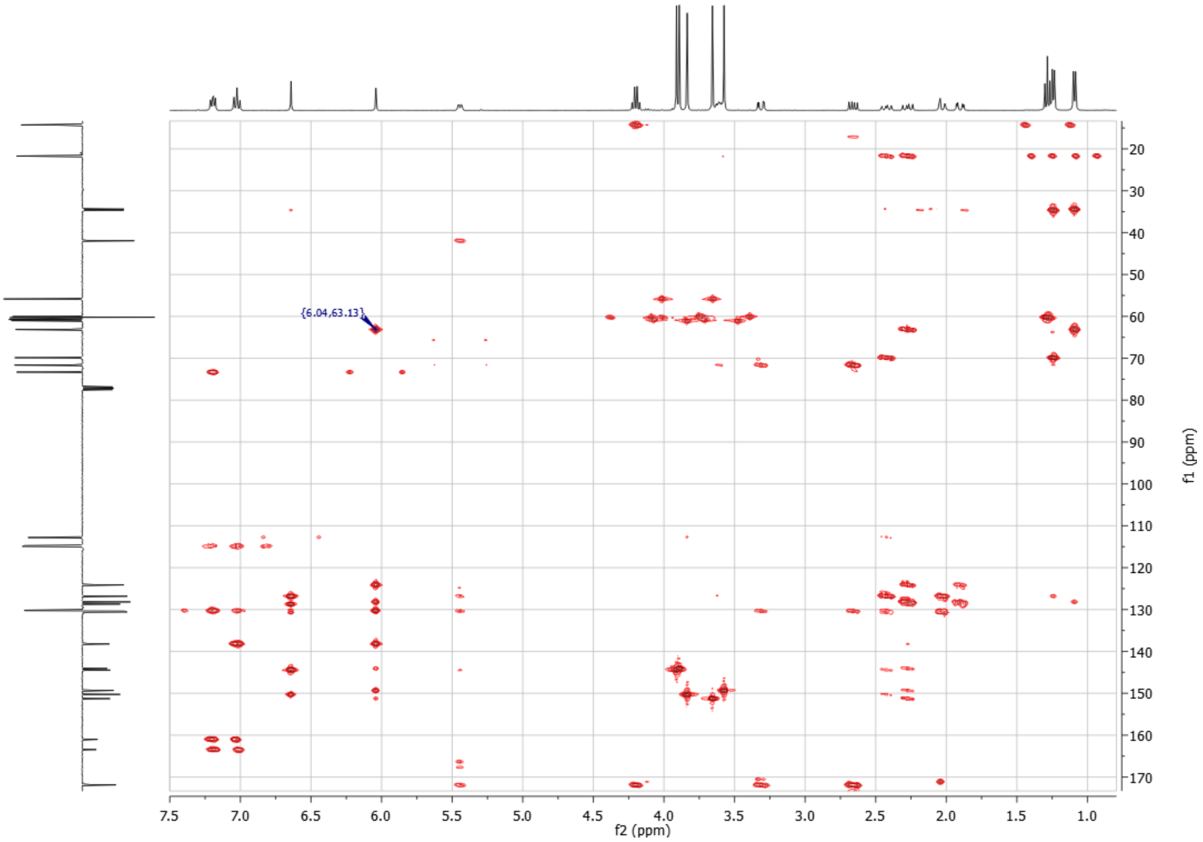

Figure S111. ^{1}H-^{13}C HMBC NMR (400 MHz) spectrum of (a*S*,1*S*,3*S*,1'*R*,3'*S*)-**23** in $CDCl_3$.

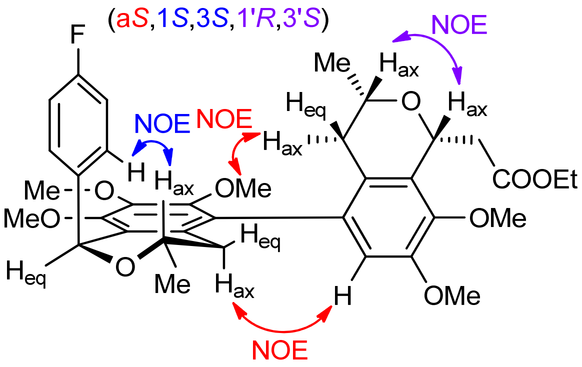

Figure S112. Characteristic NOE correlations shown on the structure of (a*S*,1*S*,3*S*,1'*R*,3'*S*)-**23** suggesting (1*S*,3*S*,1'*R*,3'*S*) configurations of isochroman subunits and (a*S*) axial chirality.

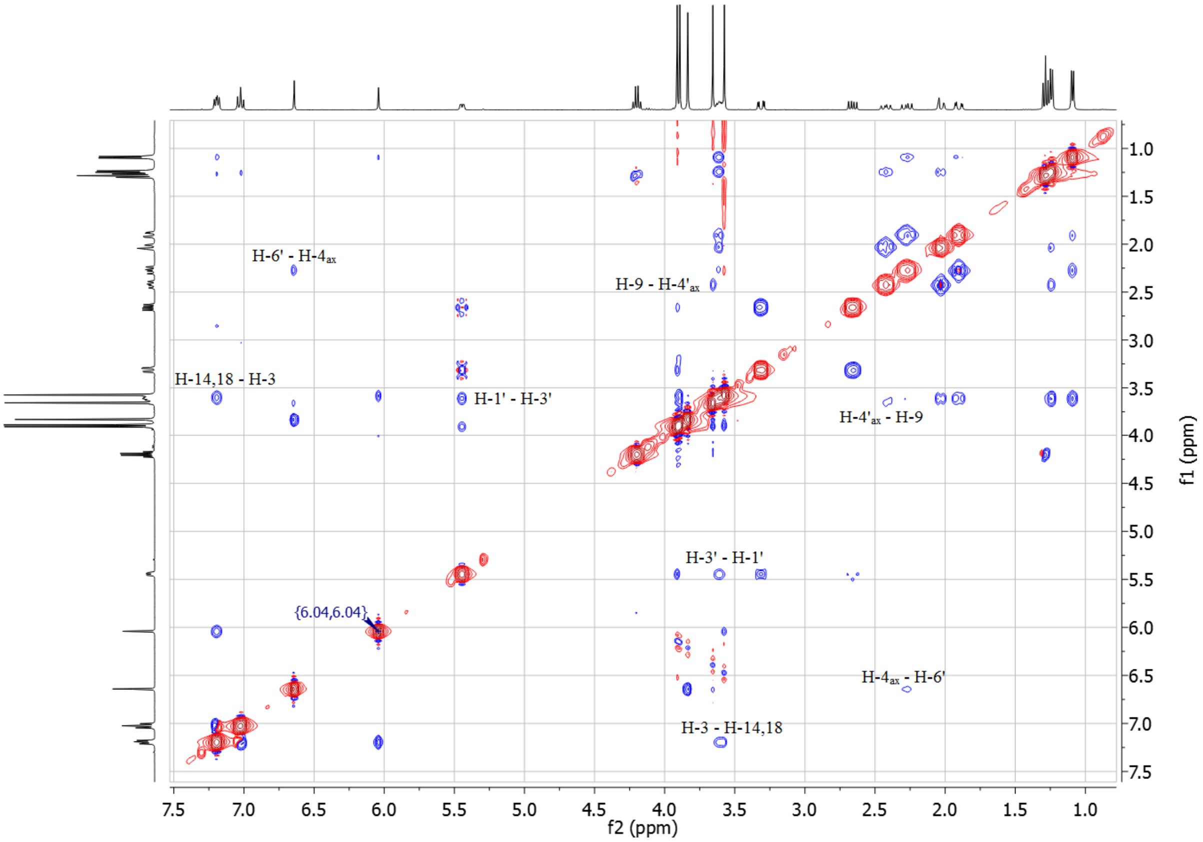

Figure S113. ^{1}H-^{1}H NOESY NMR (400 MHz) spectrum of (a*S*,1*S*,3*S*,1'*R*,3'*S*)-**23** in $CDCl_3$.

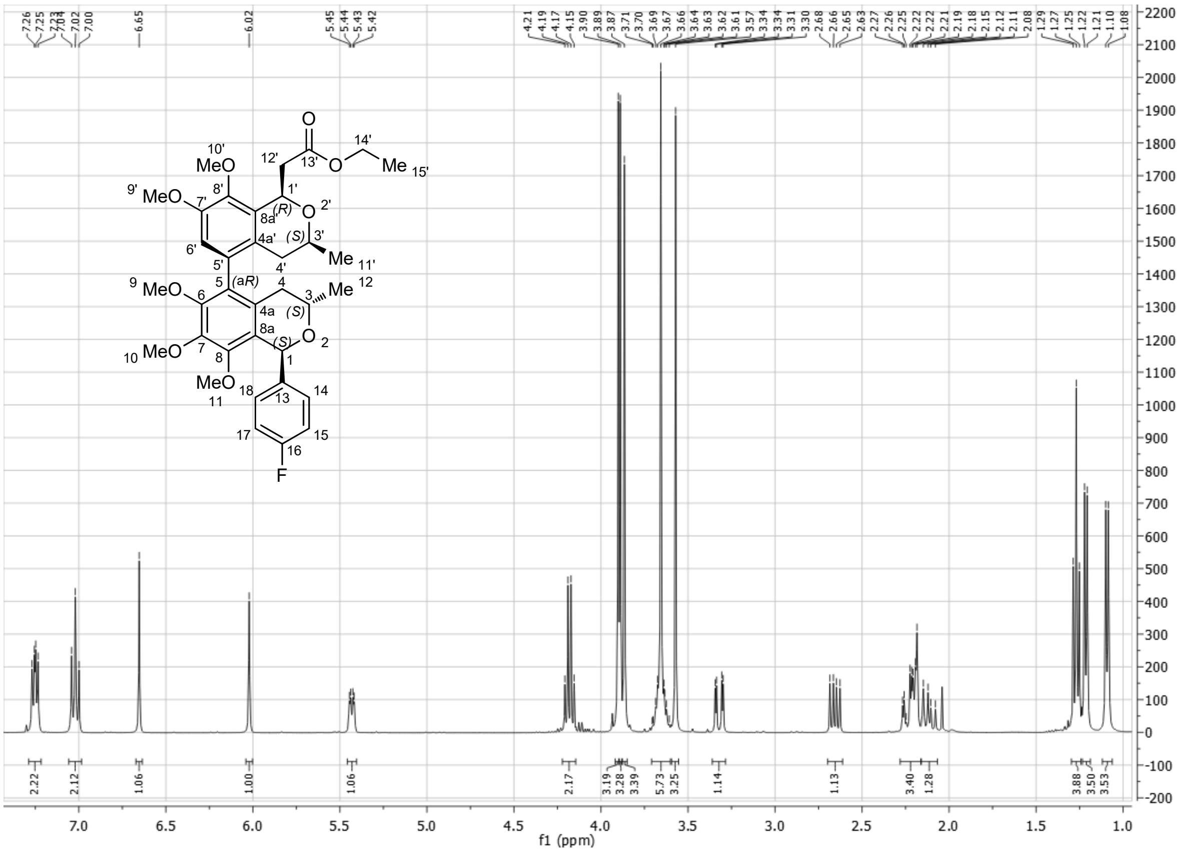

Figure S114. ^{1}H NMR (400 MHz) spectrum of (a*R*,1*S*,3*S*,1'*R*,3'*S*)-**23** in $CDCl_3$.

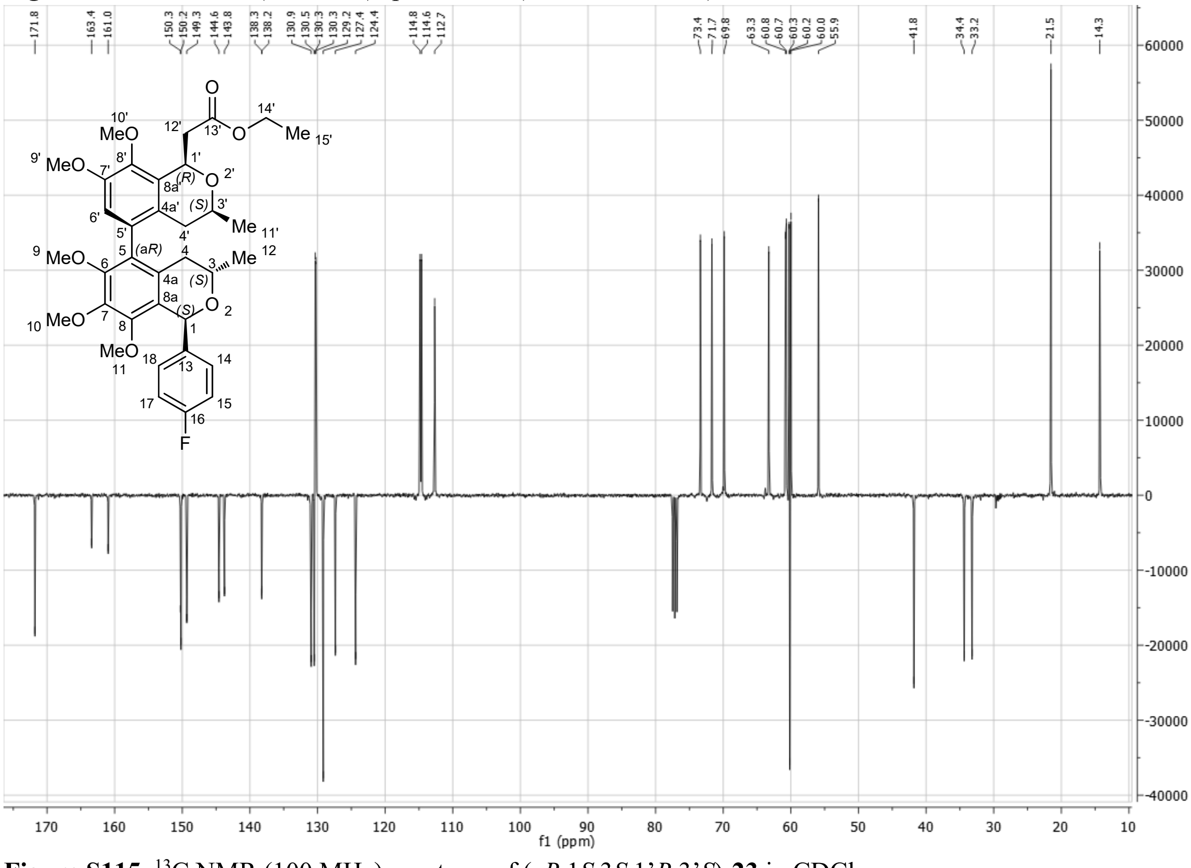

Figure S115. ^{13}C NMR (100 MHz) spectrum of (a*R*,1*S*,3*S*,1'*R*,3'*S*)-**23** in $CDCl_3$.

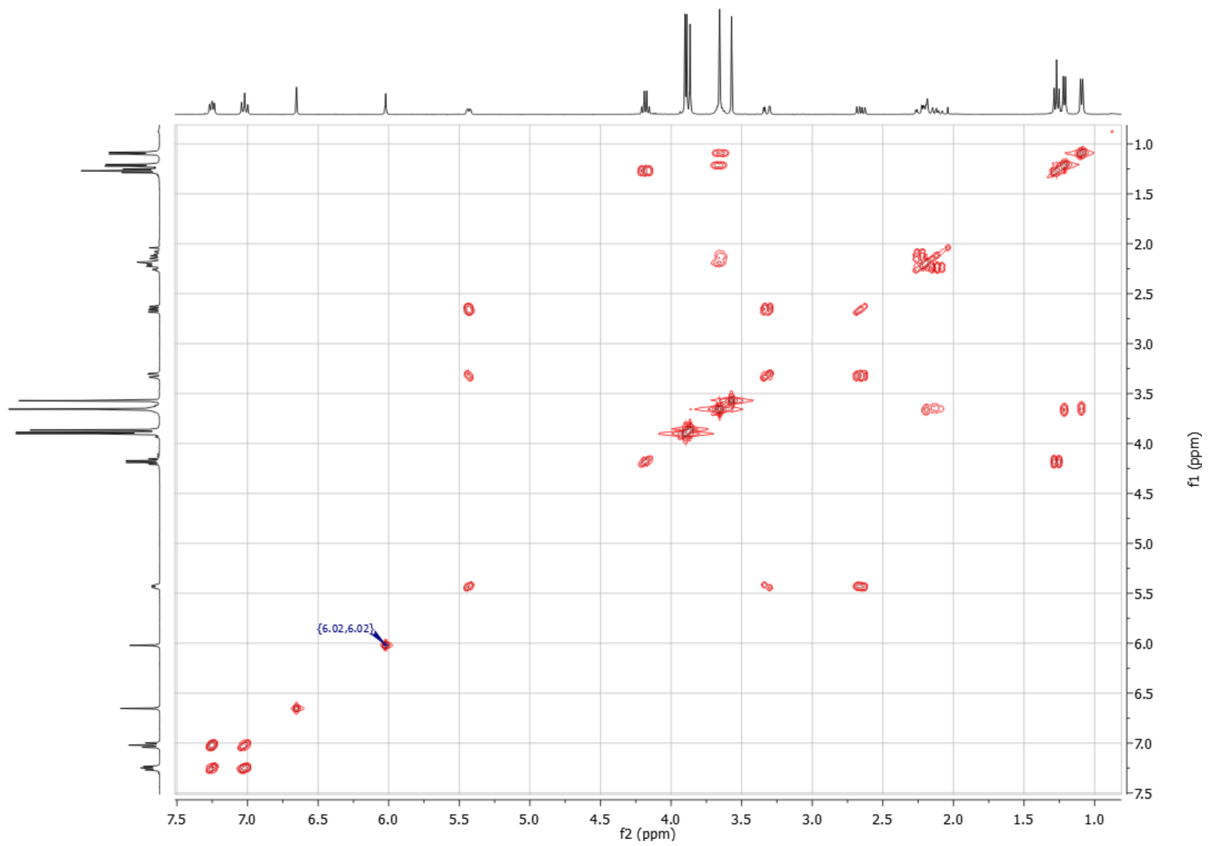

Figure S116. ^{1}H-^{1}H COSY NMR (400 MHz) spectrum of (a*R*,1*S*,3*S*,1'*R*,3'*S*)-**23** in $CDCl_3$.

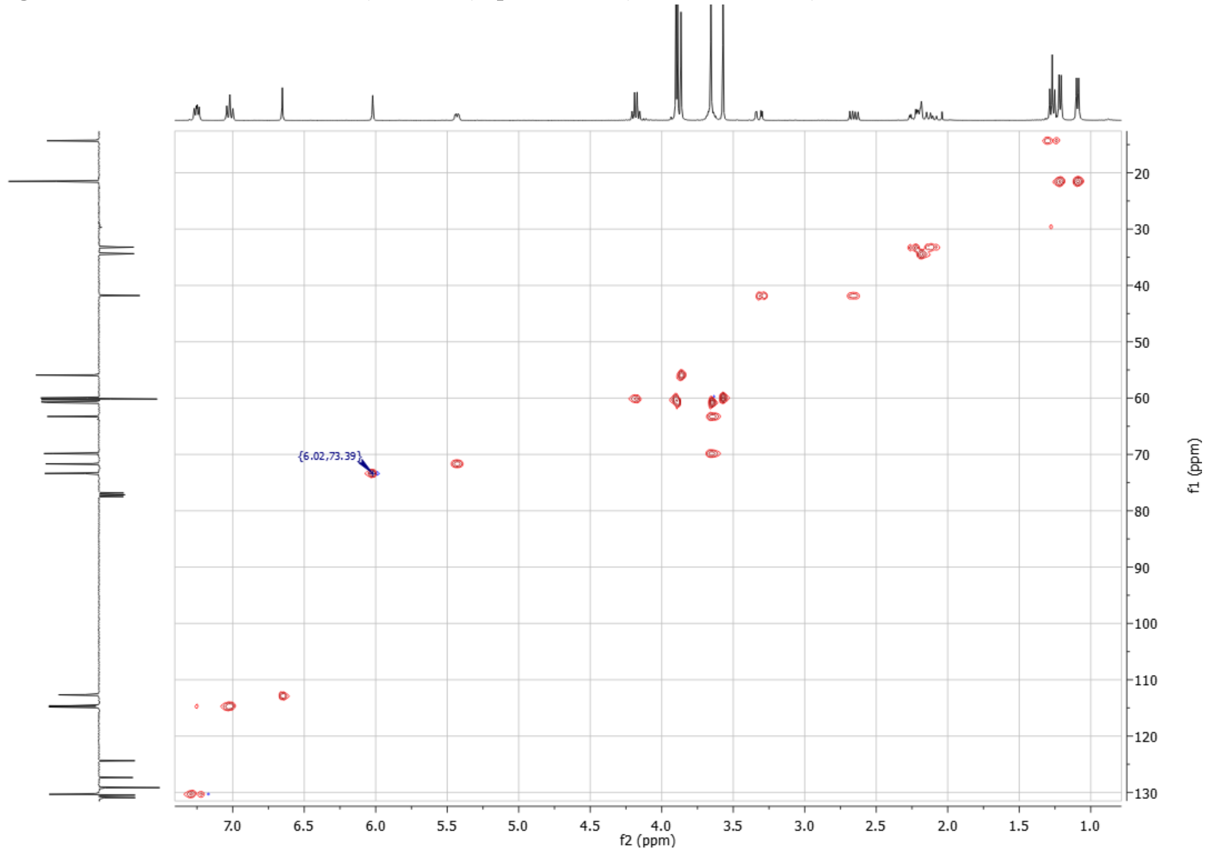

Figure S117. ^{1}H-^{13}C HSQC NMR (400 MHz) spectrum of (a*R*,1*S*,3*S*,1'*R*,3'*S*)-**23** in $CDCl_3$.

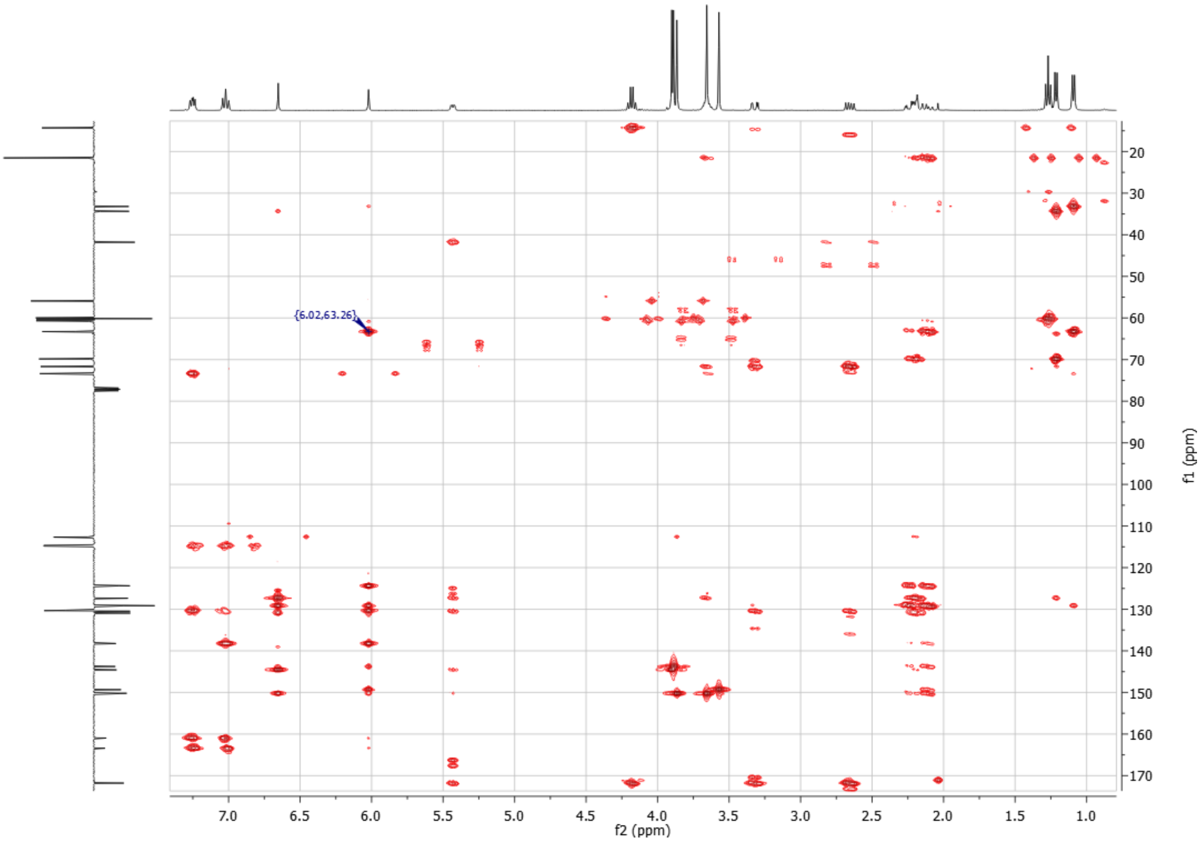

Figure S118. ^{1}H-^{13}C HMBC NMR (400 MHz) spectrum of (a*R*,1*S*,3*S*,1'*R*,3'*S*)-**23** in $CDCl_3$.

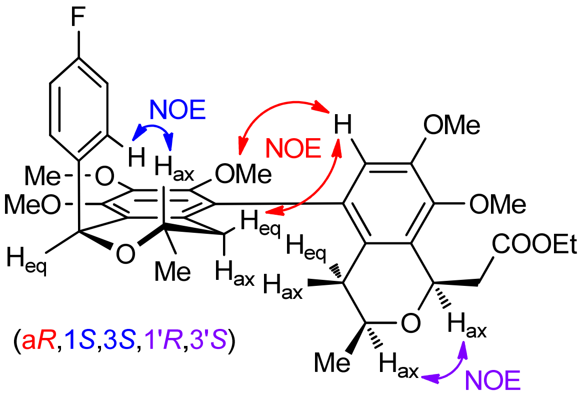

Figure S119. Characteristic NOE correlations shown on the structure of (a*R*,1*S*,3*S*,1'*R*,3'*S*)-**23** suggesting (1*S*,3*S*,1'*R*,3'*S*) configurations of isochroman subunits and (a*R*) axial chirality.

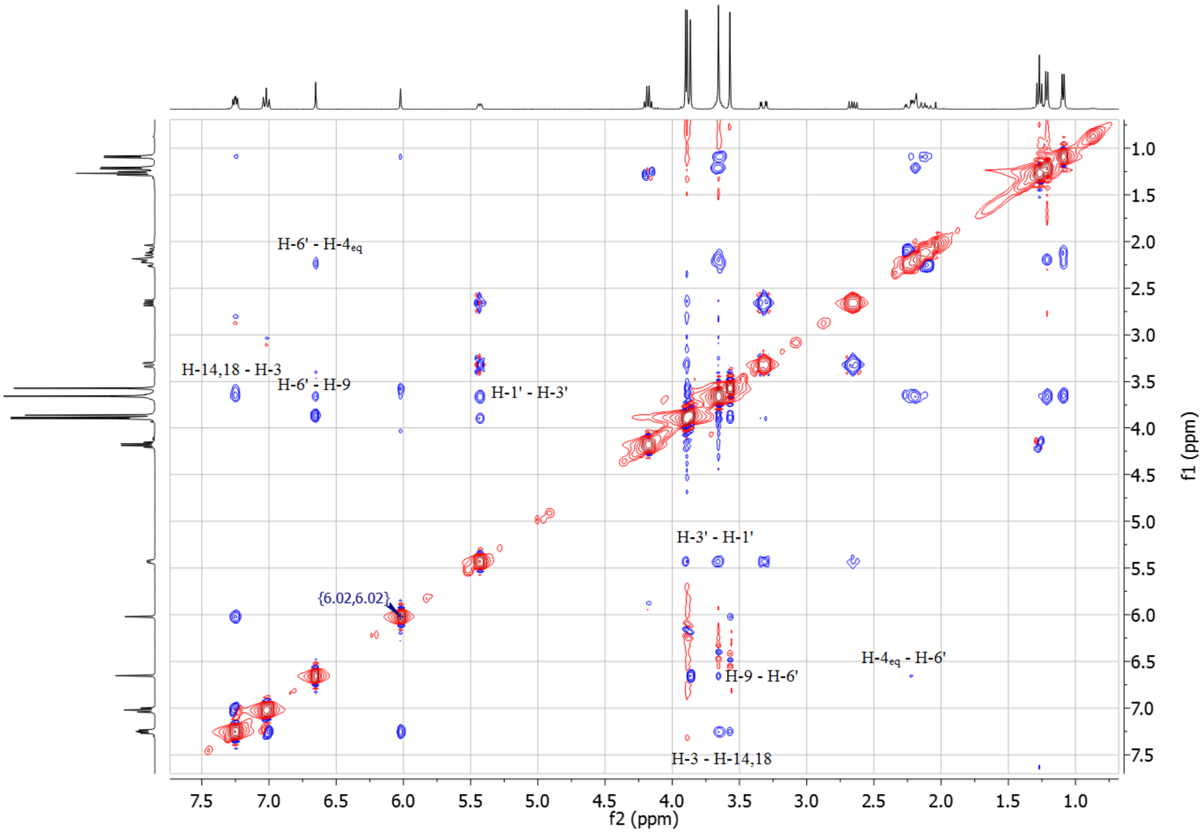

Figure S120. ^1H-^1H NOESY NMR (400 MHz) spectrum of (a*R*,1*S*,3*S*,1'*R*,3'*S*)-**23** in $CDCl_3$.

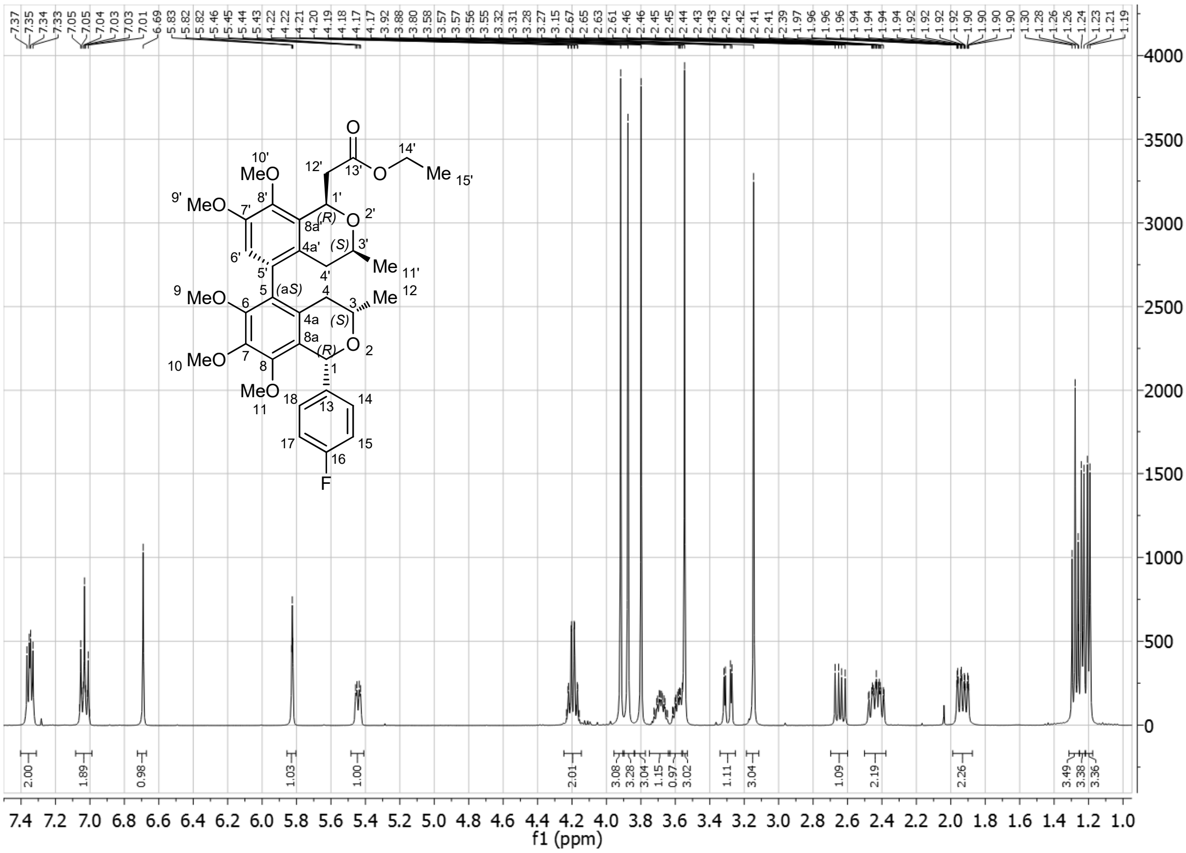

Figure S121. ^{1}H NMR (400 MHz) spectrum of (a*S*,1*R*,3*S*,1'*R*,3'*S*)-**23** in $CDCl_3$.

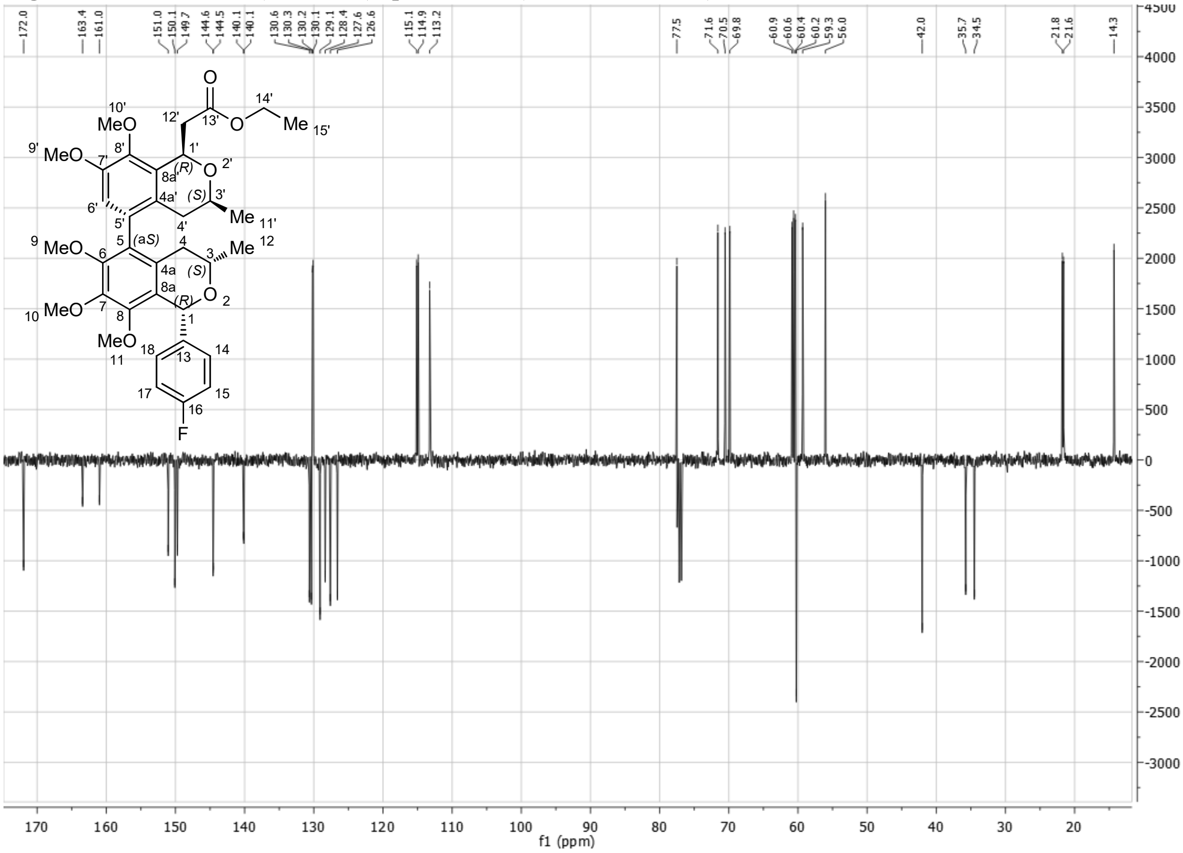

Figure S122. ^{13}C NMR (100 MHz) spectrum of (a*S*,1*R*,3*S*,1'*R*,3'*S*)-**23** in $CDCl_3$.

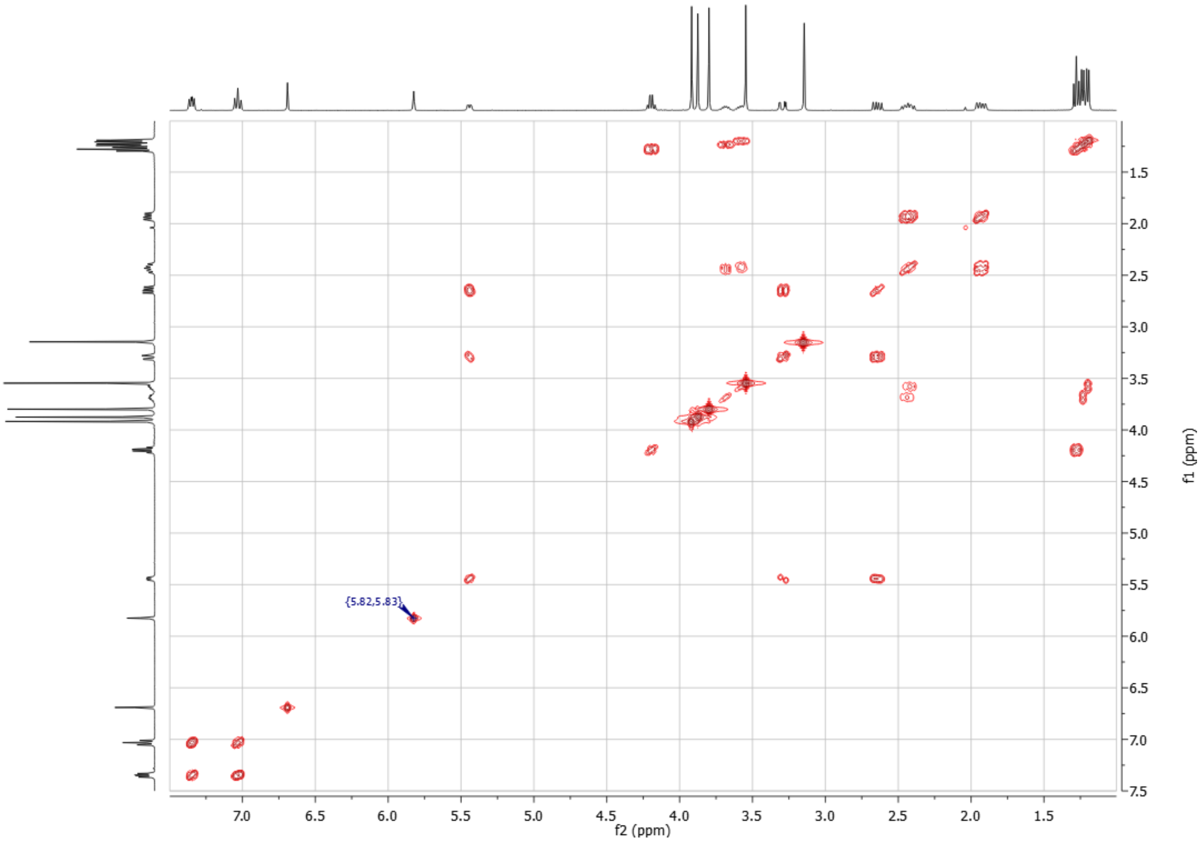

Figure S123. ^{1}H-^{1}H COSY NMR (400 MHz) spectrum of (a*S*,1*R*,3*S*,1'*R*,3'*S*)-**23** in $CDCl_3$.

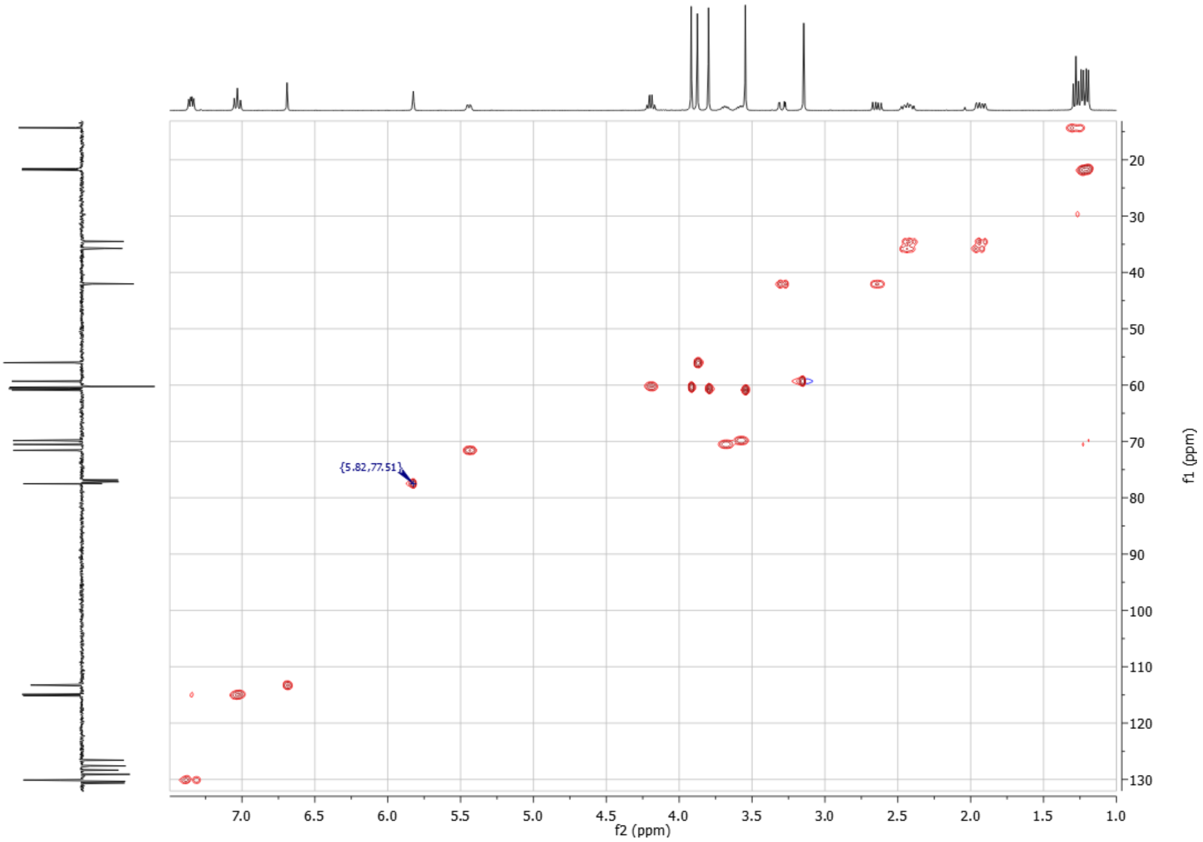

Figure S124. ^{1}H-^{13}C HSQC NMR (400 MHz) spectrum of (a*S*,1*R*,3*S*,1'*R*,3'*S*)-**23** in $CDCl_3$.

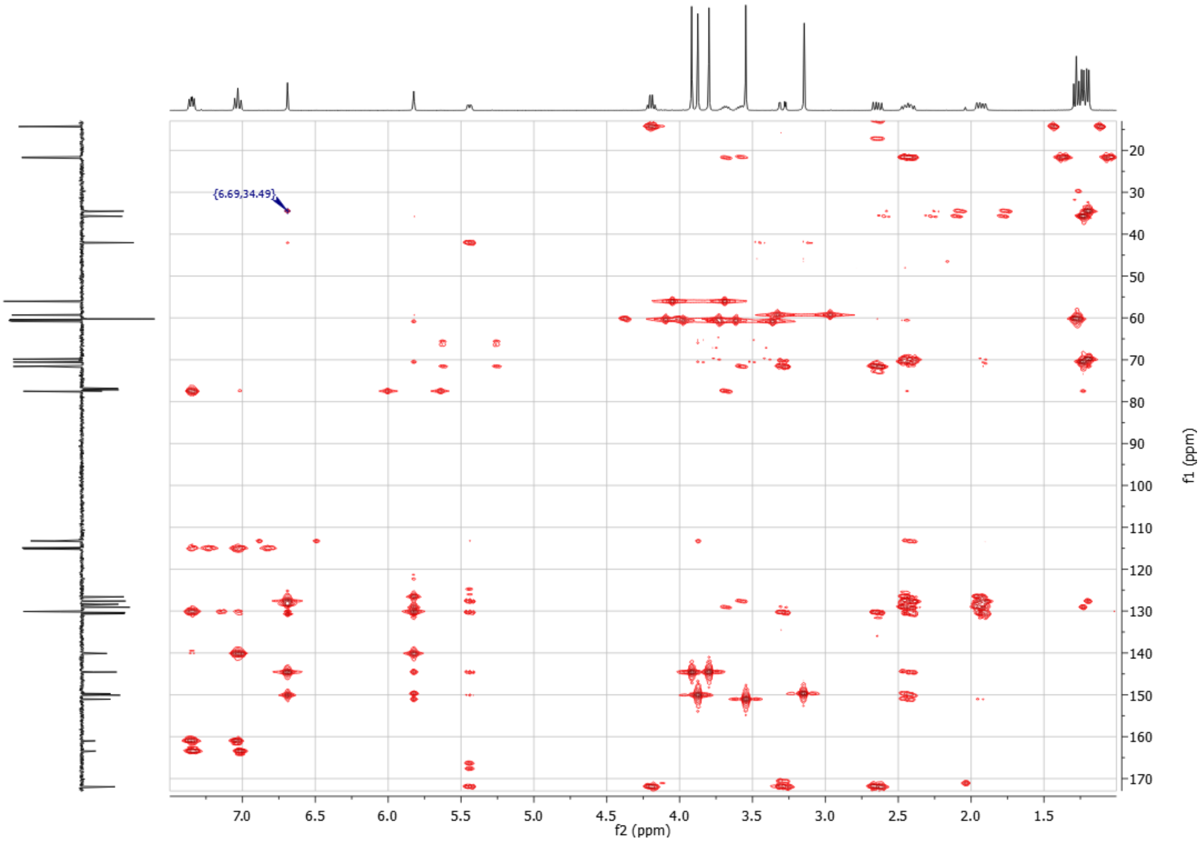

Figure S125. ^{1}H-^{13}C HMBC NMR (400 MHz) spectrum of (a*S*,1*R*,3*S*,1'*R*,3'*S*)-**23** in $CDCl_3$.

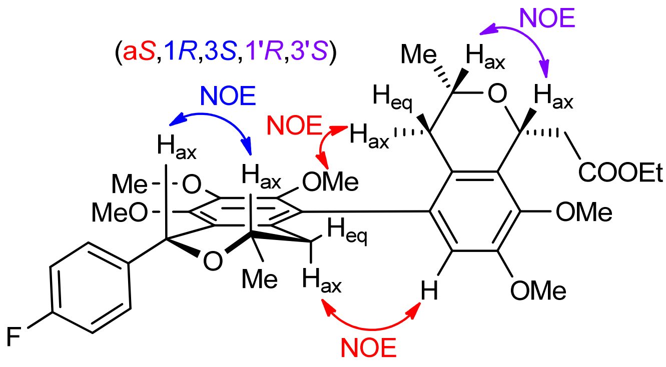

Figure S126. Characteristic NOE correlations shown on the structure of (a*S*,1*R*,3*S*,1'*R*,3'*S*)-**23** suggesting (1*R*,3*S*,1'*R*,3'*S*) configurations of isochroman subunits and (a*S*) axial chirality.

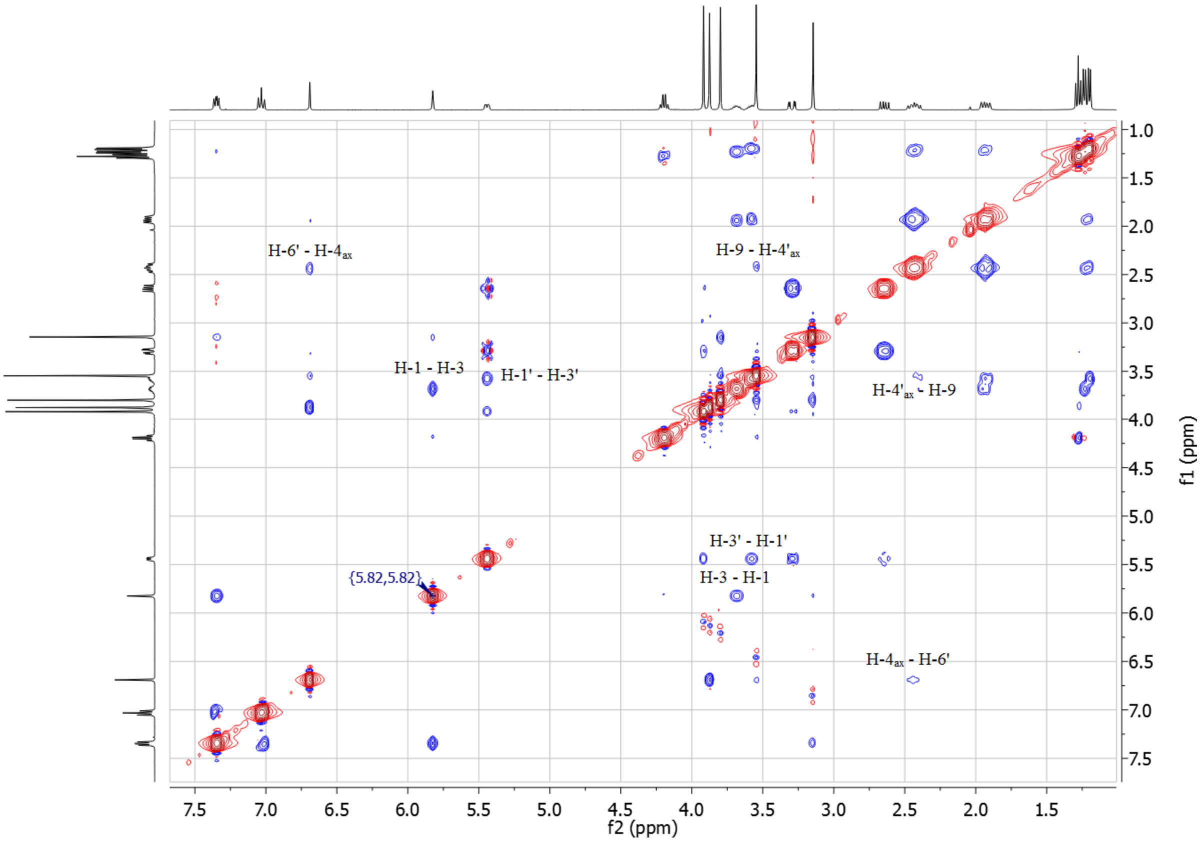

Figure S127. ^{1}H-^{1}H NOESY NMR (400 MHz) spectrum of (a*S*,1*R*,3*S*,1'*R*,3'*S*)-**23** in $CDCl_3$.

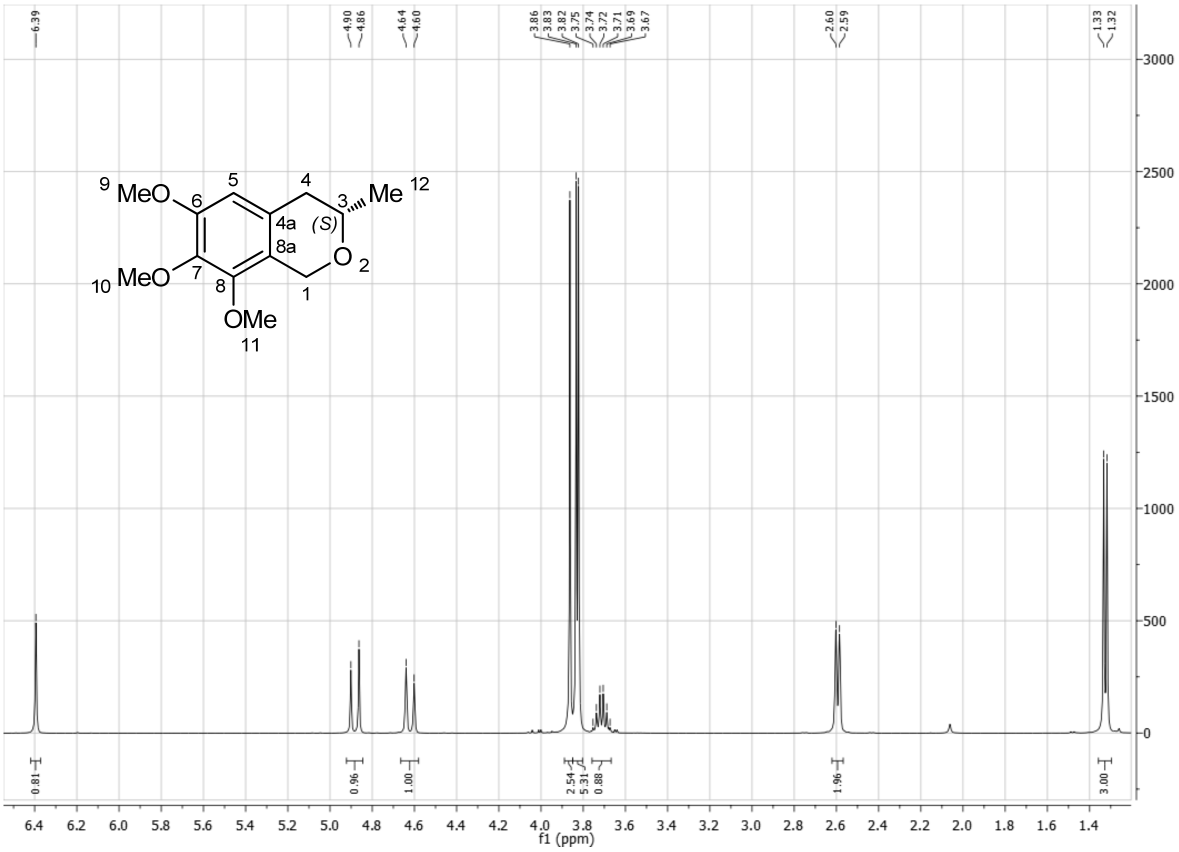

Figure S128. ^{1}H NMR (400 MHz) spectrum of (*S*)-**24** in $CDCl_3$.

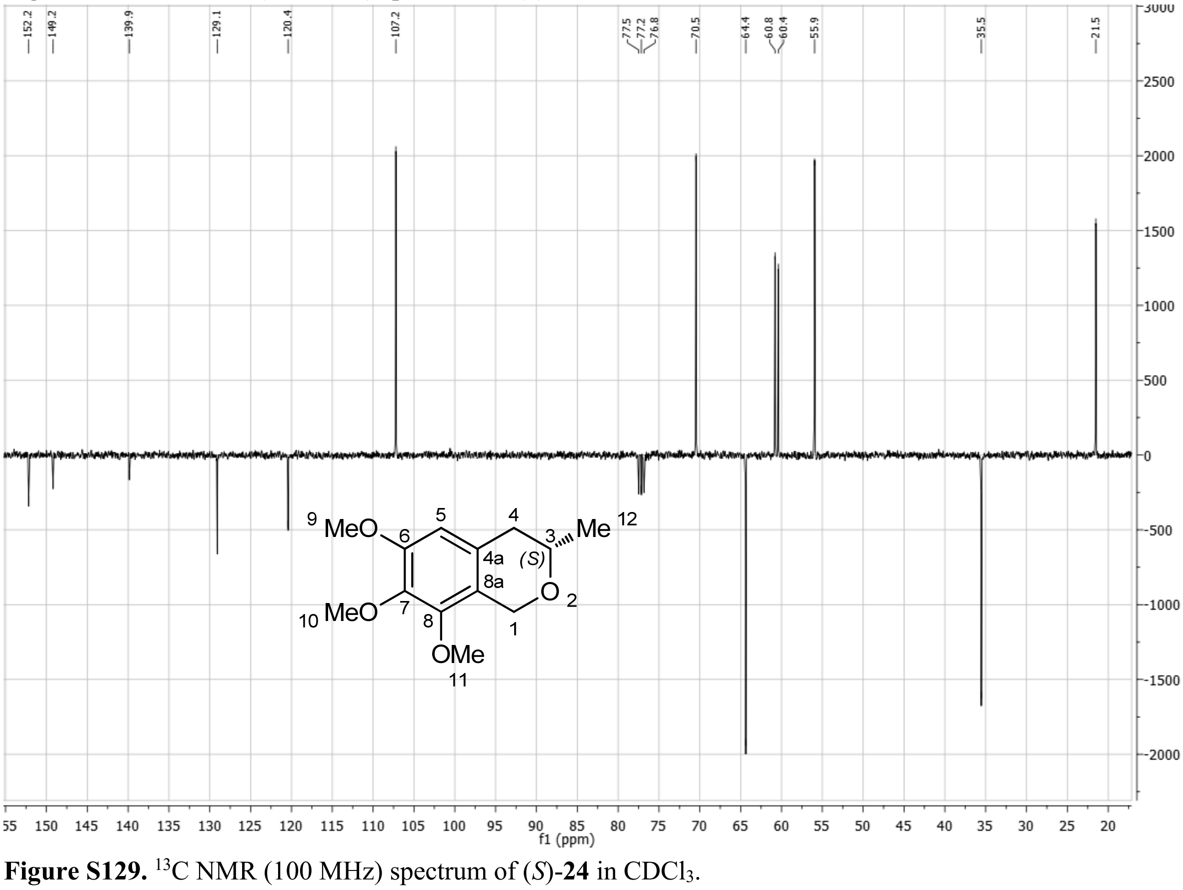

Figure S129. ^{13}C NMR (100 MHz) spectrum of (*S*)-**24** in $CDCl_3$.

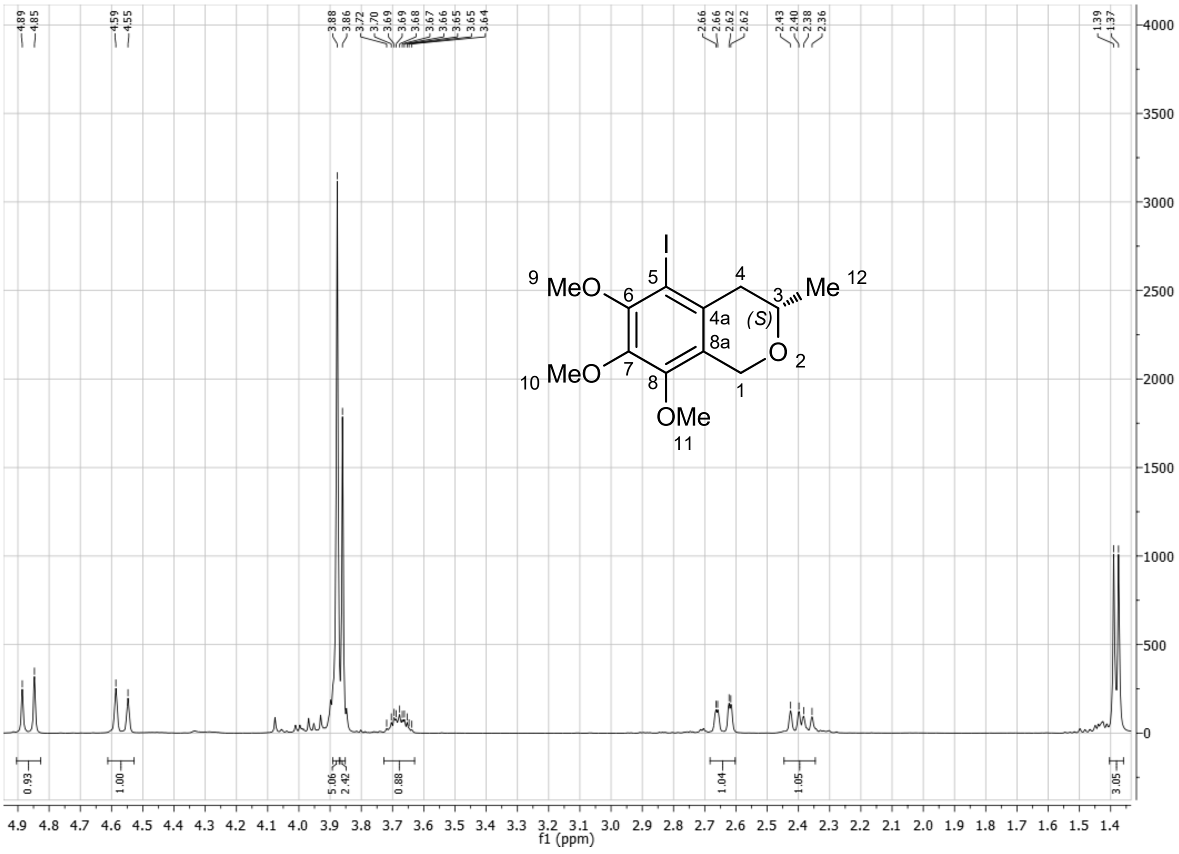

Figure S130. ^{1}H NMR (400 MHz) spectrum of (*S*)-**25** in $CDCl_3$.

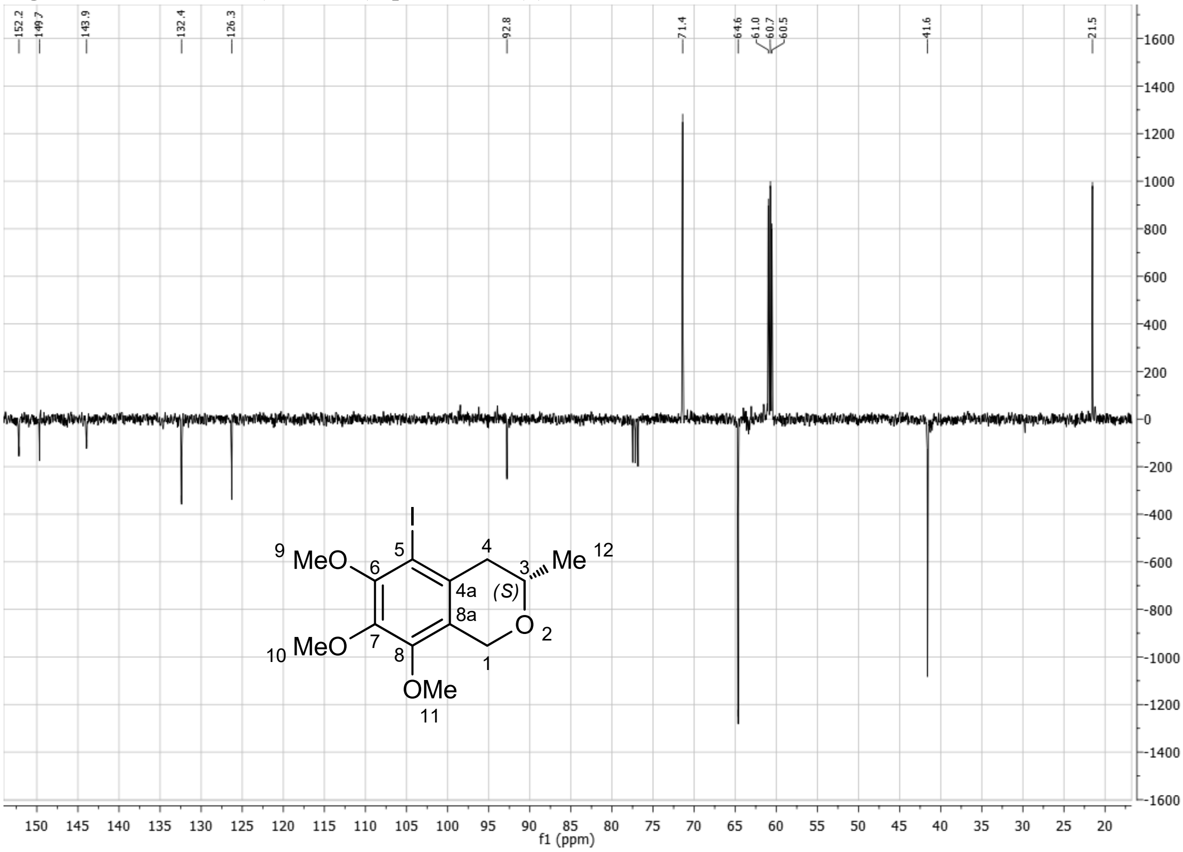

Figure S131. ^{13}C NMR (100 MHz) spectrum of (*S*)-**25** in $CDCl_3$.

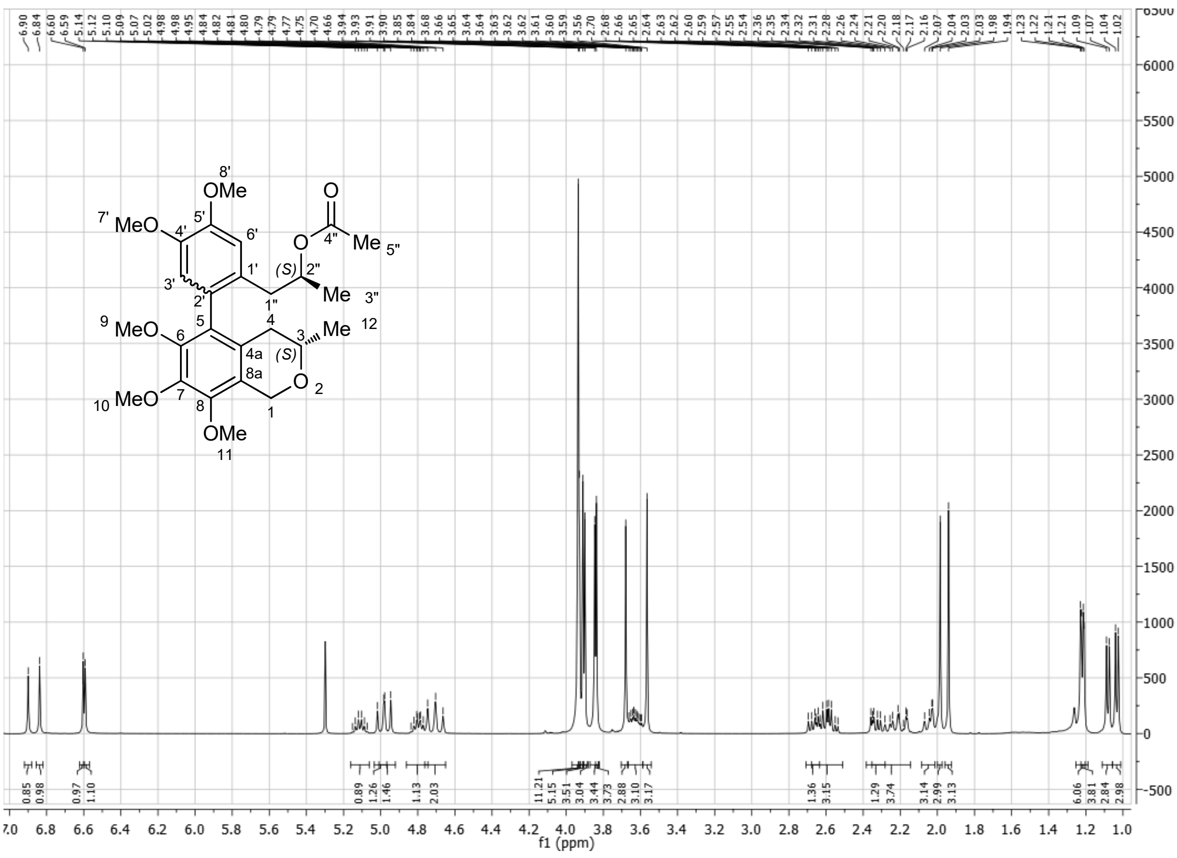

Figure S132. ^{1}H NMR (400 MHz) spectrum of the mixture of (a*R*,3*S*,2'*S*)-**26** and (a*S*,3*S*,2'*S*)-**26** in $CDCl_3$.

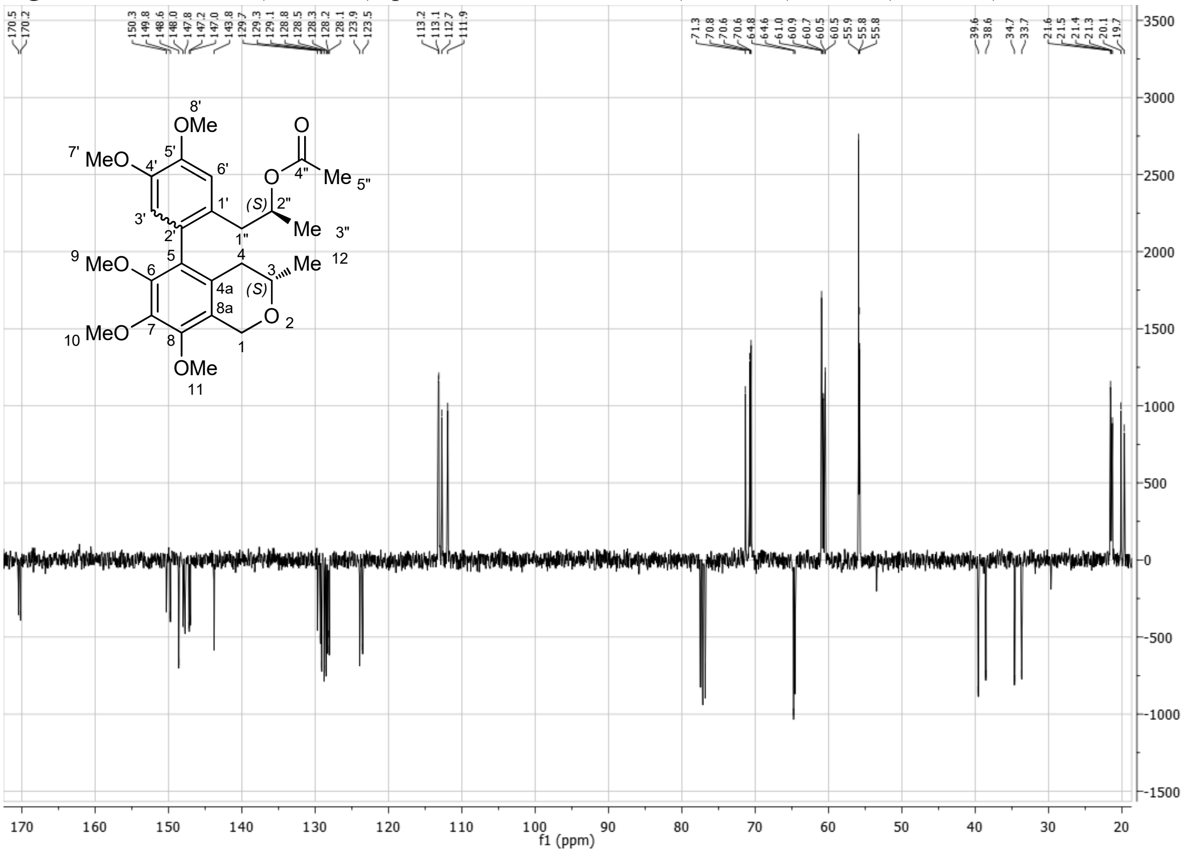

Figure S133. ^{13}C NMR (100 MHz) spectrum of the mixture of (a*R*,3*S*,2'*S*)-**26** and (a*S*,3*S*,2'*S*)-**26** in $CDCl_3$.

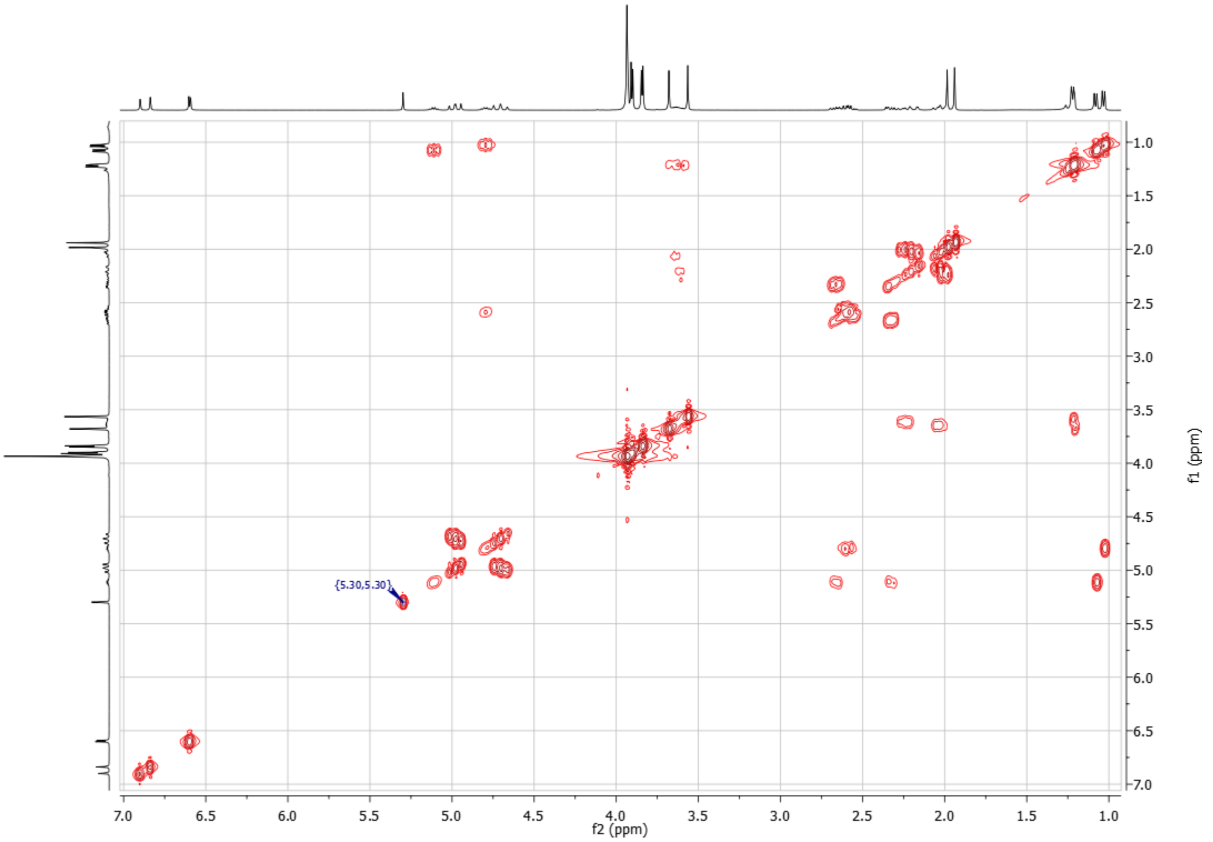

Figure S134. ^{1}H-^{1}H COSY NMR (400 MHz) spectrum of the mixture of (a*R*,3*S*,2'*S*)-**26** and (a*S*,3*S*,2'*S*)-**26** in $CDCl_3$.

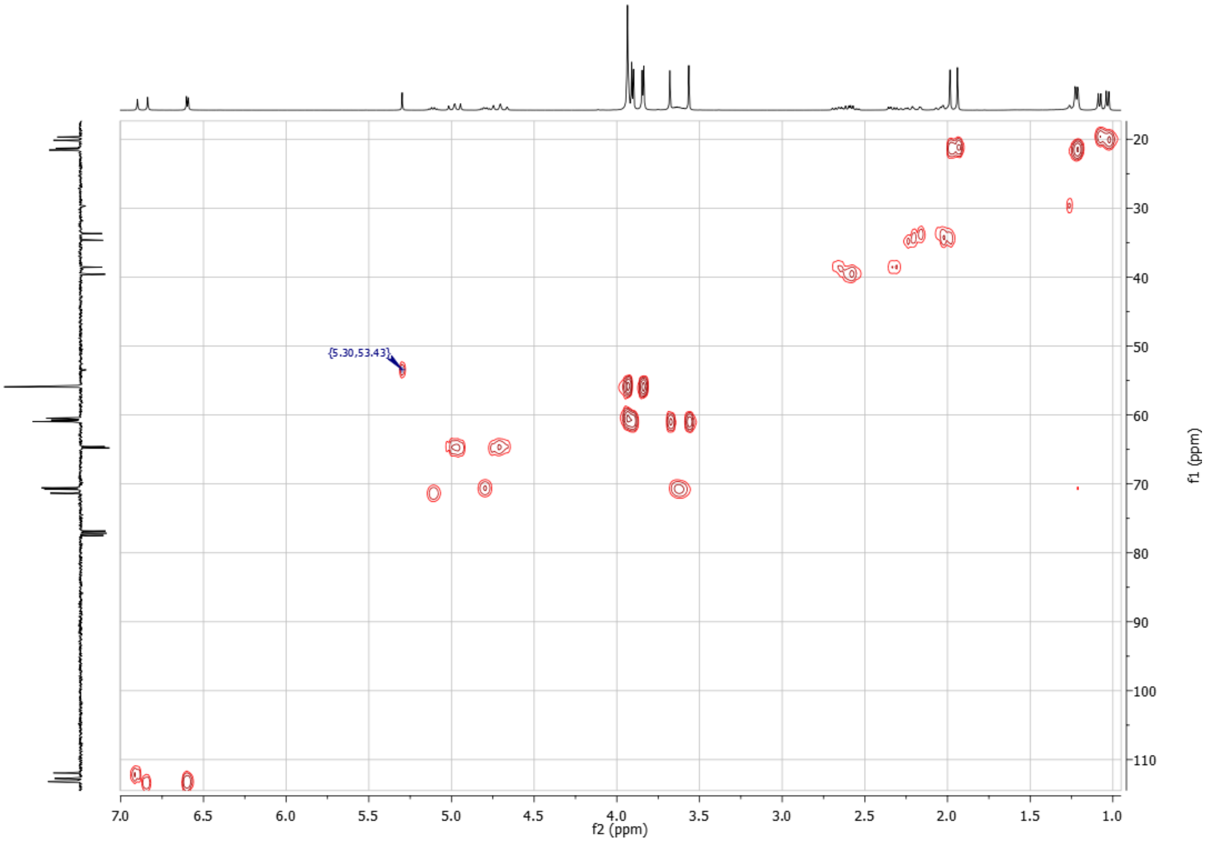

Figure S135. ^{1}H-^{13}C HSQC NMR (400 MHz) spectrum of the mixture of (a*R*,3*S*,2'*S*)-**26** and (a*S*,3*S*,2'*S*)-**26** in $CDCl_3$.

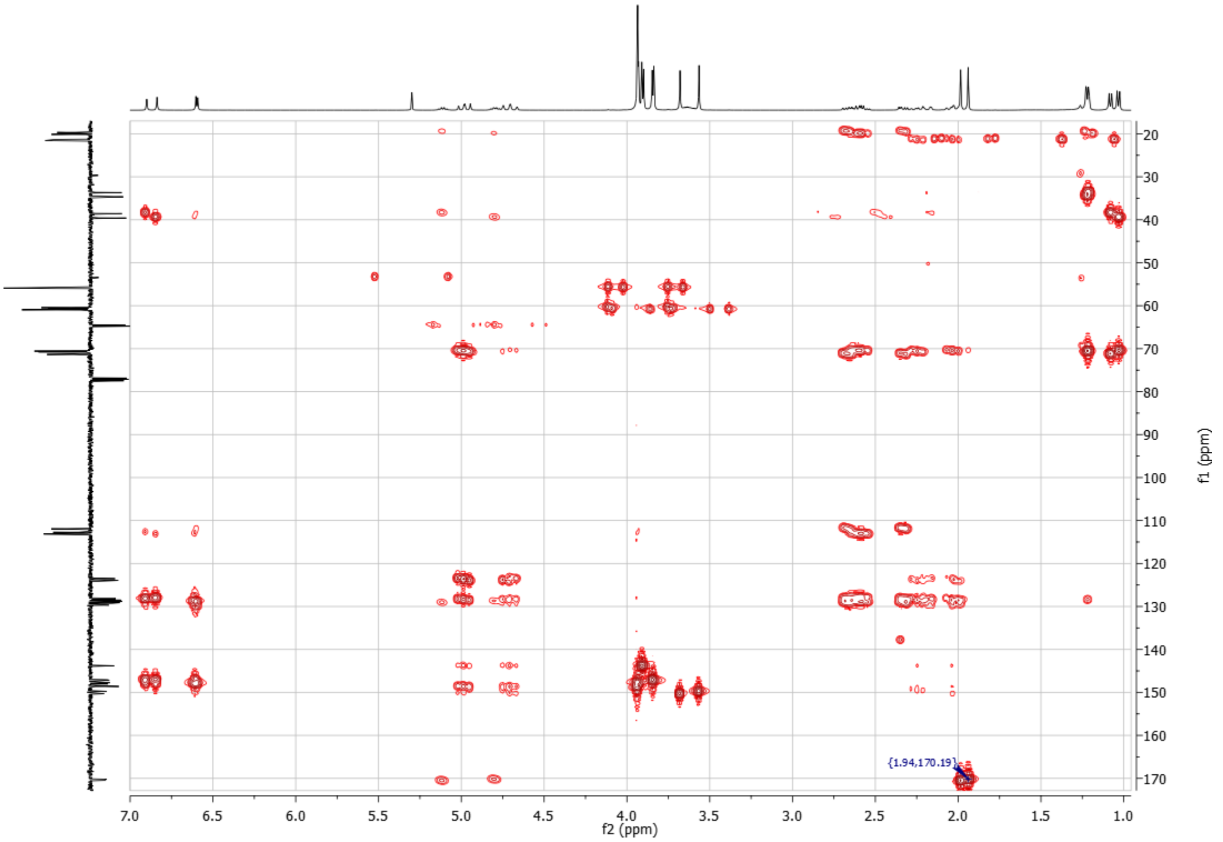

Figure S136. ^{1}H-^{13}C HMBC NMR (400 MHz) spectrum of the mixture of (a*R*,3*S*,2'*S*)-**26** and (a*S*,3*S*,2'*S*)-**26** in $CDCl_3$.

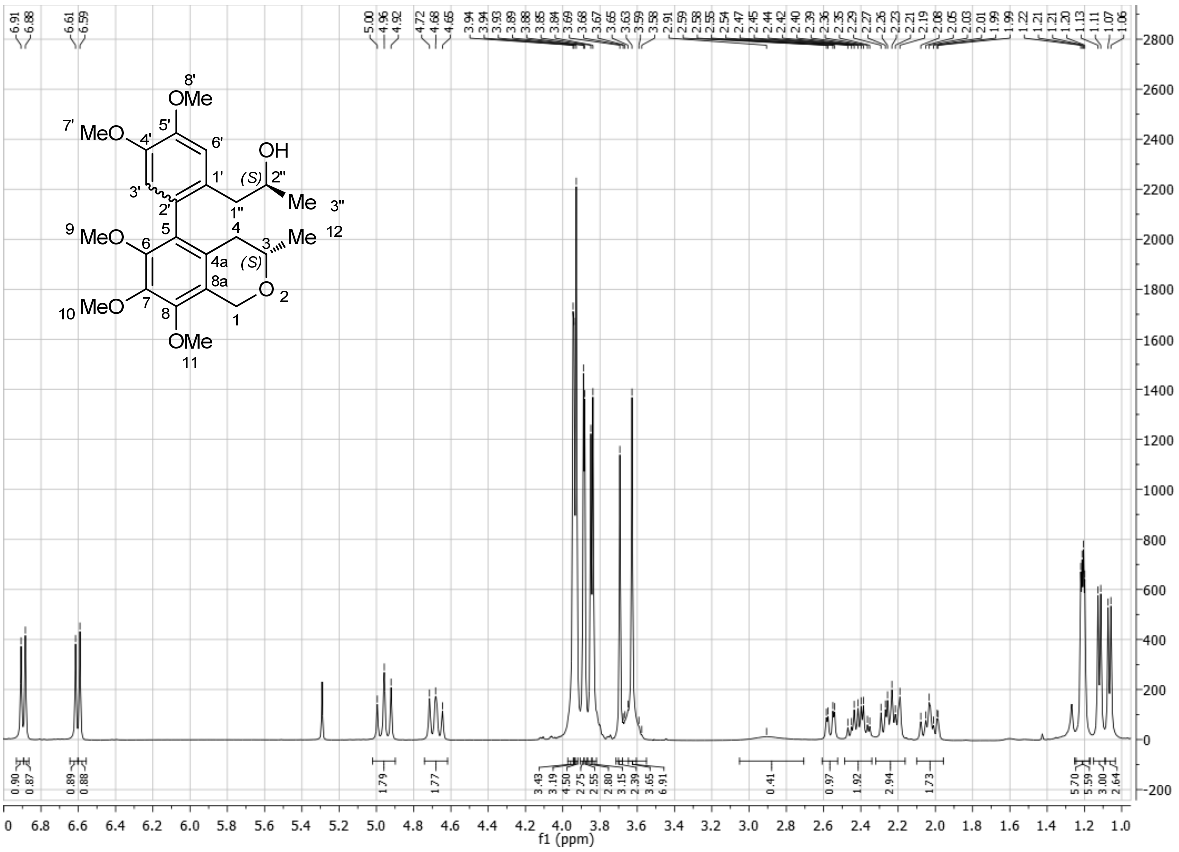

Figure S137. ^{1}H NMR (400 MHz) spectrum of the mixture of (a*R*,3*S*,2'*S*)-**27** and (a*S*,3*S*,2'*S*)-**27** in $CDCl_3$.

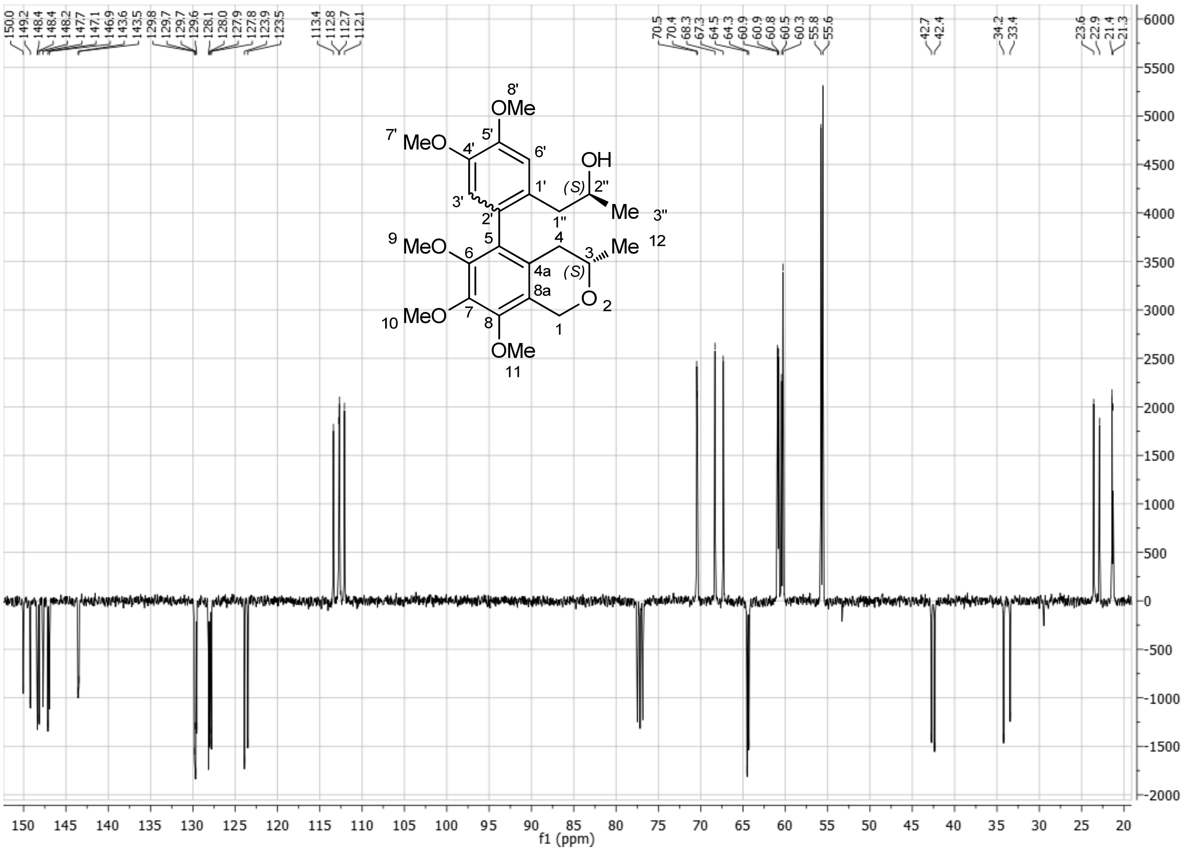

Figure S138. ^{13}C NMR (100 MHz) spectrum of the mixture of (a*R*,3*S*,2'*S*)-**27** and (a*S*,3*S*,2'*S*)-**27** in $CDCl_3$.

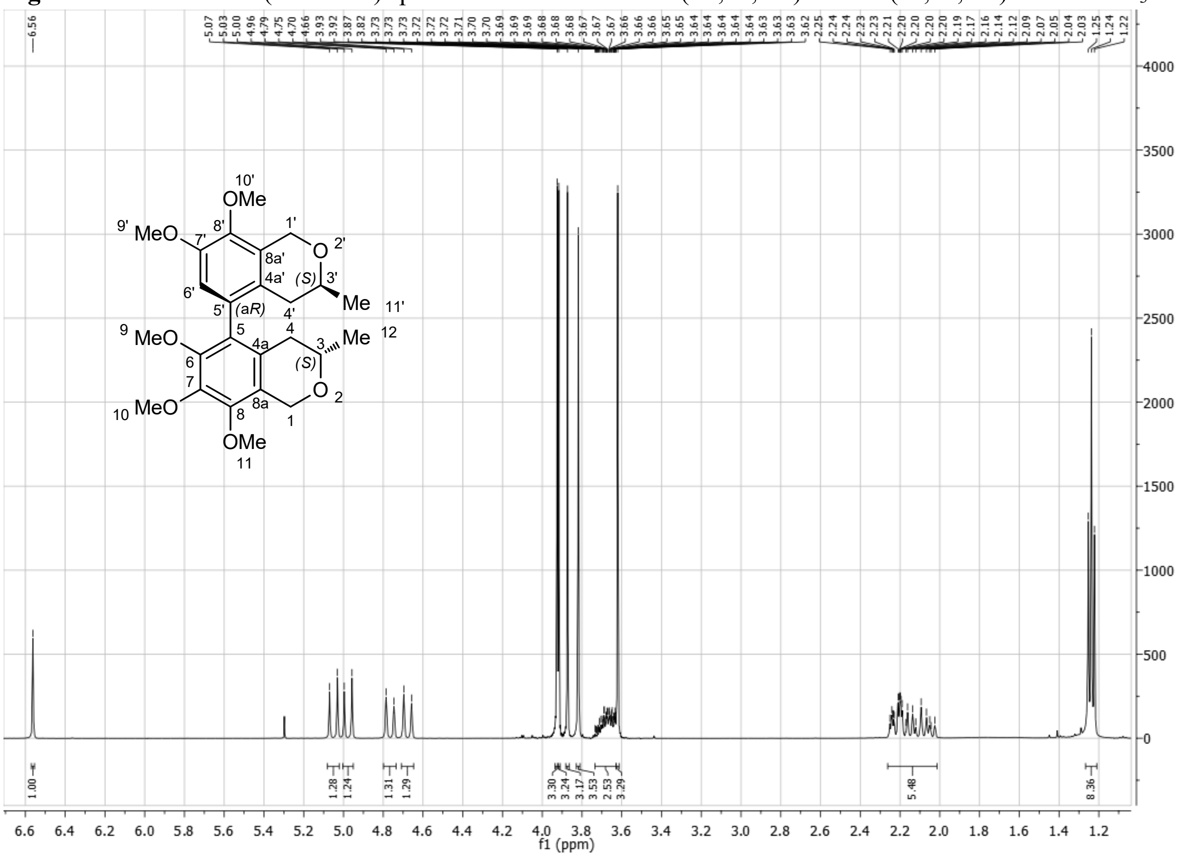

Figure S139. ^{1}H NMR (400 MHz) spectrum of (a*R*,3*S*,3'*S*)-**28** in $CDCl_3$.

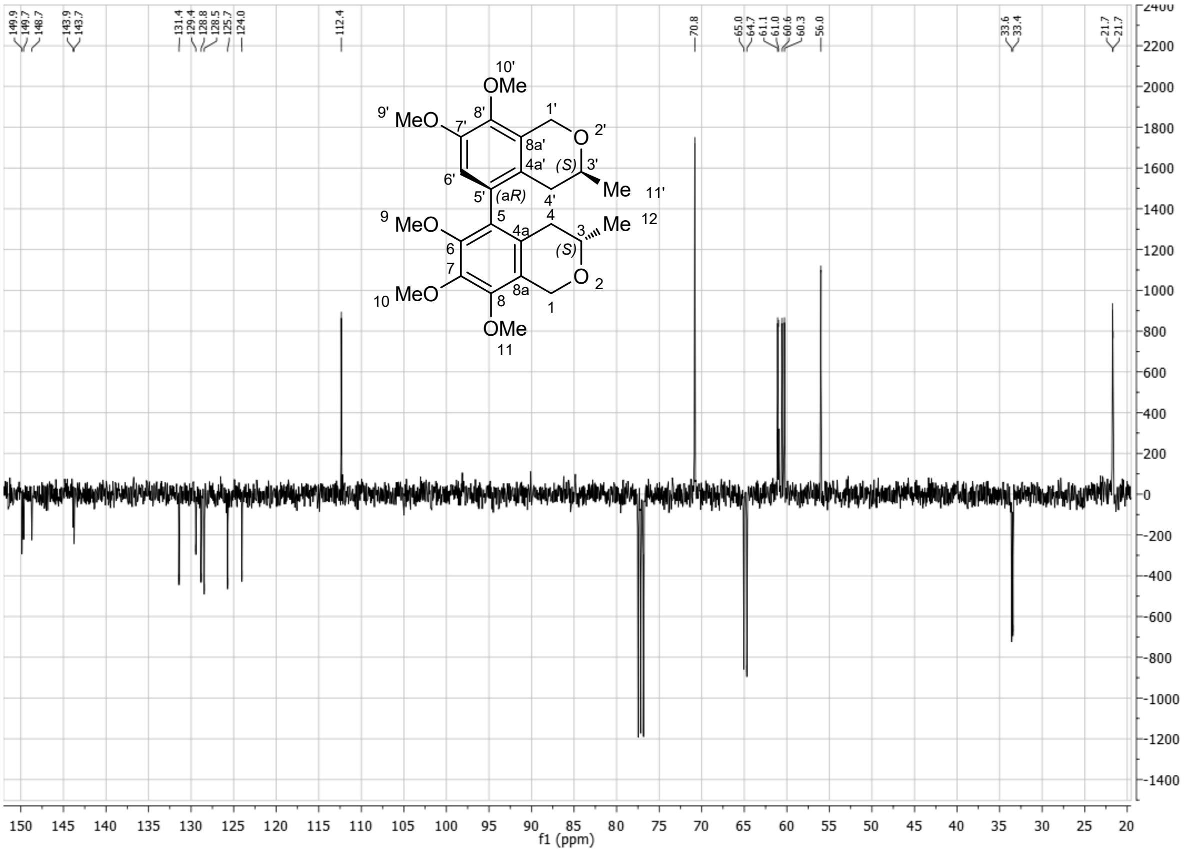

Figure S140. ^{13}C NMR (100 MHz) spectrum of (a*R*,3*S*,3'*S*)-**28** in CDCl$_3$.

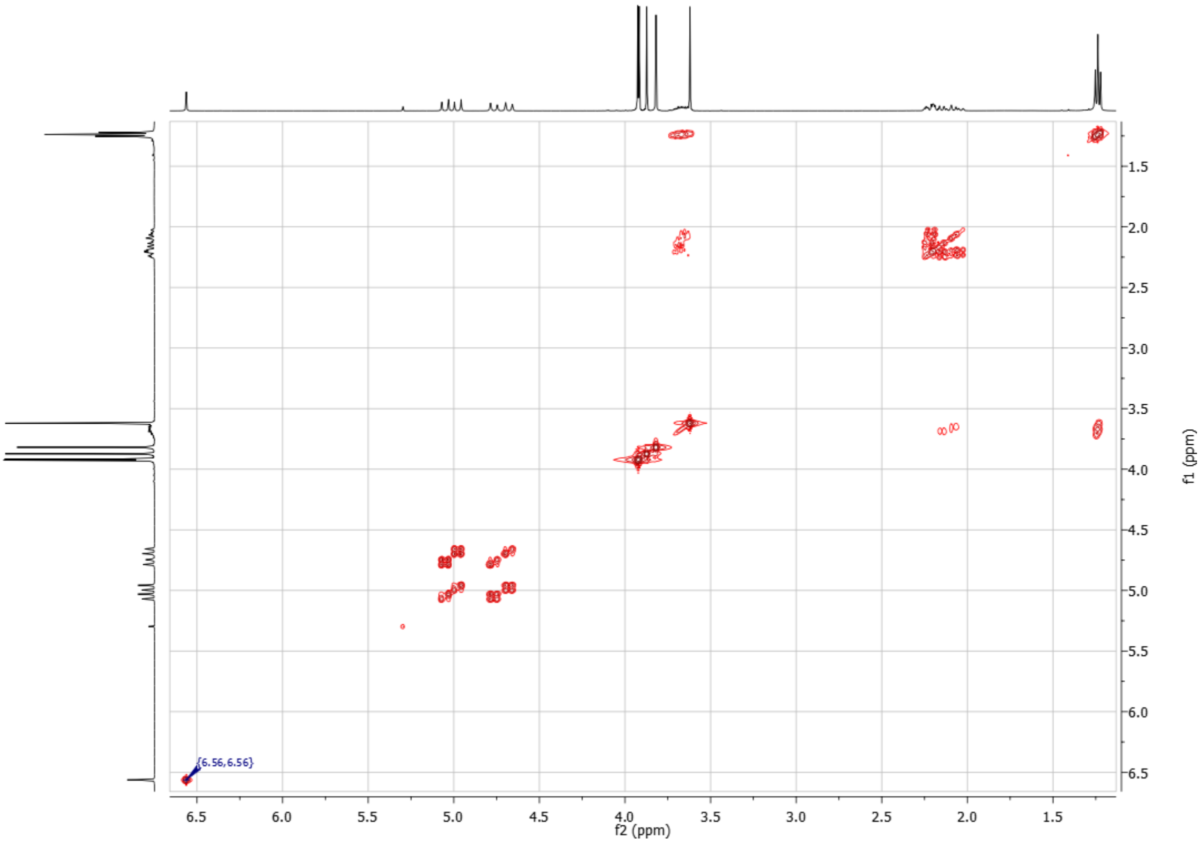

Figure S141. ^{1}H-^{1}H COSY NMR (400 MHz) spectrum of (a*R*,3*S*,3'*S*)-**28** in CDCl$_3$.

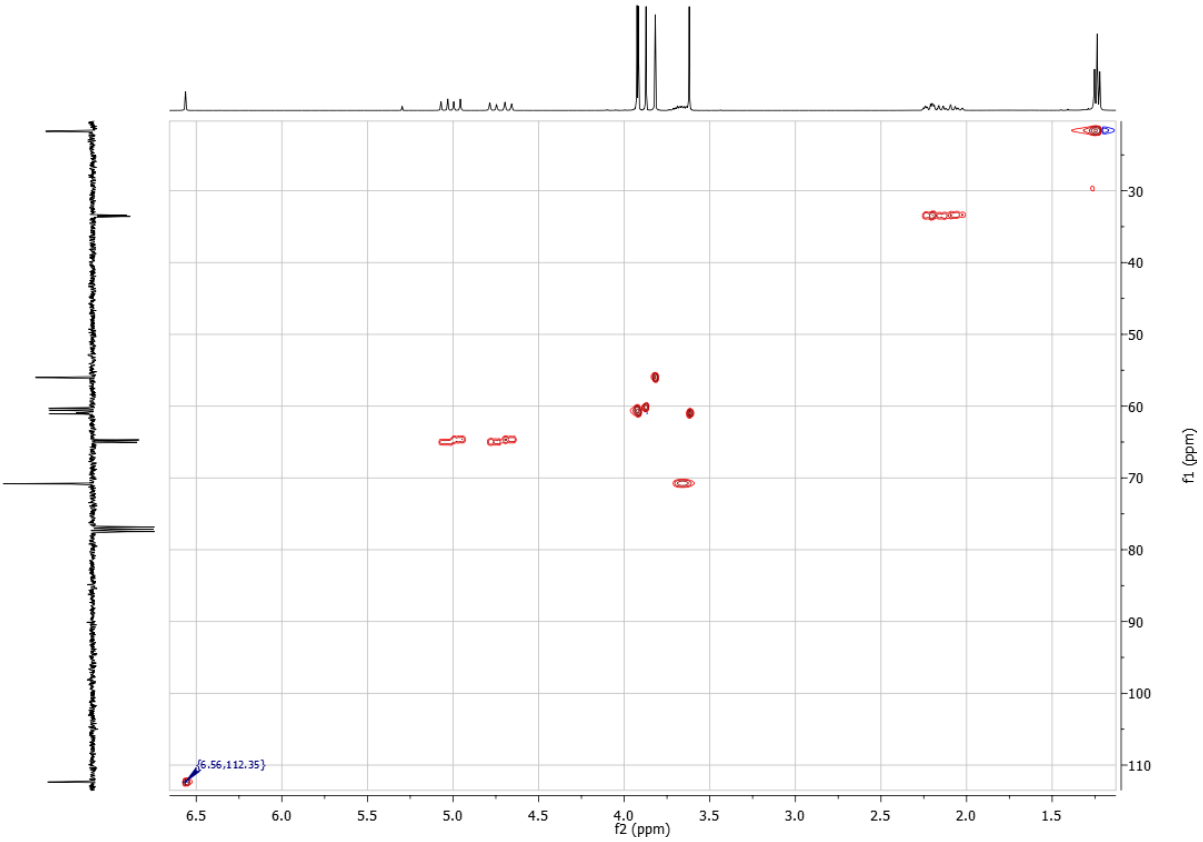

Figure S142. ^{1}H-^{13}C HSQC NMR (400 MHz) spectrum of (a*R*,3*S*,3’*S*)-**28** in $CDCl_3$.

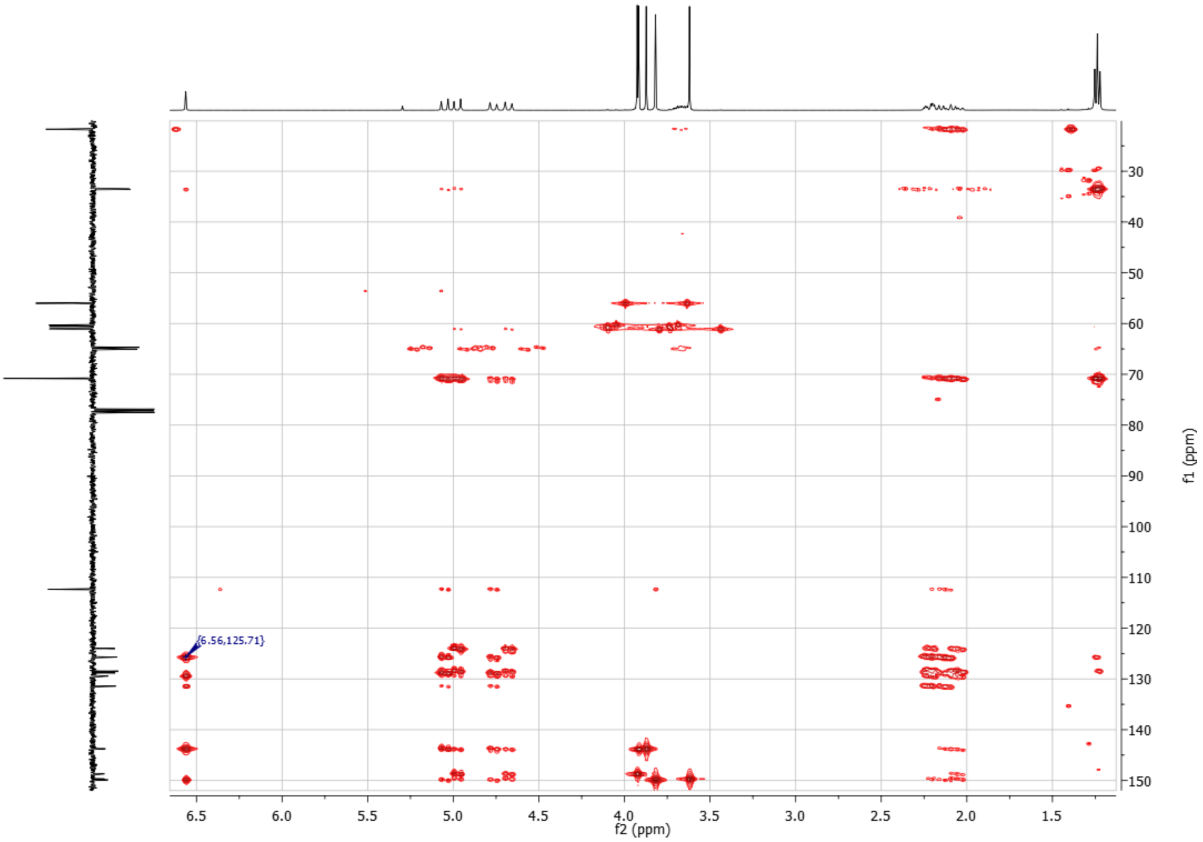

Figure S143. ^{1}H-^{13}C HMBC NMR (400 MHz) spectrum of (a*R*,3*S*,3’*S*)-**28** in $CDCl_3$.

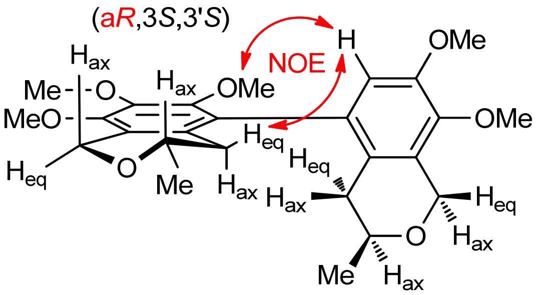

Figure S144. Characteristic NOE correlations shown on the structure of (a*R*,3*S*,3'*S*)-**28** suggesting (a*R*) axial chirality.

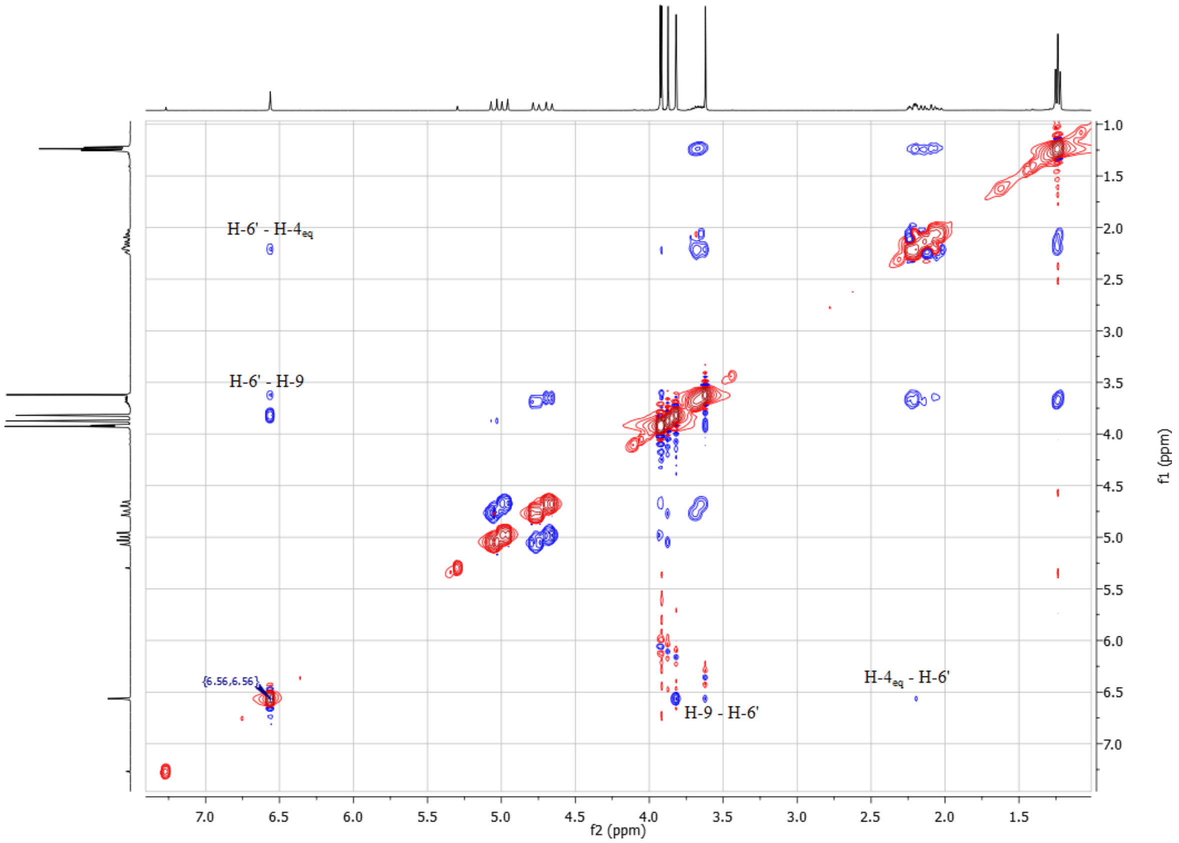

Figure S145. ^{1}H-^{1}H NOESY NMR (400 MHz) spectrum of (a*R*,3*S*,3'*S*)-**28** in $CDCl_3$.

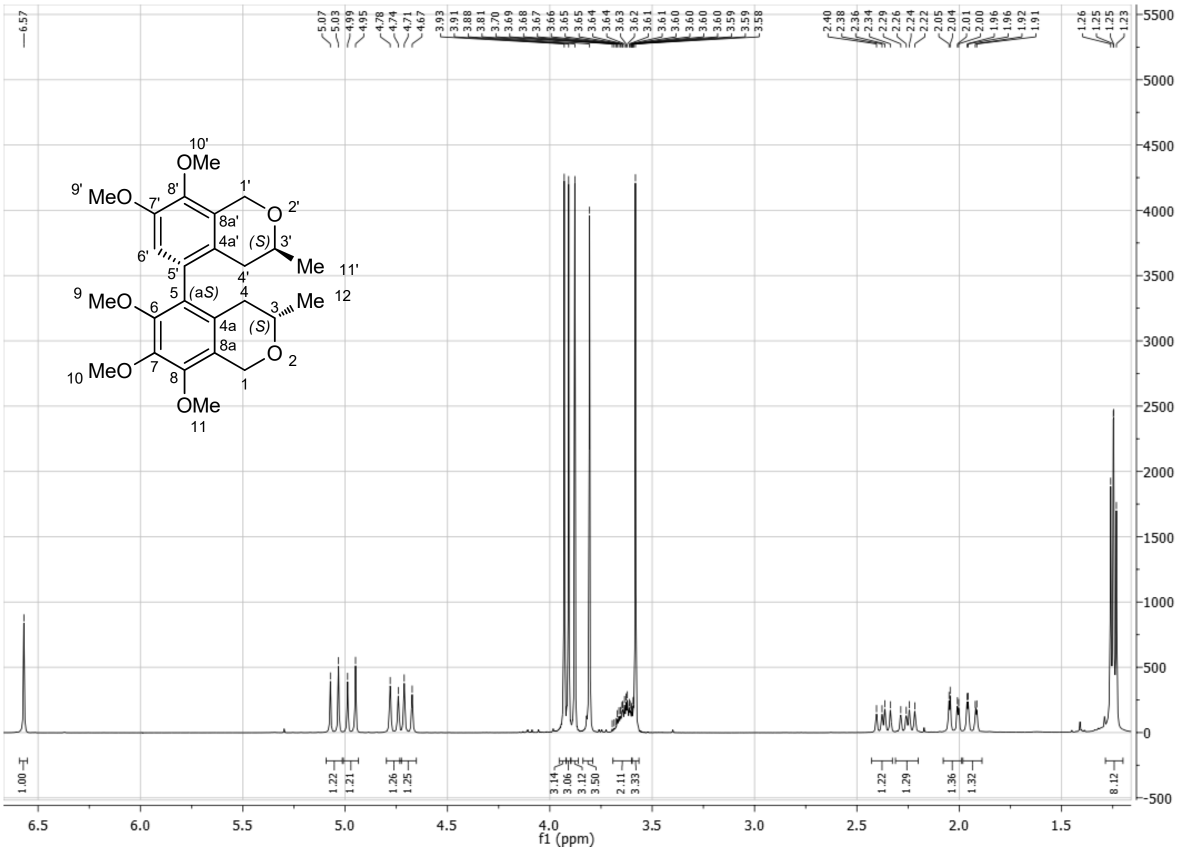

Figure S146. ^{1}H NMR (400 MHz) spectrum of (a*S*,3*S*,3'*S*)-**28** in $CDCl_3$.

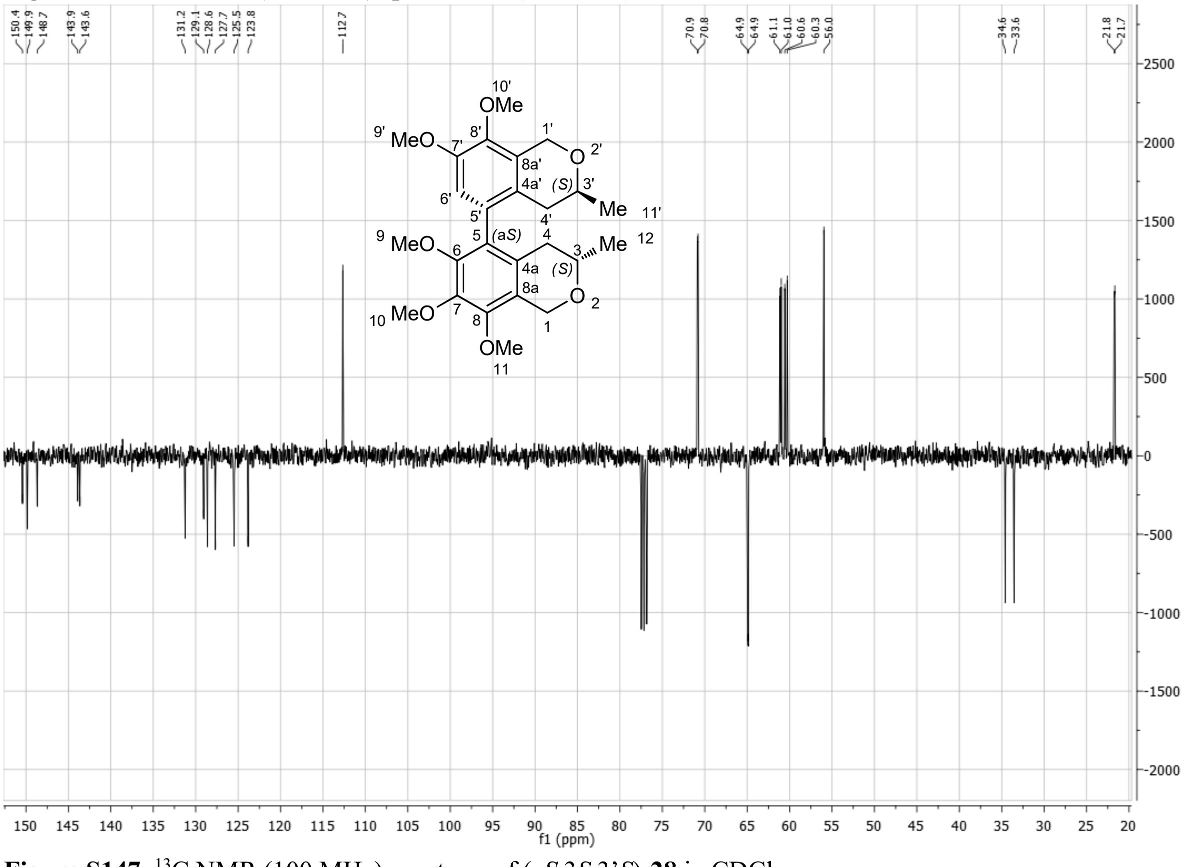

Figure S147. ^{13}C NMR (100 MHz) spectrum of (a*S*,3*S*,3'*S*)-**28** in $CDCl_3$.

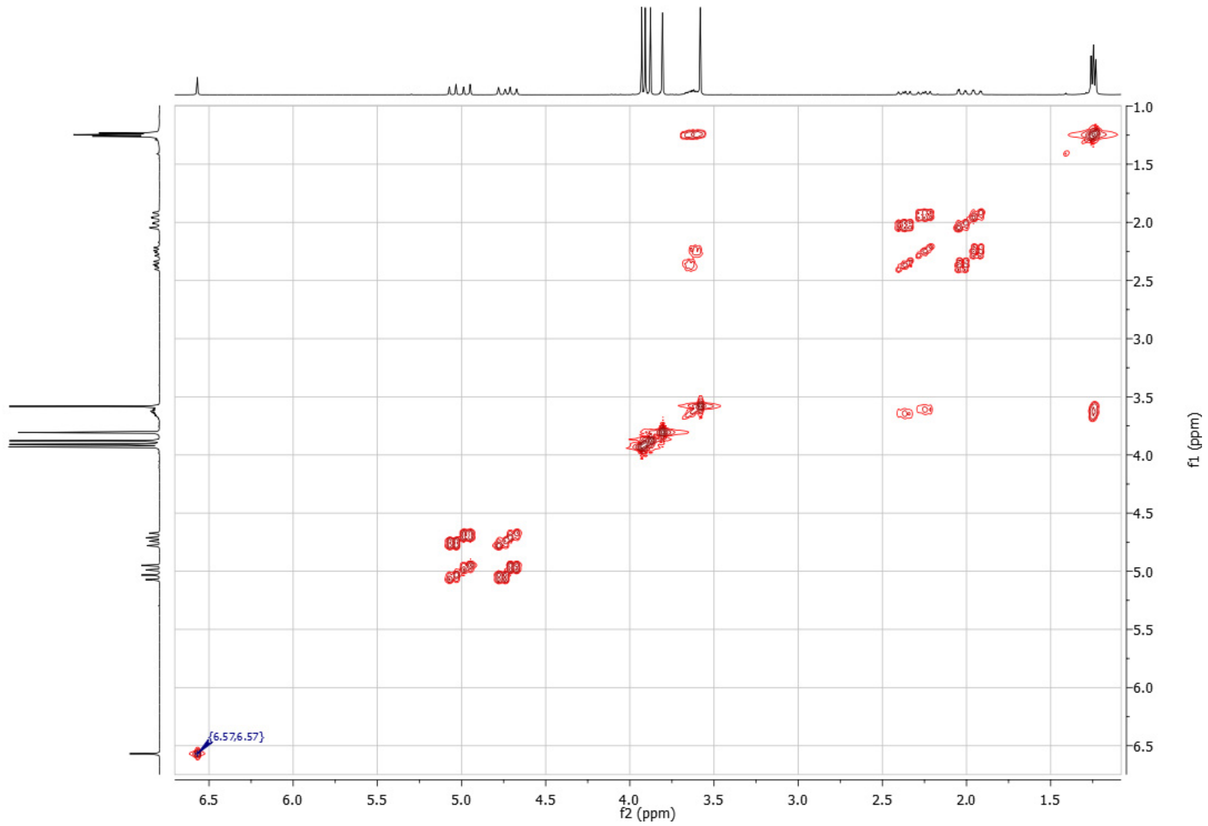

Figure S148. ^{1}H-^{1}H COSY NMR (400 MHz) spectrum of (a*S*,3*S*,3'*S*)-**28** in $CDCl_3$.

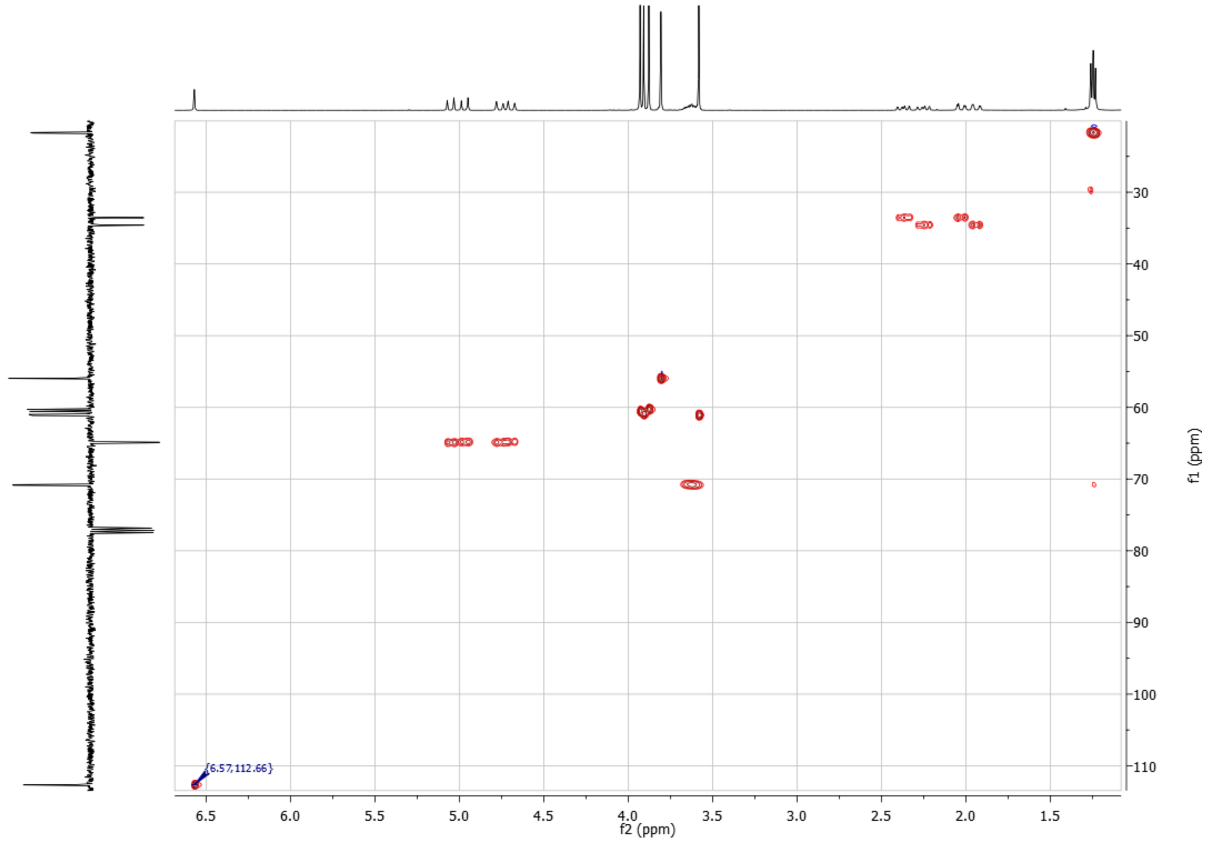

Figure S149. ^{1}H-^{13}C HSQC NMR (400 MHz) spectrum of (a*S*,3*S*,3'*S*)-**28** in $CDCl_3$.

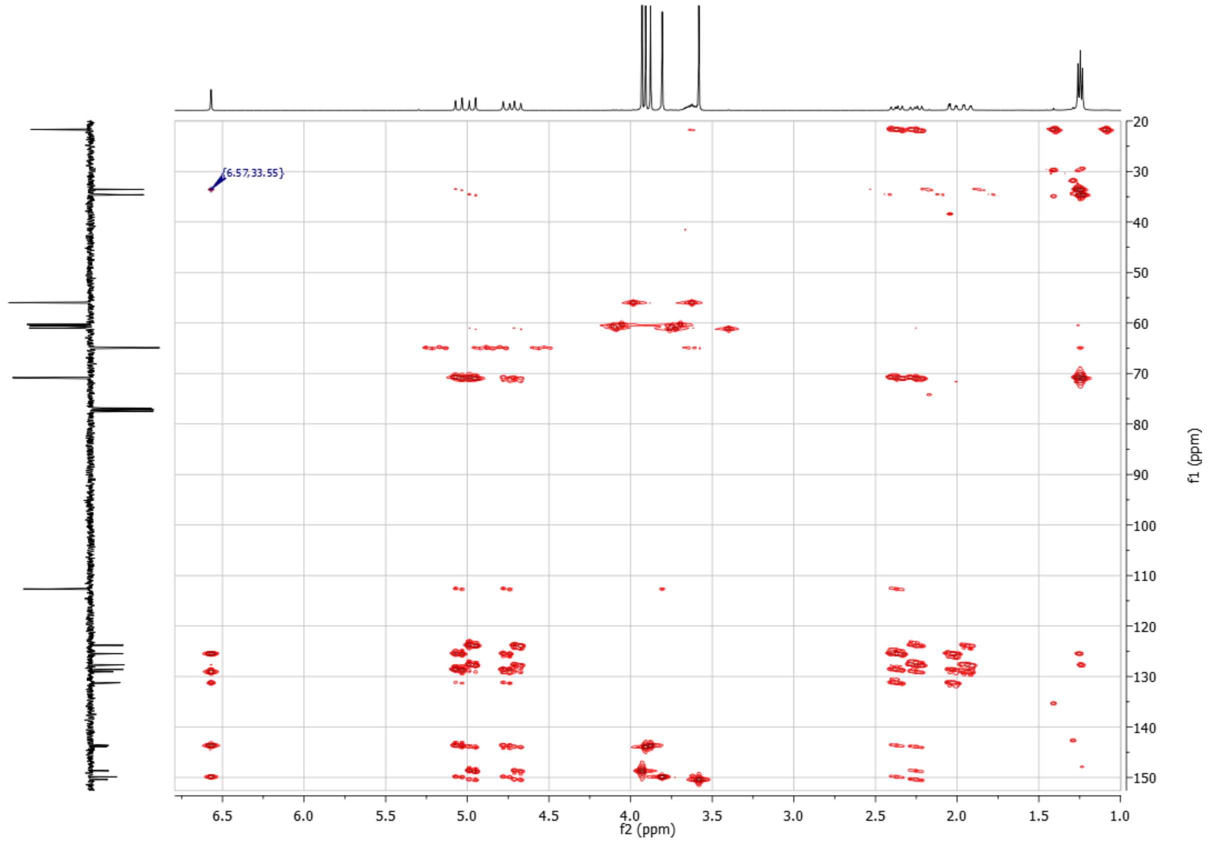

Figure S150. ^{1}H-^{13}C HMBC NMR (400 MHz) spectrum of (a*S*,3*S*,3'*S*)-**28** in $CDCl_3$.

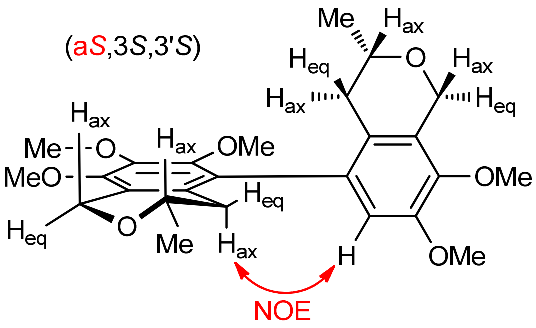

Figure S151. Characteristic NOE correlation shown on the structure of (a*S*,3*S*,3'*S*)-**28** suggesting (a*S*) axial chirality.

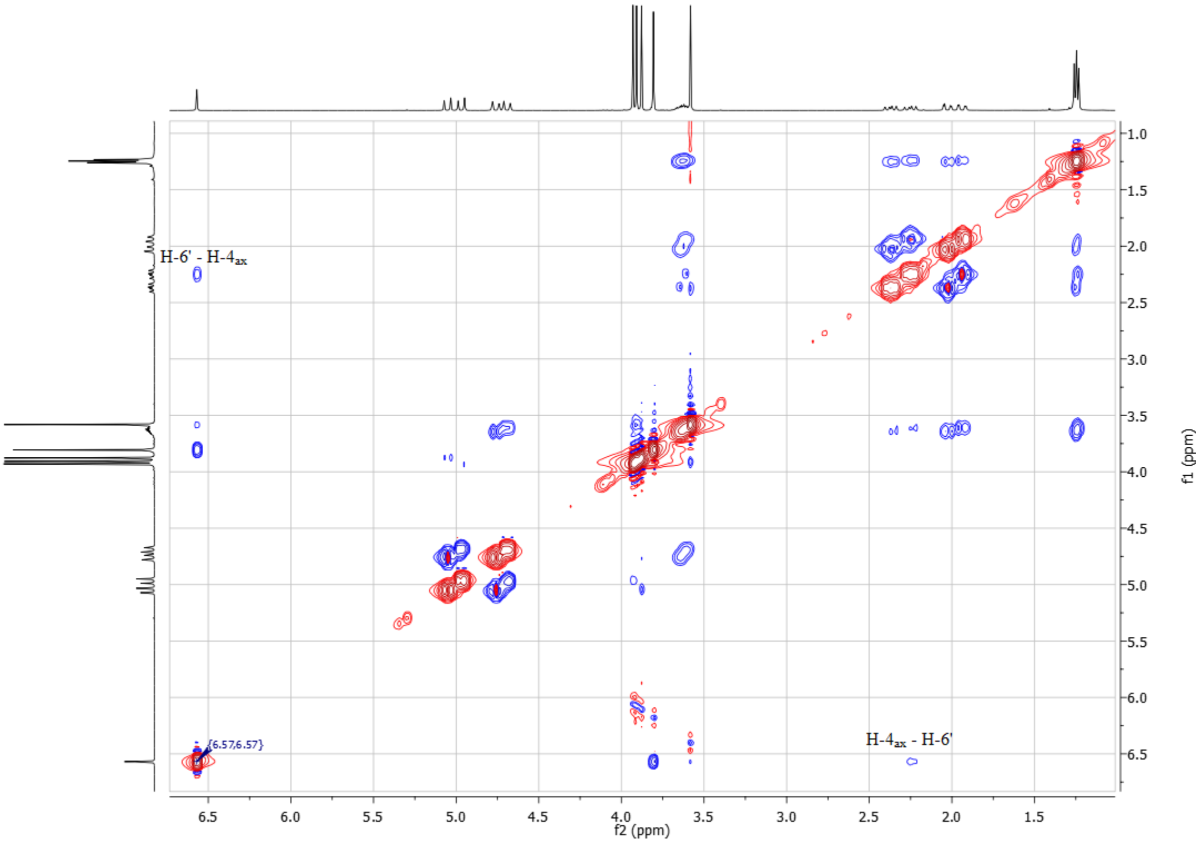

Figure S152. ^{1}H-^{1}H NOESY NMR (400 MHz) spectrum of (a*S*,3*S*,3'*S*)-**28** in $CDCl_3$.

3.2. ECD and VCD spectra

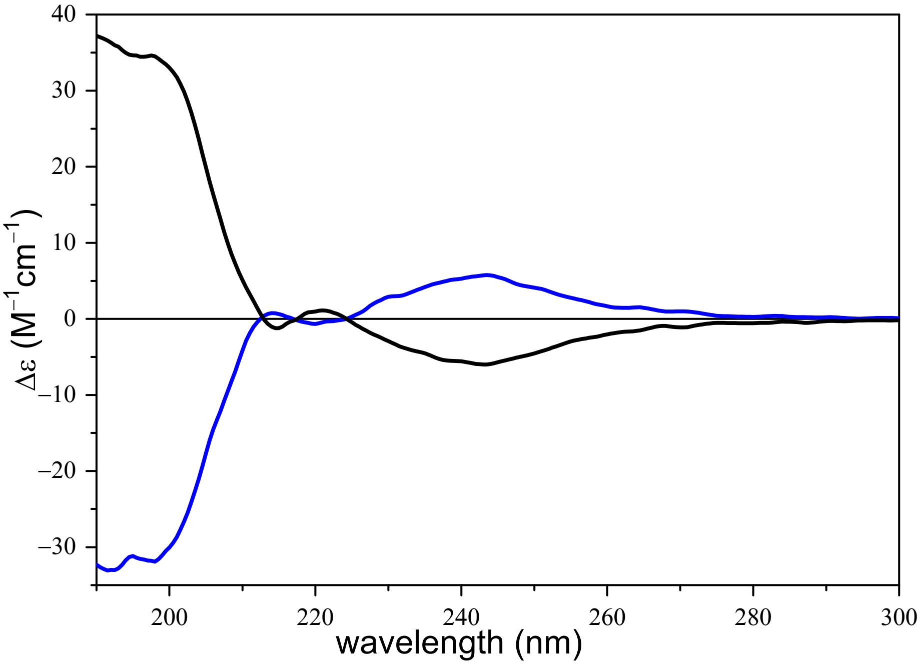

Figure S153. Experimental ECD spectra of enantiomeric (a*S*,1*S*,3*S*,3'*S*)-**21** (black) and (a*R*,1*R*,3*R*,3'*R*)-**21** (blue) in MeCN.

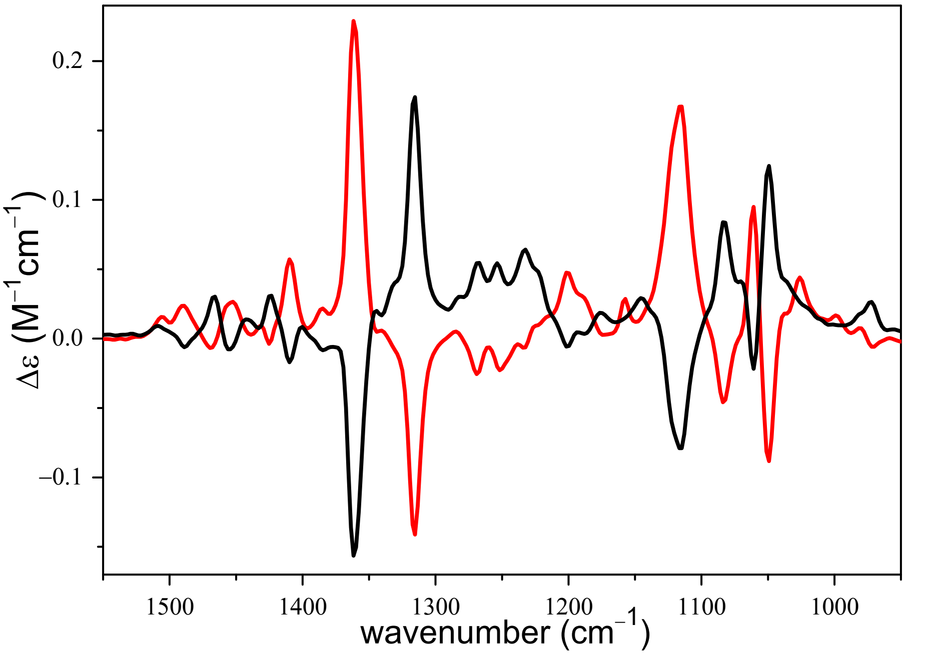

Figure S154. Experimental VCD spectra of enantiomeric (a*S*,1*S*,3*S*,3'*S*)-**21** (red) and (a*R*,1*R*,3*R*,3'*R*)-**21** (black) in $CDCl_3$.

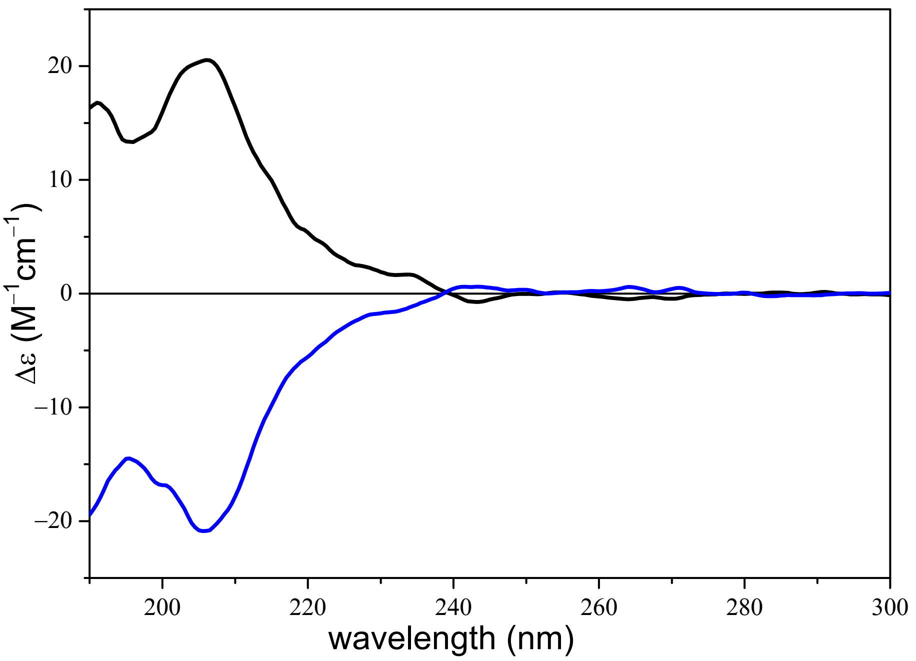

Figure S155. Experimental ECD spectra of enantiomeric (a*R*,1*S*,3*S*,3'*S*)-**21** (black) and (a*S*,1*R*,3*R*,3'*R*)-**21** (blue) in MeCN.

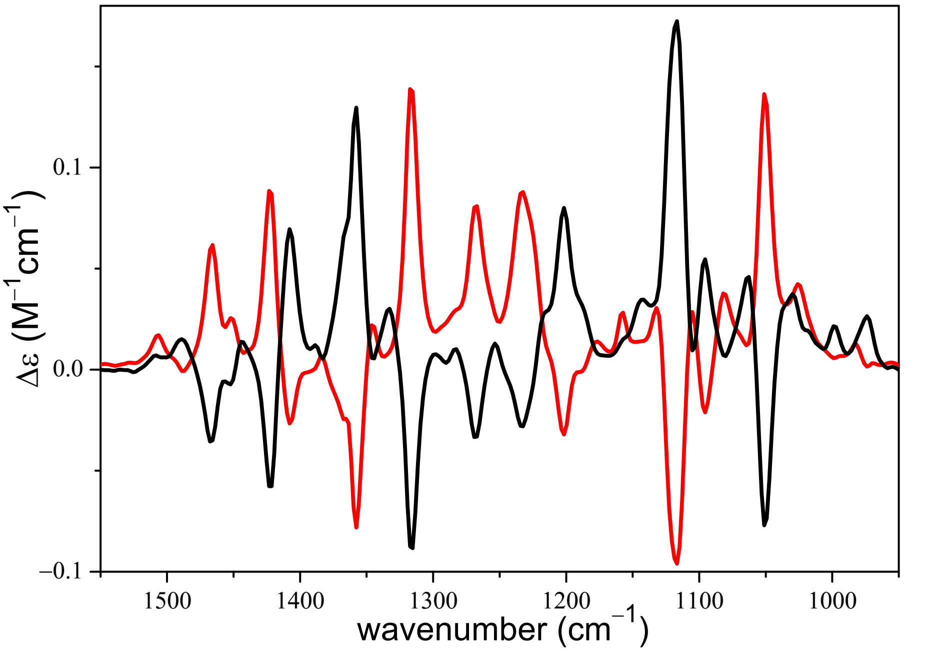

Figure S156. Experimental VCD spectra of enantiomeric (a*R*,1*S*,3*S*,3'*S*)-**21** (red) and (a*S*,1*R*,3*R*,3'*R*)-**21** (black) in $CDCl_3$.

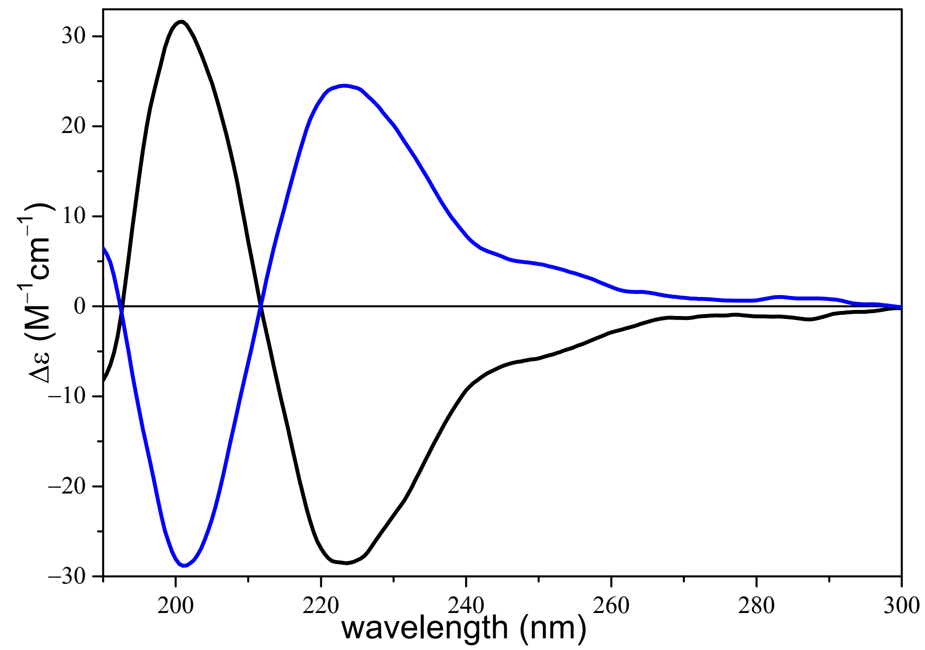

Figure S157. Experimental ECD spectra of enantiomeric (a*S*,1*R*,3*S*,3'*S*)-**21** (black) and (a*R*,1*S*,3*R*,3'*R*)-**21** (blue) in MeCN.

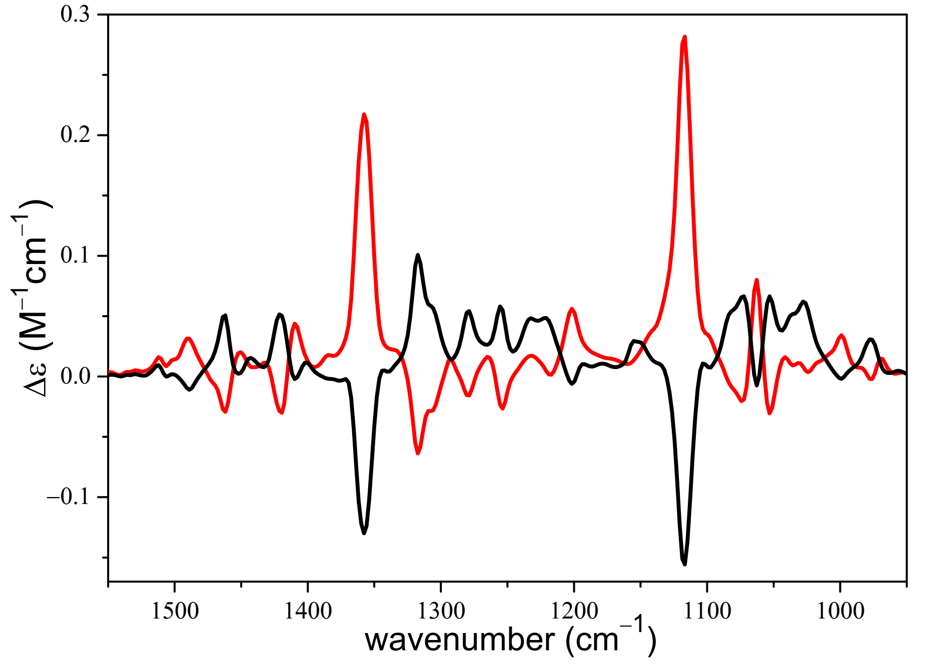

Figure S158. Experimental VCD spectra of enantiomeric (a*S*,1*R*,3*S*,3'*S*)-**21** (red) and (a*R*,1*S*,3*R*,3'*R*)-**21** (black) in $CDCl_3$.

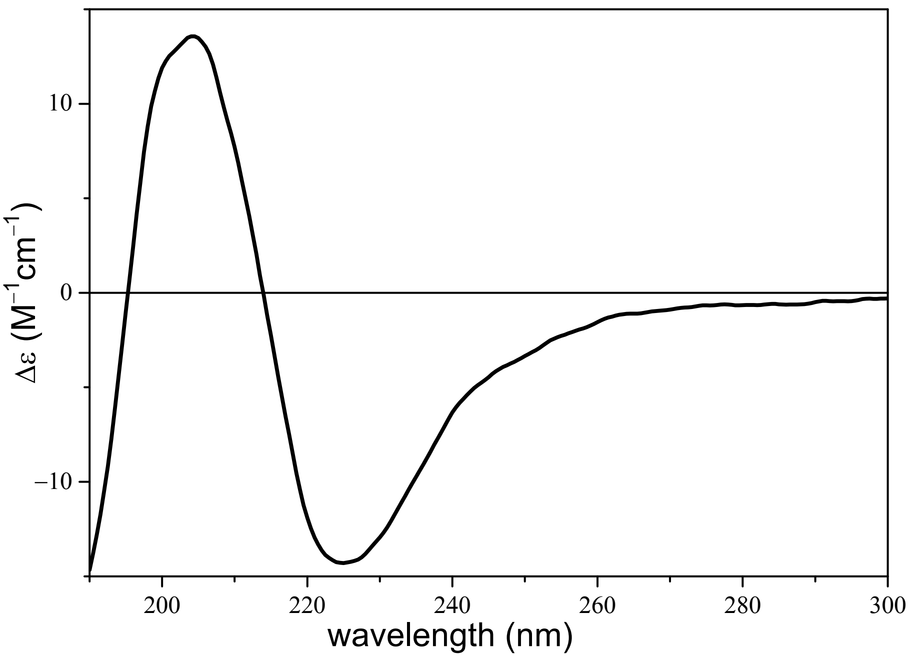

Figure S159. Experimental ECD spectrum of (a*R*,1*R*,3*S*,3'*S*)-**21** in MeCN.

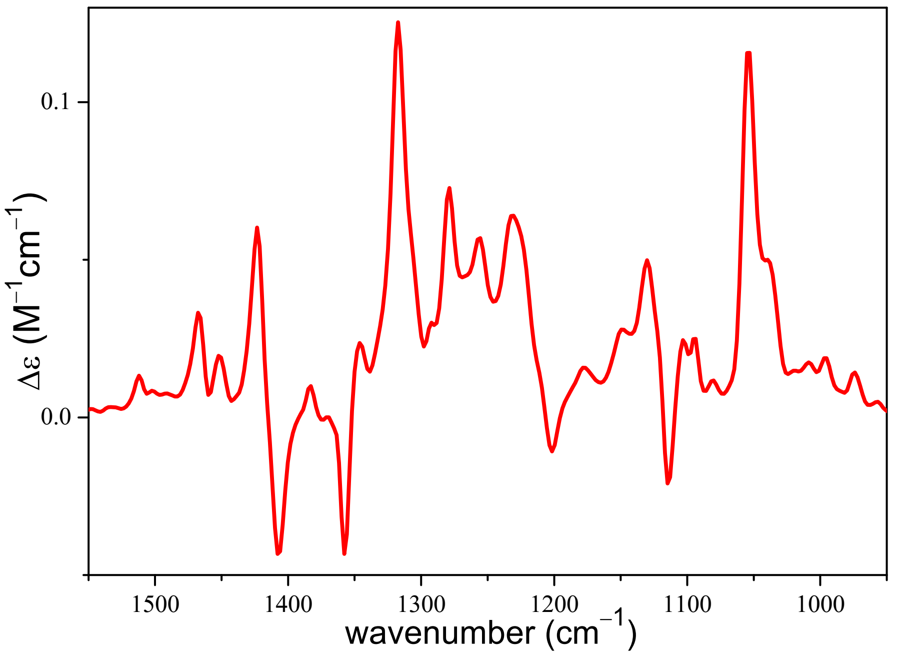

Figure S160. Experimental VCD spectrum of (a*R*,1*R*,3*S*,3'*S*)-**21** in $CDCl_3$.

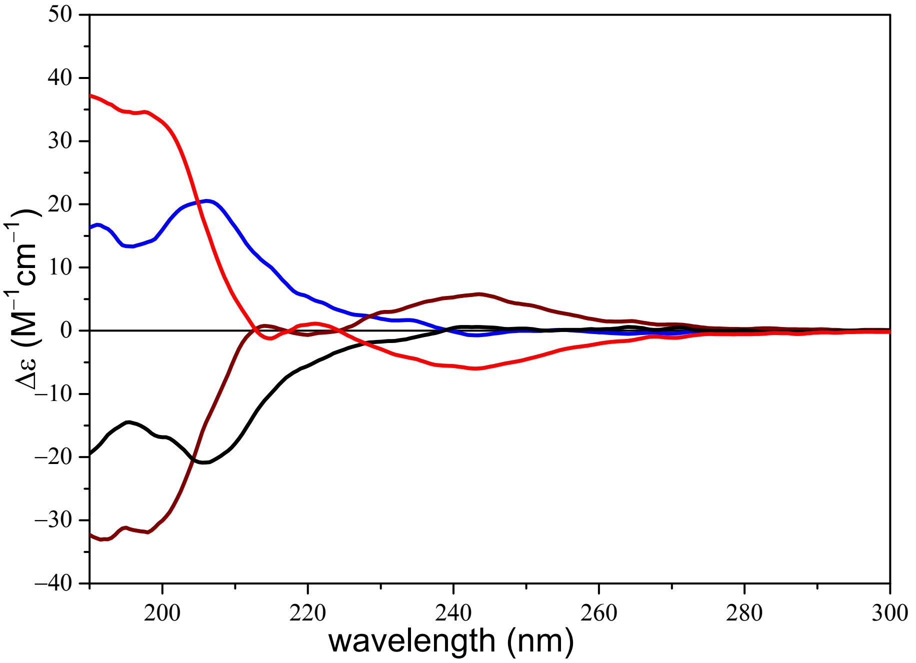

Figure S161. Overlapped experimental ECD spectra of stereoisomeric (a*S*,1*S*,3*S*,3'*S*)-**21** (red), (a*R*,1*S*,3*S*,3'*S*)-**21** (blue), (a*R*,1*R*,3*R*,3'*R*)-**21** (wine) and (a*S*,1*R*,3*R*,3'*R*)-**21** (black) in MeCN.

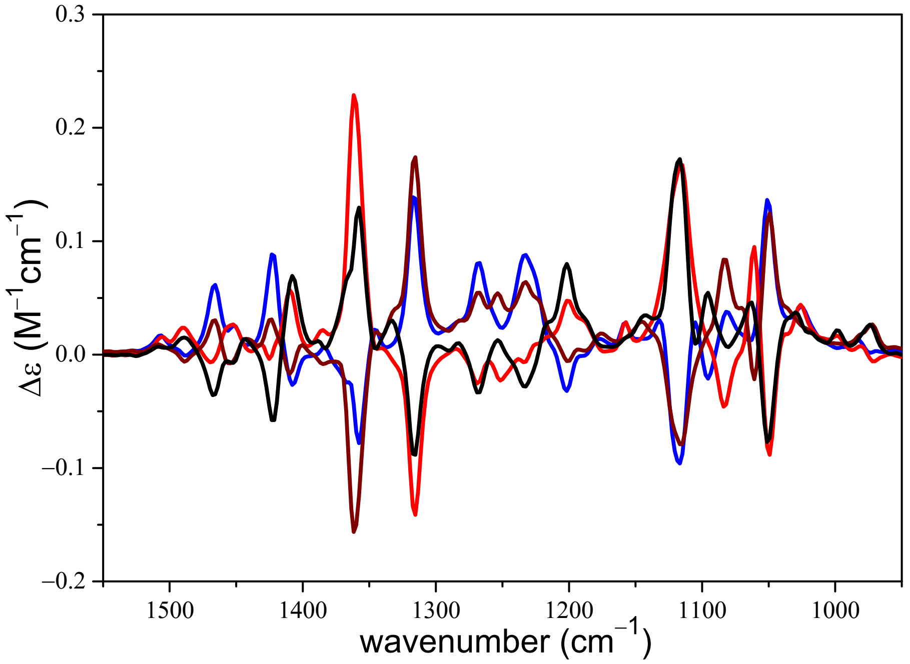

Figure S162. Overlapped experimental VCD spectra of stereoisomeric (a*S*,1*S*,3*S*,3'*S*)-**21** (red), (a*R*,1*S*,3*S*,3'*S*)-**21** (blue), (a*R*,1*R*,3*R*,3'*R*)-**21** (wine) and (a*S*,1*R*,3*R*,3'*R*)-**21** (black) in $CDCl_3$.

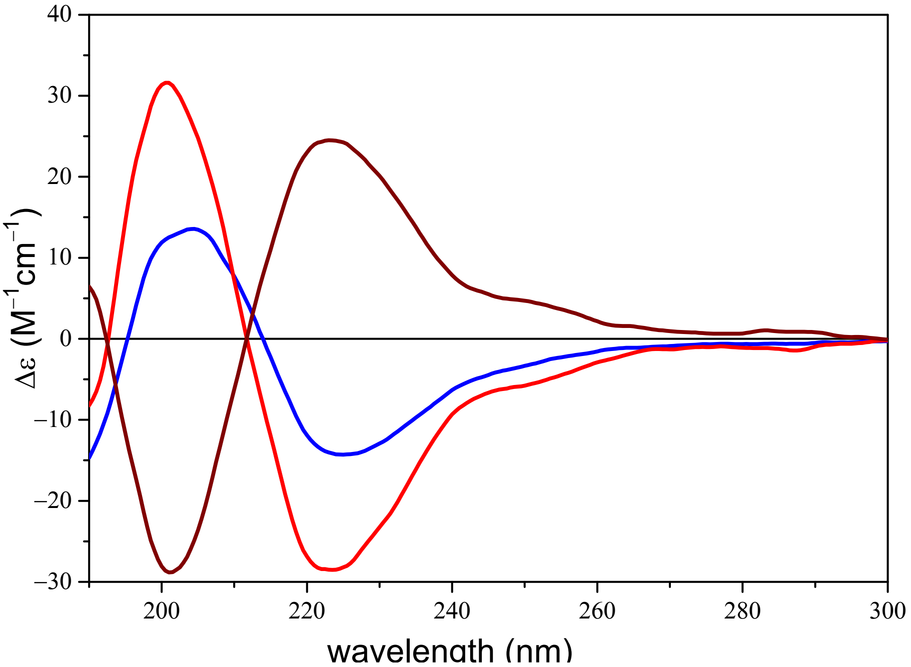

Figure S163. Overlapped experimental ECD spectra of stereoisomeric (a*S*,1*R*,3*S*,3'*S*)-**21** (red), (a*R*,1*R*,3*S*,3'*S*)-**21** (blue) and (a*R*,1*S*,3*R*,3'*R*)-**21** (wine) in MeCN.

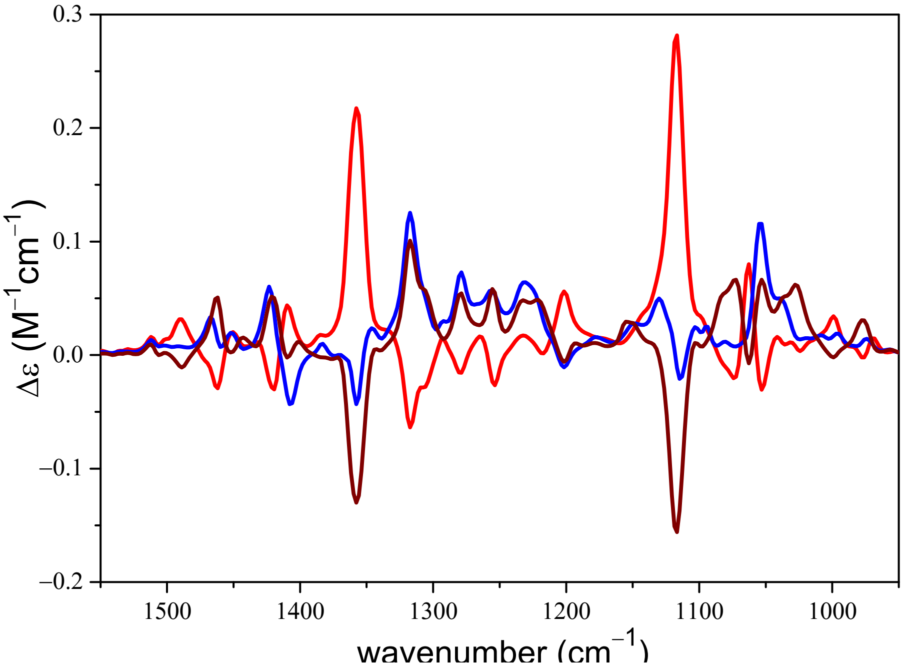

Figure S164. Overlapped experimental VCD spectra of stereoisomeric (a*S*,1*R*,3*S*,3'*S*)-**21** (red), (a*R*,1*R*,3*S*,3'*S*)-**21** (blue) and (a*R*,1*S*,3*R*,3'*R*)-**21** (wine) in $CDCl_3$.

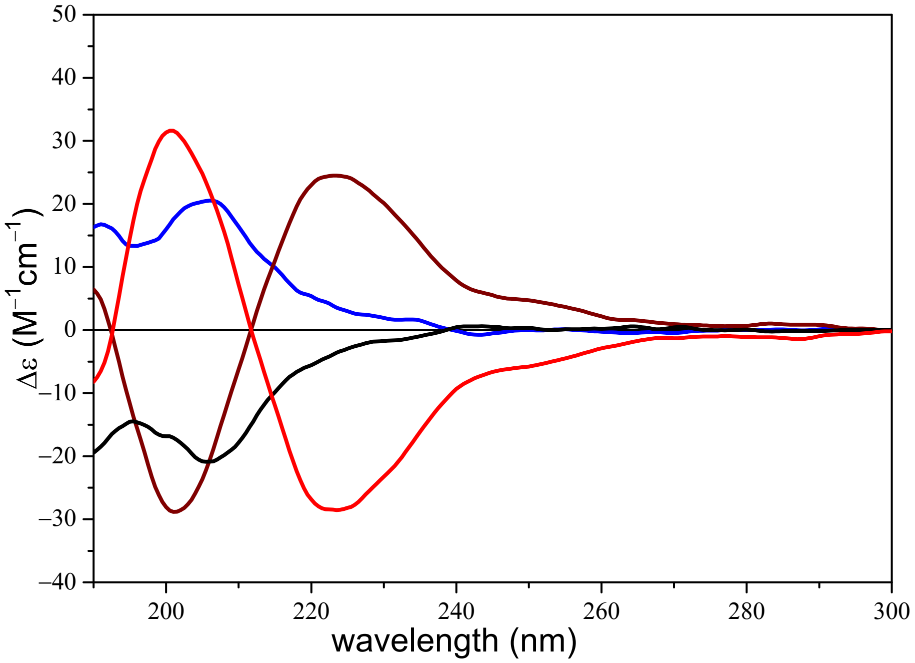

Figure S165. Overlapped experimental ECD spectra of stereoisomeric (a*R*,1*S*,3*S*,3'*S*)-**21** (blue), (a*S*,1*R*,3*S*,3'*S*)-**21** (red), (a*S*,1*R*,3*R*,3'*R*)-**21** (black) and (a*R*,1*S*,3*R*,3'*R*)-**21** (wine) in MeCN.

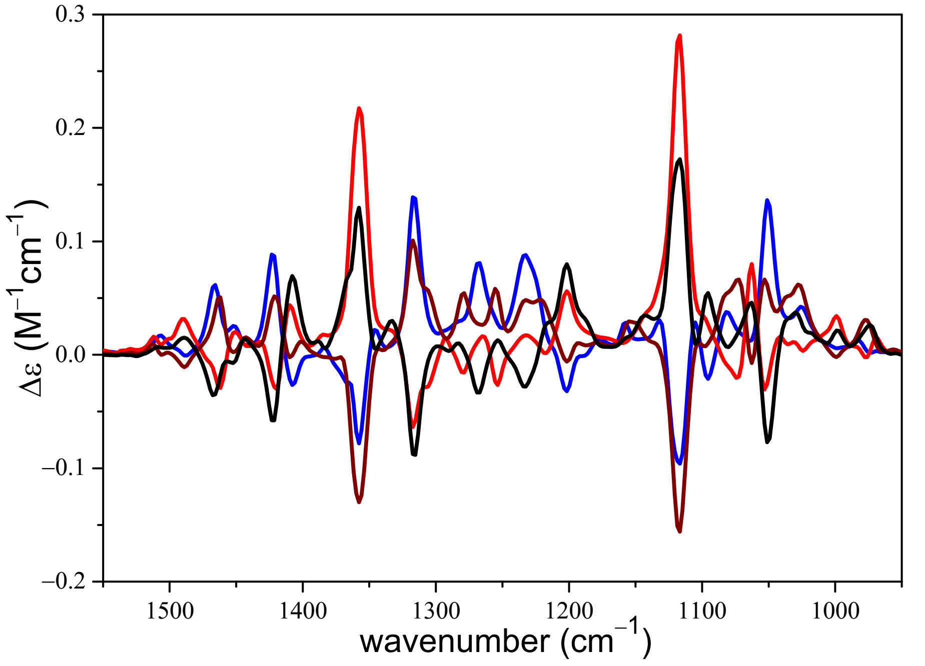

Figure S166. Overlapped experimental VCD spectra of stereoisomeric (a*R*,1*S*,3*S*,3'*S*)-**21** (blue), (a*S*,1*R*,3*S*,3'*S*)-**21** (red), (a*S*,1*R*,3*R*,3'*R*)-**21** (black) and (a*R*,1*S*,3*R*,3'*R*)-**21** (wine) in $CDCl_3$.

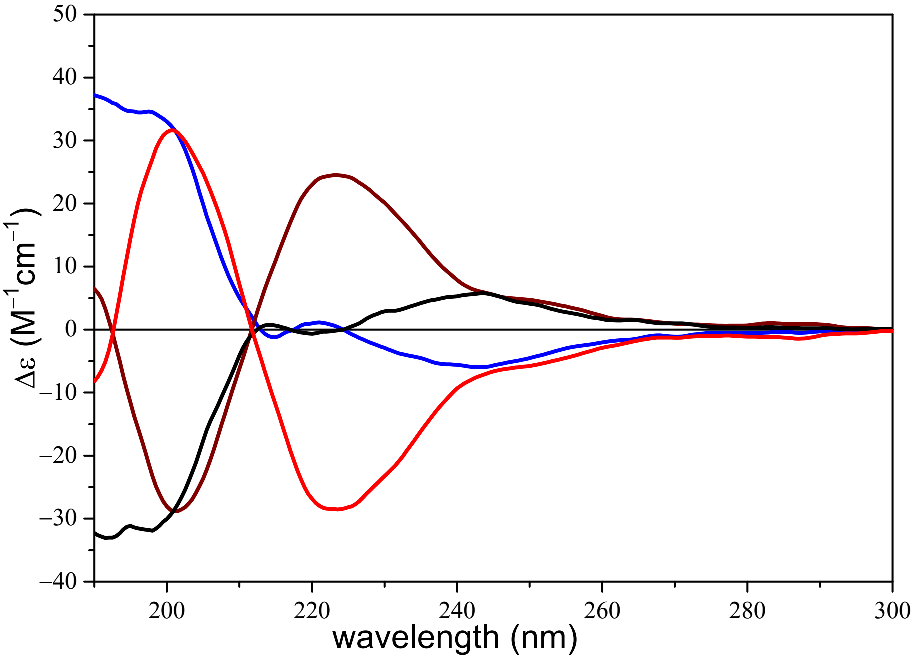

Figure S167. Overlapped experimental ECD spectra of stereoisomeric (a*S*,1*S*,3*S*,3'*S*)-**21** (blue), (a*S*,1*R*,3*S*,3'*S*)-**21** (red), (a*R*,1*R*,3*R*,3'*R*)-**21** (black) and (a*R*,1*S*,3*R*,3'*R*)-**21** (wine) in MeCN.

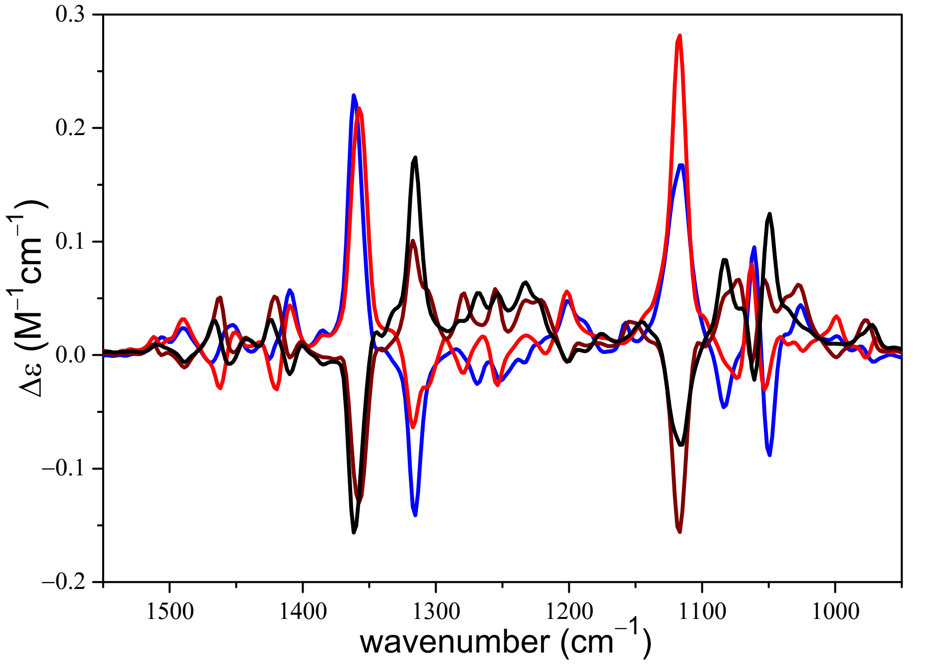

Figure S168. Overlapped experimental VCD spectra of stereoisomeric (a*S*,1*S*,3*S*,3'*S*)-**21** (blue), (a*S*,1*R*,3*S*,3'*S*)-**21** (red), (a*R*,1*R*,3*R*,3'*R*)-**21** (black) and (a*R*,1*S*,3*R*,3'*R*)-**21** (wine) in $CDCl_3$.

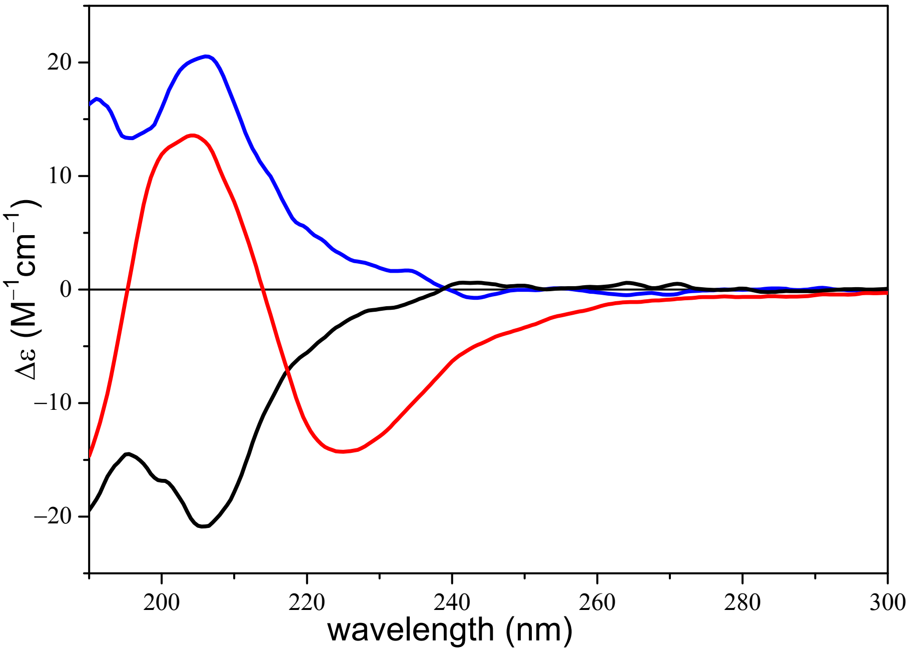

Figure S169. Overlapped experimental ECD spectra of stereoisomeric (a*R*,1*S*,3*S*,3'*S*)-**21** (blue), (a*R*,1*R*,3*S*,3'*S*)-**21** (red) and (a*S*,1*R*,3*R*,3'*R*)-**21** (black) in MeCN.

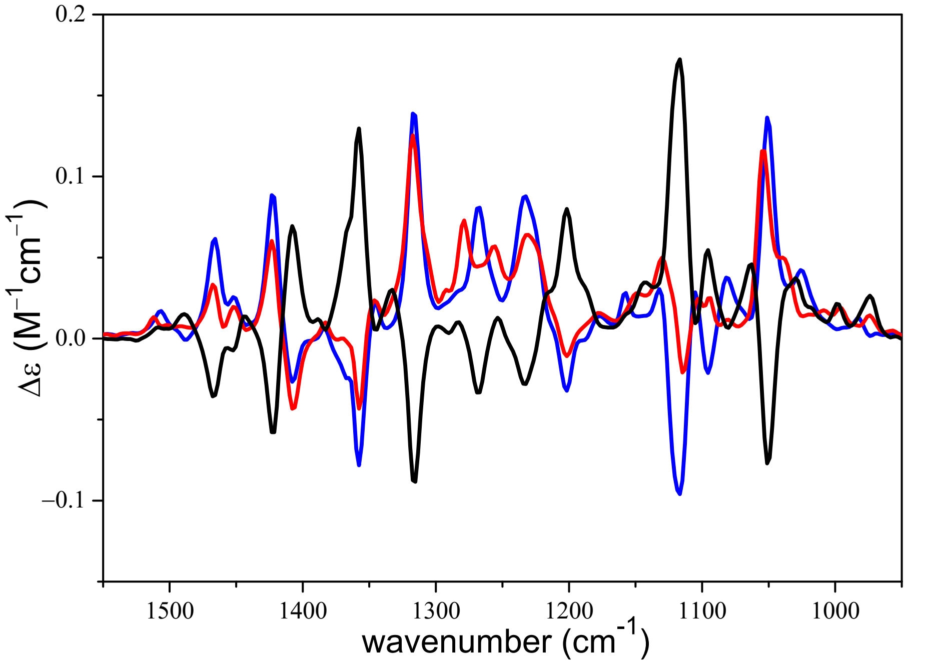

Figure S170. Overlapped experimental VCD spectra of stereoisomeric (a*R*,1*S*,3*S*,3'*S*)-**21** (blue), (a*R*,1*R*,3*S*,3'*S*)-**21** (red) and (a*S*,1*R*,3*R*,3'*R*)-**21** (black) in $CDCl_3$.

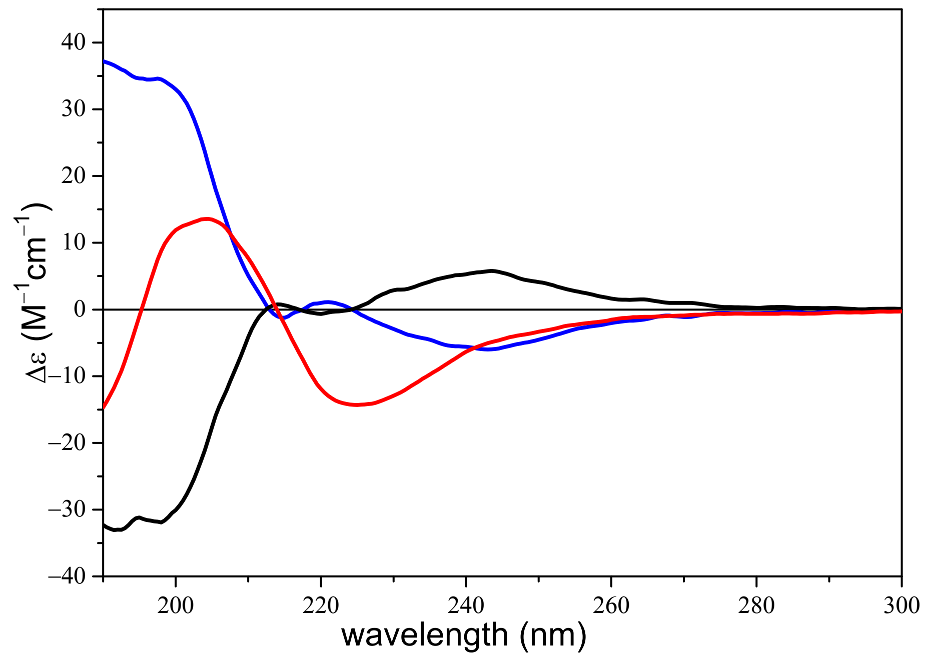

Figure S171. Overlapped experimental ECD spectra of stereoisomeric (a*S*,1*S*,3*S*,3'*S*)-**21** (blue), (a*R*,1*R*,3*S*,3'*S*)-**21** (red) and (a*R*,1*R*,3*R*,3'*R*)-**21** (black) in MeCN.

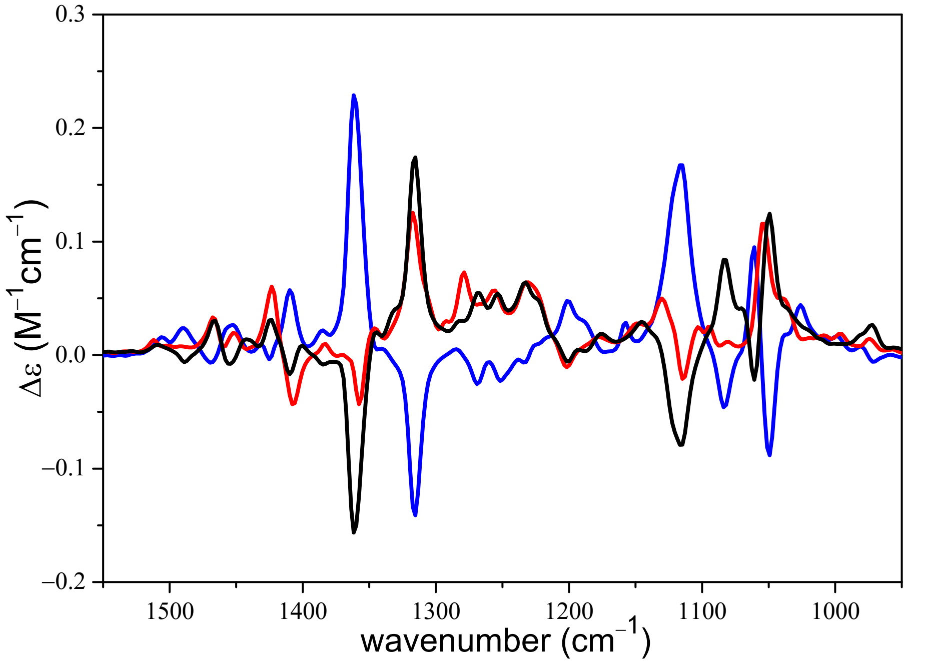

Figure S172. Overlapped experimental VCD spectra of stereoisomeric (a*S*,1*S*,3*S*,3'*S*)-**21** (blue), (a*R*,1*R*,3*S*,3'*S*)-**21** (red) and (a*R*,1*R*,3*R*,3'*R*)-**21** (black) in $CDCl_3$.

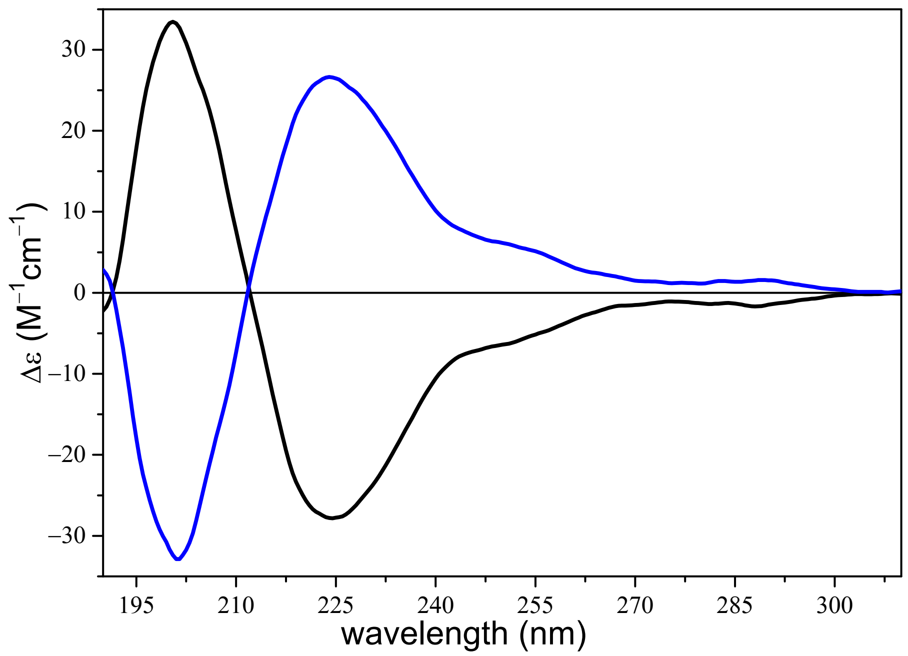

Figure S173. Experimental ECD spectra of enantiomeric (a*S*,1*R*,3*S*,1'*S*,3'*S*)-**22** (black) and (a*R*,1*S*,3*R*,1'*R*,3'*R*)-**22** (blue) in MeCN.

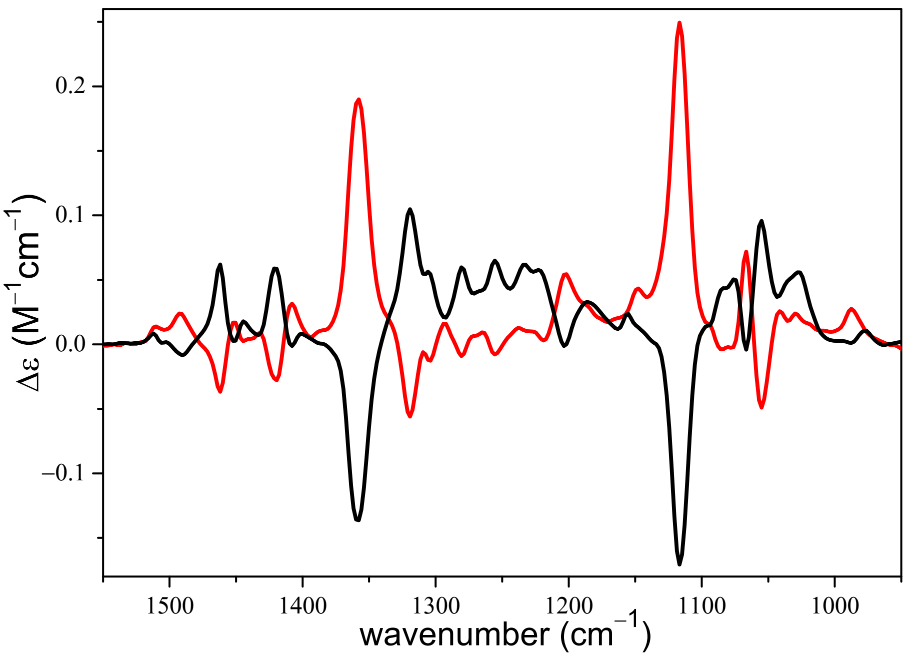

Figure S174. Experimental VCD spectra of enantiomeric (a*S*,1*R*,3*S*,1'*S*,3'*S*)-**22** (red) and (a*R*,1*S*,3*R*,1'*R*,3'*R*)-**22** (black) in $CDCl_3$.

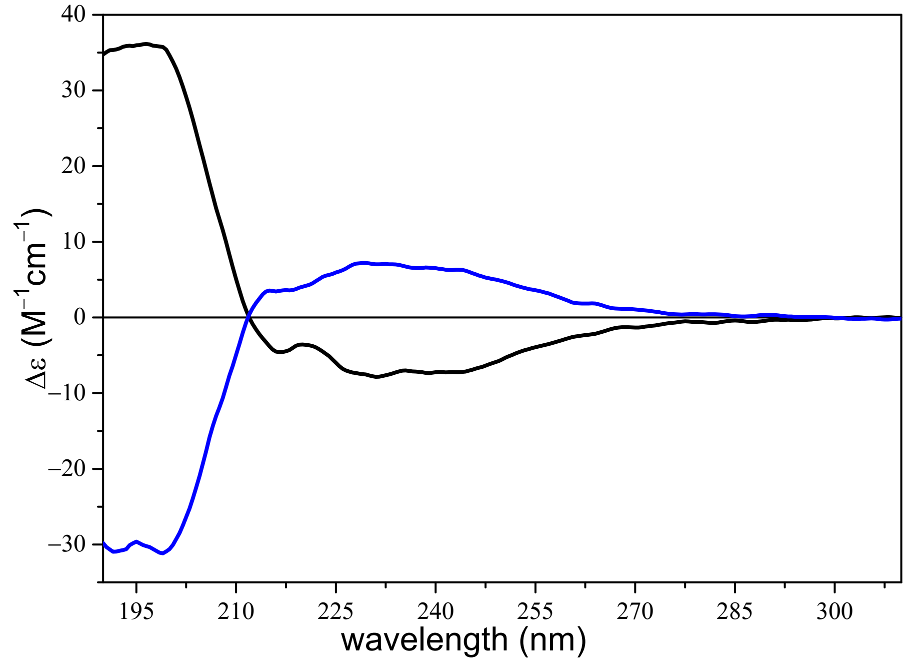

Figure S175. Experimental ECD spectra of enantiomeric (a*S*,1*S*,3*S*,1'*S*,3'*S*)-**22** (black) and (a*R*,1*R*,3*R*,1'*R*,3'*R*)-**22** (blue) in MeCN.

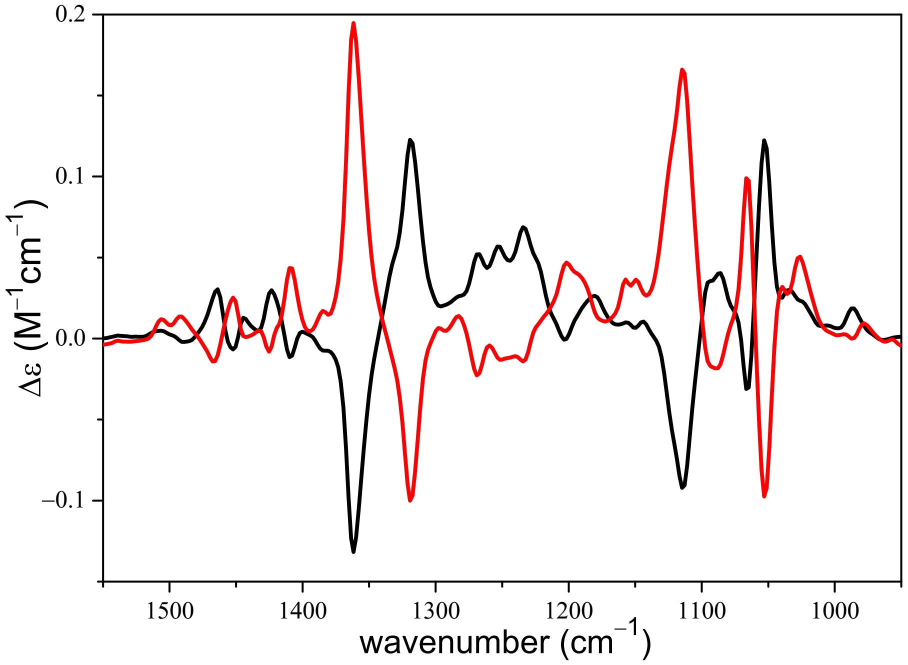

Figure S176. Experimental VCD spectra of enantiomeric (a*S*,1*S*,3*S*,1'*S*,3'*S*)-**22** (red) and (a*R*,1*R*,3*R*,1'*R*,3'*R*)-**22** (black) in $CDCl_3$.

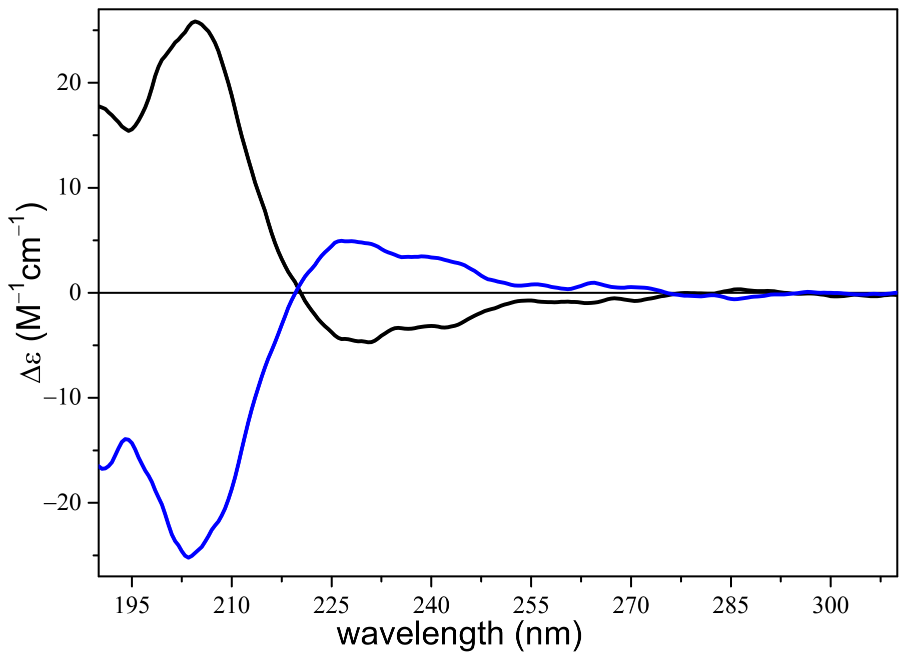

Figure S177. Experimental ECD spectra of enantiomeric (a*R*,1*S*,3*S*,1'*S*,3'*S*)-**22** (black) and (a*S*,1*R*,3*R*,1'*R*,3'*R*)-**22** (blue) in MeCN.

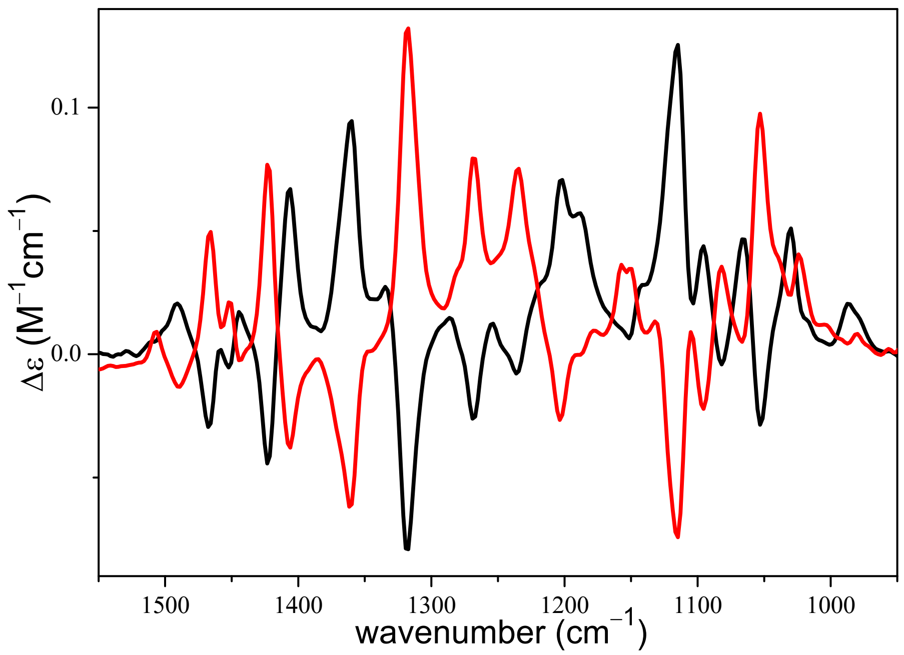

Figure S178. Experimental VCD spectra of enantiomeric (a*R*,1*S*,3*S*,1'*S*,3'*S*)-**22** (red) and (a*S*,1*R*,3*R*,1'*R*,3'*R*)-**22** (black) in $CDCl_3$.

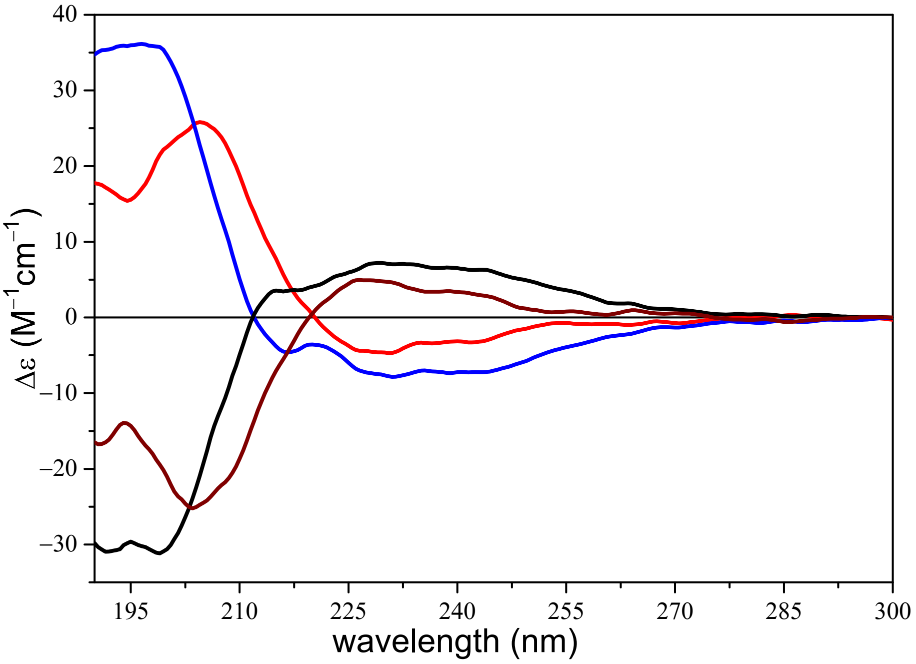

Figure S179. Overlapped experimental ECD spectra of stereoisomeric (a*S*,1*S*,3*S*,1'*S*,3'*S*)-**22** (blue), (a*R*,1*S*,3*S*,1'*S*,3'*S*)-**22** (red), (a*R*,1*R*,3*R*,1'*R*,3'*R*)-**22** (black) and (a*S*,1*R*,3*R*,1'*R*,3'*R*)-**22** (wine) in MeCN.

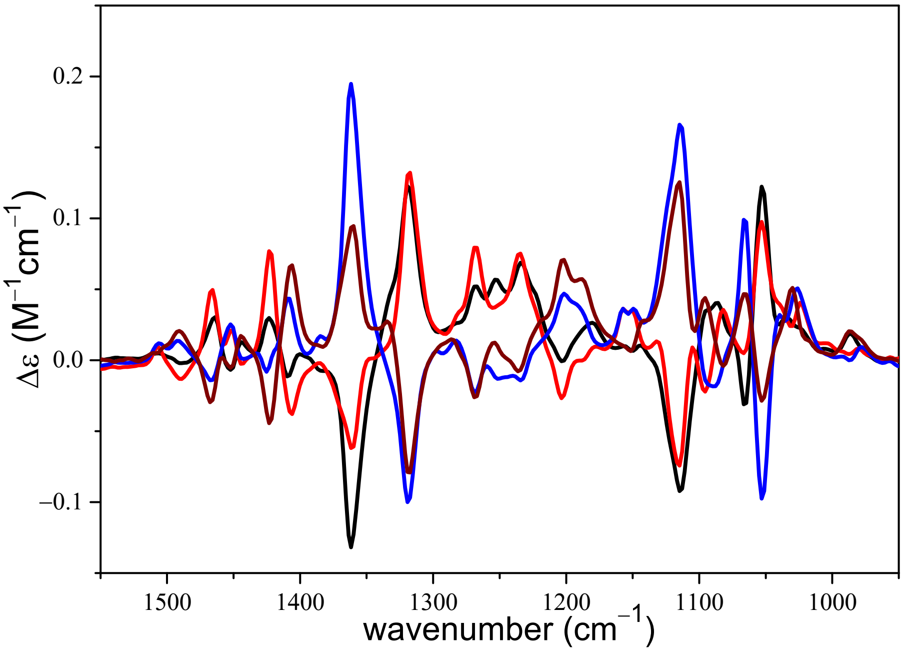

Figure S180. Overlapped experimental VCD spectra of stereoisomeric (a*S*,1*S*,3*S*,1'*S*,3'*S*)-**22** (blue), (a*R*,1*S*,3*S*,1'*S*,3'*S*)-**22** (red), (a*R*,1*R*,3*R*,1'*R*,3'*R*)-**22** (black) and (a*S*,1*R*,3*R*,1'*R*,3'*R*)-**22** (wine) in $CDCl_3$.

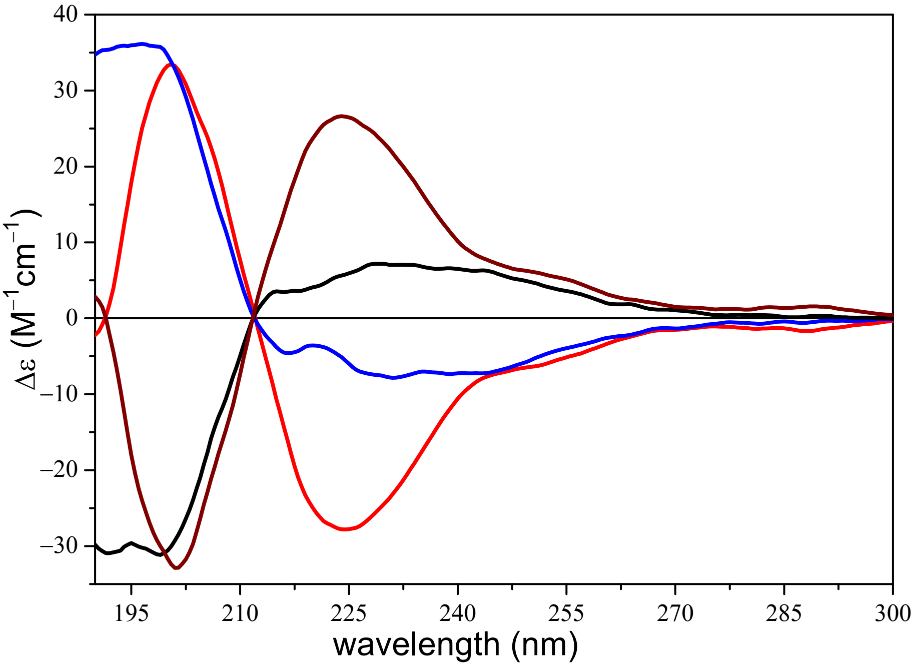

Figure S181. Overlapped experimental ECD spectra of stereoisomeric (a*S*,1*S*,3*S*,1'*S*,3'*S*)-**22** (blue), (a*S*,1*R*,3*S*,1'*S*,3'*S*)-**22** (red), (a*R*,1*R*,3*R*,1'*R*,3'*R*)-**22** (black) and (a*R*,1*S*,3*R*,1'*R*,3'*R*)-**22** (wine) in MeCN.

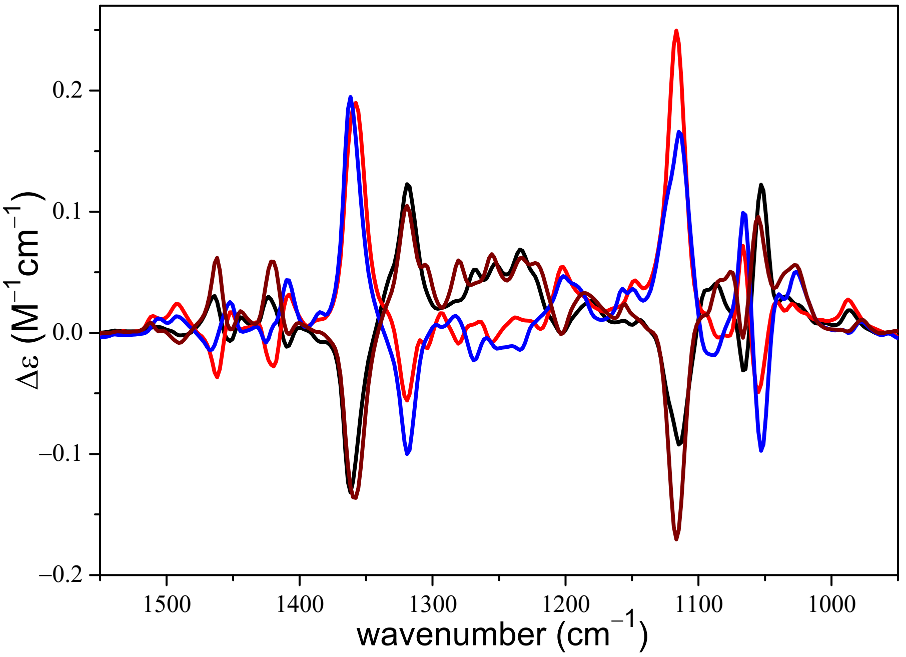

Figure S182. Overlapped experimental VCD spectra of stereoisomeric (a*S*,1*S*,3*S*,1'*S*,3'*S*)-**22** (blue), (a*S*,1*R*,3*S*,1'*S*,3'*S*)-**22** (red), (a*R*,1*R*,3*R*,1'*R*,3'*R*)-**22** (black) and (a*R*,1*S*,3*R*,1'*R*,3'*R*)-**22** (wine) in $CDCl_3$.

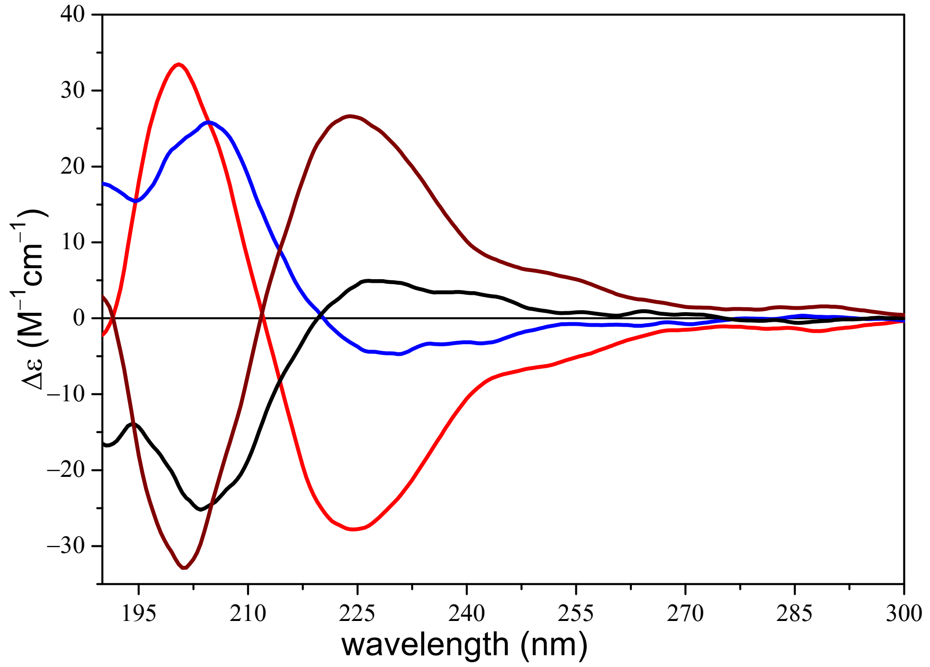

Figure S183. Overlapped experimental ECD spectra of stereoisomeric (a*R*,1*S*,3*S*,1'*S*,3'*S*)-**22** (blue), (a*S*,1*R*,3*S*,1'*S*,3'*S*)-**22** (red), (a*S*,1*R*,3*R*,1'*R*,3'*R*)-**22** (black) and (a*R*,1*S*,3*R*,1'*R*,3'*R*)-**22** (wine) in MeCN.

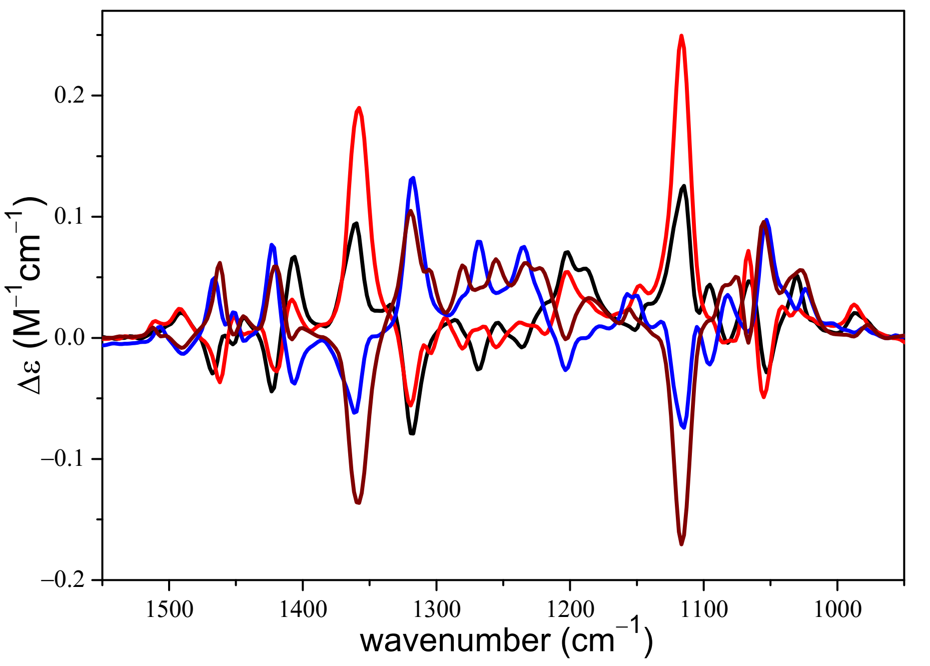

Figure S184. Overlapped experimental VCD spectra of stereoisomeric (a*R*,1*S*,3*S*,1'*S*,3'*S*)-**22** (blue), (a*S*,1*R*,3*S*,1'*S*,3'*S*)-**22** (red), (a*S*,1*R*,3*R*,1'*R*,3'*R*)-**22** (black) and (a*R*,1*S*,3*R*,1'*R*,3'*R*)-**22** (wine) in $CDCl_3$.

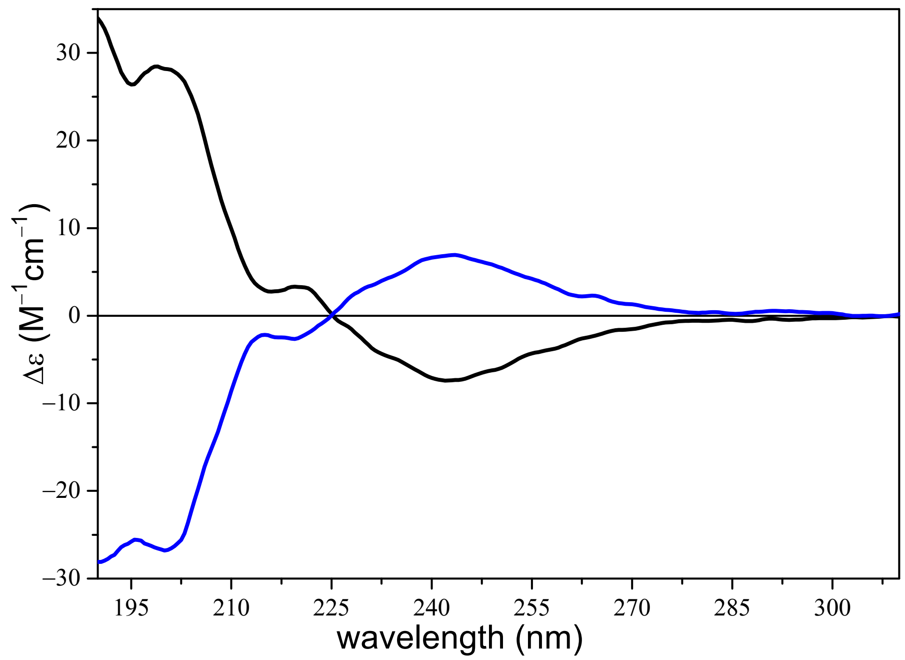

Figure S185. Experimental ECD spectra of enantiomeric (a*S*,1*S*,3*S*,1'*R*,3'*S*)-**23** (black) and (a*R*,1*R*,3*R*,1'*S*,3'*R*)-**23** (blue) in MeCN.

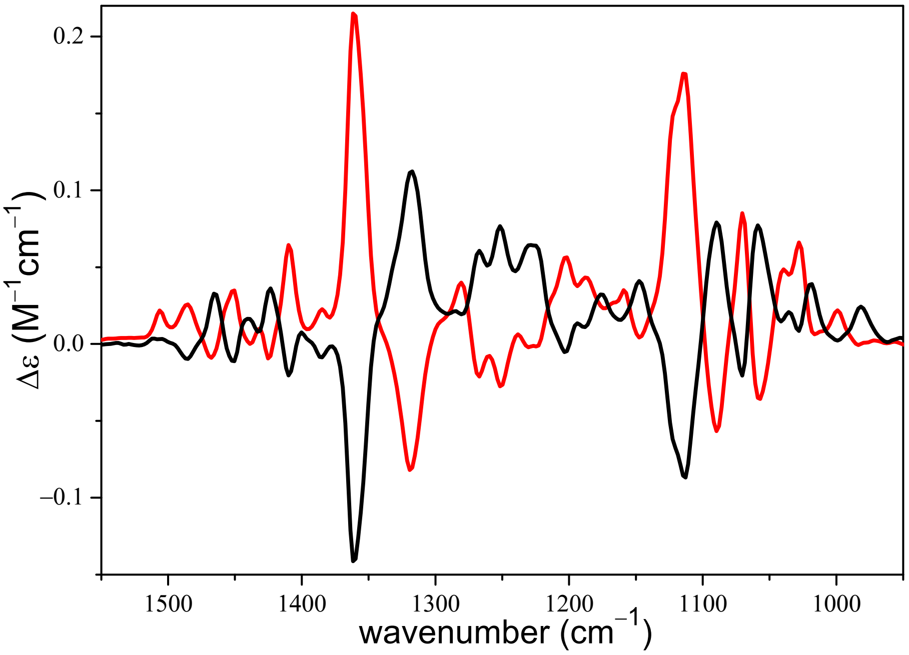

Figure S186. Experimental VCD spectra of enantiomeric (a*S*,1*S*,3*S*,1'*R*,3'*S*)-**23** (red) and (a*R*,1*R*,3*R*,1'*S*,3'*R*)-**23** (black) in $CDCl_3$.

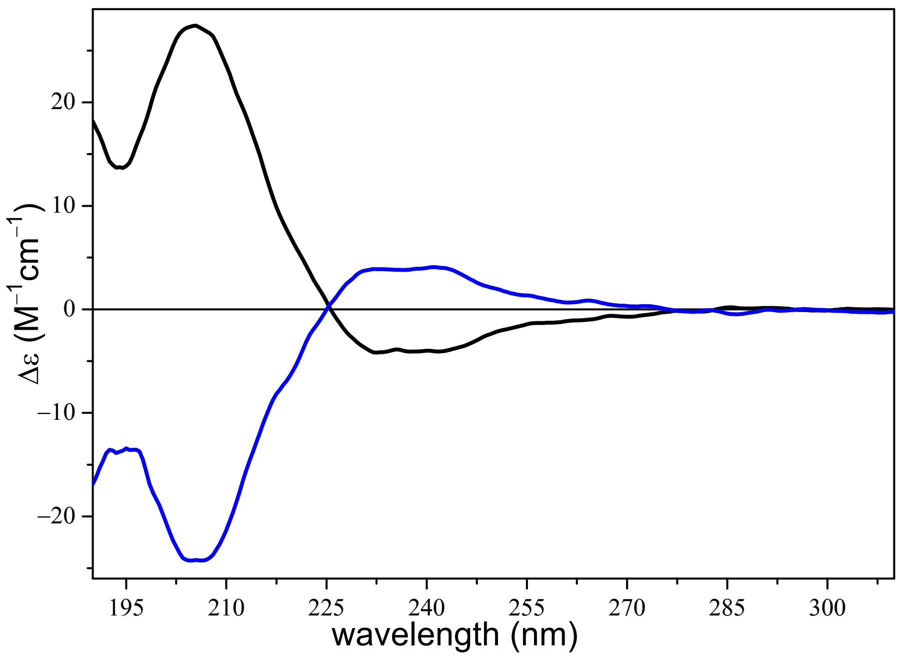

Figure S187. Experimental ECD spectra of enantiomeric (a*R*,1*S*,3*S*,1'*R*,3'*S*)-**23** (black) and (a*S*,1*R*,3*R*,1'*S*,3'*R*)-**23** (blue) in MeCN.

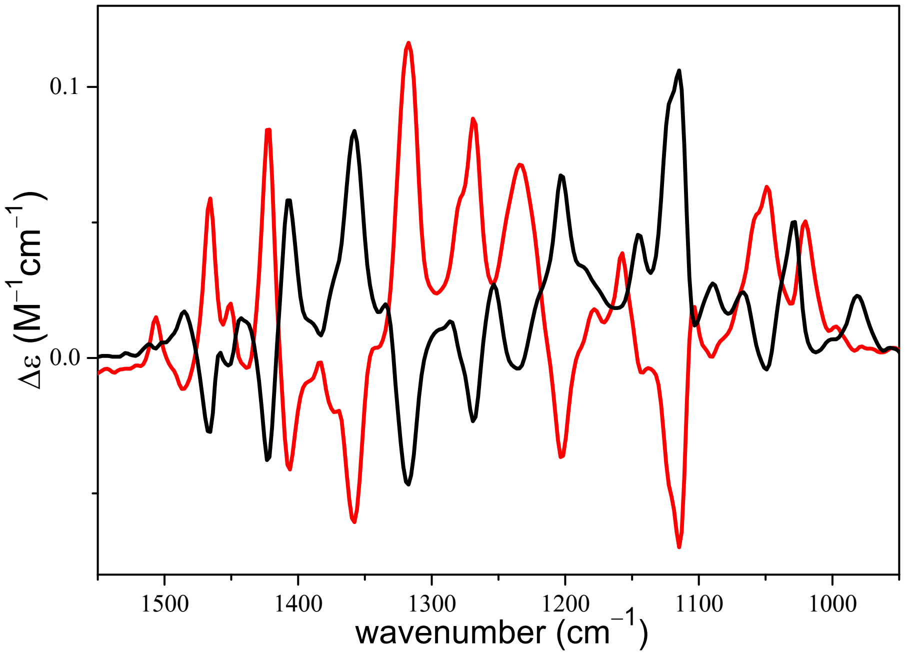

Figure S188. Experimental VCD spectra of enantiomeric (a*R*,1*S*,3*S*,1'*R*,3'*S*)-**23** (red) and (a*S*,1*R*,3*R*,1'*S*,3'*R*)-**23** (black) in $CDCl_3$.

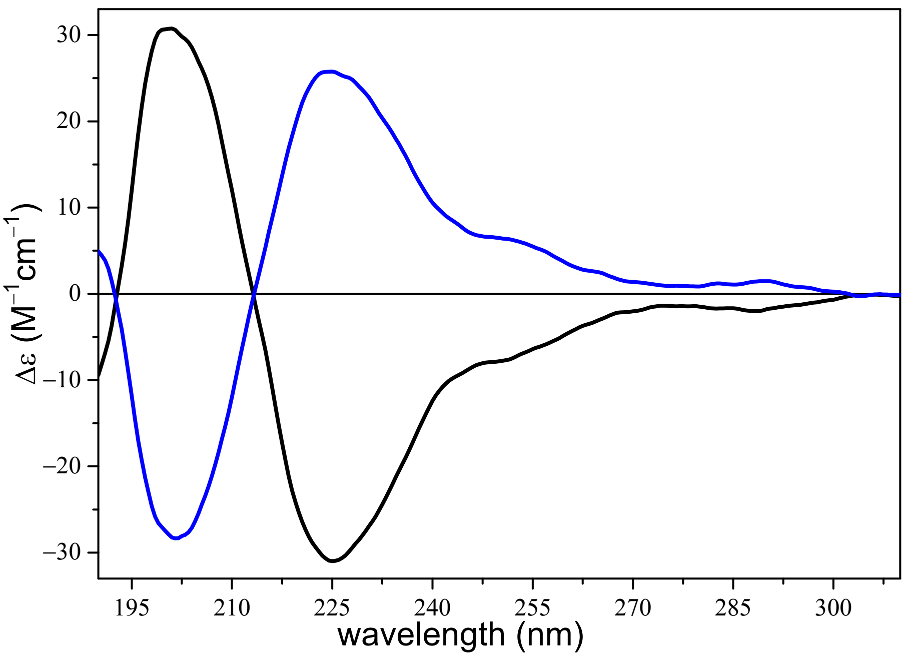

Figure S189. Experimental ECD spectra of enantiomeric (a*S*,1*R*,3*S*,1'*R*,3'*S*)-**23** (black) and (a*R*,1*S*,3*R*,1'*S*,3'*R*)-**23** (blue) in MeCN.

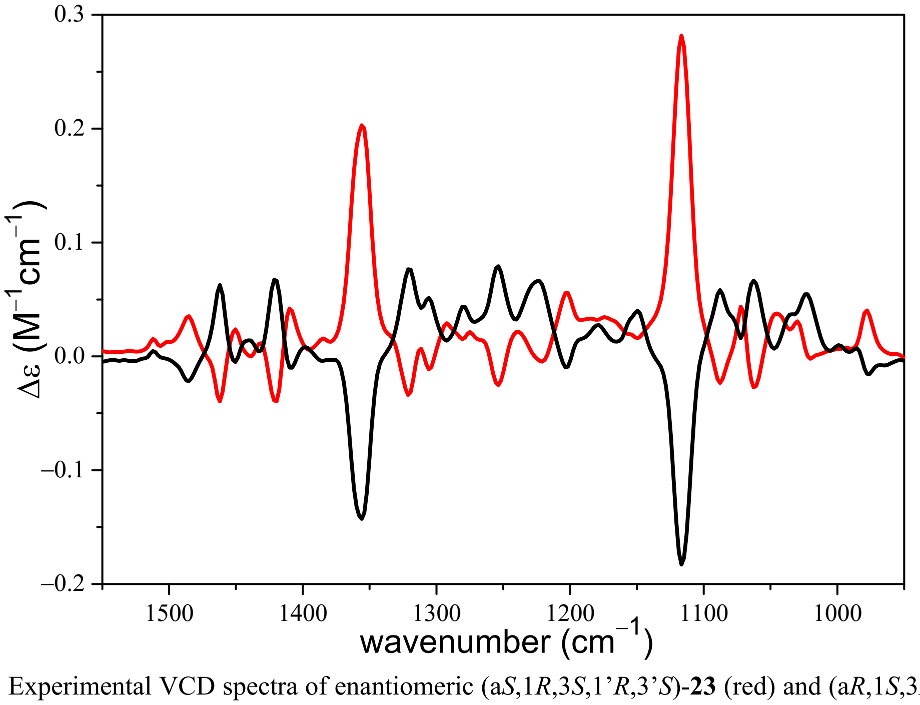

Figure S190. Experimental VCD spectra of enantiomeric (a*S*,1*R*,3*S*,1'*R*,3'*S*)-**23** (red) and (a*R*,1*S*,3*R*,1'*S*,3'*R*)-**23** (black) in $CDCl_3$.

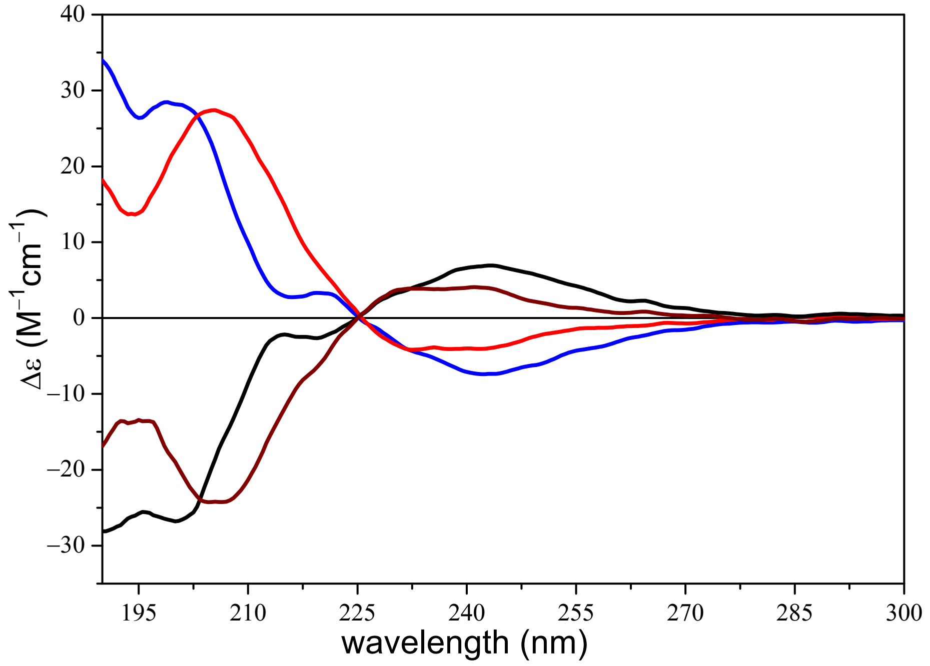

Figure S191. Overlapped experimental ECD spectra of stereoisomeric (a*S*,1*S*,3*S*,1'*R*,3'*S*)-**23** (blue), (a*R*,1*S*,3*S*,1'*R*,3'*S*)-**23** (red), (a*R*,1*R*,3*R*,1'*S*,3'*R*)-**23** (black) and (a*S*,1*R*,3*R*,1'*S*,3'*R*)-**23** (wine) in MeCN.

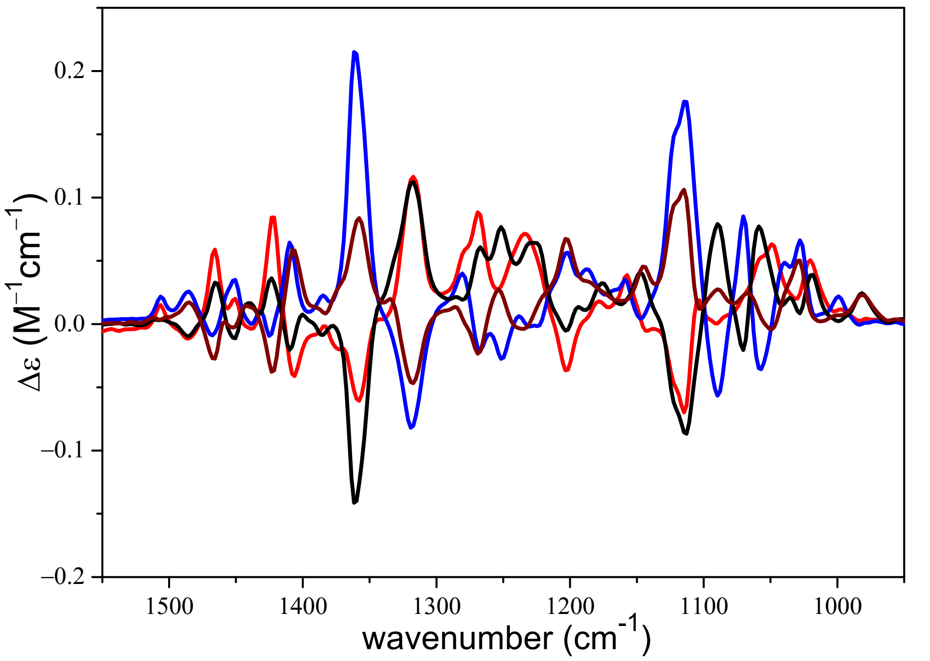

Figure S192. Overlapped experimental VCD spectra of stereoisomeric (a*S*,1*S*,3*S*,1'*R*,3'*S*)-**23** (blue), (a*R*,1*S*,3*S*,1'*R*,3'*S*)-**23** (red), (a*R*,1*R*,3*R*,1'*S*,3'*R*)-**23** (black) and (a*S*,1*R*,3*R*,1'*S*,3'*R*)-**23** (wine) in $CDCl_3$.

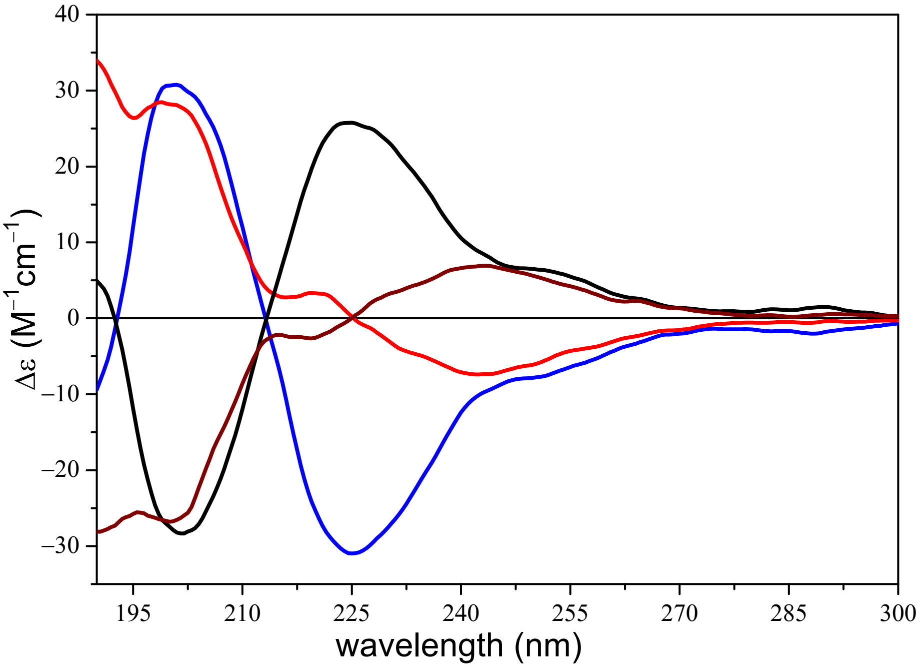

Figure S193. Overlapped experimental ECD spectra of stereoisomeric (a*S*,1*R*,3*S*,1'*R*,3'*S*)-**23** (blue), (a*S*,1*S*,3*S*,1'*R*,3'*S*)-**23** (red), (a*R*,1*S*,3*R*,1'*S*,3'*R*)-**23** (black) and (a*R*,1*R*,3*R*,1'*S*,3'*R*)-**23** (wine) in MeCN.

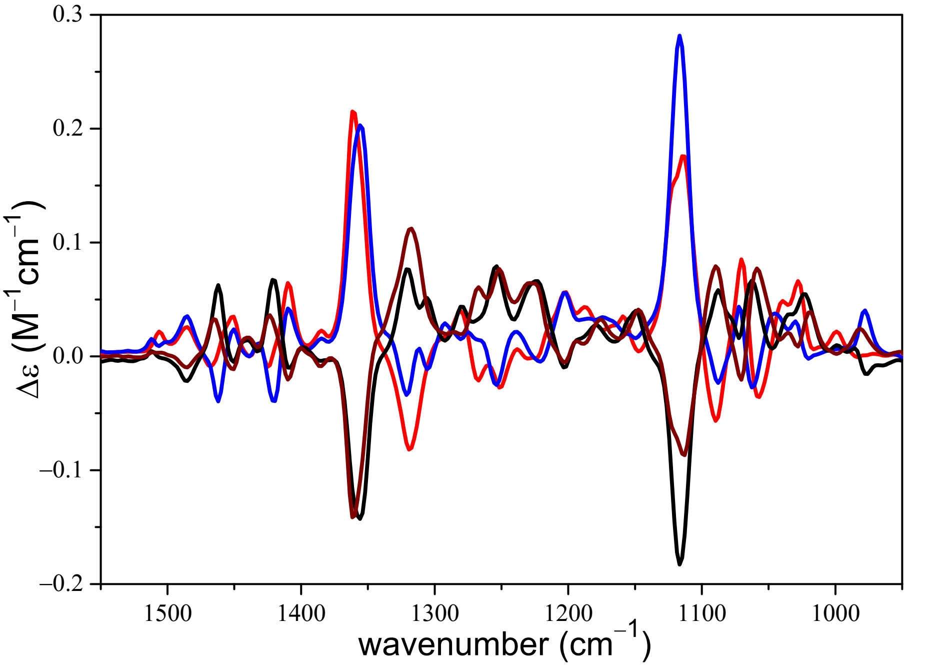

Figure S194. Overlapped experimental VCD spectra of stereoisomeric (a*S*,1*R*,3*S*,1'*R*,3'*S*)-**23** (blue), (a*S*,1*S*,3*S*,1'*R*,3'*S*)-**23** (red), (a*R*,1*S*,3*R*,1'*S*,3'*R*)-**23** (black) and (a*R*,1*R*,3*R*,1'*S*,3'*R*)-**23** (wine) in $CDCl_3$.

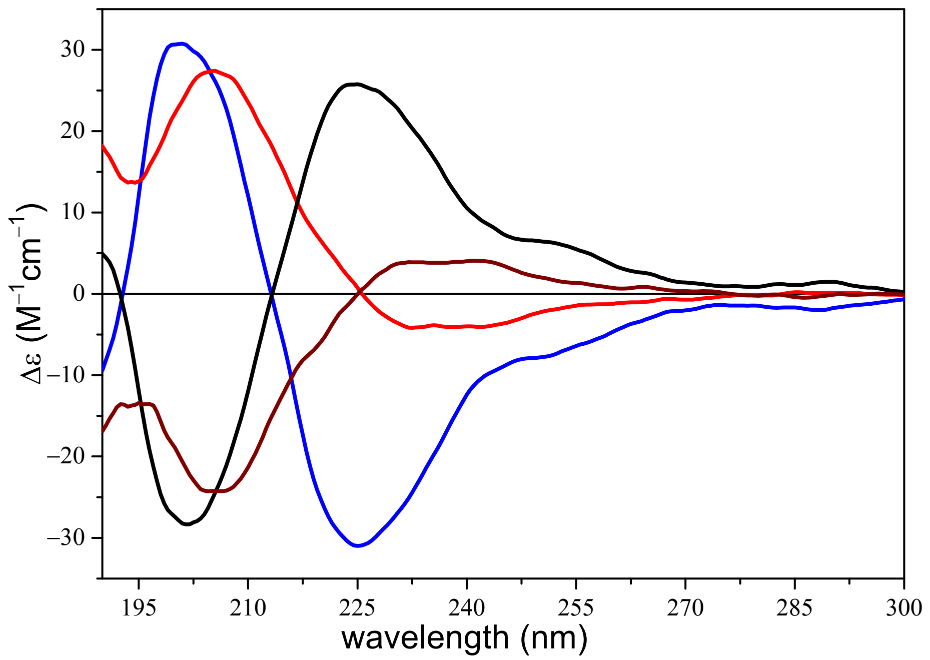

Figure S195. Overlapped experimental ECD spectra of stereoisomeric (a*S*,1*R*,3*S*,1'*R*,3'*S*)-**23** (blue), (a*R*,1*S*,3*S*,1'*R*,3'*S*)-**23** (red), (a*R*,1*S*,3*R*,1'*S*,3'*R*)-**23** (black) and (a*S*,1*R*,3*R*,1'*S*,3'*R*)-**23** (wine) in MeCN.

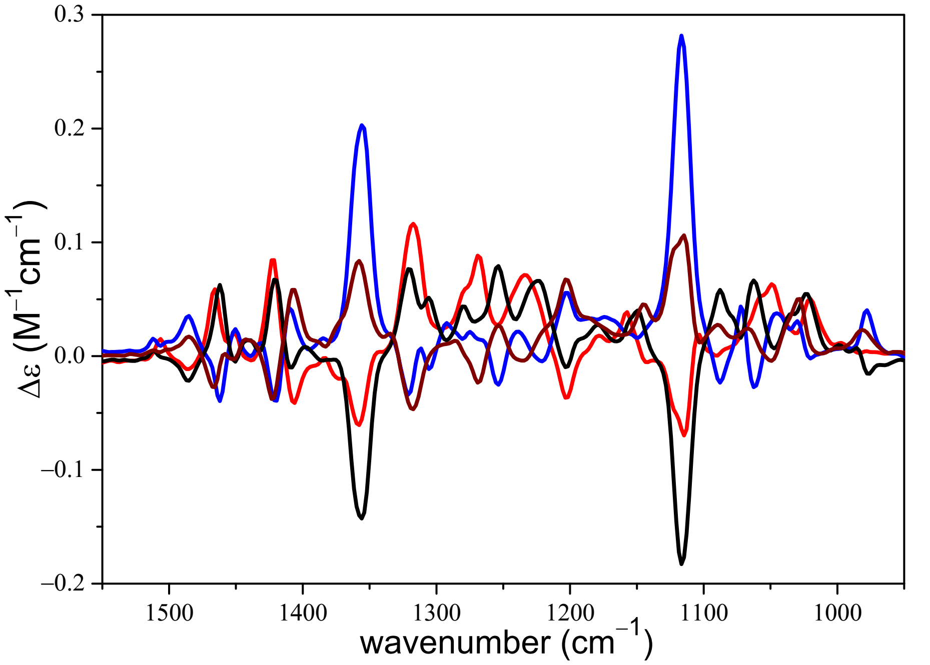

Figure S196. Overlapped experimental VCD spectra of stereoisomeric (a*S*,1*R*,3*S*,1'*R*,3'*S*)-**23** (blue), (a*R*,1*S*,3*S*,1'*R*,3'*S*)-**23** (red), (a*R*,1*S*,3*R*,1'*S*,3'*R*)-**23** (black) and (a*S*,1*R*,3*R*,1'*S*,3'*R*)-**23** (wine) in $CDCl_3$.

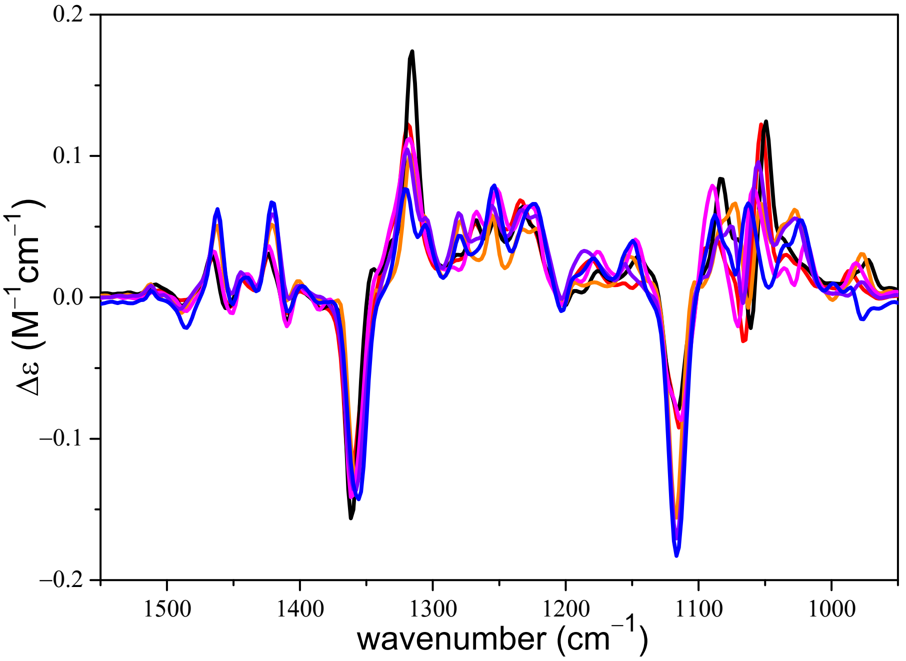

Figure S197. Overlapped experimental VCD spectra of stereoisomeric (a*R*,1*R*,3*R*,3'*R*)-**21** (black), (a*R*,1*S*,3*R*,3'*R*)-**21** (orange), (a*R*,1*S*,3*R*,1'*R*,3'*R*)-**22** (violet), (a*R*,1*R*,3*R*,1'*R*,3'*R*)-**22** (red), (a*R*,1*S*,3*R*,1'*S*,3'*R*)-**23** (blue) and (a*R*,1*R*,3*R*,1'*S*,3'*R*)-**23** (magenta) in $CDCl_3$.

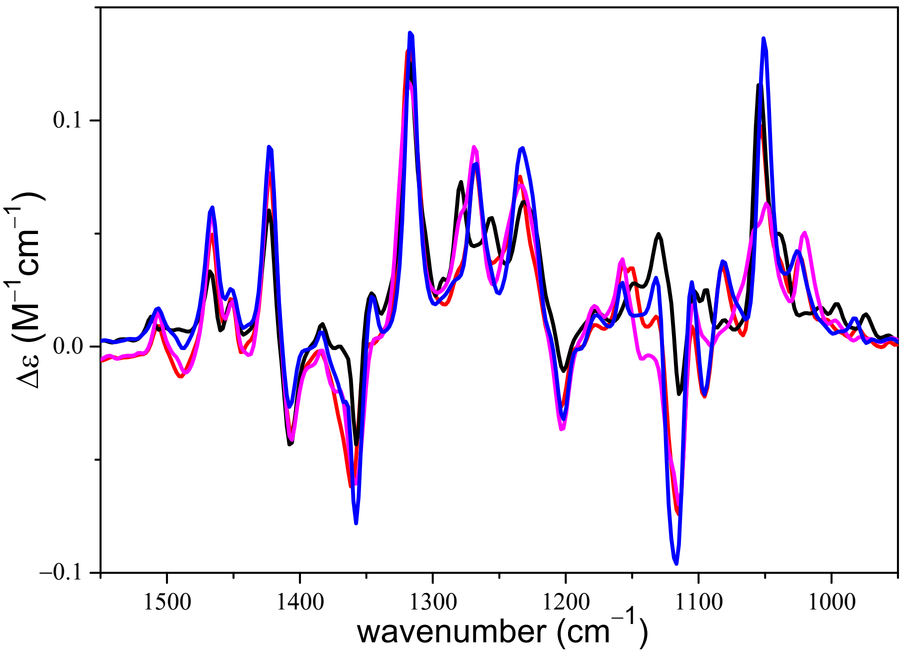

Figure S198. Overlapped experimental VCD spectra of stereoisomeric (a*R*,1*S*,3*S*,3'*S*)-**21** (blue), (a*R*,1*R*,3*S*,3'*S*)-**21** (black), (a*R*,1*S*,3*S*,1'*S*,3'*S*)-**22** (red) and (a*R*,1*S*,3*S*,1'*R*,3'*S*)-**23** (magenta) in $CDCl_3$.

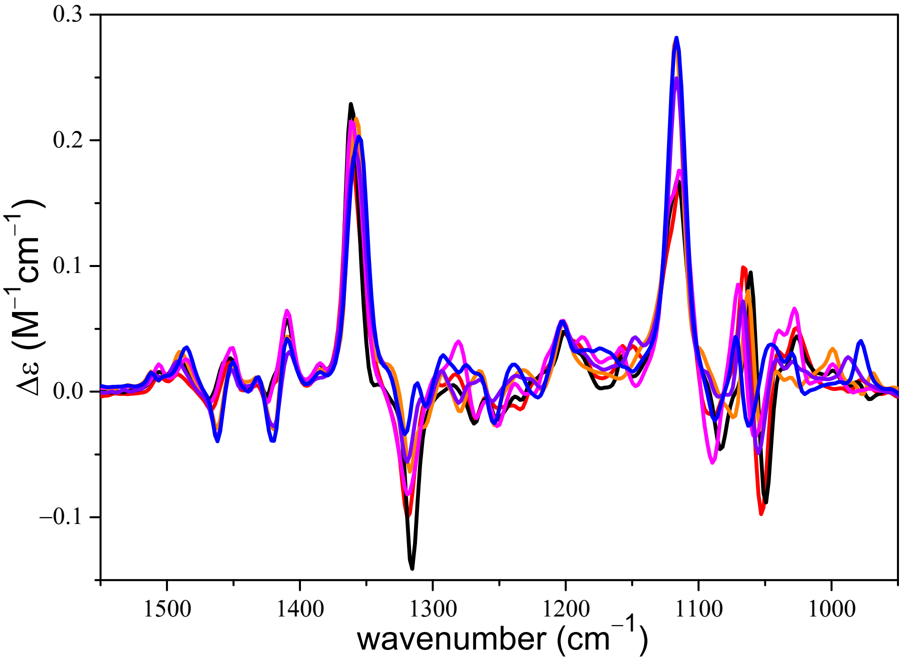

Figure S199. Overlapped experimental VCD spectra of stereoisomeric (a*S*,1*S*,3*S*,3'*S*)-**21** (black), (a*S*,1*R*,3*S*,3'*S*)-**21** (orange), (a*S*,1*R*,3*S*,1'*S*,3'*S*)-**22** (violet), (a*S*,1*S*,3*S*,1'*S*,3'*S*)-**22** (red), (a*S*,1*R*,3*S*,1'*R*,3'*S*)-**23** (blue) and (a*S*,1*S*,3*S*,1'*R*,3'*S*)-**23** (magenta) in $CDCl_3$.

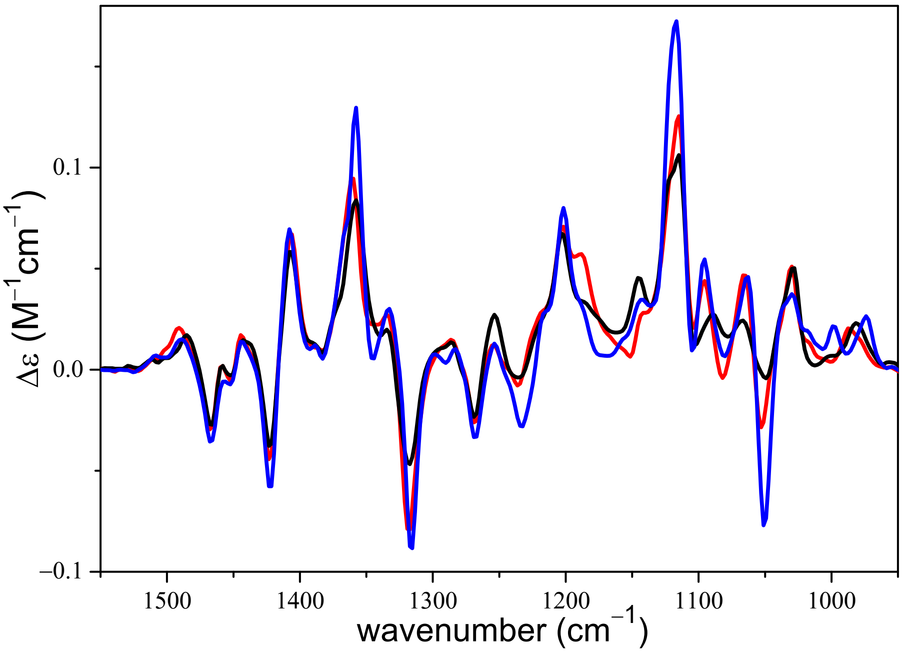

Figure S200. Overlapped experimental VCD spectra of stereoisomeric (a*S*,1*R*,3*R*,3'*R*)-**21** (blue), (a*S*,1*R*,3*R*,1'*R*,3'*R*)-**22** (red) and (a*S*,1*R*,3*R*,1'*S*,3'*R*)-**23** (black) in $CDCl_3$.

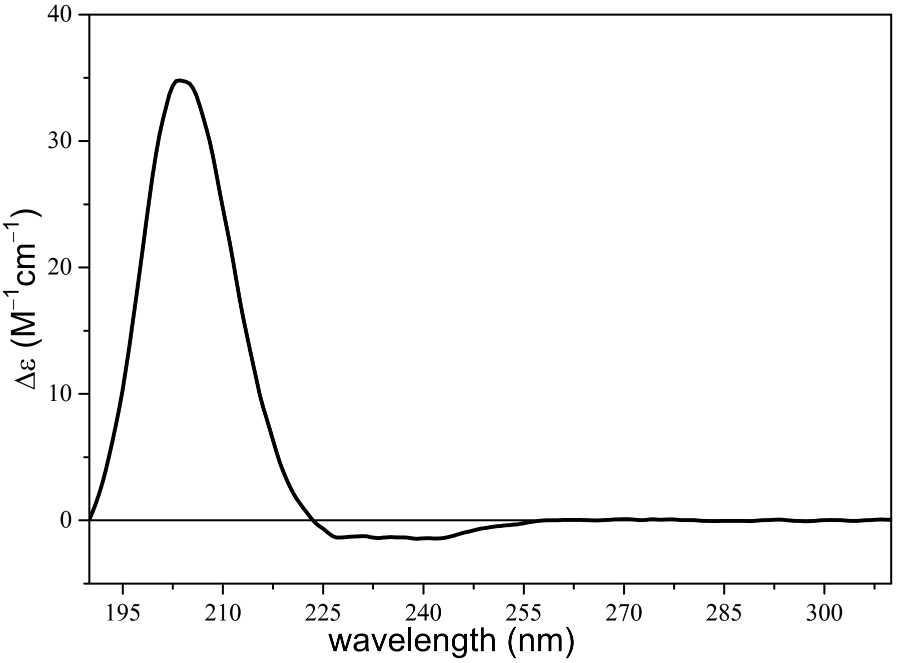

Figure S201. Experimental ECD spectrum of (a*R*,3*S*,3'*S*)-**28** in MeCN.

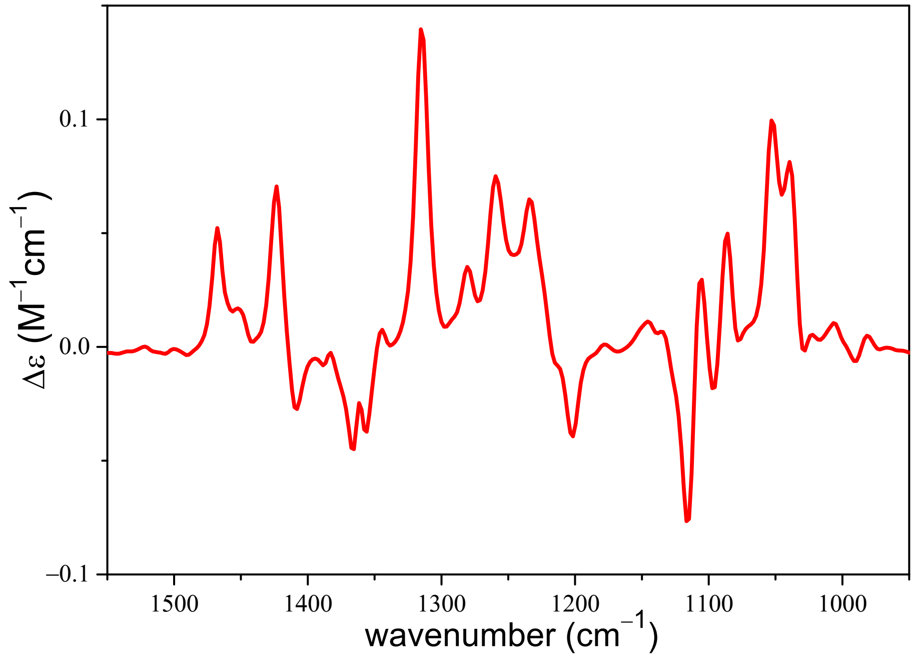

Figure S202. Experimental VCD spectrum of (a*R*,3*S*,3'*S*)-**28** in $CDCl_3$.

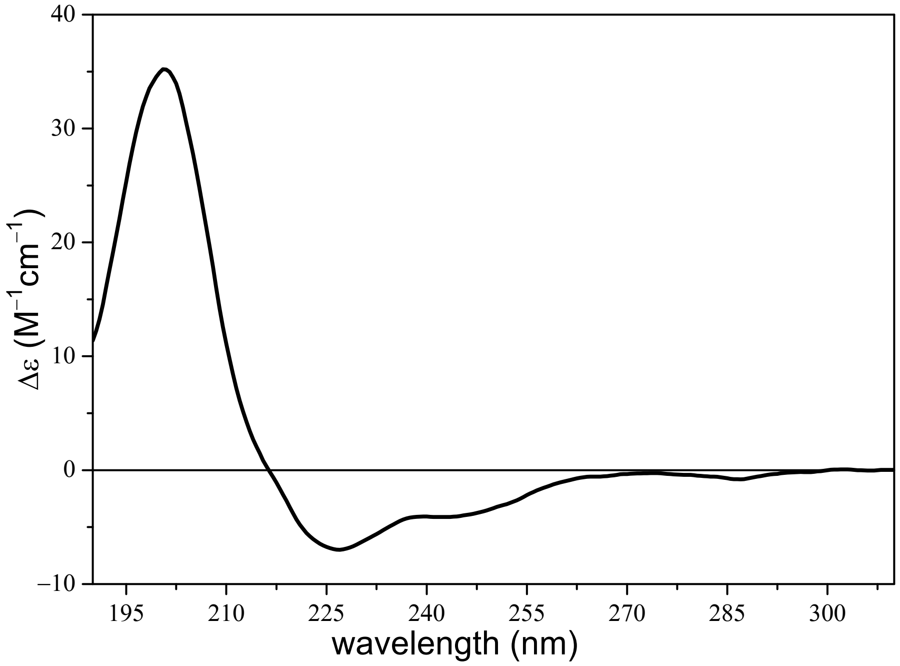

Figure S203. Experimental ECD spectrum of (a*S*,3*S*,3'*S*)-**28** in MeCN.

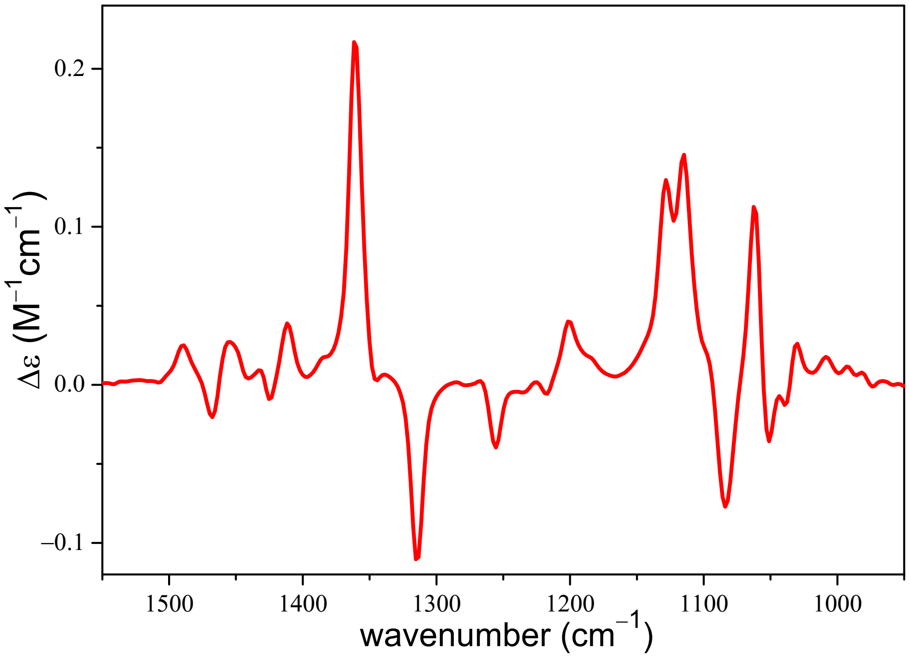

Figure S204. Experimental VCD spectrum of (a*S*,3*S*,3'*S*)-**28** in $CDCl_3$.

3.3. MS spectra

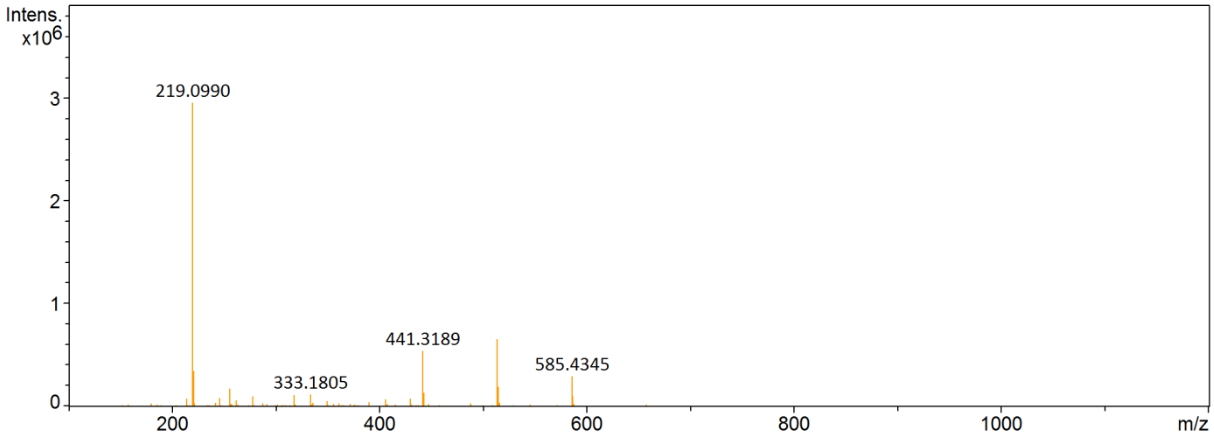

Figure S205. Experimental ESI-HRMS spectrum of (*S*)-**11**.

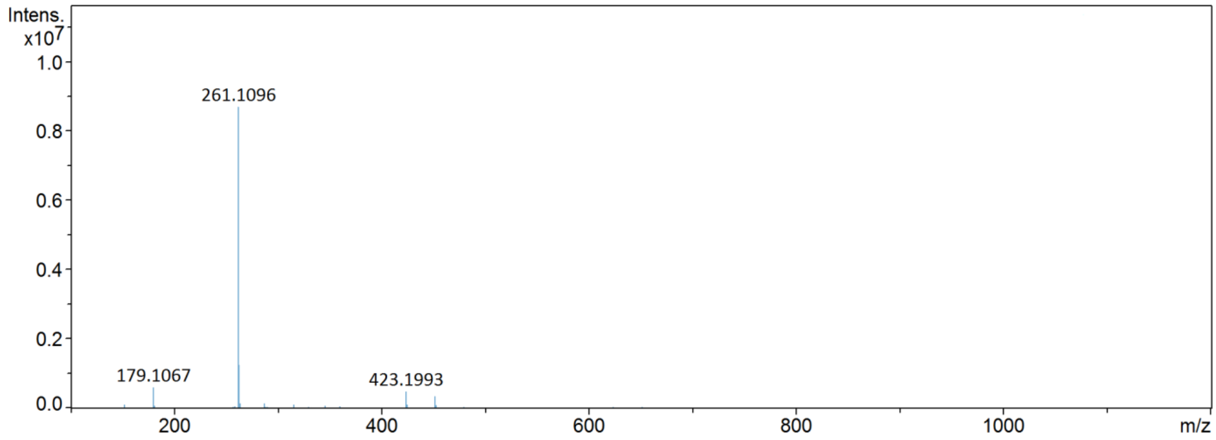

Figure S206. Experimental ESI-HRMS spectrum of (*S*)-**12**.

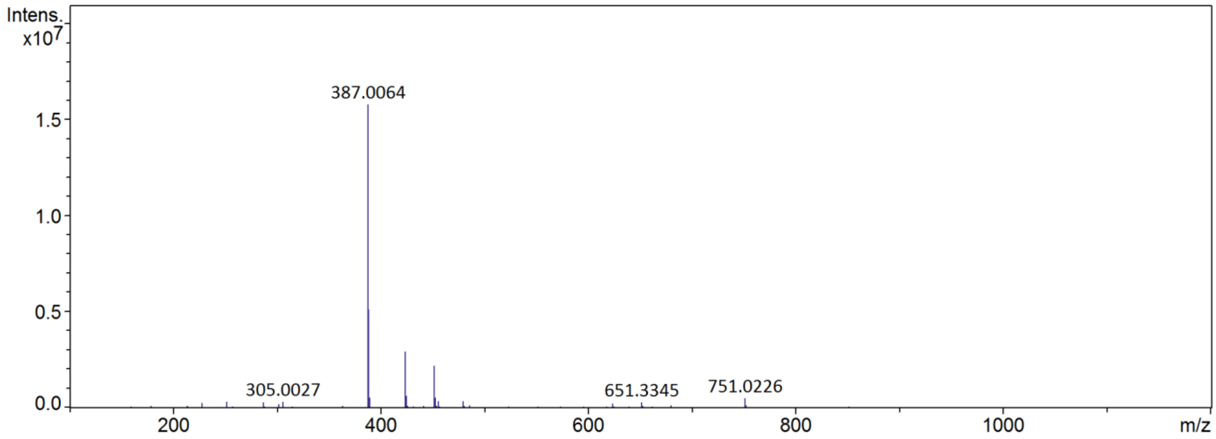

Figure S207. Experimental ESI-HRMS spectrum of (*S*)-**13**.

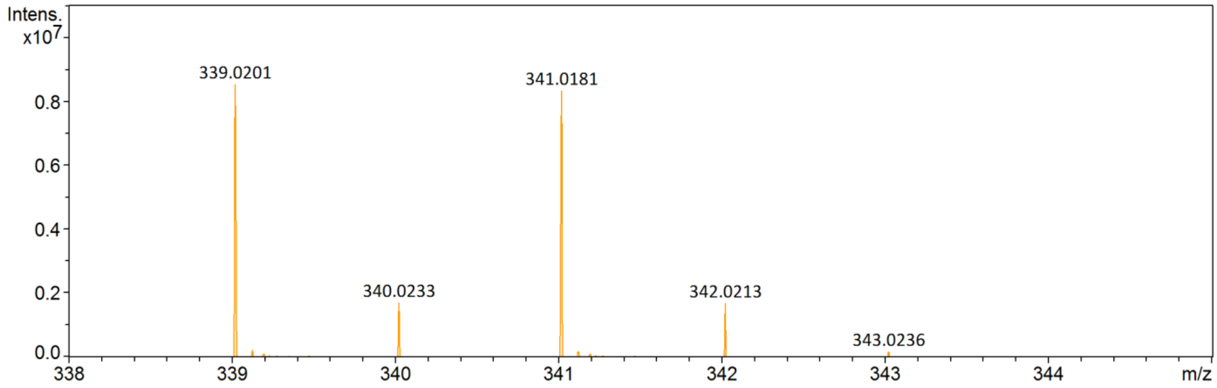

Figure S208. Experimental ESI-HRMS spectrum of (*S*)-**14**.

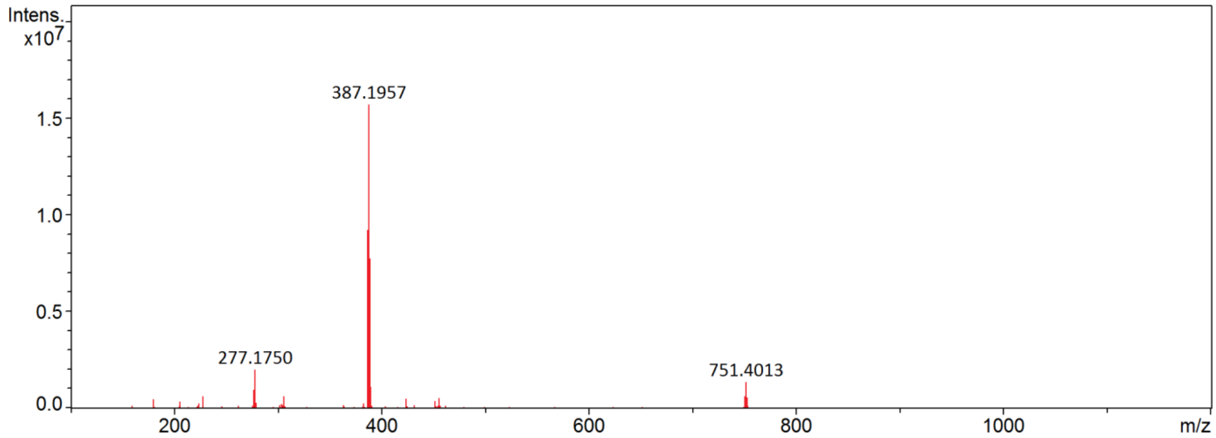

Figure S209. Experimental ESI-HRMS spectrum of (*S*)-**8**.

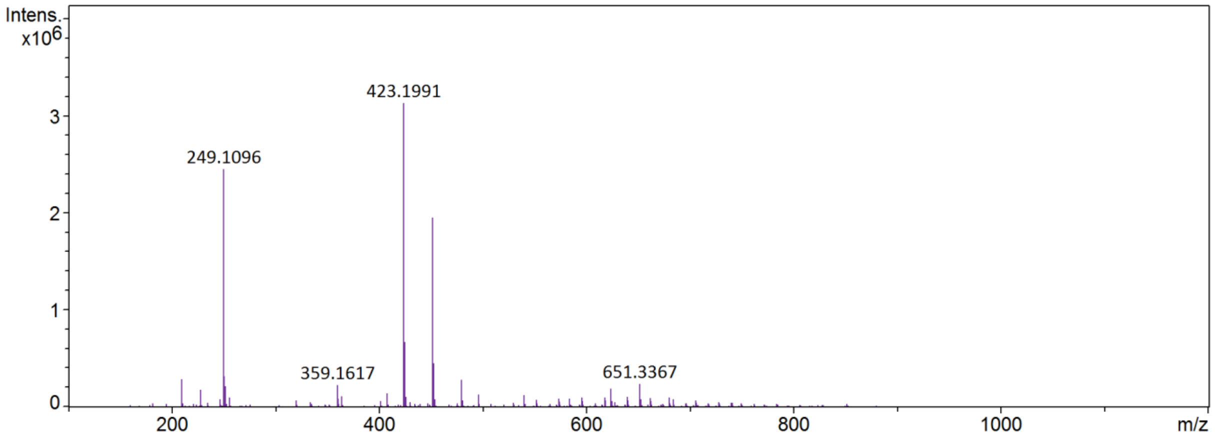

Figure S210. Experimental ESI-HRMS spectrum of (*S*)-**16**.

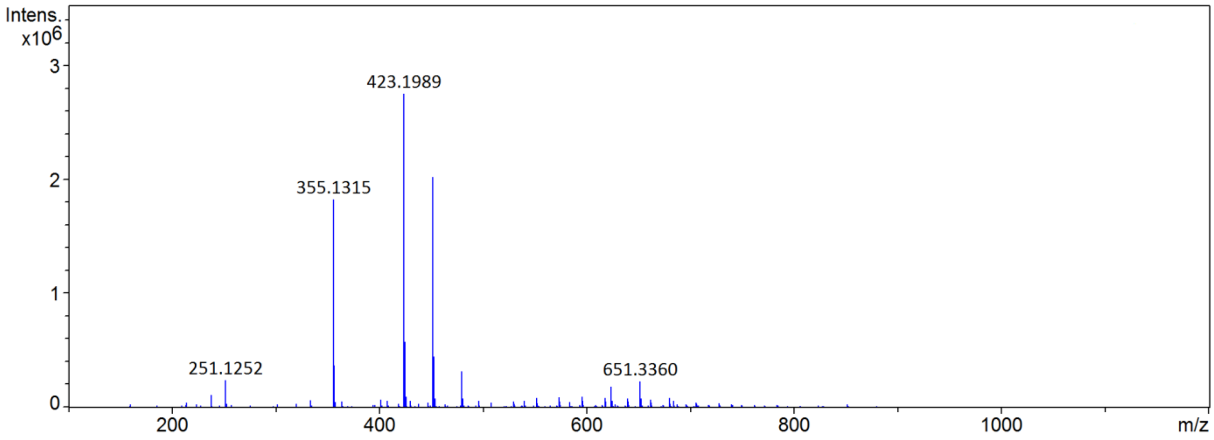

Figure S211. Experimental ESI-HRMS spectrum of *cis*-(1*R*,3*S*)-**17**.

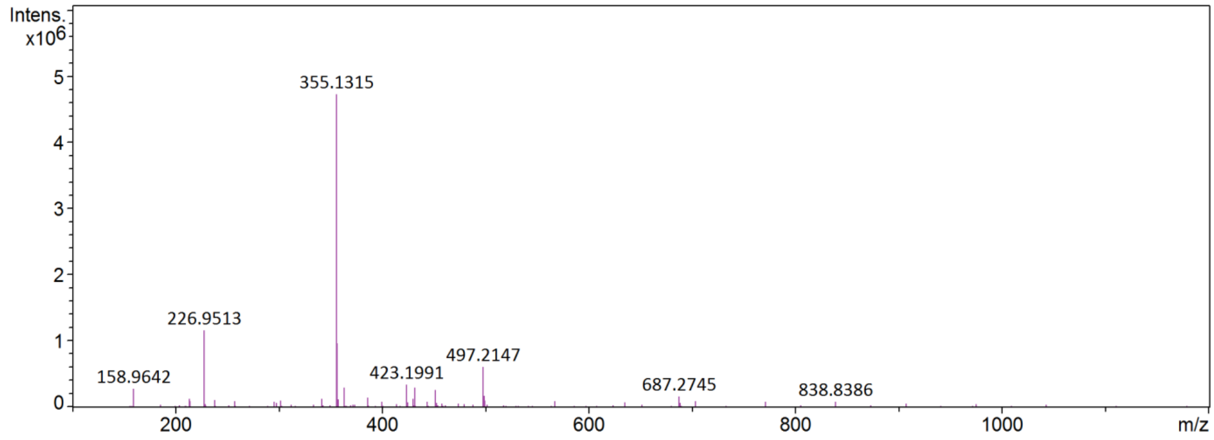

Figure S212. Experimental ESI-HRMS spectrum of *trans*-(1*S*,3*S*)-**17**.

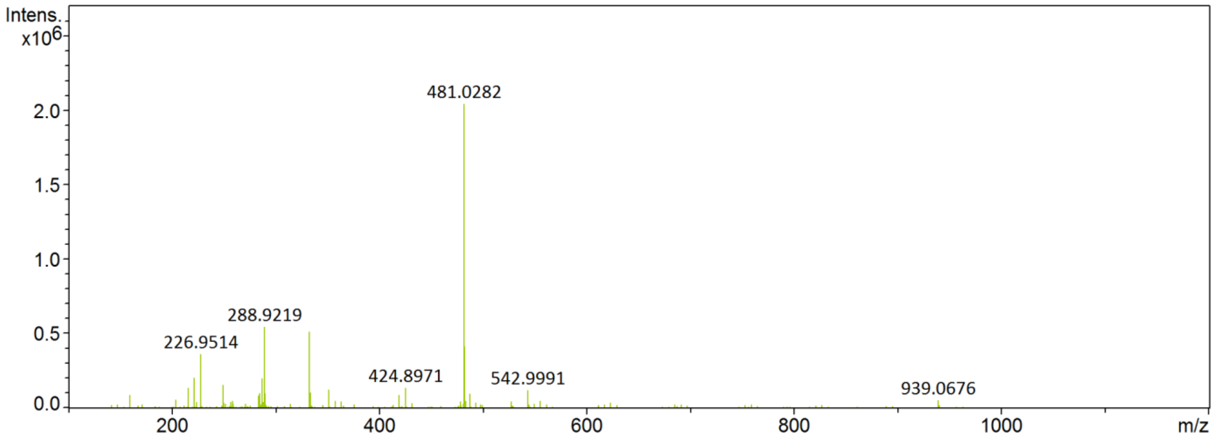

Figure S213. Experimental ESI-HRMS spectrum of *cis*-(1*R*,3*S*)-**18**.

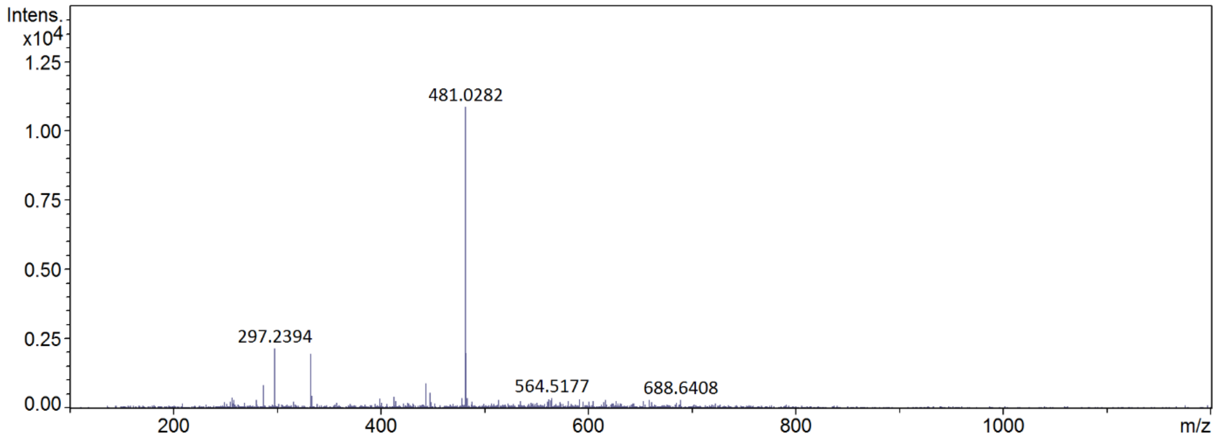

Figure S214. Experimental ESI-HRMS spectrum of *trans*-(1*S*,3*S*)-**18**.

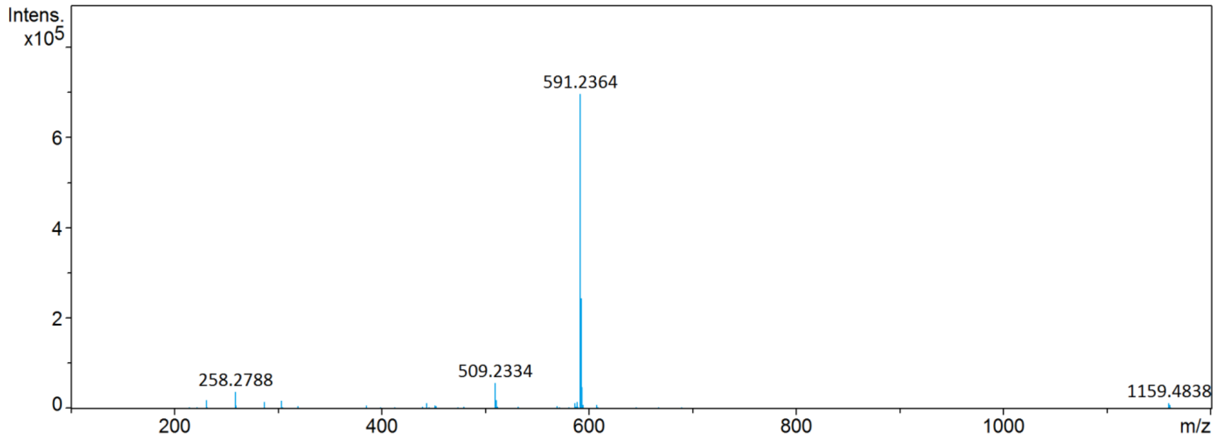

Figure S215. Experimental ESI-HRMS spectrum of the mixture of (a*R*,1*S*,3*S*,2'*S*)-**19** and (a*S*,1*S*,3*S*,2'*S*)-**19**.

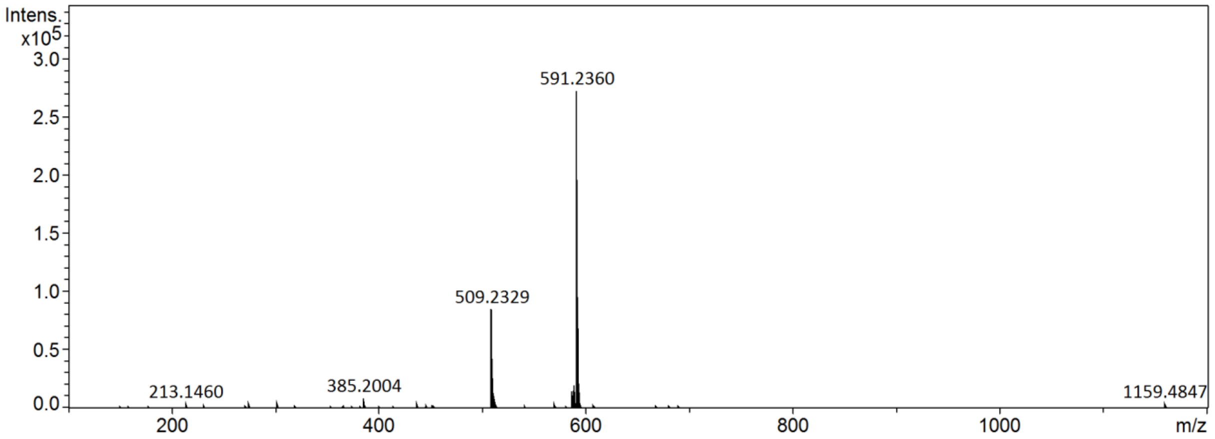

Figure S216. Experimental ESI-HRMS spectrum of the mixture of (a*R*,1*R*,3*S*,2'*S*)-**19** and (a*S*,1*R*,3*S*,2'*S*)-**19**.

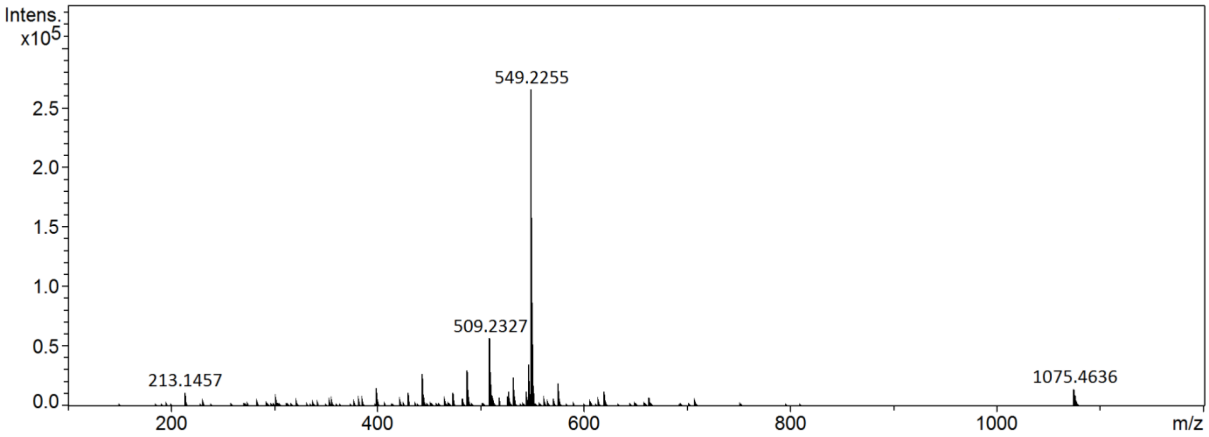

Figure S217. Experimental ESI-HRMS spectrum of (a*S*,1*S*,3*S*,2'*S*)-**20**.

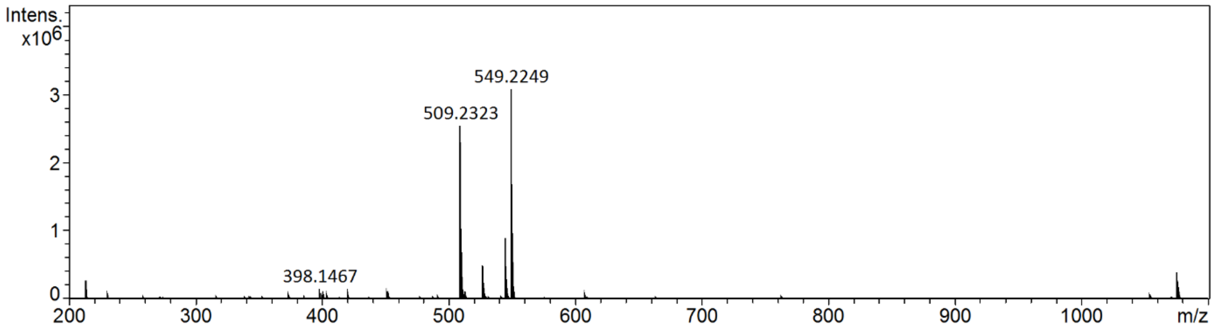

Figure S218. Experimental ESI-HRMS spectrum of (a*R*,1*S*,3*S*,2'*S*)-**20**.

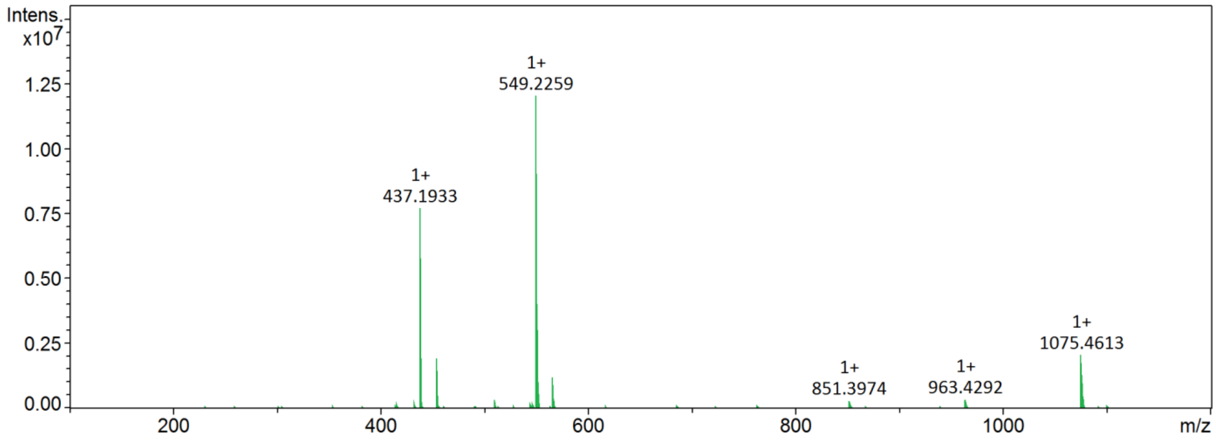

Figure S219. Experimental ESI-HRMS spectrum of (a*S*,1*R*,3*S*,2'*S*)-**20**.

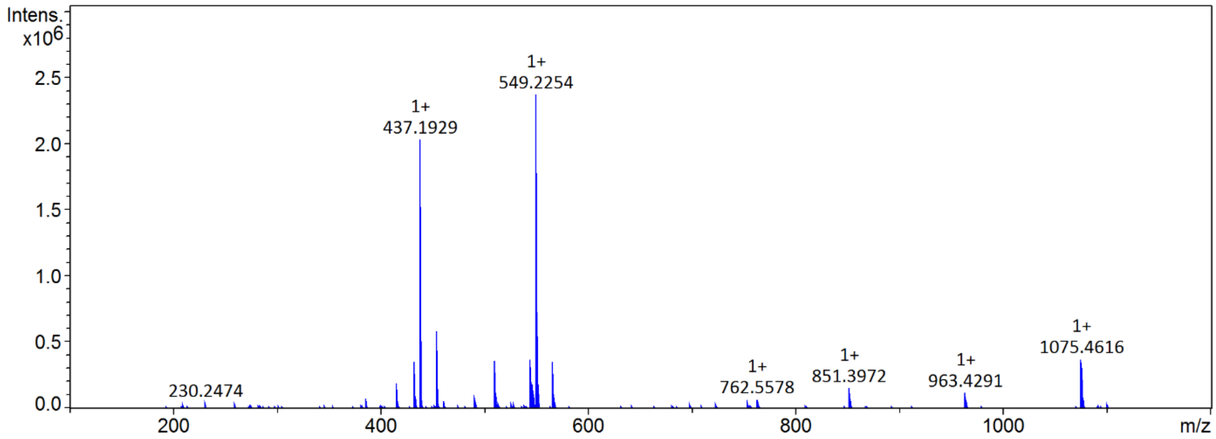

Figure S220. Experimental ESI-HRMS spectrum of (a*R*,1*R*,3*S*,2'*S*)-**20**.

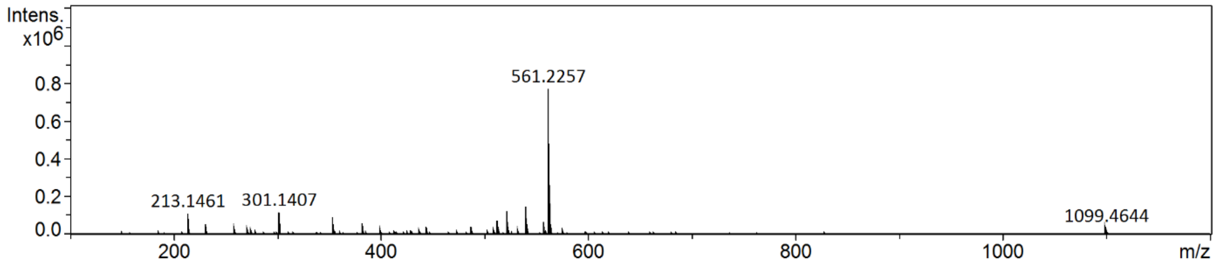

Figure S221. Experimental ESI-HRMS spectrum of (a*S*,1*S*,3*S*,3’*S*)-**21**.

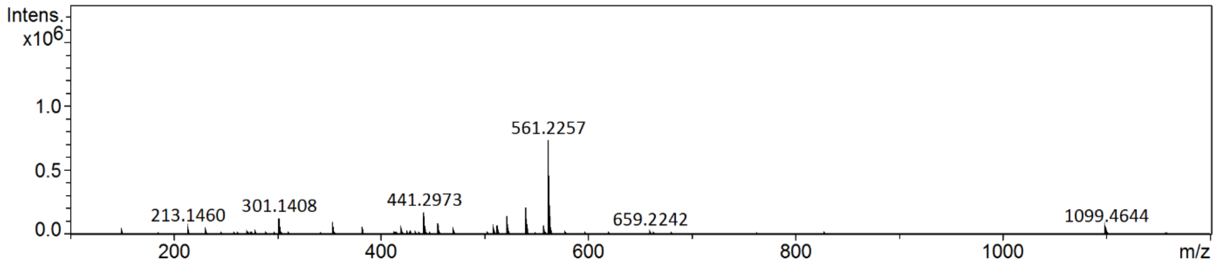

Figure S222. Experimental ESI-HRMS spectrum of (a*R*,1*S*,3*S*,3’*S*)-**21**.

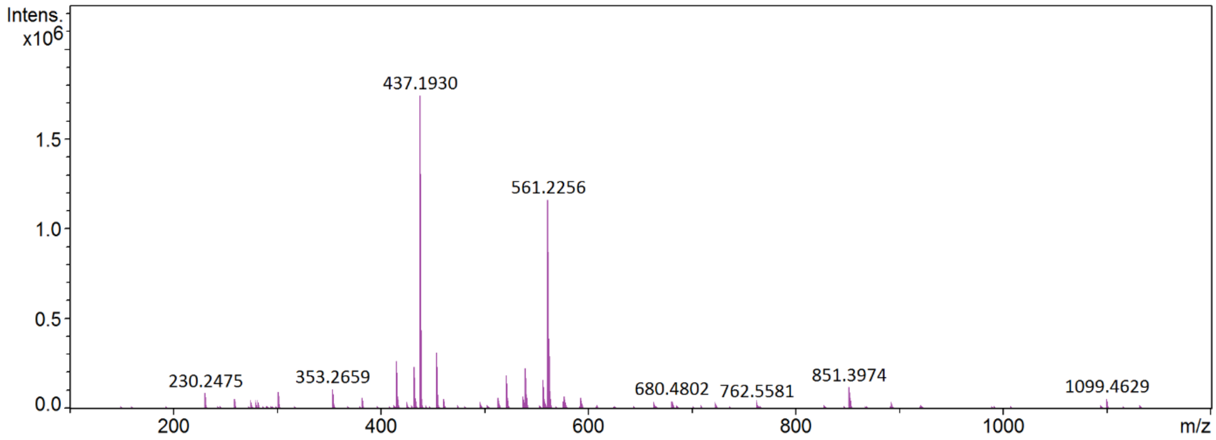

Figure S223. Experimental ESI-HRMS spectrum of (a*S*,1*R*,3*S*,3’*S*)-**21**.

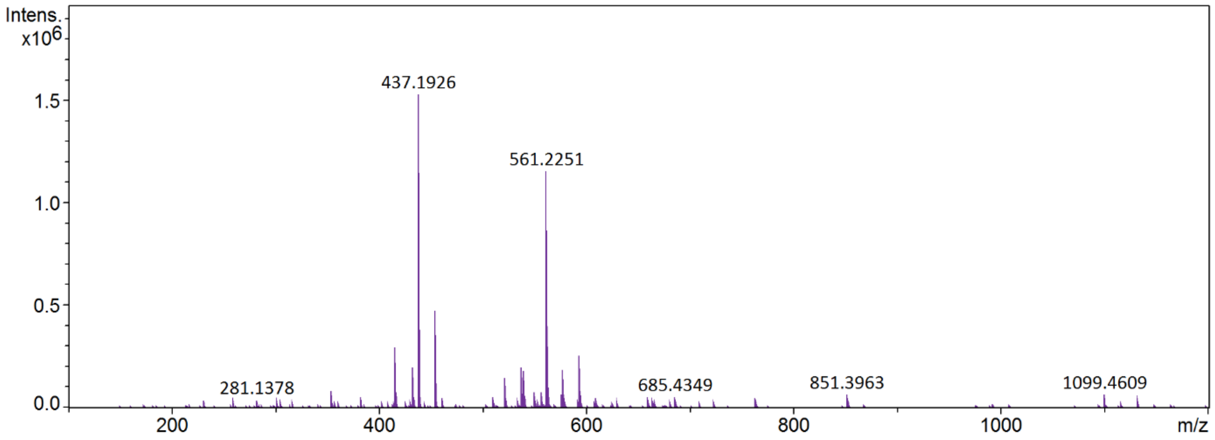

Figure S224. Experimental ESI-HRMS spectrum of (a*R*,1*R*,3*S*,3’*S*)-**21**.

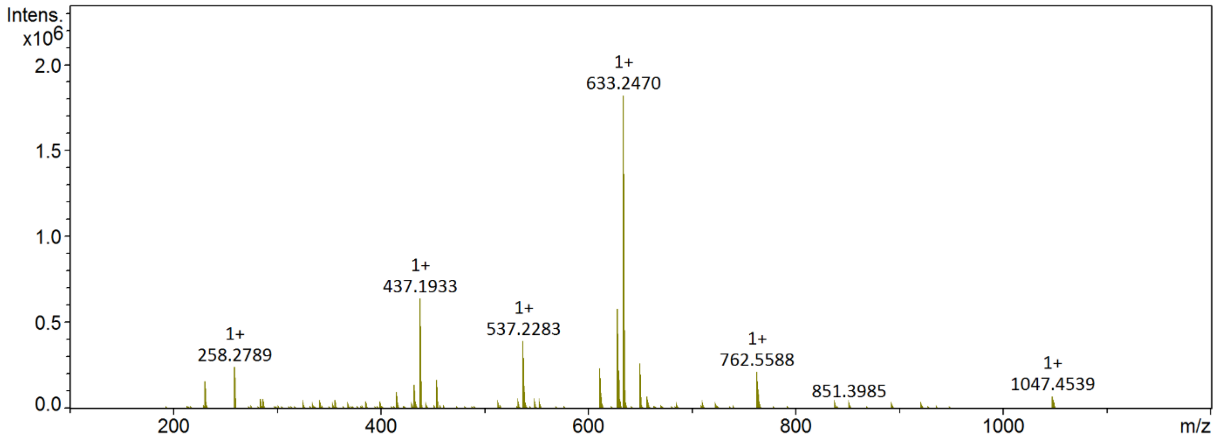

Figure S225. Experimental ESI-HRMS spectrum of (a*S*,1*R*,3*S*,1’*S*,3’*S*)-**22**.

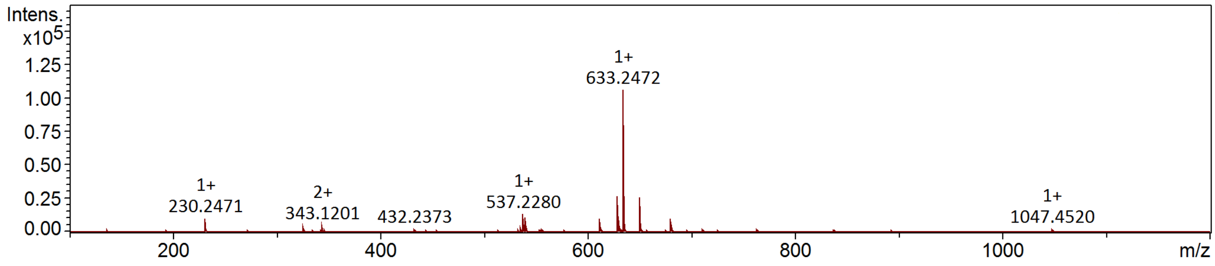

Figure S226. Experimental ESI-HRMS spectrum of (a*S*,1*S*,3*S*,1’*S*,3’*S*)-**22**.

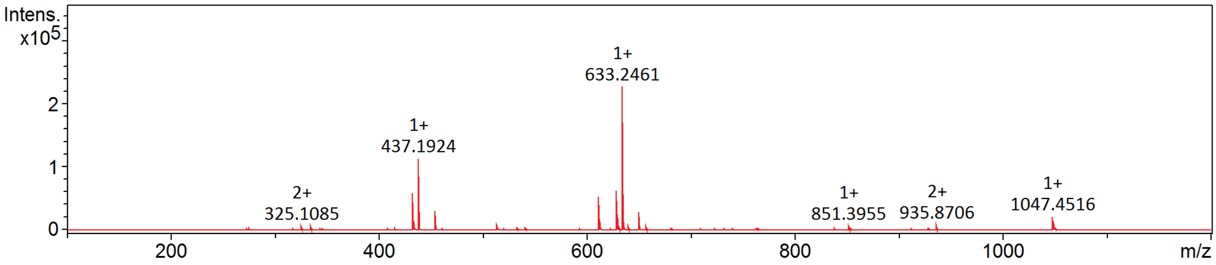

Figure S227. Experimental ESI-HRMS spectrum of (a*R*,1*S*,3*S*,1’*S*,3’*S*)-**22**.

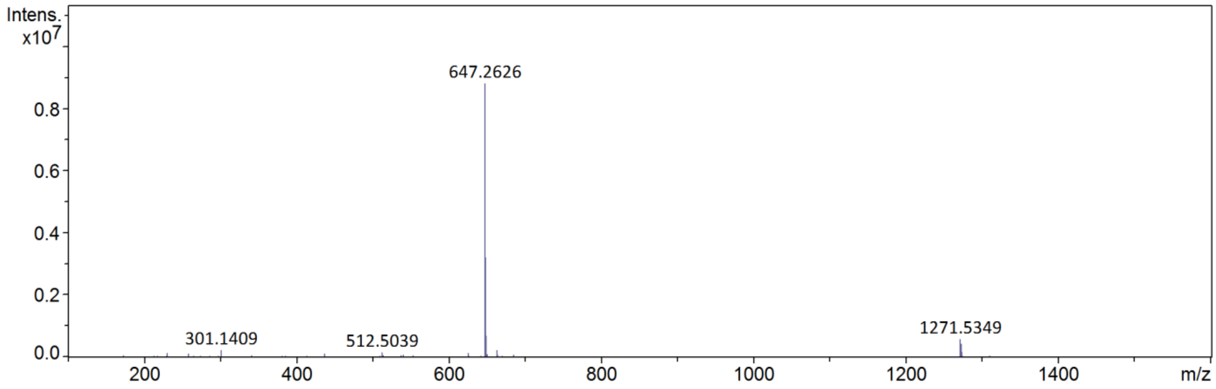

Figure S228. Experimental ESI-HRMS spectrum of (a*S*,1*S*,3*S*,1’*R*,3’*S*)-**23**.

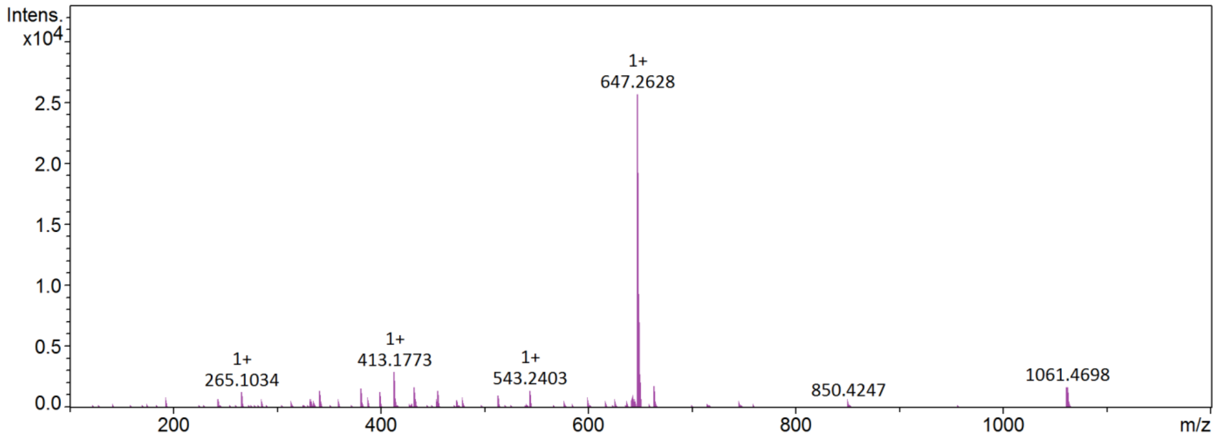

Figure S229. Experimental ESI-HRMS spectrum of (a*R*,1*S*,3*S*,1’*R*,3’*S*)-**23**.

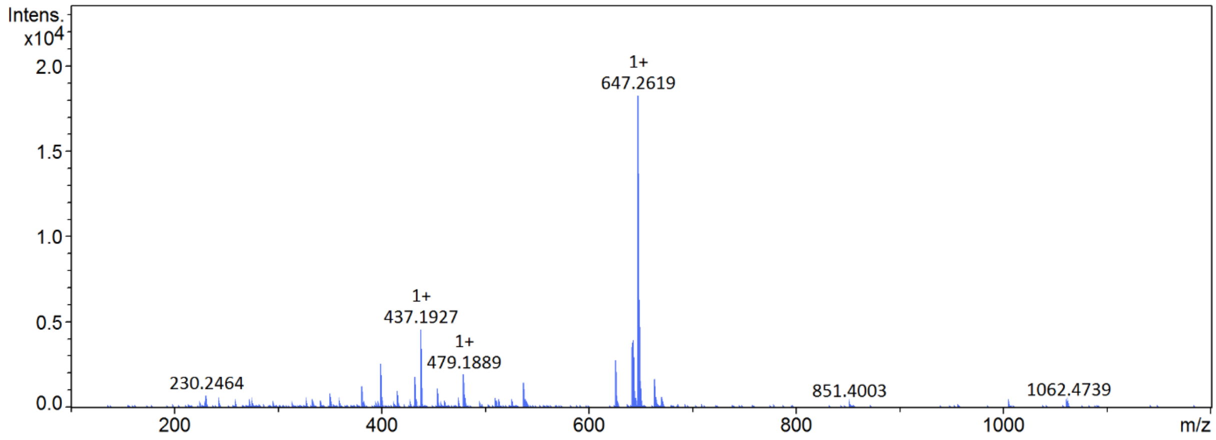

Figure S230. Experimental ESI-HRMS spectrum of (a*S*,1*R*,3*S*,1’*R*,3’*S*)-**23**.

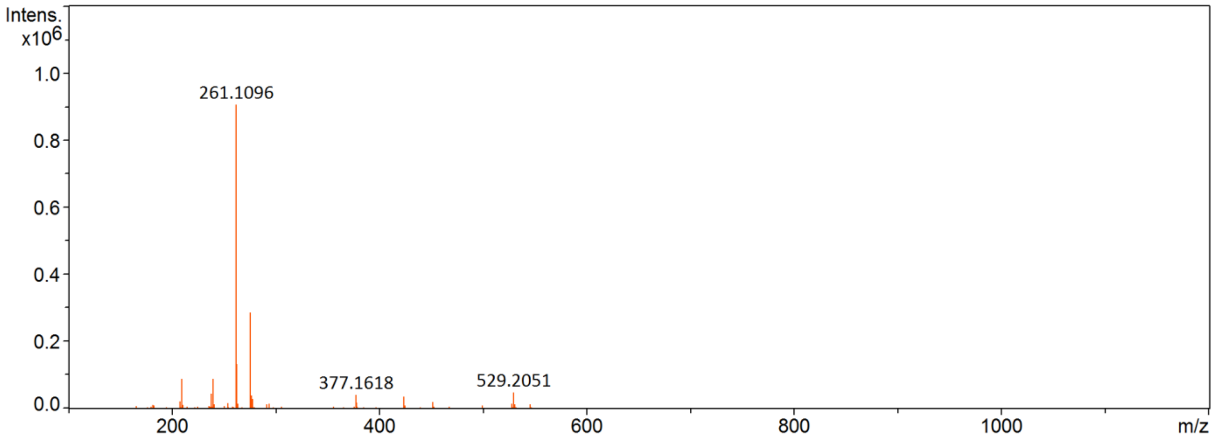

Figure S231. Experimental ESI-HRMS spectrum of (*S*)-**24**.

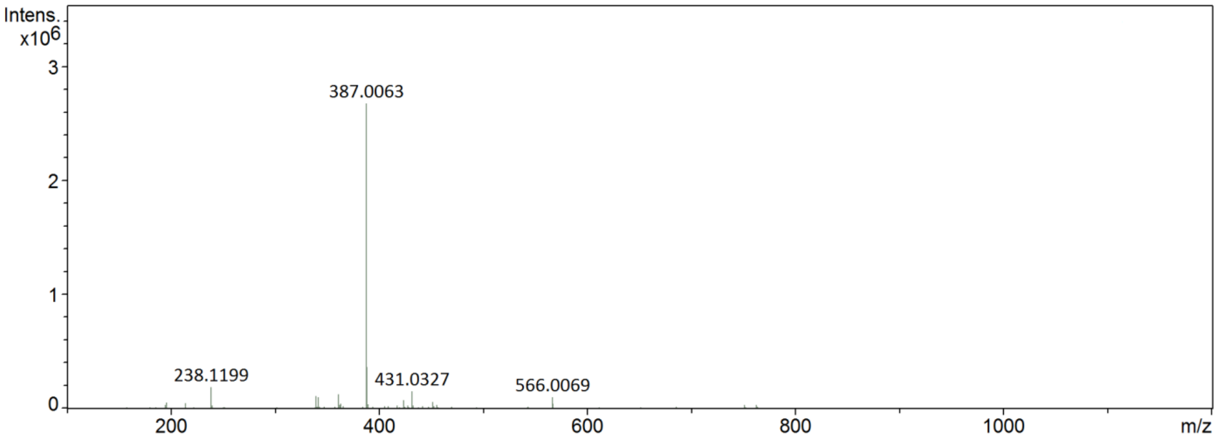

Figure S232. Experimental ESI-HRMS spectrum of (*S*)-**25**.

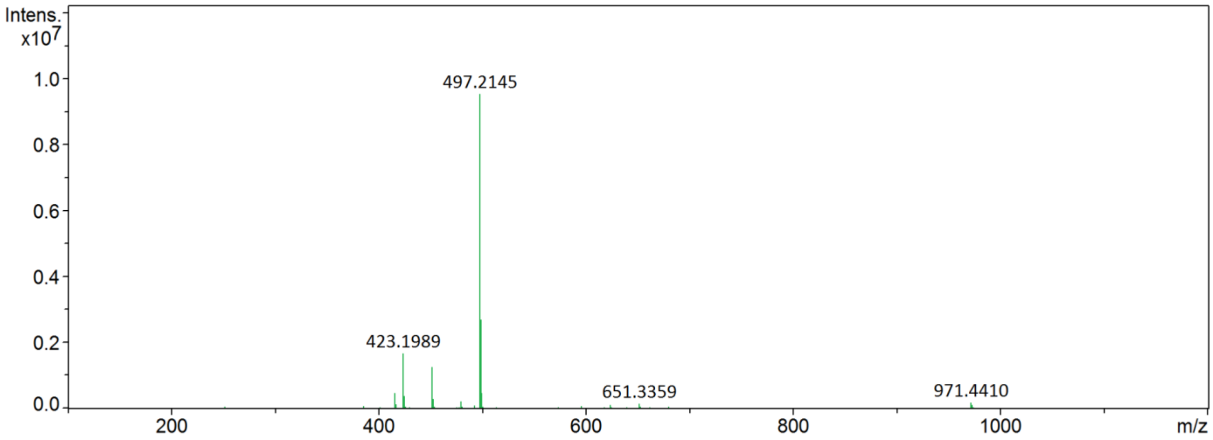

Figure S233. Experimental ESI-HRMS spectrum of the mixture of (a*R*,3*S*,2’*S*)-26 and (a*S*,3*S*,2’*S*)-**26**.

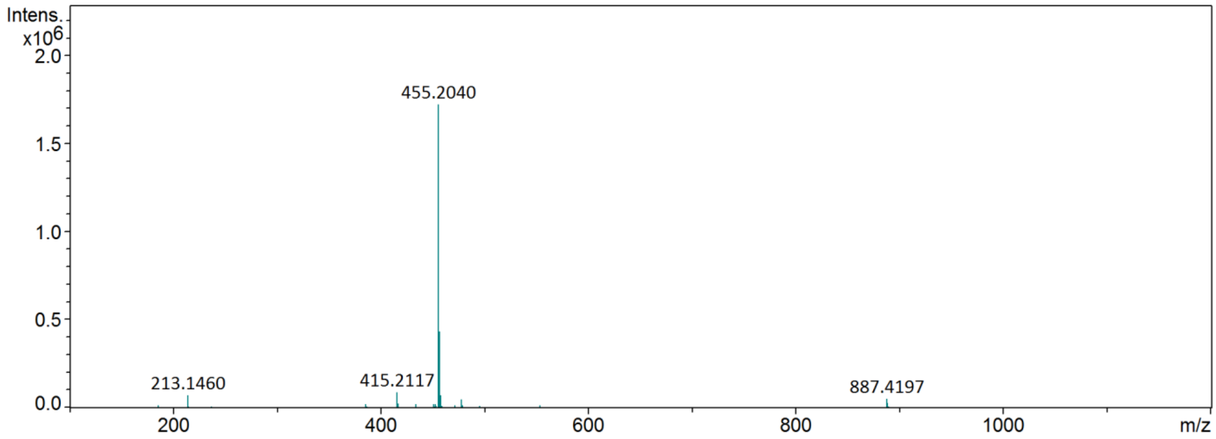

Figure S234. Experimental ESI-HRMS spectrum of the mixture of (a*R*,3*S*,2’*S*)-**27** and (a*S*,3*S*,2’*S*)-**27**.

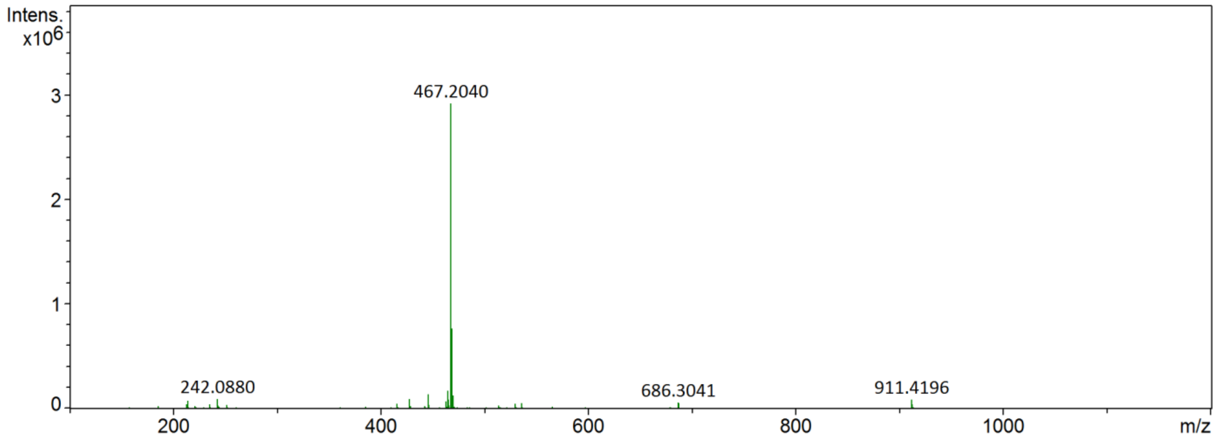

Figure S235. Experimental ESI-HRMS spectrum of the mixture of (a*R*,3*S*,3’*S*)-**28** and (a*S*,3*S*,3’*S*)-**28**.

3.4. IR spectra

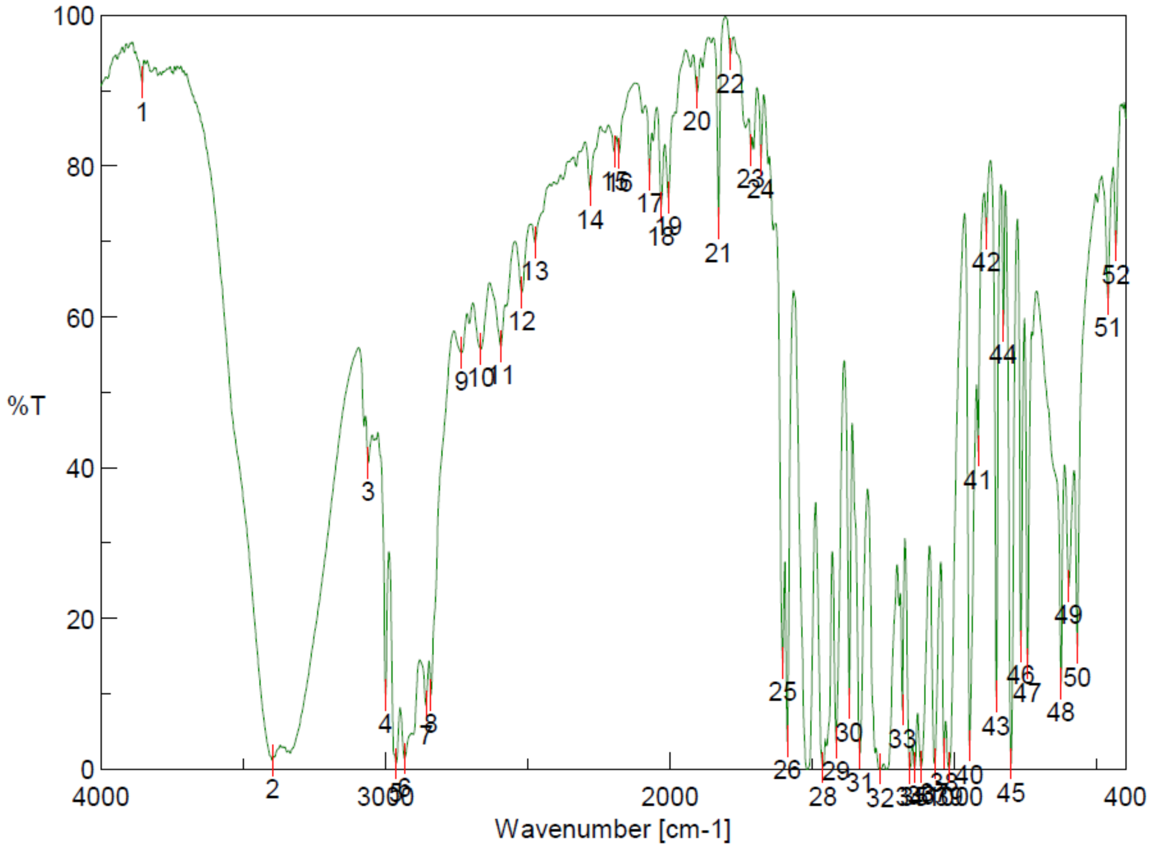

Figure S236. Experimental IR spectrum of (*S*)-**11** recorded as KBr disc.

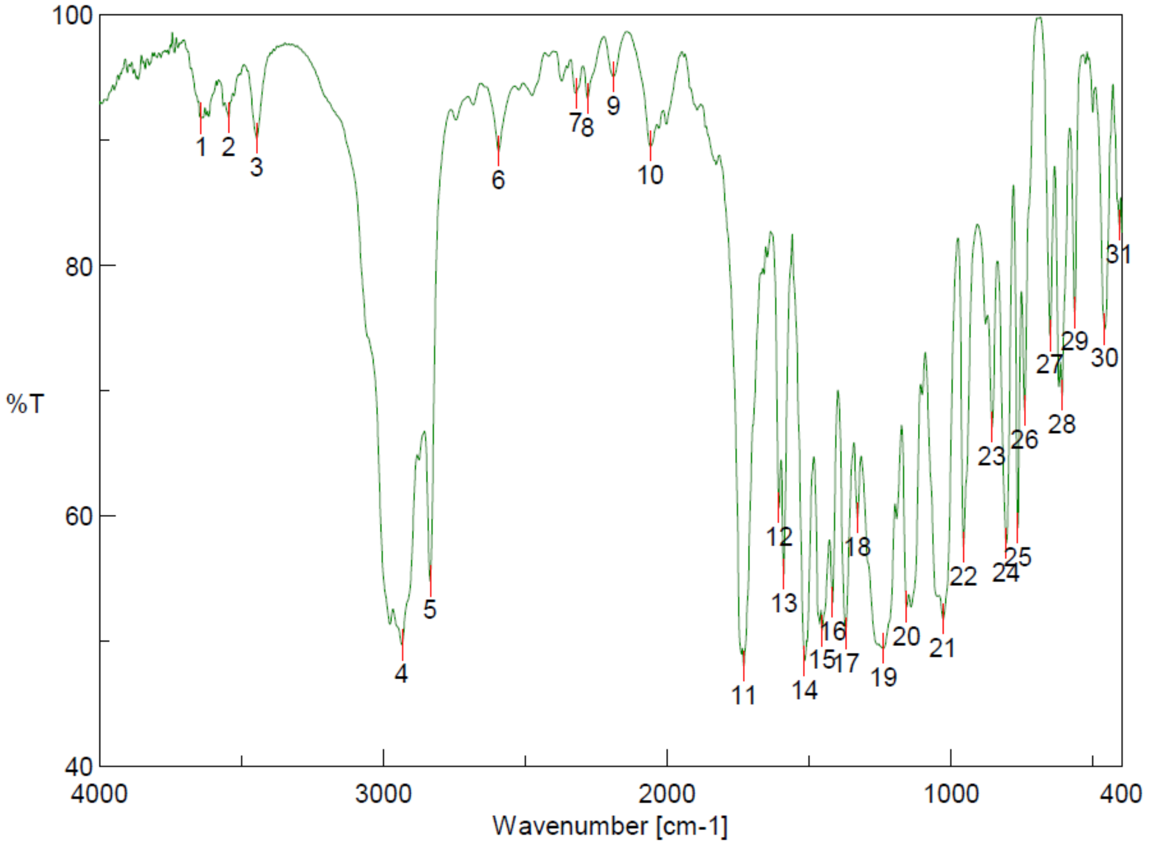

Figure S237. Experimental IR spectrum of (*S*)-**12** recorded as KBr disc.

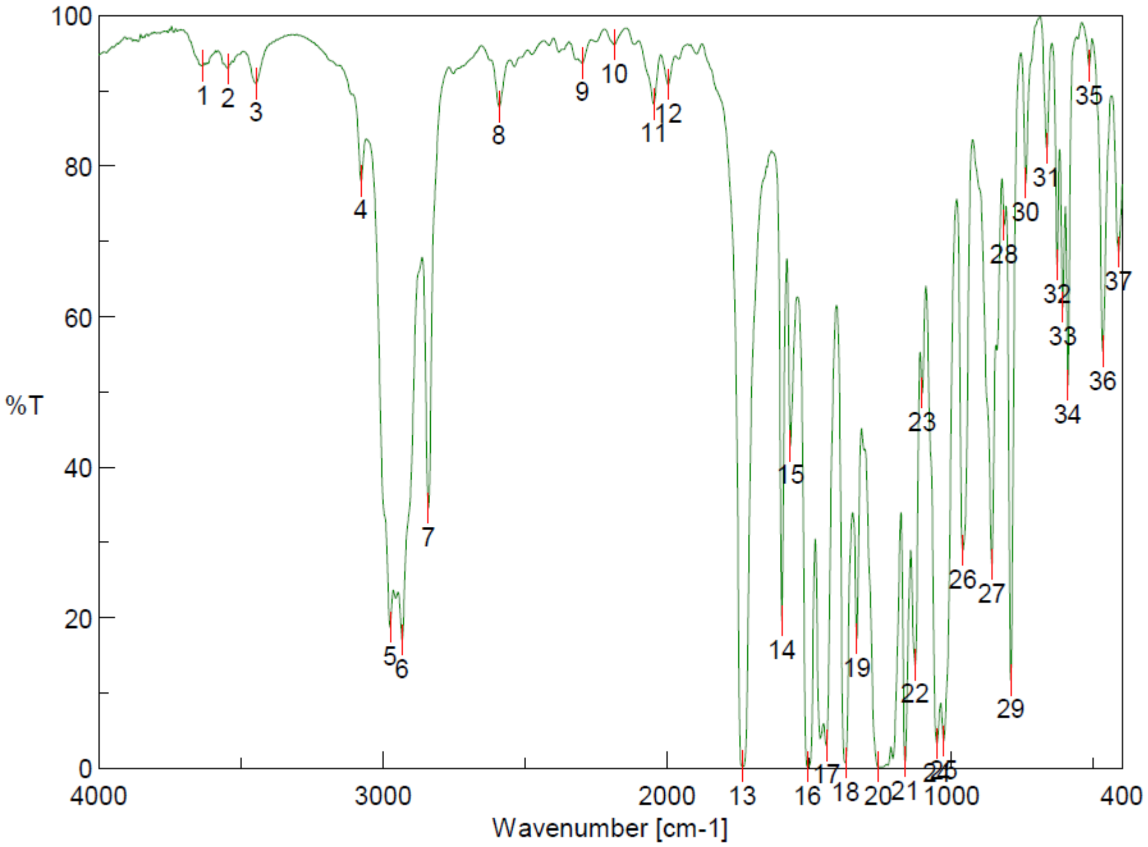

Figure S238. Experimental IR spectrum of (*S*)-**13** recorded as KBr disc.

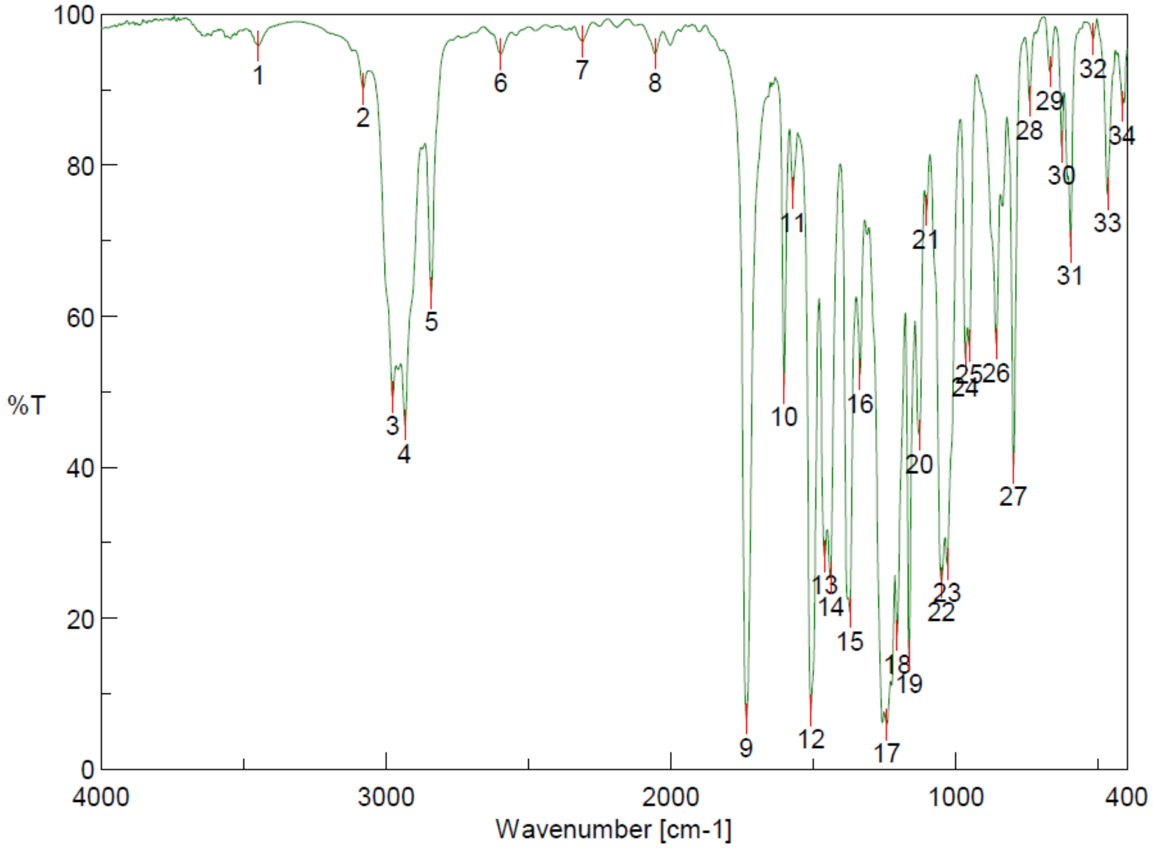

Figure S239. Experimental IR spectrum of (*S*)-**14** recorded as KBr disc.

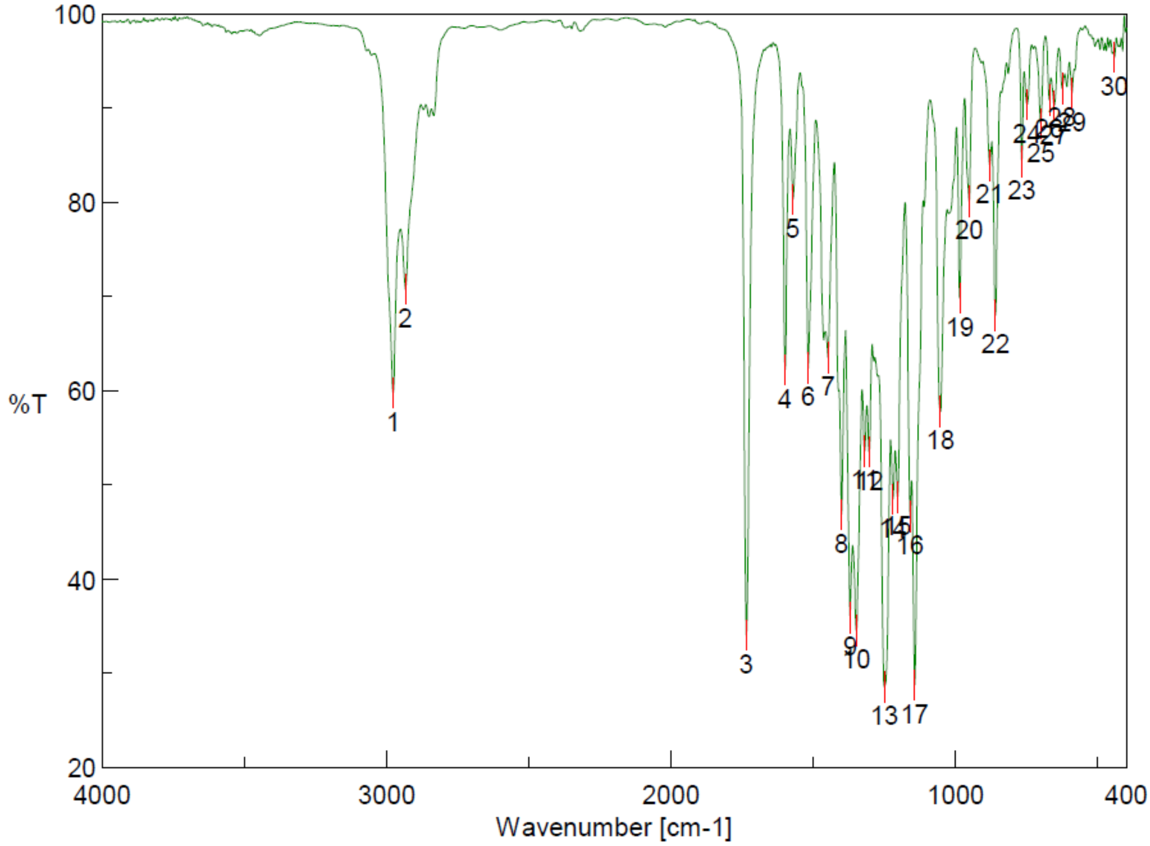

Figure S240. Experimental IR spectrum of (*S*)-**8** recorded as KBr disc.

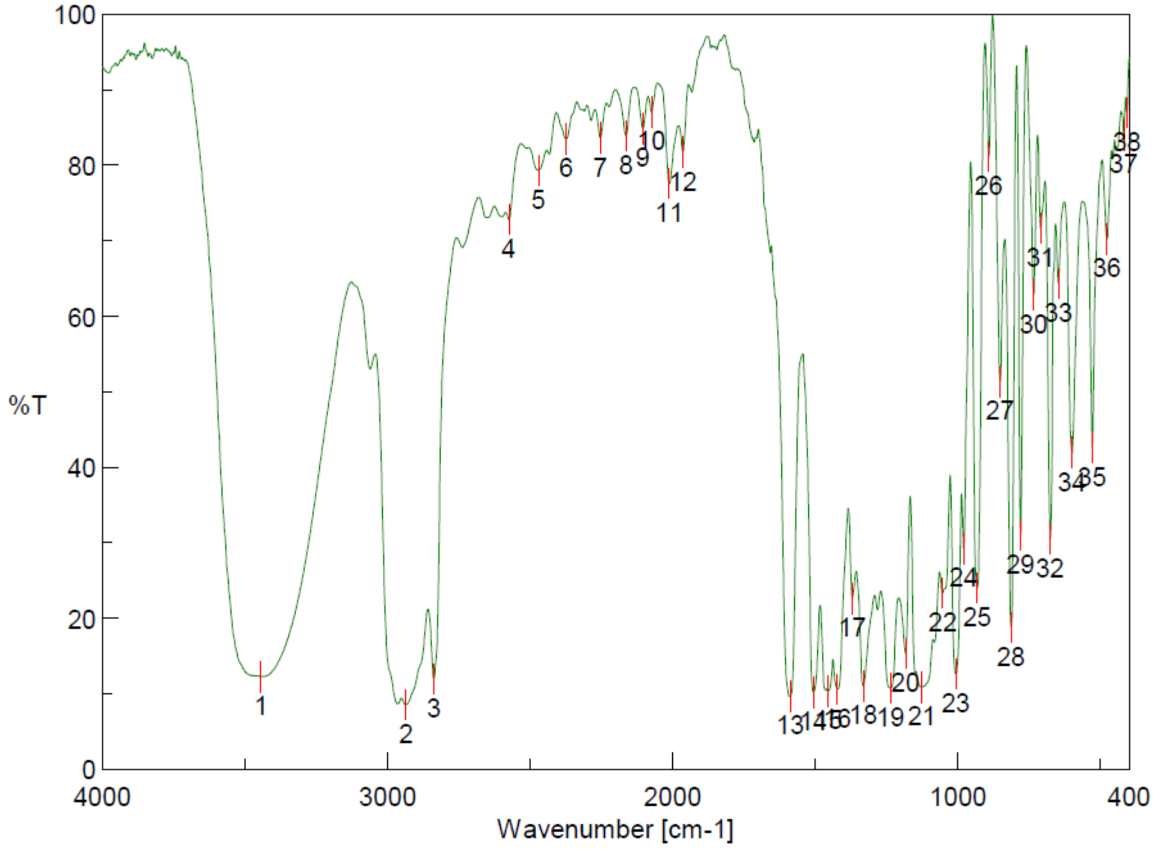

Figure S241. Experimental IR spectrum of (*S*)-**16** recorded as KBr disc.

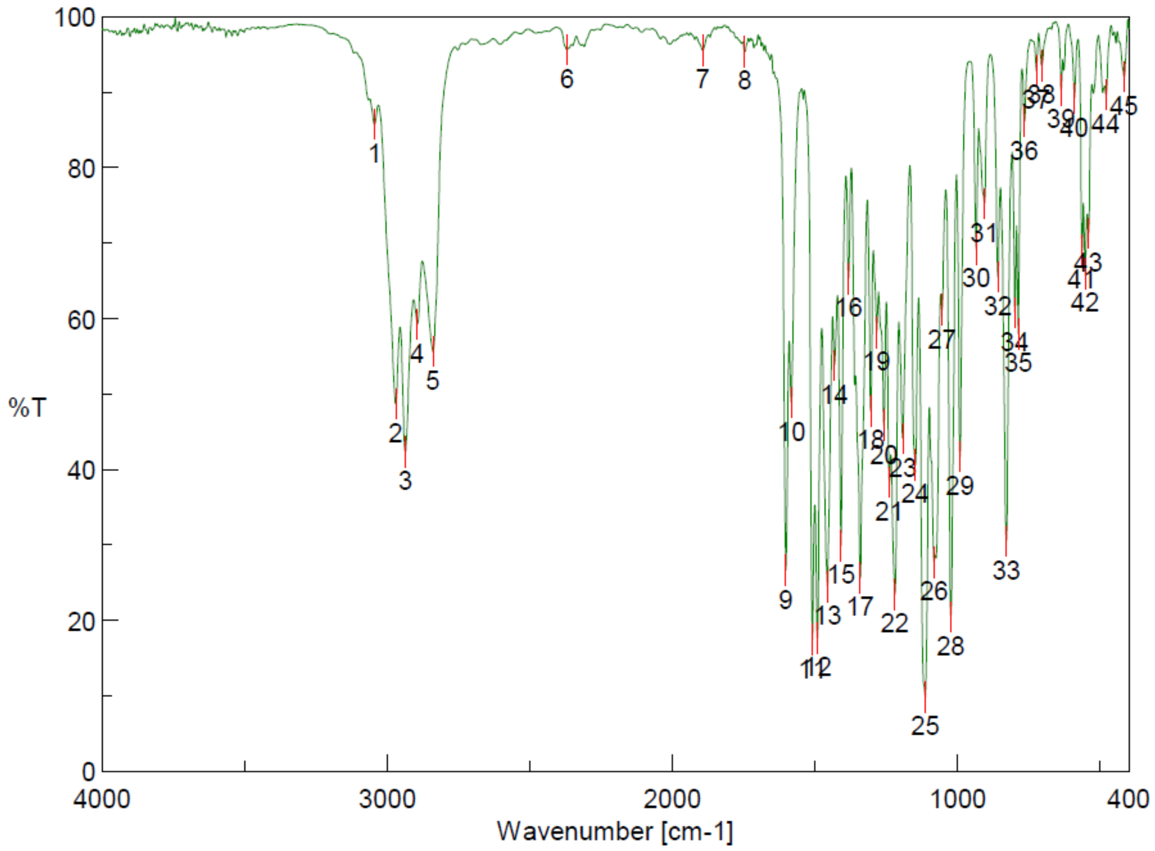

Figure S242. Experimental IR spectrum of *cis*-(1*R*,3*S*)-**17** recorded as KBr disc.

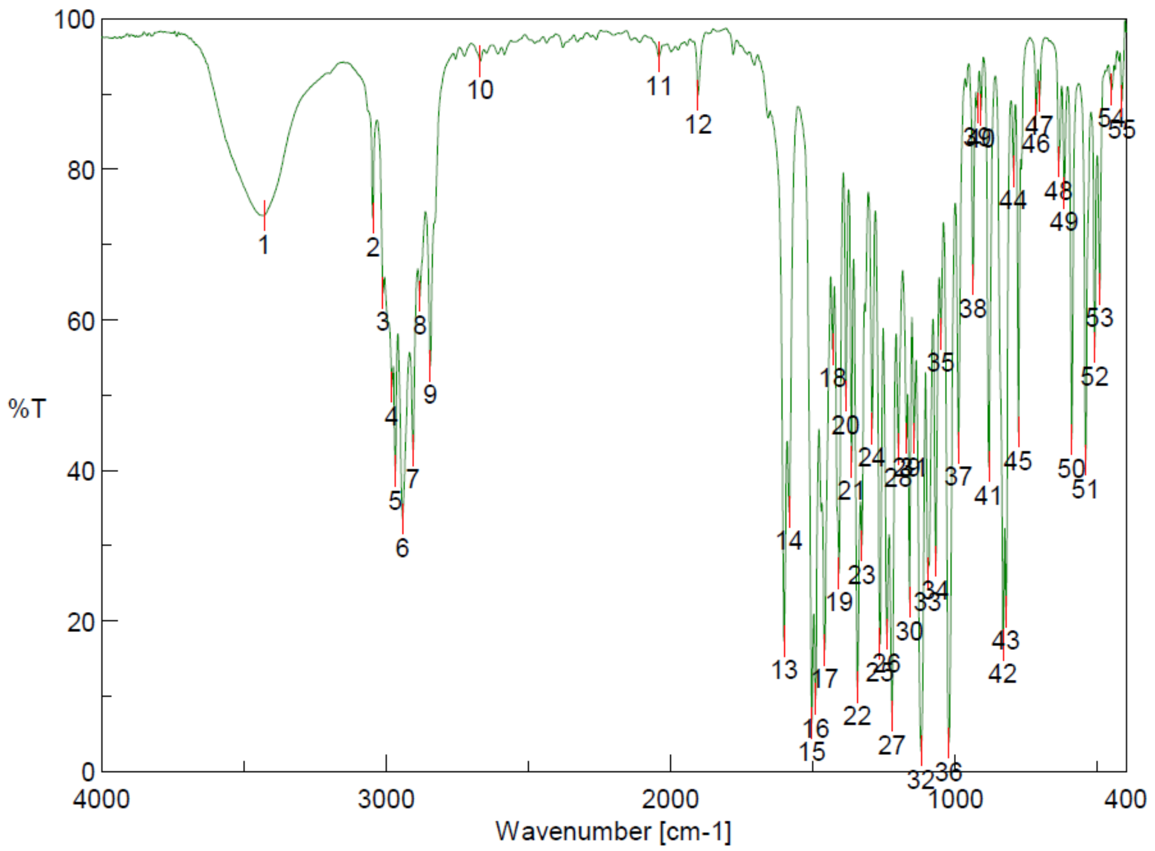

Figure S243. Experimental IR spectrum of *trans*-(1*S*,3*S*)-**17** recorded as KBr disc.

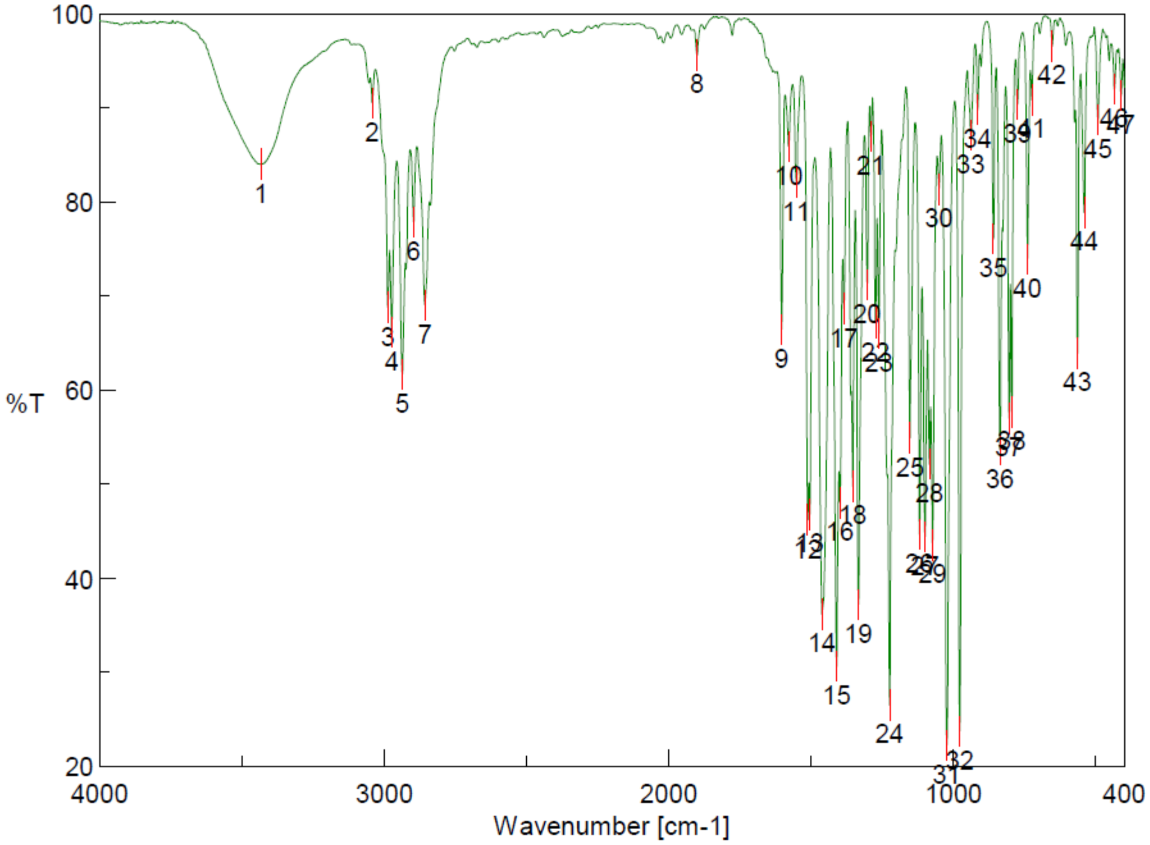

Figure S244. Experimental IR spectrum of *cis*-(1*R*,3*S*)-**18** recorded as KBr disc.

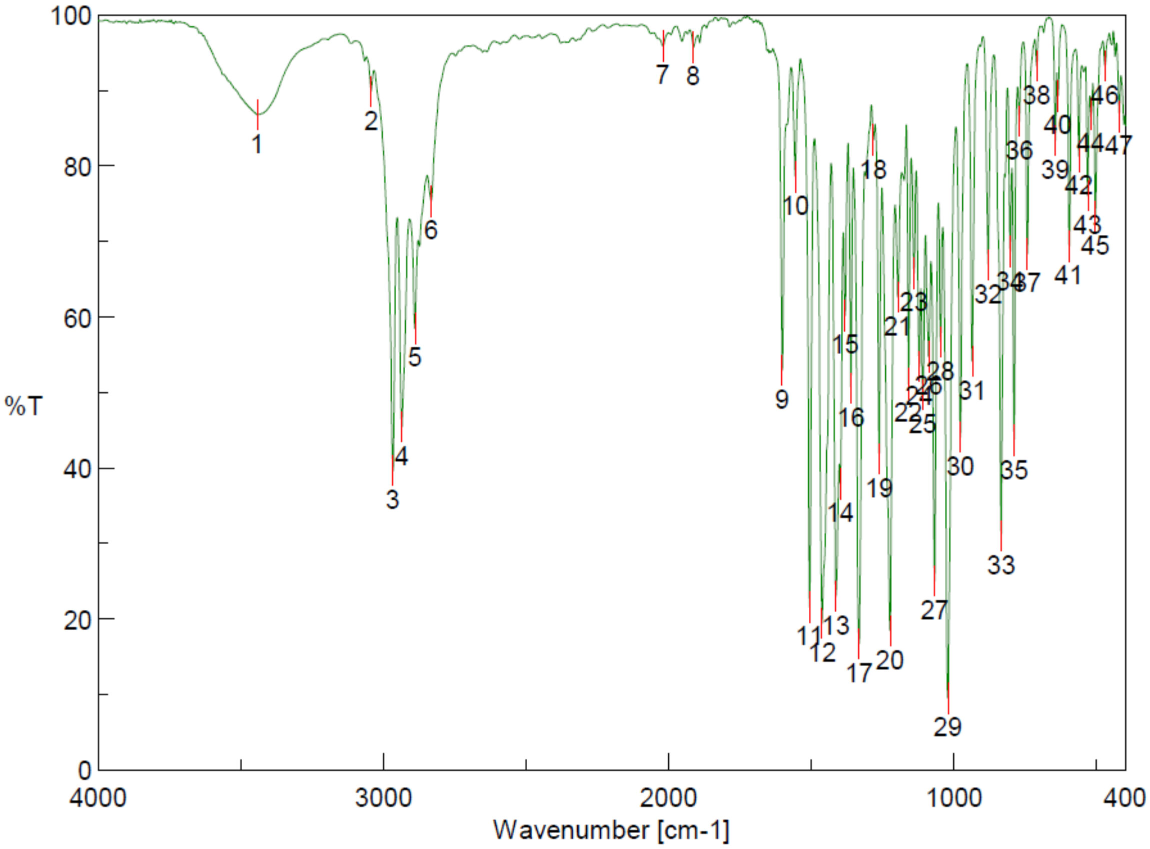

Figure S245. Experimental IR spectrum of *trans*-(1*S*,3*S*)-**18** recorded as KBr disc.

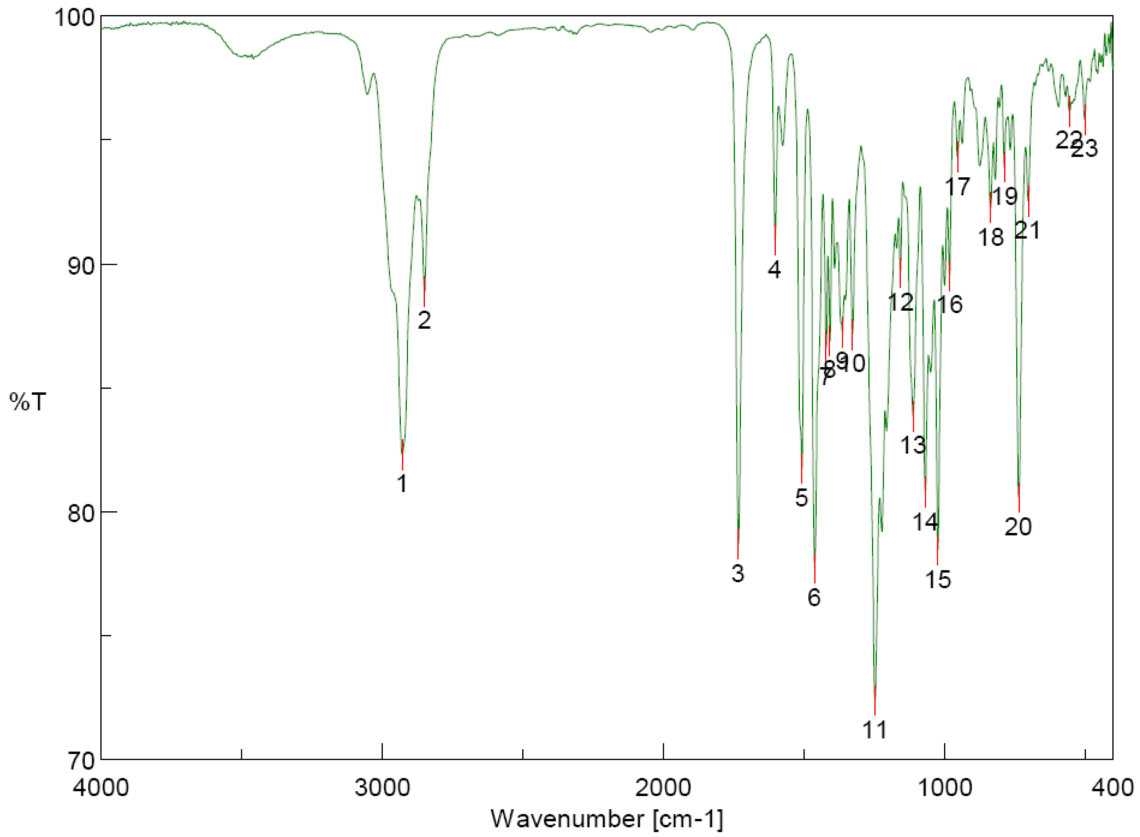

Figure S246. Experimental IR spectrum of the mixture of (a*R*,1*S*,3*S*,2'*S*)-**19** and (a*S*,1*S*,3*S*,2'*S*)-**19** recorded as KBr disc.

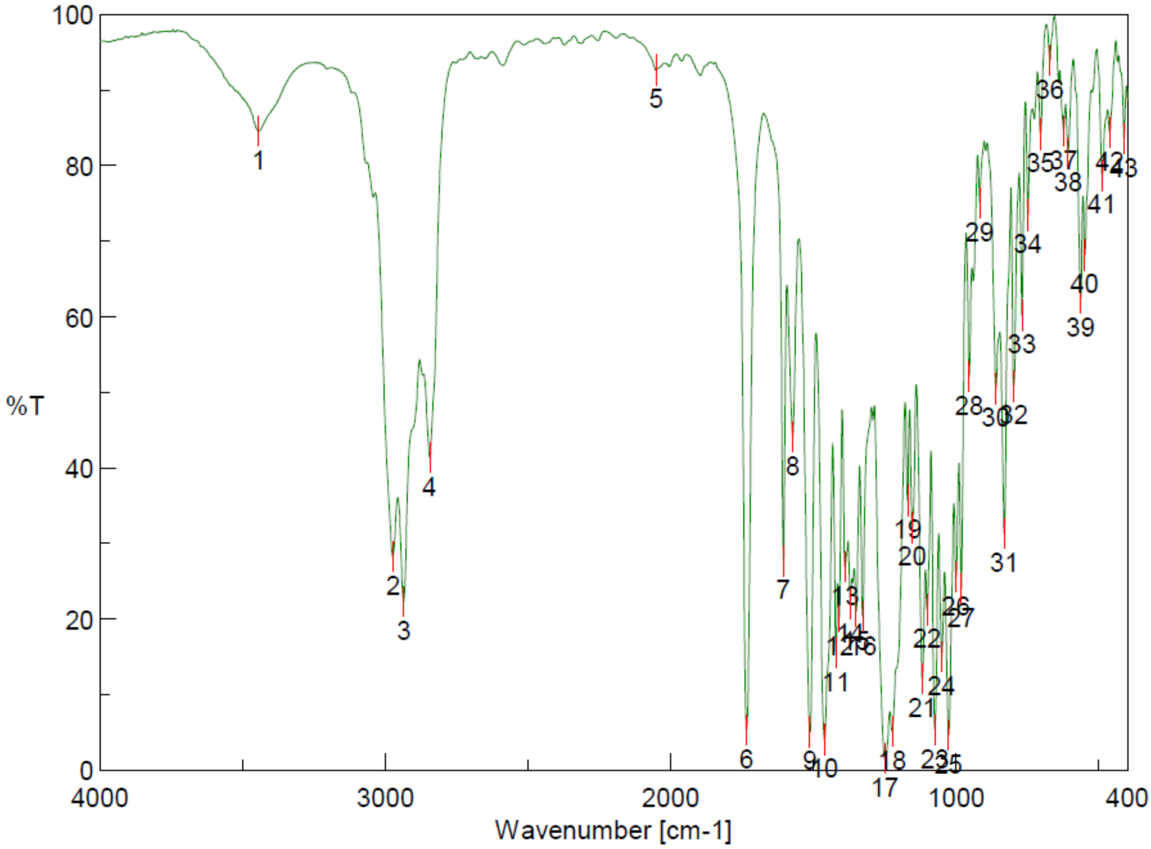

Figure S247. Experimental IR spectrum of (a*S*,1*R*,3*S*,2'*S*)-**19** recorded as KBr disc.

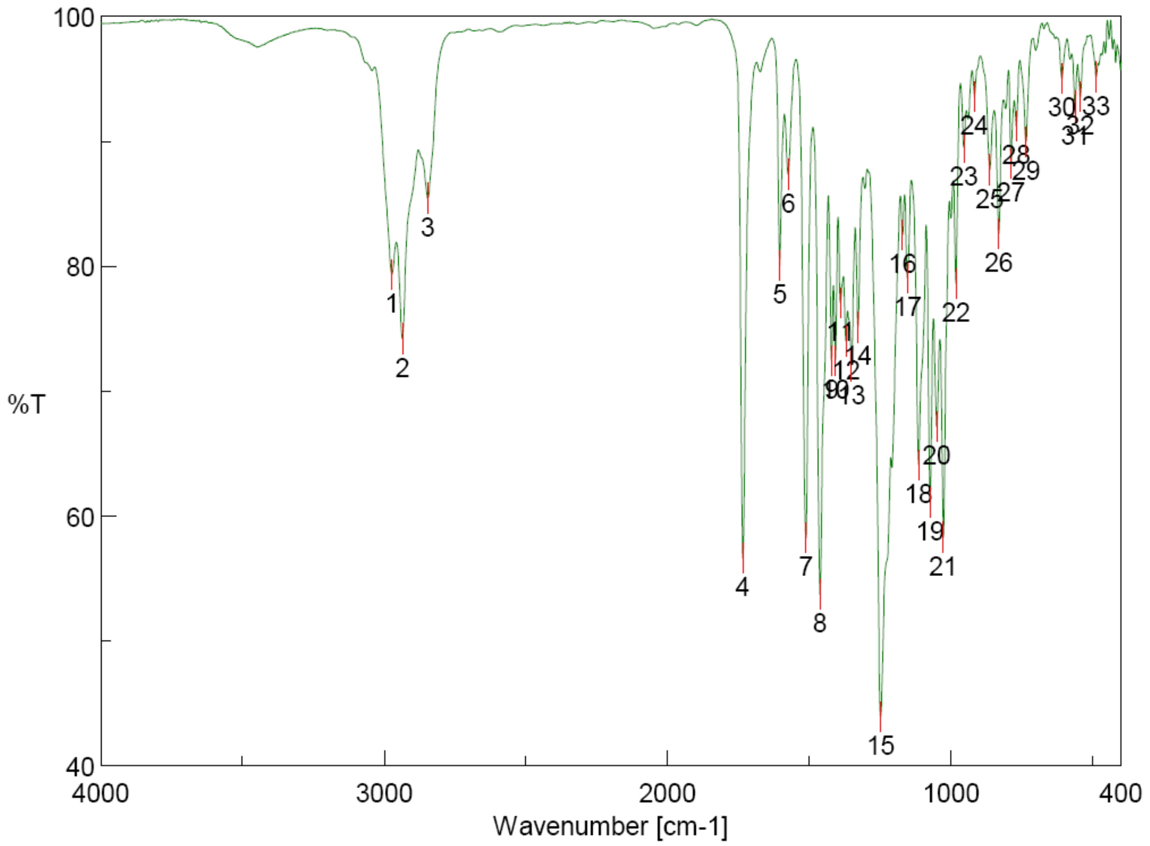

Figure S248. Experimental IR spectrum of (a*R*,1*R*,3*S*,2’*S*)-**19** recorded as KBr disc.

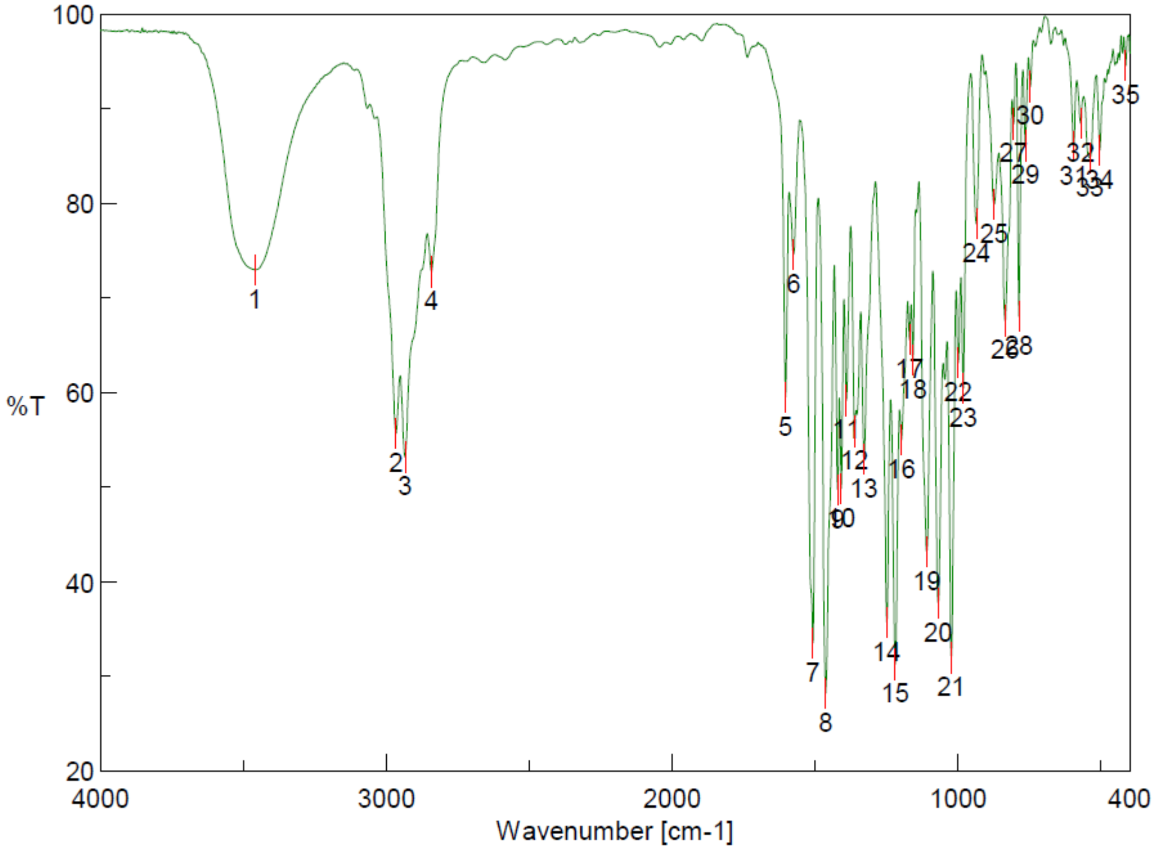

Figure S249. Experimental IR spectrum of (a*S*,1*S*,3*S*,2’*S*)-**20** recorded as KBr disc.

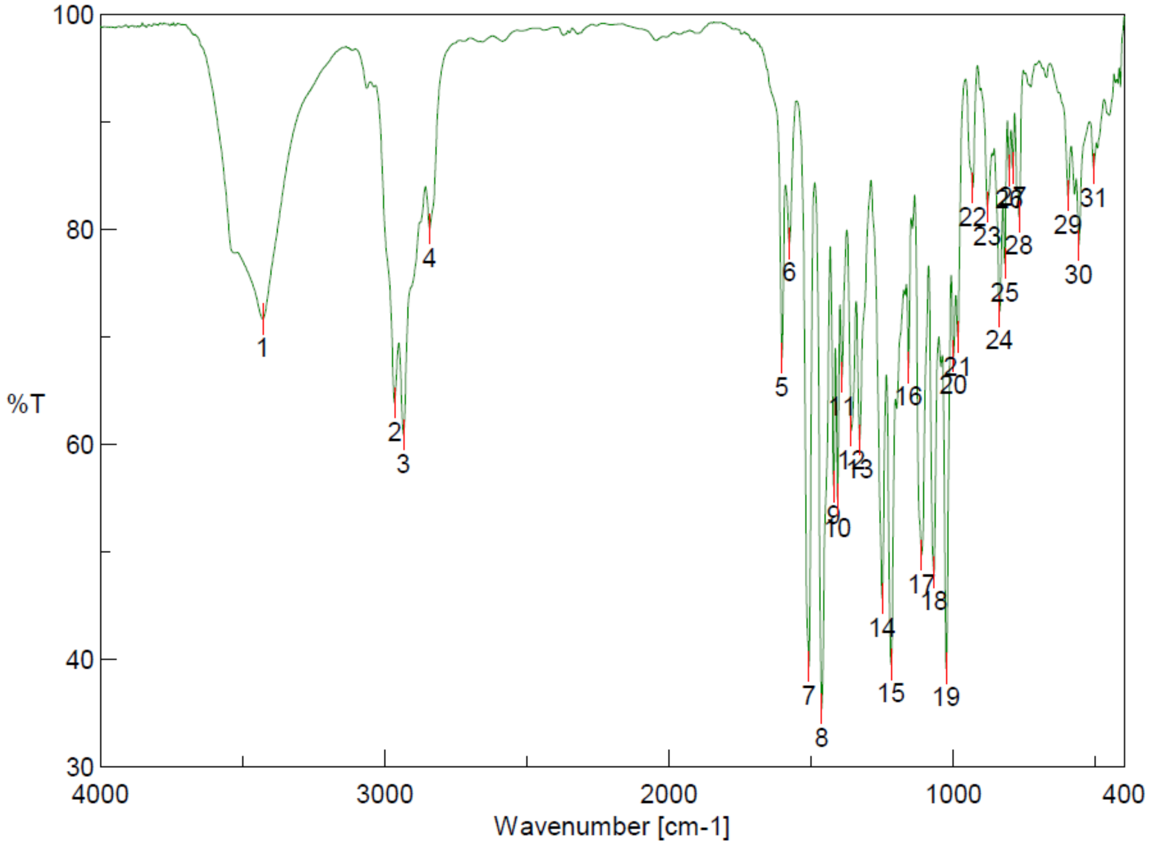

Figure S250. Experimental IR spectrum of (a*R*,1*S*,3*S*,2'*S*)-**20** recorded as KBr disc.

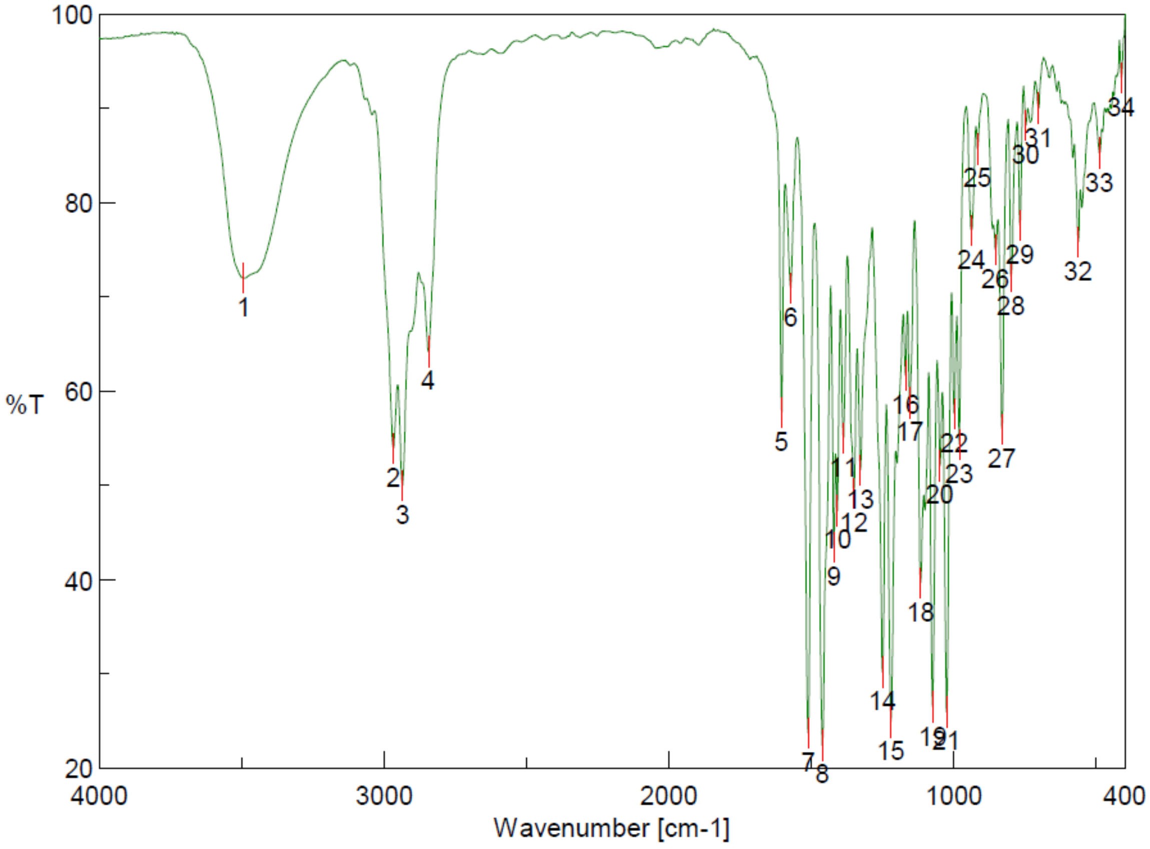

Figure S251. Experimental IR spectrum of (a*S*,1*R*,3*S*,2'*S*)-**20** recorded as KBr disc.

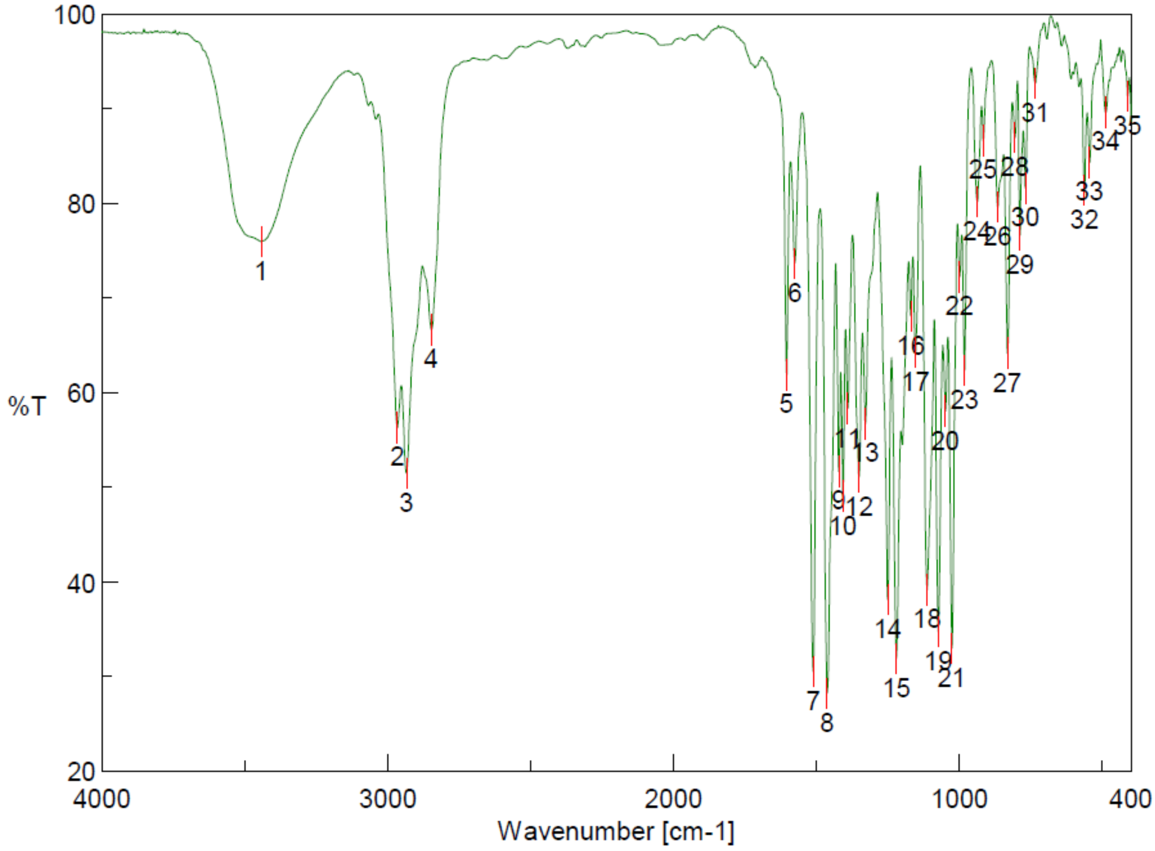

Figure S252. Experimental IR spectrum of (a*R*,1*R*,3*S*,2'*S*)-**20** recorded as KBr disc.

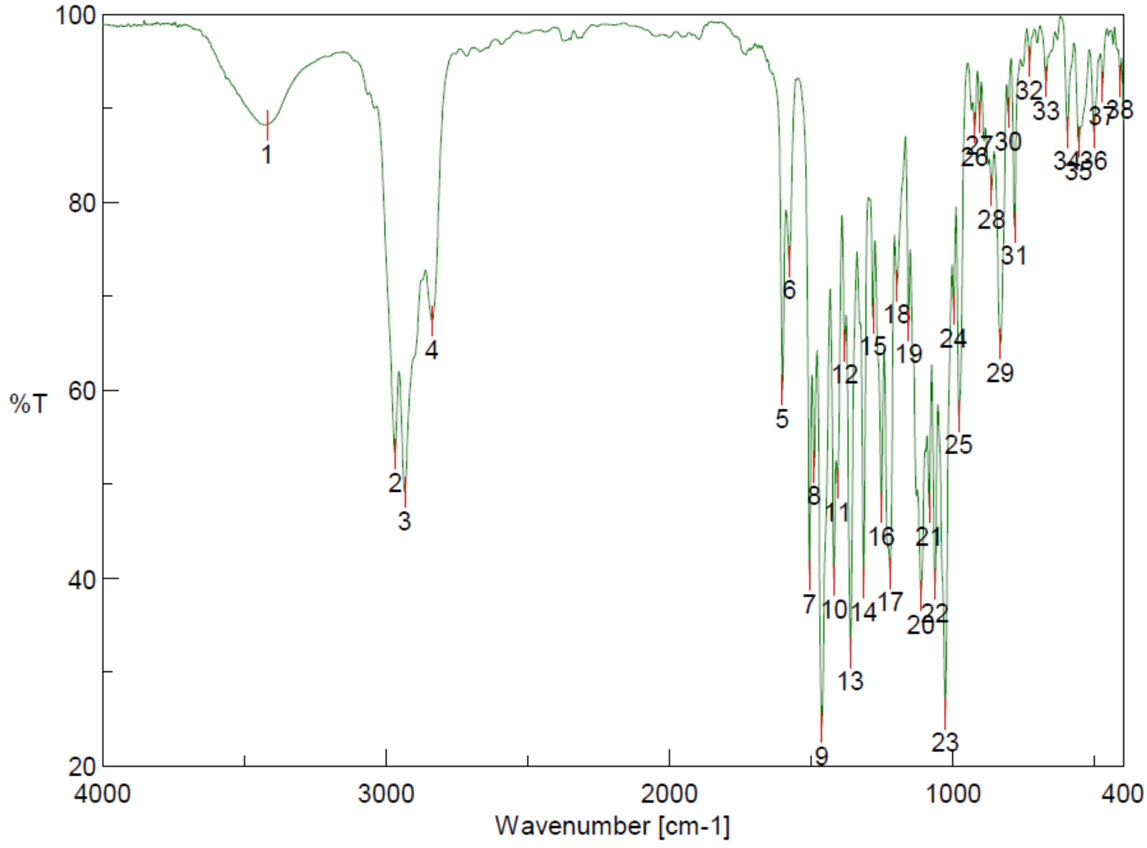

Figure S253. Experimental IR spectrum of (a*S*,1*S*,3*S*,3'*S*)-**21** recorded as KBr disc.

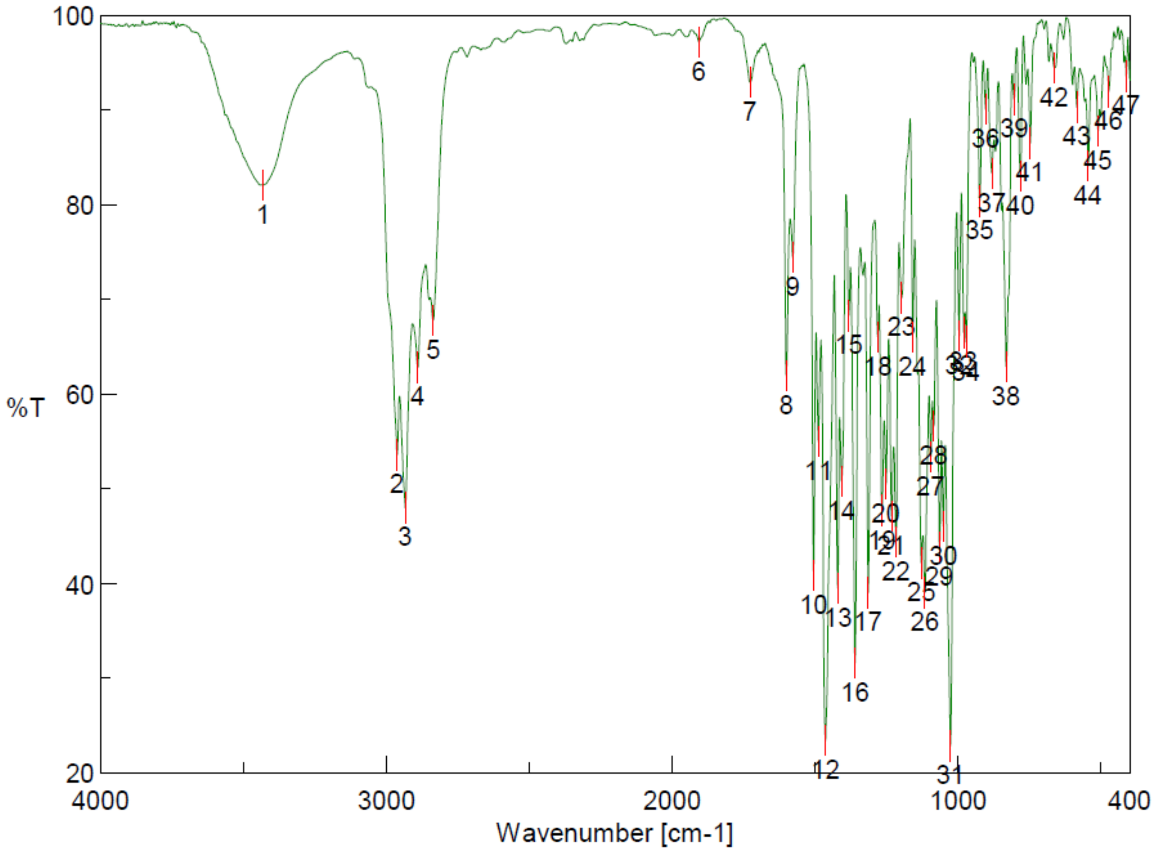

Figure S254. Experimental IR spectrum of (a*R*,1*S*,3*S*,3’*S*)-**21** recorded as KBr disc.

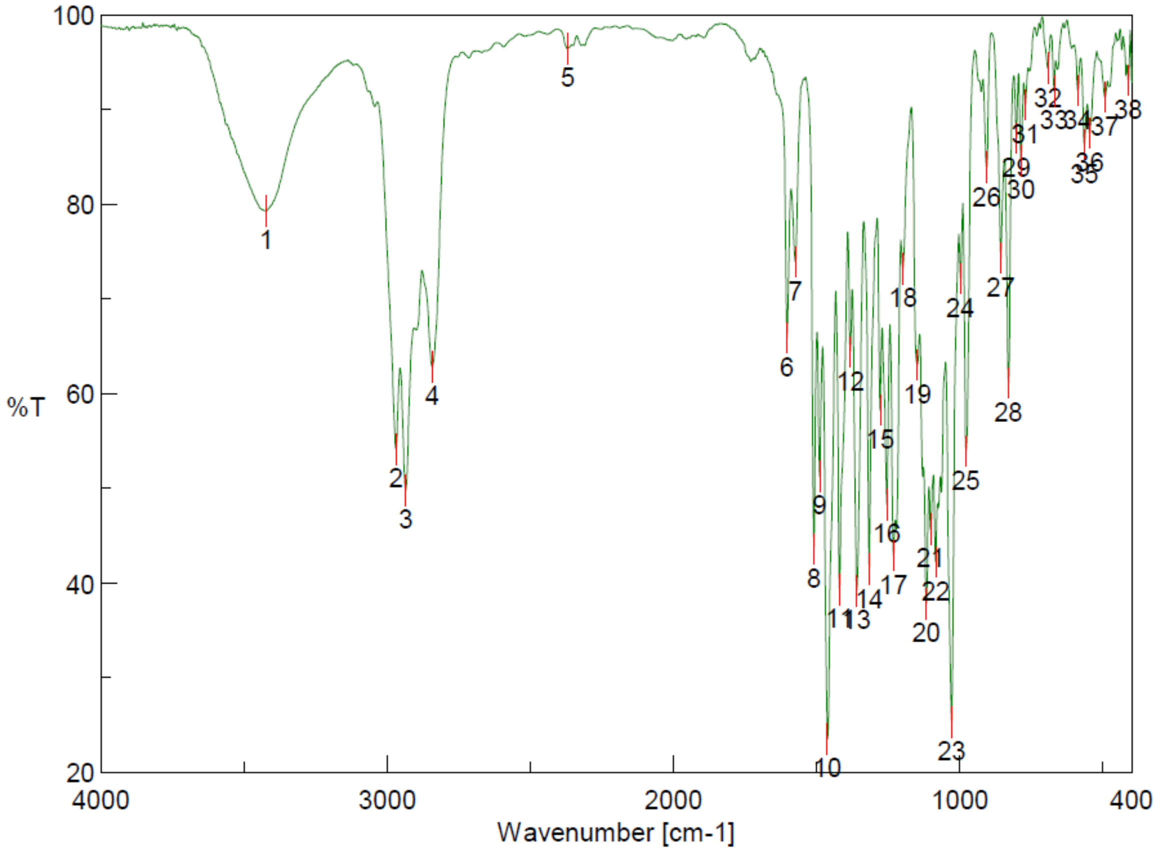

Figure S255. Experimental IR spectrum of (a*S*,1*R*,3*S*,3’*S*)-**21** recorded as KBr disc.

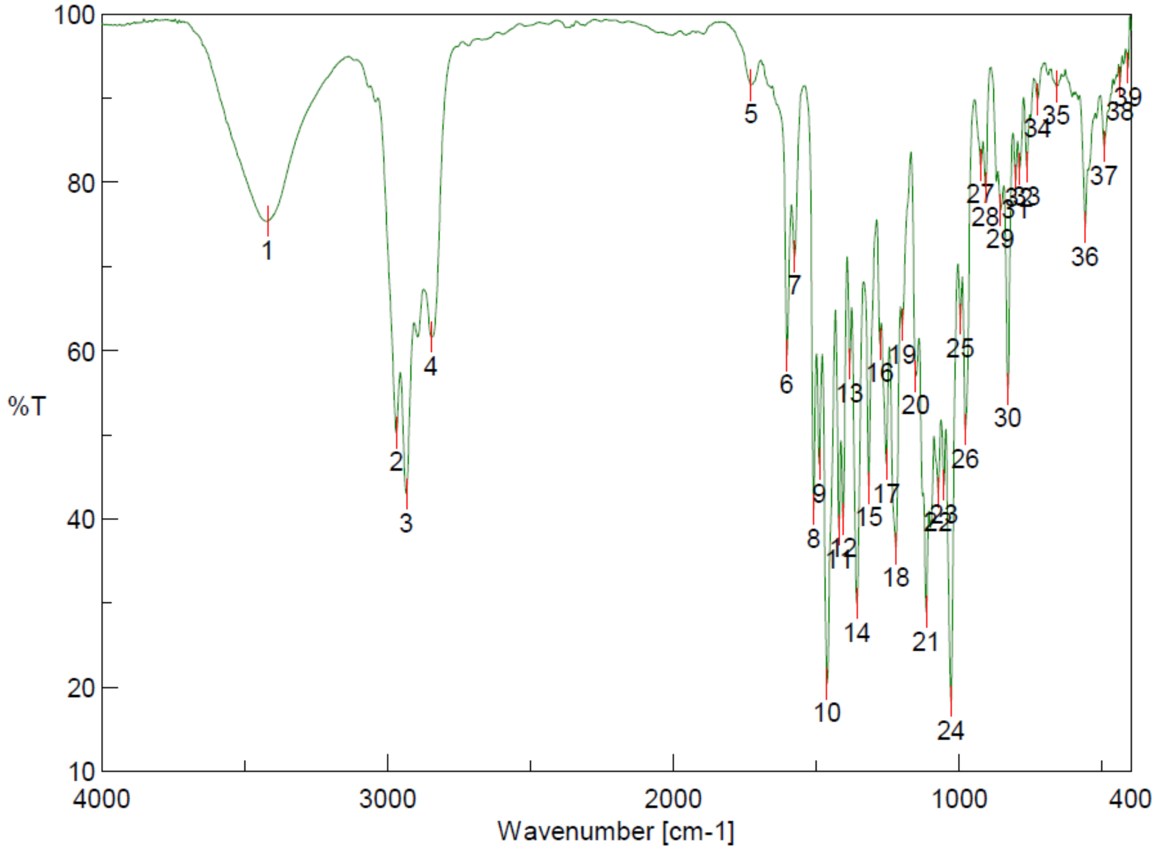

Figure S256. Experimental IR spectrum of (a*R*,1*R*,3*S*,3'*S*)-**21** recorded as KBr disc.

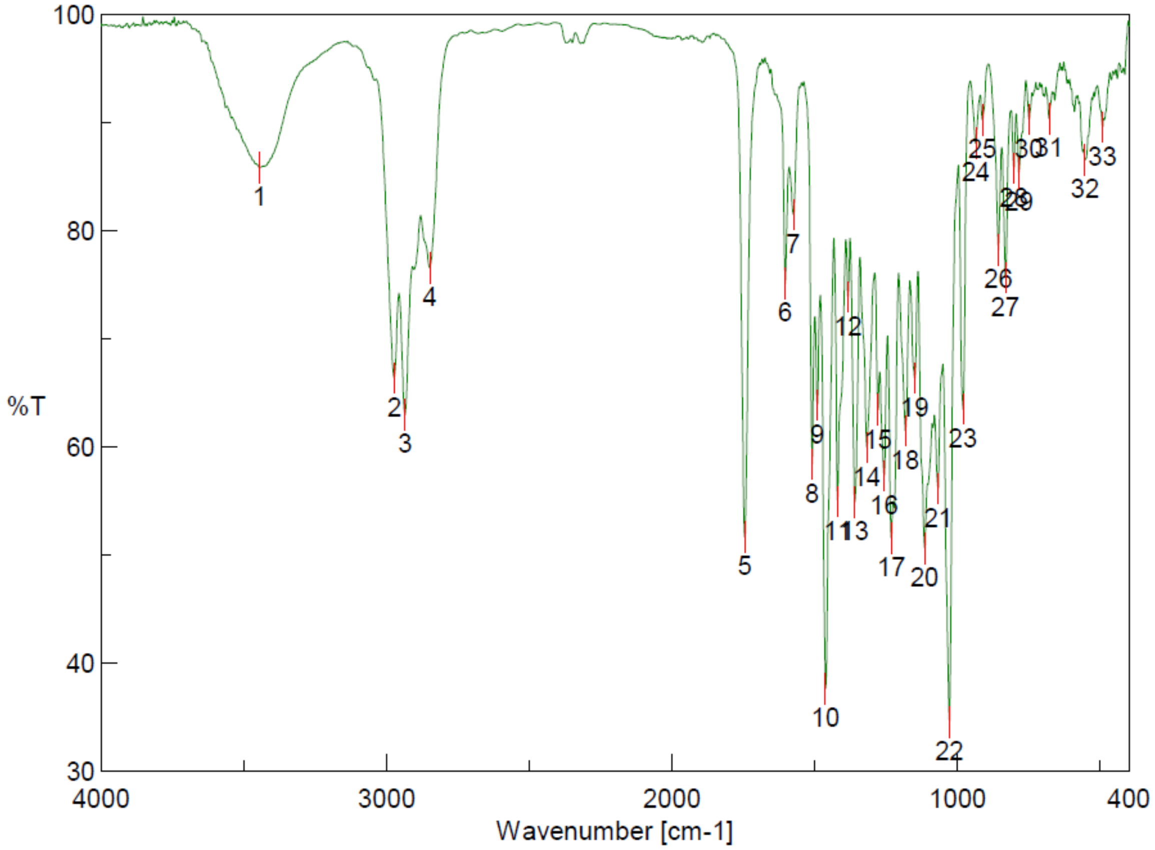

Figure S257. Experimental IR spectrum of (a*S*,1*R*,3*S*,1'*S*,3'*S*)-**22** recorded as KBr disc.

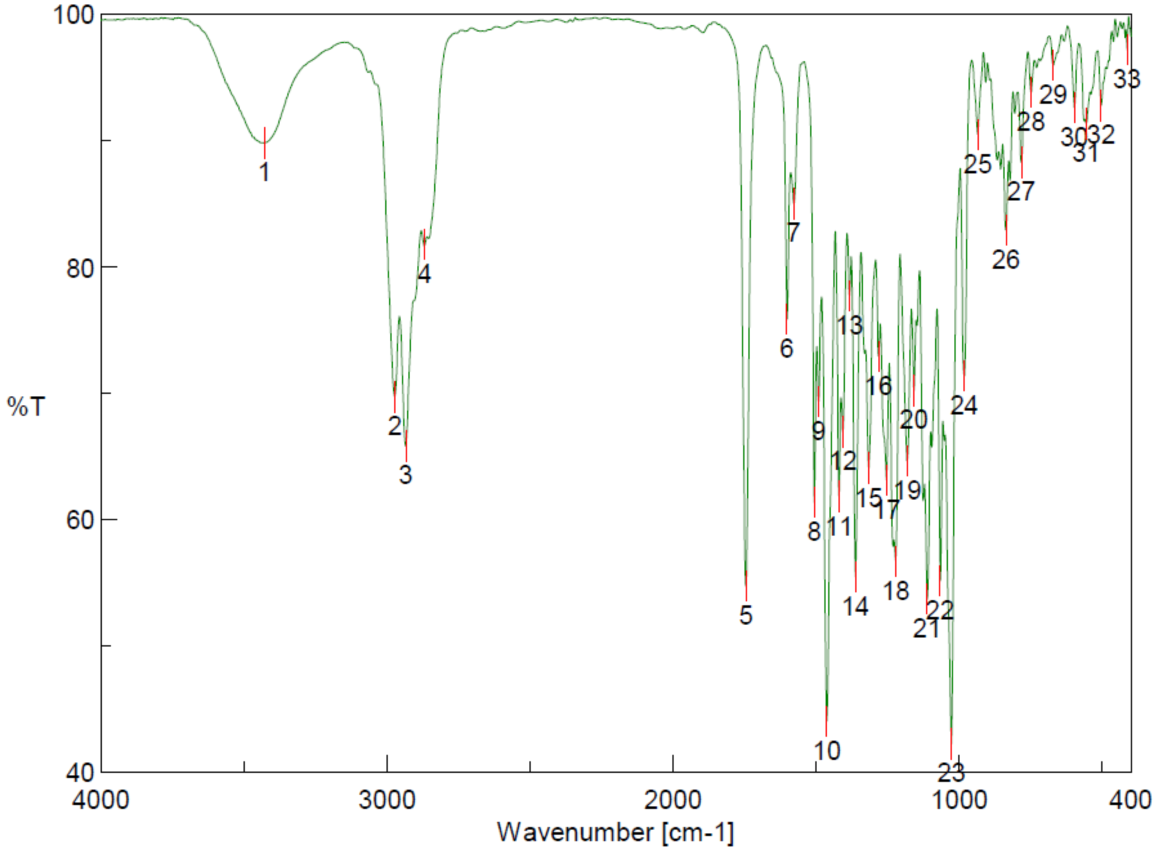

Figure S258. Experimental IR spectrum of (a*S*,1*S*,3*S*,1’*S*,3’*S*)-**22** recorded as KBr disc.

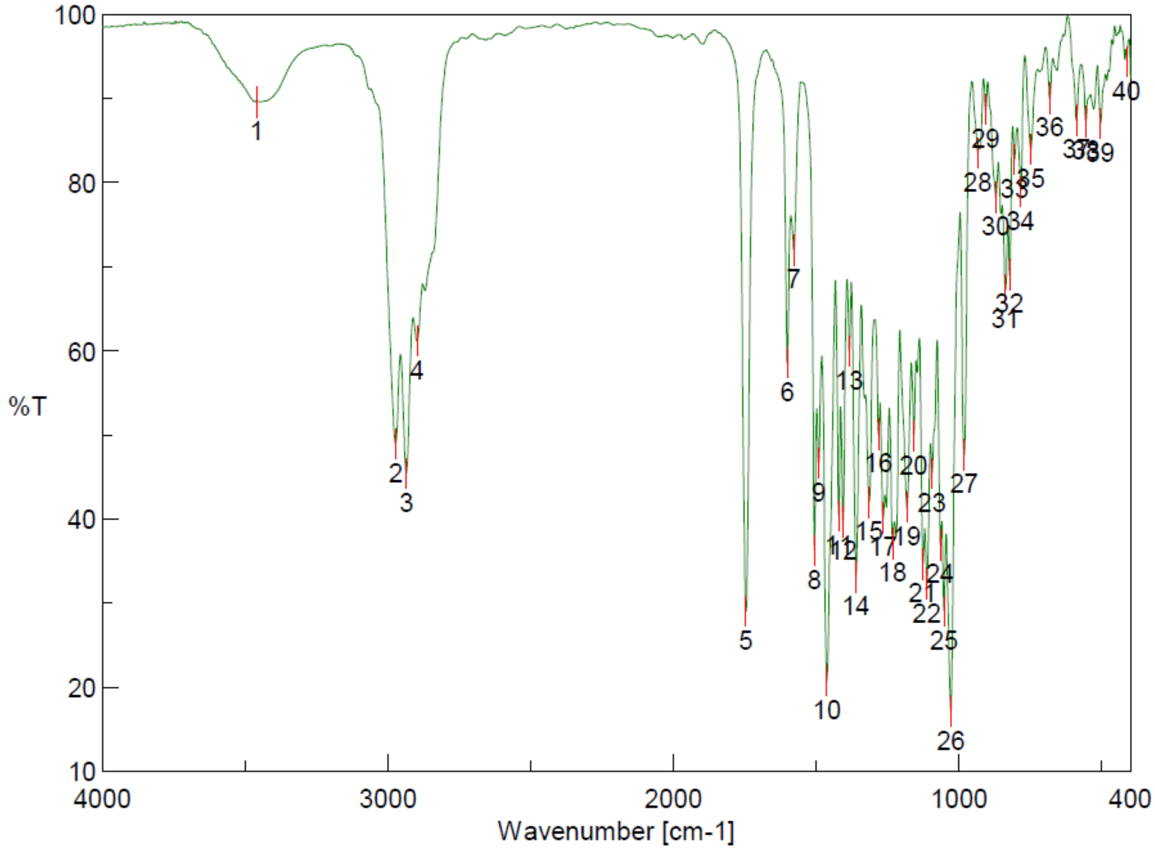

Figure S259. Experimental IR spectrum of (a*R*,1*S*,3*S*,1’*S*,3’*S*)-**22** recorded as KBr disc.

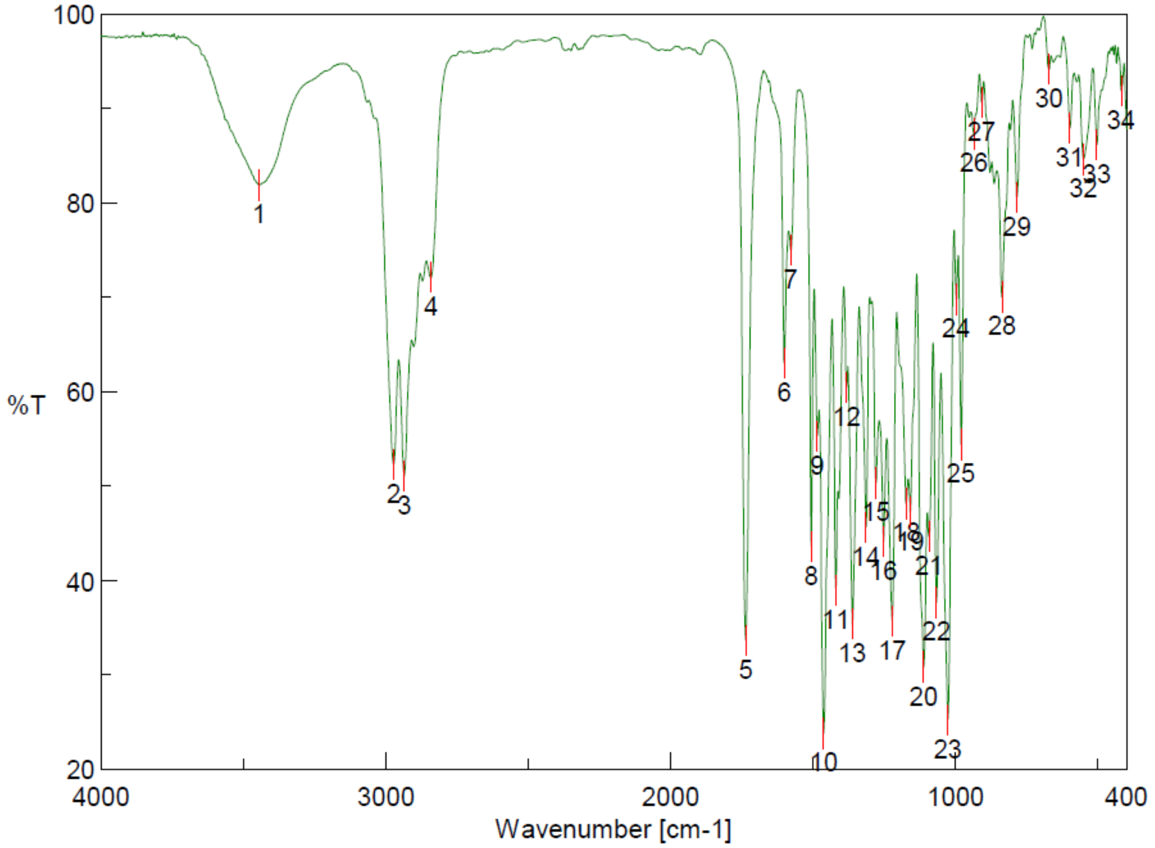

Figure S260. Experimental IR spectrum of (a*S*,1*S*,3*S*,1’*R*,3’*S*)-**23** recorded as KBr disc.

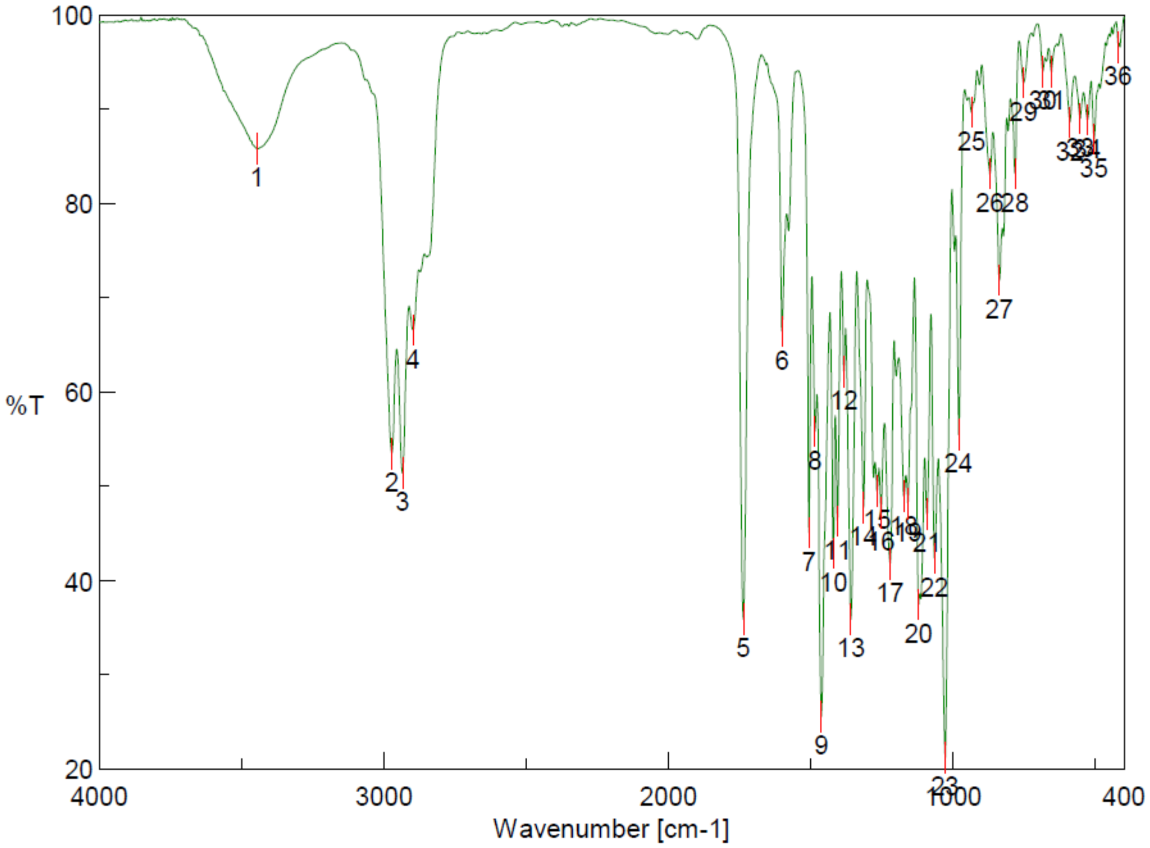

Figure S261. Experimental IR spectrum of (a*R*,1*S*,3*S*,1’*R*,3’*S*)-**23** recorded as KBr disc.

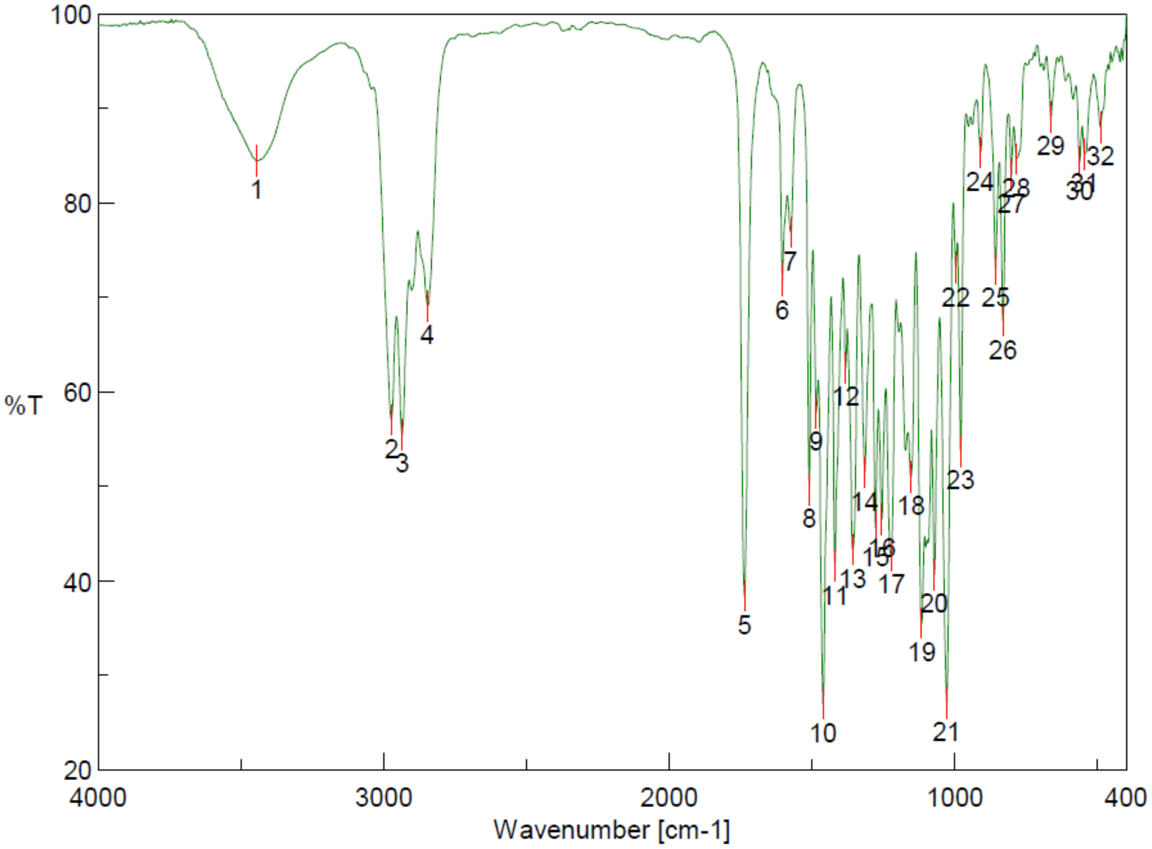

Figure S262. Experimental IR spectrum of (a*S*,1*R*,3*S*,1’*R*,3’*S*)-**23** recorded as KBr disc.

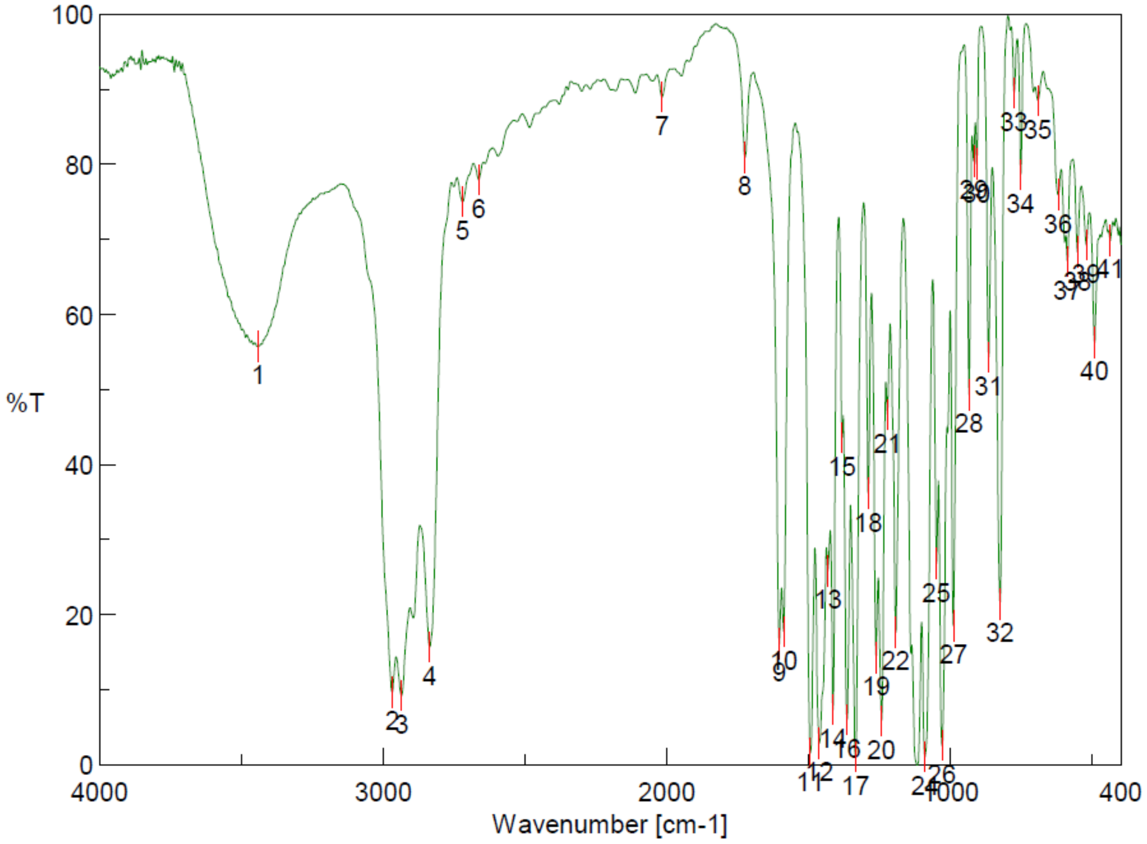

Figure S263. Experimental IR spectrum of (*S*)-**24** recorded as KBr disc.

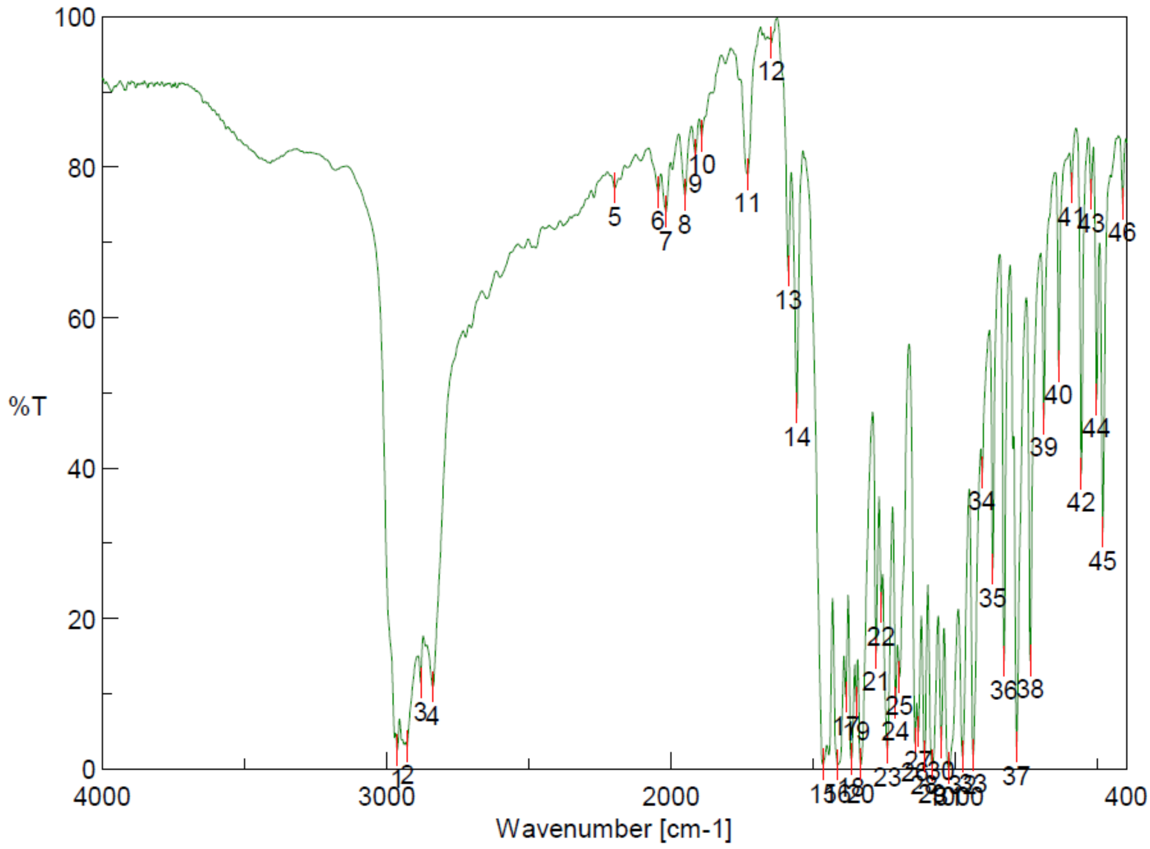

Figure S264. Experimental IR spectrum of (*S*)-**25** recorded as KBr disc.

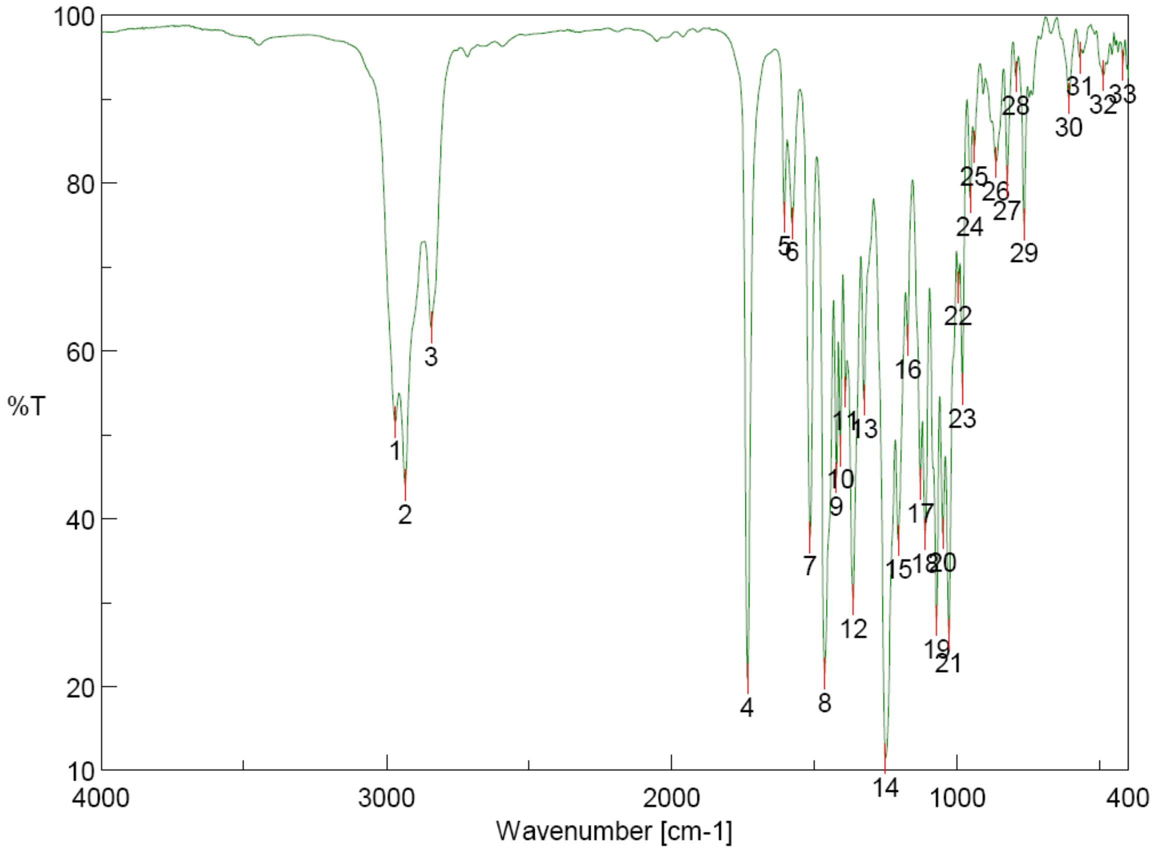

Figure S265. Experimental IR spectrum of the mixture of (a*R*,3*S*,2’*S*)-**26** and (a*S*,3*S*,2’*S*)-**26** recorded as KBr disc.

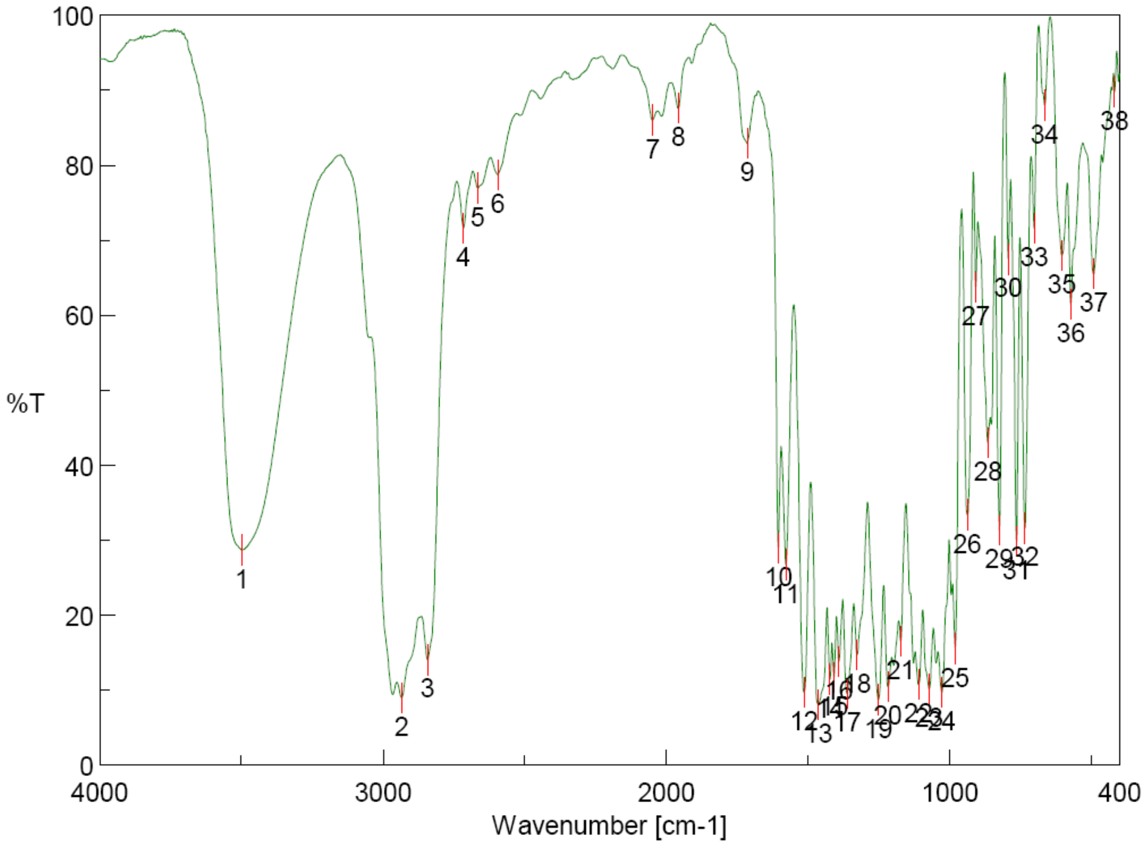

Figure S266. Experimental IR spectrum of the mixture of (a*R*,3*S*,2'*S*)-**27** and (a*S*,3*S*,2'*S*)-**27** recorded as KBr disc.

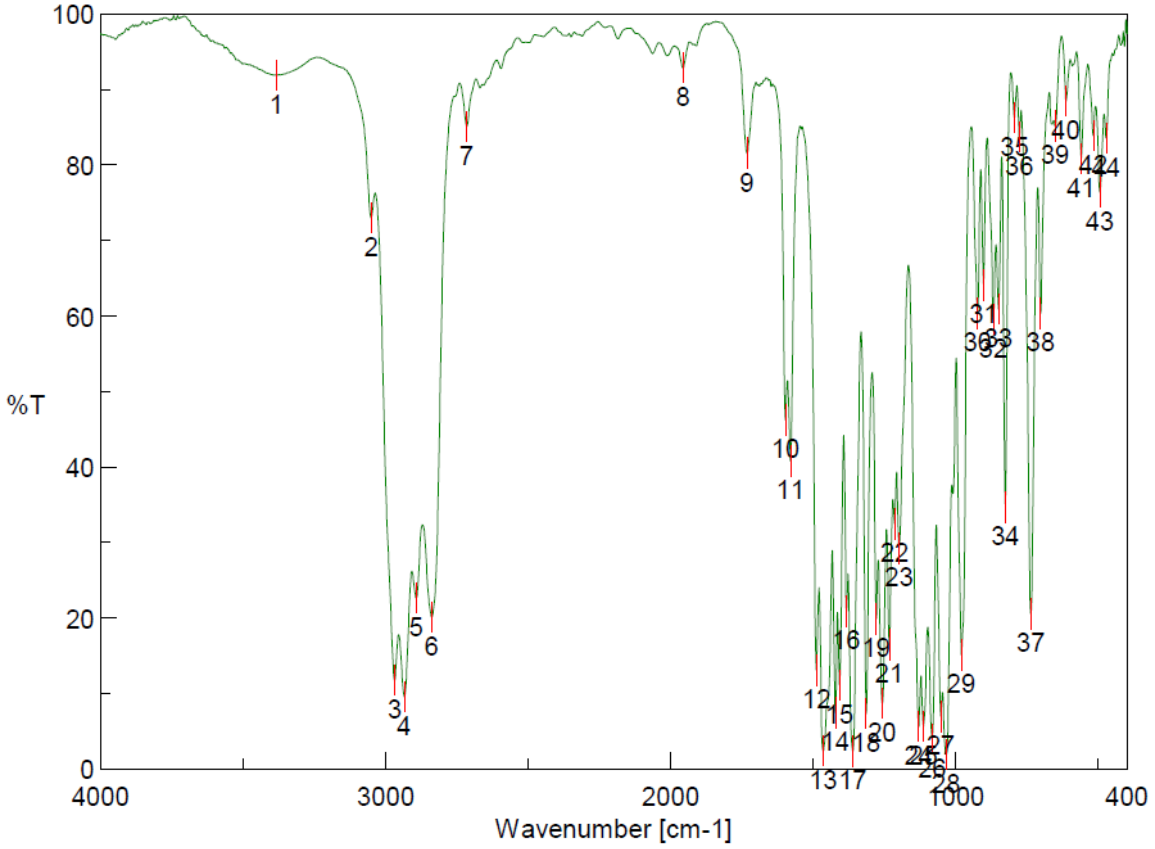

Figure S267. Experimental IR spectrum of (a*R*,3*S*,3'*S*)-**28** recorded as KBr disc.

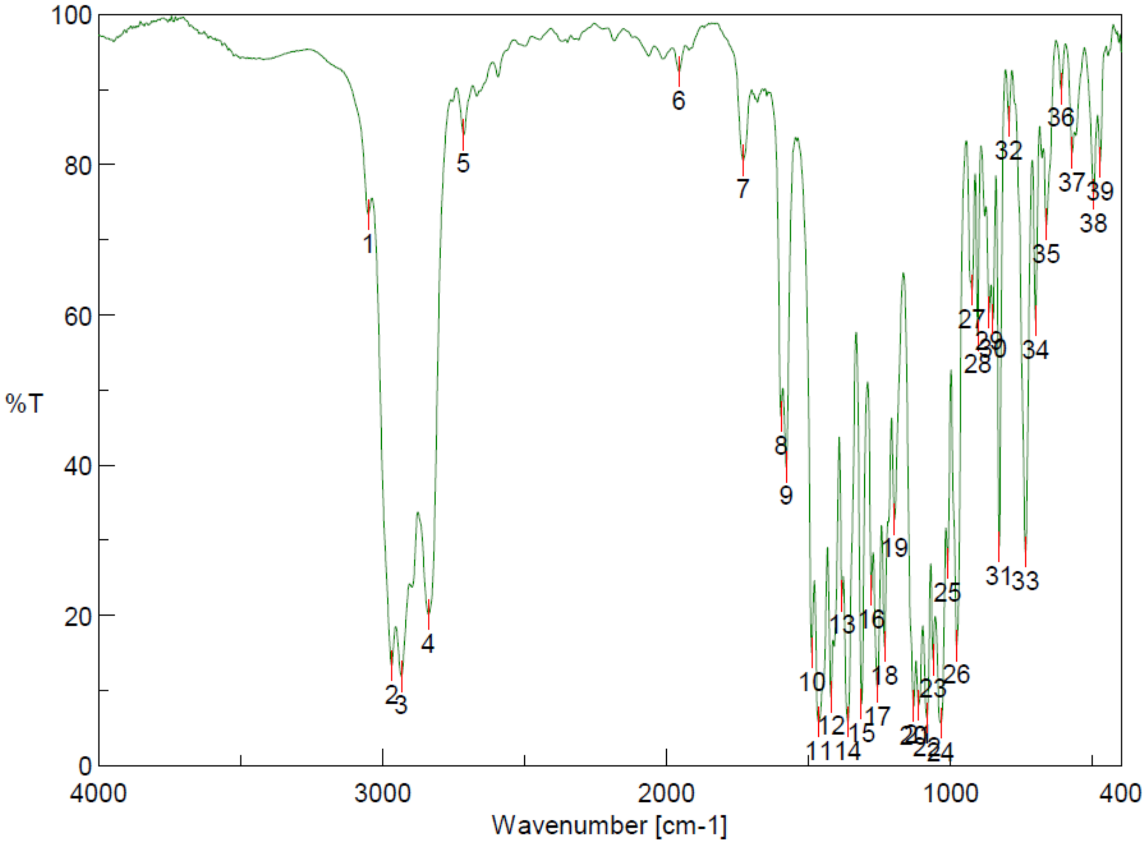

Figure S268. Experimental IR spectrum of (a*S*,3*S*,3'*S*)-**28** recorded as KBr disc.

4. X-ray analysis

X-ray-quality crystals were grown by slow evaporation of the sample dissolved in a mixture of hexane and EtOH. A crystal well-looking in polarized light microscope was fixed under a microscope onto a Mitegen loop using high-density oil. Diffraction intensity data were collected at room temperature and in case of (a*S*,1*R*,3*R*,1'*R*,3'*R*)-**22** at low temperature (120 K) using a Bruker-D8 Venture diffractometer (Bruker AXS GmbH, Karlsruhe, Germany) equipped with INCOATEC IμS 3.0 (Incoatec GmbH, Geesthacht, Germany) dual (Cu and Mo) sealed tube micro sources and a Photon II Charge-Integrating Pixel Array detector (Bruker AXS GmbH, Karlsruhe, Germany) using Mo Kα ($\lambda = 0.71073$ Å) radiation. The absolute configurations were assigned relative of the known configuration of C-3 and hence the applied radiation the Flack parameter is meaningless [11], but the assignment is unambiguous. Other spectroscopic methods (VCD, ECD) support the 100% enantiopurity of the samples. High multiplicity data collection and integration were performed using APEX4 (version 2021-4.0, Bruker AXS Inc., 2021, Madison, USA) software. Data reduction and multi-scan absorption correction were performed using SAINT (version 8.40B, Bruker AXS Inc., 2019, Madison, USA). The structures were routinely solved using direct methods and refined on F^2 using the SHELXL program [12] incorporated into the APEX4 suite. Refinement was performed anisotropically for all non-hydrogen atoms. Hydrogen atoms were placed into geometric positions except for the hydrogen atoms of solvent ethanol molecules in the structure of (a*S*,1*R*,3*R*,1'*R*,3'*R*)-**22**. These atoms could be found on the difference electron density map and the respective O-H distances should be restrained. Multi-scan absorption correction had to be applied because of the irregular shape of the crystals especially in the case of (a*S*,1*R*,3*R*,3'*R*)-**21** (large crystals) and (a*S*,1*R*,3*R*,1'*R*,3'*R*)-**22** (very small needle crystals). In latter case, RIGU command was used to regulate the refinement resulting in significant number of restrains. Further experimental details are shown in Table S1. The CIF file was manually edited using Publcif software [13], while graphics were prepared by using the Mercury program [14]. The results for the X-ray diffraction structure determinations were good enough and acceptable according to the Checkcif functionality of the PLATON software (Utrecht University, Utrecht, The Netherlands) [15]. Structural parameters such as bond length and angle data were in the expected range (Figure S269 and Table S2, Figure S270 and Table S3 as well as Figure S271 and Table S4 for (a*R*,1*S*,3*S*,3'*S*)-**21**, (a*S*,1*R*,3*R*,3'*R*)-**21** and (a*S*,1*R*,3*R*,1'*R*,3'*R*)-**22**, respectively. The solid-state structures are stabilized by van der Waals interactions as well as by weak C-H··O and in case of (a*S*,1*R*,3*R*,1'*R*,3'*R*)-**22** by strong O-H··O hydrogen bonds. As expected, (a*R*,1*S*,3*S*,3'*S*)-**21** and (a*S*,1*R*,3*R*,3'*R*)-**21** crystallized in unit cells of identical

geometric parameters. The most interesting structure is (a*S*,1*R*,3*R*,1'*R*,3'*R*)-**22** in which there were three molecules in the asymmetric unit together with two (slightly disordered) solvent ethanol molecules. There were only minor conformational differences among the molecules (Figure S272) and they have the same axial chirality.

The supplementary crystallographic data can be obtained free of charge from the Cambridge Crystallographic Data Centre via http://www.ccdc.cam.ac.uk/data_request/cif, using reference deposition number 2249290 for (a*R*,1*S*,3*S*,3'*S*)-**21**, 2249291 for (a*S*,1*R*,3*R*,3'*R*)-**21**, and 2249292 for (a*S*,1*R*,3*R*,1'*R*,3'*R*)-**22**.

4.1. Data of the X-ray analysis

Table S1. Experimental details of the structural elucidation by single crystal X-ray diffraction.

Compound	(a*R*,1*S*,3*S*,3'*S*)-**21**	(a*S*,1*R*,3*R*,3'*R*)-**21**	(a*S*,1*R*,3*R*,1'*R*,3'*R*)-**22**
Crystal data			
Chemical formula	$C_{31}H_{35}FO_7$	$C_{31}H_{35}FO_7$	$3(C_{34}H_{39}FO_9)\cdot 2(C_2H_6O)$
M_r	538.59	538.59	1924.08
Crystal system, space group	Monoclinic, $P2_1$	Monoclinic, $P2_1$	Triclinic, $P1$
Temperature (K)	295	302	120
a, b, c (Å)	8.2171 (10), 15.7142 (16), 11.5070 (14)	8.1885 (7), 15.6600 (14), 11.4825 (10)	9.8692 (15), 11.5507 (18), 22.357 (3)
α,β,γ (°)	90, 108.055 (4), 90	90, 108.025 (3), 90	85.927 (8), 82.008 (8), 87.066 (8)
V (Å^3)	1412.7 (3)	1400.2 (2)	2515.3 (7)
Z	2	2	1
Radiation type	Mo K		
μ (mm^{-1})	0.09	0.09	0.10
Crystal size (mm)	0.32 × 0.21 × 0.07	0.55 × 0.47 × 0.31	0.29 × 0.07 × 0.04
Data collection			
Diffractometer	Bruker D8 VENTURE		
Absorption correction	Multi-scan *SADABS2016*/2 - Bruker AXS area detector scaling and absorption correction		
T_{min}, T_{max}	0.97, 0.99	0.95, 0.97	0.97, 1.00
No. of measured, independent and observed [$I > 2\sigma(I)$]	13016, 5165, 2976	17212, 5645, 3805	80197, 17554, 10225

reflections			
R_{int}	0.084	0.112	0.191
(sin Θ / λ)$_{max}$ (Å^{-1})	0.604	0.627	0.604
Refinement			
$R[F^2 > 2\sigma(F^2)]$, $wR(F^2)$, S	0.070, 0.194, 1.06	0.077, 0.203, 0.99	0.104, 0.231, 1.08
No. of reflections	5165	5645	17554
No. of parameters	360	360	1272
No. of restraints	1	1	1086
H-atom treatment	H-atom parameters constrained		H atoms treated by a mixture of independent and constrained refinement
$\Delta>_{max}$, $\Delta>_{min}$ (e Å^{-3})	0.57, −0.60	0.40, −0.50	0.54, −0.53

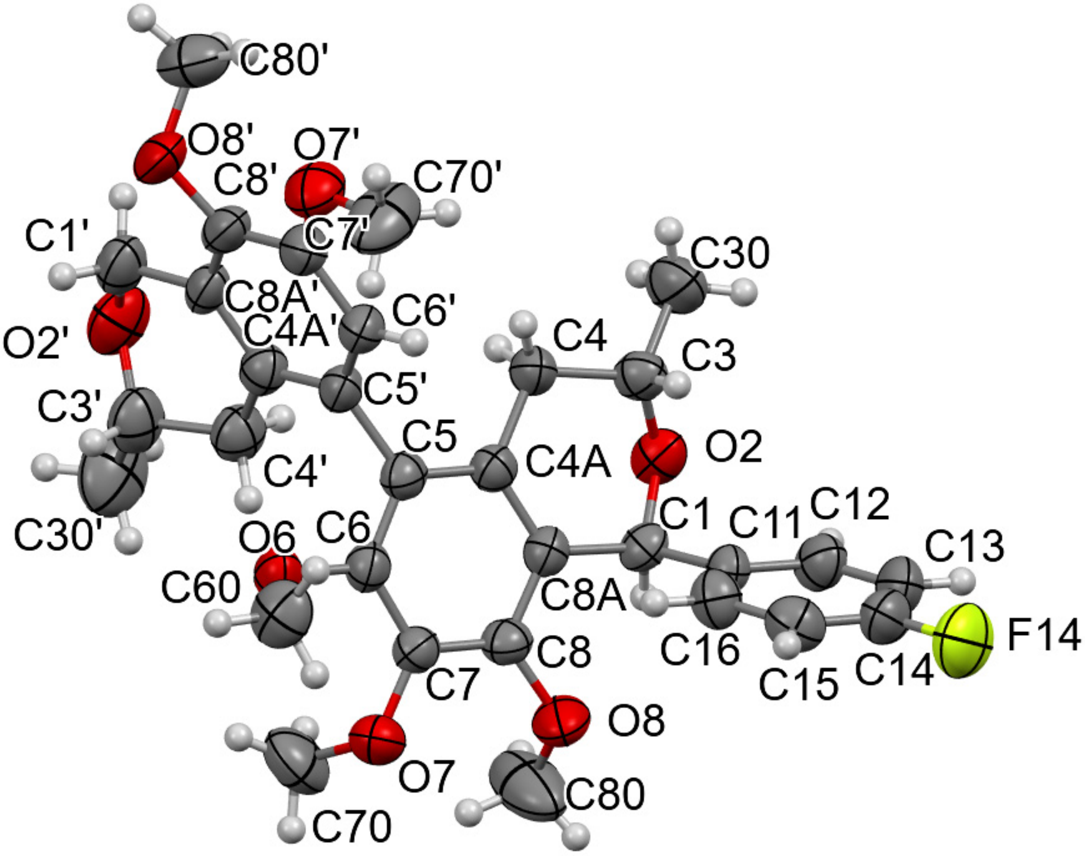

Figure S269. ORTEP view of (a*R*,1*S*,3*S*,3'*S*)-**21** at 50% probability level with numbering scheme.

Table S2. Geometric parameters (Å, º) for (a*R*,1*S*,3*S*,3'*S*)-**21**.

Bond distances (Å)

C1—O2	1.433 (8)	C8'—C8A'	1.392 (11)
C1—C8A	1.502 (9)	C11—C12	1.379 (10)
C1—C11	1.529 (9)	C11—C16	1.381 (10)
C1—H1	0.9800	C12—C13	1.380 (11)
C1'—O2'	1.430 (11)	C12—H12	0.9300
C1'—C8A'	1.520 (10)	C13—C14	1.358 (12)
C1'—H1'A	0.9700	C13—H13	0.9300
C1'—H1'B	0.9700	C14—F14	1.354 (9)
C3—O2	1.428 (9)	C14—C15	1.362 (11)
C3—C30	1.518 (10)	C15—C16	1.384 (11)
C3—C4	1.532 (10)	C15—H5'	0.9300
C3—H3	0.9800	C16—H6'	0.9300
C3'—O2'	1.427 (10)	C30—H30A	0.9600
C3'—C30'	1.490 (13)	C30—H30B	0.9600
C3'—C4'	1.496 (10)	C30—H30C	0.9600
C3'—H3'	0.9800	C30'—H30D	0.9600
C4—C4A	1.521 (9)	C30'—H30E	0.9600
C4—H4A	0.9700	C30'—H30F	0.9600
C4—H4B	0.9700	C60—O6	1.432 (9)
C4'—C4A'	1.493 (11)	C60—H60A	0.9600
C4'—H4'A	0.9700	C60—H60B	0.9600
C4'—H4'B	0.9700	C60—H60C	0.9600
C5—C4A	1.393 (9)	C70—O7	1.427 (9)
C5—C6	1.397 (9)	C70—H70A	0.9600
C5—C5'	1.509 (10)	C70—H70B	0.9600
C4A—C8A	1.401 (9)	C70—H70C	0.9600
C5'—C4A'	1.392 (9)	C70'—O7'	1.413 (11)
C5'—C6'	1.400 (10)	C70'—H70D	0.9600
C6—C7	1.378 (10)	C70'—H70E	0.9600
C6—O6	1.389 (8)	C70'—H7`E	0.9600
C6'—C7'	1.386 (10)	C80—O8	1.368 (12)
C6'—H6''	0.9300	C80—H80A	0.9600
C7—O7	1.381 (8)	C80—H80B	0.9600
C7—C8	1.389 (10)	C80—H80C	0.9600
C7'—O7'	1.386 (10)	C80'—O8'	1.422 (10)

C7'—C8'	1.392 (10)	C80'—H80D	0.9600
C8—O8	1.369 (9)	C80'—H80E	0.9600
C8—C8A	1.393 (9)	C80'—H80F	0.9600
C8'—O8'	1.385 (9)	C4A'—C8A'	1.384 (10)
Angles (º)			
O2—C1—C8A	111.6 (5)	C11—C12—H12	119.6000
O2—C1—C11	110.2 (5)	C13—C12—H12	119.6000
C8A—C1—C11	114.4 (5)	C14—C13—C12	119.3 (8)
O2—C1—H1	106.7000	C14—C13—H13	120.4000
C8A—C1—H1	106.7000	C12—C13—H13	120.4000
C11—C1—H1	106.7000	F14—C14—C13	119.1 (8)
O2'—C1'—C8A'	112.9 (7)	F14—C14—C15	119.5 (8)
O2'—C1'—H1'A	109.0000	C13—C14—C15	121.5 (8)
C8A'—C1'—H1'A	109.0000	C14—C15—C16	119.4 (8)
O2'—C1'—H1'B	109.0000	C14—C15—H5'	120.3000
C8A'—C1'—H1'B	109.0000	C16—C15—H5'	120.3000
H1'A—C1'—H1'B	107.8000	C11—C16—C15	120.3 (7)
O2—C3—C30	109.1 (6)	C11—C16—H6'	119.8000
O2—C3—C4	109.8 (5)	C15—C16—H6'	119.8000
C30—C3—C4	112.1 (7)	C3—C30—H30A	109.5000
O2—C3—H3	108.6000	C3—C30—H30B	109.5000
C30—C3—H3	108.6000	H30A—C30—H30B	109.5000
C4—C3—H3	108.6000	C3—C30—H30C	109.5000
O2'—C3'—C30'	107.8 (8)	H30A—C30—H30C	109.5000
O2'—C3'—C4'	108.8 (7)	H30B—C30—H30C	109.5000
C30'—C3'—C4'	113.4 (8)	C3'—C30'—H30D	109.5000
O2'—C3'—H3'	108.9000	C3'—C30'—H30E	109.5000
C30'—C3'—H3'	108.9000	H30D—C30'—H30E	109.5000
C4'—C3'—H3'	108.9000	C3'—C30'—H30F	109.5000
C4A—C4—C3	111.7 (6)	H30D—C30'—H30F	109.5000
C4A—C4—H4A	109.3000	H30E—C30'—H30F	109.5000
C3—C4—H4A	109.3000	O6—C60—H60A	109.5000
C4A—C4—H4B	109.3000	O6—C60—H60B	109.5000
C3—C4—H4B	109.3000	H60A—C60—H60B	109.5000
H4A—C4—H4B	107.9000	O6—C60—H60C	109.5000

C4A'—C4'—C3'	112.5 (7)	H60A—C60—H60C	109.5000
C4A'—C4'—H4'A	109.1000	H60B—C60—H60C	109.5000
C3'—C4'—H4'A	109.1000	O7—C70—H70A	109.5000
C4A'—C4'—H4'B	109.1000	O7—C70—H70B	109.5000
C3'—C4'—H4'B	109.1000	H70A—C70—H70B	109.5000
H4'A—C4'—H4'B	107.8000	O7—C70—H70C	109.5000
C4A—C5—C6	119.1 (6)	H70A—C70—H70C	109.5000
C4A—C5—C5'	122.2 (6)	H70B—C70—H70C	109.5000
C6—C5—C5'	118.7 (6)	O7'—C70'—H70D	109.5000
C5—C4A—C8A	120.0 (6)	O7'—C70'—H70E	109.5000
C5—C4A—C4	120.9 (6)	H70D—C70'—H70E	109.5000
C8A—C4A—C4	119.0 (6)	O7'—C70'—H7`E	109.5000
C4A'—C5'—C6'	120.8 (7)	H70D—C70'—H7`E	109.5000
C4A'—C5'—C5	119.4 (6)	H70E—C70'—H7`E	109.5000
C6'—C5'—C5	119.7 (6)	O8—C80—H80A	109.5000
C7—C6—O6	119.6 (6)	O8—C80—H80B	109.5000
C7—C6—C5	121.4 (6)	H80A—C80—H80B	109.5000
O6—C6—C5	118.9 (6)	O8—C80—H80C	109.5000
C7'—C6'—C5'	119.8 (6)	H80A—C80—H80C	109.5000
C7'—C6'—H6"	120.1000	H80B—C80—H80C	109.5000
C5'—C6'—H6"	120.1000	O8'—C80'—H80D	109.5000
C6—C7—O7	121.3 (6)	O8'—C80'—H80E	109.5000
C6—C7—C8	119.1 (6)	H80D—C80'—H80E	109.5000
O7—C7—C8	119.5 (6)	O8'—C80'—H80F	109.5000
C6'—C7'—O7'	123.7 (7)	H80D—C80'—H80F	109.5000
C6'—C7'—C8'	119.5 (7)	H80E—C80'—H80F	109.5000
O7'—C7'—C8'	116.7 (7)	C8A'—C4A'—C5'	118.8 (7)
O8—C8—C7	121.9 (6)	C8A'—C4A'—C4'	119.0 (7)
O8—C8—C8A	117.4 (6)	C5'—C4A'—C4'	122.2 (7)
C7—C8—C8A	120.7 (6)	C4A'—C8A'—C8'	120.7 (7)
O8'—C8'—C7'	121.7 (8)	C4A'—C8A'—C1'	120.9 (8)
O8'—C8'—C8A'	117.8 (7)	C8'—C8A'—C1'	118.3 (7)
C7'—C8'—C8A'	120.3 (7)	C3—O2—C1	112.7 (5)
C8—C8A—C4A	119.5 (6)	C3'—O2'—C1'	112.3 (6)
C8—C8A—C1	118.6 (6)	C6—O6—C60	113.7 (6)

C4A—C8A—C1	121.9 (6)	C7—O7—C70	113.4 (6)
C12—C11—C16	118.8 (7)	C7'—O7'—C70'	117.4 (6)
C12—C11—C1	118.2 (6)	C80—O8—C8	119.8 (8)
C16—C11—C1	123.0 (6)	C8'—O8'—C80'	117.3 (6)
C11—C12—C13	120.8 (7)		
Torsion angles (º)			
O2—C3—C4—C4A	47.5 (8)	C8A—C1—C11—C12	−177.3 (7)
C30—C3—C4—C4A	169.0 (7)	O2—C1—C11—C16	132.3 (7)
O2'—C3'—C4'—C4A'	52.7 (9)	C8A—C1—C11—C16	5.5 (9)
C30'—C3'—C4'—C4A'	172.7 (8)	C16—C11—C12—C13	0.4 (12)
C6—C5—C4A—C8A	0.1 (9)	C1—C11—C12—C13	−176.9 (7)
C5'—C5—C4A—C8A	−176.3 (6)	C11—C12—C13—C14	0.3 (12)
C6—C5—C4A—C4	176.6 (6)	C12—C13—C14—F14	179.4 (7)
C5'—C5—C4A—C4	0.2 (9)	C12—C13—C14—C15	−0.3 (13)
C3—C4—C4A—C5	166.1 (6)	F14—C14—C15—C16	179.8 (7)
C3—C4—C4A—C8A	−17.4 (9)	C13—C14—C15—C16	−0.5 (13)
C4A—C5—C5'—C4A'	101.6 (7)	C12—C11—C16—C15	−1.2 (11)
C6—C5—C5'—C4A'	−74.8 (8)	C1—C11—C16—C15	175.9 (7)
C4A—C5—C5'—C6'	−78.8 (8)	C14—C15—C16—C11	1.3 (12)
C6—C5—C5'—C6'	104.8 (7)	C6'—C5'—C4A'—C8A'	1.1 (10)
C4A—C5—C6—C7	−4.4 (9)	C5—C5'—C4A'—C8A'	−179.3 (6)
C5'—C5—C6—C7	172.1 (6)	C6'—C5'—C4A'—C4'	−178.4 (7)
C4A—C5—C6—O6	−179.8 (6)	C5—C5'—C4A'—C4'	1.3 (10)
C5'—C5—C6—O6	-3.3 (9)	C3'—C4'—C4A'—C8A'	−20.3 (10)
C4A'—C5'—C6'—C7'	0.5 (10)	C3'—C4'—C4A'—C5'	159.2 (7)
C5—C5'—C6'—C7'	−179.2 (6)	C5'—C4A'—C8A'—C8'	−2.2 (10)
O6—C6—C7—O7	0.1 (10)	C4'—C4A'—C8A'—C8'	177.3 (6)
C5—C6—C7—O7	−175.2 (6)	C5'—C4A'—C8A'—C1'	−179.3 (6)
O6—C6—C7—C8	−179.1 (6)	C4'—C4A'—C8A'—C1'	0.2 (10)
C5—C6—C7—C8	5.6 (10)	O8'—C8'—C8A'—C4A'	−171.8 (6)
C5'—C6'—C7'—O7'	176.2 (6)	C7'—C8'—C8A'—C4A'	1.8 (10)
C5'—C6'—C7'—C8'	−0.9 (10)	O8'—C8'—C8A'—C1'	5.3 (10)
C6—C7—C8—O8	175.1 (6)	C7'—C8'—C8A'—C1'	178.9 (7)
O7—C7—C8—O8	−4.1 (10)	O2'—C1'—C8A'—C4A'	−12.4 (10)
C6—C7—C8—C8A	−2.4 (10)	O2'—C1'—C8A'—C8'	170.5 (6)

O7—C7—C8—C8A	178.4 (6)	C30—C3—O2—C1	169.2 (6)
C6'—C7'—C8'—O8'	173.1 (7)	C4—C3—O2—C1	−67.6 (7)
O7'—C7'—C8'—O8'	−4.2 (10)	C8A—C1—O2—C3	52.0 (7)
C6'—C7'—C8'—C8A'	−0.2 (10)	C11—C1—O2—C3	−76.3 (7)
O7'—C7'—C8'—C8A'	−177.5 (6)	C30'—C3'—O2'—C1'	169.0 (8)
O8—C8—C8A—C4A	−179.4 (6)	C4'—C3'—O2'—C1'	−67.6 (9)
C7—C8—C8A—C4A	−1.8 (10)	C8A'—C1'—O2'—C3'	46.4 (9)
O8—C8—C8A—C1	3.0 (9)	C7—C6—O6—C60	68.8 (9)
C7—C8—C8A—C1	−179.4 (6)	C5—C6—O6—C60	−115.8 (7)
C5—C4A—C8A—C8	2.9 (9)	C6—C7—O7—C70	70.3 (9)
C4—C4A—C8A—C8	−173.6 (6)	C8—C7—O7—C70	−110.5 (8)
C5—C4A—C8A—C1	−179.5 (6)	C6'—C7'—O7'—C70'	−7.5 (11)
C4—C4A—C8A—C1	3.9 (9)	C8'—C7'—O7'—C70'	169.8 (8)
O2—C1—C8A—C8	157.9 (6)	C7—C8—O8—C80	64.6 (11)
C11—C1—C8A—C8	−76.1 (8)	C8A—C8—O8—C80	−117.8 (9)
O2—C1—C8A—C4A	−19.7 (8)	C7'—C8'—O8'—C80'	67.2 (9)
C11—C1—C8A—C4A	106.3 (7)	C8A'—C8'—O8'—C80'	−119.2 (8)
O2—C1—C11—C12	−50.5 (8)		

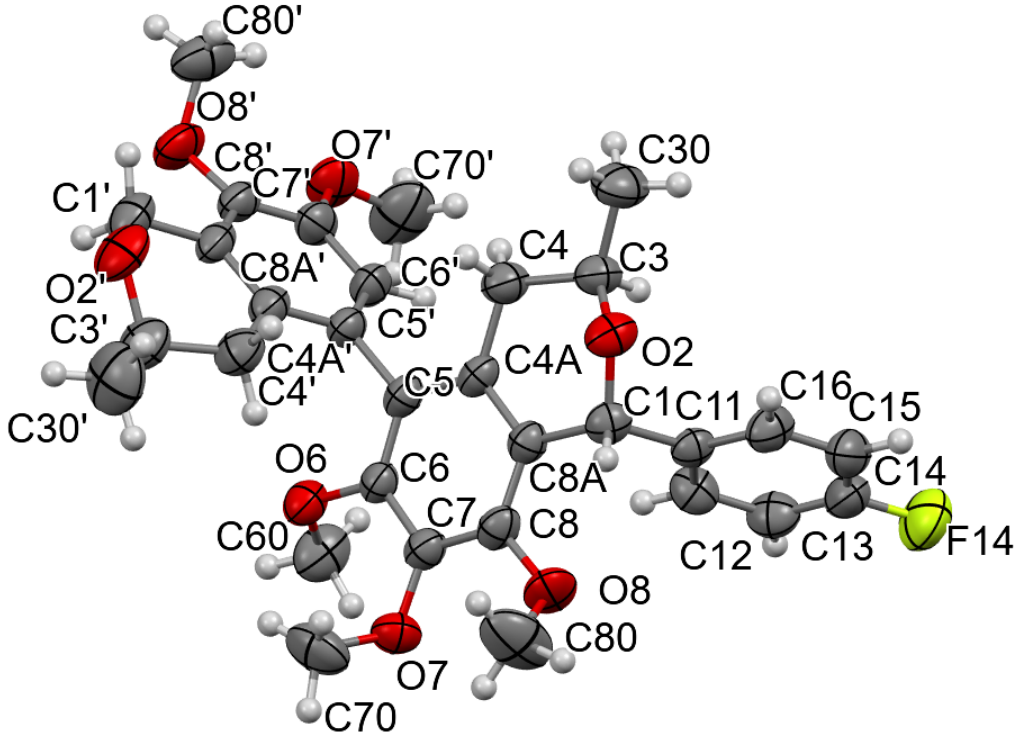

Figure S270. ORTEP view of (a*S*,1*R*,3*R*,3'*R*)-**21** at 50% probability level with numbering scheme.

Table S3. Geometric parameters (Å, º) for (a*S*,1*R*,3*R*,3'*R*)-**21**.

Distances (Å)			
C1—O2	1.425 (6)	C8'—C8A'	1.389 (8)
C1—C8A	1.508 (7)	C11—C16	1.367 (7)
C1—C11	1.533 (7)	C11—C12	1.384 (8)
C1—H1	0.9800	C12—C13	1.384 (8)
C1'—O2'	1.429 (8)	C12—H12	0.9300
C1'—C8A'	1.504 (8)	C13—C14	1.341 (9)
C1'—H1'A	0.9700	C13—H13	0.9300
C1'—H1'B	0.9700	C14—F14	1.361 (7)
C3—O2	1.433 (5)	C14—C15	1.371 (9)
C3—C30	1.505 (8)	C15—C16	1.367 (9)
C3—C4	1.518 (7)	C15—H15	0.9300
C3—H3	0.9800	C16—H16	0.9300
C3'—O2'	1.440 (8)	C30—H30A	0.9600
C3'—C4'	1.484 (8)	C30—H30B	0.9600
C3'—C30'	1.502 (10)	C30—H30C	0.9600
C3'—H3'	0.9800	C30'—H30D	0.9600
C4—C4A	1.507 (7)	C30'—H30E	0.9600
C4—H4A	0.9700	C30'—H30F	0.9600
C4—H4B	0.9700	C60—O6	1.436 (7)
C4'—C4A'	1.502 (8)	C60—H60A	0.9600
C4'—H4'A	0.9700	C60—H60B	0.9600
C4'—H4'B	0.9700	C60—H60C	0.9600
C5—C6	1.385 (7)	C70—O7	1.411 (8)
C5—C4A	1.396 (7)	C70—H70A	0.9600
C5—C5'	1.495 (6)	C70—H70B	0.9600
C4A—C8A	1.402 (6)	C70—H70C	0.9600
C5'—C6'	1.386 (7)	C70'—O7'	1.396 (9)
C5'—C4A'	1.394 (7)	C70'—H70D	0.9600
C6—O6	1.378 (6)	C70'—H70E	0.9600
C6—C7	1.409 (6)	C70'—H70F	0.9600
C6'—C7'	1.382 (6)	C80—O8	1.379 (9)
C6'—H6'	0.9300	C80—H80D	0.9600

C7—C8	1.368 (7)	C80—H80E	0.9600
C7—O7	1.370 (6)	C80—H80F	0.9600
C7'—C8'	1.387 (8)	C80'—O8'	1.413 (8)
C7'—O7'	1.387 (7)	C80'—H80A	0.9600
C8—O8	1.376 (5)	C80'—H80B	0.9600
C8—C8A	1.387 (7)	C80'—H80C	0.9600
C8'—O8'	1.380 (6)	C4A'—C8A'	1.394 (6)
Angles (º)			
O2—C1—C8A	111.6 (4)	C13—C12—H12	120.3000
O2—C1—C11	110.7 (4)	C11—C12—H12	120.3000
C8A—C1—C11	113.8 (4)	C14—C13—C12	119.6 (6)
O2—C1—H1	106.8000	C14—C13—H13	120.2000
C8A—C1—H1	106.8000	C12—C13—H13	120.2000
C11—C1—H1	106.8000	C13—C14—F14	120.3 (6)
O2'—C1'—C8A'	113.4 (4)	C13—C14—C15	122.3 (5)
O2'—C1'—H1'A	108.9000	F14—C14—C15	117.4 (6)
C8A'—C1'—H1'A	108.9000	C16—C15—C14	117.7 (5)
O2'—C1'—H1'B	108.9000	C16—C15—H15	121.1000
C8A'—C1'—H1'B	108.9000	C14—C15—H15	121.1000
H1'A—C1'—H1'B	107.7000	C15—C16—C11	122.0 (5)
O2—C3—C30	107.6 (5)	C15—C16—H16	119.0000
O2—C3—C4	109.1 (4)	C11—C16—H16	119.0000
C30—C3—C4	113.3 (5)	C3—C30—H30A	109.5000
O2—C3—H3	108.9000	C3—C30—H30B	109.5000
C30—C3—H3	108.9000	H30A—C30—H30B	109.5000
C4—C3—H3	108.9000	C3—C30—H30C	109.5000
O2'—C3'—C4'	108.3 (5)	H30A—C30—H30C	109.5000
O2'—C3'—C30'	107.6 (6)	H30B—C30—H30C	109.5000
C4'—C3'—C30'	113.3 (6)	C3'—C30'—H30D	109.5000
O2'—C3'—H3'	109.2000	C3'—C30'—H30E	109.5000
C4'—C3'—H3'	109.2000	H30D—C30'—H30E	109.5000
C30'—C3'—H3'	109.2000	C3'—C30'—H30F	109.5000
C4A—C4—C3	112.7 (4)	H30D—C30'—H30F	109.5000
C4A—C4—H4A	109.1000	H30E—C30'—H30F	109.5000
C3—C4—H4A	109.1000	O6—C60—H60A	109.5000

C4A—C4—H4B	109.1000	O6—C60—H60B	109.5000
C3—C4—H4B	109.1000	H60A—C60—H60B	109.5000
H4A—C4—H4B	107.8000	O6—C60—H60C	109.5000
C3'—C4'—C4A'	112.7 (5)	H60A—C60—H60C	109.5000
C3'—C4'—H4'A	109.0000	H60B—C60—H60C	109.5000
C4A'—C4'—H4'A	109.0000	O7—C70—H70A	109.5000
C3'—C4'—H4'B	109.0000	O7—C70—H70B	109.5000
C4A'—C4'—H4'B	109.0000	H70A—C70—H70B	109.5000
H4'A—C4'—H4'B	107.8000	O7—C70—H70C	109.5000
C6—C5—C4A	119.0 (4)	H70A—C70—H70C	109.5000
C6—C5—C5'	118.9 (4)	H70B—C70—H70C	109.5000
C4A—C5—C5'	122.0 (4)	O7'—C70'—H70D	109.5000
C5—C4A—C8A	120.0 (4)	O7'—C70'—H70E	109.5000
C5—C4A—C4	121.2 (4)	H70D—C70'—H70E	109.5000
C8A—C4A—C4	118.6 (4)	O7'—C70'—H70F	109.5000
C6'—C5'—C4A'	120.5 (4)	H70D—C70'—H70F	109.5000
C6'—C5'—C5	120.7 (4)	H70E—C70'—H70F	109.5000
C4A'—C5'—C5	118.7 (4)	O8—C80—H80D	109.5000
O6—C6—C5	119.3 (4)	O8—C80—H80E	109.5000
O6—C6—C7	119.4 (4)	H80D—C80—H80E	109.5000
C5—C6—C7	121.2 (4)	O8—C80—H80F	109.5000
C7'—C6'—C5'	120.2 (5)	H80D—C80—H80F	109.5000
C7'—C6'—H6'	119.9000	H80E—C80—H80F	109.5000
C5'—C6'—H6'	119.9000	O8'—C80'—H80A	109.5000
C8—C7—O7	121.0 (4)	O8'—C80'—H80B	109.5000
C8—C7—C6	118.5 (4)	H80A—C80'—H80B	109.5000
O7—C7—C6	120.5 (4)	O8'—C80'—H80C	109.5000
C6'—C7'—C8'	119.7 (5)	H80A—C80'—H80C	109.5000
C6'—C7'—O7'	123.9 (5)	H80B—C80'—H80C	109.5000
C8'—C7'—O7'	116.4 (4)	C8A'—C4A'—C5'	119.0 (5)
C7—C8—O8	121.5 (5)	C8A'—C4A'—C4'	118.6 (5)
C7—C8—C8A	121.7 (4)	C5'—C4A'—C4'	122.4 (4)
O8—C8—C8A	116.8 (4)	C8'—C8A'—C4A'	120.1 (5)
O8'—C8'—C7'	122.1 (5)	C8'—C8A'—C1'	119.1 (4)
O8'—C8'—C8A'	117.0 (5)	C4A'—C8A'—C1'	120.8 (5)

C7'—C8'—C8A'	120.5 (4)	C1—O2—C3	112.4 (4)
C8—C8A—C4A	119.3 (4)	C1'—O2'—C3'	111.9 (5)
C8—C8A—C1	119.0 (4)	C6—O6—C60	114.2 (4)
C4A—C8A—C1	121.6 (4)	C7—O7—C70	114.7 (5)
C16—C11—C12	118.8 (5)	C7'—O7'—C70'	117.4 (4)
C16—C11—C1	118.0 (4)	C8—O8—C80	118.7 (6)
C12—C11—C1	123.1 (4)	C8'—O8'—C80'	117.5 (5)
C13—C12—C11	119.5 (5)		
Torsion angles (º)			
O2—C3—C4—C4A	−48.3 (6)	C8A—C1—C11—C16	177.2 (4)
C30—C3—C4—C4A	−168.2 (5)	O2—C1—C11—C12	−132.0 (5)
O2'—C3'—C4'—C4A'	−52.7 (7)	C8A—C1—C11—C12	−5.4 (6)
C30'—C3'—C4'—C4A'	−172.0 (6)	C16—C11—C12—C13	1.5 (7)
C6—C5—C4A—C8A	−0.5 (7)	C1—C11—C12—C13	−175.9 (5)
C5'—C5—C4A—C8A	176.9 (4)	C11—C12—C13—C14	−0.8 (8)
C6—C5—C4A—C4	−176.4 (5)	C12—C13—C14—F14	−179.6 (5)
C5'—C5—C4A—C4	0.9 (7)	C12—C13—C14—C15	0.1 (9)
C3—C4—C4A—C5	−166.1 (5)	C13—C14—C15—C16	−0.2 (9)
C3—C4—C4A—C8A	17.9 (7)	F14—C14—C15—C16	179.5 (5)
C6—C5—C5'—C6'	−105.2 (6)	C14—C15—C16—C11	1.1 (9)
C4A—C5—C5'—C6'	77.5 (7)	C12—C11—C16—C15	−1.7 (8)
C6—C5—C5'—C4A'	75.1 (6)	C1—C11—C16—C15	175.8 (5)
C4A—C5—C5'—C4A'	−102.2 (5)	C6'—C5'—C4A'—C8A'	0.7 (8)
C4A—C5—C6—O6	−179.5 (4)	C5—C5'—C4A'—C8A'	−179.6 (5)
C5'—C5—C6—O6	3.1 (7)	C6'—C5'—C4A'—C4'	178.3 (5)

C4A—C5—C6—C7	4.1 (7)	C5—C5'—C4A'—C4'	−2.0 (7)
C5'—C5—C6—C7	−173.3 (4)	C3'—C4'—C4A'—C8A'	19.2 (7)
C4A'—C5'—C6'—C7'	−2.3 (8)	C3'—C4'—C4A'—C5'	−158.4 (5)
C5—C5'—C6'—C7'	178.1 (5)	O8'—C8'—C8A'—C4A'	172.9 (5)
O6—C6—C7—C8	179.0 (4)	C7'—C8'—C8A'—C4A'	−0.4 (8)
C5—C6—C7—C8	−4.6 (7)	O8'—C8'—C8A'—C1'	−6.2 (7)
O6—C6—C7—O7	−1.5 (7)	C7'—C8'—C8A'—C1'	−179.5 (5)
C5—C6—C7—O7	174.9 (4)	C5'—C4A'—C8A'—C8'	0.6 (7)
C5'—C6'—C7'—C8'	2.5 (8)	C4'—C4A'—C8A'—C8'	−177.1 (5)
C5'—C6'—C7'—O7'	−175.8 (5)	C5'—C4A'—C8A'—C1'	179.7 (5)
O7—C7—C8—O8	5.1 (7)	C4'—C4A'—C8A'—C1'	2.0 (8)
C6—C7—C8—O8	−175.4 (5)	O2'—C1'—C8A'—C8'	−169.9 (5)
O7—C7—C8—C8A	−178.0 (5)	O2'—C1'—C8A'—C4A'	11.0 (8)
C6—C7—C8—C8A	1.5 (7)	C8A—C1—O2—C3	−52.3 (5)
C6'—C7'—C8'—O8'	−174.1 (5)	C11—C1—O2—C3	75.6 (5)
O7'—C7'—C8'—O8'	4.3 (8)	C30—C3—O2—C1	−168.7 (5)
C6'—C7'—C8'—C8A'	−1.2 (8)	C4—C3—O2—C1	68.0 (6)
O7'—C7'—C8'—C8A'	177.3 (5)	C8A'—C1'—O2'—C3'	−46.4 (7)
C7—C8—C8A—C4A	2.0 (7)	C4'—C3'—O2'—C1'	68.2 (6)
O8—C8—C8A—C4A	179.0 (4)	C30'—C3'—O2'—C1'	−169.1 (6)
C7—C8—C8A—C1	179.2 (5)	C5—C6—O6—C60	115.4 (5)
O8—C8—C8A—C1	−3.7 (7)	C7—C6—O6—C60	−68.1 (6)
C5—C4A—C8A—C8	−2.5 (7)	C8—C7—O7—C70	111.2 (6)
C4—C4A—C8A—C8	173.5 (5)	C6—C7—O7—C70	−68.3 (7)
C5—C4A—C8A—C1	−179.7 (4)	C6'—C7'—O7'—C70'	8.3 (9)
C4—C4A—C8A—C1	−3.6 (7)	C8'—C7'—O7'—C70'	−170.0 (6)

O2—C1—C8A—C8	−157.6 (4)	C7—C8—O8—C80	−65.1 (9)
C11—C1—C8A—C8	76.2 (5)	C8A—C8—O8—C80	117.9 (7)
O2—C1—C8A—C4A	19.6 (6)	C7'—C8'—O8'—C80'	−66.9 (7)
C11—C1—C8A—C4A	−106.6 (5)	C8A'—C8'—O8'—C80'	119.9 (6)
O2—C1—C11—C16	50.5 (5)		

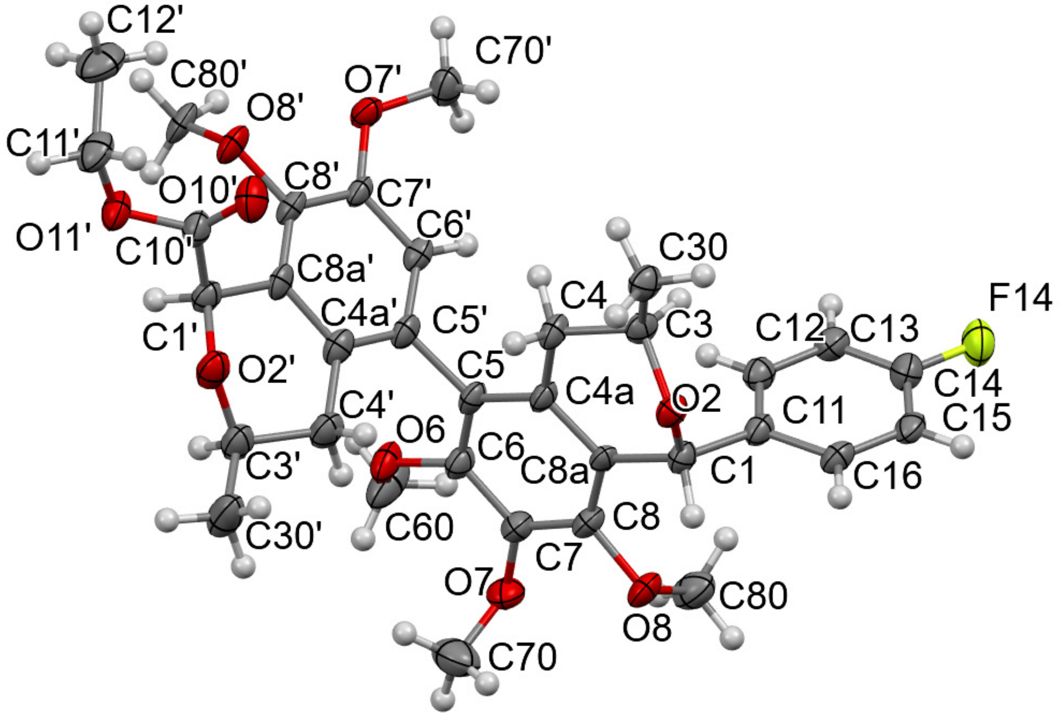

Figure S271. ORTEP view of (a*S*,1*R*,3*R*,1'*R*,3'*R*)-**22** at 50% probability level with numbering scheme. Only one molecule from the asymmetric unit is shown and solvent molecules are omitted for clarity. The numbering of the other molecules were systematically changed; *e.g.* C1 corresponds to C21 and C31.

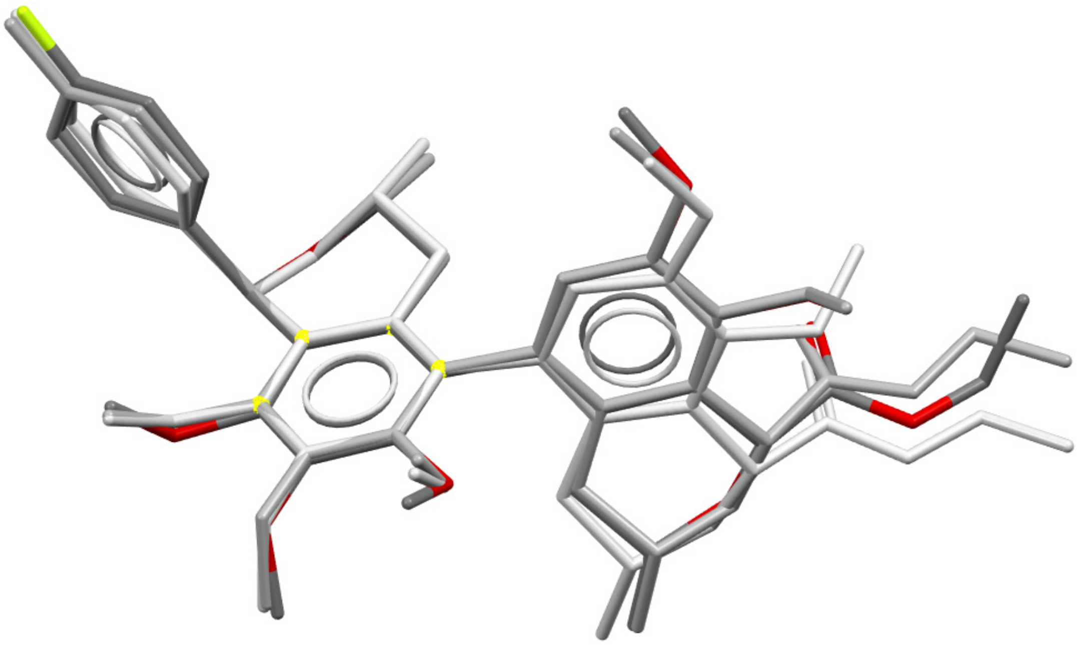

Figure S272. Overlay of the three isomers of in the asymmetric unit of structure (a*S*,1*R*,3*R*,1'*R*,3'*R*)-**22**.

Table S4. Geometric parameters (Å, º) for (a*S*,1*R*,3*R*,1'*R*,3'*R*)-**22**.

Distances (Å)			
C1—O2	1.424 (15)	C35—H35	0.9500
C1—C11	1.522 (17)	C36—H36	0.9500
C1—C8A	1.533 (17)	C41—O42	1.431 (14)
C1—H1	1.0000	C41—C51	1.500 (17)
C1'—O2'	1.428 (14)	C41—C48E	1.514 (17)
C1'—C10'	1.510 (18)	C41—H41	1.0000
C1'—C8A'	1.512 (17)	C41'—O42'	1.422 (15)
C1'—H1'	1.0000	C41'—C48F	1.525 (16)
C3—O2	1.447 (14)	C41'—C50D	1.530 (18)
C3—C30	1.504 (18)	C41'—H41'	1.0000
C3—C4	1.530 (17)	C43—O42	1.456 (14)
C3—H3	1.0000	C43—C44	1.527 (17)
C3'—O2'	1.457 (17)	C43—H43	1.0000
C3'—C30'	1.503 (19)	C43'—O42'	1.433 (15)
C3'—C4'	1.523 (18)	C43'—C44'	1.526 (16)
C3'—H3'	1.0000	C43'—H43'	1.0000

C4—C4A	1.502 (17)	C44—C44E	1.519 (17)
C4—H4A	0.9900	C44—H44A	0.9900
C4—H4B	0.9900	C44—H44B	0.9900
C4'—C4A'	1.508 (18)	C44'—C44F	1.481 (17)
C4'—H4'A	0.9900	C44'—H44C	0.9900
C4'—H4'B	0.9900	C44'—H44D	0.9900
C5—C6	1.396 (17)	C45—C46	1.390 (17)
C5—C4A	1.401 (17)	C45—C44E	1.405 (17)
C5—C5'	1.533 (17)	C45—C45'	1.506 (16)
C4A—C8A	1.399 (16)	C44E—C48E	1.389 (16)
C5'—C4A'	1.365 (18)	C44F—C45'	1.401 (16)
C5'—C6'	1.398 (18)	C44F—C48F	1.421 (16)
C6—O6	1.380 (15)	C45'—C46'	1.391 (17)
C6—C7	1.389 (17)	C46—C47	1.376 (17)
C6'—C7'	1.400 (16)	C46—O46	1.385 (14)
C6'—H6'	0.9500	C46'—C47'	1.367 (17)
C7—O7	1.379 (15)	C46'—H46'	0.9500
C7—C8	1.386 (18)	C47—O47	1.368 (15)
C7'—O7'	1.375 (15)	C47—C48	1.418 (17)
C7'—C8'	1.385 (17)	C47'—O47'	1.387 (15)
C8—C8A	1.368 (17)	C47'—C48'	1.402 (17)
C8—O8	1.418 (15)	C48—C48E	1.383 (17)
C8'—C8A'	1.368 (18)	C48—O48	1.386 (14)
C8'—O8'	1.404 (14)	C48'—C48F	1.387 (16)
C10'—O10'	1.198 (15)	C48'—O48'	1.405 (14)
C10'—O11'	1.336 (15)	C51—C56	1.387 (18)
C11—C12	1.372 (18)	C51—C52	1.394 (17)
C11—C16	1.416 (17)	C50D—O50D	1.201 (15)
C11'—O11'	1.463 (16)	C50D—O51D	1.334 (15)
C11'—C12'	1.53 (2)	C52—C53	1.413 (18)
C11'—H11A	0.9900	C52—H52	0.9500
C11'—H11B	0.9900	C51D—C52D	1.47 (2)
C12—C13	1.384 (18)	C51D—O51D	1.466 (16)
C12—H12	0.9500	C51D—H51A	0.9900
C12'—H12A	0.9800	C51D—H51B	0.9900

C12'—H12B	0.9800	C53—C54	1.347 (19)
C12'—H12C	0.9800	C53—H53	0.9500
C13—C14	1.380 (19)	C52D—H52A	0.9800
C13—H13	0.9500	C52D—H52B	0.9800
C14—C15	1.355 (19)	C52D—H52C	0.9800
C14—F14	1.366 (15)	C54—F54	1.370 (16)
C15—C16	1.375 (18)	C54—C55	1.386 (19)
C15—H15	0.9500	C55—C56	1.396 (19)
C16—H16	0.9500	C55—H55	0.9500
C21—O22	1.443 (15)	C56—H56	0.9500
C21—C28B	1.512 (17)	C60—O6	1.441 (17)
C21—C31	1.517 (17)	C60—H60A	0.9800
C21—H21	1.0000	C60—H60B	0.9800
C21'—O22'	1.420 (14)	C60—H60C	0.9800
C21'—C28C	1.492 (16)	C60B—O26	1.433 (15)
C21'—C30D	1.517 (18)	C60B—H60D	0.9800
C21'—H21'	1.0000	C60B—H60E	0.9800
C23—O22	1.445 (14)	C60B—H60F	0.9800
C23—C30B	1.508 (18)	C60C—O46	1.430 (14)
C23—C24	1.523 (16)	C60C—H60G	0.9800
C23—H23	1.0000	C60C—H60H	0.9800
C23'—O22'	1.436 (14)	C60C—H60I	0.9800
C23'—C24'	1.483 (17)	C70—O7	1.433 (17)
C23'—C30C	1.509 (17)	C70—H70A	0.9800
C23'—H23'	1.0000	C70—H70B	0.9800
C24—C24B	1.496 (17)	C70—H70C	0.9800
C24—H24A	0.9900	C70'—O7'	1.432 (15)
C24—H24B	0.9900	C70'—H70D	0.9800
C24'—C24C	1.505 (16)	C70'—H70E	0.9800
C24'—H24C	0.9900	C70'—H70F	0.9800
C24'—H24D	0.9900	C70B—O27	1.424 (16)
C25—C26	1.394 (17)	C70B—H70G	0.9800
C25—C24B	1.409 (16)	C70B—H70H	0.9800
C25—C25'	1.525 (16)	C70B—H70I	0.9800
C24B—C28B	1.387 (16)	C70C—O27'	1.437 (15)

C24C—C25'	1.400 (16)	C70C—H70J	0.9800
C24C—C28C	1.412 (16)	C70C—H70K	0.9800
C25'—C26'	1.391 (17)	C70C—H70L	0.9800
C26—O26	1.383 (14)	C70E—O47	1.425 (15)
C26—C27	1.397 (17)	C70E—H70M	0.9800
C26'—C27'	1.396 (17)	C70E—H70N	0.9800
C26'—H26'	0.9500	C70E—H70O	0.9800
C27—C28	1.380 (17)	C70F—O47'	1.439 (15)
C27—O27	1.383 (15)	C70F—H70P	0.9800
C27'—C28'	1.383 (17)	C70F—H70Q	0.9800
C27'—O27'	1.384 (14)	C70F—H70R	0.9800
C28—O28	1.385 (14)	C80—O8	1.422 (16)
C28—C28B	1.402 (17)	C80—H80A	0.9800
C28'—C28C	1.396 (17)	C80—H80B	0.9800
C28'—O28'	1.399 (14)	C80—H80C	0.9800
C30—H30A	0.9800	C80'—O8'	1.440 (15)
C30—H30B	0.9800	C80'—H80D	0.9800
C30—H30C	0.9800	C80'—H80E	0.9800
C30'—H30D	0.9800	C80'—H80F	0.9800
C30'—H30E	0.9800	C81—O81	1.422 (18)
C30'—H30F	0.9800	C81—C82	1.53 (2)
C31—C36	1.374 (17)	C81—H81A	0.9900
C31—C32	1.418 (17)	C81—H81B	0.9900
C30B—H30G	0.9800	C80B—O28	1.442 (16)
C30B—H30H	0.9800	C80B—H80G	0.9800
C30B—H30I	0.9800	C80B—H80H	0.9800
C30C—H30J	0.9800	C80B—H80I	0.9800
C30C—H30K	0.9800	C80C—O28'	1.425 (15)
C30C—H30L	0.9800	C80C—H80J	0.9800
C30D—O30D	1.222 (16)	C80C—H80K	0.9800
C30D—O31D	1.340 (15)	C80C—H80L	0.9800
C30E—C43	1.483 (18)	C80E—O48	1.442 (15)
C30E—H30M	0.9800	C80E—H80M	0.9800
C30E—H30N	0.9800	C80E—H80N	0.9800
C30E—H30O	0.9800	C80E—H80O	0.9800

C30F—C43'	1.513 (17)	C80F—O48'	1.425 (15)
C30F—H30P	0.9800	C80F—H80P	0.9800
C30F—H30Q	0.9800	C80F—H80Q	0.9800
C30F—H30R	0.9800	C80F—H80R	0.9800
C32—C33	1.385 (18)	C82—H82A	0.9800
C32—H32	0.9500	C82—H82B	0.9800
C31D—O31D	1.471 (16)	C82—H82C	0.9800
C31D—C32D	1.51 (2)	C91—C92	1.32 (3)
C31D—H31A	0.9900	C91—O91	1.34 (2)
C31D—H31B	0.9900	C91—H91A	0.9900
C33—C34	1.368 (18)	C91—H91B	0.9900
C33—H33	0.9500	C92—H92A	0.9800
C32D—H32A	0.9800	C92—H92B	0.9800
C32D—H32B	0.9800	C92—H92C	0.9800
C32D—H32C	0.9800	C4A'—C8A'	1.416 (17)
C34—F34	1.372 (15)	O81—H81	0.8500 (15)
C34—C35	1.395 (18)	O91—H91	0.8400
C35—C36	1.369 (17)		
Angles (º)			
O2—C1—C11	111.3 (10)	O42'—C41'—C48F	112.9 (10)
O2—C1—C8A	112.7 (10)	O42'—C41'—C50D	104.4 (10)
C11—C1—C8A	113.1 (11)	C48F—C41'—C50D	110.0 (10)
O2—C1—H1	106.4000	O42'—C41'—H41'	109.8000
C11—C1—H1	106.4000	C48F—C41'—H41'	109.8000
C8A—C1—H1	106.4000	C50D—C41'—H41'	109.8000
O2'—C1'—C10'	102.8 (10)	O42—C43—C30E	106.5 (10)
O2'—C1'—C8A'	113.6 (10)	O42—C43—C44	108.8 (10)
C10'—C1'—C8A'	112.3 (11)	C30E—C43—C44	112.5 (11)
O2'—C1'—H1'	109.3000	O42—C43—H43	109.7000
C10'—C1'—H1'	109.3000	C30E—C43—H43	109.7000
C8A'—C1'—H1'	109.3000	C44—C43—H43	109.7000
O2—C3—C30	105.7 (10)	O42'—C43'—C30F	106.0 (10)
O2—C3—C4	110.0 (10)	O42'—C43'—C44'	109.1 (10)
C30—C3—C4	112.4 (11)	C30F—C43'—C44'	111.5 (10)
O2—C3—H3	109.6000	O42'—C43'—H43'	110.0000

C30—C3—H3	109.6000	C30F—C43'—H43'	110.0000
C4—C3—H3	109.6000	C44'—C43'—H43'	110.0000
O2'—C3'—C30'	105.7 (12)	C44E—C44—C43	113.6 (10)
O2'—C3'—C4'	108.7 (11)	C44E—C44—H44A	108.8000
C30'—C3'—C4'	114.2 (12)	C43—C44—H44A	108.8000
O2'—C3'—H3'	109.4000	C44E—C44—H44B	108.8000
C30'—C3'—H3'	109.4000	C43—C44—H44B	108.8000
C4'—C3'—H3'	109.4000	H44A—C44—H44B	107.7000
C4A—C4—C3	113.1 (10)	C44F—C44'—C43'	110.8 (10)
C4A—C4—H4A	109.0000	C44F—C44'—H44C	109.5000
C3—C4—H4A	109.0000	C43'—C44'—H44C	109.5000
C4A—C4—H4B	109.0000	C44F—C44'—H44D	109.5000
C3—C4—H4B	109.0000	C43'—C44'—H44D	109.5000
H4A—C4—H4B	107.8000	H44C—C44'—H44D	108.1000
C4A'—C4'—C3'	111.7 (11)	C46—C45—C44E	119.4 (11)
C4A'—C4'—H4'A	109.3000	C46—C45—C45'	120.5 (11)
C3'—C4'—H4'A	109.3000	C44E—C45—C45'	120.1 (11)
C4A'—C4'—H4'B	109.3000	C48E—C44E—C45	119.6 (11)
C3'—C4'—H4'B	109.3000	C48E—C44E—C44	119.8 (11)
H4'A—C4'—H4'B	107.9000	C45—C44E—C44	120.6 (11)
C6—C5—C4A	120.3 (11)	C45'—C44F—C48F	118.2 (11)
C6—C5—C5'	120.0 (11)	C45'—C44F—C44'	122.2 (11)
C4A—C5—C5'	119.5 (11)	C48F—C44F—C44'	119.7 (10)
C8A—C4A—C5	119.1 (11)	C46'—C45'—C44F	120.8 (11)
C8A—C4A—C4	120.4 (11)	C46'—C45'—C45	120.1 (10)
C5—C4A—C4	120.5 (11)	C44F—C45'—C45	119.1 (11)
C4A'—C5'—C6'	122.0 (12)	C47—C46—O46	118.6 (11)
C4A'—C5'—C5	120.2 (12)	C47—C46—C45	122.0 (11)
C6'—C5'—C5	117.7 (11)	O46—C46—C45	119.3 (10)
O6—C6—C7	122.7 (11)	C47'—C46'—C45'	120.6 (12)
O6—C6—C5	116.9 (11)	C47'—C46'—H46'	119.7000
C7—C6—C5	120.3 (12)	C45'—C46'—H46'	119.7000
C5'—C6'—C7'	118.8 (12)	O47—C47—C46	122.1 (11)
C5'—C6'—H6'	120.6000	O47—C47—C48	119.9 (11)
C7'—C6'—H6'	120.6000	C46—C47—C48	118.0 (11)

O7—C7—C8	119.8 (11)	C46'—C47'—O47'	125.1 (11)
O7—C7—C6	122.2 (12)	C46'—C47'—C48'	120.2 (12)
C8—C7—C6	118.0 (12)	O47'—C47'—C48'	114.7 (10)
O7'—C7'—C8'	116.7 (10)	C48E—C48—O48	117.4 (11)
O7'—C7'—C6'	124.1 (11)	C48E—C48—C47	120.8 (11)
C8'—C7'—C6'	119.2 (12)	O48—C48—C47	121.6 (11)
C8A—C8—C7	123.1 (12)	C48F—C48'—C47'	120.1 (11)
C8A—C8—O8	117.6 (11)	C48F—C48'—O48'	117.3 (10)
C7—C8—O8	119.0 (11)	C47'—C48'—O48'	122.5 (11)
C8A'—C8'—C7'	121.5 (11)	C48—C48E—C44E	120.2 (11)
C8A'—C8'—O8'	117.6 (11)	C48—C48E—C41	118.4 (11)
C7'—C8'—O8'	120.7 (11)	C44E—C48E—C41	121.3 (11)
C8—C8A—C4A	118.9 (11)	C48'—C48F—C44F	120.1 (11)
C8—C8A—C1	121.2 (11)	C48'—C48F—C41'	119.2 (10)
C4A—C8A—C1	119.7 (11)	C44F—C48F—C41'	120.6 (10)
O10'—C10'—O11'	124.0 (13)	C56—C51—C52	117.2 (12)
O10'—C10'—C1'	125.1 (12)	C56—C51—C41	123.7 (11)
O11'—C10'—C1'	110.8 (11)	C52—C51—C41	119.1 (11)
C12—C11—C16	116.9 (13)	O50D—C50D—O51D	124.5 (12)
C12—C11—C1	124.9 (12)	O50D—C50D—C41'	123.5 (12)
C16—C11—C1	118.0 (12)	O51D—C50D—C41'	111.9 (11)
O11'—C11'—C12'	110.1 (12)	C51—C52—C53	122.4 (13)
O11'—C11'—H11A	109.6000	C51—C52—H52	118.8000
C12'—C11'—H11A	109.6000	C53—C52—H52	118.8000
O11'—C11'—H11B	109.6000	C52D—C51D—O51D	107.4 (13)
C12'—C11'—H11B	109.6000	C52D—C51D—H51A	110.2000
H11A—C11'—H11B	108.2000	O51D—C51D—H51A	110.2000
C11—C12—C13	122.5 (13)	C52D—C51D—H51B	110.2000
C11—C12—H12	118.8000	O51D—C51D—H51B	110.2000
C13—C12—H12	118.8000	H51A—C51D—H51B	108.5000
C11'—C12'—H12A	109.5000	C54—C53—C52	116.9 (13)
C11'—C12'—H12B	109.5000	C54—C53—H53	121.6000
H12A—C12'—H12B	109.5000	C52—C53—H53	121.6000
C11'—C12'—H12C	109.5000	C51D—C52D—H52A	109.5000
H12A—C12'—H12C	109.5000	C51D—C52D—H52B	109.5000

H12B—C12'—H12C	109.5000	H52A—C52D—H52B	109.5000
C14—C13—C12	118.0 (13)	C51D—C52D—H52C	109.5000
C14—C13—H13	121.0000	H52A—C52D—H52C	109.5000
C12—C13—H13	121.0000	H52B—C52D—H52C	109.5000
C15—C14—F14	119.6 (12)	C53—C54—F54	118.1 (13)
C15—C14—C13	122.3 (14)	C53—C54—C55	124.1 (13)
F14—C14—C13	118.2 (13)	F54—C54—C55	117.8 (13)
C14—C15—C16	118.9 (13)	C54—C55—C56	117.3 (13)
C14—C15—H15	120.6000	C54—C55—H55	121.3000
C16—C15—H15	120.6000	C56—C55—H55	121.3000
C15—C16—C11	121.5 (13)	C51—C56—C55	122.1 (13)
C15—C16—H16	119.3000	C51—C56—H56	119.0000
C11—C16—H16	119.3000	C55—C56—H56	119.0000
O22—C21—C28B	110.5 (9)	O6—C60—H60A	109.5000
O22—C21—C31	110.7 (10)	O6—C60—H60B	109.5000
C28B—C21—C31	114.0 (10)	H60A—C60—H60B	109.5000
O22—C21—H21	107.1000	O6—C60—H60C	109.5000
C28B—C21—H21	107.1000	H60A—C60—H60C	109.5000
C31—C21—H21	107.1000	H60B—C60—H60C	109.5000
O22'—C21'—C28C	113.7 (10)	O26—C60B—H60D	109.5000
O22'—C21'—C30D	102.7 (10)	O26—C60B—H60E	109.5000
C28C—C21'—C30D	112.7 (10)	H60D—C60B—H60E	109.5000
O22'—C21'—H21'	109.2000	O26—C60B—H60F	109.5000
C28C—C21'—H21'	109.2000	H60D—C60B—H60F	109.5000
C30D—C21'—H21'	109.2000	H60E—C60B—H60F	109.5000
O22—C23—C30B	106.5 (10)	O46—C60C—H60G	109.5000
O22—C23—C24	108.3 (10)	O46—C60C—H60H	109.5000
C30B—C23—C24	113.0 (11)	H60G—C60C—H60H	109.5000
O22—C23—H23	109.7000	O46—C60C—H60I	109.5000
C30B—C23—H23	109.7000	H60G—C60C—H60I	109.5000
C24—C23—H23	109.7000	H60H—C60C—H60I	109.5000
O22'—C23'—C24'	109.6 (10)	O7—C70—H70A	109.5000
O22'—C23'—C30C	107.3 (10)	O7—C70—H70B	109.5000
C24'—C23'—C30C	113.2 (11)	H70A—C70—H70B	109.5000
O22'—C23'—H23'	108.9000	O7—C70—H70C	109.5000

C24'—C23'—H23'	108.9000	H70A—C70—H70C	109.5000
C30C—C23'—H23'	108.9000	H70B—C70—H70C	109.5000
C24B—C24—C23	112.5 (10)	O7'—C70'—H70D	109.5000
C24B—C24—H24A	109.1000	O7'—C70'—H70E	109.5000
C23—C24—H24A	109.1000	H70D—C70'—H70E	109.5000
C24B—C24—H24B	109.1000	O7'—C70'—H70F	109.5000
C23—C24—H24B	109.1000	H70D—C70'—H70F	109.5000
H24A—C24—H24B	107.8000	H70E—C70'—H70F	109.5000
C23'—C24'—C24C	113.6 (11)	O27—C70B—H70G	109.5000
C23'—C24'—H24C	108.8000	O27—C70B—H70H	109.5000
C24C—C24'—H24C	108.8000	H70G—C70B—H70H	109.5000
C23'—C24'—H24D	108.8000	O27—C70B—H70I	109.5000
C24C—C24'—H24D	108.8000	H70G—C70B—H70I	109.5000
H24C—C24'—H24D	107.7000	H70H—C70B—H70I	109.5000
C26—C25—C24B	119.6 (11)	O27'—C70C—H70J	109.5000
C26—C25—C25'	118.8 (10)	O27'—C70C—H70K	109.5000
C24B—C25—C25'	121.2 (11)	H70J—C70C—H70K	109.5000
C28B—C24B—C25	119.5 (11)	O27'—C70C—H70L	109.5000
C28B—C24B—C24	120.1 (11)	H70J—C70C—H70L	109.5000
C25—C24B—C24	120.4 (11)	H70K—C70C—H70L	109.5000
C25'—C24C—C28C	118.7 (11)	O47—C70E—H70M	109.5000
C25'—C24C—C24'	122.6 (11)	O47—C70E—H70N	109.5000
C28C—C24C—C24'	118.6 (11)	H70M—C70E—H70N	109.5000
C26'—C25'—C24C	121.6 (11)	O47—C70E—H70O	109.5000
C26'—C25'—C25	120.5 (11)	H70M—C70E—H70O	109.5000
C24C—C25'—C25	117.8 (11)	H70N—C70E—H70O	109.5000
O26—C26—C25	119.0 (10)	O47'—C70F—H70P	109.5000
O26—C26—C27	120.2 (11)	O47'—C70F—H70Q	109.5000
C25—C26—C27	120.7 (11)	H70P—C70F—H70Q	109.5000
C25'—C26'—C27'	119.0 (12)	O47'—C70F—H70R	109.5000
C25'—C26'—H26'	120.5000	H70P—C70F—H70R	109.5000
C27'—C26'—H26'	120.5000	H70Q—C70F—H70R	109.5000
C28—C27—O27	120.9 (11)	O8—C80—H80A	109.5000
C28—C27—C26	119.2 (11)	O8—C80—H80B	109.5000
O27—C27—C26	119.7 (11)	H80A—C80—H80B	109.5000

C28'—C27'—O27'	116.8 (11)	O8—C80—H80C	109.5000
C28'—C27'—C26'	120.0 (11)	H80A—C80—H80C	109.5000
O27'—C27'—C26'	123.2 (11)	H80B—C80—H80C	109.5000
C27—C28—O28	122.6 (11)	O8'—C80'—H80D	109.5000
C27—C28—C28B	120.9 (11)	O8'—C80'—H80E	109.5000
O28—C28—C28B	116.5 (11)	H80D—C80'—H80E	109.5000
C27'—C28'—C28C	121.3 (11)	O8'—C80'—H80F	109.5000
C27'—C28'—O28'	121.8 (11)	H80D—C80'—H80F	109.5000
C28C—C28'—O28'	116.5 (11)	H80E—C80'—H80F	109.5000
C24B—C28B—C28	120.0 (11)	O81—C81—C82	107.7 (15)
C24B—C28B—C21	121.4 (11)	O81—C81—H81A	110.2000
C28—C28B—C21	118.4 (11)	C82—C81—H81A	110.2000
C28'—C28C—C24C	119.1 (11)	O81—C81—H81B	110.2000
C28'—C28C—C21'	120.7 (11)	C82—C81—H81B	110.2000
C24C—C28C—C21'	120.2 (11)	H81A—C81—H81B	108.5000
C3—C30—H30A	109.5000	O28—C80B—H80G	109.5000
C3—C30—H30B	109.5000	O28—C80B—H80H	109.5000
H30A—C30—H30B	109.5000	H80G—C80B—H80H	109.5000
C3—C30—H30C	109.5000	O28—C80B—H80I	109.5000
H30A—C30—H30C	109.5000	H80G—C80B—H80I	109.5000
H30B—C30—H30C	109.5000	H80H—C80B—H80I	109.5000
C3'—C30'—H30D	109.5000	O28'—C80C—H80J	109.5000
C3'—C30'—H30E	109.5000	O28'—C80C—H80K	109.5000
H30D—C30'—H30E	109.5000	H80J—C80C—H80K	109.5000
C3'—C30'—H30F	109.5000	O28'—C80C—H80L	109.5000
H30D—C30'—H30F	109.5000	H80J—C80C—H80L	109.5000
H30E—C30'—H30F	109.5000	H80K—C80C—H80L	109.5000
C36—C31—C32	118.4 (12)	O48—C80E—H80M	109.5000
C36—C31—C21	125.2 (11)	O48—C80E—H80N	109.5000
C32—C31—C21	116.0 (11)	H80M—C80E—H80N	109.5000
C23—C30B—H30G	109.5000	O48—C80E—H80O	109.5000
C23—C30B—H30H	109.5000	H80M—C80E—H80O	109.5000
H30G—C30B—H30H	109.5000	H80N—C80E—H80O	109.5000
C23—C30B—H30I	109.5000	O48'—C80F—H80P	109.5000
H30G—C30B—H30I	109.5000	O48'—C80F—H80Q	109.5000

H30H—C30B—H30I	109.5000	H80P—C80F—H80Q	109.5000
C23'—C30C—H30J	109.5000	O48'—C80F—H80R	109.5000
C23'—C30C—H30K	109.5000	H80P—C80F—H80R	109.5000
H30J—C30C—H30K	109.5000	H80Q—C80F—H80R	109.5000
C23'—C30C—H30L	109.5000	C81—C82—H82A	109.5000
H30J—C30C—H30L	109.5000	C81—C82—H82B	109.5000
H30K—C30C—H30L	109.5000	H82A—C82—H82B	109.5000
O30D—C30D—O31D	123.5 (12)	C81—C82—H82C	109.5000
O30D—C30D—C21'	123.8 (12)	H82A—C82—H82C	109.5000
O31D—C30D—C21'	112.7 (11)	H82B—C82—H82C	109.5000
C43—C30E—H30M	109.5000	C92—C91—O91	127 (3)
C43—C30E—H30N	109.5000	C92—C91—H91A	105.5000
H30M—C30E—H30N	109.5000	O91—C91—H91A	105.5000
C43—C30E—H30O	109.5000	C92—C91—H91B	105.5000
H30M—C30E—H30O	109.5000	O91—C91—H91B	105.5000
H30N—C30E—H30O	109.5000	H91A—C91—H91B	106.1000
C43'—C30F—H30P	109.5000	C91—C92—H92A	109.5000
C43'—C30F—H30Q	109.5000	C91—C92—H92B	109.5000
H30P—C30F—H30Q	109.5000	H92A—C92—H92B	109.5000
C43'—C30F—H30R	109.5000	C91—C92—H92C	109.4000
H30P—C30F—H30R	109.5000	H92A—C92—H92C	109.5000
H30Q—C30F—H30R	109.5000	H92B—C92—H92C	109.5000
C33—C32—C31	120.6 (12)	C5'—C4A'—C8A'	118.5 (12)
C33—C32—H32	119.7000	C5'—C4A'—C4'	121.1 (12)
C31—C32—H32	119.7000	C8A'—C4A'—C4'	120.3 (12)
O31D—C31D—C32D	107.4 (12)	C8'—C8A'—C4A'	119.9 (11)
O31D—C31D—H31A	110.2000	C8'—C8A'—C1'	120.3 (11)
C32D—C31D—H31A	110.2000	C4A'—C8A'—C1'	119.9 (11)
O31D—C31D—H31B	110.2000	C1—O2—C3	111.8 (9)
C32D—C31D—H31B	110.2000	C1'—O2'—C3'	112.6 (10)
H31A—C31D—H31B	108.5000	C6—O6—C60	118.3 (11)
C34—C33—C32	117.8 (12)	C7—O7—C70	112.7 (11)
C34—C33—H33	121.1000	C7'—O7'—C70'	118.2 (10)
C32—C33—H33	121.1000	C8—O8—C80	111.3 (11)
C31D—C32D—H32A	109.5000	C8'—O8'—C80'	114.1 (9)

C31D—C32D—H32B	109.5000	C10'—O11'—C11'	117.5 (10)
H32A—C32D—H32B	109.5000	C21—O22—C23	111.1 (9)
C31D—C32D—H32C	109.5000	C21'—O22'—C23'	112.1 (9)
H32A—C32D—H32C	109.5000	C26—O26—C60B	112.9 (9)
H32B—C32D—H32C	109.5000	C27—O27—C70B	115.1 (10)
C33—C34—F34	119.2 (11)	C27'—O27'—C70C	117.7 (10)
C33—C34—C35	123.4 (13)	C28—O28—C80B	116.1 (10)
F34—C34—C35	117.5 (12)	C28'—O28'—C80C	115.7 (10)
C36—C35—C34	117.3 (13)	C30D—O31D—C31D	115.5 (11)
C36—C35—H35	121.3000	C41—O42—C43	113.6 (9)
C34—C35—H35	121.3000	C41'—O42'—C43'	113.1 (9)
C35—C36—C31	122.4 (12)	C46—O46—C60C	112.2 (9)
C35—C36—H36	118.8000	C47—O47—C70E	111.4 (10)
C31—C36—H36	118.8000	C47'—O47'—C70F	115.2 (9)
O42—C41—C51	111.2 (10)	C48—O48—C80E	115.7 (10)
O42—C41—C48E	111.7 (10)	C48'—O48'—C80F	113.8 (10)
C51—C41—C48E	113.8 (10)	C50D—O51D—C51D	114.3 (10)
O42—C41—H41	106.5000	C81—O81—H81	130 (6)
C51—C41—H41	106.5000	C91—O91—H91	109.5000
C48E—C41—H41	106.5000		
Torsion angles (º)			
O2—C3—C4—C4A	−44.9 (14)	C43—C44—C44E—C45	−169.2 (11)
C30—C3—C4—C4A	−162.3 (11)	C43'—C44'—C44F—C45'	−159.9 (11)
O2'—C3'—C4'—C4A'	−50.2 (15)	C43'—C44'—C44F—C48F	19.4 (15)
C30'—C3'—C4'—C4A'	−167.9 (14)	C48F—C44F—C45'—C46'	−2.4 (17)
C6—C5—C4A—C8A	2.9 (19)	C44'—C44F—C45'—C46'	176.9 (11)
C5'—C5—C4A—C8A	178.7 (12)	C48F—C44F—C45'—C45	176.8 (10)
C6—C5—C4A—C4	−174.2 (12)	C44'—C44F—C45'—C45	−4.0 (17)
C5'—C5—C4A—C4	1.6 (19)	C46—C45—C45'—C46'	−98.3 (14)
C3—C4—C4A—C8A	15.0 (18)	C44E—C45—C45'—C46'	82.5 (15)
C3—C4—C4A—C5	−167.9 (12)	C46—C45—C45'—C44F	82.5 (15)
C6—C5—C5'—C4A'	80.7 (16)	C44E—C45—C45'—	−96.6 (14)

		C44F	
C4A—C5—C5'—C4A'	−95.1 (15)	C44E—C45—C46—C47	1.0 (18)
C6—C5—C5'—C6'	−102.9 (14)	C45'—C45—C46—C47	−178.2 (11)
C4A—C5—C5'—C6'	81.3 (15)	C44E—C45—C46—O46	176.3 (11)
C4A—C5—C6—O6	176.5 (12)	C45'—C45—C46—O46	−2.8 (17)
C5'—C5—C6—O6	0.8 (18)	C44F—C45'—C46'—C47'	0.4 (18)
C4A—C5—C6—C7	1 (2)	C45—C45'—C46'—C47'	−178.8 (11)
C5'—C5—C6—C7	−175.0 (12)	O46—C46—C47—O47	4.4 (18)
C4A'—C5'—C6'—C7'	−1.2 (19)	C45—C46—C47—O47	179.8 (11)
C5—C5'—C6'—C7'	−177.5 (11)	O46—C46—C47—C48	−178.9 (10)
O6—C6—C7—O7	2 (2)	C45—C46—C47—C48	-3.5 (18)
C5—C6—C7—O7	177.4 (13)	C45'—C46'—C47'—O47'	−179.7 (11)
O6—C6—C7—C8	−178.3 (13)	C45'—C46'—C47'—C48'	1.1 (18)
C5—C6—C7—C8	−3 (2)	O47—C47—C48—C48E	−179.6 (11)
C5'—C6'—C7'—O7'	178.2 (11)	C46—C47—C48—C48E	3.6 (18)
C5'—C6'—C7'—C8'	0.4 (17)	O47—C47—C48—O48	−5.2 (18)
O7—C7—C8—C8A	−179.0 (13)	C46—C47—C48—O48	178.1 (11)
C6—C7—C8—C8A	1 (2)	C46'—C47'—C48'—C48F	−0.5 (18)
O7—C7—C8—O8	−5 (2)	O47'—C47'—C48'—C48F	−179.7 (11)
C6—C7—C8—O8	174.7 (12)	C46'—C47'—C48'—O48'	174.8 (11)
O7'—C7'—C8'—C8A'	−175.9 (11)	O47'—C47'—C48'—O48'	−4.4 (17)
C6'—C7'—C8'—C8A'	2.0 (18)	O48—C48—C48E—C44E	−175.9 (11)
O7'—C7'—C8'—O8'	−0.4 (17)	C47—C48—C48E—C44E	−1.3 (19)
C6'—C7'—C8'—O8'	177.5 (11)	O48—C48—C48E—C41	0.9 (17)
C7—C8—C8A—C4A	3 (2)	C47—C48—C48E—C41	175.6 (11)
O8—C8—C8A—C4A	−171.2 (11)	C45—C44E—C48E—C48	−1.3 (18)
C7—C8—C8A—C1	178.3 (12)	C44—C44E—C48E—C48	175.7 (12)
O8—C8—C8A—C1	4.7 (19)	C45—C44E—C48E—C41	−178.0 (11)
C5—C4A—C8A—C8	−4.5 (19)	C44—C44E—C48E—C41	−1.1 (18)

C4—C4A—C8A—C8	172.6 (12)	O42—C41—C48E—C48	−157.7 (11)
C5—C4A—C8A—C1	179.6 (12)	C51—C41—C48E—C48	75.3 (15)
C4—C4A—C8A—C1	−3.3 (18)	O42—C41—C48E—C44E	19.1 (16)
O2—C1—C8A—C8	−154.2 (12)	C51—C41—C48E—C44E	−107.9 (13)
C11—C1—C8A—C8	78.5 (15)	C47'—C48'—C48F—C44F	−1.6 (17)
O2—C1—C8A—C4A	21.6 (16)	O48'—C48'—C48F—C44F	−177.1 (10)
C11—C1—C8A—C4A	−105.7 (13)	C47'—C48'—C48F—C41'	178.6 (11)
O2'—C1'—C10'—O10'	79.2 (16)	O48'—C48'—C48F—C41'	3.1 (16)
C8A'—C1'—C10'—O10'	−43.3 (19)	C45'—C44F—C48F—C48'	3.0 (17)
O2'—C1'—C10'—O11'	−96.3 (12)	C44'—C44F—C48F—C48'	−176.3 (11)
C8A'—C1'—C10'—O11'	141.2 (11)	C45'—C44F—C48F—C41'	−177.2 (11)
O2—C1—C11—C12	−96.6 (14)	C44'—C44F—C48F—C41'	3.5 (16)
C8A—C1—C11—C12	31.5 (17)	O42'—C41'—C48F—C48'	−172.9 (11)
O2—C1—C11—C16	77.5 (14)	C50D—C41'—C48F—C48'	−56.8 (14)
C8A—C1—C11—C16	−154.4 (11)	O42'—C41'—C48F—C44F	7.4 (16)
C16—C11—C12—C13	−1.5 (19)	C50D—C41'—C48F—C44F	123.5 (12)
C1—C11—C12—C13	172.8 (12)	O42—C41—C51—C56	−99.7 (14)
C11—C12—C13—C14	0 (2)	C48E—C41—C51—C56	27.6 (18)
C12—C13—C14—C15	1 (2)	O42—C41—C51—C52	78.4 (14)
C12—C13—C14—F14	179.5 (11)	C48E—C41—C51—C52	−154.3 (12)
F14—C14—C15—C16	−179.2 (12)	O42'—C41'—C50D—O50D	68.3 (15)
C13—C14—C15—C16	−1 (2)	C48F—C41'—C50D—O50D	−53.1 (16)
C14—C15—C16—C11	−0.5 (19)	O42'—C41'—C50D—O51D	−109.6 (11)

C12—C11—C16—C15	1.7 (19)	C48F—C41'—C50D—O51D	129.1 (11)
C1—C11—C16—C15	−172.9 (12)	C56—C51—C52—C53	2 (2)
O22—C23—C24—C24B	−46.4 (14)	C41—C51—C52—C53	−175.9 (12)
C30B—C23—C24—C24B	−164.2 (11)	C51—C52—C53—C54	-2 (2)
O22'—C23'—C24'—C24C	−47.8 (14)	C52—C53—C54—F54	178.6 (12)
C30C—C23'—C24'—C24C	−167.5 (11)	C52—C53—C54—C55	0 (2)
C26—C25—C24B—C28B	2.0 (18)	C53—C54—C55—C56	1 (2)
C25'—C25—C24B—C28B	174.9 (11)	F54—C54—C55—C56	−177.2 (13)
C26—C25—C24B—C24	−178.9 (12)	C52—C51—C56—C55	−1 (2)
C25'—C25—C24B—C24	−6.0 (18)	C41—C51—C56—C55	177.3 (13)
C23—C24—C24B—C28B	11.0 (17)	C54—C55—C56—C51	−1 (2)
C23—C24—C24B—C25	−168.1 (11)	C6'—C5'—C4A'—C8A'	−0.4 (19)
C23'—C24'—C24C—C25'	−161.9 (11)	C5—C5'—C4A'—C8A'	175.8 (11)
C23'—C24'—C24C—C28C	15.8 (16)	C6'—C5'—C4A'—C4'	−179.0 (12)
C28C—C24C—C25'—C26'	−0.2 (18)	C5—C5'—C4A'—C4'	−2.8 (19)
C24'—C24C—C25'—C26'	177.5 (12)	C3'—C4'—C4A'—C5'	−162.6 (13)
C28C—C24C—C25'—C25	176.8 (11)	C3'—C4'—C4A'—C8A'	18.8 (18)
C24'—C24C—C25'—C25	−5.5 (17)	C7'—C8'—C8A'—C4A'	−3.6 (19)
C26—C25—C25'—C26'	−102.5 (15)	O8'—C8'—C8A'—C4A'	−179.3 (11)
C24B—C25—C25'—C26'	84.5 (16)	C7'—C8'—C8A'—C1'	178.4 (11)
C26—C25—C25'—C24C	80.4 (15)	O8'—C8'—C8A'—C1'	2.7 (18)
C24B—C25—C25'—C24C	−92.5 (15)	C5'—C4A'—C8A'—C8'	2.8 (18)
C24B—C25—C26—O26	174.4 (11)	C4'—C4A'—C8A'—C8'	−178.6 (13)
C25'—C25—C26—O26	1.3 (17)	C5'—C4A'—C8A'—C1'	−179.2 (12)
C24B—C25—C26—C27	−1.0 (18)	C4'—C4A'—C8A'—C1'	−0.6 (18)
C25'—C25—C26—C27	−174.1 (11)	O2'—C1'—C8A'—C8'	−167.5 (11)
C24C—C25'—C26'—C27'	-2.2 (19)	C10'—C1'—C8A'—C8'	−51.4 (16)
C25—C25'—C26'—C27'	−179.1 (11)	O2'—C1'—C8A'—C4A'	14.4 (16)
O26—C26—C27—C28	−175.5 (11)	C10'—C1'—C8A'—C4A'	130.6 (12)
C25—C26—C27—C28	−0.1 (19)	C11—C1—O2—C3	75.0 (12)
O26—C26—C27—O27	−0.2 (18)	C8A—C1—O2—C3	−53.4 (13)
C25—C26—C27—O27	175.1 (11)	C30—C3—O2—C1	−172.4 (10)
C25'—C26'—C27'—C28'	4.6 (19)	C4—C3—O2—C1	66.0 (12)
C25'—C26'—C27'—O27'	−176.8 (12)	C10'—C1'—O2'—C3'	−170.1 (10)
O27—C27—C28—O28	2.4 (19)	C8A'—C1'—O2'—C3'	−48.5 (14)

C26—C27—C28—O28	177.6 (11)	C30'—C3'—O2'—C1'	−169.5 (11)
O27—C27—C28—C28B	−174.9 (11)	C4'—C3'—O2'—C1'	67.5 (13)
C26—C27—C28—C28B	0.3 (19)	C7—C6—O6—C60	−45.4 (19)
O27'—C27'—C28'—C28C	176.6 (12)	C5—C6—O6—C60	138.9 (13)
C26'—C27'—C28'—C28C	−4.8 (19)	C8—C7—O7—C70	95.5 (16)
O27'—C27'—C28'—O28'	3.5 (18)	C6—C7—O7—C70	−84.6 (17)
C26'—C27'—C28'—O28'	−177.9 (12)	C8'—C7'—O7'—C70'	161.9 (11)
C25—C24B—C28B—C28	−1.9 (18)	C6'—C7'—O7'—C70'	−15.9 (17)
C24—C24B—C28B—C28	179.0 (12)	C8A—C8—O8—C80	−108.8 (14)
C25—C24B—C28B—C21	−176.6 (11)	C7—C8—O8—C80	77.3 (16)
C24—C24B—C28B—C21	4.3 (18)	C8A'—C8'—O8'—C80'	−112.8 (13)
C27—C28—C28B—C24B	1 (2)	C7'—C8'—O8'—C80'	71.5 (15)
O28—C28—C28B—C24B	−176.7 (10)	O10'—C10'—O11'—C11'	−1 (2)
C27—C28—C28B—C21	175.6 (11)	C1'—C10'—O11'—C11'	174.4 (11)
O28—C28—C28B—C21	−1.9 (17)	C12'—C11'—O11'—C10'	86.7 (15)
O22—C21—C28B—C24B	16.2 (16)	C28B—C21—O22—C23	−54.0 (12)
C31—C21—C28B—C24B	−109.3 (13)	C31—C21—O22—C23	73.3 (11)
O22—C21—C28B—C28	−158.5 (11)	C30B—C23—O22—C21	−167.7 (10)
C31—C21—C28B—C28	76.0 (15)	C24—C23—O22—C21	70.5 (12)
C27'—C28'—C28C—C24C	2.3 (19)	C28C—C21'—O22'—C23'	−49.7 (14)
O28'—C28'—C28C—C24C	175.8 (10)	C30D—C21'—O22'—C23'	−171.8 (10)
C27'—C28'—C28C—C21'	−175.7 (12)	C24'—C23'—O22'—C21'	66.5 (13)
O28'—C28'—C28C—C21'	−2.2 (18)	C30C—C23'—O22'—C21'	−170.2 (10)
C25'—C24C—C28C—C28'	0.2 (17)	C25—C26—O26—C60B	117.1 (13)
C24'—C24C—C28C—C28'	−177.6 (12)	C27—C26—O26—C60B	−67.4 (15)
C25'—C24C—C28C—C21'	178.2 (11)	C28—C27—O27—C70B	82.2 (15)
C24'—C24C—C28C—C21'	0.4 (17)	C26—C27—O27—C70B	−92.9 (14)
O22'—C21'—C28C—C28'	−166.1 (11)	C28'—C27'—O27'—C70C	−171.4 (12)
C30D—C21'—C28C—C28'	−49.7 (16)	C26'—C27'—O27'—C70C	10.1 (18)
O22'—C21'—C28C—C24C	15.9 (16)	C27—C28—O28—C80B	54.1 (17)
C30D—C21'—C28C—C24C	132.3 (12)	C28B—C28—O28—C80B	−128.4 (13)

O22—C21—C31—C36	−110.2 (14)	C27'—C28'—O28'—C80C	−66.2 (15)
C28B—C21—C31—C36	15.2 (18)	C28C—C28'—O28'—C80C	120.4 (12)
O22—C21—C31—C32	62.4 (14)	O30D—C30D—O31D—C31D	0.2 (19)
C28B—C21—C31—C32	−172.2 (11)	C21'—C30D—O31D—C31D	178.3 (11)
O22'—C21'—C30D—O30D	74.4 (15)	C32D—C31D—O31D—C30D	165.1 (12)
C28C—C21'—C30D—O30D	−48.3 (17)	C51—C41—O42—C43	76.1 (12)
O22'—C21'—C30D—O31D	−103.7 (12)	C48E—C41—O42—C43	−52.3 (13)
C28C—C21'—C30D—O31D	133.5 (11)	C30E—C43—O42—C41	−173.1 (10)
C36—C31—C32—C33	−1 (2)	C44—C43—O42—C41	65.4 (12)
C21—C31—C32—C33	−174.1 (12)	C48F—C41'—O42'—C43'	−43.6 (14)
C31—C32—C33—C34	1.4 (19)	C50D—C41'—O42'—C43'	−163.1 (10)
C32—C33—C34—F34	177.2 (11)	C30F—C43'—O42'—C41'	−171.3 (10)
C32—C33—C34—C35	−3 (2)	C44'—C43'—O42'—C41'	68.5 (12)
C33—C34—C35—C36	3 (2)	C47—C46—O46—C60C	−74.9 (14)
F34—C34—C35—C36	−176.6 (12)	C45—C46—O46—C60C	109.6 (12)
C34—C35—C36—C31	−3 (2)	C46—C47—O47—C70E	−91.5 (14)
C32—C31—C36—C35	2 (2)	C48—C47—O47—C70E	91.9 (13)
C21—C31—C36—C35	174.0 (13)	C46'—C47'—O47'—C70F	−1.0 (17)
O42—C43—C44—C44E	−43.6 (14)	C48'—C47'—O47'—C70F	178.2 (11)
C30E—C43—C44—C44E	−161.3 (11)	C48E—C48—O48—C80E	−126.8 (13)
O42'—C43'—C44'—C44F	−53.8 (13)	C47—C48—O48—C80E	58.5 (16)
C30F—C43'—C44'—C44F	−170.6 (10)	C48F—C48'—O48'—C80F	−113.2 (12)
C46—C45—C44E—C48E	1.5 (18)	C47'—C48'—O48'—C80F	71.4 (15)
C45'—C45—C44E—C48E	−179.4 (11)	O50D—C50D—O51D—C51D	4.5 (19)

C46—C45—C44E—C44	−175.5 (11)	C41'—C50D—O51D—C51D	−177.7 (11)
C45'—C45—C44E—C44	3.7 (18)	C52D—C51D—O51D—C50D	−174.2 (13)
C43—C44—C44E—C48E	13.8 (17)		

5. Structures, populations and Cartesian coordinates of the low-energy DFT conformers

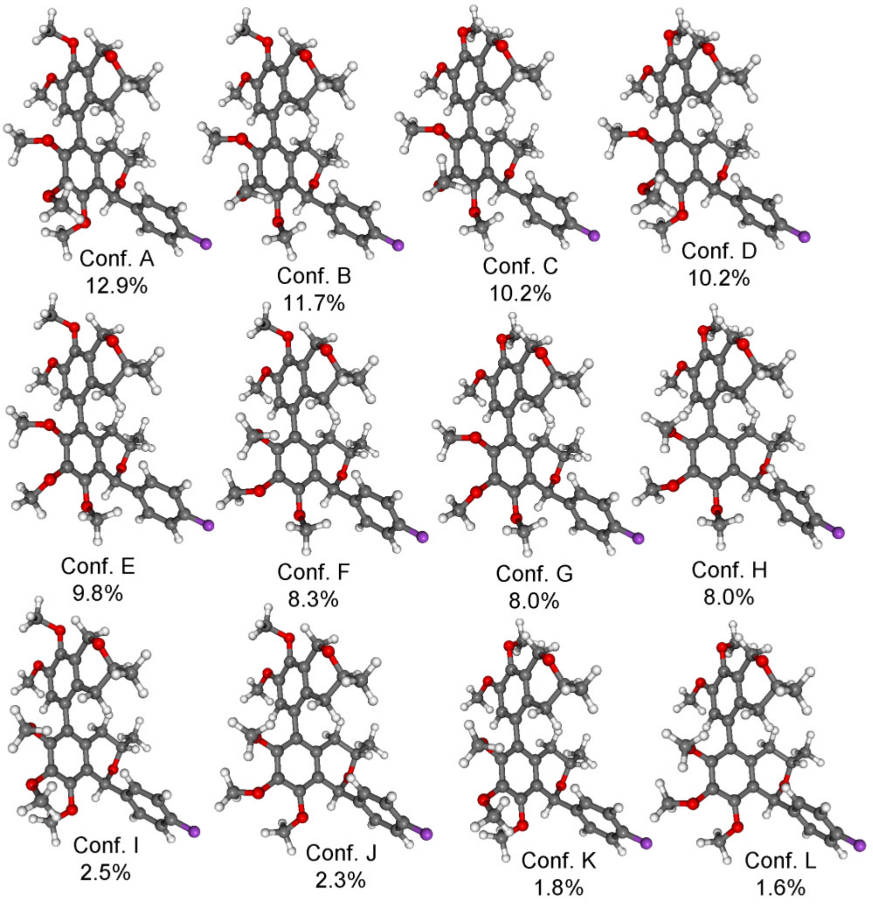

Figure S273. Structures and populations of the low-energy ωB97X/TZVP PCM/MeCN conformers (≥ 1%) of (*aS*,1*S*,3*S*,3'*S*)-**21**.

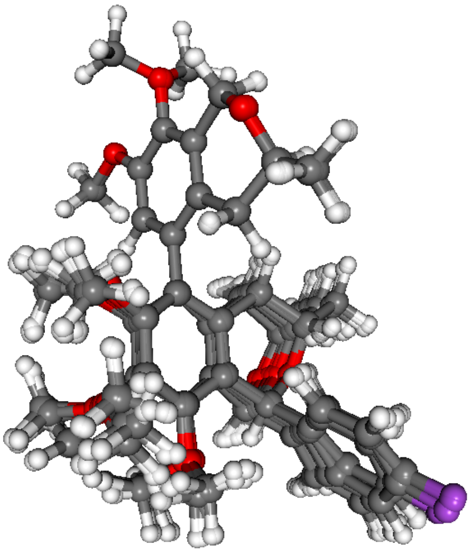

Figure S274. Overlapped structures of the 12 low-energy ωB97X/TZVP PCM/MeCN conformers (≥ 1%) of (*aS*,1*S*,3*S*,3'*S*)-**21**.

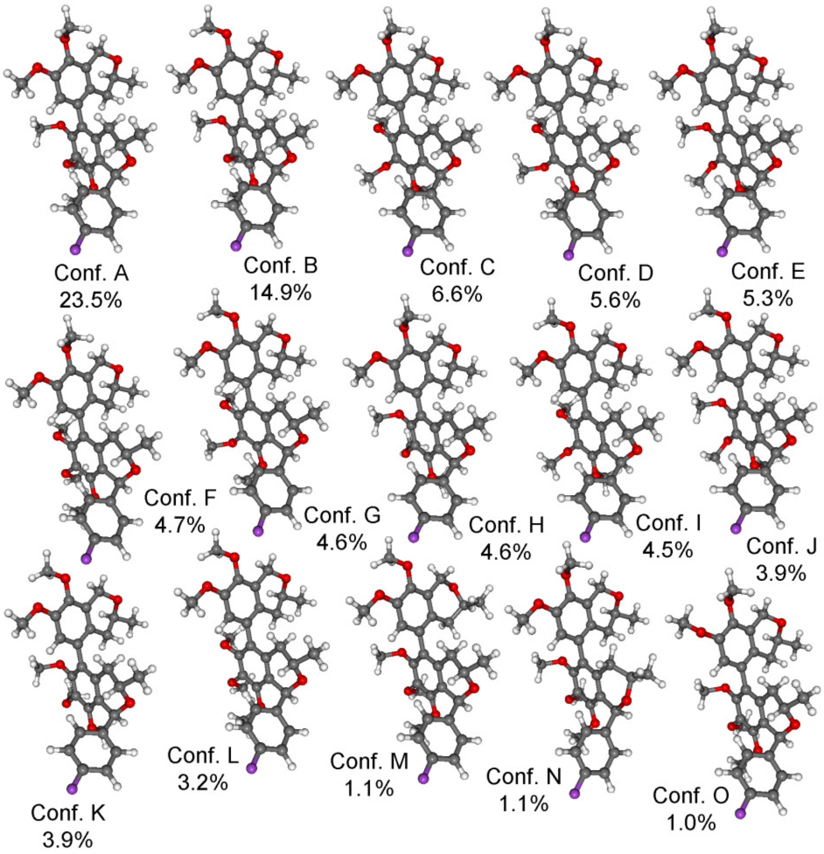

Figure S275. Structures and populations of the low-energy ωB97X/TZVP PCM/MeCN conformers (≥ 1%) of (*aR*,1*S*,3*S*,3’*S*)-**21**.

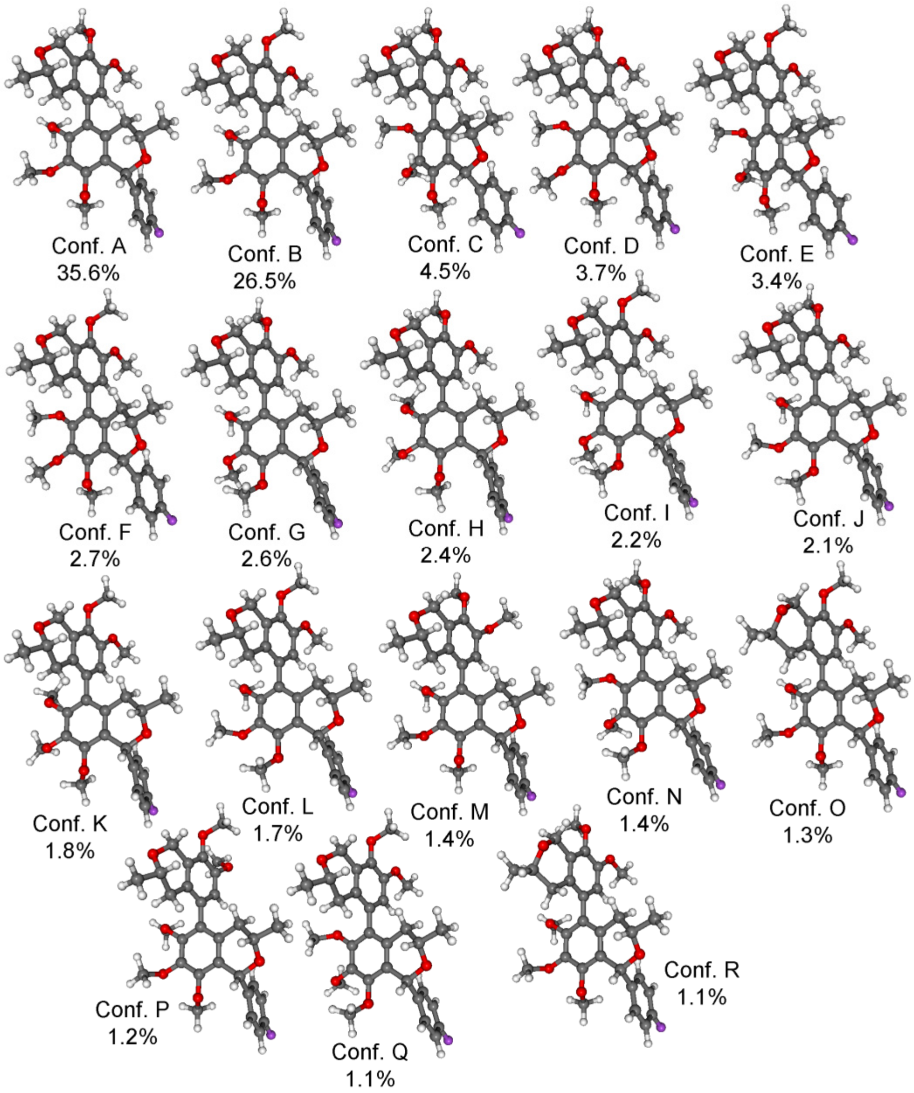

Figure S276. Structures and populations of the low-energy ωB97X/TZVP PCM/MeCN conformers (≥ 1%) of (*aS*,1*R*,3*S*,3'*S*)-**21**.

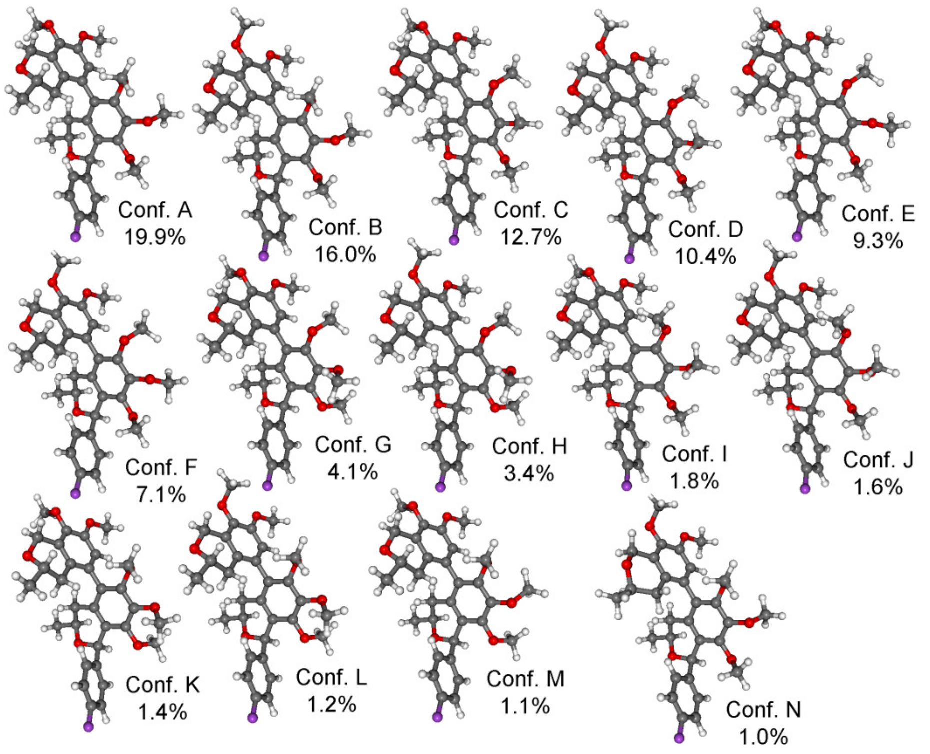

Figure S277. Structures and populations of the low-energy ωB97X/TZVP PCM/MeCN conformers (≥ 1%) of (*aR*,1*R*,3*S*,3'*S*)-**21**.

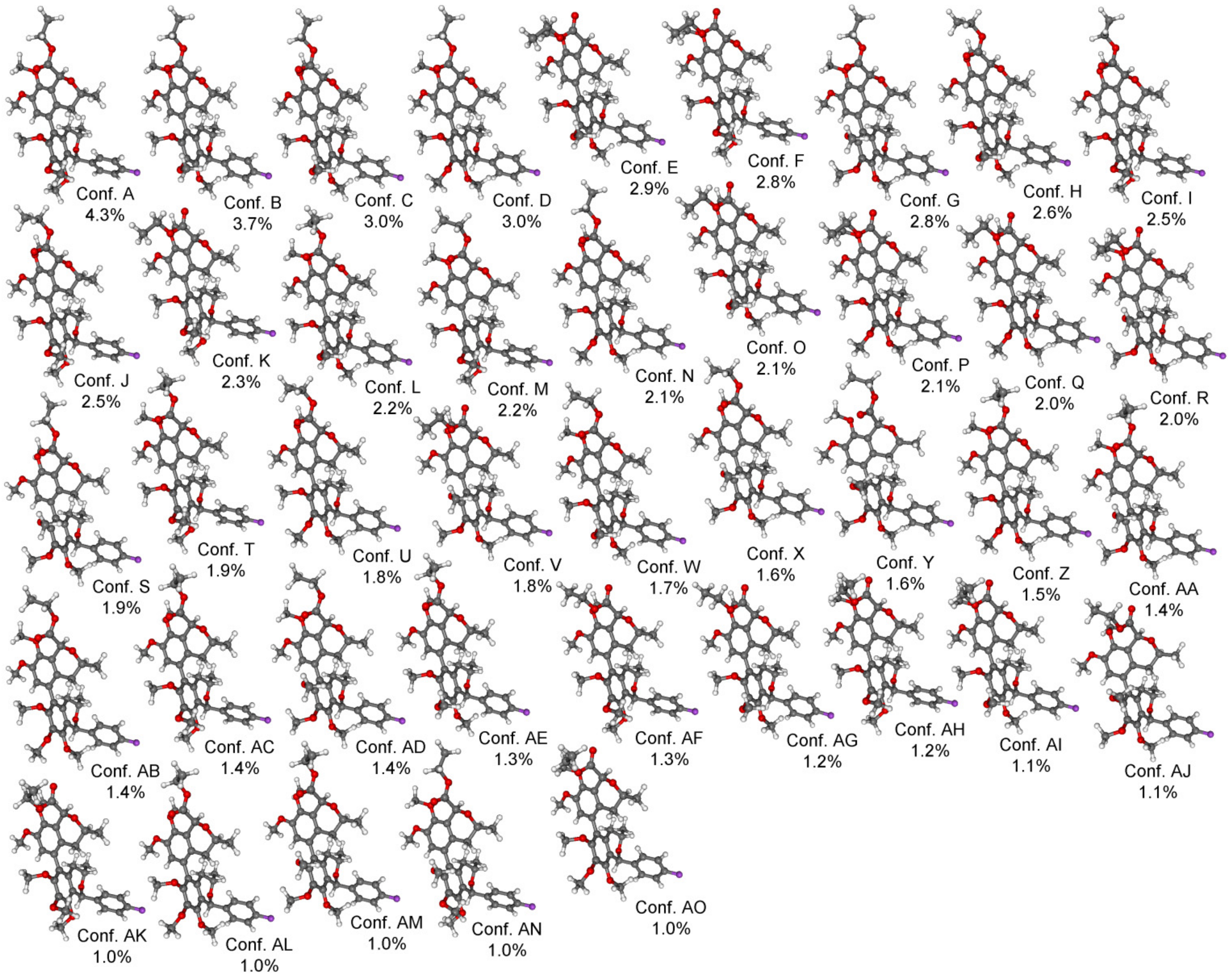

Figure S278. Structures and populations of the low-energy ωB97X/TZVP PCM/MeCN conformers (≥ 1%) of (*aS*,1*S*,3*S*,1'*S*,3'*S*)-**22**.

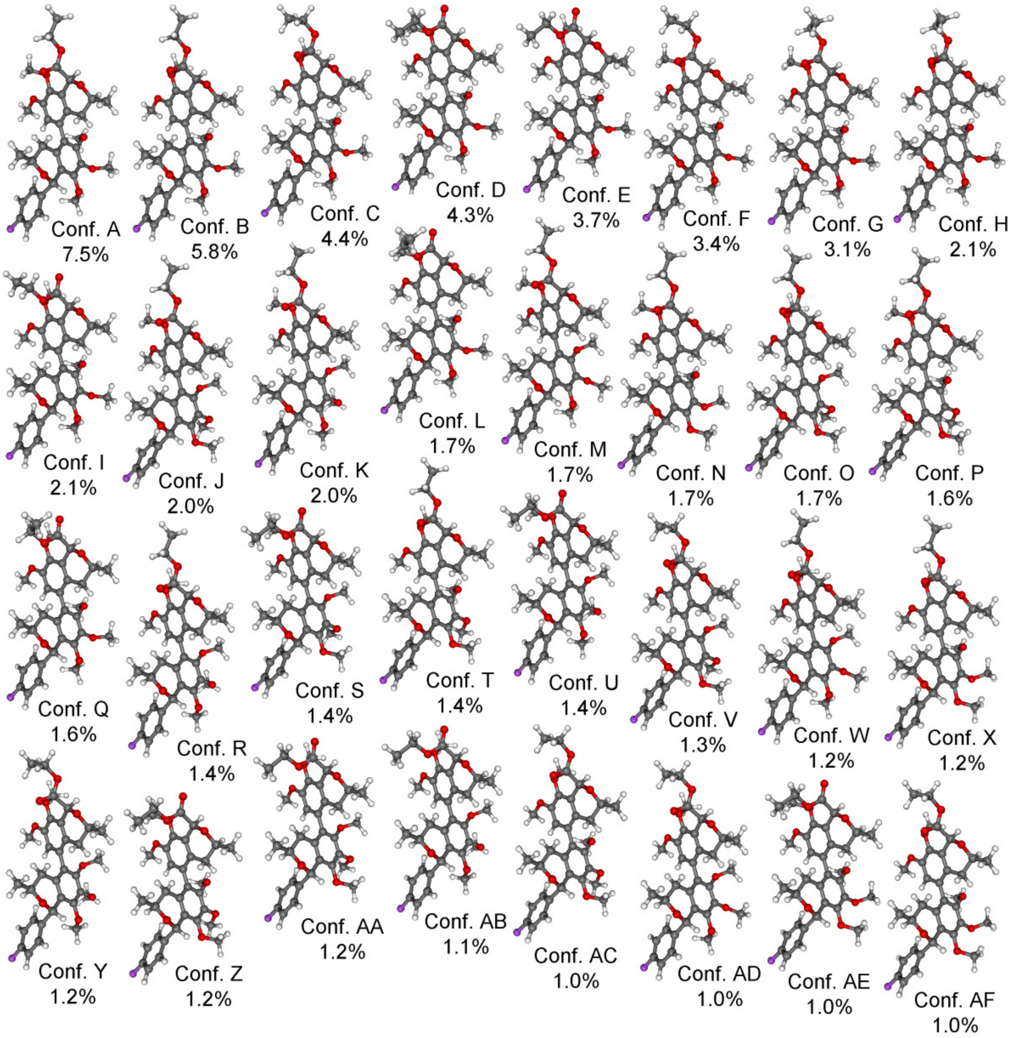

Figure S279. Structures and populations of the low-energy ωB97X/TZVP PCM/MeCN conformers (≥ 1%) of (*aR*,1*S*,3*S*,1’*S*,3’*S*)-**22**.

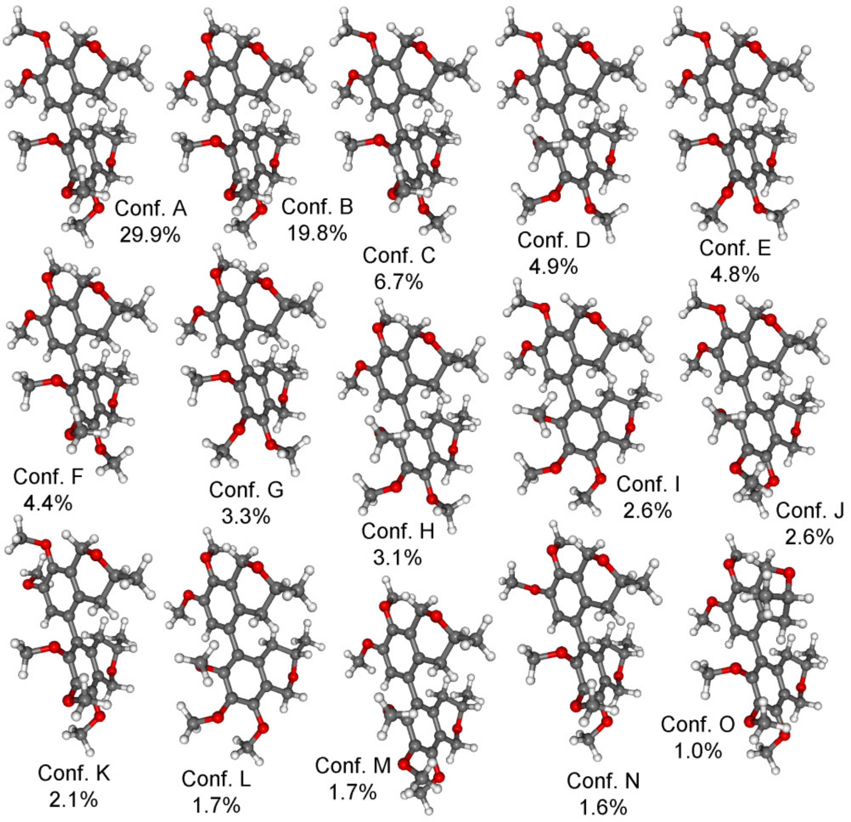

Figure S280. Structures and populations of the low-energy ωB97X/TZVP PCM/MeCN conformers (≥ 1%) of (*aS*,3*S*,3'*S*)-**28**.

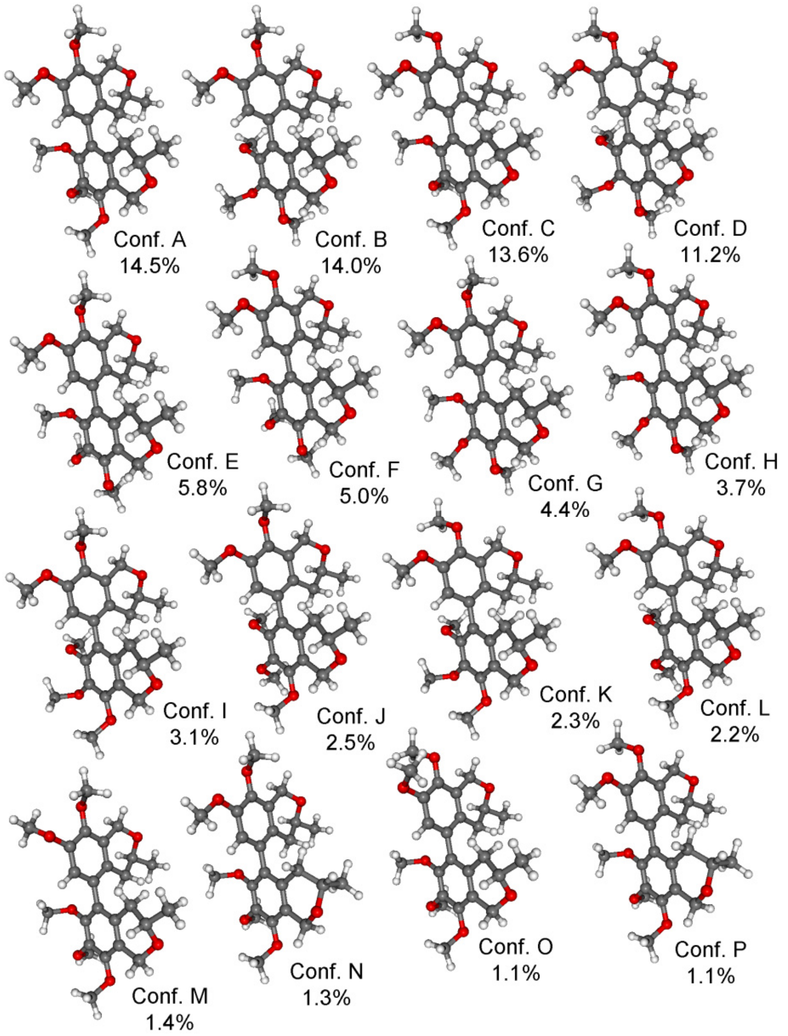

Figure S281. Structures and populations of the low-energy ωB97X/TZVP PCM/MeCN conformers (≥ 1%) of (*aR*,3*S*,3'*S*)-**28**.

Table S5. Cartesian coordinates and energies of the low-energy conformers calculated at the ωB97X/TZVP PCM/MeCN level.

(*aS*,1*S*,3*S*,3'*S*)-**21**, Conf A

C	2.709496	0.616496	−1.323124
C	3.996849	0.120643	−1.476552
C	4.506569	−0.749861	−0.506340
C	3.731917	−1.118796	0.582816
C	2.429042	−0.636030	0.725436
C	1.928692	0.242920	−0.230984
C	1.603617	−1.059134	1.916361
C	2.198233	−2.271810	2.617817
C	1.568317	−2.528798	3.967681
O	3.594920	−2.066322	2.820453
C	0.284853	1.857611	0.721096
C	−0.996360	2.403266	0.815426
C	−2.023835	1.862257	0.049566
C	−1.788030	0.767209	−0.779222
C	−0.508521	0.225554	−0.866826
C	0.540449	0.784790	−0.126126
C	−0.255294	−0.946865	−1.785209
C	−1.544966	−1.611583	−2.237224
C	−1.337564	−2.560059	−3.395935
O	−2.469290	−0.609108	−2.660138
C	−2.937073	0.201471	−1.587969
C	4.309241	−2.073058	1.602418
C	−3.975203	−0.550690	−0.764098
C	−3.723222	−1.051681	0.507370
C	−4.691391	−1.771567	1.197085
C	−5.911739	−1.976405	0.590150
C	−6.203721	−1.493729	−0.670903
C	−5.222951	−0.780479	−1.340333
F	−6.863270	−2.672802	1.256340
O	4.816573	0.413153	−2.519694
C	4.327044	1.298931	−3.515507
O	5.760894	−1.295485	−0.641155
C	6.828609	−0.377322	−0.400792
O	−1.220674	3.476885	1.636116
C	−1.806052	3.122587	2.890907
O	−3.297829	2.361402	0.140683
C	−3.470433	3.663966	−0.426043
O	1.285412	2.355613	1.514542
C	1.857585	3.571422	1.031877
H	2.290285	1.292465	−2.057821
H	1.538634	−0.228600	2.629117
H	0.580135	−1.286846	1.603918
H	2.072355	−3.157093	1.976209
H	1.737550	−1.676387	4.629703
H	0.491799	−2.674231	3.860197
H	1.994540	−3.420175	4.429951
H	0.380241	−1.682900	−1.285152
H	0.298654	−0.604877	−2.666792
H	−1.984432	−2.160701	−1.393656
H	−0.958330	−2.017666	−4.265137
H	−2.275696	−3.045348	−3.668632
H	−0.613116	−3.330952	−3.126670
H	−3.450126	1.031994	−2.077665
H	4.326230	−3.089695	1.183524
H	5.338272	−1.801715	1.838167
H	−2.761566	−0.877801	0.978085
H	−4.505830	−2.166523	2.188317
H	−7.177877	−1.671970	−1.109244
H	−5.432182	−0.392679	−2.331897
H	3.434798	0.893510	−4.000353
H	5.123458	1.396247	−4.249178
H	4.099438	2.281348	−3.092799
H	6.756843	0.036972	0.609585
H	7.754079	−0.942180	−0.495196
H	6.816763	0.432708	−1.132004
H	−1.138882	2.460845	3.449856
H	−2.772692	2.635462	2.740728
H	−1.945636	4.049922	3.442862
H	−4.522552	3.913770	−0.306181
H	−3.218132	3.651296	−1.490383
H	−2.852870	4.398622	0.092771
H	2.325457	3.411003	0.055571
H	1.098019	4.352851	0.956857
H	2.616794	3.867899	1.752919

ωB97X Energy = −1828.88132787 a.u.

(*aS*,1*S*,3*S*,3'*S*)-**21**, Conf B

C	2.736424	−0.540025	−1.366386
C	4.025015	−0.977484	−1.093517
C	4.511307	−0.875450	0.214938
C	3.711298	−0.360773	1.223573
C	2.406298	0.056790	0.952986
C	1.930936	−0.023827	−0.353101
C	1.548194	0.595725	2.072068
C	2.127659	0.265909	3.439975
C	1.461484	1.036781	4.556693
O	3.517028	0.586431	3.456960
C	0.288172	1.776647	−0.858588
C	−0.991351	2.235252	−1.190830
C	−2.013979	1.307240	−1.364813
C	−1.764863	−0.056818	−1.199764
C	−0.493328	−0.502100	−0.858749
C	0.546297	0.422049	−0.692839
C	−0.241705	−1.980406	−0.677607
C	−1.533130	−2.769570	−0.537971
C	−1.323872	−4.259701	−0.684597
O	−2.447141	−2.363692	−1.555908
C	−2.910628	−1.027857	−1.397077
C	4.265761	−0.276494	2.626941
C	−3.975873	−0.950790	−0.309696
C	−3.725532	−0.452319	0.963905

C	−4.716291	−0.443428	1.938123
C	−5.959729	−0.940235	1.611332
C	−6.251154	−1.443610	0.358275
C	−5.247188	−1.443973	−0.596479
F	−6.934316	−0.930962	2.551255
O	4.869328	−1.513229	−2.012841
C	4.402088	−1.635330	−3.347929
O	5.767198	−1.333949	0.533870
C	6.831221	−0.503863	0.065388
O	−1.228119	3.562381	−1.431568
C	−1.144084	4.415117	−0.285116
O	−3.270195	1.666775	−1.766273
C	−3.978844	2.608226	−0.959460
O	1.298263	2.689439	−0.680743
C	1.897587	3.132070	−1.899653
H	2.335183	−0.604815	−2.370021
H	1.455723	1.683689	1.972839
H	0.536492	0.185534	1.998650
H	2.023044	−0.814237	3.623481
H	1.607224	2.109646	4.410836
H	0.389154	0.832883	4.570128
H	1.880803	0.755734	5.523668
H	0.386042	−2.146981	0.202042
H	0.318608	−2.363592	−1.537973
H	−1.982260	−2.561518	0.442622
H	−0.929725	−4.488903	−1.677390
H	−2.264783	−4.794819	−0.549518
H	−0.610768	−4.615969	0.061195
H	−3.397058	−0.794406	−2.345745
H	4.302710	−1.282503	3.069294
H	5.285091	0.109457	2.610274
H	−2.746062	−0.054907	1.207877
H	−4.530820	−0.054856	2.931906
H	−7.243278	−1.820418	0.142032
H	−5.456979	−1.835341	−1.586580
H	5.215897	−2.086670	−3.910114
H	4.163874	−0.657475	−3.775447
H	3.521890	−2.281991	−3.400767
H	6.822817	−0.437850	−1.024026
H	6.751765	0.498837	0.496604
H	7.758085	−0.967631	0.397444
H	−0.141783	4.393710	0.143690
H	−1.875549	4.111724	0.469636
H	−1.377657	5.419569	−0.632612
H	−3.669574	3.629517	−1.182085
H	−3.826116	2.396482	0.102570
H	−5.033455	2.482518	−1.198874
H	1.153413	3.596317	−2.551350
H	2.654092	3.865278	−1.627173
H	2.372796	2.292895	−2.416515

ωB97X Energy = −1828.88124038 a.u.

(*aS*,1*S*,3*S*,3'*S*)-**21**, Conf C

C	2.745246	−0.364807	−1.414406
C	4.056856	−0.748114	−1.171643
C	4.564747	−0.637127	0.127556
C	3.776381	−0.131054	1.149928
C	2.453918	0.246497	0.906626
C	1.947209	0.131345	−0.385210
C	1.607944	0.769365	2.042137
C	2.231191	0.468028	3.397150
C	1.566130	1.222338	4.525775
O	3.608043	0.838064	3.377593
C	0.222650	1.867167	−0.846738
C	−1.079187	2.273838	−1.158895
C	−2.064303	1.305318	−1.328844
C	−1.756023	−0.048577	−1.180658
C	−0.462558	−0.442354	−0.858562
C	0.539561	0.523123	−0.696362
C	−0.146293	−1.910467	−0.695080
C	−1.401499	−2.755691	−0.556005
C	−1.130696	−4.234004	−0.720141
O	−2.338269	−2.377773	−1.563881
C	−2.859757	−1.066157	−1.382944
C	4.369141	−0.000338	2.534246
C	−3.920193	−1.054857	−0.288345
C	−3.688889	−0.554918	0.988170
C	−4.672391	−0.608894	1.968414
C	−5.888878	−1.170027	1.644525
C	−6.160751	−1.677189	0.388537
C	−5.164744	−1.613627	−0.572305
F	−6.856089	−1.223430	2.590622
O	4.909347	−1.226023	−2.114903
C	4.420297	−1.358973	−3.441046
O	5.870176	−0.972754	0.396601
C	6.111785	−2.378859	0.450532
O	−1.373828	3.591420	−1.387906
C	−1.310759	4.440563	−0.237610
O	−3.338879	1.615458	−1.712859
C	−4.076488	2.519616	−0.889656
O	1.195865	2.820492	−0.676784
C	1.760745	3.292067	−1.901356
H	2.324720	−0.443091	−2.409077
H	1.481387	1.853273	1.937595
H	0.607664	0.328460	1.996887
H	2.169152	−0.614039	3.588863
H	1.672374	2.298577	4.371018
H	0.501899	0.982832	4.566459
H	2.017813	0.961531	5.483825
H	0.494560	−2.058477	0.178471
H	0.423957	−2.260099	−1.563148
H	−1.852807	−2.577280	0.429458
H	−0.731610	−4.435866	−1.716881
H	−2.048045	−4.809136	−0.587439
H	−0.400472	−4.568664	0.019057
H	−3.362503	−0.840995	−2.325119
H	4.469473	−0.996793	2.989806
H	5.366917	0.433427	2.473812
H	−2.730436	−0.108127	1.230013
H	−4.501678	−0.220006	2.964714
H	−7.132064	−2.106094	0.175033

H	−5.359221	−2.007171	−1.564660
H	3.579025	−2.056276	−3.484170
H	5.246624	−1.753242	−4.027389
H	4.114784	−0.391744	−3.849529
H	7.163483	−2.506697	0.699793
H	5.495778	−2.844924	1.225649
H	5.905762	−2.846612	−0.514261
H	−0.301961	4.462968	0.175648
H	−2.015900	4.099939	0.526260
H	−1.595572	5.435178	−0.575136
H	−3.811433	3.554704	−1.105463
H	−3.904060	2.304383	0.168654
H	−5.127644	2.353568	−1.119441
H	0.990302	3.732926	−2.538663
H	2.494497	4.050231	−1.635105
H	2.258027	2.473745	−2.430775

ωB97X Energy = −1828.88111088 a.u.

(*aS*,1*S*,3*S*,3'*S*)-**21**, Conf D

C	2.789626	0.839377	−1.141741
C	4.104072	0.400958	−1.227623
C	4.574600	−0.516992	−0.282148
C	3.748072	−0.959376	0.739841
C	2.424901	−0.520303	0.823691
C	1.954249	0.385021	−0.123404
C	1.538798	−1.033169	1.933095
C	2.127406	−2.268307	2.599083
C	1.415980	−2.634898	3.881351
O	3.497124	−2.027866	2.913822
C	0.219257	1.961904	0.722954
C	−1.083730	2.461598	0.755563
C	−2.062817	1.861017	−0.028918
C	−1.757277	0.752589	−0.816261
C	−0.456605	0.257535	−0.842832
C	0.543779	0.876039	−0.082823
C	−0.126326	−0.929353	−1.716451
C	−1.373294	−1.659807	−2.186430
C	−1.092168	−2.635167	−3.306688
O	−2.322571	−0.709670	−2.670891
C	−2.856122	0.117743	−1.643024
C	4.298375	−1.934418	1.755091
C	−3.892418	−0.644497	−0.826119
C	−5.117561	−0.928717	−1.425924
C	−6.093374	−1.652295	−0.760107
C	−5.819320	−2.089520	0.521344
C	−4.621231	−1.830837	1.151704
C	−3.657976	−1.101443	0.465286
F	−6.766556	−2.794781	1.184330
O	4.991767	0.803961	−2.173338
C	4.544636	1.742216	−3.140534
O	5.879556	−0.946292	−0.315324
C	6.161820	−1.887836	−1.351010
O	−1.376794	3.547184	1.538103
C	−1.979066	3.202015	2.787403
O	−3.357268	2.311750	0.004938
C	−3.559485	3.592847	−0.599602
O	1.171059	2.516232	1.538449
C	1.710328	3.745319	1.051404
H	2.396843	1.542970	−1.864977
H	1.400655	−0.250345	2.688286
H	0.545352	−1.270692	1.540974
H	2.080176	−3.113019	1.894948
H	1.504720	−1.822374	4.606280
H	0.356019	−2.811277	3.689006
H	1.844757	−3.538723	4.316296
H	0.521936	−1.623495	−1.174455
H	0.441858	−0.589936	−2.590085
H	−1.817540	−2.199143	−1.339052
H	−0.704307	−2.106011	−4.180231
H	−2.002240	−3.163601	−3.593878
H	−0.349502	−3.369593	−2.989499
H	−3.382947	0.911283	−2.177485
H	4.404262	−2.927027	1.292402
H	5.289244	−1.618343	2.080140
H	−5.313446	−0.575740	−2.433136
H	−7.050674	−1.871955	−1.216435
H	−4.449167	−2.191094	2.158479
H	−2.714414	−0.884584	0.954406
H	5.394961	1.932370	−3.790776
H	4.232198	2.677567	−2.668037
H	3.718445	1.337954	−3.731816
H	5.535836	−2.778642	−1.241585
H	6.001802	−1.443123	−2.335151
H	7.208222	−2.166504	−1.241962
H	−1.305116	2.572647	3.374828
H	−2.927324	2.682899	2.627064
H	−2.158376	4.136089	3.316040
H	−3.273064	3.563630	−1.654924
H	−2.986267	4.362498	−0.080608
H	−4.623337	3.805940	−0.518797
H	2.439241	4.079968	1.786876
H	2.209524	3.587695	0.090299
H	0.924924	4.496880	0.944489

ωB97X Energy = −1828.88110805 a.u.

(*aS*,1*S*,3*S*,3'*S*)-**21**, Conf E

C	−2.733379	0.612778	1.273555
C	−4.017655	0.105372	1.413940
C	−4.503835	−0.781938	0.446941
C	−3.708102	−1.157795	−0.624576
C	−2.407465	−0.664985	−0.752434
C	−1.931348	0.232560	0.199198
C	−1.557555	−1.099149	−1.921827
C	−2.129919	−2.327257	−2.614928
C	−1.473951	−2.600274	−3.949096
O	−3.524308	−2.135524	−2.845786
C	−0.287218	1.838974	−0.762476
C	0.988365	2.406599	−0.844876
C	2.021222	1.851880	−0.092342
C	1.770229	0.797712	0.786094

C	0.489721	0.266327	0.892892
C	−0.546798	0.786372	0.107570
C	0.225434	−0.859853	1.864490
C	1.506391	−1.520183	2.347176
C	1.286035	−2.402759	3.554829
O	2.448141	−0.514062	2.718055
C	2.916356	0.257027	1.616853
C	−4.260545	−2.130139	−1.640846
C	3.949138	−0.522427	0.813811
C	5.249319	−0.601052	1.307766
C	6.224861	−1.334979	0.652020
C	5.873868	−1.994841	−0.509540
C	4.599646	−1.944352	−1.033585
C	3.639136	−1.199245	−0.361107
F	6.818912	−2.712881	−1.160563
O	−4.856001	0.401982	2.441075
C	−4.387974	1.299530	3.436605
O	−5.754855	−1.338075	0.568286
C	−6.827692	−0.432351	0.304538
O	1.186091	3.456027	−1.701183
C	1.586095	4.682404	−1.083007
O	3.292967	2.363899	−0.137562
C	3.966189	2.248157	−1.394968
O	−1.286014	2.307897	−1.576458
C	−1.945304	3.474265	−1.089385
H	−2.333063	1.302847	2.005709
H	−1.486049	−0.279334	−2.646013
H	−0.538364	−1.313547	−1.586601
H	−2.009267	−3.201548	−1.957405
H	−1.636370	−1.759179	−4.627097
H	−0.398657	−2.737065	−3.820190
H	−1.886170	−3.501278	−4.405382
H	−0.421005	−1.610211	1.401473
H	−0.321454	−0.469365	2.730248
H	1.933422	−2.121947	1.534033
H	0.915732	−1.809037	4.393812
H	2.217146	−2.886547	3.853019
H	0.550147	−3.176255	3.327087
H	3.431305	1.102460	2.076122
H	−4.278606	−3.140997	−1.208276
H	−5.286897	−1.868823	−1.898752
H	5.506073	−0.075988	2.222029
H	7.239241	−1.396614	1.026492
H	4.368239	−2.472754	−1.950204
H	2.637080	−1.140939	−0.772793
H	−4.163595	2.280893	3.009740
H	−3.498661	0.905747	3.936245
H	−5.194954	1.394973	4.158892
H	−6.835360	0.382847	1.030074
H	−6.743900	−0.024579	−0.707551
H	−7.748832	−1.005914	0.388282
H	0.872589	4.968886	−0.305125
H	1.586840	5.434686	−1.868885
H	2.582451	4.600566	−0.645528
H	4.054629	1.197184	−1.681798
H	4.959095	2.667892	−1.247257
H	3.442401	2.801721	−2.176152
H	−2.718184	3.723142	−1.813914
H	−2.406972	3.277034	−0.116957
H	−1.248000	4.311533	−1.003436

ωB97X Energy = −1828.88106565 a.u.

(*aS*,1*S*,3*S*,3’*S*)-**21**, Conf F

C	−2.796359	0.764722	1.226860
C	−4.082849	0.262185	1.362013
C	−4.522928	−0.720894	0.468243
C	−3.680332	−1.193889	−0.525625
C	−2.377512	−0.703797	−0.646718
C	−1.947252	0.288046	0.230556
C	−1.472866	−1.255834	−1.722993
C	−2.007998	−2.559028	−2.298809
C	−1.286611	−2.978617	−3.559185
O	−3.388590	−2.402716	−2.618235
C	−0.298732	1.923348	−0.685586
C	0.980051	2.488636	−0.731052
C	2.007201	1.911480	0.009481
C	1.757609	0.815395	0.833833
C	0.472773	0.288387	0.914187
C	−0.561311	0.840370	0.148983
C	0.199837	−0.872726	1.840811
C	1.477246	−1.557095	2.298184
C	1.250610	−2.496486	3.461005
O	2.418237	−0.571027	2.720362
C	2.895154	0.248341	1.657906
C	−4.184750	−2.269253	−1.459588
C	3.944744	−0.489137	0.837485
C	5.240409	−0.573338	1.342202
C	6.231364	−1.269134	0.668189
C	5.900318	−1.885044	−0.522938
C	4.631214	−1.827435	−1.058746
C	3.655115	−1.120758	−0.367527
F	6.860410	−2.565549	−1.192086
O	−4.966712	0.650887	2.317405
C	−4.545645	1.641966	3.242837
O	−5.775034	−1.273992	0.594946
C	−6.838913	−0.412780	0.186115
O	1.236563	3.602961	−1.487806
C	0.744675	4.813743	−0.908058
O	3.258320	2.468956	0.026066
C	3.980293	2.414070	−1.207692
O	−1.338944	2.468175	−1.387335
C	−1.177752	2.572777	−2.804003
H	−2.430426	1.526971	1.902969
H	−1.372493	−0.526993	−2.536155
H	−0.467480	−1.419694	−1.323674
H	−1.918655	−3.351096	−1.539799
H	−1.407801	−2.216909	−4.333050
H	−0.220307	−3.103684	−3.361820
H	−1.683074	−3.923026	−3.934449
H	−0.448719	−1.602719	1.348990
H	−0.347760	−0.511990	2.719134

H	1.907230	−2.119624	1.458713
H	0.877068	−1.943510	4.325965
H	2.180096	−2.994820	3.739696
H	0.515237	−3.257404	3.193090
H	3.397768	1.077855	2.158245
H	−4.229754	−3.228564	−0.924044
H	−5.195160	−2.037757	−1.796874
H	5.481308	−0.082176	2.279314
H	7.242388	−1.334446	1.051007
H	4.416165	−2.320865	−1.998543
H	2.657065	−1.055509	−0.787868
H	−3.677345	1.304394	3.815281
H	−5.383719	1.798725	3.917468
H	−4.306458	2.580681	2.735845
H	−6.864434	0.490982	0.797718
H	−6.727502	−0.141988	−0.868337
H	−7.763214	−0.971524	0.320126
H	1.205035	4.981754	0.069048
H	−0.342805	4.780306	−0.802831
H	1.022979	5.618165	−1.585940
H	4.136613	1.374580	−1.508969
H	4.943807	2.883152	−1.018324
H	3.452567	2.957426	−1.991850
H	−0.730428	1.660511	−3.207882
H	−2.177433	2.696645	−3.215975
H	−0.555576	3.426810	−3.072893

ωB97X Energy = −1828.88090967 a.u.

(*aS*,1*S*,3*S*,3’*S*)-**21**, Conf G

C	2.817741	0.839715	−1.083757
C	4.128143	0.387416	−1.158641
C	4.574368	−0.552459	−0.223055
C	3.727775	−1.003859	0.778331
C	2.408535	−0.551246	0.850684
C	1.962240	0.376986	−0.086029
C	1.498876	−1.076769	1.934664
C	2.064090	−2.330485	2.586134
C	1.328689	−2.713546	3.849960
O	3.430931	−2.110271	2.927215
C	0.226096	1.950435	0.763309
C	−1.072929	2.469916	0.780725
C	−2.056103	1.849967	0.013244
C	−1.734501	0.775834	−0.816895
C	−0.432473	0.290850	−0.858179
C	0.555223	0.879076	−0.058596
C	−0.090658	−0.862028	−1.771851
C	−1.328844	−1.589315	−2.269083
C	−1.034402	−2.515682	−3.427176
O	−2.290955	−0.635248	−2.717423
C	−2.828206	0.163200	−1.668208
C	4.251988	−2.003194	1.783622
C	−3.867908	−0.616808	−0.874746
C	−5.144823	−0.753939	−1.414494
C	−6.124207	−1.490273	−0.767292
C	−5.800717	−2.092806	0.432737
C	−4.549992	−1.983677	1.002479
C	−3.585278	−1.237143	0.337610
F	−6.749981	−2.812266	1.076129
O	5.034104	0.796270	−2.084325
C	4.613205	1.758259	−3.039754
O	5.874596	−0.996862	−0.245758
C	6.157858	−1.926968	−1.291456
O	−1.342468	3.538020	1.593512
C	−1.767089	4.726413	0.920144
O	−3.349667	2.305307	−0.000087
C	−4.054077	2.217564	1.242172
O	1.177558	2.479178	1.596711
C	1.779217	3.682359	1.124532
H	2.443676	1.559918	−1.800613
H	1.355125	−0.307529	2.702525
H	0.510225	−1.296472	1.520485
H	2.019671	−3.161334	1.865546
H	1.411954	−1.914445	4.590273
H	0.270634	−2.878046	3.637620
H	1.743408	−3.628176	4.275892
H	0.564270	−1.568150	−1.254112
H	0.474187	−0.486709	−2.632881
H	−1.764242	−2.169264	−1.444571
H	−0.652957	−1.946768	−4.278243
H	−1.937291	−3.043950	−3.736459
H	−0.282547	−3.252506	−3.138508
H	−3.351056	0.971803	−2.181968
H	4.355986	−2.988081	1.304305
H	5.240310	−1.703093	2.130763
H	−5.380194	−0.272459	−2.358001
H	−7.120907	−1.596644	−1.177452
H	−4.340210	−2.468654	1.947845
H	−2.602060	−1.132923	0.784365
H	3.792912	1.375139	−3.652964
H	5.476444	1.951856	−3.671726
H	4.303085	2.687614	−2.554050
H	5.519567	−2.811325	−1.202430
H	6.014954	−1.466385	−2.270971
H	7.199355	−2.220342	−1.174289
H	−2.740948	4.589027	0.447137
H	−1.035328	5.018584	0.161573
H	−1.830455	5.501818	1.680663
H	−3.643465	2.908407	1.980562
H	−4.012601	1.197302	1.631786
H	−5.087611	2.479392	1.024942
H	2.530102	3.965853	1.859360
H	2.261788	3.516642	0.156484
H	1.040245	4.482379	1.036389

ωB97X Energy = −1828.88087275 a.u.

(*aS*,1*S*,3*S*,3’*S*)-**21**, Conf H

C	2.894938	1.012528	−0.951530
C	4.205645	0.561263	−1.009389
C	4.589704	−0.508044	−0.191897
C	3.682265	−1.085210	0.682533

C	2.361204	−0.631628	0.739898
C	1.976884	0.424299	−0.083849
C	1.389376	−1.307415	1.679524
C	1.908157	−2.658576	2.149176
C	1.090123	−3.237010	3.281065
O	3.248534	−2.511584	2.610654
C	0.246359	2.049817	0.685795
C	−1.053303	2.565948	0.677443
C	−2.035265	1.930947	−0.073781
C	−1.718278	0.813607	−0.847742
C	−0.417646	0.321892	−0.858990
C	0.572737	0.935824	−0.079468
C	−0.074669	−0.856990	−1.738997
C	−1.312302	−1.596205	−2.219092
C	−1.014610	−2.566235	−3.339959
O	−2.265700	−0.654537	−2.709209
C	−2.806126	0.185221	−1.694513
C	4.143057	−2.229841	1.555503
C	−3.865997	−0.554795	−0.889780
C	−5.138203	−0.692347	−1.440428
C	−6.135292	−1.393856	−0.781666
C	−5.834371	−1.961082	0.441250
C	−4.589210	−1.849802	1.022752
C	−3.606594	−1.138166	0.345972
F	−6.800836	−2.646701	1.095741
O	5.169024	1.086253	−1.809563
C	4.809308	2.170067	−2.653514
O	5.886014	−0.963977	−0.200712
C	6.229669	−1.731835	−1.354598
O	−1.359967	3.686749	1.404855
C	−0.987147	4.905273	0.756771
O	−3.303467	2.442020	−0.153767
C	−4.064968	2.412583	1.057309
O	1.224938	2.702258	1.389952
C	1.137711	2.561460	2.808711
H	2.567697	1.833467	−1.576686
H	1.212666	−0.677877	2.559391
H	0.418752	−1.440533	1.193151
H	1.910514	−3.358612	1.299648
H	1.118368	−2.569154	4.145209
H	0.050097	−3.359439	2.972838
H	1.480555	−4.210806	3.579522
H	0.578931	−1.552014	−1.205733
H	0.491879	−0.502675	−2.608021
H	−1.756460	−2.143192	−1.376560
H	−0.622884	−2.031522	−4.208343
H	−1.918642	−3.099616	−3.636824
H	−0.269964	−3.296872	−3.018697
H	−3.312025	0.982541	−2.241622
H	4.285108	−3.128491	0.936930
H	5.104081	−1.992011	2.010588
H	−5.355606	−0.238512	−2.401754
H	−7.128434	−1.500275	−1.200322
H	−4.397510	−2.306563	1.985830
H	−2.627983	−1.030601	0.801681
H	4.029745	1.877322	−3.362041
H	5.711140	2.437938	−3.198518
H	4.468945	3.029676	−2.069912
H	7.259898	−2.055301	−1.218712
H	5.581666	−2.609680	−1.437856
H	6.151263	−1.128715	−2.261236
H	−1.509029	5.004336	−0.198792
H	0.092972	4.940117	0.596247
H	−1.284952	5.714754	1.420245
H	−5.036614	2.839374	0.816636
H	−3.582581	3.005639	1.834597
H	−4.196259	1.381624	1.397388
H	1.252614	1.512085	3.095058
H	1.958584	3.139531	3.228200
H	0.187054	2.946747	3.182065

ωB97X Energy = −1828.88087212 a.u.

(*aS*,1*S*,3*S*,3'*S*)-**21**, Conf I

C	−2.778045	0.778028	1.289762
C	−4.073602	0.300123	1.429049
C	−4.555452	−0.629566	0.500350
C	−3.744644	−1.075079	−0.532047
C	−2.433086	−0.610581	−0.658058
C	−1.961201	0.328760	0.254732
C	−1.564160	−1.133776	−1.777084
C	−2.139654	−2.400675	−2.393047
C	−1.450792	−2.788324	−3.681445
O	−3.521743	−2.199922	−2.680012
C	−0.285218	1.949774	−0.637024
C	1.010763	2.474222	−0.697294
C	2.016568	1.880350	0.062886
C	1.755847	0.756463	0.841577
C	0.464567	0.241835	0.900383
C	−0.562906	0.849028	0.171292
C	0.177516	−0.956512	1.773613
C	1.449182	−1.670573	2.200044
C	1.215842	−2.663726	3.315682
O	2.394503	−0.709407	2.669652
C	2.886593	0.137537	1.637030
C	−4.293744	−2.095297	−1.502196
C	3.918263	−0.597300	0.789387
C	5.154702	−0.881273	1.365983
C	6.128339	−1.580106	0.671366
C	5.840851	−1.992347	−0.615518
C	4.631456	−1.733147	−1.223467
C	3.670282	−1.029085	−0.508006
F	6.786008	−2.673242	−1.306433
O	−4.927295	0.663649	2.421233
C	−4.462601	1.600053	3.382178
O	−5.817197	−1.160265	0.626959
C	−6.870230	−0.256355	0.288496
O	1.242594	3.607473	−1.430818
C	2.126951	3.447972	−2.544426
O	3.295442	2.379155	0.041375
C	3.453732	3.637075	0.704733
O	−1.328080	2.529228	−1.302282

C	−1.199498	2.702064	−2.713780
H	−2.380070	1.499397	1.992081
H	−1.466561	−0.373280	−2.561261
H	−0.554039	−1.334574	−1.407930
H	−2.055600	−3.223140	−1.666446
H	−1.570957	−1.997315	−4.425482
H	−0.383764	−2.940938	−3.508937
H	−1.872665	−3.710939	−4.082332
H	−0.476316	−1.657112	1.247083
H	−0.368724	−0.632088	2.666749
H	1.879291	−2.191922	1.334130
H	0.843163	−2.151395	4.205719
H	2.142451	−3.179410	3.571495
H	0.477319	−3.407206	3.010195
H	3.410452	0.936068	2.166778
H	−4.355741	−3.075117	−1.007010
H	−5.303325	−1.822855	−1.810358
H	5.360587	−0.548709	2.378129
H	7.093972	−1.799714	1.109811
H	4.449007	−2.073464	−2.235338
H	2.717312	−0.812751	−0.978650
H	−3.590207	1.215836	3.917691
H	−5.281601	1.743598	4.082676
H	−4.212434	2.555875	2.914041
H	−7.802485	−0.807312	0.397370
H	−6.873224	0.605718	0.957902
H	−6.766305	0.081359	−0.747220
H	3.123221	3.150288	−2.214808
H	2.174381	4.416173	−3.038745
H	1.732641	2.702473	−3.241065
H	2.869429	4.416373	0.211870
H	4.512864	3.880456	0.653204
H	3.148703	3.554740	1.751773
H	−2.212262	2.770526	−3.107478
H	−0.649874	3.612272	−2.952675
H	−0.698241	1.840620	−3.164835

ωB97X Energy = −1828.87979073 a.u.

(*aS*,1*S*,3*S*,3'*S*)-**21**, Conf J

C	−2.822352	0.938287	1.031057
C	−4.104902	0.439523	1.209311
C	−4.487864	−0.708251	0.505539
C	−3.593850	−1.343731	−0.341675
C	−2.295000	−0.853726	−0.504871
C	−1.922109	0.300722	0.179870
C	−1.336901	−1.589661	−1.412991
C	−1.818417	−3.000888	−1.715720
C	−1.029777	−3.663031	−2.822289
O	−3.183734	−2.954623	−2.122903
C	−0.312438	1.890401	−0.870657
C	0.959236	2.462998	−0.995706
C	2.006173	1.953484	−0.232763
C	1.785792	0.913640	0.669912
C	0.511321	0.378949	0.820681
C	−0.545707	0.866702	0.042329
C	0.268228	−0.694482	1.855465
C	1.559424	−1.313331	2.366516
C	1.358292	−2.113210	3.633519
O	2.500927	−0.280960	2.656825
C	2.949683	0.406902	1.494905
C	−4.039354	−2.597630	−1.058201
C	3.942925	−0.447953	0.716503
C	3.648667	−1.036898	−0.507154
C	4.576205	−1.845431	−1.153390
C	5.799114	−2.049319	−0.551584
C	6.132681	−1.480558	0.662552
C	5.192303	−0.679419	1.288885
F	6.711105	−2.832617	−1.175187
O	−5.036102	0.982386	2.035549
C	−4.673873	2.143912	2.767739
O	−5.733231	−1.260990	0.686137
C	−6.797988	−0.521019	0.086714
O	1.215492	3.464832	−1.893216
C	0.547067	4.704272	−1.636705
O	3.286271	2.420675	−0.369333
C	3.508984	3.772626	0.034766
O	−1.359043	2.369092	−1.616796
C	−1.277530	2.103456	−3.018643
H	−2.499509	1.826660	1.558696
H	−1.222655	−1.050421	−2.360441
H	−0.342386	−1.635411	−0.959973
H	−1.752478	−3.607412	−0.799517
H	−1.136225	−3.097022	−3.750679
H	0.029566	−3.703511	−2.561831
H	−1.384471	−4.680480	−2.991943
H	−0.373396	−1.480077	1.447914
H	−0.278142	−0.256098	2.698774
H	1.980323	−1.964810	1.588609
H	0.997700	−1.464250	4.434998
H	2.294852	−2.573593	3.951049
H	0.621463	−2.901859	3.469453
H	3.495251	1.269580	1.882278
H	−4.104812	−3.424818	−0.336745
H	−5.032396	−2.459363	−1.486083
H	2.684631	−0.864949	−0.973670
H	4.357409	−2.308763	−2.107555
H	7.107414	−1.661991	1.098324
H	5.433995	−0.224278	2.243680
H	−5.548739	2.414138	3.353981
H	−4.415595	2.968945	2.098472
H	−3.835039	1.941241	3.439150
H	−6.637134	−0.423466	−0.991309
H	−7.710716	−1.085959	0.266367
H	−6.885398	0.469466	0.536539
H	0.772532	5.056821	−0.626093
H	−0.532380	4.600735	−1.753783
H	0.933056	5.415783	−2.363919
H	4.587713	3.898815	0.104091
H	3.058934	3.958639	1.014170
H	3.107257	4.474986	−0.696239
H	−2.181162	2.515789	−3.462589

H	−0.398422	2.577195	−3.459958
H	−1.243800	1.025987	−3.201136

ωB97X Energy = −1828.87968944 a.u.

(*aS*,1*S*,3*S*,3'*S*)-**21**, Conf K

C	2.776833	0.545621	−1.408630
C	4.093510	0.106688	−1.391091
C	4.588303	−0.503644	−0.233170
C	3.781837	−0.646335	0.885540
C	2.455019	−0.210117	0.864780
C	1.961181	0.391118	−0.289882
C	1.591724	−0.404639	2.088012
C	2.201400	−1.408012	3.055645
C	1.508412	−1.417274	4.398945
O	3.571182	−1.079001	3.274185
C	0.210727	2.118047	0.137965
C	−1.103055	2.591027	0.044073
C	−2.070461	1.784046	−0.552242
C	−1.753967	0.511781	−1.018306
C	−0.443909	0.050806	−0.931804
C	0.544610	0.861058	−0.364986
C	−0.094905	−1.313755	−1.477760
C	−1.327817	−2.160053	−1.752582
C	−1.023614	−3.366481	−2.611431
O	−2.300051	−1.375074	−2.442397
C	−2.845046	−0.335385	−1.638087
C	4.358670	−1.295950	2.122415
C	−3.840243	−0.902092	−0.631371
C	−5.039141	−1.420366	−1.117490
C	−5.972812	−1.989602	−0.267205
C	−5.684617	−2.032036	1.083432
C	−4.512593	−1.530041	1.606153
C	−3.590686	−0.963102	0.734260
F	−6.591355	−2.583717	1.924626
O	4.962243	0.227927	−2.428144
C	4.491937	0.849440	−3.614765
O	5.897621	−0.916819	−0.166479
C	6.172407	−2.113995	−0.894338
O	−1.390401	3.865520	0.455567
C	−2.295343	3.967907	1.559283
O	−3.366497	2.221143	−0.673164
C	−3.554119	3.259212	−1.639972
O	1.218886	2.881437	0.652349
C	1.037457	3.445000	1.951697
H	2.366447	1.018889	−2.291714
H	1.460888	0.551569	2.608692
H	0.593097	−0.741836	1.794771
H	2.155292	−2.412287	2.607679
H	1.603883	−0.439305	4.876591
H	0.446724	−1.638748	4.274945
H	1.946154	−2.170273	5.055601
H	0.561350	−1.841886	−0.780550
H	0.471564	−1.194878	−2.408419
H	−1.758722	−2.493083	−0.798724
H	−0.641918	−3.049746	−3.584917
H	−1.922587	−3.964771	−2.765910
H	−0.268519	−3.991266	−2.130641
H	−3.407989	0.284769	−2.338548
H	4.477971	−2.375858	1.949350
H	5.346952	−0.887874	2.332525
H	−5.246031	−1.378044	−2.181785
H	−6.908478	−2.391568	−0.635941
H	−4.327464	−1.582492	2.671963
H	−2.666996	−0.560118	1.135213
H	3.656279	0.293800	−4.049045
H	5.328512	0.843898	−4.309181
H	4.184527	1.881357	−3.424820
H	7.223601	−2.343915	−0.731071
H	5.557914	−2.939342	−0.521966
H	5.990040	−1.970879	−1.961181
H	−3.263215	3.528142	1.315194
H	−2.411827	5.030660	1.761659
H	−1.878577	3.472957	2.441002
H	−3.014700	4.163773	−1.352656
H	−4.622859	3.460119	−1.673115
H	−3.214959	2.926097	−2.625008
H	2.034726	3.595259	2.362294
H	0.512240	4.398316	1.899328
H	0.484838	2.757719	2.598886

ωB97X Energy = −1828.87947933 a.u.

(*aS*,1*S*,3*S*,3'*S*)-**21**, Conf L

C	−2.854912	1.051484	0.895040
C	−4.161993	0.597616	0.999078
C	−4.549813	−0.522746	0.255393
C	−3.651344	−1.146689	−0.595766
C	−2.334932	−0.688593	−0.702124
C	−1.945306	0.416482	0.051795
C	−1.374379	−1.416093	−1.614202
C	−1.883914	−2.804079	−1.972515
C	−1.077080	−3.459389	−3.070019
O	−3.232063	−2.704835	−2.424375
C	−0.226203	2.000529	−0.824054
C	1.071600	2.525374	−0.855009
C	2.060550	1.926595	−0.079220
C	1.757483	0.837936	0.738516
C	0.458299	0.346151	0.792878
C	−0.542137	0.929119	0.004838
C	0.128310	−0.788694	1.733693
C	1.372279	−1.501283	2.237393
C	1.088474	−2.403907	3.416620
O	2.334886	−0.535098	2.656956
C	2.861557	0.231499	1.579536
C	−4.116285	−2.350671	−1.382742
C	3.874198	−0.585363	0.786245
C	5.102670	−0.861314	1.383295
C	6.056852	−1.633525	0.741360
C	5.757948	−2.128043	−0.513492
C	4.556306	−1.878590	−1.140710
C	3.615000	−1.099613	−0.478594

F	6.683576	−2.882181	−1.152773
O	−5.117328	1.165467	1.779473
C	−4.753271	2.303307	2.547123
O	−5.840913	−0.989758	0.314133
C	−6.161946	−1.674652	1.525615
O	1.408871	3.566542	−1.677882
C	0.778695	4.816532	−1.378478
O	3.362659	2.347099	−0.123438
C	3.616261	3.671214	0.349079
O	−1.215428	2.571182	−1.584061
C	−1.091701	2.359861	−2.991870
H	−2.523796	1.910301	1.464838
H	−1.229801	−0.850461	−2.542011
H	−0.390515	−1.499909	−1.144037
H	−1.866855	−3.437331	−1.072085
H	−1.129754	−2.862919	−3.983810
H	−0.030241	−3.544358	−2.772439
H	−1.459274	−4.458506	−3.283352
H	−0.529921	−1.511314	1.244624
H	−0.429655	−0.391404	2.589644
H	1.803560	−2.096137	1.420772
H	0.711758	−1.818165	4.258353
H	1.994927	−2.923437	3.730280
H	0.336434	−3.148496	3.149045
H	3.406330	1.044343	2.063860
H	−4.249622	−3.202654	−0.699491
H	−5.082679	−2.148765	−1.843862
H	5.317882	−0.463169	2.369451
H	7.016244	−1.847951	1.195794
H	4.364778	−2.284311	−2.126450
H	2.668664	−0.890124	−0.965611
H	−5.649060	2.600348	3.086901
H	−4.426930	3.125164	1.904190
H	−3.961702	2.061203	3.261450
H	−5.486879	−2.522449	1.676926
H	−6.102764	−0.999108	2.381100
H	−7.181365	−2.039956	1.416830
H	−0.298610	4.762005	−1.538891
H	1.218388	5.549099	−2.052320
H	0.979858	5.105533	−0.342862
H	4.694065	3.749796	0.477532
H	3.123879	3.835072	1.311920
H	3.280301	4.418163	−0.370502
H	−0.173890	2.807122	−3.379004
H	−1.103067	1.290296	−3.218766
H	−1.955931	2.834593	−3.451711

ωB97X Energy = −1828.87934419 a.u.

(*aR*,1*S*,3*S*,3’*S*)-**21**, Conf A

C	−2.030320	−1.044059	−1.384732
C	−3.270012	−1.656111	−1.507664
C	−4.301582	−1.274423	−0.642228
C	−4.091787	−0.287051	0.308009
C	−2.851668	0.347724	0.413265
C	−1.819629	−0.048798	−0.432374
C	−2.658711	1.440285	1.437994
C	−3.987520	1.952209	1.975505
C	−3.824457	2.828311	3.196334
O	−4.809663	0.845139	2.339467
C	−0.152637	1.684136	−1.091465
C	1.107355	2.281509	−1.008766
C	2.068171	1.730229	−0.167046
C	1.763529	0.617763	0.617366
C	0.500789	0.038765	0.544525
C	−0.462069	0.565975	−0.325715
C	0.168601	−1.142079	1.425828
C	1.407740	−1.785426	2.026819
C	1.079289	−2.727860	3.162668
O	2.268596	−0.773246	2.547375
C	2.827793	0.067793	1.544474
C	−5.236295	0.113350	1.209692
C	3.966074	−0.635920	0.816735
C	5.212117	−0.701488	1.435905
C	6.276098	−1.361828	0.841979
C	6.069139	−1.961099	−0.385163
C	4.852481	−1.922475	−1.032303
C	3.801866	−1.251126	−0.420085
F	7.102414	−2.605566	−0.976479
O	−3.570426	−2.612522	−2.423854
C	−2.546738	−3.009571	−3.323725
O	−5.555103	−1.826110	−0.756546
C	−5.649676	−3.175751	−0.298185
O	1.378051	3.391748	−1.764929
C	1.308120	4.615272	−1.029066
O	3.298307	2.316076	−0.018814
C	4.139976	2.306462	−1.176566
O	−1.118407	2.258319	−1.876089
C	−0.965322	2.007205	−3.273067
H	−1.210708	−1.330343	−2.031784
H	−2.058464	1.067540	2.277055
H	−2.102466	2.271998	0.997216
H	−4.502375	2.515138	1.182292
H	−3.364689	2.260426	4.008548
H	−3.183237	3.681242	2.965952
H	−4.791166	3.202720	3.535762
H	−0.389633	−1.889839	0.857588
H	−0.490316	−0.809648	2.237192
H	1.944364	−2.336987	1.243473
H	0.596742	−2.182728	3.977312
H	1.985023	−3.199318	3.546396
H	0.398973	−3.509509	2.819432
H	3.261721	0.901925	2.098219
H	−5.965680	0.702331	0.635105
H	−5.753171	−0.770648	1.583472
H	5.356004	−0.222777	2.399003
H	7.250056	−1.411506	1.312971
H	4.733856	−2.402832	−1.995654
H	2.844624	−1.201169	−0.927536
H	−2.989770	−3.763759	−3.969423
H	−1.696036	−3.442388	−2.790330

H	−2.206391	−2.166018	−3.930839
H	−6.690232	−3.472115	−0.416161
H	−5.011052	−3.833593	−0.890263
H	−5.370673	−3.242441	0.757931
H	0.307788	4.750479	−0.609073
H	1.518173	5.415922	−1.735376
H	2.051408	4.626263	−0.228121
H	3.688075	2.869800	−1.993651
H	4.334808	1.278451	−1.493842
H	5.074938	2.774863	−0.875930
H	−1.777833	2.529348	−3.774673
H	−0.006905	2.387654	−3.632948
H	−1.042634	0.934844	−3.477555

ωB97X Energy = −1828.88182768 a.u.

(*aR*,1*S*,3*S*,3'*S*)-**21**, Conf B

C	−2.022228	−1.257989	−1.176186
C	−3.249086	−1.906272	−1.211477
C	−4.270349	−1.467722	−0.361356
C	−4.050794	−0.419564	0.519108
C	−2.818059	0.237118	0.549551
C	−1.805840	−0.193006	−0.303898
C	−2.618279	1.399324	1.493078
C	−3.943043	1.927966	2.023782
C	−3.767404	2.897141	3.170265
O	−4.734051	0.837360	2.490224
C	−0.220015	1.529105	−1.154719
C	1.021588	2.168898	−1.167337
C	2.029693	1.714092	−0.323873
C	1.791074	0.656693	0.554190
C	0.546537	0.035364	0.574928
C	−0.464404	0.464773	−0.294517
C	0.286782	−1.083090	1.556356
C	1.567789	−1.636401	2.159850
C	1.310113	−2.495997	3.376696
O	2.408100	−0.557847	2.568218
C	2.904399	0.216978	1.482629
C	−5.174766	0.013432	1.432037
C	4.043414	−0.505226	0.774461
C	5.306099	−0.490394	1.362699
C	6.374680	−1.159984	0.787893
C	6.155429	−1.849799	−0.388576
C	4.922144	−1.893488	−1.003033
C	3.866695	−1.211728	−0.410591
F	7.193173	−2.503504	−0.961776
O	−3.543839	−2.958725	−2.017252
C	−2.529911	−3.422467	−2.896046
O	−5.487702	−2.105425	−0.344466
C	−6.314805	−1.818098	−1.472492
O	1.228368	3.227596	−2.012711
C	1.105785	4.498841	−1.370791
O	3.243130	2.348557	−0.266657
C	4.043077	2.276587	−1.451373
O	−1.232088	2.012347	−1.942493
C	−1.116584	1.669395	−3.323494
H	−1.213451	−1.577675	−1.821311
H	−1.995070	1.093257	2.342340
H	−2.084169	2.206264	0.984130
H	−4.486407	2.420787	1.203247
H	−3.279343	2.400180	4.011966
H	−3.146921	3.740164	2.860497
H	−4.732584	3.280771	3.503735
H	−0.263197	−1.891335	1.068415
H	−0.355586	−0.710246	2.363349
H	2.097724	−2.230138	1.403263
H	0.834549	−1.904258	4.162325
H	2.243897	−2.905391	3.764463
H	0.647777	−3.324785	3.119443
H	3.326188	1.106450	1.953473
H	−5.943653	0.537687	0.845121
H	−5.647056	−0.858088	1.884516
H	5.458845	0.057628	2.286689
H	7.361112	−1.147614	1.234768
H	4.794640	−2.443186	−1.927415
H	2.895439	−1.227000	−0.893051
H	−2.228473	−2.641402	−3.599654
H	−2.965299	−4.254507	−3.443884
H	−1.654864	−3.771677	−2.341154
H	−5.854798	−2.179871	−2.394083
H	−7.258639	−2.334427	−1.307871
H	−6.498582	−0.742195	−1.549758
H	1.856048	4.606264	−0.583447
H	0.104605	4.617257	−0.947736
H	1.268878	5.253606	−2.137494
H	4.253967	1.233357	−1.701683
H	4.975522	2.787452	−1.219974
H	3.547252	2.768815	−2.288681
H	−0.182096	2.050210	−3.741003
H	−1.168687	0.583740	−3.451884
H	−1.959870	2.133637	−3.831150

ωB97X Energy = −1828.88139827 a.u.

(*aR*,1*S*,3*S*,3'*S*)-**21**, Conf C

C	2.075062	−0.960628	1.494490
C	3.326142	−1.546787	1.615111
C	4.316905	−1.226180	0.678860
C	4.053295	−0.329723	−0.343965
C	2.795642	0.271433	−0.458229
C	1.807897	−0.057273	0.467105
C	2.546681	1.249260	−1.583160
C	3.847803	1.762592	−2.181530
C	3.633806	2.537882	−3.461326
O	4.694258	0.654664	−2.479907
C	0.122129	1.643428	1.164210
C	−1.138779	2.237199	1.080447
C	−2.081427	1.715287	0.201835
C	−1.785098	0.591732	−0.567234
C	−0.526157	0.004148	−0.482030
C	0.442103	0.541909	0.376255
C	−0.205708	−1.197379	−1.339970

C	−1.452333	−1.837341	−1.929330
C	−1.135352	−2.809854	−3.042617
O	−2.297236	−0.823378	−2.473533
C	−2.854410	0.031219	−1.481391
C	5.162686	0.016870	−1.310466
C	−3.989887	−0.669348	−0.744335
C	−5.197284	−0.853251	−1.414705
C	−6.255217	−1.521412	−0.819577
C	−6.081931	−2.007202	0.461970
C	−4.905232	−1.846467	1.161232
C	−3.859075	−1.170627	0.545654
F	−7.109933	−2.659800	1.054611
O	3.680282	−2.417671	2.594849
C	2.699835	−2.755714	3.564641
O	5.583744	−1.746312	0.794324
C	5.689505	−3.122811	0.428090
O	−1.431525	3.333033	1.849192
C	−2.086535	3.004599	3.076164
O	−3.338398	2.256641	0.125584
C	−3.392899	3.552618	−0.478559
O	1.024108	2.120051	2.080154
C	1.685361	3.323632	1.690476
H	1.291058	−1.187065	2.205711
H	1.967401	0.767659	−2.379978
H	1.948245	2.093353	−1.230956
H	4.359207	2.399041	−1.443211
H	3.175920	1.895701	−4.217288
H	2.971554	3.387060	−3.282638
H	4.581760	2.912730	−3.849518
H	0.343225	−1.939366	−0.754896
H	0.456023	−0.892666	−2.159032
H	−1.999083	−2.362183	−1.134599
H	−0.643972	−2.290588	−3.868852
H	−2.047067	−3.277337	−3.416950
H	−0.466572	−3.592903	−2.680406
H	−3.292107	0.857299	−2.045650
H	5.897731	0.658346	−0.803252
H	5.684656	−0.884621	−1.631635
H	−5.314777	−0.463417	−2.420570
H	−7.199622	−1.663061	−1.330473
H	−4.811848	−2.241352	2.165387
H	−2.931527	−1.031096	1.090076
H	2.371268	−1.871907	4.118099
H	3.180469	−3.451868	4.247552
H	1.835330	−3.239212	3.101646
H	6.735796	−3.397334	0.547937
H	5.069989	−3.746928	1.074523
H	5.393803	−3.265446	−0.615912
H	−1.446924	2.366154	3.691334
H	−3.037195	2.501269	2.881605
H	−2.269967	3.943938	3.593960
H	−2.994804	3.512798	−1.496642
H	−2.831821	4.279187	0.110874
H	−4.443616	3.833155	−0.510963
H	2.320814	3.147095	0.817100
H	0.961149	4.110987	1.470999
H	2.307862	3.623677	2.531111

ωB97X Energy = −1828.88062572 a.u.

(*aR*,1*S*,3*S*,3’*S*)-**21**, Conf D

C	2.100263	−0.506641	1.679175
C	3.358747	−1.016478	1.961603
C	4.350841	−0.954527	0.975399
C	4.080190	−0.386278	−0.258696
C	2.815313	0.140606	−0.539208
C	1.826858	0.070276	0.440034
C	2.560585	0.758609	−1.894925
C	3.857154	1.093621	−2.617021
C	3.636587	1.465170	−4.065604
O	4.718490	−0.041694	−2.585260
C	0.123771	1.878380	0.639916
C	−1.143266	2.423282	0.398342
C	−2.072349	1.676738	−0.319758
C	−1.751440	0.394566	−0.770531
C	−0.496668	−0.144715	−0.515226
C	0.455825	0.607386	0.185727
C	−0.169326	−1.535951	−1.003532
C	−1.414836	−2.315929	−1.390992
C	−1.095752	−3.572768	−2.168531
O	−2.244472	−1.500199	−2.217182
C	−2.803757	−0.383443	−1.534562
C	5.189923	−0.310740	−1.282099
C	−3.988823	−0.809712	−0.677565
C	−3.885535	−1.035015	0.691096
C	−4.977758	−1.471232	1.430263
C	−6.173582	−1.671691	0.774346
C	−6.320708	−1.456693	−0.582098
C	−5.216047	−1.026206	−1.300071
F	−7.246456	−2.089404	1.486913
O	3.718923	−1.571582	3.147466
C	2.733453	−1.649530	4.166652
O	5.625120	−1.399807	1.233417
C	5.753600	−2.821605	1.282099
O	−1.452356	3.701393	0.781213
C	−1.555132	3.908325	2.193756
O	−3.295137	2.177511	−0.672743
C	−4.172708	2.574270	0.381893
O	1.039004	2.605413	1.360384
C	1.676636	3.652834	0.628235
H	1.315581	−0.535646	2.424279
H	1.985647	0.066884	−2.521899
H	1.954345	1.662619	−1.796192
H	4.358940	1.922551	−2.094314
H	3.183382	0.628758	−4.602758
H	2.968325	2.325236	−4.138391
H	4.582138	1.717888	−4.547124
H	0.379152	−2.082335	−0.232202
H	0.492276	−1.474155	−1.875166
H	−1.972291	−2.584011	−0.483548
H	−0.586045	−3.320799	−3.101415
H	−2.009219	−4.119707	−2.406094

H	−0.443768	−4.223184	−1.582446
H	−3.190353	0.256022	−2.329822
H	5.912068	0.460684	−0.978090
H	5.727335	−1.257854	−1.331400
H	−2.943489	−0.860070	1.199432
H	−4.906183	−1.648470	2.496275
H	−7.280280	−1.619869	−1.056995
H	−5.313284	−0.850572	−2.366439
H	1.884051	−2.260467	3.849096
H	2.381250	−0.654670	4.452355
H	3.218593	−2.119441	5.018595
H	6.806304	−3.033067	1.459681
H	5.153237	−3.239581	2.091987
H	5.449141	−3.266411	0.329712
H	−0.594600	3.744911	2.683213
H	−2.306357	3.238964	2.623177
H	−1.870019	4.941048	2.329986
H	−4.185334	1.820887	1.174534
H	−5.165272	2.644812	−0.059570
H	−3.884208	3.542277	0.791481
H	0.939598	4.343096	0.211729
H	2.314644	4.181356	1.333747
H	2.293867	3.239197	−0.174715

ωB97X Energy = −1828.88046454 a.u.

(*aR*,1*S*,3*S*,3'*S*)-**21**, Conf E

C	2.069998	−1.015994	1.368115
C	3.320990	−1.607519	1.475772
C	4.336908	−1.204682	0.601458
C	4.100271	−0.217822	−0.342925
C	2.848797	0.396670	−0.432458
C	1.833008	−0.019514	0.423203
C	2.625252	1.487268	−1.453058
C	3.938001	2.019553	−2.010141
C	3.742442	2.889142	−3.230848
O	4.773645	0.925596	−2.382743
C	0.147433	1.684680	1.108388
C	−1.127785	2.256763	1.053474
C	−2.066588	1.724282	0.173161
C	−1.763658	0.601582	−0.595222
C	−0.500692	0.024758	−0.518824
C	0.464652	0.574264	0.333631
C	−0.165826	−1.161864	−1.391279
C	−1.404108	−1.803582	−1.995951
C	−1.073909	−2.751453	−3.126589
O	−2.259588	−0.789740	−2.523395
C	−2.826221	0.044506	−1.519475
C	5.227037	0.203660	−1.257179
C	−3.956198	−0.675455	−0.793168
C	−5.163252	−0.857287	−1.464829
C	−6.215776	−1.541712	−0.878748
C	−6.037515	−2.045867	0.395039
C	−4.861099	−1.887816	1.095338
C	−3.820369	−1.195471	0.488916
F	−7.060278	−2.714420	0.978811
O	3.646948	−2.562372	2.384805
C	2.639728	−2.980226	3.293797
O	5.600763	−1.735360	0.700524
C	5.713557	−3.081738	0.236663
O	−1.385239	3.363259	1.816004
C	−2.426676	3.215427	2.785770
O	−3.320597	2.270161	0.060511
C	−3.352614	3.572566	−0.531546
O	1.117994	2.261396	1.885601
C	1.025884	1.967894	3.277600
H	1.261923	−1.319779	2.021699
H	2.018053	1.105810	−2.283180
H	2.063234	2.310321	−1.003612
H	4.454332	2.593380	−1.225802
H	3.281559	2.310626	−4.034905
H	3.089319	3.731118	−2.993781
H	4.697419	3.279653	−3.584970
H	0.385721	−1.908741	−0.815259
H	0.499999	−0.836756	−2.199962
H	−1.946826	−2.349010	−1.212295
H	−0.587087	−2.210942	−3.941789
H	−1.979352	−3.222451	−3.511591
H	−0.396613	−3.533222	−2.777687
H	−3.270285	0.874525	−2.072439
H	5.951164	0.807335	−0.691252
H	5.756677	−0.670703	−1.635656
H	−5.284663	−0.452773	−2.464401
H	−7.159878	−1.681996	−1.390573
H	−4.763823	−2.297460	2.093182
H	−2.892998	−1.057977	1.034123
H	3.102406	−3.729478	3.931410
H	1.790721	−3.425203	2.767742
H	2.291930	−2.144994	3.908165
H	6.759539	−3.361709	0.346307
H	5.089707	−3.751638	0.830897
H	5.428107	−3.149202	−0.817675
H	−3.399267	3.089794	2.307870
H	−2.420229	4.126286	3.380845
H	−2.226883	2.356096	3.432466
H	−4.402369	3.844021	−0.619939
H	−2.896404	3.548194	−1.524996
H	−2.831231	4.300755	0.092901
H	1.092038	0.889030	3.448031
H	1.869734	2.460810	3.756417
H	0.095364	2.352461	3.701468

ωB97X Energy = −1828.88042503 a.u.

(*aR*,1*S*,3*S*,3'*S*)-**21**, Conf F

C	−2.056906	−0.988761	−1.511921
C	−3.302334	−1.585048	−1.640540
C	−4.305601	−1.262915	−0.718197
C	−4.059412	−0.355689	0.299344
C	−2.806584	0.253921	0.423208
C	−1.806776	−0.075052	−0.489346
C	−2.578282	1.240466	1.545068

C	−3.890760	1.759099	2.113125
C	−3.701483	2.554218	3.384635
O	−4.738325	0.652598	2.413599
C	−0.122120	1.627258	−1.181870
C	1.128916	2.245666	−1.077512
C	2.082702	1.698311	−0.222881
C	1.770683	0.594592	0.571227
C	0.509745	0.013844	0.497047
C	−0.445034	0.532097	−0.387736
C	0.179056	−1.169168	1.376378
C	1.419106	−1.811699	1.976312
C	1.091676	−2.757685	3.109480
O	2.275520	−0.797491	2.500504
C	2.835085	0.044995	1.499158
C	−5.184233	−0.005683	1.246727
C	3.974241	−0.659067	0.773123
C	5.221401	−0.717514	1.390612
C	6.286089	−1.378917	0.799087
C	6.078521	−1.986761	−0.423712
C	4.860584	−1.955648	−1.068872
C	3.809355	−1.282917	−0.459247
F	7.112461	−2.632392	−1.012614
O	−3.639477	−2.467635	−2.615728
C	−2.644847	−2.810063	−3.569422
O	−5.567575	−1.792485	−0.843015
C	−5.668386	−3.166224	−0.465152
O	1.386995	3.335184	−1.864623
C	1.666411	4.548899	−1.161376
O	3.328785	2.255800	−0.090048
C	4.155370	2.223293	−1.257847
O	−1.019692	2.085056	−2.112214
C	−1.774597	3.226157	−1.714716
H	−1.263879	−1.215568	−2.212926
H	−2.017581	0.763899	2.357854
H	−1.970526	2.080751	1.199983
H	−4.391578	2.382851	1.356790
H	−3.253181	1.925758	4.157676
H	−3.040506	3.404027	3.204241
H	−4.657545	2.930010	3.751463
H	−0.378234	−1.915733	0.805293
H	−0.477401	−0.843773	2.191564
H	1.958513	−2.360045	1.192877
H	0.606415	−2.215964	3.924815
H	1.998209	−3.227525	3.493310
H	0.414110	−3.540450	2.763352
H	3.268157	0.878992	2.053800
H	−5.917707	0.621750	0.720078
H	−5.702786	−0.907207	1.573338
H	5.365719	−0.232414	2.350446
H	7.260958	−1.423008	1.268752
H	4.741457	−2.442363	−2.028958
H	2.851252	−1.238416	−0.965689
H	−2.314943	−1.930379	−4.128584
H	−3.112302	−3.516930	−4.250483
H	−1.783164	−3.282690	−3.090201
H	−5.040188	−3.792482	−1.101061
H	−5.380293	−3.297845	0.582420
H	−6.712103	−3.447890	−0.590782
H	2.621619	4.493747	−0.636655
H	0.871937	4.766168	−0.441700
H	1.700959	5.335112	−1.912564
H	5.111376	2.652155	−0.964670
H	3.719174	2.809856	−2.068177
H	4.305102	1.192314	−1.588108
H	−1.119610	4.060804	−1.453146
H	−2.390225	3.506037	−2.567332
H	−2.422707	2.985731	−0.866385

ωB97X Energy = −1828.88029870 a.u.

(*aR*,1*S*,3*S*,3'*S*)-**21**, Conf G

C	−2.092928	−0.319613	−1.712051
C	−3.334316	−0.825407	−2.067725
C	−4.310564	−0.982377	−1.076523
C	−4.028404	−0.669453	0.243417
C	−2.774738	−0.164649	0.603751
C	−1.811232	0.010669	−0.387539
C	−2.511058	0.179147	2.051833
C	−3.805725	0.328388	2.836428
C	−3.577198	0.437150	4.326679
O	−4.625443	−0.814287	2.603973
C	−0.181323	1.890441	−0.265054
C	1.066810	2.433703	0.062555
C	2.038267	1.604114	0.613655
C	1.775822	0.247818	0.817751
C	0.538361	−0.286306	0.480316
C	−0.456121	0.544581	−0.054137
C	0.275282	−1.757491	0.700990
C	1.556340	−2.543067	0.929633
C	1.299948	−3.931602	1.470303
O	2.368039	−1.858336	1.882589
C	2.871774	−0.614096	1.410188
C	−5.112476	−0.859067	1.279889
C	4.053942	−0.827500	0.473385
C	5.301198	−1.100870	1.030064
C	6.406844	−1.347684	0.231523
C	6.240326	−1.319582	−1.139266
C	5.024354	−1.054192	−1.732255
C	3.931261	−0.804933	−0.911903
F	7.313642	−1.556667	−1.929906
O	−3.688139	−1.191199	−3.326621
C	−2.720774	−1.041150	−4.355073
O	−5.544312	−1.502770	−1.384637
C	−6.397985	−0.608125	−2.099170
O	1.312892	3.774552	−0.066825
C	1.384373	4.257835	−1.412158
O	3.247762	2.078186	1.040937
C	4.085575	2.711459	0.073354
O	−1.134414	2.706071	−0.823704
C	−1.842796	3.517741	0.113227
H	−1.323076	−0.175914	−2.459356
H	−1.911344	−0.607256	2.524950

H	−1.929224	1.101335	2.126948
H	−4.344134	1.219538	2.478632
H	−3.089118	−0.466311	4.699597
H	−2.937592	1.292922	4.550580
H	−4.524272	0.564487	4.852607
H	−0.259381	−2.173916	−0.156301
H	−0.377240	−1.888190	1.571752
H	2.108827	−2.620203	−0.016308
H	0.800609	−3.871982	2.440130
H	2.238097	−4.474463	1.593287
H	0.660780	−4.492510	0.785899
H	3.251413	−0.117033	2.304368
H	−5.887496	−0.090521	1.140795
H	−5.589632	−1.830122	1.150707
H	5.413077	−1.117328	2.109301
H	7.381574	−1.552992	0.656572
H	4.938113	−1.038526	−2.811743
H	2.972660	−0.582102	−1.367966
H	−2.432159	0.006433	−4.476500
H	−3.195706	−1.393395	−5.267405
H	−1.831620	−1.644309	−4.152465
H	−7.348667	−1.121405	−2.230269
H	−6.557574	0.310152	−1.525907
H	−5.974986	−0.363932	−3.075326
H	2.176727	3.740874	−1.961451
H	1.622941	5.317366	−1.343583
H	0.432062	4.123681	−1.925598
H	4.097711	2.138816	−0.858446
H	5.087434	2.721915	0.499140
H	3.759510	3.733273	−0.121079
H	−1.151951	4.112685	0.715222
H	−2.482083	4.178546	−0.468933
H	−2.465166	2.897375	0.765084

ωB97X Energy = −1828.88028885 a.u.

(*aR*,1*S*,3*S*,3'*S*)-**21**, Conf H

C	2.004527	−1.118132	1.338348
C	3.242179	−1.740689	1.422558
C	4.261681	−1.339062	0.551858
C	4.042058	−0.323743	−0.366014
C	2.804272	0.320844	−0.431996
C	1.784448	−0.093093	0.420232
C	2.599604	1.440248	−1.425151
C	3.921363	1.958461	−1.973954
C	3.740828	2.865541	−3.169417
O	4.730872	0.856837	−2.379774
C	0.139351	1.626641	1.161586
C	−1.115302	2.244905	1.118035
C	−2.090348	1.732216	0.265614
C	−1.812886	0.623126	−0.536491
C	−0.556574	0.029455	−0.502123
C	0.430094	0.534556	0.353666
C	−0.251710	−1.133935	−1.416192
C	−1.506647	−1.743764	−2.019547
C	−1.204844	−2.653586	−3.188777
O	−2.362377	−0.705022	−2.493830
C	−2.901116	0.096603	−1.448302
C	5.173111	0.094834	−1.276351
C	−4.013926	−0.647240	−0.719186
C	−3.856814	−1.188682	0.551309
C	−4.883966	−1.899801	1.159352
C	−6.068547	−2.054943	0.472362
C	−6.267824	−1.529980	−0.789892
C	−5.228676	−0.826760	−1.377549
F	−7.078124	−2.742060	1.057836
O	3.551960	−2.726022	2.304270
C	2.540730	−3.147133	3.207319
O	5.513335	−1.901554	0.628790
C	5.591385	−3.236361	0.125869
O	−1.425698	3.283188	1.955445
C	−0.720230	4.504440	1.711536
O	−3.358612	2.237847	0.213121
C	−3.504721	3.630573	−0.070931
O	1.110707	2.129977	1.990687
C	0.946277	1.784184	3.367015
H	1.193576	−1.419848	1.989345
H	1.981737	1.091536	−2.261846
H	2.056025	2.263726	−0.954168
H	4.453175	2.499156	−1.176403
H	3.264262	2.320034	−3.987300
H	3.107738	3.715144	−2.906537
H	4.703093	3.244267	−3.516576
H	0.301252	−1.904227	−0.873338
H	0.404108	−0.790578	−2.225590
H	−2.039533	−2.312350	−1.245690
H	−0.728488	−2.088274	−3.993258
H	−2.120905	−3.103995	−3.573392
H	−0.527734	−3.452285	−2.880106
H	−3.357139	0.945866	−1.960416
H	5.914856	0.666026	−0.699615
H	5.679548	−0.781436	−1.681212
H	−2.923035	−1.053486	1.086073
H	−4.770138	−2.325778	2.148572
H	−7.217454	−1.669077	−1.291731
H	−5.366375	−0.406048	−2.368264
H	2.215505	−2.322750	3.848155
H	2.990077	−3.924938	3.819784
H	1.678829	−3.557444	2.674166
H	5.295833	−3.266949	−0.927416
H	6.631355	−3.543620	0.218386
H	4.956833	−3.908440	0.706172
H	−1.108978	5.229565	2.423780
H	−0.908984	4.857108	0.693457
H	0.351489	4.372600	1.862503
H	−3.347171	4.233914	0.822563
H	−4.524440	3.762712	−0.427885
H	−2.806425	3.938026	−0.854293
H	1.019674	0.700599	3.498778
H	1.756856	2.268896	3.907673
H	−0.014057	2.140529	3.746715

ωB97X Energy = −1828.88028143 a.u.

(*aR*,1*S*,3*S*,3'*S*)-**21**, Conf I

C	−2.028372	−1.234497	−1.252411
C	−3.255978	−1.878282	−1.290363
C	−4.258536	−1.475833	−0.399759
C	−4.014663	−0.475626	0.527547
C	−2.774582	0.169950	0.569944
C	−1.786413	−0.215516	−0.332480
C	−2.550668	1.263376	1.588418
C	−3.866169	1.799336	2.133303
C	−3.676889	2.720467	3.316561
O	−4.675332	0.705075	2.557329
C	−0.195447	1.470637	−1.244583
C	1.044754	2.111649	−1.271478
C	2.035260	1.714175	−0.379753
C	1.808591	0.665510	0.508873
C	0.570066	0.030448	0.533235
C	−0.445523	0.444026	−0.338288
C	0.323609	−1.083165	1.523968
C	1.611182	−1.614692	2.133677
C	1.363658	−2.465530	3.358522
O	2.437555	−0.520292	2.530958
C	2.930410	0.232539	1.429226
C	−5.125821	−0.076740	1.471779
C	4.052435	−0.520580	0.722355
C	5.266685	−0.668882	1.389788
C	6.315339	−1.376285	0.824887
C	6.126581	−1.936865	−0.423727
C	4.942934	−1.813936	−1.118394
C	3.905356	−1.098921	−0.532830
F	7.145932	−2.628238	−0.986769
O	−3.572882	−2.889916	−2.138704
C	−2.576314	−3.323925	−3.052157
O	−5.480678	−2.104191	−0.391946
C	−6.320058	−1.767533	−1.497320
O	1.264734	3.129461	−2.161646
C	2.012725	2.729246	−3.312384
O	3.274240	2.300966	−0.406634
C	3.305175	3.652547	0.061808
O	−1.148717	1.816892	−2.166691
C	−1.816149	3.049511	−1.896819
H	−1.239320	−1.514819	−1.938538
H	−1.959210	0.880732	2.428742
H	−1.975197	2.082816	1.150231
H	−4.398201	2.332708	1.330367
H	−3.194569	2.184964	4.137654
H	−3.046587	3.567511	3.039236
H	−4.637111	3.102358	3.665867
H	−0.215608	−1.902037	1.041357
H	−0.322577	−0.714478	2.329122
H	2.150343	−2.207158	1.382501
H	0.879924	−1.872557	4.138257
H	2.302350	−2.859321	3.750632
H	0.712172	−3.305218	3.108933
H	3.369566	1.124136	1.881402
H	−5.899491	0.470458	0.912705
H	−5.595896	−0.964034	1.894851
H	5.395459	−0.221334	2.369851
H	7.264297	−1.492243	1.333820
H	4.837011	−2.268093	−2.095850
H	2.971427	−0.991767	−1.073443
H	−3.024794	−4.133462	−3.622721
H	−1.693337	−3.696536	−2.525875
H	−2.283981	−2.518694	−3.731564
H	−6.500231	−0.688642	−1.527504
H	−5.872450	−2.092585	−2.438429
H	−7.263975	−2.286627	−1.342333
H	1.475613	1.952921	−3.863860
H	3.000270	2.362709	−3.021525
H	2.119533	3.612978	−3.938181
H	2.950971	3.704189	1.095441
H	2.694364	4.297947	−0.571159
H	4.345144	3.969336	0.018720
H	−2.499986	3.221548	−2.725673
H	−2.388487	2.980393	−0.966513
H	−1.101253	3.872404	−1.833787

ωB97X Energy = −1828.88027260 a.u.

(*aR*,1*S*,3*S*,3'*S*)-**21**, Conf J

C	2.056698	−1.288579	1.103236
C	3.294469	−1.916831	1.096871
C	4.301710	−1.418553	0.262972
C	4.057462	−0.331321	−0.561871
C	2.814338	0.305649	−0.549027
C	1.816384	−0.183428	0.289351
C	2.586182	1.510088	−1.431303
C	3.896158	2.083173	−1.952393
C	3.691722	3.100797	−3.051231
O	4.698511	1.028451	−2.477576
C	0.219099	1.482571	1.224634
C	−1.031476	2.106955	1.278610
C	−2.019298	1.709376	0.381286
C	−1.789176	0.666331	−0.514108
C	−0.548621	0.038861	−0.550127
C	0.465917	0.455577	0.320084
C	−0.291801	−1.054303	−1.560366
C	−1.574102	−1.576702	−2.188544
C	−1.317502	−2.398500	−3.431244
O	−2.403728	−0.477427	−2.564765
C	−2.904853	0.249821	−1.449751
C	5.164829	0.164540	−1.463136
C	−4.029544	−0.518968	−0.764773
C	−5.238186	−0.660078	−1.444051
C	−6.289367	−1.378242	−0.897951
C	−6.109103	−1.956441	0.343985
C	−4.931260	−1.841428	1.049666
C	−3.890789	−1.115594	0.482696
F	−7.131386	−2.657964	0.888729
O	3.612795	−3.003300	1.846918
C	2.615969	−3.522196	2.714495

O	5.529576	−2.033315	0.206340
C	6.357832	−1.789725	1.343910
O	−1.214256	3.132464	2.165154
C	−2.244268	2.935122	3.138324
O	−3.252408	2.312325	0.375258
C	−3.249173	3.664146	−0.093403
O	1.235666	1.929422	2.028103
C	1.163303	1.493516	3.383507
H	1.258095	−1.655087	1.736005
H	1.955153	1.236670	−2.285974
H	2.048302	2.283235	−0.876256
H	4.441579	2.546692	−1.116357
H	3.202257	2.634298	−3.909357
H	3.061126	3.918215	−2.696694
H	4.646710	3.515166	−3.377091
H	0.245407	−1.882139	−1.091350
H	0.361612	−0.666321	−2.351246
H	−2.114313	−2.188133	−1.453330
H	−0.832851	−1.785705	−4.194873
H	−2.252684	−2.787273	−3.836539
H	−0.663406	−3.240804	−3.197851
H	−3.343160	1.149803	−1.885549
H	5.929682	0.675310	−0.859312
H	5.648341	−0.676271	−1.959743
H	−5.360221	−0.198443	−2.418417
H	−7.234114	−1.488853	−1.415843
H	−4.832042	−2.309445	2.021256
H	−2.961057	−1.014542	1.031561
H	3.070955	−4.369209	3.221977
H	1.741390	−3.862542	2.153412
H	2.308369	−2.777205	3.453670
H	7.306010	−2.288834	1.153261
H	6.531292	−0.716709	1.469612
H	5.904422	−2.197917	2.249270
H	−2.142467	3.744257	3.858621
H	−2.109921	1.975752	3.646325
H	−3.234024	2.965859	2.680830
H	−2.844913	3.713232	−1.108326
H	−2.661496	4.307255	0.564919
H	−4.286906	3.990804	−0.098953
H	0.256150	1.861290	3.868302
H	1.194658	0.400950	3.438098
H	2.033999	1.905563	3.889830

ωB97X Energy = −1828.88013279 a.u.

(*aR*,1*S*,3*S*,3'*S*)-**21**, Conf K

C	−1.978027	−1.365643	−1.083536
C	−3.199159	−2.025256	−1.064350
C	−4.213871	−1.545913	−0.228286
C	−3.992825	−0.445740	0.585723
C	−2.766677	0.223196	0.559411
C	−1.761589	−0.247231	−0.281018
C	−2.564700	1.440424	1.430544
C	−3.885565	1.983192	1.956718
C	−3.699448	3.013477	3.047005
O	−4.656352	0.911786	2.495229
C	−0.214211	1.449423	−1.242829
C	1.013133	2.117672	−1.303551
C	2.039905	1.717414	−0.452150
C	1.840205	0.679931	0.458934
C	0.607385	0.040303	0.532272
C	−0.428619	0.426558	−0.326259
C	0.383988	−1.041432	1.562369
C	1.684451	−1.544874	2.168081
C	1.463968	−2.348454	3.429442
O	2.514770	−0.433908	2.506427
C	2.983568	0.277430	1.366673
C	−5.106952	0.028696	1.490205
C	4.090630	−0.501743	0.665000
C	5.308468	−0.651031	1.325902
C	6.345871	−1.377871	0.765203
C	6.142572	−1.956346	−0.473015
C	4.955037	−1.833142	−1.160774
C	3.928608	−1.098784	−0.579206
F	7.151087	−2.666713	−1.032123
O	−3.494344	−3.125677	−1.803182
C	−2.490465	−3.626419	−2.673361
O	−5.425057	−2.191657	−0.158675
C	−6.267750	−1.976862	−1.291377
O	1.251501	3.097195	−2.229777
C	0.489189	4.297777	−2.067650
O	3.287329	2.277366	−0.513187
C	3.378325	3.666510	−0.192913
O	−1.236214	1.834137	−2.074270
C	−1.095884	1.382837	−3.422616
H	−1.173774	−1.717169	−1.717641
H	−1.920761	1.190584	2.282797
H	−2.050840	2.223281	0.866196
H	−4.448842	2.426354	1.121522
H	−3.191964	2.565602	3.904551
H	−3.092868	3.844496	2.682285
H	−4.662378	3.405449	3.377215
H	−0.157293	−1.879631	1.117205
H	−0.254226	−0.647185	2.362634
H	2.211327	−2.164061	1.429705
H	0.994040	−1.726760	4.195113
H	2.411387	−2.724887	3.817678
H	0.809995	−3.198449	3.225468
H	3.431810	1.185009	1.775783
H	−5.888335	0.515358	0.887619
H	−5.566016	−0.820330	1.995908
H	5.448275	−0.189581	2.297942
H	7.297229	−1.495228	1.269334
H	4.837706	−2.301430	−2.130231
H	2.991393	−0.991654	−1.113953
H	−2.206180	−2.879784	−3.420156
H	−2.927676	−4.488290	−3.171342
H	−1.604100	−3.940673	−2.115558
H	−5.812963	−2.383292	−2.196841
H	−7.203215	−2.494648	−1.088158
H	−6.465576	−0.909062	−1.425374

H	0.644427	4.716646	−1.069023
H	−0.573481	4.112034	−2.225239
H	0.858969	4.997439	−2.814626
H	2.826958	3.884869	0.726118
H	3.001348	4.285759	−1.007157
H	4.434989	3.875004	−0.036927
H	−0.172407	1.761912	−3.866318
H	−1.104864	0.289447	−3.460972
H	−1.952308	1.770762	−3.970519

ωB97X Energy = −1828.88013235 a.u.

(*aR*,1*S*,3*S*,3’*S*)-**21**, Conf L

C	−2.028591	−1.183981	−1.340523
C	−3.253812	−1.829625	−1.410886
C	−4.256799	−1.478665	−0.499267
C	−4.016922	−0.525366	0.477139
C	−2.779589	0.122827	0.551421
C	−1.789882	−0.212485	−0.369728
C	−2.562675	1.166653	1.622198
C	−3.882100	1.673921	2.184445
C	−3.700536	2.542988	3.407719
O	−4.686681	0.558157	2.558687
C	−0.191667	1.509510	−1.198460
C	1.042048	2.169249	−1.175621
C	2.042819	1.697299	−0.329876
C	1.795046	0.630447	0.534059
C	0.551930	0.007644	0.537768
C	−0.449228	0.447225	−0.338009
C	0.290767	−1.133436	1.492834
C	1.570499	−1.699901	2.086593
C	1.310222	−2.586752	3.283131
O	2.408151	−0.629444	2.521209
C	2.907356	0.167783	1.453306
C	−5.130178	−0.179709	1.439885
C	4.043576	−0.544567	0.730833
C	3.860385	−1.240306	−0.459711
C	4.912585	−1.916259	−1.064285
C	6.149431	−1.877616	−0.456530
C	6.375133	−1.198259	0.724703
C	5.309468	−0.534611	1.311894
F	7.184066	−2.525783	−1.041504
O	−3.568045	−2.796343	−2.311010
C	−2.571565	−3.175385	−3.248667
O	−5.475818	−2.112821	−0.523525
C	−6.323044	−1.710448	−1.600511
O	1.238634	3.220557	−2.029882
C	1.502331	4.478651	−1.402333
O	3.274116	2.299593	−0.275583
C	4.060900	2.216897	−1.468250
O	−1.140296	1.887655	−2.113727
C	−1.891459	3.046887	−1.764182
H	−1.238825	−1.425596	−2.040312
H	−1.977600	0.745402	2.448113
H	−1.984456	2.006144	1.227972
H	−4.414658	2.239173	1.404043
H	−3.215044	1.974956	4.204694
H	−3.076252	3.406134	3.169082
H	−4.663812	2.902012	3.772537
H	−0.258098	−1.929735	0.984080
H	−0.350427	−0.783070	2.309796
H	2.103197	−2.276395	1.319017
H	0.832447	−2.013338	4.080969
H	2.243285	−3.004413	3.663749
H	0.648983	−3.409804	3.005508
H	3.332224	1.045314	1.943341
H	−5.910458	0.383428	0.906471
H	−5.590673	−1.089465	1.824202
H	2.886716	−1.250973	−0.937523
H	4.779924	−2.457441	−1.992950
H	7.364074	−1.189494	1.166086
H	5.467332	0.004955	2.239989
H	−2.282619	−2.332186	−3.881916
H	−3.018241	−3.952755	−3.863655
H	−1.686778	−3.574217	−2.745162
H	−7.260410	−2.250024	−1.479408
H	−6.515748	−0.634244	−1.554901
H	−5.874932	−1.963888	−2.563156
H	2.472513	4.478541	−0.902931
H	0.721535	4.713326	−0.673185
H	1.493877	5.223424	−2.195372
H	4.242014	1.171553	−1.730594
H	5.007919	2.702446	−1.241904
H	3.572703	2.726824	−2.300539
H	−1.243455	3.919549	−1.654165
H	−2.593355	3.220745	−2.577417
H	−2.448669	2.882683	−0.836900

ωB97X Energy = −1828.87993546 a.u.

(*aR*,1*S*,3*S*,3’*S*)-**21**, Conf M

C	−2.119321	−0.332403	−1.601854
C	−3.372122	−0.846973	−1.905518
C	−4.352530	−0.873676	−0.907321
C	−4.076405	−0.391707	0.362988
C	−2.813313	0.121190	0.669516
C	−1.838832	0.148031	−0.324284
C	−2.553241	0.662875	2.055280
C	−3.537938	0.098692	3.072516
C	−3.241498	−1.345714	3.451865
O	−4.868676	0.275243	2.579100
C	−0.175969	2.003547	−0.281234
C	1.093969	2.519455	−0.009231
C	2.076986	1.675061	0.495513
C	1.789603	0.336103	0.761738
C	0.521518	−0.169840	0.497051
C	−0.467720	0.666873	−0.036770
C	0.209550	−1.612182	0.819702
C	1.462768	−2.434083	1.073585
C	1.164289	−3.763717	1.728594
O	2.335589	−1.716493	1.944801
C	2.873763	−0.533442	1.364111

C	−5.155315	−0.477023	1.417199
C	4.004802	−0.865031	0.400466
C	3.819325	−0.959979	−0.974949
C	4.865953	−1.315667	−1.816213
C	6.100093	−1.566468	−1.254675
C	6.328806	−1.477954	0.104450
C	5.268319	−1.127141	0.924898
F	7.128704	−1.906692	−2.066302
O	−3.726617	−1.348974	−3.116634
C	−2.761735	−1.306098	−4.157189
O	−5.587292	−1.426301	−1.150958
C	−6.439313	−0.619566	−1.965764
O	1.351058	3.844381	−0.246883
C	1.303548	4.653311	0.930931
O	3.315850	2.154326	0.832900
C	4.123482	2.619514	−0.252949
O	−1.164645	2.846016	−0.718338
C	−1.058430	3.213206	−2.093888
H	−1.337829	−0.312505	−2.350877
H	−1.530304	0.441208	2.369646
H	−2.644591	1.753650	2.039188
H	−3.507018	0.709795	3.976214
H	−3.165534	−1.995435	2.576453
H	−2.289890	−1.393831	3.985048
H	−4.021217	−1.735812	4.108731
H	−0.364035	−2.063734	0.006670
H	−0.428694	−1.652091	1.711642
H	1.979617	−2.609570	0.120861
H	0.691338	−3.607647	2.701088
H	2.082835	−4.333831	1.874032
H	0.486991	−4.349309	1.104099
H	3.312166	0.005843	2.205361
H	−6.096408	−0.087683	1.027030
H	−5.327503	−1.531221	1.672005
H	2.848208	−0.744808	−1.407552
H	4.730927	−1.393401	−2.887929
H	7.316074	−1.675386	0.503452
H	5.429096	−1.049984	1.995214
H	−3.245060	−1.731700	−5.033097
H	−1.880211	−1.901524	−3.904341
H	−2.458723	−0.277415	−4.372002
H	−7.385727	−1.150240	−2.050273
H	−6.609304	0.353603	−1.494960
H	−6.007437	−0.477714	−2.958012
H	2.062969	4.333616	1.648786
H	0.312921	4.598669	1.390106
H	1.503603	5.675682	0.616637
H	4.267126	1.822146	−0.987349
H	5.085067	2.889877	0.178610
H	3.672452	3.489802	−0.730836
H	−1.871799	3.907605	−2.295360
H	−0.102055	3.701514	−2.291899
H	−1.167073	2.331329	−2.732956

ωB97X Energy = −1828.87896180 a.u.

(*aR*,1*S*,3*S*,3'*S*)-**21**, Conf N

C	2.118721	1.586656	−0.861372
C	3.400408	2.116069	−0.790389
C	4.413872	1.356784	−0.194850
C	4.144798	0.089078	0.297778
C	2.863271	−0.456621	0.195192
C	1.849944	0.309124	−0.375338
C	2.601206	−1.848972	0.717852
C	3.893797	−2.610904	0.972514
C	3.675629	−3.879276	1.765252
O	4.795241	−1.784752	1.706186
C	0.065447	−0.934317	−1.589675
C	−1.225227	−1.454231	−1.690517
C	−2.140791	−1.220206	−0.669244
C	−1.771058	−0.479924	0.456511
C	−0.465073	−0.007680	0.569820
C	0.453622	−0.214680	−0.466033
C	−0.049703	0.701256	1.835637
C	−0.928971	0.279137	3.003079
C	−0.677982	−1.146000	3.474117
O	−2.292668	0.509237	2.646480
C	−2.794141	−0.267261	1.564719
C	5.265734	−0.704556	0.927720
C	−4.036787	0.458006	1.090478
C	−5.298415	−0.077077	1.308412
C	−6.441018	0.594883	0.892621
C	−6.288876	1.813833	0.266389
C	−5.050762	2.384719	0.040965
C	−3.924745	1.693942	0.460133
F	−7.395133	2.479600	−0.142950
O	3.759273	3.335742	−1.267647
C	2.752949	4.128877	−1.879197
O	5.703388	1.828422	−0.137093
C	5.906952	2.872401	0.816352
O	−1.580189	−2.193970	−2.788844
C	−1.323241	−3.590892	−2.631474
O	−3.384730	−1.791594	−0.709458
C	−4.259853	−1.307690	−1.733495
O	0.980869	−1.218460	−2.569579
C	0.827490	−0.438120	−3.755433
H	1.311655	2.157352	−1.303144
H	2.030443	−1.796986	1.654173
H	1.985921	−2.406404	0.006303
H	4.362334	−2.854512	0.007130
H	3.251839	−3.643759	2.744387
H	2.982592	−4.538464	1.239158
H	4.617344	−4.410223	1.910477
H	−0.132777	1.784363	1.703319
H	0.998562	0.491960	2.059247
H	−0.760238	0.958809	3.840126
H	−0.763337	−1.870193	2.660398
H	−1.390035	−1.416329	4.255856
H	0.329433	−1.224251	3.888338
H	−3.097823	−1.254403	1.936911
H	5.940721	−1.074749	0.142587
H	5.854743	−0.070379	1.590392

H	−5.396730	−1.039903	1.798104
H	−7.430773	0.184823	1.051023
H	−4.978211	3.346198	−0.452227
H	−2.942655	2.122899	0.290395
H	2.331104	3.626562	−2.754154
H	3.243474	5.047315	−2.191965
H	1.953031	4.366552	−1.172756
H	5.311384	3.751580	0.564140
H	5.649107	2.527487	1.822443
H	6.965236	3.124018	0.781048
H	−1.894607	−3.992668	−1.790495
H	−0.256100	−3.767789	−2.473758
H	−1.641008	−4.074376	−3.553131
H	−5.202345	−1.836040	−1.603386
H	−3.850387	−1.513288	−2.722707
H	−4.428095	−0.233993	−1.611029
H	−0.166018	−0.579641	−4.186595
H	0.992591	0.621684	−3.537981
H	1.583165	−0.784799	−4.457589

ωB97X Energy = −1828.87894652 a.u.

(*aR*,1*S*,3*S*,3’*S*)-**21**, Conf O

C	−2.033272	−0.979986	−1.390879
C	−3.283206	−1.566941	−1.513988
C	−4.286592	−1.233510	−0.606057
C	−4.039399	−0.294894	0.392232
C	−2.790740	0.314829	0.495127
C	−1.776208	−0.046473	−0.395728
C	−2.554748	1.350533	1.568376
C	−3.857191	1.835602	2.188622
C	−3.635885	2.629507	3.455666
O	−4.674622	0.713296	2.513436
C	−0.117574	1.718402	−0.980216
C	1.146693	2.305417	−0.890295
C	2.125824	1.696143	−0.112336
C	1.836307	0.534589	0.603904
C	0.570421	−0.036442	0.522934
C	−0.411680	0.551175	−0.285005
C	0.255487	−1.272670	1.331630
C	1.507328	−1.953667	1.860349
C	1.205646	−2.972930	2.935525
O	2.378412	−0.977048	2.429675
C	2.918535	−0.071393	1.473298
C	−5.148215	0.057336	1.356439
C	4.046513	−0.724683	0.685750
C	5.300324	−0.828655	1.283954
C	6.355147	−1.454439	0.638735
C	6.131346	−1.980135	−0.618879
C	4.907189	−1.899345	−1.247749
C	3.865697	−1.263657	−0.583747
F	7.154941	−2.592119	−1.259446
O	−3.490079	−2.495777	−2.504179
C	−4.362087	−2.056526	−3.547721
O	−5.546407	−1.774101	−0.697388
C	−5.623099	−3.163871	−0.369637
O	1.402885	3.463824	−1.575997
C	1.351775	4.634999	−0.757957
O	3.362311	2.265352	0.044802
C	4.171554	2.341843	−1.133165
O	−1.101825	2.347987	−1.696347
C	−0.987390	2.193926	−3.111117
H	−1.258748	−1.263787	−2.095627
H	−1.919517	0.931759	2.358234
H	−2.013995	2.203115	1.148848
H	−4.397382	2.452265	1.454691
H	−3.150543	2.005537	4.209756
H	−2.994459	3.489705	3.254997
H	−4.583826	2.989718	3.857472
H	−0.316408	−1.981501	0.728338
H	−0.384058	−0.994454	2.178270
H	2.026637	−2.449058	1.029178
H	0.741314	−2.486759	3.796694
H	2.120488	−3.467410	3.264781
H	0.518532	−3.730943	2.554799
H	3.359763	0.726372	2.072995
H	−5.892982	0.685856	0.847132
H	−5.658687	−0.845447	1.692166
H	5.457267	−0.408305	2.271940
H	7.334475	−1.534369	1.094193
H	4.775257	−2.322388	−2.235944
H	2.902205	−1.182233	−1.075176
H	−4.407340	−2.864120	−4.275564
H	−3.959184	−1.158644	−4.025121
H	−5.362502	−1.851047	−3.161256
H	−5.018323	−3.760948	−1.054683
H	−5.288494	−3.331340	0.658301
H	−6.670198	−3.445460	−0.460304
H	0.359937	4.744706	−0.311203
H	1.552386	5.481204	−1.412017
H	2.109704	4.589946	0.028007
H	4.316346	1.345169	−1.558879
H	5.132346	2.741628	−0.815236
H	3.721390	3.001881	−1.875434
H	−0.038982	2.599516	−3.469983
H	−1.070186	1.138065	−3.386571
H	−1.813101	2.748079	−3.553210

ωB97X Energy = −1828.87887908 a.u.

(*aS*,1*R*,3*S*,3’*S*)-**21**, Conf A

C	2.009453	−1.497712	−0.892200
C	3.260136	−2.098059	−0.932495
C	4.343686	−1.445375	−0.333424
C	4.164819	−0.226426	0.302787
C	2.901475	0.366092	0.363094
C	1.828163	−0.272828	−0.252631
C	2.730451	1.684914	1.077327
C	3.923814	2.009366	1.963763
C	3.914077	3.439789	2.452568
O	5.129364	1.807497	1.229192

C	0.161911	1.399209	−1.051943
C	−1.100317	1.993296	−1.025041
C	−2.084914	1.473634	−0.190756
C	−1.810489	0.373289	0.626000
C	−0.526976	−0.170166	0.640123
C	0.459548	0.324527	−0.222862
C	−0.195961	−1.249519	1.642598
C	−1.185991	−1.228904	2.792473
C	−1.004911	−2.383370	3.751306
O	−2.502149	−1.320380	2.254573
C	−2.912953	−0.170979	1.527835
C	5.354419	0.440999	0.953509
C	−4.151629	−0.588405	0.763214
C	−5.398762	−0.079434	1.097259
C	−6.539272	−0.475336	0.410420
C	−6.401265	−1.397670	−0.605426
C	−5.179159	−1.936082	−0.959982
C	−4.053989	−1.522000	−0.264340
F	−7.506021	−1.792594	−1.282189
O	3.521744	−3.297918	−1.513505
C	2.441004	−3.984963	−2.126372
O	5.589689	−2.024793	−0.314921
C	6.256107	−2.028739	−1.578578
O	−1.366687	3.079177	−1.817904
C	−0.918942	4.313498	−1.253300
O	−3.303619	2.088585	−0.086353
C	−4.126614	2.053047	−1.256647
O	1.135058	1.941160	−1.852032
C	1.038254	1.551171	−3.221981
H	1.154447	−1.978563	−1.350767
H	2.601769	2.485253	0.339335
H	1.821304	1.667102	1.685629
H	3.929577	1.324569	2.825463
H	3.969000	4.127131	1.605161
H	2.993706	3.644392	3.002822
H	4.762105	3.629629	3.111947
H	0.818600	−1.100812	2.021085
H	−0.218275	−2.239152	1.173079
H	−1.090638	−0.277358	3.335974
H	−1.133250	−3.333322	3.227395
H	−1.735638	−2.329592	4.559304
H	−0.003993	−2.359856	4.185926
H	−3.194866	0.618041	2.238514
H	5.611600	−0.090615	1.881117
H	6.223822	0.391349	0.297843
H	−5.484995	0.649943	1.895378
H	−7.517020	−0.079638	0.656075
H	−5.118226	−2.661258	−1.762079
H	−3.084005	−1.929909	−0.528633
H	2.858638	−4.906105	−2.525270
H	2.010437	−3.398243	−2.942451
H	1.661853	−4.224682	−1.397640
H	7.230312	−2.485797	−1.415829
H	5.694958	−2.610537	−2.311821
H	6.390963	−1.005886	−1.943497
H	0.164097	4.298237	−1.108351
H	−1.419544	4.500067	−0.299310
H	−1.182232	5.095664	−1.962565
H	−5.057071	2.550152	−0.989024
H	−4.338231	1.017924	−1.539179
H	−3.648223	2.577700	−2.083661
H	1.847773	2.053475	−3.747983
H	1.158162	0.467784	−3.319339
H	0.079027	1.858446	−3.645138

ωB97X Energy = −1828.88139278 a.u.

(*aS*,1*R*,3*S*,3’*S*)-**21**, Conf B

C	2.022732	−1.339811	−1.141174
C	3.285777	−1.899878	−1.275242
C	4.370355	−1.282616	−0.641955
C	4.188689	−0.116283	0.085762
C	2.917017	0.444280	0.224065
C	1.836520	−0.174423	−0.400094
C	2.743633	1.701000	1.041994
C	3.955784	1.977388	1.918844
C	3.932116	3.361604	2.525779
O	5.141565	1.864813	1.134868
C	0.108880	1.514422	−1.009628
C	−1.162274	2.077740	−0.890662
C	−2.104615	1.475331	−0.063730
C	−1.780178	0.319574	0.651995
C	−0.487504	−0.195975	0.577513
C	0.457481	0.385920	−0.277592
C	−0.100091	−1.344001	1.478384
C	−1.050844	−1.439219	2.657518
C	−0.814390	−2.664420	3.510886
O	−2.382715	−1.515365	2.157018
C	−2.839821	−0.318938	1.542270
C	5.387871	0.532369	0.738666
C	−4.097767	−0.698321	0.789386
C	−4.020194	−1.511882	−0.337145
C	−5.164079	−1.892347	−1.021544
C	−6.384445	−1.442898	−0.554745
C	−6.502558	−0.641355	0.561086
C	−5.343311	−0.277004	1.233743
F	−7.507581	−1.805126	−1.219394
O	3.562374	−3.019886	−1.992388
C	2.482372	−3.670032	−2.645396
O	5.640836	−1.787462	−0.782672
C	5.889145	−2.973117	−0.026825
O	−1.476107	3.216187	−1.586805
C	−1.048399	4.412363	−0.932155
O	−3.328423	2.054394	0.138551
C	−4.198456	2.107193	−0.996403
O	1.040965	2.137767	−1.798717
C	0.892858	1.861171	−3.191667
H	1.164602	−1.797835	−1.616919
H	2.582293	2.554100	0.372743
H	1.852027	1.619294	1.670559
H	4.001427	1.223267	2.719204
H	3.946938	4.117644	1.737338

H	3.025251	3.498883	3.117704
H	4.796340	3.515323	3.173425
H	0.923532	−1.201912	1.834463
H	−0.117542	−2.293399	0.931858
H	−0.956001	−0.533635	3.274628
H	−0.941234	−3.570864	2.914541
H	−1.518343	−2.693952	4.343587
H	0.199912	−2.653952	3.913983
H	−3.110534	0.403886	2.324191
H	5.701197	−0.064244	1.608518
H	6.224964	0.558661	0.041654
H	−3.051369	−1.849542	−0.690206
H	−5.118699	−2.522856	−1.900982
H	−7.479744	−0.313744	0.893907
H	−5.414586	0.356850	2.111084
H	1.731949	−4.009352	−1.926087
H	2.912293	−4.530573	−3.151996
H	2.012088	−3.012276	−3.381454
H	6.930905	−3.238427	−0.196675
H	5.731019	−2.788248	1.040035
H	5.243802	−3.788566	−0.359123
H	0.036764	4.411036	−0.802364
H	−1.539361	4.512361	0.039699
H	−1.338724	5.241733	−1.574170
H	−3.776395	2.735916	−1.780302
H	−5.134421	2.533505	−0.640377
H	−4.383074	1.100078	−1.380608
H	−0.086068	2.189224	−3.549288
H	1.675116	2.416000	−3.705978
H	1.020098	0.791157	−3.382491

ωB97X Energy = −1828.88111681 a.u.

(*aS*,1*R*,3*S*,3’*S*)-**21**, Conf C

C	2.098650	0.728659	−1.593888
C	3.358034	0.588163	−2.158717
C	4.374683	−0.008259	−1.403859
C	4.122136	−0.459339	−0.118101
C	2.849265	−0.331473	0.445771
C	1.842246	0.276301	−0.301093
C	2.604289	−0.860098	1.840216
C	3.697827	−1.821881	2.281371
C	3.623346	−2.150523	3.755041
O	4.971528	−1.233262	2.030277
C	0.093847	1.635155	0.846105
C	−1.196742	1.814319	1.356932
C	−2.114294	0.771023	1.266650
C	−1.770471	−0.423402	0.627901
C	−0.478837	−0.598957	0.140182
C	0.458596	0.436944	0.241769
C	−0.096624	−1.931120	−0.457421
C	−1.027805	−3.021799	0.038890
C	−0.790495	−4.354669	−0.633887
O	−2.368427	−2.633906	−0.245529
C	−2.827307	−1.513770	0.499391
C	5.239746	−1.128632	0.648197

C	−4.081572	−1.042406	−0.207907
C	−5.333941	−1.247736	0.353922
C	−6.487935	−0.844582	−0.305832
C	−6.357057	−0.243659	−1.540071
C	−5.129336	−0.031057	−2.136901
C	−3.991254	−0.437743	−1.458119
F	−7.474947	0.153435	−2.194164
O	3.689529	0.982225	−3.415065
C	2.675873	1.577681	−4.211364
O	5.622131	−0.212454	−1.943606
C	6.407831	0.973517	−2.072170
O	−1.551999	2.964851	2.010676
C	−1.630258	4.132798	1.187274
O	−3.346962	0.847536	1.852309
C	−4.234332	1.868340	1.392888
O	1.005925	2.657946	0.911420
C	1.493361	2.959252	2.220355
H	1.292797	1.186235	−2.153411
H	2.562492	−0.032128	2.557078
H	1.634958	−1.363686	1.893575
H	3.623640	−2.747906	1.690917
H	3.747209	−1.241785	4.348710
H	2.654232	−2.591448	3.995808
H	4.405228	−2.858527	4.032646
H	0.936140	−2.178088	−0.201659
H	−0.149906	−1.890486	−1.551287
H	−0.911262	−3.130131	1.127584
H	−0.937974	−4.263276	−1.712411
H	−1.480765	−5.106941	−0.250023
H	0.230629	−4.694134	−0.450989
H	−3.102827	−1.845739	1.509800
H	5.422174	−2.127193	0.225423
H	6.164381	−0.559922	0.548680
H	−5.415651	−1.718078	1.327923
H	−7.470309	−0.993453	0.125039
H	−5.073366	0.442463	−3.109333
H	−3.017143	−0.277952	−1.908518
H	1.845641	0.885227	−4.374817
H	3.143100	1.812642	−5.164493
H	2.300854	2.496882	−3.753392
H	7.370415	0.667438	−2.477475
H	5.932751	1.683598	−2.751330
H	6.557323	1.441717	−1.094362
H	−0.658930	4.371058	0.751782
H	−1.955041	4.943482	1.836454
H	−2.364832	3.988124	0.389672
H	−3.983634	2.833361	1.833000
H	−5.232628	1.569148	1.708289
H	−4.210429	1.936019	0.301350
H	2.220660	3.759645	2.101008
H	0.685311	3.287575	2.876812
H	1.986662	2.085562	2.654402

ωB97X Energy = −1828.87943253 a.u.

(*aS*,1*R*,3*S*,3’*S*)-**21**, Conf D

C	2.022104	−1.453226	−1.080391
C	3.269297	−2.060481	−1.129718
C	4.337688	−1.467549	−0.447423
C	4.148060	−0.300319	0.275737
C	2.888621	0.300582	0.339167
C	1.830000	−0.279062	−0.355474
C	2.707975	1.557981	1.155270
C	3.862035	1.773670	2.122828
C	3.845605	3.148087	2.751905
O	5.095856	1.628392	1.423110
C	0.147641	1.376908	−1.166210
C	−1.124839	1.955095	−1.130789
C	−2.088630	1.432415	−0.267644
C	−1.787587	0.367646	0.581492
C	−0.498727	−0.163235	0.579969
C	0.461338	0.319714	−0.313849
C	−0.135912	−1.214480	1.600723
C	−1.095940	−1.164298	2.774869
C	−0.880926	−2.285375	3.765976
O	−2.424290	−1.284737	2.274730
C	−2.867842	−0.169512	1.513995
C	5.320469	0.293073	1.022567
C	−4.106407	−0.643439	0.782275
C	−5.360206	−0.147139	1.109821
C	−6.499633	−0.594788	0.453608
C	−6.353317	−1.555466	−0.524826
C	−5.124035	−2.082980	−0.871007
C	−4.000330	−1.617209	−0.206462
F	−7.456876	−2.001309	−1.171321
O	3.541733	−3.213230	−1.794506
C	2.475292	−3.845207	−2.486893
O	5.577540	−2.061192	−0.434424
C	6.295031	−1.943763	−1.663907
O	−1.418986	2.977196	−1.995637
C	−1.678036	4.243338	−1.382217
O	−3.335696	1.998014	−0.195975
C	−4.150695	1.869318	−1.365807
O	1.105695	1.781689	−2.050713
C	1.442104	3.168366	−2.099926
H	1.177075	−1.889294	−1.598036
H	2.641371	2.427593	0.490871
H	1.767486	1.514842	1.712195
H	3.820127	1.005413	2.909711
H	3.946538	3.915323	1.980616
H	2.903470	3.310728	3.278727
H	4.664800	3.257940	3.463709
H	0.887767	−1.054705	1.948721
H	−0.168394	−2.216013	1.157570
H	−0.994457	−0.194755	3.284628
H	−1.010873	−3.252756	3.275495
H	−1.594347	−2.212765	4.587892
H	0.128964	−2.237821	4.177285
H	−3.159375	0.636969	2.200889
H	5.546044	−0.328105	1.901469
H	6.209851	0.298713	0.392095
H	−5.453048	0.612401	1.878517
H	−7.482849	−0.209805	0.694488
H	−5.056539	−2.838963	−1.643573
H	−3.024864	−2.015513	−0.465219
H	1.675722	−4.134651	−1.799549
H	2.899951	−4.736114	−2.942907
H	2.069833	−3.195172	−3.266868
H	5.758801	−2.437043	−2.476642
H	6.455820	−0.890453	−1.913682
H	7.256957	−2.429714	−1.511947
H	−2.569663	4.201446	−0.754629
H	−1.829240	4.952507	−2.193640
H	−0.822075	4.558427	−0.778766
H	−5.106188	2.329495	−1.122041
H	−4.307252	0.813982	−1.604179
H	−3.698518	2.378528	−2.218022
H	2.467313	3.221471	−2.463382
H	0.782468	3.712423	−2.775225
H	1.393225	3.616199	−1.103318

ωB97X Energy = −1828.87926012 a.u.

(*aS*,1*R*,3*S*,3'*S*)-**21**, Conf E

C	2.141305	0.125268	−1.778429
C	3.424490	−0.186414	−2.204577
C	4.427242	−0.378298	−1.247410
C	4.148929	−0.227035	0.101967
C	2.857025	0.090698	0.531522
C	1.855800	0.265559	−0.422038
C	2.583762	0.215259	2.012965
C	3.683899	−0.429308	2.842756
C	3.571285	−0.106048	4.315118
O	4.949285	0.043784	2.389124
C	0.029517	1.914578	−0.024591
C	−1.271637	2.251049	0.365092
C	−2.142977	1.242806	0.768036
C	−1.742466	−0.096042	0.734415
C	−0.439118	−0.418167	0.367302
C	0.453093	0.590300	−0.019748
C	0.005774	−1.858650	0.434273
C	−0.883607	−2.646340	1.378284
C	−0.580735	−4.127397	1.377926
O	−2.236401	−2.486075	0.962798
C	−2.749652	−1.170700	1.127264
C	5.264334	−0.438039	1.100353
C	−4.018435	−1.123593	0.300573
C	−5.264490	−1.104365	0.911479
C	−6.430572	−1.089757	0.156612
C	−6.317626	−1.105474	−1.217636
C	−5.096359	−1.135464	−1.862688
C	−3.946188	−1.145377	−1.089034
F	−7.447201	−1.091521	−1.965320
O	3.795525	−0.319618	−3.503933
C	2.799895	−0.129026	−4.498224
O	5.715974	−0.662833	−1.630511
C	5.907200	−1.996307	−2.104307
O	−1.678492	3.557538	0.434172

C	−1.803716	4.222766	−0.826764
O	−3.381330	1.524006	1.273854
C	−4.308993	2.191234	0.416753
O	0.890491	2.898402	−0.440732
C	1.387901	3.746076	0.596434
H	1.344506	0.272540	−2.496193
H	2.507002	1.270685	2.298226
H	1.623396	−0.243355	2.263667
H	3.650361	−1.520301	2.699425
H	3.657382	0.971831	4.470827
H	2.605894	−0.435907	4.703408
H	4.360809	−0.603890	4.879650
H	1.044562	−1.919375	0.766237
H	−0.035697	−2.320059	−0.558881
H	−0.774099	−2.245454	2.397099
H	−0.724819	−4.541692	0.377468
H	−1.236909	−4.654961	2.071311
H	0.454080	−4.301091	1.678705
H	−3.016686	−1.021231	2.182408
H	5.509564	−1.509310	1.152189
H	6.162714	0.087046	0.777092
H	−5.332173	−1.086394	1.993990
H	−7.408410	−1.067941	0.621640
H	−5.054509	−1.150103	−2.944864
H	−2.977059	−1.165814	−1.576523
H	3.299556	−0.266061	−5.454004
H	2.379993	0.879206	−4.448794
H	1.996964	−0.864675	−4.399393
H	6.970437	−2.105410	−2.309578
H	5.609475	−2.721243	−1.340694
H	5.337370	−2.170135	−3.019070
H	−2.493924	3.677991	−1.477485
H	−0.834211	4.316575	−1.317589
H	−2.209407	5.209891	−0.614095
H	−5.291641	2.048081	0.863083
H	−4.300682	1.742754	−0.580820
H	−4.086653	3.256115	0.349780
H	1.996366	3.170917	1.299504
H	2.011481	4.494199	0.111211
H	0.570092	4.235892	1.128737

ωB97X Energy = −1828.87916240 a.u.

(*aS*,1*R*,3*S*,3'*S*)-**21**, Conf F

C	2.076642	−1.205328	−1.388187
C	3.347425	−1.748803	−1.517757
C	4.390259	−1.223227	−0.746894
C	4.161485	−0.162429	0.115871
C	2.881897	0.382056	0.247980
C	1.843067	−0.145347	−0.515280
C	2.655534	1.513083	1.222515
C	3.801534	1.638706	2.215391
C	3.735690	2.912940	3.025955
O	5.038573	1.636966	1.506789
C	0.078338	1.480942	−1.192189
C	−1.208166	2.017770	−1.084212
C	−2.122789	1.428634	−0.211099
C	−1.758344	0.335743	0.576195
C	−0.456709	−0.156142	0.501809
C	0.455569	0.398115	−0.401123
C	−0.028088	−1.245874	1.453794
C	−0.935542	−1.269645	2.669871
C	−0.650337	−2.426865	3.599894
O	−2.281843	−1.402163	2.223651
C	−2.785584	−0.271299	1.525144
C	5.316294	0.380317	0.926210
C	−4.037779	−0.754063	0.822954
C	−5.292337	−0.324003	1.231576
C	−6.443938	−0.783998	0.605883
C	−6.308296	−1.689982	−0.424817
C	−5.077919	−2.150596	−0.853033
C	−3.942261	−1.673483	−0.217492
F	−7.423802	−2.147960	−1.041590
O	3.669956	−2.770419	−2.352330
C	2.635055	−3.322333	−3.152408
O	5.667918	−1.714056	−0.875113
C	5.874777	−2.979360	−0.246869
O	−1.560830	3.070815	−1.887532
C	−1.831153	4.298207	−1.203711
O	−3.381247	1.952657	−0.063821
C	−4.240909	1.851858	−1.203870
O	0.986572	1.961717	−2.092706
C	1.327878	3.346206	−2.020940
H	1.250004	−1.593734	−1.969513
H	2.554440	2.461743	0.682028
H	1.718000	1.361454	1.765384
H	3.789337	0.769497	2.890679
H	3.808163	3.781175	2.366756
H	2.789238	2.968573	3.567055
H	4.551664	2.953864	3.748618
H	1.007199	−1.082950	1.763507
H	−0.063131	−2.226083	0.965349
H	−0.833174	−0.320415	3.216395
H	−0.781373	−3.375295	3.073947
H	−1.326111	−2.407109	4.455909
H	0.376234	−2.372631	3.966805
H	−3.073337	0.498577	2.254125
H	5.585314	−0.342384	1.710866
H	6.191555	0.512445	0.290734
H	−5.377074	0.393287	2.040806
H	−7.428470	−0.451314	0.910866
H	−5.018966	−2.865510	−1.664452
H	−2.965851	−2.019457	−0.540368
H	1.837858	−3.742787	−2.533372
H	3.096844	−4.116052	−3.734511
H	2.216108	−2.572333	−3.828750
H	6.920763	−3.237191	−0.401756
H	5.672811	−2.912519	0.826598
H	5.238299	−3.745100	−0.694726
H	−2.023534	5.042297	−1.974085
H	−0.964856	4.603927	−0.610263
H	−2.702095	4.203260	−0.553582

H	−3.841236	2.413630	−2.049254
H	−5.198150	2.270241	−0.899154
H	−4.376406	0.804146	−1.484897
H	0.600111	3.961458	−2.549669
H	1.399241	3.674731	−0.979992
H	2.304255	3.445269	−2.492238

ωB97X Energy = −1828.87895828 a.u.

(*aS*,1*R*,3*S*,3'*S*)-**21**, Conf G

C	−2.082404	1.457090	−0.877802
C	−3.343402	2.036639	−0.886101
C	−4.403650	1.358928	−0.273176
C	−4.191651	0.135475	0.343895
C	−2.917679	−0.436460	0.371634
C	−1.867593	0.228546	−0.255948
C	−2.710137	−1.760164	1.066730
C	−3.878921	−2.114388	1.974349
C	−3.835712	−3.550118	2.445443
O	−5.103000	−1.923945	1.267868
C	−0.169640	−1.385446	−1.108858
C	1.112935	−1.940456	−1.118954
C	2.069481	−1.446374	−0.235082
C	1.784378	−0.356216	0.587565
C	0.496527	0.173857	0.606712
C	−0.485291	−0.337971	−0.251008
C	0.155770	1.242815	1.616975
C	1.155797	1.235453	2.758860
C	0.967796	2.387786	3.718846
O	2.466718	1.342433	2.210164
C	2.884282	0.192621	1.486191
C	−5.355760	−0.558312	1.012940
C	4.128531	0.617506	0.734093
C	4.034777	1.345653	−0.447266
C	5.173454	1.787741	−1.103198
C	6.403883	1.490842	−0.550584
C	6.537836	0.778541	0.622599
C	5.383820	0.346519	1.262437
F	7.522062	1.915068	−1.187502
O	−3.635862	3.239185	−1.446452
C	−2.576532	3.956231	−2.062249
O	−5.657870	1.918288	−0.220097
C	−6.352884	1.925269	−1.468128
O	1.362487	−2.996179	−1.952058
C	2.392016	−2.789033	−2.923431
O	3.319716	−2.007984	−0.175407
C	3.348596	−3.324537	0.382401
O	−1.142255	−1.920381	−1.913118
C	−1.095207	−1.472470	−3.265544
H	−1.244432	1.958799	−1.345362
H	−2.584552	−2.550259	0.317548
H	−1.788350	−1.734183	1.655371
H	−3.877535	−1.439844	2.844143
H	−3.898013	−4.228090	1.591055
H	−2.900431	−3.746194	2.973161
H	−4.666035	−3.761546	3.120608
H	−0.852510	1.075087	2.004089
H	0.156659	2.234428	1.151198
H	1.077107	0.282898	3.303388
H	1.081732	3.339088	3.194098
H	1.704267	2.342503	4.522135
H	−0.029960	2.352579	4.159917
H	3.167096	−0.592358	2.202410
H	−5.599950	−0.041261	1.952221
H	−6.240693	−0.515150	0.377894
H	3.059543	1.566363	−0.868476
H	5.115676	2.349947	−2.027135
H	7.522385	0.569165	1.022233
H	5.467628	−0.216811	2.185884
H	−1.790859	4.196126	−1.340569
H	−3.015875	4.877113	−2.437736
H	−2.147971	3.392654	−2.895527
H	−5.815780	2.520304	−2.208696
H	−6.483756	0.904168	−1.839383
H	−7.328547	2.369065	−1.279476
H	3.374690	−2.728999	−2.453668
H	2.203985	−1.871283	−3.488214
H	2.352864	−3.643049	−3.596449
H	2.915940	−3.321178	1.386719
H	4.396582	−3.611529	0.439248
H	2.804176	−4.030318	−0.248592
H	−0.156060	−1.763765	−3.742917
H	−1.925544	−1.949469	−3.782584
H	−1.212011	−0.385344	−3.314253

ωB97X Energy = −1828.87892287 a.u.

(*aS*,1*R*,3*S*,3'*S*)-**21**, Conf H

C	2.039600	−1.381974	−0.975278
C	3.300675	−1.951053	−1.067698
C	4.375017	−1.320189	−0.428198
C	4.174907	−0.158248	0.299555
C	2.899209	0.402797	0.411821
C	1.836080	−0.209566	−0.247556
C	2.708151	1.648756	1.243316
C	3.896996	1.909417	2.155920
C	3.859063	3.285826	2.779730
O	5.103133	1.804754	1.401695
C	0.143529	1.549614	−0.810307
C	−1.113202	2.142246	−0.648389
C	−2.073417	1.496355	0.128278
C	−1.792995	0.271290	0.734215
C	−0.516565	−0.274790	0.626740
C	0.455391	0.356223	−0.160651
C	−0.174160	−1.505651	1.432749
C	−1.165530	−1.702010	2.565263
C	−0.983750	−3.019552	3.284018
O	−2.482672	−1.689029	2.022110
C	−2.889624	−0.417563	1.536132
C	5.355386	0.475476	0.999398
C	−4.146866	−0.668754	0.729605
C	−4.075072	−1.331602	−0.491847

C	−5.220453	−1.599366	−1.225668
C	−6.436199	−1.192078	−0.711292
C	−6.548938	−0.538753	0.497878
C	−5.388426	−0.284793	1.217366
F	−7.560707	−1.446065	−1.422723
O	3.584440	−3.097404	−1.738169
C	2.511850	−3.767962	−2.383020
O	5.631871	−1.874400	−0.459920
C	6.301198	−1.740427	−1.714827
O	−1.402306	3.387242	−1.133553
C	−1.408953	3.543978	−2.552499
O	−3.284021	2.073762	0.392624
C	−4.139155	2.336207	−0.720817
O	1.082889	2.246828	−1.528058
C	1.458102	1.676986	−2.783358
H	1.192339	−1.842778	−1.467557
H	2.566461	2.511602	0.583556
H	1.799763	1.559353	1.846024
H	3.921104	1.145032	2.947246
H	3.899420	4.051565	2.001610
H	2.934872	3.418377	3.345321
H	4.703455	3.428787	3.455397
H	0.836958	−1.410609	1.836659
H	−0.179948	−2.400288	0.800282
H	−1.070788	−0.872459	3.281339
H	−1.117425	−3.849635	2.586526
H	−1.711045	−3.120990	4.090594
H	0.018976	−3.083484	3.710497
H	−3.149316	0.224278	2.389462
H	5.633370	−0.133647	1.871830
H	6.219318	0.505063	0.335151
H	−3.109992	−1.639671	−0.880415
H	−5.179095	−2.111957	−2.178847
H	−7.522691	−0.239221	0.865403
H	−5.455726	0.231607	2.168884
H	1.747266	−4.071924	−1.662830
H	2.944848	−4.651660	−2.845100
H	2.058571	−3.139334	−3.154281
H	5.749661	−2.249697	−2.507131
H	6.425230	−0.683762	−1.971089
H	7.280214	−2.200793	−1.596398
H	−1.931939	2.710553	−3.030964
H	−0.395235	3.618902	−2.946066
H	−1.944442	4.469799	−2.756725
H	−3.819642	3.227727	−1.260503
H	−5.134833	2.492315	−0.308846
H	−4.162479	1.477289	−1.397731
H	2.100021	2.407500	−3.272251
H	2.005848	0.742895	−2.648189
H	0.575507	1.494781	−3.403630

ωB97X Energy = −1828.87885177 a.u.

(*aS*,1*R*,3*S*,3’*S*)-**21**, Conf I

C	2.068686	−1.310079	−1.114725
C	3.339001	−1.856984	−1.232365
C	4.411467	−1.219042	−0.598921
C	4.210481	−0.045812	0.112512
C	2.931337	0.501553	0.234276
C	1.863105	−0.137620	−0.390009
C	2.736582	1.766466	1.034229
C	3.938804	2.069296	1.916023
C	3.894175	3.461437	2.503362
O	5.131819	1.959822	1.142671
C	0.116322	1.516975	−1.037726
C	−1.171731	2.052322	−0.953912
C	−2.090515	1.468624	−0.084851
C	−1.762671	0.317569	0.631851
C	−0.468453	−0.192280	0.560974
C	0.475027	0.404325	−0.284609
C	−0.078658	−1.334035	1.468496
C	−1.034183	−1.426874	2.644576
C	−0.794616	−2.644823	3.507336
O	−2.363778	−1.513952	2.138883
C	−2.825554	−0.324206	1.513051
C	5.396592	0.624946	0.767001
C	−4.075128	−0.722887	0.755163
C	−5.328267	−0.489323	1.305463
C	−6.483562	−0.899293	0.653229
C	−6.352648	−1.551608	−0.554633
C	−5.124060	−1.809781	−1.130530
C	−3.984333	−1.390497	−0.461820
F	−7.472056	−1.953982	−1.203394
O	3.633623	−2.982758	−1.933215
C	2.565497	−3.653802	−2.584595
O	5.688787	−1.710521	−0.723720
C	5.944338	−2.885663	0.046023
O	−1.464051	3.175574	−1.677653
C	−2.520171	3.039185	−2.632876
O	−3.345464	2.002971	0.063930
C	−3.373712	3.256260	0.752073
O	1.053355	2.134318	−1.824774
C	0.971723	1.796513	−3.207163
H	1.219580	−1.784498	−1.590702
H	2.569274	2.607946	0.352204
H	1.841411	1.681790	1.657308
H	3.987249	1.326935	2.727180
H	3.907197	4.206650	1.704666
H	2.980617	3.596502	3.085415
H	4.750830	3.634135	3.156280
H	0.942672	−1.185294	1.828188
H	−0.090195	−2.286653	0.927512
H	−0.946917	−0.516519	3.255796
H	−0.913315	−3.556131	2.916790
H	−1.502210	−2.672728	4.337004
H	0.217709	−2.625402	3.915038
H	−3.110674	0.398628	2.290858
H	5.708523	0.043814	1.647760
H	6.239665	0.651100	0.077219
H	−5.409854	0.027488	2.255882
H	−7.466678	−0.718568	1.069969
H	−5.068467	−2.325708	−2.081188

H	−3.010300	−1.581756	−0.899944
H	3.008611	−4.515313	−3.078004
H	2.091840	−3.010854	−3.331477
H	1.814748	−3.993158	−1.865619
H	5.310020	−3.711290	−0.282337
H	6.990001	−3.141541	−0.113994
H	5.776813	−2.691638	1.109808
H	−3.480859	2.879544	−2.141419
H	−2.315779	2.205705	−3.311291
H	−2.541331	3.968883	−3.197654
H	−2.911812	3.158494	1.738577
H	−4.422933	3.520405	0.867181
H	−2.856926	4.030170	0.180434
H	0.018226	2.118056	−3.634203
H	1.784816	2.319920	−3.706425
H	1.093375	0.717913	−3.346674

ωB97X Energy = −1828.87875646 a.u.

(*aS*,1*R*,3*S*,3'*S*)-**21**, Conf J

C	1.989705	−1.563748	−0.808194
C	3.238653	−2.169002	−0.796335
C	4.314006	−1.490984	−0.210736
C	4.128853	−0.241122	0.360359
C	2.867569	0.358461	0.366404
C	1.802560	−0.307619	−0.234485
C	2.689975	1.713359	1.008122
C	3.868714	2.079665	1.898265
C	3.857652	3.534601	2.308536
O	5.086731	1.833361	1.198801
C	0.156540	1.315029	−1.161693
C	−1.101271	1.923521	−1.189347
C	−2.096437	1.455788	−0.334250
C	−1.845570	0.394503	0.537295
C	−0.567124	−0.153765	0.607096
C	0.436423	0.295762	−0.259143
C	−0.264968	−1.178152	1.673344
C	−1.278806	−1.083953	2.799258
C	−1.123291	−2.180354	3.828290
O	−2.584110	−1.203186	2.240558
C	−2.976488	−0.105270	1.427133
C	5.308652	0.453016	1.000544
C	−4.185216	−0.588515	0.652449
C	−4.034762	−1.502035	−0.385816
C	−5.137632	−1.997364	−1.063958
C	−6.391012	−1.562663	−0.679160
C	−6.581354	−0.661964	0.347452
C	−5.462077	−0.181462	1.014302
F	−7.475048	−2.039796	−1.337644
O	3.505618	−3.396728	−1.312873
C	2.432714	−4.110963	−1.908144
O	5.557069	−2.072886	−0.139926
C	6.247227	−2.140229	−1.388997
O	−1.403689	2.912508	−2.085527
C	−0.674368	4.134037	−1.928881
O	−3.356996	1.988897	−0.361187
C	−3.473817	3.332329	0.105147
O	1.138395	1.754933	−2.014250
C	1.007654	1.255630	−3.346359
H	1.140804	−2.064596	−1.256621
H	2.579263	2.476141	0.228483
H	1.769820	1.731040	1.599562
H	3.854743	1.442346	2.795619
H	3.931421	4.174033	1.425762
H	2.928903	3.772962	2.830381
H	4.694943	3.756508	2.971605
H	0.743235	−1.016773	2.063517
H	−0.288347	−2.192562	1.260040
H	−1.188928	−0.102091	3.286972
H	−1.247106	−3.158641	3.358266
H	−1.869200	−2.075910	4.617292
H	−0.130814	−2.135740	4.280244
H	−3.292038	0.723706	2.076610
H	5.541381	−0.030266	1.960461
H	6.191835	0.363161	0.368057
H	−3.041320	−1.830777	−0.673104
H	−5.035682	−2.707981	−1.874953
H	−7.582518	−0.348023	0.616030
H	−5.589999	0.530499	1.822903
H	1.641698	−4.313018	−1.180748
H	2.854081	−5.051433	−2.254568
H	2.016388	−3.564619	−2.758891
H	7.216655	−2.591335	−1.186113
H	5.697941	−2.755663	−2.103473
H	6.392345	−1.136794	−1.800993
H	−1.094537	4.834400	−2.648034
H	0.386893	3.985793	−2.128660
H	−0.805083	4.529862	−0.917316
H	−3.072564	4.040070	−0.621812
H	−2.959485	3.453607	1.062940
H	−4.537612	3.518745	0.241346
H	0.053869	1.561064	−3.783660
H	1.827589	1.681718	−3.921104
H	1.086468	0.164324	−3.352989

ωB97X Energy = −1828.87871108 a.u.

(*aS*,1*R*,3*S*,3'*S*)-**21**, Conf K

C	2.032330	−1.220272	−1.186332
C	3.299521	−1.753504	−1.367427
C	4.384033	−1.155377	−0.714345
C	4.194435	−0.039257	0.084787
C	2.915661	0.492963	0.274554
C	1.837395	−0.100958	−0.377210
C	2.736713	1.678117	1.192701
C	3.954490	1.896386	2.078074
C	3.917928	3.224070	2.799917
O	5.131846	1.870088	1.273578
C	0.091803	1.653978	−0.767950
C	−1.172985	2.203947	−0.532284
C	−2.092331	1.489037	0.233060
C	−1.763845	0.236962	0.754005

C	−0.480106	−0.270663	0.572301
C	0.451045	0.430225	−0.205425
C	−0.084536	−1.539223	1.290524
C	−1.027742	−1.819841	2.446220
C	−0.791451	−3.168841	3.086284
O	−2.363890	−1.809265	1.951739
C	−2.817254	−0.524496	1.548353
C	5.394247	0.580388	0.764327
C	−4.090922	−0.765580	0.764739
C	−4.037130	−1.363309	−0.490906
C	−5.195724	−1.622809	−1.206511
C	−6.406217	−1.274103	−0.639396
C	−6.501042	−0.685706	0.604141
C	−5.327219	−0.438977	1.304507
F	−7.543631	−1.521069	−1.332530
O	3.582846	−2.826903	−2.149391
C	2.502182	−3.461453	−2.816655
O	5.659376	−1.631840	−0.898590
C	5.932487	−2.856233	−0.215730
O	−1.505160	3.467765	−0.934876
C	−1.572687	3.697774	−2.342291
O	−3.306301	2.019935	0.570352
C	−4.205349	2.321881	−0.497446
O	0.989744	2.419327	−1.468714
C	1.352464	1.934214	−2.762877
H	1.176001	−1.659412	−1.682714
H	2.559204	2.581508	0.599417
H	1.852321	1.532715	1.819671
H	4.021567	1.078667	2.811734
H	3.910731	4.043272	2.077205
H	3.017224	3.296535	3.412319
H	4.788587	3.336587	3.447420
H	0.939639	−1.447783	1.660977
H	−0.099658	−2.395549	0.607297
H	−0.924272	−1.027281	3.201962
H	−0.929308	−3.964012	2.349988
H	−1.488817	−3.330741	3.909212
H	0.226276	−3.230306	3.476038
H	−3.067317	0.061927	2.443334
H	5.738482	−0.079368	1.574767
H	6.214894	0.680074	0.054223
H	−3.076033	−1.625826	−0.920564
H	−5.168673	−2.085941	−2.185169
H	−7.471104	−0.431032	1.012818
H	−5.380335	0.027578	2.282290
H	1.767575	−3.844762	−2.103046
H	2.936181	−4.290995	−3.369338
H	2.011657	−2.776962	−3.514126
H	6.975179	−3.097353	−0.413353
H	5.786109	−2.734456	0.861802
H	5.292727	−3.659316	−0.586624
H	−2.121871	2.892477	−2.839345
H	−0.576772	3.787943	−2.775945
H	−2.109819	4.635527	−2.474857
H	−3.917605	3.242390	−1.005459
H	−5.186951	2.444695	−0.042785
H	−4.242872	1.495380	−1.212979
H	1.929969	2.726327	−3.236045
H	1.960778	1.030955	−2.693297
H	0.460133	1.727267	−3.361082

ωB97X Energy = −1828.87859887 a.u.

(*aS*,1*R*,3*S*,3'*S*)-**21**, Conf L

C	1.986789	−1.423071	−1.058542
C	3.242980	−2.006118	−1.150784
C	4.330579	−1.369723	−0.541937
C	4.159144	−0.161887	0.117467
C	2.894206	0.422438	0.213113
C	1.810197	−0.216142	−0.384456
C	2.731980	1.726339	0.956036
C	3.940462	2.036225	1.826810
C	3.932011	3.453635	2.352203
O	5.131320	1.861796	1.062320
C	0.108813	1.464536	−1.084268
C	−1.156995	2.051667	−1.004385
C	−2.114071	1.484028	−0.166052
C	−1.816545	0.342346	0.580710
C	−0.529015	−0.187893	0.549653
C	0.436547	0.364242	−0.300790
C	−0.172815	−1.308229	1.496849
C	−1.153621	−1.353567	2.654398
C	−0.946723	−2.545282	3.561076
O	−2.472257	−1.443518	2.121899
C	−2.908369	−0.270528	1.446613
C	5.361715	0.505608	0.744622
C	−4.138290	−0.688311	0.667059
C	−5.403179	−0.461447	1.193001
C	−6.542849	−0.890990	0.526167
C	−6.384534	−1.555742	−0.671593
C	−5.143700	−1.807376	−1.223412
C	−4.019849	−1.368693	−0.540237
F	−7.488619	−1.977293	−1.334575
O	3.509873	−3.167134	−1.803562
C	2.425691	−3.838330	−2.427848
O	5.594789	−1.899336	−0.641117
C	5.815689	−3.049356	0.175928
O	−1.503735	3.119809	−1.786649
C	−0.784621	4.331295	−1.532559
O	−3.383107	1.991692	−0.092504
C	−3.503702	3.296750	0.472741
O	1.050925	2.005193	−1.923433
C	0.884983	1.629189	−3.291430
H	1.126489	−1.895368	−1.516035
H	2.589045	2.541458	0.237101
H	1.834058	1.693450	1.579989
H	3.967826	1.329516	2.670197
H	3.963965	4.162714	1.521709
H	3.022719	3.637853	2.927482
H	4.793249	3.632544	2.997346
H	0.843019	−1.161662	1.872836
H	−0.186327	−2.276183	0.984057

H	−1.069335	−0.424953	3.237949
H	−1.063322	−3.474016	2.997879
H	−1.672240	−2.538461	4.375531
H	0.056770	−2.523669	3.989931
H	−3.212823	0.476200	2.193898
H	5.658326	−0.045082	1.649783
H	6.205498	0.481351	0.055699
H	−5.506194	0.064983	2.136070
H	−7.534778	−0.715685	0.923964
H	−5.066495	−2.332624	−2.167442
H	−3.036686	−1.554166	−0.960071
H	1.667158	−4.129814	−1.696185
H	2.847953	−4.729576	−2.885422
H	1.967553	−3.215262	−3.200766
H	6.854423	−3.339931	0.030730
H	5.649998	−2.808934	1.230499
H	5.160004	−3.869704	−0.122235
H	−1.236513	5.092454	−2.165469
H	0.271263	4.218857	−1.778928
H	−0.887870	4.622075	−0.482958
H	−3.156140	4.062259	−0.222092
H	−2.943035	3.363546	1.409554
H	−4.562744	3.446495	0.674718
H	1.676919	2.124921	−3.849387
H	0.984416	0.545339	−3.403186
H	−0.088495	1.953007	−3.668146

ωB97X Energy = −1828.87851537 a.u.

(*aS*,1*R*,3*S*,3’*S*)-**21**, Conf M

C	1.983269	−1.364857	−1.038586
C	3.242065	−1.935073	−1.147568
C	4.328018	−1.313479	−0.533180
C	4.137135	−0.148722	0.205933
C	2.866337	0.407511	0.334503
C	1.784259	−0.199169	−0.310769
C	2.683805	1.660809	1.155428
C	3.891776	1.946896	2.034959
C	3.859538	3.334826	2.632834
O	5.081255	1.833530	1.256818
C	0.087465	1.515410	−0.932721
C	−1.183747	2.084055	−0.841755
C	−2.155408	1.468226	−0.059758
C	−1.858315	0.297555	0.643574
C	−0.565458	−0.220955	0.603304
C	0.408189	0.371891	−0.211319
C	−0.211467	−1.385456	1.496983
C	−1.205816	−1.502446	2.637441
C	−1.002709	−2.744961	3.474287
O	−2.517969	−1.567206	2.086193
C	−2.951787	−0.358860	1.477971
C	5.330029	0.498548	0.870871
C	−4.176699	−0.724822	0.666090
C	−5.441266	−0.317296	1.067425
C	−6.570148	−0.670587	0.339265
C	−6.402386	−1.446333	−0.788386
C	−5.161827	−1.881153	−1.213946
C	−4.049049	−1.512487	−0.474205
F	−7.495461	−1.797191	−1.507116
O	3.392881	−3.077560	−1.894791
C	3.710588	−4.244365	−1.133682
O	5.592901	−1.845872	−0.594686
C	6.194627	−1.808891	−1.891545
O	−1.471310	3.237434	−1.524307
C	−1.030071	4.417475	−0.849405
O	−3.385115	2.044626	0.110863
C	−4.212906	2.124981	−1.053742
O	1.046158	2.145050	−1.684381
C	0.935638	1.892356	−3.085557
H	1.150195	−1.856228	−1.530273
H	2.515053	2.511156	0.484827
H	1.790977	1.570229	1.781008
H	3.937021	1.198213	2.840262
H	3.876436	4.085846	1.839651
H	2.948564	3.472563	3.218288
H	4.719174	3.495661	3.284791
H	0.797754	−1.249849	1.894337
H	−0.207453	−2.324923	0.933366
H	−1.133772	−0.609652	3.275945
H	−1.109366	−3.638762	2.855356
H	−1.736616	−2.789386	4.279988
H	−0.003791	−2.745379	3.914218
H	−3.255886	0.347510	2.262642
H	5.630616	−0.094824	1.746690
H	6.177737	0.520240	0.185881
H	−5.551634	0.296137	1.955179
H	−7.561791	−0.353878	0.638161
H	−5.077135	−2.490932	−2.104997
H	−3.064856	−1.838682	−0.793959
H	2.925456	−4.443989	−0.398805
H	4.670379	−4.131746	−0.625101
H	3.763510	−5.069844	−1.840689
H	5.620928	−2.403112	−2.605119
H	6.269121	−0.777181	−2.247191
H	7.192899	−2.227279	−1.780504
H	0.053763	4.397317	−0.711302
H	−1.527225	4.511409	0.119902
H	−1.302687	5.260126	−1.481812
H	−5.161809	2.542269	−0.722093
H	−4.382163	1.127560	−1.469057
H	−3.763295	2.772798	−1.806154
H	−0.027975	2.238966	−3.466410
H	1.739913	2.444660	−3.567565
H	1.054624	0.823921	−3.289548

ωB97X Energy = −1828.87836779 a.u.

(*aS*,1*R*,3*S*,3’*S*)-**21**, Conf N

C	−2.036329	1.460252	−1.020492
C	−3.286875	2.061696	−1.048396
C	−4.346773	1.450337	−0.369058
C	−4.145500	0.270151	0.329655

C	−2.882302	−0.324644	0.372550
C	−1.831906	0.274399	−0.318160
C	−2.688306	−1.596915	1.161956
C	−3.834719	−1.839609	2.132227
C	−3.806056	−3.226591	2.732591
O	−5.073975	−1.687122	1.443635
C	−0.157754	−1.390548	−1.129768
C	1.122040	−1.949032	−1.119023
C	2.083564	−1.444171	−0.247529
C	1.806151	−0.348675	0.566893
C	0.522349	0.196348	0.559691
C	−0.459355	−0.315624	−0.295147
C	0.185007	1.286141	1.548710
C	1.169865	1.279572	2.703785
C	0.981906	2.443573	3.649693
O	2.488750	1.367382	2.171767
C	2.905238	0.208370	1.461033
C	−5.308918	−0.344873	1.073132
C	4.152758	0.623850	0.708774
C	5.406117	0.346480	1.237967
C	6.563066	0.769740	0.597161
C	6.433662	1.480524	−0.577284
C	5.204995	1.784112	−1.130545
C	4.063646	1.350448	−0.473942
F	7.554536	1.896545	−1.215148
O	−3.570122	3.225286	−1.689394
C	−2.510860	3.877675	−2.373786
O	−5.589183	2.037627	−0.334470
C	−6.315779	1.942121	−1.560523
O	1.448994	−2.991202	−1.948236
C	1.736982	−2.579882	−3.285794
O	3.340309	−1.989418	−0.233688
C	3.411362	−3.288373	0.357826
O	−1.127235	−1.829920	−1.984666
C	−1.400980	−3.232729	−2.026328
H	−1.197920	1.910972	−1.536396
H	−2.620515	−2.452077	0.479129
H	−1.744438	−1.558053	1.713355
H	−3.792456	−1.087671	2.934701
H	−3.906139	−3.978680	1.946422
H	−2.860142	−3.393783	3.251152
H	−4.620702	−3.356385	3.446288
H	−0.830917	1.142050	1.925117
H	0.209302	2.269893	1.067163
H	1.073202	0.333546	3.256808
H	1.110883	3.388405	3.116808
H	1.708893	2.399502	4.461659
H	−0.020993	2.422501	4.079887
H	3.185608	−0.568642	2.187026
H	−5.530613	0.256322	1.966781
H	−6.203359	−0.342111	0.449865
H	5.486400	−0.215420	2.162638
H	7.546241	0.555036	0.997384
H	5.150868	2.344816	−2.055631
H	3.089673	1.575600	−0.895754
H	−2.106266	3.246896	−3.169877
H	−1.708985	4.156418	−1.684676
H	−2.942562	4.775898	−2.808326
H	−7.277348	2.423370	−1.392280
H	−5.786530	2.451696	−2.367759
H	−6.476595	0.893429	−1.828988
H	0.860005	−2.115925	−3.746356
H	2.007630	−3.477914	−3.837660
H	2.573075	−1.875684	−3.296997
H	4.460371	−3.577653	0.342784
H	2.821547	−4.007350	−0.213310
H	3.057840	−3.255240	1.392743
H	−1.212880	−3.695155	−1.054706
H	−2.456409	−3.331400	−2.276788
H	−0.794688	−3.731927	−2.782676

ωB97X Energy = −1828.87834418 a.u.

(*aS*,1*R*,3*S*,3’*S*)-**21**, Conf O

C	2.037189	−1.100359	−1.256921
C	3.299120	−1.648482	−1.442352
C	4.353264	−1.204373	−0.637120
C	4.142471	−0.223016	0.320221
C	2.877835	0.344463	0.490736
C	1.825734	−0.108998	−0.301101
C	2.676114	1.409510	1.541944
C	3.994847	2.056080	1.950600
C	4.516257	3.044879	0.917015
O	4.943944	1.030814	2.257898
C	0.106956	1.636600	−0.759955
C	−1.170561	2.179010	−0.613592
C	−2.128139	1.488319	0.122410
C	−1.811544	0.266803	0.723541
C	−0.513286	−0.230671	0.627892
C	0.445675	0.440517	−0.140571
C	−0.137385	−1.469675	1.403477
C	−1.116449	−1.707178	2.538524
C	−0.894815	−3.026260	3.242938
O	−2.435253	−1.727675	1.999728
C	−2.886568	−0.471234	1.512390
C	5.318760	0.246607	1.143153
C	−4.124708	−0.774383	0.695036
C	−4.014304	−1.450696	−0.516297
C	−5.139878	−1.766773	−1.261161
C	−6.375402	−1.393216	−0.768141
C	−6.525946	−0.728188	0.430456
C	−5.384416	−0.426065	1.161856
F	−7.481021	−1.694280	−1.490230
O	3.600597	−2.594976	−2.368893
C	2.551577	−3.055208	−3.207501
O	5.627418	−1.686680	−0.816759
C	5.821924	−3.026229	−0.360669
O	−1.475693	3.382584	−1.194380
C	−1.066227	4.505446	−0.410769
O	−3.361662	2.036086	0.351731
C	−4.203608	2.200194	−0.793713
O	1.056682	2.345194	−1.450593

C	0.949635	2.222943	−2.869499
H	1.201064	−1.429963	−1.860788
H	1.991440	2.177195	1.173983
H	2.206956	0.965670	2.426573
H	3.856369	2.580992	2.897268
H	4.578627	2.603241	−0.080353
H	3.839757	3.899626	0.859795
H	5.505574	3.408707	1.200975
H	0.875403	−1.362335	1.800388
H	−0.131189	−2.349335	0.750094
H	−1.041983	−0.882372	3.262451
H	−1.002911	−3.852458	2.536488
H	−1.618924	−3.158281	4.047939
H	0.109271	−3.063756	3.669314
H	−3.179917	0.157652	2.364430
H	5.865665	−0.608572	1.541648
H	6.015443	0.799286	0.499133
H	−3.034097	−1.730453	−0.888014
H	−5.068489	−2.289789	−2.206835
H	−7.514209	−0.457369	0.781125
H	−5.481784	0.099235	2.105835
H	2.997651	−3.792906	−3.869957
H	2.134712	−2.237860	−3.802357
H	1.755282	−3.525284	−2.623911
H	5.194728	−3.722467	−0.919975
H	6.871150	−3.262066	−0.527971
H	5.598437	−3.103837	0.707805
H	−1.350329	5.396722	−0.966844
H	0.016565	4.494525	−0.262917
H	−1.574317	4.500529	0.557357
H	−5.146481	2.596594	−0.421687
H	−4.381795	1.234459	−1.274714
H	−3.761289	2.897669	−1.504994
H	1.734477	2.843719	−3.297117
H	1.100957	1.182791	−3.174092
H	−0.025618	2.574036	−3.214824

ωB97X Energy = −1828.87825231 a.u.

(*aS*,1*R*,3*S*,3’*S*)-**21**, Conf P

C	1.997646	−1.354768	−1.057329
C	3.265328	−1.902508	−1.175872
C	4.339846	−1.282409	−0.541455
C	4.142283	−0.103695	0.172885
C	2.866267	0.442487	0.293857
C	1.786783	−0.189799	−0.331216
C	2.673297	1.702826	1.101318
C	3.873719	1.999778	1.987435
C	3.828612	3.389240	2.580925
O	5.068226	1.892451	1.216516
C	0.063848	1.494647	−0.961548
C	−1.212438	2.050650	−0.865465
C	−2.168590	1.437047	−0.062978
C	−1.851856	0.279467	0.653309
C	−0.554303	−0.226725	0.605431
C	0.404389	0.364951	−0.227425
C	−0.178702	−1.375703	1.510163
C	−1.158978	−1.487296	2.663450
C	−0.933686	−2.716166	3.514592
O	−2.476611	−1.571754	2.128174
C	−2.928523	−0.374631	1.511162
C	5.329860	0.559654	0.832218
C	−4.162247	−0.759460	0.721729
C	−5.423468	−0.352855	1.134446
C	−6.560225	−0.722710	0.427184
C	−6.403668	−1.514448	−0.690927
C	−5.166707	−1.949052	−1.127018
C	−4.045884	−1.563520	−0.408247
F	−7.504555	−1.881756	−1.389232
O	3.432326	−3.066399	−1.886158
C	4.101513	−2.893463	−3.136683
O	5.615853	−1.779818	−0.648799
C	5.839019	−3.001758	0.058860
O	−1.518797	3.191111	−1.561486
C	−1.097817	4.385617	−0.899398
O	−3.401176	2.004916	0.114952
C	−4.243826	2.063451	−1.040273
O	1.008042	2.125104	−1.730575
C	0.887695	1.852937	−3.127183
H	1.170217	−1.853495	−1.551248
H	2.507537	2.546789	0.421957
H	1.776112	1.614884	1.720824
H	3.918839	1.253711	2.795222
H	3.844826	4.138016	1.785611
H	2.913489	3.522447	3.160919
H	4.683465	3.557978	3.237173
H	0.833539	−1.225127	1.894237
H	−0.171634	−2.322262	0.958642
H	−1.088457	−0.585255	3.289020
H	−1.037152	−3.619550	2.909197
H	−1.658446	−2.757325	4.328723
H	0.069809	−2.699637	3.943657
H	−3.226602	0.339342	2.291258
H	5.640976	−0.028800	1.708249
H	6.172799	0.588783	0.142311
H	−5.524973	0.273396	2.014282
H	−7.549369	−0.406780	0.735040
H	−5.091005	−2.572109	−2.009658
H	−3.064426	−1.889485	−0.736505
H	3.522496	−2.232410	−3.788094
H	4.172422	−3.879265	−3.592043
H	5.102909	−2.482761	−2.990293
H	6.886503	−3.257089	−0.087980
H	5.644335	−2.864469	1.126441
H	5.205140	−3.799769	−0.332317
H	−0.013829	4.385079	−0.760388
H	−1.597102	4.481991	0.068567
H	−1.383672	5.216666	−1.541239
H	−3.808978	2.705649	−1.806054
H	−5.192247	2.476533	−0.702043
H	−4.409341	1.059470	−1.441066
H	−0.084789	2.179981	−3.502735

H	1.678631	2.411205	−3.624179
H	1.020928	0.783848	−3.319069

ωB97X Energy = −1828.87820610 a.u.

(*aS*,1*R*,3*S*,3'*S*)-**21**, Conf Q

C	2.031629	−1.289687	−1.257082
C	3.293922	−1.853614	−1.382031
C	4.362044	−1.283955	−0.680205
C	4.165584	−0.160409	0.107984
C	2.894470	0.403974	0.236395
C	1.830062	−0.167433	−0.456564
C	2.705085	1.605877	1.129882
C	3.881023	1.794849	2.076499
C	3.846093	3.126788	2.790490
O	5.096105	1.730280	1.333373
C	0.098537	1.512325	−1.094181
C	−1.189090	2.044688	−0.999900
C	−2.108699	1.465572	−0.129697
C	−1.781826	0.326088	0.602029
C	−0.490417	−0.192246	0.512706
C	0.449341	0.391922	−0.342789
C	−0.098274	−1.334402	1.418478
C	−1.033877	−1.408376	2.611841
C	−0.787121	−2.618789	3.483256
O	−2.372313	−1.492678	2.130291
C	−2.839411	−0.310393	1.492800
C	5.348088	0.431460	0.840407
C	−4.091299	−0.723010	0.745754
C	−4.004321	−1.402095	−0.465182
C	−5.145639	−1.835022	−1.122116
C	−6.372191	−1.579031	−0.540688
C	−6.499313	−0.915763	0.661463
C	−5.342198	−0.492354	1.302109
F	−7.493246	−1.995028	−1.178195
O	3.584744	−2.935023	−2.150555
C	2.520579	−3.540985	−2.869010
O	5.632211	−1.794005	−0.806463
C	5.844535	−3.018740	−0.103801
O	−1.563659	3.133010	−1.745434
C	−1.888006	2.805999	−3.097987
O	−3.373739	1.982806	−0.034396
C	−3.446510	3.233533	0.652678
O	1.027146	2.026122	−1.953083
C	1.281378	3.432320	−1.902888
H	1.185654	−1.712133	−1.784456
H	2.596282	2.511987	0.522154
H	1.782665	1.502260	1.708542
H	3.884320	0.978704	2.814758
H	3.904940	3.941997	2.065496
H	2.915278	3.230626	3.351199
H	4.682127	3.215772	3.485432
H	0.931340	−1.198781	1.759188
H	−0.133170	−2.288801	0.881585
H	−0.929485	−0.492188	3.211594
H	−0.921522	−3.535452	2.904476
H	−1.480720	−2.633627	4.324999
H	0.232064	−2.601642	3.873613
H	−3.124584	0.419138	2.264498
H	5.637358	−0.232947	1.668322
H	6.203477	0.507431	0.169471
H	−3.031926	−1.590954	−0.907976
H	−5.093063	−2.359676	−2.068181
H	−7.480785	−0.737281	1.083033
H	−5.420849	0.032809	2.248228
H	2.959731	−4.377962	−3.406266
H	2.078099	−2.841912	−3.583833
H	1.746193	−3.910521	−2.191208
H	6.885507	−3.293389	−0.263377
H	5.665259	−2.882468	0.967094
H	5.194074	−3.806328	−0.489188
H	−2.716414	2.093408	−3.129957
H	−1.020418	2.384078	−3.613376
H	−2.185213	3.734268	−3.581898
H	−3.046285	3.134819	1.666247
H	−4.501154	3.496915	0.704435
H	−2.898375	4.006765	0.111469
H	2.326146	3.565016	−2.180787
H	0.642938	3.975250	−2.600424
H	1.120702	3.821443	−0.894928

ωB97X Energy = −1828.87811651 a.u.

(*aS*,1*R*,3*S*,3'*S*)-**21**, Conf R

C	2.003043	−1.277529	−1.072733
C	3.248734	−1.878317	−1.193798
C	4.307478	−1.400156	−0.414802
C	4.107210	−0.365416	0.486839
C	2.854321	0.239639	0.607600
C	1.805170	−0.224079	−0.182437
C	2.665584	1.362461	1.599394
C	3.994851	1.998977	1.989145
C	4.555633	2.912746	0.908375
O	4.912282	0.969863	2.369635
C	0.154208	1.547101	−0.773638
C	−1.108727	2.135062	−0.688437
C	−2.106884	1.509419	0.051864
C	−1.843579	0.311479	0.722018
C	−0.558765	−0.226982	0.690044
C	0.439989	0.375170	−0.084926
C	−0.241772	−1.432131	1.541996
C	−1.258640	−1.575493	2.659251
C	−1.095098	−2.854827	3.447893
O	−2.561827	−1.592964	2.083369
C	−2.963678	−0.356141	1.510773
C	5.280809	0.109308	1.311032
C	−4.177830	−0.674503	0.663344
C	−5.443985	−0.262158	1.054422
C	−6.563433	−0.573844	0.293361
C	−6.384890	−1.312778	−0.857107
C	−5.142542	−1.751056	−1.273913
C	−4.039299	−1.424302	−0.500966

F	−7.468741	−1.623291	−1.607650
O	3.528236	−2.918337	−2.021481
C	2.471260	−3.431205	−2.818487
O	5.547858	−1.987773	−0.481811
C	6.279341	−1.683412	−1.669801
O	−1.363012	3.316411	−1.335466
C	−0.898896	4.463641	−0.620329
O	−3.331232	2.099992	0.214310
C	−4.131514	2.226473	−0.965251
O	1.142534	2.187589	−1.476978
C	1.062421	1.994992	−2.889784
H	1.165342	−1.624803	−1.664517
H	2.002777	2.125385	1.184600
H	2.177895	0.975972	2.500736
H	3.857156	2.581260	2.901776
H	4.618643	2.413272	−0.061347
H	3.903649	3.780545	0.792891
H	5.550812	3.265970	1.185174
H	0.762541	−1.332015	1.961565
H	−0.244808	−2.347459	0.939589
H	−1.181192	−0.710824	3.334736
H	−1.210807	−3.719726	2.790706
H	−1.842556	−2.916094	4.239921
H	−0.103540	−2.895264	3.902367
H	−3.272447	0.326721	2.314256
H	5.783024	−0.742775	1.769149
H	6.013633	0.598128	0.654155
H	−5.563030	0.321425	1.960941
H	−7.556166	−0.253169	0.584316
H	−5.049032	−2.330890	−2.183870
H	−3.053853	−1.754455	−0.812737
H	2.897817	−4.251187	−3.390948
H	2.087231	−2.670582	−3.503854
H	1.655127	−3.808864	−2.196522
H	6.422067	−0.602797	−1.766681
H	7.248927	−2.167906	−1.571341
H	5.765031	−2.067645	−2.552644
H	0.183052	4.412989	−0.476018
H	−1.400749	4.538409	0.348200
H	−1.147124	5.332394	−1.226943
H	−5.078056	2.657336	−0.644413
H	−4.313427	1.242992	−1.407562
H	−3.651442	2.881597	−1.692135
H	0.112447	2.369817	−3.278030
H	1.884530	2.556329	−3.329379
H	1.173378	0.934447	−3.135544

ωB97X Energy = −1828.87810776 a.u.

(*aR*,1*R*,3*S*,3’*S*)-**21**, Conf A

C	2.951226	1.171189	0.723162
C	4.280921	0.776586	0.731216
C	4.644790	−0.391132	0.048981
C	3.692725	−1.133791	−0.630266
C	2.354483	−0.728787	−0.650177
C	1.992114	0.426255	0.039426
C	1.343834	−1.556804	−1.410187
C	2.013455	−2.510033	−2.389320
C	1.052760	−3.529968	−2.956613
O	3.054769	−3.217027	−1.720234
C	0.161401	1.905850	−0.783713
C	−1.164709	2.343678	−0.783902
C	−2.095606	1.713839	0.035168
C	−1.704160	0.665200	0.872485
C	−0.365466	0.283135	0.917005
C	0.569204	0.880036	0.060266
C	0.075135	−0.736587	1.939121
C	−0.934606	−0.827582	3.068448
C	−0.633814	−1.943116	4.043408
O	−2.217467	−1.086821	2.505679
C	−2.752341	−0.009476	1.748969
C	4.132294	−2.370710	−1.379385
C	−3.894752	−0.601543	0.950256
C	−5.212903	−0.296107	1.258279
C	−6.262219	−0.857976	0.541999
C	−5.959811	−1.736165	−0.477230
C	−4.661061	−2.074064	−0.806695
C	−3.630260	−1.497267	−0.081858
F	−6.974102	−2.291263	−1.182358
O	5.283046	1.450628	1.352143
C	4.946752	2.643976	2.044120
O	5.959353	−0.787408	−0.009944
C	6.463591	−1.336710	1.208498
O	−1.545713	3.382497	−1.592861
C	−1.264211	4.667394	−1.034091
O	−3.383586	2.172172	0.107847
C	−4.171010	2.025088	−1.078192
O	1.074907	2.558560	−1.571629
C	1.043205	2.173730	−2.945535
H	2.640896	2.071329	1.238094
H	0.737515	−2.146002	−0.711662
H	0.650278	−0.908446	−1.952344
H	2.462823	−1.928434	−3.208802
H	0.644676	−4.148379	−2.153714
H	0.224536	−3.030206	−3.462523
H	1.557569	−4.178253	−3.674014
H	1.054744	−0.460125	2.335868
H	0.182981	−1.727788	1.483911
H	−0.969328	0.133061	3.602876
H	−0.637028	−2.905936	3.527297
H	−1.380897	−1.970750	4.837618
H	0.348597	−1.795618	4.495574
H	−3.161712	0.742203	2.437802
H	4.675736	−2.072591	−2.287626
H	4.815886	−2.962307	−0.770368
H	−5.430074	0.401406	2.060042
H	−7.295188	−0.623607	0.767671
H	−4.469777	−2.771759	−1.612679
H	−2.602365	−1.744708	−0.326765
H	4.528506	3.389891	1.362896
H	5.876208	3.019865	2.464649

H	4.236188	2.446614	2.851355
H	6.431441	−0.597920	2.011069
H	5.887332	−2.221273	1.497019
H	7.495699	−1.624168	1.017298
H	−1.616692	5.404963	−1.752388
H	−0.189800	4.790889	−0.878617
H	−1.794599	4.796318	−0.086706
H	−4.269849	0.967524	−1.338315
H	−3.730193	2.577027	−1.908151
H	−5.153079	2.431013	−0.843195
H	1.337439	1.125472	−3.057996
H	0.048279	2.327233	−3.369779
H	1.761932	2.805207	−3.464046

ωB97X Energy = −1828.88072464 a.u.

(*aR*,1*R*,3*S*,3’*S*)-**21**, Conf B

C	2.932838	1.097209	0.845630
C	4.248217	0.667329	0.940300
C	4.614733	−0.533200	0.319638
C	3.671871	−1.293204	−0.353647
C	2.344977	−0.862015	−0.446255
C	1.985078	0.340003	0.159394
C	1.346792	−1.708223	−1.202338
C	2.035505	−2.718149	−2.108288
C	1.077056	−3.741231	−2.673789
O	3.027617	−3.417537	−1.361268
C	0.227994	1.811367	−0.823889
C	−1.084238	2.282969	−0.901603
C	−2.062916	1.726014	−0.085447
C	−1.733390	0.717995	0.825069
C	−0.408491	0.305546	0.945363
C	0.574858	0.828135	0.094513
C	−0.037221	−0.665677	2.039482
C	−1.090965	−0.665858	3.131584
C	−0.858651	−1.732588	4.177313
O	−2.358419	−0.922788	2.533777
C	−2.832831	0.122303	1.695970
C	4.110390	−2.584230	−1.006484
C	−3.960734	−0.484555	0.887603
C	−3.683988	−1.436592	−0.089456
C	−4.704045	−2.026039	−0.819404
C	−6.004522	−1.643731	−0.550989
C	−6.319205	−0.709340	0.413018
C	−5.280785	−0.135482	1.135607
F	−7.008048	−2.210727	−1.262076
O	5.231986	1.328691	1.602861
C	4.893351	2.552207	2.238590
O	5.901350	−1.005640	0.418358
C	6.847592	−0.304831	−0.389413
O	−1.405010	3.283289	−1.782190
C	−1.110846	4.590277	−1.284460
O	−3.339322	2.220830	−0.089926
C	−4.083834	2.030665	−1.297361
O	1.189637	2.388814	−1.613220
C	1.180505	1.940725	−2.968460
H	2.621731	2.025174	1.308041
H	0.705121	−2.254043	−0.500170
H	0.686047	−1.074560	−1.799941
H	2.534925	−2.183614	−2.931004
H	0.614650	−4.310043	−1.863721
H	0.287689	−3.246932	−3.243212
H	1.599218	−4.435042	−3.333923
H	0.934412	−0.394192	2.458547
H	0.058670	−1.683404	1.644036
H	−1.118652	0.323809	3.610443
H	−0.872146	−2.722120	3.714787
H	−1.633601	−1.694993	4.943895
H	0.111356	−1.587367	4.656189
H	−3.247122	0.923083	2.323785
H	4.713127	−2.355882	−1.898236
H	4.739415	−3.156052	−0.324924
H	−2.654835	−1.718097	−0.287926
H	−4.503088	−2.766379	−1.583891
H	−7.353144	−0.441746	0.592602
H	−5.507429	0.605324	1.894765
H	4.537153	3.288971	1.513530
H	5.808345	2.913676	2.701340
H	4.132356	2.400068	3.008887
H	7.808911	−0.792051	−0.237845
H	6.571767	−0.368592	−1.446414
H	6.916207	0.742386	−0.088675
H	−1.667088	4.781941	−0.362875
H	−1.423594	5.295638	−2.051807
H	−0.039174	4.698718	−1.101145
H	−5.064233	2.469264	−1.121600
H	−4.197656	0.963663	−1.508061
H	−3.598510	2.529981	−2.135859
H	1.944537	2.512175	−3.491799
H	1.426617	0.875475	−3.022411
H	0.207176	2.120251	−3.430602

ωB97X Energy = −1828.88052036 a.u.

(*aR*,1*R*,3*S*,3’*S*)-**21**, Conf C

C	−2.909739	0.952748	−0.979392
C	−4.246973	0.582365	−0.951859
C	−4.660718	−0.393223	−0.037136
C	−3.750249	−0.967146	0.836457
C	−2.408874	−0.576066	0.824500
C	−1.995025	0.382087	−0.096525
C	−1.442126	−1.200006	1.802997
C	−2.164963	−1.919609	2.932448
C	−1.241540	−2.791266	3.752538
O	−3.183694	−2.755461	2.387126
C	−0.143784	1.919860	0.546170
C	1.185416	2.351300	0.486768
C	2.093297	1.642876	−0.296027
C	1.693020	0.491791	−0.980843
C	0.360266	0.092457	−0.942587
C	−0.562830	0.802617	−0.165476
C	−0.083632	−1.079719	−1.782847

C	0.910926	−1.345432	−2.898312
C	0.610978	−2.611981	−3.667206
O	2.206892	−1.488409	−2.323963
C	2.733605	−0.288039	−1.774977
C	−4.237070	−2.001558	1.824643
C	3.924668	−0.709928	−0.939235
C	5.218664	−0.471030	−1.381055
C	6.315974	−0.883924	−0.636445
C	6.085845	−1.547137	0.550527
C	4.813702	−1.813114	1.018928
C	3.733554	−1.388408	0.260649
F	7.147790	−1.954144	1.286591
O	−5.208796	1.106688	−1.754391
C	−4.821902	2.110983	−2.680235
O	−5.983586	−0.755332	0.051196
C	−6.444506	−1.564624	−1.031778
O	1.588275	3.499793	1.115675
C	1.598954	3.438084	2.545439
O	3.373798	2.080573	−0.486745
C	4.213034	2.198999	0.662919
O	−1.042114	2.595121	1.333777
C	−1.489266	3.839671	0.792592
H	−2.560547	1.693619	−1.687427
H	−0.794896	−1.917828	1.284010
H	−0.786500	−0.432451	2.222929
H	−2.645363	−1.174038	3.583863
H	−0.805621	−3.571227	3.123832
H	−0.430612	−2.192748	4.171817
H	−1.782811	−3.265280	4.572378
H	−1.071301	−0.881048	−2.204886
H	−0.174710	−1.983087	−1.168096
H	0.921730	−0.487743	−3.586910
H	0.631000	−3.474015	−2.996484
H	1.348213	−2.766231	−4.456181
H	−0.378678	−2.550701	−4.123461
H	3.094136	0.350862	−2.593098
H	−4.809260	−1.505402	2.621934
H	−4.906028	−2.710221	1.336288
H	5.378335	0.055338	−2.315989
H	7.330355	−0.698514	−0.967502
H	4.679663	−2.338517	1.956480
H	2.725927	−1.583984	0.612999
H	−5.728965	2.401388	−3.204538
H	−4.093154	1.727513	−3.399611
H	−4.404418	2.981742	−2.166888
H	−6.353620	−1.033711	−1.981009
H	−5.879908	−2.501060	−1.077048
H	−7.492113	−1.783412	−0.833997
H	1.981251	4.396540	2.891167
H	2.262135	2.637923	2.886997
H	0.594211	3.275023	2.937341
H	4.004062	3.119395	1.208061
H	5.236496	2.215597	0.291437
H	4.086782	1.335700	1.322492
H	−0.651551	4.524580	0.644139
H	−2.008610	3.677656	−0.156442
H	−2.184182	4.260090	1.516843

ωB97X Energy = −1828.88029948 a.u.

(*aR*,1*R*,3*S*,3’*S*)-**21**, Conf D

C	2.911741	0.947250	0.981735
C	4.229389	0.514543	1.039420
C	4.615168	−0.585683	0.265118
C	3.690233	−1.243441	−0.530811
C	2.366784	−0.800371	−0.597352
C	1.985097	0.298132	0.168447
C	1.387889	−1.516165	−1.498421
C	2.095777	−2.420265	−2.497228
C	1.147477	−3.356310	−3.210878
O	3.060980	−3.215638	−1.812721
C	0.209692	1.819791	−0.694661
C	−1.105671	2.294835	−0.726067
C	−2.058939	1.707188	0.101817
C	−1.716337	0.631022	0.925667
C	−0.396822	0.191448	0.976131
C	0.570351	0.777864	0.150500
C	−0.013537	−0.882708	1.964515
C	−1.049520	−0.986299	3.068536
C	−0.811366	−2.156699	3.995262
O	−2.330536	−1.164946	2.471332
C	−2.805317	−0.027151	1.764513
C	4.140233	−2.440421	−1.336302
C	−3.970784	−0.521494	0.932307
C	−5.275430	−0.180695	1.260831
C	−6.347330	−0.656381	0.516356
C	−6.081498	−1.486193	−0.552444
C	−4.798057	−1.857437	−0.904275
C	−3.743880	−1.366899	−0.149830
F	−7.118230	−1.956942	−1.286340
O	5.194209	1.080366	1.809305
C	4.835965	2.208782	2.593038
O	5.900635	−1.067638	0.327736
C	6.861881	−0.265354	−0.358735
O	−1.453815	3.376175	−1.491729
C	−1.420224	3.160300	−2.906025
O	−3.329289	2.202812	0.198509
C	−4.132020	2.196913	−0.982952
O	1.152847	2.374169	−1.523785
C	1.621586	3.659684	−1.111941
H	2.583786	1.788271	1.579416
H	0.700364	−2.125081	−0.898378
H	0.775144	−0.789563	−2.038802
H	2.624973	−1.797091	−3.233867
H	0.659612	−4.017844	−2.491126
H	0.376764	−2.787667	−3.734552
H	1.683122	−3.967188	−3.938719
H	0.965616	−0.658834	2.393734
H	0.071868	−1.855840	1.466679
H	−1.056442	−0.051084	3.647566
H	−0.843889	−3.094256	3.435524
H	−1.572236	−2.189975	4.776043

H	0.168348	−2.069478	4.468446
H	−3.181380	0.710420	2.486908
H	4.760600	−2.103972	−2.180360
H	4.754964	−3.096128	−0.720321
H	−5.463486	0.477646	2.102196
H	−7.369692	−0.393220	0.758143
H	−4.636000	−2.514925	−1.749567
H	−2.727348	−1.642887	−0.410954
H	4.483786	3.030914	1.963558
H	5.741002	2.513145	3.112888
H	4.065206	1.952658	3.324907
H	6.932885	0.726983	0.090831
H	7.818287	−0.776466	−0.266232
H	6.600385	−0.173416	−1.417302
H	−1.790907	4.075282	−3.363954
H	−2.072879	2.326264	−3.180240
H	−0.404391	2.958639	−3.247629
H	−4.048808	1.235686	−1.498282
H	−3.848951	3.007213	−1.654529
H	−5.160042	2.335994	−0.652717
H	2.115436	3.590920	−0.138262
H	2.343153	3.981067	−1.860392
H	0.799783	4.377169	−1.060388

ωB97X Energy = −1828.88011080 a.u.

(*aR*,1*R*,3*S*,3'*S*)-**21**, Conf E

C	2.937562	1.110676	0.688356
C	4.264770	0.704975	0.718525
C	4.633841	−0.454807	0.026972
C	3.690644	−1.175845	−0.688489
C	2.359895	−0.753385	−0.738565
C	1.989873	0.389719	−0.035181
C	1.356270	−1.544910	−1.543715
C	2.032821	−2.520378	−2.496552
C	1.066855	−3.526048	−3.080121
O	3.044096	−3.242819	−1.797712
C	0.149277	1.806700	−0.942456
C	−1.173688	2.259945	−0.944131
C	−2.094006	1.670658	−0.079322
C	−1.691628	0.681923	0.821986
C	−0.353329	0.302245	0.874916
C	0.566672	0.844533	−0.030211
C	0.097207	−0.663263	1.943531
C	−0.897271	−0.685448	3.089515
C	−0.587872	−1.744567	4.123023
O	−2.188466	−0.973041	2.560681
C	−2.733130	0.049926	1.737888
C	4.128288	−2.413377	−1.437022
C	−3.866741	−0.606728	0.976899
C	−5.189249	−0.298245	1.262209
C	−6.229910	−0.918604	0.582452
C	−5.914435	−1.858042	−0.376363
C	−4.610848	−2.200699	−0.680753
C	−3.588859	−1.565457	0.006779
F	−6.920258	−2.470444	−1.045444
O	5.256598	1.360428	1.375073
C	4.914484	2.550493	2.069531
O	5.944496	−0.867124	−0.003208
C	6.412275	−1.434605	1.221401
O	−1.533425	3.230031	−1.841262
C	−1.975417	4.462805	−1.265659
O	−3.399049	2.089161	−0.050414
C	−4.160365	1.844318	−1.237348
O	1.034745	2.275850	−1.877666
C	1.557552	3.577705	−1.624204
H	2.621674	1.995029	1.227186
H	0.697691	−2.108793	−0.871470
H	0.716087	−0.867351	−2.115012
H	2.514461	−1.955030	−3.308620
H	0.628425	−4.130932	−2.282894
H	0.260175	−3.014077	−3.608177
H	1.575730	−4.188584	−3.781483
H	1.083699	−0.373208	2.312010
H	0.193114	−1.677299	1.537529
H	−0.921446	0.304484	3.568073
H	−0.597914	−2.734720	3.661783
H	−1.326946	−1.725874	4.924949
H	0.399304	−1.574063	4.556369
H	−3.153540	0.837604	2.377891
H	4.689998	−2.119199	−2.335422
H	4.793848	−3.016346	−0.819222
H	−5.416709	0.447218	2.016549
H	−7.266190	−0.682952	0.790999
H	−4.409064	−2.946534	−1.439662
H	−2.557427	−1.815799	−0.219711
H	4.511815	3.303270	1.385987
H	5.838323	2.918784	2.508673
H	4.188952	2.351680	2.862995
H	7.442770	−1.739664	1.049762
H	6.376349	−0.701343	2.028886
H	5.814130	−2.310632	1.490844
H	−2.940951	4.346363	−0.771006
H	−2.063042	5.170490	−2.087571
H	−1.241853	4.832246	−0.543282
H	−4.171022	0.775699	−1.467313
H	−3.758128	2.400256	−2.085652
H	−5.173810	2.177148	−1.022201
H	2.276873	3.782654	−2.414728
H	0.769425	4.333029	−1.649275
H	2.066333	3.607272	−0.656069

ωB97X Energy = −1828.88000819 a.u.

(*aR*,1*R*,3*S*,3'*S*)-**21**, Conf F

C	2.923214	1.031550	0.810463
C	4.235272	0.591287	0.918393
C	4.602487	−0.603344	0.288539
C	3.665064	−1.344929	−0.413634
C	2.346532	−0.897522	−0.528331
C	1.983645	0.295203	0.091816
C	1.352166	−1.710749	−1.323203

C	2.040376	−2.742575	−2.205292
C	1.073964	−3.752119	−2.781445
O	3.005797	−3.454673	−1.435094
C	0.222948	1.713382	−0.959113
C	−1.085436	2.200683	−1.039970
C	−2.056628	1.680121	−0.186706
C	−1.719886	0.728034	0.778669
C	−0.396556	0.315147	0.906284
C	0.574783	0.787058	0.015585
C	−0.018164	−0.607579	2.038686
C	−1.057907	−0.546681	3.142341
C	−0.820727	−1.564503	4.234792
O	−2.334764	−0.823357	2.574383
C	−2.815129	0.172524	1.681444
C	4.096525	−2.636796	−1.068790
C	−3.938516	−0.488308	0.909072
C	−3.653547	−1.494603	−0.009333
C	−4.668492	−2.133258	−0.704287
C	−5.972052	−1.745545	−0.459740
C	−6.294698	−0.758164	0.447122
C	−5.261274	−0.135115	1.135347
F	−6.970665	−2.360853	−1.136876
O	5.211992	1.236988	1.606410
C	4.874548	2.462776	2.238029
O	5.882645	−1.089186	0.407026
C	6.847678	−0.394976	−0.383819
O	−1.380045	3.132105	−1.999609
C	−1.812634	4.403806	−1.507295
O	−3.348924	2.135187	−0.231685
C	−4.071669	1.853645	−1.434460
O	1.159401	2.111348	−1.877443
C	1.704195	3.412325	−1.669969
H	2.609499	1.947005	1.295959
H	0.664722	−2.231365	−0.645067
H	0.741115	−1.051554	−1.945514
H	2.565855	−2.224848	−3.022061
H	0.587474	−4.307423	−1.976078
H	0.303493	−3.247109	−3.367111
H	1.594637	−4.459197	−3.428602
H	0.960814	−0.326948	2.433500
H	0.063967	−1.642867	1.686937
H	−1.071545	0.464991	3.573455
H	−0.846105	−2.574509	3.819444
H	−1.586970	−1.485567	5.007032
H	0.155400	−1.403920	4.696052
H	−3.235855	1.003499	2.263990
H	4.709136	−2.412528	−1.954856
H	4.712732	−3.218467	−0.383875
H	−2.622162	−1.779849	−0.190127
H	−4.461106	−2.915048	−1.424526
H	−7.330795	−0.488534	0.610532
H	−5.494134	0.646971	1.849931
H	4.530153	3.201182	1.508483
H	5.786903	2.819150	2.709823
H	4.104879	2.316383	3.000791
H	7.802545	−0.890166	−0.217825
H	6.588575	−0.454129	−1.445294
H	6.920157	0.651126	−0.079743
H	−2.796554	4.335204	−1.040652
H	−1.856154	5.066601	−2.369181
H	−1.095076	4.796422	−0.781048
H	−5.077678	2.238800	−1.280423
H	−4.118249	0.775056	−1.605786
H	−3.613552	2.344419	−2.294533
H	2.461147	3.557594	−2.438316
H	0.937375	4.183337	−1.769139
H	2.172033	3.482014	−0.683373

ωB97X Energy = −1828.87974789 a.u.

(*aR*,1*R*,3*S*,3’*S*)-**21**, Conf G

C	2.901418	1.162355	0.699907
C	4.238034	0.793921	0.765555
C	4.650469	−0.374753	0.114691
C	3.740620	−1.140874	−0.596910
C	2.400085	−0.756318	−0.683143
C	1.986835	0.395363	−0.019160
C	1.435404	−1.594651	−1.488685
C	2.159636	−2.568486	−2.406969
C	1.235132	−3.610710	−2.993673
O	3.172958	−3.249788	−1.670812
C	0.141292	1.753664	−0.999580
C	−1.186437	2.181474	−1.038144
C	−2.101342	1.654646	−0.132455
C	−1.715258	0.670454	0.777276
C	−0.378550	0.281806	0.839280
C	0.555085	0.820744	−0.054798
C	0.055751	−0.674517	1.922781
C	−0.946725	−0.676736	3.062878
C	−0.652241	−1.728515	4.108152
O	−2.238149	−0.957827	2.530094
C	−2.767507	0.063640	1.695264
C	4.226885	−2.384942	−1.303729
C	−3.912118	−0.584049	0.942648
C	−5.227587	−0.330048	1.305059
C	−6.281995	−0.958172	0.654856
C	−5.986565	−1.847641	−0.356423
C	−4.690652	−2.132511	−0.741000
C	−3.654557	−1.491902	−0.079787
F	−7.006218	−2.466585	−0.999441
O	5.200036	1.494200	1.420027
C	4.815490	2.696099	2.070542
O	5.972104	−0.751328	0.119133
C	6.432949	−1.271102	1.367271
O	−1.571210	3.127857	−1.951231
C	−2.238257	2.580486	−3.090885
O	−3.409411	2.062806	−0.166055
C	−3.624197	3.390842	0.318280
O	1.028441	2.213716	−1.937133
C	1.476768	3.552104	−1.720782
H	2.552277	2.054095	1.204986
H	0.781616	−2.164755	−0.817216

H	0.786397	−0.948363	−2.085813
H	2.645004	−2.005303	−3.218318
H	0.792476	−4.211369	−2.195584
H	0.429120	−3.130712	−3.551879
H	1.778971	−4.272993	−3.668550
H	1.042046	−0.388944	2.295231
H	0.145209	−1.693665	1.528454
H	−0.964733	0.317975	3.531712
H	−0.668820	−2.722908	3.656344
H	−1.395212	−1.695490	4.905987
H	0.334200	−1.562611	4.544942
H	−3.177813	0.863717	2.327849
H	4.803635	−2.098018	−2.194934
H	4.891358	−2.956342	−0.655486
H	−5.439065	0.376378	2.100830
H	−7.313120	−0.764162	0.923291
H	−4.505185	−2.840372	−1.539497
H	−2.629045	−1.700659	−0.367338
H	4.408720	3.417858	1.356596
H	5.721418	3.100010	2.515632
H	4.079311	2.503976	2.855810
H	5.857239	−2.158342	1.648598
H	7.475701	−1.547456	1.223326
H	6.357946	−0.518195	2.153748
H	−2.495450	3.418654	−3.735503
H	−3.148353	2.056553	−2.788952
H	−1.575377	1.896585	−3.627767
H	−3.096995	4.119624	−0.299970
H	−3.292311	3.476151	1.357226
H	−4.696371	3.568800	0.264807
H	2.152664	3.790417	−2.539756
H	0.635527	4.248058	−1.723790
H	2.017635	3.623975	−0.772128

ωB97X Energy = −1828.87924378 a.u.

(*aR*,1*R*,3*S*,3'*S*)-**21**, Conf H

C	−2.883363	−1.096566	0.813497
C	−4.204166	−0.696847	0.962540
C	−4.613730	0.513600	0.391839
C	−3.709130	1.309187	−0.293989
C	−2.381960	0.901920	−0.451956
C	−1.976843	−0.305673	0.110326
C	−1.426510	1.771198	−1.235124
C	−2.161065	2.815913	−2.062520
C	−1.233938	3.869703	−2.623837
O	−3.125248	3.474297	−1.244260
C	−0.218707	−1.646862	−1.037176
C	1.092683	−2.106075	−1.165606
C	2.063044	−1.663048	−0.273342
C	1.747112	−0.734479	0.718568
C	0.426588	−0.314777	0.867292
C	−0.561400	−0.766767	−0.016435
C	0.068927	0.578124	2.029870
C	1.118152	0.475898	3.122343
C	0.899483	1.465866	4.243867
O	2.393708	0.755578	2.552121
C	2.855633	−0.219724	1.626811
C	−4.186813	2.616051	−0.884375
C	3.986254	0.448731	0.871264
C	5.307732	0.161402	1.184070
C	6.352342	0.808299	0.536492
C	6.041516	1.750314	−0.421144
C	4.739519	2.069959	−0.754299
C	3.713261	1.410212	−0.096596
F	7.051442	2.387363	−1.061757
O	−5.151614	−1.397580	1.636971
C	−4.770362	−2.636698	2.215296
O	−5.904452	0.958057	0.552110
C	−6.860748	0.280869	−0.263673
O	1.411440	−2.998940	−2.155037
C	1.970468	−2.379512	−3.315404
O	3.356455	−2.098505	−0.401731
C	3.562798	−3.454689	0.000585
O	−1.159754	−2.020894	−1.960527
C	−1.637674	−3.358350	−1.811057
H	−2.537221	−2.023824	1.252347
H	−0.738420	2.284777	−0.552522
H	−0.812098	1.152127	−1.894421
H	−2.692754	2.314511	−2.885441
H	−0.743142	4.409227	−1.810403
H	−0.464771	3.405373	−3.243855
H	−1.786866	4.584936	−3.234229
H	−0.909257	0.296899	2.426163
H	−0.004206	1.623909	1.708884
H	1.124750	−0.547404	3.525360
H	0.933512	2.486553	3.856118
H	1.669603	1.357577	5.008699
H	−0.075469	1.303431	4.706967
H	3.269077	−1.072656	2.183889
H	−4.817458	2.413631	−1.762975
H	−4.797837	3.154066	−0.160165
H	5.531360	−0.585152	1.938825
H	7.387710	0.589252	0.766592
H	4.541823	2.818079	−1.512085
H	2.683236	1.645859	−0.344544
H	−4.419873	−3.337975	1.452771
H	−5.664472	−3.035152	2.688361
H	−3.991403	−2.498307	2.970073
H	−6.614487	0.399495	−1.323258
H	−6.904952	−0.780780	−0.012583
H	−7.825429	0.743674	−0.064363
H	2.175439	−3.175474	−4.028547
H	2.900265	−1.862814	−3.064642
H	1.258075	−1.673092	−3.750075
H	2.975496	−4.136232	−0.617301
H	3.297011	−3.585236	1.053787
H	4.623489	−3.657872	−0.132676
H	−2.354587	−3.523928	−2.612909
H	−0.818397	−4.075000	−1.895956
H	−2.139611	−3.477925	−0.846017

ωB97X Energy = −1828.87906502 a.u.

(*aR*,1*R*,3*S*,3'*S*)-**21**, Conf I

C	2.895365	1.132362	0.884175
C	4.239256	0.787231	0.880162
C	4.658611	−0.287665	0.087461
C	3.748489	−0.981985	−0.694071
C	2.398092	−0.622306	−0.704910
C	1.978139	0.434512	0.100926
C	1.443532	−1.367245	−1.608759
C	2.182616	−2.161544	−2.675847
C	1.279506	−3.117872	−3.420603
O	3.214768	−2.927610	−2.059452
C	0.119995	2.015807	−0.467985
C	−1.202047	2.457382	−0.338908
C	−2.105640	1.691825	0.394767
C	−1.704994	0.495438	0.991395
C	−0.374022	0.096138	0.910566
C	0.541592	0.844251	0.158464
C	0.080385	−1.098880	1.714248
C	−0.902981	−1.399161	2.831247
C	−0.588613	−2.683863	3.563400
O	−2.202063	−1.535771	2.262175
C	−2.738420	−0.321263	1.756335
C	4.253222	−2.109539	−1.564440
C	−3.925522	−0.717255	0.902715
C	−5.221673	−0.488286	1.343307
C	−6.315213	−0.873574	0.578525
C	−6.079133	−1.498881	−0.627612
C	−4.804596	−1.753383	−1.096251
C	−3.728392	−1.356451	−0.317733
F	−7.137202	−1.878732	−1.383405
O	5.202730	1.426022	1.591890
C	4.807208	2.519067	2.407895
O	5.986209	−0.633761	0.019321
C	6.487812	−1.280332	1.190518
O	−1.612988	3.671420	−0.815492
C	−1.607370	3.836736	−2.233601
O	−3.380707	2.125431	0.631251
C	−4.231966	2.298028	−0.502262
O	0.993335	2.842807	−1.128751
C	1.514692	2.356865	−2.366132
H	2.540794	1.957823	1.487828
H	0.827129	−2.060373	−1.025035
H	0.755554	−0.665782	−2.089386
H	2.649129	−1.462821	−3.387115
H	0.859631	−3.852237	−2.729160
H	0.456715	−2.575255	−3.889746
H	1.833713	−3.646912	−4.196984
H	1.071056	−0.907239	2.133046
H	0.167540	−1.988825	1.081249
H	−0.914416	−0.560490	3.542649
H	−0.607208	−3.527453	2.869588
H	−1.319544	−2.864749	4.352548
H	0.403588	−2.627983	4.014755
H	−3.103940	0.284624	2.596781
H	4.832518	−1.692387	−2.400656
H	4.921730	−2.756934	−0.996559
H	−5.385851	0.009555	2.292916
H	−7.331223	−0.695246	0.908458
H	−4.666002	−2.248936	−2.049246
H	−2.718949	−1.542776	−0.669945
H	5.714509	2.881222	2.884929
H	4.094400	2.202357	3.173949
H	4.367499	3.320443	1.808130
H	6.411364	−0.624807	2.059724
H	5.941530	−2.209751	1.378411
H	7.533839	−1.509089	0.995874
H	−0.594838	3.980004	−2.610958
H	−2.199051	4.726702	−2.441661
H	−2.066743	2.973326	−2.724148
H	−5.246075	2.367343	−0.112262
H	−4.163275	1.434358	−1.170020
H	−3.984718	3.210260	−1.045332
H	0.708084	1.994637	−3.010714
H	2.006482	3.202058	−2.844497
H	2.241987	1.558680	−2.206243

ωB97X Energy = −1828.87847184 a.u.

(*aR*,1*R*,3*S*,3'*S*)-**21**, Conf J

C	2.888463	1.059422	0.967081
C	4.216056	0.660810	1.033214
C	4.618566	−0.471046	0.314777
C	3.700500	−1.194656	−0.430492
C	2.364234	−0.791776	−0.499681
C	1.966247	0.340172	0.209912
C	1.404944	−1.566599	−1.373010
C	2.140898	−2.463920	−2.357243
C	1.221985	−3.437312	−3.059380
O	3.123356	−3.221007	−1.655170
C	0.190493	1.939749	−0.538337
C	−1.119237	2.431241	−0.496536
C	−2.077179	1.751733	0.253646
C	−1.740613	0.595436	0.958846
C	−0.421789	0.149270	0.961778
C	0.545933	0.805606	0.189934
C	−0.038071	−0.989685	1.876244
C	−1.068053	−1.157687	2.978368
C	−0.822435	−2.379774	3.833802
O	−2.350722	−1.305315	2.376132
C	−2.829506	−0.127656	1.741619
C	4.180617	−2.410802	−1.188966
C	−3.983444	−0.570709	0.866198
C	−3.740729	−1.333861	−0.272286
C	−4.784442	−1.772141	−1.071979
C	−6.073395	−1.432298	−0.708242
C	−6.354734	−0.684212	0.415473
C	−5.293010	−0.259672	1.204241
F	−7.099753	−1.851546	−1.486425
O	5.176052	1.289719	1.758199
C	4.798940	2.445386	2.492576

O	5.916049	−0.917406	0.380205
C	6.851011	−0.119967	−0.347818
O	−1.473010	3.616920	−1.077736
C	−1.368351	3.694440	−2.499850
O	−3.345995	2.243719	0.395953
C	−4.143652	2.316495	−0.786538
O	1.118119	2.685451	−1.221879
C	1.664051	2.093273	−2.401002
H	2.548202	1.934005	1.506390
H	0.747918	−2.192101	−0.758016
H	0.757625	−0.877812	−1.923362
H	2.655268	−1.837196	−3.101756
H	0.748343	−4.099418	−2.330744
H	0.439219	−2.899388	−3.597475
H	1.778643	−4.046066	−3.773063
H	0.945173	−0.797974	2.311975
H	0.035387	−1.933243	1.324186
H	−1.074687	−0.258002	3.611105
H	−0.853660	−3.283090	3.220231
H	−1.580521	−2.461670	4.613792
H	0.158492	−2.316910	4.308318
H	−3.217386	0.558521	2.506808
H	4.807303	−2.090215	−2.034514
H	4.798291	−3.035639	−0.544291
H	−2.720062	−1.585105	−0.542247
H	−4.610291	−2.363527	−1.962449
H	−7.380859	−0.443376	0.664405
H	−5.492804	0.333968	2.089825
H	4.424335	3.229527	1.829186
H	5.701161	2.792634	2.989890
H	4.039572	2.206134	3.241772
H	6.903884	0.890044	0.062812
H	7.819452	−0.606163	−0.247251
H	6.575601	−0.075275	−1.405884
H	−1.950189	4.563305	−2.803313
H	−1.786996	2.797727	−2.966617
H	−0.332467	3.824396	−2.812499
H	−4.137416	1.357048	−1.311841
H	−3.794537	3.108233	−1.449529
H	−5.157406	2.536620	−0.456831
H	0.867539	1.715893	−3.049409
H	2.205749	2.883339	−2.918233
H	2.353315	1.282499	−2.157265

ωB97X Energy = −1828.87832878 a.u.

(*aR*,1*R*,3*S*,3’*S*)-**21**, Conf K

C	2.912194	1.242996	0.752016
C	4.256579	0.907908	0.807619
C	4.694193	−0.246064	0.145681
C	3.799439	−1.032376	−0.561503
C	2.445698	−0.686794	−0.629710
C	2.010205	0.454215	0.039716
C	1.501077	−1.562153	−1.421107
C	2.249397	−2.476133	−2.379787
C	1.359293	−3.534331	−2.990605
O	3.294821	−3.140729	−1.674560
C	0.155359	1.865610	−0.842112
C	−1.182801	2.271158	−0.874242
C	−2.093994	1.662564	−0.015297
C	−1.701865	0.607232	0.809346
C	−0.362604	0.229700	0.854582
C	0.570834	0.854285	0.017118
C	0.078380	−0.797152	1.869314
C	−0.938771	−0.910587	2.990486
C	−0.639355	−2.040456	3.949102
O	−2.217754	−1.164081	2.415445
C	−2.750143	−0.068938	1.682090
C	4.320554	−2.250602	−1.288901
C	−3.916756	−0.636484	0.900397
C	−5.211306	−0.493819	1.381683
C	−6.289195	−1.058618	0.712984
C	−6.038443	−1.771251	−0.440622
C	−4.764800	−1.942315	−0.946861
C	−3.703848	−1.368578	−0.263045
F	−7.081622	−2.325189	−1.104669
O	5.206475	1.628636	1.457425
C	4.795218	2.809721	2.129868
O	6.025846	−0.584941	0.133560
C	6.514577	−1.098207	1.373821
O	−1.530613	3.304880	−1.699318
C	−2.522876	3.009241	−2.686260
O	−3.401086	2.077575	0.023738
C	−3.590759	3.371032	0.603226
O	1.075337	2.520115	−1.619452
C	1.091331	2.120676	−2.987007
H	2.545507	2.131380	1.250025
H	0.905558	−2.184530	−0.742680
H	0.791792	−0.947816	−1.982154
H	2.703079	−1.868191	−3.177496
H	0.947603	−4.176391	−2.208364
H	0.530510	−3.068924	−3.527361
H	1.921893	−4.154269	−3.689890
H	1.052465	−0.517627	2.277235
H	0.197044	−1.782110	1.403569
H	−0.980714	0.041217	3.540175
H	−0.637005	−2.995130	3.418087
H	−1.389914	−2.082819	4.739391
H	0.340526	−1.896899	4.408009
H	−3.139876	0.675722	2.391290
H	4.889803	−1.929811	−2.173308
H	5.001313	−2.813693	−0.650337
H	−5.387117	0.071902	2.290601
H	−7.303900	−0.949593	1.075521
H	−4.614865	−2.509942	−1.857006
H	−2.696048	−1.490867	−0.646648
H	4.368367	3.533439	1.430264
H	5.692903	3.227585	2.578781
H	4.066948	2.585459	2.913925
H	5.968285	−2.003877	1.654910
H	7.563637	−1.342305	1.217908
H	6.424894	−0.352456	2.165540

H	−2.541387	3.860367	−3.363877
H	−3.505495	2.871851	−2.233305
H	−2.251685	2.107095	−3.242364
H	−4.665597	3.534784	0.646077
H	−3.121823	4.147434	−0.005336
H	−3.176702	3.399150	1.615019
H	0.121233	2.292789	−3.459865
H	1.845739	2.729013	−3.481933
H	1.362538	1.064224	−3.079037

ωB97X Energy = −1828.87821641 a.u.

(*aR*,1*R*,3*S*,3’*S*)-**21**, Conf L

C	−2.904649	−1.205999	0.803105
C	−4.232282	−0.829025	0.938018
C	−4.649747	0.389073	0.387861
C	−3.744024	1.216803	−0.255669
C	−2.404314	0.838246	−0.389522
C	−1.994144	−0.381215	0.144718
C	−1.449050	1.758644	−1.113750
C	−2.188748	2.784564	−1.958970
C	−1.276987	3.866225	−2.491758
O	−3.190689	3.411817	−1.162292
C	−0.217196	−1.760954	−0.926975
C	1.105717	−2.199035	−1.046759
C	2.064741	−1.697395	−0.170907
C	1.733220	−0.716416	0.764299
C	0.408863	−0.309157	0.895963
C	−0.571282	−0.823751	0.037370
C	0.038271	0.622543	2.023780
C	1.090692	0.578390	3.117221
C	0.858440	1.605867	4.201606
O	2.360335	0.856720	2.532810
C	2.835220	−0.149890	1.648980
C	−4.237749	2.524688	−0.831080
C	3.954925	0.499193	0.860937
C	5.281261	0.227535	1.167167
C	6.315414	0.856923	0.486515
C	5.989207	1.765224	−0.498209
C	4.682054	2.067791	−0.827126
C	3.666384	1.425648	−0.136294
F	6.988729	2.385353	−1.170874
O	−5.182527	−1.559186	1.576385
C	−4.791420	−2.799239	2.146512
O	−5.951065	0.808145	0.527841
C	−6.880278	0.124399	−0.313559
O	1.393353	−3.159894	−1.976665
C	2.356808	−2.797519	−2.970048
O	3.360652	−2.144520	−0.223111
C	3.537197	−3.492523	0.220358
O	−1.183664	−2.310369	−1.729136
C	−1.228330	−1.783286	−3.052390
H	−2.555132	−2.146035	1.210408
H	−0.819656	2.294301	−0.393103
H	−0.772436	1.181309	−1.749407
H	−2.684431	2.271873	−2.797635
H	−0.818452	4.412366	−1.664102
H	−0.482582	3.426871	−3.098008
H	−1.835134	4.571242	−3.109179
H	−0.934809	0.339397	2.431528
H	−0.051720	1.655051	1.667449
H	1.115370	−0.428735	3.558237
H	0.873999	2.611767	3.775932
H	1.632410	1.539003	4.967228
H	−0.112332	1.444510	4.673757
H	3.260535	−0.972819	2.240748
H	−4.851237	2.322849	−1.721845
H	−4.870848	3.038076	−0.107952
H	5.516800	−0.493237	1.943036
H	7.354325	0.649720	0.711521
H	4.472308	2.789816	−1.606677
H	2.632435	1.648244	−0.379993
H	−4.421926	−3.487844	1.381799
H	−5.685952	−3.214471	2.604143
H	−4.023990	−2.657692	2.912429
H	−7.855886	0.569544	−0.127724
H	−6.615776	0.259108	−1.366808
H	−6.911589	−0.940446	−0.074820
H	2.336630	−3.588755	−3.716549
H	3.356492	−2.713853	−2.542085
H	2.083498	−1.847584	−3.438518
H	4.606744	−3.689667	0.187102
H	3.010940	−4.192604	−0.432187
H	3.176429	−3.606246	1.246494
H	−1.454784	−0.712486	−3.034762
H	−0.283378	−1.950160	−3.575294
H	−2.025848	−2.310408	−3.572329

ωB97X Energy = −1828.87807643 a.u.

(*aR*,1*R*,3*S*,3’*S*)-**21**, Conf M

C	2.904133	1.202469	0.661424
C	4.235411	0.817913	0.716026
C	4.615357	−0.392584	0.123147
C	3.676846	−1.188237	−0.513484
C	2.336241	−0.795296	−0.578913
C	1.958500	0.404677	0.019501
C	1.342139	−1.684896	−1.289417
C	2.032979	−2.690129	−2.198747
C	1.088982	−3.751312	−2.716151
O	3.069703	−3.345186	−1.471777
C	0.142618	1.823041	−0.930014
C	−1.180855	2.273846	−0.973506
C	−2.119972	1.696909	−0.122730
C	−1.748485	0.688302	0.768078
C	−0.413719	0.304716	0.858915
C	0.534940	0.855944	−0.011432
C	0.000272	−0.653147	1.948986
C	−1.031889	−0.663830	3.062316
C	−0.755050	−1.711864	4.116116
O	−2.304661	−0.959529	2.493735
C	−2.822285	0.053035	1.640422

C	4.135952	−2.470289	−1.169508
C	−3.918338	−0.617586	0.836919
C	−3.594800	−1.522444	−0.169302
C	−4.585666	−2.184935	−0.876670
C	−5.903632	−1.924158	−0.555295
C	−6.264262	−1.038020	0.437590
C	−5.254283	−0.387844	1.134987
F	−6.879329	−2.564662	−1.243936
O	5.224922	1.540110	1.301829
C	4.873129	2.777270	1.903032
O	5.933004	−0.782644	0.109785
C	6.423618	−1.240854	1.370643
O	−1.595466	3.210048	−1.881045
C	−1.018704	4.512356	−1.740172
O	−3.436795	2.068783	−0.170595
C	−3.723005	3.386205	0.297423
O	1.070917	2.372986	−1.778856
C	0.999059	1.888389	−3.120245
H	2.581939	2.135100	1.106558
H	0.739766	−2.236908	−0.557964
H	0.643161	−1.083254	−1.876375
H	2.490118	−2.156350	−3.046030
H	0.668706	−4.318909	−1.882629
H	0.267983	−3.290739	−3.268856
H	1.611252	−4.441091	−3.380394
H	0.975021	−0.359967	2.345824
H	0.105935	−1.672295	1.559945
H	−1.073075	0.330889	3.529506
H	−0.750531	−2.706790	3.665194
H	−1.518448	−1.685063	4.894634
H	0.218314	−1.536403	4.577807
H	−3.277713	0.840634	2.257078
H	4.693334	−2.229630	−2.086320
H	4.812675	−3.014695	−0.510798
H	−2.552762	−1.712328	−0.406551
H	−4.348692	−2.890822	−1.663172
H	−7.310520	−0.863219	0.655959
H	−5.516754	0.316252	1.917542
H	4.463686	3.472359	1.164980
H	5.794287	3.185659	2.311335
H	4.149944	2.633897	2.710507
H	7.460265	−1.532898	1.214185
H	6.374526	−0.447810	2.118882
H	5.849392	−2.107465	1.712665
H	−1.548294	5.156480	−2.439318
H	0.045390	4.499128	−1.976378
H	−1.162550	4.885575	−0.721920
H	−3.384962	4.141050	−0.414223
H	−3.254123	3.558757	1.270566
H	−4.804704	3.448165	0.402106
H	0.006047	2.055571	−3.544318
H	1.739096	2.444604	−3.692175
H	1.241868	0.822044	−3.156676

ωB97X Energy = −1828.87801889 a.u.

(*aR*,1*R*,3*S*,3'*S*)-**21**, Conf N

C	2.893729	0.993651	0.912188
C	4.217102	0.579399	0.934750
C	4.609976	−0.475663	0.102593
C	3.689431	−1.097903	−0.725547
C	2.352356	−0.688035	−0.739788
C	1.964376	0.366202	0.084364
C	1.382481	−1.383422	−1.667466
C	1.879661	−2.767610	−2.067832
C	1.742203	−3.797403	−0.954693
O	3.219936	−2.656482	−2.552816
C	0.162301	1.964000	−0.572348
C	−1.157056	2.421373	−0.522905
C	−2.108221	1.698629	0.188482
C	−1.746014	0.532686	0.869872
C	−0.413918	0.127361	0.876581
C	0.542939	0.824752	0.125911
C	−0.005892	−1.042453	1.738351
C	−1.034732	−1.287560	2.826713
C	−0.763234	−2.544703	3.621327
O	−2.312396	−1.435105	2.214398
C	−2.817917	−0.245304	1.624256
C	4.153991	−2.263065	−1.567824
C	−3.960856	−0.684853	0.733151
C	−5.276493	−0.396722	1.067281
C	−6.326624	−0.817438	0.261082
C	−6.027592	−1.538797	−0.875520
C	−4.731753	−1.855907	−1.235672
C	−3.699971	−1.421552	−0.418704
F	−7.042626	−1.954455	−1.669985
O	5.181861	1.117014	1.724909
C	4.813505	2.185766	2.584059
O	5.898262	−0.952340	0.134911
C	6.859839	−0.093318	−0.479919
O	−1.510629	3.573772	−1.175905
C	−1.220484	4.758437	−0.430925
O	−3.388904	2.164933	0.316137
C	−4.164983	2.216107	−0.885082
O	1.099234	2.702201	−1.248378
C	1.039304	2.567719	−2.668596
H	2.560524	1.804501	1.547222
H	0.395958	−1.465455	−1.205480
H	1.250907	−0.785572	−2.575087
H	1.311936	−3.112523	−2.933676
H	2.202304	−3.461167	−0.022159
H	0.683932	−3.980144	−0.757503
H	2.202789	−4.742032	−1.249751
H	0.971672	−0.847643	2.185463
H	0.094985	−1.955047	1.138182
H	−1.062493	−0.420226	3.502466
H	−0.770108	−3.415937	2.962141
H	−1.522129	−2.684254	4.392302
H	0.214165	−2.482404	4.103138
H	−3.221860	0.402608	2.414191
H	5.068827	−2.003279	−2.101545
H	4.398718	−3.107313	−0.909541

H	−5.490440	0.175691	1.963420
H	−7.357541	−0.594273	0.506599
H	−4.543375	−2.426454	−2.136758
H	−2.674203	−1.654883	−0.685285
H	4.444517	3.042889	2.014335
H	5.718404	2.468077	3.116514
H	4.052658	1.869709	3.302806
H	6.907720	0.867994	0.034515
H	7.821409	−0.597353	−0.404136
H	6.614421	0.064013	−1.534732
H	−1.553862	5.598592	−1.036974
H	−0.146815	4.842493	−0.246402
H	−1.762414	4.754514	0.518680
H	−3.727638	2.911723	−1.601122
H	−5.155526	2.558589	−0.591603
H	−4.244577	1.219607	−1.328650
H	1.251040	1.535476	−2.963153
H	0.059942	2.867994	−3.047251
H	1.806139	3.223409	−3.076182

ωB97X Energy = −1828.87792563 a.u.

(*aS*,1*S*,3*S*,1'*S*,3'*S*)-**22**, Conf A

C	0.778024	−1.552833	1.452622
C	2.098748	−1.742981	1.862198
C	3.132619	−1.450503	0.978673
C	2.858641	−0.943213	−0.289590
C	1.539263	−0.760866	−0.694711
C	0.490414	−1.083176	0.175306
O	3.593132	−0.511031	−2.561383
C	2.479866	0.364736	−2.740562
C	1.250157	−0.240972	−2.083382
C	4.014546	−0.600144	−1.205518
C	2.300945	0.574858	−4.226846
C	4.779676	0.655393	−0.799187
C	−0.935087	−0.918958	−0.239751
C	−1.658956	0.214360	0.121740
C	−2.991862	0.315737	−0.276546
C	−3.580498	−0.670897	−1.057196
C	−2.838134	−1.788888	−1.454001
C	−1.524883	−1.909436	−1.019336
C	6.015653	0.889541	−1.399147
C	6.751239	2.026656	−1.108374
C	6.226625	2.929491	−0.203483
C	5.009259	2.736490	0.412549
C	4.288674	1.588387	0.105900
C	−1.028378	1.328015	0.919442
C	−1.821800	2.616738	0.795115
O	−3.197878	2.343394	1.071369
C	−3.809739	1.540038	0.082558
C	−1.369578	3.683619	1.764723
F	6.938590	4.043253	0.090973
O	−4.868093	−0.460134	−1.479849
C	−5.836606	−1.450481	−1.113321
O	−3.460980	−2.680954	−2.266853
C	−2.726996	−3.822924	−2.684189
C	−5.162190	1.175371	0.691684
O	−5.315191	0.334197	1.542437
O	−6.134967	1.946000	0.219875
C	−8.397005	2.686903	0.084736
C	−7.454803	1.730038	0.769506
O	−0.238943	−1.778343	2.342672
C	−0.577948	−3.155877	2.503359
O	2.357606	−2.230552	3.115794
C	2.743328	−1.220015	4.050623
C	4.869424	−2.950962	1.524831
O	4.440798	−1.595316	1.362555
H	2.708779	1.326267	−2.261661
H	0.451827	0.505370	−2.046312
H	0.879477	−1.062280	−2.707276
H	4.722389	−1.431587	−1.192618
H	1.441380	1.220536	−4.416412
H	3.187310	1.041159	−4.659123
H	2.131064	−0.382593	−4.724813
H	−0.932663	−2.770000	−1.304078
H	6.409633	0.169114	−2.108420
H	7.714404	2.214191	−1.566754
H	4.635583	3.468622	1.117749
H	3.331358	1.425336	0.588801
H	−0.966973	1.043240	1.975990
H	−0.003961	1.498701	0.576866
H	−1.746051	2.993258	−0.235179
H	−3.990215	2.135636	−0.821330
H	−1.944315	4.600337	1.627927
H	−0.313382	3.908893	1.606805
H	−1.499905	3.337132	2.792333
H	−6.806880	−1.029989	−1.373215
H	−5.673864	−2.377971	−1.659805
H	−5.796485	−1.636259	−0.037233
H	−3.399484	−4.397180	−3.316481
H	−1.843360	−3.535169	−3.260328
H	−2.423844	−4.432251	−1.828469
H	−9.403371	2.549192	0.482768
H	−8.097423	3.721080	0.259345
H	−8.424657	2.504625	−0.990379
H	−7.733039	0.688714	0.596451
H	−7.411633	1.898926	1.846701
H	0.283372	−3.727148	2.856714
H	−1.373172	−3.197318	3.244995
H	−0.939666	−3.572347	1.558134
H	3.662637	−0.726388	3.725957
H	1.944967	−0.481560	4.163712
H	2.912530	−1.721410	5.001389
H	4.314907	−3.441655	2.326111
H	4.738678	−3.503490	0.590019
H	5.927032	−2.910562	1.776894

ωB97X Energy = −2096.10614559 a.u.

(*aS*,1*S*,3*S*,1'*S*,3'*S*)-**22**, Conf B

C	0.767966	−0.951342	1.862917

C	2.088855	−0.989149	2.322341
C	3.122161	−1.057365	1.392375
C	2.842049	−1.078545	0.024680
C	1.527207	−1.033508	−0.423112
C	0.478751	−0.974427	0.504210
O	3.580786	−1.498953	−2.256803
C	2.478087	−0.726564	−2.729931
C	1.243511	−1.054843	−1.906676
C	4.004388	−1.144468	−0.945054
C	2.300519	−1.042569	−4.197700
C	4.847464	0.123916	−0.970633
C	−0.946361	−0.944810	0.058054
C	−1.666743	0.247225	0.047839
C	−2.999218	0.223663	−0.364705
C	−3.589891	−0.954598	−0.802705
C	−2.850512	−2.142546	−0.832419
C	−1.538894	−2.126439	−0.377789
C	6.189473	0.023541	−1.329745
C	6.997528	1.146654	−1.413014
C	6.435039	2.376851	−1.134543
C	5.111660	2.522191	−0.776088
C	4.322560	1.381966	−0.693058
C	−1.033179	1.551549	0.462718
C	−1.827475	2.742090	−0.045087
O	−3.203836	2.564894	0.299490
C	−3.812509	1.502320	−0.405807
C	−1.376463	4.052511	0.556785
F	7.215342	3.480826	−1.209898
O	−4.875246	−0.881435	−1.275214
C	−5.849543	−1.708172	−0.626915
O	−3.474741	−3.239943	−1.332896
C	−2.743253	−4.456435	−1.380952
C	−5.175400	1.346000	0.265567
O	−5.342942	0.821148	1.338603
O	−6.139609	1.920105	−0.443870
C	−8.402786	2.539729	−0.862847
C	−7.470131	1.879069	0.120075
O	−0.255020	−0.877242	2.775340
C	−0.610373	−2.137942	3.345825
O	2.375781	−1.048401	3.659656
C	2.064440	0.133116	4.405377
C	4.985348	−0.136096	2.567466
O	4.431837	−1.180573	1.765738
H	2.716359	0.339371	−2.616031
H	0.446265	−0.345534	−2.144769
H	0.876589	−2.046316	−2.195416
H	4.658580	−1.962688	−0.639343
H	1.448777	−0.492609	−4.602247
H	3.192477	−0.765162	−4.761128
H	2.119085	−2.111156	−4.334363
H	−0.950243	−3.035197	−0.379052
H	6.614582	−0.951952	−1.542136
H	8.044177	1.076222	−1.682333
H	4.711305	3.505524	−0.562358
H	3.284023	1.483167	−0.396292
H	−0.964890	1.602663	1.555351
H	−0.011188	1.608009	0.077483
H	−1.751351	2.789946	−1.140954
H	−3.977221	1.795646	−1.450424
H	−1.952857	4.883985	0.149556
H	−0.320460	4.221807	0.338986
H	−1.507164	4.032447	1.641037
H	−6.815822	−1.392557	−1.017435
H	−5.682502	−2.760106	−0.852535
H	−5.822117	−1.546493	0.453492
H	−3.418180	−5.194182	−1.807630
H	−1.859332	−4.360522	−2.017314
H	−2.441419	−4.776037	−0.379935
H	−9.418332	2.522419	−0.464682
H	−8.118372	3.578952	−1.032832
H	−8.398633	2.012625	−1.817823
H	−7.736878	0.835370	0.296746
H	−7.454160	2.396269	1.080812
H	−1.405069	−1.943451	4.063274
H	−0.977085	−2.817178	2.570382
H	0.245189	−2.586216	3.856875
H	2.593721	0.995914	3.990320
H	0.990921	0.323645	4.402693
H	2.407703	−0.047355	5.422149
H	4.673819	−0.231403	3.607720
H	6.066700	−0.240245	2.497562
H	4.693201	0.842712	2.176637

ωB97X Energy = −2096.10599611 a.u.

(*aS*,1*S*,3*S*,1'*S*,3'*S*)-**22**, Conf C

C	−0.792309	1.091981	−1.825221
C	−2.122684	1.428965	−2.096670
C	−3.136081	0.595020	−1.632761
C	−2.826298	−0.551609	−0.898285
C	−1.502360	−0.871248	−0.621347
C	−0.473838	−0.046698	−1.095831
O	−3.501803	−2.714765	−0.014392
C	−2.408851	−2.653288	0.901036
C	−1.184940	−2.110414	0.182362
C	−3.964396	−1.433307	−0.426812
C	−2.195251	−4.045756	1.449508
C	−4.831747	−0.801989	0.654945
C	0.960184	−0.381196	−0.845409
C	1.694760	0.301081	0.121856
C	3.028836	−0.054862	0.325621
C	3.606064	−1.085004	−0.400494
C	2.871505	−1.763533	−1.377685
C	1.547679	−1.403596	−1.587636
C	−6.135043	−1.269813	0.809202
C	−6.963383	−0.777434	1.804688
C	−6.461315	0.193908	2.648913
C	−5.179066	0.686095	2.531401
C	−4.367393	0.179623	1.523646
C	1.078067	1.397544	0.954010
C	1.898366	1.680700	2.200651
O	3.269195	1.850929	1.828740

C	3.861478	0.651516	1.379442
C	1.465636	2.937632	2.918774
F	−7.261707	0.684622	3.624547
O	4.930036	−1.381063	−0.187836
C	5.159483	−2.595455	0.529984
O	3.527281	−2.736386	−2.058851
C	2.807891	−3.439661	−3.061721
C	5.229983	1.068195	0.840945
O	5.389767	1.712256	−0.165234
O	6.210330	0.682830	1.649856
C	8.500098	0.421604	2.252764
C	7.555880	0.984653	1.220979
O	0.210857	1.910001	−2.281758
C	0.546270	1.697171	−3.654352
O	−2.435958	2.505609	−2.882269
C	−2.149850	3.785559	−2.309106
C	−5.030109	2.045847	−1.513283
O	−4.452895	0.808478	−1.932594
H	−2.675752	−1.977670	1.724860
H	−0.398702	−1.891604	0.910026
H	−0.789476	−2.886901	−0.482038
H	−4.610549	−1.639344	−1.281949
H	−1.355982	−4.051856	2.147561
H	−3.086016	−4.391911	1.975503
H	−1.975611	−4.742922	0.637583
H	0.952982	−1.912717	−2.335790
H	−6.511445	−2.032535	0.135387
H	−7.979326	−1.131961	1.927900
H	−4.826730	1.450465	3.213019
H	−3.360384	0.568468	1.415456
H	1.000184	2.314381	0.358660
H	0.061004	1.119536	1.244061
H	1.833681	0.821380	2.883612
H	4.021019	−0.023835	2.230230
H	2.060900	3.093399	3.819191
H	0.415190	2.861868	3.205387
H	1.584212	3.804282	2.264687
H	4.796412	−3.453986	−0.037958
H	6.235933	−2.675167	0.669385
H	4.667316	−2.561965	1.506410
H	3.504700	−4.161933	−3.479503
H	1.949480	−3.966142	−2.635841
H	2.468066	−2.762837	−3.850245
H	9.529207	0.622404	1.951586
H	8.331816	0.881421	3.227511
H	8.373647	−0.657984	2.346616
H	7.708679	0.532725	0.238909
H	7.656904	2.066492	1.121154
H	0.926364	0.681577	−3.800124
H	−0.323051	1.864362	−4.294980
H	1.326271	2.414843	−3.900453
H	−2.681148	3.906094	−1.360403
H	−1.078627	3.910908	−2.149140
H	−2.507791	4.526364	−3.021371
H	−6.109007	1.906828	−1.554105
H	−4.737247	2.276820	−0.485195
H	−4.739839	2.860553	−2.176532

ωB97X Energy = −2096.10580625 a.u.

(*aS*,1*S*,3*S*,1'*S*,3'*S*)-**22**, Conf D

C	−0.769287	−1.585124	−1.385650
C	−2.089653	−1.811475	−1.786838
C	−3.124645	−1.447925	−0.928642
C	−2.847124	−0.941166	0.340965
C	−1.530417	−0.763840	0.751067
C	−0.482379	−1.081104	−0.122181
O	−3.594477	−0.464169	2.604914
C	−2.467239	0.392709	2.784179
C	−1.243367	−0.235410	2.136988
C	−4.007197	−0.585057	1.247920
C	−2.292332	0.604446	4.270995
C	−4.774900	0.652227	0.801662
C	0.943181	−0.888526	0.280435
C	1.661688	0.224865	−0.147746
C	2.995403	0.353881	0.239607
C	3.589651	−0.582544	1.075785
C	2.852186	−1.677452	1.540292
C	1.538928	−1.829016	1.115714
C	−4.177688	1.694638	0.099760
C	−4.897021	2.831934	−0.244056
C	−6.224717	2.901141	0.121718
C	−6.857774	1.886993	0.813362
C	−6.118592	0.764132	1.150834
C	1.024433	1.287377	−1.007315
C	1.815906	2.582558	−0.966086
O	3.191552	2.295413	−1.229899
C	3.808435	1.556858	−0.194588
C	1.357577	3.586127	−1.998455
F	−6.937238	4.001842	−0.214817
O	4.877146	−0.341133	1.482226
C	5.849465	−1.348118	1.176265
O	3.479387	−2.516849	2.404430
C	2.745866	−3.626343	2.902137
C	5.160474	1.159972	−0.784285
O	5.313677	0.270208	−1.584036
O	6.131954	1.960929	−0.362928
C	8.393710	2.710180	−0.273561
C	7.448985	1.722898	−0.910042
O	0.251831	−1.830628	−2.266375
C	0.670253	−3.192325	−2.321195
O	−2.316387	−2.332292	−3.031892
C	−2.971133	−3.603949	−3.053060
C	−4.901907	−0.855475	−2.384910
O	−4.438805	−1.631705	−1.275415
H	−2.676065	1.357865	2.304454
H	−0.434791	0.499515	2.097919
H	−0.887955	−1.056037	2.770596
H	−4.708842	−1.420693	1.249640
H	−1.424001	1.237671	4.462640
H	−3.173546	1.085208	4.697833
H	−2.139171	−0.354055	4.772320

H	0.951966	−2.674597	1.451846
H	−3.136698	1.621906	−0.197184
H	−4.440614	3.648474	−0.789797
H	−7.905023	1.979522	1.073503
H	−6.599257	−0.043952	1.692533
H	0.956961	0.937280	−2.043548
H	0.001783	1.476098	−0.668685
H	1.743277	3.022309	0.039086
H	3.988710	2.207971	0.670191
H	1.931386	4.510447	−1.922096
H	0.301638	3.819016	−1.850199
H	1.483888	3.175744	−3.002775
H	6.817767	−0.910622	1.414403
H	5.687584	−2.242952	1.774890
H	5.813270	−1.595588	0.112471
H	3.419845	−4.155196	3.571381
H	1.863833	−3.298254	3.458849
H	2.440408	−4.294418	2.092429
H	9.396743	2.560728	−0.675726
H	8.087883	3.735383	−0.486190
H	8.432664	2.570993	0.807635
H	7.730616	0.690583	−0.694363
H	7.398774	1.845359	−1.993268
H	−0.143487	−3.839240	−2.658866
H	1.488338	−3.239722	−3.037199
H	1.024151	−3.525583	−1.340695
H	−2.973175	−3.928872	−4.091373
H	−2.419990	−4.327429	−2.445523
H	−3.995666	−3.529702	−2.684607
H	−4.387820	−1.136360	−3.305766
H	−5.966119	−1.061909	−2.477936
H	−4.753750	0.209815	−2.190124

ωB97X Energy = −2096.10579153 a.u.

(*aS*,1*S*,3*S*,1'*S*,3'*S*)-**22**, Conf E

C	0.456983	−1.060351	−1.632686
C	1.720766	−1.503214	−2.036863
C	2.833030	−0.718476	−1.745490
C	2.685801	0.486436	−1.055331
C	1.426804	0.915356	−0.651632
C	0.299339	0.137827	−0.947292
O	3.606555	2.634754	−0.378402
C	2.615989	2.711525	0.645887
C	1.287130	2.220560	0.096092
C	3.927903	1.300926	−0.756451
C	2.558009	4.147950	1.113988
C	4.851590	0.655850	0.269544
C	−1.070566	0.573614	−0.542201
C	−1.708185	−0.007170	0.551997
C	−2.994822	0.418037	0.883518
C	−3.616138	1.433525	0.168853
C	−2.957775	2.044055	−0.903756
C	−1.695531	1.589003	−1.260535
C	6.197885	1.014412	0.265489
C	7.082817	0.497355	1.198126
C	6.594231	−0.386880	2.140231
C	5.270164	−0.767977	2.181397
C	4.401980	−0.239368	1.234162
C	−1.036588	−1.074525	1.379613
C	−1.698377	−1.220293	2.738540
O	−3.110044	−1.358791	2.554861
C	−3.718222	−0.176403	2.075626
C	−1.221951	−2.433346	3.502756
F	7.450322	−0.901600	3.054347
O	−4.849339	1.843885	0.603049
C	−5.919597	1.811179	−0.347066
O	−3.607011	3.066958	−1.517487
C	−2.961761	3.700316	−2.612746
C	−5.164074	−0.587913	1.801811
O	−6.060897	−0.417396	2.590378
O	−5.299449	−1.221285	0.643051
C	−6.533643	−2.374140	−1.034150
C	−6.611576	−1.741050	0.331887
O	−0.640691	−1.836281	−1.911564
C	−1.152074	−1.657489	−3.233272
O	1.868862	−2.640889	−2.783903
C	1.575473	−3.864344	−2.101149
C	4.628035	−2.296175	−1.757036
O	4.089580	−1.041609	−2.176437
H	2.922433	2.069848	1.482992
H	0.570171	2.108274	0.913851
H	0.878192	2.983964	−0.575495
H	4.495517	1.410277	−1.682044
H	1.793354	4.263002	1.884583
H	3.518329	4.456360	1.529478
H	2.309804	4.807322	0.279026
H	−1.171246	2.035615	−2.096012
H	6.562817	1.709687	−0.483432
H	8.132313	0.764818	1.197442
H	4.929400	−1.466831	2.935340
H	3.360317	−0.541799	1.250848
H	−1.079132	−2.035253	0.854080
H	0.021053	−0.832045	1.514981
H	−1.517801	−0.311814	3.331004
H	−3.765947	0.568727	2.880253
H	−1.712014	−2.493909	4.475166
H	−0.143392	−2.377003	3.660311
H	−1.442984	−3.343699	2.941082
H	−5.867937	0.898848	−0.947584
H	−6.840506	1.812976	0.234588
H	−5.888617	2.680310	−1.002025
H	−3.645008	4.472991	−2.956200
H	−2.018966	4.159538	−2.303278
H	−2.775637	2.992171	−3.424635
H	−7.507592	−2.788402	−1.298354
H	−6.257002	−1.637151	−1.789985
H	−5.800543	−3.181572	−1.047033
H	−6.888361	−2.462292	1.102516
H	−7.328053	−0.917821	0.360253
H	−0.389611	−1.896522	−3.978715
H	−1.992763	−2.340543	−3.337823

H	−1.498377	−0.628971	−3.372280
H	2.212845	−3.970506	−1.218362
H	0.526780	−3.903451	−1.805446
H	1.793712	−4.666025	−2.803950
H	4.221842	−3.116751	−2.348390
H	5.703877	−2.231086	−1.910159
H	4.427004	−2.464529	−0.695258

ωB97X Energy = −2096.10575917 a.u.

(*aS*,1*S*,3*S*,1'*S*,3'*S*)-**22**, Conf F

C	0.474441	1.768937	−0.860718
C	1.741590	2.230035	−1.220456
C	2.850642	1.804690	−0.496104
C	2.705339	0.900847	0.554295
C	1.438790	0.444173	0.908186
C	0.312054	0.895307	0.209386
O	3.606793	−0.118767	2.563956
C	2.612904	−1.139669	2.473494
C	1.288006	−0.518053	2.062872
C	3.941033	0.441347	1.299732
C	2.540427	−1.825986	3.818530
C	4.827399	−0.513869	0.507716
C	−1.061685	0.452883	0.594581
C	−1.720691	−0.548020	−0.114869
C	−3.006890	−0.917052	0.280500
C	−3.608042	−0.330470	1.386382
C	−2.929657	0.648946	2.119640
C	−1.666672	1.043948	1.700186
C	4.401534	−1.179675	−0.635048
C	5.237488	−2.069565	−1.298917
C	6.503375	−2.277374	−0.795794
C	6.966409	−1.635842	0.336911
C	6.115574	−0.753800	0.982432
C	−1.070744	−1.233444	−1.290694
C	−1.756508	−2.548118	−1.617923
O	−3.167179	−2.328642	−1.704060
C	−3.748060	−2.017045	−0.453613
C	−1.309038	−3.134895	−2.936376
F	7.327678	−3.139513	−1.437249
O	−4.840657	−0.801979	1.754566
C	−5.900286	0.153788	1.867480
O	−3.561036	1.136766	3.218701
C	−2.886366	2.120264	3.989542
C	−5.202221	−1.691756	−0.790131
O	−6.098301	−2.494065	−0.697293
O	−5.347756	−0.462493	−1.269534
C	−6.604171	1.344828	−2.177263
C	−6.672065	−0.086585	−1.709111
O	−0.611013	2.143928	−1.608549
C	−1.110167	3.444051	−1.294703
O	1.874141	3.108945	−2.262720
C	2.299862	2.487690	−3.477752
C	4.376521	3.601957	−0.592221
O	4.111780	2.217482	−0.840290
H	2.924798	−1.866005	1.710985
H	0.578925	−1.308003	1.800463
H	0.860027	0.010196	2.922396
H	4.542152	1.320814	1.540632
H	1.769603	−2.598762	3.806603
H	3.495398	−2.292131	4.064783
H	2.292299	−1.101891	4.598018
H	−1.124272	1.806011	2.245644
H	3.405118	−1.004178	−1.025648
H	4.915082	−2.591923	−2.191186
H	7.970693	−1.824312	0.695913
H	6.459980	−0.241096	1.874466
H	−1.108287	−0.578147	−2.168449
H	−0.014140	−1.418525	−1.077815
H	−1.571718	−3.268067	−0.807699
H	−3.779965	−2.916664	0.174823
H	−1.805817	−4.087448	−3.124161
H	−0.230441	−3.301622	−2.925462
H	−1.545170	−2.448444	−3.752470
H	−5.853658	0.678277	2.820291
H	−5.850684	0.877113	1.048927
H	−6.827766	−0.413031	1.797084
H	−2.682370	3.016601	3.397684
H	−3.557699	2.372440	4.806604
H	−1.949981	1.728095	4.395801
H	−7.588536	1.659704	−2.526513
H	−6.300055	2.007013	−1.364765
H	−5.895924	1.451579	−2.999833
H	−6.975677	−0.765391	−2.507688
H	−7.364089	−0.210316	−0.873799
H	−1.456850	3.479343	−0.257197
H	−0.340749	4.202705	−1.455486
H	−1.949071	3.627091	−1.963285
H	3.277456	2.016882	−3.346999
H	1.568256	1.741924	−3.800193
H	2.369513	3.275693	−4.224884
H	5.419339	3.767431	−0.854476
H	3.733595	4.236720	−1.203694
H	4.226646	3.831703	0.466582

ωB97X Energy = −2096.10573808 a.u.

(*aS*,1*S*,3*S*,1'*S*,3'*S*)-**22**, Conf G

C	−0.786131	−1.708715	−1.312505
C	−2.114078	−1.951027	−1.678334
C	−3.139300	−1.543175	−0.830160
C	−2.850839	−0.953149	0.399013
C	−1.526819	−0.765245	0.783034
C	−0.487975	−1.140261	−0.076371
O	−3.565320	−0.357971	2.645173
C	−2.436440	0.507340	2.758633
C	−1.221395	−0.157915	2.132080
C	−3.997882	−0.552277	1.302672
C	−2.240035	0.805099	4.228008
C	−4.777874	0.657611	0.804868
C	0.940111	−0.941126	0.314053
C	1.650454	0.178223	−0.112685

C	2.984852	0.314204	0.270934
C	3.588217	−0.622638	1.099480
C	2.858869	−1.723504	1.562811
C	1.544950	−1.880971	1.143100
C	−6.104563	0.801955	1.204302
C	−6.854503	1.903279	0.823117
C	−6.249859	2.862678	0.034525
C	−4.940156	2.759879	−0.383944
C	−4.209223	1.645112	0.006809
C	1.004950	1.246009	−0.960299
C	1.782556	2.548754	−0.896194
O	3.159537	2.279875	−1.170736
C	3.788620	1.526859	−0.153224
C	1.310660	3.567230	−1.907656
F	−6.973379	3.941640	−0.346250
O	4.876274	−0.375772	1.501724
C	5.852616	−1.374090	1.180966
O	3.494927	−2.561797	2.421403
C	2.767545	−3.672212	2.926326
C	5.136474	1.143644	−0.761232
O	5.285457	0.264186	−1.573163
O	6.108850	1.942907	−0.339052
C	8.367867	2.702025	−0.264002
C	7.422152	1.715053	−0.899480
O	0.254444	−2.092845	−2.109779
C	0.248999	−1.641383	−3.466364
O	−2.421170	−2.585769	−2.854301
C	−2.191411	−3.996159	−2.817339
C	−4.967304	−1.104659	−2.275981
O	−4.448971	−1.799916	−1.138193
H	−2.653013	1.443062	2.226286
H	−0.413479	0.573317	2.041099
H	−0.856770	−0.941452	2.806234
H	−4.696523	−1.388450	1.360219
H	−1.370835	1.450116	4.369798
H	−3.116320	1.307738	4.639532
H	−2.076709	−0.122731	4.781172
H	0.963987	−2.731276	1.477194
H	−6.562423	0.037266	1.823242
H	−7.888549	2.020418	1.123140
H	−4.506647	3.533030	−1.006248
H	−3.182369	1.545983	−0.329078
H	0.949938	0.918476	−2.005154
H	−0.022464	1.417362	−0.627356
H	1.708286	2.967660	0.117670
H	3.974815	2.164741	0.720047
H	1.875671	4.495449	−1.815288
H	0.253063	3.787088	−1.751643
H	1.437939	3.177695	−2.920188
H	5.815539	−1.608007	0.114095
H	6.819426	−0.935572	1.423238
H	5.695746	−2.277547	1.767990
H	3.447017	−4.197031	3.593208
H	1.887819	−3.345016	3.487153
H	2.459397	−4.343500	2.120477
H	9.368095	2.559758	−0.675645
H	8.055740	3.727286	−0.466953
H	8.416608	2.555544	0.815824
H	7.710263	0.682499	−0.693588
H	7.361970	1.845085	−1.981282
H	−0.190197	−0.643400	−3.540411
H	1.291183	−1.602670	−3.779151
H	−0.305931	−2.324315	−4.110276
H	−2.502969	−4.388420	−3.783346
H	−1.132060	−4.213447	−2.656216
H	−2.785901	−4.458348	−2.024995
H	−4.893594	−0.024086	−2.124903
H	−4.436814	−1.394053	−3.183453
H	−6.015378	−1.386865	−2.354574

ωB97X Energy = −2096.10572014 a.u.

(*aS*,1*S*,3*S*,1'*S*,3'*S*)-**22**, Conf H

C	−0.744933	−1.198162	1.763632
C	−2.070939	−1.538449	2.052330
C	−3.088119	−0.672074	1.661864
C	−2.786387	0.512201	0.986255
C	−1.467031	0.838094	0.696197
C	−0.434748	−0.021764	1.093193
O	−3.468072	2.726259	0.245880
C	−2.392269	2.720687	−0.691627
C	−1.158218	2.122391	−0.036580
C	−3.928187	1.422173	0.583021
C	−2.177249	4.146775	−1.145237
C	−4.804372	0.856709	−0.528248
C	0.994869	0.311359	0.816973
C	1.677814	−0.296841	−0.233910
C	3.008038	0.057135	−0.462949
C	3.631810	1.014994	0.320876
C	2.950943	1.614946	1.384549
C	1.630046	1.257856	1.617936
C	−6.100338	1.350846	−0.661605
C	−6.934814	0.916151	−1.678697
C	−6.446969	−0.024309	−2.565290
C	−5.172271	−0.540016	−2.470103
C	−4.353959	−0.090889	−1.440773
C	1.009723	−1.307829	−1.131949
C	1.770334	−1.482096	−2.435266
O	3.152557	−1.706466	−2.142207
C	3.786357	−0.564506	−1.606348
C	1.284795	−2.657882	−3.250381
F	−7.254142	−0.458778	−3.561777
O	4.949960	1.308382	0.072203
C	5.177205	2.595279	−0.506461
O	3.651968	2.513361	2.121047
C	2.986067	3.139154	3.208555
C	5.163326	−1.059278	−1.161884
O	5.347734	−1.752503	−0.193722
O	6.107552	−0.687429	−2.021078
C	8.095000	−0.047615	−0.772495
C	7.470300	−1.057212	−1.708981
O	0.261630	−2.051419	2.141451

C	0.641722	−1.921962	3.512765
O	−2.373948	−2.654534	2.785154
C	−2.116270	−3.903789	2.135801
C	−4.995439	−2.103664	1.510490
O	−4.399689	−0.892972	1.978806
H	−2.679585	2.105675	−1.555219
H	−0.390649	1.944945	−0.794869
H	−0.740559	2.851974	0.666504
H	−4.566548	1.575377	1.454745
H	−1.349426	4.196206	−1.855148
H	−3.073662	4.535226	−1.630523
H	−1.938858	4.783308	−0.289927
H	1.075768	1.709812	2.431014
H	−6.465560	2.088838	0.045098
H	−7.944908	1.291766	−1.786587
H	−4.830495	−1.278162	−3.185144
H	−3.352210	−0.497571	−1.351599
H	0.945140	−2.274930	−0.620487
H	−0.015177	−0.994539	−1.349452
H	1.691877	−0.559529	−3.028293
H	3.931356	0.183661	−2.396693
H	1.838249	−2.734104	−4.187020
H	0.224685	−2.540206	−3.481966
H	1.415511	−3.585571	−2.688893
H	4.647044	2.686225	−1.459052
H	4.856982	3.386067	0.173943
H	6.249249	2.670997	−0.679517
H	3.711855	3.813021	3.656765
H	2.121062	3.712699	2.864569
H	2.665589	2.403039	3.950635
H	9.128486	−0.331691	−0.566669
H	8.096488	0.945969	−1.224069
H	7.550673	−0.002711	0.171526
H	7.473640	−2.059688	−1.281818
H	7.973940	−1.076767	−2.673571
H	1.040287	−0.921118	3.704007
H	−0.209538	−2.115372	4.170117
H	1.419075	−2.661927	3.692475
H	−2.669634	−3.965372	1.194107
H	−1.050414	−4.032263	1.945633
H	−2.468231	−4.678561	2.814126
H	−6.071998	−1.959775	1.583081
H	−4.727787	−2.284517	0.465575
H	−4.696996	−2.953344	2.124397

ωB97X Energy = −2096.10566943 a.u.

(*aS*,1*S*,3*S*,1'*S*,3'*S*)-**22**, Conf I

C	0.829158	−1.799752	1.195215
C	2.166136	−2.047952	1.509953
C	3.162926	−1.573859	0.663447
C	2.835731	−0.832165	−0.469915
C	1.500615	−0.586765	−0.778204
C	0.488683	−1.089848	0.048819
O	3.467741	0.029849	−2.648915
C	2.356320	0.924448	−2.598714
C	1.152506	0.198131	−2.021075
C	3.952007	−0.325667	−1.358945
C	2.114590	1.430865	−4.002323
C	4.764386	0.816255	−0.759888
C	−0.951998	−0.876546	−0.283960
C	−1.690400	0.128183	0.336090
C	−3.028976	0.295596	−0.023579
C	−3.607199	−0.509110	−0.992990
C	−2.868296	−1.524488	−1.607822
C	−1.539073	−1.694491	−1.247113
C	6.001456	1.103465	−1.333358
C	6.779876	2.153695	−0.875801
C	6.295832	2.915530	0.170165
C	5.079233	2.665164	0.767319
C	4.315716	1.605330	0.292368
C	−1.072777	1.036735	1.369669
C	−1.900292	2.293734	1.576209
O	−3.268546	1.925533	1.773379
C	−3.859400	1.399775	0.605094
C	−1.468743	3.092728	2.783698
F	7.048588	3.943768	0.628537
O	−4.935126	−0.330646	−1.293553
C	−5.180280	0.306823	−2.548883
O	−3.524903	−2.277910	−2.525001
C	−2.796972	−3.311374	−3.172976
C	−5.236135	0.906072	1.048414
O	−5.409718	−0.031130	1.785965
O	−6.206777	1.674843	0.568327
C	−8.486678	2.191415	0.119419
C	−7.559711	1.285590	0.889911
O	−0.150562	−2.218964	2.056547
C	−0.483364	−3.601536	1.926302
O	2.477006	−2.775934	2.627568
C	2.872394	−1.971246	3.740731
C	4.922611	−3.135754	0.828047
O	4.486035	−1.779163	0.958722
H	2.615853	1.770518	−1.948109
H	0.361696	0.919823	−1.798240
H	0.748963	−0.483007	−2.778907
H	4.641814	−1.151718	−1.544883
H	1.258815	2.108491	−4.016800
H	2.988706	1.968359	−4.372396
H	1.906540	0.595828	−4.675330
H	−0.939733	−2.468362	−1.710272
H	6.363276	0.493355	−2.154427
H	7.744433	2.380593	−1.312916
H	4.739174	3.286209	1.586875
H	3.359139	1.395708	0.758766
H	−0.982438	0.503793	2.323005
H	−0.060060	1.315692	1.065276
H	−1.841979	2.920560	0.674655
H	−4.006741	2.203448	−0.128369
H	−2.068029	3.998543	2.882296
H	−0.419795	3.378722	2.687697
H	−1.582852	2.495465	3.691006
H	−4.822415	−0.313646	−3.372615

H	−6.258312	0.435593	−2.626534
H	−4.693615	1.285982	−2.583982
H	−3.493815	−3.782868	−3.861337
H	−1.950092	−2.905852	−3.733153
H	−2.438964	−4.052240	−2.453046
H	−9.521712	1.931059	0.345362
H	−8.327680	3.235707	0.391491
H	−8.330914	2.080263	−0.954770
H	−7.690531	0.237934	0.613119
H	−7.700366	1.376862	1.968321
H	−0.882451	−3.804320	0.927629
H	0.391613	−4.228463	2.112719
H	−1.248617	−3.810751	2.671114
H	3.765991	−1.390876	3.497847
H	2.060678	−1.299532	4.032785
H	3.091453	−2.655276	4.558145
H	5.986203	−3.140270	1.057072
H	4.389507	−3.784492	1.524528
H	4.769497	−3.486468	−0.196727

ωB97X Energy = −2096.10564467 a.u.

(*aS*,1*S*,3*S*,1’*S*,3’*S*)-**22**, Conf J

C	−0.751180	−1.651125	−1.346719
C	−2.074372	−1.908390	−1.708231
C	−3.098201	−1.562610	−0.832837
C	−2.811402	−0.942374	0.381335
C	−1.489587	−0.690664	0.738544
C	−0.449643	−1.059946	−0.124142
O	−3.509682	−0.344023	2.626649
C	−2.421438	0.576624	2.706353
C	−1.187811	−0.043621	2.070188
C	−3.957295	−0.557724	1.293065
C	−2.215194	0.912182	4.165948
C	−4.769851	0.636323	0.804694
C	0.978028	−0.818632	0.243131
C	1.684157	0.252270	−0.300094
C	3.011959	0.443432	0.084374
C	3.609322	−0.396178	1.011382
C	2.905274	−1.479548	1.545596
C	1.587811	−1.676794	1.155664
C	−6.013863	0.862827	1.390584
C	−6.790434	1.951610	1.029738
C	−6.298264	2.813937	0.068921
C	−5.074756	2.626096	−0.536711
C	−4.313053	1.526496	−0.159689
C	1.044366	1.210374	−1.273445
C	1.826458	2.508802	−1.370600
O	3.207034	2.205615	−1.591285
C	3.817452	1.602481	−0.470206
C	1.369530	3.389085	−2.510353
F	−7.049682	3.880652	−0.293661
O	4.925192	−0.179669	1.338938
C	5.135542	0.339769	2.653575
O	3.581960	−2.269622	2.416794
C	2.888682	−3.371351	2.984981
C	5.191862	1.158766	−0.973320
O	5.367941	0.219613	−1.707415
O	6.145045	1.987223	−0.557345
C	8.116007	0.687239	0.030780
C	7.505953	1.677220	−0.935733
O	0.252086	−1.937005	−2.234932
C	0.622065	−3.315762	−2.268815
O	−2.347153	−2.513008	−2.906777
C	−2.712973	−1.590722	−3.935459
C	−4.809300	−3.142393	−1.208472
O	−4.409552	−1.768808	−1.175545
H	−2.692337	1.489127	2.158491
H	−0.415532	0.721044	1.948943
H	−0.776479	−0.795843	2.752822
H	−4.638721	−1.407847	1.365834
H	−1.376344	1.601440	4.279958
H	−3.107795	1.380561	4.582895
H	−1.997938	0.004759	4.734311
H	1.016364	−2.504949	1.555928
H	−6.382294	0.174244	2.143872
H	−7.760072	2.132578	1.476940
H	−4.727663	3.325560	−1.287189
H	−3.350644	1.367137	−0.633774
H	0.987372	0.748685	−2.265548
H	0.018048	1.427273	−0.964223
H	1.743602	3.055786	−0.420419
H	3.967863	2.352646	0.317187
H	1.938228	4.319442	−2.532560
H	0.311503	3.631055	−2.395001
H	1.504494	2.872137	−3.462978
H	4.794471	−0.370421	3.408703
H	6.207740	0.497686	2.755542
H	4.613807	1.293354	2.777115
H	3.596180	−3.860303	3.649929
H	2.020294	−3.037553	3.559506
H	2.568064	−4.076449	2.213427
H	9.151676	0.491881	−0.252784
H	8.108589	1.087338	1.046165
H	7.568419	−0.255838	0.021095
H	7.509712	1.298742	−1.957623
H	8.020012	2.636077	−0.906663
H	−0.231299	−3.937720	−2.548495
H	1.405359	−3.410643	−3.018182
H	1.010012	−3.630252	−1.294983
H	−2.896731	−2.178023	−4.832901
H	−3.620587	−1.047592	−3.660112
H	−1.899426	−0.884212	−4.121059
H	−4.251393	−3.691860	−1.968090
H	−4.657589	−3.605140	−0.228940
H	−5.869913	−3.148498	−1.451012

ωB97X Energy = −2096.10562824 a.u.

(*aS*,1*S*,3*S*,1’*S*,3’*S*)-**22**, Conf K

C	−0.482002	1.944849	−0.553237
C	−1.754146	2.518032	−0.570580

C	−2.859813	1.712321	−0.819099
C	−2.703184	0.343797	−1.030797
C	−1.431380	−0.222092	−1.016265
C	−0.309729	0.584891	−0.786698
O	−3.597203	−1.743333	−1.884364
C	−2.583292	−2.457964	−1.177443
C	−1.267927	−1.701610	−1.274506
C	−3.933807	−0.505893	−1.267859
C	−2.501462	−3.843600	−1.776610
C	−4.775415	−0.742945	−0.018167
C	1.068306	0.008922	−0.773909
C	1.716182	−0.255876	0.430677
C	3.003375	−0.792902	0.395215
C	3.613984	−1.089582	−0.813253
C	2.966807	−0.817599	−2.021981
C	1.691624	−0.269547	−1.988066
C	−6.061283	−1.254644	−0.182635
C	−6.866719	−1.538029	0.908250
C	−6.360629	−1.300258	2.171785
C	−5.096482	−0.792695	2.379063
C	−4.306467	−0.515312	1.269966
C	1.053900	0.012281	1.758938
C	1.743510	−0.736848	2.886296
O	3.152152	−0.498476	2.812325
C	3.747764	−1.097872	1.680357
C	1.282455	−0.296538	4.255998
F	−7.139360	−1.572556	3.245754
O	4.893303	−1.587777	−0.795815
C	5.000321	−2.970160	−1.144435
O	3.654966	−1.111306	−3.153863
C	3.025093	−0.846074	−4.398921
C	5.193076	−0.600118	1.697144
O	6.107878	−1.244038	2.149649
O	5.304921	0.635409	1.230503
C	6.516103	2.596894	0.638920
C	6.629662	1.209828	1.218917
O	0.598292	2.732137	−0.248776
C	1.084005	3.497963	−1.352065
O	−1.908546	3.863074	−0.360602
C	−2.008802	4.208952	1.022458
C	−4.431326	3.103855	−1.893711
O	−4.125483	2.237086	−0.797392
H	−2.879129	−2.535285	−0.122685
H	−0.549253	−2.126901	−0.568390
H	−0.844446	−1.848215	−2.274684
H	−4.567335	0.001222	−1.998523
H	−1.715100	−4.420832	−1.286565
H	−3.447942	−4.371939	−1.654132
H	−2.271469	−3.779904	−2.842731
H	1.165213	−0.047870	−2.908079
H	−6.439931	−1.436313	−1.183088
H	−7.867973	−1.933513	0.789521
H	−4.739467	−0.618806	3.386670
H	−3.311576	−0.111051	1.421957
H	1.075565	1.086775	1.973774
H	0.001514	−0.282579	1.717581

H	1.573028	−1.816191	2.764387
H	3.803691	−2.185228	1.823664
H	1.790050	−0.865426	5.035862
H	0.206856	−0.451550	4.357501
H	1.494127	0.765049	4.401646
H	4.698460	−3.130891	−2.180794
H	6.046975	−3.240004	−1.019190
H	4.383761	−3.581789	−0.479259
H	3.731429	−1.158202	−5.164041
H	2.098984	−1.417717	−4.503697
H	2.812502	0.220175	−4.514226
H	7.503935	3.058525	0.603759
H	6.116440	2.561194	−0.375476
H	5.865800	3.222774	1.251483
H	7.012903	1.223233	2.240584
H	7.275616	0.567733	0.617180
H	1.425952	2.835940	−2.153501
H	0.307675	4.168428	−1.728073
H	1.924246	4.081918	−0.981715
H	−2.873486	3.717963	1.477277
H	−1.097101	3.925724	1.554563
H	−2.137656	5.288546	1.067827
H	−5.466562	3.411932	−1.762779
H	−3.780210	3.979076	−1.889230
H	−4.327899	2.568232	−2.841951

ωB97X Energy = −2096.10556265 a.u.

(*aS*,1*S*,3*S*,1'*S*,3'*S*)-**22**, Conf L

C	−0.667219	0.267990	2.026279
C	−1.962860	0.113886	2.530525
C	−3.042122	0.485183	1.733785
C	−2.830448	0.995719	0.451444
C	−1.539900	1.141421	−0.043211
C	−0.445826	0.778266	0.753021
O	−3.686780	2.180776	−1.494756
C	−2.604016	1.657007	−2.262926
C	−1.331562	1.696761	−1.432543
C	−4.040112	1.368108	−0.381285
C	−2.504406	2.483750	−3.524759
C	−4.862499	0.164703	−0.825427
C	0.957482	0.932281	0.265956
C	1.684367	−0.172478	−0.171697
C	3.003171	0.012750	−0.585970
C	3.572658	1.279078	−0.612656
C	2.823963	2.393949	−0.219254
C	1.528032	2.201579	0.240810
C	−6.215563	0.348342	−1.100490
C	−7.006702	−0.697924	−1.548201
C	−6.416554	−1.934849	−1.719912
C	−5.081510	−2.160735	−1.460766
C	−4.309240	−1.098469	−1.008178
C	1.072855	−1.550676	−0.216236
C	1.857304	−2.474359	−1.131336
O	3.241583	−2.411151	−0.778767
C	3.828612	−1.159640	−1.073976

C	1.430568	−3.919552	−1.021018
F	−7.180447	−2.965190	−2.153662
O	4.845628	1.390174	−1.110447
C	5.829334	1.964490	−0.241111
O	3.424066	3.606886	−0.335068
C	2.686380	4.753470	0.062799
C	5.195103	−1.230657	−0.394482
O	5.370312	−1.061895	0.786722
O	6.147194	−1.567303	−1.258235
C	7.644655	−3.143505	−0.146893
C	7.481998	−1.759368	−0.733022
O	0.400145	−0.105217	2.804572
C	0.784842	0.879644	3.765248
O	−2.178424	−0.309794	3.814631
C	−1.838769	−1.676319	4.071952
C	−4.840879	−0.828255	2.599550
O	−4.332783	0.439585	2.182416
H	−2.830087	0.615878	−2.529684
H	−0.542611	1.138499	−1.943821
H	−0.984720	2.734085	−1.362597
H	−4.690679	2.002080	0.223362
H	−1.676372	2.131937	−4.143076
H	−3.425772	2.411026	−4.104210
H	−2.329448	3.533068	−3.276210
H	0.934875	3.046044	0.568447
H	−6.662122	1.326912	−0.957617
H	−8.061237	−0.565425	−1.756388
H	−4.659007	−3.147341	−1.605668
H	−3.260725	−1.268637	−0.787991
H	1.044400	−1.980623	0.791342
H	0.038297	−1.487395	−0.565033
H	1.751743	−2.132672	−2.171029
H	3.987532	−1.069641	−2.155991
H	2.006419	−4.545224	−1.703931
H	0.371839	−4.018690	−1.266897
H	1.584022	−4.280388	−0.001557
H	5.818695	1.457564	0.726789
H	6.790065	1.799910	−0.726255
H	5.657285	3.031139	−0.106977
H	3.339557	5.604749	−0.111968
H	1.776695	4.864472	−0.533464
H	2.425618	4.706451	1.123530
H	8.676835	−3.279302	0.180539
H	6.990147	−3.284120	0.713908
H	7.418478	−3.906639	−0.893018
H	8.131555	−1.610088	−1.593281
H	7.681019	−0.982852	0.006090
H	−0.037076	1.090201	4.454071
H	1.627970	0.466398	4.315172
H	1.094068	1.801059	3.262789
H	−2.424248	−2.341274	3.430253
H	−0.774664	−1.849985	3.910262
H	−2.091041	−1.863530	5.113824
H	−5.925686	−0.736585	2.597425
H	−4.545680	−1.610573	1.894610
H	−4.496258	−1.079819	3.602569

ωB97X Energy = −2096.10550950 a.u.

(*aS*,1*S*,3*S*,1’*S*,3’*S*)-**22**, Conf M

C	0.725114	−1.600576	1.417427
C	2.040999	−1.827822	1.824521
C	3.083143	−1.527151	0.953614
C	2.822603	−0.977584	−0.299844
C	1.508447	−0.756580	−0.701933
C	0.450892	−1.084110	0.155516
O	3.570593	−0.500241	−2.557712
C	2.476715	0.403311	−2.716614
C	1.232938	−0.190794	−2.075329
C	3.987012	−0.632081	−1.203723
C	2.304038	0.652983	−4.197540
C	4.773198	0.598461	−0.763392
C	−0.969126	−0.875168	−0.258328
C	−1.654780	0.283926	0.095301
C	−2.983431	0.428418	−0.305283
C	−3.603488	−0.540191	−1.084163
C	−2.896082	−1.682369	−1.477996
C	−1.590097	−1.847274	−1.036550
C	4.295262	1.517766	0.162512
C	5.035046	2.645010	0.499487
C	6.258074	2.831113	−0.107422
C	6.770335	1.941027	−1.031818
C	6.015745	0.824674	−1.352732
C	−0.985441	1.386793	0.876153
C	−1.717103	2.705117	0.699391
O	−3.103945	2.504740	0.982576
C	−3.756680	1.685408	0.031756
C	−1.219657	3.787223	1.629108
F	6.988465	3.924595	0.216344
O	−4.880583	−0.284917	−1.514606
C	−5.879721	−1.250522	−1.165415
O	−3.540324	−2.551837	−2.298766
C	−2.837260	−3.710949	−2.722000
C	−5.106546	1.398870	0.688881
O	−5.296051	0.530419	1.503535
O	−6.013414	2.296071	0.316252
C	−8.223731	1.237688	0.357503
C	−7.301019	2.262479	0.976229
O	−0.298973	−1.836648	2.296636
C	−0.659436	−3.213038	2.415018
O	2.286677	−2.358040	3.063379
C	2.685743	−1.382938	4.029653
C	4.791572	−3.073215	1.461642
O	4.387457	−1.706149	1.336552
H	2.725943	1.347944	−2.214828
H	0.452790	0.573359	−2.017709
H	0.842120	−0.985428	−2.721197
H	4.679626	−1.476274	−1.211147
H	1.460242	1.323337	−4.371716
H	3.201491	1.108547	−4.618260
H	2.111987	−0.288053	−4.718230
H	−1.026288	−2.727279	−1.319429

H	3.333170	1.360327	0.637837
H	4.671577	3.366621	1.220677
H	7.738532	2.122310	−1.482026
H	6.399856	0.114421	−2.077509
H	−0.956566	1.133116	1.941964
H	0.051616	1.500122	0.548406
H	−1.622500	3.036931	−0.344631
H	−3.938117	2.256322	−0.887769
H	−1.755977	4.721269	1.457776
H	−0.155218	3.962890	1.463724
H	−1.363660	3.485735	2.668975
H	−5.702514	−2.197993	−1.671128
H	−5.895872	−1.398582	−0.082452
H	−6.829041	−0.827844	−1.489248
H	−2.557716	−4.337072	−1.870425
H	−3.522274	−4.259243	−3.363829
H	−1.941867	−3.443876	−3.289850
H	−9.210310	1.320310	0.816855
H	−8.330519	1.410347	−0.714877
H	−7.853330	0.225570	0.520519
H	−7.141900	2.069979	2.037478
H	−7.688167	3.271800	0.850274
H	−1.025454	−3.595153	1.456938
H	0.191998	−3.808268	2.752404
H	−1.456908	−3.264494	3.153617
H	2.845376	−1.915596	4.964960
H	3.613132	−0.893449	3.722097
H	1.898355	−0.636306	4.164040
H	5.848784	−3.058960	1.718211
H	4.225498	−3.576662	2.246754
H	4.654738	−3.596709	0.511104

ωB97X Energy = −2096.10549169 a.u.

(*aS*,1*S*,3*S*,1'*S*,3'*S*)-**22**, Conf N

C	−0.824233	−1.818030	−1.156068
C	−2.161456	−2.084972	−1.467392
C	−3.159754	−1.561671	−0.648910
C	−2.829550	−0.849992	0.504435
C	−1.496942	−0.625781	0.830617
C	−0.485588	−1.108541	−0.009404
O	−3.482757	0.009254	2.682280
C	−2.346871	0.873286	2.668584
C	−1.151804	0.131656	2.091261
C	−3.950451	−0.332342	1.382067
C	−2.112413	1.335954	4.088741
C	−4.731499	0.821117	0.766128
C	0.954610	−0.876606	0.313087
C	1.677522	0.126198	−0.328750
C	3.015898	0.314863	0.019285
C	3.608757	−0.462295	1.002123
C	2.885993	−1.475668	1.638790
C	1.557385	−1.669653	1.287175
C	−6.050545	1.017152	1.168819
C	−6.801326	2.075737	0.683013
C	−6.205108	2.940310	−0.214067
C	−4.903139	2.783862	−0.639915
C	−4.171364	1.712735	−0.143049
C	1.043521	1.012154	−1.371833
C	1.859425	2.271913	−1.605895
O	3.228466	1.910663	−1.811660
C	3.835461	1.407571	−0.641486
C	1.409624	3.049518	−2.820669
F	−6.929247	3.976762	−0.697666
O	4.935843	−0.259525	1.291550
C	5.178623	0.394383	2.539051
O	3.556581	−2.204667	2.565761
C	2.847634	−3.241441	3.229299
C	5.203931	0.899844	−1.094877
O	5.365328	−0.073112	−1.787521
O	6.181817	1.697248	−0.680026
C	8.468744	2.313245	−0.424286
C	7.527685	1.292625	−1.012435
O	0.162352	−2.230532	−2.013463
C	0.561986	−3.588933	−1.847064
O	−2.442377	−2.807044	−2.595596
C	−3.106717	−4.053923	−2.371472
C	−4.988457	−1.213511	−2.124328
O	−4.488300	−1.779792	−0.909092
H	−2.573294	1.743935	2.039029
H	−0.343937	0.841009	1.892169
H	−0.772319	−0.570597	2.842269
H	−4.654885	−1.148412	1.551361
H	−1.237266	1.986996	4.133693
H	−2.975958	1.889208	4.460328
H	−1.939464	0.477454	4.741854
H	0.970867	−2.443548	1.766430
H	−6.501895	0.326849	1.873958
H	−7.829709	2.231229	0.985005
H	−4.476306	3.482676	−1.348724
H	−3.150811	1.571401	−0.483070
H	0.948792	0.463111	−2.315377
H	0.031431	1.288229	−1.062435
H	1.805943	2.912154	−0.713503
H	3.995111	2.225145	0.073773
H	2.000509	3.958518	−2.938732
H	0.359411	3.328616	−2.718283
H	1.519262	2.439719	−3.720131
H	4.832447	−0.221715	3.370948
H	6.255207	0.537448	2.610209
H	4.679467	1.367494	2.565562
H	3.554508	−3.693099	3.920735
H	1.996434	−2.842663	3.787771
H	2.498868	−3.996624	2.519775
H	9.498356	2.028345	−0.645290
H	8.287329	3.301291	−0.849401
H	8.352601	2.369609	0.659082
H	7.694624	0.296824	−0.596940
H	7.617019	1.231735	−2.098172
H	−0.267467	−4.270188	−2.053013
H	1.361641	−3.769534	−2.562695
H	0.935979	−3.758210	−0.832713

H	−2.521493	−4.682293	−1.693942
H	−4.103436	−3.903147	−1.953323
H	−3.181552	−4.539000	−3.342364
H	−4.531259	−1.682397	−2.997184
H	−6.061007	−1.396498	−2.125211
H	−4.803649	−0.136471	−2.145765

ωB97X Energy = −2096.10547817 a.u.

(*aS*,1*S*,3*S*,1'*S*,3'*S*)-**22**, Conf O

C	−0.478533	1.446999	−1.358135
C	−1.758209	1.941054	−1.633120
C	−2.848691	1.083513	−1.519145
C	−2.665497	−0.243220	−1.123719
C	−1.391820	−0.722735	−0.842998
C	−0.285923	0.127945	−0.967436
O	−3.532977	−2.510532	−0.935575
C	−2.521778	−2.790026	0.031965
C	−1.212697	−2.159512	−0.413338
C	−3.886264	−1.133570	−1.009227
C	−2.427767	−4.292801	0.168370
C	−4.814023	−0.748515	0.135833
C	1.100457	−0.360700	−0.702782
C	1.742908	−0.069537	0.498415
C	3.039583	−0.544598	0.698041
C	3.666407	−1.318482	−0.266265
C	3.024138	−1.605384	−1.474381
C	1.738645	−1.122800	−1.678480
C	−6.166387	−1.064992	0.030721
C	−7.054277	−0.777831	1.055453
C	−6.561722	−0.170218	2.193718
C	−5.231064	0.159317	2.340489
C	−4.360754	−0.132746	1.297913
C	1.063997	0.728856	1.582587
C	1.749999	0.538168	2.924266
O	3.155322	0.751150	2.763290
C	3.777436	−0.254905	1.990578
C	1.263899	1.505883	3.977740
F	−7.420005	0.118030	3.200402
O	4.955482	−1.732304	−0.037835
C	5.094165	−3.129999	0.229575
O	3.726709	−2.341600	−2.371880
C	3.105537	−2.635188	−3.615045
C	5.208189	0.245760	1.793182
O	6.135673	−0.118893	2.473485
O	5.289663	1.173667	0.850061
C	6.440954	2.779876	−0.477022
C	6.593159	1.747141	0.610961
O	0.599911	2.289632	−1.464842
C	1.071919	2.456685	−2.802869
O	−1.942489	3.217057	−2.093992
C	−1.663891	4.254095	−1.147502
C	−4.682996	2.563166	−1.133112
O	−4.117677	1.467393	−1.853954
H	−2.823780	−2.357610	0.995217
H	−0.482758	−2.218237	0.398604
H	−0.802116	−2.742081	−1.245866
H	−4.458353	−1.048633	−1.934626
H	−1.646173	−4.558939	0.882444
H	−3.373608	−4.707369	0.519723
H	−2.184711	−4.744860	−0.795973
H	1.215976	−1.329227	−2.604046
H	−6.535606	−1.540090	−0.872272
H	−8.108812	−1.011985	0.977493
H	−4.887249	0.639115	3.248525
H	−3.314535	0.136613	1.396715
H	1.072381	1.793604	1.322617
H	0.015221	0.430281	1.665743
H	1.597686	−0.494881	3.268461
H	3.859430	−1.177622	2.580212
H	1.767975	1.327992	4.928358
H	0.189253	1.387528	4.127161
H	1.458857	2.533827	3.664125
H	4.782269	−3.720054	−0.633907
H	6.149075	−3.303199	0.432313
H	4.503112	−3.412402	1.105816
H	3.825540	−3.222227	−4.179599
H	2.192166	−3.218981	−3.472527
H	2.872207	−1.718951	−4.164156
H	7.410226	3.236511	−0.682279
H	6.072777	2.324085	−1.397137
H	5.748355	3.565453	−0.172065
H	6.956368	2.185600	1.541903
H	7.273478	0.944587	0.320062
H	1.434251	1.504069	−3.200938
H	0.281457	2.851066	−3.446103
H	1.895121	3.166927	−2.757374
H	−2.292546	4.138565	−0.259652
H	−0.612667	4.245357	−0.858044
H	−1.904827	5.192937	−1.642207
H	−4.290948	3.514589	−1.492488
H	−5.756717	2.520133	−1.307427
H	−4.489259	2.457465	−0.061902

ωB97X Energy = −2096.10547627 a.u.

(*aS*,1*S*,3*S*,1'*S*,3'*S*)-**22**, Conf P

C	0.470727	1.777679	−0.832587
C	1.740353	2.253510	−1.175705
C	2.848083	1.776929	−0.478498
C	2.686375	0.896048	0.591324
C	1.416892	0.457915	0.950777
C	0.299718	0.898305	0.230203
O	3.583012	−0.125655	2.608695
C	2.566176	−1.124615	2.538225
C	1.252808	−0.483306	2.120659
C	3.918439	0.436863	1.344677
C	2.483041	−1.783076	3.896801
C	4.807471	−0.507065	0.546799
C	−1.078256	0.450990	0.594199
C	−1.714420	−0.563773	−0.116352
C	−3.007495	−0.934993	0.253312

C	−3.637831	−0.337149	1.336742
C	−2.982223	0.655643	2.072625
C	−1.711789	1.052549	1.677756
C	6.172153	−0.529238	0.824230
C	7.026359	−1.397339	0.162714
C	6.487433	−2.249817	−0.780820
C	5.142773	−2.261942	−1.086298
C	4.306965	−1.378602	−0.415446
C	−1.032542	−1.260678	−1.267015
C	−1.704301	−2.582500	−1.594941
O	−3.113561	−2.371110	−1.717049
C	−3.725181	−2.048287	−0.484234
C	−1.222730	−3.182123	−2.895466
F	7.312125	−3.100945	−1.434877
O	−4.876470	−0.810440	1.681466
C	−5.943084	0.140983	1.758769
O	−3.642490	1.154278	3.149630
C	−2.995034	2.156812	3.919240
C	−5.173182	−1.736140	−0.857768
O	−6.066680	−2.542299	−0.773540
O	−5.314675	−0.515003	−1.358532
C	−6.559467	1.269454	−2.325415
C	−6.630169	−0.154647	−1.835906
O	−0.615776	2.160369	−1.575782
C	−1.151243	3.433773	−1.222748
O	1.849185	3.131885	−2.219413
C	2.379858	4.418600	−1.890027
C	4.610689	1.834655	−2.066528
O	4.116956	2.204745	−0.775407
H	2.858199	−1.874663	1.791722
H	0.528145	−1.263793	1.873381
H	0.838144	0.066811	2.973057
H	4.515213	1.317226	1.589109
H	1.699072	−2.542615	3.900100
H	3.429998	−2.260824	4.151532
H	2.248287	−1.039807	4.662212
H	−1.186715	1.825094	2.225492
H	6.577251	0.149564	1.567612
H	8.089994	−1.415624	0.366038
H	4.762781	−2.943958	−1.836859
H	3.250303	−1.365930	−0.661232
H	−1.050616	−0.617026	−2.153931
H	0.019255	−1.438513	−1.025525
H	−1.535490	−3.292142	−0.772220
H	−3.766215	−2.940492	0.154139
H	−1.711972	−4.138257	−3.084823
H	−0.144267	−3.345272	−2.855872
H	−1.440492	−2.505427	−3.724708
H	−5.875523	0.858413	0.936315
H	−6.865856	−0.430713	1.668563
H	−5.923872	0.673124	2.708308
H	−3.687703	2.416388	4.715891
H	−2.064979	1.780386	4.353826
H	−2.785233	3.044842	3.316911
H	−7.536985	1.572570	−2.703110
H	−6.278582	1.946381	−1.516721
H	−5.832499	1.367340	−3.132623
H	−6.910164	−0.848167	−2.630478
H	−7.341481	−0.269589	−1.015675
H	−1.470228	3.439469	−0.175895
H	−0.417446	4.226562	−1.388750
H	−2.013533	3.600444	−1.865324
H	2.258084	5.034555	−2.778557
H	1.822653	4.861655	−1.059700
H	3.435976	4.356955	−1.622843
H	5.626514	2.220036	−2.125577
H	4.627691	0.746514	−2.168638
H	4.002971	2.270323	−2.861580

ωB97X Energy = −2096.10545556 a.u.

(*aS*,1*S*,3*S*,1'*S*,3'*S*)-**22**, Conf Q

C	0.485793	2.007983	−0.139248
C	1.765745	2.551343	−0.287813
C	2.859869	1.836176	0.193735
C	2.674600	0.634662	0.877979
C	1.395056	0.120949	1.056844
C	0.291441	0.809875	0.538258
O	3.538462	−1.053592	2.399605
C	2.504296	−1.943974	1.982839
C	1.205351	−1.172380	1.814444
C	3.892663	−0.091403	1.411832
C	2.398593	−3.034557	3.024990
C	4.755200	−0.719824	0.325523
C	−1.095076	0.279515	0.705490
C	−1.735440	−0.383446	−0.338931
C	−3.028424	−0.866953	−0.134238
C	−3.654346	−0.715927	1.093264
C	−3.015423	−0.042538	2.138417
C	−1.732998	0.447218	1.932494
C	6.114936	−0.893453	0.571975
C	6.942826	−1.501302	−0.358776
C	6.382426	−1.938573	−1.542902
C	5.041812	−1.787306	−1.827927
C	4.232702	−1.169954	−0.882898
C	−1.059615	−0.596046	−1.670307
C	−1.739871	−1.693554	−2.470311
O	−3.148058	−1.442478	−2.502243
C	−3.760031	−1.603495	−1.239433
C	−1.262399	−1.763996	−3.902001
F	7.181178	−2.532293	−2.460338
O	−4.939327	−1.178041	1.236413
C	−5.064033	−2.350239	2.045306
O	−3.718077	0.086261	3.292055
C	−3.094107	0.762375	4.374137
C	−5.201877	−1.142187	−1.450526
O	−6.116607	−1.903860	−1.649007
O	−5.309516	0.178774	−1.466611
C	−6.506545	2.224459	−1.691568
C	−6.628031	0.721711	−1.694229
O	−0.587067	2.650891	−0.700283
C	−1.109964	3.718946	0.086320

O	1.897547	3.742299	−0.948553
C	2.447651	4.815576	−0.179678
C	4.635485	2.418773	−1.269500
O	4.138127	2.317291	0.068551
H	2.788652	−2.391054	1.021415
H	0.472247	−1.801555	1.302392
H	0.789788	−0.953898	2.804701
H	4.513840	0.629620	1.945532
H	1.602557	−3.733154	2.760640
H	3.335731	−3.587965	3.098956
H	2.170209	−2.601388	4.001582
H	−1.212060	0.969643	2.725148
H	6.537793	−0.542149	1.507489
H	8.002379	−1.633980	−0.178115
H	4.643573	−2.141124	−2.770857
H	3.179789	−1.030287	−1.103848
H	−1.077962	0.335253	−2.247546
H	−0.008002	−0.854941	−1.518092
H	−1.576671	−2.661510	−1.974822
H	−3.820037	−2.671559	−0.991233
H	−1.760872	−2.574087	−4.435642
H	−0.185615	−1.940124	−3.930237
H	−1.472349	−0.823056	−4.415370
H	−6.117391	−2.622903	2.034551
H	−4.471144	−3.168908	1.627104
H	−4.745801	−2.147738	3.069407
H	−3.814047	0.751825	5.188590
H	−2.181681	0.248162	4.687985
H	−2.859483	1.797290	4.110627
H	−7.489015	2.668879	−1.857165
H	−6.124290	2.582121	−0.734489
H	−5.838155	2.561754	−2.484882
H	−7.000344	0.342894	−2.647563
H	−7.287600	0.364342	−0.901145
H	−0.364335	4.506394	0.222976
H	−1.962975	4.120527	−0.457096
H	−1.440903	3.350327	1.062165
H	1.898000	4.935542	0.758255
H	3.503010	4.647161	0.040469
H	2.334285	5.713352	−0.783752
H	4.630571	1.437493	−1.750932
H	4.044535	3.122023	−1.859002
H	5.659708	2.776317	−1.185948

ωB97X Energy = −2096.10540626 a.u.

(*aS*,1*S*,3*S*,1'*S*,3'*S*)-**22**, Conf R

C	0.487770	1.889589	−0.725761
C	1.767285	2.365832	−1.028746
C	2.865216	1.850124	−0.346462
C	2.692180	0.909845	0.667878
C	1.412098	0.481112	1.003567
C	0.303325	0.970003	0.303513
O	3.567009	−0.231974	2.629993
C	2.528523	−1.202936	2.506690
C	1.228980	−0.511032	2.127566
C	3.913877	0.392429	1.398400
C	2.433117	−1.932904	3.827436
C	4.786610	−0.521956	0.549076
C	−1.080028	0.525671	0.650087
C	−1.690404	−0.523501	−0.032737
C	−2.987587	−0.893783	0.324210
C	−3.648077	−0.258297	1.366847
C	−3.018702	0.770236	2.076044
C	−1.743507	1.163815	1.693743
C	4.271160	−1.317768	−0.469286
C	5.089724	−2.173157	−1.195153
C	6.433175	−2.210336	−0.886356
C	6.986628	−1.434756	0.113365
C	6.149203	−0.593124	0.828677
C	−0.978330	−1.266989	−1.135727
C	−1.619221	−2.619122	−1.395349
O	−3.027423	−2.439127	−1.566483
C	−3.678427	−2.048280	−0.374067
C	−1.094905	−3.292305	−2.642206
F	7.242033	−3.033673	−1.593722
O	−4.890271	−0.729807	1.701863
C	−5.967085	0.213081	1.709630
O	−3.708610	1.304382	3.116731
C	−3.090462	2.345924	3.858382
C	−5.114221	−1.761625	−0.810525
O	−6.007168	−2.567393	−0.717498
O	−5.243198	−0.566220	−1.372922
C	−6.455113	1.159971	−2.476552
C	−6.543218	−0.234202	−1.909707
O	−0.620222	2.368293	−1.366635
C	−0.637073	2.290174	−2.794180
O	1.957183	3.332102	−1.982678
C	1.579168	4.641964	−1.552923
C	4.665514	2.045151	−1.880305
O	4.126744	2.326766	−0.585321
H	2.803246	−1.917096	1.719219
H	0.485016	−1.261924	1.848179
H	0.830938	0.008467	3.006781
H	4.525955	1.247399	1.690405
H	1.635952	−2.677711	3.790589
H	3.371761	−2.440036	4.054690
H	2.211931	−1.228063	4.632270
H	−1.238353	1.964446	2.219063
H	3.215990	−1.264791	−0.715717
H	4.697752	−2.795482	−1.990137
H	8.048709	−1.490582	0.317932
H	6.566366	0.025989	1.616171
H	−1.001283	−0.681893	−2.062727
H	0.074664	−1.406878	−0.875993
H	−1.459601	−3.270922	−0.524348
H	−3.739027	−2.902822	0.312242
H	−1.568655	−4.264291	−2.784240
H	−0.016395	−3.439827	−2.562782
H	−1.296676	−2.672381	−3.518614
H	−6.882073	−0.371026	1.619351
H	−5.978117	0.785302	2.635770

H	−5.883665	0.895783	0.859527
H	−3.803797	2.628889	4.628362
H	−2.165947	1.999709	4.328239
H	−2.877884	3.210857	3.224470
H	−7.421004	1.442363	−2.897662
H	−6.194467	1.881096	−1.700053
H	−5.706181	1.210932	−3.267845
H	−6.800114	−0.971166	−2.672255
H	−7.278937	−0.302886	−1.105987
H	−0.138124	1.380244	−3.137113
H	−1.685731	2.259353	−3.085308
H	−0.152561	3.156350	−3.245971
H	0.507793	4.687904	−1.339958
H	2.143929	4.928092	−0.661824
H	1.819516	5.319296	−2.369951
H	5.658403	2.490121	−1.900230
H	4.749350	0.964946	−2.029020
H	4.047539	2.486021	−2.662835

ωB97X Energy = −2096.10540617 a.u.

(*aS*,1*S*,3*S*,1'*S*,3'*S*)-**22**, Conf S

C	0.841286	1.924546	−1.080549
C	2.186367	2.185637	−1.361674
C	3.171543	1.630613	−0.550343
C	2.826797	0.871089	0.566463
C	1.486444	0.659159	0.872468
C	0.487765	1.183453	0.044132
O	3.445636	−0.083036	2.716334
C	2.295999	−0.925753	2.652368
C	1.118913	−0.136345	2.102811
C	3.932492	0.313555	1.438529
C	2.043286	−1.453621	4.046420
C	4.719282	−0.813748	0.783154
C	−0.956578	0.955081	0.349972
C	−1.652537	−0.097583	−0.240008
C	−2.996721	−0.280372	0.088490
C	−3.622496	0.553747	1.001276
C	−2.926318	1.616103	1.585888
C	−1.591513	1.802902	1.254165
C	6.039965	−1.018053	1.176051
C	6.795053	−2.054301	0.649969
C	6.201269	−2.888422	−0.276914
C	4.897576	−2.723040	−0.694333
C	4.162068	−1.674146	−0.157587
C	−0.985194	−1.051362	−1.199877
C	−1.771265	−2.344250	−1.333278
O	−3.139252	−2.029752	−1.607490
C	−3.790365	−1.426138	−0.510024
C	−1.276032	−3.223848	−2.457409
F	6.929526	−3.902695	−0.799792
O	−4.954291	0.352360	1.269576
C	−5.224076	−0.199732	2.560194
O	−3.628185	2.396657	2.445273
C	−2.946911	3.480448	3.060980
C	−5.135781	−0.960722	−1.066642
O	−5.265442	−0.036685	−1.829593
O	−6.129381	−1.740506	−0.656471
C	−8.424506	−2.354334	−0.479281
C	−7.453764	−1.393004	−1.116852
O	−0.161551	2.450332	−1.845575
C	−0.125268	2.181400	−3.249538
O	2.549212	2.978774	−2.419456
C	2.342299	4.373875	−2.185413
C	5.043670	1.375929	−1.985715
O	4.496660	1.896682	−0.770658
H	2.511887	−1.768249	1.981867
H	0.296031	−0.819844	1.876348
H	0.752651	0.546824	2.877750
H	4.636628	1.118815	1.654200
H	1.155836	−2.089346	4.054344
H	2.893925	−2.040858	4.394908
H	1.882170	−0.625515	4.740524
H	−1.025415	2.614983	1.692660
H	6.489768	−0.351423	1.904593
H	7.824971	−2.215348	0.943703
H	4.472785	−3.397584	−1.427461
H	3.141052	−1.523821	−0.492016
H	−0.894371	−0.589145	−2.189820
H	0.030005	−1.275934	−0.861110
H	−1.728816	−2.894716	−0.382407
H	−3.983411	−2.179119	0.265207
H	−1.848343	−4.151046	−2.503479
H	−0.224333	−3.471703	−2.303372
H	−1.373033	−2.703805	−3.413006
H	−4.904979	0.486575	3.346525
H	−6.300869	−0.347841	2.615359
H	−4.717796	−1.162203	2.679118
H	−3.675301	3.964267	3.706869
H	−2.105224	3.127015	3.662671
H	−2.589519	4.196063	2.315864
H	−9.437467	−2.119906	−0.809576
H	−8.199736	−3.383004	−0.764190
H	−8.390895	−2.275016	0.608317
H	−7.655255	−0.359004	−0.830979
H	−7.470258	−1.457497	−2.206138
H	0.243272	1.169936	−3.438622
H	−1.151730	2.264627	−3.602409
H	0.507150	2.896769	−3.776047
H	2.688860	4.893188	−3.076615
H	1.282570	4.587792	−2.023257
H	2.921511	4.704550	−1.319394
H	6.104063	1.620269	−1.971128
H	4.923256	0.289533	−2.020879
H	4.568861	1.833799	−2.853677

ωB97X Energy = −2096.10539029 a.u.

(*aS*,1*S*,3*S*,1'*S*,3'*S*)-**22**, Conf T

C	0.685330	−0.970280	1.838831
C	1.982070	−1.009844	2.352656
C	3.060741	−1.024607	1.475647

C	2.853494	−0.980305	0.098531
C	1.557370	−0.946198	−0.409007
C	0.463347	−0.954607	0.465884
O	3.697054	−1.351613	−2.145537
C	2.611131	−0.585407	−2.667299
C	1.341295	−0.925691	−1.904041
C	4.055530	−0.971040	−0.822605
C	2.503471	−0.894337	−4.143134
C	4.823460	0.346797	−0.836454
C	−0.939125	−0.939408	−0.048117
C	−1.685186	0.236690	−0.057682
C	−2.997526	0.195563	−0.528522
C	−3.541267	−0.981564	−1.026088
C	−2.774430	−2.151881	−1.055983
C	−1.485272	−2.120768	−0.541555
C	6.076367	0.363960	−1.446699
C	6.817161	1.531083	−1.533290
C	6.280324	2.685892	−0.997252
C	5.046644	2.712929	−0.384346
C	4.320980	1.529836	−0.309018
C	−1.101278	1.542094	0.421290
C	−1.899695	2.728903	−0.088684
O	−3.285209	2.512433	0.190832
C	−3.840660	1.453961	−0.563640
C	−1.505495	4.031056	0.568620
F	6.997097	3.832448	−1.073979
O	−4.806355	−0.921389	−1.551844
C	−5.788800	−1.787887	−0.971189
O	−3.350457	−3.247416	−1.615160
C	−2.593170	−4.448207	−1.656588
C	−5.218950	1.247238	0.061625
O	−5.405756	0.664742	1.101003
O	−6.168206	1.847169	−0.648814
C	−7.737200	2.891815	0.902186
C	−7.516675	1.804685	−0.125061
O	−0.372832	−0.892123	2.706488
C	−0.742803	−2.144598	3.284361
O	2.185509	−1.049110	3.706846
C	2.299029	0.248530	4.294981
C	4.761403	−2.250413	2.551799
O	4.345459	−1.019188	1.953798
H	2.838062	0.480929	−2.534231
H	0.557910	−0.206790	−2.159254
H	0.980867	−1.907578	−2.231218
H	4.747938	−1.748118	−0.491795
H	1.664999	−0.350721	−4.582548
H	3.416826	−0.604847	−4.664620
H	2.338619	−1.963831	−4.293395
H	−0.877650	−3.016915	−0.539422
H	6.479633	−0.552718	−1.864074
H	7.792902	1.553133	−2.002773
H	4.663058	3.639376	0.024908
H	3.350256	1.539519	0.174437
H	−1.083861	1.562576	1.516838
H	−0.064507	1.632266	0.085007
H	−1.777718	2.809731	−1.178406
H	−3.979742	1.772270	−1.604533
H	−2.086519	4.860315	0.163512
H	−0.446538	4.231749	0.396643
H	−1.676585	3.974979	1.645927
H	−6.744910	−1.478055	−1.389967
H	−5.591034	−2.828443	−1.223383
H	−5.808417	−1.661416	0.114020
H	−3.229248	−5.186154	−2.138965
H	−1.678514	−4.318511	−2.241381
H	−2.338910	−4.789855	−0.649627
H	−8.777195	2.871925	1.232568
H	−7.098060	2.744402	1.773299
H	−7.532615	3.873880	0.473377
H	−8.147828	1.946761	−1.000126
H	−7.697523	0.813583	0.291742
H	−1.070054	−2.841371	2.506565
H	0.093089	−2.574331	3.841071
H	−1.569777	−1.943873	3.962532
H	2.465352	0.097245	5.359642
H	3.145636	0.790051	3.864521
H	1.377505	0.816946	4.144563
H	4.146310	−2.487776	3.421083
H	4.705225	−3.064305	1.822948
H	5.795808	−2.110025	2.858502

ωB97X Energy = −2096.10537681 a.u.

(*aS*,1*S*,3*S*,1'*S*,3'*S*)-**22**, Conf U

C	0.754385	1.822740	−1.107165
C	2.083193	2.139942	−1.407379
C	3.095639	1.629593	−0.598145
C	2.785512	0.878882	0.535997
C	1.459901	0.603282	0.851446
C	0.435628	1.074427	0.020424
O	3.460182	−0.022834	2.690606
C	2.353396	−0.922613	2.643674
C	1.136434	−0.199409	2.089778
C	3.920780	0.376580	1.404284
C	2.133005	−1.444309	4.045448
C	4.748475	−0.726838	0.758625
C	−0.996865	0.785103	0.330464
C	−1.682658	−0.223324	−0.342736
C	−3.014511	−0.468145	−0.006380
C	−3.636873	0.255582	0.998810
C	−2.952659	1.276632	1.665242
C	−1.630646	1.527712	1.324214
C	6.074959	−0.877832	1.156036
C	6.868629	−1.891498	0.643336
C	6.307707	−2.757299	−0.275115
C	4.999840	−2.644327	−0.696828
C	4.224748	−1.617495	−0.172979
C	−1.014754	−1.059429	−1.405394
C	−1.781615	−2.344619	−1.665495
O	−3.161897	−2.031013	−1.873776
C	−3.796782	−1.564966	−0.702110
C	−1.296775	−3.083573	−2.890918

F	7.073714	−3.750227	−0.784862
O	−4.956441	−0.005748	1.274562
C	−5.186559	−0.671147	2.518147
O	−3.651359	1.957125	2.608619
C	−2.982965	3.003398	3.298765
C	−5.168617	−1.080844	−1.173565
O	−5.344121	−0.068700	−1.803474
O	−6.119401	−1.956729	−0.862642
C	−8.109314	−0.745004	−0.160458
C	−7.477827	−1.616213	−1.222786
O	−0.242186	2.223857	−1.958531
C	−0.691185	3.561682	−1.756328
O	2.344299	2.895778	−2.518062
C	2.966940	4.158400	−2.265058
C	4.940206	1.377346	−2.074224
O	4.417241	1.896827	−0.847811
H	2.608490	−1.762142	1.983544
H	0.349058	−0.925378	1.869751
H	0.738189	0.469339	2.861315
H	4.592782	1.212987	1.603442
H	1.283114	−2.129199	4.063729
H	3.015927	−1.977997	4.399766
H	1.926402	−0.617415	4.728865
H	−1.073913	2.308755	1.826832
H	6.498032	−0.187060	1.878085
H	7.902919	−2.011900	0.941190
H	4.601639	−3.342186	−1.423032
H	3.199012	−1.509490	−0.509698
H	−0.942343	−0.487352	−2.337190
H	0.007495	−1.302115	−1.101647
H	−1.708802	−2.997665	−0.783831
H	−3.950140	−2.400189	−0.006280
H	−1.852134	−4.012079	−3.028290
H	−0.237281	−3.324592	−2.787444
H	−1.424944	−2.462589	−3.780219
H	−4.865676	−0.048222	3.354809
H	−6.259084	−0.846564	2.580661
H	−4.658383	−1.628852	2.543584
H	−3.707981	3.412493	3.997878
H	−2.119307	2.623272	3.851073
H	−2.660106	3.787069	2.608157
H	−9.141773	−0.523872	−0.436713
H	−8.115033	−1.256523	0.803672
H	−7.567592	0.195665	−0.054707
H	−7.470634	−1.124931	−2.195465
H	−7.983977	−2.575811	−1.309380
H	0.115535	4.277688	−1.932923
H	−1.489785	3.735479	−2.474819
H	−1.080676	3.687817	−0.741501
H	2.357181	4.755540	−1.580974
H	3.964799	4.031321	−1.841752
H	3.033771	4.663597	−3.226225
H	4.483383	1.863882	−2.937561
H	6.009118	1.579969	−2.057797
H	4.776123	0.298265	−2.130844

ωB97X Energy = −2096.10531262 a.u.

(*aS*,1*S*,3*S*,1'*S*,3'*S*)-**22**, Conf V

C	0.499114	2.060756	0.081266
C	1.787740	2.596716	−0.006858
C	2.872754	1.833806	0.415294
C	2.678171	0.573338	0.976931
C	1.389011	0.068833	1.116679
C	0.293518	0.812037	0.663124
O	3.522136	−1.263338	2.330110
C	2.467770	−2.089435	1.838433
C	1.182494	−1.281299	1.762380
C	3.886652	−0.213310	1.440332
C	2.354343	−3.276740	2.767945
C	4.742859	−0.741378	0.297125
C	−1.097827	0.283819	0.793364
C	−1.715149	−0.372194	−0.269205
C	−3.013775	−0.854108	−0.097591
C	−3.668325	−0.707044	1.115094
C	−3.051552	−0.042107	2.178966
C	−1.763564	0.445800	2.005700
C	4.214806	−1.063730	−0.949168
C	5.017362	−1.589455	−1.953259
C	6.357231	−1.778738	−1.687985
C	6.922950	−1.468147	−0.466981
C	6.101329	−0.950097	0.522024
C	−1.008720	−0.589228	−1.584397
C	−1.666068	−1.696221	−2.390465
O	−3.071621	−1.442744	−2.466225
C	−3.721432	−1.587626	−1.220100
C	−1.146279	−1.785230	−3.806311
F	7.149962	−2.282476	−2.662728
O	−4.958149	−1.165657	1.223986
C	−5.107525	−2.342603	2.021590
O	−3.780356	0.080543	3.316786
C	−3.179254	0.744813	4.419084
C	−5.150362	−1.109521	−1.476416
O	−6.066711	−1.860336	−1.706097
O	−5.242621	0.212637	−1.492985
C	−6.409191	2.272173	−1.750603
C	−6.546224	0.770815	−1.765526
O	−0.597151	2.770440	−0.320292
C	−0.596705	3.285324	−1.654231
O	2.000346	3.858192	−0.499579
C	1.635079	4.896529	0.412524
C	4.687111	2.573274	−0.922514
O	4.143042	2.344756	0.380701
H	2.733653	−2.443067	0.833467
H	0.427493	−1.849472	1.212139
H	0.788144	−1.140105	2.775275
H	4.515291	0.445607	2.041425
H	1.543532	−3.933029	2.446148
H	3.282687	−3.849531	2.774045
H	2.144561	−2.940301	3.785934
H	−1.259832	0.963438	2.812281
H	3.162855	−0.893286	−1.152290

H	4.615196	−1.841975	−2.926592
H	7.981667	−1.626486	−0.302864
H	6.528342	−0.699000	1.487426
H	−1.022029	0.333254	−2.176717
H	0.041626	−0.838421	−1.409790
H	−1.517903	−2.657629	−1.877912
H	−3.802201	−2.653232	−0.967540
H	−1.630956	−2.600566	−4.344530
H	−0.069645	−1.964452	−3.799672
H	−1.338366	−0.850212	−4.337309
H	−6.158431	−2.619995	1.969612
H	−4.495485	−3.156469	1.621974
H	−4.828892	−2.144390	3.057944
H	−3.917575	0.728708	5.216813
H	−2.275678	0.224980	4.748745
H	−2.935915	1.781337	4.170804
H	−7.380578	2.727654	−1.948361
H	−6.057509	2.620947	−0.778658
H	−5.709762	2.606492	−2.518015
H	−6.887177	0.400612	−2.733873
H	−7.238101	0.416719	−0.999027
H	−0.091125	2.595501	−2.334537
H	−1.641828	3.380261	−1.944194
H	−0.108409	4.258929	−1.705038
H	2.203824	4.805260	1.341463
H	1.878810	5.838596	−0.074412
H	0.564554	4.862123	0.632037
H	5.683676	2.982605	−0.769091
H	4.762741	1.630698	−1.471753
H	4.077720	3.285320	−1.479519

ωB97X Energy = −2096.10529428 a.u.

(*aS*,1*S*,3*S*,1'*S*,3'*S*)-**22**, Conf W

C	0.708633	−0.585026	1.997427
C	2.019553	−0.562404	2.486026
C	3.068475	−0.815175	1.606682
C	2.813231	−1.078758	0.259506
C	1.508665	−1.091902	−0.218650
C	0.444308	−0.848848	0.659552
O	3.582094	−1.935288	−1.884087
C	2.508778	−1.245453	−2.522907
C	1.250508	−1.374806	−1.679851
C	3.990339	−1.339607	−0.657593
C	2.346598	−1.840497	−3.903433
C	4.848512	−0.106138	−0.910315
C	−0.971488	−0.878098	0.184797
C	−1.667796	0.302927	−0.059300
C	−2.993068	0.226004	−0.489161
C	−3.598842	−1.002547	−0.719394
C	−2.879869	−2.187652	−0.520732
C	−1.578466	−2.109345	−0.043853
C	4.343125	1.188250	−0.846954
C	5.145643	2.286027	−1.130742
C	6.462581	2.062657	−1.471619
C	7.005807	0.794743	−1.542330
C	6.184929	−0.285744	−1.259782
C	−1.012602	1.650289	0.112121
C	−1.754745	2.726311	−0.659822
O	−3.143232	2.660859	−0.325645
C	−3.775369	1.485573	−0.795286
C	−1.280412	4.123375	−0.333940
F	7.255762	3.126017	−1.742559
O	−4.872695	−0.995759	−1.228053
C	−5.868951	−1.692068	−0.469665
O	−3.509346	−3.348984	−0.836901
C	−2.795661	−4.563723	−0.657937
C	−5.139197	1.528859	−0.106805
O	−5.341987	1.162227	1.023813
O	−6.042757	2.116099	−0.884467
C	−8.242096	1.166700	−0.362754
C	−7.346942	2.383631	−0.316610
O	−0.329216	−0.329825	2.858976
C	−0.727608	−1.464275	3.630683
O	2.278029	−0.386587	3.818970
C	1.988103	0.917470	4.332873
C	4.928043	0.267598	2.644154
O	4.368904	−0.889994	2.022076
H	2.775258	−0.184120	−2.615598
H	0.484625	−0.694217	−2.061798
H	0.849554	−2.388729	−1.790401
H	4.629302	−2.090568	−0.190286
H	1.516498	−1.361422	−4.426009
H	3.254851	−1.700539	−4.491121
H	2.138574	−2.910457	−3.829814
H	−1.007258	−3.012275	0.131665
H	3.308812	1.354062	−0.564953
H	4.760360	3.297103	−1.083210
H	8.047986	0.664074	−1.806433
H	6.594879	−1.289257	−1.308932
H	−0.985001	1.921988	1.173533
H	0.024345	1.605125	−0.232081
H	−1.650739	2.538496	−1.738168
H	−3.936506	1.559443	−1.878304
H	−1.826936	4.863074	−0.920132
H	−0.216312	4.220751	−0.556326
H	−1.433040	4.333619	0.726956
H	−5.905669	−1.300014	0.550109
H	−6.815987	−1.496396	−0.969009
H	−5.672138	−2.762432	−0.453551
H	−3.470217	−5.355194	−0.974802
H	−1.893352	−4.585958	−1.275091
H	−2.526842	−4.713815	0.391060
H	−9.244965	1.446341	−0.035068
H	−8.311774	0.776061	−1.379554
H	−7.873145	0.381060	0.296412
H	−7.213843	2.745591	0.703231
H	−7.740698	3.189644	−0.932820
H	−1.099551	−2.258028	2.975907
H	0.107247	−1.837124	4.229149
H	−1.528637	−1.130539	4.287246
H	2.566068	1.675952	3.796641

H	0.923884	1.139753	4.252367
H	2.287055	0.907324	5.379199
H	6.008988	0.150827	2.588063
H	4.637015	1.172896	2.103732
H	4.620794	0.341666	3.687437

ωB97X Energy = −2096.10524763 a.u.

(*aS*,1*S*,3*S*,1’*S*,3’*S*)-**22**, Conf X

C	0.767670	1.926148	−1.008453
C	2.103115	2.244122	−1.276048
C	3.105930	1.695893	−0.482011
C	2.786670	0.885581	0.606198
C	1.453937	0.615533	0.900267
C	0.438474	1.133687	0.088560
O	3.435077	−0.126151	2.721190
C	2.316836	−1.006588	2.619913
C	1.113209	−0.238078	2.099227
C	3.910371	0.338888	1.462108
C	2.081474	−1.598938	3.990929
C	4.744977	−0.729165	0.768418
C	−0.996981	0.842464	0.382078
C	−1.665079	−0.187279	−0.276815
C	−3.000243	−0.434286	0.044920
C	−3.644590	0.310813	1.019803
C	−2.977862	1.352668	1.671758
C	−1.651952	1.604235	1.346487
C	6.076798	−0.881622	1.146548
C	6.876185	−1.864484	0.584596
C	6.315240	−2.698379	−0.362691
C	5.001879	−2.583008	−0.766813
C	4.221696	−1.586647	−0.194426
C	−0.975729	−1.052211	−1.302806
C	−1.721868	−2.357858	−1.517652
O	−3.100860	−2.069329	−1.764851
C	−3.763734	−1.558010	−0.627834
C	−1.206374	−3.146129	−2.698965
F	7.086609	−3.661074	−0.920197
O	−4.967402	0.046937	1.277861
C	−5.218530	−0.578633	2.538041
O	−3.696803	2.051235	2.586269
C	−3.045713	3.115122	3.266184
C	−5.123535	−1.094171	−1.151475
O	−5.285882	−0.098873	−1.811062
O	−6.078052	−1.968521	−0.848107
C	−8.098454	−0.755961	−0.241430
C	−7.426407	−1.647666	−1.260902
O	−0.252244	2.442219	−1.756238
C	−0.193106	2.255880	−3.172849
O	2.438859	3.087378	−2.303681
C	2.190873	4.465329	−2.015330
C	4.987535	1.554407	−1.919231
O	4.420855	2.017269	−0.689870
H	2.563735	−1.813174	1.916912
H	0.314458	−0.940174	1.845097
H	0.723726	0.401477	2.899537
H	4.580642	1.162645	1.712886
H	1.221893	−2.271555	3.969433
H	2.954985	−2.163144	4.320433
H	1.883024	−0.806419	4.716232
H	−1.108608	2.401822	1.837226
H	6.500202	−0.215559	1.891282
H	7.914872	−1.985165	0.866612
H	4.603900	−3.255204	−1.516948
H	3.192260	−1.474421	−0.518047
H	−0.908318	−0.522861	−2.260433
H	0.048893	−1.265824	−0.986062
H	−1.657051	−2.967888	−0.605162
H	−3.934067	−2.365591	0.096061
H	−1.751281	−4.084850	−2.804974
H	−0.147244	−3.372747	−2.564107
H	−1.322337	−2.567504	−3.618091
H	−6.291402	−0.756711	2.585913
H	−4.687261	−1.532624	2.605069
H	−4.916205	0.072981	3.359499
H	−3.784589	3.533820	3.944818
H	−2.189413	2.751105	3.840358
H	−2.715623	3.886644	2.565617
H	−9.123594	−0.551821	−0.555438
H	−8.129698	−1.243111	0.734700
H	−7.569079	0.192514	−0.143423
H	−7.390266	−1.180055	−2.244520
H	−7.922685	−2.613003	−1.340449
H	−1.223544	2.260788	−3.524653
H	0.366804	3.057342	−3.655685
H	0.270307	1.296942	−3.417439
H	2.764573	4.780409	−1.139884
H	2.515882	5.029020	−2.887549
H	1.125980	4.640593	−1.839948
H	4.906298	0.466013	−1.988197
H	4.497303	2.021461	−2.773726
H	6.038405	1.836256	−1.895057

ωB97X Energy = −2096.10521619 a.u.

(*aS*,1*S*,3*S*,1’*S*,3’*S*)-**22**, Conf Y

C	0.769773	1.956908	−1.081727
C	2.105458	2.241207	−1.384041
C	3.112725	1.702172	−0.592666
C	2.794854	0.924003	0.521569
C	1.464543	0.672988	0.838038
C	0.442132	1.183167	0.025895
O	3.461002	−0.014645	2.664778
C	2.333824	−0.887638	2.610833
C	1.131094	−0.129141	2.073831
C	3.923756	0.396805	1.383011
C	2.108970	−1.424865	4.006033
C	4.738620	−0.705558	0.719901
C	−0.993949	0.927729	0.350049
C	−1.635091	−0.234601	−0.072479
C	−2.978358	−0.420130	0.261401
C	−3.658451	0.513321	1.031500

C	−2.997312	1.658529	1.492711
C	−1.675210	1.862170	1.124556
C	6.066957	−0.870784	1.105536
C	6.849840	−1.883115	0.573981
C	6.276344	−2.733128	−0.351378
C	4.966272	−2.605868	−0.762053
C	4.202302	−1.580402	−0.219606
C	−0.910211	−1.310356	−0.844450
C	−1.606694	−2.651167	−0.696900
O	−2.984361	−2.492137	−1.042647
C	−3.703726	−1.691609	−0.124845
C	−1.037011	−3.717482	−1.603167
F	7.031828	−3.724402	−0.879606
O	−4.949356	0.220405	1.391220
C	−5.949694	1.171200	1.006258
O	−3.702330	2.488793	2.303393
C	−3.045055	3.641829	2.809290
C	−5.024901	−1.429815	−0.847106
O	−5.193374	−0.558091	−1.662927
O	−5.928346	−2.349978	−0.526517
C	−8.154544	−1.334044	−0.661286
C	−7.185280	−2.337033	−1.243668
O	−0.228089	2.514634	−1.837673
C	−0.363759	1.963005	−3.148915
O	2.422604	3.040063	−2.451931
C	2.286632	4.436785	−2.177130
C	4.973331	1.499651	−2.050965
O	4.429505	1.997897	−0.824841
H	2.565277	−1.722909	1.936208
H	0.323119	−0.834713	1.863190
H	0.757179	0.548368	2.850275
H	4.605298	1.222801	1.592667
H	1.242760	−2.089106	4.019810
H	2.980854	−1.984898	4.346702
H	1.926746	−0.603430	4.702809
H	−1.145136	2.744826	1.458662
H	6.500204	−0.192026	1.832928
H	7.885524	−2.014181	0.862324
H	4.558142	−3.291453	−1.494404
H	3.175529	−1.460459	−0.548718
H	−0.864258	−1.057982	−1.909973
H	0.122421	−1.395126	−0.496294
H	−1.547348	−2.979363	0.350980
H	−3.919598	−2.270971	0.781878
H	−1.553762	−4.666312	−1.454735
H	0.023760	−3.863306	−1.391517
H	−1.144433	−3.419949	−2.648605
H	−5.918809	1.329711	−0.074919
H	−6.904927	0.728576	1.282270
H	−5.812862	2.116559	1.528358
H	−3.774163	4.151236	3.434419
H	−2.176619	3.366835	3.413891
H	−2.732670	4.306924	2.000004
H	−9.120982	−1.439285	−1.157356
H	−8.297863	−1.510153	0.406291
H	−7.802152	−0.313369	−0.810141
H	−6.983267	−2.135664	−2.295966
H	−7.557750	−3.354351	−1.139755
H	−0.606867	0.898213	−3.089726
H	−1.185930	2.493506	−3.624727
H	0.551466	2.103257	−3.726831
H	2.567064	4.962246	−3.087809
H	1.253347	4.677811	−1.916940
H	2.954920	4.731638	−1.363671
H	4.871600	0.411788	−2.096586
H	4.481275	1.957808	−2.909145
H	6.029318	1.762589	−2.044282

ωB97X Energy = −2096.10517991 a.u.

(*aS*,1*S*,3*S*,1’*S*,3’*S*)-**22**, Conf Z

C	−0.702177	−1.497448	−1.491782
C	−2.012721	−1.685139	−1.943131
C	−3.068352	−1.392053	−1.082800
C	−2.822076	−0.996205	0.231894
C	−1.515782	−0.859546	0.688005
C	−0.446969	−1.104163	−0.183163
O	−3.624617	−0.700344	2.507904
C	−2.497845	0.130681	2.784538
C	−1.261235	−0.452890	2.120390
C	−4.002998	−0.706092	1.135708
C	−2.361224	0.223147	4.287404
C	−4.749192	0.571395	0.773563
C	0.967660	−0.942351	0.268657
C	1.669794	0.230259	0.001093
C	2.992774	0.333660	0.430096
C	3.592228	−0.691393	1.150230
C	2.870908	−1.851181	1.454498
C	1.568193	−1.971099	0.988251
C	−4.131403	1.653611	0.155103
C	−4.831945	2.822798	−0.111959
C	−6.162043	2.883900	0.246132
C	−6.815110	1.831459	0.857421
C	−6.094603	0.676721	1.118735
C	1.026803	1.385152	−0.725104
C	1.784482	2.678682	−0.483009
O	3.171020	2.461902	−0.757905
C	3.789350	1.595892	0.172587
C	1.320736	3.811387	−1.368725
F	−6.856567	4.015839	−0.016153
O	4.867753	−0.480263	1.607315
C	5.864757	−1.422749	1.194019
O	3.502019	−2.783716	2.213728
C	2.790978	−3.970895	2.532864
C	5.152707	1.303832	−0.452171
O	5.329960	0.509823	−1.342457
O	6.097973	2.082518	0.063863
C	7.552190	2.813640	−1.755181
C	7.425787	1.976877	−0.502428
O	0.341613	−1.664287	−2.364449
C	0.751191	−3.017370	−2.548100
O	−2.210140	−2.094811	−3.233899

C	−2.865122	−3.357874	−3.381436
C	−4.807343	−0.677249	−2.532846
O	−4.374076	−1.539013	−1.475719
H	−2.688603	1.132272	2.377150
H	−0.446674	0.275066	2.164405
H	−0.928903	−1.327818	2.690815
H	−4.711028	−1.532101	1.051738
H	−1.495444	0.833807	4.550653
H	−3.251212	0.674787	4.727680
H	−2.225343	−0.772450	4.716242
H	0.992367	−2.862849	1.201788
H	−3.088367	1.588089	−0.136118
H	−4.359422	3.670094	−0.593301
H	−7.863424	1.919766	1.114767
H	−6.590809	−0.161353	1.597157
H	0.995385	1.182222	−1.801584
H	−0.008644	1.504270	−0.393998
H	1.684176	2.966187	0.573516
H	3.952244	2.124109	1.120541
H	1.872403	4.725903	−1.147790
H	0.257598	4.000087	−1.209257
H	1.473124	3.554918	−2.419393
H	6.822776	−0.985716	1.471130
H	5.730840	−2.378849	1.697401
H	5.827205	−1.560232	0.110651
H	3.467137	−4.573185	3.134417
H	1.890455	−3.748091	3.111569
H	2.518211	−4.522588	1.629167
H	8.575973	2.754002	−2.128647
H	6.881485	2.453944	−2.536143
H	7.324106	3.859695	−1.545641
H	8.083208	2.335883	0.286993
H	7.638670	0.925442	−0.696807
H	1.591536	−2.998795	−3.239256
H	1.071161	−3.454886	−1.597492
H	−0.055778	−3.616820	−2.976727
H	−3.894725	−3.315808	−3.022145
H	−2.852765	−3.585005	−4.445350
H	−2.323409	−4.135855	−2.835777
H	−4.279617	−0.895243	−3.462920
H	−5.871826	−0.863312	−2.659563
H	−4.651404	0.369002	−2.257671

ωB97X Energy = −2096.10516274 a.u.

(*aS*,1*S*,3*S*,1'*S*,3'*S*)-**22**, Conf AA

C	0.722288	−1.499074	1.543704
C	2.039632	−1.667734	1.982551
C	3.087613	−1.406056	1.104919
C	2.832867	−1.039998	−0.215575
C	1.520054	−0.931963	−0.663440
C	0.458123	−1.156400	0.220125
O	3.612949	−0.809990	−2.507795
C	2.477694	0.005123	−2.795282
C	1.251429	−0.570222	−2.105314
C	4.004209	−0.777605	−1.139321
C	2.326962	0.057901	−4.298786
C	4.754349	0.508788	−0.819356
C	−0.958269	−1.017426	−0.233505
C	−1.656024	0.171416	−0.033500
C	−2.978424	0.255945	−0.469773
C	−3.581416	−0.805807	−1.130830
C	−2.864758	−1.984343	−1.366774
C	−1.563323	−2.083144	−0.892589
C	4.141340	1.608683	−0.227680
C	4.844540	2.783823	0.003198
C	6.172928	2.833083	−0.363321
C	6.821422	1.763317	−0.948666
C	6.098065	0.602745	−1.174311
C	−1.009874	1.366638	0.622714
C	−1.759179	2.646829	0.297254
O	−3.146954	2.455328	0.582499
C	−3.767992	1.536549	−0.294080
C	−1.291355	3.830794	1.110954
F	6.870201	3.970868	−0.135795
O	−4.854930	−0.615332	−1.603034
C	−5.857412	−1.526801	−1.137051
O	−3.499196	−2.955427	−2.073108
C	−2.790933	−4.159101	−2.331040
C	−5.135727	1.293897	0.342027
O	−5.322488	0.558810	1.279729
O	−6.073157	2.045039	−0.226231
C	−7.533293	2.891852	1.537324
C	−7.404942	1.981828	0.336966
O	−0.339403	−1.739186	2.369237
C	−0.371557	−1.046991	3.620091
O	2.313434	−2.082013	3.260417
C	2.068252	−3.473844	3.475296
C	4.872289	−0.711298	2.505593
O	4.389102	−1.593392	1.488176
H	2.664328	1.018511	−2.415795
H	0.429712	0.148541	−2.165256
H	0.923187	−1.464943	−2.646660
H	4.712826	−1.601285	−1.039147
H	1.453870	0.654602	−4.569796
H	3.209353	0.505214	−4.758356
H	2.195610	−0.949408	−4.700840
H	−0.991848	−2.988791	−1.051682
H	3.100161	1.552082	0.071727
H	4.375815	3.644694	0.463710
H	7.868525	1.842933	−1.213682
H	6.590777	−0.248926	−1.631873
H	−0.988101	1.236427	1.711089
H	0.028769	1.458715	0.293464
H	−1.654501	2.865755	−0.775143
H	−3.923817	2.006034	−1.273600
H	−1.841247	4.731018	0.834063
H	−0.227900	4.006987	0.939700
H	−1.444089	3.640038	2.175510
H	−5.819156	−1.604833	−0.047800
H	−6.813097	−1.099574	−1.436746
H	−5.730308	−2.509923	−1.587403

H	−3.466682	−4.787027	−2.906284
H	−1.887068	−3.967451	−2.915544
H	−2.524474	−4.667725	−1.400772
H	−8.560993	2.865095	1.903769
H	−6.873560	2.571564	2.344384
H	−7.291909	3.921402	1.269003
H	−8.054810	2.298180	−0.476600
H	−7.626261	0.944908	0.591080
H	−1.422697	−0.937726	3.882222
H	0.147532	−1.608540	4.397577
H	0.084343	−0.058301	3.526693
H	2.327242	−3.678602	4.512169
H	1.015235	−3.713543	3.305155
H	2.694969	−4.077807	2.814048
H	4.782492	0.328703	2.179741
H	4.330004	−0.859121	3.439752
H	5.923617	−0.954814	2.646023

ωB97X Energy = −2096.10510860 a.u.

(*aS*,1*S*,3*S*,1'*S*,3'*S*)-**22**, Conf AB

C	−0.721122	−1.645132	−1.334071
C	−2.037291	−1.915392	−1.722510
C	−3.078800	−1.535155	−0.879643
C	−2.810710	−0.966573	0.365643
C	−1.497639	−0.743175	0.764224
C	−0.444116	−1.078020	−0.095619
O	−3.562972	−0.414882	2.610236
C	−2.455971	0.474801	2.751477
C	−1.219130	−0.148913	2.124859
C	−3.976201	−0.597597	1.260292
C	−2.281456	0.748956	4.228138
C	−4.769912	0.605785	0.768658
C	0.977244	−0.840073	0.297911
C	1.658869	0.294833	−0.133195
C	2.989930	0.466545	0.248845
C	3.616298	−0.449740	1.083975
C	2.912338	−1.564210	1.556139
C	1.604005	−1.758513	1.134641
C	−4.201291	1.623705	0.009635
C	−4.945041	2.732062	−0.374795
C	−6.267682	2.797184	0.009355
C	−6.872735	1.806511	0.757825
C	−6.109783	0.712426	1.134284
C	0.982315	1.342109	−0.981349
C	1.714591	2.669616	−0.904662
O	3.100395	2.449654	−1.177640
C	3.755865	1.703808	−0.169899
C	1.212146	3.680135	−1.909387
F	−7.003701	3.869559	−0.365911
O	4.895803	−0.167730	1.490108
C	5.892800	−1.152470	1.191525
O	3.562582	−2.377731	2.428001
C	2.861767	−3.503552	2.936130
C	5.111829	1.387757	−0.799983
O	5.309623	0.479585	−1.568027
O	6.013539	2.305502	−0.466913
C	8.224053	1.244402	−0.449892
C	7.305825	2.243416	−1.115606
O	0.305215	−1.910192	−2.202919
C	0.738290	−3.268501	−2.210712
O	−2.254125	−2.495528	−2.942945
C	−2.886072	−3.778379	−2.906967
C	−4.868745	−1.044545	−2.357819
O	−4.389261	−1.760920	−1.215525
H	−2.689467	1.415072	2.234998
H	−0.429765	0.604186	2.051217
H	−0.839691	−0.932915	2.790247
H	−4.660968	−1.446279	1.297355
H	−1.429575	1.411918	4.390873
H	−3.173488	1.222929	4.639861
H	−2.101341	−0.184955	4.765623
H	1.043388	−2.618973	1.477689
H	−3.163927	1.554181	−0.300472
H	−4.511232	3.529044	−0.966023
H	−7.917029	1.894504	1.031097
H	−6.568141	−0.076729	1.721331
H	0.942635	1.013079	−2.025929
H	−0.051402	1.477848	−0.651429
H	1.624344	3.077834	0.112317
H	3.926839	2.338551	0.708898
H	1.748255	4.624634	−1.809761
H	0.148204	3.866461	−1.752757
H	1.351789	3.301907	−2.924504
H	5.915793	−1.347762	0.116159
H	6.841885	−0.720541	1.503417
H	5.707091	−2.075772	1.737309
H	2.575317	−4.187472	2.132739
H	3.551446	−4.006164	3.609603
H	1.970971	−3.196224	3.490708
H	9.214888	1.312217	−0.902462
H	8.320150	1.457229	0.616293
H	7.857200	0.226189	−0.578742
H	7.153686	2.008440	−2.169362
H	7.692825	3.256840	−1.028024
H	−0.066861	−3.935578	−2.528788
H	1.559247	−3.330749	−2.922285
H	1.092755	−3.564399	−1.218538
H	−2.331424	−4.459064	−2.254850
H	−3.917144	−3.704045	−2.557319
H	−2.866725	−4.157255	−3.926671
H	−4.352873	−1.359188	−3.266679
H	−5.929674	−1.272545	−2.437011
H	−4.737862	0.030795	−2.212620

ωB97X Energy = −2096.10505961 a.u.

(*aS*,1*S*,3*S*,1'*S*,3'*S*)-**22**, Conf AC

C	0.747948	−1.503285	1.569768
C	2.069312	−1.643665	1.997521
C	3.103585	−1.392226	1.101642
C	2.828792	−0.978378	−0.200068

C	1.509088	−0.848985	−0.623641
C	0.460880	−1.126708	0.262084
O	3.570094	−0.685022	−2.491876
C	2.438400	0.149230	−2.739637
C	1.217938	−0.435369	−2.047141
C	3.982723	−0.669274	−1.130494
C	2.265836	0.248849	−4.238092
C	4.708363	0.632598	−0.805976
C	−0.963639	−0.999739	−0.169341
C	−1.680462	0.166801	0.086932
C	−3.003759	0.248843	−0.346624
C	−3.587189	−0.796021	−1.046075
C	−2.872394	−1.972134	−1.291319
C	−1.558280	−2.059119	−0.850430
C	5.943023	0.861232	−1.410742
C	6.642644	2.036687	−1.192488
C	6.083528	2.983411	−0.355625
C	4.866048	2.797556	0.262396
C	4.182017	1.610609	0.029125
C	−1.054085	1.342836	0.793556
C	−1.835150	2.619834	0.535372
O	−3.217550	2.384584	0.817605
C	−3.824678	1.498065	−0.099072
C	−1.388399	3.772986	1.403253
F	6.760460	4.134885	−0.132461
O	−4.898440	−0.671798	−1.434946
C	−5.084211	−0.486860	−2.840533
O	−3.534366	−2.955635	−1.951076
C	−2.835140	−4.164720	−2.209339
C	−5.180286	1.177373	0.531614
O	−5.328034	0.428780	1.464325
O	−6.154665	1.879435	−0.038257
C	−7.700956	2.586989	1.715774
C	−7.488161	1.716375	0.498148
O	−0.270688	−1.682013	2.468436
C	−0.592288	−3.050497	2.718909
O	2.328320	−2.042180	3.282004
C	2.695074	−0.967152	4.150296
C	4.867736	−2.817878	1.750434
O	4.411408	−1.484377	1.503426
H	2.641708	1.147795	−2.329868
H	0.403644	0.294162	−2.067128
H	0.868839	−1.307491	−2.611753
H	4.713196	−1.477532	−1.056453
H	1.392892	0.858950	−4.477926
H	3.144372	0.704427	−4.696977
H	2.121839	−0.744938	−4.668496
H	−0.977098	−2.954457	−1.032536
H	6.364588	0.106217	−2.066119
H	7.604591	2.220715	−1.654839
H	4.464629	3.564518	0.913253
H	3.225005	1.452787	0.514391
H	−1.015595	1.156357	1.872831
H	−0.022085	1.475827	0.456904
H	−1.742166	2.893663	−0.525457
H	−4.002888	2.013538	−1.051789
H	−1.955197	4.674963	1.169494
H	−0.328670	3.976278	1.239262
H	−1.535392	3.529772	2.457834
H	−4.549645	0.404140	−3.182914
H	−4.738093	−1.361479	−3.393786
H	−6.152434	−0.351121	−2.996890
H	−3.533537	−4.808271	−2.738309
H	−1.957628	−3.987178	−2.836874
H	−2.527796	−4.648742	−1.278398
H	−8.732342	2.484361	2.057682
H	−7.038835	2.290981	2.529992
H	−7.521893	3.636317	1.476686
H	−8.143252	2.008580	−0.320386
H	−7.645334	0.661527	0.723944
H	−0.943558	−3.533201	1.801697
H	0.275058	−3.584902	3.112865
H	−1.390619	−3.053688	3.458315
H	3.608582	−0.482308	3.797430
H	1.885536	−0.234923	4.212327
H	2.867132	−1.404052	5.131852
H	5.920523	−2.738272	2.013079
H	4.313423	−3.273279	2.572393
H	4.762590	−3.427785	0.848563

ωB97X Energy = −2096.10505931 a.u.

(*aS*,1*S*,3*S*,1'*S*,3'*S*)-**22**, Conf AD

C	−0.734916	−1.704010	−1.312756
C	−2.056642	−1.978043	−1.678883
C	−3.091525	−1.593343	−0.831835
C	−2.817408	−0.994038	0.396246
C	−1.498491	−0.775251	0.780985
C	−0.450631	−1.127272	−0.077580
O	−3.548730	−0.405961	2.639275
C	−2.437993	0.482263	2.751942
C	−1.208411	−0.160292	2.130006
C	−3.974358	−0.615087	1.296878
C	−2.250585	0.789031	4.220692
C	−4.779025	0.575499	0.791788
C	0.971979	−0.894596	0.314239
C	1.651228	0.250483	−0.093888
C	2.981531	0.418304	0.293405
C	3.609855	−0.513171	1.109381
C	2.908542	−1.639002	1.557985
C	1.601322	−1.828239	1.131606
C	−6.111741	0.689664	1.180551
C	−6.884249	1.772254	0.790667
C	−6.296358	2.743916	0.004436
C	−4.981049	2.670816	−0.403231
C	−4.227442	1.574397	−0.004276
C	0.973425	1.318248	−0.915803
C	1.697185	2.647224	−0.793789
O	3.083567	2.443501	−1.074736
C	3.744283	1.667763	−0.093093
C	1.188924	3.688207	−1.763881
F	−7.041732	3.804260	−0.384737

O	4.888569	−0.235591	1.522051
C	5.888810	−1.208989	1.199043
O	3.560852	−2.468069	2.413541
C	2.859222	−3.599691	2.907346
C	5.097120	1.370129	−0.738184
O	5.293089	0.479902	−1.527327
O	5.998597	2.282163	−0.388860
C	8.212939	1.230456	−0.400866
C	7.287759	2.237464	−1.044759
O	0.313482	−2.064824	−2.111698
C	0.303480	−1.589406	−3.460014
O	−2.348794	−2.619491	−2.854841
C	−2.075720	−4.022248	−2.823565
C	−4.930898	−1.199660	−2.276890
O	−4.394571	−1.882081	−1.139881
H	−2.671987	1.411706	2.215933
H	−0.414300	0.586096	2.040863
H	−0.831035	−0.936141	2.806078
H	−4.654759	−1.465985	1.356429
H	−1.395922	1.453283	4.361804
H	−3.138286	1.274070	4.628864
H	−2.068443	−0.133211	4.777322
H	1.043208	−2.698026	1.454103
H	−6.556587	−0.084258	1.797520
H	−7.923157	1.865761	1.082122
H	−4.560943	3.452551	−1.024034
H	−3.196156	1.498197	−0.332407
H	0.946703	1.026972	−1.972443
H	−0.064436	1.436230	−0.592724
H	1.604119	3.019525	0.236577
H	3.919383	2.275990	0.803387
H	1.720311	4.631461	−1.632532
H	0.124162	3.863878	−1.600911
H	1.330609	3.345693	−2.791365
H	6.837155	−0.779754	1.517011
H	5.709349	−2.144642	1.725607
H	5.909078	−1.381451	0.119708
H	3.548383	−4.111227	3.574614
H	1.968517	−3.298653	3.465535
H	2.572347	−4.273510	2.095769
H	9.200887	1.308431	−0.858016
H	8.314556	1.426257	0.668045
H	7.848141	0.213357	−0.543905
H	7.131860	2.020325	−2.101752
H	7.671214	3.250718	−0.941079
H	1.343378	−1.558935	−3.780821
H	−0.266579	−2.252827	−4.111142
H	−0.122454	−0.584215	−3.511978
H	−2.649956	−4.504378	−2.028123
H	−2.382746	−4.421230	−3.788238
H	−1.009180	−4.207755	−2.670926
H	−4.888984	−0.117671	−2.123788
H	−4.391142	−1.471806	−3.184294
H	−5.970162	−1.512227	−2.357801

ωB97X Energy = −2096.10504969 a.u.

(*aS*,1*S*,3*S*,1'*S*,3'*S*)-**22**, Conf AE

C	−0.713056	0.213223	2.094939
C	−2.026178	0.052048	2.549983
C	−3.075912	0.446517	1.725649
C	−2.818777	0.986960	0.464066
C	−1.511559	1.138477	0.017759
C	−0.447149	0.751692	0.842237
O	−3.603116	2.221749	−1.480756
C	−2.497012	1.710438	−2.223559
C	−1.253172	1.725048	−1.350114
C	−3.998037	1.384941	−0.399504
C	−2.351196	2.564495	−3.462521
C	−4.810929	0.197165	−0.899556
C	0.971845	0.908641	0.403471
C	1.692831	−0.185227	−0.070307
C	3.018049	0.004585	−0.462400
C	3.598904	1.262294	−0.414155
C	2.877554	2.358754	0.067698
C	1.562506	2.168323	0.470418
C	−4.258702	−1.065046	−1.091801
C	−5.020405	−2.111610	−1.595912
C	−6.344241	−1.871281	−1.896307
C	−6.933157	−0.634869	−1.716739
C	−6.152466	0.395732	−1.217379
C	1.068975	−1.553599	−0.186667
C	1.863113	−2.445874	−1.125277
O	3.242379	−2.408963	−0.748769
C	3.843781	−1.153239	−0.985766
C	1.423369	−3.890668	−1.081058
F	−7.098120	−2.886397	−2.380485
O	4.914267	1.391134	−0.787001
C	5.116310	2.066724	−2.030688
O	3.536667	3.543868	0.110071
C	2.839190	4.671085	0.620261
C	5.202061	−1.251768	−0.290589
O	5.351081	−1.198110	0.903992
O	6.177121	−1.470657	−1.167201
C	7.748143	−3.058595	−0.178912
C	7.514349	−1.638409	−0.641632
O	0.325916	−0.183782	2.899176
C	0.676376	0.770463	3.903116
O	−2.287538	−0.401449	3.815245
C	−1.961078	−1.774934	4.051996
C	−4.905434	−0.879526	2.503840
O	−4.381531	0.394582	2.127220
H	−2.717952	0.676640	−2.521288
H	−0.449779	1.174575	−1.847296
H	−0.904036	2.758485	−1.243682
H	−4.665374	2.008338	0.197814
H	−1.503607	2.222685	−4.059641
H	−3.252115	2.508959	−4.075081
H	−2.180918	3.607324	−3.185092
H	0.978711	2.999162	0.846630
H	−3.219475	−1.246837	−0.839269
H	−4.598616	−3.097334	−1.748648

H	−7.979075	−0.491052	−1.958250
H	−6.598248	1.373629	−1.067696
H	1.015646	−2.025786	0.800878
H	0.042402	−1.465683	−0.553416
H	1.776700	−2.061138	−2.151691
H	4.017582	−1.022271	−2.061764
H	2.003445	−4.492569	−1.781519
H	0.367403	−3.970190	−1.345324
H	1.559620	−4.295093	−0.075613
H	4.601224	1.541100	−2.840185
H	4.760161	3.096595	−1.972136
H	6.188365	2.057937	−2.217011
H	3.540495	5.500680	0.578593
H	1.962793	4.904480	0.009560
H	2.531033	4.507441	1.656425
H	8.783697	−3.167062	0.148265
H	7.096094	−3.311823	0.657592
H	7.570776	−3.763612	−0.992478
H	8.163843	−1.378439	−1.475390
H	7.663245	−0.920837	0.165439
H	−0.172878	0.969454	4.561364
H	1.491374	0.334139	4.477092
H	1.015120	1.702399	3.440729
H	−2.536679	−2.423477	3.385059
H	−0.894734	−1.951672	3.909504
H	−2.236439	−1.981221	5.084366
H	−5.989280	−0.788460	2.456662
H	−4.579706	−1.651332	1.800717
H	−4.603072	−1.145298	3.516786

ωB97X Energy = −2096.10504262 a.u.

(*aS*,1*S*,3*S*,1’*S*,3’*S*)-**22**, Conf AF

C	0.568305	1.885897	−0.854526
C	1.864634	2.308451	−1.154032
C	2.929588	1.817013	−0.405794
C	2.712162	0.886450	0.608391
C	1.417485	0.469279	0.902933
C	0.335476	0.985384	0.179362
O	3.503090	−0.232490	2.610869
C	2.469070	−1.205767	2.463061
C	1.186369	−0.518734	2.022033
C	3.901633	0.346163	1.373784
C	2.321201	−1.915756	3.789503
C	4.760075	−0.628987	0.575346
C	−1.066143	0.577380	0.496035
C	−1.705059	−0.417512	−0.239875
C	−3.010544	−0.772546	0.103008
C	−3.650089	−0.167474	1.173779
C	−3.014587	0.842810	1.901891
C	−1.718697	1.201412	1.556674
C	4.322628	−1.250265	−0.588144
C	5.128020	−2.161808	−1.260003
C	6.375767	−2.436554	−0.743866
C	6.849609	−1.841047	0.409314
C	6.029100	−0.936110	1.062657
C	−1.013518	−1.119580	−1.381564
C	−1.704393	−2.426204	−1.732133
O	−3.106734	−2.187323	−1.881988
C	−3.738332	−1.861711	−0.660881
C	−1.207493	−3.026329	−3.026664
F	7.170767	−3.320376	−1.392614
O	−4.943472	−0.529693	1.456865
C	−5.095184	−1.316942	2.640104
O	−3.732599	1.405421	2.906400
C	−3.116045	2.439687	3.659776
C	−5.172246	−1.504944	−1.054186
O	−6.090378	−2.282549	−0.962660
O	−5.257720	−0.290713	−1.580878
C	−7.381557	0.691163	−0.884659
C	−6.558422	0.151266	−2.032008
O	−0.475989	2.321727	−1.627063
C	−0.914578	3.645378	−1.320395
O	2.066881	3.212088	−2.163447
C	2.528264	2.614755	−3.377685
C	4.527254	3.553063	−0.389673
O	4.217505	2.189067	−0.693159
H	2.775511	−1.928988	1.695541
H	0.457217	−1.272159	1.711412
H	0.746967	0.003232	2.879723
H	4.539155	1.186059	1.657624
H	1.523768	−2.659112	3.733361
H	3.248894	−2.421856	4.060054
H	2.071333	−1.198558	4.574807
H	−1.197706	1.974472	2.107654
H	3.340696	−1.022874	−0.988546
H	4.795989	−2.649978	−2.167950
H	7.839181	−2.081591	0.777978
H	6.382981	−0.458098	1.970187
H	−1.002730	−0.470236	−2.264577
H	0.030059	−1.319235	−1.122238
H	−1.565719	−3.144178	−0.910940
H	−3.813845	−2.759797	−0.033331
H	−1.709976	−3.972121	−3.232454
H	−0.133191	−3.209286	−2.966123
H	−1.395898	−2.340330	−3.855470
H	−6.155783	−1.544572	2.726918
H	−4.528209	−2.248755	2.555876
H	−4.765195	−0.760602	3.519180
H	−2.219980	2.076127	4.169891
H	−2.855544	3.289590	3.023066
H	−3.850719	2.752404	4.397422
H	−8.329938	1.070914	−1.269252
H	−7.591310	−0.088210	−0.151654
H	−6.859301	1.509503	−0.386674
H	−6.337913	0.925227	−2.764750
H	−7.057222	−0.680792	−2.529278
H	−0.102061	4.363074	−1.453860
H	−1.722273	3.875132	−2.012530
H	−1.290426	3.694184	−0.293660
H	1.796523	1.891353	−3.747378
H	2.640106	3.420545	−4.100328

H	3.491602	2.122614	−3.222994
H	3.922839	4.232217	−0.992590
H	4.360718	3.751434	0.672993
H	5.581315	3.688670	−0.622443

ωB97X Energy = −2096.10498380 a.u.

(*aS*,1*S*,3*S*,1'*S*,3'*S*)-**22**, Conf AG

C	0.553026	−1.477237	−1.407989
C	1.849427	−1.925464	−1.683980
C	2.912239	−1.037786	−1.542333
C	2.686403	0.273933	−1.120265
C	1.396576	0.707814	−0.838838
C	0.318366	−0.173376	−0.990521
O	3.480177	2.563233	−0.881547
C	2.455036	2.792542	0.084297
C	1.169300	2.128808	−0.380058
C	3.877546	1.199808	−0.978557
C	2.312326	4.288847	0.247012
C	4.809086	0.821888	0.165700
C	−1.082616	0.270869	−0.724536
C	−1.715113	−0.041043	0.476554
C	−3.021033	0.405320	0.683200
C	−3.666546	1.172726	−0.273888
C	−3.036539	1.474262	−1.485071
C	−1.741538	1.021032	−1.695691
C	6.152802	1.176891	0.073589
C	7.042676	0.897864	1.098843
C	6.560801	0.259021	2.224468
C	5.238950	−0.109383	2.358133
C	4.366654	0.175164	1.315147
C	−1.013775	−0.826424	1.556034
C	−1.694915	−0.648861	2.901918
O	−3.096315	−0.890816	2.750183
C	−3.745415	0.099828	1.979457
C	−1.183163	−1.606000	3.952946
F	7.421143	−0.021478	3.231584
O	−4.959653	1.566062	−0.034298
C	−5.118791	2.962082	0.229753
O	−3.758434	2.197585	−2.377764
C	−3.148579	2.507827	−3.622522
C	−5.166512	−0.434573	1.797346
O	−6.089380	−0.091703	2.494977
O	−5.234978	−1.367982	0.857217
C	−7.384010	−1.131180	−0.277469
C	−6.522302	−1.984692	0.625146
O	−0.499282	−2.348800	−1.539846
C	−0.951764	−2.508894	−2.885505
O	2.078275	−3.184277	−2.171174
C	1.819840	−4.252186	−1.253795
C	4.789262	−2.462785	−1.161462
O	4.194570	−1.377690	−1.874887
H	2.765378	2.352887	1.041548
H	0.434748	2.146867	0.429659
H	0.742608	2.714708	−1.202110
H	4.458727	1.151267	−1.900931
H	1.518923	4.517116	0.961200
H	3.242737	4.726604	0.611202
H	2.060119	4.750195	−0.710557
H	−1.226341	1.243995	−2.621618
H	6.513710	1.675968	−0.819774
H	8.090800	1.161998	1.030852
H	4.903692	−0.612887	3.256483
H	3.327921	−0.125179	1.403277
H	−1.002120	−1.891224	1.296308
H	0.029318	−0.506908	1.632627
H	−1.561169	0.387379	3.244346
H	−3.843953	1.021521	2.568149
H	−1.684365	−1.436800	4.906674
H	−0.109995	−1.467272	4.095345
H	−1.360869	−2.637705	3.641470
H	−6.174786	3.118809	0.440566
H	−4.525363	3.256933	1.100289
H	−4.823790	3.554576	−0.638000
H	−3.884218	3.078755	−4.183441
H	−2.248712	3.112690	−3.482003
H	−2.895667	1.598318	−4.174180
H	−8.326376	−1.647286	−0.469945
H	−7.604640	−0.169788	0.186926
H	−6.886577	−0.954720	−1.232232
H	−6.282024	−2.939721	0.161803
H	−7.001963	−2.163798	1.587507
H	−0.143947	−2.872334	−3.525377
H	−1.755882	−3.241549	−2.859471
H	−1.335425	−1.560620	−3.273855
H	2.416430	−4.127182	−0.345367
H	0.761519	−4.296919	−0.995439
H	2.117801	−5.168630	−1.759352
H	5.861770	−2.387406	−1.332118
H	4.589507	−2.371905	−0.090034
H	4.426105	−3.422189	−1.529946

ωB97X Energy = −2096.10490117 a.u.

(*aS*,1*S*,3*S*,1'*S*,3'*S*)-**22**, Conf AH

C	−0.512989	0.981694	1.780523
C	−1.786349	1.080752	2.342866
C	−2.897391	1.122541	1.507419
C	−2.746330	1.044459	0.124642
C	−1.473383	0.949677	−0.431175
C	−0.346753	0.932233	0.400655
O	−3.658743	1.418356	−2.091253
C	−2.626185	0.601075	−2.643020
C	−1.314721	0.894980	−1.932399
C	−3.981738	1.069472	−0.750460
C	−2.561508	0.888501	−4.125726
C	−4.800327	−0.217004	−0.715925
C	1.031818	0.851004	−0.168865
C	1.700168	−0.367870	−0.252766
C	2.991372	−0.391390	−0.780376
C	3.588157	0.769389	−1.254005
C	2.900653	1.986508	−1.202653

C	1.631833	2.015330	−0.639574
C	−4.323908	−1.412849	−0.193191
C	−5.098431	−2.566711	−0.223473
C	−6.354146	−2.497528	−0.786300
C	−6.866240	−1.328616	−1.315956
C	−6.076818	−0.191216	−1.274789
C	1.057473	−1.651105	0.211172
C	1.753061	−2.864338	−0.380477
O	3.160919	−2.746623	−0.157685
C	3.747547	−1.699534	−0.904209
C	1.303227	−4.165745	0.241211
F	−7.118252	−3.615258	−0.818434
O	4.828359	0.650339	−1.823498
C	5.876782	1.462259	−1.283550
O	3.530516	3.065907	−1.734072
C	2.856459	4.315218	−1.690524
C	5.190637	−1.648001	−0.404045
O	6.106314	−2.187057	−0.974899
O	5.296472	−1.009201	0.756146
C	6.855802	−2.257148	2.160435
C	6.597564	−0.983626	1.387877
O	0.576391	0.876295	2.605488
C	1.010336	2.119710	3.157497
O	−1.931807	1.151427	3.703259
C	−2.174650	−0.119722	4.309763
C	−4.504286	2.433982	2.627224
O	−4.161728	1.177539	2.034859
H	−2.892454	−0.453107	−2.487925
H	−0.574989	0.136759	−2.203800
H	−0.921291	1.853810	−2.288983
H	−4.630553	1.877702	−0.406179
H	−1.763222	0.305494	−4.588880
H	−3.504900	0.631509	−4.609395
H	−2.358656	1.948510	−4.295461
H	1.082390	2.946331	−0.579578
H	−3.335500	−1.456251	0.251081
H	−4.735564	−3.502819	0.182697
H	−7.860213	−1.316814	−1.745920
H	−6.460291	0.735875	−1.687818
H	1.094997	−1.713446	1.304807
H	0.001093	−1.664296	−0.071512
H	1.579522	−2.890885	−1.465878
H	3.807652	−1.986301	−1.962095
H	1.816176	−5.011193	−0.218727
H	0.228332	−4.295082	0.102711
H	1.516626	−4.165825	1.312443
H	5.840165	2.469511	−1.695353
H	5.801010	1.508622	−0.194051
H	6.810467	0.975747	−1.562982
H	2.654916	4.619156	−0.659828
H	3.527283	5.033616	−2.154976
H	1.918627	4.277457	−2.251337
H	7.815306	−2.179233	2.674578
H	6.077855	−2.418886	2.908015
H	6.892602	−3.119348	1.493829
H	7.355705	−0.816639	0.622106
H	6.557471	−0.117928	2.046794
H	1.321467	2.802714	2.360980
H	0.215452	2.577456	3.750611
H	1.862271	1.897105	3.796921
H	−3.092483	−0.563476	3.915193
H	−1.331507	−0.793269	4.134412
H	−2.284527	0.057496	5.377729
H	−5.524858	2.335108	2.991113
H	−3.836384	2.667126	3.457707
H	−4.459682	3.230874	1.879142

ωB97X Energy = −2096.10489948 a.u.

(*aS*,1*S*,3*S*,1’*S*,3’*S*)-**22**, Conf AI

C	−0.504262	−0.383919	1.964032
C	−1.775333	−0.682214	2.466532
C	−2.886447	−0.075296	1.888023
C	−2.730931	0.810129	0.819605
C	−1.464861	1.096880	0.323313
C	−0.339175	0.498665	0.903863
O	−3.654694	2.563329	−0.589667
C	−2.630073	2.307741	−1.549632
C	−1.314123	2.061279	−0.829961
C	−3.971671	1.432592	0.214051
C	−2.573163	3.499529	−2.478120
C	−4.833432	0.442625	−0.560501
C	1.038637	0.795378	0.409759
C	1.698938	−0.093676	−0.434907
C	2.993453	0.213492	−0.854585
C	3.601664	1.402786	−0.475437
C	2.920348	2.313983	0.338702
C	1.648381	1.987503	0.789586
C	−6.183055	0.737718	−0.740501
C	−7.010223	−0.097725	−1.474142
C	−6.460553	−1.237313	−2.028510
C	−5.131025	−1.567595	−1.875580
C	−4.321580	−0.716953	−1.133164
C	1.042012	−1.368357	−0.902432
C	1.729352	−1.925853	−2.136069
O	3.137082	−1.992499	−1.893109
C	3.738146	−0.717124	−1.791649
C	1.266933	−3.319188	−2.493019
F	−7.259855	−2.062520	−2.744980
O	4.845622	1.664796	−0.987355
C	5.895547	1.912571	−0.045860
O	3.558805	3.479625	0.617726
C	2.891730	4.420270	1.446779
C	5.178174	−1.019425	−1.379791
O	6.091603	−1.074827	−2.165565
O	5.282372	−1.282848	−0.081819
C	6.799367	−3.174566	0.192962
C	6.576674	−1.695967	0.415226
O	0.594987	−0.985522	2.524736
C	1.039897	−0.363151	3.731401
O	−1.934179	−1.486062	3.563476
C	−1.610110	−2.865771	3.363609

C	−4.666872	−1.583751	2.403499
O	−4.152167	−0.251886	2.374290
H	−2.901058	1.412825	−2.125787
H	−0.574270	1.682447	−1.540485
H	−0.927560	3.016425	−0.456503
H	−4.583923	1.835454	1.022378
H	−1.790234	3.359634	−3.225977
H	−3.525718	3.630013	−2.993421
H	−2.353772	4.408638	−1.913372
H	1.103840	2.670070	1.429934
H	−6.597174	1.637050	−0.296420
H	−8.061782	0.120566	−1.613947
H	−4.740656	−2.472345	−2.325002
H	−3.276023	−0.972698	−0.998113
H	1.071695	−2.118638	−0.104005
H	−0.012537	−1.184193	−1.126037
H	1.560105	−1.247726	−2.984818
H	3.801406	−0.256458	−2.785946
H	1.768242	−3.673564	−3.394383
H	0.189930	−3.323006	−2.670042
H	1.485770	−4.009683	−1.675477
H	5.849345	1.190527	0.773623
H	6.828502	1.782349	−0.592260
H	5.831766	2.923568	0.353215
H	3.569641	5.264316	1.546267
H	1.957151	4.756282	0.989570
H	2.686697	3.999598	2.434830
H	7.748238	−3.469370	0.644357
H	6.002696	−3.757330	0.657600
H	6.837604	−3.410199	−0.871052
H	7.346630	−1.094664	−0.069142
H	6.545087	−1.451972	1.475784
H	1.339409	0.671281	3.538006
H	0.254778	−0.386083	4.491017
H	1.901313	−0.931343	4.076718
H	−2.225886	−3.290240	2.565187
H	−0.554557	−2.987053	3.119066
H	−1.833948	−3.371172	4.301012
H	−4.258544	−2.145390	3.243608
H	−5.745494	−1.489540	2.515904
H	−4.447638	−2.100328	1.464737

ωB97X Energy = −2096.10487362 a.u.

(*aS*,1*S*,3*S*,1'*S*,3'*S*)-**22**, Conf AJ

C	0.589446	2.034123	−0.791368
C	1.903092	2.437898	−1.051314
C	2.947185	1.861247	−0.337669
C	2.685732	0.925296	0.663813
C	1.375352	0.555116	0.944764
C	0.318011	1.103314	0.205685
O	3.428128	−0.249445	2.660420
C	2.349258	−1.169518	2.502439
C	1.100939	−0.419012	2.066571
C	3.851421	0.348243	1.440139
C	2.166594	−1.881103	3.823971
C	4.705227	−0.614922	0.625964
C	−1.098001	0.717406	0.487394
C	−1.661668	−0.419039	−0.090073
C	−2.986583	−0.738745	0.215257
C	−3.719637	0.042031	1.094237
C	−3.157655	1.189698	1.662335
C	−1.843314	1.511557	1.355599
C	4.190906	−1.375136	−0.419574
C	4.989308	−2.276119	−1.112087
C	6.312116	−2.395402	−0.741875
C	6.864307	−1.657436	0.286747
C	6.046910	−0.768762	0.967291
C	−0.873089	−1.324297	−1.005563
C	−1.508779	−2.700365	−1.103946
O	−2.894381	−2.548852	−1.421441
C	−3.639192	−1.977715	−0.365286
C	−0.890744	−3.562194	−2.180012
F	7.101855	−3.264114	−1.415525
O	−5.027326	−0.296877	1.337555
C	−5.273742	−0.824381	2.643283
O	−3.961763	1.912952	2.481139
C	−3.419943	3.084193	3.075011
C	−5.025882	−1.739924	−0.964427
O	−5.940731	−2.514214	−0.825006
O	−5.067421	−0.645464	−1.712330
C	−7.271711	0.365586	−1.422820
C	−6.318111	−0.331086	−2.366715
O	−0.447051	2.623286	−1.466951
C	−0.548050	2.266170	−2.846872
O	2.165644	3.387981	−2.003792
C	1.935040	4.725517	−1.554111
C	4.809773	1.956926	−1.804639
O	4.241000	2.267492	−0.528970
H	2.619696	−1.902976	1.731236
H	0.333312	−1.137646	1.767745
H	0.694354	0.129299	2.924295
H	4.496296	1.171912	1.750415
H	1.339329	−2.590542	3.760629
H	3.071560	−2.426866	4.094325
H	1.944427	−1.158866	4.612927
H	−1.379477	2.391425	1.782825
H	3.153685	−1.258279	−0.714863
H	4.597680	−2.870616	−1.928236
H	7.910541	−1.777737	0.539477
H	6.463797	−0.178868	1.777055
H	−0.812866	−0.893389	−2.011405
H	0.154381	−1.425495	−0.646215
H	−1.431680	−3.206370	−0.130550
H	−3.779847	−2.721348	0.430462
H	−1.364678	−4.543983	−2.206595
H	0.175382	−3.695848	−1.988282
H	−1.010718	−3.088451	−3.156822
H	−6.332605	−1.072349	2.682178
H	−4.681333	−1.728979	2.808945
H	−5.040525	−0.082809	3.409062
H	−4.215764	3.507404	3.682743

H	−2.564371	2.844079	3.711899
H	−3.118501	3.809030	2.314285
H	−8.174184	0.649426	−1.967338
H	−7.556738	−0.288709	−0.598640
H	−6.817551	1.269046	−1.013323
H	−6.030397	0.316293	−3.192969
H	−6.745643	−1.251345	−2.765225
H	0.355271	2.548426	−3.390267
H	−0.723467	1.191559	−2.950906
H	−1.402861	2.809137	−3.244646
H	0.883867	4.868062	−1.292939
H	2.567217	4.951680	−0.691287
H	2.199290	5.382173	−2.380604
H	5.833221	2.325908	−1.778883
H	4.818481	0.874774	−1.962167
H	4.258826	2.449005	−2.606510

ωB97X Energy = −2096.10483296 a.u.

(*aS*,1*S*,3*S*,1'*S*,3'*S*)-**22**, Conf AK

C	−0.534457	−0.565567	1.949114
C	−1.816422	−0.869489	2.408930
C	−2.912999	−0.239096	1.830295
C	−2.738689	0.666886	0.786126
C	−1.457168	0.970589	0.334933
C	−0.345139	0.363112	0.930894
O	−3.620087	2.481096	−0.563911
C	−2.574606	2.266650	−1.512274
C	−1.275816	1.978106	−0.776400
C	−3.961461	1.307336	0.164479
C	−2.491669	3.501003	−2.381104
C	−4.785734	0.359355	−0.698635
C	1.044434	0.694149	0.494033
C	1.745674	−0.147399	−0.365983
C	3.042975	0.205853	−0.740353
C	3.613418	1.385702	−0.288663
C	2.912281	2.226497	0.580800
C	1.625689	1.870734	0.961335
C	−4.275347	−0.817771	−1.233488
C	−5.052017	−1.627522	−2.053029
C	−6.345982	−1.237140	−2.322766
C	−6.892883	−0.077080	−1.809325
C	−6.099429	0.715895	−0.996341
C	1.130793	−1.413551	−0.906686
C	1.869292	−1.905825	−2.139360
O	3.270575	−1.953836	−1.854878
C	3.841043	−0.672677	−1.683622
C	1.448345	−3.292751	−2.565710
F	−7.113029	−2.021944	−3.116222
O	4.904457	1.673758	−0.656369
C	5.029472	2.731313	−1.610215
O	3.562829	3.342197	0.996436
C	2.879873	4.212793	1.886807
C	5.271516	−0.952953	−1.222779
O	6.218648	−0.934852	−1.969986
O	5.325828	−1.284542	0.060511
C	6.955780	−3.086996	0.304924
C	6.617068	−1.643940	0.602799
O	0.540191	−1.232329	2.476954
C	0.992475	−0.708127	3.725620
O	−1.977572	−1.763219	3.434247
C	−2.324361	−3.077570	2.992769
C	−4.520452	−0.075715	3.548682
O	−4.187219	−0.540200	2.236806
H	−2.837667	1.401483	−2.135598
H	−0.525287	1.621031	−1.487127
H	−0.887587	2.913222	−0.356945
H	−4.609460	1.663230	0.968074
H	−1.692314	3.393172	−3.116753
H	−3.431718	3.660260	−2.911046
H	−2.281993	4.381011	−1.768551
H	1.056553	2.503324	1.631072
H	−3.257631	−1.119161	−1.010031
H	−4.662835	−2.546118	−2.474523
H	−7.917147	0.188654	−2.040060
H	−6.511453	1.630700	−0.582974
H	1.151167	−2.194711	−0.138395
H	0.080294	−1.241473	−1.157342
H	1.711849	−1.197416	−2.965408
H	3.931942	−0.174797	−2.658143
H	1.982449	−3.599189	−3.465818
H	0.377032	−3.312000	−2.773466
H	1.659391	−4.011202	−1.770567
H	6.091295	2.830040	−1.826354
H	4.491448	2.483456	−2.529986
H	4.648450	3.667729	−1.199103
H	3.569006	5.026893	2.096936
H	1.971654	4.613848	1.428852
H	2.625573	3.702489	2.819727
H	7.897737	−3.344734	0.792309
H	6.179534	−3.751864	0.686741
H	7.068167	−3.251485	−0.767060
H	7.366318	−0.961475	0.200928
H	6.513372	−1.471543	1.672355
H	1.334202	0.324381	3.604017
H	0.197632	−0.751479	4.473769
H	1.827007	−1.330651	4.042226
H	−3.275110	−3.063678	2.453894
H	−1.538445	−3.482878	2.349791
H	−2.418399	−3.692701	3.885418
H	−5.565444	−0.331872	3.710014
H	−3.898075	−0.560051	4.302486
H	−4.398404	1.009645	3.609311

ωB97X Energy = −2096.10480941 a.u.

(*aS*,1*S*,3*S*,1'*S*,3'*S*)-**22**, Conf AL

C	−0.739598	−1.561477	−1.464663
C	−2.060660	−1.756706	−1.880351
C	−3.095833	−1.415798	−1.012822
C	−2.819180	−0.964273	0.277663
C	−1.502592	−0.819030	0.700317

C	−0.454118	−1.111980	−0.180653
O	−3.570769	−0.569575	2.555775
C	−2.434211	0.265480	2.774177
C	−1.215203	−0.351814	2.107930
C	−3.978769	−0.629110	1.193251
C	−2.264538	0.416676	4.268908
C	−4.726004	0.637175	0.795997
C	0.970421	−0.946401	0.237739
C	1.692625	0.186260	−0.130580
C	3.016386	0.304533	0.293387
C	3.594475	−0.669434	1.092671
C	2.873811	−1.812241	1.452104
C	1.560095	−1.936369	1.020185
C	−6.063632	0.763267	1.163512
C	−6.783467	1.911434	0.872985
C	−6.137339	2.936296	0.209647
C	−4.815176	2.853713	−0.172692
C	−4.115212	1.691418	0.124428
C	1.070080	1.286475	−0.952375
C	1.864260	2.577112	−0.846093
O	3.244822	2.298074	−1.095380
C	3.840393	1.521667	−0.077036
C	1.426425	3.621107	−1.846734
F	−6.831084	4.061771	−0.081001
O	4.906472	−0.516244	1.468440
C	5.091787	−0.176093	2.844485
O	3.530994	−2.728975	2.206137
C	2.827681	−3.906544	2.575008
C	5.207158	1.138711	−0.645569
O	5.369021	0.311796	−1.506890
O	6.174717	1.880556	−0.115538
C	7.777183	2.428450	−1.875222
C	7.519213	1.658162	−0.600061
O	0.283934	−1.779856	−2.349549
C	0.682887	−3.143278	−2.469189
O	−2.287640	−2.222534	−3.146903
C	−2.957262	−3.484250	−3.224379
C	−4.858906	−0.747812	−2.455130
O	−4.410388	−1.569240	−1.372524
H	−2.628714	1.251124	2.331352
H	−0.397157	0.373584	2.102169
H	−0.873592	−1.202599	2.708449
H	−4.692527	−1.453693	1.158296
H	−1.389376	1.030912	4.489322
H	−3.141938	0.891609	4.709906
H	−2.125917	−0.562349	4.733499
H	0.974997	−2.807287	1.288073
H	−6.554424	−0.053328	1.682907
H	−7.825896	2.015535	1.147733
H	−4.348077	3.679604	−0.694805
H	−3.078639	1.608859	−0.185075
H	1.015905	0.981352	−2.003497
H	0.043409	1.464776	−0.620260
H	1.773745	2.977819	0.173832
H	4.001572	2.140587	0.815234
H	1.999581	4.540743	−1.723559
H	0.368172	3.849928	−1.708918
H	1.570754	3.250209	−2.863888
H	4.730784	−0.976443	3.492804
H	6.161645	−0.040118	2.989299
H	4.570821	0.756301	3.081487
H	3.524318	−4.502188	3.159524
H	1.951875	−3.669187	3.185092
H	2.516698	−4.472119	1.692429
H	8.816062	2.287709	−2.178610
H	7.133228	2.077816	−2.682327
H	7.606035	3.495077	−1.722517
H	8.157116	2.004413	0.210967
H	7.665912	0.587033	−0.739714
H	−0.137531	−3.760326	−2.844131
H	1.505526	−3.167121	−3.181104
H	1.024801	−3.530217	−1.504295
H	−2.408573	−4.243676	−2.659933
H	−3.977022	−3.417007	−2.841634
H	−2.973837	−3.756165	−4.277663
H	−4.346331	−1.003059	−3.384183
H	−5.925618	−0.935834	−2.557904
H	−4.696403	0.307815	−2.222958

ωB97X Energy = −2096.10477165 a.u.

(*aS*,1*S*,3*S*,1’*S*,3’*S*)-**22**, Conf AM

C	−0.748650	−1.629732	−1.463232
C	−2.076095	−1.820238	−1.860851
C	−3.104039	−1.474889	−0.988507
C	−2.819993	−1.003948	0.292113
C	−1.497583	−0.873301	0.704150
C	−0.455617	−1.180515	−0.178069
O	−3.551141	−0.592887	2.574395
C	−2.405893	0.234128	2.775552
C	−1.197314	−0.401405	2.107465
C	−3.970568	−0.661936	1.215503
C	−2.224073	0.400302	4.267310
C	−4.720609	0.602109	0.815987
C	0.970491	−1.022765	0.237791
C	1.676983	0.138045	−0.069445
C	3.001501	0.248319	0.354519
C	3.596863	−0.762051	1.092802
C	2.892380	−1.932445	1.390166
C	1.577711	−2.047966	0.958688
C	−4.118378	1.646421	0.121553
C	−4.820159	2.806970	−0.178264
C	−6.135576	2.897932	0.224842
C	−6.773396	1.882950	0.911060
C	−6.051813	0.736390	1.203631
C	1.038837	1.284348	−0.813748
C	1.806758	2.576951	−0.596449
O	3.189758	2.347845	−0.879945
C	3.811963	1.493207	0.057003
C	1.342514	3.698997	−1.495397
F	−6.831076	4.021803	−0.068052
O	4.909069	−0.610103	1.468885

C	5.100500	−0.370222	2.865363
O	3.565379	−2.882178	2.087412
C	2.873996	−4.080984	2.407781
C	5.161119	1.157831	−0.579649
O	5.301895	0.371695	−1.482066
O	6.137421	1.891807	−0.055099
C	7.644326	2.546892	−1.862252
C	7.462980	1.721026	−0.608877
O	0.294248	−1.950565	−2.285014
C	0.305977	−1.371299	−3.592470
O	−2.378996	−2.338932	−3.093235
C	−2.155239	−3.747384	−3.191637
C	−4.908460	−0.878680	−2.407752
O	−4.414500	−1.679370	−1.330085
H	−2.594506	1.216695	2.322923
H	−0.370682	0.314163	2.095271
H	−0.862660	−1.253593	2.710089
H	−4.685459	−1.485973	1.192518
H	−1.342063	1.009186	4.474858
H	−3.094217	0.887527	4.709268
H	−2.090643	−0.574818	4.741518
H	1.005259	−2.940582	1.177167
H	−3.087492	1.556800	−0.204351
H	−4.359654	3.625200	−0.718022
H	−7.810862	1.993302	1.201719
H	−6.536143	−0.072531	1.740801
H	1.006104	1.069977	−1.888408
H	0.004399	1.415949	−0.484663
H	1.715921	2.880442	0.456425
H	3.999376	2.039370	0.990579
H	1.900625	4.613602	−1.291937
H	0.281593	3.895746	−1.331132
H	1.486257	3.426478	−2.543282
H	6.168355	−0.220252	3.010959
H	4.560387	0.528995	3.176334
H	4.764198	−1.225876	3.453309
H	3.579152	−4.695481	2.961791
H	1.999941	−3.877717	3.032410
H	2.562592	−4.610001	1.503204
H	8.669412	2.439389	−2.221186
H	6.968594	2.215652	−2.651311
H	7.461300	3.603011	−1.658773
H	8.129824	2.050112	0.185764
H	7.626465	0.659968	−0.797953
H	−0.134799	−0.371544	−3.576188
H	1.351988	−1.301805	−3.886570
H	−0.239073	−1.991038	−4.305148
H	−1.101474	−3.986014	−3.024720
H	−2.773418	−4.283255	−2.466748
H	−2.440983	−4.037613	−4.200734
H	−4.802511	0.183511	−2.170346
H	−4.384734	−1.108632	−3.335889
H	−5.964425	−1.121433	−2.509147

ωB97X Energy = −2096.10474404 a.u.

(*aS*,1*S*,3*S*,1'*S*,3'*S*)-**22**, Conf AN

C	0.788124	−1.647219	1.414389
C	2.120930	−1.835477	1.796790
C	3.134560	−1.498803	0.901877
C	2.837906	−0.945819	−0.340484
C	1.510752	−0.769628	−0.719392
C	0.482070	−1.134737	0.154700
O	3.529996	−0.440422	−2.610508
C	2.406357	0.430168	−2.742640
C	1.193159	−0.207730	−2.085094
C	3.976000	−0.570171	−1.265517
C	2.200193	0.686250	−4.218224
C	4.745032	0.674073	−0.834313
C	−0.949280	−0.980901	−0.245230
C	−1.671991	0.158653	0.099230
C	−3.008283	0.250198	−0.291055
C	−3.602099	−0.753064	−1.045172
C	−2.860829	−1.876910	−1.427052
C	−1.544028	−1.986475	−1.001134
C	5.979779	0.918632	−1.432634
C	6.717959	2.048496	−1.121170
C	6.196897	2.933689	−0.197026
C	4.981071	2.729831	0.418650
C	4.257926	1.588966	0.091254
C	−1.038284	1.295595	0.861728
C	−1.830140	2.580774	0.695961
O	−3.203696	2.319570	0.994991
C	−3.826018	1.481554	0.042077
C	−1.368654	3.682943	1.620651
F	6.911064	4.040424	0.117788
O	−4.894588	−0.553192	−1.458691
C	−5.855890	−1.538254	−1.060451
O	−3.489218	−2.784920	−2.217603
C	−2.755171	−3.930601	−2.624918
C	−5.167713	1.128474	0.681256
O	−5.305035	0.307263	1.553951
O	−6.149982	1.885208	0.206667
C	−8.412876	2.628659	0.101213
C	−7.460645	1.678378	0.781250
O	−0.250970	−2.017028	2.219124
C	−0.290944	−1.481848	3.542633
O	2.393570	−2.422009	3.003800
C	3.096389	−1.602187	3.942764
C	4.866174	−3.051042	1.296144
O	4.453140	−1.682210	1.236737
H	2.635135	1.378118	−2.237152
H	0.389504	0.530292	−2.011635
H	0.818456	−1.011902	−2.728666
H	4.686608	−1.398691	−1.292328
H	1.332785	1.330875	−4.372201
H	3.075581	1.172828	−4.650503
H	2.028528	−0.256246	−4.743371
H	−0.952650	−2.851807	−1.272261
H	6.371013	0.211707	−2.156891
H	7.680259	2.243749	−1.578136
H	4.610566	3.448389	1.139324

H	3.301899	1.416907	0.573691
H	−0.979930	1.052354	1.929104
H	−0.012773	1.452594	0.516246
H	−1.762345	2.917259	−0.348640
H	−4.022996	2.046848	−0.877662
H	−1.943093	4.594650	1.452888
H	−0.313353	3.900334	1.446561
H	−1.492239	3.377130	2.661945
H	−6.830632	−1.124799	−1.314903
H	−5.698205	−2.475560	−1.591592
H	−5.800442	−1.703956	0.018282
H	−3.431819	−4.516613	−3.241824
H	−1.878512	−3.648298	−3.214249
H	−2.441140	−4.525678	−1.763282
H	−9.412710	2.496552	0.517205
H	−8.109324	3.664284	0.259649
H	−8.458438	2.434727	−0.971237
H	−7.743389	0.635507	0.625301
H	−7.399175	1.859951	1.855441
H	0.319817	−2.071940	4.225446
H	0.049580	−0.442225	3.550548
H	−1.333339	−1.517667	3.854844
H	4.099530	−1.366683	3.584921
H	2.546283	−0.674893	4.126274
H	3.156745	−2.176059	4.865313
H	5.932143	−3.041545	1.513587
H	4.331521	−3.587980	2.082120
H	4.694657	−3.541203	0.333641

ωB97X Energy = −2096.10473283 a.u.

(*aS*,1*S*,3*S*,1'*S*,3'*S*)-**22**, Conf AO

C	0.503436	−0.926891	1.751604
C	1.774393	−1.046501	2.322357
C	2.886978	−1.075925	1.485001
C	2.730302	−1.063431	0.098976
C	1.460132	−0.988526	−0.461822
C	0.336707	−0.912116	0.371411
O	3.656210	−1.490834	−2.108658
C	2.610895	−0.708905	−2.685631
C	1.303719	−0.995830	−1.964399
C	3.970025	−1.122769	−0.769860
C	2.549363	−1.051953	−4.156891
C	4.801436	0.152719	−0.738078
C	−1.041602	−0.814871	−0.196222
C	−1.711416	0.405592	−0.240377
C	−3.004856	0.443798	−0.761414
C	−3.601415	−0.701594	−1.271598
C	−2.911829	−1.918454	−1.262478
C	−1.642273	−1.964052	−0.702264
C	4.244433	1.409166	−0.522167
C	5.028336	2.555504	−0.553419
C	6.378506	2.418169	−0.796963
C	6.972601	1.189926	−1.010772
C	6.169715	0.060434	−0.981015
C	−1.068335	1.674092	0.261461
C	−1.774766	2.906485	−0.275267
O	−3.180627	2.772273	−0.049142
C	−3.765957	1.752870	−0.834021
C	−1.328511	4.183112	0.398427
F	7.152946	3.528229	−0.821543
O	−4.843886	−0.565297	−1.832217
C	−5.888105	−1.401028	−1.321281
O	−3.540950	−2.980958	−1.828289
C	−2.863313	−4.228955	−1.830462
C	−5.206936	1.676957	−0.331000
O	−6.126845	2.233584	−0.877723
O	−5.306058	0.994940	0.804869
C	−6.867061	2.180812	2.260788
C	−6.605117	0.939473	1.438733
O	−0.588993	−0.786250	2.567810
C	−1.105539	−2.012629	3.079132
O	1.880632	−1.075036	3.686296
C	2.431359	−2.275727	4.234234
C	4.608969	−0.072766	2.775274
O	4.158840	−1.178923	1.987194
H	2.860535	0.354250	−2.573544
H	0.552292	−0.261282	−2.266115
H	0.928242	−1.974484	−2.284425
H	4.603878	−1.933132	−0.406215
H	1.743937	−0.496097	−4.640669
H	3.489407	−0.801653	−4.650400
H	2.359433	−2.119858	−4.287414
H	−1.093227	−2.896732	−0.670990
H	3.183777	1.504708	−0.314956
H	4.603180	3.537488	−0.386717
H	8.038555	1.125897	−1.191226
H	6.619008	−0.913468	−1.145462
H	−1.091812	1.693580	1.356831
H	−0.015820	1.701742	−0.034502
H	−1.608527	2.980173	−1.359600
H	−3.830717	2.080308	−1.879733
H	−1.847267	5.044580	−0.023713
H	−0.254759	4.323300	0.261527
H	−1.537507	4.136671	1.469487
H	−6.824214	−0.904919	−1.574646
H	−5.852410	−2.389897	−1.775364
H	−5.806075	−1.493386	−0.235202
H	−3.530142	−4.930724	−2.325127
H	−1.923573	−4.166965	−2.385920
H	−2.664620	−4.572551	−0.811700
H	−7.824698	2.078262	2.774090
H	−6.087786	2.317182	3.012047
H	−6.909970	3.068319	1.628623
H	−7.364597	0.799189	0.668932
H	−6.559643	0.048857	2.063199
H	−1.396989	−2.678202	2.260767
H	−0.371188	−2.512172	3.716237
H	−1.983387	−1.760296	3.670791
H	2.318069	−2.197653	5.313479
H	1.881864	−3.149219	3.871949
H	3.486954	−2.382891	3.978692

H	4.044851	0.005993	3.706196
H	5.657934	−0.262508	2.993366
H	4.518267	0.858423	2.210227

ωB97X Energy = −2096.10472392 a.u.

(*aR*,1*S*,3*S*,1’*S*,3’*S*)-**22**, Conf A

C	−1.029441	2.050541	0.700135
C	−2.321383	2.448631	0.348339
C	−3.128127	1.580594	−0.378175
C	−2.640893	0.337089	−0.782213
C	−1.347079	−0.047111	−0.444926
C	−0.536692	0.810243	0.311194
O	−2.795356	−1.573754	−2.278539
C	−1.918638	−2.300321	−1.418112
C	−0.816352	−1.379526	−0.919455
C	−3.537699	−0.559947	−1.609998
C	−1.382313	−3.474258	−2.205643
C	−4.695704	−1.160535	−0.824506
C	0.854472	0.421818	0.692849
C	1.922660	0.655886	−0.169347
C	3.198317	0.239648	0.213997
C	3.414440	−0.354278	1.450257
C	2.346233	−0.544688	2.334410
C	1.069787	−0.176952	1.930718
C	−4.618512	−1.432410	0.537864
C	−5.678491	−2.025059	1.211700
C	−6.817258	−2.332666	0.497715
C	−6.937769	−2.075286	−0.853833
C	−5.864847	−1.488628	−1.506670
C	1.732031	1.364537	−1.488155
C	3.045023	1.919207	−2.012299
O	4.026831	0.879615	−1.992974
C	4.392416	0.496943	−0.682743
C	2.948491	2.423932	−3.433032
F	−7.859436	−2.903044	1.146509
O	4.708271	−0.655962	1.791361
C	4.986197	−2.026072	2.105597
O	2.643337	−1.065595	3.552509
C	1.581733	−1.227253	4.482070
C	5.245233	−0.754000	−0.885740
O	4.796014	−1.847225	−1.126905
O	6.541444	−0.473761	−0.830081
C	8.855545	−1.029631	−0.920207
C	7.455083	−1.571583	−1.053614
O	−0.213038	2.931739	1.360284
C	−0.504617	3.078476	2.750770
O	−2.784378	3.684246	0.719223
C	−2.473268	4.709457	−0.226744
C	−5.348128	2.144494	0.239859
O	−4.375955	1.962995	−0.794617
H	−2.494040	−2.673677	−0.560847
H	−0.267444	−1.873287	−0.113807
H	−0.097661	−1.213158	−1.730954
H	−3.966521	0.038924	−2.415076
H	−0.685089	−4.051359	−1.595314
H	−2.194758	−4.131476	−2.518523
H	−0.854745	−3.121968	−3.095007
H	0.223770	−0.336337	2.587239
H	−3.724883	−1.169666	1.093558
H	−5.627403	−2.239003	2.272125
H	−7.853601	−2.325978	−1.374769
H	−5.942303	−1.277527	−2.568217
H	1.317266	0.677135	−2.234852
H	1.013235	2.179516	−1.371718
H	3.387948	2.728056	−1.351396
H	5.024826	1.272530	−0.232382
H	3.904177	2.831916	−3.764152
H	2.193357	3.208991	−3.500797
H	2.662766	1.610486	−4.103629
H	4.657817	−2.673826	1.288878
H	6.067530	−2.095771	2.212478
H	4.501671	−2.319469	3.035493
H	2.033476	−1.630065	5.385067
H	1.107653	−0.268142	4.708295
H	0.831088	−1.928322	4.107734
H	9.573304	−1.836273	−1.076031
H	9.044749	−0.253357	−1.662760
H	9.017717	−0.612250	0.074446
H	7.246066	−2.348734	−0.315991
H	7.267905	−1.982173	−2.047125
H	−0.355981	2.128200	3.272679
H	0.194427	3.816768	3.138633
H	−1.528488	3.428290	2.897596
H	−1.390980	4.805708	−0.345314
H	−2.935378	4.491464	−1.193233
H	−2.880759	5.636642	0.171347
H	−5.039023	2.928805	0.931353
H	−6.271807	2.433006	−0.257810
H	−5.505014	1.206627	0.779746

ωB97X Energy = −2096.10658614 a.u.

(*aR*,1*S*,3*S*,1’*S*,3’*S*)-**22**, Conf B

C	−0.978231	2.038952	0.557678
C	−2.265903	2.451188	0.206763
C	−3.108409	1.564429	−0.453327
C	−2.661846	0.286255	−0.791186
C	−1.372008	−0.112740	−0.455954
C	−0.525090	0.765845	0.232624
O	−2.901807	−1.709091	−2.162005
C	−2.024085	−2.403813	−1.276895
C	−0.886461	−1.484620	−0.861158
C	−3.600025	−0.633132	−1.545586
C	−1.537706	−3.639071	−2.000432
C	−4.760370	−1.143968	−0.702427
C	0.864391	0.367084	0.610527
C	1.921528	0.544952	−0.278885
C	3.200192	0.144876	0.112641
C	3.420896	−0.391338	1.371348
C	2.358377	−0.576623	2.260909

C	1.083642	−0.193049	1.867007
C	−4.656156	−1.353537	0.669240
C	−5.721205	−1.865044	1.399006
C	−6.892698	−2.153904	0.731401
C	−7.040707	−1.955689	−0.627247
C	−5.961846	−1.450707	−1.336326
C	1.720299	1.180438	−1.632907
C	3.033411	1.677452	−2.213345
O	4.001417	0.626675	−2.140506
C	4.385492	0.332945	−0.814666
C	2.919924	2.086775	−3.663244
F	−7.939873	−2.644424	1.435049
O	4.691377	−0.794360	1.702332
C	5.363802	0.062647	2.628023
O	2.664542	−1.131184	3.460072
C	1.607917	−1.314982	4.391410
C	5.229077	−0.936885	−0.926413
O	4.776716	−2.026255	−1.174174
O	6.522886	−0.676420	−0.780154
C	8.820260	−1.287119	−0.637338
C	7.421861	−1.804413	−0.860345
O	−0.128247	2.934247	1.154029
C	−0.379241	3.145760	2.544158
O	−2.691594	3.716773	0.515525
C	−2.337492	4.687905	−0.471562
C	−5.302919	2.217404	0.168549
O	−4.353694	1.954959	−0.869665
H	−2.588243	−2.707664	−0.385291
H	−0.330404	−1.944335	−0.040582
H	−0.184697	−1.386340	−1.698003
H	−4.026407	−0.072469	−2.379001
H	−0.844917	−4.198497	−1.369003
H	−2.375226	−4.289325	−2.256672
H	−1.018722	−3.358575	−2.919895
H	0.240087	−0.330938	2.531794
H	−3.736802	−1.103850	1.187701
H	−5.649010	−2.031081	2.466736
H	−7.981421	−2.188190	−1.110898
H	−6.060789	−1.285942	−2.404243
H	1.277870	0.458994	−2.329994
H	1.019957	2.015592	−1.552520
H	3.399012	2.523558	−1.614213
H	5.026838	1.136112	−0.428800
H	3.874579	2.459886	−4.035907
H	2.172189	2.874277	−3.772619
H	2.614753	1.232950	−4.272316
H	6.357469	−0.357134	2.772252
H	5.451070	1.073937	2.219961
H	4.833925	0.092073	3.581821
H	2.061984	−1.749358	5.278428
H	1.140656	−0.361071	4.651212
H	0.850774	−1.998990	3.998804
H	9.527198	−2.116403	−0.688710
H	9.091767	−0.556696	−1.400591
H	8.910019	−0.819803	0.344372
H	7.127743	−2.529988	−0.099657
H	7.308684	−2.269676	−1.840855
H	0.337573	3.892348	2.880178
H	−1.395054	3.513759	2.703018
H	−0.226219	2.217611	3.103227
H	−2.719090	5.644468	−0.119971
H	−1.251608	4.744205	−0.581928
H	−2.796979	4.441450	−1.432482
H	−4.966945	3.036005	0.805593
H	−6.230209	2.493173	−0.329683
H	−5.467342	1.317374	0.767426

ωB97X Energy = −2096.10634044 a.u.

(*aR*,1*S*,3*S*,1’*S*,3’*S*)-**22**, Conf C

C	−0.972039	2.051791	0.597888
C	−2.272743	2.447246	0.276394
C	−3.108877	1.559911	−0.390855
C	−2.643804	0.298605	−0.765261
C	−1.341459	−0.083154	−0.459736
C	−0.500083	0.795641	0.235583
O	−2.866123	−1.666581	−2.181061
C	−1.963420	−2.366350	−1.325524
C	−0.837075	−1.436416	−0.903121
C	−3.575758	−0.620141	−1.527963
C	−1.464780	−3.573994	−2.086467
C	−4.713172	−1.175385	−0.681568
C	0.902463	0.414261	0.581648
C	1.938668	0.614895	−0.327443
C	3.229982	0.226713	0.033012
C	3.484402	−0.316680	1.282079
C	2.443029	−0.525293	2.191196
C	1.155415	−0.155000	1.827612
C	−5.914791	−1.497039	−1.307872
C	−6.972961	−2.041952	−0.597284
C	−6.804157	−2.264470	0.755185
C	−5.631726	−1.962215	1.415110
C	−4.587532	−1.410973	0.683979
C	1.703222	1.263575	−1.669679
C	2.998832	1.784441	−2.268469
O	3.980150	0.744357	−2.230020
C	4.393497	0.434668	−0.916185
C	2.851647	2.213068	−3.709741
F	−7.831213	−2.793790	1.460396
O	4.767027	−0.706703	1.579604
C	5.444273	0.133356	2.516781
O	2.780886	−1.089209	3.377581
C	1.746553	−1.295050	4.329056
C	5.241990	−0.828805	−1.066293
O	4.785449	−1.922868	−1.282345
O	6.539266	−0.545910	−1.001932
C	7.668406	−2.307596	0.241048
C	7.466147	−1.651012	−1.105813
O	−0.129309	2.948468	1.202337
C	−0.370777	3.133766	2.597984
O	−2.717931	3.697056	0.620106
C	−2.385177	4.699761	−0.342607

C	−5.305848	2.154705	0.278951
O	−4.367537	1.936148	−0.779111
H	−2.509297	−2.702454	−0.434186
H	−0.261783	−1.904559	−0.100712
H	−0.148571	−1.306681	−1.746642
H	−4.025353	−0.047606	−2.340883
H	−0.751745	−4.134974	−1.479408
H	−2.293397	−4.234100	−2.346398
H	−0.965395	−3.260662	−3.006159
H	0.327840	−0.311450	2.508066
H	−6.030141	−1.312964	−2.370980
H	−7.913672	−2.286587	−1.074916
H	−5.542922	−2.148181	2.478296
H	−3.667919	−1.151133	1.196941
H	1.257164	0.545306	−2.367654
H	0.993742	2.088414	−1.565755
H	3.366285	2.626279	−1.664477
H	5.038879	1.235741	−0.532866
H	3.794490	2.602049	−4.095931
H	2.093479	2.993754	−3.792736
H	2.543569	1.364731	−4.324978
H	4.928794	0.133583	3.478727
H	6.443858	−0.280941	2.635861
H	5.516468	1.155232	2.132837
H	2.224030	−1.734122	5.201366
H	1.274777	−0.349232	4.609519
H	0.988635	−1.983057	3.945073
H	8.400961	−3.111254	0.147804
H	8.042667	−1.585225	0.968513
H	6.734623	−2.729265	0.614666
H	7.090997	−2.356597	−1.846759
H	8.385096	−1.199272	−1.474575
H	0.341282	3.881922	2.940542
H	−1.388934	3.488146	2.771538
H	−0.203554	2.197947	3.139927
H	−2.781400	5.639978	0.035841
H	−1.301009	4.777660	−0.456210
H	−2.845117	4.470758	−1.307625
H	−6.245328	2.425250	−0.198862
H	−5.444537	1.236973	0.857055
H	−4.975757	2.963372	0.931558

ωB97X Energy = −2096.10609784 a.u.

(*aR*,1*S*,3*S*,1'*S*,3'*S*)-**22**, Conf D

C	−0.926383	2.218321	0.493923
C	−2.233881	2.524078	0.108827
C	−3.005352	1.548991	−0.514421
C	−2.467269	0.290959	−0.786384
C	−1.160077	−0.003162	−0.413207
C	−0.386055	0.962288	0.243881
O	−2.546415	−1.779079	−2.060896
C	−1.642293	−2.369578	−1.128184
C	−0.576516	−1.357165	−0.741125
C	−3.328170	−0.726044	−1.507294
C	−1.060676	−3.604155	−1.779089
C	−4.463459	−1.279238	−0.656732
C	1.017400	0.665958	0.660898
C	2.079921	0.870385	−0.215745
C	3.367427	0.527742	0.198920
C	3.598033	0.036532	1.476708
C	2.534870	−0.125055	2.371294
C	1.248137	0.169957	1.940721
C	−4.378402	−1.394073	0.727206
C	−5.414553	−1.951940	1.464906
C	−6.537416	−2.384785	0.791800
C	−6.665458	−2.284053	−0.579652
C	−5.616157	−1.729694	−1.295763
C	1.867308	1.464469	−1.586669
C	3.163919	2.007039	−2.161760
O	4.175270	1.001543	−2.055376
C	4.554208	0.747081	−0.717794
C	3.052101	2.382292	−3.620948
F	−7.556182	−2.923315	1.502158
O	4.899638	−0.206141	1.829445
C	5.213815	−1.519844	2.303340
O	2.846823	−0.551120	3.622134
C	1.787485	−0.693739	4.557384
C	5.504253	−0.444916	−0.821391
O	6.704234	−0.332055	−0.870133
O	4.857718	−1.599143	−0.931567
C	4.730751	−3.967192	−1.144771
C	5.663114	−2.782553	−1.130337
O	−0.143729	3.197585	1.047760
C	−0.423954	3.468142	2.421410
O	−2.738176	3.774435	0.353722
C	−2.676467	4.642739	−0.780151
C	−5.242510	2.102078	0.056574
O	−4.271741	1.825834	−0.958134
H	−2.202685	−2.665526	−0.231470
H	−0.007012	−1.736904	0.110476
H	0.133689	−1.252518	−1.570312
H	−3.778157	−0.237537	−2.373118
H	−0.341964	−4.080158	−1.109276
H	−1.846894	−4.323525	−2.012047
H	−0.546391	−3.336389	−2.704992
H	0.405484	0.030539	2.606142
H	−3.497460	−1.034250	1.247789
H	−5.356846	−2.046392	2.542221
H	−7.568365	−2.629900	−1.067578
H	−5.699615	−1.640237	−2.373925
H	1.465811	0.708718	−2.272261
H	1.129427	2.268784	−1.532512
H	3.485061	2.879722	−1.575286
H	5.158574	1.581030	−0.338220
H	3.996350	2.784092	−3.990219
H	2.276550	3.138478	−3.754917
H	2.787036	1.505334	−4.215819
H	6.290326	−1.634805	2.184367
H	4.937658	−1.634598	3.350214
H	4.697474	−2.274955	1.704168
H	2.249138	−1.024149	5.484442

H	1.278494	0.259810	4.723311
H	1.063834	−1.442564	4.224736
H	5.305621	−4.880541	−1.303834
H	4.200497	−4.057110	−0.195039
H	3.998142	−3.877077	−1.947824
H	6.204444	−2.675388	−2.071789
H	6.392688	−2.846803	−0.320920
H	−0.208930	2.587365	3.034496
H	0.232425	4.283497	2.719064
H	−1.465618	3.768205	2.553233
H	−3.105437	5.593372	−0.469740
H	−1.638388	4.792656	−1.088744
H	−3.255552	4.232138	−1.611145
H	−4.990401	3.012459	0.601306
H	−6.192712	2.229188	−0.458174
H	−5.315780	1.259720	0.749897

ωB97X Energy = −2096.10605516 a.u.

(*aR*,1*S*,3*S*,1'*S*,3'*S*)-**22**, Conf E

C	−0.869220	2.216215	0.320760
C	−2.167230	2.534386	−0.085884
C	−2.971714	1.542138	−0.634797
C	−2.475754	0.249385	−0.809348
C	−1.175425	−0.056309	−0.421901
C	−0.367428	0.930680	0.158275
O	−2.632076	−1.912204	−1.915150
C	−1.735428	−2.454816	−0.946809
C	−0.635465	−1.447983	−0.651850
C	−3.374529	−0.793930	−1.441193
C	−1.197658	−3.753279	−1.504557
C	−4.517285	−1.241688	−0.539957
C	1.030477	0.624816	0.587459
C	2.088312	0.728567	−0.312580
C	3.372446	0.397078	0.121969
C	3.598818	0.004969	1.431745
C	2.536425	−0.102861	2.334038
C	1.256010	0.210630	1.898049
C	−4.428745	−1.229465	0.848327
C	−5.474228	−1.691465	1.637404
C	−6.609254	−2.159824	1.010272
C	−6.740287	−2.185910	−0.364444
C	−5.682078	−1.724803	−1.131640
C	1.881305	1.214309	−1.726110
C	3.187899	1.676579	−2.347916
O	4.174751	0.656491	−2.168494
C	4.557418	0.496545	−0.818096
C	3.075212	1.939388	−3.831330
F	−7.637253	−2.605289	1.770013
O	4.875018	−0.343071	1.799692
C	5.525427	0.596253	2.659419
O	2.846498	−0.522515	3.586210
C	1.788379	−0.631041	4.527698
C	5.456889	−0.739476	−0.819641
O	6.660952	−0.678110	−0.870213
O	4.763096	−1.868901	−0.835911
C	4.540672	−4.239922	−0.824432
C	5.522307	−3.096828	−0.873645
O	−0.057337	3.207558	0.807955
C	−0.334093	3.582751	2.157879
O	−2.637700	3.813607	0.058900
C	−2.343146	4.650800	−1.061496
C	−5.188754	2.208843	−0.110709
O	−4.227659	1.828228	−1.100387
H	−2.293409	−2.661765	−0.023988
H	−0.064629	−1.778706	0.219071
H	0.064819	−1.427975	−1.495622
H	−3.818216	−0.361869	−2.339592
H	−0.480956	−4.193833	−0.808843
H	−2.006029	−4.467118	−1.668461
H	−0.691241	−3.573655	−2.455776
H	0.412478	0.127096	2.571837
H	−3.537934	−0.842321	1.331100
H	−5.414384	−1.684594	2.718737
H	−7.652182	−2.556470	−0.816181
H	−5.767487	−1.736542	−2.213333
H	1.458363	0.415673	−2.347015
H	1.163717	2.038510	−1.735373
H	3.534992	2.583708	−1.833092
H	5.197947	1.334634	−0.512633
H	4.025950	2.288957	−4.235400
H	2.316323	2.700673	−4.020309
H	2.786265	1.025106	−4.354452
H	6.528664	0.212515	2.833175
H	5.588162	1.576177	2.177392
H	4.993897	0.683262	3.608524
H	2.245533	−0.966656	5.455089
H	1.303577	0.335710	4.689262
H	1.045146	−1.364055	4.202965
H	5.083112	−5.185909	−0.857240
H	3.956537	−4.210448	0.096449
H	3.858576	−4.205257	−1.675032
H	6.117585	−3.107620	−1.788397
H	6.202934	−3.106443	−0.020476
H	0.357266	4.385580	2.406243
H	−1.361646	3.938312	2.259132
H	−0.163988	2.736766	2.830888
H	−2.771287	5.627456	−0.844803
H	−1.262519	4.745471	−1.195488
H	−2.796222	4.246071	−1.970379
H	−4.893706	3.135126	0.382637
H	−6.128854	2.353614	−0.639251
H	−5.307862	1.411372	0.627960

ωB97X Energy = −2096.10591385 a.u.

(*aR*,1*S*,3*S*,1'*S*,3'*S*)-**22**, Conf F

C	−1.034924	2.086242	0.675565
C	−2.339996	2.445784	0.329690
C	−3.129195	1.544273	−0.376816
C	−2.609932	0.312546	−0.775267
C	−1.304673	−0.033858	−0.441857

C	−0.514020	0.852684	0.300905
O	−2.722358	−1.616087	−2.251429
C	−1.822690	−2.311084	−1.388815
C	−0.742267	−1.357344	−0.904408
C	−3.488049	−0.616359	−1.587777
C	−1.259140	−3.477994	−2.167697
C	−4.624563	−1.241419	−0.790080
C	0.887129	0.500837	0.681242
C	1.946069	0.737065	−0.191409
C	3.230515	0.344981	0.188634
C	3.465413	−0.220155	1.435187
C	2.406459	−0.404669	2.332335
C	1.121149	−0.068662	1.929159
C	−5.781957	−1.620588	−1.465960
C	−6.833634	−2.233591	−0.803099
C	−6.703842	−2.465029	0.552291
C	−5.576259	−2.106896	1.260350
C	−4.537348	−1.488923	0.576170
C	1.738681	1.432178	−1.514488
C	3.038616	2.022015	−2.031535
O	4.041935	1.003608	−2.022450
C	4.414910	0.601937	−0.718253
C	2.933165	2.539067	−3.447224
F	−7.725416	−3.061038	1.210901
O	4.766683	−0.494908	1.770689
C	5.060889	−1.847602	2.139361
O	2.718454	−0.887773	3.562568
C	1.664843	−1.043139	4.502199
C	5.256310	−0.650043	−0.965850
O	4.801500	−1.756325	−1.118310
O	6.542439	−0.338443	−1.086160
C	7.880527	−2.225685	−0.282938
C	7.456234	−1.392934	−1.470810
O	−0.232768	2.995318	1.313953
C	−0.519471	3.166161	2.702160
O	−2.821169	3.674986	0.697266
C	−2.833694	4.627024	−0.369423
C	−5.353752	2.063855	0.268123
O	−4.396013	1.878030	−0.779155
H	−2.383943	−2.690982	−0.525014
H	−0.178200	−1.828682	−0.095839
H	−0.030880	−1.181513	−1.720335
H	−3.937912	−0.037471	−2.396017
H	−0.544409	−4.030876	−1.555177
H	−2.055315	−4.159170	−2.471023
H	−0.744606	−3.120252	−3.062516
H	0.282470	−0.228148	2.594897
H	−5.866828	−1.429518	−2.530724
H	−7.740148	−2.524630	−1.319322
H	−5.517138	−2.302758	2.323847
H	−3.652302	−1.188339	1.126408
H	1.349014	0.730685	−2.261635
H	0.996107	2.226296	−1.404098
H	3.362797	2.831202	−1.361640
H	5.061122	1.364837	−0.265845
H	3.881754	2.966701	−3.773908
H	2.164859	3.311851	−3.507189
H	2.661615	1.727682	−4.126152
H	4.567723	−2.114854	3.072219
H	4.753119	−2.529036	1.341887
H	6.141124	−1.896183	2.263318
H	1.176600	−0.086410	4.707252
H	0.923473	−1.765282	4.150192
H	2.128221	−1.415860	5.412206
H	8.648078	−2.935217	−0.596895
H	8.299449	−1.593314	0.501842
H	7.039777	−2.787995	0.123246
H	6.980316	−2.002498	−2.239257
H	8.301170	−0.863710	−1.907141
H	−1.548094	3.501943	2.847938
H	−0.352136	2.229697	3.243174
H	0.169198	3.923783	3.070913
H	−3.489464	4.292803	−1.176976
H	−3.211939	5.557809	0.048377
H	−1.821823	4.782172	−0.753239
H	−5.447831	1.149353	0.860090
H	−5.072838	2.898326	0.911795
H	−6.302079	2.275582	−0.221527

ωB97X Energy = −2096.10585458 a.u.

(*aR*,1*S*,3*S*,1’*S*,3’*S*)-**22**, Conf G

C	−1.022674	2.102280	0.562004
C	−2.311095	2.466244	0.164157
C	−3.101896	1.542917	−0.510212
C	−2.601358	0.277522	−0.817891
C	−1.311787	−0.074538	−0.432675
C	−0.518598	0.839276	0.273549
O	−2.720851	−1.733848	−2.179354
C	−1.856213	−2.393401	−1.255475
C	−0.767226	−1.434340	−0.801506
C	−3.479664	−0.680241	−1.596104
C	−1.299593	−3.616112	−1.949127
C	−4.648809	−1.230498	−0.790708
C	0.865811	0.487209	0.710937
C	1.954678	0.681262	−0.135112
C	3.223799	0.308154	0.308960
C	3.412551	−0.204315	1.585637
C	2.321807	−0.359350	2.448632
C	1.053216	−0.033886	1.987961
C	−5.807245	−1.603954	−1.467694
C	−6.888708	−2.150933	−0.794875
C	−6.787295	−2.322621	0.571710
C	−5.659813	−1.967213	1.281536
C	−4.591163	−1.415166	0.587210
C	1.791329	1.301626	−1.501202
C	3.112592	1.830905	−2.031429
O	4.103537	0.806342	−1.913375
C	4.438984	0.524080	−0.570226
C	3.047608	2.230704	−3.486965
F	−7.837565	−2.854451	1.239929
O	4.700589	−0.461542	1.979390

C	4.993709	−1.801069	2.394133
O	2.590326	−0.804559	3.702873
C	1.504411	−0.930574	4.609576
C	5.317282	−0.722654	−0.659900
O	4.889260	−1.835461	−0.842064
O	6.606741	−0.414009	−0.575207
C	7.837757	−1.794489	−2.169128
C	7.559899	−1.493971	−0.713851
O	−0.220234	3.033971	1.167665
C	−0.523448	3.268338	2.543369
O	−2.784176	3.722627	0.440298
C	−2.521748	4.664544	−0.602132
C	−5.333007	2.136130	0.035828
O	−4.346512	1.884251	−0.969978
H	−2.445276	−2.710394	−0.384769
H	−0.233039	−1.867924	0.047358
H	−0.033033	−1.321894	−1.608356
H	−3.896968	−0.141652	−2.448415
H	−0.611712	−4.145400	−1.287072
H	−2.102956	−4.298111	−2.230946
H	−0.756397	−3.324129	−2.850887
H	0.190727	−0.164414	2.629272
H	−5.869570	−1.459431	−2.541309
H	−7.796321	−2.436524	−1.312199
H	−5.623793	−2.115093	2.353788
H	−3.706098	−1.115669	1.138054
H	1.396991	0.564193	−2.210414
H	1.065695	2.117510	−1.454411
H	3.431653	2.688846	−1.422488
H	5.045124	1.341401	−0.159439
H	4.007556	2.624424	−3.823041
H	2.286890	3.000111	−3.630449
H	2.787004	1.367249	−4.103145
H	6.077893	−1.858790	2.477471
H	4.534269	−2.020963	3.356353
H	4.648334	−2.513505	1.640999
H	0.773538	−1.661215	4.253119
H	1.935201	−1.277739	5.545343
H	1.012121	0.032622	4.770077
H	8.607073	−2.565441	−2.238674
H	6.941441	−2.157860	−2.672867
H	8.198258	−0.902971	−2.684324
H	8.449986	−1.131228	−0.203465
H	7.175066	−2.368212	−0.188036
H	−1.553885	3.609999	2.661152
H	−0.362827	2.357646	3.128501
H	0.160709	4.042316	2.885500
H	−1.445066	4.774604	−0.755829
H	−3.000002	4.348565	−1.533006
H	−2.943299	5.614438	−0.279401
H	−5.047808	2.985585	0.657182
H	−6.257572	2.358287	−0.493265
H	−5.475815	1.247457	0.656770

ωB97X Energy = −2096.10575637 a.u.

(*aR*,1*S*,3*S*,1'*S*,3'*S*)-**22**, Conf H

C	−0.977379	2.089200	0.387047
C	−2.257579	2.457083	−0.032929
C	−3.076603	1.507071	−0.632444
C	−2.611447	0.208804	−0.845065
C	−1.327963	−0.146464	−0.443119
C	−0.506932	0.796441	0.189170
O	−2.805682	−1.907827	−2.030222
C	−1.941892	−2.508011	−1.066096
C	−0.821077	−1.543056	−0.714258
C	−3.525048	−0.784460	−1.533606
C	−1.428446	−3.800019	−1.660450
C	−4.702443	−1.224580	−0.674511
C	0.871446	0.441794	0.643852
C	1.960715	0.570678	−0.214383
C	3.226612	0.213739	0.252364
C	3.404100	−0.231722	1.552712
C	2.309082	−0.370709	2.410859
C	1.047457	−0.029462	1.942894
C	−4.631237	−1.301424	0.712994
C	−5.709639	−1.753992	1.461918
C	−6.860531	−2.119762	0.796235
C	−6.975770	−2.053259	−0.578358
C	−5.884144	−1.605049	−1.305761
C	1.808352	1.115973	−1.612928
C	3.138064	1.603440	−2.162768
O	4.117380	0.574777	−1.991235
C	4.446728	0.352283	−0.636307
C	3.082772	1.932268	−3.636316
F	−7.920529	−2.554105	1.517514
O	4.666571	−0.589164	1.957552
C	5.281538	0.329744	2.863879
O	2.572226	−0.840797	3.655601
C	1.481602	−0.973233	4.555975
C	5.291275	−0.922188	−0.655757
O	4.838250	−2.025595	−0.827772
O	6.584672	−0.645126	−0.525573
C	7.847226	−2.118140	−2.007052
C	7.509399	−1.756162	−0.578469
O	−0.149714	3.040007	0.925707
C	−0.441609	3.370516	2.284094
O	−2.696123	3.742834	0.149178
C	−2.385349	4.602720	−0.949364
C	−5.284803	2.211987	−0.123940
O	−4.315461	1.843485	−1.110135
H	−2.522396	−2.733262	−0.161669
H	−0.276505	−1.921034	0.154153
H	−0.104312	−1.511194	−1.543671
H	−3.934232	−0.308320	−2.426062
H	−0.738872	−4.286001	−0.967701
H	−2.252858	−4.483988	−1.866528
H	−0.897925	−3.600596	−2.594421
H	0.180088	−0.130369	2.583161
H	−3.727619	−0.992579	1.227424
H	−5.663234	−1.817724	2.542042
H	−7.901269	−2.342866	−1.060557

H	−5.957187	−1.543362	−2.386641
H	1.410784	0.344441	−2.282920
H	1.091134	1.940496	−1.615233
H	3.463997	2.486686	−1.595142
H	5.075166	1.173685	−0.268029
H	4.048048	2.299024	−3.987308
H	2.330800	2.701594	−3.820368
H	2.815336	1.042365	−4.210505
H	5.352774	1.323212	2.411500
H	4.719009	0.387100	3.797371
H	6.281493	−0.052003	3.059895
H	1.904184	−1.345740	5.485686
H	0.999024	−0.008497	4.735233
H	0.744210	−1.687597	4.180190
H	8.596478	−2.911647	−2.012040
H	6.965629	−2.475206	−2.540303
H	8.257492	−1.256441	−2.535670
H	8.385383	−1.401044	−0.039000
H	7.073179	−2.598220	−0.040995
H	−0.311056	2.493648	2.925703
H	0.268781	4.141016	2.577033
H	−1.459688	3.753729	2.380220
H	−2.851551	4.234275	−1.867001
H	−2.788216	5.583419	−0.704275
H	−1.303046	4.674256	−1.085226
H	−6.213635	2.390878	−0.661891
H	−5.430583	1.394950	0.588132
H	−4.979868	3.116808	0.402310

ωB97X Energy = −2096.10540689 a.u.

(*aR*,1*S*,3*S*,1'*S*,3'*S*)-**22**, Conf I

C	−0.900928	2.128402	0.514536
C	−2.208163	2.486030	0.175692
C	−3.026453	1.553596	−0.453163
C	−2.534694	0.289760	−0.779821
C	−1.226719	−0.055084	−0.455094
C	−0.405926	0.865757	0.208640
O	−2.720534	−1.740791	−2.106012
C	−1.806569	−2.383708	−1.218425
C	−0.696568	−1.415536	−0.841876
C	−3.449555	−0.678449	−1.501193
C	−1.287161	−3.618586	−1.919256
C	−4.577329	−1.216791	−0.631270
C	1.000342	0.523485	0.579782
C	2.043699	0.724664	−0.320676
C	3.339534	0.379065	0.067270
C	3.589215	−0.124424	1.333870
C	2.540210	−0.337210	2.233190
C	1.249331	−0.007346	1.843407
C	−5.765214	−1.605358	−1.245949
C	−6.811824	−2.142992	−0.513351
C	−6.645397	−2.290223	0.849710
C	−5.486746	−1.919604	1.498850
C	−4.453840	−1.377276	0.745161
C	1.809169	1.332208	−1.682147
C	3.098201	1.872216	−2.277767
O	4.107124	0.861501	−2.197658
C	4.510635	0.598639	−0.869872
C	2.960169	2.257818	−3.732003
F	−7.661189	−2.812524	1.576288
O	4.876606	−0.472121	1.659922
C	5.515349	0.411223	2.584871
O	2.874465	−0.863662	3.437886
C	1.832040	−1.067427	4.380953
C	5.457128	−0.596625	−0.985805
O	6.657656	−0.480580	−1.019780
O	4.802872	−1.741413	−1.130198
C	6.052797	−3.473625	0.052693
C	5.590665	−2.944047	−1.285903
O	−0.073016	3.062723	1.079576
C	−0.323794	3.311038	2.463216
O	−2.663193	3.743079	0.476100
C	−2.666409	4.632876	−0.642840
C	−5.228421	2.159497	0.200507
O	−4.297321	1.878069	−0.849218
H	−2.347059	−2.684864	−0.311323
H	−0.109167	−1.837019	−0.022673
H	−0.014112	−1.308428	−1.693558
H	−3.908543	−0.151805	−2.339531
H	−0.567028	−4.138148	−1.284296
H	−2.104575	−4.303622	−2.148369
H	−0.790329	−3.342878	−2.852319
H	0.416508	−0.164603	2.517271
H	−5.878613	−1.479945	−2.317757
H	−7.741631	−2.440229	−0.982433
H	−5.399156	−2.048960	2.570486
H	−3.544445	−1.066290	1.247881
H	1.391248	0.584394	−2.366545
H	1.077052	2.140237	−1.608433
H	3.434373	2.739530	−1.691754
H	5.123274	1.430989	−0.498618
H	3.898761	2.658590	−4.116479
H	2.185091	3.017716	−3.846158
H	2.680557	1.385869	−4.327558
H	4.989511	0.414931	3.541210
H	6.527063	0.035016	2.723127
H	5.555984	1.426294	2.179035
H	1.337469	−0.124842	4.631411
H	1.093241	−1.780112	4.004711
H	2.306725	−1.474924	5.269951
H	6.591848	−4.410826	−0.097483
H	6.718798	−2.765243	0.545880
H	5.200942	−3.667683	0.706139
H	4.917801	−3.641620	−1.781016
H	6.431326	−2.727789	−1.945362
H	−0.153275	2.402832	3.049541
H	0.381065	4.079353	2.774775
H	−1.345189	3.666102	2.614567
H	−3.025920	5.592398	−0.276544
H	−1.654724	4.748539	−1.040898
H	−3.333475	4.265265	−1.426468

H	−5.296003	1.309378	0.884834
H	−4.940690	3.056901	0.749205
H	−6.191901	2.312000	−0.281581

ωB97X Energy = −2096.10536729 a.u.

(*aR*,1*S*,3*S*,1'*S*,3'*S*)-**22**, Conf J

C	−1.061353	2.094372	0.639420
C	−2.358605	2.458794	0.273228
C	−3.143695	1.558029	−0.438556
C	−2.658900	0.289558	−0.749923
C	−1.366261	−0.071081	−0.379406
C	−0.553133	0.842887	0.303067
O	−2.806084	−1.717121	−2.104947
C	−1.946437	−2.386961	−1.182775
C	−0.841123	−1.440480	−0.742324
C	−3.557066	−0.672141	−1.499011
C	−1.413534	−3.622973	−1.870794
C	−4.693749	−1.247199	−0.660467
C	0.847364	0.493255	0.688654
C	1.890711	0.595862	−0.229059
C	3.179191	0.241294	0.178335
C	3.427063	−0.178540	1.477365
C	2.381837	−0.246238	2.406781
C	1.097265	0.072813	1.992361
C	−5.724653	−1.909180	−1.324960
C	−6.771879	−2.490250	−0.629036
C	−6.769264	−2.397676	0.749651
C	−5.770680	−1.752106	1.445845
C	−4.730190	−1.176190	0.726673
C	1.671603	1.093641	−1.637775
C	2.964241	1.605752	−2.248337
O	3.967952	0.597113	−2.108944
C	4.355564	0.388298	−0.766715
C	2.839064	1.917096	−3.721450
F	−7.789446	−2.959591	1.440767
O	4.729314	−0.431586	1.825412
C	5.019754	−1.750781	2.303366
O	2.713445	−0.600348	3.674731
C	1.673968	−0.662737	4.640869
C	5.219754	−0.870079	−0.819870
O	4.779220	−1.987300	−0.933291
O	6.513650	−0.575411	−0.794187
C	8.829933	−1.128892	−0.755183
C	7.435291	−1.685148	−0.889874
O	−0.310316	2.959820	1.390987
C	0.244712	4.052714	0.659620
O	−2.832011	3.701660	0.601330
C	−3.697025	3.700507	1.739572
C	−4.544105	2.886133	−1.795499
O	−4.430708	1.876403	−0.787645
H	−2.537228	−2.685760	−0.306367
H	−0.308134	−1.874005	0.107575
H	−0.110973	−1.347354	−1.554519
H	−4.008216	−0.137395	−2.337539
H	−0.728478	−4.157425	−1.209956
H	−2.229263	−4.295005	−2.140971
H	−0.872966	−3.346489	−2.779060
H	0.271071	0.026052	2.690113
H	−5.706927	−1.973214	−2.407987
H	−7.579569	−3.002418	−1.137305
H	−5.808661	−1.700744	2.527018
H	−3.941139	−0.662235	1.264477
H	1.285256	0.288087	−2.272824
H	0.921656	1.888090	−1.647402
H	3.296701	2.501459	−1.703913
H	4.985919	1.220843	−0.429210
H	3.783350	2.291392	−4.118488
H	2.070480	2.675525	−3.880486
H	2.558053	1.017591	−4.273757
H	4.666163	−2.497166	1.587581
H	6.104176	−1.808879	2.383304
H	4.565945	−1.921642	3.278186
H	2.149473	−0.958705	5.572574
H	1.196664	0.312261	4.769846
H	0.921596	−1.405859	4.364134
H	9.553679	−1.941909	−0.828038
H	9.040705	−0.409057	−1.547128
H	8.960623	−0.638181	0.210234
H	7.201143	−2.397864	−0.097019
H	7.281803	−2.178556	−1.851082
H	0.967222	3.692765	−0.079537
H	−0.540470	4.626784	0.163444
H	0.757373	4.683705	1.382907
H	−4.582910	3.089734	1.549353
H	−3.167975	3.324570	2.619297
H	−3.993538	4.733931	1.907187
H	−5.607745	3.012376	−1.986069
H	−4.114419	3.828354	−1.452258
H	−4.045087	2.562696	−2.713533

ωB97X Energy = −2096.10534483 a.u.

(*aR*,1*S*,3*S*,1'*S*,3'*S*)-**22**, Conf K

C	−1.078716	2.179496	0.006280
C	−2.381655	2.409795	−0.450934
C	−3.140072	1.331494	−0.896344
C	−2.613550	0.038397	−0.869272
C	−1.324146	−0.184212	−0.401862
C	−0.543015	0.897053	0.029287
O	−2.720308	−2.278840	−1.605122
C	−1.866258	−2.638887	−0.520189
C	−0.775489	−1.591283	−0.368143
C	−3.482064	−1.101686	−1.360520
C	−1.312480	−4.014460	−0.815148
C	−4.668677	−1.403100	−0.454310
C	0.858282	0.692706	0.505223
C	1.906356	0.526356	−0.398173
C	3.192322	0.306230	0.100912
C	3.435380	0.285511	1.466896
C	2.386072	0.490641	2.371035
C	1.103190	0.671616	1.875730

C	−5.800355	−1.990168	−1.015327
C	−6.895032	−2.331155	−0.236609
C	−6.834765	−2.076731	1.119594
C	−5.735293	−1.497249	1.716300
C	−4.651913	−1.159426	0.915106
C	1.696116	0.593702	−1.891944
C	2.993360	0.904695	−2.617416
O	3.991267	−0.024566	−2.187562
C	4.372062	0.159402	−0.839170
C	2.875836	0.778293	−4.118419
F	−7.898777	−2.403573	1.890511
O	4.736637	0.145757	1.876640
C	5.026156	−0.966366	2.732490
O	2.711427	0.521320	3.688596
C	1.667647	0.742379	4.626305
C	5.214593	−1.074896	−0.521888
O	4.754901	−2.164915	−0.286171
O	6.513009	−0.813392	−0.607530
C	8.821692	−1.391077	−0.513264
C	7.415879	−1.921213	−0.389132
O	−0.333947	3.241483	0.456655
C	0.262514	4.025063	−0.577225
O	−2.887436	3.678521	−0.547134
C	−3.122419	4.340324	0.700362
C	−5.393292	2.064812	−0.605658
O	−4.385182	1.491637	−1.439564
H	−2.461948	−2.678931	0.401479
H	−0.237084	−1.758942	0.567980
H	−0.047342	−1.722701	−1.176489
H	−3.881807	−0.827890	−2.338360
H	−0.623793	−4.321409	−0.025770
H	−2.116780	−4.748597	−0.878882
H	−0.769990	−4.006436	−1.763403
H	0.273662	0.825665	2.553794
H	−5.829913	−2.180795	−2.083154
H	−7.781507	−2.781276	−0.666121
H	−5.731261	−1.311573	2.783304
H	−3.787361	−0.689990	1.371974
H	1.311580	−0.360746	−2.269164
H	0.947566	1.350961	−2.136815
H	3.327937	1.918434	−2.353043
H	5.015202	1.044338	−0.753420
H	3.825571	1.013178	−4.600270
H	2.115856	1.465498	−4.494286
H	2.587723	−0.239942	−4.389046
H	6.111709	−1.017146	2.801377
H	4.596822	−0.819875	3.722319
H	4.645292	−1.890747	2.291292
H	2.139234	0.732756	5.605716
H	1.189194	1.711505	4.462155
H	0.916765	−0.050514	4.576399
H	9.531510	−2.205358	−0.360910
H	8.991575	−0.967674	−1.504032
H	9.014883	−0.622129	0.235915
H	7.221702	−2.335143	0.602177
H	7.202021	−2.692277	−1.131041
H	1.023490	3.445291	−1.108209
H	−0.492383	4.379611	−1.282890
H	0.735681	4.875573	−0.090488
H	−3.596701	5.289546	0.458986
H	−3.794907	3.746049	1.325941
H	−2.186341	4.515711	1.230975
H	−5.384930	1.596606	0.382642
H	−5.259623	3.142237	−0.509452
H	−6.344835	1.856889	−1.091676

ωB97X Energy = −2096.10531944 a.u.

(*aR*,1*S*,3*S*,1’*S*,3’*S*)-**22**, Conf L

C	−0.974845	2.224834	0.361127
C	−2.270437	2.504853	−0.079930
C	−3.026110	1.492603	−0.660805
C	−2.483663	0.218394	−0.832990
C	−1.186285	−0.049309	−0.409452
C	−0.427848	0.957052	0.203582
O	−2.537347	−1.935915	−1.962054
C	−1.652009	−2.459450	−0.973040
C	−0.594819	−1.420725	−0.635675
C	−3.329282	−0.847396	−1.499725
C	−1.057850	−3.734383	−1.527776
C	−4.481376	−1.340630	−0.634640
C	0.962790	0.686723	0.677450
C	2.050732	0.835196	−0.179145
C	3.324067	0.512641	0.291181
C	3.515957	0.095743	1.601346
C	2.427346	−0.009128	2.473392
C	1.155023	0.265735	1.990173
C	−5.613463	−1.854589	−1.262731
C	−6.676747	−2.357247	−0.529377
C	−6.583883	−2.341319	0.848622
C	−5.482103	−1.843257	1.511210
C	−4.431084	−1.339720	0.755616
C	1.880952	1.348519	−1.587890
C	3.197003	1.852748	−2.153879
O	4.198817	0.849763	−1.963415
C	4.537994	0.666448	−0.603627
C	3.129227	2.148625	−3.633834
F	−7.617136	−2.827513	1.575552
O	4.805165	−0.133877	2.003663
C	5.098069	−1.421690	2.556197
O	2.702607	−0.364729	3.754429
C	1.616439	−0.461809	4.664311
C	5.472321	−0.542957	−0.610736
O	6.674750	−0.448205	−0.610870
O	4.805886	−1.689699	−0.687860
C	5.988264	−3.156820	−2.238867
C	5.578698	−2.906881	−0.805436
O	−0.209827	3.238234	0.877909
C	−0.531691	3.589947	2.224012
O	−2.785363	3.766888	0.064832
C	−2.514246	4.615864	−1.052489
C	−5.278561	2.079435	−0.194182

O	−4.277219	1.742856	−1.159606
H	−2.229715	−2.694355	−0.069166
H	−0.040055	−1.741562	0.249203
H	0.129591	−1.369315	−1.457493
H	−3.761700	−0.421421	−2.406486
H	−0.347015	−4.159621	−0.816744
H	−1.838751	−4.471130	−1.720916
H	−0.531709	−3.529334	−2.463003
H	0.293043	0.168008	2.638078
H	−5.668982	−1.857522	−2.346436
H	−7.563935	−2.751772	−1.009152
H	−5.452174	−1.845706	2.593822
H	−3.566680	−0.928882	1.265981
H	1.496160	0.555635	−2.240272
H	1.146023	2.157212	−1.602471
H	3.506275	2.754578	−1.606571
H	5.143815	1.512466	−0.254415
H	4.085761	2.526367	−3.996860
H	2.361913	2.899351	−3.830895
H	2.876723	1.241673	−4.187756
H	4.807552	−1.472010	3.604348
H	4.581224	−2.204803	1.995159
H	6.174595	−1.555127	2.457840
H	2.051159	−0.745220	5.619539
H	1.103185	0.498142	4.768652
H	0.902534	−1.227004	4.348266
H	6.501982	−4.117315	−2.306475
H	5.112818	−3.189744	−2.889069
H	6.665529	−2.379838	−2.594808
H	6.443156	−2.838701	−0.144319
H	4.908801	−3.683427	−0.440150
H	0.113322	4.424745	2.491072
H	−1.577593	3.893001	2.306408
H	−0.332845	2.749121	2.895688
H	−2.968934	5.579656	−0.832275
H	−1.436539	4.740724	−1.185753
H	−2.955869	4.202024	−1.962882
H	−6.208990	2.191930	−0.747168
H	−5.387157	1.273082	0.536367
H	−5.033537	3.012891	0.312940

ωB97X Energy = −2096.10520155 a.u.

(*aR*,1*S*,3*S*,1’*S*,3’*S*)-**22**, Conf M

C	−1.070001	2.022663	0.827739
C	−2.362603	2.425593	0.474506
C	−3.156815	1.561795	−0.274895
C	−2.646555	0.345282	−0.728861
C	−1.346424	−0.030548	−0.410056
C	−0.551264	0.813015	0.377164
O	−2.788964	−1.532281	−2.267413
C	−1.896562	−2.268245	−1.431169
C	−0.802446	−1.344424	−0.920494
C	−3.539655	−0.546322	−1.567492
C	−1.350942	−3.415536	−2.250916
C	−4.677926	−1.186507	−0.784132
C	0.847881	0.435425	0.741808
C	1.898296	0.616013	−0.155900
C	3.183380	0.225743	0.229083
C	3.422004	−0.303741	1.489130
C	2.369972	−0.449255	2.401844
C	1.088616	−0.095254	2.006519
C	−4.581565	−1.488712	0.570472
C	−5.622472	−2.118863	1.240206
C	−6.761661	−2.433357	0.530018
C	−6.900692	−2.147092	−0.813931
C	−5.846650	−1.523122	−1.462854
C	1.693403	1.235567	−1.517659
C	2.991412	1.804116	−2.063207
O	3.993699	0.786025	−2.010601
C	4.367474	0.446793	−0.690864
C	2.881767	2.255269	−3.500943
F	−7.785478	−3.040313	1.174963
O	4.721992	−0.587036	1.821934
C	5.007476	−1.939833	2.198296
O	2.691258	−0.911158	3.637298
C	1.644304	−1.050214	4.587279
C	5.214862	−0.813450	−0.857439
O	4.760043	−1.913819	−1.050868
O	6.512214	−0.534712	−0.829382
C	8.823806	−1.101926	−0.912940
C	7.420775	−1.641790	−1.026212
O	−0.326003	2.816222	1.662359
C	0.324735	3.914417	1.029464
O	−2.817286	3.633959	0.928059
C	−3.158065	4.583731	−0.085812
C	−5.370350	2.038681	0.435893
O	−4.434762	1.901602	−0.638455
H	−2.460144	−2.669757	−0.579013
H	−0.239665	−1.848384	−0.130767
H	−0.095391	−1.154068	−1.735968
H	−3.987700	0.063507	−2.353669
H	−0.639971	−3.996496	−1.660424
H	−2.156723	−4.076985	−2.571994
H	−0.836589	−3.034692	−3.136268
H	0.257338	−0.201579	2.691522
H	−3.687938	−1.220903	1.123825
H	−5.556224	−2.356284	2.294774
H	−7.816116	−2.404808	−1.332158
H	−5.938877	−1.289546	−2.518500
H	1.321311	0.488010	−2.227349
H	0.938272	2.023620	−1.467398
H	3.317671	2.643925	−1.432439
H	5.006447	1.234857	−0.272717
H	3.830575	2.663984	−3.850346
H	2.115786	3.027118	−3.594160
H	2.605444	1.413874	−4.140266
H	6.091491	−2.007494	2.275178
H	4.551029	−2.182654	3.156509
H	4.652699	−2.628504	1.427447
H	2.111874	−1.423110	5.495069
H	1.167760	−0.087861	4.791633

H	0.892868	−1.766493	4.244755
H	9.537968	−1.914644	−1.052830
H	9.012059	−0.344143	−1.674593
H	8.992249	−0.661562	0.070707
H	7.211918	−2.398138	−0.267311
H	7.227930	−2.078091	−2.007645
H	−0.397109	4.571959	0.539109
H	0.839050	4.466299	1.813742
H	1.057962	3.561636	0.297502
H	−3.333991	5.527279	0.426529
H	−2.332383	4.701438	−0.793425
H	−4.055634	4.282537	−0.628402
H	−5.085656	2.851173	1.106719
H	−6.331213	2.258146	−0.024910
H	−5.441712	1.103769	0.997530

ωB97X Energy = −2096.10519018 a.u.

(*aR*,1*S*,3*S*,1’*S*,3’*S*)-**22**, Conf N

C	−1.058052	1.984247	0.907562
C	−2.350073	2.415369	0.585731
C	−3.153068	1.603831	−0.212306
C	−2.669051	0.384460	−0.691026
C	−1.376800	−0.025238	−0.384086
C	−0.562433	0.781119	0.420507
O	−2.819837	−1.404567	−2.324488
C	−1.948862	−2.198870	−1.519492
C	−0.846241	−1.321291	−0.950257
C	−3.569199	−0.460606	−1.567718
C	−1.414376	−3.313326	−2.389978
C	−4.702536	−1.152842	−0.819530
C	0.831092	0.363414	0.759963
C	1.892409	0.682817	−0.083351
C	3.168889	0.219689	0.237494
C	3.393420	−0.506143	1.399608
C	2.333436	−0.782484	2.270850
C	1.055268	−0.365683	1.924012
C	−5.761560	−1.667241	−1.565168
C	−6.807528	−2.342041	−0.957264
C	−6.774273	−2.495048	0.415443
C	−5.747397	−2.001502	1.190587
C	−4.709523	−1.326724	0.559084
C	1.691685	1.526963	−1.317924
C	3.002335	2.125276	−1.797625
O	3.977458	1.084505	−1.899922
C	4.354703	0.559032	−0.643308
C	2.891311	2.775420	−3.156971
F	−7.793016	−3.150541	1.021360
O	4.687517	−0.856738	1.688720
C	4.953307	−2.255978	1.844672
O	2.640232	−1.434768	3.421386
C	1.586872	−1.697663	4.337008
C	5.185783	−0.673254	−0.995600
O	4.715506	−1.719556	−1.368704
O	6.487319	−0.428124	−0.907526
C	8.788094	−1.035246	−1.036463
C	7.376732	−1.505824	−1.279224
O	−0.251680	2.781076	1.681157
C	−0.568547	2.765820	3.074289
O	−2.862302	3.574234	1.105974
C	−2.253472	4.781203	0.635427
C	−4.688956	3.177453	−1.151681
O	−4.447115	1.921353	−0.514739
H	−2.530021	−2.629732	−0.693140
H	−0.302759	−1.873550	−0.179761
H	−0.122128	−1.097504	−1.743099
H	−4.023178	0.192473	−2.315593
H	−0.718022	−3.933802	−1.822778
H	−2.227799	−3.944861	−2.749820
H	−0.885879	−2.898105	−3.251236
H	0.214494	−0.592983	2.567160
H	−5.769059	−1.536434	−2.642302
H	−7.637192	−2.740671	−1.528019
H	−5.762245	−2.141585	2.264444
H	−3.899357	−0.927985	1.159676
H	1.261259	0.924523	−2.126743
H	0.981470	2.330426	−1.107633
H	3.358493	2.858932	−1.060435
H	5.003546	1.273452	−0.121066
H	3.845013	3.211700	−3.456115
H	2.139365	3.566027	−3.133769
H	2.593390	2.036452	−3.904041
H	4.594539	−2.807201	0.971815
H	6.035891	−2.349899	1.914486
H	4.488279	−2.644019	2.749486
H	2.046367	−2.197180	5.186209
H	1.115874	−0.769536	4.672500
H	0.831910	−2.353115	3.894841
H	9.486534	−1.823711	−1.320365
H	9.011177	−0.148431	−1.631107
H	8.944212	−0.801167	0.017440
H	7.129052	−2.381979	−0.677126
H	7.201288	−1.749736	−2.328351
H	−1.597728	3.089997	3.243566
H	−0.422448	1.763416	3.487617
H	0.118396	3.458525	3.556373
H	−2.784767	5.599841	1.116848
H	−1.196878	4.816137	0.902381
H	−2.362615	4.865685	−0.449665
H	−4.693458	3.991909	−0.427946
H	−3.936615	3.368111	−1.922046
H	−5.668377	3.096464	−1.619383

ωB97X Energy = −2096.10517428 a.u.

(*aR*,1*S*,3*S*,1’*S*,3’*S*)-**22**, Conf O

C	−1.006846	2.068830	0.529248
C	−2.297083	2.456241	0.161849
C	−3.116853	1.550267	−0.502702
C	−2.674637	0.255152	−0.763857
C	−1.388332	−0.127803	−0.393595
C	−0.540747	0.789344	0.240648

O	−2.908722	−1.810967	−2.015841
C	−2.048300	−2.459562	−1.079504
C	−0.907925	−1.525378	−0.707038
C	−3.614869	−0.712932	−1.450886
C	−1.563722	−3.741174	−1.718104
C	−4.754116	−1.205459	−0.564973
C	0.853995	0.415842	0.625588
C	1.892386	0.481410	−0.301248
C	3.180436	0.130186	0.111253
C	3.423871	−0.265708	1.415985
C	2.376844	−0.351628	2.339798
C	1.098186	−0.001049	1.932640
C	−4.743897	−1.093886	0.820123
C	−5.788925	−1.595072	1.586707
C	−6.839826	−2.208100	0.939629
C	−6.890041	−2.338985	−0.434923
C	−5.836867	−1.832420	−1.178769
C	1.672882	0.937821	−1.723665
C	2.969111	1.414773	−2.355673
O	3.963275	0.400524	−2.187596
C	4.355784	0.232807	−0.842364
C	2.840725	1.679115	−3.837596
F	−7.865289	−2.696628	1.677061
O	4.700672	−0.631244	1.764107
C	5.382289	0.304901	2.602323
O	2.704700	−0.778305	3.584791
C	1.662317	−0.878386	4.544834
C	5.207033	−1.036807	−0.844834
O	4.760218	−2.146877	−0.989157
O	6.500129	−0.755979	−0.734676
C	8.799662	−1.338445	−0.525212
C	7.407455	−1.880170	−0.728426
O	−0.220699	2.939079	1.238042
C	0.341046	4.000004	0.465596
O	−2.730283	3.724718	0.444476
C	−3.592325	3.793581	1.583100
C	−4.490811	2.860513	−1.902165
O	−4.397564	1.893812	−0.851075
H	−2.627667	−2.699723	−0.177829
H	−0.366266	−1.936380	0.148392
H	−0.195666	−1.487107	−1.539342
H	−4.063605	−0.206337	−2.307921
H	−0.879288	−4.262634	−1.046378
H	−2.402877	−4.402049	−1.939677
H	−1.035529	−3.522680	−2.649177
H	0.269673	−0.044604	2.628063
H	−3.914195	−0.604902	1.318595
H	−5.790534	−1.511094	2.666486
H	−7.738054	−2.822793	−0.903743
H	−5.857289	−1.926772	−2.259528
H	1.274902	0.115955	−2.330127
H	0.931902	1.739935	−1.757090
H	3.313438	2.324404	−1.842545
H	4.995326	1.071085	−0.536267
H	3.787005	2.030584	−4.250382
H	2.079132	2.439761	−4.018124
H	2.548302	0.765035	−4.359093
H	4.870138	0.409530	3.560371
H	6.382297	−0.094083	2.760948
H	5.452553	1.279355	2.110165
H	2.130149	−1.234762	5.459067
H	1.199353	0.094889	4.727930
H	0.899479	−1.592862	4.224337
H	9.513835	−2.163102	−0.523184
H	9.071397	−0.650833	−1.327207
H	8.877380	−0.814792	0.428715
H	7.110211	−2.556165	0.075349
H	7.308542	−2.409597	−1.677536
H	1.040620	3.605250	−0.277858
H	−0.442094	4.577384	−0.029572
H	0.882035	4.638709	1.160942
H	−4.502803	3.213992	1.412918
H	−3.076909	3.423634	2.473390
H	−3.845662	4.842965	1.719385
H	−5.552131	3.014459	−2.085152
H	−4.024746	3.801948	−1.607483
H	−4.015955	2.479019	−2.810683

ωB97X Energy = −2096.10516780 a.u.

(*aR*,1*S*,3*S*,1’*S*,3’*S*)-**22**, Conf P

C	−1.013803	2.098577	0.587576
C	−2.313337	2.475605	0.233352
C	−3.104197	1.575624	−0.477117
C	−2.634732	0.297733	−0.775255
C	−1.349265	−0.078211	−0.399659
C	−0.529767	0.831846	0.278332
O	−2.784325	−1.692240	−2.153935
C	−1.937920	−2.380581	−1.233217
C	−0.830725	−1.448323	−0.767973
C	−3.533503	−0.649079	−1.542649
C	−1.407018	−3.610327	−1.933904
C	−4.682846	−1.224512	−0.722151
C	0.865402	0.466801	0.669094
C	1.926035	0.663184	−0.211545
C	3.206623	0.273313	0.182787
C	3.434273	−0.259723	1.444573
C	2.372501	−0.417011	2.342772
C	1.091650	−0.073960	1.931357
C	−5.707103	−1.879504	−1.403956
C	−6.765838	−2.459823	−0.725324
C	−6.782113	−2.373353	0.653761
C	−5.791038	−1.734665	1.366650
C	−4.738292	−1.159583	0.664628
C	1.720537	1.301783	−1.563450
C	3.026141	1.832273	−2.129517
O	4.014075	0.800548	−2.060831
C	4.392557	0.494193	−0.734521
C	2.914588	2.255130	−3.575681
F	−7.813871	−2.934617	1.327941
O	4.732491	−0.535378	1.790470
C	5.023725	−1.884389	2.175332

O	2.679745	−0.881800	3.580965
C	1.622718	−1.019116	4.519623
C	5.259515	−0.755710	−0.874249
O	4.822713	−1.863710	−1.065556
O	6.552546	−0.459369	−0.827599
C	8.872341	−0.996491	−0.869683
C	7.478475	−1.556049	−0.999363
O	−0.180175	3.007709	1.184522
C	−0.418312	3.221500	2.573996
O	−2.733464	3.743197	0.530803
C	−3.869955	3.831381	1.395443
C	−4.431917	2.913827	−1.895853
O	−4.373673	1.911612	−0.875857
H	−2.539387	−2.687400	−0.366797
H	−0.313321	−1.896228	0.083961
H	−0.086947	−1.349513	−1.567801
H	−3.972358	−0.101821	−2.379366
H	−0.731027	−4.158438	−1.274926
H	−2.224578	−4.273266	−2.220513
H	−0.856870	−3.324420	−2.833441
H	0.250942	−0.206781	2.600511
H	−5.674266	−1.938713	−2.486884
H	−7.568181	−2.966913	−1.246983
H	−5.843990	−1.688094	2.447407
H	−3.954579	−0.651352	1.215453
H	1.299757	0.574810	−2.268370
H	1.000746	2.119981	−1.482458
H	3.370278	2.678933	−1.518514
H	5.018295	1.300694	−0.331592
H	3.864895	2.649012	−3.937936
H	2.153982	3.030707	−3.681369
H	2.628349	1.402872	−4.196064
H	6.108126	−1.946895	2.251863
H	4.569029	−2.123030	3.135356
H	4.671637	−2.579593	1.409214
H	2.081319	−1.383537	5.435384
H	1.139687	−0.057053	4.711692
H	0.878034	−1.741010	4.174110
H	9.599272	−1.800738	−0.991735
H	9.062023	−0.242777	−1.635016
H	9.020200	−0.545527	0.112475
H	7.267737	−2.307857	−0.236446
H	7.306605	−2.004274	−1.979340
H	−1.414833	3.634921	2.744475
H	−0.305452	2.286405	3.130965
H	0.329902	3.935747	2.911875
H	−4.766532	3.438454	0.914313
H	−3.686730	3.283086	2.324121
H	−4.001384	4.888166	1.618158
H	−5.485061	3.051927	−2.130998
H	−4.003658	3.855241	−1.546128
H	−3.898675	2.577102	−2.789230

ωB97X Energy = −2096.10514261 a.u.

(*aR*,1*S*,3*S*,1'*S*,3'*S*)-**22**, Conf Q

C	−0.919794	2.213484	0.107957
C	−2.209626	2.486005	−0.353378
C	−3.003574	1.441425	−0.813538
C	−2.504378	0.138872	−0.848357
C	−1.211323	−0.123408	−0.408061
C	−0.415496	0.918477	0.086178
O	−2.636646	−2.118478	−1.744156
C	−1.754780	−2.565239	−0.715043
C	−0.664766	−1.529052	−0.492385
C	−3.390235	−0.964575	−1.388820
C	−1.200772	−3.904423	−1.146326
C	−4.543537	−1.330363	−0.464032
C	0.970585	0.660298	0.580319
C	2.054346	0.675400	−0.294309
C	3.326067	0.397221	0.209425
C	3.513823	0.141489	1.558565
C	2.425000	0.120976	2.435340
C	1.157928	0.382793	1.932367
C	−4.474692	−1.184674	0.917801
C	−5.529691	−1.573873	1.733102
C	−6.654270	−2.105237	1.138213
C	−6.765776	−2.263569	−0.229324
C	−5.698414	−1.872862	−1.022637
C	1.888229	1.011653	−1.755733
C	3.209512	1.427029	−2.379513
O	4.199471	0.441653	−2.070009
C	4.539710	0.417466	−0.698993
C	3.142498	1.542271	−3.884372
F	−7.691431	−2.480599	1.922957
O	4.779494	−0.158283	1.997790
C	5.397486	0.865186	2.781944
O	2.698957	−0.166263	3.732431
C	1.613187	−0.190825	4.647969
C	5.452344	−0.800629	−0.555341
O	6.656236	−0.722494	−0.545579
O	4.767038	−1.936024	−0.516213
C	5.993801	−3.601895	−1.815792
C	5.520735	−3.168001	−0.446834
O	−0.116233	3.250332	0.505789
C	−0.417193	3.764413	1.803747
O	−2.683205	3.772176	−0.347211
C	−2.385236	4.487404	−1.548185
C	−5.230626	2.146102	−0.396804
O	−4.250330	1.678983	−1.329154
H	−2.327953	−2.692524	0.212798
H	−0.112694	−1.773530	0.418279
H	0.054207	−1.585480	−1.318647
H	−3.823947	−0.621827	−2.329633
H	−0.492241	−4.276530	−0.403888
H	−2.002347	−4.635703	−1.258379
H	−0.680756	−3.805334	−2.101958
H	0.294769	0.365356	2.585879
H	−3.591993	−0.749679	1.373873
H	−5.485220	−1.462407	2.809424
H	−7.670065	−2.679605	−0.656041
H	−5.768578	−1.988750	−2.099199

H	1.495439	0.147403	−2.304379
H	1.161642	1.819660	−1.871948
H	3.531290	2.384558	−1.945603
H	5.161917	1.289088	−0.456029
H	4.103201	1.860660	−4.290654
H	2.384755	2.273545	−4.170898
H	2.877683	0.578498	−4.324801
H	6.408868	0.524200	2.993740
H	5.439230	1.804042	2.222040
H	4.854700	1.015778	3.716577
H	2.044458	−0.427536	5.617323
H	1.117097	0.782339	4.698517
H	0.885121	−0.959550	4.375811
H	6.489951	−4.570726	−1.735915
H	5.150468	−3.704231	−2.500421
H	6.703385	−2.886002	−2.231620
H	6.353294	−3.028660	0.243128
H	4.820474	−3.884732	−0.021997
H	−0.265257	2.991942	2.563897
H	0.274112	4.585927	1.981052
H	−1.444362	4.132486	1.847276
H	−2.810269	5.482570	−1.433424
H	−1.304124	4.564380	−1.689877
H	−2.838987	3.992478	−2.410893
H	−4.933668	3.102864	0.033340
H	−6.154649	2.263981	−0.959262
H	−5.380765	1.407567	0.395509

ωB97X Energy = −2096.10512907 a.u.

(*aR*,1*S*,3*S*,1'*S*,3'*S*)-**22**, Conf R

C	−1.028305	2.136692	−0.156470
C	−2.328165	2.369623	−0.621391
C	−3.120319	1.283581	−0.980940
C	−2.631460	−0.018665	−0.859176
C	−1.346289	−0.243204	−0.381271
C	−0.531710	0.844018	−0.036262
O	−2.807149	−2.378574	−1.426268
C	−1.953801	−2.685470	−0.324541
C	−0.835452	−1.658602	−0.248731
C	−3.534663	−1.166562	−1.261242
C	−1.436846	−4.091445	−0.529177
C	−4.720241	−1.367638	−0.326249
C	0.866253	0.633990	0.447558
C	1.904016	0.387963	−0.449834
C	3.190508	0.185761	0.055941
C	3.435614	0.244165	1.417736
C	2.390815	0.475291	2.318558
C	1.112309	0.673939	1.818610
C	−4.679364	−1.035054	1.023888
C	−5.763514	−1.283091	1.856152
C	−6.888715	−1.862744	1.309605
C	−6.973684	−2.202847	−0.026307
C	−5.877565	−1.951130	−0.836442
C	1.688174	0.356546	−1.943807
C	2.988379	0.596060	−2.691798
O	3.973349	−0.318702	−2.203872
C	4.364653	−0.050457	−0.874242
C	2.863263	0.372000	−4.180704
F	−7.953430	−2.102435	2.110860
O	4.712577	0.007852	1.863651
C	5.400088	1.165873	2.343515
O	2.719555	0.484824	3.634228
C	1.679976	0.717465	4.574046
C	5.183216	−1.274405	−0.462948
O	4.705036	−2.354226	−0.221415
O	6.485386	−1.015211	−0.472494
C	8.775970	−1.579027	−0.141343
C	7.363226	−2.104767	−0.112470
O	−0.245522	3.204502	0.206610
C	0.328321	3.919323	−0.888676
O	−2.799969	3.641307	−0.808615
C	−3.004630	4.401467	0.387041
C	−5.351746	2.093434	−0.730902
O	−4.363675	1.440389	−1.529208
H	−2.543370	−2.648499	0.601128
H	−0.299003	−1.775017	0.696240
H	−0.112971	−1.864879	−1.046571
H	−3.936235	−0.952870	−2.253129
H	−0.748118	−4.361120	0.273648
H	−2.259572	−4.807667	−0.535931
H	−0.904090	−4.162123	−1.480300
H	0.284542	0.862898	2.490171
H	−3.794796	−0.565556	1.440483
H	−5.740398	−1.028793	2.908647
H	−7.879706	−2.649574	−0.416816
H	−5.926315	−2.209367	−1.889251
H	1.287436	−0.615656	−2.252777
H	0.951182	1.107005	−2.239098
H	3.340206	1.619592	−2.496618
H	5.025782	0.825764	−0.852305
H	3.814325	0.561019	−4.679800
H	2.111318	1.043396	−4.598911
H	2.560114	−0.657746	−4.382497
H	4.905624	1.569617	3.228659
H	6.407859	0.843971	2.598787
H	5.448979	1.932073	1.564278
H	2.149715	0.685518	5.553833
H	1.221738	1.698237	4.421487
H	0.912795	−0.058966	4.513153
H	9.467164	−2.380596	0.122907
H	9.035812	−1.214856	−1.136184
H	8.902610	−0.766434	0.575486
H	7.079710	−2.459846	0.879986
H	7.215012	−2.918393	−0.824449
H	−0.445068	4.263580	−1.578942
H	0.846505	4.776228	−0.462946
H	1.048132	3.291555	−1.422376
H	−2.059990	4.585651	0.899361
H	−3.449800	5.344762	0.076887
H	−3.691691	3.877888	1.058200
H	−6.310871	1.880602	−1.199590

H	−5.351226	1.689800	0.285552
H	−5.189073	3.170934	−0.705438

ωB97X Energy = −2096.10501745 a.u.

(*aR*,1*S*,3*S*,1’*S*,3’*S*)-**22**, Conf S

C	−0.972853	2.262748	0.451269
C	−2.284594	2.527115	0.052164
C	−3.023986	1.526383	−0.567760
C	−2.476966	0.259390	−0.761651
C	−1.169677	−0.000400	−0.360689
C	−0.404025	1.011534	0.234253
O	−2.531798	−1.885278	−1.898042
C	−1.637957	−2.410632	−0.916742
C	−0.577693	−1.371088	−0.592088
C	−3.329011	−0.814581	−1.406159
C	−1.049841	−3.687074	−1.473800
C	−4.444261	−1.344050	−0.511320
C	1.010240	0.757631	0.643014
C	2.044479	0.817480	−0.288916
C	3.343875	0.525530	0.131612
C	3.611233	0.213514	1.456775
C	2.575863	0.190360	2.398109
C	1.280192	0.445723	1.972751
C	−5.470934	−2.073626	−1.107807
C	−6.497523	−2.618057	−0.353657
C	−6.477559	−2.420410	1.013597
C	−5.481933	−1.705680	1.643663
C	−4.463224	−1.167495	0.866842
C	1.802322	1.194406	−1.730773
C	3.079111	1.685393	−2.389559
O	4.103668	0.709067	−2.186079
C	4.508515	0.605182	−0.835704
C	2.937039	1.886500	−3.880075
F	−7.477594	−2.945255	1.760955
O	4.921212	0.004105	1.799387
C	5.239452	−1.239933	2.432295
O	2.925723	−0.067064	3.684320
C	1.892286	−0.106042	4.657992
C	5.427072	−0.616054	−0.818842
O	6.626850	−0.543486	−0.919921
O	4.749991	−1.756371	−0.760397
C	4.550035	−4.126481	−0.695766
C	5.517932	−2.978553	−0.831576
O	−0.270232	3.231411	1.120127
C	0.238480	4.277250	0.291865
O	−2.832479	3.765815	0.259658
C	−3.481664	3.886165	1.527362
C	−4.481926	2.628293	−2.054016
O	−4.325268	1.751519	−0.934482
H	−2.209420	−2.640635	−0.007448
H	−0.013620	−1.688556	0.288440
H	0.137379	−1.324956	−1.421580
H	−3.798157	−0.392145	−2.297251
H	−0.332063	−4.110506	−0.768611
H	−1.832813	−4.424208	−1.656978
H	−0.532686	−3.485043	−2.414731
H	0.460017	0.426244	2.678873
H	−5.468048	−2.218405	−2.183172
H	−7.302733	−3.180983	−0.809438
H	−5.506420	−1.571987	2.718134
H	−3.678270	−0.597699	1.351774
H	1.430881	0.331629	−2.295838
H	1.033983	1.968146	−1.797743
H	3.397861	2.624454	−1.914425
H	5.141062	1.462142	−0.570818
H	3.871734	2.247052	−4.311023
H	2.154354	2.618498	−4.086802
H	2.667934	0.944864	−4.363684
H	4.700046	−2.061234	1.952221
H	6.311075	−1.381287	2.298863
H	4.993825	−1.212956	3.492599
H	2.380068	−0.330358	5.603259
H	1.383530	0.858738	4.731726
H	1.163652	−0.888307	4.429270
H	5.094461	−5.070106	−0.751829
H	4.030931	−4.085942	0.263408
H	3.809459	−4.107771	−1.496295
H	6.046826	−2.998499	−1.785860
H	6.258237	−2.971329	−0.029393
H	0.989341	3.887210	−0.402280
H	−0.567982	4.759591	−0.264291
H	0.707293	4.999901	0.956735
H	−4.291725	3.156781	1.612026
H	−2.763820	3.741005	2.338747
H	−3.890870	4.893227	1.577164
H	−5.551888	2.714674	−2.231405
H	−4.062363	3.611965	−1.838397
H	−3.999064	2.203701	−2.939068

ωB97X Energy = −2096.10500889 a.u.

(*aR*,1*S*,3*S*,1’*S*,3’*S*)-**22**, Conf T

C	−0.968031	2.079275	0.451436
C	−2.262747	2.470444	0.095387
C	−3.085685	1.555100	−0.556403
C	−2.652462	0.251791	−0.793435
C	−1.370060	−0.135790	−0.419280
C	−0.518839	0.787446	0.199293
O	−2.886398	−1.813827	−2.045950
C	−2.031429	−2.465895	−1.107208
C	−0.891268	−1.535214	−0.725503
C	−3.592434	−0.714126	−1.484215
C	−1.546208	−3.746743	−1.746894
C	−4.737868	−1.202073	−0.604005
C	0.873455	0.409793	0.589240
C	1.926591	0.556277	−0.310323
C	3.207816	0.173979	0.090537
C	3.434271	−0.316620	1.366808
C	2.375386	−0.471473	2.266489
C	1.098573	−0.103854	1.864278
C	−4.736061	−1.086155	0.780743

C	−5.787119	−1.582523	1.542348
C	−6.835271	−2.195179	0.890590
C	−6.877200	−2.330230	−0.483910
C	−5.818358	−1.828356	−1.222691
C	1.716609	1.138213	−1.686778
C	3.025189	1.614050	−2.294415
O	3.997306	0.570453	−2.182909
C	4.388711	0.333724	−0.847936
C	2.902511	1.962161	−3.759448
F	−7.866394	−2.679171	1.623023
O	4.706466	−0.706128	1.706995
C	5.381238	0.182060	2.600909
O	2.686748	−0.982670	3.483534
C	1.632853	−1.138613	4.423017
C	5.244019	−0.931419	−0.911989
O	4.802554	−2.032357	−1.125923
O	6.534727	−0.654618	−0.768007
C	8.835298	−1.241076	−0.582144
C	7.444068	−1.776407	−0.807542
O	−0.106133	2.994978	0.996152
C	−0.321109	3.271024	2.378557
O	−2.647894	3.761020	0.335176
C	−3.772508	3.919513	1.205497
C	−4.400451	2.846470	−2.027986
O	−4.352541	1.902732	−0.953291
H	−2.615844	−2.707251	−0.208991
H	−0.359376	−1.948359	0.134845
H	−0.169148	−1.496585	−1.549979
H	−4.035247	−0.206810	−2.343688
H	−0.865423	−4.270993	−1.073634
H	−2.385403	−4.405748	−1.973844
H	−1.013693	−3.526596	−2.675102
H	0.258106	−0.218505	2.537349
H	−3.908050	−0.597905	1.282690
H	−5.795391	−1.495295	2.621837
H	−7.723399	−2.813582	−0.956464
H	−5.832161	−1.925919	−2.303266
H	1.270165	0.389170	−2.351607
H	1.016178	1.975314	−1.634984
H	3.391168	2.485568	−1.733164
H	5.023946	1.157603	−0.497057
H	3.853973	2.321959	−4.152773
H	2.151895	2.742413	−3.897398
H	2.596496	1.082693	−4.330411
H	5.463793	1.179820	2.159936
H	4.856313	0.241084	3.556088
H	6.376773	−0.230408	2.753276
H	2.090178	−1.542456	5.322699
H	1.163562	−0.177903	4.652228
H	0.876762	−1.837217	4.054834
H	9.550306	−2.064607	−0.607933
H	9.107900	−0.525189	−1.358677
H	8.910691	−0.751372	0.389827
H	7.147341	−2.484558	−0.031626
H	7.346371	−2.267793	−1.776942
H	−1.306689	3.712025	2.543947
H	−0.220675	2.357375	2.972369
H	0.445861	3.982718	2.677508
H	−4.685691	3.536573	0.748089
H	−3.597494	3.402104	2.153268
H	−3.868286	4.987800	1.387804
H	−3.935116	3.791705	−1.741530
H	−3.897298	2.441931	−2.910611
H	−5.453391	3.005620	−2.250464

ωB97X Energy = −2096.10499030 a.u.

(*aR*,1*S*,3*S*,1’*S*,3’*S*)-**22**, Conf U

C	−0.969590	2.266028	0.094925
C	−2.284623	2.477183	−0.336513
C	−3.019305	1.395729	−0.812938
C	−2.457051	0.117708	−0.841858
C	−1.155288	−0.086220	−0.401197
C	−0.398028	0.999563	0.060217
O	−2.512153	−2.172284	−1.663616
C	−1.632235	−2.548269	−0.605180
C	−0.567490	−1.477595	−0.430883
C	−3.301457	−1.026396	−1.364771
C	−1.046641	−3.896355	−0.959856
C	−4.467353	−1.393586	−0.455910
C	1.018641	0.818901	0.498556
C	2.049294	0.726447	−0.435188
C	3.353693	0.527326	0.023430
C	3.628652	0.454304	1.381254
C	2.596229	0.582061	2.317625
C	1.296442	0.744672	1.860917
C	−5.585358	−2.000634	−1.023237
C	−6.660522	−2.398619	−0.244471
C	−6.594606	−2.181272	1.117944
C	−5.507824	−1.584804	1.721023
C	−4.443866	−1.189967	0.919802
C	1.797726	0.846941	−1.919358
C	3.065936	1.218422	−2.667050
O	4.102216	0.307263	−2.293243
C	4.513856	0.465356	−0.950390
C	2.912805	1.136653	−4.167865
F	−7.640117	−2.563334	1.888805
O	4.942337	0.330349	1.750084
C	5.284115	−0.781017	2.585188
O	2.954255	0.557839	3.626904
C	1.929557	0.711206	4.598484
C	5.479278	−0.695964	−0.720176
O	6.675504	−0.593855	−0.836707
O	4.849008	−1.835230	−0.460660
C	4.757080	−4.164868	0.026098
C	5.669606	−3.017207	−0.324556
O	−0.248858	3.331444	0.575497
C	0.320201	4.163611	−0.435666
O	−2.824213	3.734753	−0.380927
C	−3.063127	4.345161	0.891632
C	−5.286316	2.065630	−0.473921
O	−4.275183	1.539793	−1.334924

H	−2.211884	−2.638212	0.323169
H	−0.008529	−1.665796	0.489190
H	0.149531	−1.558469	−1.255708
H	−3.721688	−0.726369	−2.326201
H	−0.338617	−4.214424	−0.192248
H	−1.832218	−4.648826	−1.040823
H	−0.518638	−3.837297	−1.914475
H	0.478923	0.840990	2.563759
H	−5.619772	−2.161828	−2.095751
H	−7.536753	−2.864182	−0.678547
H	−5.498625	−1.429579	2.792843
H	−3.589408	−0.707702	1.382222
H	1.425021	−0.101386	−2.322929
H	1.025037	1.594359	−2.114663
H	3.378353	2.232677	−2.378482
H	5.114667	1.378787	−0.852882
H	3.842239	1.416109	−4.665238
H	2.123152	1.811932	−4.501862
H	2.648322	0.119295	−4.464655
H	6.355567	−0.933423	2.462067
H	5.052980	−0.572269	3.628395
H	4.748155	−1.678323	2.264113
H	2.425113	0.669001	5.565123
H	1.425330	1.675056	4.489365
H	1.195746	−0.096283	4.530968
H	5.344426	−5.078774	0.125172
H	4.245799	−3.981868	0.972749
H	4.008768	−4.319259	−0.752369
H	6.192255	−3.182958	−1.268053
H	6.414555	−2.835241	0.452413
H	1.101582	3.625725	−0.981019
H	−0.446515	4.510704	−1.132225
H	0.763189	5.016633	0.074517
H	−3.552180	5.295764	0.688168
H	−3.724039	3.718167	1.497328
H	−2.127134	4.514513	1.424228
H	−5.247777	1.578370	0.504429
H	−5.182843	3.144393	−0.358042
H	−6.238924	1.838653	−0.949220

ωB97X Energy = −2096.10497249 a.u.

(*aR*,1*S*,3*S*,1'*S*,3'*S*)-**22**, Conf V

C	−0.999183	2.069466	0.605411
C	−2.302655	2.448224	0.277525
C	−3.118283	1.555967	−0.410152
C	−2.657775	0.281621	−0.734011
C	−1.358617	−0.093202	−0.402582
C	−0.515660	0.812129	0.254699
O	−2.877721	−1.732541	−2.069686
C	−1.991133	−2.405489	−1.175661
C	−0.860453	−1.468171	−0.782152
C	−3.592987	−0.672732	−1.446320
C	−1.496990	−3.650697	−1.875934
C	−4.707262	−1.223847	−0.563163
C	0.891852	0.447672	0.599573
C	1.909853	0.554879	−0.345989
C	3.209879	0.204702	0.027995
C	3.485595	−0.226112	1.315053
C	2.459425	−0.352320	2.257477
C	1.168450	−0.005357	1.887956
C	−5.790340	−1.842267	−1.184967
C	−6.819958	−2.401509	−0.445915
C	−6.745701	−2.332185	0.932034
C	−5.693780	−1.729378	1.587034
C	−4.672737	−1.174380	0.825190
C	1.655320	1.054959	−1.747850
C	2.934259	1.556257	−2.396277
O	3.937324	0.543803	−2.279095
C	4.362299	0.345143	−0.947737
C	2.769966	1.860390	−3.867049
F	−7.747745	−2.873136	1.665067
O	4.773323	−0.588982	1.623539
C	5.462322	0.320383	2.484384
O	2.818065	−0.813120	3.481794
C	1.797068	−0.954039	4.459479
C	5.226918	−0.914843	−1.004003
O	4.784210	−2.027665	−1.137028
O	6.520523	−0.611110	−0.965781
C	7.667125	−2.283646	0.383502
C	7.461332	−1.708740	−0.999618
O	−0.215797	2.924824	1.335162
C	0.317613	4.021787	0.593646
O	−2.751870	3.696193	0.619878
C	−3.596661	3.703858	1.773360
C	−4.541877	2.898791	−1.727317
O	−4.411232	1.888775	−0.721873
H	−2.551566	−2.693406	−0.276056
H	−0.297977	−1.904980	0.046707
H	−0.162715	−1.383436	−1.623251
H	−4.065519	−0.137846	−2.272880
H	−0.796303	−4.190825	−1.236453
H	−2.329691	−4.313634	−2.114968
H	−0.984440	−3.384748	−2.803360
H	0.355310	−0.080964	2.598514
H	−5.829845	−1.887909	−2.268361
H	−7.668015	−2.879413	−0.920669
H	−5.675875	−1.695050	2.669398
H	−3.842357	−0.693339	1.330214
H	1.244629	0.251665	−2.370433
H	0.911829	1.855468	−1.737595
H	3.285978	2.453415	−1.866545
H	5.000048	1.182210	−0.634732
H	3.704618	2.228496	−4.291676
H	2.000489	2.621299	−4.009019
H	2.470198	0.959245	−4.406600
H	4.959051	0.396018	3.449702
H	6.463111	−0.084939	2.622423
H	5.530062	1.309183	2.021312
H	2.287564	−1.331219	5.353265
H	1.326716	0.007779	4.680324
H	1.036148	−1.668129	4.133574

H	8.407304	−3.084367	0.337736
H	8.033592	−1.517146	1.068595
H	6.736797	−2.691953	0.779646
H	7.098100	−2.461005	−1.699534
H	8.376014	−1.267781	−1.391319
H	−0.481941	4.602565	0.129543
H	0.857317	4.645020	1.303891
H	1.013183	3.665209	−0.172445
H	−4.498258	3.113035	1.594755
H	−3.060084	3.310501	2.640835
H	−3.868283	4.741985	1.953574
H	−5.608848	3.032680	−1.892242
H	−4.096946	3.838100	−1.395615
H	−4.067626	2.571140	−2.656893

ωB97X Energy = −2096.10496600 a.u.

(*aR*,1*S*,3*S*,1’*S*,3’*S*)-**22**, Conf W

C	−1.019715	2.039490	0.657437
C	−2.311322	2.444666	0.302288
C	−3.138245	1.547423	−0.368005
C	−2.663508	0.290284	−0.742566
C	−1.366018	−0.091995	−0.421267
C	−0.537312	0.788176	0.287248
O	−2.881908	−1.691872	−2.134354
C	−1.988028	−2.384337	−1.263509
C	−0.862317	−1.452052	−0.844753
C	−3.594053	−0.637618	−1.496806
C	−1.486843	−3.602536	−2.005648
C	−4.732816	−1.185579	−0.647172
C	0.859322	0.405031	0.656474
C	1.898273	0.503258	−0.267348
C	3.184928	0.131742	0.131786
C	3.427781	−0.310917	1.421358
C	2.381295	−0.425097	2.342572
C	1.103347	−0.058504	1.947854
C	−4.610364	−1.407576	0.720875
C	−5.654452	−1.956282	1.454154
C	−6.823448	−2.270130	0.793659
C	−6.989047	−2.061190	−0.561369
C	−5.931048	−1.518603	−1.273959
C	1.683440	1.020004	−1.669847
C	2.984051	1.508480	−2.284170
O	3.968047	0.478660	−2.156285
C	4.360287	0.257939	−0.818738
C	2.856917	1.830314	−3.754870
F	−7.850325	−2.797469	1.500727
O	4.703660	−0.692791	1.755194
C	5.390414	0.214145	2.620998
O	2.708590	−0.894520	3.572149
C	1.667048	−1.019036	4.530274
C	5.197287	−1.020368	−0.867207
O	4.736986	−2.120390	−1.042692
O	6.494149	−0.757675	−0.757008
C	8.788808	−1.372064	−0.587881
C	7.388314	−1.891953	−0.790938
O	−0.239879	2.871477	1.418645
C	0.396605	3.927669	0.704715
O	−2.733126	3.690300	0.680586
C	−3.080832	4.575266	−0.388311
C	−5.328738	2.116410	0.348092
O	−4.416604	1.886907	−0.730427
H	−2.541707	−2.708323	−0.372685
H	−0.294033	−1.910915	−0.031913
H	−0.169459	−1.338708	−1.686295
H	−4.042158	−0.075740	−2.317952
H	−0.780675	−4.158222	−1.385822
H	−2.315373	−4.263293	−2.264242
H	−0.978834	−3.302298	−2.925013
H	0.274750	−0.125417	2.641248
H	−3.693268	−1.139513	1.234228
H	−5.567857	−2.131947	2.519269
H	−7.927085	−2.314771	−1.039608
H	−6.043871	−1.345539	−2.339188
H	1.277194	0.228980	−2.310348
H	0.950713	1.830537	−1.669409
H	3.338593	2.394304	−1.737673
H	5.009302	1.077599	−0.483718
H	3.805833	2.189274	−4.154970
H	2.101480	2.603489	−3.905648
H	2.556457	0.939171	−4.310422
H	4.877545	0.294730	3.580971
H	6.387422	−0.196042	2.769525
H	5.468041	1.201804	2.157022
H	2.134727	−1.403206	5.433254
H	1.207412	−0.049966	4.741742
H	0.901540	−1.721543	4.190196
H	9.492823	−2.205063	−0.612206
H	9.061631	−0.668703	−1.375709
H	8.881616	−0.872389	0.377473
H	7.091238	−2.585976	−0.002602
H	7.273428	−2.394527	−1.752737
H	1.092803	3.529428	−0.039902
H	−0.336516	4.572082	0.214207
H	0.951948	4.509177	1.437927
H	−3.235824	5.552522	0.064079
H	−2.266760	4.634717	−1.116356
H	−3.991778	4.250813	−0.893754
H	−5.034097	2.986397	0.937761
H	−6.300530	2.291338	−0.108903
H	−5.383147	1.235435	0.992632

ωB97X Energy = −2096.10488537 a.u.

(*aR*,1*S*,3*S*,1’*S*,3’*S*)-**22**, Conf X

C	−1.010631	2.003476	0.763356
C	−2.300234	2.442606	0.443095
C	−3.137630	1.601351	−0.285810
C	−2.690294	0.343624	−0.696904
C	−1.400649	−0.075710	−0.391331
C	−0.551763	0.761318	0.343297
O	−2.914040	−1.539719	−2.211894

C	−2.044181	−2.302562	−1.376103
C	−0.910818	−1.417034	−0.884712
C	−3.626731	−0.533151	−1.501649
C	−1.553821	−3.481508	−2.185573
C	−4.758325	−1.151490	−0.689202
C	0.841267	0.338964	0.680019
C	1.886405	0.578552	−0.209089
C	3.166808	0.136091	0.126647
C	3.402438	−0.500181	1.334859
C	2.352684	−0.744054	2.225550
C	1.075397	−0.320093	1.884639
C	−5.852444	−1.672087	−1.377542
C	−6.897342	−2.286663	−0.706979
C	−6.826928	−2.372993	0.670150
C	−5.764960	−1.869989	1.390001
C	−4.729215	−1.256009	0.696241
C	1.670522	1.320260	−1.505509
C	2.979945	1.851997	−2.062574
O	3.936365	0.788828	−2.095141
C	4.338090	0.378445	−0.805521
C	2.849082	2.386671	−3.469506
F	−7.843542	−2.970252	1.336740
O	4.673061	−0.944160	1.608101
C	5.372856	−0.175337	2.589181
O	2.672732	−1.395143	3.371463
C	1.629481	−1.645662	4.302237
C	5.150896	−0.894957	−1.039900
O	4.669186	−1.952167	−1.360841
O	6.453242	−0.672996	−0.906191
C	8.739650	−1.339655	−0.871710
C	7.324244	−1.810820	−1.089563
O	−0.170161	2.829082	1.467250
C	−0.453459	2.905804	2.865686
O	−2.775266	3.642892	0.901032
C	−2.154826	4.803920	0.338907
C	−4.661853	3.152914	−1.281403
O	−4.430550	1.928557	−0.581818
H	−2.617147	−2.667978	−0.513376
H	−0.364351	−1.932557	−0.091180
H	−0.198369	−1.261709	−1.703925
H	−4.081814	0.081757	−2.280407
H	−0.862131	−4.084082	−1.593880
H	−2.389516	−4.112621	−2.491084
H	−1.032254	−3.133935	−3.080356
H	0.240776	−0.506081	2.548858
H	−5.888948	−1.594414	−2.459254
H	−7.753912	−2.689972	−1.232919
H	−5.750994	−1.956961	2.469472
H	−3.891443	−0.849923	1.252385
H	1.212310	0.659649	−2.251018
H	0.978264	2.151298	−1.348907
H	3.363926	2.639588	−1.398566
H	5.004008	1.133289	−0.367329
H	3.802113	2.781088	−3.823901
H	2.109062	3.188338	−3.497780
H	2.524680	1.592719	−4.145824
H	5.467288	0.865282	2.265095
H	4.859606	−0.218550	3.551489
H	6.362895	−0.617811	2.680904
H	2.093814	−2.153445	5.143839
H	1.175070	−0.712453	4.646362
H	0.859962	−2.289891	3.868633
H	9.424964	−2.179670	−0.993535
H	9.009396	−0.569105	−1.595097
H	8.865027	−0.937012	0.134446
H	7.034505	−2.580196	−0.371377
H	7.174721	−2.206030	−2.095556
H	−1.470132	3.265763	3.038054
H	−0.321268	1.926561	3.335323
H	0.261368	3.608941	3.288554
H	−2.655689	5.662875	0.781014
H	−1.090811	4.831860	0.575647
H	−2.291926	4.821908	−0.746176
H	−4.647947	4.004335	−0.601643
H	−3.914871	3.292516	−2.067767
H	−5.646863	3.061264	−1.735258

ωB97X Energy = −2096.10485835 a.u.

(*aR*,1*S*,3*S*,1'*S*,3'*S*)-**22**, Conf Y

C	−1.011403	2.157789	−0.043642
C	−2.322388	2.408669	−0.465388
C	−3.116112	1.339959	−0.870465
C	−2.616942	0.036163	−0.835353
C	−1.319967	−0.206787	−0.400343
C	−0.503387	0.864584	−0.012550
O	−2.790820	−2.288333	−1.533529
C	−1.915163	−2.651768	−0.467101
C	−0.800281	−1.624484	−0.356389
C	−3.521845	−1.092699	−1.285032
C	−1.396477	−4.041580	−0.759059
C	−4.688260	−1.356199	−0.341626
C	0.908036	0.638411	0.422114
C	1.921199	0.438735	−0.514285
C	3.221493	0.216084	−0.054406
C	3.504284	0.213023	1.301310
C	2.484971	0.401414	2.240364
C	1.192357	0.617480	1.786144
C	−4.626053	−1.098335	1.023873
C	−5.692803	−1.403000	1.859880
C	−6.822150	−1.963267	1.301850
C	−6.927945	−2.230175	−0.049123
C	−5.849035	−1.922689	−0.863051
C	1.663991	0.478017	−2.001753
C	2.943911	0.745640	−2.774848
O	3.938100	−0.192463	−2.354182
C	4.368633	0.023031	−1.027136
C	2.776326	0.586113	−4.267867
F	−7.870015	−2.257881	2.107045
O	4.792183	−0.045740	1.700789
C	5.497793	1.084988	2.216685
O	2.850833	0.353979	3.545451

C	1.838038	0.543938	4.523334
C	5.203357	−1.212527	−0.687246
O	4.732811	−2.298023	−0.457850
O	6.504184	−0.947036	−0.753564
C	7.621450	−2.127715	1.058959
C	7.416659	−2.023003	−0.435458
O	−0.229902	3.210744	0.363582
C	0.347726	3.963910	−0.703250
O	−2.803393	3.686561	−0.566989
C	−2.988845	4.369722	0.677397
C	−5.345919	2.120467	−0.528122
O	−4.371929	1.518955	−1.382310
H	−2.486768	−2.669281	0.470311
H	−0.244838	−1.789096	0.570299
H	−0.092710	−1.783271	−1.177898
H	−3.943431	−0.824664	−2.255220
H	−0.693579	−4.352581	0.016068
H	−2.216486	−4.760165	−0.792456
H	−0.878880	−4.056209	−1.721014
H	0.383062	0.772432	2.488144
H	−3.738408	−0.643901	1.450522
H	−5.652954	−1.206772	2.924234
H	−7.836417	−2.664485	−0.447858
H	−5.914218	−2.123457	−1.927420
H	1.249058	−0.476541	−2.344754
H	0.923400	1.245045	−2.240590
H	3.305842	1.758604	−2.546057
H	5.027111	0.900713	−0.990146
H	3.714679	0.791133	−4.784396
H	2.017781	1.278889	−4.636314
H	2.460780	−0.432377	−4.504703
H	5.020781	1.457778	3.124642
H	6.505519	0.741897	2.444307
H	5.544383	1.881006	1.467711
H	2.334800	0.471000	5.487483
H	1.374873	1.529411	4.425523
H	1.070111	−0.230783	4.450845
H	8.334965	−2.925197	1.273619
H	8.020214	−1.194016	1.459474
H	6.682897	−2.353941	1.566014
H	7.025978	−2.949664	−0.855412
H	8.338262	−1.757107	−0.949704
H	−0.422864	4.320569	−1.390645
H	0.852365	4.813459	−0.247440
H	1.079649	3.359607	−1.247694
H	−2.034805	4.527533	1.180981
H	−3.445477	5.327591	0.436224
H	−3.659310	3.800226	1.327696
H	−6.313591	1.929562	−0.988586
H	−5.322588	1.659783	0.463460
H	−5.187101	3.195584	−0.444346

ωB97X Energy = −2096.10485223 a.u.

(*aR*,1*S*,3*S*,1'*S*,3'*S*)-**22**, Conf Z

C	−0.937546	2.264026	0.321899
C	−2.254049	2.520140	−0.075763
C	−2.990218	1.494299	−0.663608
C	−2.450236	0.216024	−0.796167
C	−1.147454	−0.035922	−0.380651
C	−0.382640	0.997520	0.174092
O	−2.503789	−1.961824	−1.869810
C	−1.613697	−2.459568	−0.870943
C	−0.554204	−1.412078	−0.570139
C	−3.300268	−0.872935	−1.418029
C	−1.024327	−3.750782	−1.391657
C	−4.428052	−1.366323	−0.519249
C	1.027269	0.754563	0.604111
C	2.077924	0.879051	−0.301493
C	3.371893	0.581268	0.126901
C	3.620930	0.214453	1.442394
C	2.569920	0.134736	2.361911
C	1.276408	0.383004	1.921917
C	−4.435882	−1.182992	0.858408
C	−5.465427	−1.689006	1.642376
C	−6.483551	−2.378378	1.019880
C	−6.515564	−2.581152	−0.346353
C	−5.477616	−2.069186	−1.107789
C	1.845939	1.339736	−1.719709
C	3.133738	1.829899	−2.358120
O	4.148935	0.839536	−2.173100
C	4.546113	0.708820	−0.822991
C	3.002647	2.070562	−3.843781
F	−7.494474	−2.871386	1.774084
O	4.927941	0.008487	1.798249
C	5.253260	−1.253757	2.389729
O	2.899565	−0.170624	3.643168
C	1.850850	−0.247393	4.597858
C	5.487927	−0.494433	−0.827500
O	6.687750	−0.395375	−0.902562
O	4.833351	−1.649390	−0.820457
C	4.684972	−4.025003	−0.849584
C	5.629248	−2.851725	−0.917320
O	−0.158919	3.284356	0.802156
C	−0.429683	3.664482	2.149438
O	−2.745330	3.789248	0.062118
C	−3.896992	3.920746	0.900937
C	−4.378162	2.558795	−2.244831
O	−4.274155	1.708509	−1.098545
H	−2.188311	−2.664626	0.042369
H	0.005506	−1.706627	0.320927
H	0.165961	−1.385189	−1.396815
H	−3.757545	−0.475101	−2.326159
H	−0.308352	−4.155151	−0.673561
H	−1.806835	−4.492589	−1.557113
H	−0.504747	−3.573790	−2.336260
H	0.442857	0.302786	2.608213
H	−3.633087	−0.632778	1.336762
H	−5.481256	−1.549959	2.716330
H	−7.338733	−3.122679	−0.795916
H	−5.484831	−2.217808	−2.182625
H	1.439915	0.521361	−2.326124

H	1.105539	2.143271	−1.732852
H	3.459697	2.752620	−1.857426
H	5.160047	1.570659	−0.531082
H	3.941254	2.438449	−4.259838
H	2.223764	2.810486	−4.036211
H	2.732583	1.142759	−4.353013
H	4.725640	−2.062983	1.877099
H	6.327263	−1.380506	2.260243
H	4.999255	−1.266633	3.448343
H	1.329682	0.709713	4.688054
H	1.135137	−1.031562	4.337198
H	2.325650	−0.490898	5.544969
H	5.252142	−4.953460	−0.928775
H	4.142664	−4.034093	0.097563
H	3.963035	−3.991468	−1.666501
H	6.182201	−2.824007	−1.857685
H	6.349238	−2.859102	−0.096768
H	−1.443612	4.057441	2.252877
H	−0.292640	2.813909	2.824126
H	0.286058	4.444037	2.402763
H	−4.054921	4.988726	1.035991
H	−4.777062	3.469112	0.441291
H	−3.716259	3.453161	1.873163
H	−3.983951	3.554666	−2.032572
H	−3.838859	2.123335	−3.090609
H	−5.437440	2.624665	−2.483918

ωB97X Energy = −2096.10483125 a.u.

(*aR*,1*S*,3*S*,1'*S*,3'*S*)-**22**, Conf AA

C	−0.902787	2.255562	0.322269
C	−2.209898	2.535551	−0.081683
C	−2.981629	1.522236	−0.640699
C	−2.474945	0.229743	−0.759914
C	−1.172750	−0.045435	−0.353184
C	−0.373350	0.977037	0.174782
O	−2.603791	−1.974443	−1.771080
C	−1.714508	−2.469320	−0.769935
C	−0.622660	−1.443525	−0.512039
C	−3.363668	−0.852125	−1.337720
C	−1.164986	−3.788715	−1.263015
C	−4.489263	−1.291867	−0.408940
C	1.036129	0.706863	0.590011
C	2.063495	0.683780	−0.350973
C	3.360212	0.394508	0.080439
C	3.626263	0.158474	1.419066
C	2.592753	0.172954	2.361513
C	1.303080	0.452573	1.933532
C	−5.569327	−1.975176	−0.963981
C	−6.607232	−2.441369	−0.173483
C	−6.543738	−2.212852	1.187494
C	−5.494816	−1.541076	1.777354
C	−4.466085	−1.080839	0.964646
C	1.822376	0.980535	−1.811297
C	3.108267	1.394509	−2.505005
O	4.108050	0.404881	−2.247242
C	4.519510	0.371466	−0.895789
C	2.962977	1.508389	−4.004425
F	−7.554066	−2.661374	1.969839
O	4.911107	−0.160833	1.782482
C	5.593860	0.853482	2.524291
O	2.940832	−0.100099	3.643793
C	1.910591	−0.105767	4.621812
C	5.389743	−0.881728	−0.798530
O	6.591759	−0.860314	−0.901137
O	4.667795	−1.985747	−0.667619
C	4.374994	−4.332442	−0.403525
C	5.388627	−3.235907	−0.612575
O	−0.160885	3.237084	0.925879
C	0.356367	4.225012	0.034515
O	−2.707861	3.803936	0.058788
C	−3.570304	3.955567	1.188950
C	−4.409726	2.594373	−2.180282
O	−4.276073	1.763558	−1.022781
H	−2.282042	−2.634670	0.155532
H	−0.058891	−1.728031	0.379914
H	0.084957	−1.464113	−1.348922
H	−3.825049	−0.466160	−2.249068
H	−0.450999	−4.192463	−0.542544
H	−1.967867	−4.514342	−1.399952
H	−0.652119	−3.649488	−2.217567
H	0.483119	0.473624	2.639979
H	−5.601310	−2.144639	−2.035284
H	−7.453905	−2.967439	−0.596984
H	−5.486032	−1.380720	2.848412
H	−3.639133	−0.544628	1.417154
H	1.422243	0.096647	−2.321484
H	1.075829	1.770785	−1.920665
H	3.455499	2.351901	−2.089876
H	5.186031	1.219327	−0.689987
H	3.905066	1.814989	−4.460242
H	2.200119	2.248440	−4.252292
H	2.664555	0.547384	−4.429065
H	5.105985	1.022209	3.485550
H	6.607061	0.490185	2.683456
H	5.626005	1.787333	1.955538
H	2.394524	−0.344894	5.565396
H	1.431682	0.874079	4.697055
H	1.157496	−0.865400	4.396007
H	4.884942	−5.296004	−0.362891
H	3.836784	−4.188640	0.534398
H	3.654846	−4.358312	−1.222439
H	5.938158	−3.359743	−1.547327
H	6.108184	−3.183808	0.206299
H	1.066811	3.775847	−0.666668
H	−0.450850	4.713566	−0.514911
H	0.875715	4.957181	0.649610
H	−4.433342	3.290467	1.106711
H	−3.026216	3.746063	2.113785
H	−3.904673	4.991068	1.189952
H	−5.476860	2.689848	−2.369308
H	−3.975173	3.579541	−2.004360

H	−3.927451	2.124360	−3.042210

ωB97X Energy = −2096.10482849 a.u.

(*aR*,1*S*,3*S*,1'*S*,3'*S*)-**22**, Conf AB

C	−0.897886	2.251245	−0.050029
C	−2.208499	2.472296	−0.489546
C	−2.976350	1.384532	−0.893687
C	−2.452784	0.090997	−0.839139
C	−1.156757	−0.122438	−0.386084
C	−0.365279	0.968596	−0.000687
O	−2.573033	−2.243687	−1.515338
C	−1.705138	−2.578666	−0.433514
C	−0.611030	−1.529575	−0.319990
C	−3.329671	−1.059299	−1.290104
C	−1.156445	−3.961611	−0.702556
C	−4.505355	−1.335030	−0.362058
C	1.047100	0.773743	0.445738
C	2.068075	0.580475	−0.483589
C	3.369691	0.383657	−0.015661
C	3.646169	0.402828	1.341290
C	2.619297	0.584801	2.273282
C	1.325094	0.773300	1.811041
C	−5.650520	−1.920491	−0.896695
C	−6.736457	−2.238971	−0.096438
C	−6.653485	−1.963662	1.254432
C	−5.540111	−1.384599	1.825321
C	−4.466075	−1.069092	1.002788
C	1.817233	0.601934	−1.972423
C	3.096527	0.884155	−2.740935
O	4.102336	−0.036532	−2.309689
C	4.523097	0.189730	−0.980269
C	2.939393	0.713313	−4.233812
F	−7.708425	−2.268794	2.046353
O	4.936373	0.171102	1.750026
C	5.614946	1.322546	2.258761
O	2.979351	0.557531	3.580649
C	1.959748	0.746606	4.551583
C	5.412092	−1.010323	−0.653665
O	6.613318	−0.990643	−0.766308
O	4.709202	−2.080598	−0.310765
C	4.467063	−4.334876	0.417021
C	5.454912	−3.282241	−0.017614
O	−0.141426	3.322937	0.355408
C	0.432760	4.077893	−0.712046
O	−2.712976	3.739387	−0.610449
C	−2.928054	4.432168	0.623788
C	−5.225325	2.123097	−0.586647
O	−4.228499	1.534810	−1.423517
H	−2.288767	−2.596436	0.496471
H	−0.064702	−1.673644	0.615540
H	0.109825	−1.684251	−1.130686
H	−3.741213	−0.809285	−2.269388
H	−0.457638	−4.250122	0.084938
H	−1.962089	−4.696168	−0.737767
H	−0.626662	−3.977311	−1.657876
H	0.510096	0.922313	2.507719
H	−5.697909	−2.127331	−1.960846
H	−7.633222	−2.687688	−0.505577
H	−5.518324	−1.182187	2.889049
H	−3.591059	−0.599634	1.439224
H	1.419643	−0.362043	−2.309699
H	1.065594	1.354822	−2.221452
H	3.442401	1.903560	−2.516007
H	5.179666	1.069247	−0.944837
H	3.877293	0.929469	−4.746592
H	2.172453	1.392338	−4.610359
H	2.640687	−0.311267	−4.466218
H	6.630289	1.006988	2.489941
H	5.642219	2.113956	1.504100
H	5.126986	1.689997	3.163006
H	2.453272	0.693267	5.518679
H	1.483913	1.724371	4.438612
H	1.202522	−0.039211	4.486413
H	4.998332	−5.260804	0.641980
H	3.932421	−4.018548	1.313744
H	3.741660	−4.538335	−0.371909
H	6.000376	−3.578296	−0.915398
H	6.179512	−3.054541	0.766053
H	−0.337325	4.414527	−1.409974
H	0.917109	4.940299	−0.258407
H	1.181275	3.482854	−1.243952
H	−3.401591	5.377426	0.365990
H	−3.594427	3.856064	1.272463
H	−1.984182	4.616128	1.137391
H	−5.209806	1.666152	0.406822
H	−5.085294	3.200898	−0.504407
H	−6.182541	1.914910	−1.061232

ωB97X Energy = −2096.10474300 a.u.

(*aR*,1*S*,3*S*,1'*S*,3'*S*)-**22**, Conf AC

C	−0.961305	2.085128	0.516151
C	−2.269604	2.464970	0.199496
C	−3.088385	1.559440	−0.470853
C	−2.636098	0.274673	−0.765960
C	−1.340607	−0.103357	−0.429060
C	−0.494468	0.811649	0.208518
O	−2.854919	−1.744745	−2.093671
C	−1.974110	−2.417369	−1.193897
C	−0.843674	−1.482152	−0.794785
C	−3.570611	−0.680244	−1.478802
C	−1.478898	−3.664783	−1.889513
C	−4.692498	−1.222371	−0.600289
C	0.910305	0.444484	0.561766
C	1.943849	0.628139	−0.353630
C	3.237626	0.254229	0.013161
C	3.496007	−0.261232	1.273288
C	2.456509	−0.456167	2.187655
C	1.166997	−0.098009	1.818795
C	−4.664365	−1.169673	0.788087
C	−5.692614	−1.716331	1.546355

C	−6.745063	−2.314119	0.887684
C	−6.813161	−2.386328	−0.490491
C	−5.776569	−1.835391	−1.225884
C	1.700816	1.241474	−1.710920
C	2.991810	1.751128	−2.328964
O	3.978977	0.717904	−2.263913
C	4.397980	0.449350	−0.942897
C	2.837544	2.137420	−3.781407
F	−7.753999	−2.846977	1.617103
O	4.780673	−0.638555	1.578346
C	5.454177	0.225186	2.496434
O	2.798066	−0.995169	3.384574
C	1.764245	−1.193694	4.338152
C	5.262361	−0.806706	−1.059876
O	4.819936	−1.910404	−1.255305
O	6.555868	−0.507161	−0.994601
C	7.695243	−2.238823	0.282609
C	7.496078	−1.602987	−1.074592
O	−0.104952	2.996385	1.076832
C	−0.297001	3.220303	2.471959
O	−2.673371	3.738369	0.494439
C	−3.782610	3.843172	1.392200
C	−4.455945	2.881907	−1.865020
O	−4.369088	1.896950	−0.830423
H	−2.540006	−2.702563	−0.296733
H	−0.291513	−1.917577	0.041548
H	−0.135318	−1.401099	−1.628021
H	−4.035923	−0.147922	−2.310842
H	−0.781969	−4.204847	−1.245889
H	−2.311537	−4.326825	−2.131245
H	−0.961690	−3.401240	−2.815028
H	0.341083	−0.243583	2.503752
H	−3.833220	−0.692694	1.295675
H	−5.679845	−1.679525	2.628707
H	−7.661947	−2.860054	−0.968115
H	−5.811133	−1.883268	−2.309354
H	1.253306	0.503390	−2.387175
H	0.989565	2.066664	−1.625180
H	3.356844	2.611845	−1.750666
H	5.033556	1.268852	−0.582741
H	3.777179	2.519540	−4.182017
H	2.075417	2.911918	−3.883995
H	2.531364	1.270205	−4.370730
H	4.938116	0.245422	3.457867
H	6.455199	−0.182585	2.625479
H	5.522875	1.238216	2.089172
H	2.243855	−1.616169	5.217460
H	1.285503	−0.247105	4.603757
H	1.011597	−1.892747	3.963823
H	8.436136	−3.036487	0.205277
H	8.057670	−1.503125	1.002669
H	6.763041	−2.664455	0.655568
H	7.135160	−2.323040	−1.808660
H	8.412756	−1.145872	−1.442378
H	−0.168837	2.288383	3.030927
H	0.463593	3.934637	2.780771
H	−1.286366	3.637805	2.671770
H	−4.695933	3.452172	0.941901
H	−3.575260	3.301881	2.319928
H	−3.899618	4.902894	1.608836
H	−5.515585	3.024166	−2.066002
H	−4.008931	3.825808	−1.546799
H	−3.956516	2.525699	−2.770298

ωB97X Energy = −2096.10473443 a.u.

(*aR*,1*S*,3*S*,1’*S*,3’*S*)-**22**, Conf AD

C	−1.005341	2.017437	0.764370
C	−2.305941	2.423927	0.446408
C	−3.130736	1.552250	−0.259552
C	−2.645508	0.322365	−0.704941
C	−1.338707	−0.058316	−0.421301
C	−0.511408	0.794905	0.321000
O	−2.855559	−1.587770	−2.195286
C	−1.942253	−2.312308	−1.371991
C	−0.822972	−1.387641	−0.921108
C	−3.573347	−0.577627	−1.495648
C	−1.436175	−3.484592	−2.181797
C	−4.694546	−1.185045	−0.663086
C	0.897258	0.414413	0.643930
C	1.915878	0.579125	−0.293208
C	3.215144	0.204134	0.058764
C	3.490232	−0.305434	1.316663
C	2.464084	−0.487361	2.249794
C	1.173558	−0.117138	1.902157
C	−5.890726	−1.514035	−1.296030
C	−6.932730	−2.107674	−0.600826
C	−6.753457	−2.371128	0.742957
C	−5.585921	−2.063470	1.408806
C	−4.557794	−1.463587	0.693108
C	1.666100	1.167615	−1.661315
C	2.947648	1.708799	−2.271556
O	3.947497	0.687646	−2.221685
C	4.368796	0.396437	−0.906156
C	2.787176	2.112302	−3.718677
F	−7.765041	−2.948118	1.433167
O	4.777256	−0.689552	1.601701
C	5.469618	0.166454	2.513227
O	2.822449	−1.023386	3.442973
C	1.801963	−1.221884	4.411105
C	5.217007	−0.868557	−1.044391
O	4.759149	−1.966891	−1.234354
O	6.514580	−0.583019	−1.003252
C	7.656501	−2.329496	0.250258
C	7.441033	−1.689017	−1.102255
O	−0.227053	2.820024	1.558288
C	0.408076	3.902514	0.883906
O	−2.737067	3.644233	0.891149
C	−3.100442	4.578168	−0.129550
C	−5.317495	2.063748	0.510404
O	−4.417040	1.894977	−0.589050
H	−2.480119	−2.687913	−0.491804

H	−0.237015	−1.879432	−0.140715
H	−0.143762	−1.221703	−1.765102
H	−4.038442	0.019582	−2.281654
H	−0.712521	−4.059929	−1.601331
H	−2.259319	−4.143740	−2.460849
H	−0.947041	−3.130590	−3.092209
H	0.360100	−0.235747	2.606429
H	−6.014431	−1.298154	−2.352200
H	−7.869143	−2.358827	−1.083510
H	−5.488358	−2.282394	2.464936
H	−3.642046	−1.200488	1.211387
H	1.259265	0.406213	−2.336435
H	0.921711	1.965406	−1.603568
H	3.300436	2.567312	−1.681687
H	5.016935	1.202881	−0.539103
H	3.723470	2.506136	−4.115656
H	2.019651	2.882824	−3.810156
H	2.486508	1.250581	−4.318759
H	4.967094	0.187257	3.481665
H	6.469378	−0.248938	2.626827
H	5.539823	1.180243	2.108121
H	2.292649	−1.652441	5.280339
H	1.332519	−0.274803	4.689873
H	1.040158	−1.914502	4.043481
H	8.388596	−3.133850	0.159476
H	8.037260	−1.598471	0.965628
H	6.726626	−2.747211	0.637660
H	7.059110	−2.403476	−1.831222
H	8.356131	−1.241306	−1.485202
H	1.113679	3.531914	0.133833
H	−0.326015	4.556321	0.407014
H	0.952430	4.464893	1.639956
H	−3.261779	5.530330	0.371522
H	−2.292027	4.682242	−0.858935
H	−4.011700	4.270377	−0.644884
H	−5.363186	1.148950	1.106714
H	−5.017851	2.901241	1.142959
H	−6.294549	2.260468	0.073998

ωB97X Energy = −2096.10472808 a.u.

(*aR*,1*S*,3*S*,1’*S*,3’*S*)-**22**, Conf AE

C	−0.944354	2.184569	0.634392
C	−2.251587	2.529339	0.273246
C	−3.037591	1.590019	−0.389797
C	−2.522022	0.330050	−0.699373
C	−1.215445	0.003382	−0.354416
C	−0.418552	0.937929	0.317894
O	−2.627180	−1.662637	−2.078737
C	−1.728531	−2.319612	−1.185703
C	−0.651273	−1.343833	−0.740033
C	−3.402208	−0.653426	−1.441564
C	−1.161350	−3.519420	−1.909737
C	−4.500934	−1.279831	−0.590485
C	0.988628	0.614628	0.701405
C	2.037624	0.846391	−0.184840
C	3.330869	0.486610	0.196643
C	3.580277	−0.049425	1.452585
C	2.530443	−0.243397	2.356592
C	1.237902	0.069707	1.957608
C	−5.532331	−1.951632	−1.244142
C	−6.544506	−2.578018	−0.535642
C	−6.505947	−2.520751	0.844396
C	−5.505862	−1.866375	1.530210
C	−4.501377	−1.244320	0.798478
C	1.804675	1.486090	−1.531902
C	3.092740	2.044920	−2.110577
O	4.103890	1.035437	−2.052779
C	4.503375	0.738326	−0.729916
C	2.957414	2.466656	−3.555010
F	−7.491588	−3.127464	1.547852
O	4.886869	−0.303994	1.777682
C	5.207676	−1.634450	2.197440
O	2.860972	−0.716269	3.585658
C	1.815530	−0.895353	4.530165
C	5.455765	−0.445772	−0.887669
O	6.654289	−0.326065	−0.953377
O	4.812099	−1.598827	−1.023396
C	4.692477	−3.960435	−1.303222
C	5.619065	−2.771582	−1.272917
O	−0.153774	3.106338	1.273794
C	−0.453508	3.280121	2.660101
O	−2.792596	3.736831	0.626244
C	−2.223777	4.877936	−0.024541
C	−4.611180	2.948057	−1.562952
O	−4.343585	1.829267	−0.715319
H	−2.293375	−2.659339	−0.307158
H	−0.097153	−1.773226	0.098194
H	0.070221	−1.211291	−1.555409
H	−3.887923	−0.121875	−2.262264
H	−0.446969	−4.042808	−1.271350
H	−1.955689	−4.215087	−2.183777
H	−0.645206	−3.202178	−2.818876
H	0.405492	−0.092613	2.630572
H	−5.543591	−1.986564	−2.328608
H	−7.352572	−3.098119	−1.035193
H	−5.515288	−1.843680	2.613006
H	−3.711584	−0.722408	1.327454
H	1.390605	0.753459	−2.234962
H	1.069208	2.289240	−1.440354
H	3.425068	2.898197	−1.502183
H	5.111121	1.561431	−0.332430
H	3.896047	2.879010	−3.926879
H	2.181042	3.227634	−3.651830
H	2.681080	1.609539	−4.173195
H	4.682568	−2.366277	1.577441
H	6.282437	−1.744461	2.059171
H	4.946459	−1.788021	3.243104
H	1.309343	0.050876	4.740372
H	1.086834	−1.630908	4.179359
H	2.290433	−1.261386	5.436930
H	5.268845	−4.865592	−1.499442

H	4.181421	−4.080304	−0.346311
H	3.943923	−3.851602	−2.089072
H	6.140854	−2.634036	−2.221421
H	6.364934	−2.855498	−0.480331
H	−1.486405	3.607460	2.797865
H	−0.284033	2.347130	3.205698
H	0.226825	4.044052	3.031059
H	−2.786540	5.741283	0.324827
H	−1.170515	4.991122	0.233716
H	−2.329844	4.789221	−1.109604
H	−4.606558	3.880966	−0.999553
H	−3.877541	2.999387	−2.372311
H	−5.600274	2.781208	−1.985377

ωB97X Energy = −2096.10468085 a.u.

(*aR*,1*S*,3*S*,1’*S*,3’*S*)-**22**, Conf AF

C	−1.004712	1.985925	0.850872
C	−2.306222	2.415642	0.567858
C	−3.136577	1.596133	−0.193266
C	−2.671126	0.369317	−0.671582
C	−1.369771	−0.038960	−0.403824
C	−0.527600	0.776178	0.362468
O	−2.881829	−1.441614	−2.274039
C	−1.983358	−2.226308	−1.490325
C	−0.860657	−1.343018	−0.971577
C	−3.602733	−0.486433	−1.503916
C	−1.480913	−3.352612	−2.364700
C	−4.707748	−1.166966	−0.704157
C	0.875804	0.359856	0.661092
C	1.908519	0.670197	−0.220575
C	3.197301	0.221273	0.071260
C	3.453691	−0.491609	1.231739
C	2.416773	−0.803923	2.116239
C	1.131434	−0.372317	1.818080
C	−5.799287	−1.683248	−1.399844
C	−6.820940	−2.349034	−0.742441
C	−6.729779	−2.491469	0.628778
C	−5.669192	−1.995528	1.355526
C	−4.656933	−1.329675	0.675056
C	1.668855	1.493169	−1.462425
C	2.965000	2.079037	−1.995742
O	3.936869	1.035570	−2.107182
C	4.356207	0.542436	−0.852067
C	2.811575	2.701661	−3.363752
F	−7.723913	−3.138620	1.282578
O	4.731242	−0.942212	1.456029
C	5.435936	−0.254185	2.491460
O	2.755794	−1.525865	3.213360
C	1.726620	−1.845028	4.138816
C	5.184486	−0.698363	−1.187824
O	4.709791	−1.738620	−1.568153
O	6.486310	−0.452464	−1.080161
C	7.553303	−2.441365	−0.158539
C	7.392071	−1.543559	−1.364455
O	−0.171881	2.789581	1.588676
C	−0.441138	2.785905	2.991940
O	−2.799167	3.580973	1.092129
C	−2.204868	4.781809	0.588588
C	−4.700017	3.166286	−1.092993
O	−4.438809	1.912898	−0.458142
H	−2.534898	−2.646234	−0.638508
H	−0.291149	−1.886466	−0.213832
H	−0.164420	−1.130445	−1.792035
H	−4.083753	0.156484	−2.243527
H	−0.766015	−3.967057	−1.814273
H	−2.307252	−3.987171	−2.687968
H	−0.982179	−2.949310	−3.249068
H	0.306460	−0.610997	2.477517
H	−5.852706	−1.560881	−2.476673
H	−7.675561	−2.749187	−1.273937
H	−5.638093	−2.127413	2.430069
H	−3.820635	−0.928862	1.237192
H	1.210762	0.877223	−2.245445
H	0.967945	2.302605	−1.243814
H	3.344333	2.827419	−1.285310
H	5.016172	1.274246	−0.368230
H	3.755475	3.130864	−3.701723
H	2.060934	3.493261	−3.332859
H	2.490074	1.948236	−4.086217
H	4.938767	−0.389060	3.453520
H	6.432426	−0.690692	2.528887
H	5.513773	0.812167	2.259808
H	2.205571	−2.403401	4.939193
H	1.270724	−0.939817	4.549391
H	0.955653	−2.465175	3.673861
H	8.273522	−3.228859	−0.387376
H	7.925347	−1.874891	0.696911
H	6.605093	−2.905981	0.113162
H	7.018925	−2.092628	−2.228829
H	8.327930	−1.054371	−1.627700
H	0.262425	3.481783	3.444627
H	−1.463738	3.112803	3.192933
H	−0.282260	1.786485	3.407751
H	−2.721182	5.606507	1.075939
H	−1.140617	4.819388	0.822840
H	−2.346977	4.853096	−0.493625
H	−4.691301	3.982729	−0.371536
H	−3.965768	3.357859	−1.880336
H	−5.689188	3.080011	−1.538748

ωB97X Energy = −2096.10467808 a.u.

(*aS*,3*S*,3’*S*)-**28**, Conf A

C	−1.365688	1.140715	−1.122244
C	−2.739172	1.334514	−1.160280
C	−3.556858	0.546125	−0.342665
C	−2.999447	−0.401989	0.501089
C	−1.615451	−0.582410	0.554071
C	−0.803482	0.188425	−0.273414
C	−1.036223	−1.616179	1.488846

C	−2.046187	−2.068005	2.533719
C	−1.595356	−3.300626	3.284132
O	−3.282501	−2.376458	1.897563
C	1.256403	−1.079794	−0.876765
C	2.640547	−1.256874	−0.887227
C	3.452049	−0.304536	−0.280635
C	2.890816	0.807936	0.340046
C	1.508429	0.969240	0.365096
C	0.681605	0.027485	−0.260495
C	0.921977	2.163431	1.082201
C	1.933879	2.814668	2.013477
C	1.464656	4.153209	2.536716
O	3.154602	3.025035	1.311176
C	3.803442	1.808623	1.010484
C	−3.911927	−1.223681	1.382028
O	4.812290	−0.471294	−0.223860
C	5.480948	−0.330274	−1.478147
O	0.460008	−2.047738	−1.434869
C	0.343655	−1.950964	−2.852838
O	−3.368282	2.251856	−1.941476
C	−2.563870	3.077418	−2.766115
O	−4.917052	0.734228	−0.315669
C	−5.599772	0.285283	−1.485230
O	3.198056	−2.344764	−1.507079
C	3.166218	−3.529906	−0.710443
H	−0.710165	1.735205	−1.746420
H	−0.707917	−2.485245	0.907163
H	−0.148891	−1.215558	1.987903
H	−2.221764	−1.246466	3.245832
H	−2.327543	−3.580257	4.042563
H	−1.475530	−4.136124	2.590808
H	−0.637140	−3.117694	3.774381
H	0.038630	1.859312	1.650563
H	0.582870	2.908364	0.353809
H	2.133585	2.136862	2.857843
H	1.323932	4.851352	1.708389
H	2.196861	4.575404	3.226081
H	0.513854	4.043290	3.061804
H	4.220293	1.368687	1.928096
H	4.642733	2.052240	0.358490
H	−4.284184	−0.598298	2.206763
H	−4.779601	−1.564457	0.817249
H	5.313012	0.669856	−1.889880
H	6.542441	−0.465994	−1.280253
H	5.137787	−1.084652	−2.187900
H	1.323497	−2.042655	−3.328173
H	−0.296236	−2.771777	−3.171167
H	−0.119986	−0.999925	−3.133345
H	−1.995867	2.484715	−3.489147
H	−3.249960	3.732825	−3.297245
H	−1.874861	3.681574	−2.168759
H	−5.275289	0.843592	−2.365097
H	−6.660218	0.458781	−1.311957
H	−5.427658	−0.784264	−1.642913
H	3.723531	−3.380092	0.218403
H	2.134886	−3.812352	−0.485385
H	3.640787	−4.314159	−1.297215

ωB97X Energy = −1498.55731613 a.u.

(*aS*,3*S*,3'*S*)-**28**, Conf B

C	−1.363283	0.969984	−1.274467
C	−2.741564	1.070493	−1.399291
C	−3.554536	0.258422	−0.600624
C	−2.989122	−0.653670	0.277236
C	−1.601611	−0.753229	0.403564
C	−0.793114	0.064599	−0.380853
C	−1.014209	−1.745705	1.376628
C	−2.050808	−2.244951	2.372490
C	−1.569813	−3.439446	3.164730
O	−3.229072	−2.635200	1.674618
C	1.378991	−1.061771	−0.858825
C	2.770897	−1.138785	−0.796998
C	3.478444	−0.132875	−0.147866
C	2.807712	0.935104	0.441339
C	1.418054	0.999219	0.389401
C	0.695904	0.002774	−0.279135
C	0.708839	2.147116	1.070405
C	1.619568	2.873813	2.049531
C	1.030942	4.178988	2.534742
O	2.859327	3.166108	1.413217
C	3.607468	1.996903	1.159836
C	−3.899155	−1.532783	1.104220
O	4.842434	−0.204157	−0.019233
C	5.563730	0.004860	−1.234156
O	0.685805	−2.085863	−1.453143
C	0.590629	−1.969410	−2.871194
O	−3.380485	1.907754	−2.258307
C	−2.581396	2.725798	−3.095607
O	−4.921820	0.300907	−0.721122
C	−5.528363	1.470648	−0.176699
O	3.436671	−2.178763	−1.390710
C	3.460371	−3.365336	−0.596372
H	−0.710641	1.588729	−1.877690
H	−0.607912	−2.599112	0.821803
H	−0.177811	−1.291546	1.916270
H	−2.313891	−1.426566	3.061066
H	−2.323866	−3.752683	3.887834
H	−1.364094	−4.274846	2.491795
H	−0.651526	−3.194131	3.701996
H	−0.177408	1.777670	1.593948
H	0.352749	2.863646	0.321755
H	1.819949	2.216773	2.910050
H	0.881664	4.859257	1.693173
H	1.697276	4.657363	3.253544
H	0.066075	4.005776	3.015269
H	3.996667	1.588297	2.103852
H	4.466626	2.296342	0.558985
H	−4.370851	−0.930980	1.896171
H	−4.697420	−1.934980	0.481386
H	6.620444	−0.067542	−0.984160
H	5.306344	−0.755038	−1.973516

H	5.353115	1.000524	−1.637106
H	0.043144	−2.842270	−3.221509
H	0.040849	−1.062850	−3.143342
H	1.584994	−1.952622	−3.324858
H	−1.957495	3.405826	−2.508034
H	−1.945471	2.121190	−3.748736
H	−3.273812	3.304772	−3.701988
H	−5.191144	2.366295	−0.701921
H	−5.301604	1.560948	0.890500
H	−6.602452	1.353306	−0.307962
H	3.966696	−3.177654	0.354585
H	2.444650	−3.724832	−0.414434
H	4.015817	−4.109935	−1.163494

ωB97X Energy = −1498.55692462 a.u.

(*aS*,3*S*,3’*S*)-**28**, Conf C

C	−1.451474	1.127274	−1.085600
C	−2.831531	1.267122	−1.062796
C	−3.581815	0.437767	−0.221357
C	−2.951447	−0.498168	0.583614
C	−1.560145	−0.623494	0.576423
C	−0.814911	0.189351	−0.273672
C	−0.902426	−1.643960	1.473615
C	−1.850225	−2.146968	2.552627
C	−1.320755	−3.368648	3.269045
O	−3.098002	−2.498385	1.963239
C	1.272769	−0.988987	−0.956224
C	2.664206	−1.109607	−1.034367
C	3.459001	−0.112517	−0.473724
C	2.868381	0.991080	0.143737
C	1.485683	1.089056	0.237627
C	0.674810	0.094165	−0.324279
C	0.880272	2.263361	0.971142
C	1.907238	2.970707	1.842304
C	1.401366	4.288198	2.384610
O	3.072939	3.236799	1.070622
C	3.766643	2.055897	0.730841
C	−3.793902	−1.365912	1.489689
O	4.823163	−0.107786	−0.554454
C	5.525326	−1.279722	−0.147922
O	0.491662	−1.973704	−1.511403
C	0.270912	−1.805574	−2.910849
O	−3.528573	2.167723	−1.804724
C	−2.792555	3.038480	−2.646843
O	−4.945915	0.572862	−0.132986
C	−5.660698	0.102695	−1.274641
O	3.242894	−2.137349	−1.732094
C	3.087592	−3.434247	−1.151794
H	−0.846569	1.753331	−1.729638
H	−0.564154	−2.492761	0.868276
H	−0.012275	−1.213212	1.941573
H	−2.029089	−1.340826	3.281318
H	−2.012450	−3.687874	4.049678
H	−1.192281	−4.190034	2.560580
H	−0.353615	−3.152022	3.727081
H	0.042142	1.924576	1.586359
H	0.471265	2.984652	0.254990
H	2.187353	2.310004	2.677577
H	1.174769	4.970060	1.561858
H	2.150681	4.754497	3.025474
H	0.490764	4.134696	2.966962
H	4.280736	1.653516	1.616412
H	4.534031	2.338968	0.011001
H	−4.156581	−0.766295	2.337465
H	−4.669680	−1.735316	0.956375
H	5.091233	−1.690592	0.768557
H	5.519269	−2.037949	−0.930418
H	6.548814	−0.966585	0.051074
H	−0.345951	−2.642216	−3.233579
H	−0.259188	−0.867699	−3.103010
H	1.218956	−1.814593	−3.455014
H	−2.101182	3.657620	−2.067831
H	−2.235137	2.481501	−3.405572
H	−3.525723	3.676760	−3.134150
H	−5.386946	0.668453	−2.167026
H	−5.464882	−0.961912	−1.438373
H	−6.718206	0.246749	−1.061040
H	3.560941	−3.471346	−0.165725
H	2.033215	−3.698286	−1.064417
H	3.590515	−4.131257	−1.819649

ωB97X Energy = −1498.55590620 a.u.

(*aS*,3*S*,3’*S*)-**28**, Conf D

C	−1.423899	0.761770	−1.438851
C	−2.802702	0.908296	−1.491162
C	−3.581089	0.362523	−0.464014
C	−2.979530	−0.302013	0.593302
C	−1.589975	−0.429891	0.655846
C	−0.817556	0.097590	−0.375013
C	−0.962455	−1.142061	1.830065
C	−1.938758	−1.297374	2.987534
C	−1.436542	−2.251055	4.047874
O	−3.175915	−1.804413	2.499042
C	1.289475	−1.167556	−0.817124
C	2.683135	−1.266906	−0.837069
C	3.449149	−0.216698	−0.339450
C	2.844171	0.944090	0.129785
C	1.456248	1.049653	0.123111
C	0.672875	−0.006758	−0.353098
C	0.814540	2.309908	0.653944
C	1.789344	3.138877	1.477602
C	1.258725	4.520919	1.784462
O	3.007268	3.291567	0.756927
C	3.709897	2.073653	0.638466
C	−3.850866	−0.861671	1.693970
O	4.818747	−0.272729	−0.380837
C	5.406098	−1.208549	0.522702
O	0.497030	−2.163269	−1.317168
C	0.679804	−3.476843	−0.788243
O	−3.472443	1.559791	−2.477891

C	−2.709613	2.132789	−3.526371
O	−4.945252	0.524184	−0.450031
C	−5.641907	−0.246616	−1.427785
O	3.309041	−2.380270	−1.337445
C	3.336625	−2.421731	−2.764460
H	−0.797081	1.170150	−2.221481
H	−0.624418	−2.138569	1.521839
H	−0.074426	−0.600552	2.169390
H	−2.123982	−0.309230	3.436927
H	−2.150438	−2.324075	4.869248
H	−1.295974	−3.246149	3.619605
H	−0.480172	−1.906774	4.446486
H	−0.059386	2.057524	1.261152
H	0.448194	2.919264	−0.179903
H	2.007463	2.608901	2.418019
H	1.096338	5.072720	0.855848
H	1.965161	5.078132	2.400901
H	0.308203	4.453012	2.317145
H	4.145430	1.797725	1.610902
H	4.536087	2.251809	−0.049307
H	−4.227598	−0.037791	2.317541
H	−4.717177	−1.370847	1.271925
H	6.484076	−1.115747	0.405460
H	5.131741	−0.967760	1.554552
H	5.095493	−2.227341	0.284925
H	0.913246	−3.435637	0.279027
H	−0.265755	−3.997311	−0.931634
H	1.479702	−4.007936	−1.305607
H	−2.018229	2.889983	−3.145443
H	−2.148396	1.369132	−4.072489
H	−3.425287	2.603056	−4.196524
H	−6.702599	−0.054109	−1.277243
H	−5.354183	0.050879	−2.437667
H	−5.443222	−1.313925	−1.287069
H	2.322229	−2.458567	−3.171860
H	3.860744	−1.548402	−3.161536
H	3.875248	−3.326533	−3.040045

ωB97X Energy = −1498.55560804 a.u.

(*aS*,3*S*,3'*S*)-**28**, Conf E

C	−1.433658	1.004859	−1.183702
C	−2.811670	1.165084	−1.210360
C	−3.596472	0.421243	−0.321929
C	−3.002516	−0.449959	0.577950
C	−1.613874	−0.594968	0.618685
C	−0.834573	0.130933	−0.277604
C	−0.995639	−1.545507	1.614538
C	−1.976151	−1.936220	2.710644
C	−1.485280	−3.097698	3.544704
O	−3.216619	−2.318864	2.124441
C	1.248645	−1.121856	−0.830458
C	2.639485	−1.254080	−0.872467
C	3.426827	−0.252947	−0.309159
C	2.845173	0.891737	0.227215
C	1.460615	1.022785	0.254238
C	0.654537	0.010601	−0.280554
C	0.851959	2.249756	0.892217
C	1.855261	2.985059	1.768569
C	1.358656	4.344584	2.205289
O	3.064884	3.176518	1.042264
C	3.741339	1.960618	0.809982
C	−3.881224	−1.223590	1.533560
O	4.797595	−0.350996	−0.300113
C	5.311329	−1.351459	0.580885
O	0.458880	−2.142605	−1.292131
C	0.299781	−2.172029	−2.705651
O	−3.474948	2.008862	−2.044790
C	−2.702441	2.800152	−2.931135
O	−4.960027	0.581780	−0.281295
C	−5.653901	0.034573	−1.401304
O	3.176011	−2.393171	−1.408407
C	4.019572	−2.200864	−2.545075
H	−0.802433	1.566378	−1.861133
H	−0.659748	−2.448109	1.091862
H	−0.107723	−1.092165	2.065165
H	−2.157766	−1.065497	3.360080
H	−2.201062	−3.335986	4.332477
H	−1.353276	−3.980469	2.915203
H	−0.525870	−2.855852	4.006303
H	−0.020058	1.965386	1.487529
H	0.491357	2.936803	0.118551
H	2.077595	2.370845	2.655100
H	1.193183	4.980606	1.332770
H	2.086426	4.830499	2.856243
H	0.415572	4.248007	2.746641
H	4.188971	1.595597	1.746764
H	4.558489	2.180643	0.123401
H	−4.256427	−0.545655	2.314306
H	−4.748644	−1.626538	1.010901
H	6.396013	−1.293193	0.515403
H	4.997514	−1.151888	1.609632
H	4.974707	−2.347233	0.283557
H	−0.145538	−1.237656	−3.062503
H	1.257621	−2.338714	−3.205814
H	−0.370929	−2.999541	−2.928908
H	−2.020874	3.456696	−2.382455
H	−2.128995	2.177262	−3.623856
H	−3.411370	3.404387	−3.492138
H	−6.714645	0.197762	−1.219866
H	−5.358710	0.534432	−2.325506
H	−5.460858	−1.039740	−1.485229
H	3.502004	−1.615766	−3.311430
H	4.947215	−1.694891	−2.272783
H	4.237383	−3.193447	−2.934510

ωB97X Energy = −1498.55558051 a.u.

(*aS*,3*S*,3'*S*)-**28**, Conf F

C	−1.456346	0.955859	−1.235563
C	−2.841521	0.996494	−1.300355
C	−3.582967	0.138989	−0.479697

C	−2.939457	−0.756326	0.360820
C	−1.544624	−0.794107	0.428089
C	−0.806996	0.067352	−0.379488
C	−0.874798	−1.767318	1.367103
C	−1.847010	−2.316931	2.400383
C	−1.280266	−3.491324	3.165879
O	−3.032982	−2.757672	1.747499
C	1.392808	−0.964365	−0.935953
C	2.791731	−0.982249	−0.941554
C	3.480936	0.071722	−0.346201
C	2.780519	1.129552	0.235415
C	1.391361	1.126525	0.256101
C	0.686101	0.074981	−0.343772
C	0.664035	2.253740	0.951837
C	1.589011	3.036188	1.871974
C	0.962387	4.314995	2.379188
O	2.771216	3.384964	1.161051
C	3.566147	2.257536	0.864251
C	−3.775297	−1.683616	1.213122
O	4.843311	0.177818	−0.357741
C	5.609283	−0.937518	0.090696
O	0.716107	−2.006572	−1.521650
C	0.547354	−1.859405	−2.930763
O	−3.552573	1.815253	−2.119742
C	−2.827404	2.677215	−2.980192
O	−4.954717	0.118810	−0.541447
C	−5.591355	1.258976	0.030984
O	3.480147	−1.964939	−1.603524
C	3.397649	−3.268356	−1.023318
H	−0.857907	1.609299	−1.858036
H	−0.456501	−2.599327	0.789360
H	−0.036916	−1.280864	1.875390
H	−2.119097	−1.514301	3.103783
H	−1.991723	−3.844393	3.913470
H	−1.059574	−4.312353	2.480079
H	−0.355958	−3.204938	3.671627
H	−0.177888	1.854197	1.524004
H	0.240827	2.942180	0.211951
H	1.871743	2.399676	2.725083
H	0.728889	4.975773	1.541295
H	1.643325	4.836757	3.052677
H	0.037335	4.098353	2.917079
H	4.060642	1.894675	1.777664
H	4.348214	2.594964	0.184633
H	−4.244010	−1.111161	2.028194
H	−4.577296	−2.118232	0.617464
H	6.597453	−0.548932	0.330655
H	5.165678	−1.372228	0.991564
H	5.691788	−1.699425	−0.683814
H	−0.054897	−0.973056	−3.152564
H	1.516917	−1.784077	−3.430092
H	0.023455	−2.747887	−3.278051
H	−3.570977	3.230800	−3.548536
H	−2.209320	3.378174	−2.411477
H	−2.194607	2.108769	−3.667769
H	−5.314958	2.169881	−0.503331
H	−5.327555	1.356518	1.089010
H	−6.663454	1.094171	−0.059219
H	3.806367	−3.261738	−0.008081
H	2.366667	−3.622737	−1.002836
H	4.001567	−3.921474	−1.650683

ωB97X Energy = −1498.55550722 a.u.

(*aS*,3*S*,3’*S*)-**28**, Conf G

C	−1.427006	0.859920	−1.302422
C	−2.809082	0.924924	−1.405919
C	−3.589438	0.134640	−0.554598
C	−2.987941	−0.719257	0.357133
C	−1.596451	−0.781464	0.463313
C	−0.820342	0.012614	−0.376387
C	−0.971969	−1.709245	1.476004
C	−1.980104	−2.168239	2.518909
C	−1.458984	−3.300321	3.374803
O	−3.160558	−2.625292	1.866706
C	1.368229	−1.090497	−0.832886
C	2.764899	−1.129581	−0.803380
C	3.452858	−0.088796	−0.183614
C	2.768540	1.006061	0.334997
C	1.378608	1.045597	0.288817
C	0.671621	−0.008989	−0.300832
C	0.655362	2.219284	0.907680
C	1.560077	3.015519	1.836896
C	0.952106	4.336889	2.249151
O	2.790318	3.290932	1.175699
C	3.557230	2.123575	0.978535
C	−3.864778	−1.573759	1.243909
O	4.824445	−0.094224	−0.100877
C	5.359911	−1.088560	0.774436
O	0.677606	−2.154757	−1.351045
C	0.519093	−2.121257	−2.764536
O	−3.481780	1.706849	−2.291089
C	−2.717341	2.505925	−3.177462
O	−4.959400	0.140627	−0.652167
C	−5.582704	1.321439	−0.152382
O	3.405805	−2.217952	−1.329704
C	4.270825	−1.943353	−2.432556
H	−0.798922	1.462910	−1.946270
H	−0.562674	−2.585400	0.960887
H	−0.132658	−1.213028	1.972348
H	−2.250303	−1.314634	3.160670
H	−2.197072	−3.588669	4.124304
H	−1.237244	−4.169478	2.751573
H	−0.542344	−2.998744	3.885591
H	−0.221431	1.866756	1.457854
H	0.283590	2.886938	0.122396
H	1.776661	2.413136	2.732885
H	0.784178	4.962226	1.369377
H	1.614684	4.869790	2.932165
H	−0.006016	4.175663	2.747036
H	3.969330	1.778989	1.939121
H	4.399188	2.401899	0.345152

H	−4.342158	−0.939789	2.006884
H	−4.660360	−2.029852	0.655695
H	4.953496	−0.966705	1.782635
H	5.136888	−2.093945	0.410434
H	6.436748	−0.933347	0.796673
H	−0.016139	−1.216773	−3.071101
H	1.488332	−2.165059	−3.269638
H	−0.067224	−2.997114	−3.035657
H	−3.434337	3.039838	−3.796543
H	−2.101606	3.227017	−2.631739
H	−2.077100	1.888970	−3.814650
H	−5.344702	1.463837	0.906682
H	−5.270070	2.199085	−0.721521
H	−6.655796	1.177606	−0.264100
H	5.127198	−1.340054	−2.126295
H	4.610158	−2.908725	−2.802902
H	3.726895	−1.421243	−3.225779

ωB97X Energy = −1498.55524483 a.u.

(*aS*,3*S*,3’*S*)-**28**, Conf H

C	−1.413390	0.588807	−1.537563
C	−2.795802	0.618306	−1.654147
C	−3.572831	0.021558	−0.655037
C	−2.967268	−0.615314	0.417180
C	−1.575681	−0.642191	0.534583
C	−0.803562	−0.037139	−0.452960
C	−0.946493	−1.320283	1.727656
C	−1.955480	−1.547231	2.844208
C	−1.429666	−2.459731	3.929086
O	−3.127696	−2.147977	2.305499
C	1.414349	−1.118667	−0.830909
C	2.811182	−1.104832	−0.795937
C	3.469099	−0.009168	−0.244548
C	2.753867	1.085750	0.227523
C	1.363091	1.078867	0.167958
C	0.688027	−0.023130	−0.366753
C	0.599439	2.267431	0.704024
C	1.472514	3.152824	1.581657
C	0.820002	4.479708	1.897321
O	2.699285	3.420951	0.912194
C	3.503344	2.267038	0.798623
C	−3.840678	−1.267307	1.464515
O	4.839385	0.046313	−0.234176
C	5.465395	−0.857625	0.675775
O	0.725411	−2.161716	−1.384284
C	0.988609	−3.467326	−0.869748
O	−3.471567	1.188366	−2.685874
C	−2.710931	1.769089	−3.731768
O	−4.942889	−0.003060	−0.750552
C	−5.574540	1.255123	−0.525209
O	3.545621	−2.151283	−1.292840
C	3.628299	−2.159115	−2.718395
H	−0.787489	1.044917	−2.294132
H	−0.531547	−2.290327	1.429102
H	−0.110669	−0.722541	2.103320
H	−2.233279	−0.575044	3.280933
H	−2.167885	−2.579510	4.723040
H	−1.205132	−3.444136	3.512292
H	−0.515259	−2.049060	4.361803
H	−0.269795	1.926893	1.273856
H	0.211618	2.865033	−0.128580
H	1.698794	2.619568	2.518298
H	0.641694	5.036754	0.974771
H	1.458804	5.079204	2.546971
H	−0.137682	4.323050	2.397604
H	3.917957	2.002574	1.783323
H	4.340827	2.528188	0.152278
H	−4.332589	−0.489749	2.068571
H	−4.625315	−1.852868	0.986530
H	6.537334	−0.690783	0.589111
H	5.147502	−0.648207	1.701941
H	5.231612	−1.892196	0.418809
H	1.162017	−3.430000	0.209079
H	0.098032	−4.059769	−1.072795
H	1.855685	−3.919430	−1.352726
H	−2.099662	2.598956	−3.365135
H	−2.066855	1.026833	−4.211512
H	−3.431377	2.143450	−4.455102
H	−6.646839	1.082427	−0.596601
H	−5.334040	1.633110	0.473771
H	−5.270611	1.984927	−1.277868
H	2.635669	−2.267871	−3.164663
H	4.094774	−1.238223	−3.078227
H	4.247930	−3.011584	−2.990611

ωB97X Energy = −1498.55519086 a.u.

(*aS*,3*S*,3’*S*)-**28**, Conf I

C	−1.334509	−1.077843	−1.297232
C	−2.706442	−1.122987	−1.495261
C	−3.539834	−0.411988	−0.624075
C	−2.999151	0.329154	0.414318
C	−1.615575	0.385959	0.605758
C	−0.787545	−0.330866	−0.256054
C	−1.064307	1.218213	1.740795
C	−2.091528	2.211909	2.263076
C	−1.661742	2.869655	3.554775
O	−3.319501	1.535284	2.508051
C	1.351720	−1.339705	0.537198
C	2.742335	−1.332983	0.700628
C	3.474021	−0.250691	0.219385
C	2.828800	0.795420	−0.441720
C	1.449320	0.778907	−0.604126
C	0.699047	−0.295021	−0.106843
C	0.781255	1.916136	−1.341941
C	1.692435	3.128410	−1.465002
C	1.158501	4.159609	−2.433232
O	2.971131	2.714683	−1.932206
C	3.658189	1.941803	−0.972019
C	−3.933773	1.106923	1.312002
O	4.821388	−0.119054	0.407517

C	5.662721	−1.195606	−0.001928
O	0.631066	−2.414502	0.996968
C	0.417432	−2.425816	2.407519
O	−3.320395	−1.808908	−2.494285
C	−2.502455	−2.534963	−3.396498
O	−4.899972	−0.385798	−0.814278
C	−5.563810	−1.606207	−0.489062
O	3.376722	−2.328955	1.396740
C	3.385312	−3.609033	0.761258
H	−0.666115	−1.619768	−1.953902
H	−0.757098	0.570151	2.569787
H	−0.166822	1.754882	1.421551
H	−2.267790	2.982795	1.496134
H	−2.406205	3.595548	3.883874
H	−1.541615	2.116053	4.336420
H	−0.708194	3.383997	3.420398
H	−0.145007	2.205548	−0.839064
H	0.496178	1.581695	−2.346062
H	1.819380	3.585462	−0.470865
H	1.073964	3.726501	−3.432449
H	1.824945	5.021550	−2.482785
H	0.169948	4.500328	−2.119120
H	3.981968	2.581632	−0.137868
H	4.557399	1.561867	−1.456259
H	−4.328206	1.973705	0.762047
H	−4.786377	0.491189	1.598850
H	5.332343	−1.598004	−0.964215
H	6.660602	−0.775070	−0.114112
H	5.679451	−1.989790	0.743930
H	−0.089903	−3.360892	2.637423
H	1.366447	−2.379265	2.946902
H	−0.220620	−1.588626	2.704888
H	−1.928819	−3.308358	−2.877810
H	−3.180596	−3.002731	−4.106257
H	−1.818557	−1.871187	−3.932954
H	−5.394899	−1.865600	0.561019
H	−6.626242	−1.435800	−0.652998
H	−5.219306	−2.420073	−1.129381
H	2.371496	−3.990852	0.634832
H	3.879341	−3.548498	−0.213356
H	3.952880	−4.270733	1.413027

ωB97X Energy = −1498.55501614 a.u.

(*aS*,3*S*,3’*S*)-**28**, Conf J

C	−1.405459	0.855678	−1.437970
C	−2.781878	1.012455	−1.516104
C	−3.588474	0.412370	−0.542388
C	−3.016829	−0.316233	0.488897
C	−1.629984	−0.457409	0.576963
C	−0.829665	0.125406	−0.401226
C	−1.035920	−1.244991	1.720116
C	−2.040440	−1.455333	2.844102
C	−1.570224	−2.467626	3.863883
O	−3.269357	−1.924233	2.300128
C	1.278520	−1.104858	−0.919330
C	2.671780	−1.225423	−0.896101
C	3.427935	−0.215564	−0.302598
C	2.817360	0.885486	0.284970
C	1.430120	0.995603	0.268246
C	0.658879	0.006753	−0.348001
C	0.778259	2.191633	0.921363
C	1.741486	2.930824	1.838984
C	1.208859	4.275495	2.279231
O	2.971675	3.151831	1.157142
C	3.673303	1.947762	0.935072
C	−3.918517	−0.932176	1.533610
O	4.800017	−0.300054	−0.267792
C	5.432396	−0.080504	−1.530481
O	0.481244	−2.018256	−1.547494
C	0.613470	−3.387678	−1.176779
O	−3.424145	1.723501	−2.479792
C	−2.633571	2.346231	−3.478276
O	−4.951972	0.580141	−0.553398
C	−5.625476	−0.125861	−1.594525
O	3.268573	−2.284280	−1.529749
C	3.976611	−3.187531	−0.678238
H	−0.757195	1.304417	−2.179868
H	−0.700069	−2.225278	1.360618
H	−0.150641	−0.734841	2.111261
H	−2.228811	−0.490717	3.340993
H	−2.303084	−2.576559	4.664361
H	−1.428167	−3.440684	3.388057
H	−0.620379	−2.153410	4.300994
H	−0.101141	1.875435	1.489586
H	0.417952	2.883600	0.151737
H	1.945342	2.307354	2.723601
H	1.059947	4.921656	1.411230
H	1.908192	4.763080	2.959487
H	0.251158	4.155896	2.789473
H	4.074893	1.567027	1.885743
H	4.524391	2.193440	0.299633
H	−4.305984	−0.142882	2.194271
H	−4.776975	−1.409821	1.061505
H	5.192098	0.917309	−1.909595
H	6.504276	−0.154028	−1.357424
H	5.122076	−0.832262	−2.259063
H	0.773399	−3.484515	−0.098292
H	−0.327927	−3.867599	−1.439567
H	1.433779	−3.866460	−1.711679
H	−1.947947	3.078254	−3.041821
H	−2.063090	1.609159	−4.050559
H	−3.330695	2.855932	−4.139096
H	−5.435086	−1.200855	−1.513469
H	−5.308475	0.231390	−2.575849
H	−6.688971	0.063606	−1.460820
H	4.351651	−3.982632	−1.320005
H	4.809003	−2.688332	−0.180079
H	3.302950	−3.612928	0.071772

ωB97X Energy = −1498.55499976 a.u.

(*aS*,3*S*,3’*S*)-**28**, Conf K

C	−1.348116	0.984931	−1.253936
C	−2.725487	1.123083	−1.317302
C	−3.527240	0.368132	−0.463335
C	−2.943644	−0.493085	0.461464
C	−1.557500	−0.611642	0.539046
C	−0.755846	0.123981	−0.338838
C	−0.950233	−1.547728	1.555024
C	−1.955528	−1.964747	2.618440
C	−1.463252	−3.116274	3.465244
O	−3.166940	−2.374050	1.991472
C	1.350940	−1.116105	−0.822294
C	2.740545	−1.242344	−0.813524
C	3.512800	−0.214923	−0.282868
C	2.908195	0.922223	0.245379
C	1.520739	1.034231	0.251987
C	0.733248	0.015768	−0.299412
C	0.886448	2.260600	0.866098
C	1.868040	3.031162	1.737201
C	1.345648	4.393466	2.133265
O	3.084908	3.222375	1.023364
C	3.778644	2.008281	0.834014
C	−3.836452	−1.291871	1.382873
O	4.877844	−0.328030	−0.210010
C	5.546398	−0.257225	−1.470449
O	0.592164	−2.153790	−1.301878
C	0.484382	−2.180858	−2.724126
O	−3.271717	1.971371	−2.249576
C	−3.863816	3.145909	−1.695503
O	−4.896501	0.484767	−0.475542
C	−5.526738	−0.042335	−1.644463
O	3.341200	−2.354591	−1.342915
C	3.356429	−3.469081	−0.449951
H	−0.739157	1.571741	−1.933652
H	−0.575464	−2.440194	1.041251
H	−0.088346	−1.072623	2.032892
H	−2.177964	−1.100474	3.263687
H	−2.196301	−3.372480	4.231092
H	−1.292167	−3.994759	2.839103
H	−0.523976	−2.852846	3.955559
H	0.015011	1.971213	1.460188
H	0.519323	2.926235	0.077044
H	2.087111	2.442399	2.641537
H	1.182026	5.004373	1.242638
H	2.057598	4.906719	2.780677
H	0.397230	4.296149	2.665199
H	4.200828	1.664041	1.789427
H	4.615569	2.224524	0.169588
H	−4.255370	−0.627760	2.153273
H	−4.674777	−1.712168	0.827364
H	5.226684	−1.069453	−2.125227
H	5.351712	0.706284	−1.951778
H	6.610678	−0.348699	−1.262379
H	−0.005059	−1.271853	−3.087233
H	1.470256	−2.282298	−3.184595
H	−0.127453	−3.045260	−2.975034
H	−4.684443	2.890999	−1.021135
H	−3.113625	3.732388	−1.156551
H	−4.243419	3.727428	−2.533499
H	−5.302182	−1.107780	−1.751829
H	−6.597792	0.089459	−1.503841
H	−5.201485	0.491954	−2.539189
H	3.854920	−4.282975	−0.973089
H	3.913709	−3.222472	0.458111
H	2.337041	−3.767514	−0.193135

ωB97X Energy = −1498.55479398 a.u.

(*aS*,3*S*,3’*S*)-**28**, Conf L

C	−1.308241	−1.235058	−1.195049
C	−2.680762	−1.388916	−1.323008
C	−3.523125	−0.726886	−0.422722
C	−2.990378	0.040526	0.601189
C	−1.607206	0.192946	0.732192
C	−0.769880	−0.451557	−0.176811
C	−1.067544	1.058544	1.847229
C	−2.140011	1.977863	2.412543
C	−1.701256	2.676965	3.679234
O	−3.299506	1.211788	2.719260
C	1.468808	−1.311530	0.506792
C	2.860940	−1.205605	0.611516
C	3.491686	−0.068476	0.113939
C	2.745090	0.935145	−0.504980
C	1.364460	0.820303	−0.608745
C	0.715401	−0.309433	−0.093668
C	0.586383	1.913294	−1.304304
C	1.399761	3.191369	−1.442649
C	0.751843	4.194370	−2.370178
O	2.684280	2.880316	−1.969224
C	3.466303	2.144356	−1.053419
C	−3.938804	0.723943	1.559898
O	4.833284	0.157751	0.247039
C	5.729849	−0.848255	−0.220394
O	0.849329	−2.441487	0.980927
C	0.681240	−2.477282	2.397179
O	−3.287361	−2.150003	−2.270836
C	−2.458474	−2.881944	−3.158199
O	−4.886307	−0.877070	−0.493302
C	−5.498902	−0.213431	−1.596882
O	3.593166	−2.160615	1.267513
C	3.668567	−3.428799	0.612489
H	−0.632971	−1.733047	−1.878873
H	−0.683849	0.433600	2.661909
H	−0.223040	1.655992	1.493353
H	−2.409785	2.726601	1.650919
H	−2.482077	3.347552	4.040032
H	−1.487771	1.940924	4.457520
H	−0.796543	3.260423	3.497519
H	−0.339424	2.126663	−0.763778
H	0.289807	1.570679	−2.302398
H	1.533931	3.641812	−0.446417
H	0.662320	3.772165	−3.373602

H	1.346962	5.106464	−2.428113
H	−0.247191	4.452044	−2.012840
H	3.782469	2.794004	−0.224016
H	4.367005	1.836455	−1.583941
H	−4.451059	1.548565	1.041013
H	−4.704451	0.023591	1.892050
H	5.388914	−1.253267	−1.177901
H	6.691078	−0.357193	−0.361991
H	5.832780	−1.654236	0.505806
H	−0.009518	−1.693516	2.720940
H	0.253416	−3.449483	2.634399
H	1.640348	−2.361699	2.907599
H	−1.815477	−3.578301	−2.612737
H	−3.130134	−3.441490	−3.804848
H	−1.840479	−2.215484	−3.766835
H	−5.145348	−0.624509	−2.544284
H	−6.570527	−0.379240	−1.503091
H	−5.294056	0.861395	−1.560351
H	2.678562	−3.873608	0.505563
H	4.132776	−3.322767	−0.372867
H	4.293781	−4.061247	1.240107

ωB97X Energy = −1498.55459787 a.u.

(*aS*,3*S*,3'*S*)-**28**, Conf M

C	−1.391363	0.894494	−1.412447
C	−2.769888	0.970067	−1.552096
C	−3.579329	0.194416	−0.714848
C	−3.009403	−0.657992	0.218143
C	−1.621784	−0.731155	0.359348
C	−0.816989	0.049608	−0.465574
C	−1.032556	−1.652345	1.399693
C	−2.071364	−2.085729	2.423611
C	−1.583403	−3.207825	3.311584
O	−3.236902	−2.543473	1.747143
C	1.392638	−1.002280	−0.962055
C	2.789017	−1.027505	−0.883423
C	3.445983	−0.013525	−0.187437
C	2.735161	0.996192	0.448794
C	1.345222	1.011686	0.375797
C	0.672188	0.021206	−0.344160
C	0.584047	2.108745	1.083352
C	1.454378	2.840592	2.094908
C	0.810973	4.108982	2.607251
O	2.690791	3.196968	1.485782
C	3.486274	2.064694	1.208654
C	−3.916762	−1.496182	1.089437
O	4.818285	−0.001904	−0.100022
C	5.480083	0.345089	−1.318349
O	0.686447	−1.916507	−1.687948
C	0.937307	−3.300977	−1.461150
O	−3.412524	1.750668	−2.460072
C	−2.618175	2.530062	−3.338252
O	−4.946794	0.212344	−0.845359
C	−5.570126	1.406195	−0.377893
O	3.486350	−1.990287	−1.565733
C	4.232319	−2.900133	−0.755131
H	−0.741313	1.484359	−2.046177
H	−0.625284	−2.547523	0.914888
H	−0.197233	−1.161745	1.908081
H	−2.349043	−1.218384	3.043223
H	−2.342666	−3.475549	4.047334
H	−1.357015	−4.090710	2.709313
H	−0.676455	−2.907130	3.839899
H	−0.293057	1.690656	1.585560
H	0.207810	2.831426	0.350554
H	1.667481	2.167262	2.939806
H	0.649044	4.806894	1.782682
H	1.448654	4.590928	3.349315
H	−0.153797	3.887434	3.067670
H	3.878614	1.644372	2.146409
H	4.340415	2.415430	0.629226
H	−4.413532	−0.850549	1.829613
H	−4.696661	−1.956562	0.483855
H	5.162630	1.336188	−1.656109
H	6.545863	0.361453	−1.098854
H	5.273020	−0.390038	−2.098807
H	1.131250	−3.492220	−0.401071
H	0.030363	−3.826559	−1.756331
H	1.780263	−3.653574	−2.055152
H	−2.006862	3.250549	−2.786877
H	−1.971076	1.897905	−3.952712
H	−3.314667	3.064849	−3.979623
H	−5.238528	2.271819	−0.954549
H	−5.352575	1.563967	0.683435
H	−6.641924	1.269151	−0.509219
H	4.685679	−3.616735	−1.437520
H	5.008976	−2.380321	−0.192158
H	3.569716	−3.427800	−0.062574

ωB97X Energy = −1498.55458641 a.u.

(*aS*,3*S*,3'*S*)-**28**, Conf N

C	−1.348557	0.936778	−1.287060
C	−2.727534	1.039699	−1.377637
C	−3.525551	0.307892	−0.500857
C	−2.939462	−0.548304	0.427119
C	−1.552623	−0.647332	0.517805
C	−0.752646	0.103361	−0.349116
C	−0.943772	−1.564399	1.549716
C	−1.952313	−1.971234	2.613766
C	−1.452825	−3.097890	3.489282
O	−3.152738	−2.409047	1.985397
C	1.386238	−1.099421	−0.782251
C	2.777842	−1.196132	−0.752416
C	3.520832	−0.144727	−0.226850
C	2.885136	0.987803	0.274108
C	1.495623	1.071199	0.258601
C	0.737547	0.027343	−0.286455
C	0.826656	2.294566	0.840967
C	1.778708	3.104307	1.709439
C	1.222203	4.463975	2.066056

O	3.002471	3.305152	1.010170
C	3.723236	2.101875	0.857411
C	−3.831896	−1.351498	1.345710
O	4.886734	−0.228627	−0.132866
C	5.571611	−0.163398	−1.384896
O	0.656429	−2.160521	−1.255664
C	0.565710	−2.210225	−2.678098
O	−3.277122	1.890371	−2.305446
C	−3.942228	1.230452	−3.382452
O	−4.897048	0.367500	−0.563788
C	−5.457194	1.606233	−0.126755
O	3.408345	−2.303440	−1.256981
C	3.442451	−3.400923	−0.343835
H	−0.741620	1.520379	−1.971317
H	−0.560690	−2.462189	1.051723
H	−0.087861	−1.076143	2.024845
H	−2.190548	−1.095967	3.238449
H	−2.189734	−3.347888	4.253510
H	−1.263825	−3.987172	2.883859
H	−0.522037	−2.811114	3.982644
H	−0.045913	1.997189	1.429409
H	0.454752	2.935991	0.034457
H	1.995790	2.540438	2.629964
H	1.057856	5.049823	1.158904
H	1.914095	5.007544	2.710529
H	0.268665	4.358904	2.587230
H	4.133972	1.784677	1.827090
H	4.568263	2.321985	0.204612
H	−4.279653	−0.684104	2.097936
H	−4.648887	−1.796714	0.778768
H	5.364175	0.788513	−1.883620
H	6.634496	−0.229739	−1.160766
H	5.277573	−0.992013	−2.031175
H	1.558879	−2.293914	−3.126325
H	−0.022067	−3.092737	−2.923420
H	0.058769	−1.318397	−3.059768
H	−3.242735	0.587359	−3.924839
H	−4.782068	0.634223	−3.018879
H	−4.305650	2.010770	−4.048463
H	−5.120839	2.431676	−0.757462
H	−6.537690	1.500617	−0.202541
H	−5.183884	1.803234	0.914256
H	3.988794	−3.125964	0.562714
H	2.428389	−3.717279	−0.087343
H	3.961515	−4.213105	−0.849357

ωB97X Energy = −1498.55454131 a.u.

(*aS*,3*S*,3’*S*)-**28**, Conf O

C	1.336540	0.743974	1.351975
C	2.714340	0.851193	1.478997
C	3.530505	0.254980	0.511823
C	2.968930	−0.441605	−0.547750
C	1.582747	−0.567847	−0.663070
C	0.770781	0.037899	0.292015
C	1.001462	−1.333509	−1.827512
C	2.028988	−2.268859	−2.454460
C	2.273423	−3.521811	−1.624281
O	3.229704	−1.539669	−2.715261
C	−1.378934	−1.180806	0.633074
C	−2.770102	−1.269778	0.577362
C	−3.500847	−0.192945	0.086567
C	−2.852978	0.957763	−0.353196
C	−1.463579	1.034193	−0.309828
C	−0.718776	−0.035298	0.200678
C	−0.778327	2.280611	−0.819168
C	−1.710225	3.134994	−1.666148
C	−1.142292	4.507785	−1.946039
O	−2.946980	3.307345	−0.981553
C	−3.675242	2.101333	−0.900722
C	3.891762	−1.096138	−1.548705
O	−4.865374	−0.267355	−0.034047
C	−5.570890	−0.254990	1.208042
O	−0.659723	−2.270266	1.055857
C	−0.572082	−2.390689	2.474047
O	3.348530	1.494591	2.494375
C	2.548016	2.086616	3.503050
O	4.898727	0.291342	0.622667
C	5.481416	1.569377	0.373248
O	−3.410849	−2.397736	1.019794
C	−3.459790	−3.438589	0.043444
H	0.679936	1.200072	2.082482
H	0.134319	−1.911351	−1.500237
H	0.647755	−0.629319	−2.587933
H	1.681118	−2.568947	−3.444639
H	3.091113	−4.106905	−2.049564
H	2.512377	−3.286281	−0.584739
H	1.372922	−4.138918	−1.623034
H	0.104962	2.007466	−1.403433
H	−0.420364	2.879609	0.025770
H	−1.910510	2.616243	−2.616308
H	−0.994294	5.048933	−1.008711
H	−1.819178	5.084680	−2.577346
H	−0.179123	4.423420	−2.453010
H	−4.066015	1.832273	−1.892865
H	−4.533361	2.294337	−0.256451
H	4.651780	−0.387751	−1.880054
H	4.421403	−1.927106	−1.064234
H	−5.294304	−1.115405	1.819560
H	−5.364276	0.670845	1.753942
H	−6.630381	−0.301426	0.963692
H	−0.003066	−3.295905	2.677185
H	−0.047503	−1.528919	2.898627
H	−1.567888	−2.475873	2.916348
H	1.900595	2.866375	3.091342
H	3.240465	2.530739	4.214145
H	1.935909	1.337330	4.013448
H	5.237862	1.911358	−0.637846
H	5.138119	2.304286	1.103387
H	6.558707	1.441608	0.461399
H	−4.008185	−3.105881	−0.842206
H	−2.449593	−3.747512	−0.237066

H	−3.982659	−4.275219	0.503039

ωB97X Energy = −1498.55411257 a.u.

(*aR*,3*S*,3’*S*)-**28**, Conf A

C	−1.365688	1.140715	−1.122244
C	−2.739172	1.334514	−1.160280
C	−3.556858	0.546125	−0.342665
C	−2.999447	−0.401989	0.501089
C	−1.615451	−0.582410	0.554071
C	−0.803482	0.188425	−0.273414
C	−1.036223	−1.616179	1.488846
C	−2.046187	−2.068005	2.533719
C	−1.595356	−3.300626	3.284132
O	−3.282501	−2.376458	1.897563
C	1.256403	−1.079794	−0.876765
C	2.640547	−1.256874	−0.887227
C	3.452049	−0.304536	−0.280635
C	2.890816	0.807936	0.340046
C	1.508429	0.969240	0.365096
C	0.681605	0.027485	−0.260495
C	0.921977	2.163431	1.082201
C	1.933879	2.814668	2.013477
C	1.464656	4.153209	2.536716
O	3.154602	3.025035	1.311176
C	3.803442	1.808623	1.010484
C	−3.911927	−1.223681	1.382028
O	4.812290	−0.471294	−0.223860
C	5.480948	−0.330274	−1.478147
O	0.460008	−2.047738	−1.434869
C	0.343655	−1.950964	−2.852838
O	−3.368282	2.251856	−1.941476
C	−2.563870	3.077418	−2.766115
O	−4.917052	0.734228	−0.315669
C	−5.599772	0.285283	−1.485230
O	3.198056	−2.344764	−1.507079
C	3.166218	−3.529906	−0.710443
H	−0.710165	1.735205	−1.746420
H	−0.707917	−2.485245	0.907163
H	−0.148891	−1.215558	1.987903
H	−2.221764	−1.246466	3.245832
H	−2.327543	−3.580257	4.042563
H	−1.475530	−4.136124	2.590808
H	−0.637140	−3.117694	3.774381
H	0.038630	1.859312	1.650563
H	0.582870	2.908364	0.353809
H	2.133585	2.136862	2.857843
H	1.323932	4.851352	1.708389
H	2.196861	4.575404	3.226081
H	0.513854	4.043290	3.061804
H	4.220293	1.368687	1.928096
H	4.642733	2.052240	0.358490
H	−4.284184	−0.598298	2.206763
H	−4.779601	−1.564457	0.817249
H	5.313012	0.669856	−1.889880
H	6.542441	−0.465994	−1.280253
H	5.137787	−1.084652	−2.187900
H	1.323497	−2.042655	−3.328173
H	−0.296236	−2.771777	−3.171167
H	−0.119986	−0.999925	−3.133345
H	−1.995867	2.484715	−3.489147
H	−3.249960	3.732825	−3.297245
H	−1.874861	3.681574	−2.168759
H	−5.275289	0.843592	−2.365097
H	−6.660218	0.458781	−1.311957
H	−5.427658	−0.784264	−1.642913
H	3.723531	−3.380092	0.218403
H	2.134886	−3.812352	−0.485385
H	3.640787	−4.314159	−1.297215

ωB97X Energy = −1498.55731613 a.u.

(*aR*,3*S*,3’*S*)-**28**, Conf B

C	−1.363283	0.969984	−1.274467
C	−2.741564	1.070493	−1.399291
C	−3.554536	0.258422	−0.600624
C	−2.989122	−0.653670	0.277236
C	−1.601611	−0.753229	0.403564
C	−0.793114	0.064599	−0.380853
C	−1.014209	−1.745705	1.376628
C	−2.050808	−2.244951	2.372490
C	−1.569813	−3.439446	3.164730
O	−3.229072	−2.635200	1.674618
C	1.378991	−1.061771	−0.858825
C	2.770897	−1.138785	−0.796998
C	3.478444	−0.132875	−0.147866
C	2.807712	0.935104	0.441339
C	1.418054	0.999219	0.389401
C	0.695904	0.002774	−0.279135
C	0.708839	2.147116	1.070405
C	1.619568	2.873813	2.049531
C	1.030942	4.178988	2.534742
O	2.859327	3.166108	1.413217
C	3.607468	1.996903	1.159836
C	−3.899155	−1.532783	1.104220
O	4.842434	−0.204157	−0.019233
C	5.563730	0.004860	−1.234156
O	0.685805	−2.085863	−1.453143
C	0.590629	−1.969410	−2.871194
O	−3.380485	1.907754	−2.258307
C	−2.581396	2.725798	−3.095607
O	−4.921820	0.300907	−0.721122
C	−5.528363	1.470648	−0.176699
O	3.436671	−2.178763	−1.390710
C	3.460371	−3.365336	−0.596372
H	−0.710641	1.588729	−1.877690
H	−0.607912	−2.599112	0.821803
H	−0.177811	−1.291546	1.916270
H	−2.313891	−1.426566	3.061066
H	−2.323866	−3.752683	3.887834
H	−1.364094	−4.274846	2.491795

H	−0.651526	−3.194131	3.701996
H	−0.177408	1.777670	1.593948
H	0.352749	2.863646	0.321755
H	1.819949	2.216773	2.910050
H	0.881664	4.859257	1.693173
H	1.697276	4.657363	3.253544
H	0.066075	4.005776	3.015269
H	3.996667	1.588297	2.103852
H	4.466626	2.296342	0.558985
H	−4.370851	−0.930980	1.896171
H	−4.697420	−1.934980	0.481386
H	6.620444	−0.067542	−0.984160
H	5.306344	−0.755038	−1.973516
H	5.353115	1.000524	−1.637106
H	0.043144	−2.842270	−3.221509
H	0.040849	−1.062850	−3.143342
H	1.584994	−1.952622	−3.324858
H	−1.957495	3.405826	−2.508034
H	−1.945471	2.121190	−3.748736
H	−3.273812	3.304772	−3.701988
H	−5.191144	2.366295	−0.701921
H	−5.301604	1.560948	0.890500
H	−6.602452	1.353306	−0.307962
H	3.966696	−3.177654	0.354585
H	2.444650	−3.724832	−0.414434
H	4.015817	−4.109935	−1.163494

ωB97X Energy = −1498.55692462 a.u.

(*aR*,3*S*,3’*S*)-**28**, Conf C

C	−1.451474	1.127274	−1.085600
C	−2.831531	1.267122	−1.062796
C	−3.581815	0.437767	−0.221357
C	−2.951447	−0.498168	0.583614
C	−1.560145	−0.623494	0.576423
C	−0.814911	0.189351	−0.273672
C	−0.902426	−1.643960	1.473615
C	−1.850225	−2.146968	2.552627
C	−1.320755	−3.368648	3.269045
O	−3.098002	−2.498385	1.963239
C	1.272769	−0.988987	−0.956224
C	2.664206	−1.109607	−1.034367
C	3.459001	−0.112517	−0.473724
C	2.868381	0.991080	0.143737
C	1.485683	1.089056	0.237627
C	0.674810	0.094165	−0.324279
C	0.880272	2.263361	0.971142
C	1.907238	2.970707	1.842304
C	1.401366	4.288198	2.384610
O	3.072939	3.236799	1.070622
C	3.766643	2.055897	0.730841
C	−3.793902	−1.365912	1.489689
O	4.823163	−0.107786	−0.554454
C	5.525326	−1.279722	−0.147922
O	0.491662	−1.973704	−1.511403
C	0.270912	−1.805574	−2.910849
O	−3.528573	2.167723	−1.804724
C	−2.792555	3.038480	−2.646843
O	−4.945915	0.572862	−0.132986
C	−5.660698	0.102695	−1.274641
O	3.242894	−2.137349	−1.732094
C	3.087592	−3.434247	−1.151794
H	−0.846569	1.753331	−1.729638
H	−0.564154	−2.492761	0.868276
H	−0.012275	−1.213212	1.941573
H	−2.029089	−1.340826	3.281318
H	−2.012450	−3.687874	4.049678
H	−1.192281	−4.190034	2.560580
H	−0.353615	−3.152022	3.727081
H	0.042142	1.924576	1.586359
H	0.471265	2.984652	0.254990
H	2.187353	2.310004	2.677577
H	1.174769	4.970060	1.561858
H	2.150681	4.754497	3.025474
H	0.490764	4.134696	2.966962
H	4.280736	1.653516	1.616412
H	4.534031	2.338968	0.011001
H	−4.156581	−0.766295	2.337465
H	−4.669680	−1.735316	0.956375
H	5.091233	−1.690592	0.768557
H	5.519269	−2.037949	−0.930418
H	6.548814	−0.966585	0.051074
H	−0.345951	−2.642216	−3.233579
H	−0.259188	−0.867699	−3.103010
H	1.218956	−1.814593	−3.455014
H	−2.101182	3.657620	−2.067831
H	−2.235137	2.481501	−3.405572
H	−3.525723	3.676760	−3.134150
H	−5.386946	0.668453	−2.167026
H	−5.464882	−0.961912	−1.438373
H	−6.718206	0.246749	−1.061040
H	3.560941	−3.471346	−0.165725
H	2.033215	−3.698286	−1.064417
H	3.590515	−4.131257	−1.819649

ωB97X Energy = −1498.55590620 a.u.

(*aR*,3*S*,3’*S*)-**28**, Conf D

C	−1.423899	0.761770	−1.438851
C	−2.802702	0.908296	−1.491162
C	−3.581089	0.362523	−0.464014
C	−2.979530	−0.302013	0.593302
C	−1.589975	−0.429891	0.655846
C	−0.817556	0.097590	−0.375013
C	−0.962455	−1.142061	1.830065
C	−1.938758	−1.297374	2.987534
C	−1.436542	−2.251055	4.047874
O	−3.175915	−1.804413	2.499042
C	1.289475	−1.167556	−0.817124
C	2.683135	−1.266906	−0.837069
C	3.449149	−0.216698	−0.339450
C	2.844171	0.944090	0.129785

C	1.456248	1.049653	0.123111
C	0.672875	−0.006758	−0.353098
C	0.814540	2.309908	0.653944
C	1.789344	3.138877	1.477602
C	1.258725	4.520919	1.784462
O	3.007268	3.291567	0.756927
C	3.709897	2.073653	0.638466
C	−3.850866	−0.861671	1.693970
O	4.818747	−0.272729	−0.380837
C	5.406098	−1.208549	0.522702
O	0.497030	−2.163269	−1.317168
C	0.679804	−3.476843	−0.788243
O	−3.472443	1.559791	−2.477891
C	−2.709613	2.132789	−3.526371
O	−4.945252	0.524184	−0.450031
C	−5.641907	−0.246616	−1.427785
O	3.309041	−2.380270	−1.337445
C	3.336625	−2.421731	−2.764460
H	−0.797081	1.170150	−2.221481
H	−0.624418	−2.138569	1.521839
H	−0.074426	−0.600552	2.169390
H	−2.123982	−0.309230	3.436927
H	−2.150438	−2.324075	4.869248
H	−1.295974	−3.246149	3.619605
H	−0.480172	−1.906774	4.446486
H	−0.059386	2.057524	1.261152
H	0.448194	2.919264	−0.179903
H	2.007463	2.608901	2.418019
H	1.096338	5.072720	0.855848
H	1.965161	5.078132	2.400901
H	0.308203	4.453012	2.317145
H	4.145430	1.797725	1.610902
H	4.536087	2.251809	−0.049307
H	−4.227598	−0.037791	2.317541
H	−4.717177	−1.370847	1.271925
H	6.484076	−1.115747	0.405460
H	5.131741	−0.967760	1.554552
H	5.095493	−2.227341	0.284925
H	0.913246	−3.435637	0.279027
H	−0.265755	−3.997311	−0.931634
H	1.479702	−4.007936	−1.305607
H	−2.018229	2.889983	−3.145443
H	−2.148396	1.369132	−4.072489
H	−3.425287	2.603056	−4.196524
H	−6.702599	−0.054109	−1.277243
H	−5.354183	0.050879	−2.437667
H	−5.443222	−1.313925	−1.287069
H	2.322229	−2.458567	−3.171860
H	3.860744	−1.548402	−3.161536
H	3.875248	−3.326533	−3.040045

ωB97X Energy = −1498.55560804 a.u.

(*aR*,3*S*,3'*S*)-**28**, Conf E

C	−1.433658	1.004859	−1.183702
C	−2.811670	1.165084	−1.210360
C	−3.596472	0.421243	−0.321929
C	−3.002516	−0.449959	0.577950
C	−1.613874	−0.594968	0.618685
C	−0.834573	0.130933	−0.277604
C	−0.995639	−1.545507	1.614538
C	−1.976151	−1.936220	2.710644
C	−1.485280	−3.097698	3.544704
O	−3.216619	−2.318864	2.124441
C	1.248645	−1.121856	−0.830458
C	2.639485	−1.254080	−0.872467
C	3.426827	−0.252947	−0.309159
C	2.845173	0.891737	0.227215
C	1.460615	1.022785	0.254238
C	0.654537	0.010601	−0.280554
C	0.851959	2.249756	0.892217
C	1.855261	2.985059	1.768569
C	1.358656	4.344584	2.205289
O	3.064884	3.176518	1.042264
C	3.741339	1.960618	0.809982
C	−3.881224	−1.223590	1.533560
O	4.797595	−0.350996	−0.300113
C	5.311329	−1.351459	0.580885
O	0.458880	−2.142605	−1.292131
C	0.299781	−2.172029	−2.705651
O	−3.474948	2.008862	−2.044790
C	−2.702441	2.800152	−2.931135
O	−4.960027	0.581780	−0.281295
C	−5.653901	0.034573	−1.401304
O	3.176011	−2.393171	−1.408407
C	4.019572	−2.200864	−2.545075
H	−0.802433	1.566378	−1.861133
H	−0.659748	−2.448109	1.091862
H	−0.107723	−1.092165	2.065165
H	−2.157766	−1.065497	3.360080
H	−2.201062	−3.335986	4.332477
H	−1.353276	−3.980469	2.915203
H	−0.525870	−2.855852	4.006303
H	−0.020058	1.965386	1.487529
H	0.491357	2.936803	0.118551
H	2.077595	2.370845	2.655100
H	1.193183	4.980606	1.332770
H	2.086426	4.830499	2.856243
H	0.415572	4.248007	2.746641
H	4.188971	1.595597	1.746764
H	4.558489	2.180643	0.123401
H	−4.256427	−0.545655	2.314306
H	−4.748644	−1.626538	1.010901
H	6.396013	−1.293193	0.515403
H	4.997514	−1.151888	1.609632
H	4.974707	−2.347233	0.283557
H	−0.145538	−1.237656	−3.062503
H	1.257621	−2.338714	−3.205814
H	−0.370929	−2.999541	−2.928908
H	−2.020874	3.456696	−2.382455
H	−2.128995	2.177262	−3.623856
H	−3.411370	3.404387	−3.492138

H	−6.714645	0.197762	−1.219866
H	−5.358710	0.534432	−2.325506
H	−5.460858	−1.039740	−1.485229
H	3.502004	−1.615766	−3.311430
H	4.947215	−1.694891	−2.272783
H	4.237383	−3.193447	−2.934510

ωB97X Energy = −1498.55558051 a.u.

(*aR*,3*S*,3’*S*)-**28**, Conf F

C	−1.456346	0.955859	−1.235563
C	−2.841521	0.996494	−1.300355
C	−3.582967	0.138989	−0.479697
C	−2.939457	−0.756326	0.360820
C	−1.544624	−0.794107	0.428089
C	−0.806996	0.067352	−0.379488
C	−0.874798	−1.767318	1.367103
C	−1.847010	−2.316931	2.400383
C	−1.280266	−3.491324	3.165879
O	−3.032982	−2.757672	1.747499
C	1.392808	−0.964365	−0.935953
C	2.791731	−0.982249	−0.941554
C	3.480936	0.071722	−0.346201
C	2.780519	1.129552	0.235415
C	1.391361	1.126525	0.256101
C	0.686101	0.074981	−0.343772
C	0.664035	2.253740	0.951837
C	1.589011	3.036188	1.871974
C	0.962387	4.314995	2.379188
O	2.771216	3.384964	1.161051
C	3.566147	2.257536	0.864251
C	−3.775297	−1.683616	1.213122
O	4.843311	0.177818	−0.357741
C	5.609283	−0.937518	0.090696
O	0.716107	−2.006572	−1.521650
C	0.547354	−1.859405	−2.930763
O	−3.552573	1.815253	−2.119742
C	−2.827404	2.677215	−2.980192
O	−4.954717	0.118810	−0.541447
C	−5.591355	1.258976	0.030984
O	3.480147	−1.964939	−1.603524
C	3.397649	−3.268356	−1.023318
H	−0.857907	1.609299	−1.858036
H	−0.456501	−2.599327	0.789360
H	−0.036916	−1.280864	1.875390
H	−2.119097	−1.514301	3.103783
H	−1.991723	−3.844393	3.913470
H	−1.059574	−4.312353	2.480079
H	−0.355958	−3.204938	3.671627
H	−0.177888	1.854197	1.524004
H	0.240827	2.942180	0.211951
H	1.871743	2.399676	2.725083
H	0.728889	4.975773	1.541295
H	1.643325	4.836757	3.052677
H	0.037335	4.098353	2.917079
H	4.060642	1.894675	1.777664
H	4.348214	2.594964	0.184633
H	−4.244010	−1.111161	2.028194
H	−4.577296	−2.118232	0.617464
H	6.597453	−0.548932	0.330655
H	5.165678	−1.372228	0.991564
H	5.691788	−1.699425	−0.683814
H	−0.054897	−0.973056	−3.152564
H	1.516917	−1.784077	−3.430092
H	0.023455	−2.747887	−3.278051
H	−3.570977	3.230800	−3.548536
H	−2.209320	3.378174	−2.411477
H	−2.194607	2.108769	−3.667769
H	−5.314958	2.169881	−0.503331
H	−5.327555	1.356518	1.089010
H	−6.663454	1.094171	−0.059219
H	3.806367	−3.261738	−0.008081
H	2.366667	−3.622737	−1.002836
H	4.001567	−3.921474	−1.650683

ωB97X Energy = −1498.55550722 a.u.

(*aR*,3*S*,3’*S*)-**28**, Conf G

C	−1.427006	0.859920	−1.302422
C	−2.809082	0.924924	−1.405919
C	−3.589438	0.134640	−0.554598
C	−2.987941	−0.719257	0.357133
C	−1.596451	−0.781464	0.463313
C	−0.820342	0.012614	−0.376387
C	−0.971969	−1.709245	1.476004
C	−1.980104	−2.168239	2.518909
C	−1.458984	−3.300321	3.374803
O	−3.160558	−2.625292	1.866706
C	1.368229	−1.090497	−0.832886
C	2.764899	−1.129581	−0.803380
C	3.452858	−0.088796	−0.183614
C	2.768540	1.006061	0.334997
C	1.378608	1.045597	0.288817
C	0.671621	−0.008989	−0.300832
C	0.655362	2.219284	0.907680
C	1.560077	3.015519	1.836896
C	0.952106	4.336889	2.249151
O	2.790318	3.290932	1.175699
C	3.557230	2.123575	0.978535
C	−3.864778	−1.573759	1.243909
O	4.824445	−0.094224	−0.100877
C	5.359911	−1.088560	0.774436
O	0.677606	−2.154757	−1.351045
C	0.519093	−2.121257	−2.764536
O	−3.481780	1.706849	−2.291089
C	−2.717341	2.505925	−3.177462
O	−4.959400	0.140627	−0.652167
C	−5.582704	1.321439	−0.152382
O	3.405805	−2.217952	−1.329704
C	4.270825	−1.943353	−2.432556
H	−0.798922	1.462910	−1.946270
H	−0.562674	−2.585400	0.960887

H	−0.132658	−1.213028	1.972348
H	−2.250303	−1.314634	3.160670
H	−2.197072	−3.588669	4.124304
H	−1.237244	−4.169478	2.751573
H	−0.542344	−2.998744	3.885591
H	−0.221431	1.866756	1.457854
H	0.283590	2.886938	0.122396
H	1.776661	2.413136	2.732885
H	0.784178	4.962226	1.369377
H	1.614684	4.869790	2.932165
H	−0.006016	4.175663	2.747036
H	3.969330	1.778989	1.939121
H	4.399188	2.401899	0.345152
H	−4.342158	−0.939789	2.006884
H	−4.660360	−2.029852	0.655695
H	4.953496	−0.966705	1.782635
H	5.136888	−2.093945	0.410434
H	6.436748	−0.933347	0.796673
H	−0.016139	−1.216773	−3.071101
H	1.488332	−2.165059	−3.269638
H	−0.067224	−2.997114	−3.035657
H	−3.434337	3.039838	−3.796543
H	−2.101606	3.227017	−2.631739
H	−2.077100	1.888970	−3.814650
H	−5.344702	1.463837	0.906682
H	−5.270070	2.199085	−0.721521
H	−6.655796	1.177606	−0.264100
H	5.127198	−1.340054	−2.126295
H	4.610158	−2.908725	−2.802902
H	3.726895	−1.421243	−3.225779

ωB97X Energy = −1498.55524483 a.u.

(*aR*,3*S*,3’*S*)-**28**, Conf H

C	−1.413390	0.588807	−1.537563
C	−2.795802	0.618306	−1.654147
C	−3.572831	0.021558	−0.655037
C	−2.967268	−0.615314	0.417180
C	−1.575681	−0.642191	0.534583
C	−0.803562	−0.037139	−0.452960
C	−0.946493	−1.320283	1.727656
C	−1.955480	−1.547231	2.844208
C	−1.429666	−2.459731	3.929086
O	−3.127696	−2.147977	2.305499
C	1.414349	−1.118667	−0.830909
C	2.811182	−1.104832	−0.795937
C	3.469099	−0.009168	−0.244548
C	2.753867	1.085750	0.227523
C	1.363091	1.078867	0.167958
C	0.688027	−0.023130	−0.366753
C	0.599439	2.267431	0.704024
C	1.472514	3.152824	1.581657
C	0.820002	4.479708	1.897321
O	2.699285	3.420951	0.912194
C	3.503344	2.267038	0.798623
C	−3.840678	−1.267307	1.464515
O	4.839385	0.046313	−0.234176
C	5.465395	−0.857625	0.675775
O	0.725411	−2.161716	−1.384284
C	0.988609	−3.467326	−0.869748
O	−3.471567	1.188366	−2.685874
C	−2.710931	1.769089	−3.731768
O	−4.942889	−0.003060	−0.750552
C	−5.574540	1.255123	−0.525209
O	3.545621	−2.151283	−1.292840
C	3.628299	−2.159115	−2.718395
H	−0.787489	1.044917	−2.294132
H	−0.531547	−2.290327	1.429102
H	−0.110669	−0.722541	2.103320
H	−2.233279	−0.575044	3.280933
H	−2.167885	−2.579510	4.723040
H	−1.205132	−3.444136	3.512292
H	−0.515259	−2.049060	4.361803
H	−0.269795	1.926893	1.273856
H	0.211618	2.865033	−0.128580
H	1.698794	2.619568	2.518298
H	0.641694	5.036754	0.974771
H	1.458804	5.079204	2.546971
H	−0.137682	4.323050	2.397604
H	3.917957	2.002574	1.783323
H	4.340827	2.528188	0.152278
H	−4.332589	−0.489749	2.068571
H	−4.625315	−1.852868	0.986530
H	6.537334	−0.690783	0.589111
H	5.147502	−0.648207	1.701941
H	5.231612	−1.892196	0.418809
H	1.162017	−3.430000	0.209079
H	0.098032	−4.059769	−1.072795
H	1.855685	−3.919430	−1.352726
H	−2.099662	2.598956	−3.365135
H	−2.066855	1.026833	−4.211512
H	−3.431377	2.143450	−4.455102
H	−6.646839	1.082427	−0.596601
H	−5.334040	1.633110	0.473771
H	−5.270611	1.984927	−1.277868
H	2.635669	−2.267871	−3.164663
H	4.094774	−1.238223	−3.078227
H	4.247930	−3.011584	−2.990611

ωB97X Energy = −1498.55519086 a.u.

(*aR*,3*S*,3’*S*)-**28**, Conf I

C	−1.334509	−1.077843	−1.297232
C	−2.706442	−1.122987	−1.495261
C	−3.539834	−0.411988	−0.624075
C	−2.999151	0.329154	0.414318
C	−1.615575	0.385959	0.605758
C	−0.787545	−0.330866	−0.256054
C	−1.064307	1.218213	1.740795
C	−2.091528	2.211909	2.263076
C	−1.661742	2.869655	3.554775
O	−3.319501	1.535284	2.508051

C	1.351720	−1.339705	0.537198
C	2.742335	−1.332983	0.700628
C	3.474021	−0.250691	0.219385
C	2.828800	0.795420	−0.441720
C	1.449320	0.778907	−0.604126
C	0.699047	−0.295021	−0.106843
C	0.781255	1.916136	−1.341941
C	1.692435	3.128410	−1.465002
C	1.158501	4.159609	−2.433232
O	2.971131	2.714683	−1.932206
C	3.658189	1.941803	−0.972019
C	−3.933773	1.106923	1.312002
O	4.821388	−0.119054	0.407517
C	5.662721	−1.195606	−0.001928
O	0.631066	−2.414502	0.996968
C	0.417432	−2.425816	2.407519
O	−3.320395	−1.808908	−2.494285
C	−2.502455	−2.534963	−3.396498
O	−4.899972	−0.385798	−0.814278
C	−5.563810	−1.606207	−0.489062
O	3.376722	−2.328955	1.396740
C	3.385312	−3.609033	0.761258
H	−0.666115	−1.619768	−1.953902
H	−0.757098	0.570151	2.569787
H	−0.166822	1.754882	1.421551
H	−2.267790	2.982795	1.496134
H	−2.406205	3.595548	3.883874
H	−1.541615	2.116053	4.336420
H	−0.708194	3.383997	3.420398
H	−0.145007	2.205548	−0.839064
H	0.496178	1.581695	−2.346062
H	1.819380	3.585462	−0.470865
H	1.073964	3.726501	−3.432449
H	1.824945	5.021550	−2.482785
H	0.169948	4.500328	−2.119120
H	3.981968	2.581632	−0.137868
H	4.557399	1.561867	−1.456259
H	−4.328206	1.973705	0.762047
H	−4.786377	0.491189	1.598850
H	5.332343	−1.598004	−0.964215
H	6.660602	−0.775070	−0.114112
H	5.679451	−1.989790	0.743930
H	−0.089903	−3.360892	2.637423
H	1.366447	−2.379265	2.946902
H	−0.220620	−1.588626	2.704888
H	−1.928819	−3.308358	−2.877810
H	−3.180596	−3.002731	−4.106257
H	−1.818557	−1.871187	−3.932954
H	−5.394899	−1.865600	0.561019
H	−6.626242	−1.435800	−0.652998
H	−5.219306	−2.420073	−1.129381
H	2.371496	−3.990852	0.634832
H	3.879341	−3.548498	−0.213356
H	3.952880	−4.270733	1.413027

ωB97X Energy = −1498.55501614 a.u.

(*aR*,3*S*,3'*S*)-**28**, Conf J

C	−1.405459	0.855678	−1.437970
C	−2.781878	1.012455	−1.516104
C	−3.588474	0.412370	−0.542388
C	−3.016829	−0.316233	0.488897
C	−1.629984	−0.457409	0.576963
C	−0.829665	0.125406	−0.401226
C	−1.035920	−1.244991	1.720116
C	−2.040440	−1.455333	2.844102
C	−1.570224	−2.467626	3.863883
O	−3.269357	−1.924233	2.300128
C	1.278520	−1.104858	−0.919330
C	2.671780	−1.225423	−0.896101
C	3.427935	−0.215564	−0.302598
C	2.817360	0.885486	0.284970
C	1.430120	0.995603	0.268246
C	0.658879	0.006753	−0.348001
C	0.778259	2.191633	0.921363
C	1.741486	2.930824	1.838984
C	1.208859	4.275495	2.279231
O	2.971675	3.151831	1.157142
C	3.673303	1.947762	0.935072
C	−3.918517	−0.932176	1.533610
O	4.800017	−0.300054	−0.267792
C	5.432396	−0.080504	−1.530481
O	0.481244	−2.018256	−1.547494
C	0.613470	−3.387678	−1.176779
O	−3.424145	1.723501	−2.479792
C	−2.633571	2.346231	−3.478276
O	−4.951972	0.580141	−0.553398
C	−5.625476	−0.125861	−1.594525
O	3.268573	−2.284280	−1.529749
C	3.976611	−3.187531	−0.678238
H	−0.757195	1.304417	−2.179868
H	−0.700069	−2.225278	1.360618
H	−0.150641	−0.734841	2.111261
H	−2.228811	−0.490717	3.340993
H	−2.303084	−2.576559	4.664361
H	−1.428167	−3.440684	3.388057
H	−0.620379	−2.153410	4.300994
H	−0.101141	1.875435	1.489586
H	0.417952	2.883600	0.151737
H	1.945342	2.307354	2.723601
H	1.059947	4.921656	1.411230
H	1.908192	4.763080	2.959487
H	0.251158	4.155896	2.789473
H	4.074893	1.567027	1.885743
H	4.524391	2.193440	0.299633
H	−4.305984	−0.142882	2.194271
H	−4.776975	−1.409821	1.061505
H	5.192098	0.917309	−1.909595
H	6.504276	−0.154028	−1.357424
H	5.122076	−0.832262	−2.259063
H	0.773399	−3.484515	−0.098292
H	−0.327927	−3.867599	−1.439567

H	1.433779	−3.866460	−1.711679
H	−1.947947	3.078254	−3.041821
H	−2.063090	1.609159	−4.050559
H	−3.330695	2.855932	−4.139096
H	−5.435086	−1.200855	−1.513469
H	−5.308475	0.231390	−2.575849
H	−6.688971	0.063606	−1.460820
H	4.351651	−3.982632	−1.320005
H	4.809003	−2.688332	−0.180079
H	3.302950	−3.612928	0.071772

ωB97X Energy = −1498.55499976 a.u.

(*aR*,3*S*,3'*S*)-**28**, Conf K

C	−1.348116	0.984931	−1.253936
C	−2.725487	1.123083	−1.317302
C	−3.527240	0.368132	−0.463335
C	−2.943644	−0.493085	0.461464
C	−1.557500	−0.611642	0.539046
C	−0.755846	0.123981	−0.338838
C	−0.950233	−1.547728	1.555024
C	−1.955528	−1.964747	2.618440
C	−1.463252	−3.116274	3.465244
O	−3.166940	−2.374050	1.991472
C	1.350940	−1.116105	−0.822294
C	2.740545	−1.242344	−0.813524
C	3.512800	−0.214923	−0.282868
C	2.908195	0.922223	0.245379
C	1.520739	1.034231	0.251987
C	0.733248	0.015768	−0.299412
C	0.886448	2.260600	0.866098
C	1.868040	3.031162	1.737201
C	1.345648	4.393466	2.133265
O	3.084908	3.222375	1.023364
C	3.778644	2.008281	0.834014
C	−3.836452	−1.291871	1.382873
O	4.877844	−0.328030	−0.210010
C	5.546398	−0.257225	−1.470449
O	0.592164	−2.153790	−1.301878
C	0.484382	−2.180858	−2.724126
O	−3.271717	1.971371	−2.249576
C	−3.863816	3.145909	−1.695503
O	−4.896501	0.484767	−0.475542
C	−5.526738	−0.042335	−1.644463
O	3.341200	−2.354591	−1.342915
C	3.356429	−3.469081	−0.449951
H	−0.739157	1.571741	−1.933652
H	−0.575464	−2.440194	1.041251
H	−0.088346	−1.072623	2.032892
H	−2.177964	−1.100474	3.263687
H	−2.196301	−3.372480	4.231092
H	−1.292167	−3.994759	2.839103
H	−0.523976	−2.852846	3.955559
H	0.015011	1.971213	1.460188
H	0.519323	2.926235	0.077044
H	2.087111	2.442399	2.641537
H	1.182026	5.004373	1.242638
H	2.057598	4.906719	2.780677
H	0.397230	4.296149	2.665199
H	4.200828	1.664041	1.789427
H	4.615569	2.224524	0.169588
H	−4.255370	−0.627760	2.153273
H	−4.674777	−1.712168	0.827364
H	5.226684	−1.069453	−2.125227
H	5.351712	0.706284	−1.951778
H	6.610678	−0.348699	−1.262379
H	−0.005059	−1.271853	−3.087233
H	1.470256	−2.282298	−3.184595
H	−0.127453	−3.045260	−2.975034
H	−4.684443	2.890999	−1.021135
H	−3.113625	3.732388	−1.156551
H	−4.243419	3.727428	−2.533499
H	−5.302182	−1.107780	−1.751829
H	−6.597792	0.089459	−1.503841
H	−5.201485	0.491954	−2.539189
H	3.854920	−4.282975	−0.973089
H	3.913709	−3.222472	0.458111
H	2.337041	−3.767514	−0.193135

ωB97X Energy = −1498.55479398 a.u.

(*aR*,3*S*,3'*S*)-**28**, Conf L

C	−1.308241	−1.235058	−1.195049
C	−2.680762	−1.388916	−1.323008
C	−3.523125	−0.726886	−0.422722
C	−2.990378	0.040526	0.601189
C	−1.607206	0.192946	0.732192
C	−0.769880	−0.451557	−0.176811
C	−1.067544	1.058544	1.847229
C	−2.140011	1.977863	2.412543
C	−1.701256	2.676965	3.679234
O	−3.299506	1.211788	2.719260
C	1.468808	−1.311530	0.506792
C	2.860940	−1.205605	0.611516
C	3.491686	−0.068476	0.113939
C	2.745090	0.935145	−0.504980
C	1.364460	0.820303	−0.608745
C	0.715401	−0.309433	−0.093668
C	0.586383	1.913294	−1.304304
C	1.399761	3.191369	−1.442649
C	0.751843	4.194370	−2.370178
O	2.684280	2.880316	−1.969224
C	3.466303	2.144356	−1.053419
C	−3.938804	0.723943	1.559898
O	4.833284	0.157751	0.247039
C	5.729849	−0.848255	−0.220394
O	0.849329	−2.441487	0.980927
C	0.681240	−2.477282	2.397179
O	−3.287361	−2.150003	−2.270836
C	−2.458474	−2.881944	−3.158199
O	−4.886307	−0.877070	−0.493302
C	−5.498902	−0.213431	−1.596882

O	3.593166	−2.160615	1.267513
C	3.668567	−3.428799	0.612489
H	−0.632971	−1.733047	−1.878873
H	−0.683849	0.433600	2.661909
H	−0.223040	1.655992	1.493353
H	−2.409785	2.726601	1.650919
H	−2.482077	3.347552	4.040032
H	−1.487771	1.940924	4.457520
H	−0.796543	3.260423	3.497519
H	−0.339424	2.126663	−0.763778
H	0.289807	1.570679	−2.302398
H	1.533931	3.641812	−0.446417
H	0.662320	3.772165	−3.373602
H	1.346962	5.106464	−2.428113
H	−0.247191	4.452044	−2.012840
H	3.782469	2.794004	−0.224016
H	4.367005	1.836455	−1.583941
H	−4.451059	1.548565	1.041013
H	−4.704451	0.023591	1.892050
H	5.388914	−1.253267	−1.177901
H	6.691078	−0.357193	−0.361991
H	5.832780	−1.654236	0.505806
H	−0.009518	−1.693516	2.720940
H	0.253416	−3.449483	2.634399
H	1.640348	−2.361699	2.907599
H	−1.815477	−3.578301	−2.612737
H	−3.130134	−3.441490	−3.804848
H	−1.840479	−2.215484	−3.766835
H	−5.145348	−0.624509	−2.544284
H	−6.570527	−0.379240	−1.503091
H	−5.294056	0.861395	−1.560351
H	2.678562	−3.873608	0.505563
H	4.132776	−3.322767	−0.372867
H	4.293781	−4.061247	1.240107

ωB97X Energy = −1498.55459787 a.u.

(*aR*,3*S*,3'*S*)-**28**, Conf M

C	−1.391363	0.894494	−1.412447
C	−2.769888	0.970067	−1.552096
C	−3.579329	0.194416	−0.714848
C	−3.009403	−0.657992	0.218143
C	−1.621784	−0.731155	0.359348
C	−0.816989	0.049608	−0.465574
C	−1.032556	−1.652345	1.399693
C	−2.071364	−2.085729	2.423611
C	−1.583403	−3.207825	3.311584
O	−3.236902	−2.543473	1.747143
C	1.392638	−1.002280	−0.962055
C	2.789017	−1.027505	−0.883423
C	3.445983	−0.013525	−0.187437
C	2.735161	0.996192	0.448794
C	1.345222	1.011686	0.375797
C	0.672188	0.021206	−0.344160
C	0.584047	2.108745	1.083352
C	1.454378	2.840592	2.094908
C	0.810973	4.108982	2.607251
O	2.690791	3.196968	1.485782
C	3.486274	2.064694	1.208654
C	−3.916762	−1.496182	1.089437
O	4.818285	−0.001904	−0.100022
C	5.480083	0.345089	−1.318349
O	0.686447	−1.916507	−1.687948
C	0.937307	−3.300977	−1.461150
O	−3.412524	1.750668	−2.460072
C	−2.618175	2.530062	−3.338252
O	−4.946794	0.212344	−0.845359
C	−5.570126	1.406195	−0.377893
O	3.486350	−1.990287	−1.565733
C	4.232319	−2.900133	−0.755131
H	−0.741313	1.484359	−2.046177
H	−0.625284	−2.547523	0.914888
H	−0.197233	−1.161745	1.908081
H	−2.349043	−1.218384	3.043223
H	−2.342666	−3.475549	4.047334
H	−1.357015	−4.090710	2.709313
H	−0.676455	−2.907130	3.839899
H	−0.293057	1.690656	1.585560
H	0.207810	2.831426	0.350554
H	1.667481	2.167262	2.939806
H	0.649044	4.806894	1.782682
H	1.448654	4.590928	3.349315
H	−0.153797	3.887434	3.067670
H	3.878614	1.644372	2.146409
H	4.340415	2.415430	0.629226
H	−4.413532	−0.850549	1.829613
H	−4.696661	−1.956562	0.483855
H	5.162630	1.336188	−1.656109
H	6.545863	0.361453	−1.098854
H	5.273020	−0.390038	−2.098807
H	1.131250	−3.492220	−0.401071
H	0.030363	−3.826559	−1.756331
H	1.780263	−3.653574	−2.055152
H	−2.006862	3.250549	−2.786877
H	−1.971076	1.897905	−3.952712
H	−3.314667	3.064849	−3.979623
H	−5.238528	2.271819	−0.954549
H	−5.352575	1.563967	0.683435
H	−6.641924	1.269151	−0.509219
H	4.685679	−3.616735	−1.437520
H	5.008976	−2.380321	−0.192158
H	3.569716	−3.427800	−0.062574

ωB97X Energy = −1498.55458641 a.u.

(*aR*,3*S*,3'*S*)-**28**, Conf N

C	−1.348557	0.936778	−1.287060
C	−2.727534	1.039699	−1.377637
C	−3.525551	0.307892	−0.500857
C	−2.939462	−0.548304	0.427119
C	−1.552623	−0.647332	0.517805
C	−0.752646	0.103361	−0.349116

C	−0.943772	−1.564399	1.549716
C	−1.952313	−1.971234	2.613766
C	−1.452825	−3.097890	3.489282
O	−3.152738	−2.409047	1.985397
C	1.386238	−1.099421	−0.782251
C	2.777842	−1.196132	−0.752416
C	3.520832	−0.144727	−0.226850
C	2.885136	0.987803	0.274108
C	1.495623	1.071199	0.258601
C	0.737547	0.027343	−0.286455
C	0.826656	2.294566	0.840967
C	1.778708	3.104307	1.709439
C	1.222203	4.463975	2.066056
O	3.002471	3.305152	1.010170
C	3.723236	2.101875	0.857411
C	−3.831896	−1.351498	1.345710
O	4.886734	−0.228627	−0.132866
C	5.571611	−0.163398	−1.384896
O	0.656429	−2.160521	−1.255664
C	0.565710	−2.210225	−2.678098
O	−3.277122	1.890371	−2.305446
C	−3.942228	1.230452	−3.382452
O	−4.897048	0.367500	−0.563788
C	−5.457194	1.606233	−0.126755
O	3.408345	−2.303440	−1.256981
C	3.442451	−3.400923	−0.343835
H	−0.741620	1.520379	−1.971317
H	−0.560690	−2.462189	1.051723
H	−0.087861	−1.076143	2.024845
H	−2.190548	−1.095967	3.238449
H	−2.189734	−3.347888	4.253510
H	−1.263825	−3.987172	2.883859
H	−0.522037	−2.811114	3.982644
H	−0.045913	1.997189	1.429409
H	0.454752	2.935991	0.034457
H	1.995790	2.540438	2.629964
H	1.057856	5.049823	1.158904
H	1.914095	5.007544	2.710529
H	0.268665	4.358904	2.587230
H	4.133972	1.784677	1.827090
H	4.568263	2.321985	0.204612
H	−4.279653	−0.684104	2.097936
H	−4.648887	−1.796714	0.778768
H	5.364175	0.788513	−1.883620
H	6.634496	−0.229739	−1.160766
H	5.277573	−0.992013	−2.031175
H	1.558879	−2.293914	−3.126325
H	−0.022067	−3.092737	−2.923420
H	0.058769	−1.318397	−3.059768
H	−3.242735	0.587359	−3.924839
H	−4.782068	0.634223	−3.018879
H	−4.305650	2.010770	−4.048463
H	−5.120839	2.431676	−0.757462
H	−6.537690	1.500617	−0.202541
H	−5.183884	1.803234	0.914256
H	3.988794	−3.125964	0.562714
H	2.428389	−3.717279	−0.087343
H	3.961515	−4.213105	−0.849357

ωB97X Energy = −1498.55454131 a.u.

(*aR*,3*S*,3'*S*)-**28**, Conf O

C	1.336540	0.743974	1.351975
C	2.714340	0.851193	1.478997
C	3.530505	0.254980	0.511823
C	2.968930	−0.441605	−0.547750
C	1.582747	−0.567847	−0.663070
C	0.770781	0.037899	0.292015
C	1.001462	−1.333509	−1.827512
C	2.028988	−2.268859	−2.454460
C	2.273423	−3.521811	−1.624281
O	3.229704	−1.539669	−2.715261
C	−1.378934	−1.180806	0.633074
C	−2.770102	−1.269778	0.577362
C	−3.500847	−0.192945	0.086567
C	−2.852978	0.957763	−0.353196
C	−1.463579	1.034193	−0.309828
C	−0.718776	−0.035298	0.200678
C	−0.778327	2.280611	−0.819168
C	−1.710225	3.134994	−1.666148
C	−1.142292	4.507785	−1.946039
O	−2.946980	3.307345	−0.981553
C	−3.675242	2.101333	−0.900722
C	3.891762	−1.096138	−1.548705
O	−4.865374	−0.267355	−0.034047
C	−5.570890	−0.254990	1.208042
O	−0.659723	−2.270266	1.055857
C	−0.572082	−2.390689	2.474047
O	3.348530	1.494591	2.494375
C	2.548016	2.086616	3.503050
O	4.898727	0.291342	0.622667
C	5.481416	1.569377	0.373248
O	−3.410849	−2.397736	1.019794
C	−3.459790	−3.438589	0.043444
H	0.679936	1.200072	2.082482
H	0.134319	−1.911351	−1.500237
H	0.647755	−0.629319	−2.587933
H	1.681118	−2.568947	−3.444639
H	3.091113	−4.106905	−2.049564
H	2.512377	−3.286281	−0.584739
H	1.372922	−4.138918	−1.623034
H	0.104962	2.007466	−1.403433
H	−0.420364	2.879609	0.025770
H	−1.910510	2.616243	−2.616308
H	−0.994294	5.048933	−1.008711
H	−1.819178	5.084680	−2.577346
H	−0.179123	4.423420	−2.453010
H	−4.066015	1.832273	−1.892865
H	−4.533361	2.294337	−0.256451
H	4.651780	−0.387751	−1.880054
H	4.421403	−1.927106	−1.064234
H	−5.294304	−1.115405	1.819560

H	−5.364276	0.670845	1.753942
H	−6.630381	−0.301426	0.963692
H	−0.003066	−3.295905	2.677185
H	−0.047503	−1.528919	2.898627
H	−1.567888	−2.475873	2.916348
H	1.900595	2.866375	3.091342
H	3.240465	2.530739	4.214145
H	1.935909	1.337330	4.013448
H	5.237862	1.911358	−0.637846
H	5.138119	2.304286	1.103387
H	6.558707	1.441608	0.461399
H	−4.008185	−3.105881	−0.842206
H	−2.449593	−3.747512	−0.237066
H	−3.982659	−4.275219	0.503039

ωB97X Energy = −1498.55411257 a.u.

Table S6. Cartesian coordinates and energies of the low-energy conformers calculated at the B3LYP/TZVP PCM/$CHCl_3$ level.

(*aS*,1*S*,3*S*,3'*S*)-**21**, Conf A

C	2.712249	0.607559	−1.315173
C	4.007708	0.123147	−1.470156
C	4.529491	−0.743683	−0.496919
C	3.750859	−1.120607	0.594666
C	2.440153	−0.645931	0.738946
C	1.926304	0.230966	−0.222560
C	1.624966	−1.067491	1.941408
C	2.211196	−2.293073	2.637603
C	1.588584	−2.560873	3.994542
O	3.617790	−2.099237	2.835934
C	0.282540	1.876874	0.697133
C	−1.000252	2.433236	0.776869
C	−2.032376	1.888415	0.010944
C	−1.805132	0.762863	−0.787839
C	−0.523550	0.207475	−0.859264
C	0.534566	0.775463	−0.125553
C	−0.271861	−0.976818	−1.769332
C	−1.558203	−1.647065	−2.238617
C	−1.343961	−2.588592	−3.408685
O	−2.490355	−0.640451	−2.662194
C	−2.959252	0.181553	−1.584976
C	4.334224	−2.080185	1.610311
C	−4.002121	−0.568290	−0.755665
C	−3.766236	−1.029426	0.538252
C	−4.737401	−1.744047	1.236658
C	−5.948026	−1.986682	0.616959
C	−6.225140	−1.544992	−0.666046
C	−5.242046	−0.835434	−1.343939
F	−6.904540	−2.678823	1.291490
O	4.823968	0.422775	−2.522945
C	4.330999	1.295382	−3.536720
O	5.782212	−1.302435	−0.631557
C	6.893636	−0.401980	−0.492888
O	−1.226039	3.528618	1.579512
C	−1.704351	3.209482	2.897663
O	−3.308752	2.396457	0.083525
C	−3.490635	3.720080	−0.451503
O	1.287252	2.369819	1.496099
C	1.834341	3.637072	1.098292
H	2.291832	1.277287	−2.051910
H	1.579638	−0.239643	2.657582
H	0.594747	−1.278264	1.643160
H	2.073890	−3.173789	1.992284
H	1.762566	−1.716683	4.665280
H	0.510976	−2.707265	3.897044
H	2.018667	−3.456021	4.446113
H	0.359269	−1.711440	−1.263696
H	0.292907	−0.642655	−2.646356
H	−2.001262	−2.206191	−1.405109
H	−0.960666	−2.042188	−4.273242
H	−2.281452	−3.069568	−3.690821
H	−0.623298	−3.365153	−3.144474
H	−3.475369	1.001956	−2.085367
H	4.367607	−3.093206	1.182583
H	5.358048	−1.802587	1.856677
H	−2.818900	−0.824421	1.019604
H	−4.561831	−2.102366	2.242521
H	−7.188346	−1.750930	−1.114140
H	−5.442204	−0.485385	−2.349687
H	3.448752	0.876440	−4.027810
H	5.135477	1.390698	−4.261827
H	4.089199	2.281488	−3.131067
H	6.868554	0.092204	0.482475
H	7.790109	−1.014770	−0.564699
H	6.894810	0.346061	−1.285500
H	−0.973571	2.600152	3.433997
H	−2.661112	2.685478	2.845087
H	−1.835904	4.158357	3.413900
H	−4.551366	3.940685	−0.352440
H	−3.210782	3.745480	−1.508144
H	−2.905047	4.451930	0.104004
H	2.289432	3.561760	0.107194
H	1.066850	4.412013	1.099809
H	2.602389	3.879182	1.830117

B3LYP Energy = −1829.33666153 a.u.

(*aS*,1*S*,3*S*,3'*S*)-**21**, Conf B

C	2.732486	0.475033	−1.361297
C	4.050895	0.029751	−1.386458
C	4.571032	−0.616991	−0.254647
C	3.786388	−0.773196	0.885420
C	2.458281	−0.326771	0.907827
C	1.934594	0.303444	−0.226702
C	1.628346	−0.529860	2.156263
C	2.248558	−1.558263	3.098204
C	1.595209	−1.582366	4.466952
O	3.636063	−1.251910	3.284405
C	0.224141	2.040145	0.325962
C	−1.078413	2.554137	0.288443
C	−2.080553	1.834741	−0.364186
C	−1.801380	0.586859	−0.932024
C	−0.501621	0.073789	−0.883246
C	0.525186	0.807852	−0.260930
C	−0.197008	−1.252749	−1.547764
C	−1.452202	−2.045566	−1.896507
C	−1.184978	−3.177271	−2.870895
O	−2.413200	−1.171795	−2.508138
C	−2.924066	−0.177105	−1.610142
C	4.394376	−1.443494	2.100066

C	−3.949730	−0.792233	−0.658809
C	−5.185586	−1.180417	−1.184536
C	−6.149829	−1.788101	−0.390351
C	−5.857629	−2.004006	0.946008
C	−4.650710	−1.634283	1.507513
C	−3.698967	−1.024835	0.692500
F	−6.795100	−2.595409	1.733528
O	4.900904	0.183645	−2.443689
C	4.425386	0.855532	−3.607537
O	5.884783	−1.031418	−0.208992
C	6.223688	−2.148184	−1.045580
O	−1.360177	3.773156	0.862413
C	−1.688928	3.707522	2.261066
O	−3.376565	2.292937	−0.396943
C	−3.605858	3.493001	−1.157219
O	1.199277	2.719579	1.017723
C	1.712315	3.896748	0.373035
H	2.312093	0.972916	−2.223551
H	1.525876	0.421846	2.688857
H	0.617699	−0.847895	1.887126
H	2.174804	−2.556797	2.641229
H	1.703085	−0.612049	4.956495
H	0.530444	−1.806625	4.376923
H	2.055432	−2.342196	5.100152
H	0.449039	−1.855061	−0.904341
H	0.371713	−1.071354	−2.466036
H	−1.887708	−2.458703	−0.978157
H	−0.803566	−2.784580	−3.816025
H	−2.100353	−3.735460	−3.071473
H	−0.444039	−3.865837	−2.459543
H	−3.462244	0.513055	−2.260984
H	4.516691	−2.520128	1.907823
H	5.385043	−1.036757	2.298496
H	−5.397814	−1.007012	−2.232937
H	−7.110055	−2.086081	−0.790526
H	−4.463152	−1.816094	2.557637
H	−2.755130	−0.721477	1.126480
H	5.260546	0.867696	−4.303577
H	4.128354	1.882902	−3.380846
H	3.584092	0.323950	−4.060584
H	5.607253	−3.016942	−0.797343
H	6.104334	−1.900326	−2.100473
H	7.267533	−2.375998	−0.838532
H	−0.848856	3.312230	2.835612
H	−2.574634	3.087900	2.419237
H	−1.897650	4.728355	2.574910
H	−3.291682	3.352532	−2.195050
H	−3.077273	4.340344	−0.722094
H	−4.679267	3.667417	−1.124530
H	2.455861	4.313582	1.049242
H	2.191013	3.634791	−0.574008
H	0.919198	4.625802	0.201824

B3LYP Energy = −1829.33643339 a.u.

(*aS*,1*S*,3*S*,3'*S*)-**21**, Conf C

C	−2.846642	0.885597	1.063854
C	−4.143816	0.406915	1.215821
C	−4.555901	−0.693754	0.447497
C	−3.668015	−1.302847	−0.435333
C	−2.354125	−0.831865	−0.571279
C	−1.951055	0.278139	0.180446
C	−1.417697	−1.523299	−1.539779
C	−1.917338	−2.910685	−1.934566
C	−1.151525	−3.510733	−3.098400
O	−3.295135	−2.825554	−2.318689
C	−0.328906	1.922200	−0.778884
C	0.941132	2.513614	−0.846694
C	1.993895	1.966606	−0.111453
C	1.769054	0.883459	0.747273
C	0.488443	0.335890	0.857085
C	−0.567386	0.841472	0.075433
C	0.233083	−0.769967	1.859618
C	1.515642	−1.410405	2.379195
C	1.296651	−2.251493	3.622845
O	2.458337	−0.383666	2.718570
C	2.923033	0.366764	1.588066
C	−4.138197	−2.509002	−1.220569
C	3.973636	−0.425499	0.812420
C	5.262668	−0.518789	1.345351
C	6.251248	−1.267982	0.719553
C	5.929536	−1.928895	−0.454085
C	4.669445	−1.863579	−1.017324
C	3.694569	−1.103538	−0.374229
F	6.889874	−2.662380	−1.076508
O	−5.064034	0.927948	2.078680
C	−4.683806	2.038757	2.887973
O	−5.809179	−1.247040	0.599996
C	−6.911565	−0.447392	0.140933
O	1.151883	3.624966	−1.633253
C	0.798998	4.868534	−1.001523
O	3.240836	2.543006	−0.107142
C	3.951768	2.591060	−1.357541
O	−1.383597	2.472839	−1.467105
C	−1.320347	2.411024	−2.902031
H	−2.510726	1.736963	1.638275
H	−1.309576	−0.921469	−2.449098
H	−0.417882	−1.606799	−1.107094
H	−1.842747	−3.580600	−1.064731
H	−1.254873	−2.882906	−3.986105
H	−0.090306	−3.592695	−2.855122
H	−1.528844	−4.506773	−3.333803
H	−0.406644	−1.540277	1.424062
H	−0.325293	−0.355831	2.706768
H	1.950225	−2.041619	1.593878
H	0.913015	−1.634722	4.438504
H	2.232686	−2.709288	3.945407
H	0.574863	−3.045991	3.422476
H	3.429454	1.223912	2.032157
H	−4.216340	−3.377045	−0.549557
H	−5.130072	−2.334336	−1.635041
H	5.498555	0.002542	2.265569

H	7.252041	−1.339054	1.124935
H	4.459192	−2.391828	−1.938065
H	2.708272	−1.036511	−0.814898
H	−3.850484	1.782884	3.547653
H	−5.557687	2.279542	3.488357
H	−4.413445	2.903947	2.276799
H	−6.997943	0.471919	0.719689
H	−6.796185	−0.207087	−0.919766
H	−7.803989	−1.054660	0.279233
H	1.392542	5.021330	−0.097227
H	−0.264793	4.889249	−0.756523
H	1.023741	5.652620	−1.721889
H	4.075762	1.584376	−1.762877
H	4.929270	3.009178	−1.126677
H	3.435658	3.223170	−2.078272
H	−1.255818	1.373272	−3.238131
H	−2.250288	2.846669	−3.261392
H	−0.471977	2.977516	−3.285421

B3LYP Energy = −1829.33568997 a.u.

(*aS*,1*S*,3*S*,3'*S*)-**21**, Conf D

C	2.852200	0.958088	−0.992627
C	4.172889	0.532113	−1.089077
C	4.607460	−0.521619	−0.269749
C	3.739078	−1.091378	0.657743
C	2.409618	−0.656443	0.757999
C	1.968845	0.373827	−0.081307
C	1.494981	−1.312967	1.770573
C	2.042102	−2.654239	2.252253
C	1.286146	−3.214799	3.441977
O	3.409539	−2.489480	2.645870
C	0.252262	2.011136	0.716286
C	−1.038258	2.560921	0.693284
C	−2.043406	1.924528	−0.036825
C	−1.751184	0.786950	−0.799936
C	−0.450232	0.277715	−0.820090
C	0.559539	0.880979	−0.046984
C	−0.123636	−0.898550	−1.715365
C	−1.367474	−1.624994	−2.213188
C	−1.080250	−2.576361	−3.359895
O	−2.322065	−0.663421	−2.683689
C	−2.853650	0.174031	−1.646550
C	4.258752	−2.203770	1.544584
C	−3.931099	−0.568985	−0.859709
C	−3.705812	−1.138657	0.393214
C	−4.703190	−1.857901	1.048712
C	−5.931449	−1.992101	0.430165
C	−6.199935	−1.438829	−0.810637
C	−5.189510	−0.729207	−1.447405
F	−6.913680	−2.685763	1.063929
O	5.101559	1.073417	−1.929892
C	4.715188	2.173579	−2.750784
O	5.914170	−0.958633	−0.297319
C	6.324455	−1.647576	−1.488733
O	−1.317588	3.719067	1.384898
C	−0.927355	4.924040	0.701901
O	−3.306277	2.456081	−0.133349
C	−4.067510	2.579019	1.081731
O	1.259783	2.646613	1.401772
C	1.141888	2.677121	2.834128
H	2.493455	1.762678	−1.618370
H	1.368243	−0.659916	2.641347
H	0.498688	−1.456963	1.345978
H	2.004812	−3.376983	1.423117
H	1.355145	−2.532795	4.292219
H	0.231745	−3.352035	3.194079
H	1.698643	−4.179921	3.739208
H	0.525726	−1.604520	−1.193523
H	0.448183	−0.540051	−2.578798
H	−1.814041	−2.189212	−1.384747
H	−0.691734	−2.028975	−4.221421
H	−1.988926	−3.098353	−3.662557
H	−0.338555	−3.319309	−3.059847
H	−3.349650	0.979311	−2.188976
H	4.408240	−3.114803	0.945979
H	5.226452	−1.932001	1.964038
H	−2.745592	−1.015865	0.877174
H	−4.534551	−2.301659	2.021174
H	−7.177403	−1.560721	−1.258606
H	−5.383806	−0.292323	−2.419839
H	3.920558	1.892522	−3.447187
H	5.604613	2.450188	−3.311603
H	4.387752	3.023792	−2.146916
H	7.341990	−1.987824	−1.306505
H	5.680988	−2.512252	−1.674927
H	6.308072	−0.983312	−2.352831
H	−1.446945	5.007049	−0.255508
H	0.152337	4.946091	0.542494
H	−1.219183	5.749607	1.347968
H	−5.049041	2.939443	0.781212
H	−3.602644	3.286774	1.766045
H	−4.172340	1.604673	1.564071
H	1.097426	1.661767	3.235376
H	2.042149	3.167428	3.198337
H	0.260629	3.237156	3.146523

B3LYP Energy = −1829.33532556 a.u.

(*aS*,1*S*,3*S*,3'*S*)-**21**, Conf E

C	2.714087	−0.473605	−1.387057
C	4.008359	−0.936474	−1.170983
C	4.536681	−0.892812	0.128876
C	3.763687	−0.412223	1.182918
C	2.452609	0.032830	0.966623
C	1.933607	0.009980	−0.332975
C	1.639970	0.544226	2.135714
C	2.231752	0.125045	3.478884
C	1.608887	0.851498	4.655900
O	3.636916	0.409041	3.487379
C	0.281193	1.840426	−0.742460
C	−1.004473	2.317615	−1.043429

C	−2.036018	1.397727	−1.255907
C	−1.787202	0.021391	−1.144322
C	−0.508364	−0.441351	−0.831232
C	0.541617	0.474605	−0.629227
C	−0.253567	−1.931149	−0.734848
C	−1.539428	−2.743678	−0.643045
C	−1.324193	−4.226004	−0.885245
O	−2.464106	−2.276839	−1.635769
C	−2.933710	−0.942208	−1.399180
C	4.354255	−0.411255	2.577352
C	−4.019106	−0.943014	−0.323847
C	−3.782300	−0.570495	0.999113
C	−4.787598	−0.646416	1.960950
C	−6.035117	−1.097167	1.573836
C	−6.315228	−1.472597	0.270813
C	−5.296476	−1.392251	−0.670644
F	−7.024988	−1.169361	2.503246
O	4.818018	−1.451366	−2.142367
C	4.309151	−1.547598	−3.470799
O	5.789139	−1.394074	0.411204
C	6.899422	−0.651453	−0.118866
O	−1.234465	3.659191	−1.243945
C	−1.123830	4.508037	−0.086618
O	−3.293568	1.752857	−1.663377
C	−3.997940	2.804698	−0.987635
O	1.298693	2.746469	−0.532031
C	1.879511	3.298242	−1.725563
H	2.290013	−0.494696	−2.380857
H	1.591138	1.637928	2.096918
H	0.610719	0.182968	2.064006
H	2.099902	−0.959767	3.608278
H	1.776582	1.927363	4.570423
H	0.532381	0.671887	4.687772
H	2.043978	0.507297	5.595239
H	0.379244	−2.147238	0.129260
H	0.308376	−2.260857	−1.615266
H	−1.986791	−2.605711	0.349335
H	−0.936910	−4.396240	−1.892160
H	−2.262132	−4.772586	−0.778368
H	−0.606440	−4.627651	−0.167112
H	−3.410606	−0.661720	−2.338690
H	4.397501	−1.441917	2.959430
H	5.375309	−0.032670	2.561133
H	−2.805399	−0.207014	1.290703
H	−4.610243	−0.359057	2.989073
H	−7.307287	−1.814456	0.006306
H	−5.499389	−1.684945	−1.694089
H	5.105536	−1.994774	−4.060614
H	4.065992	−0.562683	−3.878513
H	3.424354	−2.188276	−3.512061
H	6.885333	−0.647598	−1.208572
H	6.887644	0.376223	0.255219
H	7.796912	−1.154717	0.235459
H	−0.121594	4.464827	0.337450
H	−1.858294	4.220859	0.670457
H	−1.337357	5.517166	−0.432988
H	−3.674780	3.783716	−1.335760
H	−3.861848	2.730210	0.093637
H	−5.049498	2.655771	−1.225479
H	1.128512	3.821478	−2.320133
H	2.642799	4.000570	−1.396548
H	2.344784	2.510911	−2.323433

B3LYP Energy = −1829.33509463 a.u.

(*aS*,1*S*,3*S*,3'*S*)-**21**, Conf F

C	2.740092	−0.258553	−1.428538
C	4.058259	−0.663048	−1.240200
C	4.585619	−0.673148	0.060324
C	3.810078	−0.242792	1.134256
C	2.483689	0.166280	0.942157
C	1.950402	0.155694	−0.352051
C	1.666480	0.614296	2.133394
C	2.287591	0.173385	3.455904
C	1.647511	0.831569	4.663467
O	3.679004	0.517032	3.461448
C	0.222251	1.930996	−0.683826
C	−1.083993	2.365702	−0.959091
C	−2.078717	1.412494	−1.198333
C	−1.773950	0.044404	−1.138733
C	−0.475224	−0.376837	−0.849398
C	0.538109	0.573381	−0.621131
C	−0.160542	−1.857671	−0.807365
C	−1.413146	−2.721908	−0.730473
C	−1.143336	−4.187070	−1.018573
O	−2.362706	−2.262039	−1.702387
C	−2.882703	−0.954739	−1.422234
C	4.425305	−0.237030	2.518572
C	−3.964145	−1.031897	−0.345748
C	−3.739711	−0.688211	0.987074
C	−4.739332	−0.831532	1.947167
C	−5.968736	−1.320261	1.548324
C	−6.236148	−1.669376	0.235298
C	−5.223189	−1.521863	−0.704281
F	−6.953214	−1.457818	2.476039
O	4.900119	−1.051165	−2.242491
C	4.412933	−1.035416	−3.582309
O	5.898040	−1.021375	0.296406
C	6.224772	−2.407850	0.114538
O	−1.371716	3.702067	−1.111561
C	−1.278964	4.518392	0.070562
O	−3.352163	1.733684	−1.585080
C	−4.098575	2.711354	−0.845900
O	1.204625	2.868903	−0.449131
C	1.755735	3.482314	−1.626693
H	2.312229	−0.250218	−2.420916
H	1.581344	1.706391	2.130142
H	0.649224	0.220877	2.059911
H	2.201987	−0.920005	3.548067
H	1.769919	1.915729	4.614352
H	0.579763	0.606175	4.698961
H	2.106574	0.473406	5.586050

H	0.490155	−2.077895	0.042325
H	0.403375	−2.134380	−1.704619
H	−1.857256	−2.630158	0.268727
H	−0.755888	−4.311704	−2.032129
H	−2.059224	−4.771902	−0.923776
H	−0.406634	−4.582765	−0.316427
H	−3.372960	−0.663075	−2.351487
H	4.539825	−1.271061	2.877254
H	5.420089	0.205308	2.487764
H	−2.777935	−0.293315	1.287940
H	−4.571660	−0.566460	2.982888
H	−7.214513	−2.042263	−0.038139
H	−5.416309	−1.793111	−1.735493
H	3.569877	−1.719996	−3.708622
H	5.242330	−1.366337	−4.202507
H	4.113479	−0.028563	−3.884966
H	7.269149	−2.513212	0.401895
H	5.606044	−3.037533	0.760374
H	6.098091	−2.709142	−0.925514
H	−0.269963	4.503368	0.480132
H	−1.990525	4.178182	0.827475
H	−1.538148	5.528033	−0.241352
H	−3.820087	3.722596	−1.136049
H	−3.953668	2.578309	0.228634
H	−5.143844	2.530581	−1.089200
H	0.981706	3.998613	−2.197428
H	2.496430	4.199288	−1.278460
H	2.243840	2.733386	−2.254754

B3LYP Energy = −1829.33476331 a.u.

(*aS*,1*S*,3*S*,3'*S*)-**21**, Conf G

C	−2.734933	0.560633	1.292526
C	−4.022342	0.048911	1.425626
C	−4.518162	−0.809532	0.431709
C	−3.721572	−1.152850	−0.658049
C	−2.418336	−0.652321	−0.779687
C	−1.930765	0.217896	0.201918
C	−1.582456	−1.038388	−1.979463
C	−2.140980	−2.257810	−2.709003
C	−1.501042	−2.482360	−4.065670
O	−3.549025	−2.084386	−2.916494
C	−0.304244	1.883946	−0.707306
C	0.969641	2.468430	−0.773981
C	2.021598	1.882575	−0.063383
C	1.789834	0.790511	0.780640
C	0.505920	0.249266	0.886535
C	−0.547590	0.787312	0.124315
C	0.252268	−0.896583	1.844212
C	1.535134	−1.560647	2.332182
C	1.320909	−2.442966	3.547720
O	2.484906	−0.549341	2.700047
C	2.948136	0.232531	1.590237
C	−4.276597	−2.103657	−1.697554
C	3.981430	−0.548343	0.781294
C	5.283712	−0.642874	1.280245
C	6.256701	−1.387486	0.624943
C	5.905225	−2.043562	−0.542740
C	4.630777	−1.976975	−1.072867
C	3.672449	−1.220808	−0.401327
F	6.849695	−2.772999	−1.193186
O	−4.855133	0.313737	2.474966
C	−4.386897	1.169075	3.514681
O	−5.762095	−1.392681	0.543416
C	−6.887487	−0.509506	0.406887
O	1.157074	3.557260	−1.591941
C	1.544780	4.778646	−0.937060
O	3.294294	2.407025	−0.109758
C	3.966790	2.354221	−1.382405
O	−1.314208	2.349903	−1.515889
C	−1.973373	3.548598	−1.090276
H	−2.335520	1.225206	2.045460
H	−1.539103	−0.194990	−2.677024
H	−0.553400	−1.241172	−1.671674
H	−1.994360	−3.151033	−2.083124
H	−1.684022	−1.625639	−4.717774
H	−0.421856	−2.610983	−3.961037
H	−1.910405	−3.374375	−4.542050
H	−0.390443	−1.644810	1.374768
H	−0.301009	−0.521714	2.712075
H	1.961789	−2.166578	1.523547
H	0.948617	−1.852550	4.387818
H	2.256324	−2.918196	3.846001
H	0.591891	−3.225026	3.325774
H	3.468953	1.069134	2.056666
H	−4.293813	−3.125419	−1.290032
H	−5.303464	−1.841352	−1.947986
H	5.543165	−0.126105	2.196701
H	7.267781	−1.459130	1.003801
H	4.397083	−2.500656	−1.990530
H	2.675646	−1.151326	−0.817231
H	−5.199435	1.231965	4.234329
H	−4.159193	2.169468	3.136660
H	−3.501864	0.753362	4.003507
H	−6.861076	−0.000075	−0.560571
H	−7.773929	−1.138586	0.460033
H	−6.909590	0.226188	1.210728
H	2.520537	4.683919	−0.460545
H	0.802320	5.064338	−0.187471
H	1.583623	5.536841	−1.716194
H	4.958551	2.767535	−1.212592
H	3.441070	2.943731	−2.133297
H	4.056894	1.320756	−1.721177
H	−2.394245	3.423807	−0.089203
H	−1.293360	4.401754	−1.102830
H	−2.779098	3.721263	−1.801035

B3LYP Energy = −1829.33421666 a.u.

(*aS*,1*S*,3*S*,3'*S*)-**21**, Conf H

C	−2.761101	0.781580	1.261397
C	−4.068592	0.335058	1.424959

C	−4.581707	−0.607711	0.520337
C	−3.781481	−1.099122	−0.507738
C	−2.457264	−0.663738	−0.657632
C	−1.954356	0.293185	0.230894
C	−1.615851	−1.227221	−1.783119
C	−2.206112	−2.514538	−2.352803
C	−1.541978	−2.955956	−3.643319
O	−3.598503	−2.314990	−2.627038
C	−0.294813	1.917664	−0.696541
C	0.998376	2.461207	−0.765935
C	2.016129	1.893069	0.007315
C	1.780207	0.763068	0.792687
C	0.490608	0.227220	0.860173
C	−0.553039	0.811457	0.124602
C	0.217541	−0.950282	1.772005
C	1.493032	−1.641180	2.240665
C	1.263272	−2.583372	3.407387
O	2.439316	−0.650415	2.669465
C	2.922944	0.171450	1.598509
C	−4.358911	−2.142924	−1.440125
C	3.967613	−0.582491	0.775158
C	5.198399	−0.866731	1.374709
C	6.182378	−1.579390	0.701435
C	5.915628	−2.006837	−0.588596
C	4.714449	−1.747010	−1.219577
C	3.742145	−1.029522	−0.525534
F	6.873155	−2.701805	−1.258779
O	−4.906018	0.742442	2.423263
C	−4.420661	1.691327	3.370507
O	−5.849347	−1.128848	0.668607
C	−6.935788	−0.223025	0.415120
O	1.213610	3.602743	−1.503245
C	2.114836	3.480336	−2.619303
O	3.287082	2.428655	−0.010744
C	3.428711	3.693678	0.661923
O	−1.366688	2.479910	−1.334888
C	−1.278212	2.789829	−2.732669
H	−2.348127	1.514344	1.939931
H	−1.535502	−0.493123	−2.592962
H	−0.597478	−1.416926	−1.435199
H	−2.113717	−3.314967	−1.603340
H	−1.666859	−2.193922	−4.415747
H	−0.473445	−3.117125	−3.486806
H	−1.981403	−3.887295	−4.003260
H	−0.427051	−1.675714	1.270592
H	−0.340482	−0.602471	2.648303
H	1.929075	−2.203949	1.405645
H	0.887711	−2.033820	4.273308
H	2.192894	−3.079785	3.688879
H	0.530742	−3.347580	3.139973
H	3.441507	0.984652	2.107742
H	−4.443769	−3.103267	−0.910399
H	−5.362616	−1.862050	−1.756259
H	5.390538	−0.527487	2.385716
H	7.138533	−1.798355	1.158377
H	4.546953	−2.094828	−2.230503
H	2.802126	−0.810993	−1.015133
H	−3.558798	1.300652	3.917822
H	−5.240407	1.862568	4.063930
H	−4.150022	2.634206	2.888028
H	−7.847840	−0.807769	0.518267
H	−6.940397	0.596268	1.133949
H	−6.873316	0.176946	−0.600912
H	3.109400	3.179211	−2.292130
H	2.157755	4.465004	−3.080018
H	1.731340	2.756553	−3.343014
H	2.824100	4.464507	0.182905
H	4.482751	3.954831	0.595929
H	3.140508	3.602090	1.712268
H	−2.304704	2.825260	−3.093473
H	−0.795408	3.751547	−2.896154
H	−0.737023	2.008589	−3.271954

B3LYP Energy = −1829.33408435 a.u.

(*aS*,1*S*,3*S*,3’*S*)-**21**, Conf I

C	2.663618	−0.185141	−1.486825
C	3.972556	−0.632406	−1.358384
C	4.537248	−0.742602	−0.084315
C	3.772172	−0.443055	1.045936
C	2.445794	−0.017812	0.913202
C	1.892426	0.128712	−0.368991
C	1.643064	0.301346	2.154952
C	2.279555	−0.268605	3.419861
C	1.659621	0.273550	4.693571
O	3.673950	0.063589	3.439880
C	0.211148	1.973951	−0.481370
C	−1.083396	2.459678	−0.702930
C	−2.103191	1.566310	−1.035047
C	−1.849541	0.191580	−1.093220
C	−0.556717	−0.289154	−0.863239
C	0.487951	0.606758	−0.569252
C	−0.281272	−1.774010	−0.979742
C	−1.553806	−2.613638	−0.983631
C	−1.320115	−4.039754	−1.445602
O	−2.508841	−2.023718	−1.878307
C	−2.991943	−0.750388	−1.428898
C	4.397226	−0.601211	2.415833
C	−4.022778	−0.932902	−0.315176
C	−3.767552	−0.634819	1.022326
C	−4.726155	−0.863621	2.007346
C	−5.944947	−1.392549	1.628739
C	−6.241747	−1.699832	0.311423
C	−5.270321	−1.465482	−0.653552
F	−6.889618	−1.614184	2.581363
O	4.701901	−0.910096	−2.496352
C	4.937147	−2.306753	−2.738804
O	5.823964	−1.204780	0.097865
C	6.870617	−0.328305	−0.357582
O	−1.337216	3.810265	−0.623985
C	−1.714823	4.265307	0.686968
O	−3.391852	2.003167	−1.232361

C	−3.610568	2.836087	−2.385573
O	1.204074	2.843779	−0.096076
C	1.730207	3.685829	−1.135403
H	2.250794	−0.090777	−2.483808
H	1.553782	1.388052	2.258210
H	0.626223	−0.086526	2.052981
H	2.187797	−1.365111	3.407277
H	1.789549	1.356519	4.747717
H	0.590745	0.052682	4.721185
H	2.127405	−0.175455	5.570862
H	0.369734	−2.100231	−0.165033
H	0.269948	−1.965541	−1.906457
H	−1.981910	−2.629199	0.026401
H	−0.942919	−4.051307	−2.470582
H	−2.248392	−4.611487	−1.411127
H	−0.587436	−4.531911	−0.802759
H	−3.520908	−0.349546	−2.294370
H	4.489088	−1.670576	2.657886
H	5.402813	−0.182960	2.427643
H	−2.815003	−0.208390	1.308767
H	−4.535237	−0.632198	3.047051
H	−7.211117	−2.106478	0.054640
H	−5.486176	−1.703446	−1.688460
H	5.515588	−2.364333	−3.658737
H	3.989734	−2.836315	−2.871843
H	5.499868	−2.759709	−1.920716
H	6.822565	0.629379	0.167415
H	7.807906	−0.826198	−0.117810
H	6.803361	−0.161143	−1.432811
H	−1.888009	5.336232	0.601689
H	−0.912224	4.080627	1.404125
H	−2.631512	3.771343	1.016933
H	−3.318637	2.308380	−3.297495
H	−3.057101	3.770956	−2.305339
H	−4.679404	3.037680	−2.410450
H	0.948664	4.318587	−1.557881
H	2.491943	4.305567	−0.666782
H	2.188327	3.080427	−1.921635

B3LYP Energy = −1829.33401202 a.u.

(*aS*,1*S*,3*S*,3’*S*)-**21**, Conf J

C	2.761453	0.620096	−1.245560
C	4.073630	0.160890	−1.309361
C	4.569990	−0.624745	−0.257506
C	3.771008	−0.897255	0.850606
C	2.450942	−0.430973	0.914302
C	1.947779	0.330247	−0.146704
C	1.608301	−0.751011	2.128506
C	2.198413	−1.888740	2.957789
C	1.537103	−2.040609	4.314373
O	3.592218	−1.638256	3.179925
C	0.234689	2.000717	0.577186
C	−1.060561	2.539883	0.555430
C	−2.069693	1.852461	−0.126024
C	−1.775185	0.695569	−0.856741
C	−0.470006	0.197274	−0.878765
C	0.541371	0.844433	−0.145489
C	−0.148991	−1.026686	−1.710541
C	−1.394077	−1.789048	−2.149156
C	−1.117268	−2.788961	−3.256278
O	−2.369736	−0.860642	−2.644665
C	−2.890002	0.017829	−1.635679
C	4.352151	−1.717276	1.983957
C	−3.922363	−0.710036	−0.778044
C	−5.208741	−0.889150	−1.294673
C	−6.178066	−1.594523	−0.592274
C	−5.838683	−2.124844	0.640991
C	−4.580126	−1.971152	1.190862
C	−3.625484	−1.255698	0.471224
F	−6.779582	−2.815199	1.337702
O	4.937947	0.427240	−2.332330
C	4.485787	1.246383	−3.407788
O	5.874909	−1.068260	−0.250673
C	6.202531	−2.079763	−1.215883
O	−1.310577	3.692220	1.261955
C	−1.710269	4.834357	0.483198
O	−3.360736	2.330158	−0.163400
C	−4.068024	2.377888	1.090505
O	1.202020	2.574553	1.367812
C	1.846623	3.743259	0.847054
H	2.357326	1.219548	−2.048923
H	1.523133	0.140681	2.758873
H	0.592585	−1.014334	1.822119
H	2.103507	−2.831175	2.397283
H	1.667457	−1.130629	4.903797
H	0.467286	−2.225169	4.197973
H	1.973797	−2.875306	4.864655
H	0.512965	−1.695068	−1.154885
H	0.406927	−0.719179	−2.603004
H	−1.818224	−2.319668	−1.287912
H	−0.753321	−2.277748	−4.150176
H	−2.024487	−3.336155	−3.515796
H	−0.359751	−3.507535	−2.936962
H	−3.421456	0.782894	−2.202301
H	4.444414	−2.768462	1.671410
H	5.352908	−1.365099	2.229907
H	−5.458196	−0.470621	−2.262561
H	−7.177098	−1.731349	−0.984897
H	−4.356275	−2.396659	2.160290
H	−2.642080	−1.115656	0.901139
H	3.643379	0.787861	−3.932816
H	5.329514	1.334087	−4.087870
H	4.199090	2.241162	−3.056518
H	5.558155	−2.954710	−1.091035
H	6.112209	−1.697565	−2.232871
H	7.235377	−2.361698	−1.020649
H	−2.667635	4.666214	−0.010383
H	−0.952550	5.070720	−0.268335
H	−1.794618	5.660887	1.185480
H	−3.596355	3.071870	1.785905
H	−4.115450	1.383492	1.537615

H	−5.073949	2.716112	0.851999
H	2.617982	4.010990	1.566323
H	2.311367	3.530521	−0.118987
H	1.144572	4.572538	0.746725

B3LYP Energy = −1829.33390621 a.u.

(*aS*,1*S*,3*S*,3'*S*)-**21**, Conf K

C	−2.822472	0.929067	1.012421
C	−4.117342	0.454260	1.193245
C	−4.523664	−0.692860	0.493033
C	−3.631354	−1.352560	−0.347698
C	−2.318472	−0.886386	−0.510097
C	−1.922134	0.272097	0.169463
C	−1.377524	−1.637569	−1.429223
C	−1.873351	−3.047916	−1.738303
C	−1.102659	−3.716661	−2.860696
O	−3.250225	−2.989321	−2.130691
C	−0.310933	1.893136	−0.848351
C	0.954776	2.493997	−0.947955
C	2.013956	1.976612	−0.195564
C	1.801765	0.896876	0.672933
C	0.527436	0.343071	0.808058
C	−0.540853	0.836148	0.036159
C	0.287875	−0.740198	1.839037
C	1.578227	−1.360194	2.364128
C	1.375478	−2.163751	3.634842
O	2.521402	−0.319966	2.659824
C	2.970198	0.381507	1.492902
C	−4.095757	−2.608029	−1.055882
C	3.981618	−0.462969	0.716124
C	5.210581	−0.738770	1.323442
C	6.167320	−1.524314	0.693502
C	5.875612	−2.032676	−0.561432
C	4.675791	−1.783225	−1.198862
C	3.730665	−0.992226	−0.548238
F	6.806549	−2.800098	−1.189019
O	−5.040630	1.021562	2.023134
C	−4.664068	2.176871	2.769189
O	−5.775284	−1.240033	0.676745
C	−6.878715	−0.474417	0.165150
O	1.192204	3.518735	−1.834674
C	0.527778	4.766596	−1.560150
O	3.300404	2.437201	−0.301288
C	3.558389	3.839380	−0.132446
O	−1.360669	2.390859	−1.588910
C	−1.315973	2.129097	−3.001708
H	−2.491379	1.815649	1.534006
H	−1.265257	−1.096416	−2.375095
H	−0.379378	−1.693123	−0.989056
H	−1.800265	−3.662617	−0.828589
H	−1.204860	−3.145027	−3.785745
H	−0.041968	−3.780729	−2.610004
H	−1.477195	−4.726187	−3.035361
H	−0.350019	−1.525392	1.428614
H	−0.267049	−0.308255	2.679697
H	2.007205	−2.011802	1.592377
H	1.001036	−1.523152	4.436358
H	2.315691	−2.611385	3.959577
H	0.651969	−2.964213	3.467180
H	3.508844	1.241657	1.891937
H	−4.170870	−3.432123	−0.331204
H	−5.088095	−2.464213	−1.480953
H	5.421429	−0.336137	2.306979
H	7.121615	−1.738271	1.156713
H	4.488190	−2.194574	−2.182017
H	2.792131	−0.782213	−1.044438
H	−3.829064	1.960719	3.440798
H	−5.538243	2.447262	3.356395
H	−4.397697	3.007877	2.110659
H	−7.770242	−1.072671	0.342835
H	−6.966971	0.481013	0.681935
H	−6.763018	−0.304551	−0.909097
H	0.843741	5.163071	−0.591882
H	−0.554966	4.647748	−1.571518
H	0.834917	5.449758	−2.349242
H	2.994968	4.236771	0.715606
H	3.315749	4.398080	−1.034164
H	4.623728	3.920997	0.074918
H	−1.345652	1.054372	−3.192840
H	−2.205562	2.592990	−3.422625
H	−0.423172	2.559724	−3.456831

B3LYP Energy = −1829.33385979 a.u.

(*aS*,1*S*,3*S*,3'*S*)-**21**, Conf L

C	2.674028	0.240754	−1.462534
C	3.981102	−0.229981	−1.447883
C	4.514652	−0.731086	−0.257135
C	3.755436	−0.699347	0.915727
C	2.440931	−0.221578	0.897515
C	1.893514	0.247887	−0.307944
C	1.637700	−0.217014	2.179096
C	2.254118	−1.108914	3.253663
C	1.637620	−0.900681	4.623888
O	3.654673	−0.823453	3.360237
C	0.196171	2.048532	0.056789
C	−1.103956	2.561890	−0.030854
C	−2.113932	1.778339	−0.591381
C	−1.844547	0.472092	−1.014337
C	−0.546934	−0.039841	−0.913887
C	0.487600	0.755917	−0.386755
C	−0.254577	−1.439024	−1.415982
C	−1.518670	−2.256627	−1.654163
C	−1.270574	−3.509322	−2.473155
O	−2.471919	−1.456936	−2.369949
C	−2.974189	−0.357513	−1.598706
C	4.373324	−1.211633	2.200167
C	−4.007968	−0.847706	−0.584417
C	−3.778541	−0.882985	0.789757
C	−4.738028	−1.382821	1.668061
C	−5.931054	−1.843744	1.145923

C	−6.201854	−1.823798	−0.212081
C	−5.230424	−1.322443	−1.069167
F	−6.875972	−2.329587	1.994594
O	4.704631	−0.237697	−2.622306
C	5.765684	0.729434	−2.689141
O	5.809841	−1.200412	−0.188564
C	6.059997	−2.435907	−0.880750
O	−1.374185	3.840752	0.400758
C	−1.705633	3.937014	1.796910
O	−3.407429	2.237251	−0.669130
C	−3.636373	3.345948	−1.557629
O	1.179752	2.799987	0.656995
C	1.698599	3.882938	−0.132233
H	2.273133	0.610778	−2.398250
H	1.571448	0.807404	2.560737
H	0.613657	−0.543054	1.979653
H	2.138715	−2.162700	2.958138
H	1.791047	0.128072	4.956672
H	0.563995	−1.096702	4.592319
H	2.089520	−1.572025	5.355364
H	0.392255	−1.963503	−0.708524
H	0.307558	−1.376058	−2.353898
H	−1.954332	−2.540091	−0.687966
H	−0.890249	−3.249200	−3.463416
H	−2.193829	−4.077440	−2.594527
H	−0.535341	−4.147183	−1.978272
H	−3.504665	0.255675	−2.328377
H	4.453671	−2.308487	2.162046
H	5.382613	−0.818678	2.316061
H	−2.846033	−0.508081	1.191129
H	−4.566939	−1.410888	2.736203
H	−7.151473	−2.188483	−0.581177
H	−5.425573	−1.303611	−2.134858
H	5.364219	1.742995	−2.604852
H	6.231346	0.604676	−3.664712
H	6.504141	0.560252	−1.903746
H	5.892135	−2.327296	−1.952782
H	7.102892	−2.682917	−0.692880
H	5.421695	−3.231449	−0.487177
H	−0.873851	3.592976	2.415018
H	−2.602837	3.355157	2.020549
H	−1.895671	4.990057	1.994832
H	−4.709156	3.526300	−1.540357
H	−3.327050	3.089336	−2.574370
H	−3.103596	4.234546	−1.221275
H	2.173897	3.500301	−1.039081
H	0.908961	4.588709	−0.393307
H	2.445192	4.378246	0.485225

B3LYP Energy = −1829.33380006 a.u.

(*aS*,1*S*,3*S*,3'*S*)-**21**, Conf M

C	2.799223	0.830332	−1.212291
C	4.127001	0.421334	−1.287862
C	4.622115	−0.461262	−0.315214
C	3.803243	−0.881847	0.730088
C	2.467151	−0.464869	0.806272
C	1.968080	0.396622	−0.177086
C	1.604385	−0.950664	1.950660
C	2.206705	−2.168035	2.647279
C	1.510568	−2.516300	3.949210
O	3.583076	−1.905784	2.947017
C	0.230647	2.044790	0.544953
C	−1.081932	2.544617	0.530494
C	−2.057958	1.868798	−0.209336
C	−1.761897	0.678611	−0.876964
C	−0.453319	0.187216	−0.862233
C	0.548879	0.875366	−0.159748
C	−0.115151	−1.059571	−1.651844
C	−1.352430	−1.840886	−2.080079
C	−1.058449	−2.879134	−3.146596
O	−2.323550	−0.933129	−2.621702
C	−2.862302	−0.030331	−1.645772
C	4.382001	−1.810734	1.777133
C	−3.898661	−0.741396	−0.775537
C	−3.679099	−1.074488	0.559944
C	−4.639566	−1.762997	1.298293
C	−5.823397	−2.109488	0.676186
C	−6.084355	−1.795304	−0.647141
C	−5.112079	−1.109976	−1.364409
F	−6.769322	−2.777391	1.388870
O	5.007504	0.826339	−2.249310
C	4.561029	1.765160	−3.225385
O	5.941058	−0.861674	−0.316171
C	6.332774	−1.741949	−1.380740
O	−1.356552	3.740419	1.152470
C	−2.282704	3.687769	2.253551
O	−3.347269	2.354918	−0.272062
C	−3.517363	3.545148	−1.063928
O	1.263898	2.703707	1.153679
C	1.131779	3.126253	2.518115
H	2.396467	1.508123	−1.951289
H	1.481564	−0.151560	2.690669
H	0.602234	−1.195448	1.590226
H	2.159796	−3.031478	1.966426
H	1.589923	−1.687782	4.656392
H	0.452358	−2.719782	3.773121
H	1.960720	−3.400859	4.401968
H	0.543815	−1.708816	−1.070832
H	0.450167	−0.773658	−2.545806
H	−1.788142	−2.339322	−1.204932
H	−0.683989	−2.399088	−4.053344
H	−1.960809	−3.438020	−3.398007
H	−0.303265	−3.583785	−2.792437
H	−3.397034	0.709283	−2.242630
H	4.526345	−2.812292	1.344735
H	5.359656	−1.455042	2.099321
H	−2.753099	−0.788723	1.041427
H	−4.476367	−2.023090	2.335940
H	−7.027338	−2.078685	−1.095958
H	−5.299967	−0.858738	−2.401633
H	3.747832	1.355585	−3.830717

H	5.420486	1.961060	−3.861857
H	4.234741	2.698016	−2.758543
H	7.368571	−2.012445	−1.184697
H	5.716027	−2.645324	−1.380390
H	6.259771	−1.245940	−2.348740
H	−1.898080	3.040861	3.046105
H	−3.259651	3.329730	1.930647
H	−2.364990	4.706752	2.625771
H	−3.193555	3.369557	−2.093083
H	−2.958911	4.381921	−0.643062
H	−4.582474	3.766359	−1.051215
H	0.621192	2.365704	3.114083
H	2.147660	3.246554	2.890145
H	0.597409	4.071810	2.590939

B3LYP Energy = −1829.33377123 a.u.

(*aS*,1*S*,3*S*,3'*S*)-**21**, Conf N

C	−2.842599	1.007374	0.918144
C	−4.158136	0.569367	1.030595
C	−4.566633	−0.549446	0.287991
C	−3.677723	−1.175036	−0.582316
C	−2.353245	−0.728921	−0.698655
C	−1.938480	0.369638	0.064859
C	−1.416858	−1.447514	−1.647267
C	−1.936756	−2.831002	−2.028368
C	−1.155409	−3.470910	−3.160222
O	−3.300921	−2.718883	−2.451477
C	−0.237605	1.987658	−0.804053
C	1.050420	2.548060	−0.824926
C	2.063605	1.952612	−0.066589
C	1.782903	0.834277	0.731173
C	0.487048	0.318258	0.788175
C	−0.535196	0.891408	0.009374
C	0.174651	−0.814093	1.744378
C	1.424137	−1.509033	2.272835
C	1.149551	−2.384681	3.481127
O	2.386616	−0.521097	2.667624
C	2.902031	0.235238	1.563676
C	−4.169318	−2.360398	−1.387383
C	3.920247	−0.591834	0.777696
C	5.121039	−0.929221	1.409682
C	6.080310	−1.704762	0.771163
C	5.820030	−2.139795	−0.517738
C	4.648992	−1.827404	−1.180506
C	3.700666	−1.048056	−0.520636
F	6.753950	−2.896305	−1.154023
O	−5.105743	1.157477	1.817227
C	−4.745172	2.320410	2.559317
O	−5.867706	−1.001625	0.332048
C	−6.284080	−1.606851	1.566052
O	1.353054	3.612180	−1.642882
C	0.720418	4.864799	−1.319588
O	3.368139	2.369634	−0.097620
C	3.671934	3.756322	0.116964
O	−1.243795	2.560527	−1.549684
C	−1.149134	2.392867	−2.974564
H	−2.504172	1.862511	1.485494
H	−1.291702	−0.863542	−2.565850
H	−0.422561	−1.540134	−1.204787
H	−1.900834	−3.485886	−1.144540
H	−1.218758	−2.857092	−4.061327
H	−0.103594	−3.575134	−2.886661
H	−1.552001	−4.461154	−3.388491
H	−0.474721	−1.550484	1.266923
H	−0.394137	−0.412622	2.590936
H	1.861069	−2.124438	1.476162
H	0.765710	−1.784172	4.308859
H	2.062655	−2.883179	3.809043
H	0.408437	−3.148386	3.236418
H	3.447654	1.053235	2.035208
H	−4.315816	−3.223808	−0.721265
H	−5.134484	−2.135916	−1.839421
H	5.308303	−0.582459	2.419009
H	7.013246	−1.965614	1.253454
H	4.486375	−2.181434	−2.190065
H	2.785396	−0.788207	−1.036320
H	−5.644749	2.623502	3.089415
H	−4.423622	3.129176	1.898056
H	−3.954240	2.102945	3.282167
H	−5.635933	−2.450793	1.819184
H	−6.280807	−0.882227	2.380463
H	−7.297241	−1.967588	1.399620
H	−0.364677	4.782394	−1.368712
H	1.072874	5.579099	−2.060707
H	1.018737	5.197878	−0.322247
H	4.725912	3.788768	0.386574
H	3.077037	4.159925	0.940156
H	3.503705	4.342365	−0.784306
H	−0.248683	2.863879	−3.370701
H	−1.154702	1.332780	−3.236639
H	−2.032206	2.870874	−3.393107

B3LYP Energy = −1829.33345252 a.u.

(*aR*,1*S*,3*S*,3'*S*)-**21**, Conf A

C	−1.992368	−1.120850	−1.331123
C	−3.218995	−1.765859	−1.459860
C	−4.277929	−1.383419	−0.621599
C	−4.103064	−0.357390	0.303755
C	−2.873827	0.307027	0.415422
C	−1.811132	−0.092724	−0.401750
C	−2.722685	1.428708	1.420572
C	−4.071472	1.946947	1.913449
C	−3.954355	2.859235	3.119329
O	−4.899698	0.837030	2.286528
C	−0.186334	1.695174	−1.049580
C	1.058765	2.332915	−0.957591
C	2.056450	1.775480	−0.156158
C	1.795653	0.624059	0.598018
C	0.535633	0.022617	0.541804

C	−0.462303	0.550066	−0.299302
C	0.237862	−1.173488	1.421161
C	1.494331	−1.807217	2.007233
C	1.198929	−2.760397	3.150342
O	2.355333	−0.780123	2.518867
C	2.887780	0.083019	1.504267
C	−5.278194	0.053929	1.164782
C	4.043152	−0.598423	0.773815
C	5.284702	−0.667477	1.412382
C	6.363042	−1.320255	0.827839
C	6.179370	−1.909921	−0.411565
C	4.970478	−1.865489	−1.079390
C	3.904116	−1.201877	−0.476237
F	7.228261	−2.548838	−0.994315
O	−3.482456	−2.754234	−2.364244
C	−2.440531	−3.164164	−3.246147
O	−5.529466	−1.948031	−0.743646
C	−5.643346	−3.322049	−0.339018
O	1.304594	3.489850	−1.662662
C	0.780921	4.682288	−1.051084
O	3.272001	2.393320	0.010549
C	4.101998	2.547362	−1.154942
O	−1.186025	2.262751	−1.805556
C	−1.020187	2.169384	−3.229747
H	−1.161416	−1.404902	−1.961095
H	−2.143713	1.082629	2.284900
H	−2.159348	2.252950	0.978039
H	−4.568833	2.486259	1.093761
H	−3.512495	2.321854	3.961205
H	−3.320121	3.717138	2.887337
H	−4.936221	3.227109	3.420595
H	−0.318546	−1.926307	0.859588
H	−0.416051	−0.856897	2.241837
H	2.025754	−2.351974	1.216897
H	0.719451	−2.229443	3.975562
H	2.119500	−3.214877	3.518924
H	0.529869	−3.556408	2.817295
H	3.308441	0.918440	2.064207
H	−6.006843	0.605999	0.552961
H	−5.790158	−0.822839	1.559064
H	5.411760	−0.202531	2.382920
H	7.327964	−1.371172	1.314951
H	4.869182	−2.335692	−2.048758
H	2.957384	−1.151000	−0.998144
H	−2.871415	−3.942059	−3.871663
H	−1.588613	−3.572411	−2.695540
H	−2.105190	−2.336785	−3.877065
H	−6.696160	−3.579912	−0.435848
H	−5.045340	−3.971251	−0.978523
H	−5.334385	−3.442856	0.703265
H	−0.306730	4.632225	−0.975908
H	1.065380	5.508363	−1.699831
H	1.217752	4.828016	−0.060365
H	3.632995	3.202931	−1.886773
H	4.311687	1.573658	−1.603220
H	5.031382	2.988155	−0.800503
H	−1.882692	2.666668	−3.668715
H	−0.104051	2.666104	−3.551446
H	−1.007565	1.122569	−3.544222

B3LYP Energy = −1829.33650259 a.u.

(*aR*,1*S*,3*S*,3’*S*)-**21**, Conf B

C	−1.993171	−1.260535	−1.183498
C	−3.207569	−1.938826	−1.233169
C	−4.251522	−1.524528	−0.391779
C	−4.056649	−0.474416	0.502138
C	−2.833896	0.209278	0.548860
C	−1.798826	−0.193473	−0.302135
C	−2.666124	1.367373	1.508953
C	−4.005841	1.876394	2.034503
C	−3.863319	2.838976	3.197843
O	−4.790733	0.763837	2.484108
C	−0.236766	1.585811	−1.107481
C	0.996498	2.252111	−1.095989
C	2.030082	1.767294	−0.293164
C	1.818334	0.661051	0.539666
C	0.571528	0.030437	0.559834
C	−0.462934	0.483070	−0.280983
C	0.326295	−1.114130	1.520672
C	1.611103	−1.676108	2.119379
C	1.365102	−2.547964	3.336542
O	2.459403	−0.595465	2.533626
C	2.946528	0.203140	1.446878
C	−5.197598	−0.074175	1.413924
C	4.093637	−0.507113	0.731154
C	5.352224	−0.514394	1.339410
C	6.426263	−1.187611	0.770517
C	6.220790	−1.860904	−0.421945
C	4.994485	−1.879851	−1.058580
C	3.932557	−1.194391	−0.472165
F	7.265169	−2.520851	−0.989244
O	−3.467882	−3.003418	−2.047069
C	−2.428049	−3.479929	−2.897307
O	−5.456610	−2.193261	−0.368247
C	−6.288324	−2.031837	−1.527906
O	1.193190	3.366986	−1.879978
C	0.698861	4.590345	−1.306517
O	3.234824	2.421810	−0.204278
C	4.029964	2.507807	−1.400770
O	−1.272961	2.088574	−1.860936
C	−1.152280	1.915870	−3.282127
H	−1.176811	−1.561535	−1.824667
H	−2.049408	1.063237	2.362800
H	−2.136074	2.185465	1.016448
H	−4.547496	2.371578	1.214774
H	−3.374296	2.346914	4.041332
H	−3.261318	3.701920	2.906217
H	−4.841371	3.194883	3.524860
H	−0.222009	−1.915362	1.021455
H	−0.318386	−0.762317	2.334067
H	2.137291	−2.265905	1.358527

H	0.893477	−1.967357	4.132259
H	2.304696	−2.953651	3.713905
H	0.706808	−3.380890	3.081241
H	3.366548	1.082847	1.934942
H	−5.982281	0.426594	0.827200
H	−5.647309	−0.957250	1.865987
H	5.495811	0.015769	2.273506
H	7.404111	−1.191475	1.233897
H	4.876495	−2.415319	−1.991511
H	2.971737	−1.192974	−0.970389
H	−2.115506	−2.715115	−3.613493
H	−2.849755	−4.325619	−3.434961
H	−1.562733	−3.814205	−2.318686
H	−5.815969	−2.458475	−2.413074
H	−7.213406	−2.563614	−1.313922
H	−6.509277	−0.974547	−1.699762
H	1.197909	4.797783	−0.357066
H	−0.381082	4.537656	−1.156837
H	0.931253	5.378447	−2.020034
H	4.260504	1.508338	−1.776153
H	4.952910	3.004055	−1.108214
H	3.521478	3.088296	−2.168867
H	−0.255453	2.406258	−3.662449
H	−1.133365	0.852939	−3.536752
H	−2.036398	2.375161	−3.719718

B3LYP Energy = −1829.33624493 a.u.

(*aR*,1*S*,3*S*,3'*S*)-**21**, Conf C

C	2.107308	−0.888896	1.523434
C	3.359462	−1.477985	1.664118
C	4.345901	−1.215853	0.699565
C	4.075739	−0.357390	−0.362630
C	2.819575	0.253782	−0.490187
C	1.828322	−0.030817	0.456552
C	2.570277	1.192677	−1.651994
C	3.869371	1.673234	−2.293003
C	3.651081	2.396517	−3.608255
O	4.712977	0.543488	−2.551311
C	0.138405	1.703062	1.097097
C	−1.133069	2.284481	1.002629
C	−2.079880	1.728258	0.140628
C	−1.792352	0.559959	−0.571809
C	−0.525592	−0.022720	−0.467927
C	0.457225	0.562317	0.352317
C	−0.205805	−1.261062	−1.278458
C	−1.448943	−1.931373	−1.853120
C	−1.128258	−2.941491	−2.938634
O	−2.304279	−0.934146	−2.432427
C	−2.865650	−0.040472	−1.461764
C	5.181180	−0.059147	−1.353676
C	−4.014272	−0.717621	−0.713694
C	−5.196392	−0.974628	−1.414344
C	−6.267192	−1.622381	−0.811401
C	−6.137129	−2.013173	0.510796
C	−4.988073	−1.778302	1.240885
C	−3.927328	−1.125332	0.616393
F	−7.180384	−2.644870	1.112175
O	3.717312	−2.297414	2.695465
C	2.761380	−2.554151	3.721910
O	5.620764	−1.725502	0.820178
C	5.751188	−3.144959	0.639819
O	−1.425339	3.411028	1.737756
C	−2.173185	3.160260	2.940789
O	−3.341695	2.267468	0.038531
C	−3.420748	3.567609	−0.573968
O	1.052779	2.201110	1.994361
C	1.624434	3.481684	1.683324
H	1.333445	−1.076152	2.253901
H	1.972310	0.691320	−2.421559
H	1.985862	2.054706	−1.323385
H	4.391927	2.340346	−1.591272
H	3.178862	1.731454	−4.334617
H	3.002933	3.262902	−3.462469
H	4.600477	2.741011	−4.020530
H	0.343522	−1.978783	−0.665223
H	0.460645	−0.990176	−2.104435
H	−1.994397	−2.434688	−1.045191
H	−0.635276	−2.452822	−3.781907
H	−2.039473	−3.420336	−3.299463
H	−0.461921	−3.714904	−2.551350
H	−3.299790	0.760561	−2.061461
H	5.932414	0.589739	−0.879981
H	5.690279	−0.975634	−1.648894
H	−5.281386	−0.665440	−2.449389
H	−7.186516	−1.819369	−1.347163
H	−4.927603	−2.095451	2.273715
H	−3.027699	−0.926534	1.184323
H	2.455565	−1.630157	4.219270
H	3.263063	−3.200591	4.437814
H	1.879425	−3.066203	3.327553
H	6.816092	−3.361216	0.698968
H	5.220858	−3.690617	1.420136
H	5.373864	−3.443532	−0.342443
H	−1.612256	2.504476	3.610677
H	−3.142736	2.715262	2.708987
H	−2.318924	4.127695	3.417153
H	−3.020435	3.534404	−1.590731
H	−2.880974	4.311424	0.011619
H	−4.478614	3.819060	−0.609272
H	2.197673	3.430591	0.754175
H	0.853322	4.248086	1.604808
H	2.296883	3.718624	2.505310

B3LYP Energy = −1829.33575129 a.u.

(*aR*,1*S*,3*S*,3'*S*)-**21**, Conf D

C	−2.061070	−1.140944	−1.319547
C	−3.290234	−1.788284	−1.387168
C	−4.295184	−1.434254	−0.472922
C	−4.042750	−0.483033	0.512011
C	−2.800827	0.165422	0.581819

C	−1.808344	−0.169909	−0.346962
C	−2.572278	1.207077	1.656561
C	−3.881605	1.704964	2.263158
C	−3.679021	2.544888	3.509783
O	−4.689159	0.578043	2.625931
C	−0.196917	1.556875	−1.175089
C	1.057736	2.180769	−1.173126
C	2.045963	1.731550	−0.295676
C	1.815031	0.625813	0.528334
C	0.564290	0.000517	0.518232
C	−0.458354	0.478378	−0.322745
C	0.305815	−1.164950	1.450530
C	1.584951	−1.747280	2.041953
C	1.328286	−2.657791	3.227946
O	2.429409	−0.678740	2.496531
C	2.933530	0.135158	1.429817
C	−5.145871	−0.145980	1.493908
C	4.072928	−0.579821	0.703051
C	3.947431	−1.116994	−0.577094
C	5.003097	−1.798164	−1.179660
C	6.186200	−1.930011	−0.478735
C	6.354904	−1.409786	0.793546
C	5.288601	−0.735830	1.375472
F	7.224677	−2.588044	−1.059768
O	−3.602584	−2.765158	−2.287688
C	−2.607039	−3.167758	−3.225622
O	−5.517136	−2.071699	−0.471797
C	−6.375742	−1.790643	−1.588423
O	1.296253	3.241402	−2.017741
C	2.001403	2.893631	−3.222382
O	3.293717	2.311841	−0.285163
C	3.352502	3.663072	0.206799
O	−1.152151	1.945657	−2.084360
C	−1.750175	3.234240	−1.872326
H	−1.278512	−1.386232	−2.023130
H	−1.958339	0.789528	2.462536
H	−2.012883	2.053119	1.251460
H	−4.427236	2.293630	1.510411
H	−3.183352	1.958725	4.286596
H	−3.058931	3.415384	3.286604
H	−4.636860	2.893489	3.898465
H	−0.242761	−1.950182	0.926267
H	−0.340388	−0.833906	2.270638
H	2.118498	−2.310889	1.266554
H	0.850584	−2.103302	4.038620
H	2.264314	−3.077040	3.599303
H	0.671397	−3.481028	2.939898
H	3.366826	0.997443	1.938173
H	−5.934207	0.425441	0.981195
H	−5.604962	−1.057128	1.875113
H	3.020915	−1.000186	−1.124096
H	4.912654	−2.215306	−2.174043
H	7.299788	−1.528999	1.307324
H	5.404143	−0.325619	2.371768
H	−3.066090	−3.948695	−3.826854
H	−1.725265	−3.570823	−2.720470
H	−2.310165	−2.338358	−3.872973
H	−5.940777	−2.152523	−2.520179
H	−7.309328	−2.314189	−1.391780
H	−6.570721	−0.717108	−1.663716
H	1.427204	2.170902	−3.806645
H	2.987313	2.486114	−2.990305
H	2.110587	3.815620	−3.789941
H	4.402470	3.946770	0.181193
H	2.987973	3.709418	1.236460
H	2.770282	4.335915	−0.422389
H	−2.289752	3.255325	−0.921998
H	−1.000034	4.024834	−1.892903
H	−2.456807	3.375946	−2.687626

B3LYP Energy = −1829.33549686 a.u.

(*aR*,1*S*,3*S*,3’*S*)-**21**, Conf E

C	1.965370	−1.200412	1.265300
C	3.190510	−1.854056	1.358967
C	4.248369	−1.433388	0.537858
C	4.074047	−0.362232	−0.334820
C	2.847071	0.311598	−0.408612
C	1.784932	−0.124947	0.390666
C	2.695476	1.480633	−1.358604
C	4.043004	2.011722	−1.841507
C	3.920309	2.974165	−3.007235
O	4.862280	0.914627	−2.267836
C	0.170002	1.643559	1.113729
C	−1.072754	2.294085	1.062969
C	−2.076077	1.775242	0.237375
C	−1.829080	0.634148	−0.539957
C	−0.575388	0.020373	−0.513474
C	0.437633	0.525934	0.321909
C	−0.294703	−1.153447	−1.428654
C	−1.559157	−1.754315	−2.032392
C	−1.277104	−2.662467	−3.214649
O	−2.415993	−0.700608	−2.495520
C	−2.937971	0.109404	−1.433874
C	5.245925	0.084178	−1.182561
C	−4.061310	−0.626372	−0.702485
C	−3.944793	−1.099951	0.603003
C	−4.985689	−1.797419	1.212806
C	−6.145154	−2.010249	0.492752
C	−6.304716	−1.554511	−0.805261
C	−5.253684	−0.862297	−1.393427
F	−7.169235	−2.685741	1.079805
O	3.453808	−2.886571	2.212549
C	2.411903	−3.341418	3.072318
O	5.498177	−2.007867	0.626433
C	5.604696	−3.361712	0.156904
O	−1.353036	3.361910	1.883352
C	−0.617383	4.574714	1.636087
O	−3.346387	2.283486	0.185441
C	−3.541241	3.694274	0.004849
O	1.162390	2.148668	1.926426
C	1.014887	1.860127	3.326545

H	1.134533	−1.515680	1.880453
H	2.103957	1.179775	−2.231348
H	2.143845	2.287579	−0.871635
H	4.550386	2.513983	−1.004521
H	3.467213	2.474796	−3.866421
H	3.293829	3.825586	−2.733802
H	4.901616	3.348394	−3.302393
H	0.252116	−1.929804	−0.890242
H	0.363908	−0.821066	−2.239370
H	−2.091185	−2.325074	−1.260837
H	−0.801417	−2.101018	−4.021737
H	−2.202587	−3.097777	−3.593922
H	−0.609224	−3.474372	−2.919584
H	−3.389603	0.959432	−1.946531
H	5.978501	0.609163	−0.551904
H	5.754555	−0.775684	−1.616351
H	−3.036994	−0.918768	1.163687
H	−4.902286	−2.164487	2.227351
H	−7.231407	−1.736251	−1.333707
H	−5.361874	−0.502154	−2.409633
H	2.076443	−2.547712	3.744959
H	2.843069	−4.150177	3.657179
H	1.560091	−3.721199	2.501599
H	5.286974	−3.432318	−0.887332
H	6.657487	−3.626555	0.232973
H	5.009858	−4.038604	0.770025
H	−0.988557	5.301953	2.355244
H	−0.805880	4.936684	0.622161
H	0.451546	4.422528	1.778667
H	−3.411297	4.234894	0.940123
H	−4.564863	3.806860	−0.347511
H	−2.855860	4.087125	−0.750293
H	1.051584	0.782050	3.499976
H	1.857691	2.334022	3.825662
H	0.079469	2.266615	3.714856

B3LYP Energy = −1829.33484405 a.u.

(*aR*,1*S*,3*S*,3’*S*)-**21**, Conf F

C	−1.945236	−1.369435	−1.065912
C	−3.154338	−2.058588	−1.072622
C	−4.210347	−1.584426	−0.279231
C	−4.031464	−0.464007	0.528458
C	−2.814747	0.232093	0.529680
C	−1.767727	−0.230625	−0.275260
C	−2.662875	1.463585	1.396843
C	−4.008407	1.994515	1.884594
C	−3.875446	3.037514	2.977951
O	−4.783971	0.909930	2.410482
C	−0.221815	1.520911	−1.169636
C	1.003947	2.203714	−1.201344
C	2.044553	1.767380	−0.374385
C	1.851726	0.677197	0.486408
C	0.613449	0.034434	0.542935
C	−0.436500	0.455713	−0.293618
C	0.389511	−1.076968	1.547085
C	1.685997	−1.606146	2.150270
C	1.462497	−2.432320	3.402837
O	2.531954	−0.503274	2.508991
C	3.000777	0.238979	1.375849
C	−5.181212	−0.004418	1.400616
C	4.114973	−0.525076	0.659366
C	5.329976	−0.698768	1.329123
C	6.375899	−1.410460	0.755432
C	6.188113	−1.949507	−0.506431
C	5.005487	−1.800660	−1.204415
C	3.969993	−1.082327	−0.609660
F	7.206925	−2.644886	−1.079299
O	−3.398160	−3.189125	−1.797857
C	−2.344455	−3.729638	−2.591131
O	−5.410582	−2.258566	−0.211648
C	−6.229201	−2.206883	−1.390495
O	1.231969	3.220460	−2.099091
C	0.472770	4.428894	−1.908051
O	3.301074	2.312156	−0.400558
C	3.455335	3.734542	−0.282325
O	−1.248950	1.943160	−1.987311
C	−1.133459	1.550277	−3.364721
H	−1.119048	−1.718708	−1.668843
H	−2.039771	1.234934	2.269271
H	−2.145671	2.248598	0.840923
H	−4.553562	2.426311	1.031971
H	−3.378085	2.611063	3.851791
H	−3.285196	3.885621	2.625187
H	−4.857096	3.402977	3.282764
H	−0.157199	−1.900538	1.084139
H	−0.249888	−0.701457	2.354328
H	2.207541	−2.219133	1.404427
H	0.996441	−1.825493	4.182241
H	2.409751	−2.817036	3.783108
H	0.807158	−3.278844	3.187844
H	3.451228	1.131621	1.811306
H	−5.964059	0.450153	0.775035
H	−5.630220	−0.854855	1.911931
H	5.459954	−0.273975	2.317421
H	7.319754	−1.544555	1.267477
H	4.899864	−2.233084	−2.190743
H	3.043538	−0.952824	−1.153777
H	−2.024955	−3.025969	−3.364644
H	−2.755114	−4.619621	−3.061638
H	−1.485807	−4.009686	−1.975087
H	−5.742664	−2.705166	−2.229284
H	−7.152536	−2.726234	−1.141287
H	−6.456521	−1.170687	−1.656627
H	0.680096	4.859766	−0.925103
H	−0.595544	4.242638	−2.009409
H	0.805119	5.116976	−2.682596
H	2.783444	4.135068	0.480840
H	3.275787	4.232754	−1.232956
H	4.486142	3.894730	0.028328
H	−0.213065	1.935512	−3.806893
H	−1.161660	0.461690	−3.454168

H	−1.993802	1.976522	−3.876914

B3LYP Energy = −1829.33467513 a.u.

(*aR*,1*S*,3*S*,3’*S*)-**21**, Conf G

C	2.058543	−1.090432	1.317409
C	3.304114	−1.702429	1.423191
C	4.342035	−1.279613	0.578092
C	4.128048	−0.246510	−0.331069
C	2.879949	0.385233	−0.418799
C	1.838299	−0.055616	0.404257
C	2.683863	1.515760	−1.406284
C	4.010072	2.071516	−1.919762
C	3.847629	2.987092	−3.117772
O	4.861133	0.986096	−2.313616
C	0.174237	1.681509	1.088452
C	−1.096022	2.275520	1.028791
C	−2.051095	1.742888	0.158020
C	−1.780075	0.589897	−0.584398
C	−0.517463	−0.004265	−0.510883
C	0.470879	0.549952	0.322021
C	−0.203589	−1.203884	−1.379899
C	−1.451098	−1.844440	−1.978160
C	−1.138474	−2.801744	−3.112998
O	−2.310087	−0.821762	−2.504182
C	−2.861279	0.031525	−1.492440
C	5.279139	0.208072	−1.202630
C	−4.006656	−0.670986	−0.763216
C	−5.195403	−0.895971	−1.463850
C	−6.264084	−1.562704	−0.878118
C	−6.125563	−2.005090	0.426885
C	−4.969907	−1.803294	1.156399
C	−3.911232	−1.130863	0.549207
F	−7.166706	−2.655413	1.011555
O	3.606006	−2.695165	2.310826
C	2.586550	−3.147461	3.198084
O	5.609810	−1.811815	0.677592
C	5.755450	−3.177022	0.254101
O	−1.329133	3.417124	1.757203
C	−2.374487	3.343753	2.744275
O	−3.295098	2.324879	0.036102
C	−3.313614	3.599634	−0.633042
O	1.180604	2.255408	1.826528
C	1.069984	2.175121	3.253172
H	1.243144	−1.406478	1.952541
H	2.094495	1.167084	−2.262480
H	2.112247	2.321783	−0.941353
H	4.507436	2.619410	−1.105882
H	3.405912	2.442658	−3.955245
H	3.194863	3.826359	−2.869582
H	4.813884	3.382996	−3.433557
H	0.346910	−1.952437	−0.806841
H	0.460685	−0.891010	−2.193597
H	−1.991457	−2.385726	−1.191326
H	−0.652038	−2.272639	−3.935373
H	−2.052338	−3.263012	−3.489693
H	−0.469135	−3.592514	−2.768308
H	−3.297019	0.857425	−2.055604
H	5.997156	0.779260	−0.595771
H	5.813940	−0.649147	−1.609312
H	−5.287247	−0.546609	−2.485430
H	−7.188309	−1.735129	−1.413905
H	−4.902880	−2.160648	2.175615
H	−3.006018	−0.958809	1.116891
H	3.046178	−3.922748	3.806155
H	1.739548	−3.570521	2.651083
H	2.236169	−2.339646	3.846008
H	6.816070	−3.407303	0.334017
H	5.183801	−3.850107	0.892979
H	5.436116	−3.293380	−0.785541
H	−3.346693	3.172370	2.283402
H	−2.369031	4.303495	3.256505
H	−2.166546	2.546527	3.462788
H	−4.357804	3.901360	−0.678766
H	−2.916315	3.504591	−1.646779
H	−2.736048	4.343022	−0.082410
H	0.931221	1.138726	3.572348
H	2.011032	2.550028	3.650626
H	0.250042	2.790477	3.623968

B3LYP Energy = −1829.33464125 a.u.

(*aR*,1*S*,3*S*,3’*S*)-**21**, Conf H

C	2.044609	−1.282113	1.120260
C	3.275315	−1.932194	1.133145
C	4.306947	−1.449517	0.313354
C	4.084521	−0.359627	−0.524869
C	2.845768	0.295943	−0.534057
C	1.822604	−0.176601	0.295156
C	2.646023	1.498660	−1.431283
C	3.970059	2.061212	−1.941646
C	3.796501	3.073890	−3.057392
O	4.776756	0.989531	−2.447770
C	0.231033	1.544542	1.163992
C	−1.021453	2.177738	1.186895
C	−2.017611	1.737490	0.310503
C	−1.803601	0.637215	−0.524513
C	−0.557033	0.005733	−0.536975
C	0.470654	0.467361	0.304347
C	−0.302040	−1.130201	−1.505832
C	−1.581938	−1.684906	−2.122126
C	−1.325409	−2.544921	−3.345330
O	−2.426459	−0.598986	−2.533006
C	−2.926181	0.177345	−1.436958
C	5.211144	0.113154	−1.419803
C	−4.062608	−0.560075	−0.728585
C	−5.277599	−0.704494	−1.405116
C	−6.341499	−1.395994	−0.839818
C	−6.171321	−1.945470	0.420005
C	−4.989103	−1.825721	1.124542
C	−3.935632	−1.126709	0.538604
F	−7.207359	−2.621176	0.984668

O	3.563173	−3.031753	1.889652
C	2.535254	−3.580650	2.709982
O	5.527554	−2.087393	0.253700
C	6.358632	−1.964564	1.418361
O	−1.199911	3.271015	2.000393
C	−2.213022	3.151757	3.016547
O	−3.246313	2.362606	0.272666
C	−3.246979	3.679323	−0.310043
O	1.279577	2.027302	1.908278
C	1.191789	1.900261	3.333299
H	1.237496	−1.635433	1.746374
H	2.025169	1.227184	−2.293132
H	2.107278	2.279911	−0.890849
H	4.506527	2.530128	−1.103320
H	3.308782	2.611938	−3.918540
H	3.180578	3.909724	−2.719670
H	4.763901	3.464624	−3.376164
H	0.238947	−1.937289	−1.008216
H	0.352870	−0.773164	−2.308705
H	−2.116581	−2.280084	−1.371158
H	−0.846129	−1.956916	−4.131015
H	−2.261596	−2.946661	−3.735182
H	−0.669661	−3.380519	−3.092243
H	−3.361823	1.055066	−1.915194
H	5.985002	0.606647	−0.812868
H	5.681731	−0.735522	−1.914507
H	−5.394012	−0.271354	−2.391541
H	−7.285689	−1.506714	−1.356760
H	−4.897601	−2.266494	2.108581
H	−3.009141	−1.019970	1.087594
H	2.978076	−4.443148	3.202046
H	1.679505	−3.905111	2.111816
H	2.201626	−2.864343	3.465818
H	7.294388	−2.465246	1.177351
H	6.557314	−0.912351	1.641008
H	5.898075	−2.443890	2.282598
H	−2.173653	4.076924	3.587578
H	−1.995230	2.307275	3.675549
H	−3.202652	3.025451	2.578899
H	−2.884917	3.641236	−1.340566
H	−2.629870	4.367550	0.268720
H	−4.282365	4.013065	−0.300770
H	0.446861	2.578723	3.748826
H	0.956488	0.870795	3.616435
H	2.174585	2.162774	3.720013

B3LYP Energy = −1829.33434393 a.u.

(*aR*,1*S*,3*S*,3'*S*)-**21**, Conf I

C	−1.984489	−1.080893	−1.324049
C	−3.217728	−1.710072	−1.442371
C	−4.253046	−1.355420	−0.572638
C	−4.055261	−0.344912	0.372274
C	−2.819844	0.304537	0.470069
C	−1.768174	−0.083558	−0.375179
C	−2.636687	1.401811	1.496199
C	−3.967416	1.911451	2.044359
C	−3.808430	2.786648	3.272644
O	−4.791263	0.795635	2.408874
C	−0.140750	1.716845	−0.979736
C	1.112497	2.337395	−0.887050
C	2.120137	1.737655	−0.129942
C	1.862816	0.559372	0.582572
C	0.596543	−0.028758	0.524737
C	−0.412049	0.542762	−0.273862
C	0.304048	−1.258569	1.358055
C	1.564305	−1.922493	1.901212
C	1.277885	−2.916916	3.010883
O	2.440196	−0.921398	2.439142
C	2.964622	−0.024534	1.449770
C	−5.204083	0.046148	1.276983
C	4.103633	−0.686203	0.677303
C	5.356489	−0.779442	1.290225
C	6.420867	−1.418348	0.666127
C	6.211635	−1.969542	−0.586838
C	4.990732	−1.900171	−1.230261
C	3.938755	−1.250847	−0.587643
F	7.246694	−2.594830	−1.207821
O	−3.373056	−2.707467	−2.382607
C	−4.205908	−2.364224	−3.502670
O	−5.500778	−1.937200	−0.659265
C	−5.568395	−3.321143	−0.271304
O	1.351751	3.521369	−1.548705
C	0.929136	4.694783	−0.831792
O	3.344703	2.338601	0.034682
C	4.154001	2.518442	−1.141977
O	−1.150966	2.324110	−1.689290
C	−1.010585	2.298704	−3.119224
H	−1.189371	−1.382016	−1.995132
H	−2.029378	1.033207	2.330932
H	−2.085571	2.234508	1.054285
H	−4.485397	2.477967	1.256430
H	−3.345636	2.221784	4.084742
H	−3.175451	3.647222	3.047354
H	−4.778415	3.151167	3.613700
H	−0.263274	−1.985455	0.773719
H	−0.337463	−0.972693	2.199694
H	2.080279	−2.439993	1.082964
H	0.815010	−2.414977	3.863337
H	2.199901	−3.392700	3.347588
H	0.597462	−3.693970	2.656925
H	3.399945	0.786126	2.034359
H	−5.947712	0.617399	0.701626
H	−5.708033	−0.840032	1.660480
H	5.503744	−0.345317	2.272144
H	7.394318	−1.488048	1.133559
H	4.868841	−2.340739	−2.211103
H	2.982656	−1.181327	−1.090108
H	−4.225931	−3.240374	−4.147737
H	−3.779599	−1.519229	−4.050138
H	−5.219615	−2.119670	−3.181124
H	−4.952257	−3.942467	−0.921885

H	−5.246542	−3.443387	0.766372
H	−6.613113	−3.610888	−0.363147
H	−0.149974	4.678760	−0.666848
H	1.190465	5.546270	−1.457022
H	1.451296	4.768974	0.124897
H	4.351119	1.555247	−1.617645
H	5.091457	2.947313	−0.794309
H	3.674003	3.193525	−1.848673
H	−0.103343	2.816200	−3.432607
H	−0.997260	1.267888	−3.482668
H	−1.883633	2.810920	−3.518501

B3LYP Energy = −1829.33406422 a.u.

(*aR*,1*S*,3*S*,3'*S*)-**21**, Conf J

C	−1.975587	−1.146177	−1.274895
C	−3.201423	−1.793187	−1.371522
C	−4.252788	−1.394597	−0.541648
C	−4.049524	−0.391925	0.410383
C	−2.813182	0.256220	0.505479
C	−1.766912	−0.125003	−0.349947
C	−2.632442	1.362005	1.522722
C	−3.965691	1.880419	2.055974
C	−3.813715	2.776158	3.270226
O	−4.788674	0.769169	2.435569
C	−0.175591	1.678241	−1.028844
C	1.068106	2.321885	−0.972727
C	2.093370	1.766050	−0.205797
C	1.862469	0.609986	0.551039
C	0.605574	−0.000350	0.528446
C	−0.420366	0.526014	−0.278919
C	0.342288	−1.205011	1.407445
C	1.619038	−1.827629	1.961022
C	1.361485	−2.785717	3.109110
O	2.482058	−0.792734	2.453116
C	2.982563	0.074383	1.425839
C	−5.197690	−0.001818	1.317307
C	4.122542	−0.599469	0.664964
C	3.957574	−1.194036	−0.586100
C	5.011833	−1.851522	−1.216743
C	6.234754	−1.898930	−0.575312
C	6.444023	−1.318061	0.664243
C	5.377588	−0.671271	1.276433
F	7.271899	−2.532202	−1.184651
O	−3.366798	−2.780986	−2.320163
C	−3.456713	−4.116446	−1.796756
O	−5.483674	−2.015221	−0.591470
C	−6.260173	−1.758066	−1.774523
O	1.281603	3.484589	−1.679011
C	0.823200	4.674200	−1.012623
O	3.309936	2.390600	−0.074998
C	4.101763	2.552177	−1.266080
O	−1.202595	2.245617	−1.747311
C	−1.085997	2.155824	−3.177167
H	−1.176780	−1.459809	−1.935784
H	−2.034058	0.998976	2.366326
H	−2.074484	2.188349	1.077538
H	−4.480479	2.433061	1.256124
H	−3.353491	2.225769	4.093590
H	−3.181577	3.634287	3.033488
H	−4.785976	3.144185	3.600932
H	−0.217734	−1.961288	0.854238
H	−0.296272	−0.901032	2.244846
H	2.136215	−2.365350	1.156632
H	0.898467	−2.260934	3.947573
H	2.295400	−3.232247	3.452996
H	0.691614	−3.587412	2.791680
H	3.412421	0.911996	1.975481
H	−5.954244	0.550924	0.740399
H	−5.683510	−0.891650	1.715819
H	2.999738	−1.141738	−1.087302
H	4.890078	−2.315067	−2.186959
H	7.419112	−1.371344	1.130413
H	5.524615	−0.213823	2.247727
H	−2.537787	−4.381228	−1.266399
H	−3.580832	−4.773322	−2.655397
H	−4.311254	−4.221034	−1.126188
H	−6.455037	−0.687544	−1.879725
H	−5.752446	−2.128360	−2.665766
H	−7.202172	−2.285673	−1.639520
H	−0.256145	4.635817	−0.853780
H	1.066522	5.506438	−1.670200
H	1.336970	4.800601	−0.056750
H	4.304933	1.580526	−1.721596
H	5.038182	3.000318	−0.940628
H	3.604588	3.204220	−1.982331
H	−1.082964	1.109961	−3.494759
H	−1.963558	2.653664	−3.584645
H	−0.182244	2.655203	−3.528260

B3LYP Energy = −1829.33384284 a.u.

(*aR*,1*S*,3*S*,3'*S*)-**21**, Conf K

C	2.114736	−0.344765	1.699395
C	3.368352	−0.843562	2.035906
C	4.358444	−0.917510	1.042793
C	4.087337	−0.477110	−0.249732
C	2.827914	0.043647	−0.582980
C	1.835269	0.097620	0.403299
C	2.582782	0.526755	−1.997324
C	3.884831	0.784945	−2.750166
C	3.676852	1.025366	−4.233208
O	4.741396	−0.356010	−2.610221
C	0.148919	1.941877	0.473865
C	−1.127839	2.475526	0.232704
C	−2.084667	1.683483	−0.408277
C	−1.781032	0.361558	−0.766163
C	−0.517372	−0.168383	−0.501301
C	0.465087	0.629342	0.115779
C	−0.205342	−1.595661	−0.900145
C	−1.454622	−2.399641	−1.240370
C	−1.145951	−3.698145	−1.962087

O	−2.296656	−1.620490	−2.102008
C	−2.850247	−0.461389	−1.464633
C	5.199434	−0.523132	−1.277046
C	−4.033647	−0.854226	−0.581596
C	−3.947849	−0.963236	0.806234
C	−5.038512	−1.382482	1.565417
C	−6.217158	−1.686517	0.911479
C	−6.347483	−1.588504	−0.463613
C	−5.245933	−1.171845	−1.200820
F	−7.289335	−2.090052	1.643869
O	3.725435	−1.259029	3.286182
C	2.765302	−1.158877	4.335497
O	5.638119	−1.337677	1.334362
C	5.790625	−2.729330	1.657207
O	−1.414737	3.790001	0.522007
C	−1.463548	4.138188	1.918092
O	−3.310437	2.155963	−0.797555
C	−4.157752	2.809512	0.158549
O	1.093131	2.718055	1.111217
C	1.697505	3.750216	0.314316
H	1.340126	−0.272943	2.449143
H	2.007195	−0.218729	−2.557069
H	1.977907	1.435892	−1.988634
H	4.392232	1.654404	−2.306118
H	3.217887	0.151113	−4.699956
H	3.021671	1.884171	−4.391776
H	4.629015	1.221542	−4.727976
H	0.345073	−2.095035	−0.099688
H	0.454885	−1.594024	−1.773738
H	−2.004777	−2.625042	−0.318364
H	−0.646522	−3.496736	−2.912323
H	−2.063435	−4.252765	−2.163354
H	−0.490100	−4.324225	−1.353708
H	−3.246180	0.129422	−2.291070
H	5.946861	0.248395	−1.040591
H	5.711304	−1.484154	−1.246046
H	−3.024449	−0.711568	1.311768
H	−4.978483	−1.469170	2.642434
H	−7.289794	−1.829785	−0.937784
H	−5.331376	−1.092296	−2.278123
H	1.888324	−1.780580	4.135475
H	2.451690	−0.123091	4.488989
H	3.266542	−1.520460	5.230003
H	6.858411	−2.894065	1.787721
H	5.264583	−2.979744	2.578337
H	5.424201	−3.356116	0.839215
H	−0.505168	3.955588	2.402711
H	−2.247781	3.571498	2.426672
H	−1.701980	5.199145	1.955043
H	−4.173723	2.257928	1.101323
H	−5.155339	2.799439	−0.276144
H	−3.840381	3.836004	0.331215
H	0.944571	4.429940	−0.087041
H	2.366831	4.292743	0.978840
H	2.277570	3.314967	−0.502513

B3LYP Energy = −1829.33375072 a.u.

(*aR*,1*S*,3*S*,3’*S*)-**21**, Conf L

C	−2.096674	−0.275759	−1.699204
C	−3.329272	−0.794122	−2.081043
C	−4.317082	−0.986555	−1.102248
C	−4.047192	−0.695611	0.232193
C	−2.802030	−0.176075	0.616767
C	−1.823443	0.032519	−0.363561
C	−2.562384	0.150085	2.076125
C	−3.867572	0.268548	2.858347
C	−3.657830	0.353405	4.358241
O	−4.674255	−0.886723	2.596663
C	−0.192402	1.924262	−0.230702
C	1.070395	2.464953	0.063432
C	2.056680	1.632476	0.600135
C	1.795359	0.269009	0.802103
C	0.545261	−0.263927	0.485679
C	−0.467702	0.570947	−0.024689
C	0.282763	−1.738547	0.708485
C	1.560258	−2.538136	0.935390
C	1.300827	−3.924976	1.493601
O	2.389758	−1.845012	1.878768
C	2.897866	−0.597589	1.386631
C	−5.138655	−0.929644	1.256419
C	4.083962	−0.837338	0.453828
C	5.319426	−1.163213	1.020461
C	6.424931	−1.450856	0.229302
C	6.274751	−1.410972	−1.146566
C	5.072681	−1.093608	−1.749717
C	3.978568	−0.805107	−0.936622
F	7.350188	−1.688326	−1.931097
O	−3.661109	−1.143201	−3.358171
C	−2.682647	−0.982491	−4.382528
O	−5.538903	−1.540510	−1.418314
C	−6.419523	−0.721405	−2.203307
O	1.315310	3.812439	−0.071607
C	1.342590	4.322958	−1.417281
O	3.275122	2.086759	1.028978
C	4.073852	2.914263	0.170689
O	−1.164014	2.740595	−0.769820
C	−1.794201	3.650628	0.146375
H	−1.325679	−0.108039	−2.437387
H	−1.955740	−0.632116	2.545663
H	−1.993762	1.077533	2.170175
H	−4.415713	1.158863	2.514997
H	−3.157242	−0.545720	4.723853
H	−3.039578	1.217755	4.608466
H	−4.613609	0.451137	4.874965
H	−0.261807	−2.153391	−0.142543
H	−0.366131	−1.866213	1.580971
H	2.105648	−2.631343	−0.012066
H	0.813523	−3.859032	2.468724
H	2.236748	−4.472939	1.610610
H	0.651514	−4.489403	0.821345
H	3.283142	−0.101002	2.277656

H	−5.938828	−0.187244	1.117161
H	−5.584666	−1.913389	1.115732
H	5.420596	−1.192233	2.098982
H	7.384928	−1.698010	0.663259
H	4.997399	−1.070716	−2.829012
H	3.036220	−0.544349	−1.400868
H	−2.390029	0.064938	−4.494296
H	−3.155988	−1.324749	−5.299546
H	−1.795851	−1.590377	−4.184213
H	−7.348696	−1.280020	−2.297926
H	−6.617062	0.227443	−1.696552
H	−6.002627	−0.531480	−3.192490
H	2.132842	3.836954	−1.995295
H	1.558421	5.385883	−1.331276
H	0.383382	4.177102	−1.912658
H	4.049724	2.543821	−0.856623
H	5.090449	2.840330	0.552106
H	3.741713	3.950140	0.200075
H	−1.061114	4.306482	0.618220
H	−2.490492	4.242233	−0.444641
H	−2.347990	3.102585	0.911915

B3LYP Energy = −1829.33351521 a.u.

(*aS*,1*R*,3*S*,3'*S*)-**21**, Conf A

C	2.001337	−1.476411	−0.921408
C	3.242787	−2.103141	−0.973950
C	4.342078	−1.484272	−0.358288
C	4.180041	−0.272979	0.309775
C	2.924528	0.345719	0.380238
C	1.833549	−0.259546	−0.254277
C	2.783749	1.658264	1.119903
C	3.970067	1.932982	2.039972
C	3.994809	3.354065	2.570121
O	5.189789	1.717256	1.318149
C	0.181149	1.434443	−1.065520
C	−1.078737	2.045957	−1.041082
C	−2.082945	1.523012	−0.223545
C	−1.823069	0.414175	0.595478
C	−0.535685	−0.133784	0.628279
C	0.469342	0.356192	−0.226735
C	−0.222523	−1.213933	1.640477
C	−1.212129	−1.174030	2.796670
C	−1.046044	−2.323683	3.772309
O	−2.537736	−1.257266	2.260293
C	−2.941536	−0.120868	1.488238
C	5.381695	0.349941	0.988474
C	−4.178686	−0.572608	0.732382
C	−5.445219	−0.168978	1.148684
C	−6.593131	−0.622845	0.504306
C	−6.446122	−1.495933	−0.557482
C	−5.206100	−1.928220	−0.996205
C	−4.073456	−1.459247	−0.340364
F	−7.559359	−1.946835	−1.195552
O	3.478788	−3.303258	−1.580984
C	2.386394	−3.977873	−2.200511
O	5.580854	−2.087223	−0.326117
C	6.271303	−2.181727	−1.582899
O	−1.332875	3.142856	−1.833308
C	−0.847728	4.388243	−1.301311
O	−3.304896	2.138922	−0.114081
C	−4.117419	2.223536	−1.299674
O	1.174054	1.970552	−1.853015
C	1.057800	1.696148	−3.258209
H	1.143483	−1.933377	−1.393898
H	2.698496	2.476659	0.396950
H	1.861157	1.662585	1.706234
H	3.942369	1.229202	2.885592
H	4.074751	4.065609	1.745477
H	3.079017	3.569959	3.124082
H	4.844871	3.501434	3.237815
H	0.795211	−1.083409	2.013865
H	−0.260613	−2.207440	1.181389
H	−1.103955	−0.220194	3.333172
H	−1.190703	−3.279322	3.264065
H	−1.774150	−2.248933	4.581092
H	−0.044442	−2.311163	4.206481
H	−3.238760	0.681631	2.176086
H	5.630222	−0.221079	1.895347
H	6.253111	0.309811	0.336373
H	−5.542125	0.515341	1.982952
H	−7.580637	−0.311212	0.818250
H	−5.135962	−2.614986	−1.829544
H	−3.096335	−1.789251	−0.670361
H	2.798758	−4.898755	−2.605478
H	1.959120	−3.383725	−3.012842
H	1.605192	−4.219419	−1.474905
H	7.245746	−2.612354	−1.360851
H	5.734012	−2.825513	−2.279007
H	6.404220	−1.189622	−2.023588
H	0.237705	4.364885	−1.190880
H	−1.317086	4.602346	−0.338129
H	−1.125767	5.156404	−2.020253
H	−5.060201	2.663150	−0.981470
H	−4.301704	1.226326	−1.704443
H	−3.646232	2.851788	−2.053212
H	1.899272	2.193460	−3.736378
H	1.119850	0.620529	−3.442818
H	0.121915	2.089833	−3.657775

B3LYP Energy = −1829.33591122 a.u.

(*aS*,1*R*,3*S*,3'*S*)-**21**, Conf B

C	1.988829	−1.330484	−1.139869
C	3.232973	−1.937145	−1.283443
C	4.342156	−1.373031	−0.634335
C	4.200914	−0.198164	0.100351
C	2.947744	0.413474	0.240780
C	1.836552	−0.163348	−0.385657
C	2.828829	1.679239	1.060500
C	4.041674	1.899116	1.960123

C	4.078405	3.282212	2.581893
O	5.237578	1.739305	1.186308
C	0.135668	1.565934	−1.004857
C	−1.132516	2.150433	−0.891353
C	−2.101972	1.543696	−0.089943
C	−1.797645	0.379509	0.630640
C	−0.501221	−0.145059	0.580041
C	0.467580	0.429355	−0.264218
C	−0.139857	−1.293712	1.496739
C	−1.086727	−1.348240	2.687459
C	−0.868730	−2.556617	3.578741
O	−2.428952	−1.420090	2.192794
C	−2.878630	−0.242774	1.512012
C	5.427743	0.399976	0.757542
C	−4.126241	−0.670018	0.758899
C	−4.032011	−1.461085	−0.386961
C	−5.172193	−1.912478	−1.042129
C	−6.408564	−1.559286	−0.528706
C	−6.544584	−0.780789	0.605646
C	−5.389103	−0.343338	1.247826
F	−7.529347	−1.993664	−1.164932
O	3.468113	−3.056963	−2.028398
C	2.374756	−3.656827	−2.718845
O	5.603210	−1.910737	−0.775025
C	5.820430	−3.183408	−0.146189
O	−1.430239	3.304357	−1.581012
C	−0.941456	4.509452	−0.966029
O	−3.330700	2.124418	0.104330
C	−4.181054	2.283767	−1.046391
O	1.095166	2.178458	−1.776726
C	0.921174	2.045787	−3.197042
H	1.120494	−1.753082	−1.625007
H	2.725328	2.539185	0.390355
H	1.922948	1.647833	1.671565
H	4.043247	1.140211	2.757184
H	4.125669	4.047042	1.803705
H	3.181689	3.454641	3.180462
H	4.950925	3.391340	3.227613
H	0.890253	−1.179436	1.840025
H	−0.188885	−2.251700	0.968441
H	−0.974060	−0.430192	3.282194
H	−1.011024	−3.479643	3.012681
H	−1.572737	−2.550794	4.411980
H	0.145320	−2.551942	3.983396
H	−3.174714	0.503062	2.261221
H	5.732208	−0.223585	1.611690
H	6.261445	0.415539	0.056860
H	−3.057430	−1.728540	−0.775677
H	−5.110407	−2.525525	−1.931710
H	−7.529476	−0.528607	0.976085
H	−5.477341	0.266769	2.138756
H	1.601897	−3.997201	−2.024250
H	2.790545	−4.513827	−3.243045
H	1.935459	−2.967328	−3.444617
H	6.872562	−3.418786	−0.294373
H	5.609586	−3.124830	0.925468
H	5.202946	−3.957686	−0.601691
H	0.147993	4.497331	−0.904064
H	−1.371545	4.632496	0.030843
H	−1.262459	5.329962	−1.604738
H	−3.740949	2.971681	−1.765888
H	−5.118279	2.686260	−0.668246
H	−4.366463	1.315952	−1.516669
H	−0.027294	2.479400	−3.517085
H	1.746596	2.586102	−3.655921
H	0.969145	0.994013	−3.490688

B3LYP Energy = −1829.33562322 a.u.

(*aS*,1*R*,3*S*,3’*S*)-**21**, Conf C

C	−2.124303	1.427610	−1.027471
C	−3.389052	2.006827	−1.009150
C	−4.412789	1.378800	−0.282133
C	−4.153727	0.205299	0.421238
C	−2.873955	−0.368096	0.414699
C	−1.859357	0.248337	−0.327273
C	−2.630702	−1.639510	1.200684
C	−3.718714	−1.888089	2.242090
C	−3.646665	−3.271286	2.860713
O	−5.003466	−1.755590	1.621528
C	−0.128242	−1.190116	−1.420698
C	1.165703	−1.721724	−1.495908
C	2.105358	−1.392593	−0.515940
C	1.801252	−0.458071	0.480206
C	0.501596	0.056292	0.560136
C	−0.471741	−0.313374	−0.385295
C	0.146799	0.965565	1.715442
C	1.103498	0.759823	2.882018
C	0.892490	1.744813	4.016517
O	2.442568	0.938890	2.406611
C	2.888191	−0.051932	1.473714
C	−5.273319	−0.418175	1.228052
C	4.132629	0.538577	0.833260
C	5.395464	0.159592	1.282505
C	6.549336	0.733244	0.755775
C	6.411022	1.699466	−0.223133
C	5.174299	2.110498	−0.690091
C	4.035720	1.522609	−0.150967
F	7.530515	2.267063	−0.748246
O	−3.716445	3.169001	−1.645384
C	−2.705447	3.841745	−2.392825
O	−5.666705	1.941136	−0.175839
C	−6.459186	1.947221	−1.374724
O	1.496868	−2.578969	−2.521588
C	2.252098	−1.970723	−3.584807
O	3.375268	−1.918235	−0.568688
C	3.477328	−3.331666	−0.323145
O	−1.050128	−1.446209	−2.405531
C	−1.493267	−2.805349	−2.554193
H	−1.322588	1.890305	−1.584876
H	−2.598696	−2.500142	0.523406
H	−1.656433	−1.600860	1.693560

H	−3.638093	−1.128944	3.034634
H	−3.773196	−4.038226	2.093442
H	−2.679350	−3.423100	3.343874
H	−4.429831	−3.399049	3.609273
H	−0.879469	0.779119	2.035522
H	0.190517	2.016488	1.409081
H	0.994907	−0.266161	3.263270
H	1.030499	2.768921	3.663553
H	1.603166	1.558877	4.822933
H	−0.118339	1.651058	4.418218
H	3.189316	−0.947950	2.033663
H	−5.472117	0.198085	2.117184
H	−6.194183	−0.448538	0.647261
H	5.485896	−0.598828	2.051089
H	7.534386	0.441478	1.095486
H	5.110966	2.872182	−1.456159
H	3.061359	1.833764	−0.506591
H	−2.331049	3.217528	−3.208450
H	−1.872673	4.144202	−1.752433
H	−3.182738	4.727398	−2.804985
H	−7.427023	2.359123	−1.095499
H	−6.002770	2.569055	−2.144664
H	−6.592484	0.929399	−1.752136
H	1.680816	−1.161104	−4.044906
H	2.430101	−2.753691	−4.319272
H	3.205771	−1.590233	−3.214735
H	4.540421	−3.562555	−0.336572
H	2.963590	−3.903545	−1.095796
H	3.063674	−3.579982	0.657952
H	−1.940507	−3.166059	−1.624755
H	−2.253082	−2.787124	−3.332784
H	−0.672981	−3.458357	−2.849218

B3LYP Energy = −1829.33406158 a.u.

(*aS*,1*R*,3*S*,3’*S*)-**21**, Conf D

C	2.130605	−1.122172	−1.385312
C	3.405748	−1.666415	−1.502049
C	4.428917	−1.183488	−0.670919
C	4.175812	−0.142700	0.218861
C	2.891842	0.409592	0.329500
C	1.865220	−0.092271	−0.479702
C	2.652443	1.525040	1.324447
C	3.759974	1.607816	2.371766
C	3.685017	2.862143	3.221376
O	5.031012	1.606741	1.710680
C	0.070316	1.477610	−1.246595
C	−1.235193	1.982734	−1.184359
C	−2.132440	1.463068	−0.248039
C	−1.775834	0.373961	0.554554
C	−0.466149	−0.117168	0.497767
C	0.466542	0.438351	−0.396627
C	−0.056625	−1.208013	1.462047
C	−0.964790	−1.215631	2.684272
C	−0.693977	−2.370038	3.631042
O	−2.320598	−1.340608	2.240882
C	−2.819391	−0.223420	1.494985
C	5.312996	0.366433	1.080463
C	−4.068458	−0.737077	0.798672
C	−3.981300	−1.516390	−0.354942
C	−5.121516	−2.042370	−0.951714
C	−6.349828	−1.778252	−0.370639
C	−6.478377	−1.015471	0.775052
C	−5.323037	−0.500359	1.356221
F	−7.470911	−2.286693	−0.950012
O	3.750238	−2.649581	−2.383508
C	2.753192	−3.141378	−3.276652
O	5.718167	−1.660443	−0.770987
C	5.927970	−3.009643	−0.326356
O	−1.617132	3.000074	−2.029762
C	−2.418719	2.576362	−3.147395
O	−3.412358	1.959984	−0.163238
C	−3.527120	3.304421	0.334943
O	0.951332	1.928491	−2.197823
C	1.361754	3.303454	−2.116068
H	1.326561	−1.483295	−2.010355
H	2.599257	2.488029	0.804570
H	1.688494	1.390140	1.820904
H	3.707604	0.722258	3.023163
H	3.784938	3.751790	2.595899
H	2.727394	2.913001	3.743336
H	4.483841	2.870962	3.964138
H	0.981560	−1.067203	1.766829
H	−0.109983	−2.190885	0.981061
H	−0.851254	−0.264606	3.225020
H	−0.836067	−3.324126	3.119268
H	−1.371354	−2.332551	4.485243
H	0.332032	−2.325533	4.001650
H	−3.125962	0.558011	2.203628
H	5.565191	−0.387120	1.841572
H	6.204682	0.523839	0.475198
H	−3.013726	−1.713967	−0.799145
H	−5.065493	−2.645325	−1.848560
H	−7.457128	−0.832743	1.198712
H	−5.405552	0.099715	2.254682
H	3.248359	−3.892215	−3.887466
H	2.369347	−2.345429	−3.919943
H	1.924598	−3.604562	−2.734111
H	6.997435	−3.193216	−0.409553
H	5.620811	−3.123478	0.717224
H	5.384100	−3.718803	−0.950497
H	−3.356260	2.133866	−2.806445
H	−1.869226	1.858876	−3.761189
H	−2.625798	3.471258	−3.730777
H	−3.099140	3.377253	1.338351
H	−4.592827	3.518640	0.379679
H	−3.033815	4.013681	−0.329486
H	2.096282	3.442167	−2.906579
H	0.519109	3.976809	−2.266972
H	1.831331	3.505344	−1.150483

B3LYP Energy = −1829.33375499 a.u.

(*aS*,1*R*,3*S*,3'*S*)-**21**, Conf E

C	1.967166	−1.543976	−0.816310
C	3.207178	−2.174932	−0.840225
C	4.311821	−1.522022	−0.270987
C	4.155703	−0.273504	0.326515
C	2.901426	0.349968	0.369498
C	1.804863	−0.289335	−0.221094
C	2.767220	1.702449	1.034510
C	3.961175	2.029442	1.927448
C	3.991456	3.479207	2.372853
O	5.175151	1.770138	1.210350
C	0.163926	1.387138	−1.088308
C	−1.091720	2.012132	−1.094846
C	−2.099396	1.524310	−0.254679
C	−1.857072	0.412769	0.568017
C	−0.578227	−0.148167	0.621589
C	0.442708	0.330497	−0.220531
C	−0.290592	−1.214452	1.655325
C	−1.294565	−1.140847	2.797957
C	−1.148893	−2.268491	3.802193
O	−2.613241	−1.229006	2.246334
C	−3.000335	−0.117334	1.429188
C	5.362622	0.385434	0.960296
C	−4.202698	−0.605146	0.638292
C	−4.044537	−1.415559	−0.485812
C	−5.147200	−1.915745	−1.169129
C	−6.410668	−1.593012	−0.704941
C	−6.610007	−0.797300	0.407828
C	−5.491282	−0.308661	1.076764
F	−7.495392	−2.075428	−1.369896
O	3.437208	−3.409523	−1.376276
C	2.340647	−4.115047	−1.952652
O	5.550707	−2.122968	−0.215074
C	6.229138	−2.290673	−1.470858
O	−1.383007	3.019788	−1.983829
C	−0.663944	4.256108	−1.819210
O	−3.368621	2.034867	−0.236267
C	−3.561515	3.451869	−0.123082
O	1.149724	1.858209	−1.929346
C	1.057090	1.393481	−3.286044
H	1.105664	−2.026220	−1.255761
H	2.674933	2.478116	0.266731
H	1.849991	1.739566	1.628091
H	3.939633	1.376562	2.813134
H	4.071354	4.140691	1.507500
H	3.078103	3.730291	2.915941
H	4.844013	3.663027	3.028217
H	0.724244	−1.089716	2.038599
H	−0.337946	−2.215969	1.215022
H	−1.185864	−0.175412	3.313393
H	−1.294017	−3.234735	3.314629
H	−1.886612	−2.170241	4.599723
H	−0.152772	−2.252428	4.248689
H	−3.335955	0.698048	2.084050
H	5.615182	−0.131891	1.897720
H	6.230053	0.304976	0.306702
H	−3.049757	−1.657100	−0.838761
H	−5.035944	−2.541628	−2.044862
H	−7.614437	−0.568680	0.739076
H	−5.629068	0.316988	1.950640
H	1.561559	−4.313264	−1.211825
H	2.749156	−5.058403	−2.306585
H	1.911806	−3.566513	−2.795643
H	7.203326	−2.713915	−1.234019
H	5.682311	−2.968807	−2.125705
H	6.363198	−1.325236	−1.966990
H	−1.053803	4.932047	−2.577500
H	0.405397	4.110885	−1.963762
H	−0.848101	4.677008	−0.827292
H	−3.436956	3.947379	−1.083753
H	−2.871929	3.880980	0.608318
H	−4.583332	3.582529	0.228699
H	0.107823	1.689684	−3.736749
H	1.880404	1.857768	−3.825261
H	1.164572	0.306791	−3.325874

B3LYP Energy = −1829.33368509 a.u.

(*aS*,1*R*,3*S*,3'*S*)-**21**, Conf F

C	−2.084508	1.461508	−0.869887
C	−3.345697	2.049542	−0.884604
C	−4.416799	1.373580	−0.279380
C	−4.207358	0.144847	0.342615
C	−2.931971	−0.434755	0.376254
C	−1.869117	0.228207	−0.248334
C	−2.739043	−1.767512	1.065881
C	−3.905637	−2.117578	1.985901
C	−3.878601	−3.558618	2.458612
O	−5.140311	−1.913114	1.286311
C	−0.176232	−1.391058	−1.123684
C	1.107944	−1.954060	−1.141165
C	2.071977	−1.473238	−0.250050
C	1.802724	−0.374278	0.573429
C	0.512486	0.166819	0.598975
C	−0.484945	−0.342059	−0.253097
C	0.187442	1.239595	1.615124
C	1.175404	1.206549	2.773333
C	0.999497	2.355543	3.748004
O	2.502109	1.298455	2.240086
C	2.914835	0.163042	1.471869
C	−5.379099	−0.540978	1.012937
C	4.156869	0.615183	0.723839
C	4.059101	1.422041	−0.409781
C	5.197593	1.891289	−1.055120
C	6.435266	1.541067	−0.543417
C	6.574708	0.748512	0.580621
C	5.420963	0.291674	1.211605
F	7.554493	1.992830	−1.171087
O	−3.626588	3.263188	−1.443974
C	−2.565038	3.991112	−2.056651
O	−5.674050	1.934056	−0.212406

C	−6.381279	2.051244	−1.457928
O	1.349087	−3.018991	−1.973502
C	2.378837	−2.839304	−2.962784
O	3.319844	−2.054834	−0.193551
C	3.353854	−3.370306	0.388319
O	−1.169021	−1.932685	−1.905572
C	−1.161000	−1.551209	−3.286335
H	−1.247986	1.962847	−1.335524
H	−2.630229	−2.554578	0.312126
H	−1.811649	−1.760153	1.644624
H	−3.891337	−1.447353	2.858803
H	−3.948084	−4.238857	1.607167
H	−2.948716	−3.767503	2.991345
H	−4.714141	−3.759121	3.130916
H	−0.829473	1.098626	1.986630
H	0.218095	2.234840	1.159118
H	1.072602	0.252110	3.309951
H	1.139365	3.311689	3.239400
H	1.725975	2.286132	4.558727
H	−0.003145	2.336908	4.179525
H	3.210564	−0.636299	2.164979
H	−5.631010	−0.013212	1.944796
H	−6.261125	−0.502703	0.375174
H	3.083904	1.687095	−0.798678
H	5.133447	2.515574	−1.936706
H	7.560619	0.498662	0.949897
H	5.511970	−0.330062	2.094341
H	−1.777385	4.228323	−1.336599
H	−3.010412	4.914042	−2.419874
H	−2.136402	3.439832	−2.898039
H	−5.872701	2.736729	−2.135595
H	−6.487561	1.071831	−1.932901
H	−7.366370	2.442065	−1.210642
H	3.359107	−2.721377	−2.502028
H	2.162893	−1.966999	−3.585111
H	2.360959	−3.736558	−3.577677
H	2.976859	−3.344807	1.413957
H	4.399221	−3.671565	0.393277
H	2.767330	−4.077760	−0.199339
H	−0.255293	−1.895704	−3.788688
H	−2.027085	−2.028865	−3.740115
H	−1.250860	−0.466294	−3.387188

B3LYP Energy = −1829.33360796 a.u.

(*aS*,1*R*,3*S*,3’*S*)-**21**, Conf G

C	1.969409	−1.349360	−1.062908
C	3.216252	−1.950015	−1.182990
C	4.320117	−1.367335	−0.553321
C	4.153923	−0.216069	0.220990
C	2.892176	0.372950	0.357769
C	1.791317	−0.191711	−0.307287
C	2.744896	1.618806	1.203114
C	3.942499	1.840530	2.122792
C	3.952162	3.215141	2.763849
O	5.152339	1.705470	1.365633
C	0.118054	1.548197	−0.958991
C	−1.150913	2.136040	−0.882108
C	−2.147820	1.525861	−0.117703
C	−1.870223	0.356010	0.604592
C	−0.574140	−0.171988	0.592718
C	0.422176	0.405376	−0.216295
C	−0.245898	−1.329673	1.510469
C	−1.233011	−1.394122	2.667453
C	−1.048074	−2.612651	3.552347
O	−2.557764	−1.457022	2.127027
C	−2.981561	−0.271666	1.443899
C	5.359758	0.373818	0.921522
C	−4.204257	−0.688186	0.645571
C	−5.481760	−0.353807	1.088977
C	−6.616021	−0.782036	0.404071
C	−6.443850	−1.559124	−0.726329
C	−5.191975	−1.919938	−1.195037
C	−4.073406	−1.477741	−0.497742
F	−7.543632	−1.984258	−1.404187
O	3.343235	−3.079648	−1.964936
C	3.572469	−4.299005	−1.239754
O	5.575440	−1.934746	−0.623737
C	6.207189	−1.883906	−1.915789
O	−1.422579	3.295675	−1.572805
C	−0.956673	4.494921	−0.929625
O	−3.381021	2.108650	0.037684
C	−4.192072	2.272295	−1.140704
O	1.103923	2.161037	−1.697023
C	0.974538	2.032648	−3.122958
H	1.129723	−1.807291	−1.571189
H	2.633508	2.489083	0.547898
H	1.831850	1.559960	1.801239
H	3.941527	1.070590	2.908872
H	4.002872	3.991257	1.997098
H	3.043553	3.368404	3.349499
H	4.812320	3.325559	3.425647
H	0.771677	−1.220581	1.890864
H	−0.277345	−2.282396	0.971390
H	−1.138437	−0.482805	3.275640
H	−1.174148	−3.528918	2.971704
H	−1.779421	−2.613531	4.361679
H	−0.048052	−2.615629	3.990542
H	−3.300331	0.467475	2.190517
H	5.639607	−0.264016	1.773258
H	6.216307	0.403277	0.249332
H	−5.598287	0.254960	1.977583
H	−7.612037	−0.523877	0.738960
H	−5.101760	−2.531831	−2.082971
H	−3.087139	−1.751845	−0.850675
H	2.733268	−4.509288	−0.571022
H	4.497812	−4.247764	−0.663582
H	3.646980	−5.088163	−1.985269
H	5.631037	−2.438380	−2.656932
H	6.325638	−0.847384	−2.242390
H	7.187578	−2.339236	−1.791715
H	0.130045	4.482725	−0.829808

H	−1.421161	4.608177	0.052885
H	−1.255782	5.321494	−1.571146
H	−5.138005	2.682747	−0.794144
H	−4.369352	1.305023	−1.615088
H	−3.723361	2.955631	−1.846398
H	0.037268	2.468942	−3.470979
H	1.814614	2.573402	−3.553979
H	1.029856	0.981668	−3.417945

B3LYP Energy = −1829.33335425 a.u.

(*aS*,1*R*,3*S*,3'*S*)-**21**, Conf H

C	1.963482	−1.407900	−1.043026
C	3.209281	−2.016824	−1.158822
C	4.319838	−1.410441	−0.551635
C	4.176995	−0.197041	0.117118
C	2.921735	0.415874	0.230896
C	1.809599	−0.199505	−0.356564
C	2.802081	1.723624	0.981885
C	4.017933	1.993874	1.863427
C	4.052122	3.406633	2.414798
O	5.210899	1.799804	1.093014
C	0.115348	1.507623	−1.046095
C	−1.151206	2.105988	−0.970964
C	−2.122014	1.541507	−0.135287
C	−1.832488	0.381267	0.600686
C	−0.543537	−0.157886	0.573631
C	0.440607	0.398039	−0.264940
C	−0.204384	−1.286883	1.522276
C	−1.166685	−1.301785	2.702133
C	−0.968137	−2.486615	3.628869
O	−2.502515	−1.379730	2.192785
C	−2.937021	−0.227112	1.460084
C	5.405137	0.440941	0.732941
C	−4.153585	−0.690389	0.676315
C	−5.434015	−0.439764	1.164305
C	−6.563038	−0.911547	0.500519
C	−6.382163	−1.644445	−0.657587
C	−5.127106	−1.920044	−1.172296
C	−4.014049	−1.437347	−0.493306
F	−7.477128	−2.110567	−1.317274
O	3.445025	−3.177564	−1.837816
C	2.351361	−3.816708	−2.491982
O	5.582748	−1.948499	−0.673239
C	5.814878	−3.176810	0.033683
O	−1.490054	3.162469	−1.783034
C	−0.789347	4.400759	−1.563374
O	−3.399512	2.021445	−0.040057
C	−3.620451	3.425622	0.153259
O	1.063547	2.056880	−1.882606
C	0.953127	1.661487	−3.259775
H	1.094692	−1.861057	−1.498749
H	2.693665	2.546413	0.267194
H	1.898600	1.721883	1.597245
H	4.026415	1.276847	2.698420
H	4.094589	4.131746	1.599294
H	3.157030	3.606066	3.007447
H	4.926425	3.550448	3.051225
H	0.822559	−1.173489	1.875375
H	−0.257554	−2.257689	1.018451
H	−1.054238	−0.368311	3.272590
H	−1.110559	−3.423614	3.086388
H	−1.682171	−2.453139	4.452878
H	0.040964	−2.477949	4.045639
H	−3.267996	0.537613	2.175429
H	5.718615	−0.136229	1.615876
H	6.233706	0.423838	0.026244
H	−5.557325	0.136225	2.073766
H	−7.561377	−0.718154	0.870363
H	−5.030174	−2.497450	−2.082358
H	−3.025702	−1.643002	−0.884970
H	1.580984	−4.120475	−1.778037
H	2.767964	−4.699946	−2.969895
H	1.908870	−3.168228	−3.252696
H	6.863312	−3.423062	−0.122771
H	5.629048	−3.046597	1.103680
H	5.187194	−3.978923	−0.354907
H	−1.206122	5.110339	−2.275185
H	0.279002	4.282999	−1.736516
H	−0.961174	4.762797	−0.546330
H	−3.548664	3.971315	−0.785269
H	−2.910503	3.835347	0.876207
H	−4.628955	3.511871	0.553747
H	1.746366	2.182404	−3.792165
H	1.096684	0.583075	−3.360199
H	−0.016912	1.947578	−3.671358

B3LYP Energy = −1829.33334384 a.u.

(*aS*,1*R*,3*S*,3'*S*)-**21**, Conf I

C	2.064898	−1.305213	−1.116020
C	3.329467	−1.874237	−1.233911
C	4.412064	−1.262821	−0.583053
C	4.223931	−0.081375	0.130530
C	2.950081	0.491199	0.246903
C	1.866242	−0.131533	−0.383438
C	2.779512	1.764733	1.045266
C	3.974413	2.039030	1.953801
C	3.958728	3.432657	2.552651
O	5.184210	1.905660	1.196674
C	0.124516	1.532644	−1.056931
C	−1.167110	2.074523	−0.980494
C	−2.092587	1.503239	−0.102115
C	−1.778447	0.345229	0.617833
C	−0.480236	−0.172685	0.555046
C	0.478125	0.420312	−0.287029
C	−0.105406	−1.315668	1.472995
C	−1.045839	−1.374912	2.669166
C	−0.815010	−2.580842	3.560473
O	−2.390942	−1.456389	2.182661
C	−2.852633	−0.284448	1.501181
C	5.422663	0.566699	0.792097

C	−4.096706	−0.721988	0.747218
C	−5.360157	−0.458237	1.271188
C	−6.512740	−0.907813	0.632805
C	−6.372687	−1.632854	−0.535793
C	−5.135442	−1.921900	−1.085524
C	−3.998303	−1.460878	−0.432065
F	−7.490691	−2.077808	−1.170339
O	3.608803	−3.000026	−1.954402
C	2.544162	−3.642968	−2.650708
O	5.691505	−1.761388	−0.701746
C	5.944604	−3.010852	−0.040834
O	−1.451840	3.205795	−1.705277
C	−2.515501	3.099557	−2.669515
O	−3.346580	2.055327	0.046005
C	−3.380658	3.316983	0.736977
O	1.086781	2.146414	−1.821320
C	0.981801	1.974094	−3.240215
H	1.216367	−1.763800	−1.603616
H	2.651887	2.610042	0.360879
H	1.869192	1.711066	1.648239
H	3.991800	1.293997	2.763803
H	3.990740	4.185753	1.762456
H	3.049291	3.585599	3.137192
H	4.818940	3.580657	3.207157
H	0.925425	−1.193156	1.810894
H	−0.151132	−2.275500	0.947618
H	−0.935764	−0.455394	3.262195
H	−0.954718	−3.505305	2.996164
H	−1.514114	−2.578781	4.397848
H	0.201352	−2.569216	3.959101
H	−3.156442	0.457289	2.251980
H	5.736207	−0.033539	1.659612
H	6.264085	0.598004	0.101218
H	−5.451827	0.110121	2.189125
H	−7.498192	−0.704156	1.030572
H	−5.070606	−2.494499	−2.001469
H	−3.023452	−1.678984	−0.849980
H	2.992693	−4.495435	−3.154926
H	2.095701	−2.978081	−3.393628
H	1.771301	−3.995004	−1.961950
H	5.351082	−3.814169	−0.477658
H	7.003502	−3.219005	−0.181446
H	5.729860	−2.931751	1.028785
H	−3.474320	2.913521	−2.186889
H	−2.306191	2.298314	−3.383066
H	−2.539708	4.053206	−3.192438
H	−2.957402	3.216033	1.739722
H	−4.430589	3.591820	0.812164
H	−2.835352	4.084274	0.185896
H	0.088012	2.459412	−3.634227
H	1.864934	2.443351	−3.668984
H	0.973667	0.912396	−3.500953

B3LYP Energy = −1829.33328584 a.u.

(*aS*,1*R*,3*S*,3’*S*)-**21**, Conf J

C	1.958721	−1.337049	−1.067773
C	3.205502	−1.935520	−1.197979
C	4.307259	−1.368909	−0.550983
C	4.158096	−0.180710	0.169308
C	2.901128	0.420904	0.295576
C	1.788156	−0.168046	−0.327798
C	2.769031	1.691359	1.105759
C	3.974341	1.928568	2.011040
C	3.996221	3.317788	2.619548
O	5.176685	1.770045	1.246775
C	0.088593	1.559360	−0.953898
C	−1.185999	2.132472	−0.858422
C	−2.166148	1.508025	−0.084134
C	−1.865809	0.339169	0.630650
C	−0.563923	−0.173301	0.601149
C	0.415808	0.418036	−0.218609
C	−0.210755	−1.328937	1.512327
C	−1.183167	−1.407496	2.680872
C	−0.972963	−2.625182	3.561292
O	−2.513459	−1.485738	2.156138
C	−2.959759	−0.304635	1.480305
C	5.376827	0.428515	0.831201
C	−4.185190	−0.735235	0.693559
C	−5.462381	−0.419898	1.151586
C	−6.597867	−0.861405	0.477205
C	−6.427080	−1.632579	−0.657439
C	−5.175434	−1.974876	−1.140411
C	−4.055709	−1.519573	−0.453506
F	−7.528100	−2.070721	−1.324987
O	3.316822	−3.105034	−1.921138
C	3.984701	−2.976247	−3.187204
O	5.567985	−1.917770	−0.657903
C	5.755339	−3.180673	0.004188
O	−1.479380	3.291510	−1.541439
C	−1.021453	4.493670	−0.897985
O	−3.404503	2.074897	0.088268
C	−4.230957	2.234489	−1.079899
O	1.056887	2.184693	−1.704526
C	0.903998	2.065656	−3.128719
H	1.114686	−1.799585	−1.564546
H	2.661202	2.543501	0.426729
H	1.859283	1.656162	1.710660
H	3.976149	1.177590	2.815410
H	4.043307	4.075471	1.834414
H	3.093970	3.489008	3.210050
H	4.863149	3.439559	3.270461
H	0.809884	−1.207797	1.880738
H	−0.236441	−2.281049	0.972009
H	−1.092715	−0.496083	3.289588
H	−1.094330	−3.541960	2.980506
H	−1.694715	−2.636415	4.379165
H	0.032120	−2.616543	3.987684
H	−3.280336	0.428877	2.231670
H	5.677929	−0.186721	1.692488
H	6.215917	0.442069	0.136677
H	−5.577815	0.184214	2.043501

H	−7.593731	−0.618099	0.823468
H	−5.086251	−2.583088	−2.030977
H	−3.069577	−1.779367	−0.817437
H	3.433128	−2.296611	−3.842545
H	3.998710	−3.971295	−3.627465
H	5.006765	−2.615444	−3.059816
H	6.799108	−3.449198	−0.145232
H	5.554393	−3.085481	1.074612
H	5.110042	−3.948494	−0.423538
H	0.066231	4.493796	−0.808281
H	−1.477973	4.597857	0.089243
H	−1.336088	5.319137	−1.533476
H	−3.781725	2.931739	−1.784660
H	−5.180062	2.625418	−0.719718
H	−4.396561	1.268398	−1.560886
H	−0.040428	2.500839	−3.458120
H	1.735049	2.612011	−3.570039
H	0.957553	1.016794	−3.431666

B3LYP Energy = −1829.33317067 a.u.

(*aS*,1*R*,3*S*,3'*S*)-**21**, Conf K

C	2.138936	0.455972	−1.678467
C	3.402124	0.223728	−2.212255
C	4.419083	−0.249534	−1.367964
C	4.153298	−0.499631	−0.024251
C	2.873963	−0.281896	0.508304
C	1.866347	0.210663	−0.330226
C	2.626369	−0.575530	1.973449
C	3.702457	−1.480210	2.567715
C	3.625503	−1.585973	4.078829
O	4.993581	−0.956113	2.233043
C	0.122683	1.760608	0.572482
C	−1.175319	2.043119	1.026288
C	−2.113663	1.008576	1.102287
C	−1.785328	−0.279577	0.651524
C	−0.487477	−0.551514	0.210050
C	0.476984	0.471928	0.166191
C	−0.126506	−1.967223	−0.181322
C	−1.070594	−2.967807	0.469900
C	−0.856669	−4.394634	0.000819
O	−2.414623	−2.605660	0.134134
C	−2.858481	−1.365994	0.697925
C	5.266299	−1.050223	0.842800
C	−4.131831	−1.023376	−0.056480
C	−5.375589	−1.275847	0.518264
C	−6.554840	−1.033429	−0.181107
C	−6.461914	−0.542708	−1.470257
C	−5.245961	−0.287026	−2.080597
C	−4.081192	−0.533780	−1.362126
F	−7.606519	−0.300717	−2.164808
O	3.734675	0.411282	−3.522535
C	2.729216	0.879973	−4.418339
O	5.671790	−0.555187	−1.854900
C	6.473659	0.560263	−2.276999
O	−1.511317	3.297100	1.484338
C	−1.571062	4.335379	0.488930
O	−3.345200	1.161464	1.678719
C	−4.204325	2.238106	1.273266
O	1.054331	2.770976	0.482962
C	1.540698	3.306179	1.726121
H	1.342953	0.825547	−2.308825
H	2.608677	0.358015	2.546197
H	1.645331	−1.036239	2.109809
H	3.612600	−2.484447	2.126814
H	3.763041	−0.604297	4.537203
H	2.652691	−1.975966	4.384629
H	4.399727	−2.255002	4.456903
H	0.903699	−2.184144	0.104071
H	−0.179223	−2.091912	−1.268526
H	−0.951201	−2.920134	1.562313
H	−1.006376	−4.468403	−1.078376
H	−1.558144	−5.069265	0.493514
H	0.158654	−4.722688	0.232041
H	−3.124152	−1.536288	1.749865
H	5.458316	−2.098688	0.571235
H	6.191689	−0.501399	0.673243
H	−5.429665	−1.660447	1.529697
H	−7.524911	−1.222660	0.259384
H	−5.218075	0.093019	−3.093453
H	−3.122331	−0.341350	−1.826928
H	1.892810	0.178611	−4.478839
H	3.210003	0.951472	−5.390879
H	2.359214	1.865312	−4.122663
H	7.437426	0.147010	−2.568046
H	6.020524	1.071123	−3.126328
H	6.614540	1.265928	−1.453336
H	−0.599177	4.488205	0.020213
H	−1.876087	5.238514	1.013424
H	−2.312254	4.090045	−0.275976
H	−3.929825	3.167791	1.767484
H	−5.208466	1.941853	1.570379
H	−4.177091	2.368789	0.189441
H	2.279452	4.059025	1.459651
H	0.736681	3.762255	2.304422
H	2.020510	2.524877	2.318905

B3LYP Energy = −1829.33254463 a.u.

(*aR*,1*R*,3*S*,3'*S*)-**21**, Conf A

C	2.923127	1.120526	0.774649
C	4.263241	0.753367	0.831247
C	4.681839	−0.396339	0.142203
C	3.768746	−1.137542	−0.602540
C	2.421154	−0.754319	−0.675264
C	2.000073	0.377175	0.033577
C	1.463490	−1.578720	−1.510871
C	2.193966	−2.484521	−2.498887
C	1.285373	−3.506340	−3.155613
O	3.224739	−3.200435	−1.806827
C	0.182964	1.887057	−0.791112

C	−1.136881	2.358073	−0.772885
C	−2.092616	1.704357	0.007002
C	−1.727799	0.611168	0.806229
C	−0.389103	0.206383	0.851978
C	0.570121	0.820429	0.024612
C	0.024090	−0.852505	1.850672
C	−0.983817	−0.945227	2.987670
C	−0.710051	−2.089927	3.945066
O	−2.282985	−1.161416	2.424439
C	−2.795094	−0.056681	1.671514
C	4.268613	−2.343619	−1.369970
C	−3.972919	−0.616365	0.893605
C	−5.277047	−0.361394	1.311698
C	−6.367285	−0.916450	0.646991
C	−6.123638	−1.739547	−0.436820
C	−4.842819	−2.025764	−0.877771
C	−3.769287	−1.457172	−0.201478
F	−7.179949	−2.288473	−1.094596
O	5.227508	1.444232	1.506338
C	4.854336	2.639871	2.187716
O	6.007481	−0.771684	0.112758
C	6.554535	−1.255127	1.350448
O	−1.491337	3.456117	−1.525326
C	−1.192294	4.719106	−0.905340
O	−3.376146	2.180359	0.106771
C	−4.168802	2.213420	−1.094617
O	1.131083	2.551851	−1.533317
C	1.021390	2.417002	−2.959645
H	2.579244	2.002841	1.294995
H	0.842879	−2.207221	−0.862354
H	0.777812	−0.924770	−2.054283
H	2.666545	−1.864063	−3.274923
H	0.844610	−4.163635	−2.402882
H	0.476929	−3.008068	−3.694519
H	1.844922	−4.119067	−3.863813
H	1.013411	−0.616262	2.246474
H	0.103195	−1.836064	1.375206
H	−0.990645	0.003409	3.543908
H	−0.740477	−3.044867	3.416225
H	−1.456142	−2.111732	4.740551
H	0.276372	−1.977397	4.399253
H	−3.179997	0.695782	2.372802
H	4.861867	−2.008561	−2.233499
H	4.924255	−2.950495	−0.746998
H	−5.450019	0.285073	2.163655
H	−7.383658	−0.720406	0.962304
H	−4.696220	−2.678393	−1.728399
H	−2.761077	−1.672145	−0.532555
H	4.449652	3.381744	1.494495
H	5.768632	3.023427	2.633853
H	4.123167	2.438818	2.975473
H	6.558211	−0.474013	2.110501
H	5.987641	−2.118715	1.709889
H	7.576121	−1.559482	1.131270
H	−1.534238	5.487506	−1.595903
H	−0.118083	4.824581	−0.741845
H	−1.725432	4.816617	0.043290
H	−4.213241	1.220439	−1.546471
H	−3.768165	2.930519	−1.808803
H	−5.167018	2.512348	−0.782446
H	1.141662	1.371823	−3.255860
H	0.064596	2.797438	−3.318395
H	1.833831	3.004803	−3.381907

B3LYP Energy = −1829.33508181 a.u.

(*aR*,1*R*,3*S*,3’*S*)-**21**, Conf B

C	2.862562	1.081118	0.842093
C	4.203346	0.721207	0.943307
C	4.663863	−0.391817	0.222440
C	3.792252	−1.104693	−0.596807
C	2.447922	−0.725816	−0.716564
C	1.983690	0.367970	0.022061
C	1.532908	−1.509073	−1.634064
C	2.310782	−2.384414	−2.613249
C	1.435891	−3.385429	−3.343471
O	3.311409	−3.121453	−1.898121
C	0.152539	1.751859	−0.972713
C	−1.177764	2.185267	−1.029786
C	−2.113109	1.659655	−0.136434
C	−1.748358	0.649658	0.762363
C	−0.410812	0.242766	0.833361
C	0.548530	0.792676	−0.036847
C	−0.003600	−0.741982	1.906414
C	−1.002582	−0.731242	3.055753
C	−0.730824	−1.796460	4.101454
O	−2.309349	−0.980406	2.525238
C	−2.820011	0.046526	1.667177
C	4.331990	−2.276806	−1.388853
C	−3.977252	−0.598735	0.923412
C	−5.282835	−0.413942	1.372749
C	−6.355070	−1.045862	0.749218
C	−6.091136	−1.871617	−0.327606
C	−4.807824	−2.088399	−0.799088
C	−3.752470	−1.445799	−0.161799
F	−7.130171	−2.493350	−0.948090
O	5.128254	1.385881	1.695925
C	4.710070	2.535886	2.426788
O	5.992675	−0.757003	0.238295
C	6.486063	−1.293363	1.476719
O	−1.556356	3.142362	−1.944337
C	−1.966958	2.613164	−3.217312
O	−3.425368	2.065826	−0.180076
C	−3.676168	3.427622	0.208157
O	1.059929	2.209567	−1.899267
C	1.491707	3.570635	−1.740862
H	2.485083	1.928167	1.397167
H	0.868611	−2.150824	−1.043605
H	0.891095	−0.824208	−2.192488
H	2.817335	−1.739214	−3.346128
H	0.965536	−4.069388	−2.633614
H	0.649572	−2.870579	−3.899196

H	2.027866	−3.972001	−4.047480
H	0.992693	−0.495039	2.275917
H	0.054389	−1.758687	1.501951
H	−0.993584	0.259989	3.532344
H	−0.770719	−2.790938	3.652320
H	−1.472557	−1.748158	4.899834
H	0.258812	−1.654421	4.540164
H	−3.228772	0.852091	2.292413
H	4.956367	−1.905972	−2.215020
H	4.966630	−2.903030	−0.763228
H	−5.471845	0.237256	2.217890
H	−7.372823	−0.904004	1.088192
H	−4.645244	−2.744092	−1.644436
H	−2.743037	−1.604911	−0.519811
H	4.329336	3.313833	1.759732
H	5.598010	2.901694	2.936505
H	3.945797	2.282774	3.166582
H	5.909519	−2.175145	1.770639
H	7.518638	−1.582520	1.291022
H	6.449889	−0.547471	2.270610
H	−2.243996	3.468839	−3.829863
H	−2.828760	1.952544	−3.099767
H	−1.144944	2.073451	−3.692163
H	−3.195819	4.126195	−0.476339
H	−3.324128	3.605223	1.228101
H	−4.755923	3.556241	0.170800
H	2.185056	3.764869	−2.556667
H	0.648315	4.259048	−1.801157
H	2.011541	3.696997	−0.787732

B3LYP Energy = −1829.33497895 a.u.

(*aR*,1*R*,3*S*,3’*S*)-**21**, Conf C

C	2.916653	1.061351	0.863118
C	4.241871	0.656451	0.983265
C	4.651269	−0.525790	0.346073
C	3.730793	−1.296469	−0.358934
C	2.393743	−0.889058	−0.475960
C	1.991277	0.301108	0.142660
C	1.430854	−1.744009	−1.273333
C	2.157588	−2.723872	−2.190464
C	1.236092	−3.759503	−2.806188
O	3.152224	−3.423985	−1.433013
C	0.243820	1.806630	−0.827773
C	−1.063161	2.309156	−0.885494
C	−2.061468	1.727977	−0.101739
C	−1.751584	0.677997	0.775013
C	−0.426365	0.245377	0.893046
C	0.576040	0.783051	0.063372
C	−0.076185	−0.759402	1.968080
C	−1.124376	−0.757789	3.072410
C	−0.911219	−1.847087	4.106481
O	−2.408900	−0.978383	2.477576
C	−2.866268	0.085128	1.635098
C	4.209888	−2.574716	−1.016127
C	−4.019981	−0.501479	0.840394
C	−3.785557	−1.410802	−0.192119
C	−4.839502	−1.999089	−0.882193
C	−6.132415	−1.662335	−0.519121
C	−6.406798	−0.770707	0.501211
C	−5.335853	−0.196734	1.181170
F	−7.169579	−2.229789	−1.191574
O	5.196055	1.328668	1.690594
C	4.821257	2.526208	2.367465
O	5.941803	−0.993928	0.467830
C	6.954138	−0.233676	−0.210414
O	−1.360557	3.364798	−1.718886
C	−1.075878	4.658115	−1.157699
O	−3.335995	2.236504	−0.076955
C	−4.083466	2.224588	−1.307389
O	1.231003	2.403244	−1.577739
C	1.177388	2.158346	−2.992020
H	2.582319	1.974484	1.334208
H	0.788334	−2.319896	−0.597912
H	0.766684	−1.111195	−1.866267
H	2.663119	−2.162239	−2.990380
H	0.759388	−4.356156	−2.025404
H	0.455045	−3.273975	−3.394796
H	1.794439	−4.430205	−3.460785
H	0.905144	−0.524573	2.384134
H	−0.007685	−1.771881	1.556129
H	−1.125996	0.223395	3.568955
H	−0.945351	−2.831889	3.635938
H	−1.684751	−1.803882	4.874378
H	0.061284	−1.727804	4.587972
H	−3.266109	0.887077	2.269772
H	4.850190	−2.329715	−1.876781
H	4.814557	−3.154130	−0.319692
H	−2.768447	−1.664703	−0.462978
H	−4.668745	−2.705089	−1.684318
H	−7.431884	−0.536383	0.756558
H	−5.533167	0.503903	1.983558
H	4.470868	3.287868	1.665891
H	5.721730	2.879279	2.864049
H	4.047026	2.334139	3.114995
H	7.885381	−0.780130	−0.074229
H	6.729643	−0.160032	−1.278313
H	7.048037	0.765085	0.216309
H	−1.666015	4.822665	−0.253070
H	−1.356908	5.388741	−1.913684
H	−0.012214	4.755306	−0.931647
H	−5.087666	2.551340	−1.046553
H	−4.126771	1.212547	−1.714992
H	−3.646173	2.901828	−2.038729
H	2.010029	2.708110	−3.426256
H	1.301542	1.092638	−3.200755
H	0.238681	2.514439	−3.417746

B3LYP Energy = −1829.33481287 a.u.

(*aR*,1*R*,3*S*,3’*S*)-**21**, Conf D

C	−2.848008	−1.073797	0.868630

C	−4.177750	−0.706464	1.053842
C	−4.648475	0.469029	0.448236
C	−3.781913	1.272899	−0.288246
C	−2.442625	0.902065	−0.471986
C	−1.979944	−0.282074	0.111911
C	−1.536098	1.783315	−1.304489
C	−2.324727	2.767069	−2.164000
C	−1.454374	3.834753	−2.798913
O	−3.300085	3.429555	−1.348095
C	−0.221772	−1.586755	−1.092597
C	1.093754	−2.040682	−1.245566
C	2.072343	−1.636125	−0.335219
C	1.768447	−0.718736	0.677752
C	0.445406	−0.290193	0.840203
C	−0.557668	−0.723752	−0.045493
C	0.102005	0.587975	2.022478
C	1.138484	0.437253	3.127768
C	0.932733	1.398351	4.283386
O	2.432816	0.706970	2.576075
C	2.885421	−0.242726	1.604850
C	−4.319710	2.547647	−0.905111
C	4.041086	0.438041	0.891404
C	5.354864	0.136007	1.242257
C	6.428214	0.791218	0.645031
C	6.157718	1.759854	−0.303392
C	4.866631	2.095746	−0.673126
C	3.810414	1.426844	−0.065230
F	7.197544	2.406635	−0.896273
O	−5.079054	−1.411268	1.798478
C	−4.637048	−2.594562	2.458774
O	−5.945073	0.898049	0.634251
C	−6.963417	0.117685	−0.011288
O	1.407304	−2.904828	−2.270409
C	1.968120	−2.268706	−3.432430
O	3.368825	−2.075042	−0.469547
C	3.585679	−3.472518	−0.208337
O	−1.176844	−1.922141	−2.022978
C	−1.622626	−3.287482	−2.006115
H	−2.466576	−1.977188	1.322816
H	−0.862618	2.352582	−0.653261
H	−0.903105	1.166542	−1.946345
H	−2.853787	2.210589	−2.951941
H	−0.959597	4.430063	−2.028353
H	−0.687299	3.376503	−3.426316
H	−2.054216	4.502233	−3.419190
H	−0.888166	0.329335	2.400712
H	0.060080	1.641988	1.726195
H	1.118711	−0.596391	3.503195
H	0.986148	2.431763	3.934705
H	1.699010	1.251146	5.045698
H	−0.045517	1.238217	4.740849
H	3.284480	−1.119625	2.132912
H	−4.986515	2.299324	−1.744446
H	−4.911037	3.100159	−0.176071
H	5.549652	−0.625373	1.988168
H	7.451993	0.559633	0.907979
H	4.698757	2.862605	−1.417937
H	2.794874	1.679314	−0.343474
H	−4.293626	−3.346796	1.743426
H	−5.503104	−2.975684	2.994266
H	−3.837446	−2.376638	3.171715
H	−6.787816	0.072074	−1.089806
H	−7.004225	−0.891655	0.398802
H	−7.904578	0.629519	0.180034
H	2.168822	−3.062725	−4.148958
H	2.899219	−1.756594	−3.181993
H	1.256553	−1.559990	−3.862312
H	3.049305	−4.094176	−0.925065
H	3.272632	−3.724626	0.808578
H	4.657079	−3.634002	−0.307253
H	−2.360849	−3.374462	−2.800857
H	−0.795585	−3.972410	−2.194110
H	−2.093650	−3.522396	−1.047982

B3LYP Energy = −1829.33468082 a.u.

(*aR*,1*R*,3*S*,3’*S*)-**21**, Conf E

C	−2.867028	0.563271	−1.260797
C	−4.207927	0.196566	−1.194040
C	−4.676974	−0.455795	−0.042824
C	−3.812532	−0.710637	1.018777
C	−2.467159	−0.321947	0.957670
C	−1.995527	0.310240	−0.197711
C	−1.556806	−0.605041	2.133854
C	−2.337521	−0.938143	3.402943
C	−1.465035	−1.507373	4.505425
O	−3.345610	−1.911806	3.099949
C	−0.162415	1.980288	0.108839
C	1.169178	2.404649	−0.013279
C	2.106042	1.546961	−0.599637
C	1.729410	0.257473	−1.007178
C	0.394380	−0.144381	−0.900916
C	−0.559692	0.715810	−0.328112
C	−0.017256	−1.490192	−1.455765
C	0.978883	−1.972426	−2.500314
C	0.710009	−3.384487	−2.985535
O	2.286983	−1.963186	−1.917918
C	2.792675	−0.659154	−1.610499
C	−4.360214	−1.384860	2.258772
C	3.993612	−0.903217	−0.712817
C	5.284297	−0.831857	−1.232340
C	6.395159	−1.117229	−0.443048
C	6.186022	−1.485581	0.873008
C	4.919714	−1.578008	1.424625
C	3.824996	−1.285652	0.618557
F	7.262985	−1.767043	1.655149
O	−5.125490	0.436401	−2.175914
C	−4.697221	1.105325	−3.359615
O	−6.007555	−0.785157	0.102790
C	−6.492840	−1.840143	−0.743388
O	1.543331	3.682936	0.332289
C	1.506419	4.000587	1.735983

O	3.386661	1.934942	−0.887339
C	4.208402	2.503416	0.143433
O	−1.089131	2.813851	0.697593
C	−1.553304	3.899724	−0.121851
H	−2.483761	1.059267	−2.141231
H	−0.893924	−1.446193	1.899630
H	−0.912660	0.256492	2.322700
H	−2.837032	−0.027489	3.765329
H	−1.002767	−2.441974	4.180044
H	−0.672566	−0.802986	4.765997
H	−2.056516	−1.707413	5.399948
H	−1.013559	−1.420354	−1.894635
H	−0.078394	−2.237922	−0.656902
H	0.965915	−1.282873	−3.357147
H	0.755567	−4.089286	−2.152768
H	1.449658	−3.681486	−3.730279
H	−0.281442	−3.447974	−3.438298
H	3.152496	−0.196145	−2.539229
H	−4.978971	−0.671287	2.822634
H	−5.002299	−2.221994	1.988437
H	5.430439	−0.542162	−2.266008
H	7.401085	−1.060085	−0.837693
H	4.799744	−1.874890	2.458320
H	2.827767	−1.357535	1.034336
H	−5.580564	1.188826	−3.987940
H	−3.929954	0.532306	−3.887322
H	−4.316483	2.105421	−3.136201
H	−6.441784	−1.556383	−1.794505
H	−5.921343	−2.758313	−0.579848
H	−7.529897	−2.004624	−0.457443
H	1.847635	5.030337	1.819835
H	2.181538	3.347056	2.294324
H	0.496109	3.910264	2.133054
H	3.987714	3.559740	0.283811
H	5.235247	2.380523	−0.195358
H	4.076210	1.965047	1.084329
H	−0.727863	4.547657	−0.420947
H	−2.064753	3.517135	−1.008059
H	−2.259440	4.459718	0.487824

B3LYP Energy = −1829.33373011 a.u.

(*aR*,1*R*,3*S*,3'*S*)-**21**, Conf F

C	2.849117	0.705851	1.197736
C	4.173923	0.282376	1.254611
C	4.640404	−0.620644	0.286795
C	3.774708	−1.112809	−0.687043
C	2.441516	−0.684358	−0.742729
C	1.981872	0.231804	0.209757
C	1.535830	−1.214235	−1.832806
C	2.322668	−1.861912	−2.969080
C	1.445743	−2.639754	−3.931588
O	3.283218	−2.773956	−2.420869
C	0.217396	1.841794	−0.525973
C	−1.098148	2.329490	−0.535913
C	−2.074633	1.669120	0.217024
C	−1.752971	0.500438	0.925458
C	−0.433593	0.039429	0.947131
C	0.560836	0.705408	0.207460
C	−0.084392	−1.150210	1.813882
C	−1.111980	−1.331172	2.921738
C	−0.905238	−2.593162	3.738286
O	−2.411440	−1.417729	2.325613
C	−2.862194	−0.212881	1.697117
C	4.306929	−2.107438	−1.697846
C	−4.045276	−0.631253	0.840583
C	−5.346949	−0.362263	1.257339
C	−6.444179	−0.795632	0.517554
C	−6.210230	−1.511836	−0.641509
C	−4.932707	−1.808269	−1.085027
C	−3.852027	−1.362962	−0.331891
F	−7.273328	−1.941178	−1.374057
O	5.073549	0.679637	2.201640
C	4.634505	1.571827	3.223057
O	5.930600	−1.104022	0.323957
C	6.963931	−0.162231	−0.004528
O	−1.415577	3.502396	−1.182171
C	−1.359431	3.472262	−2.620158
O	−3.343803	2.153521	0.378438
C	−4.120875	2.527915	−0.769022
O	1.181336	2.480053	−1.277712
C	1.676111	3.713023	−0.728996
H	2.470342	1.401208	1.933177
H	0.843154	−1.956094	−1.418458
H	0.923181	−0.403757	−2.233618
H	2.865207	−1.079303	−3.520270
H	0.937333	−3.453749	−3.410090
H	0.689554	−1.985321	−4.369706
H	2.043134	−3.065911	−4.738754
H	0.909144	−1.016629	2.244035
H	−0.046588	−2.068607	1.217367
H	−1.083307	−0.456116	3.587351
H	−0.969548	−3.476363	3.099323
H	−1.664583	−2.674814	4.517259
H	0.077565	−2.581595	4.213615
H	−3.230750	0.471016	2.473174
H	4.984008	−1.596254	−2.398649
H	4.886050	−2.882142	−1.197020
H	−5.511917	0.199501	2.168866
H	−7.458379	−0.588080	0.832578
H	−4.793922	−2.376791	−1.995305
H	−2.846640	−1.591620	−0.662526
H	4.303352	2.526046	2.804457
H	5.498507	1.738873	3.861518
H	3.826739	1.132539	3.814334
H	7.009751	0.643140	0.728943
H	7.897788	−0.720949	0.005711
H	6.801718	0.254801	−1.002497
H	−1.634071	4.471169	−2.952811
H	−2.075538	2.747586	−3.016295
H	−0.356818	3.227277	−2.968661
H	−3.974626	1.817155	−1.585116

H	−3.870332	3.533211	−1.102051
H	−5.159907	2.491256	−0.447338
H	2.176149	3.532206	0.225358
H	2.398011	4.097312	−1.446888
H	0.869172	4.434909	−0.594190

B3LYP Energy = −1829.33357990 a.u.

(*aR*,1*R*,3*S*,3'*S*)-**21**, Conf G

C	2.909448	1.036384	0.796969
C	4.241461	0.639383	0.868834
C	4.656034	−0.483214	0.135091
C	3.748952	−1.167515	−0.669726
C	2.413436	−0.751004	−0.760247
C	1.994418	0.350206	−0.006152
C	1.459549	−1.501706	−1.665493
C	2.194568	−2.391052	−2.665013
C	1.278559	−3.360768	−3.386866
O	3.186438	−3.161723	−1.973050
C	0.189177	1.818753	−0.921414
C	−1.130645	2.294169	−0.934189
C	−2.085675	1.683358	−0.113849
C	−1.722881	0.657668	0.768231
C	−0.385453	0.252176	0.837007
C	0.569205	0.809429	−0.030588
C	0.023752	−0.746526	1.896270
C	−0.965372	−0.732454	3.053536
C	−0.690543	−1.799360	4.096773
O	−2.276766	−0.977134	2.532746
C	−2.792364	0.044606	1.670965
C	4.240215	−2.350295	−1.477139
C	−3.940825	−0.614811	0.926696
C	−5.259127	−0.353183	1.292432
C	−6.321619	−0.997549	0.663859
C	−6.035494	−1.915668	−0.329322
C	−4.738939	−2.211313	−0.715092
C	−3.694021	−1.552969	−0.076824
F	−7.064131	−2.552181	−0.950742
O	5.200469	1.275201	1.604012
C	4.829728	2.432135	2.349005
O	5.974146	−0.886003	0.123672
C	6.475501	−1.441733	1.350153
O	−1.470955	3.301521	−1.808024
C	−1.861791	4.547478	−1.201602
O	−3.388358	2.127366	−0.101407
C	−4.146787	1.952066	−1.313998
O	1.122686	2.271314	−1.819700
C	1.526369	3.645125	−1.748023
H	2.566909	1.892239	1.361252
H	0.788800	−2.129389	−1.067183
H	0.826435	−0.795546	−2.207336
H	2.706206	−1.754714	−3.402021
H	0.803222	−4.038165	−2.673948
H	0.496408	−2.819026	−3.922421
H	1.840252	−3.956884	−4.107467
H	1.025684	−0.511335	2.258007
H	0.068299	−1.761368	1.485487
H	−0.948740	0.258412	3.530476
H	−0.735955	−2.793444	3.647254
H	−1.427451	−1.749409	4.899524
H	0.302030	−1.660422	4.529783
H	−3.209221	0.848792	2.291423
H	4.860985	−1.993261	−2.312040
H	4.866773	−2.998503	−0.865900
H	−5.465577	0.367925	2.074177
H	−7.348893	−0.797600	0.938866
H	−4.558477	−2.939616	−1.494807
H	−2.674311	−1.773942	−0.366140
H	4.457219	3.224117	1.693896
H	5.737836	2.770317	2.842064
H	4.074036	2.196437	3.103289
H	7.495436	−1.760089	1.143429
H	6.476439	−0.698553	2.147461
H	5.879320	−2.307608	1.652146
H	−2.752291	4.425713	−0.585125
H	−2.069864	5.230029	−2.022893
H	−1.047875	4.948064	−0.591819
H	−4.197185	0.894998	−1.579363
H	−3.713742	2.519656	−2.136984
H	−5.146989	2.318434	−1.094222
H	2.438339	3.720283	−2.337218
H	0.767200	4.304745	−2.166971
H	1.741599	3.933133	−0.715696

B3LYP Energy = −1829.33284002 a.u.

(*aR*,1*R*,3*S*,3'*S*)-**21**, Conf H

C	2.888944	1.157937	0.850896
C	4.238323	0.829463	0.930165
C	4.711226	−0.277591	0.207437
C	3.842148	−1.013920	−0.593002
C	2.486612	−0.667214	−0.689581
C	2.010978	0.418924	0.053712
C	1.577349	−1.477250	−1.589937
C	2.362200	−2.323561	−2.588965
C	1.502518	−3.341534	−3.314006
O	3.391498	−3.039404	−1.894423
C	0.158558	1.848848	−0.830233
C	−1.180301	2.270833	−0.837925
C	−2.096154	1.647319	0.015283
C	−1.719871	0.561332	0.810833
C	−0.375020	0.175351	0.845969
C	0.566877	0.814526	0.022663
C	0.056520	−0.887118	1.832606
C	−0.949257	−1.012443	2.968806
C	−0.658091	−2.168285	3.907508
O	−2.247102	−1.237951	2.405159
C	−2.776343	−0.129215	1.670252
C	4.398136	−2.173029	−1.393057
C	−3.948876	−0.691147	0.885708
C	−5.249570	−0.518887	1.353328
C	−6.335073	−1.078694	0.685161

C	−6.089092	−1.820745	−0.454903
C	−4.811105	−2.022801	−0.947273
C	−3.742514	−1.452343	−0.265122
F	−7.140812	−2.372603	−1.118570
O	5.161245	1.520959	1.660593
C	4.730710	2.673844	2.380743
O	6.048459	−0.612357	0.200450
C	6.576561	−1.127869	1.433214
O	−1.538953	3.357176	−1.601421
C	−2.503969	3.116161	−2.642818
O	−3.401206	2.089026	0.065788
C	−3.590934	3.345766	0.739744
O	1.127372	2.474788	−1.567740
C	0.930993	2.651469	−2.977672
H	2.503702	2.007256	1.396797
H	0.948609	−2.144864	−0.989739
H	0.897247	−0.814699	−2.130107
H	2.840842	−1.660116	−3.324655
H	1.059475	−4.041841	−2.602600
H	0.695645	−2.843383	−3.855433
H	2.099273	−3.908237	−4.030115
H	1.041367	−0.639756	2.232197
H	0.152032	−1.862457	1.342983
H	−0.968524	−0.072919	3.540204
H	−0.677032	−3.115016	3.363610
H	−1.401932	−2.212926	4.704189
H	0.327834	−2.049800	4.361258
H	−3.170197	0.604957	2.386587
H	5.005845	−1.787426	−2.224875
H	5.052774	−2.786797	−0.775728
H	−5.424249	0.064724	2.249388
H	−7.349164	−0.946062	1.038504
H	−4.662985	−2.613518	−1.841781
H	−2.736680	−1.601451	−0.637313
H	4.321143	3.431868	1.708183
H	5.619351	3.066365	2.868976
H	3.985121	2.415824	3.137750
H	7.612583	−1.392995	1.231294
H	6.536010	−0.377597	2.222761
H	6.026997	−2.021416	1.743005
H	−2.645518	4.070012	−3.146631
H	−3.450518	2.768391	−2.230986
H	−2.121354	2.381134	−3.355515
H	−3.076720	4.154067	0.218054
H	−3.230218	3.286642	1.769999
H	−4.663165	3.530079	0.740812
H	1.924925	2.782962	−3.401816
H	0.467359	1.766355	−3.420244
H	0.322010	3.529394	−3.186333

B3LYP Energy = −1829.33275266 a.u.

(*aR*,1*R*,3*S*,3’*S*)-**21**, Conf I

C	2.851901	0.958069	0.934070
C	4.170141	0.545663	1.107022
C	4.625113	−0.584641	0.410312
C	3.753552	−1.302568	−0.404998
C	2.426005	−0.886815	−0.576238
C	1.978950	0.252263	0.101691
C	1.514003	−1.670991	−1.495359
C	2.295254	−2.599521	−2.420579
C	1.413606	−3.589215	−3.157952
O	3.239743	−3.351220	−1.646809
C	0.243819	1.636145	−1.045310
C	−1.059088	2.140511	−1.164588
C	−2.061145	1.649007	−0.320973
C	−1.758815	0.722667	0.686115
C	−0.436320	0.296595	0.854784
C	0.565650	0.730347	−0.030750
C	−0.097419	−0.595381	2.027940
C	−1.125283	−0.432780	3.138705
C	−0.921881	−1.392237	4.296227
O	−2.424389	−0.694528	2.595662
C	−2.877147	0.242191	1.611910
C	4.272348	−2.534268	−1.118184
C	−4.023932	−0.458807	0.903951
C	−3.780508	−1.535985	0.050096
C	−4.827978	−2.226723	−0.548494
C	−6.123762	−1.822488	−0.274790
C	−6.406856	−0.764930	0.569342
C	−5.342165	−0.090213	1.161199
F	−7.154499	−2.490540	−0.858582
O	5.073857	1.162741	1.923513
C	4.645720	2.291438	2.681026
O	5.908287	−1.059073	0.578082
C	6.956528	−0.246674	0.026727
O	−1.326824	3.053818	−2.157378
C	−1.735848	4.361377	−1.716589
O	−3.351068	2.119922	−0.414622
C	−4.061734	1.816398	−1.630781
O	1.208399	1.972665	−1.965245
C	1.764703	3.290181	−1.877463
H	2.482579	1.828621	1.457584
H	0.813215	−2.273633	−0.905701
H	0.911305	−0.985149	−2.094475
H	2.852090	−1.992796	−3.149946
H	0.889699	−4.234288	−2.449212
H	0.670510	−3.062498	−3.760015
H	2.011211	−4.217670	−3.819874
H	0.898333	−0.354344	2.402081
H	−0.073017	−1.648411	1.726048
H	−1.094554	0.601753	3.510871
H	−0.982978	−2.426239	3.950514
H	−1.684868	−1.238070	5.060475
H	0.058732	−1.237051	4.750295
H	−3.285234	1.122376	2.126217
H	4.958297	−2.233524	−1.924260
H	4.839032	−3.159188	−0.429074
H	−2.761757	−1.842470	−0.151075
H	−4.650110	−3.062426	−1.212504
H	−7.433805	−0.482508	0.760393
H	−5.546491	0.740016	1.826388

H	4.329994	3.112452	2.031547
H	5.510535	2.603733	3.261258
H	3.829960	2.028602	3.359764
H	7.881494	−0.798334	0.183152
H	6.802064	−0.093727	−1.045113
H	7.013633	0.717489	0.532254
H	−2.697640	4.327310	−1.204983
H	−1.814839	4.969690	−2.615142
H	−0.985854	4.792984	−1.048617
H	−5.064770	2.214855	−1.496735
H	−4.116055	0.736700	−1.777818
H	−3.586772	2.282628	−2.493591
H	2.576303	3.324579	−2.601653
H	1.026269	4.052086	−2.128381
H	2.165471	3.475019	−0.877469

B3LYP Energy = −1829.33265097 a.u.

(*aR*,1*R*,3*S*,3’*S*)-**21**, Conf J

C	2.870904	1.132156	0.888310
C	4.207488	0.781734	1.048790
C	4.683795	−0.383319	0.426748
C	3.816687	−1.191574	−0.303644
C	2.468831	−0.837766	−0.461923
C	1.999969	0.334403	0.141847
C	1.564135	−1.727444	−1.288221
C	2.355448	−2.682208	−2.177414
C	1.493532	−3.753622	−2.817979
O	3.352483	−3.341942	−1.387046
C	0.221279	1.718517	−0.940382
C	−1.102114	2.177276	−1.034071
C	−2.064446	1.665116	−0.158178
C	−1.748431	0.649553	0.748753
C	−0.418387	0.230967	0.868217
C	0.569403	0.760431	0.021034
C	−0.054073	−0.741982	1.967215
C	−1.097517	−0.723324	3.076351
C	−0.871123	−1.786838	4.134195
O	−2.384182	−0.967044	2.494291
C	−2.854581	0.072215	1.629183
C	4.364316	−2.451562	−0.942198
C	−4.008043	−0.541087	0.854227
C	−5.322171	−0.293580	1.244233
C	−6.393371	−0.891264	0.585869
C	−6.120031	−1.746476	−0.465249
C	−4.828364	−2.025749	−0.877751
C	−3.774338	−1.416263	−0.206774
F	−7.157521	−2.335207	−1.119457
O	5.111658	1.493579	1.782971
C	4.668528	2.677840	2.441804
O	5.988562	−0.798005	0.588754
C	6.988268	0.002671	−0.060903
O	−1.400306	3.194933	−1.909703
C	−2.339271	2.882968	−2.956105
O	−3.355647	2.147259	−0.195660
C	−3.525722	3.467598	0.349796
O	1.231517	2.242767	−1.702616
C	1.088592	2.266609	−3.129807
H	2.486213	2.032189	1.346111
H	0.918605	−2.323016	−0.632778
H	0.900535	−1.118125	−1.906101
H	2.863847	−2.104108	−2.963697
H	1.019222	−4.369503	−2.050817
H	0.710288	−3.298606	−3.427617
H	2.095691	−4.401050	−3.456975
H	0.926569	−0.489258	2.373591
H	0.020859	−1.763261	1.578187
H	−1.103973	0.269019	3.550344
H	−0.899589	−2.782483	3.686702
H	−1.641480	−1.732045	4.904564
H	0.102579	−1.648953	4.608274
H	−3.258722	0.883908	2.249428
H	5.019589	−2.184172	−1.784751
H	4.970022	−3.005866	−0.226443
H	−5.518634	0.379880	2.070009
H	−7.417445	−0.701009	0.879026
H	−4.658660	−2.703586	−1.704034
H	−2.758258	−1.624063	−0.517848
H	4.307708	3.421270	1.726127
H	5.538949	3.069848	2.962212
H	3.881389	2.457478	3.167749
H	7.938044	−0.500185	0.110849
H	6.797148	0.059567	−1.136296
H	7.022639	1.007248	0.361042
H	−3.310385	2.608666	−2.546060
H	−1.960333	2.068557	−3.578810
H	−2.430330	3.786038	−3.555999
H	−3.215412	3.492270	1.397712
H	−4.588559	3.689641	0.280880
H	−2.956273	4.204613	−0.217982
H	0.632625	1.341376	−3.491125
H	0.496945	3.120521	−3.455424
H	2.098454	2.345719	−3.528707

B3LYP Energy = −1829.33257105 a.u.

(*aR*,1*R*,3*S*,3’*S*)-**21**, Conf K

C	2.884034	1.159287	0.718066
C	4.226083	0.805469	0.802618
C	4.654598	−0.383722	0.190729
C	3.748481	−1.179118	−0.504956
C	2.397542	−0.812699	−0.602700
C	1.967443	0.362187	0.025927
C	1.448143	−1.701878	−1.379125
C	2.187444	−2.653185	−2.316005
C	1.289550	−3.721292	−2.910651
O	3.226804	−3.316796	−1.585341
C	0.156448	1.823856	−0.889533
C	−1.161037	2.308118	−0.908249
C	−2.120068	1.706246	−0.086837
C	−1.766763	0.631356	0.743591
C	−0.433975	0.217951	0.817400

C	0.536959	0.801536	−0.017787
C	−0.040523	−0.798003	1.866511
C	−1.059634	−0.825168	2.997742
C	−0.801306	−1.920100	4.015822
O	−2.354128	−1.064967	2.434293
C	−2.853260	−0.012317	1.600286
C	4.260782	−2.425845	−1.196040
C	−3.988608	−0.637937	0.807697
C	−3.735460	−1.431910	−0.310822
C	−4.771913	−2.057990	−0.994117
C	−6.065405	−1.877771	−0.535639
C	−6.357671	−1.103586	0.571844
C	−5.304001	−0.487602	1.241122
F	−7.086215	−2.483693	−1.200720
O	5.183588	1.545230	1.434034
C	4.799672	2.776297	2.042388
O	5.983312	−0.749425	0.188173
C	6.527587	−1.155922	1.454261
O	−1.547156	3.294767	−1.784797
C	−0.966215	4.597046	−1.584391
O	−3.438607	2.075707	−0.091573
C	−3.778451	3.456824	0.095791
O	1.098978	2.407670	−1.708927
C	1.029460	2.029907	−3.093659
H	2.532756	2.071549	1.178243
H	0.844875	−2.302004	−0.689044
H	0.745240	−1.094491	−1.953495
H	2.651901	−2.072641	−3.127069
H	0.855607	−4.337086	−2.119841
H	0.475926	−3.263914	−3.477185
H	1.855380	−4.369013	−3.581748
H	0.948480	−0.554914	2.259186
H	0.028991	−1.804137	1.439504
H	−1.064988	0.151350	3.503636
H	−0.829153	−2.900809	3.536406
H	−1.556805	−1.898773	4.802387
H	0.180073	−1.787477	4.475493
H	−3.282230	0.768437	2.242734
H	4.855174	−2.137751	−2.075597
H	4.918940	−2.988659	−0.535400
H	−2.718367	−1.562177	−0.658665
H	−4.587193	−2.672759	−1.865282
H	−7.382749	−0.988407	0.898688
H	−5.515103	0.123319	2.110794
H	4.389657	3.472381	1.306113
H	5.710339	3.193402	2.465248
H	4.069454	2.616762	2.840515
H	7.552174	−1.465718	1.257932
H	6.522823	−0.331534	2.167190
H	5.964499	−2.000685	1.861238
H	0.115887	4.567531	−1.702715
H	−1.217587	4.979406	−0.591724
H	−1.406500	5.241018	−2.342805
H	−3.657619	4.023393	−0.825476
H	−3.170950	3.901413	0.888396
H	−4.824175	3.465500	0.397764
H	1.236369	0.963681	−3.211283
H	0.052190	2.266272	−3.518027
H	1.800672	2.603053	−3.604232

B3LYP Energy = −1829.33254835 a.u.

(*aR*,1*R*,3*S*,3’*S*)-**21**, Conf L

C	2.871873	1.029765	0.959953
C	4.207696	0.655138	1.005885
C	4.643763	−0.423653	0.230487
C	3.745759	−1.086778	−0.609125
C	2.404068	−0.690643	−0.670769
C	1.960706	0.368813	0.137978
C	1.462479	−1.423176	−1.603901
C	2.209352	−2.242108	−2.653703
C	1.307978	−3.190993	−3.420475
O	3.218848	−3.027714	−2.007406
C	0.160626	1.949468	−0.579922
C	−1.155886	2.427360	−0.532651
C	−2.125262	1.711600	0.172674
C	−1.777852	0.546493	0.871973
C	−0.441982	0.130408	0.897212
C	0.530979	0.811464	0.141600
C	−0.047627	−1.018767	1.799180
C	−1.070948	−1.209430	2.909805
C	−0.816222	−2.438324	3.762154
O	−2.363380	−1.364727	2.311674
C	−2.859689	−0.190849	1.659787
C	4.256767	−2.224064	−1.468014
C	−4.031013	−0.666679	0.819283
C	−5.338939	−0.420481	1.230476
C	−6.424714	−0.904074	0.505219
C	−6.172771	−1.647635	−0.632728
C	−4.888129	−1.922785	−1.069809
C	−3.819168	−1.426102	−0.332432
F	−7.224332	−2.126773	−1.350043
O	5.066693	1.328927	1.849287
C	5.994039	2.207865	1.190871
O	5.965196	−0.817693	0.217300
C	6.451117	−1.411275	1.434722
O	−1.495552	3.593491	−1.182112
C	−1.173457	4.791994	−0.454067
O	−3.405951	2.188939	0.300428
C	−4.185430	2.327400	−0.901886
O	1.122845	2.669377	−1.249572
C	1.019800	2.675972	−2.682767
H	2.551042	1.862113	1.573880
H	0.818900	−2.099570	−1.030549
H	0.797619	−0.713023	−2.100722
H	2.704049	−1.558059	−3.359253
H	0.851137	−3.914017	−2.741274
H	0.511542	−2.638608	−3.923006
H	1.877161	−3.737075	−4.173945
H	0.937332	−0.826440	2.228216
H	0.031983	−1.954609	1.235383
H	−1.080174	−0.315594	3.550141

H	−0.846665	−3.341459	3.149004
H	−1.571910	−2.526402	4.543944
H	0.165261	−2.374752	4.236028
H	−3.247991	0.496441	2.423136
H	4.876686	−1.820160	−2.282118
H	4.890149	−2.890484	−0.883950
H	−5.518115	0.162673	2.125660
H	−7.443818	−0.714166	0.815412
H	−4.735278	−2.512629	−1.964079
H	−2.808270	−1.635182	−0.659152
H	6.647470	1.655557	0.513134
H	6.587282	2.670631	1.977064
H	5.457674	2.982237	0.635941
H	6.406793	−0.703295	2.262568
H	5.870739	−2.304006	1.682632
H	7.484387	−1.692921	1.242590
H	−1.509232	5.623447	−1.070611
H	−0.096855	4.867243	−0.290492
H	−1.698378	4.811580	0.503848
H	−4.265128	1.366196	−1.413765
H	−3.750264	3.071256	−1.566823
H	−5.173928	2.646679	−0.579015
H	1.130390	1.663440	−3.079559
H	0.069397	3.100459	−3.007093
H	1.840934	3.293123	−3.041610

B3LYP Energy = −1829.33234045 a.u.

(*aR*,1*R*,3*S*,3’*S*)-**21**, Conf M

C	2.869047	1.094608	0.820014
C	4.196376	0.709305	0.973811
C	4.621750	−0.502582	0.406561
C	3.713909	−1.320742	−0.260250
C	2.373697	−0.933856	−0.408899
C	1.956041	0.286511	0.136313
C	1.425622	−1.843349	−1.162097
C	2.169054	−2.859700	−2.024160
C	1.262679	−3.935874	−2.590818
O	3.166775	−3.508639	−1.225792
C	0.216853	1.743310	−0.915301
C	−1.085257	2.260413	−1.002960
C	−2.088141	1.723137	−0.189113
C	−1.792079	0.684790	0.707202
C	−0.473557	0.244253	0.850207
C	0.540119	0.758219	0.020273
C	−0.143988	−0.723143	1.964768
C	−1.201919	−0.663269	3.058622
C	−1.005838	−1.702995	4.145778
O	−2.481122	−0.906006	2.461748
C	−2.924898	0.108309	1.552421
C	4.211417	−2.627256	−0.844011
C	−4.037422	−0.538573	0.744797
C	−5.368302	−0.324237	1.095954
C	−6.403977	−0.954206	0.411576
C	−6.078159	−1.806896	−0.626674
C	−4.768559	−2.052156	−1.001883
C	−3.750633	−1.410964	−0.305007
F	−7.080850	−2.426875	−1.306177
O	5.138423	1.428058	1.651081
C	4.748001	2.657742	2.257855
O	5.915934	−0.949674	0.562919
C	6.925812	−0.217135	−0.148781
O	−1.414317	3.216144	−1.935568
C	−0.801225	4.508843	−1.772838
O	−3.396173	2.123312	−0.265113
C	−3.705421	3.517543	−0.127443
O	1.203049	2.256639	−1.730756
C	1.150898	1.830665	−3.102325
H	2.522576	2.029436	1.236456
H	0.790340	−2.393217	−0.458926
H	0.753203	−1.252915	−1.788402
H	2.673244	−2.334281	−2.849175
H	0.789454	−4.498244	−1.782978
H	0.478628	−3.489274	−3.205560
H	1.831652	−4.631685	−3.208985
H	0.837498	−0.485454	2.378945
H	−0.088894	−1.752435	1.594977
H	−1.201471	0.340539	3.507848
H	−1.039292	−2.709251	3.723084
H	−1.788144	−1.619842	4.901499
H	−0.038583	−1.564632	4.632770
H	−3.364678	0.930418	2.132745
H	4.857713	−2.421060	−1.710358
H	4.815105	−3.162945	−0.112560
H	−5.605497	0.348490	1.911617
H	−7.440737	−0.790009	0.674388
H	−4.557435	−2.727331	−1.820737
H	−2.721175	−1.591083	−0.588156
H	4.396342	3.376473	1.512911
H	5.641627	3.045895	2.740445
H	3.969923	2.500565	3.009584
H	6.709719	−0.206071	−1.220862
H	7.005134	0.804935	0.222035
H	7.861714	−0.744491	0.025373
H	−1.201449	5.132454	−2.569494
H	0.282462	4.444007	−1.858909
H	−1.068102	4.939459	−0.804274
H	−3.510057	4.060369	−1.050255
H	−3.135718	3.961306	0.693247
H	−4.767331	3.563660	0.106934
H	0.200292	2.102717	−3.563960
H	1.966319	2.341498	−3.610450
H	1.301319	0.750908	−3.174957

B3LYP Energy = −1829.33231506 a.u.

(*aR*,1*R*,3*S*,3’*S*)-**21**, Conf N

C	2.816990	0.917148	1.077046
C	4.154170	0.544810	1.141269
C	4.633336	−0.439255	0.271540
C	3.780755	−1.002765	−0.681543
C	2.441913	−0.604560	−0.761134

C	1.951796	0.351365	0.142757
C	1.546062	−1.220688	−1.813943
C	2.340834	−1.930015	−2.907450
C	1.475303	−2.793541	−3.804881
O	3.326390	−2.779106	−2.304167
C	0.134931	1.871694	−0.652802
C	−1.193225	2.313553	−0.657395
C	−2.140470	1.655512	0.129492
C	−1.791618	0.513578	0.860643
C	−0.455134	0.096121	0.884219
C	0.515942	0.775881	0.126203
C	−0.062969	−1.051183	1.788863
C	−1.081131	−1.231333	2.906249
C	−0.827122	−2.457185	3.763299
O	−2.378261	−1.384477	2.318082
C	−2.874523	−0.219008	1.650640
C	4.335773	−2.035685	−1.638923
C	−4.040531	−0.709477	0.810216
C	−5.346634	−0.548435	1.266457
C	−6.427573	−1.049927	0.546568
C	−6.171539	−1.722754	−0.633544
C	−4.887977	−1.911914	−1.116341
C	−3.823996	−1.400665	−0.382077
F	−7.218780	−2.217110	−1.347861
O	4.964691	1.121994	2.096851
C	5.944381	2.042136	1.587427
O	5.955272	−0.832533	0.281475
C	6.378643	−1.562158	1.447172
O	−1.549923	3.411251	−1.407500
C	−2.103598	3.094256	−2.696655
O	−3.448988	2.078735	0.134873
C	−3.691426	3.342341	0.777522
O	1.059168	2.467494	−1.478487
C	1.485750	3.785697	−1.097647
H	2.459968	1.666544	1.772839
H	0.865869	−1.942561	−1.347732
H	0.919576	−0.448345	−2.265503
H	2.861833	−1.176830	−3.516767
H	0.989691	−3.579756	−3.222659
H	0.701127	−2.189255	−4.281686
H	2.076981	−3.261943	−4.584911
H	0.925928	−0.868365	2.211799
H	0.004541	−1.987457	1.223481
H	−1.081638	−0.334111	3.542171
H	−0.864192	−3.362884	3.154387
H	−1.579409	−2.538775	4.549067
H	0.156886	−2.395289	4.232115
H	−3.270251	0.473355	2.406473
H	4.983464	−1.541352	−2.378039
H	4.951620	−2.759336	−1.106562
H	−5.529142	−0.019766	2.194487
H	−7.445822	−0.925372	0.890798
H	−4.731995	−2.447836	−2.043431
H	−2.814018	−1.542016	−0.746052
H	6.630518	1.546927	0.898411
H	6.493545	2.412139	2.450929
H	5.455108	2.878959	1.081606
H	5.785139	−2.472691	1.564533
H	7.419844	−1.827747	1.276337
H	6.293899	−0.951110	2.346191
H	−2.339548	4.045018	−3.170533
H	−3.013984	2.500859	−2.591171
H	−1.374128	2.554318	−3.304651
H	−3.175392	4.151295	0.260492
H	−3.372444	3.307315	1.822808
H	−4.766815	3.501315	0.732690
H	2.198110	4.103922	−1.856191
H	0.643137	4.476923	−1.069735
H	1.981556	3.759780	−0.123745

B3LYP Energy = −1829.33230346 a.u.

(*aR*,1*R*,3*S*,3’*S*)-**21**, Conf O

C	2.804926	0.914825	1.100024
C	4.143064	0.548848	1.179284
C	4.647243	−0.398837	0.284371
C	3.795450	−1.008790	−0.640161
C	2.448157	−0.639272	−0.715447
C	1.950650	0.336541	0.163272
C	1.558384	−1.291496	−1.751581
C	2.361460	−2.000104	−2.839441
C	1.509167	−2.892132	−3.721531
O	3.364519	−2.822429	−2.228298
C	0.150299	1.848698	−0.676127
C	−1.174946	2.297941	−0.703575
C	−2.135034	1.658046	0.082810
C	−1.801402	0.525984	0.835947
C	−0.467846	0.100520	0.881291
C	0.515822	0.763111	0.124526
C	−0.093780	−1.035023	1.808275
C	−1.124871	−1.189700	2.917543
C	−0.888705	−2.403590	3.796548
O	−2.416888	−1.343216	2.318628
C	−2.897664	−0.185567	1.626995
C	4.363644	−2.054340	−1.576680
C	−4.058949	−0.680332	0.782781
C	−5.369366	−0.482428	1.211137
C	−6.447061	−0.984605	0.486910
C	−6.183589	−1.695486	−0.668984
C	−4.895696	−1.921911	−1.123488
C	−3.835019	−1.409364	−0.385416
F	−7.227499	−2.191013	−1.387320
O	4.954959	1.169192	2.105946
C	5.365241	0.340258	3.206171
O	5.965327	−0.802744	0.330138
C	6.928579	0.176638	−0.096125
O	−1.516099	3.385496	−1.475183
C	−2.062446	3.050972	−2.763020
O	−3.440868	2.089099	0.066685
C	−3.682568	3.365096	0.684751
O	1.087462	2.426116	−1.500105
C	1.522763	3.744972	−1.130382

H	2.435473	1.660559	1.793226
H	0.898806	−2.021935	−1.269308
H	0.911065	−0.541007	−2.210352
H	2.865694	−1.245437	−3.460928
H	1.039534	−3.678288	−3.126334
H	0.722961	−2.309344	−4.205410
H	2.117350	−3.361233	−4.496103
H	0.892146	−0.852804	2.238289
H	−0.027336	−1.980937	1.259116
H	−1.125569	−0.282418	3.539114
H	−0.925492	−3.318413	3.201444
H	−1.649963	−2.467653	4.575233
H	0.090625	−2.341342	4.275062
H	−3.295420	0.522197	2.367360
H	5.008079	−1.570198	−2.325715
H	4.984602	−2.758529	−1.024323
H	−5.557789	0.075862	2.120424
H	−7.468495	−0.831911	0.809713
H	−4.733997	−2.487056	−2.032059
H	−2.821748	−1.579546	−0.727263
H	4.494561	0.003491	3.775628
H	5.993059	0.963031	3.840313
H	5.933876	−0.523750	2.858587
H	6.734486	0.481078	−1.127929
H	6.912907	1.052013	0.554068
H	7.900536	−0.309095	−0.038915
H	−2.287253	3.995353	−3.254725
H	−2.978206	2.466444	−2.654603
H	−1.332557	2.495179	−3.356041
H	−3.151534	4.161147	0.162939
H	−3.380550	3.344505	1.735464
H	−4.755685	3.532388	0.620140
H	2.242189	4.049340	−1.887917
H	0.685507	4.443136	−1.115294
H	2.012887	3.725343	−0.153577

B3LYP Energy = −1829.33217882 a.u.

(*aS*,1*S*,3*S*,1'*S*,3'*S*)-**22**, Conf A

C	−0.493914	1.895754	−0.684918
C	−1.774998	2.457299	−0.761444
C	−2.878422	1.623850	−0.952479
C	−2.714750	0.235462	−1.013566
C	−1.434328	−0.320327	−0.929838
C	−0.310375	0.513776	−0.778098
O	−3.589840	−1.944133	−1.675384
C	−2.573024	−2.582427	−0.888354
C	−1.262091	−1.820690	−1.047342
C	−3.941934	−0.641325	−1.190034
C	−2.480240	−4.024860	−1.349421
C	−4.834232	−0.756543	0.045665
C	1.075227	−0.052526	−0.738068
C	1.771117	−0.211941	0.466276
C	3.056565	−0.768485	0.436936
C	3.626006	−1.182959	−0.764100
C	2.941711	−0.995257	−1.973987
C	1.667246	−0.438181	−1.943761
C	−6.131958	−1.248754	−0.123213
C	−6.985886	−1.419540	0.958918
C	−6.519954	−1.089165	2.220610
C	−5.246025	−0.598632	2.432721
C	−4.407105	−0.433939	1.332408
C	1.163191	0.184906	1.791005
C	1.846135	−0.519465	2.955973
O	3.266291	−0.328459	2.846535
C	3.841051	−0.982980	1.722161
C	1.426535	0.011881	4.312756
F	−7.348832	−1.248799	3.286486
O	4.905757	−1.688314	−0.734466
C	5.050506	−3.077847	−1.074517
O	3.596509	−1.374553	−3.108958
C	2.954142	−1.167192	−4.365189
C	5.289484	−0.472617	1.691693
O	6.214068	−1.084217	2.173775
O	5.386045	0.742685	1.150262
C	6.570453	2.718041	0.497204
C	6.705642	1.350478	1.126743
O	0.585572	2.712098	−0.440368
C	1.047968	3.480585	−1.562880
O	−1.941540	3.821245	−0.686190
C	−2.018456	4.339082	0.653570
C	−4.457955	2.963264	−2.142433
O	−4.153368	2.134777	−1.005787
H	−2.879825	−2.562119	0.164801
H	−0.540898	−2.174285	−0.306566
H	−0.834657	−2.058769	−2.027210
H	−4.551125	−0.217905	−1.989484
H	−1.701406	−4.551603	−0.794233
H	−3.427469	−4.541174	−1.188321
H	−2.237066	−4.071283	−2.413235
H	1.118021	−0.291550	−2.862896
H	−6.480579	−1.505382	−1.116582
H	−7.993053	−1.795083	0.834040
H	−4.921263	−0.347991	3.434054
H	−3.411416	−0.039355	1.487334
H	1.251490	1.267547	1.930231
H	0.095993	−0.048396	1.797539
H	1.637952	−1.597829	2.903598
H	3.915990	−2.058514	1.926312
H	1.941310	−0.523613	5.111405
H	0.350685	−0.112684	4.450612
H	1.664896	1.074119	4.396740
H	4.792697	−3.251419	−2.119066
H	6.097052	−3.321684	−0.905844
H	4.423750	−3.698336	−0.428459
H	3.653391	−1.524477	−5.117032
H	2.023294	−1.736068	−4.436437
H	2.749118	−0.107071	−4.535491
H	7.549264	3.200694	0.465748
H	6.191904	2.644040	−0.523161
H	5.895319	3.350518	1.075381

H	7.078430	1.407022	2.150255
H	7.371495	0.702130	0.555750
H	1.408198	2.819221	−2.354964
H	0.257183	4.127477	−1.945452
H	1.873809	4.086865	−1.196551
H	−2.867551	3.903966	1.185801
H	−1.093561	4.138880	1.197792
H	−2.160797	5.413664	0.558158
H	−5.510465	3.222694	−2.049922
H	−3.850102	3.867191	−2.143028
H	−4.300464	2.408375	−3.071341

B3LYP Energy = −2096.62250751 a.u.

(*aS*,1*S*,3*S*,1'*S*,3'*S*)-**22**, Conf B

C	0.782920	−1.503033	1.512144
C	2.105078	−1.670669	1.943037
C	3.152262	−1.405251	1.059193
C	2.888647	−0.931754	−0.230652
C	1.566862	−0.765011	−0.655676
C	0.502331	−1.062298	0.215953
O	3.646992	−0.588908	−2.524265
C	2.522892	0.272708	−2.759474
C	1.290799	−0.305750	−2.072430
C	4.055606	−0.619031	−1.150096
C	2.356357	0.401354	−4.261973
C	4.829897	0.650765	−0.795521
C	−0.923307	−0.926227	−0.221127
C	−1.687664	0.197541	0.112855
C	−3.019523	0.263312	−0.314418
C	−3.572646	−0.749528	−1.097804
C	−2.792728	−1.858501	−1.462577
C	−1.483436	−1.941511	−0.998624
C	6.095015	0.829782	−1.363135
C	6.840055	1.976654	−1.119961
C	6.297840	2.948435	−0.295562
C	5.052840	2.811953	0.287392
C	4.323707	1.652709	0.030517
C	−1.104154	1.341159	0.909006
C	−1.893203	2.626595	0.700779
O	−3.284178	2.361951	0.945104
C	−3.871987	1.481763	−0.007221
C	−1.480520	3.747650	1.634676
F	7.019927	4.073532	−0.047519
O	−4.853746	−0.559072	−1.559846
C	−5.832745	−1.577565	−1.275214
O	−3.377839	−2.781912	−2.280781
C	−2.616537	−3.919594	−2.679943
C	−5.226636	1.131928	0.623734
O	−5.401201	0.271944	1.453089
O	−6.179029	1.965413	0.188543
C	−8.397164	2.866685	0.149885
C	−7.499562	1.827233	0.781400
O	−0.246930	−1.700153	2.401626
C	−0.566488	−3.071300	2.688582
O	2.371952	−2.125183	3.214294
C	2.378931	−1.101250	4.224554
C	4.918180	−2.864956	1.727073
O	4.462434	−1.526819	1.458548
H	2.744087	1.260775	−2.336939
H	0.487442	0.434624	−2.078309
H	0.926233	−1.154745	−2.660548
H	4.760668	−1.449836	−1.102809
H	1.499198	1.035991	−4.495999
H	3.246357	0.844387	−4.710708
H	2.192151	−0.579819	−4.712945
H	−0.871691	−2.793442	−1.259255
H	6.504088	0.061384	−2.008408
H	7.821785	2.118624	−1.552426
H	4.666526	3.592403	0.929751
H	3.352444	1.532587	0.492318
H	−1.103568	1.093529	1.975791
H	−0.062592	1.502533	0.621780
H	−1.782385	2.957159	−0.341963
H	−4.066384	2.028853	−0.937989
H	−2.069010	4.645457	1.441762
H	−0.425495	3.988950	1.491318
H	−1.629325	3.451620	2.675053
H	−6.790653	−1.151568	−1.566858
H	−5.635758	−2.480482	−1.848655
H	−5.843338	−1.803365	−0.207383
H	−3.273558	−4.504649	−3.318713
H	−1.728878	−3.625655	−3.246184
H	−2.317819	−4.522261	−1.818174
H	−9.398191	2.790164	0.578765
H	−8.021635	3.873848	0.335943
H	−8.474426	2.716476	−0.927935
H	−7.856962	0.813354	0.597624
H	−7.409115	1.966019	1.859474
H	0.291950	−3.590451	3.117205
H	−1.381077	−3.048566	3.409429
H	−0.899568	−3.583228	1.782144
H	3.142269	−0.350982	4.005471
H	1.398632	−0.626914	4.299639
H	2.616792	−1.599048	5.162484
H	4.368470	−3.306880	2.557397
H	4.812074	−3.489515	0.835767
H	5.971738	−2.777179	1.984048

B3LYP Energy = −2096.62241488 a.u.

(*aS*,1*S*,3*S*,1'*S*,3'*S*)-**22**, Conf C

C	0.785254	−1.514567	1.503020
C	2.110413	−1.694617	1.919478
C	3.151074	−1.418263	1.031330
C	2.878050	−0.920918	−0.247598
C	1.553496	−0.740051	−0.657804
C	0.495108	−1.048776	0.217489
O	3.617824	−0.543220	−2.541719
C	2.497914	0.330032	−2.751067
C	1.267960	−0.252431	−2.063370
C	4.038803	−0.600018	−1.171939

C	2.318621	0.486756	−4.249497
C	4.825261	0.658022	−0.803089
C	−0.933422	−0.903950	−0.207218
C	−1.709313	0.191722	0.189581
C	−3.036229	0.275166	−0.249375
C	−3.572082	−0.693777	−1.093636
C	−2.805138	−1.807564	−1.465907
C	−1.488772	−1.893068	−1.023030
C	6.087400	0.836723	−1.377352
C	6.842502	1.974412	−1.122329
C	6.313384	2.937253	−0.279165
C	5.071877	2.800535	0.311199
C	4.332505	1.650560	0.042236
C	−1.146307	1.295310	1.052805
C	−1.953664	2.579059	0.911570
O	−3.341482	2.284016	1.140464
C	−3.921367	1.442691	0.150452
C	−1.557803	3.653894	1.905254
F	7.045225	4.053372	−0.019566
O	−4.891292	−0.566291	−1.465794
C	−5.142479	−0.357390	−2.865991
O	−3.424843	−2.743379	−2.241278
C	−2.703050	−3.922180	−2.592249
C	−5.242142	0.985153	0.789364
O	−5.337943	0.105937	1.609526
O	−6.261853	1.744738	0.369432
C	−8.549773	2.452595	0.355043
C	−7.567241	1.467488	0.946198
O	−0.236793	−1.725964	2.397694
C	−0.564676	−3.101331	2.654072
O	2.386181	−2.173154	3.179806
C	2.399587	−1.168848	4.209540
C	4.915631	−2.896618	1.662520
O	4.464274	−1.552678	1.415759
H	2.729706	1.308792	−2.312886
H	0.470264	0.494049	−2.047735
H	0.891735	−1.087216	−2.664258
H	4.738257	−1.436464	−1.145539
H	1.463961	1.131559	−4.464267
H	3.207568	0.931563	−4.698523
H	2.143482	−0.485069	−4.716318
H	−0.877517	−2.739295	−1.302888
H	6.486176	0.075313	−2.037209
H	7.822011	2.115955	−1.559933
H	4.696179	3.573667	0.968539
H	3.364028	1.530106	0.509806
H	−1.149984	0.986992	2.103445
H	−0.105371	1.487389	0.782651
H	−1.845799	2.966389	−0.111801
H	−4.164143	2.038197	−0.738084
H	−2.158457	4.552664	1.759914
H	−0.505973	3.916983	1.777664
H	−1.704381	3.299630	2.927584
H	−4.857581	−1.232896	−3.448628
H	−6.212826	−0.185698	−2.957618
H	−4.600641	0.521262	−3.226329
H	−3.391095	−4.525991	−3.178679
H	−1.822382	−3.687378	−3.196056
H	−2.398753	−4.479939	−1.702855
H	−9.542264	2.268844	0.770998
H	−8.265469	3.479314	0.590215
H	−8.609026	2.346240	−0.729180
H	−7.833135	0.435364	0.713972
H	−7.493720	1.564632	2.030006
H	−0.909337	−3.588478	1.738480
H	0.293479	−3.636832	3.062838
H	−1.373173	−3.089793	3.381892
H	3.160844	−0.413857	3.999454
H	1.419528	−0.696972	4.300759
H	2.644549	−1.684433	5.135947
H	5.973456	−2.817931	1.904421
H	4.375739	−3.344665	2.495936
H	4.792178	−3.510414	0.766029

B3LYP Energy = −2096.62235658 a.u.

(*aS*,1*S*,3*S*,1’*S*,3’*S*)-**22**, Conf D

C	−0.817273	−1.928199	−1.140982
C	−2.157899	−2.196368	−1.455140
C	−3.171688	−1.631514	−0.679420
C	−2.856037	−0.863489	0.448413
C	−1.518840	−0.647105	0.790377
C	−0.488659	−1.165178	−0.016587
O	−3.529891	0.004234	2.630862
C	−2.379207	0.860920	2.635418
C	−1.184769	0.108940	2.058833
C	−3.987237	−0.337372	1.314421
C	−2.158667	1.313777	4.066741
C	−4.778857	0.818551	0.706676
C	0.951906	−0.960223	0.338595
C	1.669911	0.159990	−0.095997
C	3.017912	0.283560	0.266593
C	3.632528	−0.668665	1.078691
C	2.899115	−1.772176	1.543525
C	1.573284	−1.912524	1.147901
C	−6.086083	1.033687	1.152942
C	−6.843639	2.099998	0.684896
C	−6.270097	2.955446	−0.240837
C	−4.982909	2.779549	−0.711643
C	−4.242977	1.700938	−0.231390
C	1.026278	1.246169	−0.927975
C	1.780458	2.563089	−0.801145
O	3.166192	2.329230	−1.097883
C	3.823652	1.504470	−0.140999
C	1.293549	3.635292	−1.756496
F	−7.002881	4.000857	−0.707526
O	4.928311	−0.422219	1.467987
C	5.918251	−1.433058	1.193047
O	3.544507	−2.630735	2.385610
C	2.834251	−3.765075	2.879309
C	5.151017	1.153176	−0.826984
O	5.301347	0.256845	−1.622018

O	6.105638	2.025974	−0.483928
C	8.297242	2.981063	−0.617443
C	7.397418	1.882768	−1.136072
O	0.192972	−2.509608	−1.868095
C	0.329659	−2.087326	−3.235750
O	−2.478091	−3.008573	−2.520672
C	−2.415161	−4.416324	−2.229246
C	−5.038837	−1.486065	−2.182925
O	−4.495464	−1.909641	−0.919423
H	−2.590514	1.738714	2.011913
H	−0.366471	0.809695	1.879786
H	−0.817627	−0.601779	2.807724
H	−4.689440	−1.153801	1.484637
H	−1.276898	1.954557	4.130992
H	−3.020678	1.875573	4.428882
H	−2.007173	0.451804	4.720086
H	0.995961	−2.761309	1.485141
H	−6.519810	0.356668	1.879396
H	−7.857903	2.267688	1.022668
H	−4.573362	3.467686	−1.439491
H	−3.238803	1.547242	−0.604829
H	1.008920	0.959995	−1.985340
H	−0.013411	1.385891	−0.625229
H	1.703638	2.928823	0.232702
H	4.054292	2.097158	0.752711
H	1.862859	4.555949	−1.623029
H	0.239108	3.852306	−1.575226
H	1.406278	3.304496	−2.790963
H	5.884571	−1.718372	0.140010
H	6.877899	−0.970599	1.415176
H	5.770512	−2.306743	1.823731
H	3.536648	−4.298407	3.515056
H	1.966506	−3.463596	3.471795
H	2.512329	−4.419060	2.064893
H	9.277997	2.899980	−1.090046
H	7.887026	3.965276	−0.847863
H	8.430437	2.902461	0.462509
H	7.790837	0.891249	−0.908831
H	7.248523	1.950929	−2.214389
H	0.503144	−1.009944	−3.287648
H	1.201875	−2.608820	−3.623764
H	−0.552153	−2.348782	−3.820354
H	−2.705602	−4.931105	−3.142924
H	−1.401129	−4.708391	−1.949540
H	−3.111359	−4.672831	−1.427412
H	−4.912469	−0.408422	−2.308336
H	−4.568207	−2.016402	−3.009181
H	−6.100167	−1.721867	−2.143274

B3LYP Energy = −2096.62168880 a.u.

(*aS*,1*S*,3*S*,1’*S*,3’*S*)-**22**, Conf E

C	0.521554	2.069497	−0.602576
C	1.824350	2.532567	−0.839657
C	2.902721	1.895939	−0.223608
C	2.682833	0.848349	0.679919
C	1.379627	0.429225	0.957302
C	0.288125	1.028266	0.300352
O	3.489697	−0.476617	2.568670
C	2.416359	−1.405502	2.362098
C	1.146973	−0.640774	2.002921
C	3.878261	0.229317	1.381696
C	2.276763	−2.223131	3.632560
C	4.750208	−0.654144	0.492472
C	−1.118977	0.606870	0.593530
C	−1.722209	−0.467364	−0.071994
C	−3.037971	−0.817667	0.262214
C	−3.727982	−0.128096	1.255240
C	−3.132381	0.964529	1.902884
C	−1.828046	1.312486	1.568326
C	4.263144	−1.319083	−0.633079
C	5.081009	−2.162167	−1.382497
C	6.395595	−2.323700	−0.988353
C	6.921606	−1.679410	0.118793
C	6.087033	−0.846178	0.853303
C	−0.985748	−1.272001	−1.119467
C	−1.611061	−2.648034	−1.310311
O	−3.019646	−2.487428	−1.541511
C	−3.724976	−1.997414	−0.407302
C	−1.046180	−3.410287	−2.493068
F	7.203719	−3.138585	−1.716594
O	−5.031351	−0.489756	1.509610
C	−5.294041	−1.070835	2.798236
O	−3.897774	1.617410	2.823131
C	−3.348977	2.763494	3.471648
C	−5.138238	−1.713646	−0.937354
O	−6.053414	−2.495503	−0.826114
O	−5.208010	−0.554275	−1.592989
C	−6.333270	1.136200	−2.860831
C	−6.489173	−0.206517	−2.184300
O	−0.548829	2.715785	−1.173430
C	−0.689149	2.584338	−2.597995
O	2.041453	3.603161	−1.678373
C	1.858158	4.888113	−1.057110
C	4.745201	2.312035	−1.710421
O	4.190155	2.347435	−0.382880
H	2.683179	−2.074770	1.534469
H	0.382740	−1.344077	1.665519
H	0.748404	−0.169964	2.908757
H	4.509175	1.037337	1.752394
H	1.458649	−2.939875	3.535984
H	3.195727	−2.774231	3.836215
H	2.067082	−1.572321	4.484158
H	−1.346490	2.147308	2.057023
H	3.236162	−1.174740	−0.943052
H	4.710086	−2.680034	−2.257275
H	7.958718	−1.826867	0.390077
H	6.483319	−0.335198	1.722855
H	−1.000375	−0.749170	−2.081924
H	0.064559	−1.381972	−0.841553
H	−1.476134	−3.239540	−0.393479
H	−3.845530	−2.807541	0.322923

H	−1.523893	−4.386385	−2.584809
H	0.027574	−3.561722	−2.366860
H	−1.210766	−2.855032	−3.418764
H	−6.339965	−1.369373	2.786150
H	−4.666419	−1.951713	2.957339
H	−5.124595	−0.345699	3.593945
H	−4.129042	3.129088	4.134741
H	−2.465003	2.503736	4.060181
H	−3.091894	3.541941	2.748875
H	−7.283061	1.425579	−3.314888
H	−6.048268	1.905866	−2.142333
H	−5.577412	1.094001	−3.646366
H	−6.767649	−0.990008	−2.890327
H	−7.237052	−0.179510	−1.390897
H	−0.837176	1.537075	−2.871929
H	−1.577064	3.153003	−2.865755
H	0.180051	2.986149	−3.118814
H	2.554559	5.014349	−0.224893
H	2.068374	5.631042	−1.823896
H	0.831410	5.003951	−0.704999
H	5.774478	2.649753	−1.609954
H	4.736206	1.291017	−2.098288
H	4.198758	2.972466	−2.381520

B3LYP Energy = −2096.62167486 a.u.

(*aS*,1*S*,3*S*,1'*S*,3'*S*)-**22**, Conf F

C	−0.525739	−1.249901	1.608908
C	−1.810974	−1.707675	1.926103
C	−2.916437	−0.909592	1.626656
C	−2.750058	0.312649	0.965939
C	−1.465359	0.760963	0.643432
C	−0.340848	−0.016543	0.978160
O	−3.637027	2.509381	0.380612
C	−2.592907	2.662433	−0.592424
C	−1.290719	2.109253	−0.024656
C	−3.978883	1.139135	0.631186
C	−2.508963	4.137058	−0.939348
C	−4.835651	0.585951	−0.507256
C	1.048418	0.462055	0.689611
C	1.773496	−0.007567	−0.411717
C	3.065454	0.487514	−0.633138
C	3.612275	1.452830	0.208291
C	2.896183	1.904439	1.326609
C	1.615958	1.406828	1.548744
C	−4.362168	−0.326685	−1.448384
C	−5.168743	−0.764253	−2.496916
C	−6.457393	−0.274021	−2.584325
C	−6.969517	0.630122	−1.668861
C	−6.146993	1.053953	−0.632727
C	1.190419	−1.016785	−1.372935
C	1.907098	−0.989486	−2.716523
O	3.321448	−1.103379	−2.490162
C	3.883569	0.022366	−1.827889
C	1.508315	−2.126500	−3.636961
F	−7.254118	−0.697911	−3.601262
O	4.900114	1.872130	−0.036639
C	5.057043	3.239330	−0.452864
O	3.527552	2.809140	2.128731
C	2.849294	3.275263	3.293555
C	5.318619	−0.417764	−1.500384
O	6.266266	−0.137988	−2.196419
O	5.369004	−1.192395	−0.414244
C	6.934258	−3.057820	−0.716022
C	6.661561	−1.735453	−0.026321
O	0.560337	−2.057025	1.851688
C	0.962107	−2.162394	3.227273
O	−1.979844	−2.919555	2.556315
C	−2.062212	−4.045109	1.664433
C	−4.536712	−1.466914	3.290867
O	−4.194307	−1.337351	1.899007
H	−2.865938	2.098153	−1.492792
H	−0.544503	2.040362	−0.819881
H	−0.893923	2.823359	0.704832
H	−4.611893	1.183843	1.518218
H	−1.712499	4.311757	−1.665595
H	−3.449533	4.484240	−1.368914
H	−2.296955	4.728319	−0.045759
H	1.044249	1.745374	2.400995
H	−3.355208	−0.714450	−1.365771
H	−4.808008	−1.473806	−3.229985
H	−7.986909	0.985117	−1.767957
H	−6.532071	1.763388	0.090231
H	1.270021	−2.024031	−0.950229
H	0.126235	−0.820334	−1.522339
H	1.713682	−0.028605	−3.214679
H	3.987630	0.850518	−2.540081
H	2.042763	−2.059587	−4.585350
H	0.436451	−2.088618	−3.840787
H	1.738771	−3.088840	−3.175559
H	6.112484	3.364229	−0.684624
H	4.462150	3.438942	−1.348153
H	4.768900	3.924497	0.344255
H	3.535492	3.965220	3.778266
H	1.927407	3.802946	3.034747
H	2.620428	2.452444	3.975778
H	7.874911	−3.471483	−0.345472
H	6.139580	−3.775795	−0.508068
H	7.019721	−2.930105	−1.795322
H	7.430951	−0.998473	−0.251236
H	6.583883	−1.856980	1.052445
H	1.259525	−1.184006	3.613217
H	0.159513	−2.577640	3.838091
H	1.820141	−2.831222	3.241963
H	−2.910640	−3.936048	0.984939
H	−1.137704	−4.152611	1.093575
H	−2.208160	−4.922402	2.291453
H	−5.584233	−1.759686	3.315278
H	−3.926036	−2.227451	3.775894
H	−4.414724	−0.509082	3.803927

B3LYP Energy = −2096.62161168 a.u.

(*aS*,1*S*,3*S*,1'*S*,3'*S*)-**22**, Conf G

C	0.837373	2.001297	−1.071170
C	2.184866	2.275883	−1.348601
C	3.181887	1.665849	−0.585163
C	2.842650	0.844605	0.497381
C	1.498533	0.621434	0.806021
C	0.485633	1.185342	0.008164
O	3.473883	−0.128247	2.647537
C	2.316984	−0.974648	2.596593
C	1.137420	−0.190548	2.031684
C	3.955027	0.266073	1.354333
C	2.068441	−1.484639	4.003948
C	4.740031	−0.871880	0.705528
C	−0.961712	0.968147	0.326862
C	−1.670205	−0.131642	−0.171712
C	−3.018841	−0.281639	0.177795
C	−3.640950	0.626538	1.029466
C	−2.938662	1.743609	1.505944
C	−1.601407	1.894262	1.153716
C	6.039166	−1.123545	1.156461
C	6.788785	−2.177651	0.649470
C	6.215654	−2.983639	−0.319872
C	4.936342	−2.770372	−0.796733
C	4.204419	−1.704732	−0.276992
C	−1.017217	−1.178030	−1.045906
C	−1.777418	−2.497207	−0.992767
O	−3.157842	−2.246382	−1.300426
C	−3.832060	−1.463390	−0.322280
C	−1.278264	−3.524319	−1.990410
F	6.940895	−4.016344	−0.824778
O	−4.975680	0.437971	1.310181
C	−5.303219	0.135691	2.677032
O	−3.637193	2.614612	2.288565
C	−2.975935	3.789588	2.755087
C	−5.125789	−1.029803	−1.029142
O	−5.197977	−0.134947	−1.834835
O	−6.145469	−1.827208	−0.687352
C	−8.412525	−2.589335	−0.810454
C	−7.423542	−1.571479	−1.331172
O	−0.157074	2.622521	−1.786722
C	−0.272623	2.265457	−3.174905
O	2.529866	3.136429	−2.366992
C	2.451735	4.529819	−2.015690
C	5.072519	1.569558	−2.060657
O	4.510799	1.947465	−0.790696
H	2.532526	−1.827594	1.941014
H	0.320140	−0.878638	1.804828
H	0.758485	0.487270	2.804877
H	4.664811	1.064649	1.570835
H	1.182020	−2.121869	4.026377
H	2.920601	−2.066552	4.357400
H	1.910588	−0.649251	4.689540
H	−1.038395	2.743643	1.513140
H	6.472985	−0.485254	1.917124
H	7.796950	−2.373239	0.990502
H	4.526896	−3.419809	−1.559344
H	3.206847	−1.521060	−0.654541
H	−0.984495	−0.842023	−2.088035
H	0.017641	−1.336670	−0.735529
H	−1.716842	−2.911776	0.023665
H	−4.108102	−2.100006	0.526981
H	−1.851288	−4.448785	−1.909421
H	−0.227062	−3.752459	−1.804235
H	−1.375399	−3.144264	−3.009359
H	−5.074913	0.978660	3.328495
H	−6.372431	−0.064321	2.693067
H	−4.762005	−0.752307	3.014980
H	−3.719334	4.338329	3.327888
H	−2.130453	3.541107	3.402129
H	−2.630617	4.406953	1.922044
H	−9.384456	−2.423277	−1.279048
H	−8.089759	−3.605035	−1.043859
H	−8.535039	−2.501407	0.270092
H	−7.726971	−0.549949	−1.098518
H	−7.287926	−1.649504	−2.410499
H	−0.446465	1.192086	−3.280058
H	−1.138000	2.806244	−3.551450
H	0.618946	2.552430	−3.732208
H	2.768046	5.085175	−2.896388
H	1.428437	4.806293	−1.754854
H	3.121908	4.752572	−1.182193
H	6.131681	1.810719	−2.000389
H	4.954702	0.495827	−2.222747
H	4.608605	2.124531	−2.874530

B3LYP Energy = −2096.62160619 a.u.

(*aS*,1*S*,3*S*,1'*S*,3'*S*)-**22**, Conf H

C	0.679673	−0.909709	1.863332
C	1.980312	−0.942858	2.381768
C	3.066456	−0.986650	1.506274
C	2.863116	−0.948077	0.122598
C	1.562453	−0.913319	−0.390274
C	0.458513	−0.908730	0.483106
O	3.711817	−1.369794	−2.125760
C	2.622439	−0.609280	−2.669916
C	1.350925	−0.924027	−1.890019
C	4.070927	−0.966318	−0.797293
C	2.514980	−0.958399	−4.142383
C	4.872452	0.334914	−0.834797
C	−0.945659	−0.924852	−0.036353
C	−1.730165	0.233453	−0.075729
C	−3.043912	0.146663	−0.552352
C	−3.556896	−1.060035	−1.027827
C	−2.754734	−2.212050	−1.025308
C	−1.466648	−2.132685	−0.505356
C	6.155420	0.300052	−1.389335
C	6.927338	1.449829	−1.496937
C	6.393867	2.643089	−1.039097
C	5.132007	2.722710	−0.482647
C	4.375905	1.556327	−0.383578

C	−1.187364	1.571022	0.369360
C	−1.978734	2.722300	−0.235901
O	−3.375324	2.515678	0.031366
C	−3.922380	1.382010	−0.634122
C	−1.610032	4.077287	0.336196
F	7.142106	3.774284	−1.136233
O	−4.819814	−1.036085	−1.571193
C	−5.802158	−1.945511	−1.038024
O	−3.298845	−3.343735	−1.562191
C	−2.511778	−4.532444	−1.589732
C	−5.285317	1.197011	0.048055
O	−5.462525	0.592394	1.077846
O	−6.238105	1.860454	−0.620224
C	−7.722630	3.028712	0.946898
C	−7.574685	1.890803	−0.044205
O	−0.386855	−0.798896	2.723686
C	−0.749190	−2.000166	3.423526
O	2.182005	−0.966183	3.743062
C	2.264683	0.333425	4.353580
C	4.770494	−2.167102	2.691836
O	4.357687	−0.986962	1.979189
H	2.855526	0.458220	−2.570119
H	0.567548	−0.213365	−2.163972
H	0.987133	−1.910750	−2.195244
H	4.747240	−1.751849	−0.457431
H	1.683555	−0.418878	−4.600488
H	3.431847	−0.690684	−4.669109
H	2.342367	−2.029479	−4.268954
H	−0.841103	−3.013538	−0.480112
H	6.556991	−0.640928	−1.746167
H	7.922901	1.428139	−1.920468
H	4.752925	3.673714	−0.132101
H	3.390427	1.607632	0.060522
H	−1.225720	1.646874	1.461029
H	−0.136852	1.660645	0.083430
H	−1.833095	2.731116	−1.325598
H	−4.103278	1.629316	−1.687328
H	−2.199787	4.866099	−0.132461
H	−0.552902	4.285966	0.160648
H	−1.792800	4.099502	1.412405
H	−6.751837	−1.635733	−1.469600
H	−5.579645	−2.971629	−1.321967
H	−5.848336	−1.856897	0.048816
H	−3.135690	−5.288333	−2.060258
H	−1.602922	−4.394081	−2.181102
H	−2.245981	−4.858784	−0.580819
H	−8.754527	3.066027	1.303044
H	−7.069326	2.888657	1.808257
H	−7.487792	3.985401	0.478050
H	−8.229373	2.022697	−0.903549
H	−7.777563	0.926447	0.419251
H	−1.063741	−2.772304	2.716800
H	0.081079	−2.363183	4.030938
H	−1.587115	−1.736495	4.065386
H	2.425001	0.162784	5.416275
H	3.104574	0.898661	3.943217
H	1.334718	0.886751	4.208189
H	4.184419	−2.302327	3.600199
H	4.676288	−3.049853	2.053598
H	5.817492	−2.013468	2.944585

B3LYP Energy = −2096.62153607 a.u.

(*aS*,1*S*,3*S*,1’*S*,3’*S*)-**22**, Conf I

C	−0.460032	1.549868	−1.203098
C	−1.727913	2.091445	−1.449541
C	−2.849446	1.262421	−1.395405
C	−2.717522	−0.085910	−1.045911
C	−1.450168	−0.620607	−0.795170
C	−0.307483	0.196619	−0.887670
O	−3.648384	−2.342224	−1.034171
C	−2.643069	−2.740545	−0.089724
C	−1.314686	−2.092600	−0.463978
C	−3.966245	−0.945279	−0.965495
C	−2.586978	−4.256584	−0.099108
C	−4.863918	−0.659670	0.238035
C	1.065721	−0.357099	−0.666606
C	1.751855	−0.161824	0.537799
C	3.040817	−0.691143	0.677667
C	3.621295	−1.444141	−0.343402
C	2.916763	−1.671215	−1.535777
C	1.656111	−1.102200	−1.689247
C	−4.413993	−0.023734	1.394083
C	−5.258494	0.169957	2.485263
C	−6.561278	−0.281318	2.396630
C	−7.050487	−0.914410	1.266342
C	−6.190133	−1.098843	0.191301
C	1.128948	0.595532	1.687107
C	1.797331	0.250725	3.011026
O	3.217692	0.412811	2.868604
C	3.811134	−0.525607	1.977890
C	1.358694	1.138369	4.159488
F	−7.395511	−0.092565	3.453566
O	4.846722	−2.003292	−0.077732
C	5.915765	−1.819467	−1.023076
O	3.521380	−2.465607	−2.467766
C	2.833382	−2.730333	−3.688051
C	5.251560	−0.015747	1.828109
O	6.175779	−0.436489	2.482958
O	5.342463	0.997475	0.961129
C	6.492729	2.771119	−0.162982
C	6.635517	1.650930	0.841896
O	0.638741	2.376434	−1.195893
C	1.103346	2.802323	−2.486921
O	−1.858860	3.422966	−1.773282
C	−2.054235	4.290379	−0.642935
C	−4.398111	2.226445	−2.937128
O	−4.112622	1.763514	−1.604670
H	−2.944850	−2.400146	0.908610
H	−0.598856	−2.229711	0.350059
H	−0.897622	−2.619913	−1.328598
H	−4.561943	−0.766505	−1.861470

H	−1.818857	−4.612258	0.590603
H	−3.545928	−4.677605	0.205710
H	−2.349222	−4.624171	−1.099764
H	1.104005	−1.254319	−2.605756
H	−3.395168	0.337137	1.450122
H	−4.916038	0.664295	3.384889
H	−8.079537	−1.247542	1.233989
H	−6.557203	−1.594972	−0.699412
H	1.214825	1.673588	1.514977
H	0.061763	0.369668	1.747617
H	1.593098	−0.800426	3.260009
H	3.895470	−1.499881	2.474820
H	1.864411	0.851262	5.082221
H	0.281503	1.050899	4.314397
H	1.593457	2.183014	3.945218
H	5.820547	−2.506486	−1.860924
H	5.929831	−0.791643	−1.391937
H	6.832210	−2.022619	−0.472583
H	2.645651	−1.810463	−4.248175
H	3.492207	−3.375199	−4.264346
H	1.887730	−3.248003	−3.507245
H	7.449908	3.284920	−0.271445
H	6.197723	2.387158	−1.140639
H	5.748732	3.498300	0.164738
H	6.925965	2.020299	1.826178
H	7.371547	0.910277	0.526873
H	1.419365	1.941458	−3.081938
H	0.329268	3.358858	−3.016855
H	1.959953	3.447665	−2.303569
H	−2.965880	4.022325	−0.104250
H	−1.195524	4.243954	0.030161
H	−2.149827	5.297926	−1.042774
H	−5.445622	2.520305	−2.933920
H	−3.772882	3.079337	−3.198910
H	−4.248560	1.420453	−3.660659

B3LYP Energy = −2096.62152489 a.u.

(*aS*,1*S*,3*S*,1’*S*,3’*S*)-**22**, Conf J

C	0.712839	−0.696600	1.990899
C	2.024964	−0.664921	2.480246
C	3.091287	−0.808896	1.591193
C	2.856313	−0.930283	0.216969
C	1.544587	−0.953776	−0.267142
C	0.460560	−0.851082	0.625053
O	3.656356	−1.594672	−1.990642
C	2.552282	−0.899837	−2.589392
C	1.299687	−1.130514	−1.751759
C	4.043691	−1.048654	−0.722261
C	2.415919	−1.405881	−4.013146
C	4.840312	0.241305	−0.918937
C	−0.953885	−0.921928	0.139679
C	−1.741629	0.227213	0.002542
C	−3.052332	0.095408	−0.472666
C	−3.557686	−1.149234	−0.838182
C	−2.779779	−2.304456	−0.671812
C	−1.481017	−2.173454	−0.189909
C	6.117430	0.147543	−1.480156
C	6.882545	1.280301	−1.727397
C	6.348037	2.516307	−1.403975
C	5.091512	2.654842	−0.846834
C	4.342469	1.504911	−0.606026
C	−1.206936	1.602435	0.324531
C	−2.006732	2.690211	−0.380301
O	−3.401814	2.501958	−0.092017
C	−3.948288	1.310335	−0.643648
C	−1.645366	4.092749	0.069100
F	7.089466	3.631618	−1.638968
O	−4.861206	−1.216028	−1.276572
C	−5.050109	−1.598538	−2.649325
O	−3.372119	−3.489824	−0.995199
C	−2.632700	−4.693777	−0.801881
C	−5.292632	1.157258	0.085757
O	−5.415637	0.726523	1.205443
O	−6.294923	1.633422	−0.666074
C	−7.894089	2.889623	0.709849
C	−7.631423	1.628457	−0.089799
O	−0.335006	−0.495468	2.857523
C	−0.675802	−1.615329	3.691284
O	2.261325	−0.528689	3.829016
C	2.254059	0.830088	4.301001
C	4.825683	−1.837934	2.870372
O	4.392971	−0.753259	2.029614
H	2.782411	0.172663	−2.611016
H	0.507333	−0.455305	−2.083833
H	0.934169	−2.145772	−1.939488
H	4.729542	−1.792028	−0.313742
H	1.571862	−0.922174	−4.509001
H	3.320177	−1.192113	−4.584473
H	2.248335	−2.485137	−4.020748
H	−0.860815	−3.048750	−0.057965
H	6.520444	−0.826706	−1.730279
H	7.873840	1.213685	−2.156235
H	4.711363	3.638477	−0.604151
H	3.361940	1.603272	−0.158873
H	−1.247374	1.776235	1.404754
H	−0.156805	1.672892	0.031298
H	−1.862178	2.603598	−1.466808
H	−4.156844	1.462908	−1.709464
H	−2.238867	4.833886	−0.467639
H	−0.589144	4.290518	−0.123472
H	−1.829886	4.209139	1.138893
H	−4.512408	−0.918180	−3.315396
H	−4.717597	−2.622437	−2.818545
H	−6.118521	−1.521649	−2.839257
H	−3.296710	−5.499812	−1.103992
H	−1.732734	−4.713517	−1.422346
H	−2.356843	−4.825452	0.247559
H	−8.926647	2.883254	1.066161
H	−7.235177	2.951847	1.576060
H	−7.751221	3.778421	0.093282
H	−8.291114	1.559660	−0.952855

H	−7.744033	0.734401	0.521589
H	−1.000015	−2.461419	3.080137
H	0.169042	−1.907507	4.316635
H	−1.502115	−1.286515	4.317872
H	3.035996	1.412045	3.807476
H	1.279829	1.292187	4.130943
H	2.454776	0.783430	5.369452
H	5.877936	−1.654755	3.077540
H	4.261560	−1.861173	3.801950
H	4.719102	−2.791746	2.346424

B3LYP Energy = −2096.62151038 a.u.

(*aS*,1*S*,3*S*,1'*S*,3'*S*)-**22**, Conf K

C	0.545688	2.048302	−0.039973
C	1.837584	2.577462	−0.154250
C	2.922750	1.846516	0.332248
C	2.730814	0.574017	0.881541
C	1.439971	0.046798	0.985712
C	0.334353	0.792450	0.535053
O	3.561396	−1.244009	2.281160
C	2.533886	−2.101470	1.762078
C	1.237561	−1.310732	1.626983
C	3.940025	−0.203709	1.369412
C	2.411664	−3.284864	2.703734
C	4.833588	−0.763821	0.262833
C	−1.062227	0.271766	0.676685
C	−1.742303	−0.309039	−0.400209
C	−3.041371	−0.793582	−0.199043
C	−3.638112	−0.730066	1.057403
C	−2.968087	−0.122452	2.129438
C	−1.680800	0.364965	1.926111
C	4.412194	−0.921720	−1.056281
C	5.250845	−1.480183	−2.018720
C	6.518774	−1.874320	−1.637065
C	6.979113	−1.732489	−0.338605
C	6.125366	−1.175063	0.604709
C	−1.102084	−0.442949	−1.761833
C	−1.780694	−1.521693	−2.596083
O	−3.197059	−1.279630	−2.604389
C	−3.811937	−1.451372	−1.333286
C	−1.323265	−1.545670	−4.041427
F	7.347229	−2.415703	−2.569353
O	−4.929006	−1.189115	1.185357
C	−5.114185	−2.355541	2.005270
O	−3.647940	−0.050167	3.309725
C	−3.015493	0.583203	4.419899
C	−5.240873	−0.925055	−1.540155
O	−6.178269	−1.647505	−1.785248
O	−5.291347	0.408539	−1.504938
C	−7.394852	1.204602	−0.517061
C	−6.568394	1.049918	−1.778511
O	−0.517693	2.741226	−0.569230
C	−0.957292	3.878871	0.190291
O	2.033106	3.820410	−0.711716
C	2.133468	3.817943	−2.146691
C	4.526983	3.489519	0.987204
O	4.207757	2.320042	0.211784
H	2.843845	−2.461822	0.773320
H	0.512642	−1.892662	1.052696
H	0.801057	−1.177242	2.622581
H	4.554851	0.464882	1.973265
H	1.623087	−3.959830	2.364899
H	3.348589	−3.841882	2.743796
H	2.166219	−2.946710	3.712922
H	−1.142125	0.829759	2.739698
H	3.421858	−0.597945	−1.348713
H	4.930654	−1.603776	−3.044957
H	7.981795	−2.047876	−0.081393
H	6.469512	−1.060053	1.625735
H	−1.162228	0.510350	−2.297732
H	−0.040735	−0.678427	−1.654421
H	−1.600822	−2.504686	−2.137460
H	−3.922008	−2.522642	−1.123401
H	−1.833292	−2.336220	−4.593282
H	−0.247811	−1.725905	−4.094506
H	−1.538026	−0.590500	−4.524891
H	−6.163978	−2.624992	1.912580
H	−4.493980	−3.180704	1.645149
H	−4.877005	−2.140796	3.047114
H	−2.100841	0.059406	4.710196
H	−2.784603	1.629085	4.201395
H	−3.732832	0.535261	5.235367
H	−8.307401	1.757766	−0.751044
H	−7.677250	0.234826	−0.107984
H	−6.843534	1.758620	0.243785
H	−6.292761	2.017144	−2.194812
H	−7.095151	0.469605	−2.534701
H	−0.151587	4.604744	0.308285
H	−1.773565	4.323976	−0.374845
H	−1.325368	3.563972	1.170135
H	1.210131	3.448283	−2.596837
H	2.296371	4.851998	−2.443781
H	2.978733	3.206778	−2.471510
H	3.927960	4.343627	0.673650
H	4.369711	3.295299	2.051699
H	5.581318	3.689037	0.807680

B3LYP Energy = −2096.62142704 a.u.

(*aS*,1*S*,3*S*,1'*S*,3'*S*)-**22**, Conf L

C	−0.727347	−1.552762	−1.429824
C	−2.045455	−1.799887	−1.834649
C	−3.095125	−1.519888	−0.958291
C	−2.838865	−0.951955	0.294590
C	−1.521857	−0.701653	0.691922
C	−0.453093	−1.014516	−0.169302
O	−3.590742	−0.489829	2.569335
C	−2.504976	0.434812	2.732255
C	−1.254088	−0.132808	2.070380
C	−4.010268	−0.630376	1.204997
C	−2.330405	0.672116	4.220562

C	−4.843689	0.576935	0.775747
C	0.969462	−0.800436	0.246433
C	1.710866	0.296848	−0.207314
C	3.034850	0.443519	0.223872
C	3.599585	−0.461294	1.118646
C	2.866440	−1.575230	1.553060
C	1.554513	−1.726147	1.114462
C	−6.119893	0.725892	1.326820
C	−6.917406	1.820466	1.017591
C	−6.416209	2.770706	0.143757
C	−5.162104	2.662314	−0.425512
C	−4.380236	1.555203	−0.102375
C	1.114034	1.339080	−1.122450
C	1.877218	2.654400	−1.035672
O	3.273700	2.396983	−1.256698
C	3.885549	1.613077	−0.238794
C	1.444360	3.673964	−2.071232
F	−7.189151	3.844768	−0.168833
O	4.913956	−0.273140	1.482580
C	5.153574	0.033962	2.866438
O	3.512136	−2.445296	2.381852
C	2.818031	−3.612582	2.817476
C	5.208070	1.159386	−0.878263
O	5.313713	0.233313	−1.643491
O	6.205621	1.985082	−0.532050
C	8.330139	0.779751	−0.279591
C	7.516331	1.745754	−1.118239
O	0.300899	−1.772560	−2.315473
C	0.676071	−3.146856	−2.503249
O	−2.303755	−2.346287	−3.070830
C	−2.343281	−1.393016	−4.147455
C	−4.804250	−3.087492	−1.527500
O	−4.403414	−1.719440	−1.330498
H	−2.774664	1.381028	2.247148
H	−0.487062	0.643425	2.014053
H	−0.843645	−0.920190	2.711430
H	−4.677367	−1.493076	1.221842
H	−1.501914	1.360159	4.400933
H	−3.236007	1.102703	4.649737
H	−2.116165	−0.267836	4.734015
H	0.970114	−2.574803	1.440223
H	−6.496644	−0.025285	2.010931
H	−7.908006	1.938079	1.436913
H	−4.809257	3.424063	−1.108316
H	−3.401307	1.456944	−0.553079
H	1.133732	0.985256	−2.158568
H	0.065810	1.506684	−0.864381
H	1.757688	3.079233	−0.028637
H	4.126624	2.251827	0.619488
H	2.015001	4.597357	−1.964491
H	0.384535	3.906891	−1.951533
H	1.601259	3.283102	−3.078524
H	4.885591	−0.807105	3.505178
H	6.219318	0.235388	2.950200
H	4.590587	0.922248	3.166082
H	3.520831	−4.157480	3.442803
H	1.933368	−3.355172	3.406138
H	2.524829	−4.239030	1.971146
H	9.326390	0.675221	−0.715251
H	8.441715	1.148497	0.741310
H	7.864982	−0.205546	−0.249519
H	7.382145	1.378290	−2.134434
H	7.976218	2.731836	−1.149821
H	−0.163926	−3.732184	−2.880208
H	1.481361	−3.144187	−3.234712
H	1.040751	−3.573935	−1.565699
H	−2.572215	−1.958907	−5.048240
H	−3.125576	−0.650323	−3.973839
H	−1.376611	−0.898896	−4.260506
H	−4.254305	−3.542812	−2.350214
H	−4.649691	−3.665177	−0.612096
H	−5.866316	−3.057550	−1.761583

B3LYP Energy = −2096.62125425 a.u.

(*aS*,1*S*,3*S*,1'*S*,3'*S*)-**22**, Conf M

C	−0.742088	0.587280	1.996565
C	−2.057081	0.533870	2.484515
C	−3.117400	0.745563	1.597789
C	−2.864896	0.980671	0.238123
C	−1.553672	1.016287	−0.239746
C	−0.477035	0.822509	0.646628
O	−3.641281	1.812108	−1.927520
C	−2.553751	1.121859	−2.559385
C	−1.300064	1.284178	−1.708934
C	−4.047801	1.222534	−0.684591
C	−2.403327	1.694687	−3.956315
C	−4.923286	−0.003110	−0.940713
C	0.942613	0.900778	0.179017
C	1.720964	−0.248451	−0.002555
C	3.047785	−0.108727	−0.427873
C	3.582176	1.147807	−0.712893
C	2.788775	2.296556	−0.565191
C	1.485461	2.157084	−0.098452
C	−6.244783	0.199868	−1.348666
C	−7.079809	−0.867874	−1.653858
C	−6.569719	−2.150764	−1.547094
C	−5.270620	−2.398092	−1.146690
C	−4.452438	−1.312122	−0.841874
C	1.159427	−1.630106	0.238639
C	1.957997	−2.694905	−0.500624
O	3.348754	−2.539353	−0.175697
C	3.917377	−1.330357	−0.667794
C	1.568191	−4.111702	−0.125871
F	−7.378324	−3.203560	−1.840962
O	4.858620	1.186872	−1.222515
C	5.833129	2.007413	−0.549240
O	3.355729	3.487861	−0.917314
C	2.582919	4.678172	−0.778606
C	5.272044	−1.259119	0.050383
O	5.440351	−0.831300	1.166857
O	6.233422	−1.811458	−0.699196

C	8.460654	−2.586007	−1.115205
C	7.556438	−1.916864	−0.105393
O	0.304947	0.378245	2.867138
C	0.665666	1.513048	3.672510
O	−2.313092	0.390989	3.828070
C	−1.968683	−0.878139	4.414593
C	−4.986992	−0.218229	2.803596
O	−4.424551	0.828096	1.998280
H	−2.806907	0.057006	−2.635188
H	−0.514840	0.621195	−2.080204
H	−0.923455	2.304767	−1.834177
H	−4.682716	1.982653	−0.228408
H	−1.571814	1.214582	−4.476244
H	−3.312587	1.533490	−4.536940
H	−2.207609	2.768175	−3.909757
H	0.864102	3.031108	0.035153
H	−6.628689	1.209969	−1.429860
H	−8.105171	−0.716088	−1.964668
H	−4.910920	−3.416002	−1.072086
H	−3.437678	−1.497724	−0.514371
H	1.172380	−1.857480	1.309838
H	0.115167	−1.668437	−0.080393
H	1.838290	−2.554853	−1.584461
H	4.111813	−1.429826	−1.742758
H	2.162931	−4.835218	−0.684828
H	0.513816	−4.284789	−0.349785
H	1.727831	−4.280844	0.940940
H	6.792883	1.737728	−0.985373
H	5.633311	3.064310	−0.710467
H	5.842204	1.785221	0.519331
H	3.226583	5.488380	−1.112130
H	1.687427	4.646235	−1.404585
H	2.295871	4.847931	0.262450
H	9.464654	−2.679864	−0.696839
H	8.099106	−3.584795	−1.363732
H	8.526027	−1.999955	−2.033059
H	7.901742	−0.914359	0.150375
H	7.475418	−2.494335	0.816339
H	1.487206	1.187073	4.306974
H	1.001614	2.338854	3.040886
H	−0.173970	1.835551	4.290997
H	−2.523850	−1.686328	3.931095
H	−0.899290	−1.068944	4.337481
H	−2.260465	−0.813780	5.460787
H	−4.702061	−0.108794	3.848136
H	−6.066202	−0.121443	2.702306
H	−4.679462	−1.198385	2.432285

B3LYP Energy = −2096.62094005 a.u.

(*aS*,1*S*,3*S*,1’*S*,3’*S*)-**22**, Conf N

C	−0.475079	1.534722	−1.252072
C	−1.750690	2.059952	−1.511481
C	−2.859825	1.211140	−1.438886
C	−2.693552	−0.138379	−1.091488
C	−1.421600	−0.647966	−0.827898
C	−0.295811	0.193827	−0.909837
O	−3.580518	−2.416861	−1.058129
C	−2.581092	−2.772362	−0.092204
C	−1.261431	−2.111413	−0.471901
C	−3.925915	−1.024793	−1.036387
C	−2.499539	−4.287030	−0.059752
C	−4.873089	−0.730379	0.126401
C	1.087397	−0.327856	−0.674730
C	1.777942	−0.065574	0.514768
C	3.067984	−0.587181	0.678257
C	3.647197	−1.378783	−0.310031
C	2.965412	−1.622907	−1.511191
C	1.687169	−1.098588	−1.674480
C	−6.202253	−1.149888	0.020554
C	−7.104145	−0.963669	1.060904
C	−6.653855	−0.349073	2.217232
C	−5.349672	0.082861	2.364599
C	−4.463289	−0.112146	1.307266
C	1.157714	0.745431	1.628036
C	1.841545	0.480255	2.962907
O	3.259920	0.641869	2.798512
C	3.848126	−0.346286	1.961300
C	1.407453	1.428836	4.063286
F	−7.528474	−0.159162	3.240799
O	4.933451	−1.825567	−0.110519
C	5.102998	−3.245724	0.038208
O	3.627787	−2.360315	−2.448366
C	2.983757	−2.609307	−3.696288
C	5.290756	0.142281	1.763082
O	6.219300	−0.257339	2.426003
O	5.376901	1.102830	0.841501
C	6.547445	2.734615	−0.461459
C	6.690241	1.681232	0.613163
O	0.616674	2.373545	−1.316832
C	1.123082	2.613480	−2.640553
O	−1.910300	3.354812	−1.946643
C	−1.606631	4.394176	−0.998118
C	−4.673976	2.818460	−1.262169
O	−4.129587	1.593802	−1.775006
H	−2.898714	−2.410697	0.893903
H	−0.547436	−2.221093	0.347991
H	−0.833459	−2.648370	−1.325078
H	−4.490647	−0.879935	−1.957796
H	−1.734576	−4.610891	0.648940
H	−3.455180	−4.716032	0.244376
H	−2.243109	−4.677267	−1.047096
H	1.141830	−1.275829	−2.590531
H	−6.538746	−1.631283	−0.890093
H	−8.135394	−1.282019	0.982343
H	−5.037872	0.561847	3.283532
H	−3.442406	0.232781	1.409127
H	1.231915	1.813133	1.396392
H	0.093380	0.513179	1.710697
H	1.646083	−0.556302	3.273140
H	3.933295	−1.289153	2.516051
H	1.920461	1.195388	4.997034

H	0.331630	1.346569	4.230103
H	1.636872	2.460667	3.789612
H	4.848465	−3.766642	−0.884549
H	6.153965	−3.400181	0.272572
H	4.488260	−3.621879	0.860263
H	3.688131	−3.196450	−4.280207
H	2.060313	−3.178850	−3.562197
H	2.765122	−1.676751	−4.222920
H	7.521296	3.188373	−0.655735
H	6.181306	2.297778	−1.391549
H	5.859128	3.521523	−0.149943
H	7.050282	2.102382	1.552750
H	7.369706	0.882301	0.313854
H	1.485767	1.683841	−3.085182
H	0.355244	3.056546	−3.277542
H	1.953556	3.307385	−2.528138
H	−2.252846	4.311845	−0.120272
H	−0.562366	4.352816	−0.691932
H	−1.807534	5.333890	−1.508445
H	−4.332778	3.674795	−1.840304
H	−5.754303	2.718831	−1.348339
H	−4.410037	2.950214	−0.210440

B3LYP Energy = −2096.62091544 a.u.

(*aS*,1*S*,3*S*,1'*S*,3'*S*)-**22**, Conf O

C	0.729112	−1.395976	1.578576
C	2.043925	−1.580901	2.024819
C	3.102391	−1.394669	1.133992
C	2.858553	−0.975984	−0.178824
C	1.544115	−0.788971	−0.618046
C	0.467263	−1.013414	0.260309
O	3.633780	−0.760535	−2.482843
C	2.539302	0.126672	−2.758859
C	1.287330	−0.384023	−2.054820
C	4.037698	−0.744232	−1.107146
C	2.383054	0.200116	−4.266077
C	4.855297	0.512271	−0.806026
C	−0.951233	−0.862724	−0.194256
C	−1.693852	0.289626	0.086274
C	−3.019817	0.368740	−0.358069
C	−3.585640	−0.659736	−1.112043
C	−2.825484	−1.798236	−1.425564
C	−1.525517	−1.894332	−0.939071
C	6.123338	0.626126	−1.383941
C	6.908329	1.755118	−1.187097
C	6.403207	2.775765	−0.399007
C	5.156526	2.704652	0.191849
C	4.386952	1.562206	−0.018145
C	−1.091275	1.453950	0.836291
C	−1.835755	2.748050	0.539080
O	−3.235964	2.541837	0.784619
C	−3.849191	1.614823	−0.107245
C	−1.397672	3.912400	1.406136
F	7.164479	3.883975	−0.196144
O	−4.854485	−0.458049	−1.602088
C	−5.858411	−1.446764	−1.300512
O	−3.417978	−2.737126	−2.220810
C	−2.676020	−3.903987	−2.568512
C	−5.197801	1.330464	0.571337
O	−5.396234	0.457557	1.381096
O	−6.099130	2.251109	0.204000
C	−8.406931	1.411955	0.124714
C	−7.392057	2.253354	0.872636
O	−0.310646	−1.515841	2.470266
C	−0.667469	−2.859453	2.834146
O	2.291732	−1.975026	3.320038
C	2.314958	−0.900394	4.276065
C	4.815693	−2.870112	1.901565
O	4.407062	−1.537833	1.543202
H	2.790851	1.123452	−2.375455
H	0.506710	0.379247	−2.095813
H	0.899425	−1.245932	−2.608176
H	4.712969	−1.596575	−1.020824
H	1.546255	0.850075	−4.529746
H	3.287836	0.598436	−4.726949
H	2.191768	−0.792819	−4.678976
H	−0.930340	−2.769198	−1.158845
H	6.502936	−0.179833	−2.000791
H	7.892488	1.846449	−1.627634
H	4.799784	3.522026	0.804730
H	3.414078	1.493546	0.450694
H	−1.123644	1.265531	1.914620
H	−0.038837	1.566829	0.566499
H	−1.704422	3.008855	−0.520845
H	−4.053173	2.110344	−1.064436
H	−1.959789	4.812524	1.154217
H	−0.335519	4.115622	1.256269
H	−1.560965	3.684857	2.461440
H	−5.919064	−1.604921	−0.222126
H	−6.796016	−1.030344	−1.662184
H	−5.648157	−2.385727	−1.807139
H	−3.336225	−4.496701	−3.196874
H	−1.773837	−3.649139	−3.130732
H	−2.403438	−4.482557	−1.681948
H	−9.383486	1.515871	0.602887
H	−8.498030	1.741137	−0.911568
H	−8.133070	0.357112	0.137593
H	−7.256696	1.904307	1.895093
H	−7.680482	3.302938	0.885685
H	−1.001454	−3.417170	1.955477
H	0.172183	−3.372129	3.305489
H	−1.490654	−2.773704	3.540327
H	2.535995	−1.354002	5.240164
H	3.095956	−0.179235	4.023945
H	1.345022	−0.401457	4.319335
H	4.261366	−3.230306	2.767322
H	4.673367	−3.551452	1.058358
H	5.875395	−2.804746	2.139206

B3LYP Energy = −2096.62091453 a.u.

(*aS*,1*S*,3*S*,1'*S*,3'*S*)-**22**, Conf P

C	−0.760287	−0.152776	2.109251
C	−2.083974	−0.356763	2.530023
C	−3.128291	0.157010	1.754709
C	−2.850673	0.842632	0.562502
C	−1.531579	1.027224	0.145824
C	−0.471238	0.529637	0.926667
O	−3.586329	2.378577	−1.193230
C	−2.490042	1.941778	−2.008534
C	−1.250666	1.783491	−1.136100
C	−4.016179	1.396782	−0.239226
C	−2.310855	2.965103	−3.114411
C	−4.884957	0.340030	−0.919523
C	0.956556	0.752026	0.535256
C	1.727835	−0.266984	−0.036643
C	3.055396	0.005973	−0.388046
C	3.596566	1.275864	−0.206661
C	2.833655	2.289355	0.391758
C	1.516898	2.014481	0.748101
C	−6.195671	0.681806	−1.264776
C	−7.022780	−0.209983	−1.936374
C	−6.515747	−1.456635	−2.262060
C	−5.227238	−1.837362	−1.939275
C	−4.416823	−0.927973	−1.262596
C	1.158873	−1.639132	−0.309744
C	1.958729	−2.368227	−1.381232
O	3.347488	−2.357890	−1.012689
C	3.935402	−1.062341	−1.013934
C	1.555828	−3.820087	−1.553157
F	−7.316605	−2.338649	−2.917198
O	4.916436	1.470198	−0.547217
C	5.166885	2.380766	−1.631346
O	3.457697	3.485965	0.589740
C	2.729119	4.537005	1.221057
C	5.252058	−1.260347	−0.246573
O	5.342989	−1.313564	0.954951
O	6.274277	−1.441820	−1.092229
C	8.564893	−1.893784	−1.626393
C	7.574802	−1.708035	−0.499305
O	0.270057	−0.659356	2.870193
C	0.624437	0.126936	4.020478
O	−2.361643	−0.950400	3.739029
C	−2.035124	−2.347966	3.855565
C	−5.019497	−1.133809	2.558208
O	−4.442492	0.110527	2.135799
H	−2.746878	0.973485	−2.456025
H	−0.464240	1.279400	−1.703105
H	−0.863100	2.778774	−0.894292
H	−4.660111	1.955608	0.440452
H	−1.470763	2.688651	−3.754822
H	−3.208897	3.025910	−3.730560
H	−2.112758	3.952672	−2.692045
H	0.909005	2.781764	1.206086
H	−6.577287	1.662165	−1.005111
H	−8.039953	0.047758	−2.200845
H	−4.869766	−2.822748	−2.208212
H	−3.410280	−1.222242	−0.994528
H	1.166446	−2.237841	0.607208
H	0.116325	−1.555277	−0.625304
H	1.849811	−1.840366	−2.339543
H	4.183626	−0.774399	−2.042738
H	2.153485	−4.295843	−2.331733
H	0.503342	−3.889256	−1.834979
H	1.701280	−4.368211	−0.620144
H	4.873534	3.395582	−1.364261
H	6.238489	2.342648	−1.815048
H	4.632005	2.063649	−2.530765
H	3.418653	5.374772	1.288646
H	1.858522	4.830772	0.628530
H	2.408129	4.249939	2.225620
H	9.553659	−2.096980	−1.210594
H	8.282456	−2.734147	−2.262275
H	8.631716	−0.996162	−2.242936
H	7.838234	−0.866170	0.142260
H	7.494225	−2.599301	0.124228
H	0.976853	1.114570	3.714071
H	−0.223672	0.228234	4.700208
H	1.432999	−0.406273	4.515880
H	−2.597868	−2.934381	3.124385
H	−0.967705	−2.513839	3.718044
H	−2.330761	−2.642341	4.860419
H	−6.096250	−1.006906	2.464049
H	−4.696977	−1.949733	1.907473
H	−4.761793	−1.358172	3.591380

B3LYP Energy = −2096.62082184 a.u.

(*aS*,1*S*,3*S*,1'*S*,3'*S*)-**22**, Conf Q

C	0.745104	−1.822622	1.266327
C	2.074346	−2.074892	1.636459
C	3.113474	−1.593587	0.838714
C	2.832994	−0.922963	−0.358480
C	1.507202	−0.723077	−0.751076
C	0.451406	−1.158530	0.071671
O	3.573092	−0.235335	−2.583814
C	2.430345	0.626158	−2.685069
C	1.215377	−0.074689	−2.088154
C	3.991671	−0.477791	−1.233123
C	2.253955	0.973609	−4.151474
C	4.777747	0.716156	−0.694611
C	−0.977718	−0.974111	−0.336918
C	−1.691574	0.190276	−0.029982
C	−3.029912	0.289819	−0.432228
C	−3.637853	−0.730875	−1.162059
C	−2.908017	−1.881681	−1.500638
C	−1.592881	−1.995486	−1.062664
C	4.230753	1.661441	0.172958
C	4.967122	2.768224	0.590752
C	6.261528	2.909182	0.128253
C	6.845555	1.992377	−0.729826
C	6.091764	0.898254	−1.135673
C	−1.053282	1.347631	0.704131

C	−1.792638	2.651443	0.433882
O	−3.185814	2.458854	0.725096
C	−3.834149	1.547710	−0.156814
C	−1.313018	3.808645	1.288461
F	6.990782	3.982141	0.534017
O	−4.922106	−0.509772	−1.600981
C	−5.930978	−1.479155	−1.255738
O	−3.545573	−2.812734	−2.268388
C	−2.841508	−4.001204	−2.623904
C	−5.170727	1.265787	0.544074
O	−5.330051	0.444160	1.414231
O	−6.117330	2.105852	0.105489
C	−7.479550	2.939706	1.969003
C	−7.424565	2.048975	0.743248
O	−0.284785	−2.331518	2.020402
C	−0.467482	−1.759392	3.326298
O	2.352868	−2.790119	2.780261
C	2.330845	−4.217686	2.602309
C	4.951151	−1.358591	2.372294
O	4.428167	−1.864351	1.130434
H	2.631345	1.546352	−2.121936
H	0.393190	0.637982	−1.994835
H	0.871244	−0.844680	−2.787916
H	4.689606	−1.310747	−1.320255
H	1.383102	1.618161	−4.287207
H	3.132888	1.497169	−4.529903
H	2.108193	0.067728	−4.743806
H	−1.018389	−2.879278	−1.299852
H	3.220435	1.535794	0.540330
H	4.548968	3.504774	1.264311
H	7.864925	2.135122	−1.063666
H	6.533740	0.172908	−1.808523
H	−1.056751	1.166332	1.784394
H	−0.007202	1.449498	0.408408
H	−1.692796	2.913000	−0.629243
H	−4.051682	2.051940	−1.106089
H	−1.872741	4.715476	1.056291
H	−0.254047	4.000499	1.104841
H	−1.445206	3.581288	2.348186
H	−5.915365	−1.675767	−0.182171
H	−6.880366	−1.022938	−1.528933
H	−5.788821	−2.405048	−1.808458
H	−3.536431	−4.588278	−3.219223
H	−1.954800	−3.774438	−3.221608
H	−2.549916	−4.571344	−1.738117
H	−8.491690	2.927319	2.379453
H	−6.794225	2.590695	2.741707
H	−7.227144	3.969713	1.712862
H	−8.110536	2.391342	−0.029247
H	−7.648112	1.012306	0.990531
H	−1.326362	−2.267911	3.758859
H	0.411223	−1.915750	3.952077
H	−0.685125	−0.691732	3.247884
H	2.562928	−4.650840	3.573241
H	1.342912	−4.551203	2.278662
H	3.085334	−4.525143	1.874493
H	4.826300	−0.274804	2.424320
H	4.465361	−1.831103	3.224296
H	6.012294	−1.598336	2.365446

B3LYP Energy = −2096.62076943 a.u.

(*aS*,1*S*,3*S*,1'*S*,3'*S*)-**22**, Conf R

C	−0.493348	−0.323379	1.965607
C	−1.763546	−0.595296	2.489441
C	−2.889993	−0.046339	1.875097
C	−2.758073	0.727128	0.716538
C	−1.488349	0.987212	0.191440
C	−0.342299	0.470490	0.825341
O	−3.699271	2.389252	−0.800074
C	−2.678448	2.079040	−1.760875
C	−1.354061	1.864274	−1.036932
C	−4.008969	1.288707	0.064810
C	−2.631292	3.221958	−2.757394
C	−4.872564	0.260964	−0.666754
C	1.033109	0.768331	0.314229
C	1.754290	−0.164064	−0.440769
C	3.044994	0.166097	−0.873071
C	3.593141	1.419967	−0.602167
C	2.853618	2.367472	0.122297
C	1.590928	2.018026	0.591236
C	−4.408200	−0.994216	−1.055274
C	−5.222955	−1.877713	−1.760457
C	−6.510709	−1.482808	−2.067303
C	−7.013584	−0.246438	−1.698050
C	−6.183204	0.618702	−0.996908
C	1.167564	−1.508191	−0.804790
C	1.871891	−2.116442	−2.009575
O	3.288101	−2.111654	−1.768328
C	3.851652	−0.805607	−1.719157
C	1.470245	−3.552542	−2.284597
F	−7.315559	−2.339057	−2.751194
O	4.823415	1.688950	−1.149199
C	5.868867	2.185703	−0.294139
O	3.428589	3.593445	0.299802
C	2.701969	4.590063	1.015157
C	5.290717	−1.054104	−1.244742
O	6.226080	−1.160221	−2.001858
O	5.361631	−1.215502	0.081524
C	6.807898	−3.108985	0.661338
C	6.643286	−1.601918	0.652614
O	0.611398	−0.908117	2.539487
C	1.041429	−0.329507	3.782218
O	−1.896273	−1.368284	3.620535
C	−1.951031	−2.783434	3.369603
C	−4.479144	0.214678	3.640102
O	−4.155049	−0.314333	2.341533
H	−2.959660	1.157547	−2.285553
H	−0.625424	1.433097	−1.727638
H	−0.952957	2.839334	−0.741033
H	−4.628699	1.732745	0.844821
H	−1.856215	3.042777	−3.505399

H	−3.588505	3.323150	−3.270505
H	−2.408980	4.162858	−2.249150
H	1.013093	2.729887	1.163240
H	−3.401630	−1.300209	−0.801482
H	−4.868854	−2.854484	−2.062981
H	−8.030249	0.023383	−1.952185
H	−6.560941	1.591245	−0.704328
H	1.255052	−2.196567	0.042395
H	0.101259	−1.404494	−1.018568
H	1.670925	−1.501691	−2.898555
H	3.943498	−0.408178	−2.737447
H	2.000203	−3.939507	−3.155735
H	0.397361	−3.614607	−2.476882
H	1.704829	−4.185111	−1.425970
H	5.743166	3.248340	−0.098654
H	5.884658	1.637721	0.650342
H	6.797365	2.009580	−0.833617
H	2.499075	4.275927	2.042456
H	3.340793	5.469698	1.026580
H	1.761143	4.832232	0.513988
H	7.744253	−3.367427	1.160918
H	5.988650	−3.586029	1.201088
H	6.841210	−3.508145	−0.352620
H	7.439233	−1.114717	0.091502
H	6.615038	−1.198979	1.663404
H	1.333049	0.713926	3.636907
H	0.255818	−0.396277	4.536119
H	1.907836	−0.904916	4.101877
H	−2.814084	−3.030287	2.746941
H	−1.032686	−3.125042	2.888017
H	−2.053734	−3.262851	4.341069
H	−5.523008	−0.037902	3.814002
H	−3.854097	−0.231447	4.412661
H	−4.361710	1.301676	3.648641

B3LYP Energy = −2096.62066153 a.u.

(*aS*,1*S*,3*S*,1’*S*,3’*S*)-**22**, Conf S

C	−0.769446	−1.924555	−1.188515
C	−2.107963	−2.178503	−1.522410
C	−3.126797	−1.634039	−0.739133
C	−2.817498	−0.899859	0.413042
C	−1.482798	−0.696938	0.772130
C	−0.447169	−1.193577	−0.041431
O	−3.509673	−0.082663	2.609874
C	−2.354054	0.766184	2.646832
C	−1.158681	0.018981	2.066327
C	−3.954839	−0.390548	1.280961
C	−2.147048	1.185679	4.090301
C	−4.734366	0.783815	0.692707
C	0.991538	−0.996761	0.325596
C	1.695519	0.156444	−0.042163
C	3.036207	0.282083	0.345142
C	3.655245	−0.705691	1.105951
C	2.959255	−1.875563	1.445893
C	1.629624	−2.000512	1.057480
C	−4.187259	1.680264	−0.225356
C	−4.916934	2.773290	−0.688484
C	−6.205148	2.949638	−0.220713
C	−6.789415	2.080999	0.685856
C	−6.042120	1.000185	1.136836
C	1.045837	1.283226	−0.812004
C	1.781488	2.598708	−0.589897
O	3.171484	2.406993	−0.896004
C	3.844345	1.518004	−0.011632
C	1.283459	3.727882	−1.471194
F	−6.927955	4.009113	−0.670810
O	4.982767	−0.535653	1.428646
C	5.284365	−0.382946	2.826005
O	3.656595	−2.820745	2.138520
C	3.004759	−4.049776	2.454786
C	5.141697	1.176028	−0.761606
O	5.218517	0.372707	−1.658010
O	6.153447	1.941214	−0.329836
C	7.551175	2.694526	−2.201995
C	7.442469	1.791081	−0.989269
O	0.240442	−2.492201	−1.927110
C	0.417199	−1.989979	−3.262556
O	−2.417997	−2.953778	−2.617688
C	−2.369593	−4.370628	−2.371170
C	−4.981905	−1.448984	−2.255791
O	−4.449803	−1.899013	−0.996502
H	−2.552633	1.659018	2.040523
H	−0.330630	0.715626	1.919696
H	−0.810291	−0.717359	2.799529
H	−4.662568	−1.207284	1.424392
H	−1.263772	1.821602	4.178308
H	−3.010789	1.742629	4.455755
H	−2.005854	0.308764	4.725817
H	1.071030	−2.889701	1.311796
H	−3.182436	1.526431	−0.596986
H	−4.498804	3.471991	−1.401241
H	−7.804066	2.249746	1.021928
H	−6.484495	0.312526	1.847912
H	1.045302	1.065688	−1.885641
H	0.000981	1.390053	−0.514198
H	1.693683	2.890895	0.466425
H	4.114697	2.052564	0.906701
H	1.841192	4.644070	−1.273488
H	0.226054	3.919396	−1.278608
H	1.402687	3.470317	−2.525481
H	6.351801	−0.180892	2.883438
H	4.731110	0.460357	3.248266
H	5.050648	−1.293060	3.377702
H	3.745723	−4.650962	2.975768
H	2.143828	−3.888175	3.108936
H	2.684291	−4.571841	1.549782
H	8.553007	2.608084	−2.628609
H	6.829078	2.413942	−2.969007
H	7.386057	3.737365	−1.926735
H	8.165352	2.064634	−0.222899
H	7.577702	0.743823	−1.255008

H	0.653854	−0.923708	−3.241038
H	1.261863	−2.534768	−3.678467
H	−0.471891	−2.162276	−3.869395
H	−1.363940	−4.677761	−2.078006
H	−3.085997	−4.648951	−1.594730
H	−2.641209	−4.853427	−3.307791
H	−4.843109	−0.370956	−2.363378
H	−4.513018	−1.970747	−3.088318
H	−6.045951	−1.673498	−2.224645

B3LYP Energy = −2096.62059795 a.u.

(*aS*,1*S*,3*S*,1’*S*,3’*S*)-**22**, Conf T

C	0.481905	1.963981	−0.783082
C	1.769833	2.419707	−1.100770
C	2.873620	1.874394	−0.443255
C	2.694231	0.930875	0.576070
C	1.405413	0.521774	0.927784
C	0.288462	1.025198	0.234462
O	3.563432	−0.160091	2.582833
C	2.508168	−1.128327	2.501120
C	1.215642	−0.434431	2.086384
C	3.915840	0.414234	1.316292
C	2.408825	−1.805065	3.855493
C	4.788117	−0.550162	0.514894
C	−1.103218	0.609788	0.600610
C	−1.706810	−0.523448	0.041869
C	−3.015574	−0.850795	0.420966
C	−3.697528	−0.090526	1.370864
C	−3.077185	1.025269	1.954838
C	−1.794403	1.372005	1.543552
C	4.297069	−1.334007	−0.529170
C	5.115580	−2.244303	−1.194692
C	6.435381	−2.352002	−0.799814
C	6.965465	−1.590255	0.228070
C	6.130165	−0.691653	0.879955
C	−0.977638	−1.410127	−0.942525
C	−1.580496	−2.807823	−0.985252
O	−2.993930	−2.692745	−1.211368
C	−3.697008	−2.094229	−0.127241
C	−1.018797	−3.676520	−2.093973
F	7.244423	−3.232030	−1.446607
O	−4.937526	−0.542188	1.750230
C	−6.046851	0.374299	1.729333
O	−3.782140	1.688912	2.917013
C	−3.192119	2.837013	3.523350
C	−5.107889	−1.877412	−0.691872
O	−6.015481	−2.657409	−0.525600
O	−5.186332	−0.781080	−1.452696
C	−6.297370	0.726387	−2.945423
C	−6.441930	−0.549761	−2.148477
O	−0.613879	2.525063	−1.394306
C	−0.779527	2.245674	−2.794347
O	1.946500	3.395455	−2.056786
C	1.780853	4.738058	−1.566798
C	4.675469	2.142212	−2.009145
O	4.147085	2.330445	−0.683625
H	2.775579	−1.878047	1.745932
H	0.462142	−1.185638	1.840557
H	0.820289	0.121513	2.943965
H	4.538455	1.269375	1.580220
H	1.606463	−2.545565	3.851710
H	3.343712	−2.310187	4.101648
H	2.195916	−1.069858	4.634571
H	−1.303644	2.234622	1.970971
H	3.265572	−1.232133	−0.840760
H	4.740686	−2.854894	−2.005623
H	8.006429	−1.699714	0.502636
H	6.530017	−0.089832	1.687508
H	−1.022888	−0.983019	−1.950464
H	0.080032	−1.474690	−0.679229
H	−1.423656	−3.302130	−0.015941
H	−3.814638	−2.826739	0.680589
H	−1.478590	−4.665333	−2.077393
H	0.059535	−3.795204	−1.971627
H	−1.208685	−3.222223	−3.068601
H	−6.940417	−0.246809	1.728317
H	−6.038505	1.019405	2.605059
H	−6.023561	0.984872	0.824105
H	−3.924017	3.200886	4.240233
H	−2.268664	2.578963	4.048155
H	−2.987967	3.616407	2.784603
H	−7.226619	0.924788	−3.483029
H	−6.091137	1.576142	−2.293101
H	−5.490896	0.643467	−3.675234
H	−6.643243	−1.410074	−2.788019
H	−7.241413	−0.482071	−1.409737
H	−0.906321	1.172782	−2.956977
H	−1.686992	2.763064	−3.098306
H	0.068206	2.612535	−3.372854
H	0.768709	4.887909	−1.185940
H	2.510009	4.952857	−0.782194
H	1.953875	5.396884	−2.415426
H	5.697993	2.512241	−1.976124
H	4.683092	1.080829	−2.266496
H	4.099328	2.701866	−2.744213

B3LYP Energy = −2096.62058368 a.u.

(*aS*,1*S*,3*S*,1’*S*,3’*S*)-**22**, Conf U

C	0.556261	1.953561	−0.960404
C	1.861971	2.333618	−1.304469
C	2.942129	1.796520	−0.602238
C	2.723996	0.944421	0.487342
C	1.419367	0.615156	0.863528
C	0.324965	1.104363	0.126275
O	3.544652	−0.003767	2.583943
C	2.453101	−0.934352	2.582260
C	1.188867	−0.234434	2.095864
C	3.922040	0.440980	1.273011
C	2.321848	−1.479630	3.992205
C	4.748983	−0.628289	0.562122

C	−1.082825	0.772059	0.515655
C	−1.727388	−0.375992	0.038919
C	−3.044538	−0.631145	0.445926
C	−3.693420	0.222967	1.333091
C	−3.054906	1.384501	1.792857
C	−1.750547	1.639865	1.382745
C	4.224393	−1.470834	−0.417894
C	4.999036	−2.474142	−0.996535
C	6.309158	−2.615209	−0.580404
C	6.872277	−1.796358	0.384143
C	6.080352	−0.805075	0.950086
C	−1.034115	−1.358361	−0.878655
C	−1.699580	−2.727975	−0.835917
O	−3.106708	−2.564388	−1.071955
C	−3.780179	−1.876089	−0.024895
C	−1.177609	−3.687663	−1.887440
F	7.075435	−3.586995	−1.142282
O	−4.999670	−0.057763	1.662605
C	−5.253900	−0.412185	3.032530
O	−3.781677	2.192122	2.616686
C	−3.183207	3.398794	3.086563
C	−5.187456	−1.626228	−0.588269
O	−6.127379	−2.347980	−0.351174
O	−5.217355	−0.576328	−1.412298
C	−6.623121	−1.015475	−3.374585
C	−6.478034	−0.262220	−2.066762
O	−0.518447	2.502517	−1.617024
C	−0.672906	2.156884	−3.004023
O	2.080703	3.229510	−2.327594
C	1.966374	4.609337	−1.936013
C	4.751923	1.863188	−2.179115
O	4.233286	2.179160	−0.874271
H	2.694944	−1.760075	1.901452
H	0.412602	−0.978816	1.905281
H	0.805272	0.404999	2.898851
H	4.582326	1.287296	1.463093
H	1.490508	−2.185146	4.050573
H	3.234749	−1.996801	4.290328
H	2.136202	−0.667998	4.699076
H	−1.237618	2.527240	1.725044
H	3.201416	−1.342287	−0.747696
H	4.598243	−3.131046	−1.757343
H	7.904912	−1.934309	0.676728
H	6.505805	−0.158367	1.708370
H	−1.055604	−0.997211	−1.912696
H	0.018407	−1.453584	−0.603555
H	−1.564614	−3.167717	0.162615
H	−3.922613	−2.555314	0.824818
H	−1.680629	−4.652321	−1.810790
H	−0.105414	−3.845921	−1.756243
H	−1.348564	−3.286042	−2.888308
H	−6.304731	−0.689036	3.080960
H	−4.639134	−1.266183	3.329338
H	−5.061198	0.431285	3.695072
H	−3.936809	3.880363	3.704654
H	−2.295177	3.194260	3.690531
H	−2.917506	4.059081	2.257191
H	−7.543396	−0.701795	−3.872452
H	−5.785192	−0.803596	−4.040450
H	−6.676025	−2.091371	−3.206606
H	−7.292485	−0.487918	−1.380186
H	−6.431028	0.812999	−2.228597
H	−0.772764	1.075046	−3.119562
H	−1.591991	2.638140	−3.331240
H	0.167675	2.516547	−3.596886
H	2.174879	5.198518	−2.826803
H	0.957790	4.827226	−1.579224
H	2.696063	4.849239	−1.159257
H	5.792601	2.180132	−2.169287
H	4.703629	0.786431	−2.355565
H	4.206470	2.395011	−2.957062

B3LYP Energy = −2096.62053124 a.u.

(*aS*,1*S*,3*S*,1’*S*,3’*S*)-**22**, Conf V

C	0.565702	2.053524	−0.750673
C	1.875720	2.490270	−0.996842
C	2.940144	1.881925	−0.329983
C	2.698886	0.889868	0.628920
C	1.388358	0.495302	0.909076
C	0.310956	1.064754	0.203878
O	3.464174	−0.326563	2.605354
C	2.391276	−1.262542	2.431703
C	1.131937	−0.515381	2.007317
C	3.878437	0.304425	1.385394
C	2.225042	−2.007998	3.742720
C	4.758144	−0.638218	0.566552
C	−1.102618	0.666095	0.497928
C	−1.700506	−0.440964	−0.117206
C	−3.020067	−0.771999	0.221444
C	−3.718361	−0.032085	1.171319
C	−3.130343	1.095960	1.762800
C	−1.821943	1.424400	1.424544
C	4.292119	−1.347363	−0.540454
C	5.114784	−2.241165	−1.223018
C	6.413079	−2.408599	−0.780532
C	6.918184	−1.721658	0.310686
C	6.079226	−0.837932	0.977982
C	−0.954365	−1.306657	−1.107177
C	−1.570865	−2.696282	−1.205343
O	−2.976351	−2.560628	−1.467467
C	−3.705458	−1.983272	−0.390328
C	−0.987345	−3.540423	−2.321773
F	7.226033	−3.273329	−1.442856
O	−5.022096	−0.383908	1.435308
C	−5.293283	−0.887227	2.754466
O	−3.905974	1.798683	2.636616
C	−3.365819	2.981700	3.223015
C	−5.095071	−1.713764	−0.988757
O	−6.022995	−2.477891	−0.863826
O	−5.116264	−0.598292	−1.722191
C	−7.289668	0.536255	−1.554078

C	−6.347287	−0.269898	−2.425960
O	−0.489751	2.681197	−1.368469
C	−0.625181	2.461122	−2.782305
O	2.111032	3.507626	−1.894708
C	1.967961	4.828509	−1.342430
C	4.807059	2.191401	−1.811240
O	4.232925	2.315582	−0.497118
H	2.670590	−1.977303	1.647540
H	0.371307	−1.233589	1.694226
H	0.717724	0.005092	2.878277
H	4.509737	1.128690	1.717885
H	1.407942	−2.728634	3.669339
H	3.138769	−2.547360	3.995531
H	1.998738	−1.310552	4.552125
H	−1.344166	2.284039	1.872169
H	3.278057	−1.198622	−0.888330
H	4.759742	−2.793569	−2.083136
H	7.943218	−1.875867	0.621291
H	6.459494	−0.293769	1.834403
H	−0.969702	−0.850713	−2.103207
H	0.095559	−1.390649	−0.819560
H	−1.445345	−3.218489	−0.246046
H	−3.864624	−2.740007	0.387811
H	−1.456862	−4.524532	−2.344692
H	0.086050	−3.673388	−2.173534
H	−1.146367	−3.058020	−3.288348
H	−6.337753	−1.191022	2.751970
H	−4.663155	−1.753847	2.971556
H	−5.133624	−0.114813	3.506434
H	−4.153366	3.383491	3.855610
H	−2.487662	2.758648	3.834718
H	−3.102403	3.717297	2.458824
H	−8.163714	0.825577	−2.142134
H	−7.630137	−0.045642	−0.697911
H	−6.804591	1.443497	−1.191540
H	−6.011959	0.304952	−3.287242
H	−6.809669	−1.194718	−2.767926
H	0.260578	2.799257	−3.320261
H	−0.807389	1.403740	−2.988417
H	−1.491301	3.040538	−3.094942
H	0.949313	4.988587	−0.983868
H	2.679060	4.982821	−0.527600
H	2.184456	5.523227	−2.151519
H	5.836668	2.530148	−1.717904
H	4.797791	1.147362	−2.131717
H	4.274072	2.809162	−2.532045

B3LYP Energy = −2096.62051940 a.u.

(*aS*,1*S*,3*S*,1'*S*,3'*S*)-**22**, Conf W

C	0.754381	1.992138	−1.022161
C	2.090282	2.321869	−1.295212
C	3.110452	1.735211	−0.544407
C	2.803636	0.880392	0.521642
C	1.469248	0.599806	0.825929
C	0.434703	1.141727	0.040622
O	3.474637	−0.120379	2.647725
C	2.350083	−1.007661	2.575555
C	1.140986	−0.253605	2.032677
C	3.938735	0.327416	1.365681
C	2.123746	−1.563603	3.969222
C	4.771363	−0.758108	0.687663
C	−1.003275	0.863939	0.355376
C	−1.673188	−0.245532	−0.174339
C	−3.015917	−0.451570	0.168844
C	−3.668199	0.407955	1.047765
C	−3.004645	1.533826	1.557806
C	−1.674211	1.742598	1.208906
C	6.088841	−0.949354	1.113799
C	6.882728	−1.956263	0.578772
C	6.335196	−2.776507	−0.393338
C	5.038846	−2.621941	−0.846628
C	4.262506	−1.602750	−0.299234
C	−0.984735	−1.247173	−1.072995
C	−1.695477	−2.594314	−1.042654
O	−3.083255	−2.389976	−1.350853
C	−3.791553	−1.641222	−0.369163
C	−1.157059	−3.586656	−2.054998
F	7.103321	−3.763494	−0.925448
O	−4.995491	0.164739	1.321613
C	−5.312787	−0.186508	2.679090
O	−3.732296	2.355349	2.366779
C	−3.108313	3.531305	2.879356
C	−5.079822	−1.222153	−1.095988
O	−5.158424	−0.294352	−1.862650
O	−6.074278	−2.078348	−0.824803
C	−8.243014	−0.929828	−0.722309
C	−7.346870	−1.872517	−1.501319
O	−0.262783	2.590976	−1.724483
C	−0.366945	2.258899	−3.119694
O	2.400041	3.214986	−2.296747
C	2.286915	4.596911	−1.911632
C	5.002755	1.734495	−2.020255
O	4.427324	2.071973	−0.744996
H	2.595867	−1.834690	1.897737
H	0.348254	−0.964829	1.790404
H	0.739870	0.390747	2.823076
H	4.613780	1.149582	1.603913
H	1.264936	−2.237951	3.974574
H	2.999740	−2.118310	4.307879
H	1.931270	−0.753712	4.676239
H	−1.141467	2.601494	1.591337
H	6.502377	−0.300757	1.877043
H	7.905118	−2.104813	0.900645
H	4.650459	−3.280357	−1.612546
H	3.250974	−1.464073	−0.658693
H	−0.970135	−0.890109	−2.108447
H	0.056845	−1.372362	−0.770096
H	−1.621649	−3.022054	−0.032641
H	−4.070155	−2.300481	0.461547
H	−1.696816	−4.532228	−1.990608
H	−0.098835	−3.780152	−1.869189

H	−1.265297	−3.194108	−3.068069
H	−6.374788	−0.421958	2.688469
H	−4.742554	−1.065490	2.992049
H	−5.112217	0.644670	3.354404
H	−3.868156	4.033230	3.473129
H	−2.254746	3.284153	3.516223
H	−2.783425	4.191799	2.071598
H	−9.209729	−0.854186	−1.225278
H	−8.413500	−1.301308	0.289409
H	−7.809564	0.068429	−0.662931
H	−7.150959	−1.500170	−2.505686
H	−7.776867	−2.870469	−1.564675
H	−1.252430	2.774792	−3.484248
H	0.512537	2.590630	−3.671261
H	−0.500840	1.182242	−3.246991
H	2.961868	4.819659	−1.082037
H	2.574735	5.181636	−2.783039
H	1.259731	4.837188	−1.630760
H	4.935574	0.658110	−2.193275
H	4.510723	2.275496	−2.827043
H	6.049434	2.025081	−1.960083

B3LYP Energy = −2096.62044233 a.u.

(*aR*,1*S*,3*S*,1’*S*,3’*S*)-**22**, Conf A

C	−0.973719	2.046342	0.660274
C	−2.251156	2.488750	0.289729
C	−3.103403	1.623626	−0.398024
C	−2.665612	0.345211	−0.766711
C	−1.373737	−0.073000	−0.436591
C	−0.522548	0.775842	0.295956
O	−2.887610	−1.585173	−2.248116
C	−2.010131	−2.331506	−1.391858
C	−0.883333	−1.426563	−0.906618
C	−3.599963	−0.541208	−1.571292
C	−1.510846	−3.524152	−2.185907
C	−4.772222	−1.118183	−0.780173
C	0.860146	0.346308	0.678077
C	1.954351	0.595670	−0.158129
C	3.221140	0.146761	0.235290
C	3.402620	−0.501703	1.453480
C	2.303798	−0.766566	2.283781
C	1.042589	−0.332952	1.885151
C	−4.729614	−1.335542	0.597203
C	−5.800720	−1.922069	1.268128
C	−6.916613	−2.282319	0.537054
C	−7.003392	−2.080283	−0.830147
C	−5.921868	−1.497579	−1.479112
C	1.811709	1.367472	−1.449647
C	3.142996	1.964452	−1.886662
O	4.131648	0.921315	−1.916754
C	4.441965	0.385957	−0.635975
C	3.103556	2.580342	−3.271650
F	−7.969648	−2.849552	1.183046
O	4.667508	−0.945645	1.768682
C	5.304458	−0.304689	2.886868
O	2.562368	−1.448129	3.436505
C	1.468253	−1.791793	4.283718
C	5.192770	−0.915386	−0.958115
O	4.661447	−1.949784	−1.279379
O	6.517224	−0.727338	−0.908653
C	8.796191	−1.424567	−1.148635
C	7.353900	−1.863168	−1.260668
O	−0.111739	2.913739	1.291612
C	−0.419937	3.213106	2.662699
O	−2.666085	3.762163	0.610658
C	−2.234569	4.775690	−0.314704
C	−5.307368	2.399717	0.164588
O	−4.339115	2.034579	−0.835658
H	−2.583575	−2.691502	−0.528529
H	−0.333016	−1.928219	−0.108389
H	−0.169360	−1.282225	−1.725271
H	−4.019958	0.057192	−2.380018
H	−0.816096	−4.115591	−1.586307
H	−2.343160	−4.163887	−2.482388
H	−0.990843	−3.191309	−3.086646
H	0.180829	−0.525793	2.508190
H	−3.856997	−1.038145	1.164266
H	−5.773752	−2.093916	2.336183
H	−7.897846	−2.370559	−1.365569
H	−5.973217	−1.335027	−2.549209
H	1.443147	0.716133	−2.249927
H	1.077910	2.165975	−1.326315
H	3.458355	2.721588	−1.155085
H	5.135470	1.058237	−0.116613
H	4.076627	2.996536	−3.535568
H	2.363542	3.382076	−3.307186
H	2.834309	1.827972	−4.015701
H	6.313837	−0.708176	2.930094
H	5.351053	0.777038	2.734733
H	4.777484	−0.525643	3.814820
H	1.900965	−2.338946	5.117548
H	0.959091	−0.900692	4.660708
H	0.750167	−2.431647	3.764271
H	9.450096	−2.259180	−1.408782
H	9.009104	−0.598770	−1.829041
H	9.033484	−1.109280	−0.131491
H	7.121768	−2.684265	−0.581277
H	7.100587	−2.177338	−2.273930
H	0.333583	3.924545	2.994605
H	−1.411822	3.656988	2.753798
H	−0.357442	2.308929	3.273901
H	−2.645164	5.715923	0.048007
H	−1.144861	4.836360	−0.339634
H	−2.619985	4.570088	−1.316000
H	−4.975297	3.266003	0.734518
H	−6.219187	2.637892	−0.378977
H	−5.496309	1.558686	0.835339

B3LYP Energy = −2096.62272909 a.u.

(*aR*,1*S*,3*S*,1’*S*,3’*S*)-**22**, Conf B

C	−1.014459	2.036786	0.793859
C	−2.291899	2.480161	0.423766
C	−3.117788	1.642096	−0.326987
C	−2.652620	0.393250	−0.758303
C	−1.360477	−0.023078	−0.427375
C	−0.536210	0.795698	0.367582
O	−2.813277	−1.451609	−2.351617
C	−1.940764	−2.232738	−1.521939
C	−0.838803	−1.340558	−0.961815
C	−3.557033	−0.461271	−1.628875
C	−1.406483	−3.369865	−2.372532
C	−4.731086	−1.104684	−0.893352
C	0.845546	0.363673	0.749511
C	1.949693	0.663564	−0.055927
C	3.211567	0.185860	0.318997
C	3.384533	−0.534379	1.500544
C	2.282859	−0.791685	2.331907
C	1.022101	−0.359781	1.930227
C	−4.713978	−1.384983	0.473041
C	−5.784260	−2.028996	1.090501
C	−6.873446	−2.382550	0.317132
C	−6.934568	−2.118872	−1.040973
C	−5.854370	−1.479343	−1.636364
C	1.813495	1.508381	−1.302198
C	3.144865	2.131167	−1.699036
O	4.136425	1.093264	−1.774151
C	4.440005	0.504382	−0.514078
C	3.112488	2.817890	−3.050475
F	−7.925699	−3.005613	0.910928
O	4.669939	−0.883695	1.843033
C	4.945898	−2.276463	2.089834
O	2.535110	−1.440556	3.506382
C	1.443709	−1.720466	4.379870
C	5.248286	−0.744085	−0.893194
O	4.768714	−1.806573	−1.209236
O	6.558577	−0.474412	−0.902635
C	8.860674	−0.993391	−1.307665
C	7.449200	−1.532681	−1.351368
O	−0.178354	2.880249	1.488910
C	−0.515052	3.096734	2.868837
O	−2.734665	3.726190	0.808140
C	−2.283151	4.801982	−0.033804
C	−5.342658	2.352375	0.238750
O	−4.352687	2.056872	−0.763212
H	−2.525037	−2.650026	−0.692496
H	−0.298726	−1.878049	−0.179869
H	−0.108919	−1.139743	−1.754219
H	−3.974722	0.175170	−2.409283
H	−0.716523	−3.986069	−1.792631
H	−2.222578	−4.002168	−2.724778
H	−0.871417	−2.977661	−3.240035
H	0.158714	−0.565912	2.546517
H	−3.862442	−1.092084	1.073557
H	−5.777090	−2.249705	2.149863
H	−7.808958	−2.405820	−1.610268
H	−5.885805	−1.267946	−2.698651
H	1.447007	0.904375	−2.139851
H	1.078479	2.297925	−1.135949
H	3.453435	2.850064	−0.927242
H	5.098705	1.173433	0.052901
H	4.084891	3.253051	−3.284574
H	2.367822	3.616105	−3.050062
H	2.853347	2.103910	−3.834861
H	4.615000	−2.882614	1.244399
H	6.027232	−2.347529	2.188121
H	4.465383	−2.613953	3.005318
H	1.875771	−2.229030	5.238164
H	0.954592	−0.801091	4.712378
H	0.708750	−2.374704	3.903389
H	9.555102	−1.767389	−1.640147
H	8.970658	−0.129966	−1.965247
H	9.137133	−0.698061	−0.294492
H	7.320745	−2.394022	−0.694768
H	7.154691	−1.826299	−2.359654
H	−0.457245	2.158622	3.426907
H	0.226712	3.792029	3.256531
H	−1.512035	3.527770	2.966357
H	−1.194132	4.874269	−0.016814
H	−2.631861	4.658969	−1.059219
H	−2.716895	5.712487	0.374856
H	−5.038325	3.197569	0.853988
H	−6.252272	2.595581	−0.306269
H	−5.521043	1.476424	0.866272

B3LYP Energy = −2096.62266221 a.u.

(*aR*,1*S*,3*S*,1’*S*,3’*S*)-**22**, Conf C

C	−0.845211	2.195211	0.398354
C	−2.135554	2.567048	−0.003749
C	−2.980332	1.608937	−0.566503
C	−2.522322	0.303148	−0.784123
C	−1.217872	−0.048789	−0.426561
C	−0.374199	0.897977	0.184034
O	−2.727490	−1.801065	−2.009596
C	−1.822690	−2.418945	−1.083079
C	−0.706819	−1.440515	−0.734265
C	−3.451270	−0.694118	−1.454650
C	−1.312857	−3.692366	−1.731606
C	−4.597049	−1.190396	−0.575391
C	1.019130	0.544219	0.604281
C	2.106004	0.713757	−0.260901
C	3.386187	0.355743	0.183002
C	3.582719	−0.128341	1.473622
C	2.490481	−0.318665	2.332641
C	1.218605	0.028483	1.887091
C	−4.530811	−1.220788	0.817579
C	−5.576657	−1.739775	1.578054
C	−6.691257	−2.223204	0.919676
C	−6.801065	−2.207616	−0.460681
C	−5.744671	−1.688823	−1.198999
C	1.932169	1.298087	−1.644438

C	3.245199	1.843205	−2.191028
O	4.257845	0.830965	−2.063196
C	4.600214	0.548515	−0.712434
C	3.177355	2.226916	−3.656193
F	−7.719754	−2.726055	1.652482
O	4.857082	−0.495459	1.841975
C	5.481525	0.303298	2.861690
O	2.766392	−0.846133	3.559846
C	1.684908	−1.074623	4.460569
C	5.539044	−0.663040	−0.805798
O	6.743818	−0.564762	−0.802664
O	4.873820	−1.808415	−0.965878
C	4.714419	−4.183062	−1.227068
C	5.668476	−3.016456	−1.110962
O	0.007117	3.145467	0.912023
C	−0.289495	3.597387	2.243107
O	−2.571080	3.861828	0.168174
C	−2.172463	4.765552	−0.877981
C	−5.189891	2.407713	−0.062198
O	−4.230964	1.940210	−1.028346
H	−2.374741	−2.679734	−0.171388
H	−0.133364	−1.828085	0.109961
H	−0.009646	−1.385144	−1.577914
H	−3.897208	−0.209601	−2.323502
H	−0.602802	−4.196162	−1.072669
H	−2.138530	−4.375108	−1.936454
H	−0.808460	−3.465465	−2.673317
H	0.362011	−0.105475	2.532395
H	−3.659459	−0.827211	1.324899
H	−5.531709	−1.766254	2.658925
H	−7.693935	−2.589507	−0.938070
H	−5.814496	−1.671677	−2.280219
H	1.555022	0.536268	−2.336150
H	1.189470	2.097549	−1.620147
H	3.551465	2.714903	−1.595731
H	5.217117	1.364280	−0.314902
H	4.136279	2.620979	−3.995058
H	2.414382	2.992192	−3.811084
H	2.924071	1.357710	−4.266663
H	6.502773	−0.061623	2.946017
H	5.494514	1.356864	2.570392
H	4.966338	0.188577	3.815254
H	2.130807	−1.501878	5.355264
H	1.176908	−0.141519	4.718734
H	0.962460	−1.781014	4.043261
H	5.284561	−5.107488	−1.338343
H	4.092137	−4.269873	−0.335333
H	4.065622	−4.074913	−2.097440
H	6.296246	−2.912730	−1.997070
H	6.318748	−3.105928	−0.240014
H	0.461465	4.347381	2.483042
H	−1.283932	4.042040	2.294171
H	−0.212716	2.769011	2.952259
H	−2.592643	5.735726	−0.620383
H	−1.084579	4.839362	−0.930791
H	−2.571470	4.438098	−1.840805
H	−4.868930	3.346561	0.386132
H	−6.117146	2.553884	−0.612087
H	−5.344847	1.654348	0.713230

B3LYP Energy = −2096.62262112 a.u.

(*aR*,1*S*,3*S*,1'*S*,3'*S*)-**22**, Conf D

C	−1.044216	2.171366	0.251043
C	−2.350185	2.469385	−0.159959
C	−3.141628	1.462303	−0.714551
C	−2.662801	0.151138	−0.802958
C	−1.363098	−0.145518	−0.381633
C	−0.538214	0.872343	0.133361
O	−2.824534	−2.071248	−1.797788
C	−1.939637	−2.578550	−0.786852
C	−0.837318	−1.558971	−0.521963
C	−3.571162	−0.923963	−1.371943
C	−1.406165	−3.911091	−1.278047
C	−4.719292	−1.342776	−0.454022
C	0.859699	0.581168	0.584816
C	1.924970	0.516980	−0.321616
C	3.206914	0.224103	0.162956
C	3.430060	0.031788	1.522907
C	2.358934	0.075043	2.428097
C	1.085526	0.356985	1.945217
C	−4.711874	−1.134740	0.924379
C	−5.760265	−1.581134	1.726195
C	−6.816449	−2.236466	1.123098
C	−6.866400	−2.460560	−0.242527
C	−5.809124	−2.008655	−1.022258
C	1.745005	0.789383	−1.797544
C	3.057021	1.213202	−2.445405
O	4.057747	0.225421	−2.149321
C	4.401901	0.146066	−0.771056
C	2.976143	1.326229	−3.955336
F	−7.847261	−2.671624	1.895936
O	4.706699	−0.284914	1.929813
C	5.371631	0.688229	2.753714
O	2.656489	−0.173637	3.735420
C	1.594696	−0.165397	4.687798
C	5.143060	−1.195280	−0.657126
O	4.602053	−2.272012	−0.596806
O	6.468277	−1.015016	−0.708294
C	8.739316	−1.773999	−0.754743
C	7.294475	−2.210913	−0.673184
O	−0.280137	3.140967	0.856967
C	0.179449	4.209354	0.013640
O	−2.836118	3.751545	−0.032789
C	−3.580455	3.983561	1.176115
C	−4.596658	2.595804	−2.231481
O	−4.436018	1.714674	−1.104936
H	−2.514410	−2.739732	0.133469
H	−0.283637	−1.844574	0.374873
H	−0.120187	−1.597460	−1.348658
H	−4.016652	−0.542845	−2.291725
H	−0.714059	−4.334965	−0.547798

H	−2.222671	−4.617872	−1.431738
H	−0.873366	−3.784195	−2.222929
H	0.246454	0.406658	2.624085
H	−3.885702	−0.610780	1.387122
H	−5.761119	−1.421822	2.796528
H	−7.714914	−2.972232	−0.677469
H	−5.831851	−2.178819	−2.092189
H	1.382361	−0.105825	−2.314446
H	0.992553	1.565404	−1.949574
H	3.379762	2.174505	−2.020820
H	5.109706	0.946622	−0.524963
H	3.937776	1.631005	−4.370094
H	2.226885	2.067826	−4.238812
H	2.696914	0.366803	−4.395289
H	4.867617	0.796127	3.713652
H	6.381444	0.313570	2.906662
H	5.414887	1.655716	2.246254
H	2.055731	−0.393610	5.645533
H	1.113928	0.814968	4.740512
H	0.847278	−0.928103	4.454566
H	9.386053	−2.653167	−0.729681
H	8.933931	−1.233026	−1.681967
H	9.003712	−1.131636	0.086555
H	7.080901	−2.748158	0.251728
H	7.013242	−2.849759	−1.511240
H	0.827699	3.822335	−0.776757
H	−0.657749	4.752945	−0.424074
H	0.754744	4.873518	0.655488
H	−4.458454	3.335678	1.220320
H	−2.950102	3.817057	2.052588
H	−3.895773	5.024654	1.146840
H	−5.664824	2.629644	−2.435106
H	−4.229203	3.595857	−2.003497
H	−4.072374	2.197572	−3.104305

B3LYP Energy = −2096.62196716 a.u.

(*aR*,1*S*,3*S*,1'*S*,3'*S*)-**22**, Conf E

C	−1.072958	2.141353	0.503060
C	−2.378268	2.469058	0.112652
C	−3.152836	1.521136	−0.557563
C	−2.657981	0.232844	−0.784600
C	−1.359220	−0.094651	−0.383270
C	−0.549872	0.869191	0.247367
O	−2.781643	−1.869547	−2.018363
C	−1.906147	−2.474336	−1.054202
C	−0.817330	−1.480284	−0.666163
C	−3.546568	−0.781239	−1.483009
C	−1.355364	−3.743700	−1.676151
C	−4.706039	−1.306780	−0.637777
C	0.848181	0.546563	0.675674
C	1.920637	0.591872	−0.223081
C	3.201344	0.251663	0.233217
C	3.421272	−0.090629	1.566581
C	2.350319	−0.094460	2.475154
C	1.074252	0.203898	2.010102
C	−5.789816	−1.895800	−1.295353
C	−6.856063	−2.437691	−0.588638
C	−6.821046	−2.382965	0.794532
C	−5.770981	−1.809079	1.485170
C	−4.713524	−1.269595	0.755872
C	1.741448	1.021786	−1.661751
C	3.046791	1.540067	−2.250437
O	4.064528	0.544456	−2.056179
C	4.404331	0.334475	−0.689545
C	2.971250	1.818636	−3.738892
F	−7.860655	−2.907632	1.496736
O	4.721939	−0.316276	1.951722
C	5.027916	−1.569673	2.593472
O	2.648882	−0.369935	3.778245
C	1.591961	−0.365003	4.735845
C	5.227720	−0.960335	−0.720334
O	4.761819	−2.074530	−0.713511
O	6.532600	−0.687884	−0.833198
C	8.840252	−1.271770	−1.091631
C	7.435522	−1.818418	−0.977934
O	−0.330632	3.047263	1.223490
C	0.146466	4.196020	0.505144
O	−2.878669	3.725500	0.370970
C	−3.632937	3.821816	1.591867
C	−4.603198	2.797781	−1.960421
O	−4.445677	1.802496	−0.932803
H	−2.492250	−2.737111	−0.164797
H	−0.274410	−1.854690	0.204334
H	−0.087497	−1.425220	−1.480654
H	−3.979800	−0.305580	−2.363742
H	−0.663397	−4.234038	−0.988563
H	−2.163439	−4.438911	−1.907709
H	−0.818828	−3.515170	−2.599542
H	0.236476	0.203013	2.692226
H	−5.801353	−1.932523	−2.378355
H	−7.700223	−2.889972	−1.092438
H	−5.783887	−1.782375	2.566876
H	−3.891936	−0.807472	1.287832
H	1.398403	0.182256	−2.276343
H	0.973157	1.793967	−1.730724
H	3.348801	2.453541	−1.719016
H	5.062415	1.143472	−0.349792
H	3.929316	2.184315	−4.110383
H	2.210317	2.574288	−3.943296
H	2.711182	0.909480	−4.284744
H	4.669786	−2.403040	1.985987
H	6.113391	−1.607129	2.659320
H	4.592185	−1.616203	3.589029
H	2.057870	−0.598260	5.690061
H	1.114655	0.616424	4.795323
H	0.840712	−1.124603	4.503982
H	9.543523	−2.099496	−1.201609
H	8.938821	−0.621379	−1.961857
H	9.114603	−0.706068	−0.200053
H	7.319425	−2.467348	−0.109087
H	7.142850	−2.381500	−1.865055

H	0.840953	3.894369	−0.283147
H	−0.680066	4.763968	0.077805
H	0.675758	4.808005	1.232685
H	−4.500127	3.158649	1.563595
H	−3.004541	3.577965	2.451464
H	−3.965759	4.855216	1.665862
H	−5.669952	2.847503	−2.168135
H	−4.243637	3.769788	−1.624225
H	−4.069876	2.497708	−2.866455

B3LYP Energy = −2096.62184912 a.u.

(*aR*,1*S*,3*S*,1'*S*,3'*S*)-**22**, Conf F

C	−0.898546	2.259515	0.227557
C	−2.216225	2.536382	−0.160586
C	−3.004776	1.513638	−0.690357
C	−2.511566	0.207475	−0.773458
C	−1.201082	−0.068491	−0.371936
C	−0.379224	0.965077	0.115331
O	−2.661209	−2.026985	−1.742949
C	−1.756016	−2.512095	−0.739787
C	−0.661096	−1.477394	−0.504298
C	−3.415406	−0.883652	−1.318726
C	−1.215934	−3.845101	−1.222640
C	−4.546741	−1.306688	−0.382399
C	1.030715	0.697842	0.544361
C	2.082204	0.650076	−0.379087
C	3.378980	0.387326	0.086016
C	3.625375	0.203330	1.443202
C	2.568029	0.230001	2.365162
C	1.281923	0.485953	1.902219
C	−5.639836	−1.982953	−0.931706
C	−6.681625	−2.440211	−0.134440
C	−6.612782	−2.210765	1.229470
C	−5.552823	−1.544817	1.814149
C	−4.520195	−1.093293	0.994990
C	1.864113	0.901365	−1.854374
C	3.154723	1.318046	−2.547636
O	4.174972	0.354079	−2.240268
C	4.560411	0.357653	−0.871103
C	3.036714	1.380541	−4.057888
F	−7.628129	−2.651236	2.019369
O	4.911491	−0.092503	1.834020
C	5.584752	0.903425	2.623456
O	2.891967	−0.006854	3.668349
C	1.848472	0.002907	4.640711
C	5.475880	−0.868072	−0.735353
O	6.681204	−0.800902	−0.794360
O	4.786021	−2.005161	−0.626983
C	4.576722	−4.383734	−0.445842
C	5.554849	−3.236144	−0.550170
O	−0.133354	3.243743	0.807195
C	0.285346	4.316701	−0.051851
O	−2.715907	3.813331	−0.035396
C	−3.463015	4.039797	1.173014
C	−4.503632	2.616091	−2.188920
O	−4.309103	1.745212	−1.059618
H	−2.314577	−2.667960	0.191373
H	−0.091110	−1.745806	0.387692
H	0.043578	−1.517597	−1.341747
H	−3.876769	−0.517088	−2.236555
H	−0.508998	−4.253003	−0.497425
H	−2.027116	−4.562134	−1.355329
H	−0.699046	−3.724390	−2.177183
H	0.453253	0.525354	2.594424
H	−5.677677	−2.156950	−2.000601
H	−7.532670	−2.959891	−0.554675
H	−5.539007	−1.381238	2.883748
H	−3.691384	−0.560997	1.443286
H	1.487771	−0.002857	−2.345461
H	1.105168	1.672621	−1.998110
H	3.476008	2.296600	−2.163096
H	5.203552	1.225143	−0.677036
H	3.982851	1.688658	−4.504390
H	2.267405	2.099076	−4.347122
H	2.765097	0.402678	−4.460590
H	5.102100	1.022548	3.593206
H	6.602335	0.543250	2.757623
H	5.604226	1.861653	2.097710
H	2.329941	−0.207958	5.592339
H	1.358595	0.978912	4.689705
H	1.104349	−0.770241	4.432076
H	5.127371	−5.324701	−0.387944
H	3.959645	−4.293033	0.449148
H	3.923618	−4.422914	−1.318798
H	6.177909	−3.310259	−1.442348
H	6.209619	−3.177562	0.320168
H	0.912332	3.936441	−0.862362
H	−0.572264	4.848980	−0.462788
H	0.872917	4.988529	0.570530
H	−4.335574	3.384727	1.217310
H	−2.831678	3.879138	2.049906
H	−3.786707	5.078257	1.142819
H	−5.575705	2.631871	−2.373188
H	−4.147786	3.623302	−1.974625
H	−3.989192	2.219897	−3.068429

B3LYP Energy = −2096.62184399 a.u.

(*aR*,1*S*,3*S*,1'*S*,3'*S*)-**22**, Conf G

C	−1.000639	2.077041	0.709090
C	−2.273712	2.498828	0.301090
C	−3.087364	1.623924	−0.420351
C	−2.613498	0.357751	−0.786305
C	−1.325193	−0.039238	−0.417801
C	−0.514500	0.817912	0.350110
O	−2.747157	−1.559697	−2.294139
C	−1.885209	−2.298883	−1.416328
C	−0.792990	−1.378265	−0.883915
C	−3.503180	−0.538496	−1.629574
C	−1.337090	−3.473440	−2.205010
C	−4.687525	−1.149932	−0.883281

C	0.860384	0.407402	0.778221
C	1.982079	0.682160	−0.011742
C	3.237104	0.228267	0.412696
C	3.385032	−0.442719	1.626411
C	2.264647	−0.675794	2.439439
C	1.011853	−0.267878	1.990650
C	−5.798554	−1.560799	−1.625659
C	−6.886454	−2.175892	−1.018683
C	−6.845763	−2.378028	0.350690
C	−5.769480	−1.986664	1.123876
C	−4.691061	−1.368301	0.494439
C	1.870723	1.474113	−1.294732
C	3.208214	2.086046	−1.688367
O	4.206243	1.051780	−1.694345
C	4.483565	0.522888	−0.402243
C	3.203981	2.711521	−3.069545
F	−7.905566	−2.976963	0.955700
O	4.663727	−0.766875	2.015138
C	4.945981	−2.145577	2.324561
O	2.491433	−1.277904	3.643722
C	1.380974	−1.526105	4.502613
C	5.312742	−0.732965	−0.704464
O	4.847430	−1.810290	−0.989175
O	6.621494	−0.449695	−0.677424
C	7.802060	−1.512484	−2.547536
C	7.554020	−1.502862	−1.051836
O	−0.175201	2.955220	1.372781
C	−0.530554	3.240332	2.735434
O	−2.722155	3.761647	0.618639
C	−2.286425	4.789527	−0.288733
C	−5.321642	2.354670	0.079323
O	−4.316580	2.015038	−0.893241
H	−2.480258	−2.678319	−0.576341
H	−0.260932	−1.877390	−0.071696
H	−0.054149	−1.211434	−1.675958
H	−3.909753	0.060066	−2.444992
H	−0.654106	−4.060132	−1.587359
H	−2.146997	−4.123696	−2.538308
H	−0.790964	−3.121155	−3.082636
H	0.134992	−0.455036	2.593893
H	−5.814064	−1.397893	−2.696816
H	−7.751195	−2.490886	−1.587884
H	−5.778061	−2.159364	2.192107
H	−3.849415	−1.046993	1.094289
H	1.525305	0.833660	−2.114048
H	1.129292	2.266464	−1.177713
H	3.495566	2.840194	−0.942387
H	5.120778	1.223830	0.150452
H	4.180579	3.138254	−3.301675
H	2.458120	3.506971	−3.121254
H	2.963319	1.962376	−3.826636
H	6.026807	−2.205000	2.435581
H	4.458887	−2.446182	3.249413
H	4.627110	−2.790269	1.503403
H	0.658354	−2.201013	4.036161
H	1.794419	−1.998663	5.390157
H	0.882527	−0.595613	4.786665
H	8.558533	−2.264249	−2.783485
H	6.893464	−1.759214	−3.097280
H	8.166891	−0.542151	−2.887498
H	8.460308	−1.262368	−0.499177
H	7.161697	−2.457191	−0.703973
H	−1.530734	3.670297	2.798175
H	−0.475020	2.332531	3.341812
H	0.202726	3.958862	3.095955
H	−1.197716	4.869899	−0.286207
H	−2.643283	4.584609	−1.300721
H	−2.722719	5.719560	0.070244
H	−5.026738	3.227043	0.660264
H	−6.222912	2.572182	−0.489871
H	−5.509577	1.507936	0.743149

B3LYP Energy = −2096.62177992 a.u.

(*aR*,1*S*,3*S*,1'*S*,3'*S*)-**22**, Conf H

C	−0.960933	2.097715	0.500018
C	−2.233806	2.506099	0.078443
C	−3.075493	1.586693	−0.549364
C	−2.630736	0.284453	−0.810609
C	−1.342510	−0.102913	−0.431426
C	−0.503336	0.803272	0.243749
O	−2.830044	−1.759821	−2.134674
C	−1.962223	−2.432282	−1.210178
C	−0.843301	−1.488041	−0.785372
C	−3.553101	−0.666498	−1.553488
C	−1.451364	−3.683729	−1.899313
C	−4.735040	−1.181032	−0.734461
C	0.871492	0.408328	0.687067
C	1.984340	0.590614	−0.141526
C	3.243533	0.186457	0.319507
C	3.398146	−0.352375	1.593889
C	2.279910	−0.554401	2.416023
C	1.027152	−0.164489	1.951588
C	−4.710519	−1.287170	0.656245
C	−5.789436	−1.821113	1.357865
C	−6.894700	−2.242290	0.643373
C	−6.963615	−2.150487	−0.736608
C	−5.874641	−1.618335	−1.415837
C	1.866679	1.243092	−1.499567
C	3.204456	1.806491	−1.960905
O	4.199303	0.772714	−1.870905
C	4.484678	0.367411	−0.537861
C	3.193711	2.290568	−3.397788
F	−7.955139	−2.759133	1.318787
O	4.657390	−0.755192	1.978197
C	5.258943	−0.020939	3.057938
O	2.512090	−1.134631	3.628376
C	1.400040	−1.395267	4.481859
C	5.271184	−0.940902	−0.714563
O	4.768754	−2.008925	−0.963297
O	6.589258	−0.716886	−0.622748
C	7.844344	−1.986637	−2.305309

C	7.483567	−1.844487	−0.839540
O	−0.109106	3.012284	1.075848
C	−0.440031	3.419096	2.413368
O	−2.653967	3.800016	0.292261
C	−2.234326	4.732381	−0.719712
C	−5.287794	2.393779	−0.073098
O	−4.306749	1.958984	−1.032220
H	−2.545624	−2.724293	−0.327892
H	−0.301134	−1.922166	0.056820
H	−0.120077	−1.407508	−1.604623
H	−3.963196	−0.136042	−2.413144
H	−0.765983	−4.225082	−1.244134
H	−2.279792	−4.345433	−2.155726
H	−0.918002	−3.423854	−2.816137
H	0.151406	−0.309240	2.568228
H	−3.845985	−0.942989	1.208943
H	−5.776483	−1.906557	2.436544
H	−7.850225	−2.485391	−1.258844
H	−5.912175	−1.542225	−2.496110
H	1.517826	0.519714	−2.245118
H	1.126533	2.044422	−1.464371
H	3.498971	2.630845	−1.296299
H	5.148959	1.101985	−0.066945
H	4.170031	2.689214	−3.676454
H	2.449259	3.078859	−3.524828
H	2.947770	1.469980	−4.074799
H	5.296383	1.046323	2.823584
H	4.712305	−0.176757	3.987675
H	6.271731	−0.405902	3.155803
H	1.813548	−1.865760	5.370413
H	0.889270	−0.471088	4.765410
H	0.688030	−2.076881	4.009261
H	8.567335	−2.796901	−2.423156
H	6.966347	−2.223384	−2.906703
H	8.294815	−1.068713	−2.686081
H	8.358003	−1.610069	−0.235163
H	7.009150	−2.745192	−0.453240
H	−0.400671	2.563479	3.092643
H	0.315969	4.145355	2.705060
H	−1.427622	3.879934	2.450436
H	−2.630012	4.443326	−1.696086
H	−2.642255	5.699615	−0.432926
H	−1.144976	4.791607	−0.761670
H	−6.195060	2.583786	−0.642610
H	−5.478943	1.605234	0.658003
H	−4.966235	3.303221	0.432002

B3LYP Energy = −2096.62172130 a.u.

(*aR*,1*S*,3*S*,1’*S*,3’*S*)-**22**, Conf I

C	−0.884471	2.193348	0.296974
C	−2.162853	2.540148	−0.161867
C	−2.993445	1.549229	−0.687765
C	−2.530492	0.232910	−0.813773
C	−1.236786	−0.096235	−0.401006
C	−0.409015	0.886112	0.174559
O	−2.705487	−1.941148	−1.916364
C	−1.824599	−2.501787	−0.932335
C	−0.718042	−1.503003	−0.613031
C	−3.442530	−0.803471	−1.447213
C	−1.297351	−3.810263	−1.490979
C	−4.610389	−1.246184	−0.568497
C	0.973067	0.556681	0.647841
C	2.081517	0.691088	−0.195848
C	3.349034	0.345672	0.291491
C	3.512751	−0.090621	1.603709
C	2.399622	−0.241497	2.443604
C	1.139984	0.091473	1.954326
C	−4.574172	−1.202370	0.825365
C	−5.639468	−1.674234	1.589433
C	−6.742901	−2.186109	0.933671
C	−6.822949	−2.244065	−0.447532
C	−5.747473	−1.771279	−1.189348
C	1.944966	1.227362	−1.602523
C	3.272846	1.756065	−2.128717
O	4.279927	0.746428	−1.947311
C	4.585479	0.493845	−0.581674
C	3.244424	2.100797	−3.604807
F	−7.790353	−2.642990	1.669820
O	4.775478	−0.452872	2.013901
C	5.383972	0.376684	3.018475
O	2.643557	−0.721127	3.697046
C	1.539649	−0.910791	4.579344
C	5.500893	−0.739051	−0.623715
O	6.706594	−0.664264	−0.588621
O	4.808827	−1.871321	−0.773586
C	5.936244	−3.401267	−2.327778
C	5.556609	−3.113960	−0.888324
O	−0.045426	3.174086	0.774541
C	−0.371498	3.695494	2.072836
O	−2.601310	3.842758	−0.081067
C	−2.154025	4.687201	−1.156844
C	−5.212881	2.384480	−0.294855
O	−4.231861	1.847970	−1.201108
H	−2.399857	−2.709007	−0.021352
H	−0.167036	−1.839899	0.267047
H	0.001171	−1.496083	−1.439869
H	−3.866217	−0.372391	−2.354420
H	−0.607429	−4.274332	−0.783302
H	−2.117180	−4.504447	−1.680226
H	−0.764935	−3.637724	−2.428812
H	0.267943	−0.015692	2.583798
H	−3.711146	−0.787409	1.329969
H	−5.617989	−1.643087	2.670902
H	−7.707933	−2.645997	−0.923141
H	−5.794042	−1.811428	−2.271218
H	1.587434	0.442701	−2.278957
H	1.201984	2.026512	−1.624748
H	3.564380	2.643096	−1.548925
H	5.209564	1.307355	−0.191165
H	4.212587	2.484704	−3.928666
H	2.487381	2.862855	−3.799528

H	3.005373	1.216267	−4.198743
H	6.398669	0.004409	3.141211
H	5.416319	1.418517	2.688913
H	4.844740	0.302979	3.962725
H	1.962312	−1.301779	5.501488
H	1.027891	0.032901	4.786355
H	0.825858	−1.632570	4.173590
H	6.432031	−4.373052	−2.383176
H	5.051164	−3.432785	−2.964975
H	6.621642	−2.646644	−2.714059
H	6.433347	−3.052765	−0.245429
H	4.876181	−3.868379	−0.497803
H	−0.307471	2.906554	2.826780
H	0.371637	4.460607	2.288164
H	−1.368293	4.137953	2.078868
H	−2.586758	5.668895	−0.974787
H	−1.064990	4.761459	−1.162385
H	−2.506563	4.304355	−2.117418
H	−4.912377	3.363854	0.073754
H	−6.132852	2.467165	−0.869627
H	−5.370068	1.700754	0.542149

B3LYP Energy = −2096.62160191 a.u.

(*aR*,1*S*,3*S*,1'*S*,3'*S*)-**22**, Conf J

C	−0.962418	2.029259	0.721359
C	−2.251320	2.469670	0.390576
C	−3.105461	1.620827	−0.314899
C	−2.659519	0.362410	−0.738617
C	−1.357469	−0.052452	−0.446540
C	−0.502986	0.778801	0.302054
O	−2.885935	−1.514867	−2.286367
C	−1.985451	−2.281318	−1.473068
C	−0.860145	−1.381915	−0.974542
C	−3.597994	−0.504756	−1.559383
C	−1.487570	−3.439396	−2.317539
C	−4.749298	−1.123987	−0.769377
C	0.891793	0.353663	0.642823
C	1.968738	0.658536	−0.197512
C	3.249618	0.218600	0.158761
C	3.461108	−0.476172	1.345984
C	2.379232	−0.802078	2.176743
C	1.104444	−0.375698	1.815221
C	−5.907747	−1.490394	−1.460577
C	−6.970630	−2.108741	−0.813733
C	−6.856039	−2.359973	0.543216
C	−5.730458	−2.014226	1.266442
C	−4.678604	−1.391177	0.598072
C	1.792972	1.481588	−1.452788
C	3.105679	2.124498	−1.880576
O	4.115034	1.104800	−1.968532
C	4.453861	0.522007	−0.715587
C	3.034269	2.799029	−3.236720
F	−7.890653	−2.962314	1.187131
O	4.739915	−0.901770	1.627754
C	5.372434	−0.296500	2.767903
O	2.667081	−1.531403	3.292965
C	1.591998	−1.931605	4.139735
C	5.232128	−0.744920	−1.105729
O	4.719552	−1.779631	−1.454273
O	6.551793	−0.512162	−1.085919
C	7.826873	−2.474875	−0.343467
C	7.436106	−1.588245	−1.509680
O	−0.099773	2.883543	1.369982
C	−0.381315	3.117602	2.759331
O	−2.676417	3.723714	0.769082
C	−2.262132	4.781416	−0.113789
C	−5.307703	2.348073	0.315063
O	−4.353574	2.032483	−0.715170
H	−2.539655	−2.678171	−0.613418
H	−0.291323	−1.906034	−0.204265
H	−0.161319	−1.199420	−1.798734
H	−4.039062	0.117838	−2.338072
H	−0.778023	−4.045034	−1.750366
H	−2.318513	−4.076203	−2.624032
H	−0.984794	−3.069210	−3.213501
H	0.255720	−0.612665	2.440988
H	−5.981078	−1.289082	−2.522771
H	−7.871932	−2.389041	−1.342951
H	−5.681663	−2.224754	2.326769
H	−3.798866	−1.104340	1.159591
H	1.426351	0.857437	−2.275354
H	1.045050	2.258760	−1.286158
H	3.415234	2.855767	−1.120786
H	5.138472	1.187297	−0.175838
H	3.994208	3.248345	−3.494321
H	2.276184	3.584432	−3.227619
H	2.771664	2.073861	−4.009562
H	4.856508	−0.566397	3.689074
H	6.389391	−0.682248	2.787814
H	5.397876	0.791324	2.661079
H	2.046319	−2.510000	4.940341
H	1.075398	−1.067246	4.565709
H	0.875509	−2.558030	3.602032
H	8.540773	−3.227158	−0.686550
H	8.300663	−1.891670	0.447728
H	6.959687	−2.989967	0.069672
H	6.941988	−2.156008	−2.296390
H	8.300061	−1.072985	−1.925599
H	0.368746	3.825661	3.106012
H	−1.377450	3.541327	2.891652
H	−0.291749	2.188564	3.328353
H	−2.678082	5.700576	0.294021
H	−1.173358	4.853391	−0.145159
H	−2.654582	4.617062	−1.119981
H	−6.231937	2.595413	−0.202843
H	−5.474613	1.480176	0.956756
H	−4.975313	3.196289	0.911360

B3LYP Energy = −2096.62157562 a.u.

(*aR*,1*S*,3*S*,1'*S*,3'*S*)-**22**, Conf K

C	−0.877208	2.154714	0.544558
C	−2.175886	2.542273	0.186397
C	−3.019161	1.620191	−0.435394
C	−2.553234	0.338331	−0.753622
C	−1.242043	−0.027036	−0.436426
C	−0.398678	0.880377	0.231755
O	−2.752799	−1.667522	−2.134233
C	−1.835156	−2.346467	−1.264675
C	−0.723883	−1.388138	−0.851373
C	−3.480207	−0.612195	−1.490933
C	−1.321139	−3.562495	−2.012591
C	−4.615867	−1.183869	−0.645023
C	1.001930	0.507974	0.609373
C	2.076055	0.740798	−0.257076
C	3.362787	0.355688	0.142609
C	3.579289	−0.213005	1.394604
C	2.500251	−0.465287	2.254209
C	1.220975	−0.094457	1.850783
C	−5.768227	−1.632897	−1.296790
C	−6.814815	−2.217504	−0.594545
C	−6.690264	−2.350331	0.778221
C	−5.570676	−1.919207	1.463848
C	−4.535103	−1.332153	0.739707
C	1.887057	1.426115	−1.591105
C	3.191765	2.028851	−2.094978
O	4.210658	1.015312	−2.068822
C	4.564924	0.598628	−0.755929
C	3.110549	2.544260	−3.518562
F	−7.708621	−2.919448	1.475890
O	4.859798	−0.600964	1.715964
C	5.497986	0.124976	2.780472
O	2.795750	−1.072599	3.439152
C	1.727033	−1.374825	4.333284
C	5.459497	−0.629886	−0.985339
O	6.666386	−0.572260	−0.996888
O	4.743406	−1.725635	−1.251552
C	5.794179	−3.745087	−0.329852
C	5.462671	−2.948213	−1.576337
O	−0.025281	3.074552	1.111299
C	−0.309249	3.434960	2.472920
O	−2.620749	3.817138	0.456334
C	−2.245467	4.796424	−0.528551
C	−5.230671	2.355949	0.149410
O	−4.277175	1.974443	−0.858975
H	−2.377642	−2.680209	−0.371262
H	−0.141107	−1.833779	−0.042869
H	−0.033993	−1.264469	−1.693713
H	−3.935944	−0.066060	−2.317113
H	−0.599828	−4.108114	−1.400819
H	−2.142698	−4.235748	−2.260729
H	−0.828695	−3.260196	−2.939285
H	0.374016	−0.276911	2.496844
H	−5.849468	−1.524665	−2.371908
H	−7.711103	−2.561805	−1.093628
H	−5.513763	−2.037785	2.537947
H	−3.660164	−0.979829	1.270495
H	1.520101	0.715016	−2.339891
H	1.133524	2.210798	−1.503075
H	3.496932	2.844794	−1.424869
H	5.215164	1.353018	−0.296094
H	4.065436	2.971335	−3.827484
H	2.344164	3.317731	−3.596631
H	2.854835	1.733615	−4.203948
H	6.521469	−0.240974	2.822659
H	5.503347	1.196760	2.565230
H	4.998319	−0.059546	3.731328
H	2.187308	−1.858876	5.191018
H	1.212767	−0.467638	4.661852
H	1.007003	−2.057967	3.875107
H	6.262106	−4.688563	−0.620194
H	6.486911	−3.202754	0.313311
H	4.891373	−3.970804	0.239267
H	4.778532	−3.491508	−2.225655
H	6.359530	−2.687121	−2.136203
H	−1.309618	3.858842	2.566094
H	−0.209200	2.564296	3.126323
H	0.433500	4.180159	2.750437
H	−2.675558	5.740580	−0.200308
H	−1.159241	4.888463	−0.586692
H	−2.650649	4.530552	−1.507607
H	−4.908277	3.253823	0.674206
H	−6.161465	2.547114	−0.380364
H	−5.380379	1.539001	0.858695

B3LYP Energy = −2096.62148517 a.u.

(*aR*,1*S*,3*S*,1'*S*,3'*S*)-**22**, Conf L

C	−0.887273	2.195939	0.517307
C	−2.179266	2.560633	0.113836
C	−2.997181	1.613257	−0.503916
C	−2.510833	0.327731	−0.773220
C	−1.205047	−0.015390	−0.411649
C	−0.388047	0.918439	0.252908
O	−2.654001	−1.718100	−2.101230
C	−1.754317	−2.362666	−1.188279
C	−0.662950	−1.382148	−0.774416
C	−3.408860	−0.653158	−1.506889
C	−1.210935	−3.595914	−1.885612
C	−4.559987	−1.214447	−0.674626
C	1.004434	0.569625	0.679847
C	2.107004	0.821552	−0.144127
C	3.381986	0.440156	0.294199
C	3.564201	−0.138001	1.550492
C	2.461312	−0.354251	2.391167
C	1.191276	−0.016917	1.932933
C	−4.521789	−1.305447	0.716721
C	−5.569832	−1.882722	1.430835
C	−6.658312	−2.362811	0.727931
C	−6.740200	−2.287857	−0.652437
C	−5.682104	−1.711545	−1.344130
C	1.951318	1.510299	−1.481164
C	3.261563	2.131803	−1.944520

O	4.294274	1.134378	−1.880487
C	4.610621	0.733652	−0.552035
C	3.218657	2.640962	−3.372061
F	−7.688555	−2.923012	1.415271
O	4.857928	−0.387610	1.937515
C	5.195884	−1.705395	2.405687
O	2.721936	−0.867577	3.629346
C	1.631430	−1.082850	4.521982
C	5.574230	−0.444243	−0.751708
O	6.776536	−0.328615	−0.726799
O	4.933175	−1.581417	−1.042066
C	4.823513	−3.868365	−1.744755
C	5.753366	−2.727646	−1.399839
O	−0.060436	3.139051	1.082544
C	−0.381970	3.528154	2.427750
O	−2.642722	3.837927	0.336735
C	−2.250962	4.793816	−0.664607
C	−5.229379	2.346568	0.000733
O	−4.246945	1.942005	−0.970163
H	−2.317286	−2.674968	−0.299676
H	−0.097579	−1.798564	0.061513
H	0.047727	−1.275043	−1.601808
H	−3.848928	−0.135402	−2.359408
H	−0.502663	−4.117409	−1.238550
H	−2.020618	−4.282800	−2.135519
H	−0.695885	−3.317370	−2.807441
H	0.328185	−0.186586	2.560799
H	−3.671001	−0.914833	1.259799
H	−5.546188	−1.956388	2.510204
H	−7.613357	−2.669218	−1.165387
H	−5.730155	−1.648087	−2.424811
H	1.614662	0.797376	−2.242395
H	1.185765	2.284962	−1.412792
H	3.532561	2.954311	−1.267867
H	5.203899	1.517014	−0.064574
H	4.176360	3.081680	−3.651698
H	2.443346	3.402132	−3.478079
H	2.997525	1.823878	−4.061937
H	6.279837	−1.772650	2.337374
H	4.874748	−1.850126	3.434828
H	4.740450	−2.466342	1.768170
H	2.070112	−1.483274	5.432569
H	1.114210	−0.147214	4.750033
H	0.918641	−1.805173	4.115087
H	5.412595	−4.744280	−2.023395
H	4.195158	−4.135820	−0.893859
H	4.179347	−3.607544	−2.585501
H	6.385551	−2.447104	−2.243019
H	6.401203	−2.969139	−0.556241
H	−0.294471	2.672862	3.102835
H	0.349443	4.283485	2.707710
H	−1.386684	3.948264	2.485086
H	−2.695779	5.742822	−0.371902
H	−1.164397	4.893237	−0.699221
H	−2.630400	4.499100	−1.645749
H	−4.931327	3.267034	0.500077
H	−6.149523	2.505255	−0.557572
H	−5.385366	1.553824	0.735687

B3LYP Energy = −2096.62144283 a.u.

(*aR*,1*S*,3*S*,1'*S*,3'*S*)-**22**, Conf M

C	−1.017258	2.051213	0.779563
C	−2.305739	2.471555	0.420978
C	−3.125277	1.616557	−0.317438
C	−2.643918	0.373734	−0.748205
C	−1.341829	−0.019880	−0.428483
C	−0.523178	0.815848	0.354513
O	−2.789050	−1.478848	−2.333950
C	−1.895621	−2.242681	−1.510506
C	−0.803585	−1.330593	−0.963010
C	−3.542319	−0.498732	−1.607038
C	−1.350086	−3.372998	−2.362989
C	−4.698476	−1.159171	−0.858353
C	0.868797	0.407659	0.725598
C	1.961437	0.722330	−0.089590
C	3.235070	0.268072	0.276519
C	3.429795	−0.441585	1.461402
C	2.338567	−0.712220	2.302934
C	1.067183	−0.306357	1.908382
C	−5.822555	−1.554465	−1.589403
C	−6.885948	−2.210660	−0.982006
C	−6.807145	−2.470005	0.376027
C	−5.716631	−2.096114	1.137862
C	−4.663428	−1.435704	0.508470
C	1.802498	1.566081	−1.333519
C	3.116320	2.231078	−1.718705
O	4.134021	1.220769	−1.810934
C	4.451642	0.607489	−0.564553
C	3.067129	2.939378	−3.058486
F	−7.842784	−3.109395	0.981505
O	4.723074	−0.764087	1.799821
C	5.023423	−2.149356	2.058732
O	2.610408	−1.346098	3.481374
C	1.530641	−1.635972	4.365885
C	5.264858	−0.624620	−0.988987
O	4.798977	−1.711163	−1.234261
O	6.554148	−0.294706	−1.138124
C	8.119622	−2.118769	−0.627062
C	7.457020	−1.285444	−1.705892
O	−0.187691	2.912560	1.460423
C	−0.512121	3.131702	2.842859
O	−2.764741	3.712099	0.804144
C	−2.346031	4.789544	−0.052461
C	−5.355711	2.290905	0.268765
O	−4.371295	2.009292	−0.742609
H	−2.464930	−2.667672	−0.674589
H	−0.248055	−1.856563	−0.184071
H	−0.083864	−1.120511	−1.762284
H	−3.977970	0.128161	−2.385319
H	−0.644780	−3.976016	−1.787664
H	−2.158615	−4.019824	−2.706182

H	−0.829356	−2.974343	−3.236277
H	0.212637	−0.523884	2.532991
H	−5.867912	−1.346370	−2.651844
H	−7.760842	−2.513745	−1.542093
H	−5.695386	−2.314048	2.197608
H	−3.811156	−1.127336	1.100098
H	1.460750	0.954553	−2.176189
H	1.041955	2.330933	−1.167864
H	3.404908	2.944662	−0.934406
H	5.114305	1.267406	0.008556
H	4.028828	3.401496	−3.285025
H	2.303657	3.719498	−3.044249
H	2.824874	2.233000	−3.855071
H	4.531283	−2.493792	2.965297
H	4.722992	−2.764915	1.208612
H	6.103311	−2.196534	2.180880
H	1.029635	−0.721743	4.694897
H	0.802319	−2.305646	3.900784
H	1.977586	−2.130509	5.224767
H	8.859642	−2.778530	−1.085505
H	8.631746	−1.482809	0.096648
H	7.392548	−2.737539	−0.101382
H	6.899060	−1.905239	−2.406014
H	8.187995	−0.692847	−2.253124
H	−1.516825	3.542069	2.949718
H	−0.427299	2.199126	3.406716
H	0.219210	3.845473	3.216553
H	−2.707589	4.632393	−1.071327
H	−2.790067	5.695105	0.356130
H	−1.258360	4.881713	−0.052721
H	−5.513219	1.413617	0.899975
H	−5.058867	3.142438	0.878913
H	−6.274756	2.517866	−0.267369

B3LYP Energy = −2096.62115145 a.u.

(*aR*,1*S*,3*S*,1’*S*,3’*S*)-**22**, Conf N

C	−0.854903	2.124855	0.595534
C	−2.141266	2.551817	0.229980
C	−2.993055	1.654518	−0.423048
C	−2.551909	0.358306	−0.727462
C	−1.254346	−0.040313	−0.400882
C	−0.394637	0.847932	0.270946
O	−2.792507	−1.632259	−2.117826
C	−1.888645	−2.340236	−1.256630
C	−0.761739	−1.408073	−0.826051
C	−3.501168	−0.579861	−1.450241
C	−1.393608	−3.554530	−2.019731
C	−4.621174	−1.154941	−0.582970
C	0.993968	0.444338	0.661070
C	2.092648	0.730144	−0.157703
C	3.367440	0.315315	0.250992
C	3.547160	−0.343208	1.464179
C	2.443460	−0.651870	2.272639
C	1.177406	−0.245111	1.862591
C	−4.587614	−1.153916	0.810443
C	−5.615385	−1.727164	1.556543
C	−6.677233	−2.298746	0.882598
C	−6.752633	−2.318617	−0.500084
C	−5.716330	−1.742212	−1.223613
C	1.938921	1.494768	−1.452991
C	3.257352	2.114588	−1.896741
O	4.272776	1.097363	−1.895384
C	4.593994	0.624486	−0.593298
C	3.210070	2.698919	−3.294988
F	−7.687850	−2.857249	1.601121
O	4.816773	−0.759186	1.794827
C	5.426828	−0.111951	2.924521
O	2.704010	−1.345940	3.417448
C	1.608557	−1.721582	4.249506
C	5.525562	−0.569485	−0.848580
O	6.730879	−0.485286	−0.814831
O	4.853010	−1.671306	−1.186282
C	4.677594	−3.958648	−1.871542
C	5.639234	−2.849487	−1.511506
O	−0.010403	3.002494	1.241764
C	−0.295085	3.217225	2.634384
O	−2.608913	3.793324	0.593707
C	−1.975539	4.925158	−0.031819
C	−4.604495	3.169553	−1.419328
O	−4.292947	1.943970	−0.739776
H	−2.440471	−2.674837	−0.369128
H	−0.194244	−1.875019	−0.019018
H	−0.062413	−1.288188	−1.661484
H	−3.973845	−0.023487	−2.260335
H	−0.681103	−4.119048	−1.414862
H	−2.225247	−4.211918	−2.276726
H	−0.895757	−3.248161	−2.942265
H	0.312641	−0.467214	2.471603
H	−3.756750	−0.693546	1.329194
H	−5.596748	−1.727260	2.638501
H	−7.604931	−2.770601	−0.990302
H	−5.759402	−1.751497	−2.306312
H	1.580343	0.830496	−2.247522
H	1.190470	2.280215	−1.335245
H	3.549898	2.895477	−1.180731
H	5.210238	1.370805	−0.076378
H	4.173540	3.134461	−3.562564
H	2.449572	3.480050	−3.351906
H	2.964733	1.923920	−4.024098
H	6.445084	−0.491297	2.974222
H	5.449079	0.971540	2.781133
H	4.895267	−0.353533	3.844825
H	2.043873	−2.270241	5.080975
H	1.078191	−0.845426	4.632063
H	0.908150	−2.368975	3.715390
H	5.241049	−4.859529	−2.122397
H	4.016041	−4.190490	−1.035705
H	4.068365	−3.685002	−2.734205
H	6.305800	−2.602043	−2.338637
H	6.250097	−3.104351	−0.644562
H	0.432739	3.946279	2.984803

H	−1.304634	3.607218	2.773140
H	−0.175675	2.287859	3.196391
H	−2.469599	5.806441	0.371910
H	−0.911468	4.956747	0.198485
H	−2.117867	4.892700	−1.115074
H	−3.868029	3.378541	−2.198932
H	−5.578237	3.011596	−1.879329
H	−4.656128	4.005345	−0.724468

B3LYP Energy = −2096.62100273 a.u.

(*aR*,1*S*,3*S*,1'*S*,3'*S*)-**22**, Conf O

C	−1.078735	2.200005	0.272861
C	−2.378416	2.464884	−0.178777
C	−3.127124	1.438343	−0.756445
C	−2.612779	0.139719	−0.829445
C	−1.319407	−0.123549	−0.368758
C	−0.536000	0.914361	0.170133
O	−2.690405	−2.087188	−1.824016
C	−1.821897	−2.571082	−0.787882
C	−0.754135	−1.523366	−0.492028
C	−3.477514	−0.958738	−1.421180
C	−1.240939	−3.889642	−1.262758
C	−4.639031	−1.405113	−0.533537
C	0.855669	0.659358	0.660258
C	1.947836	0.623411	−0.214877
C	3.221176	0.347185	0.301943
C	3.413250	0.148491	1.668279
C	2.321966	0.227589	2.548789
C	1.053917	0.460323	2.027848
C	−5.695566	−2.100784	−1.128433
C	−6.761359	−2.577718	−0.375669
C	−6.754063	−2.348362	0.989984
C	−5.731956	−1.663762	1.618782
C	−4.674377	−1.192578	0.843736
C	1.796358	0.897383	−1.694262
C	3.109647	1.361744	−2.309718
O	4.131334	0.404442	−1.987191
C	4.443288	0.347627	−0.598896
C	3.062452	1.473925	−3.821078
F	−7.793591	−2.808026	1.736435
O	4.705660	−0.021914	2.106415
C	5.005992	−1.210008	2.864532
O	2.593738	0.091586	3.879479
C	1.518660	0.202694	4.810070
C	5.283521	−0.931160	−0.477564
O	4.828907	−2.042954	−0.353893
O	6.587004	−0.648781	−0.598970
C	7.723729	−2.288786	−2.026758
C	7.525172	−1.761756	−0.619135
O	−0.360115	3.188119	0.903725
C	0.102238	4.268497	0.077183
O	−2.902456	3.733639	−0.068911
C	−3.649736	3.960694	1.138950
C	−4.565635	2.534318	−2.315820
O	−4.415528	1.657656	−1.184404
H	−2.418328	−2.747199	0.115846
H	−0.220431	−1.794081	0.421481
H	−0.012094	−1.544089	−1.297110
H	−3.907048	−0.590430	−2.353667
H	−0.557345	−4.294708	−0.513993
H	−2.034268	−4.617875	−1.436490
H	−0.687505	−3.749918	−2.193868
H	0.200966	0.519521	2.688203
H	−5.685227	−2.274561	−2.197972
H	−7.584358	−3.112479	−0.831394
H	−5.765972	−1.500906	2.688036
H	−3.875024	−0.645328	1.326244
H	1.471497	−0.005511	−2.222970
H	1.025209	1.651585	−1.860960
H	3.392997	2.331399	−1.876358
H	5.084029	1.197618	−0.334874
H	4.024973	1.805803	−4.212285
H	2.299952	2.194764	−4.122295
H	2.820296	0.507647	−4.268162
H	4.682629	−2.097900	2.317964
H	6.088662	−1.222573	2.972515
H	4.533905	−1.178568	3.843963
H	1.965587	0.074857	5.792887
H	1.043891	1.185481	4.752277
H	0.768968	−0.576553	4.649034
H	8.484590	−3.072354	−2.015076
H	6.801717	−2.715067	−2.422772
H	8.060647	−1.495166	−2.695257
H	8.444783	−1.339820	−0.218010
H	7.161108	−2.537956	0.052117
H	−0.732005	4.788389	−0.393837
H	0.633416	4.948449	0.740085
H	0.791844	3.899727	−0.686417
H	−4.505999	3.285112	1.196231
H	−3.011754	3.829020	2.015698
H	−3.999442	4.990179	1.094140
H	−5.628822	2.546361	−2.546292
H	−4.224464	3.541727	−2.079787
H	−4.011583	2.145920	−3.174613

B3LYP Energy = −2096.62099960 a.u.

(*aR*,1*S*,3*S*,1'*S*,3'*S*)-**22**, Conf P

C	−0.996326	1.947295	0.861812
C	−2.268579	2.438139	0.529713
C	−3.117981	1.645791	−0.250246
C	−2.693644	0.387922	−0.701513
C	−1.408886	−0.070617	−0.404018
C	−0.547351	0.715586	0.382232
O	−2.938911	−1.421581	−2.319736
C	−2.069014	−2.242531	−1.525967
C	−0.932164	−1.390368	−0.974053
C	−3.643089	−0.437892	−1.550575
C	−1.584564	−3.377302	−2.408608
C	−4.797164	−1.083818	−0.783003
C	0.838417	0.254284	0.713056

C	1.924063	0.587199	−0.105558
C	3.197019	0.111448	0.232560
C	3.393363	−0.646725	1.383220
C	2.302831	−0.998994	2.191746
C	1.035448	−0.538865	1.845975
C	−5.881268	−1.575572	−1.516241
C	−6.948595	−2.208363	−0.891748
C	−6.915830	−2.342917	0.486211
C	−5.866433	−1.869787	1.249824
C	−4.806949	−1.237414	0.602207
C	1.764770	1.472969	−1.319641
C	3.088646	2.113749	−1.715143
O	4.081572	1.083205	−1.848825
C	4.408519	0.439233	−0.623220
C	3.030900	2.852159	−3.038291
F	−7.957004	−2.957957	1.108651
O	4.664155	−1.106397	1.647677
C	5.302179	−0.568449	2.818323
O	2.574798	−1.787957	3.270564
C	1.490510	−2.210632	4.094675
C	5.170894	−0.819875	−1.065042
O	4.649101	−1.826817	−1.476442
O	6.493699	−0.623434	−1.003117
C	8.778417	−1.281537	−1.294794
C	7.339565	−1.715226	−1.457688
O	−0.151777	2.719116	1.631718
C	−0.459184	2.749160	3.035192
O	−2.729010	3.631591	1.035217
C	−2.056174	4.822257	0.584629
C	−4.652136	3.291200	−1.142810
O	−4.404259	2.004643	−0.555868
H	−2.646414	−2.658420	−0.690831
H	−0.391051	−1.956878	−0.213828
H	−0.212687	−1.193527	−1.777012
H	−4.084002	0.219433	−2.300824
H	−0.899032	−4.022290	−1.855537
H	−2.425229	−3.980852	−2.753594
H	−1.058039	−2.983969	−3.280866
H	0.180167	−0.800391	2.452698
H	−5.890284	−1.464704	−2.594056
H	−7.792180	−2.587179	−1.453780
H	−5.880933	−1.989941	2.325153
H	−3.985902	−0.854070	1.193834
H	1.388237	0.895347	−2.171386
H	1.029651	2.253828	−1.116996
H	3.408253	2.803910	−0.921738
H	5.099697	1.070257	−0.051593
H	3.998140	3.298576	−3.272613
H	2.284803	3.647804	−2.993540
H	2.759580	2.168259	−3.845007
H	5.346017	0.522732	2.765230
H	4.778086	−0.874672	3.723385
H	6.312553	−0.971743	2.822466
H	1.932825	−2.834099	4.867668
H	0.986969	−1.359027	4.560090
H	0.765475	−2.798516	3.525910
H	9.438802	−2.083364	−1.630677
H	8.990443	−0.392415	−1.890418
H	9.007861	−1.065399	−0.250334
H	7.108751	−2.599452	−0.862330
H	7.093973	−1.931363	−2.498187
H	−1.455558	3.158132	3.210979
H	−0.390061	1.745879	3.462452
H	0.288883	3.390336	3.497117
H	−2.565140	5.654405	1.066539
H	−1.005324	4.812518	0.870612
H	−2.141703	4.922380	−0.500388
H	−4.652141	4.076727	−0.389612
H	−3.910144	3.513762	−1.913759
H	−5.636480	3.221607	−1.601880

B3LYP Energy = −2096.62099305 a.u.

(*aR*,1*S*,3*S*,1’*S*,3’*S*)-**22**, Conf Q

C	−1.037349	2.208399	−0.024755
C	−2.339650	2.427843	−0.492791
C	−3.109248	1.342947	−0.915843
C	−2.614097	0.038765	−0.815339
C	−1.318201	−0.177720	−0.336730
C	−0.513738	0.913073	0.043809
O	−2.726548	−2.301067	−1.499856
C	−1.859076	−2.650198	−0.409639
C	−0.773636	−1.589319	−0.269024
C	−3.496355	−1.117650	−1.251181
C	−1.303103	−4.032163	−0.696394
C	−4.662183	−1.426578	−0.312107
C	0.879030	0.705694	0.552810
C	1.960119	0.527555	−0.319118
C	3.236056	0.318603	0.221404
C	3.438349	0.321973	1.598243
C	2.351698	0.480954	2.471429
C	1.084099	0.677651	1.934369
C	−5.727829	−2.181544	−0.810581
C	−6.799696	−2.540014	−0.002755
C	−6.789183	−2.130906	1.320149
C	−5.757987	−1.382252	1.853812
C	−4.694206	−1.032481	1.024628
C	1.802762	0.592957	−1.821362
C	3.121568	0.942255	−2.498870
O	4.126733	0.015735	−2.056236
C	4.447286	0.124844	−0.674011
C	3.068437	0.850920	−4.011573
F	−7.834736	−2.473462	2.119346
O	4.710806	0.080423	2.065378
C	5.353051	1.167458	2.753651
O	2.628391	0.422080	3.805358
C	1.551587	0.562901	4.730075
C	5.193316	−1.184038	−0.369556
O	4.654170	−2.242494	−0.159233
O	6.518787	−0.999863	−0.437146
C	7.606122	−2.895291	−1.555521
C	7.372178	−2.164015	−0.248173

O	−0.297266	3.264487	0.452970
C	0.179363	4.200224	−0.527070
O	−2.839061	3.709371	−0.554605
C	−3.656501	4.081448	0.569082
C	−4.540507	2.227227	−2.609451
O	−4.397936	1.524919	−1.361312
H	−2.452425	−2.686494	0.512309
H	−0.235813	−1.739390	0.669446
H	−0.038580	−1.732497	−1.067935
H	−3.923385	−0.871964	−2.224314
H	−0.615774	−4.336140	0.095520
H	−2.108730	−4.765242	−0.755588
H	−0.759235	−4.035987	−1.643439
H	0.233715	0.812106	2.587279
H	−5.720214	−2.496226	−1.847475
H	−7.629566	−3.120133	−0.384526
H	−5.789363	−1.078283	2.891888
H	−3.887680	−0.436658	1.431806
H	1.457977	−0.368692	−2.216986
H	1.044598	1.331365	−2.089066
H	3.426255	1.955669	−2.201256
H	5.146959	0.955933	−0.526454
H	4.034175	1.112275	−4.446015
H	2.315545	1.536206	−4.405691
H	2.810881	−0.163118	−4.323668
H	6.364791	0.830993	2.969807
H	5.392550	2.055466	2.116947
H	4.834609	1.401324	3.682928
H	1.997717	0.475514	5.717617
H	1.069155	1.539162	4.634256
H	0.808401	−0.227616	4.597246
H	8.302749	−3.719958	−1.388911
H	6.676616	−3.308423	−1.947618
H	8.038676	−2.228365	−2.302925
H	8.297819	−1.751641	0.149149
H	6.916624	−2.812171	0.498850
H	0.706953	4.974578	0.026211
H	0.874538	3.712891	−1.215644
H	−0.647424	4.642848	−1.082688
H	−3.975233	5.106296	0.389662
H	−4.531572	3.432564	0.643150
H	−3.078612	4.035313	1.494993
H	−4.170327	3.249016	−2.530166
H	−4.008232	1.700303	−3.405848
H	−5.605978	2.234437	−2.829207

B3LYP Energy = −2096.62094387 a.u.

(*aR*,1*S*,3*S*,1’*S*,3’*S*)-**22**, Conf R

C	−1.017647	2.132695	0.446629
C	−2.332444	2.467448	0.096364
C	−3.136986	1.518029	−0.535580
C	−2.661184	0.222926	−0.763480
C	−1.351636	−0.110913	−0.404233
C	−0.514726	0.852928	0.188847
O	−2.847059	−1.892528	−1.966351
C	−1.938224	−2.493357	−1.030671
C	−0.829284	−1.502338	−0.694937
C	−3.583025	−0.793248	−1.413789
C	−1.419019	−3.771725	−1.661121
C	−4.709794	−1.300782	−0.514673
C	0.893617	0.524195	0.577404
C	1.939260	0.563871	−0.352983
C	3.235630	0.245225	0.073921
C	3.490692	−0.077626	1.403210
C	2.438275	−0.144366	2.328892
C	1.151648	0.167434	1.903192
C	−5.816514	−1.903720	−1.119324
C	−6.855806	−2.430116	−0.362548
C	−6.770415	−2.345257	1.016947
C	−5.696296	−1.756527	1.656139
C	−4.666442	−1.233273	0.877183
C	1.722839	0.978385	−1.790571
C	3.012022	1.498080	−2.413902
O	4.038645	0.507616	−2.240798
C	4.412942	0.294291	−0.884831
C	2.898867	1.767173	−3.901822
F	−7.783324	−2.854345	1.767967
O	4.781377	−0.406011	1.752508
C	5.441489	0.493480	2.659769
O	2.765734	−0.520062	3.598274
C	1.723838	−0.619370	4.567429
C	5.191675	−1.030117	−0.925195
O	4.678722	−2.121500	−0.953146
O	6.509434	−0.800228	−1.005669
C	7.800944	−2.459720	0.256816
C	7.391801	−1.953099	−1.112299
O	−0.242494	3.038935	1.131443
C	0.211247	4.181214	0.388180
O	−2.811753	3.731730	0.356221
C	−3.555527	3.843246	1.582398
C	−4.621570	2.786664	−1.908777
O	−4.439533	1.807059	−0.870187
H	−2.490859	−2.743835	−0.116645
H	−0.253943	−1.872618	0.156149
H	−0.132230	−1.457965	−1.538390
H	−4.049053	−0.325535	−2.281995
H	−0.704388	−4.260086	−0.995717
H	−2.239481	−4.463792	−1.855499
H	−0.916331	−3.554698	−2.606083
H	0.327208	0.137074	2.600934
H	−5.867413	−1.964027	−2.200069
H	−7.717409	−2.893311	−0.825206
H	−5.669561	−1.706656	2.736775
H	−3.826117	−0.760364	1.368918
H	1.371565	0.130715	−2.389115
H	0.948745	1.745636	−1.849195
H	3.322698	2.416269	−1.895601
H	5.104846	1.084225	−0.569088
H	3.847079	2.130976	−4.299579
H	2.132295	2.520778	−4.092200
H	2.626441	0.854098	−4.434913

H	4.950686	0.495886	3.632525
H	6.460114	0.125341	2.760995
H	5.459196	1.507076	2.250170
H	2.206532	−0.938293	5.487848
H	1.235919	0.345540	4.728223
H	0.978192	−1.362380	4.272977
H	8.499027	−3.291774	0.140607
H	8.297541	−1.675423	0.830260
H	6.936806	−2.813031	0.819411
H	6.888316	−2.725262	−1.691674
H	8.247519	−1.582041	−1.673472
H	−0.628658	4.752515	−0.007135
H	0.774090	4.793236	1.090149
H	0.870327	3.871530	−0.427005
H	−4.435501	3.197050	1.561524
H	−2.925621	3.588025	2.437634
H	−3.867507	4.883021	1.657781
H	−5.694235	2.845494	−2.080502
H	−4.239753	3.760020	−1.602056
H	−4.123222	2.465236	−2.827285

B3LYP Energy = −2096.62089225 a.u.

(*aR*,1*S*,3*S*,1’*S*,3’*S*)-**22**, Conf S

C	−1.011793	1.906552	0.996663
C	−2.285509	2.418410	0.704085
C	−3.132909	1.687541	−0.135710
C	−2.699951	0.475751	−0.692919
C	−1.413502	−0.000541	−0.432946
C	−0.558412	0.717487	0.423195
O	−2.928001	−1.176509	−2.472402
C	−2.051555	−2.062907	−1.761071
C	−0.925454	−1.258893	−1.121184
C	−3.642618	−0.280438	−1.611669
C	−1.551513	−3.097740	−2.751429
C	−4.781943	−1.014365	−0.903306
C	0.816487	0.217361	0.742765
C	1.936576	0.647978	0.022939
C	3.189994	0.104023	0.330358
C	3.338669	−0.816535	1.367597
C	2.220813	−1.210531	2.119824
C	0.969420	−0.706607	1.778251
C	−5.849080	−1.477286	−1.678985
C	−6.900967	−2.184971	−1.111260
C	−6.870148	−2.423892	0.252629
C	−5.837623	−1.981938	1.056850
C	−4.793351	−1.273281	0.465896
C	1.825421	1.696136	−1.060612
C	3.164300	2.374617	−1.315707
O	4.159174	1.364269	−1.549207
C	4.436391	0.563780	−0.404908
C	3.159930	3.285987	−2.527531
F	−7.896206	−3.113476	0.819575
O	4.616696	−1.224786	1.669193
C	4.889973	−2.639593	1.677936
O	2.448282	−2.059059	3.165134
C	1.341072	−2.468893	3.964530
C	5.260850	−0.597223	−0.976806
O	4.794997	−1.582616	−1.496999
O	6.570314	−0.335637	−0.893290
C	8.887356	−0.805339	−1.274451
C	7.476486	−1.301103	−1.494375
O	−0.171267	2.619102	1.825355
C	−0.474272	2.533711	3.227795
O	−2.748362	3.566875	1.303154
C	−2.084051	4.791374	0.938790
C	−4.690478	3.393198	−0.864951
O	−4.423018	2.059123	−0.406685
H	−2.627019	−2.567705	−0.974751
H	−0.385202	−1.891669	−0.414777
H	−0.201778	−0.981644	−1.896219
H	−4.097289	0.437959	−2.294664
H	−0.866554	−3.792736	−2.261550
H	−2.385266	−3.667537	−3.163902
H	−1.020373	−2.613722	−3.573972
H	0.094352	−1.014441	2.332779
H	−5.856220	−1.284639	−2.745206
H	−7.731449	−2.542629	−1.705735
H	−5.853150	−2.184724	2.119693
H	−3.984955	−0.914391	1.089461
H	1.476628	1.245059	−1.996436
H	1.086595	2.446841	−0.775520
H	3.455196	2.947892	−0.424234
H	5.077144	1.124372	0.286650
H	4.136887	3.752136	−2.661756
H	2.414985	4.074286	−2.403502
H	2.918045	2.720199	−3.429604
H	4.576985	−3.090958	0.734565
H	5.969377	−2.728075	1.783624
H	4.392535	−3.129509	2.511966
H	1.754099	−3.125616	4.726160
H	0.860539	−1.613158	4.446054
H	0.603429	−3.018192	3.373604
H	9.592731	−1.510992	−1.717574
H	9.038417	0.168445	−1.742452
H	9.112002	−0.719855	−0.210326
H	7.306884	−2.271797	−1.027002
H	7.233890	−1.386877	−2.554361
H	−1.477531	2.909053	3.436321
H	−0.384512	1.501900	3.575716
H	0.263296	3.150984	3.736628
H	−2.595222	5.583136	1.482457
H	−1.032067	4.766246	1.219593
H	−2.174519	4.970337	−0.135709
H	−4.709642	4.099506	−0.037294
H	−3.947425	3.704667	−1.603334
H	−5.670166	3.352066	−1.337298

B3LYP Energy = −2096.62086968 a.u.

(*aR*,1*S*,3*S*,1’*S*,3’*S*)-**22**, Conf T

C	−0.941585	2.267675	−0.007413

C	−2.252724	2.483109	−0.452387
C	−3.022439	1.397204	−0.872992
C	−2.518725	0.095087	−0.790507
C	−1.214642	−0.117538	−0.332935
C	−0.410194	0.974588	0.043138
O	−2.625891	−2.238372	−1.495765
C	−1.742408	−2.592523	−0.420628
C	−0.661382	−1.526561	−0.283908
C	−3.400168	−1.062681	−1.224115
C	−1.182302	−3.968555	−0.727657
C	−4.549702	−1.389629	−0.271150
C	0.991779	0.770791	0.528284
C	2.061267	0.617816	−0.362567
C	3.348180	0.411022	0.154749
C	3.568609	0.388956	1.528819
C	2.493594	0.523727	2.420375
C	1.216818	0.721226	1.906138
C	−4.571482	−0.997711	1.066351
C	−5.620499	−1.363390	1.907464
C	−6.647247	−2.125817	1.384914
C	−6.667542	−2.533413	0.061612
C	−5.610469	−2.158862	−0.758238
C	1.873898	0.700600	−1.860997
C	3.178192	1.049496	−2.565953
O	4.193200	0.126683	−2.138688
C	4.547725	0.263071	−0.767602
C	3.094310	0.953559	−4.076862
F	−7.678335	−2.484077	2.195800
O	4.846673	0.142025	1.975573
C	5.502539	1.219711	2.666064
O	2.791287	0.441195	3.748488
C	1.730305	0.570985	4.692903
C	5.430715	−0.963334	−0.490294
O	6.637644	−0.928961	−0.538935
O	4.704880	−2.061948	−0.267084
C	5.684778	−4.018648	−1.377535
C	5.414601	−3.315348	−0.061741
O	−0.198522	3.322305	0.467741
C	0.245579	4.279416	−0.507319
O	−2.761380	3.761739	−0.493805
C	−3.566356	4.116514	0.644530
C	−4.485998	2.289685	−2.534866
O	−4.318932	1.574924	−1.296755
H	−2.323094	−2.641012	0.508802
H	−0.112221	−1.681156	0.647314
H	0.064953	−1.658369	−1.092792
H	−3.843174	−0.810467	−2.188387
H	−0.484265	−4.276610	0.053367
H	−1.984396	−4.705631	−0.784450
H	−0.650137	−3.960092	−1.681366
H	0.375117	0.838776	2.573375
H	−3.768652	−0.391106	1.464785
H	−5.644101	−1.060934	2.946183
H	−7.493650	−3.124606	−0.311220
H	−5.610513	−2.472302	−1.795517
H	1.514549	−0.255440	−2.257639
H	1.113451	1.444639	−2.105512
H	3.488222	2.064026	−2.277802
H	5.207045	1.131401	−0.646637
H	4.050233	1.215860	−4.531765
H	2.331622	1.635760	−4.457296
H	2.832563	−0.061988	−4.380678
H	5.524413	2.118005	2.043408
H	5.005193	1.436151	3.611112
H	6.520035	0.882615	2.851384
H	0.983651	−0.216767	4.562556
H	2.192775	0.470229	5.671611
H	1.247668	1.548944	4.617933
H	6.151253	−4.986543	−1.180698
H	4.756396	−4.191595	−1.923971
H	6.359502	−3.436412	−2.005443
H	6.335843	−3.109392	0.480940
H	4.746547	−3.896708	0.570960
H	0.787379	5.043934	0.045812
H	0.921166	3.808748	−1.226250
H	−0.598706	4.730036	−1.028917
H	−4.435757	3.460667	0.723634
H	−2.975747	4.065280	1.562102
H	−3.894644	5.140772	0.479714
H	−5.555013	2.293363	−2.736665
H	−4.119896	3.312523	−2.450832
H	−3.964427	1.773999	−3.345563

B3LYP Energy = −2096.62085627 a.u.

(*aR*,1*S*,3*S*,1'*S*,3'*S*)-**22**, Conf U

C	−0.969577	2.068823	0.545532
C	−2.263250	2.474771	0.182578
C	−3.080292	1.581479	−0.516106
C	−2.653163	0.277455	−0.783336
C	−1.367845	−0.124376	−0.409766
C	−0.516304	0.779520	0.249873
O	−2.874563	−1.751022	−2.122432
C	−2.009155	−2.440780	−1.207147
C	−0.880548	−1.510691	−0.776075
C	−3.589062	−0.665105	−1.518946
C	−1.511593	−3.689897	−1.909662
C	−4.755260	−1.188511	−0.680899
C	0.873813	0.385278	0.643703
C	1.957916	0.592715	−0.216427
C	3.233512	0.187793	0.195709
C	3.432694	−0.374691	1.453314
C	2.343201	−0.601846	2.306627
C	1.073365	−0.212593	1.890269
C	−4.758676	−1.182684	0.713110
C	−5.826310	−1.714733	1.433294
C	−6.890186	−2.249944	0.733046
C	−6.929399	−2.273153	−0.651032
C	−5.853490	−1.738777	−1.348535
C	1.792614	1.273227	−1.555713
C	3.112833	1.849461	−2.050366
O	4.113169	0.817305	−2.013829

C	4.444538	0.388964	−0.698899
C	3.052923	2.361876	−3.476116
F	−7.939315	−2.767359	1.426203
O	4.706915	−0.774579	1.789115
C	5.336714	−0.051856	2.860532
O	2.618681	−1.203984	3.499113
C	1.536087	−1.495302	4.379968
C	5.227379	−0.913990	−0.923558
O	4.722492	−1.981582	−1.170300
O	6.546878	−0.691325	−0.882548
C	8.843372	−1.349299	−1.055310
C	7.412693	−1.829526	−1.143765
O	−0.090152	2.962109	1.109023
C	−0.341403	3.354153	2.464335
O	−2.647938	3.768229	0.440379
C	−3.792668	3.938163	1.296624
C	−4.365728	2.903292	−2.019696
O	−4.339396	1.959947	−0.932087
H	−2.593983	−2.734102	−0.326171
H	−0.342511	−1.957605	0.062134
H	−0.156219	−1.430430	−1.594359
H	−4.017175	−0.130537	−2.367372
H	−0.827875	−4.242669	−1.262358
H	−2.346287	−4.342686	−2.168586
H	−0.979601	−3.426074	−2.826188
H	0.218696	−0.376903	2.531061
H	−3.925203	−0.751894	1.252663
H	−5.835956	−1.712269	2.515371
H	−7.784203	−2.696004	−1.162378
H	−5.867933	−1.751662	−2.431993
H	1.419129	0.564650	−2.303512
H	1.053791	2.072870	−1.477934
H	3.427264	2.661597	−1.380012
H	5.122780	1.116719	−0.237019
H	4.018203	2.769803	−3.778865
H	2.302279	3.149760	−3.562252
H	2.787242	1.554034	−4.160992
H	5.357261	1.019188	2.641913
H	4.821420	−0.227157	3.804647
H	6.355393	−0.429047	2.920113
H	1.981237	−1.981501	5.244467
H	1.025262	−0.583215	4.700424
H	0.816502	−2.173363	3.913777
H	9.518715	−2.184773	−1.249515
H	9.042271	−0.570562	−1.793049
H	9.065255	−0.954802	−0.062598
H	7.194625	−2.603365	−0.406657
H	7.175402	−2.224041	−2.132518
H	−1.256474	3.940969	2.546266
H	−0.402070	2.477684	3.115092
H	0.506922	3.966282	2.764082
H	−4.694790	3.532129	0.840642
H	−3.623474	3.453835	2.261980
H	−3.898964	5.010896	1.442947
H	−3.899041	3.847289	−1.735297
H	−3.855510	2.492054	−2.894400
H	−5.415703	3.065076	−2.253826

B3LYP Energy = −2096.62084443 a.u.

(*aR*,1*S*,3*S*,1'*S*,3'*S*)-**22**, Conf V

C	−1.005399	2.037095	0.762326
C	−2.299347	2.454537	0.413440
C	−3.088528	1.617565	−0.380780
C	−2.634971	0.350436	−0.758504
C	−1.351139	−0.065442	−0.395672
C	−0.526452	0.785994	0.360790
O	−2.792856	−1.545525	−2.286708
C	−1.938436	−2.306055	−1.418512
C	−0.834200	−1.405291	−0.876764
C	−3.538684	−0.531312	−1.601659
C	−1.406237	−3.478479	−2.220891
C	−4.716237	−1.148376	−0.846843
C	0.862589	0.375310	0.740275
C	1.958222	0.677734	−0.075442
C	3.228460	0.223343	0.300370
C	3.416852	−0.476841	1.491644
C	2.322795	−0.736824	2.332108
C	1.054435	−0.327630	1.930800
C	−5.783831	−1.656891	−1.592747
C	−6.867491	−2.270784	−0.977593
C	−6.867768	−2.370029	0.403710
C	−5.835453	−1.879608	1.179773
C	−4.759246	−1.266452	0.541403
C	1.803228	1.501174	−1.333812
C	3.123433	2.136229	−1.747694
O	4.129825	1.111630	−1.811085
C	4.448250	0.548483	−0.542988
C	3.073994	2.800264	−3.109836
F	−7.924869	−2.966356	1.016767
O	4.708533	−0.803500	1.833549
C	5.004269	−2.188705	2.098710
O	2.589377	−1.364782	3.514957
C	1.506337	−1.643584	4.398993
C	5.276240	−0.692066	−0.904809
O	4.813480	−1.765721	−1.207987
O	6.582366	−0.402604	−0.915359
C	8.893796	−0.893349	−1.301011
C	7.490358	−1.453136	−1.346813
O	−0.147320	2.888996	1.414727
C	−0.430264	3.178360	2.789592
O	−2.712886	3.712170	0.781942
C	−3.868174	3.778493	1.638471
C	−4.370752	3.060197	−1.773311
O	−4.346021	2.012939	−0.785451
H	−2.538285	−2.685861	−0.582049
H	−0.309415	−1.920399	−0.069756
H	−0.092193	−1.239686	−1.666015
H	−3.955052	0.075196	−2.406574
H	−0.729120	−4.079848	−1.610983
H	−2.224513	−4.115477	−2.559349
H	−0.857444	−3.123524	−3.095796

H	0.197172	−0.535048	2.555210
H	−5.767412	−1.574064	−2.673031
H	−7.698539	−2.662113	−1.549602
H	−5.875477	−1.973027	2.257111
H	−3.950658	−0.870725	1.141950
H	1.440839	0.878526	−2.159588
H	1.058638	2.283304	−1.175380
H	3.425285	2.871917	−0.989278
H	5.097488	1.237939	0.010331
H	4.038868	3.244996	−3.356742
H	2.318218	3.587893	−3.117529
H	2.820359	2.069932	−3.880934
H	6.086824	−2.243809	2.193865
H	4.531751	−2.519754	3.020705
H	4.678474	−2.811089	1.263122
H	1.948887	−2.134975	5.261951
H	1.009366	−0.724712	4.721169
H	0.775484	−2.312377	3.936599
H	9.601214	−1.661135	−1.620244
H	8.995629	−0.036011	−1.967810
H	9.159695	−0.582432	−0.289671
H	7.370293	−2.308760	−0.681222
H	7.206093	−1.762314	−2.353404
H	−1.342856	3.765331	2.893459
H	−0.513470	2.255446	3.369762
H	0.415022	3.757496	3.155876
H	−4.755759	3.389971	1.140376
H	−3.693231	3.217462	2.560285
H	−4.004368	4.831560	1.875113
H	−5.419151	3.224915	−2.012452
H	−3.929366	3.979139	−1.385764
H	−3.835340	2.748313	−2.673627

B3LYP Energy = −2096.62079718 a.u.

(*aR*,1*S*,3*S*,1’*S*,3’*S*)-**22**, Conf W

C	−0.934005	2.194921	0.426268
C	−2.254007	2.508461	0.075557
C	−3.047422	1.541722	−0.544345
C	−2.555363	0.250253	−0.758103
C	−1.240870	−0.062289	−0.398185
C	−0.414911	0.919057	0.181020
O	−2.717216	−1.883072	−1.932854
C	−1.799147	−2.459593	−0.991624
C	−0.701508	−1.450549	−0.672844
C	−3.465965	−0.785608	−1.392928
C	−1.266306	−3.740697	−1.605128
C	−4.583723	−1.295523	−0.484078
C	0.999459	0.613416	0.566962
C	2.041519	0.666543	−0.366940
C	3.345174	0.370375	0.056790
C	3.607233	0.054375	1.386486
C	2.558691	−0.025118	2.315627
C	1.266147	0.266081	1.893562
C	−4.534565	−1.214625	0.906922
C	−5.555174	−1.741327	1.695616
C	−6.626071	−2.347171	1.067050
C	−6.717185	−2.445456	−0.311191
C	−5.687043	−1.915294	−1.077797
C	1.809075	1.069340	−1.805781
C	3.086999	1.594684	−2.446920
O	4.126716	0.620157	−2.263528
C	4.519166	0.451357	−0.906556
C	2.959839	1.838149	−3.938055
F	−7.629861	−2.859945	1.827590
O	4.900774	−0.261075	1.733045
C	5.563877	0.651316	2.625379
O	2.896711	−0.392445	3.584692
C	1.862209	−0.488833	4.562028
C	5.424622	−0.790032	−0.934479
O	6.629174	−0.722726	−1.004070
O	4.719340	−1.923933	−0.961694
C	5.861584	−3.714354	0.274901
C	5.450451	−3.176788	−1.081796
O	−0.169047	3.117443	1.100294
C	0.263842	4.262187	0.348087
O	−2.749864	3.768631	0.323463
C	−3.488619	3.883887	1.552420
C	−4.552835	2.777189	−1.925791
O	−4.354291	1.809086	−0.879332
H	−2.346007	−2.703783	−0.072459
H	−0.119317	−1.802143	0.181556
H	−0.007971	−1.407975	−1.519435
H	−3.939777	−0.335523	−2.266176
H	−0.545676	−4.211836	−0.933670
H	−2.079030	−4.444728	−1.788591
H	−0.767670	−3.531307	−2.553999
H	0.444259	0.226280	2.593896
H	−3.697038	−0.728411	1.390374
H	−5.523975	−1.681007	2.775599
H	−7.576232	−2.921599	−0.765368
H	−5.742629	−1.985793	−2.157683
H	1.458150	0.214262	−2.394187
H	1.027761	1.829302	−1.862959
H	3.390384	2.524835	−1.945998
H	5.171404	1.282074	−0.610324
H	3.900732	2.206668	−4.348465
H	2.182675	2.579119	−4.135097
H	2.694591	0.913285	−4.454283
H	6.588310	0.296008	2.711965
H	5.563309	1.662696	2.210429
H	5.087202	0.652754	3.605301
H	2.353381	−0.794285	5.482519
H	1.368496	0.474269	4.715546
H	1.119706	−1.240202	4.280941
H	6.343084	−4.686330	0.145081
H	6.567062	−3.045478	0.767288
H	4.993066	−3.845964	0.921656
H	4.748549	−3.844196	−1.578416
H	6.312595	−3.016052	−1.727434
H	−0.586400	4.817684	−0.047460
H	0.819647	4.887154	1.044178

H	0.924763	3.957744	−0.467501
H	−4.359285	3.224917	1.543943
H	−2.850518	3.647845	2.407061
H	−3.815144	4.919875	1.617287
H	−4.184807	3.758592	−1.628154
H	−4.051689	2.455271	−2.842586
H	−5.626603	2.818599	−2.095609

B3LYP Energy = −2096.62074552 a.u.

(*aR*,1*S*,3*S*,1'*S*,3'*S*)-**22**, Conf X

C	−0.848416	2.204297	0.301566
C	−2.152496	2.534724	−0.098897
C	−2.951672	1.544443	−0.677630
C	−2.496435	0.226674	−0.782150
C	−1.202307	−0.099001	−0.368070
C	−0.367645	0.898417	0.166779
O	−2.680321	−1.960279	−1.850602
C	−1.798909	−2.509574	−0.859600
C	−0.687369	−1.510303	−0.558498
C	−3.415167	−0.819608	−1.387559
C	−1.278644	−3.829129	−1.397919
C	−4.568583	−1.253171	−0.483356
C	1.034515	0.586612	0.591264
C	2.109579	0.736063	−0.292286
C	3.398804	0.410639	0.149901
C	3.615603	−0.021498	1.455701
C	2.535734	−0.189368	2.334710
C	1.254688	0.125301	1.891126
C	−4.567228	−1.071176	0.898747
C	−5.621935	−1.527642	1.686622
C	−6.678058	−2.166344	1.065831
C	−6.722084	−2.364197	−0.304122
C	−5.658826	−1.902689	−1.069779
C	1.912415	1.266156	−1.694330
C	3.212701	1.804209	−2.277063
O	4.238800	0.808878	−2.123221
C	4.600081	0.581620	−0.767065
C	3.122533	2.131464	−3.754630
F	−7.714630	−2.611105	1.824976
O	4.897856	−0.362029	1.822373
C	5.526831	0.483060	2.801087
O	2.831572	−0.665352	3.578413
C	1.762621	−0.873453	4.498696
C	5.549948	−0.623371	−0.826667
O	6.753702	−0.513883	−0.838641
O	4.894174	−1.779677	−0.941157
C	4.754410	−4.162157	−1.131847
C	5.698682	−2.984260	−1.057106
O	0.011468	3.181933	0.742644
C	−0.220595	3.702693	2.057145
O	−2.565971	3.840474	0.005201
C	−3.709967	4.084373	0.844428
C	−4.274308	2.653732	−2.315408
O	−4.220523	1.837718	−1.130126
H	−2.374438	−2.697802	0.055429
H	−0.137669	−1.835399	0.326859
H	0.032076	−1.519848	−1.385008
H	−3.855740	−0.405993	−2.295141
H	−0.589517	−4.284766	−0.684039
H	−2.101815	−4.522680	−1.574510
H	−0.746773	−3.674123	−2.339130
H	0.407440	0.006734	2.551631
H	−3.740701	−0.560060	1.375094
H	−5.627806	−1.388804	2.759788
H	−7.570923	−2.863112	−0.753018
H	−5.676942	−2.052044	−2.142868
H	1.534185	0.475467	−2.352300
H	1.162764	2.059340	−1.689437
H	3.515596	2.701090	−1.718860
H	5.213342	1.417899	−0.408406
H	4.073158	2.521017	−4.120946
H	2.350481	2.883597	−3.927670
H	2.869362	1.237569	−4.328508
H	6.550393	0.126520	2.893005
H	5.532777	1.522931	2.463992
H	5.019873	0.407882	3.762983
H	1.246157	0.062437	4.728520
H	1.044224	−1.602885	4.115352
H	2.223110	−1.262574	5.403353
H	5.331981	−5.084365	−1.221030
H	4.139767	−4.228701	−0.233087
H	4.097882	−4.084867	−1.999727
H	6.318798	−2.900809	−1.950699
H	6.356362	−3.042591	−0.189080
H	0.595794	4.393681	2.257611
H	−1.168337	4.239158	2.111363
H	−0.204565	2.899566	2.798772
H	−4.605709	3.609744	0.445406
H	−3.525809	3.717998	1.857779
H	−3.838458	5.164360	0.866771
H	−3.747956	2.165149	−3.139234
H	−5.328265	2.753800	−2.565427
H	−3.839474	3.637966	−2.137172

B3LYP Energy = −2096.62073045 a.u.

(*aR*,1*S*,3*S*,1'*S*,3'*S*)-**22**, Conf Y

C	−0.959538	2.259257	0.347490
C	−2.279353	2.520832	−0.044180
C	−3.034890	1.505878	−0.633410
C	−2.506158	0.218740	−0.774588
C	−1.193345	−0.042271	−0.370996
C	−0.403997	0.986171	0.176908
O	−2.589645	−1.972694	−1.846302
C	−1.680538	−2.479102	−0.857507
C	−0.614808	−1.428492	−0.565398
C	−3.376616	−0.868571	−1.379081
C	−1.103572	−3.775504	−1.394354
C	−4.504889	−1.360666	−0.473509
C	1.008785	0.734380	0.605241
C	2.069314	0.761728	−0.308495

C	3.367209	0.494787	0.150427
C	3.611113	0.240388	1.499498
C	2.550358	0.250452	2.419647
C	1.259772	0.477102	1.954341
C	−5.586552	−2.020785	−1.063394
C	−6.622891	−2.538185	−0.296293
C	−6.559956	−2.385988	1.078590
C	−5.511218	−1.738653	1.703264
C	−4.484237	−1.225493	0.914046
C	1.854717	1.093628	−1.768388
C	3.133231	1.609283	−2.414881
O	4.185866	0.664312	−2.164996
C	4.556990	0.575854	−0.793613
C	3.026818	1.774466	−3.918342
F	−7.569898	−2.885996	1.839432
O	4.922514	0.082323	1.875797
C	5.284189	−1.085060	2.635311
O	2.873180	0.059326	3.732038
C	1.829861	0.095814	4.703698
C	5.516135	−0.621718	−0.757153
O	6.717604	−0.508977	−0.814539
O	4.869600	−1.792204	−0.744621
C	4.747981	−4.181960	−0.848887
C	5.681898	−2.993559	−0.852990
O	−0.230712	3.233353	0.987615
C	0.178850	4.355818	0.189723
O	−2.815278	3.776462	0.135349
C	−3.544612	3.937285	1.364740
C	−4.545826	2.641835	−2.093779
O	−4.340735	1.721505	−1.006339
H	−2.242570	−2.690722	0.060581
H	−0.044237	−1.723221	0.317924
H	0.096471	−1.410348	−1.398013
H	−3.838457	−0.472838	−2.284463
H	−0.390615	−4.196931	−0.682882
H	−1.895464	−4.506330	−1.563657
H	−0.584946	−3.600465	−2.339468
H	0.430147	0.488717	2.646259
H	−5.620221	−2.133412	−2.140628
H	−7.465439	−3.045350	−0.747790
H	−5.502039	−1.636229	2.780488
H	−3.664292	−0.706926	1.393868
H	1.525927	0.206399	−2.320906
H	1.062828	1.837446	−1.872804
H	3.412507	2.568692	−1.957015
H	5.167691	1.446117	−0.524054
H	3.967111	2.140971	−4.331834
H	2.238805	2.488557	−4.165126
H	2.788149	0.819575	−4.391125
H	4.816938	−1.976799	2.211572
H	6.366192	−1.165536	2.553267
H	4.993204	−0.980049	3.678175
H	2.315054	−0.065879	5.663090
H	1.327274	1.066335	4.710246
H	1.095787	−0.695798	4.530996
H	5.331128	−5.101052	−0.932928
H	4.171313	−4.226437	0.076231
H	4.054986	−4.138514	−1.690146
H	6.262073	−2.933505	−1.774571
H	6.379148	−3.018159	−0.014658
H	0.844181	4.032399	−0.614829
H	−0.682694	4.878321	−0.225724
H	0.723821	5.017853	0.859496
H	−4.393350	3.251098	1.403411
H	−2.890876	3.770285	2.223761
H	−3.904513	4.964158	1.375362
H	−5.616288	2.641661	−2.287940
H	−4.215956	3.645285	−1.826896
H	−4.014202	2.301228	−2.986301

B3LYP Energy = −2096.62071839 a.u.

(*aR*,1*S*,3*S*,1’*S*,3’*S*)-**22**, Conf Z

C	−0.924283	2.200101	0.455802
C	−2.206441	2.544596	0.005510
C	−3.006978	1.570629	−0.593485
C	−2.512076	0.275963	−0.796342
C	−1.215335	−0.048340	−0.388745
C	−0.416095	0.914658	0.255713
O	−2.620248	−1.826908	−2.036805
C	−1.742404	−2.428460	−1.074649
C	−0.662720	−1.428366	−0.677728
C	−3.390499	−0.737869	−1.509059
C	−1.180148	−3.690127	−1.702672
C	−4.561835	−1.263180	−0.681727
C	0.968509	0.586125	0.721930
C	2.083214	0.796638	−0.097446
C	3.349196	0.422725	0.371029
C	3.512257	−0.103406	1.652757
C	2.398312	−0.271226	2.489732
C	1.136105	0.053998	2.001463
C	−5.672485	−1.779559	−1.355626
C	−6.747617	−2.325211	−0.665225
C	−6.694348	−2.349649	0.718307
C	−5.617901	−1.849108	1.425546
C	−4.552436	−1.303291	0.712558
C	1.950019	1.433896	−1.461827
C	3.272177	2.025777	−1.930215
O	4.293843	1.021054	−1.819129
C	4.589858	0.659713	−0.474852
C	3.252183	2.485486	−3.374894
F	−7.741595	−2.879125	1.404353
O	4.799251	−0.351909	2.061915
C	5.116694	−1.650101	2.595129
O	2.640062	−0.730197	3.752926
C	1.536793	−0.899599	4.639730
C	5.522780	−0.550492	−0.625147
O	6.727316	−0.463387	−0.602101
O	4.844934	−1.680444	−0.859936
C	5.899954	−2.979772	−2.652508
C	5.607605	−2.881039	−1.168086
O	−0.114013	3.167293	1.004267

C	−0.469429	3.605414	2.325498
O	−2.677486	3.828502	0.165237
C	−2.259124	4.745898	−0.861161
C	−5.252217	2.319791	−0.176803
O	−4.245137	1.875889	−1.104331
H	−2.326620	−2.702835	−0.187275
H	−0.118348	−1.806083	0.189907
H	0.068784	−1.356711	−1.490670
H	−3.809328	−0.258836	−2.394272
H	−0.487573	−4.180110	−1.015253
H	−1.982314	−4.389686	−1.941477
H	−0.642490	−3.451888	−2.622915
H	0.264256	−0.084283	2.624949
H	−5.698160	−1.755270	−2.438668
H	−7.612210	−2.721058	−1.181696
H	−5.616901	−1.883023	2.507152
H	−3.710663	−0.896854	1.258136
H	1.615923	0.696065	−2.200151
H	1.191033	2.217478	−1.432202
H	3.543270	2.868610	−1.279121
H	5.199382	1.444147	−0.009899
H	4.217761	2.906896	−3.657274
H	2.486037	3.250061	−3.517062
H	3.031602	1.647206	−4.038983
H	4.785653	−1.741483	3.627294
H	4.657705	−2.435805	1.991005
H	6.200238	−1.733569	2.539588
H	1.961338	−1.259941	5.573472
H	1.021289	0.047740	4.817854
H	0.825782	−1.636543	4.256604
H	6.403845	−3.926525	−2.859462
H	4.976830	−2.949025	−3.233035
H	6.549979	−2.168788	−2.981558
H	6.523014	−2.877009	−0.578498
H	4.966584	−3.694931	−0.833993
H	0.256546	4.368974	2.597170
H	−1.474095	4.029274	2.341246
H	−0.402264	2.774913	3.033107
H	−2.711529	5.704931	−0.616773
H	−1.172032	4.845061	−0.870797
H	−2.612254	4.413674	−1.840148
H	−6.160083	2.445062	−0.763004
H	−5.420272	1.562246	0.591810
H	−4.971263	3.265223	0.284341

B3LYP Energy = −2096.62059567 a.u.

6. References

1. MacroModel; Schrödinger LLC, 2015. Available online: https://newsite.schrodinger.com/platform/products/macromodel (accessed on 12 July 2024).
2. Frisch, M. J.; Trucks, G. W.; Schlegel, H. B.; Scuseria, G. E.; Robb, M. A.; Cheeseman, J. R.; Scalmani, V.; Barone, G.; Mennucci, B.; Petersson, G. A., et al. *Gaussian 09 Revisions C.01*; Gaussian, Inc.: Wallingford, CT, USA, 2010.
3. Frisch, M. J.; Trucks, G. W.; Schlegel, H. B.; Scuseria, G. E.; Robb, M. A.; Cheeseman, J. R.; Scalmani, V.; Barone, G.; Mennucci, B.; Petersson, G. A., et al. *Gaussian 09 Revisions E.01*; Gaussian, Inc.: Wallingford, CT, USA, 2013.
4. Chai, J.-D.; Head-Gordon, M. Systematic optimization of long-range corrected hybrid density functionals. *J. Chem. Phys.* **2008,** *128*, 084106, https://doi.org/10.1063/1.2834918.
5. Stephens, P. J.; Harada, N. ECD cotton effect approximated by the Gaussian curve and other methods. *Chirality* **2010,** *22*, 229–233, https://doi.org/10.1002/chir.20733.
6. Varetto, U. *MOLEKEL 5.4*; Swiss National Supercomputing Centre: Manno, Switzerland, 2009.
7. Kerti, G.; Kurtán, T.; Illyés, T.-Z.; Kövér, K. E.; Sólyom, S.; Pescitelli, G.; Fujioka, N.; Berova, N.; Antus, S. Enantioselective Synthesis of 3-Methylisochromans and Determination of Their Absolute Configurations by Circular Dichroism. *Eur. J. Org. Chem.* **2007**, 296-305.
8. Inoue, K.; Makino, Y.; Itoh, N. Production of (R)-chiral alcohols by a hydrogen-transfer bioreduction with NADH-dependent *Leifsonia* alcohol dehydrogenase (LSADH). *Tetrahedron: Asymmetry* **2005,** *16*, 2539–2549, https://doi.org/10.1016/j.tetasy.2005.06.036.
9. Ren, X.; She, X.; Peng, K.; Su, Y.; Xie, X.; Pan, X.; Zhang, H. First Enantioselective Synthesis of the Neolignans Rhaphidecursinol A and Virolongin B. *J. Chin. Chem. Soc.* **2004,** *51*, 969–974, https://doi.org/10.1002/jccs.200400144.
10. González-Liste, P. J.; León, F.; Arribas, I.; Rubio, M.; García-Garrido, S. E.; Cadierno, V.; Pizzano, A. Highly Stereoselective Synthesis and Hydrogenation of (Z)-1-Alkyl-2-arylvinyl Acetates: a Wide Scope Procedure for the Preparation of Chiral Homobenzylic Esters. *ACS Catal.* **2016,** *6*, 3056–3060, https://doi.org/10.1021/acscatal.6b00282.
11. Parsons, S.; Flack, H. D.; Wagner, T. Use of intensity quotients and differences in absolute structure refinement. *Acta Crystallogr., Sect. B: Struct. Sci., Cryst. Eng. Mater.* **2013,** *69*, 249–259, https://doi.org/10.1107/S2052519213010014.
12. Sheldrick, G. A short history of SHELX. *Acta Crystallogr., Sect. A: Found. Adv.* **2008,** *64*, 112–122, https://doi.org/10.1107/S0108767307043930.

13. Westrip, S. publCIF: software for editing, validating and formatting crystallographic information files. *J. Appl. Crystallogr.* **2010,** *43*, 920–925, https://doi.org/10.1107/S0021889810022120.
14. Edgington, P. R.; McCabe, P.; Macrae, C. F.; Pidcock, E.; Shields, G. P.; Taylor, R.; Towler, M.; Van De Streek, J. Mercury: visualization and analysis of crystal structures. *J. Appl. Crystallogr.* **2006,** *39*, 453–457, https://doi.org/10.1107/S002188980600731X.
15. Spek, A. Single-crystal structure validation with the program PLATON. *J. Appl. Crystallogr.* **2003,** *36*, 7–13, https://doi.org/10.1107/S0021889802022112.
